# A Porphyrin‐Based Organic Network Comprising Sustainable Carbon Dots for Photopolymerization

**DOI:** 10.1002/anie.202208180

**Published:** 2022-09-01

**Authors:** Xiongfei Luo, Jianyong Wan, Nicolai Meckbach, Veronika Strehmel, Shujun Li, Zhijun Chen, Bernd Strehmel

**Affiliations:** ^1^ Northeast Forestry University Key Laboratory of Bio-based Material Science and Technology of Ministry of Education Hexing Road 26 150040 Harbin China; ^2^ Department of Chemistry Institute for Coatings and Surface Chemistry Niederrhein University of Applied Sciences Adlerstr. 1 47798 Krefeld Germany

**Keywords:** Carbon Dots, Photo-ATRP, Porphyrins, Sensitization, Visible-Light LEDs

## Abstract

Sustainable carbon dots (**CD**s) based on furfuraldehyde (F‐**CD**) resulted in a photosensitive material after pursuing the Alder‐Longo reaction. The porphyrin moiety formed connects the F‐**CD**s in a covalent organic network. This heterogeneous material (P‐**CD**) was characterized by XPS indicating incorporation of the respective C, N and O moieties. Time resolved fluorescence including global analysis showed contribution of three linked components to the overall dynamics of the excited state. Electrochemical and photonic properties of this heterogeneous material facilitated photopolymerization in a photo‐ATRP setup where either CuBr_2_/TPMA, FeBr_3_/Br^−^ or a metal free reaction setup activated controlled polymerization. Chain extension experiments worked in all three cases showing end group fidelity for activation of controlled block copolymerization using **MMA** and styrene as monomers. Traditional radical polymerization using a diaryl iodonium salt as co‐initiator failed.

## Graphical abstract

Alder‐Longo reaction between aldehyde groups of **CD**s based on furfuraldehyde (F‐**CD**s) and dipyrrolo methane results in porphyrin patterns connecting the F‐**CD**s. This gives a light‐sensitive organic network. This can initiate photopolymerization following a photo‐ATRP protocol upon 405 LED excitation. End‐group fidelity was shown by block polymerization where CuBr_2_/TPMA, FeBr_3_/Br^−^, and a metal‐free photocatalyst resulted in formation of polymer.

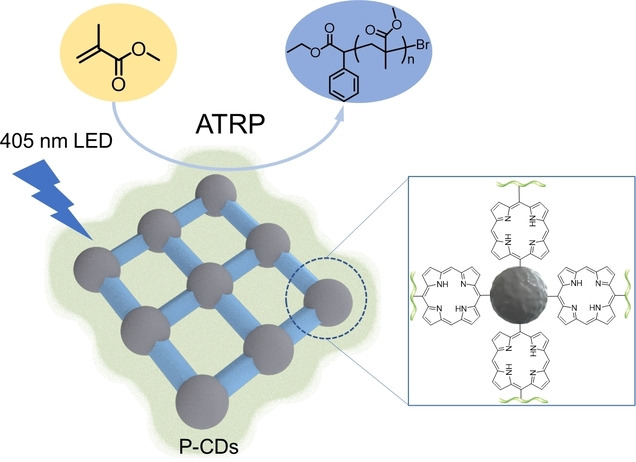

## Introduction

Photochemistry has demonstrated great potential to establish new objectives in different fields of material synthesis, biology, environmental science, and advanced manufacturing.[^1^, ^2^, ^3^, ^4^, ^5^, ^6^, ^7^, ^8^, ^9^, ^10^] Among all types of photoreactions disclosed, those requesting to operate with a sensitizer following photoinduced electron transfer (**PET**) have received big attention.[^2^, ^3^] Sensitized formation of radicals to initiate photopolymerization of vinyl monomers represents one interesting example since it connects many applications in different technology fields.[^11^, ^12^] Furthermore, photoinduced reversible‐deactivation radical polymerization (RDRP) has received increased attention^[13]^ after its first report.^[14]^ It enables therefore the tailor‐made photoinduced synthesis at the nanoscale.

The design of the photoinitiating system represents one of the most challenging elements to activate the ATRP process.^[3]^ This can proceed either by sensitized reduction of the metal bromide into its lower oxidation number^[15]^ or without any metal ion; that is the metal‐free photo‐ATRP.[^2^, ^16^, ^17^, ^18^] Sensitizers bring the benefit to use either visible[^17^, ^18^] or NIR[^19^, ^20^] light for excitation. On the other hand, activation of ATRP also proceeds with UV light without any sensitizer in the case of CuBr_2_/L (L=ligand)^[21]^ or FeBr_3_ catalysts.[^13^, ^22^, ^23^] Moreover, up‐conversion nanoparticles converting NIR light into visible and UV light operated as internal lamps to excite directly the CuBr_2_‐complex^[24]^ or thioxanthone^[25]^ in a metal‐free photo‐ATRP protocol.

Generally, effective sensitizers for activation of ATRP could be divided into two types; that is material with either fossil or biomass origin. Biomass‐derived sensitizers have moved into the focus because they are renewable, show low toxic response of living cells, and are sustainable.[^26^, ^27^] Preliminary work demonstrated activation of ATRP by using biomass‐derived carbon dots (**CD**s) in combination with a 405 nm emitting LED.[^26^, ^27^] Furthermore, **CD**s have been known as novel fluorescent carbon nanomaterials exhibiting sizes below 10 nm.[^26^, ^27^] It can exhibit intensive photoluminescence.^[28]^ Their simple preparation and the fact that they are sustainable raw materials enabled **CD**s for use in wide areas including bioimaging, optoelectronics, and photocatalysis[^29^, ^30^, ^31^, ^32^, ^33^, ^34^] In addition, **CD**s represent an ensemble of different structures unified in the solid material. Each of it uptakes a certain function in a photoredox system requesting the necessity to employ different methods for exploration of the material. This relates to spectroscopic methods such as XPS to receive quantitative information of the composition, electronic spectroscopy to obtain information about properties of the excited state, and cyclic voltammetry providing quantities for oxidation and reduction capabilities. These experiments often form the prerequisite to study the photoinduced electron transfer in the aforementioned ATRP systems.

Recently, **CD**s were also employed as photoreduction reagents in a Cu^2+^ based system resulting in activation of a photo‐ATRP.^[35]^ Here, excitation of up‐conversion nanoparticles resulted in excitation of **CD**s whose excited state resulted in formation of the respective hole **CD** ^+^⋅. Dispersity of molecular weight remained around 1.2. Nevertheless, several shortages still remained in **CD**‐assisted photo‐ATRP. There exists a certain demand to receive materials that operate better with visible light. Additionally, most of these **CD**s showed no sufficient electron/hole separation resulting in a diminished efficiency for the reduction of the metal ion.^[36]^ This can lead to lower conversion in the **CD**s‐assisted photo‐ATRP as long as the photoinduced electron transfer from photo‐excited **CD** (CD*) to the metal ion proceeds from the first excited singlet state resulting also in efficient back electron transfer. Thus, a better electron/hole separation of **CD** ^+^⋅ makes the electron back transfer of the heterogeneous sensitizer less favorable.

Recent results reported attempts to widen the absorbance and enhancement of electron/hole separation efficiency of **CD**s by integrating them with a large, conjugated structure to prepare a **CD**‐based network with covalent organic frameworks structure.[^37^, ^38^, ^39^] Such **CD**‐based networks can be of interest for photocatalysis,^[40]^ photodynamic therapy,^[37]^ and to construct light emitting devices.^[38]^ Nevertheless, almost no attention has been paid to use these materials for photopolymerization.

Motivated by these points, novel **CD**‐based organic network materials comprising porphyrin (P‐**CD**s) were employed as heterogeneous sensitizer to activate photo‐ATRP using either CuBr_2_/TPMA or FeBr_3_/Br^−^ in amounts <100 ppm. Experiments pursued under metal‐free conditions complement the studies. The simple separation of P‐**CD**s by reaction with aldehyde moieties of the **CD**s resulted in P‐**CD**s with efficient sensitizing properties in a photo‐ATRP reaction protocol. This describes a new possibility to synthesize tailor‐made polymers on the one hand while the heterogeneous nature of the network materials brings new unknown parameters and challenges into this system on the other hand. Consequently, results are critically compared of a previous heterogeneous system whose activation worked with red light.^[41]^ From this point of view, this type of polymerization represents an interesting example where redox processes control the efficiency of the overall process.

## Results and Discussion

**Figure 1:**
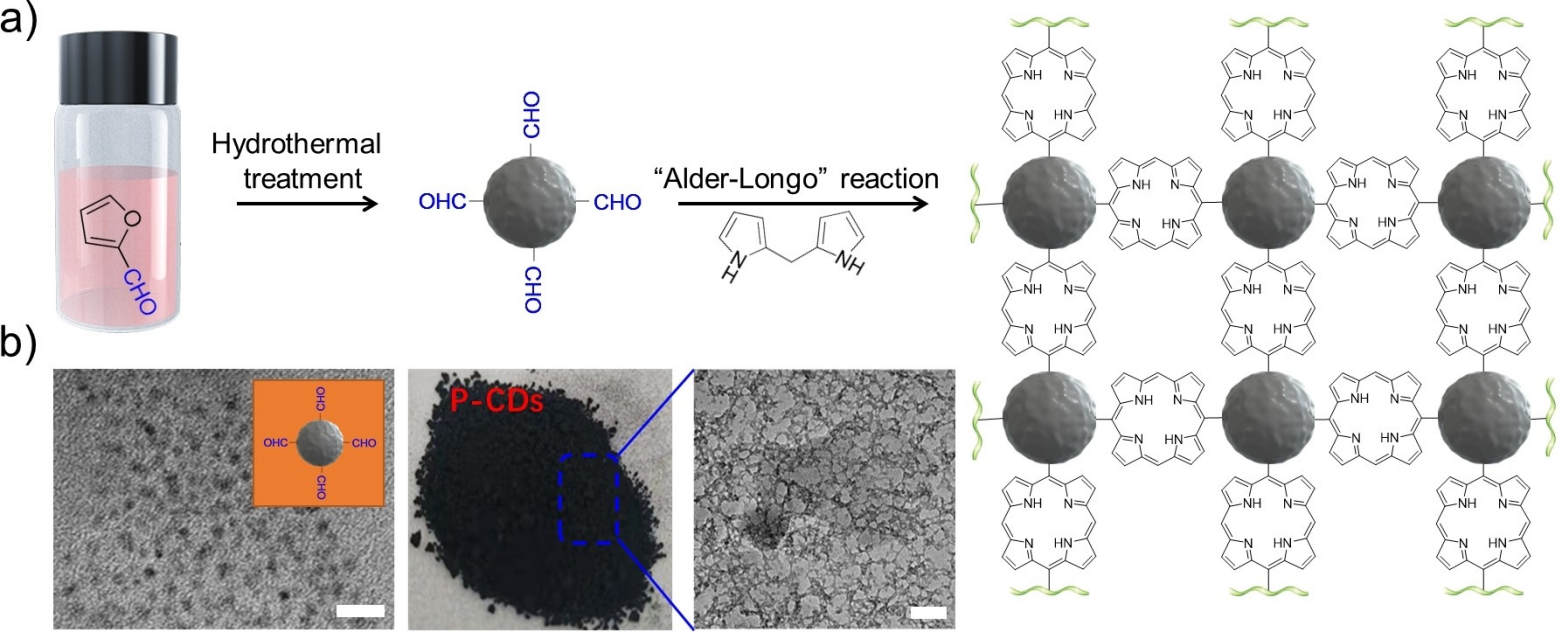
Part (a) schematically shows synthesis of P‐**CD**s. This results as shown in part (b) in P‐**CD**s (middle) with the respective TEM images of furfuraldehyde **CD**s (left slide, scale bar=20 nm) as well as P‐**CD**s (right slide of part (b), scale bar=50 nm).

P‐**CD**s were prepared via post‐chemical modification of **CD**s derived from furfuraldehyde (F‐**CD**s) via solvothermal method with di(1*H*‐pyrrol‐2‐yl)methane to produce P‐**CD**s via “Alder‐Longo” reaction[^42^, ^43^] (Figure 1a; see Supporting Information for the details). The furfural used originated from hemicellulose. It can be therefore seen as a sustainable resource. The **CD**s exhibited a reactive surface decorated with aldehyde moieties (100 mmol g^−1^, determined by titration method). Thus, binding of porphyrin moieties can successfully proceed by reaction of aldehyde groups of the **CD** with dipyrrolomethane (Figure 1a). After reaction, the amount of aldehyde groups on the surface of **CD**s decreased to 7.5 mmol g^−1^. This suggests efficient conversion of aldehyde groups bound on **CD**s resulting in formation of the porphyrin pattern shown in Figure 1a. In addition, the signal of C=O in FT‐IR spectra almost disappeared in P‐**CD**s as concluded by comparison to the **CD**s derived from furfuraldehyde. It furthermore confirmed that dipyrrolomethane successfully reacted with the aldehyde on the surface of **CD**s (Figure S1). Figure 1b shows the visual appearance of the material used. TEM images exhibited a dispersed sphere morphology of **CD**s with a diameter of 5–7 nm (Figure 1b). TEM images depict the crosslinked morphology of P‐**CD**s in Figure 1b, which completely differs from that of the **CD**s derived from furfural (Figure 1b). These results indicate the successful strategy of forming the porphyrin pattern after post‐chemical modification.

**Figure 2:**
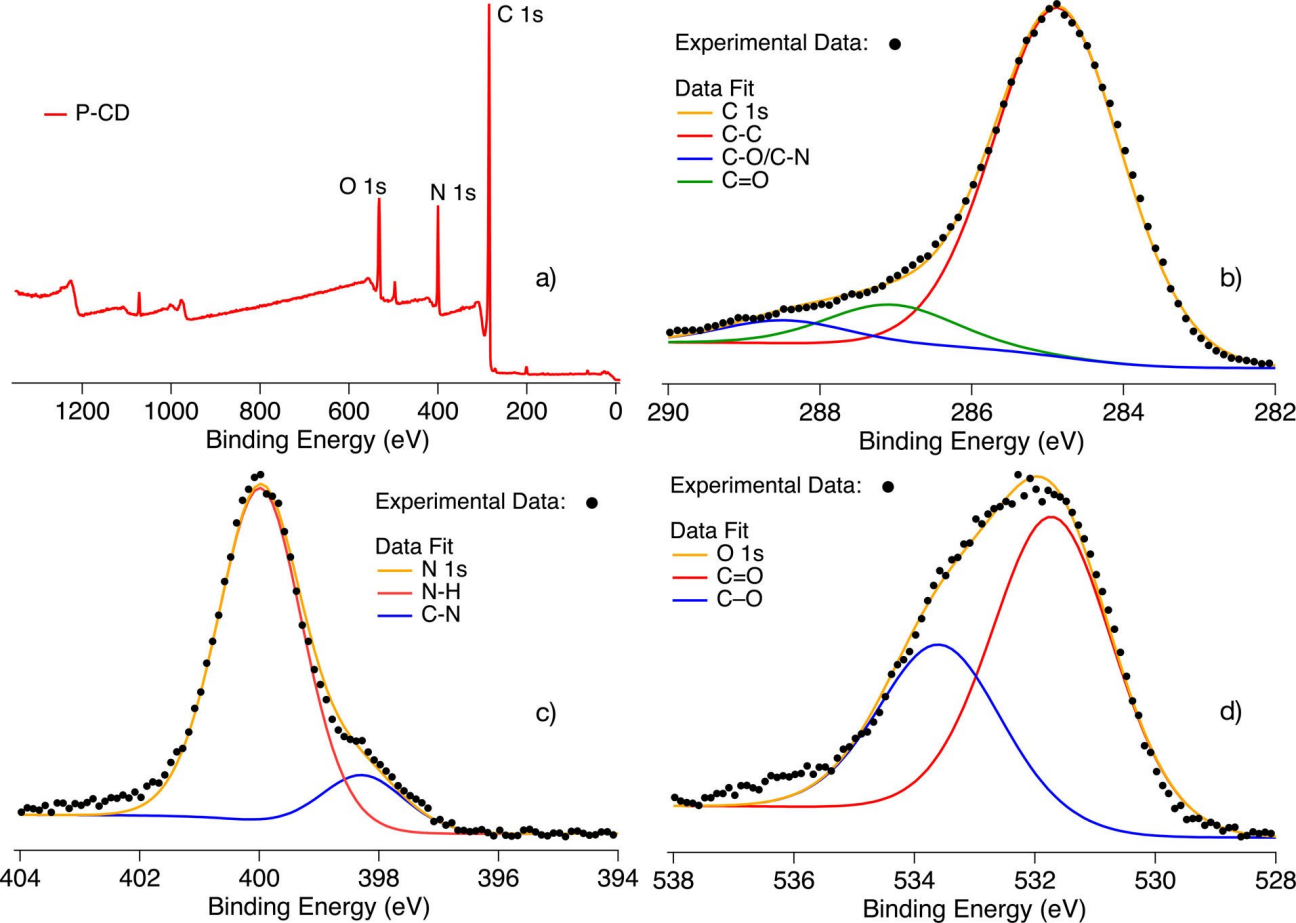
Full XPS spectrum of P‐**CD**s (a) with respective XPS spectra (⋅) of C1s (b), N1s (c), and O1s (d). The graph also shows the data fit of the C−C and C−O/C−N in b), N−H and C−N in (c), and the contribution of C−O and C=O in (d). Adding of the fit function including a background results in an envelope for C 1s, N 1s and O 1s as shown in (b), (c) and (d) respectively.

XPS provided deeper understanding of the chemical structure in such morphology. Full XPS spectra showed that P‐**CD**s consist of C (75.0 %), N (13.4 %) and O (11.6 %) elements (Figure 2a). High resolution XPS furthermore suggested that there are three peaks in the C1s spectrum, at 284.8, 287.08 and 288.48 eV, attributed to C−C, C−O/C−N and C=O groups, respectively (Figure 2b). Peaks at 398.4 and 400.0 eV in the N1s spectrum, were assigned to C−N and N−H, respectively (Figure 2c). Additionally, peaks at 531.8 and 533.7 eV in the O1s spectrum, belong to C−O and C=O, respectively (Figure 2d). Thus, the P‐**CD** combines all of these substructures resulting in a material that absorbs light in the UV and visible part while the excited state either fluoresces or reacts either with CuBr_2_, FeBr_3_ or the halide initiator in a metal free photo‐ATRP approach resulting in activation of the ATRP. In addition, the XRD spectrum showed a broad peak of around 22°, indicating the amorphous nature of P‐**CD**s (see Figure S2 in the Supporting Information).

**Figure 3:**
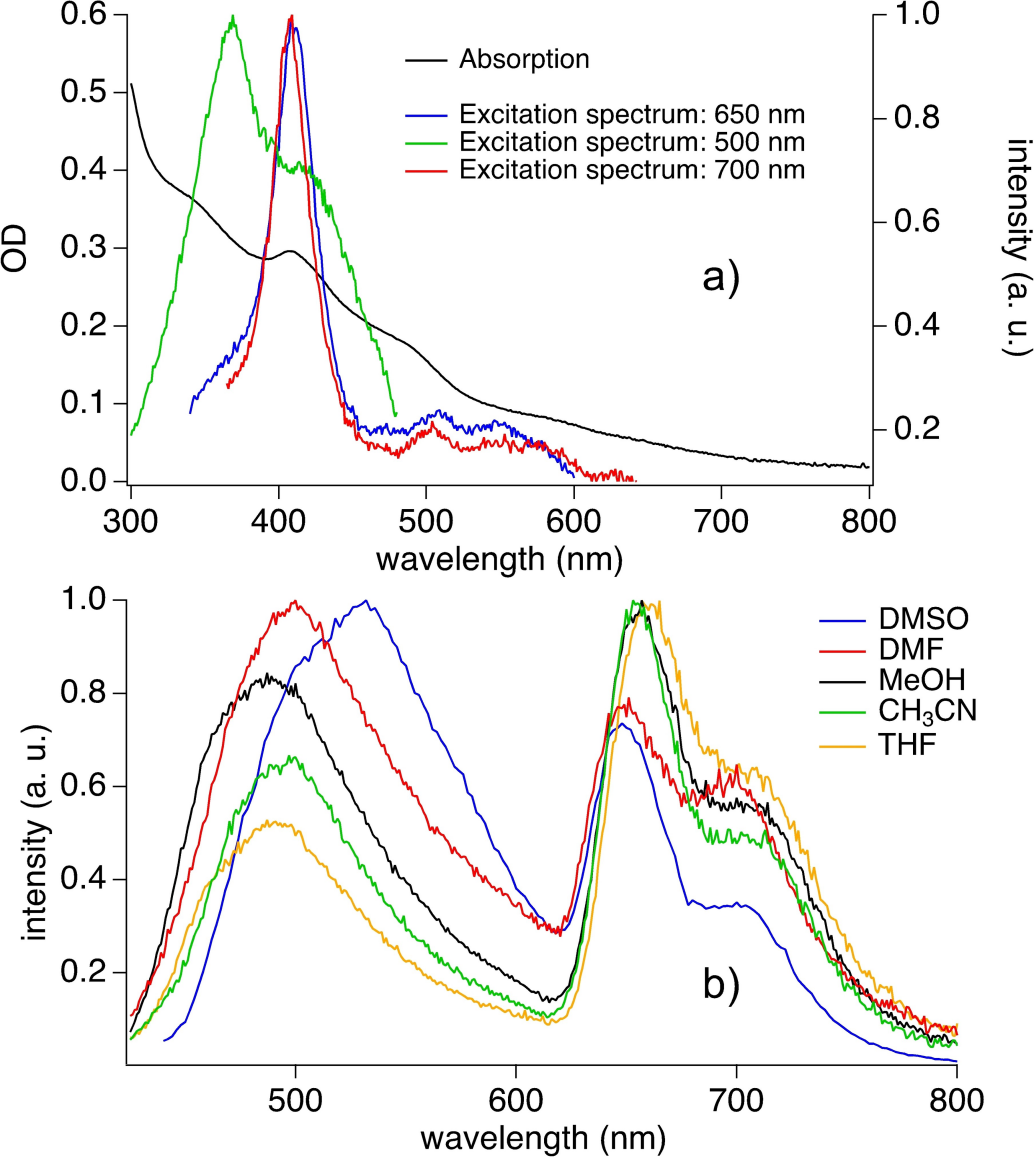
UV/Vis spectrum of P‐**CD**s in comparison with fluorescence excitation spectra obtained by excitation at 500 nm, 650 nm, and 700 nm in DMSO (a). Part (b) shows fluorescence spectra of P‐**CD**s obtained in different solvents excited at 420 nm.

Figure 3a shows the absorption of the P‐**CD**s dispersed in DMSO. It covers the UV and entire visible range explaining the black appearance of the material, see also Figure 1b. There are some slight bands on the spectrum, which may be attributed to the porphyrin pattern. Particularly the feature appearing around 400 nm can be assigned to the porphyrin absorption.^[44]^ This is concluded from the comparison with the absorption spectrum of the respective **CD** comprising no porphyrin, see Figure S3 in Supporting Information. Thus, the absorption of the **CD** derived from furfural without porphyrin shows too much hypsochromic shifted overlap in some parts with monomer and alkyl bromide absorption. Interestingly, the P‐**CD**s exhibited a wider visible absorbance than most of **CD**s used for photopolymerization examined in previous work,[^26^, ^27^] indicating their capacity in utilizing visible light for photopolymerization (Figure 3a).[^26^, ^27^] Here, the material made by **CD**s derived from furfural and dipyrrolomethane results in partially comparable properties with that of materials comprising porphyrin moieties^[44]^ although, porphyrin was not used for the synthesis. The excitation spectrum supports these findings by appearance of a band in the excitation spectrum at around 400 nm. It demonstrates good overlap with the band appearing in the absorption spectrum. The spectral envelope of the band obtained at around 400 nm appeared nearly at the same wavelength indicating that this transition originated from the same excited state. Nevertheless, this differed if the emission was fixed at around 500 nm. Here, a dual band in the excitation spectrum indicated a composition of the excited state emission originating from two different states where one may assign to the porphyrin.

Consideration of the emission spectra in Figure 3b demonstrates a dual emission in solvents of different polarity. One band appearing at 649 nm with a shoulder at 699 nm assigns to the porphyrin^[44]^ while the other relates to material in which the carbon dot material must be involved as obtained at shorter wavelengths. Furthermore, the ratio of both bands; that is the emission appearing at shorter wavelength around 500–550 nm with respect to that at 650 nm increases with increasing solvent polarity^[45]^ in aprotic solvents. This indicates that the shorter wavelength emission receives somehow emission depending on the surrounding from the longer wavelength as assigned to that of the porphyrin (Table 1). Data obtained in polar aprotic solvents such as CH_3_CN and THF exhibited the lowest ratio of the intensities *I*_blue_/*I*_red_ indicating that a polar surrounding favors formation of the emitting state that receives somehow intensity of the emission at 650 nm. However, data obtained in protic solvents remain between them indicating an influence of these solvents on the emission of the excited state. The P‐**CD**s had a wider and longer visible absorbance than **CD**s. P‐**CD**s show a certain better capacity in utilizing visible light for photopolymerization (Figure S3).

**Table 1:** Summary of P‐**CD**s fluorescence lifetime in different solvents. Excitation was carried out at 376 nm. The decay times *τ*_1_–*τ*_3_ were obtained by global analysis studies where fluorescence decays were collected between 450–720 nm in an interval of 10 nm. The global *χ* ^2^ obtained was 1.122 using DMSO as solvent. The table also includes the ratio of the fluorescence bands shown in Figure 3 obtained around 500–550 nm (*I*_blue_) and around 650 nm (*I*_red_).

Head 1^[a]^	*I*_blue_/*I*_red_	*τ*_1_ [ns]	*τ*_2_ [ns]	*τ*_3_ [ns]
DMSO	1.37	0.73	2.91	9.56
DMF	1.28	0.52	2.25	7.68
CH_3_CN	0.64	0.45	2.22	7.09
THF	0.52	0.53	1.94	7.17
EtOH	0.70	0.46	2.22	7.77
MeOH	0.83	0.54	2.51	8.04

**Figure 4:**
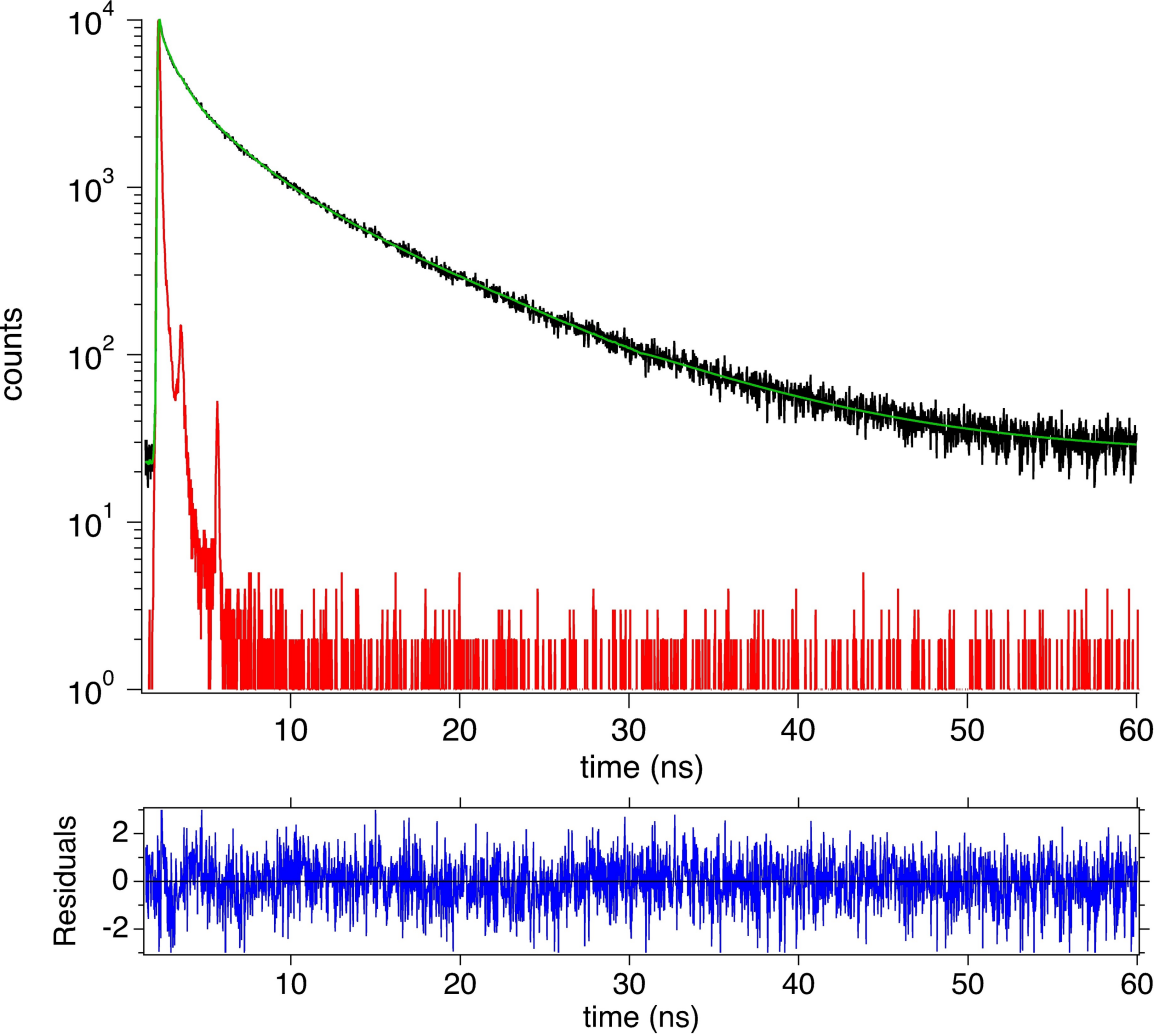
Fluorescence decay curve recorded at 620 nm in DMSO. Excitation occurred at 376 nm resulting in a *χ* ^2^ of 1.146 at this wavelength. The global analysis started to collect data between 450–720 nm where a fluorescence decay was taken every 10 nm. This resulted in a global *χ* ^2^ of 1.122.

Figure 4 depicts the emission decay taken at 620 nm. Three exponential components were necessary to fit the curve by iterative convolution. Decays were additionally taken at different wavelengths over the entire emission spectrum and linked together. A global fit of this data set resulted in three decay times as well whose amplitudes differed. Results in Table 1 show that there exists a short component of less than 1 ns, a further component of a few nanoseconds, and a long component between 7–9.5 ns. The latter mainly contributes to the long wavelength emission obtained by global analysis, see Supporting Information for more information. The use of DMSO resulted in the longest emission of the third component. Supporting Information also provides more details about the global fits obtained in different solvents. Thus, results obtained demonstrate that the surrounding affects the photophysical properties mostly for the short component *τ*_1_ while *τ*_3_ only slightly changes. This may have an influence on the sensitizing efficiency.

**Figure 5:**
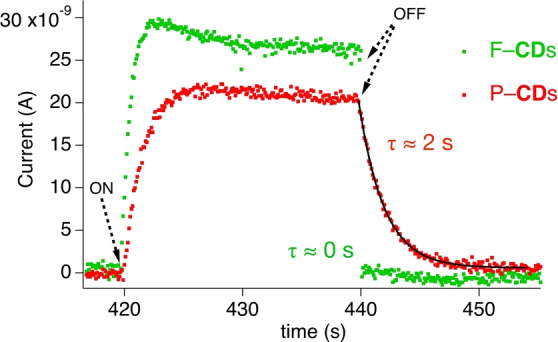
Photocurrent of P‐**CD**s and F‐**CD**s upon 405 nm LED irradiation and turning off in the dark period.

In addition, photocurrent experiments provide information about the stability of holes generated in electron transfer reactions.[^40^, ^46^] F‐**CD**s immediately switched the current to the background level after turning the source OFF. On the other hand, the hole generated in P‐**CD**s possesses with 2 s a significant longer stability in the dark period. Comparison with data of previous examined carbon dots showed different values depending on the origin.[^26^, ^27^] Here, those **CD**s originating from lac dye, alginate, and cellulose resulted in a decay of 0.5 s, 0.3, and 0.1 s, respectively, in the dark period. Interestingly, **CD**s comprising aromatic moieties showed the longest decay; that is **CD**‐1 with origin from lac dye. Thus, a charge generated can longer migrate in P‐**CD**s, which must have an impact on electron transfer reactions. Nevertheless, these data can be seen as a rough estimate. Figures 5 and S5 show the respective data obtained for photocurrent studies of lac dye, alginate and cellulose based **CD**s.

Electrochemical properties of P‐**CD**s determined by cyclic voltammetry disclose from a thermodynamic point of view how the material can participate in an initiation mechanism based on photo‐redox chemistry. P‐**CD**s exhibited an oxidation potential (*E*_ox_) of 0.58 V and reduction potential (*E*_red_) of −0.29 V (Figure S4). These data remain somehow in a range of large conjugated molecules as represented by cyanines[^47^, ^48^] and of course **CD**s derived from different natural origins.[^26^, ^27^] Porphyrin such as H_2_‐Tetraphenyl porphyrin (H_2_‐TPP) exhibits higher *E*_ox_ and much less negative *E*_red_ values.^[49]^ Thus, P‐**CD** appears different demonstrating that connection between the porphyrin and F‐**CD**s results in distinct electrochemical properties. Consequently, this must affect reactions based on **PET**.

An attempt to use P‐**CD**s as sensitizer for free radical polymerization in combination with a bis[4‐(*t*‐butyl)phenyl] iodonium tetra(nonafluoro‐*t*‐butoxy)aluminate following a previous reaction protocol^[50]^ as initiator failed using a 405 nm emitting LED (intensity: 60 mW cm^−2^). This surprised since the difference between *E*_ox_ of the P‐**CD**s and the *E*_red_ of the diaryl iodonium cation (−0.7 V^[51]^) results in a value of about 1.28 V, which is smaller as the band gap energy (*E*_00_) of the P‐**CD**s (2.18 eV, as concluded from the longest absorption band in the spectrum in Figure 3a). In other words, traditional radical photopolymerization should proceed from a thermodynamic point of view since the free reaction enthalpy of the photoinduced electron transfer (Δ*G*_el_), Equation 1, appears negative (*F*=Faraday constant, *E*_00_=excitation energy).^[52]^

(1) $$\Delta G_{el}=F\left(E_{ox}-E_{red}\right)-E_{00}$$

These findings motivated to change the initiation system. The photo‐ATRP served as fundament to study polymer formation following a previous setup.[^26^, ^27^] Here, CuBr_2_/TPMA or FeBr_3_/Br^−^ served as metal catalysts following previous studies.[^13^, ^19^, ^20^, ^22^, ^23^, ^53^] Furthermore, a metal free system based on an oxidative mechanism in the initiation step was additionally investigated.[^16^, ^18^] Ethyl α‐bromo phenylacetate (EBPA) served in all experiments as alkyl halide initiator. This compound performed well by comparison with other alkyl bromides.^[54]^

**Scheme 1:**
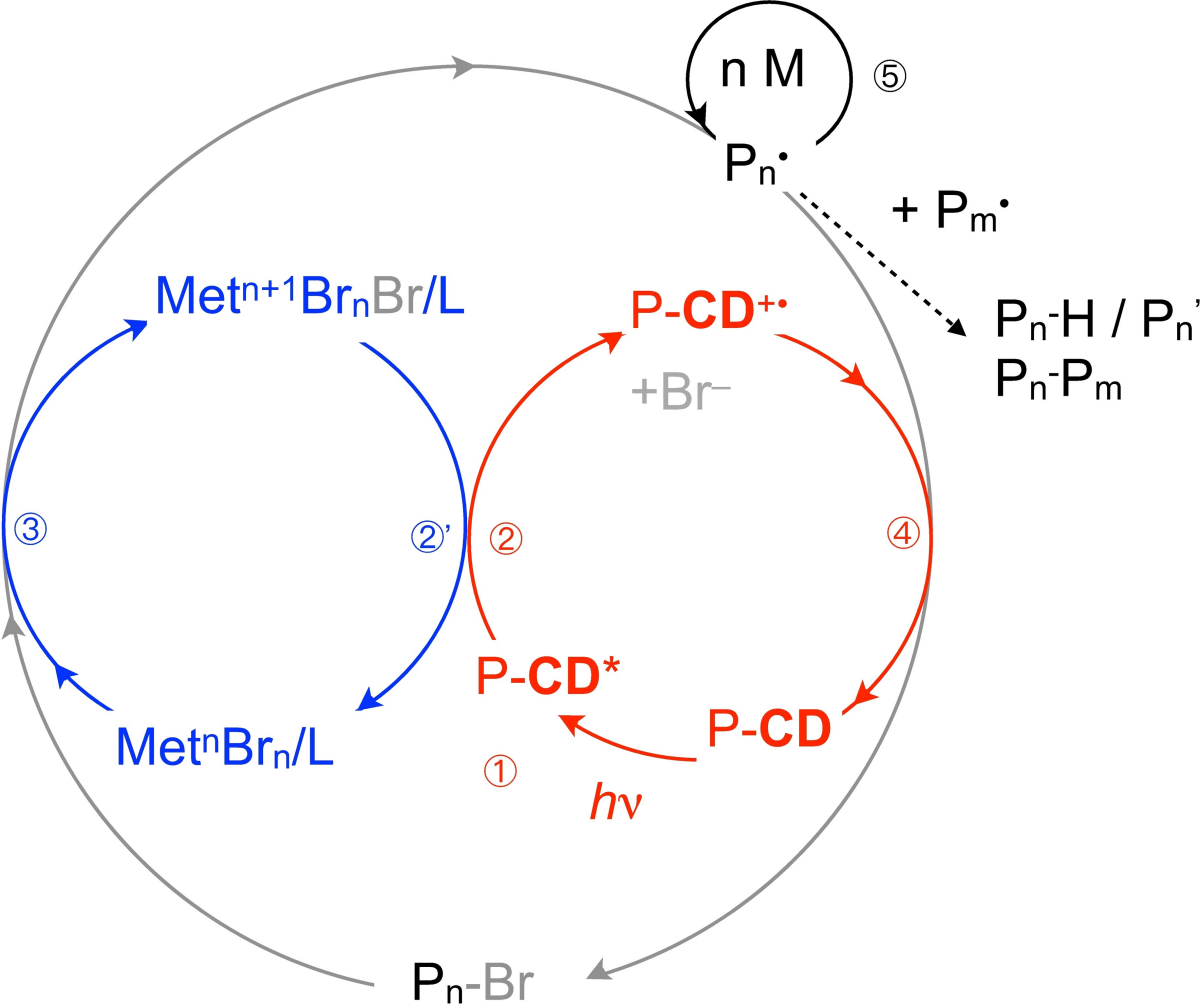
Reaction cycle for the photoinduced ATRP using P‐**CD** as light‐sensitive material excited with a 405 nm LED. The excited state P‐CD* reacts with the deactivator such as the metal halide (Met^*n*+1^Br_*n*+1_/L) in step ② resulting in formation of the activator (Met^n^Br_*n*_/L). Here, Met stands either for Cu^I/II^ or Fe^II/III^ ions. P_*n*_⋅ stands for the polymer radical formed in step ③, which can add monomer resulting in an increase of molecular weight in step ⑤ or react with Br^−^ and P‐CD^+^⋅ resulting in formation of P_*n*_‐Br and P‐**CD** in step ④. Alternatively, P_*n*_⋅ can terminate either by recombination or disproportionation resulting in formation of P_*n*_−P_*m*_ and P_*n*_−H, respectively.

Surprisingly, such systems resulted in polymer formation although their use in the aforementioned traditional radical photopolymerization attempt failed (see Table 1 below). Scheme 1 shows the occurring reactions of the system investigated from the simplest point of view. In general, the proceeding processes appear more complex compared to those disclosed previously using carbon dots as photocatalyst[^26^, ^27^] or NIR‐sensitizers.[^19^, ^20^, ^53^]

The reaction cycle depicted in Scheme 1 discloses formation of the activator (Met^*n*^Br_*n*_/L) by reaction between the deactivator (Met^*n*+1^Br_*n*+1_/L) and P‐CD* resulting in formation of the oxidized P‐**CD** species (P‐**CD^+^** ⋅) and bromide. Here, a metal ion catalyst comprising either iron or copper ions works in the ATRP cycle. Met^*n*^Br_*n*_/L activates the ATRP by reaction with the alkyl bromide (P_*n*_‐Br) resulting in formation of the deactivator (Met^*n*+1^Br_*n*+1_/L) and the polymer radical P_*n*_⋅. This can add n monomer molecules M, which leads to an increase of the average molecular weight. P_*n*_⋅ can either terminate by traditional recombination and disproportionation resulting in formation of P_*n*_−P_*m*_ and P_*n*_−H, respectively, or it can react with P‐**CD^+^** ⋅, and Br^−^ resulting in P_*n*_−Br exhibiting larger molecular weight after leaving this cycle. After this, it can enter a new ATRP cycle. Formation of Br⋅ does not appear as likely because Br⋅ and Br^−^ fast react to Br_2_⋅^−^. The latter consecutively reacts to Br^−^ and Br_3_^−^ exhibiting yellow color.[^55^, ^56^] Thus, P‐**CD^+^** ⋅, Br^−^ and P_*n*_⋅ react in a more complex mechanism resulting in back formation of P‐**CD** and P_*n*_‐Br. Copper and iron‐based systems functioned well as catalysts in such ATRP cycles.[^19^, ^20^, ^22^, ^57^] Comparative studies based on a heterogeneous photocatalyst using phenothiazine as light sensitive moiety in a network worked well using CuBr_2_/L as metal catalyst either while a metal free approach did not brought the expected success.^[58]^ Thus, the system based on P‐**CD**s behaves different explainable by the different morphology of the light sensitive heterogeneous matter of the photocatalyst.

According to Scheme 1, polymer formation shall proceed mainly when light would be ON and the reaction between Met^*n*+1^Br_*n*+1_/L and P‐CD* occurs fast. As aforementioned noticed, the reaction between P‐**CD^+^** ⋅ and bromide does not proceed as simple as shown. P‐**CD^+^** ⋅ exhibits as heterogeneous material a size of several micrometers comprising further sub‐structures with a size of several nanometers (Figure 1b). Here, distinct mesomeric structures of the radical cation can be distributed over the entire material. They can locate either on the surface or migrate into the material. In case of the latter, it should not be always available for the reactions shown in Scheme 1. The photocurrent experiments shown in Figure 5 may be a hint that particular this material can host intermediates such as radical cations much longer compared to materials previously investigated.[^26^, ^27^] Thus, P‐**CD^+^** ⋅ needs more time to react with bromide and P_*n*_⋅. The reactions mainly occur on the surface of the heterogeneous material since one would not expect significant swelling, and therefore, penetration of reaction solution inside the material. If the charge of the hole migrates much longer after turning the source off, P‐**CD** ^+^⋅ shall have a longer lifetime, and therefore, a lower access for recombination due to reduced mobility. Thus, P‐**CD** ^+^⋅ can also migrate into the material according to Equation 2 and might not be always available at the surface of the heterogeneous photocatalyst to operate in Scheme 1. In other words, P‐**CD** ^+^⋅ loses activity by formation of [P‐**CD** ^+^⋅], Equation (2). As a result, the polymer radical can add more monomer resulting in a higher molecular weight as expected because the reaction between P‐**CD^+^** ⋅, Br^−^ and P_*n*_⋅ may be slower as the reaction between the deactivator and P‐**CD**.

(2) $${P-CD}^{+\cdot }\;\;\overset{}{\underset{}{\to }}\;{[P-CD}^{+\cdot }]\;$$

Δ*G*_el_ results in negative values according to Equation (1) for the reaction with P‐CD* and Met^*n*+1^Br_*n*+1_/L. P‐**CD** possesses an oxidation potential of 0.58 V and its respective *E*_00_ energy of 2.18 eV (569 nm) results in case of reaction with Cu^II^Br_2_/TPMA, [Fe^III^Br_4_]^−^, and EBPA in negative Δ*G*_el_, Equation (1) (*E*_red_: EBPA=−0.46 V,^[59]^ [Cu/TPMA]^2+^=−0.24 V,^[60]^ FeBr_4_^−^=−0.6 V.^[61]^ It demonstrates again that photoinduced electron transfer shall proceed from a thermodynamic point of view. These steps were already discussed in previous reports where the reaction system was homogeneous and explains why polymer formation mainly proceeds when light was ON.^[3]^ However, the system introduced here possesses heterogeneous habitus of the photocatalyst, where the positive charge of P‐**CD** ^+^⋅ can migrate into the large material where it would not be always available for the reactions shown in Scheme 1. One also needs to keep in mind that **CD**s do not exhibit a defined structure as typically low molecular weight materials do. All polymers reported here were isolated by centrifugation and precipitation of the soluble parts from centrifugate. Polymer can be also grafted polymers on the surface of the P‐**CD**. NMR spectrum of the material isolated after polymerization by centrifugation supports this hypothesis, see Figure S6. It showed remarkable amount of structures in the sample related to PMMA even after three times washing with THF. This can lead to circumstances that grafted polymer around the particle may deactivate the surface after turning off the light source. Thus, the polymer would surround/encapsulate the particles surface whose fine structure as shown in Figure 1c could not contribute to activation of the ATRP. Once deactivation completed, the photocatalytic system could not be reactivated after turning ON the light source. Furthermore, the addition of surfactant such as sodium dodecylsulfate (SDS) did not brought any effort to solve this issue (see details in Supporting Information). Thus, the addition of SDS even led to opposite conditions resulting in a deactivation of the active surface of the **P**‐**CD**. Nevertheless, there exists potential to succeed by selective functionalization of the surface. Future work might focus to work out conditions where functionalization keeps the surface in better conditions.

**Scheme 2:**
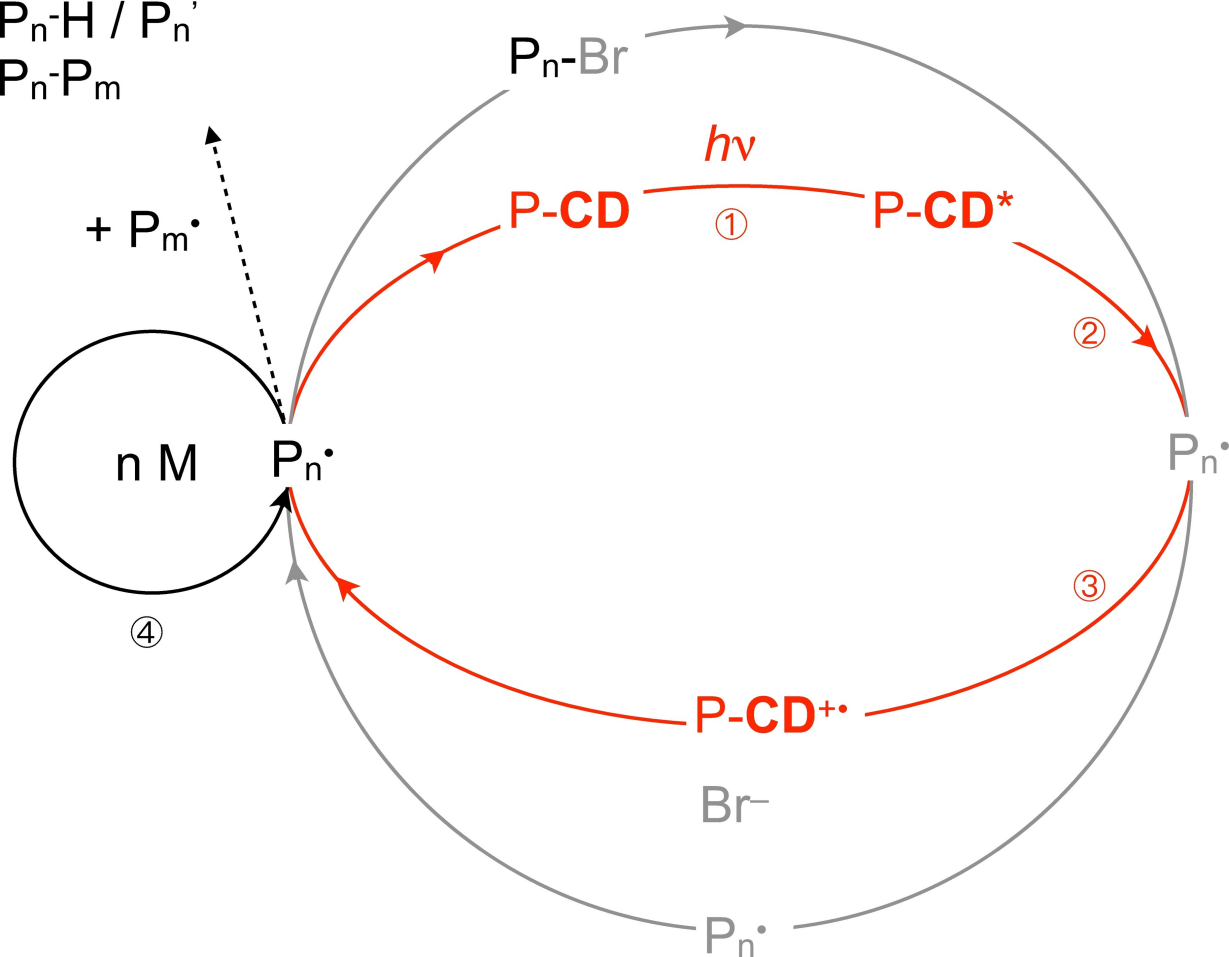
Scheme for the photoinduced ATRP using P‐**CD** as light‐sensitive material excited with a 405 nm LED in an oxidative metal‐free approach. The excited state P‐CD* reacts with P_*n*_−Br in step ② resulting in formation of P_*n*_⋅. P_*n*_⋅ can either add monomer resulting in an increase of molecular weight as shown in step ⑤ before it reacts with Br^−^ and P−CD^+^⋅ resulting in formation of P_*n*_−Br and P‐**CD** in step ④. Alternatively, P_*n*_⋅ can terminate either by recombination or disproportionation resulting in formation of P_*n*_−P_*m*_ and P_*n*_−H, respectively.

Alternatively, photopolymerization also proceeds according to a metal‐free approach, see Scheme 2.[^16^, ^18^] Here, reaction between P‐CD* and P_*n*_−Br results in formation of the respective hole P‐**CD^+^** ⋅, Br^−^ and P_*n*_⋅. The latter adds n monomers resulting in an increase of molecular weight. The availability of P‐**CD^+^** ⋅ for the back formation by reaction between P‐**CD** also depends on the stability of P‐**CD^+^** ⋅ as discussed above. Again, the reaction between the alkyl halide initiator and P‐CD* should also occur from a thermodynamic point of view according to Equation (1). Furthermore, it cannot be excluded that both mechanisms shown in Schemes 1 and 2 proceed competitively.

**Scheme 3:**
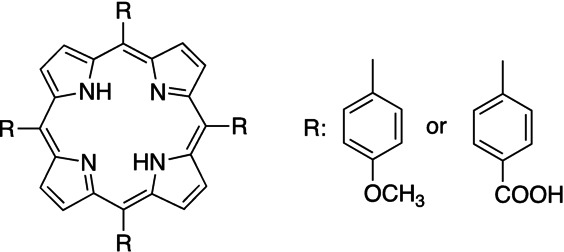
Molecular structure of porphyrins **P‐CAR** and **P‐Met**.^[44]^

P‐**CD**s exhibited a good efficiency in the photoinitiated ATRP with (Cu/TPMA)Br_2_ as catalyst. For comparison, polymers were also obtained upon 405 nm LED exposure using either the porphyrins P‐**CAr** (4,4′,4′′,4′′′‐(Porphyrin‐5,10,15,20‐tetrayl) tetrakis (benzoic acid)) and P‐**Met** (5,10,15,20‐Tetrakis(4‐methoxyphenyl)‐21*H*, 23*H*‐porphyrin), see Scheme 3 for structural details. The photo‐ATRP pursued resulted in polymers exhibiting a *Đ* close to 1.1 but a much lower yield compared to P‐**CD**, see entries 1 and 2 in Table 2. **MMA** as monomer resulted in a dispersity of the molecular weight close the 1.1, which was similar for polymers obtained using P‐**CD** (entry 3 of Table 2). Change of the monomer to styrene (**St**) also gave polymers with low dispersity but the molecular weight was significantly lower (see entry 5 in Table 2). The metal‐free system in entry 4 of Table 2 also worked but the dispersity was larger compared to entry 3 in Table 2. As mentioned above, the heterogeneous nature of the photocatalyst causes a longer lifetime of P‐**CD** ^+^⋅ formed, which would be for the ATRP only available at the surface. Consequently, the molecular weight growths faster but in the same way also the probability of side reactions such as recombination/disproportionation increases explaining the higher dispersity and molecular weight in the metal free system. Formation of polymers under different conditions can also explain a higher dispersity of molecular weight.^[62]^ From a practical point of view, a higher dispersity would not in general limit the use of a block copolymer as long it works in inks to stabilize pigments, medical applications such as manufacturing of bone implanters or fabrication of tissue related materials.

**Table 2:** Photoinduced ATRP of MMA under different conditions as shown for the average number of molecular weight *M*n and dispersity *Đ*.^[a]^

	Sens	[MMA] : [I] : [Met] : [TPMA]	**M**	MeBr_*n*_	*x* ^[b]^ [%]	*M*_n_^[c]^ [kDa]	*Đ*
1	P‐**Ar**	300 : 1 : 0.03 : 0.135	**MMA**	CuBr_2_	9	13.7	1.11
2	P‐**Me**	300 : 1 : 0.03 : 0.135	**MMA**	CuBr_2_	4	11.3	1.14
3	P‐**CD**	300 : 1 : 0.03 : 0.135	**MMA**	CuBr_2_	46	23.5	1.12
4	P‐**CD**	300 : 1 : 0 : 0	**MMA**	none	25	39.3	1.63
5	P‐**CD**	300 : 1 : 0.03 : 0.135	**styrene**	CuBr_2_	2	1.85	1.10
6	P‐**CD**	300 : 1 : 0.015 : 0.0625	**MMA**	CuBr_2_	6	12.8	1.11
7	P‐**CD**	300 : 1 : 0.06 : 0.27	**MMA**	CuBr_2_	6	11.6	1.10
8	P‐**CD**	100 : 1 : 0.04 : 0.04	**MMA**	FeBr_3_	37	55.8	1.97
9	P‐**CD**	100 : 1 : 0.02 : 0.02	**MMA**	FeBr_3_	4	36.6	1.58
10	P‐**CD**	100 : 1 : 0.08 : 0.08	**MMA**	FeBr_3_	12	33.2	1.62
11	P‐**CD**	100 : 1 : 0 : 0	**MMA**	none	25	88.4	1.82

[a] Polymerization experiments were conducted in 75 % DMSO : monomer=75 : 25 (vol %), m_P‐CDs_=3 mg and irradiated under 405 nm LED exposure (60 mW cm^−2^). [b] Conversion (*x*) determined gravimetrically. [c] Number average molecular weight determined by gel permeation chromatography using PMMA standards.

The conversion obtained was significant higher (46 %) compared to those **CD**s investigated previously.[^26^, ^27^] Thus, P‐**CD**s possess a higher capability to use light in a photo‐ATRP setup. Thus, the combination of porphyrin and furfural‐based **CD**s (P‐**CD**s) appeared to work more efficient regarding the conversion while the dispersity of molecular weight showed similar value. P‐**CD**s would be suitable to make polymers with low dispersity while conversion exceeds higher in comparison with the porphyrins **P‐CAR** and **P‐Met**.

Change of the monomer from **MMA** to styrene (**St**) results in less conversion while *Đ* remains similarly indicating nearly no effect on molecular weight distribution. Nevertheless, both monomer conversion and *M*_n_ drop. Thus, **St** does not appear as reactive as MMA in the system investigated agreeing with kinetic constants disclosed for such systems.[^15^, ^63^] In addition, decrease of catalyst concentration (CuBr_2_ and TPMA) also showed the same direction for changes of *Đ* and *M*_n_, see Entries 5 and 6 in Table 2. Moreover, photo‐ATRP following a metal‐free approach resulted in polymer formation, entry 4 in Table 2. Higher *M*_n_ and *Đ* demonstrated that polymerization under these conditions gave material with less uniformity of molecular weight distribution.

Environmental and sustainability aspects motivated to change the copper catalyst by an iron‐based catalyst needing no addition of amine. Addition of tetrabutyl ammonium bromide resulted in the necessary compatibility of FeBr_3_ with the surrounding used, entries 8–11 in Table 2. Polymerization was carried out at higher catalyst concentration and initiator concentration with no change of solvent. The lower reactivity of the iron complex explains the change of concentration.^[64]^ The systems investigated resulted in polymers exhibiting a much higher *M*_n_ and *Đ* compared to the photo‐ATRP systems comprising CuBr_2_/TPMA.

**Figure 6:**
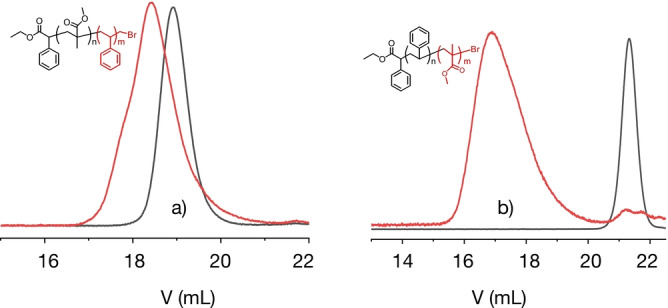
GPC chromatograms of block copolymers obtained in slide a) for **PMMA**‐*b*‐**PS** with **PMMA**‐Br as macroinitiator reacting with styrene and b) **PS**‐ *b*‐**PMMA** with **PS**‐Br as macroinitiator reacting with MMA ([monomer]:[P_*n*_‐Br] : [CuBr_2_] : [TPMA]=300 : 1 : 0.03 : 0.135; a: P_*n*_‐Br=23.5 kDa, *Đ*=1.1, **PMMA**‐*b*‐**PSt**: *M*_n_=31 kDa, *Đ*=1.4; b: P_*n*_‐Br=1.85 kDa, *Đ*=1.1 **PSt**‐*b*‐**PMMA**: *M*_n_=8.9 kDa, *Đ*=1.6).

To verify the end‐group fidelity, the **PMMA**‐Br and **PS**‐Br of entries 3 and 5 in Table 2, respectively, were used as macroinitiator for block copolymerization. Addition of the second monomer should result in formation of a block copolymer if the fidelity of the end group facilitates chain extension by a second monomer. Figure 6a shows successful block copolymerization with styrene in the case that **PMMA** operated as macroinitiator. It exhibited a *M*_n_ of 30.9 kDa. Thus, the polymerization degree ${\bar{P}}_{n}$ of the **PS** block was 71 while that of the macroinitiator exhibited a ${\bar{P}}_{n}$ of 234. Obviously, styrene results in a higher ${\bar{P}}_{n}$ if the initiating radical belongs to the **PMMA**‐Br macroinitiator while formation of **PS**‐Br macroinitiator yielded significant lower ${\bar{P}}_{n}$ ; Use of the **PS** macroinitiator of entry 5 gave **PS**‐*b*‐**PMMA**, where **PMMA** exhibited a ${\bar{P}}_{n}$ of 880. These data show that origin of the preparation of the first block affects the molecular weight of the second block. **PMMA** possesses a 10 times higher propagation rate constant compared to **PS** explaining the differences of molecular weight between both polymers.^[63]^ Somehow, the P‐**CD** must have an impact on ${\bar{P}}_{n}$ particularly in the case of styrene polymerization. The data shown above exhibit a shorter chain length if the polystyrene chain grows in the first step. In addition, block copolymerization results in higher dispersity of the block copolymer; that is *Đ*=1.4 and *Đ*=1.6 in the case of **PMMA**‐*b*‐**PS** and **PS**‐*b*‐**PMMA**, respectively. Different activity of the heterogeneous surface of the photocatalyst may explain the higher dispersity of molecular weight. Thus, the block‐copolymerization experiment more sensitively images these differences in case that styrene operated as second monomer. It typically growth slower then **MMA** and appears in this experiment to have a better selectivity to image these differences as shown in Figure 6a by the shoulder visible at elution volume of about 17.5 mL. A change of the order of the monomer used gave a different pattern. The dispersity was broader when MMA operated as second monomer, which growths faster then styrene. Here, side reactions such as recombination and disproportionation can competitively proceed resulting in higher dispersity of molecular weight. Also, photocurrent experiments showed a much longer lifetime of charged species migrating through the heterogeneous photocatalyst. Thus, not every species, i.e. P‐**CD** ^+^⋅, formed will be available in the photo‐ATRP cycle to bring back P_*n*_‐Br. It therefore also favors termination by free radical polymerization and thus explains the higher dispersity.

In addition, chain extension experiments of the **PMMA** obtained for the metal free (entry 11) and Fe‐catalyzed system (entry 8) also resulted in an increase of molecular weight. The first block of **PMMA** in entry 5 showed a polymerization degree of 88 (*M*_n_=88.4 kDa), which extended to 130 (*M*_n_=130 kDa) in the chain extended polymer. Dispersity increased to 2.2. This material may comprise some polymer with no capability of chain extension. Although the molecular weight of the macroinitiator was significantly larger, overall chain extension was successful. The chain extension experiments of the macroinitiator made by an iron catalyst also successfully worked. The molecular weight of the **PMMA** macroinitiator increased from 55.8 kDa to 93 kDa corresponding to a ${\bar{P}}_{n}$ of 55.7 and 37 for the **PMMA** macroinitiator and the chain extended polymer, respectively. The dispersity of the latter was 1.78 showing a small decrease of this quantity. Thus, the use of FeBr_3_/Br^−^ does not really affect dispersity in the chain extension experiment while it does in the metal free system.

Temporal studies operating the LED in the ON/OFF mode failed. Polymer was formed after exposure of 12 h resulting in a conversion of 23 % while the dark period showed no polymerization, see Figure S7. Nevertheless, no polymer was formed in the next cycle. This repeated in the third cycle as well. These results surprised since homogeneous systems and those comprising much smaller particles as light sensitive component behaved differently in previous studies with excellent temporal control in ON/OFF cycles applying either visible[^17^, ^18^] or NIR light excitation.^[19]^ There exist from our best knowledge only one study where a heterogeneous catalyst was applied for successful temporal controlled polymerization experiments.^[58]^ Here, covalently bound phenothiazine operated as sensitizer in a network. It requested to work in combination with CuBr_2_/L while the metal‐free approach failed.^[58]^ Such a material can certainly uptake reaction components by swelling resulting in a better availability of reactive centers of the crosslinked material also inside of the material. However, the sustainable carbon dots connected by porphyrin moieties shall not behave in this way. Upon exposure, polymerization proceeds at available reactive centers at the surface. Turning off the light source may lead to the scenario that the photocatalyst can be surrounded by polymer formed resulting in deactivation of reactive centers of the heterogeneous photocatalyst. ^1^H NMR measurements (Figure S6) gave a good hint that the heterogeneous catalyst, which was separated after exposure, was surrounded somehow by poly(methyl methacrylate) resulting in deactivation of reactive sites at the surface of the photocatalyst (see above). Thus, these parts may be not anymore available for photopolymerization after turning ON the light again.

Nevertheless, chain extension successfully succeeded as shown by block copolymerization, which requested to stop the polymerization procedure and to isolate the macroinitiator. More competitive studies would be necessary to understand the reactivity of such heterogeneous systems that show depending on size of the reactive group different behavior in the controlled polymerization setup. These are several Angstrom in case of the phenothiazine system^[58]^ while the sustainable photocatalyst **P‐CD** exhibited significant larger size. By comparison, the system comprising phenothiazine in the network should possess more reactive sites. Studies have been in progress in our lab using alternative heterogeneous photocatalysts exhibiting long afterglow room‐temperature phosphorescence.^[28]^ Here, recent lab results showed successful chain extension while temporal control of such heterogeneous systems failed again. Results obtained will be published elsewhere.

## Conclusion

Sustainable carbon dots where porphyrin moieties connected sustainable carbon dots derived from furfuraldehyde resulted in polymer formation following an ATRP reaction protocol where CuBr_2_ or FeBr_3_ functioned as metal catalyst. Polymer formation also proceeded according to a metal free photo‐ATRP approach applying a 405 nm LED excitation. The heterogeneous nature of the **CD**s connected by porphyrin brought new insight in this field because chain extension worked on the one hand while temporal control applying ON/OFF experiments failed for P‐**CD**s. This will have some consequences for future design of similar systems.

Although the sustainable **CD**s connected by porphyrin possess interesting electrochemical and photonic properties, the direct connection of the porphyrin moiety with the **CD** resulted in a scenario that less active centers would be available at the surface of the photocatalyst. Here, alternative solutions must be worked out in the future to increase this ratio. Perhaps the introduction of additional structural elements facilitating a better uptake of reaction solution would be a possible alternative.

In addition, the heterogeneous appearance will work out new challenges because the easy removal of the catalyst from the reaction mixture can be seen as one benefit. Future work may focus on alternative materials exhibiting higher accessible surface for the polymerization reaction. This may bring such materials also back to traditional radical photopolymerization studies using onium salts as co‐initiator.

## Experimental Section

Supporting Information gives additional details regarding the instrumentation, materials, photopolymerization and P‐**CD** synthesis. TEM measurements, XPS, time resolved fluorescence experiments, photocurrent studies, FTIR, UV/Vis, NMR, photo current studies, and cyclic voltammetry are discussed in more detail. Supporting Information also provides original data of time resolved fluorescence measurements.

## Conflict of interest

The authors declare no conflict of interest.

1

## Supporting information

As a service to our authors and readers, this journal provides supporting information supplied by the authors. Such materials are peer reviewed and may be re‐organized for online delivery, but are not copy‐edited or typeset. Technical support issues arising from supporting information (other than missing files) should be addressed to the authors.

Supporting Information

Supporting Information

Supporting Information

Supporting Information

Supporting Information

Supporting Information

Supporting Information

Supporting Information

## Data Availability

The data that support the findings of this study are available from the corresponding author upon reasonable request.
