# Supplementary material for: A Porphyrin‐Based Organic Network Comprising Sustainable Carbon Dots for Photopolymerization
Source: Angew Chem Int Ed Engl. 2022 Sep 1;61(40):e202208180. doi: 10.1002/anie.202208180 (PMC9826160; doi:10.1002/anie.202208180)
Supplement: Supplementary file 1 — Supporting Information [file ANIE-61-0-s007.pdf]

EasyTau Report

3/11/2022 7:09:02 PM
F:\Paper Xiongfei\CD-1 CH3CN tau global.html

Container Curves

TRES+IRF_20201222_1747.etc: crv[0 - 11]

Container Properties

Measurement Context: Time Resolved Emission Scan
Detection Wavelength: 450.0 - 650.0 (Step: 20.0) nm
Summary:

Sample:	CD_X_1
Solvent:	Acetonitrile
Excitation:	V pol 376±1nm with LDH-P-C-375
Detection:	M pol 450...650nm 20nm steps<br>10000 peak counts<br>grating 1200/500-<br>detector UV-red [PMT]

Comment

Model: Exponential [Reconv.]

$$\mathrm{Dec}(t)=\left[\int_{-\infty}^{t} dt' \left[\mathrm{IRF}(t-Shift_{IRF})-Bkgr_{IRF}\right]\left[\sum_{i=1}^{n_{Exp}} A_i\, e^{-\frac{t-t'}{\tau_i}} + A_{Scatt}\,\delta(t-t')\right]\right] + Bkgr_{Dec}$$

$$I_m = A_m \tau_m$$

$$A_{Rel\,m} = A_m \Big/ \sum_{\substack{i=1\\A_i>0}}^{n_{Exp}} A_i \qquad I_{Rel\,m} = I_m \Big/ \sum_{\substack{i=1\\I_i>0}}^{n_{Exp}} I_i$$

$$\tau_{Av\,Amp} = \sum_{\substack{i=1\\A_i>0}}^{n_{Exp}} A_i\tau_i \Big/ \sum_{\substack{i=1\\A_i>0}}^{n_{Exp}} A_i \qquad \tau_{Av\,Int} = \sum_{\substack{i=1\\I_i>0}}^{n_{Exp}} I_i\tau_i \Big/ \sum_{\substack{i=1\\I_i>0}}^{n_{Exp}} I_i$$

Data Set: 1 / 11

Decay: crv[1]; IRF: crv[0]

Fit

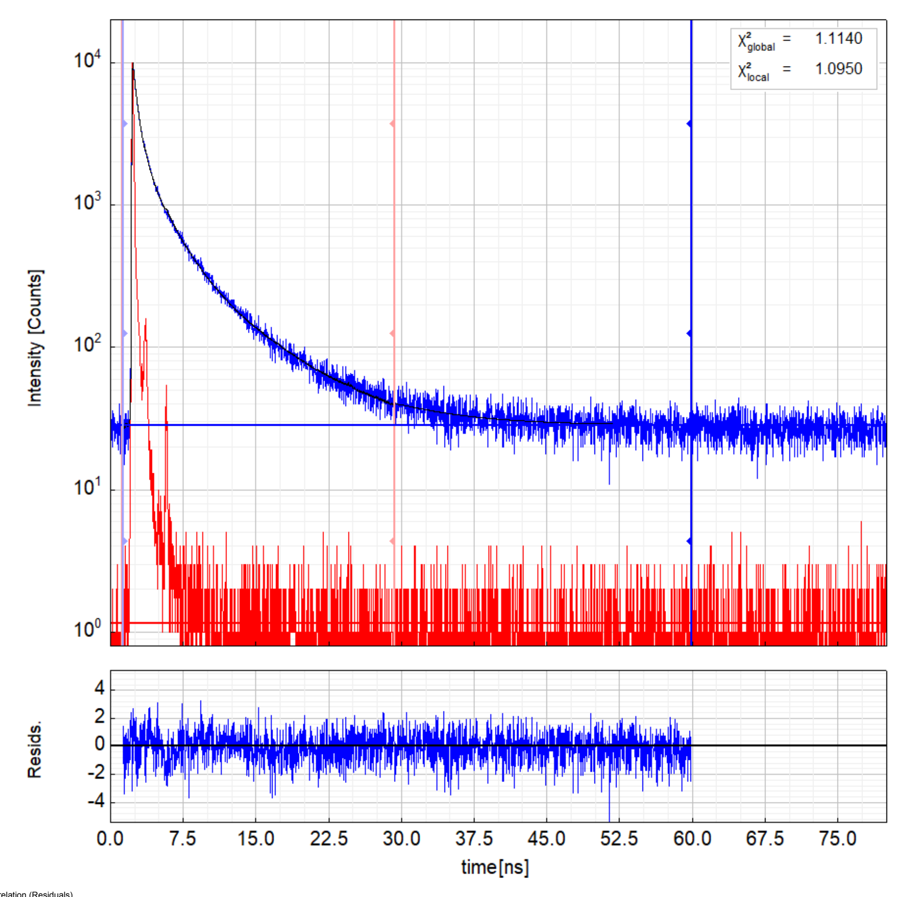

Autocorrelation (Residuals)

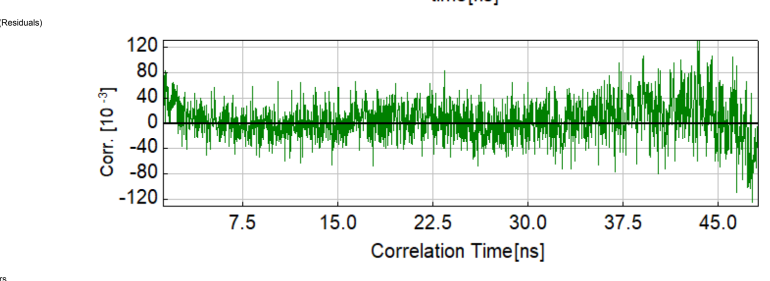

Fitted Parameters

Parameter	Value	Δ	δ
A_1[kCnts/Chnl]	2.279	±0.019	0.8%
τ_1[ns]	2.215	±0.035	1.6%
I_1[kCnts]	201.9	±2.1	1.0%
A_2[kCnts/Chnl]	8.92	±0.12	1.3%
τ_2[ns]	0.4455	±0.0055	1.2%
I_2[kCnts]	158.8	±1.7	1.0%
A_3[kCnts/Chnl]	0.613	±0.011	1.6%
τ_3[ns]	7.088	±0.041	0.6%
I_3[kCnts]	173.7	±2.3	1.3%
$Bkgr_{Dec}$[kCnts]	0.0285	±0.0002	0.6%
$Bkgr_{IRF}$[Cnts/Chnl]	1.147	±0.083	7.2%
$Shift_{IRF}$[ps]	2.4	±1.8	74%
A_{Scatt}[kCnts]	40.5	±1.2	2.9%
τ_{AvInt}[ns]	3.273	±0.015	0.4%
τ_{AvAmp}[ns]	1.1318	±0.0072	0.6%

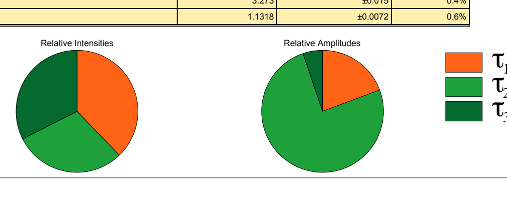

Data Set: 2 / 11

Decay: crv[2]; IRF: crv[0]

Fit

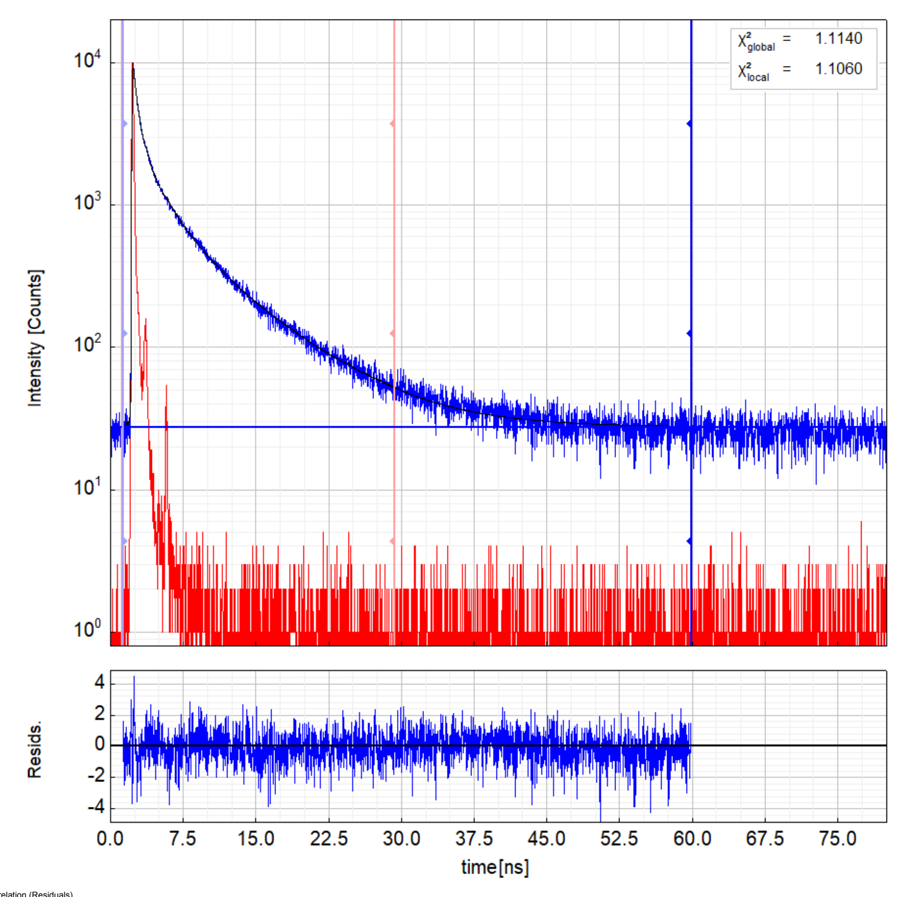

Autocorrelation (Residuals)

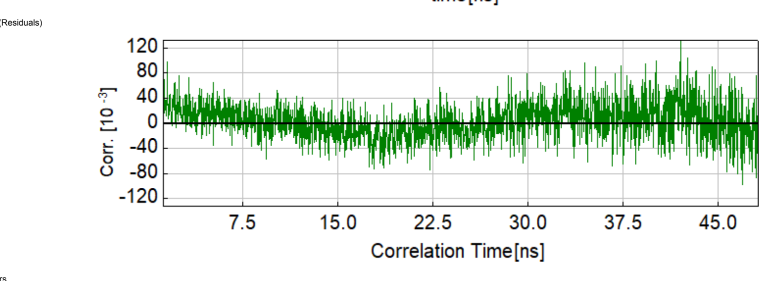

Fitted Parameters

Parameter	Value	Δ	δ
A_1[kCnts/Chnl]	2.051	±0.026	1.2%
τ_1[ns]	2.215	±0.035	1.6%
I_1[kCnts]	181.7	±1.6	0.8%
A_2[kCnts/Chnl]	8.63	±0.12	1.4%
τ_2[ns]	0.4455	±0.0055	1.2%
I_2[kCnts]	153.7	±1.9	1.2%
A_3[kCnts/Chnl]	1.018	±0.013	1.3%
τ_3[ns]	7.088	±0.041	0.6%
I_3[kCnts]	288.4	±2.0	0.7%
$Bkgr_{Dec}$[kCnts]	0.0277	±0.0002	0.7%
$Bkgr_{IRF}$[Cnts/Chnl]	0.49	±0.11	22%
$Shift_{IRF}$[ps]	6.63	±0.91	14%
A_{Scatt}[kCnts]	41.1	±1.2	2.9%
τ_{AvInt}[ns]	4.032	±0.023	0.6%
τ_{AvAmp}[ns]	1.3334	±0.0076	0.6%

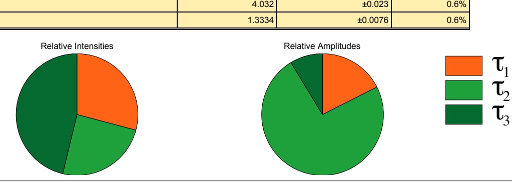

Data Set: 3 / 11

Decay: crv[3]; IRF: crv[0]

Fit

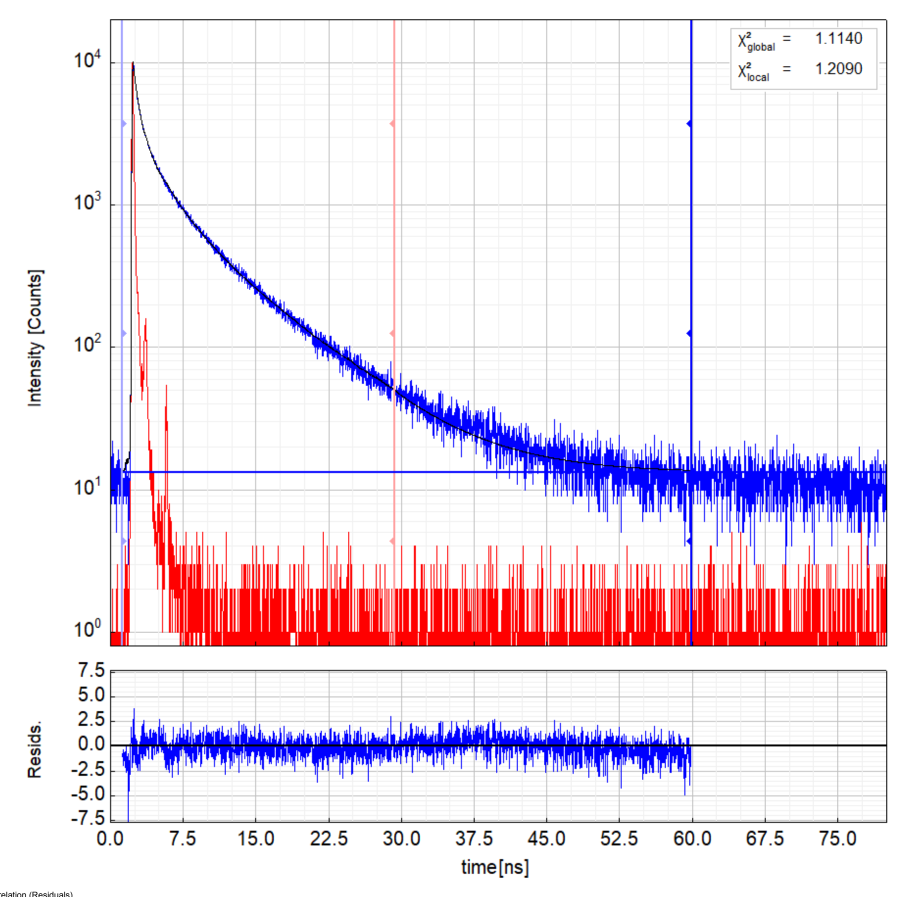

Autocorrelation (Residuals)

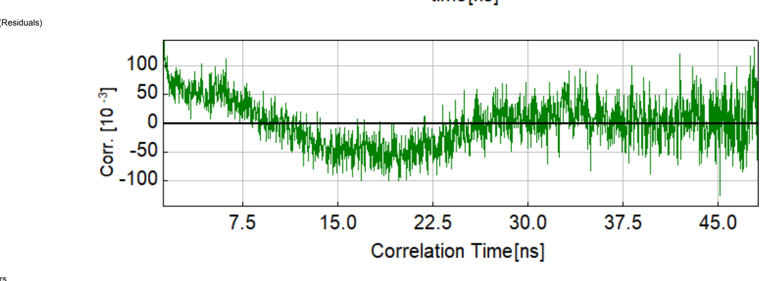

Fitted Parameters

Parameter	Value	Δ	δ
A_1[kCnts/Chnl]	2.453	±0.035	1.4%
τ_1[ns]	2.215	±0.035	1.6%
I_1[kCnts]	217.26	±0.92	0.4%
A_2[kCnts/Chnl]	8.332	±0.081	1.0%
τ_2[ns]	0.4455	±0.0055	1.2%
I_2[kCnts]	148.5	±2.4	1.6%
A_3[kCnts/Chnl]	1.382	±0.014	1.0%
τ_3[ns]	7.088	±0.041	0.6%
I_3[kCnts]	391.6	±2.5	0.6%
$Bkgr_{Dec}$[kCnts]	0.0133	±0.0002	1.0%
$Bkgr_{IRF}$[Cnts/Chnl]	0.03	±0.13	483%
$Shift_{IRF}$[ps]	6.6	±1.1	16%
A_{Scatt}[kCnts]	38.91	±0.73	1.9%
τ_{AvInt}[ns]	4.388	±0.018	0.4%
τ_{AvAmp}[ns]	1.5562	±0.0099	0.6%

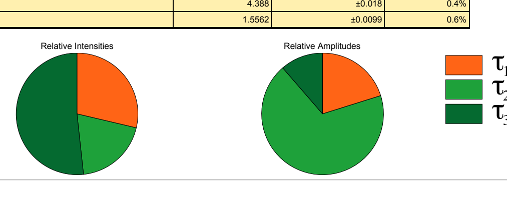

Data Set: 4 / 11

Decay: crv[4]; IRF: crv[0]

Fit

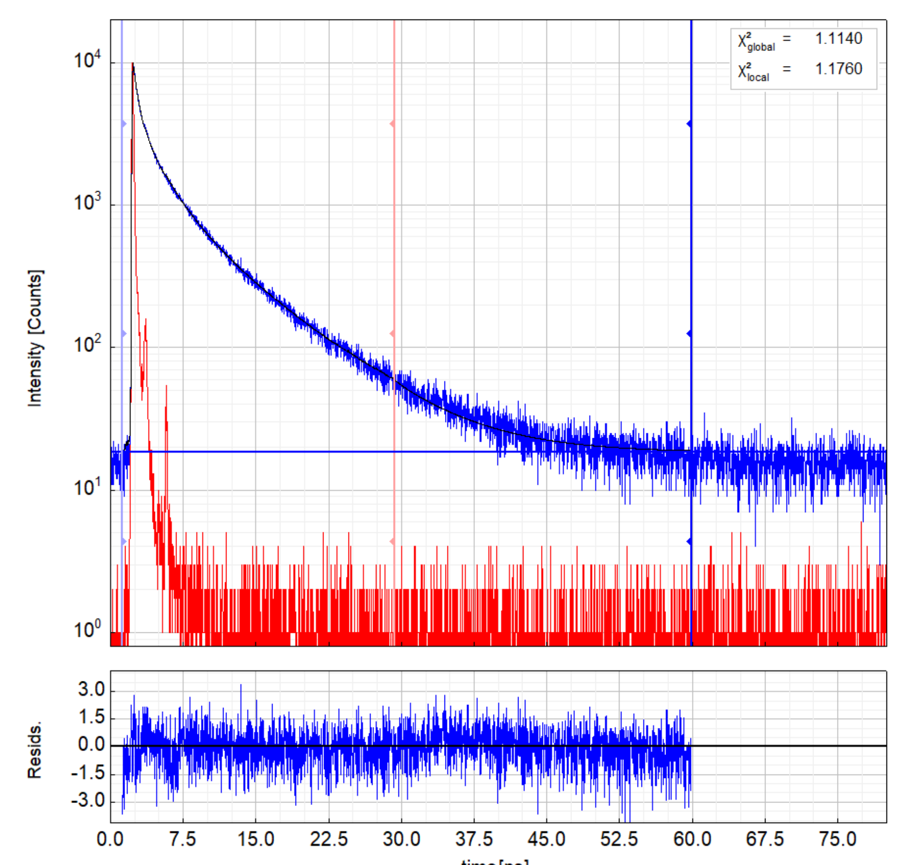

Autocorrelation (Residuals)

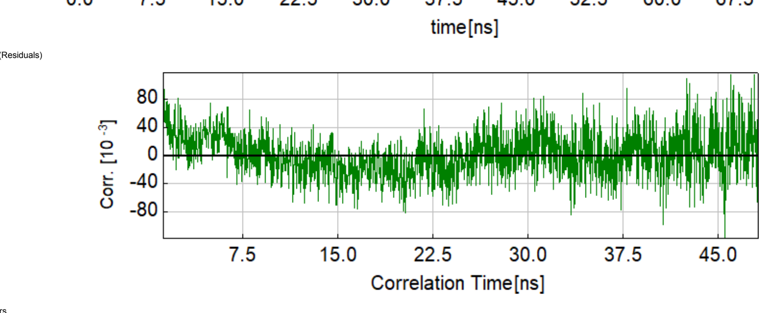

Fitted Parameters

Parameter	Value	Δ	δ
A_1[kCnts/Chnl]	2.950	±0.044	1.5%
τ_1[ns]	2.215	±0.035	1.6%
I_1[kCnts]	261.3	±1.7	0.6%
A_2[kCnts/Chnl]	7.735	±0.066	0.9%
τ_2[ns]	0.4455	±0.0055	1.2%
I_2[kCnts]	137.8	±2.9	2.1%
A_3[kCnts/Chnl]	1.481	±0.020	1.3%
τ_3[ns]	7.088	±0.041	0.6%
I_3[kCnts]	419.7	±2.4	0.6%
$Bkgr_{Dec}$[kCnts]	0.0184	±0.0002	0.9%
$Bkgr_{IRF}$[Cnts/Chnl]	-0.092	±0.097	105%
$Shift_{IRF}$[ps]	6.70	±0.83	12%
A_{Scatt}[kCnts]	37.44	±0.97	2.6%
τ_{AvInt}[ns]	4.415	±0.019	0.4%
τ_{AvAmp}[ns]	1.6826	±0.0086	0.5%

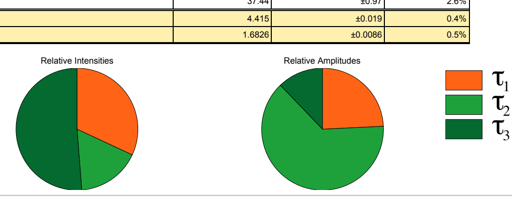

Data Set: 5 / 11

Decay: crv[5]; IRF: crv[0]

Fit

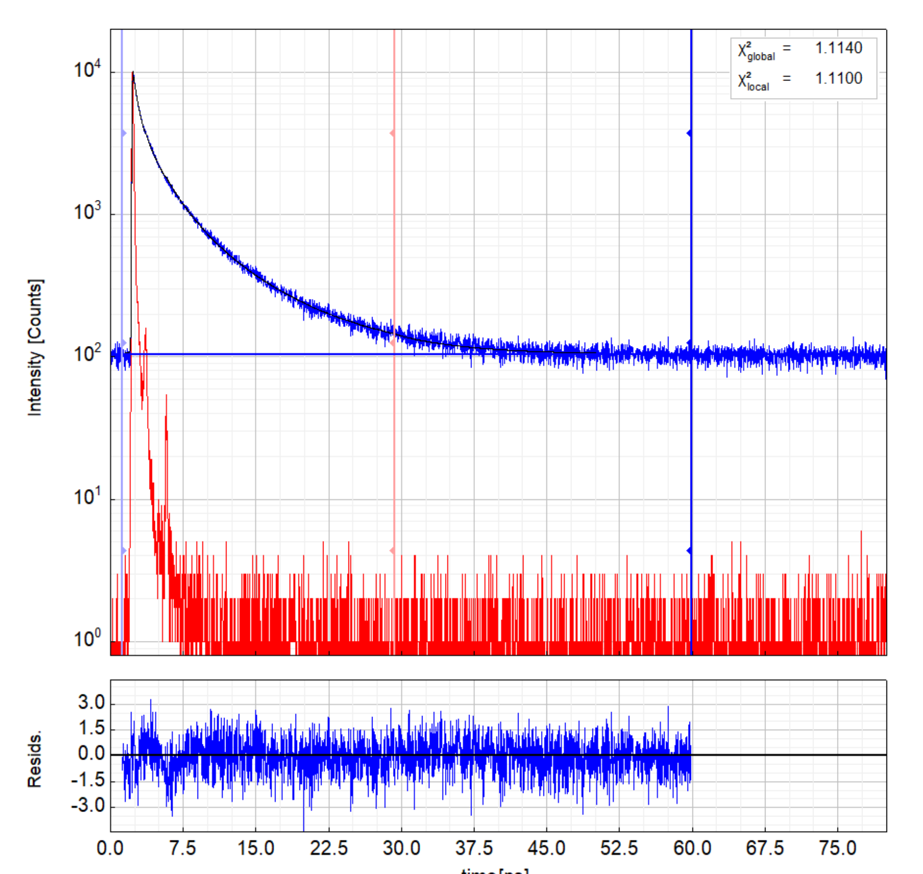

Autocorrelation (Residuals)

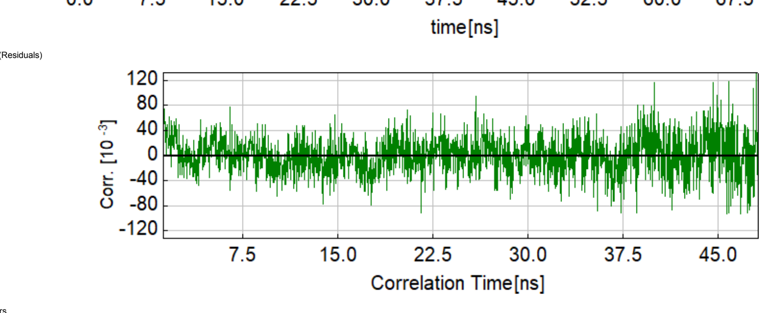

Fitted Parameters

Parameter	Value	Δ	δ
A_1[kCnts/Chnl]	3.533	±0.030	0.8%
τ_1[ns]	2.215	±0.035	1.6%
I_1[kCnts]	313.0	±1.5	0.5%
A_2[kCnts/Chnl]	7.027	±0.069	1.0%
τ_2[ns]	0.4455	±0.0055	1.2%
I_2[kCnts]	125.2	±1.7	1.3%
A_3[kCnts/Chnl]	1.485	±0.023	1.5%
τ_3[ns]	7.088	±0.041	0.6%
I_3[kCnts]	421.0	±4.1	1.0%
$Bkgr_{Dec}$[kCnts]	0.1043	±0.0004	0.3%
$Bkgr_{IRF}$[Cnts/Chnl]	-0.019	±0.057	308%
$Shift_{IRF}$[ps]	8.46	±0.64	7.5%
A_{Scatt}[kCnts]	37.31	±0.47	1.3%
τ_{AvInt}[ns]	4.345	±0.015	0.3%
τ_{AvAmp}[ns]	1.7832	±0.0056	0.3%

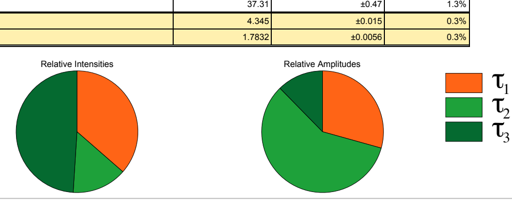

Data Set: 6 / 11

Decay: crv[6]; IRF: crv[0]

Fit

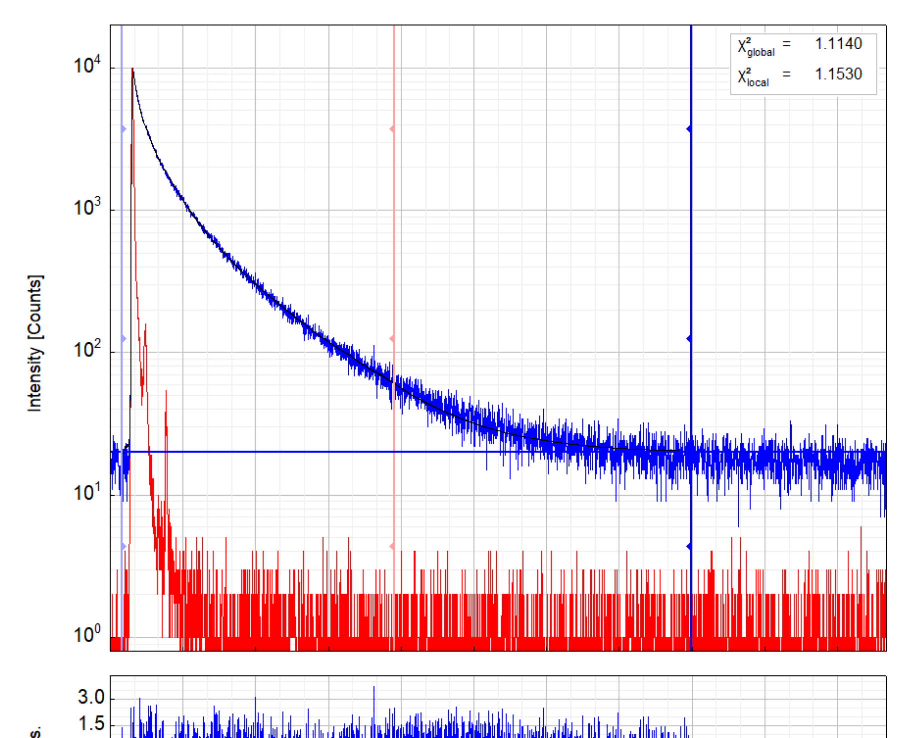

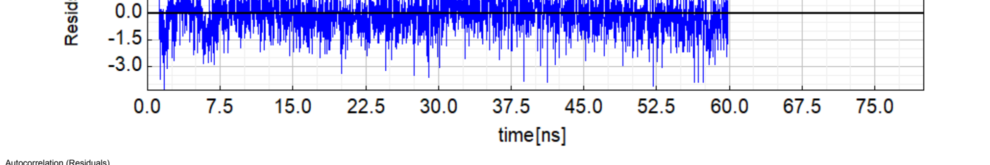

Autocorrelation Residuals

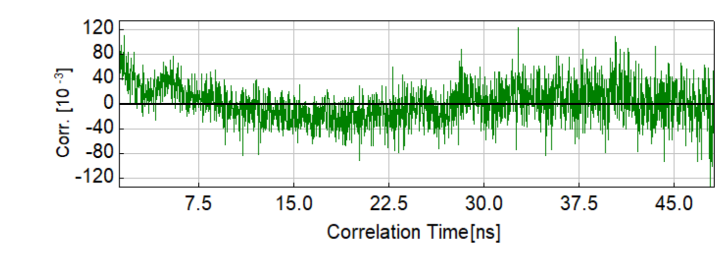

Fitted Parameters

Parameter	Value	Δ	δ
A_1[kCnts/Chnl]	3.948	±0.034	0.9%
τ_1[ns]	2.215	±0.035	1.6%
I_1[kCnts]	349.7	±1.5	0.4%
A_2[kCnts/Chnl]	6.414	±0.072	1.1%
τ_2[ns]	0.4455	±0.0055	1.2%
I_2[kCnts]	114.3	±2.0	1.7%
A_3[kCnts/Chnl]	1.566	±0.022	1.4%
τ_3[ns]	7.088	±0.041	0.6%
I_3[kCnts]	444.0	±3.4	0.8%
$Bkgr_{Dec}$[kCnts]	0.0199	±0.0003	1.1%
$Bkgr_{IRF}$[Cnts/Chnl]	0.187	±0.058	31%
$Shift_{IRF}$[ps]	9.63	±0.57	5.9%
A_{Scatt}[kCnts]	38.00	±0.63	1.6%
τ_{AvInt}[ns]	4.375	±0.012	0.3%
τ_{AvAmp}[ns]	1.9031	±0.0093	0.5%

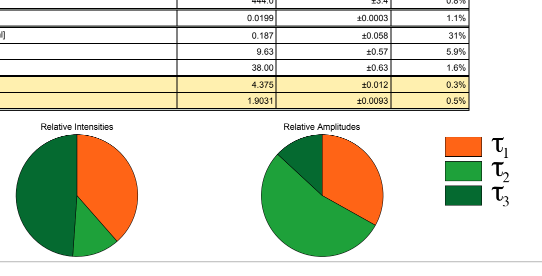

Data Set: 7 / 11

Decay: crv[7]; IRF: crv[0]

Fit

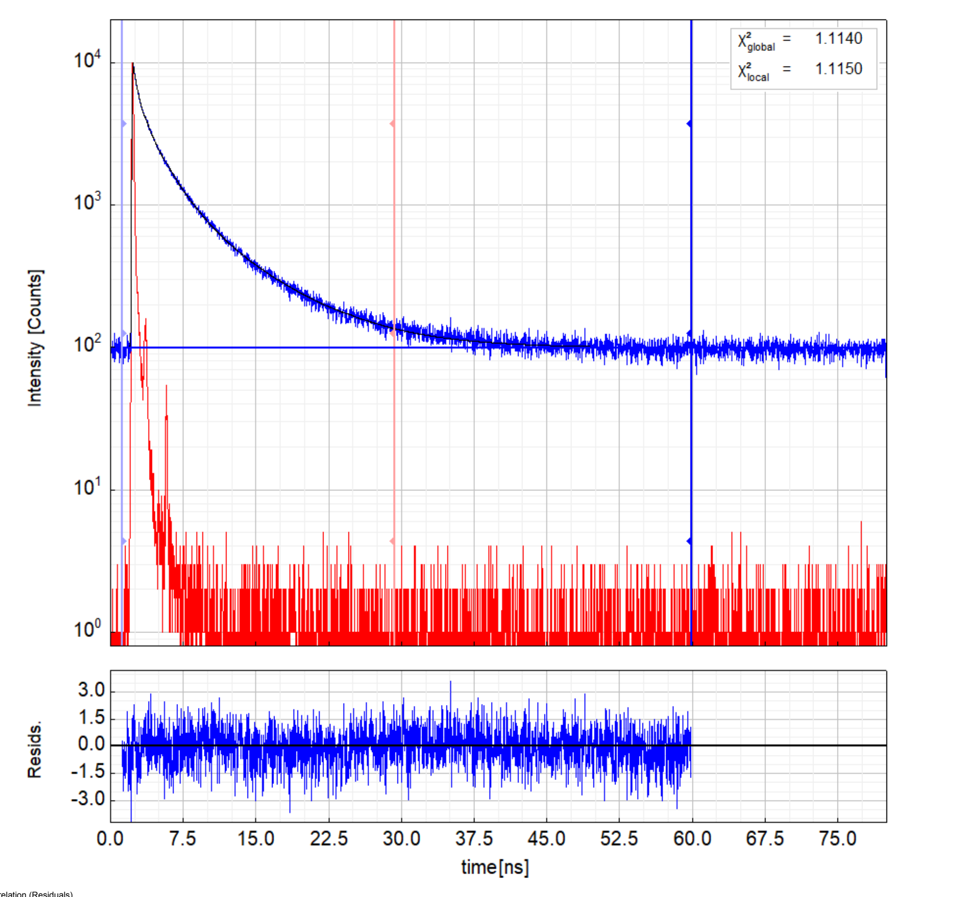

Autocorrelation Residuals

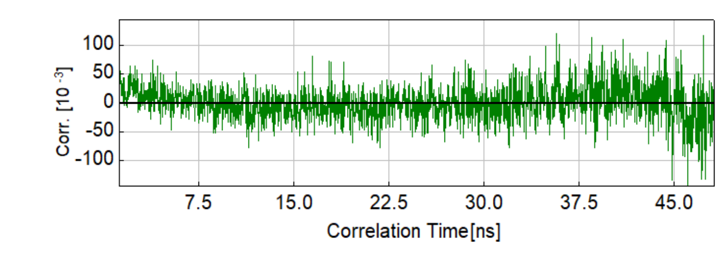

Fitted Parameters

Parameter	Value	Δ	δ
A_1[kCnts/Chnl]	4.138	±0.030	0.7%
τ_1[ns]	2.215	±0.035	1.6%
I_1[kCnts]	366.6	±3.0	0.8%
A_2[kCnts/Chnl]	5.908	±0.048	0.8%
τ_2[ns]	0.4455	±0.0055	1.2%
I_2[kCnts]	105.3	±1.6	1.5%
A_3[kCnts/Chnl]	1.570	±0.030	1.9%
τ_3[ns]	7.088	±0.041	0.6%
I_3[kCnts]	444.9	±5.9	1.3%
$Bkgr_{Dec}$[kCnts]	0.0993	±0.0004	0.4%
$Bkgr_{IRF}$[Cnts/Chnl]	0.694	±0.043	6.2%
$Shift_{IRF}$[ps]	11.81	±0.53	4.5%
A_{Scatt}[kCnts]	38.09	±0.54	1.4%
τ_{AvInt}[ns]	4.377	±0.015	0.3%
τ_{AvAmp}[ns]	1.974	±0.012	0.6%

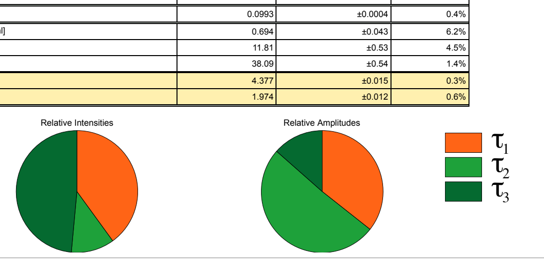

Data Set: 8 / 11

Decay: crv[8]; IRF: crv[0]

Fit

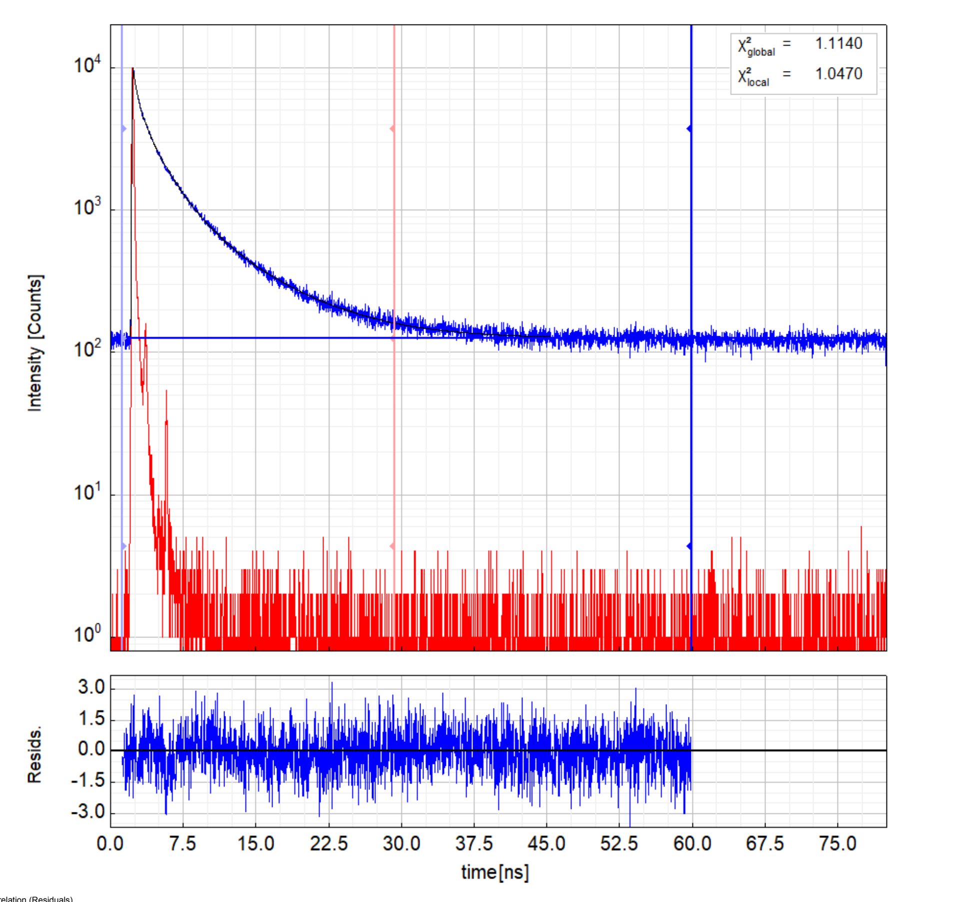

Autocorrelation Residuals

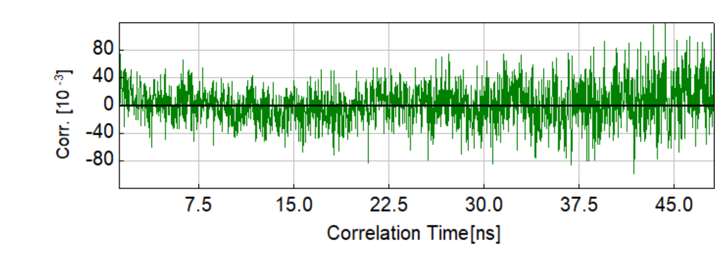

Fitted Parameters

Parameter	Value	Δ	δ
A_1[kCnts/Chnl]	4.124	±0.012	0.3%
τ_1[ns]	2.215	±0.035	1.6%
I_1[kCnts]	365.3	±2.0	0.5%
A_2[kCnts/Chnl]	5.786	±0.071	1.2%
τ_2[ns]	0.4455	±0.0055	1.2%
I_2[kCnts]	103.1	±1.6	1.5%
A_3[kCnts/Chnl]	1.588	±0.024	1.5%
τ_3[ns]	7.088	±0.041	0.6%
I_3[kCnts]	450.2	±6.1	1.4%
$Bkgr_{Dec}$[kCnts]	0.1249	±0.0004	0.3%
$Bkgr_{IRF}$[Cnts/Chnl]	0.80	±0.15	19%
$Shift_{IRF}$[ps]	12.62	±0.46	3.6%
A_{Scatt}[kCnts]	39.27	±0.43	1.1%
τ_{AvInt}[ns]	4.405	±0.018	0.4%
τ_{AvAmp}[ns]	1.9974	±0.0100	0.5%

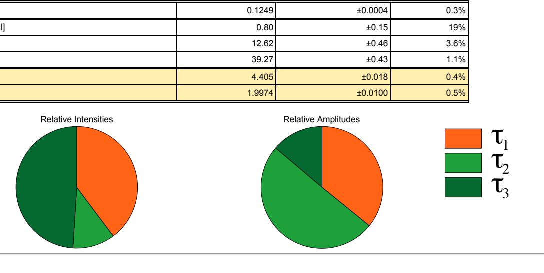

Data Set: 9 / 11

Decay: crv[9]; IRF: crv[0]

Fit

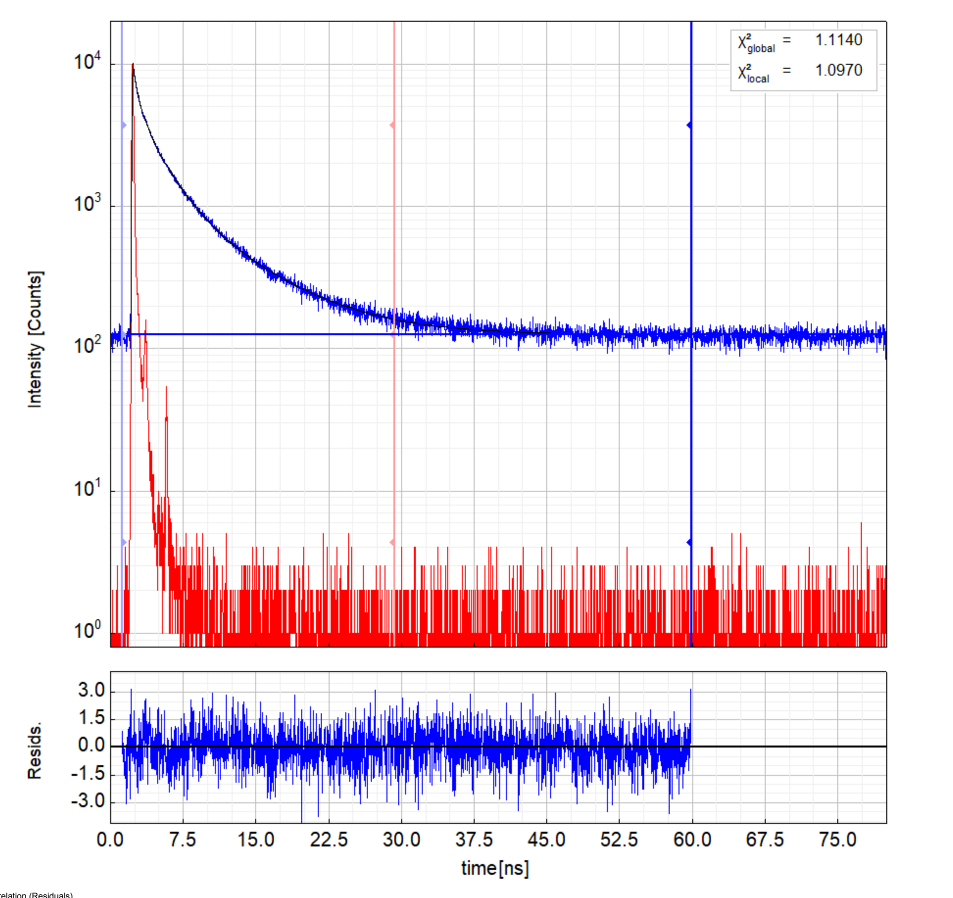

Autocorrelation Residuals

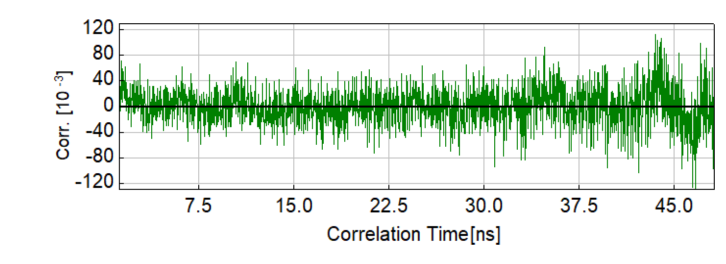

Fitted Parameters

Parameter	Value	Δ	δ
A_1[kCnts/Chnl]	3.955	±0.031	0.8%
τ_1[ns]	2.215	±0.035	1.6%
I_1[kCnts]	350.3	±2.9	0.8%
A_2[kCnts/Chnl]	5.691	±0.087	1.5%
τ_2[ns]	0.4455	±0.0055	1.2%
I_2[kCnts]	101.4	±2.8	2.7%
A_3[kCnts/Chnl]	1.5952	±0.0094	0.6%
τ_3[ns]	7.088	±0.041	0.6%
I_3[kCnts]	452.3	±1.9	0.4%
$Bkgr_{Dec}$[kCnts]	0.1248	±0.0004	0.3%
$Bkgr_{IRF}$[Cnts/Chnl]	0.793	±0.087	11%
$Shift_{IRF}$[ps]	13.67	±0.54	3.9%
A_{Scatt}[kCnts]	41.11	±0.56	1.4%
τ_{AvInt}[ns]	4.454	±0.014	0.3%
τ_{AvAmp}[ns]	2.0105	±0.0096	0.5%

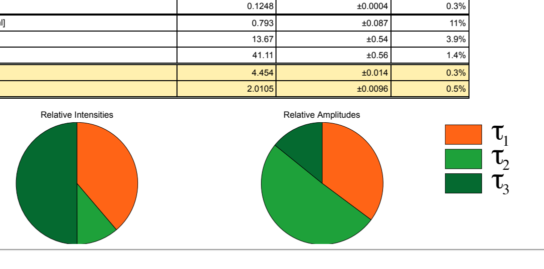

Data Set: 10 / 11

Decay: crv[10]; IRF: crv[0]

Fit

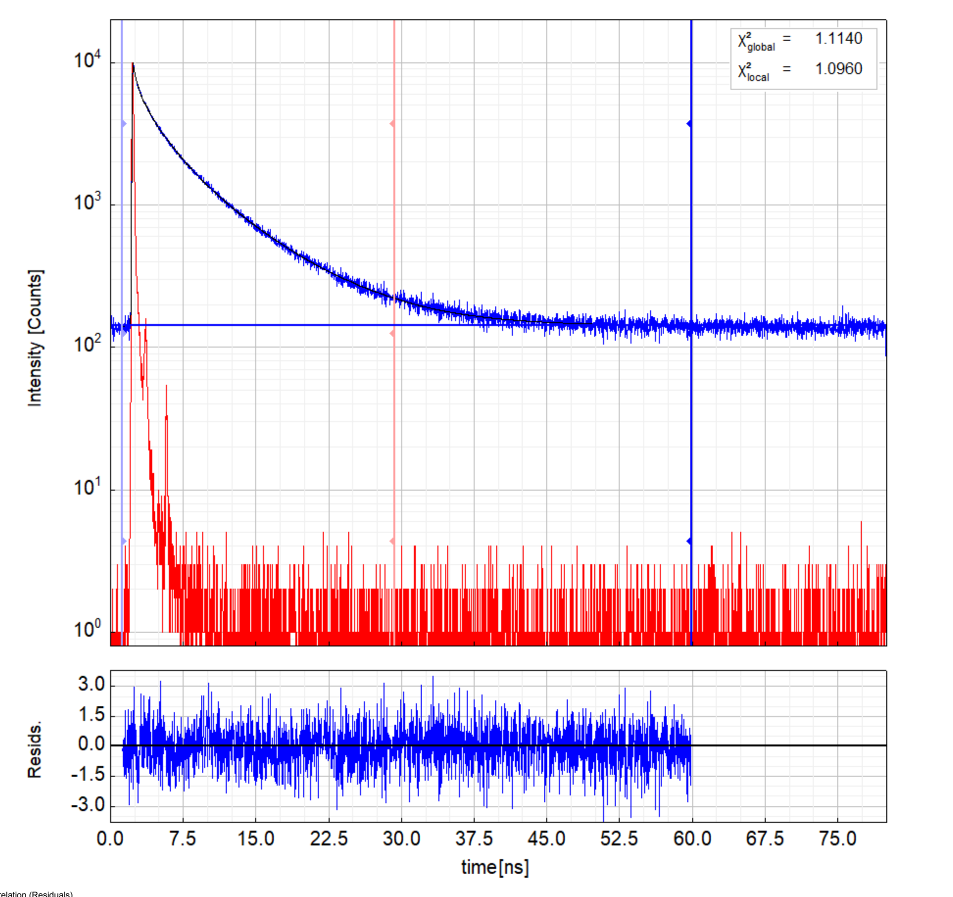

Autocorrelation Residuals

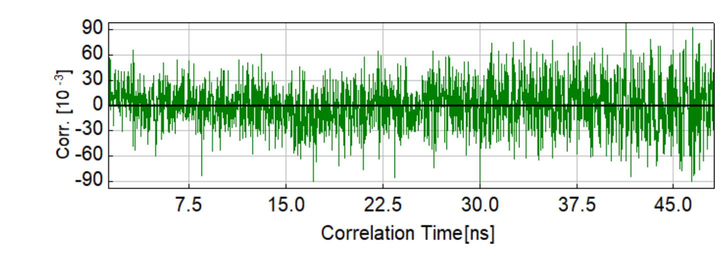

Fitted Parameters

Parameter	Value	Δ	δ
A_1[kCnts/Chnl]	3.577	±0.035	1.0%
τ_1[ns]	2.215	±0.035	1.6%
I_1[kCnts]	316.8	±5.3	1.7%
A_2[kCnts/Chnl]	4.211	±0.079	1.9%
τ_2[ns]	0.4455	±0.0055	1.2%
I_2[kCnts]	75.02	±0.49	0.6%
A_3[kCnts/Chnl]	3.274	±0.036	1.1%
τ_3[ns]	7.088	±0.041	0.6%
I_3[kCnts]	928.0	±5.4	0.6%
$Bkgr_{Dec}$[kCnts]	0.1415	±0.0007	0.5%
$Bkgr_{IRF}$[Cnts/Chnl]	0.34	±0.16	45%
$Shift_{IRF}$[ps]	14.62	±0.60	4.0%
A_{Scatt}[kCnts]	38.79	±0.71	1.8%
τ_{AvInt}[ns]	5.541	±0.020	0.4%
τ_{AvAmp}[ns]	2.984	±0.019	0.6%

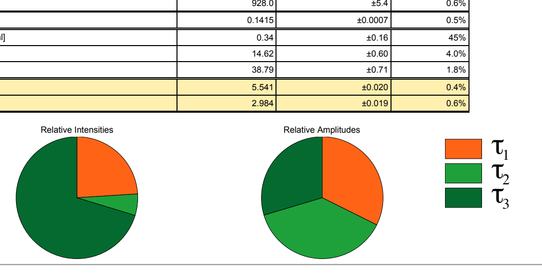

Data Set: 11 / 11

Decay: crv[11]; IRF: crv[0]

Fit

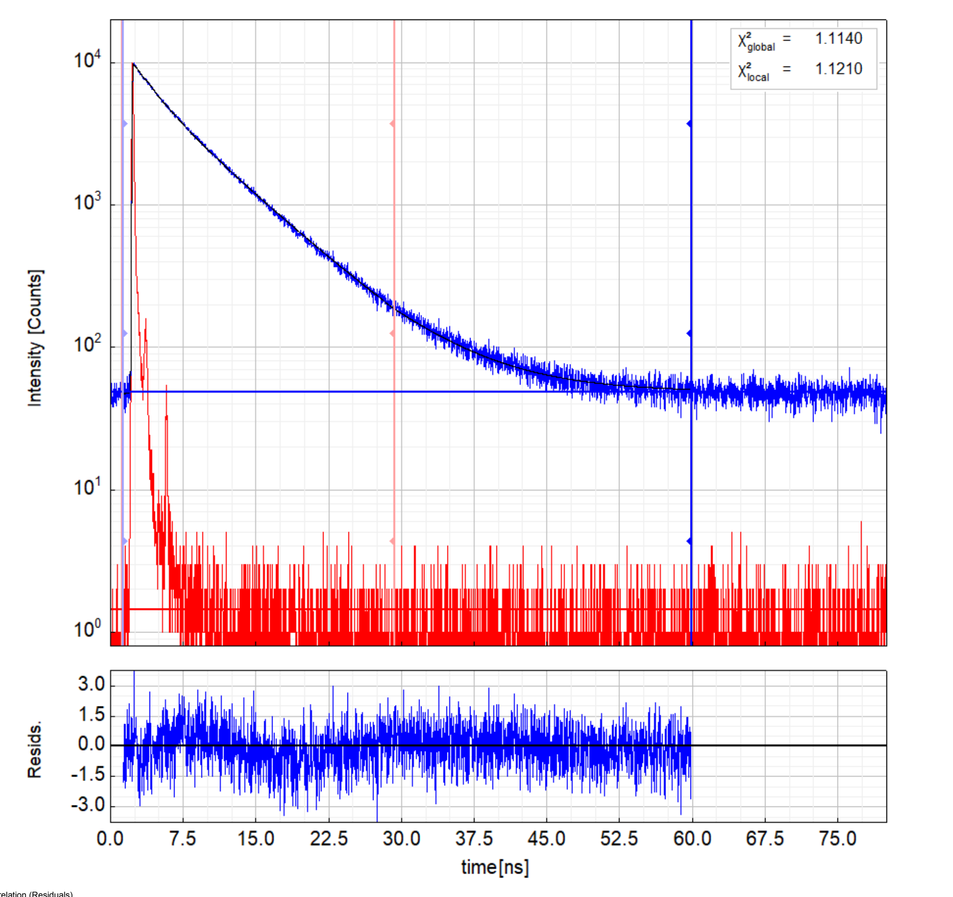

Autocorrelation Residuals

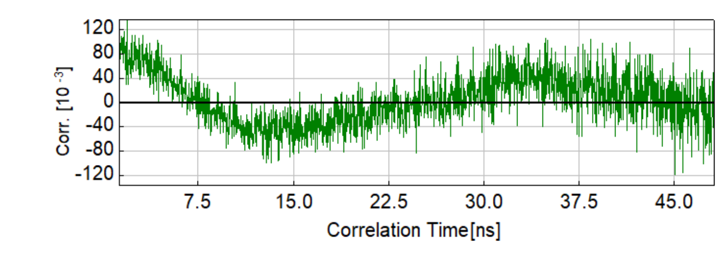

Fitted Parameters

Parameter	Value	Δ	δ
A_1[kCnts/Chnl]	3.60	±0.12	3.3%
τ_1[ns]	2.215	±0.035	1.6%
I_1[kCnts]	319	±11	3.2%
A_2[kCnts/Chnl]	0.912	±0.056	6.1%
τ_2[ns]	0.4455	±0.0055	1.2%
I_2[kCnts]	16.25	±0.96	5.9%
A_3[kCnts/Chnl]	6.825	±0.066	1.0%
τ_3[ns]	7.088	±0.041	0.6%
I_3[kCnts]	1 934.7	±8.8	0.5%
$Bkgr_{Dec}$[kCnts]	0.0489	±0.0003	0.4%
$Bkgr_{IRF}$[Cnts/Chnl]	1.440	±0.062	4.3%
$Shift_{IRF}$[ps]	17.98	±0.40	2.2%
A_{Scatt}[kCnts]	30.55	±0.28	0.9%
τ_{AvInt}[ns]	6.356	±0.016	0.3%
τ_{AvAmp}[ns]	5.007	±0.026	0.5%

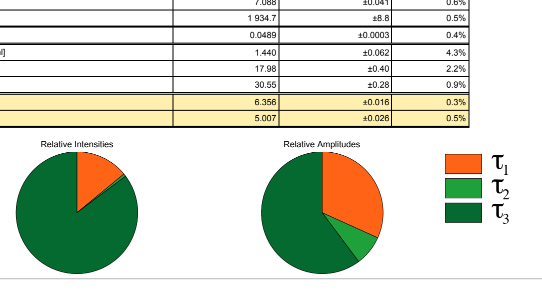

Parameter Plot

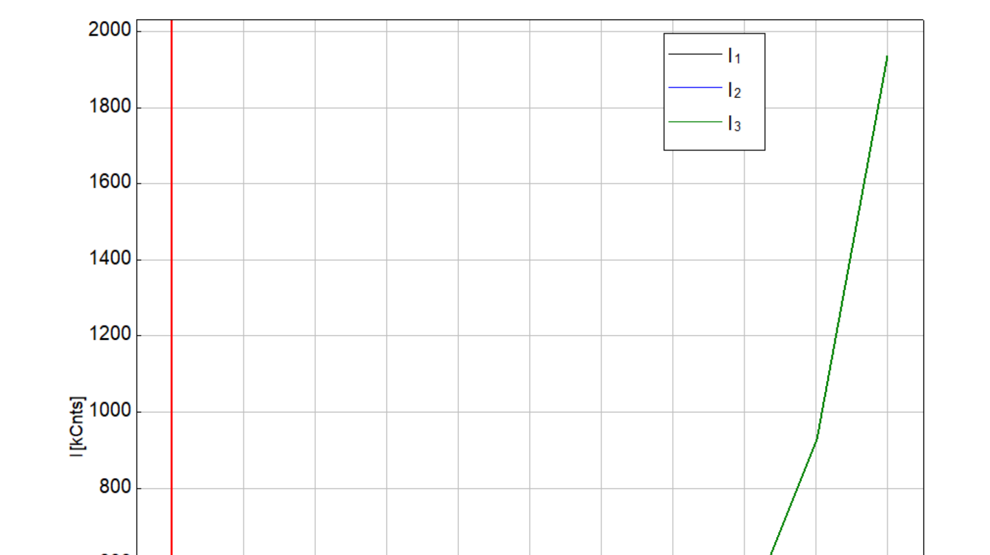

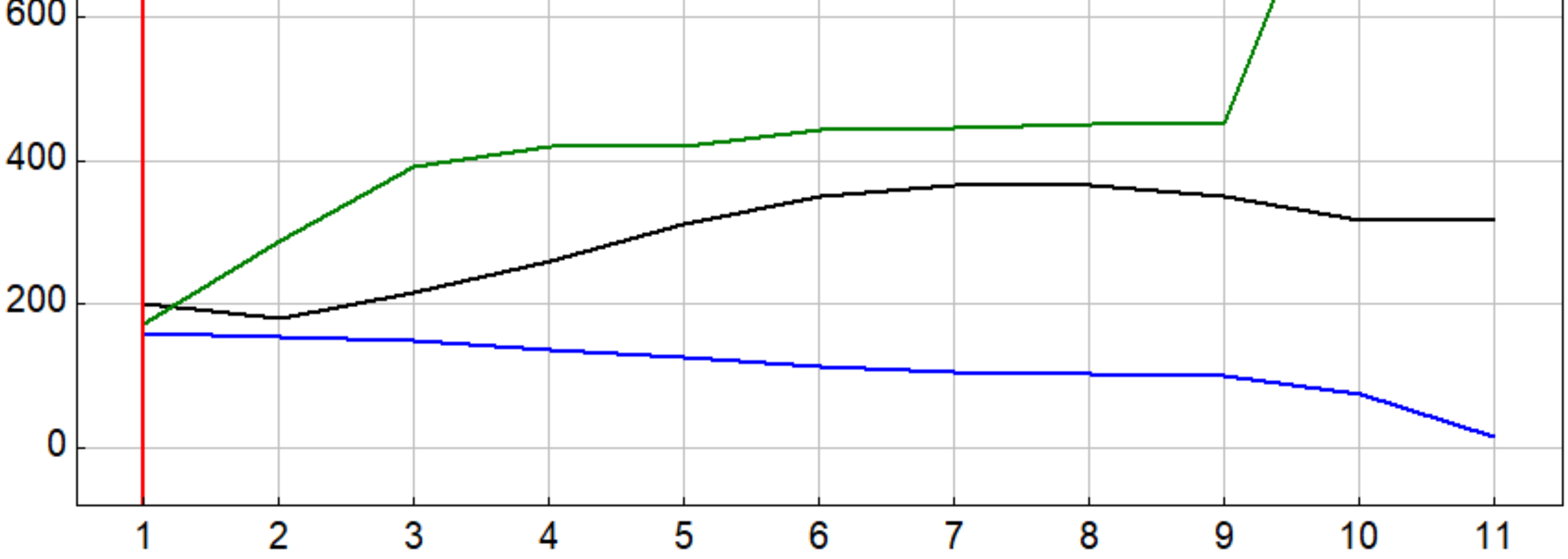

Global Parameters

Parameter	Value	Δ	δ
τ_1[ns]	2.215	±0.035	1.6%
τ_2[ns]	0.4455	±0.0055	1.2%
τ_3[ns]	7.088	±0.041	0.6%
