# Supplementary material for: A Porphyrin‐Based Organic Network Comprising Sustainable Carbon Dots for Photopolymerization
Source: Angew Chem Int Ed Engl. 2022 Sep 1;61(40):e202208180. doi: 10.1002/anie.202208180 (PMC9826160; doi:10.1002/anie.202208180)
Supplement: Supplementary file 2 — Supporting Information [file ANIE-61-0-s002.pdf]

EasyTau Report

3/11/2022 7:15:42 PM
F:\Paper Xiongfei\CD-1 DMF tau global.html

Container Curves

TRES+IRF_20201222_1558.etc: crv[0 - 31]

Container Properties

Measurement Context: Time Resolved Emission Scan
Detection Wavelength: 400.0 - 700.0 (Step: 10.0) nm
Summary:

Sample:	CD_X_1
Solvent:	Dimethyl formamide
Excitation:	V pol 376±1nm with LDH-P-C-375
Detection:	M pol 400...700nm 10nm steps<br>10000 peak counts<br>grating 1200/500-<br>detector UV-red [PMT]

Comment

Model: Exponential [Reconv.]

$$\mathrm{Dec}(t)=\left[\int_{-\infty}^{t} dt'\,[\mathrm{IRF}(t-Shift_{IRF})-Bkgr_{IRF}]\left[\sum_{i=1}^{n_{Exp}} A_i\, e^{-\frac{t-t'}{\tau_i}} + A_{Scatt}\,\delta(t-t')\right]\right]+Bkgr_{Dec}$$

$$I_m = A_m \tau_m$$

$$A_{Rel\,m}=A_m \Big/ \sum_{\substack{i=1\\A_i>0}}^{n_{Exp}} A_i \qquad I_{Rel\,m}=I_m \Big/ \sum_{\substack{i=1\\I_i>0}}^{n_{Exp}} I_i$$

$$\tau_{Av\,Amp}=\sum_{\substack{i=1\\A_i>0}}^{n_{Exp}} A_i\tau_i \Big/ \sum_{\substack{i=1\\A_i>0}}^{n_{Exp}} A_i \qquad \tau_{Av\,Int}=\sum_{\substack{i=1\\I_i>0}}^{n_{Exp}} I_i\tau_i \Big/ \sum_{\substack{i=1\\I_i>0}}^{n_{Exp}} I_i$$

Data Set: 1 / 31

Decay: crv[1]; IRF: crv[0]

Fit

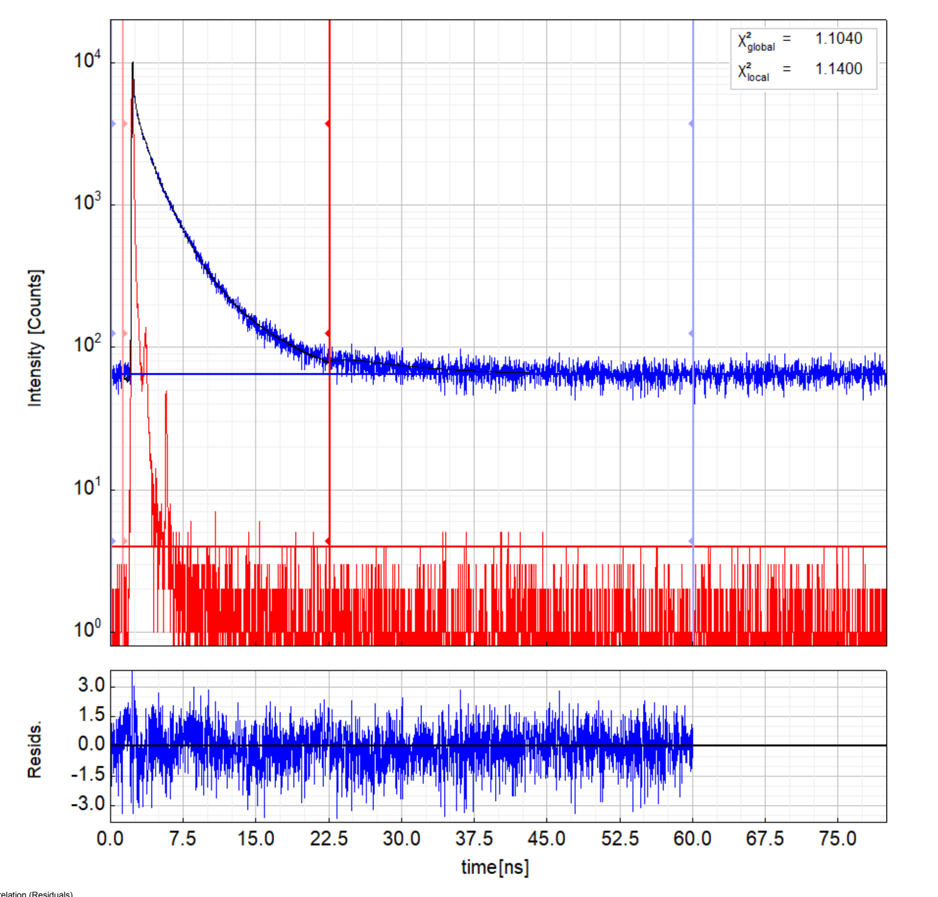

Autocorrelation (Residuals)

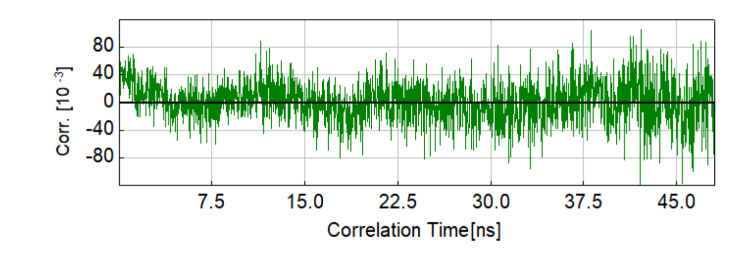

Fitted Parameters

Parameter	Value	Δ	δ
A_1[kCnts/Chnl]	0.4647	±0.0097	2.1%
τ_1[ns]	7.675	±0.012	0.2%
I_1[kCnts]	142.7	±3.0	2.1%
A_2[kCnts/Chnl]	3.716	±0.025	0.7%
τ_2[ns]	2.250	±0.013	0.6%
I_2[kCnts]	334.3	±2.1	0.6%
A_3[kCnts/Chnl]	2.876	±0.095	3.3%
τ_3[ns]	0.5216	±0.0058	1.1%
I_3[kCnts]	60.0	±2.7	4.4%
$Bkgr_{Dec}$[kCnts]	0.0641	±0.0004	0.5%
$Bkgr_{IRF}$[Cnts/Chnl]	3.95	±0.16	3.9%
$Shift_{IRF}$[ps]	-2.82	±0.76	27%
A_{Scatt}[kCnts]	60.9	±1.7	2.8%
τ_{AvInt}[ns]	3.498	±0.021	0.6%
τ_{AvAmp}[ns]	1.903	±0.021	1.1%
λ_{Det}[nm]	400.0000	---	---

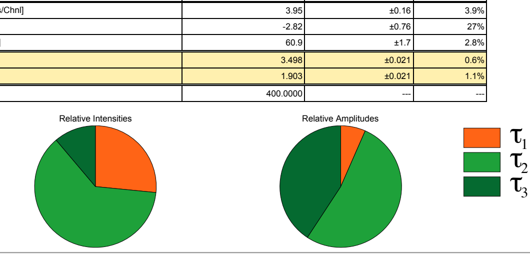

Data Set: 2 / 31

Decay: crv[2]; IRF: crv[0]

Fit

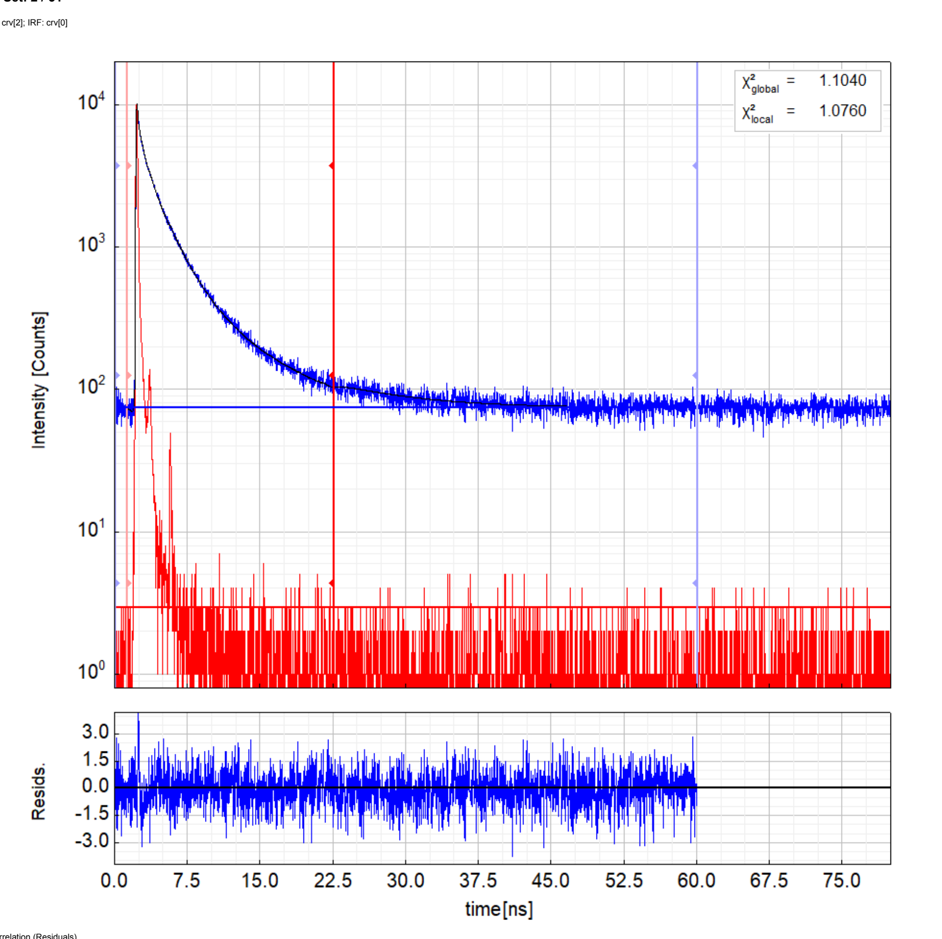

Autocorrelation (Residuals)

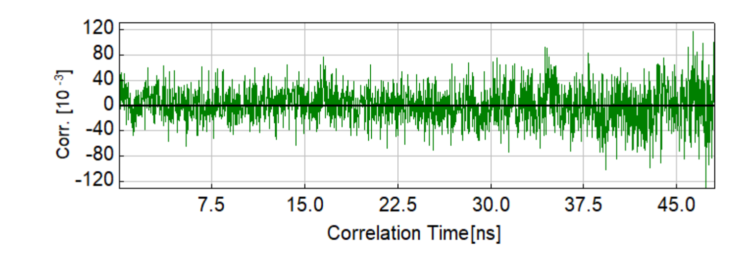

Fitted Parameters

Parameter	Value	Δ	δ
A_1[kCnts/Chnl]	0.6079	±0.0091	1.5%
τ_1[ns]	7.675	±0.012	0.2%
I_1[kCnts]	186.6	±2.7	1.4%
A_2[kCnts/Chnl]	4.154	±0.032	0.7%
τ_2[ns]	2.250	±0.013	0.6%
I_2[kCnts]	373.7	±1.5	0.4%
A_3[kCnts/Chnl]	4.95	±0.13	2.5%
τ_3[ns]	0.5216	±0.0058	1.1%
I_3[kCnts]	103.3	±2.1	2.0%
$Bkgr_{Dec}$[kCnts]	0.0745	±0.0003	0.3%
$Bkgr_{IRF}$[Cnts/Chnl]	2.95	±0.13	4.2%
$Shift_{IRF}$[ps]	11.3	±1.3	11%
A_{Scatt}[kCnts]	50.3	±1.4	2.6%
τ_{AvInt}[ns]	3.507	±0.015	0.4%
τ_{AvAmp}[ns]	1.709	±0.017	1.0%
λ_{Det}[nm]	410.0000	---	---

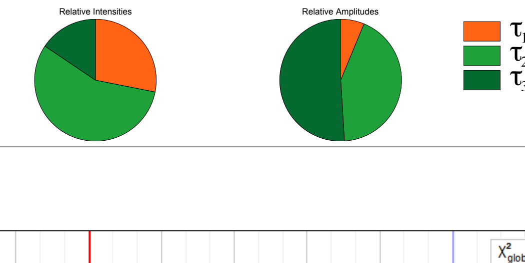

Data Set: 3 / 31

Decay: crv[3]; IRF: crv[0]

Fit

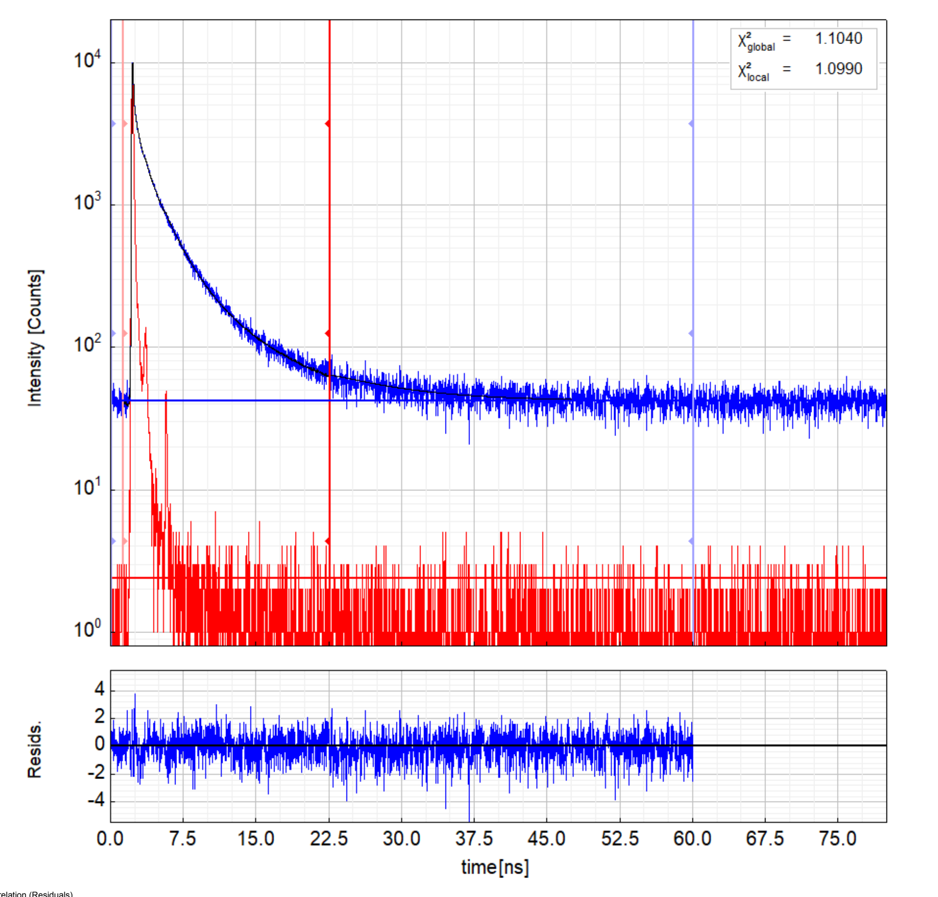

Autocorrelation (Residuals)

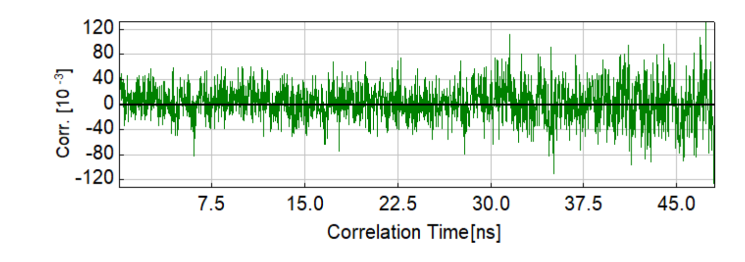

Fitted Parameters

Parameter	Value	Δ	δ
A_1[kCnts/Chnl]	0.3806	±0.0063	1.6%
τ_1[ns]	7.675	±0.012	0.2%
I_1[kCnts]	116.9	±1.8	1.5%
A_2[kCnts/Chnl]	2.519	±0.022	0.8%
τ_2[ns]	2.250	±0.013	0.6%
I_2[kCnts]	226.6	±1.7	0.7%
A_3[kCnts/Chnl]	3.115	±0.084	2.7%
τ_3[ns]	0.5216	±0.0058	1.1%
I_3[kCnts]	65.0	±2.1	3.2%
$Bkgr_{Dec}$[kCnts]	0.0421	±0.0002	0.4%
$Bkgr_{IRF}$[Cnts/Chnl]	2.39	±0.18	7.1%
$Shift_{IRF}$[ps]	-4.29	±0.63	15%
A_{Scatt}[kCnts]	65.06	±0.87	1.3%
τ_{AvInt}[ns]	3.527	±0.024	0.7%
τ_{AvAmp}[ns]	1.698	±0.014	0.8%
λ_{Det}[nm]	420.0000	---	---

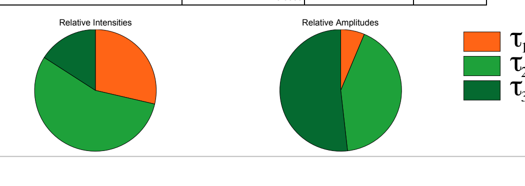

Data Set: 4 / 31

Decay: crv[4]; IRF: crv[0]

Fit

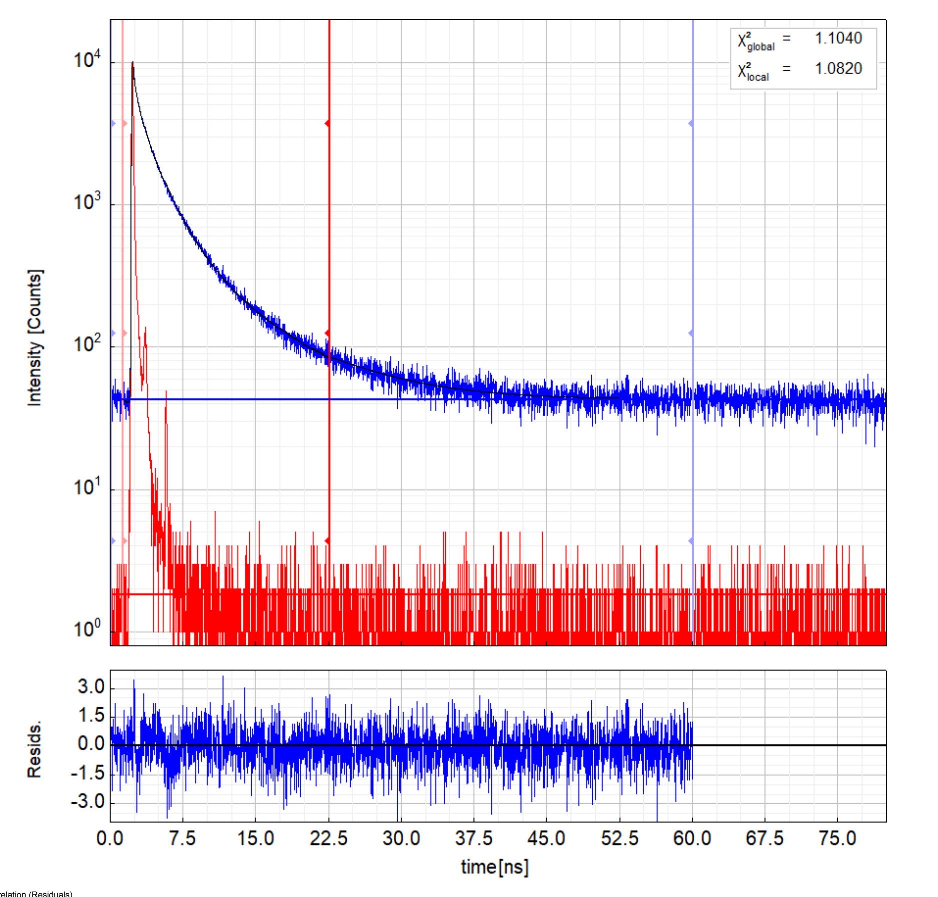

Autocorrelation (Residuals)

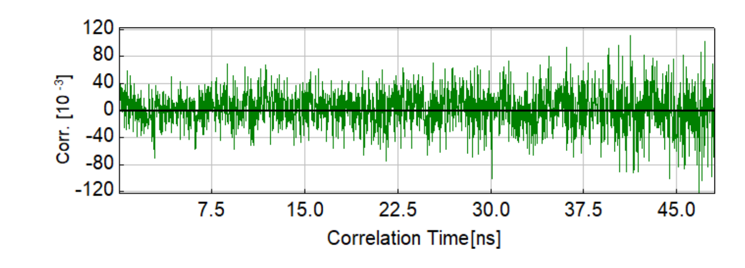

Fitted Parameters

Parameter	Value	Δ	δ
A_1[kCnts/Chnl]	0.6642	±0.0080	1.2%
τ_1[ns]	7.675	±0.012	0.2%
I_1[kCnts]	203.9	±2.6	1.2%
A_2[kCnts/Chnl]	4.066	±0.038	0.9%
τ_2[ns]	2.250	±0.013	0.6%
I_2[kCnts]	365.9	±1.2	0.3%
A_3[kCnts/Chnl]	5.340	±0.060	1.1%
τ_3[ns]	0.5216	±0.0058	1.1%
I_3[kCnts]	111.4	±2.0	1.7%
$Bkgr_{Dec}$[kCnts]	0.0429	±0.0002	0.4%
$Bkgr_{IRF}$[Cnts/Chnl]	1.83	±0.17	9.2%
$Shift_{IRF}$[ps]	10.72	±0.95	8.8%
A_{Scatt}[kCnts]	49.0	±1.1	2.2%
τ_{AvInt}[ns]	3.591	±0.018	0.5%
τ_{AvAmp}[ns]	1.692	±0.014	0.8%
λ_{Det}[nm]	430.0000	---	---

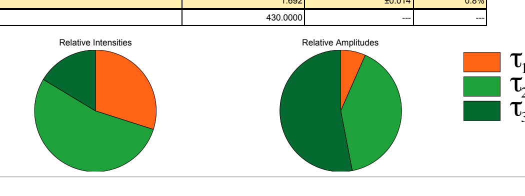

Data Set: 5 / 31

Decay: crv[5]; IRF: crv[0]

Fit

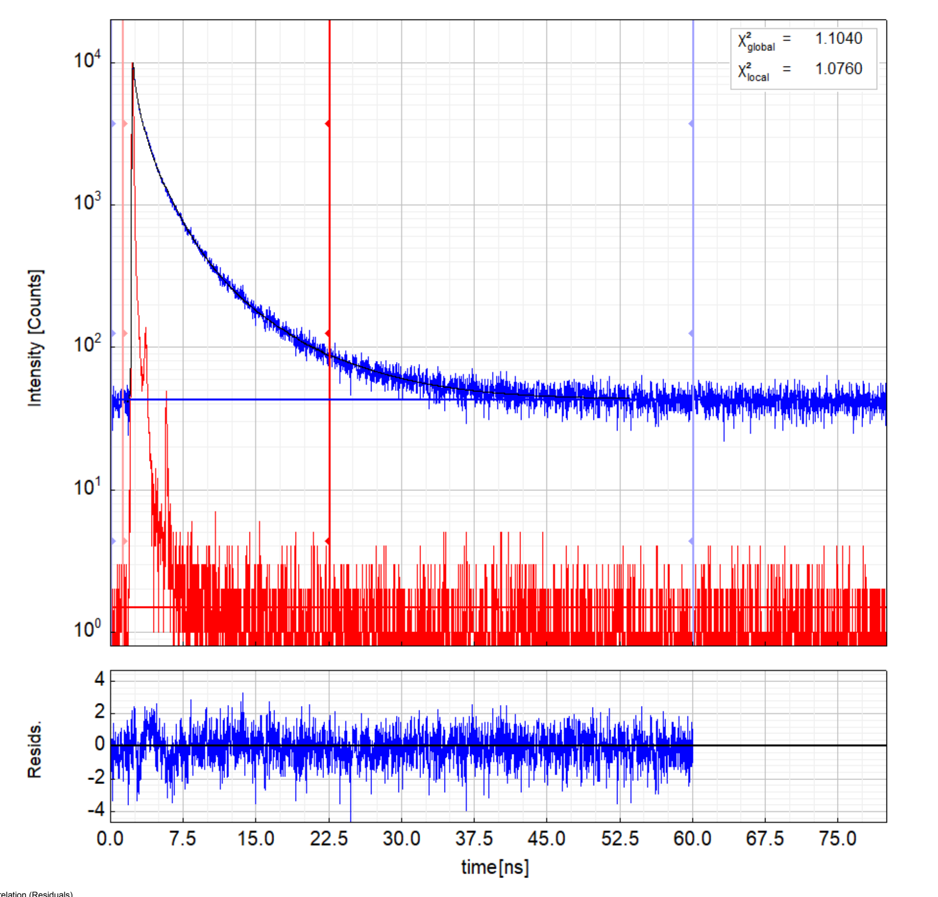

Autocorrelation (Residuals)

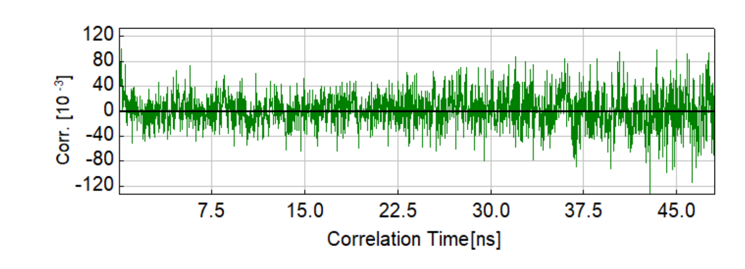

Fitted Parameters

Parameter	Value	Δ	δ
A_1[kCnts/Chnl]	0.6630	±0.0046	0.7%
τ_1[ns]	7.675	±0.012	0.2%
I_1[kCnts]	203.6	±1.6	0.7%
A_2[kCnts/Chnl]	3.702	±0.035	0.9%
τ_2[ns]	2.250	±0.013	0.6%
I_2[kCnts]	333.0	±2.3	0.7%
A_3[kCnts/Chnl]	5.916	±0.065	1.1%
τ_3[ns]	0.5216	±0.0058	1.1%
I_3[kCnts]	123.5	±1.8	1.4%
$Bkgr_{Dec}$[kCnts]	0.0430	±0.0003	0.6%
$Bkgr_{IRF}$[Cnts/Chnl]	1.481	±0.084	5.7%
$Shift_{IRF}$[ps]	11.4	±1.1	8.8%
A_{Scatt}[kCnts]	46.19	±0.75	1.6%
τ_{AvInt}[ns]	3.600	±0.013	0.4%
τ_{AvAmp}[ns]	1.6051	±0.0086	0.5%
λ_{Det}[nm]	440.0000	---	---

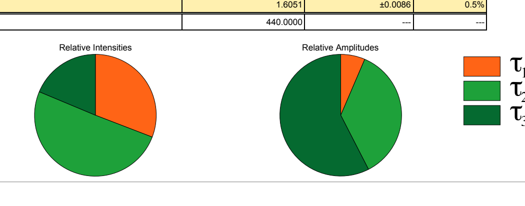

Data Set: 6 / 31

Decay: crv[6]; IRF: crv[0]

Fit

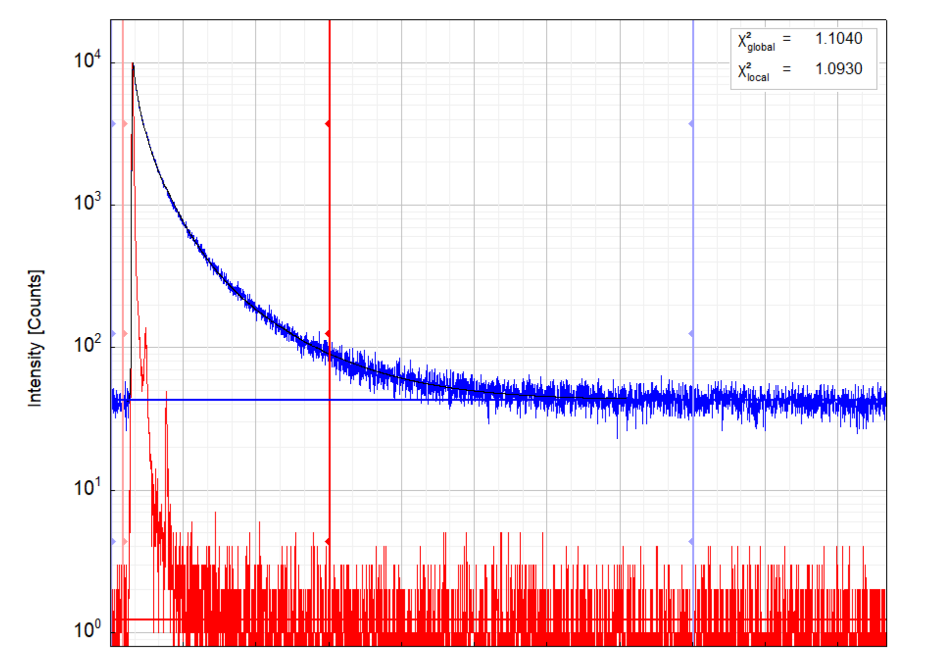

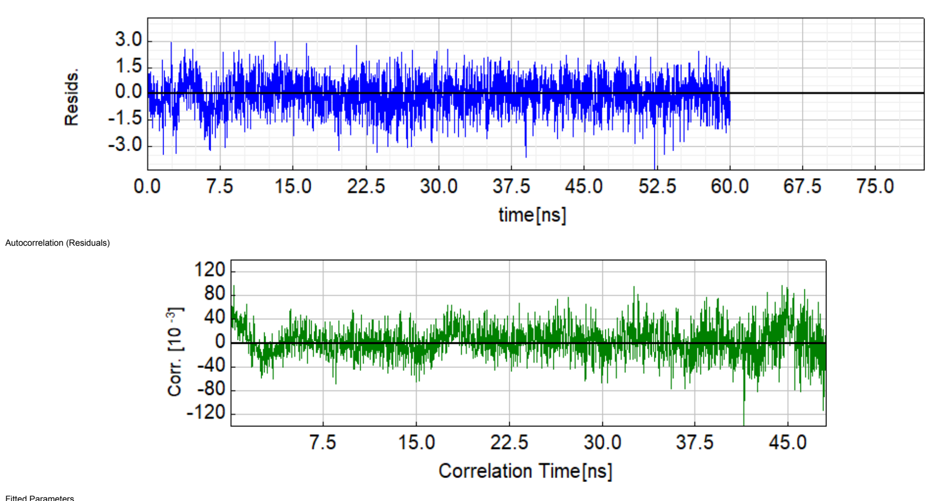

Fitted Parameters

Parameter	Value	Δ	δ
A_1[kCnts/Chnl]	0.6730	±0.0092	1.4%
τ_1[ns]	7.675	±0.012	0.2%
I_1[kCnts]	206.7	±3.1	1.5%
A_2[kCnts/Chnl]	3.672	±0.042	1.1%
τ_2[ns]	2.250	±0.013	0.6%
I_2[kCnts]	330.3	±2.3	0.7%
A_3[kCnts/Chnl]	6.647	±0.054	0.8%
τ_3[ns]	0.5216	±0.0058	1.1%
I_3[kCnts]	138.7	±1.8	1.3%
$Bkgr_{Dec}$[kCnts]	0.0429	±0.0002	0.4%
$Bkgr_{IRF}$[Cnts/Chnl]	1.24	±0.15	12%
$Shift_{IRF}$[ps]	11.42	±0.41	3.6%
A_{Scatt}[kCnts]	43.50	±0.45	1.0%
τ_{AvInt}[ns]	3.554	±0.022	0.6%
τ_{AvAmp}[ns]	1.5367	±0.0053	0.3%
λ_{Det}[nm]	450.0000	---	---

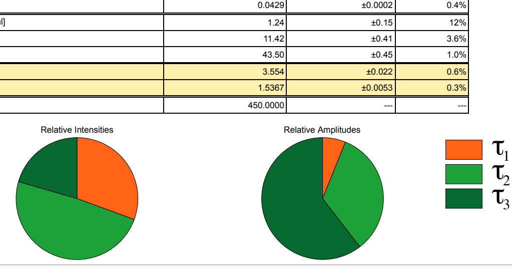

Data Set: 7 / 31

Decay: crv[7]; IRF: crv[0]

Fit

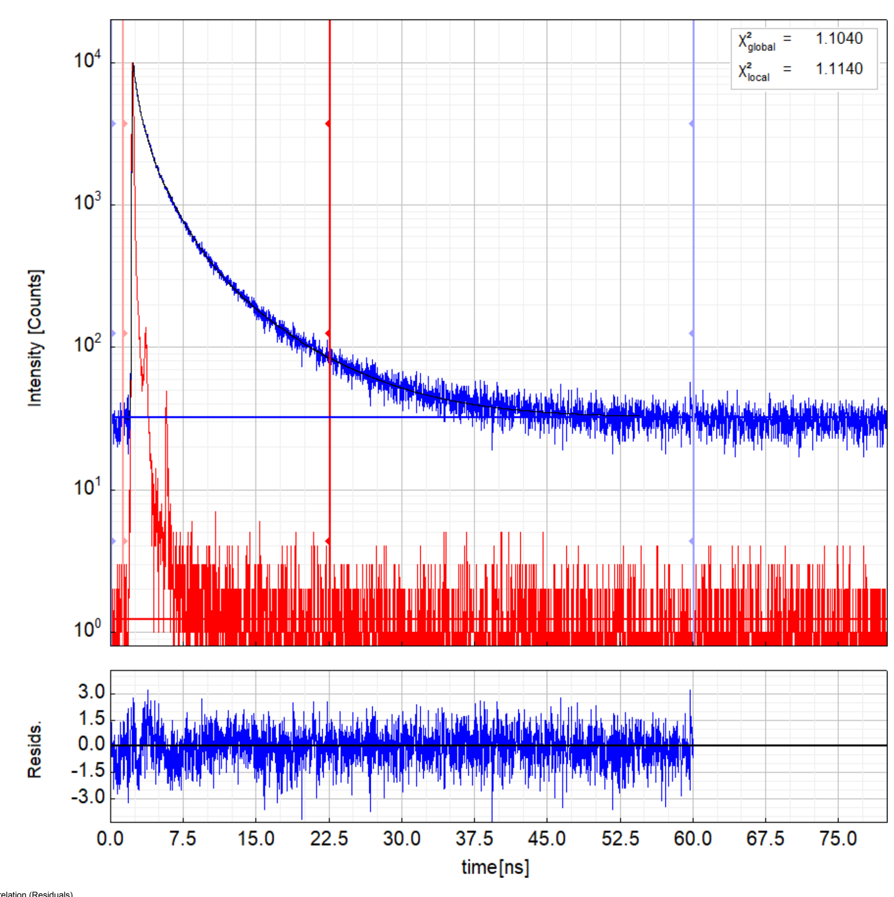

Autocorrelation Residuals

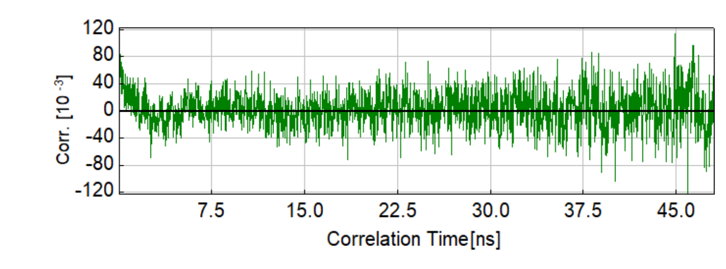

Fitted Parameters

Parameter	Value	Δ	δ
A_1[kCnts/Chnl]	0.7366	±0.0082	1.1%
τ_1[ns]	7.675	±0.012	0.2%
I_1[kCnts]	226.2	±2.5	1.1%
A_2[kCnts/Chnl]	3.522	±0.019	0.5%
τ_2[ns]	2.250	±0.013	0.6%
I_2[kCnts]	316.9	±2.1	0.6%
A_3[kCnts/Chnl]	7.014	±0.069	1.0%
τ_3[ns]	0.5216	±0.0058	1.1%
I_3[kCnts]	146.4	±2.6	1.8%
$Bkgr_{Dec}$[kCnts]	0.0321	±0.0002	0.4%
$Bkgr_{IRF}$[Cnts/Chnl]	1.24	±0.14	11%
$Shift_{IRF}$[ps]	11.17	±0.58	5.2%
A_{Scatt}[kCnts]	40.85	±0.89	2.2%
τ_{AvInt}[ns]	3.663	±0.016	0.4%
τ_{AvAmp}[ns]	1.5289	±0.0069	0.4%
λ_{Det}[nm]	460.0000	---	---

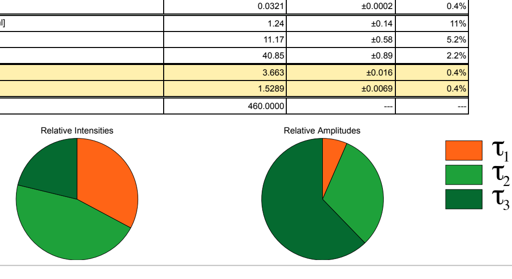

Data Set: 8 / 31

Decay: crv[8]; IRF: crv[0]

Fit

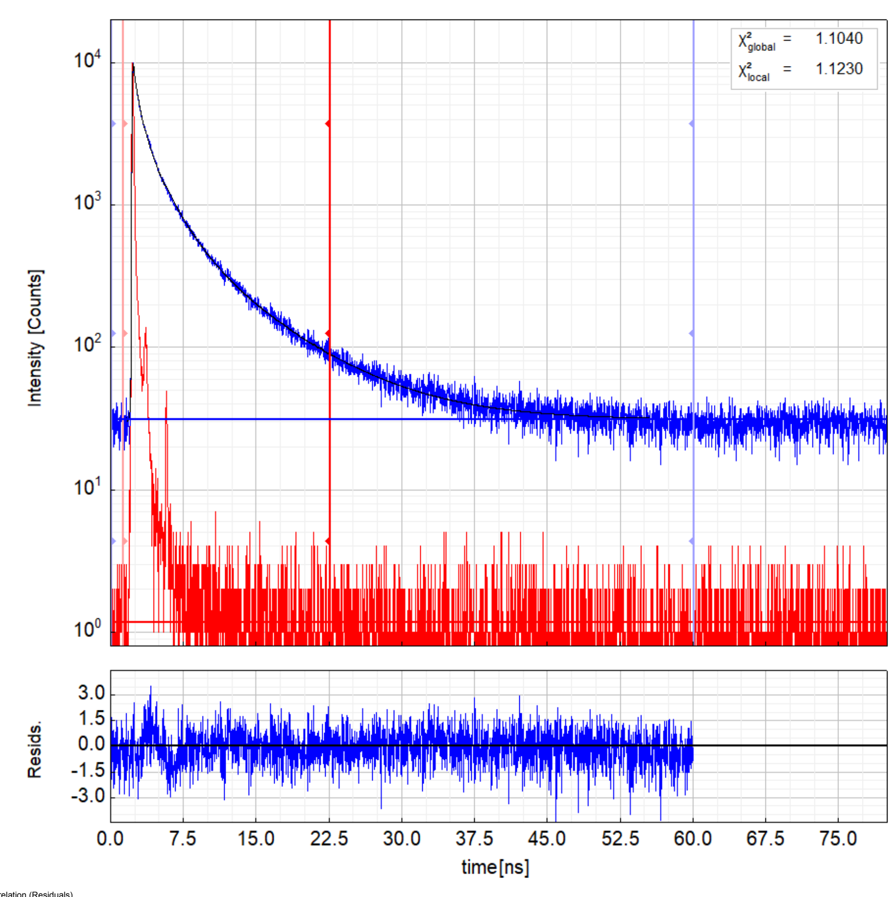

Autocorrelation Residuals

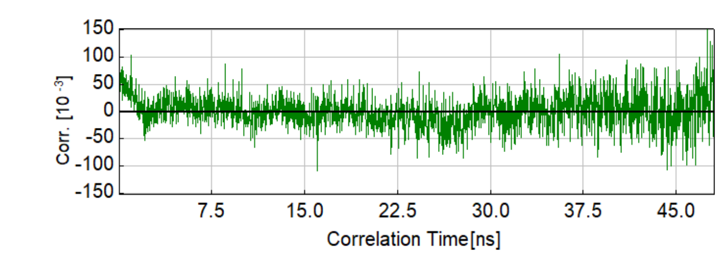

Fitted Parameters

Parameter	Value	Δ	δ
A_1[kCnts/Chnl]	0.8249	±0.0079	0.9%
τ_1[ns]	7.675	±0.012	0.2%
I_1[kCnts]	253.3	±2.1	0.8%
A_2[kCnts/Chnl]	3.395	±0.025	0.7%
τ_2[ns]	2.250	±0.013	0.6%
I_2[kCnts]	305.4	±2.0	0.7%
A_3[kCnts/Chnl]	7.174	±0.085	1.2%
τ_3[ns]	0.5216	±0.0058	1.1%
I_3[kCnts]	149.7	±1.9	1.2%
$Bkgr_{Dec}$[kCnts]	0.0311	±0.0003	0.7%
$Bkgr_{IRF}$[Cnts/Chnl]	1.178	±0.063	5.3%
$Shift_{IRF}$[ps]	10.03	±0.87	8.6%
A_{Scatt}[kCnts]	39.1	±1.4	3.5%
τ_{AvInt}[ns]	3.825	±0.013	0.3%
τ_{AvAmp}[ns]	1.555	±0.013	0.8%
λ_{Det}[nm]	470.0000	---	---

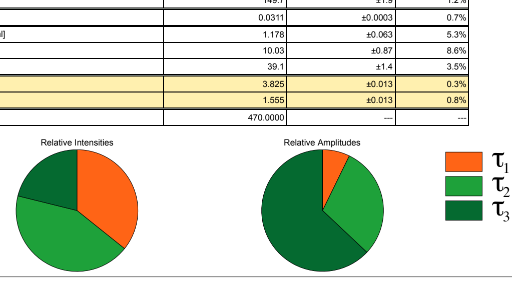

Data Set: 9 / 31

Decay: crv[9]; IRF: crv[0]

Fit

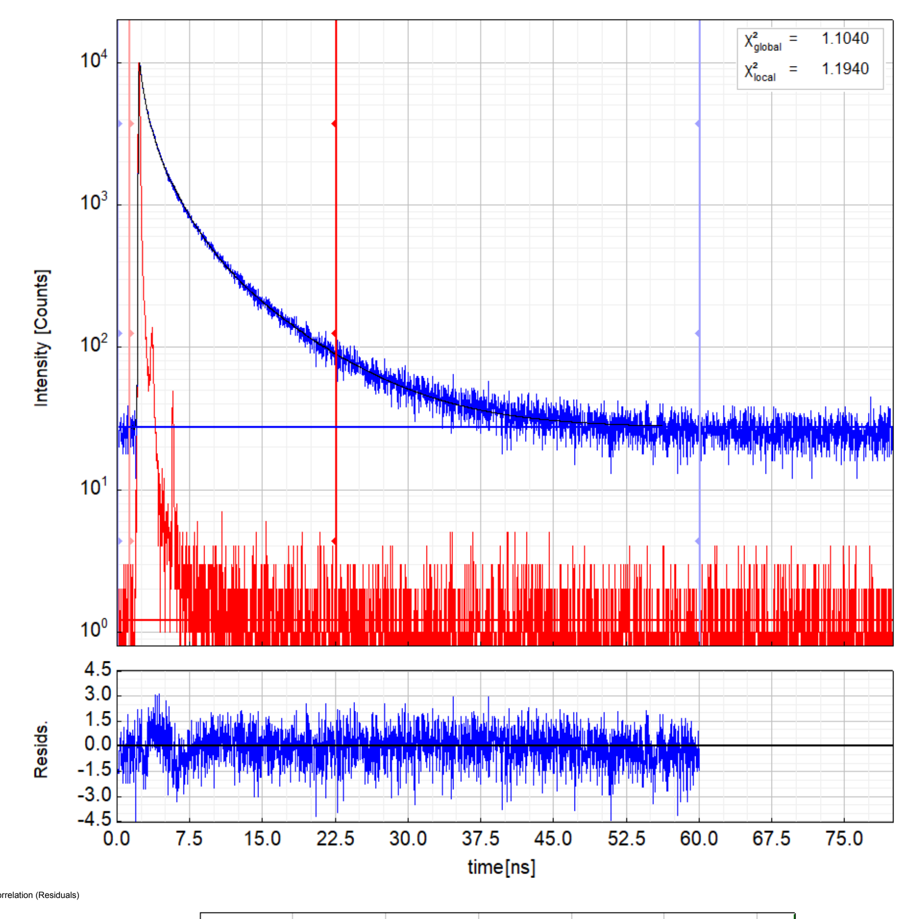

Autocorrelation Residuals

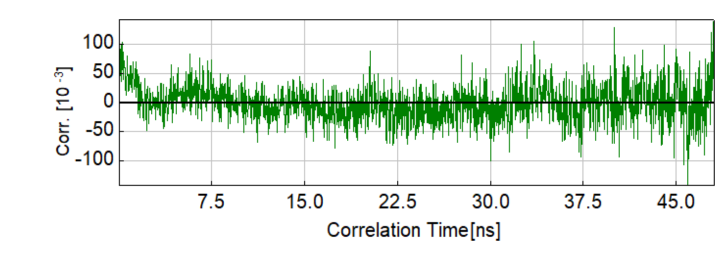

Fitted Parameters

Parameter	Value	Δ	δ
A_1[kCnts/Chnl]	0.881	±0.011	1.2%
τ_1[ns]	7.675	±0.012	0.2%
I_1[kCnts]	270.2	±3.0	1.1%
A_2[kCnts/Chnl]	3.496	±0.026	0.7%
τ_2[ns]	2.250	±0.013	0.6%
I_2[kCnts]	314.6	±1.8	0.6%
A_3[kCnts/Chnl]	7.184	±0.071	1.0%
τ_3[ns]	0.5216	±0.0058	1.1%
I_3[kCnts]	149.9	±1.7	1.1%
$Bkgr_{Dec}$[kCnts]	0.0273	±0.0002	0.4%
$Bkgr_{IRF}$[Cnts/Chnl]	1.21	±0.16	13%
$Shift_{IRF}$[ps]	10.8	±1.1	9.8%
A_{Scatt}[kCnts]	39.0	±1.4	3.5%
τ_{AvInt}[ns]	3.893	±0.013	0.3%
τ_{AvAmp}[ns]	1.589	±0.011	0.7%
λ_{Det}[nm]	480.0000	---	---

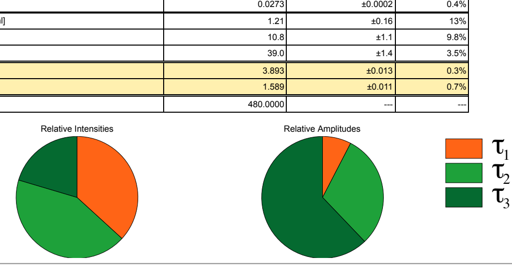

Data Set: 10 / 31

Decay: crv[10]; IRF: crv[0]

Fit

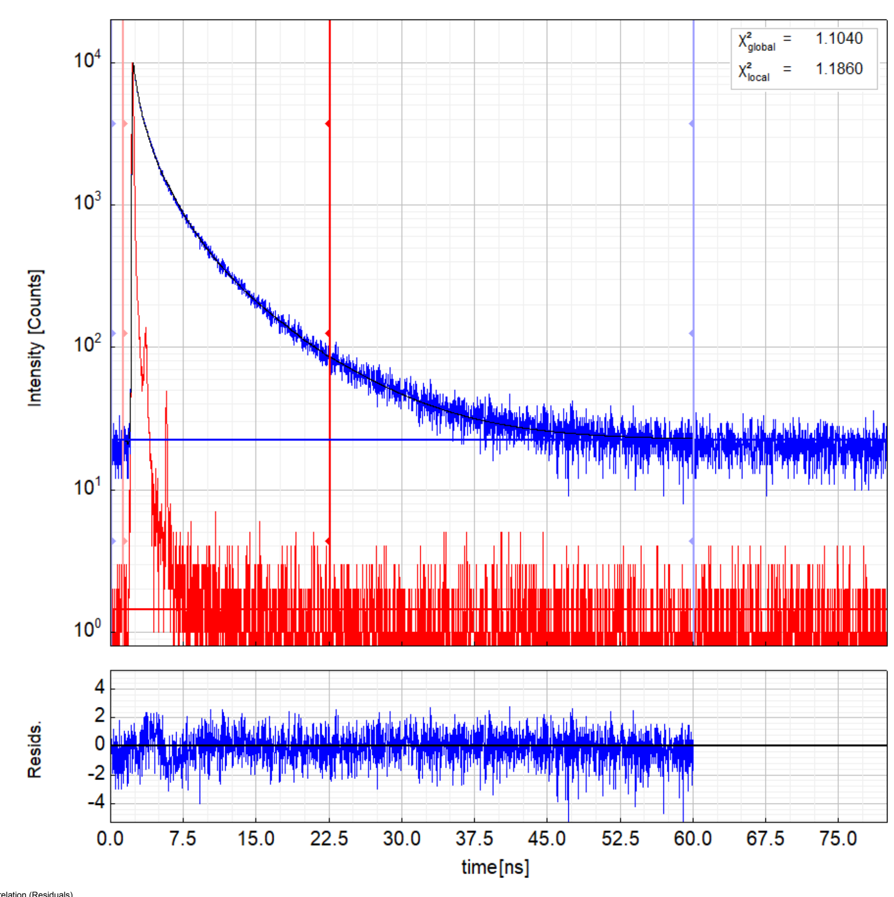

Autocorrelation Residuals

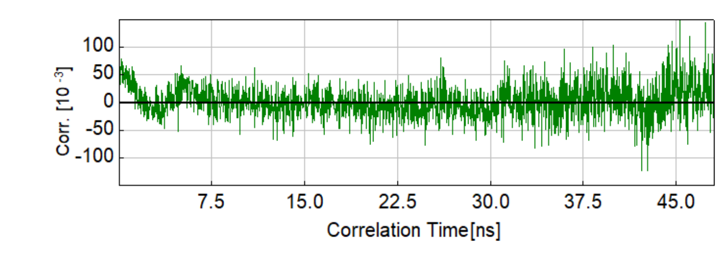

Fitted Parameters

Parameter	Value	Δ	δ
A_1[kCnts/Chnl]	0.9228	±0.0067	0.7%
τ_1[ns]	7.675	±0.012	0.2%
I_1[kCnts]	283.3	±2.1	0.7%
A_2[kCnts/Chnl]	3.714	±0.053	1.4%
τ_2[ns]	2.250	±0.013	0.6%
I_2[kCnts]	334.2	±3.3	1.0%
A_3[kCnts/Chnl]	7.096	±0.058	0.8%
τ_3[ns]	0.5216	±0.0058	1.1%
I_3[kCnts]	148.1	±2.2	1.5%
$Bkgr_{Dec}$[kCnts]	0.0224	±0.0001	0.4%
$Bkgr_{IRF}$[Cnts/Chnl]	1.45	±0.15	10%
$Shift_{IRF}$[ps]	10.64	±0.59	5.5%
A_{Scatt}[kCnts]	37.83	±0.68	1.8%
τ_{AvInt}[ns]	3.924	±0.020	0.5%
τ_{AvAmp}[ns]	1.6312	±0.0070	0.4%
λ_{Det}[nm]	490.0000	---	---

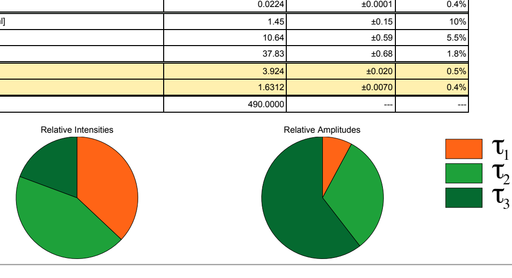

Data Set: 11 / 31

Decay: crv[11]; IRF: crv[0]

Fit

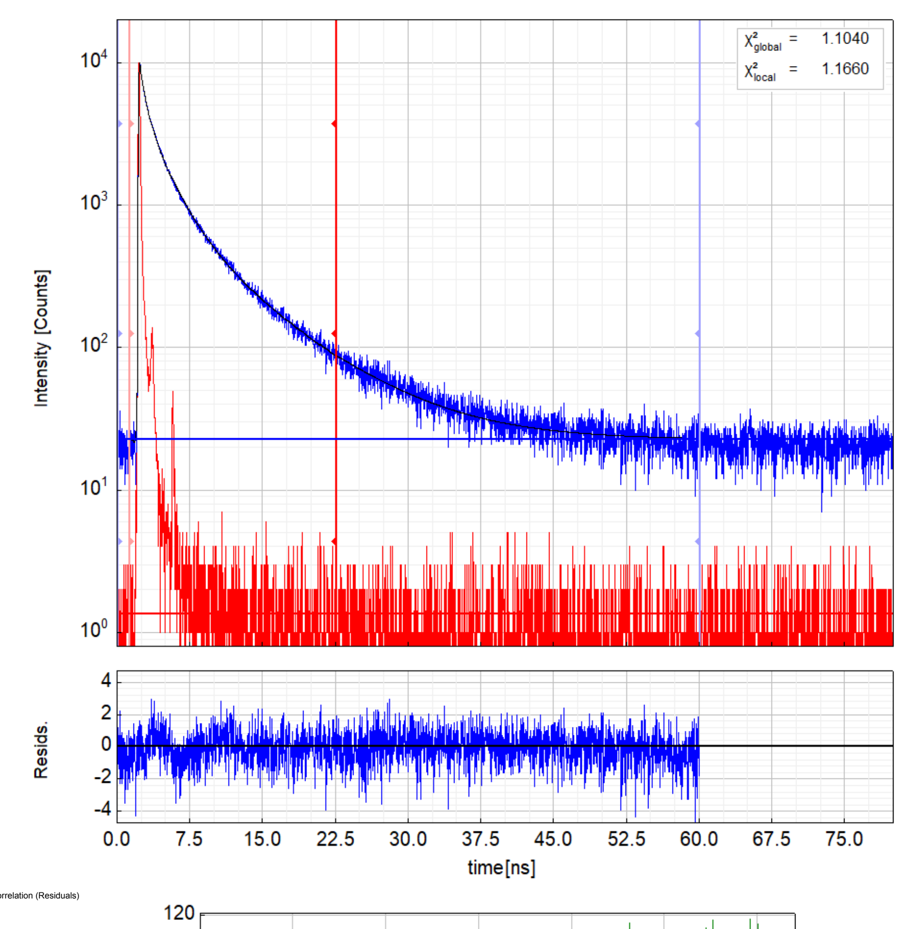

Autocorrelation Residuals

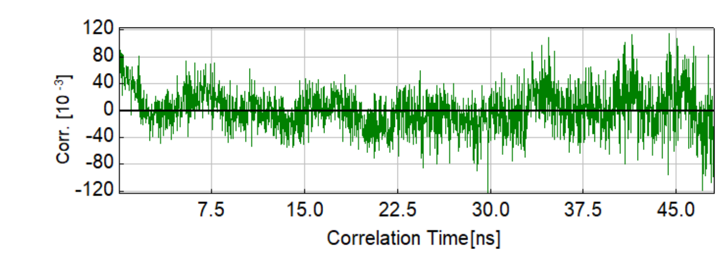

Fitted Parameters

Parameter	Value	Δ	δ
A_1[kCnts/Chnl]	0.9403	±0.0045	0.5%
τ_1[ns]	7.675	±0.012	0.2%
I_1[kCnts]	288.65	±0.96	0.3%
A_2[kCnts/Chnl]	3.912	±0.032	0.8%
τ_2[ns]	2.250	±0.013	0.6%
I_2[kCnts]	351.9	±2.8	0.8%
A_3[kCnts/Chnl]	6.794	±0.047	0.7%
τ_3[ns]	0.5216	±0.0058	1.1%
I_3[kCnts]	141.8	±1.8	1.2%
$Bkgr_{Dec}$[kCnts]	0.0226	±0.0002	0.8%
$Bkgr_{IRF}$[Cnts/Chnl]	1.348	±0.086	6.4%
$Shift_{IRF}$[ps]	11.72	±0.73	6.2%
A_{Scatt}[kCnts]	38.45	±0.47	1.2%
τ_{AvInt}[ns]	3.9382	±0.0049	0.1%
τ_{AvAmp}[ns]	1.6795	±0.0067	0.4%
λ_{Det}[nm]	500.0000	---	---

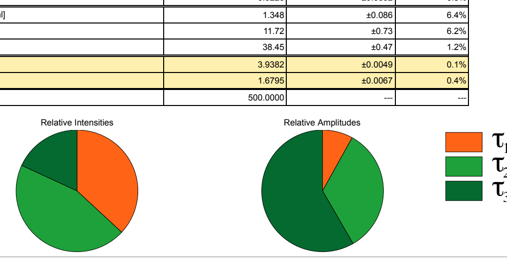

Data Set: 12 / 31

Decay: crv[12]; IRF: crv[0]

Fit

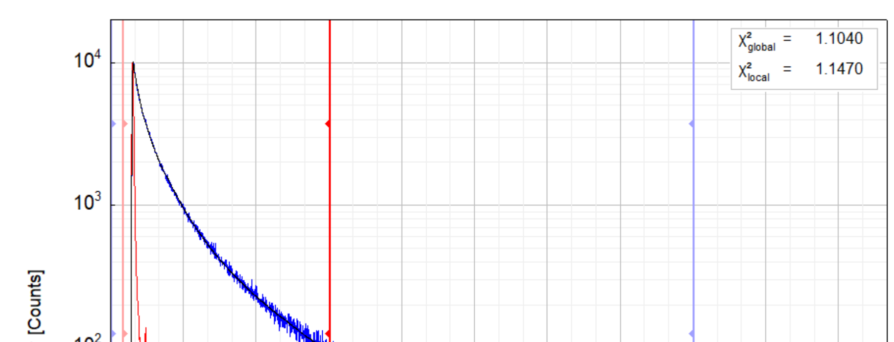

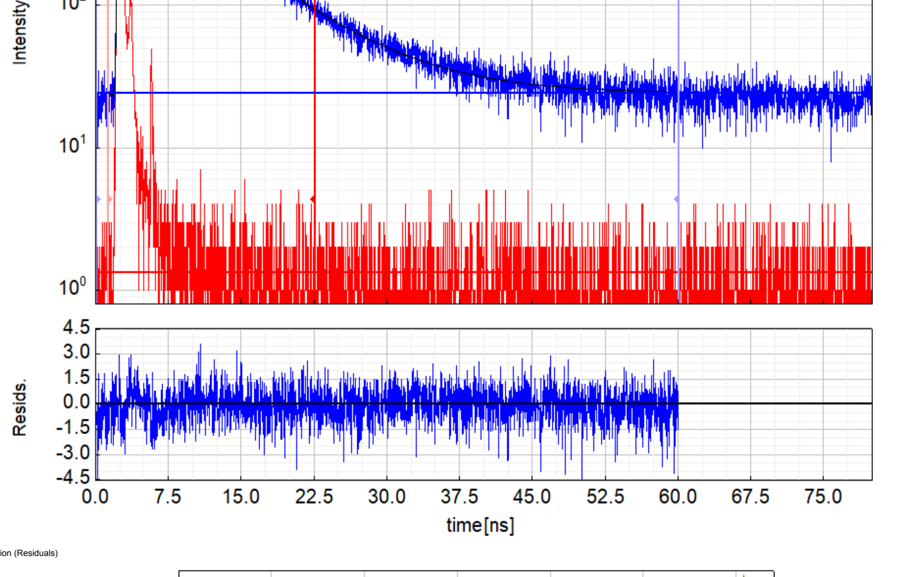

Autocorrelation (Residuals)

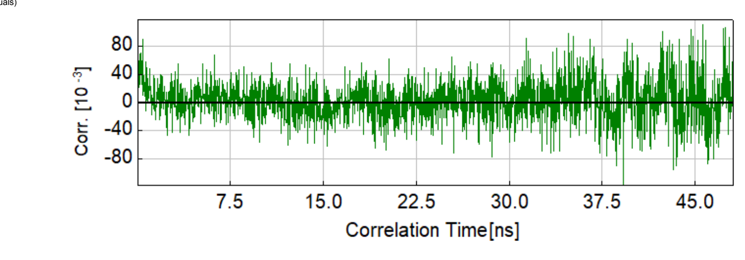

Fitted Parameters

Parameter	Value	Δ	δ
A_1[kCnts/Chnl]	0.9932	±0.0057	0.6%
τ_1[ns]	7.675	±0.012	0.2%
I_1[kCnts]	305.0	±1.9	0.6%
A_2[kCnts/Chnl]	4.149	±0.047	1.1%
τ_2[ns]	2.250	±0.013	0.6%
I_2[kCnts]	373.3	±2.5	0.7%
A_3[kCnts/Chnl]	6.703	±0.064	1.0%
τ_3[ns]	0.5216	±0.0058	1.1%
I_3[kCnts]	139.9	±2.1	1.5%
$Bkgr_{Dec}$[kCnts]	0.0242	±0.0002	0.5%
$Bkgr_{IRF}$[Cnts/Chnl]	1.32	±0.13	9.5%
$Shift_{IRF}$[ps]	11.69	±0.60	5.1%
A_{Scatt}[kCnts]	39.21	±0.50	1.3%
τ_{AvInt}[ns]	3.977	±0.012	0.3%
τ_{AvAmp}[ns]	1.727	±0.011	0.6%
λ_{Det}[nm]	510.0000	---	---

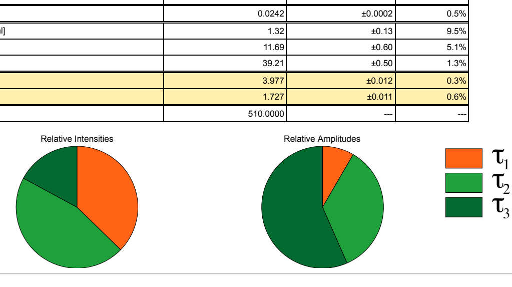

Data Set: 13 / 31

Decay: crv[13]; IRF: crv[0]

Fit

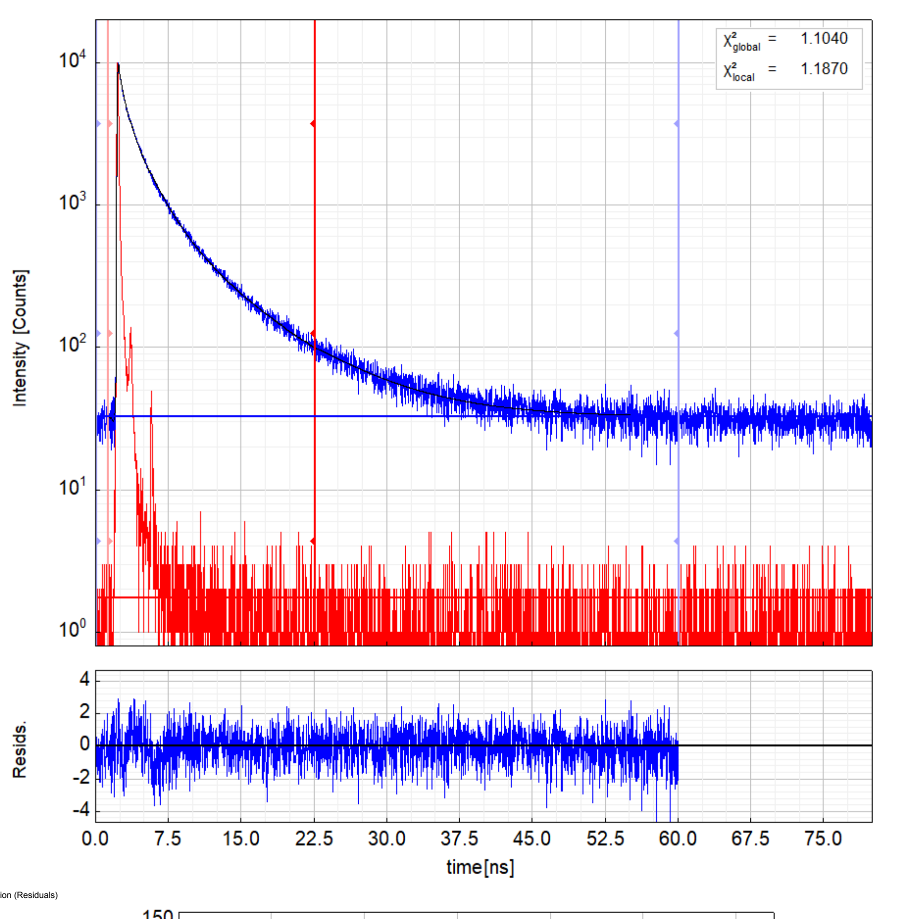

Autocorrelation (Residuals)

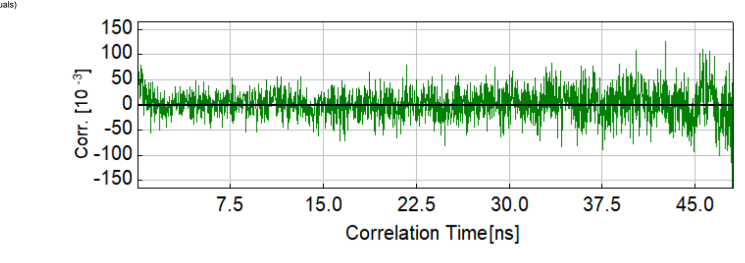

Fitted Parameters

Parameter	Value	Δ	δ
A_1[kCnts/Chnl]	1.0190	±0.0083	0.8%
τ_1[ns]	7.675	±0.012	0.2%
I_1[kCnts]	312.9	±2.5	0.8%
A_2[kCnts/Chnl]	4.220	±0.044	1.0%
τ_2[ns]	2.250	±0.013	0.6%
I_2[kCnts]	379.6	±2.1	0.5%
A_3[kCnts/Chnl]	6.320	±0.069	1.1%
τ_3[ns]	0.5216	±0.0058	1.1%
I_3[kCnts]	131.9	±2.1	1.6%
$Bkgr_{Dec}$[kCnts]	0.0324	±0.0002	0.4%
$Bkgr_{IRF}$[Cnts/Chnl]	1.745	±0.096	5.5%
$Shift_{IRF}$[ps]	11.56	±0.84	7.2%
A_{Scatt}[kCnts]	37.66	±0.78	2.1%
τ_{AvInt}[ns]	4.033	±0.020	0.5%
τ_{AvAmp}[ns]	1.783	±0.011	0.6%
λ_{Det}[nm]	520.0000	---	---

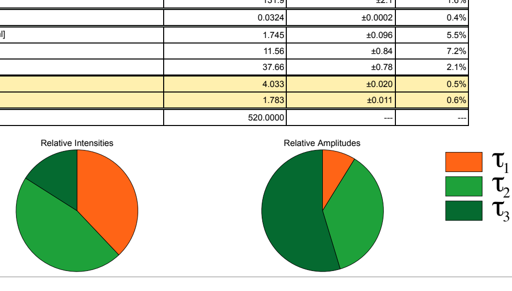

Data Set: 14 / 31

Decay: crv[14]; IRF: crv[0]

Fit

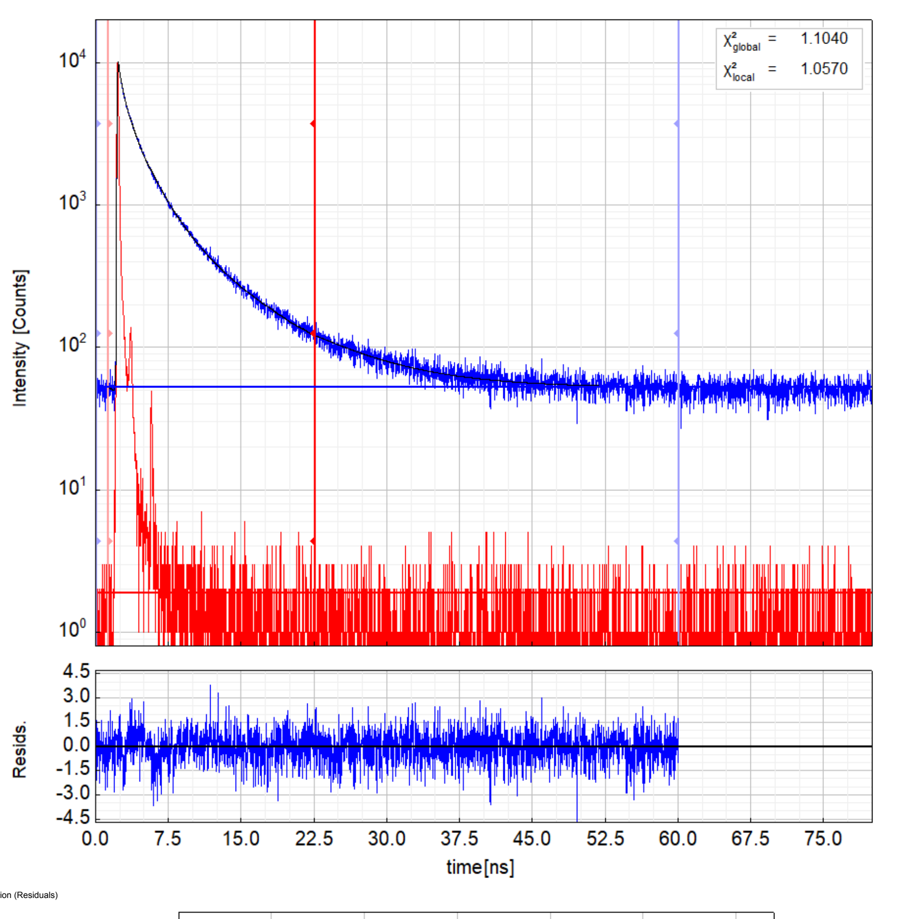

Autocorrelation (Residuals)

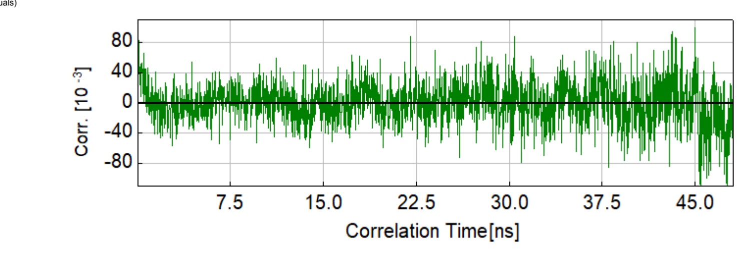

Fitted Parameters

Parameter	Value	Δ	δ
A_1[kCnts/Chnl]	1.0643	±0.0062	0.6%
τ_1[ns]	7.675	±0.012	0.2%
I_1[kCnts]	326.8	±2.1	0.6%
A_2[kCnts/Chnl]	4.474	±0.062	1.4%
τ_2[ns]	2.250	±0.013	0.6%
I_2[kCnts]	402.5	±3.7	0.9%
A_3[kCnts/Chnl]	6.117	±0.043	0.7%
τ_3[ns]	0.5216	±0.0058	1.1%
I_3[kCnts]	127.7	±1.2	0.9%
$Bkgr_{Dec}$[kCnts]	0.0522	±0.0003	0.5%
$Bkgr_{IRF}$[Cnts/Chnl]	1.89	±0.13	6.4%
$Shift_{IRF}$[ps]	13.60	±0.42	3.1%
A_{Scatt}[kCnts]	38.50	±0.59	1.5%
τ_{AvInt}[ns]	4.061	±0.013	0.3%
τ_{AvAmp}[ns]	1.8381	±0.0099	0.5%
λ_{Det}[nm]	530.0000	---	---

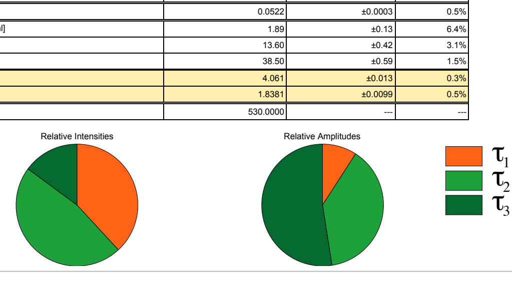

Data Set: 15 / 31

Decay: crv[15]; IRF: crv[0]

Fit

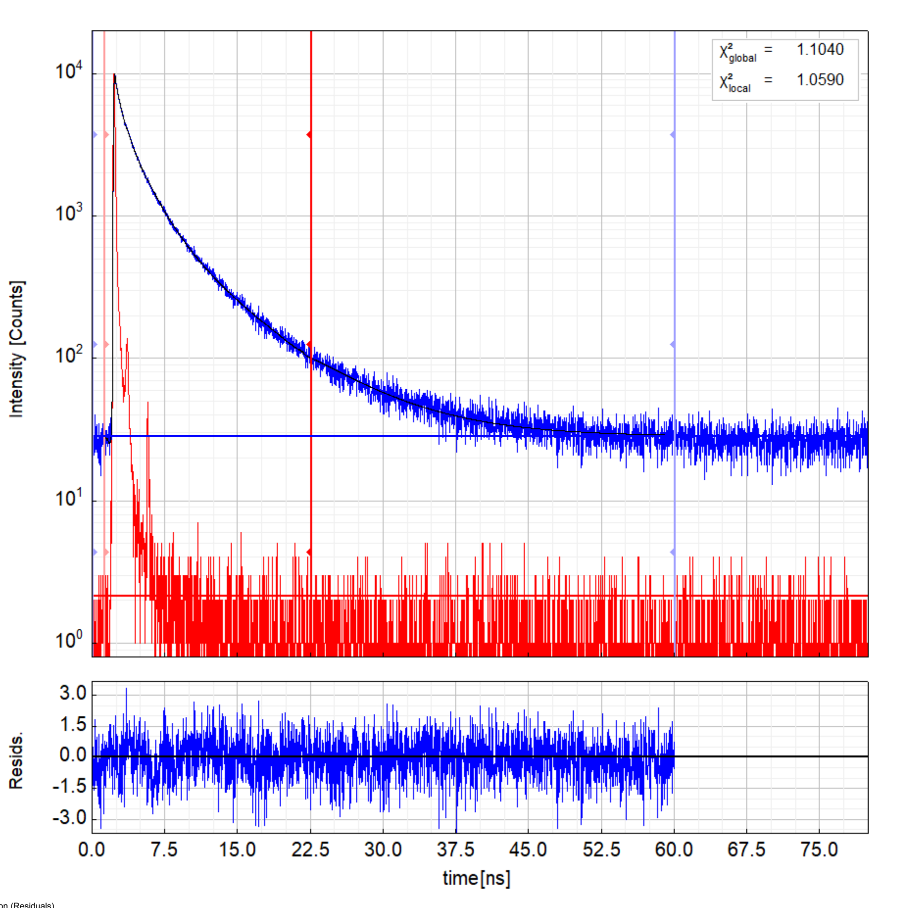

Autocorrelation (Residuals)

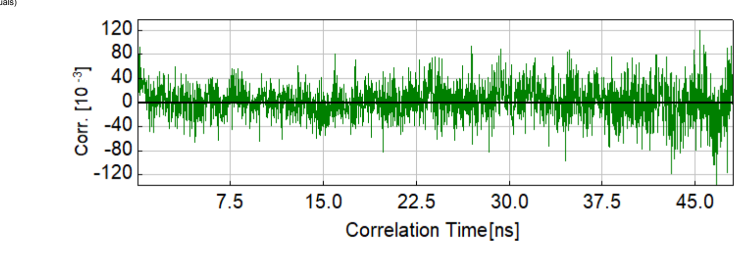

Fitted Parameters

Parameter	Value	Δ	δ
A_1[kCnts/Chnl]	1.146	±0.012	1.0%
τ_1[ns]	7.675	±0.012	0.2%
I_1[kCnts]	351.6	±3.9	1.1%
A_2[kCnts/Chnl]	4.563	±0.040	0.9%
τ_2[ns]	2.250	±0.013	0.6%
I_2[kCnts]	410.5	±3.0	0.7%
A_3[kCnts/Chnl]	5.913	±0.089	1.5%
τ_3[ns]	0.5216	±0.0058	1.1%
I_3[kCnts]	123.4	±2.7	2.2%
$Bkgr_{Dec}$[kCnts]	0.0284	±0.0002	0.5%
$Bkgr_{IRF}$[Cnts/Chnl]	2.13	±0.11	5.2%
$Shift_{IRF}$[ps]	13.62	±0.59	4.3%
A_{Scatt}[kCnts]	37.91	±0.75	2.0%
τ_{AvInt}[ns]	4.163	±0.013	0.3%
τ_{AvAmp}[ns]	1.9049	±0.0076	0.4%
λ_{Det}[nm]	540.0000	---	---

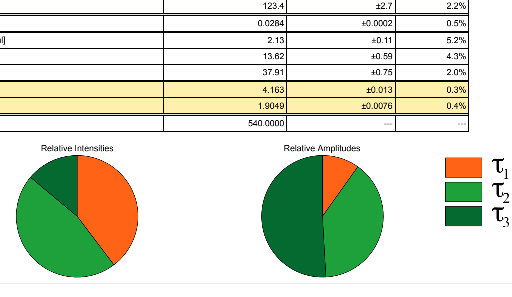

Data Set: 16 / 31

Decay: crv[16]; IRF: crv[0]

Fit

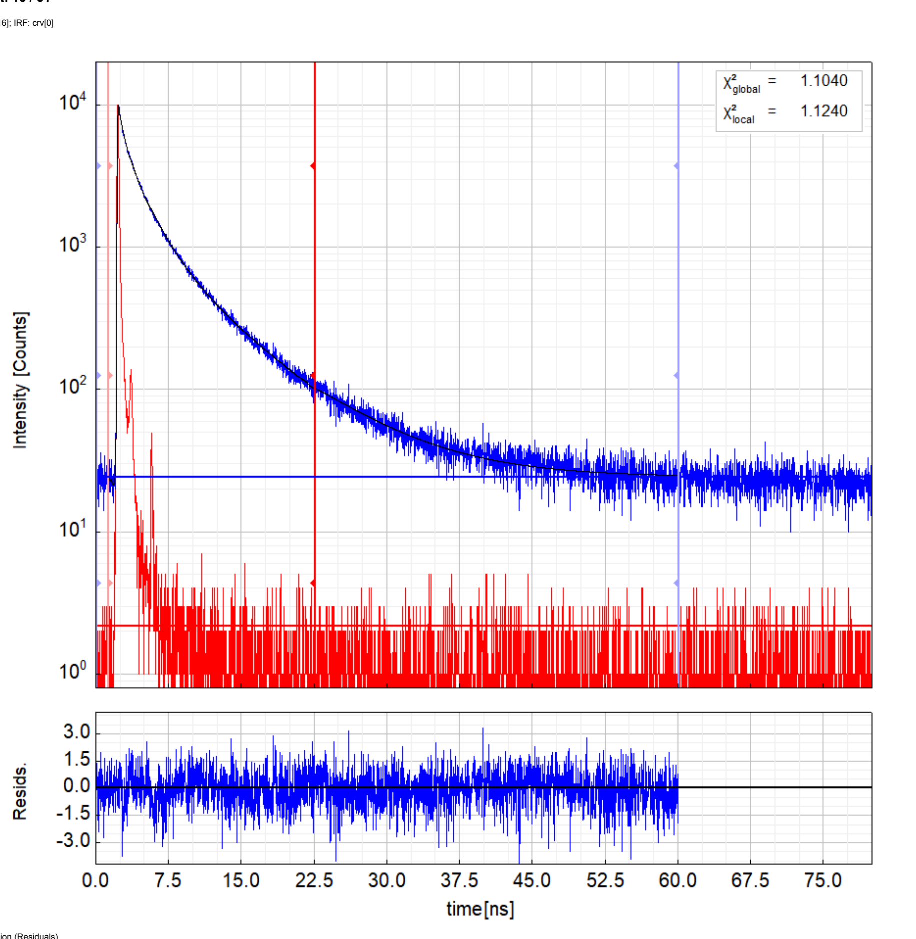

Autocorrelation (Residuals)

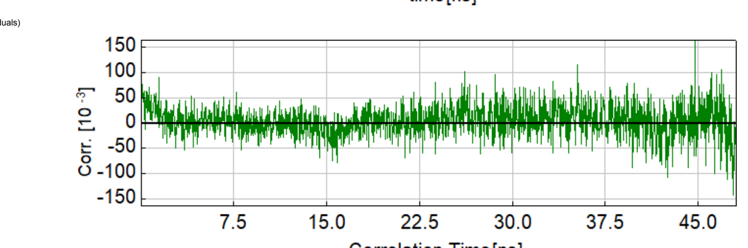

Fitted Parameters

Parameter	Value	Δ	δ
A_1[kCnts/Chnl]	1.2246	±0.0093	0.8%
τ_1[ns]	7.675	±0.012	0.2%
I_1[kCnts]	376.0	±2.1	0.6%
A_2[kCnts/Chnl]	4.545	±0.038	0.8%
τ_2[ns]	2.250	±0.013	0.6%
I_2[kCnts]	408.9	±1.6	0.4%
A_3[kCnts/Chnl]	5.708	±0.052	0.9%
τ_3[ns]	0.5216	±0.0058	1.1%
I_3[kCnts]	119.1	±2.7	2.2%
$Bkgr_{Dec}$[kCnts]	0.0243	±0.0002	0.8%
$Bkgr_{IRF}$[Cnts/Chnl]	2.164	±0.094	4.3%
$Shift_{IRF}$[ps]	14.56	±0.44	3.0%
A_{Scatt}[kCnts]	38.54	±0.43	1.1%
τ_{AvInt}[ns]	4.279	±0.011	0.3%
τ_{AvAmp}[ns]	1.9689	±0.0089	0.4%
λ_{Det}[nm]	550.0000	---	---

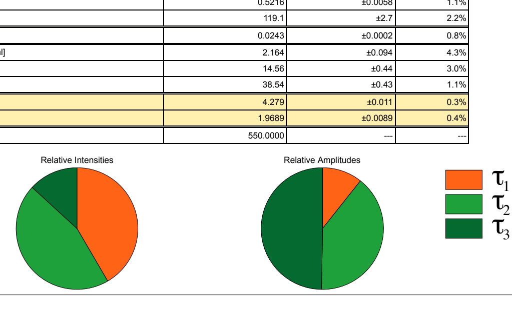

Data Set: 17 / 31

Decay: crv[17]; IRF: crv[0]

Fit

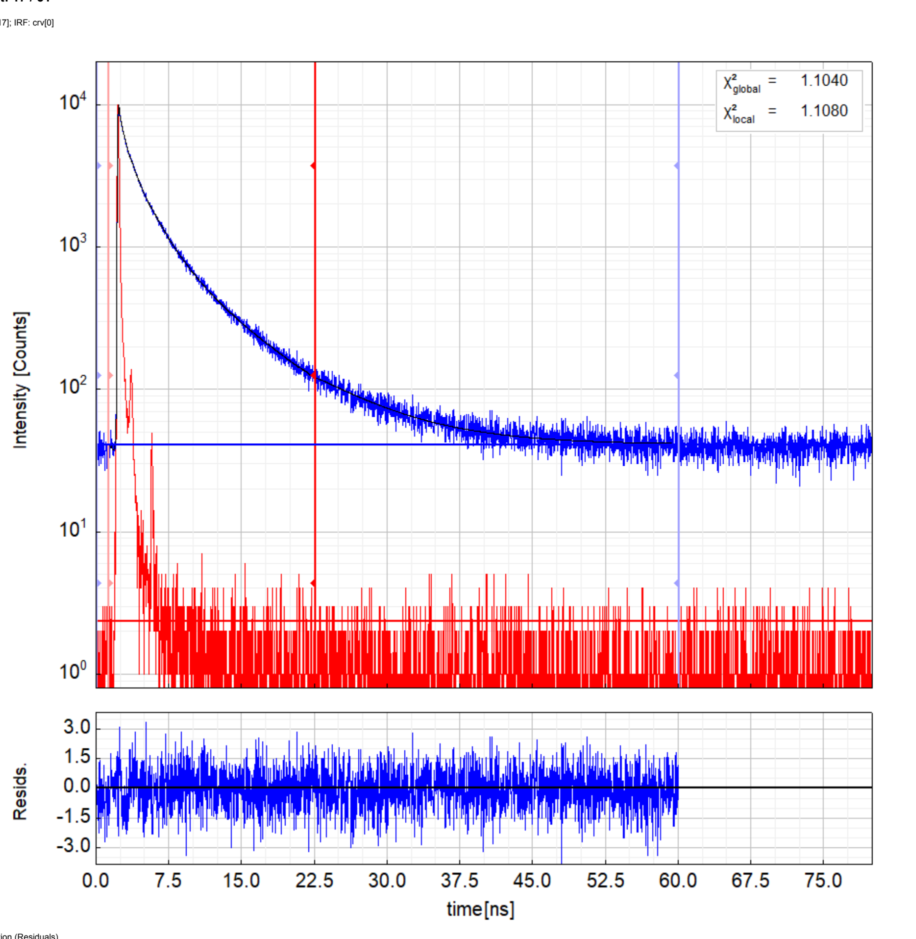

Autocorrelation (Residuals)

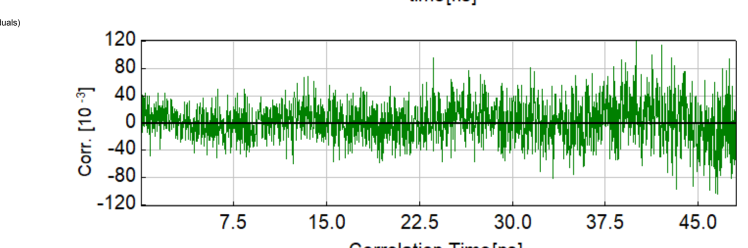

Fitted Parameters

Parameter	Value	Δ	δ
A_1[kCnts/Chnl]	1.294	±0.018	1.3%
τ_1[ns]	7.675	±0.012	0.2%
I_1[kCnts]	397.2	±5.0	1.2%
A_2[kCnts/Chnl]	4.475	±0.048	1.1%
τ_2[ns]	2.250	±0.013	0.6%
I_2[kCnts]	402.6	±4.5	1.1%
A_3[kCnts/Chnl]	5.45	±0.11	2.0%
τ_3[ns]	0.5216	±0.0058	1.1%
I_3[kCnts]	113.6	±1.9	1.6%
$Bkgr_{Dec}$[kCnts]	0.0410	±0.0003	0.5%
$Bkgr_{IRF}$[Cnts/Chnl]	2.36	±0.19	7.8%
$Shift_{IRF}$[ps]	14.58	±0.79	5.4%
A_{Scatt}[kCnts]	39.40	±0.96	2.4%
τ_{AvInt}[ns]	4.394	±0.019	0.4%
τ_{AvAmp}[ns]	2.037	±0.014	0.6%
λ_{Det}[nm]	560.0000	---	---

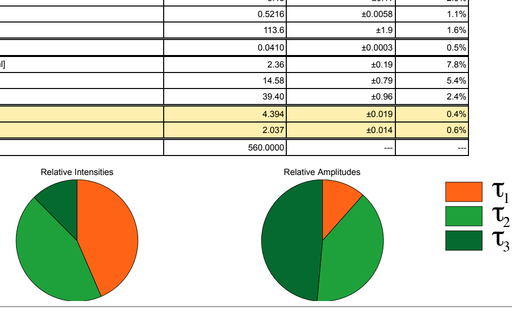

Data Set: 18 / 31

Decay: crv[18]; IRF: crv[0]

Fit

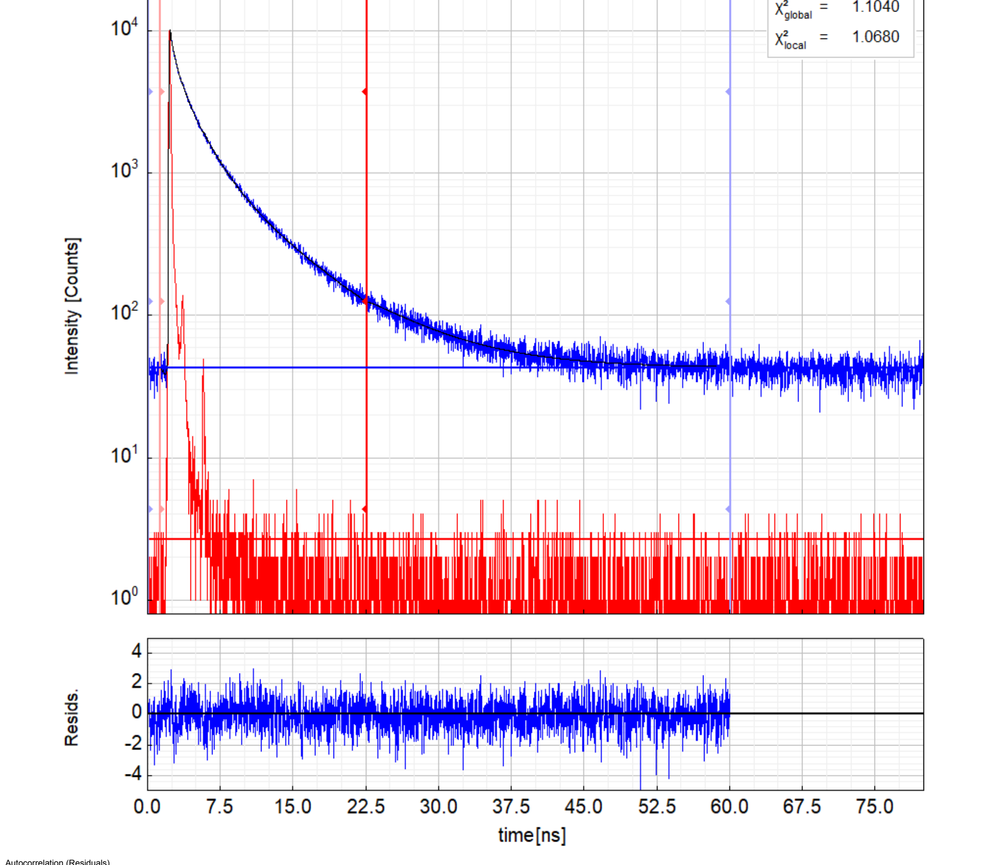

Autocorrelation Residuals

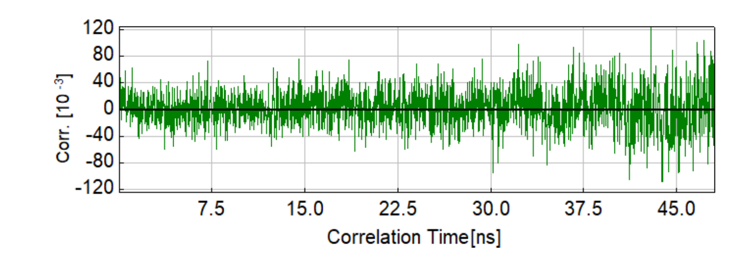

Fitted Parameters

Parameter	Value	Δ	δ
A_1[kCnts/Chnl]	1.381	±0.017	1.2%
τ_1[ns]	7.675	±0.012	0.2%
I_1[kCnts]	424.0	±4.5	1.1%
A_2[kCnts/Chnl]	4.514	±0.024	0.5%
τ_2[ns]	2.250	±0.013	0.6%
I_2[kCnts]	406.1	±4.6	1.1%
A_3[kCnts/Chnl]	5.489	±0.072	1.3%
τ_3[ns]	0.5216	±0.0058	1.1%
I_3[kCnts]	114.6	±1.9	1.6%
$Bkgr_{Dec}$[kCnts]	0.0430	±0.0002	0.4%
$Bkgr_{IRF}$[Cnts/Chnl]	2.69	±0.21	7.5%
$Shift_{IRF}$[ps]	15.46	±0.56	3.6%
A_{Scatt}[kCnts]	40.12	±0.48	1.2%
τ_{AvInt}[ns]	4.475	±0.019	0.4%
τ_{AvAmp}[ns]	2.0744	±0.0048	0.2%
λ_{Det}[nm]	570.0000	---	---

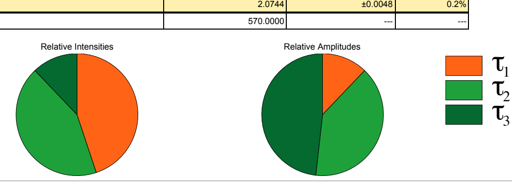

Data Set: 19 / 31

Decay: crv[19]; IRF: crv[0]

Fit

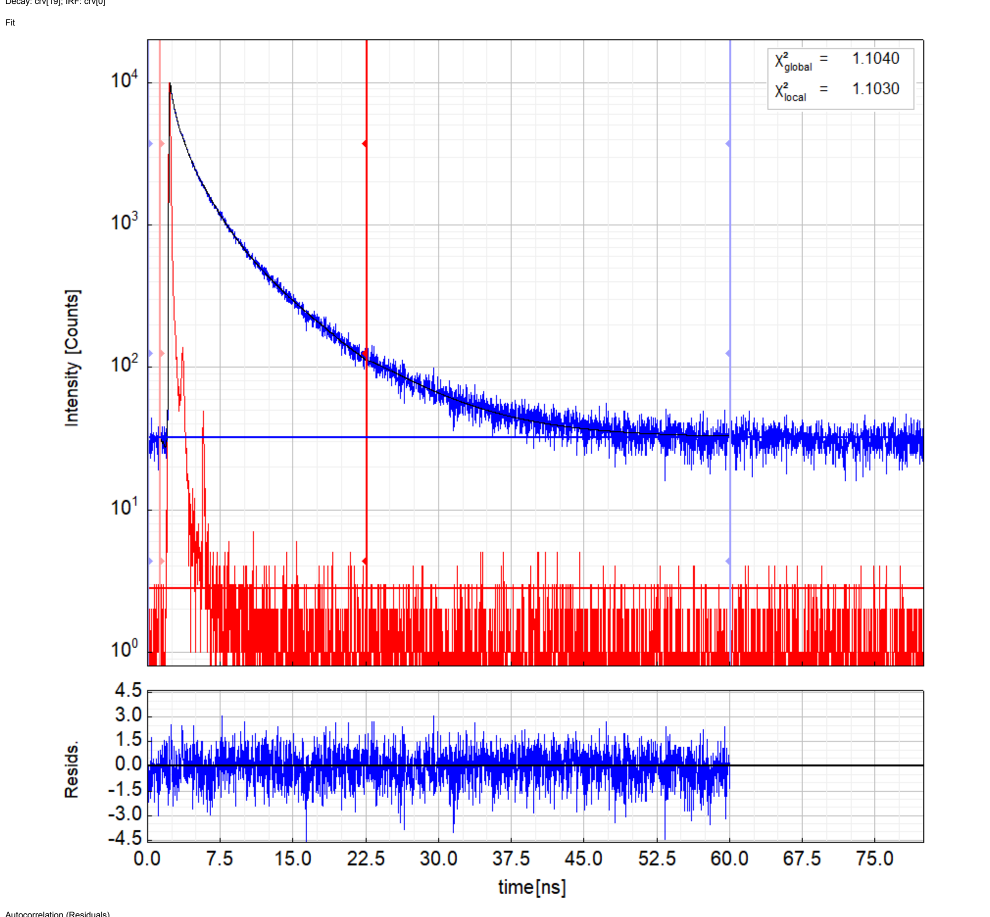

Autocorrelation Residuals

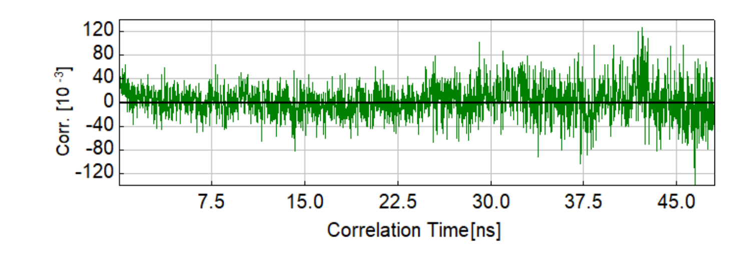

Fitted Parameters

Parameter	Value	Δ	δ
A_1[kCnts/Chnl]	1.371	±0.018	1.3%
τ_1[ns]	7.675	±0.012	0.2%
I_1[kCnts]	420.8	±4.6	1.1%
A_2[kCnts/Chnl]	4.467	±0.024	0.5%
τ_2[ns]	2.250	±0.013	0.6%
I_2[kCnts]	401.9	±1.7	0.4%
A_3[kCnts/Chnl]	5.317	±0.084	1.6%
τ_3[ns]	0.5216	±0.0058	1.1%
I_3[kCnts]	111.0	±1.5	1.3%
$Bkgr_{Dec}$[kCnts]	0.0322	±0.0002	0.4%
$Bkgr_{IRF}$[Cnts/Chnl]	2.80	±0.12	4.1%
$Shift_{IRF}$[ps]	16.32	±0.99	6.1%
A_{Scatt}[kCnts]	41.15	±0.84	2.0%
τ_{AvInt}[ns]	4.490	±0.014	0.3%
τ_{AvAmp}[ns]	2.0924	±0.0093	0.4%
λ_{Det}[nm]	580.0000	---	---

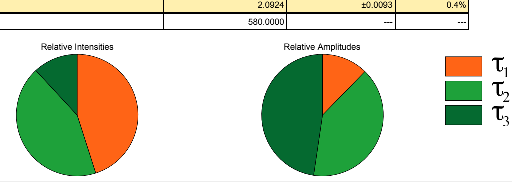

Data Set: 20 / 31

Decay: crv[20]; IRF: crv[0]

Fit

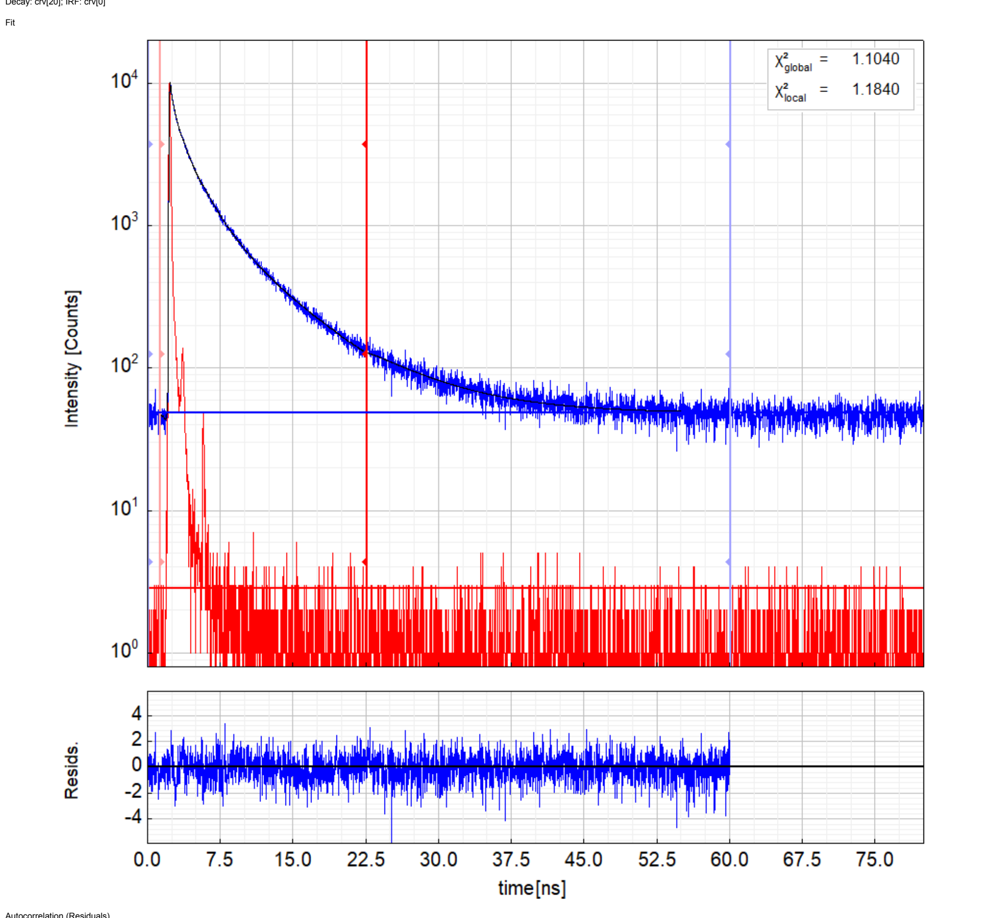

Autocorrelation Residuals

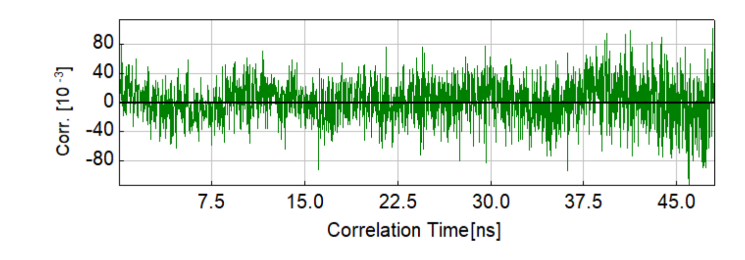

Fitted Parameters

Parameter	Value	Δ	δ
A_1[kCnts/Chnl]	1.3639	±0.0037	0.3%
τ_1[ns]	7.675	±0.012	0.2%
I_1[kCnts]	418.7	±1.5	0.3%
A_2[kCnts/Chnl]	4.364	±0.051	1.2%
τ_2[ns]	2.250	±0.013	0.6%
I_2[kCnts]	392.6	±2.8	0.7%
A_3[kCnts/Chnl]	5.268	±0.081	1.5%
τ_3[ns]	0.5216	±0.0058	1.1%
I_3[kCnts]	109.9	±1.5	1.3%
$Bkgr_{Dec}$[kCnts]	0.0484	±0.0002	0.3%
$Bkgr_{IRF}$[Cnts/Chnl]	2.85	±0.11	3.6%
$Shift_{IRF}$[ps]	16.78	±0.17	1.0%
A_{Scatt}[kCnts]	42.64	±0.30	0.7%
τ_{AvInt}[ns]	4.510	±0.016	0.3%
τ_{AvAmp}[ns]	2.095	±0.013	0.6%
λ_{Det}[nm]	590.0000	---	---

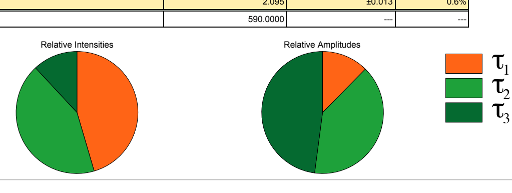

Data Set: 21 / 31

Decay: crv[21]; IRF: crv[0]

Fit

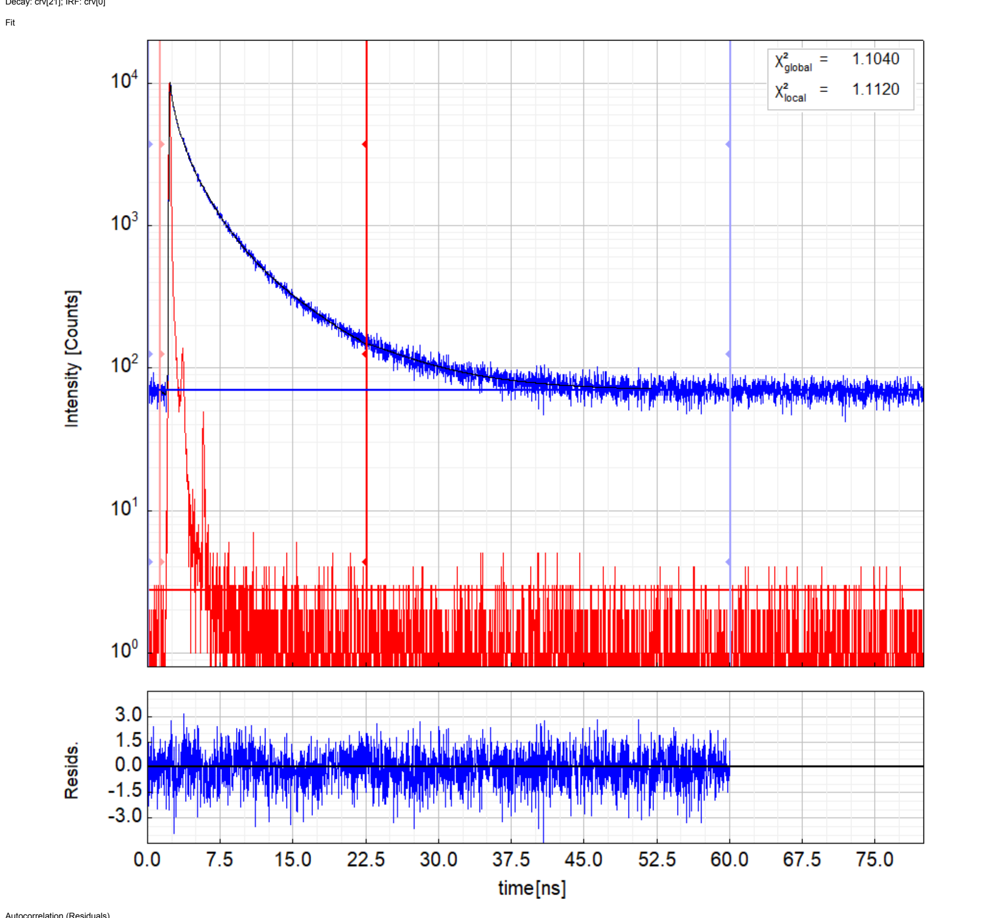

Autocorrelation Residuals

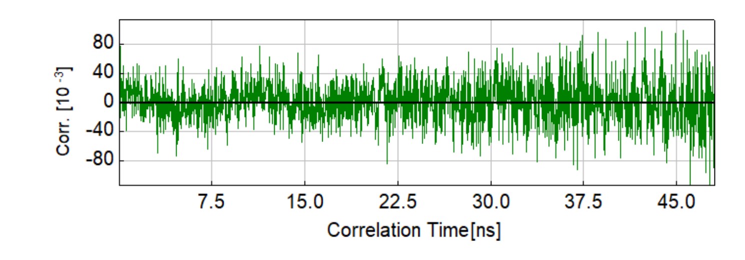

Fitted Parameters

Parameter	Value	Δ	δ
A_1[kCnts/Chnl]	1.3253	±0.0074	0.6%
τ_1[ns]	7.675	±0.012	0.2%
I_1[kCnts]	406.9	±2.2	0.5%
A_2[kCnts/Chnl]	4.228	±0.025	0.6%
τ_2[ns]	2.250	±0.013	0.6%
I_2[kCnts]	380.38	±0.99	0.3%
A_3[kCnts/Chnl]	5.275	±0.065	1.2%
τ_3[ns]	0.5216	±0.0058	1.1%
I_3[kCnts]	110.05	±0.70	0.6%
$Bkgr_{Dec}$[kCnts]	0.0696	±0.0002	0.2%
$Bkgr_{IRF}$[Cnts/Chnl]	2.742	±0.097	3.5%
$Shift_{IRF}$[ps]	16.85	±0.31	1.8%
A_{Scatt}[kCnts]	43.27	±0.82	1.9%
τ_{AvInt}[ns]	4.498	±0.013	0.3%
τ_{AvAmp}[ns]	2.072	±0.017	0.8%
λ_{Det}[nm]	600.0000	---	---

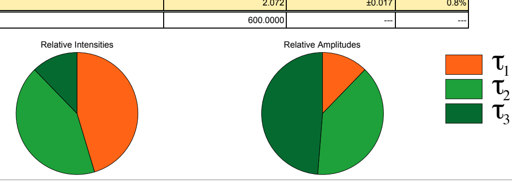

Data Set: 22 / 31

Decay: crv[22]; IRF: crv[0]

Fit

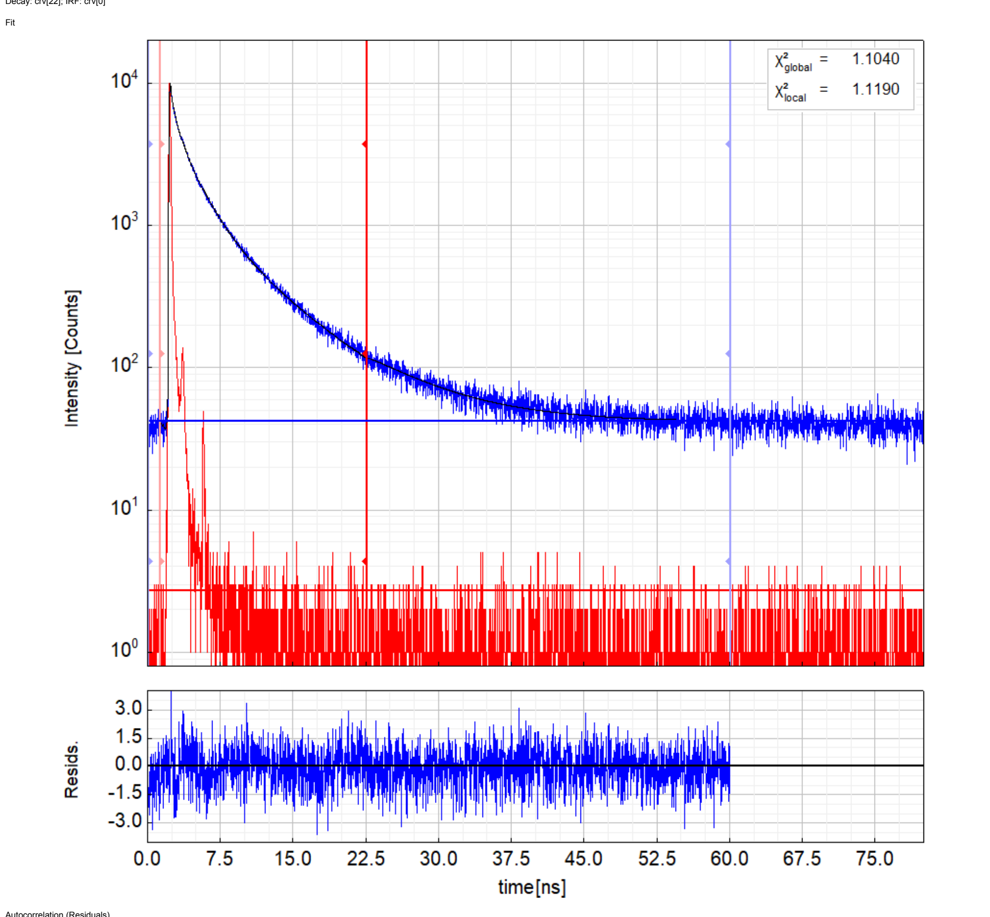

Autocorrelation Residuals

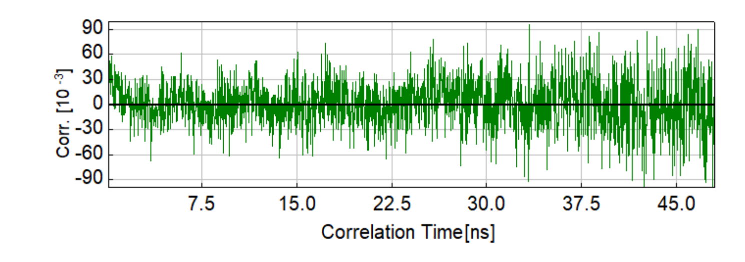

Fitted Parameters

Parameter	Value	Δ	δ
A_1[kCnts/Chnl]	1.2764	±0.0084	0.7%
τ_1[ns]	7.675	±0.012	0.2%
I_1[kCnts]	391.9	±2.3	0.6%
A_2[kCnts/Chnl]	4.155	±0.040	0.9%
τ_2[ns]	2.250	±0.013	0.6%
I_2[kCnts]	373.8	±2.0	0.5%
A_3[kCnts/Chnl]	5.185	±0.079	1.5%
τ_3[ns]	0.5216	±0.0058	1.1%
I_3[kCnts]	108.2	±2.5	2.2%
$Bkgr_{Dec}$[kCnts]	0.0418	±0.0002	0.5%
$Bkgr_{IRF}$[Cnts/Chnl]	2.73	±0.11	3.9%
$Shift_{IRF}$[ps]	17.43	±0.31	1.8%
A_{Scatt}[kCnts]	44.58	±0.68	1.5%
τ_{AvInt}[ns]	4.469	±0.013	0.3%
τ_{AvAmp}[ns]	2.058	±0.011	0.5%
λ_{Det}[nm]	610.0000	---	---

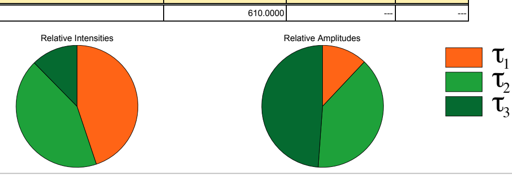

Data Set: 23 / 31

Decay: crv[23]; IRF: crv[0]

Fit

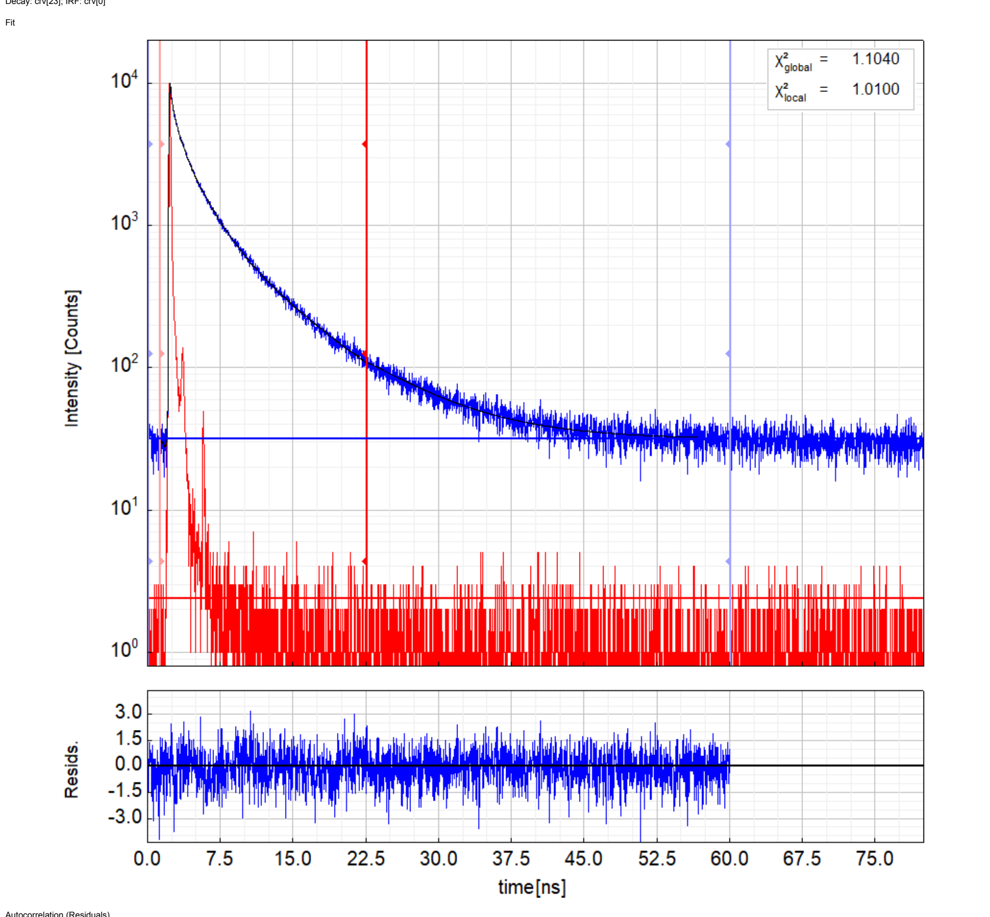

Autocorrelation Residuals

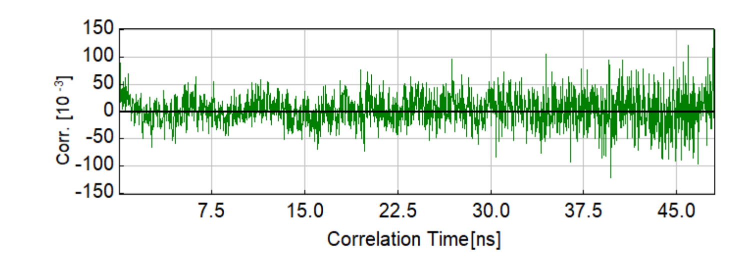

Fitted Parameters

Parameter	Value	Δ	δ
A_1[kCnts/Chnl]	1.2491	±0.0041	0.3%
τ_1[ns]	7.675	±0.012	0.2%
I_1[kCnts]	383.5	±1.8	0.5%
A_2[kCnts/Chnl]	3.956	±0.023	0.6%
τ_2[ns]	2.250	±0.013	0.6%
I_2[kCnts]	355.9	±1.3	0.4%
A_3[kCnts/Chnl]	4.978	±0.081	1.6%
τ_3[ns]	0.5216	±0.0058	1.1%
I_3[kCnts]	103.9	±2.7	2.5%
$Bkgr_{Dec}$[kCnts]	0.0314	±0.0002	0.4%
$Bkgr_{IRF}$[Cnts/Chnl]	2.39	±0.12	4.8%
$Shift_{IRF}$[ps]	19.11	±0.53	2.7%

Parameter	Value	Δ	δ
A_{Scatt}[kCnts]	45.60	±0.85	1.9%
τ_{AvInt}[ns]	4.505	±0.013	0.3%
τ_{AvAmp}[ns]	2.071	±0.016	0.8%
λ_{Det}[nm]	620.0000	---	---

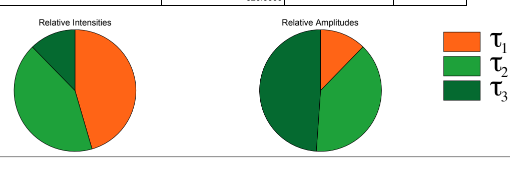

Data Set: 24 / 31

Decay: crv[24]; IRF: crv[0]

Fit

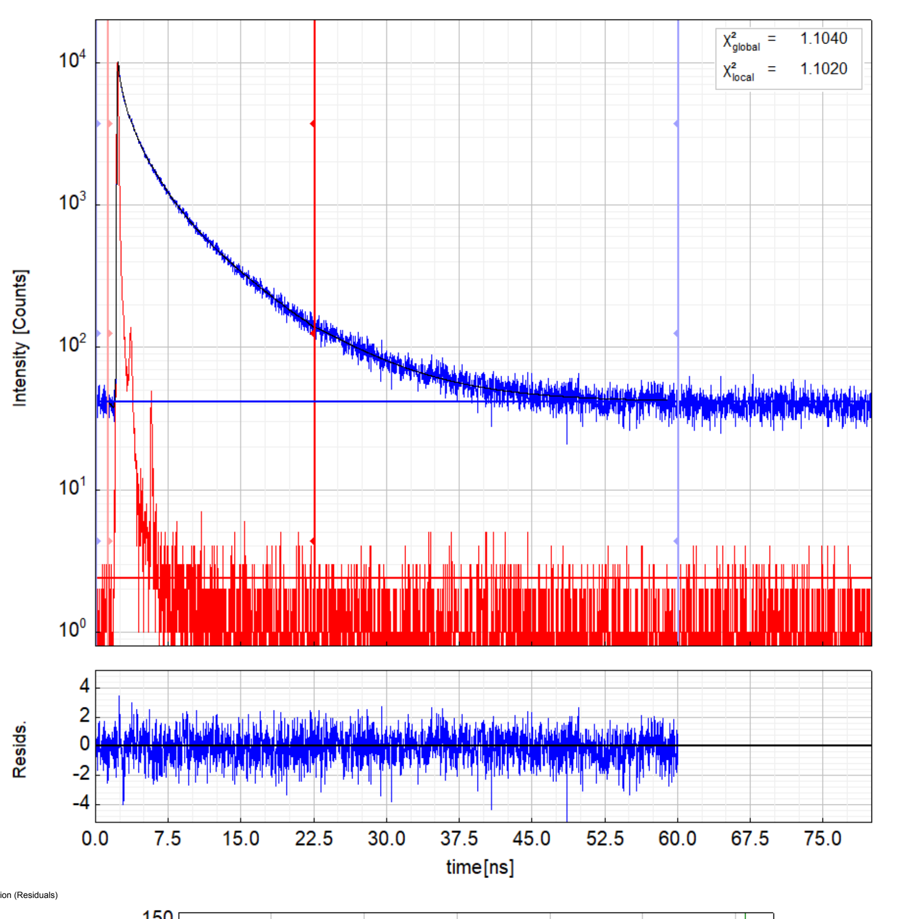

Autocorrelation (Residuals)

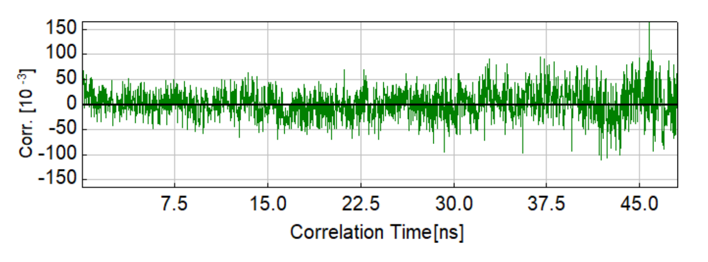

Fitted Parameters

Parameter	Value	Δ	δ
A_1[kCnts/Chnl]	1.547	±0.018	1.1%
τ_1[ns]	7.675	±0.012	0.2%
I_1[kCnts]	474.7	±4.1	0.9%
A_2[kCnts/Chnl]	3.920	±0.031	0.8%
τ_2[ns]	2.250	±0.013	0.6%
I_2[kCnts]	352.7	±2.7	0.8%
A_3[kCnts/Chnl]	4.989	±0.084	1.7%
τ_3[ns]	0.5216	±0.0058	1.1%
I_3[kCnts]	104.1	±1.8	1.6%
$Bkgr_{Dec}$[kCnts]	0.0415	±0.0003	0.6%
$Bkgr_{IRF}$[Cnts/Chnl]	2.38	±0.13	5.2%
$Shift_{IRF}$[ps]	19.13	±0.58	3.0%
A_{Scatt}[kCnts]	45.79	±0.85	1.8%
τ_{AvInt}[ns]	4.822	±0.011	0.2%
τ_{AvAmp}[ns]	2.228	±0.013	0.6%
λ_{Det}[nm]	630.0000	---	---

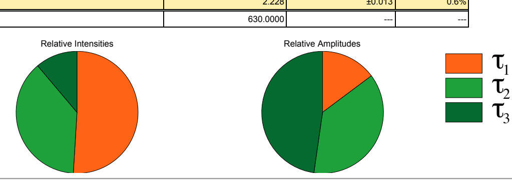

Data Set: 25 / 31

Decay: crv[25]; IRF: crv[0]

Fit

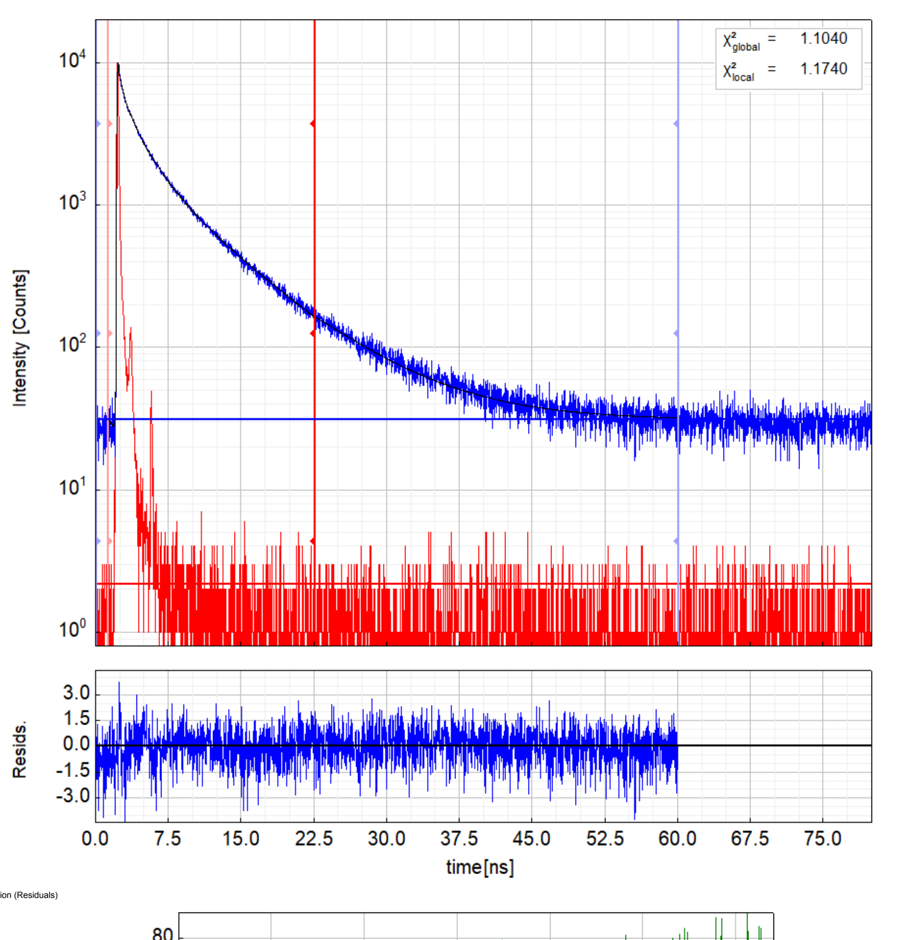

Autocorrelation (Residuals)

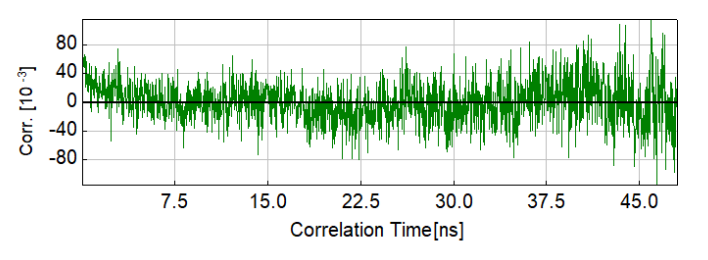

Fitted Parameters

Parameter	Value	Δ	δ
A_1[kCnts/Chnl]	2.044	±0.013	0.6%
τ_1[ns]	7.675	±0.012	0.2%
I_1[kCnts]	627.5	±3.7	0.6%
A_2[kCnts/Chnl]	3.949	±0.039	1.0%
τ_2[ns]	2.250	±0.013	0.6%
I_2[kCnts]	355.3	±3.4	0.9%
A_3[kCnts/Chnl]	4.426	±0.075	1.7%
τ_3[ns]	0.5216	±0.0058	1.1%
I_3[kCnts]	92.4	±2.9	3.1%
$Bkgr_{Dec}$[kCnts]	0.0310	±0.0002	0.4%
$Bkgr_{IRF}$[Cnts/Chnl]	2.17	±0.18	7.9%
$Shift_{IRF}$[ps]	19.96	±0.40	2.0%
A_{Scatt}[kCnts]	44.64	±0.69	1.5%
τ_{AvInt}[ns]	5.2677	±0.0092	0.2%
τ_{AvAmp}[ns]	2.5798	±0.0097	0.4%
λ_{Det}[nm]	640.0000	---	---

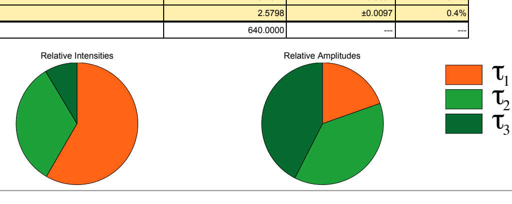

Data Set: 26 / 31

Decay: crv[26]; IRF: crv[0]

Fit

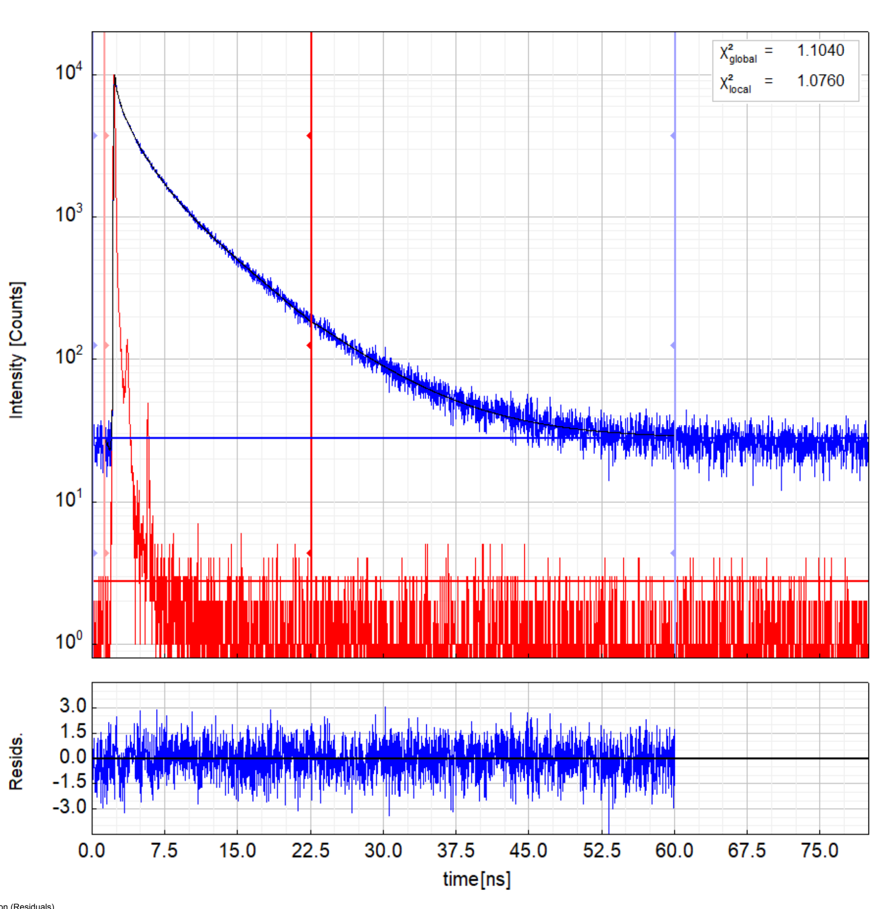

Autocorrelation (Residuals)

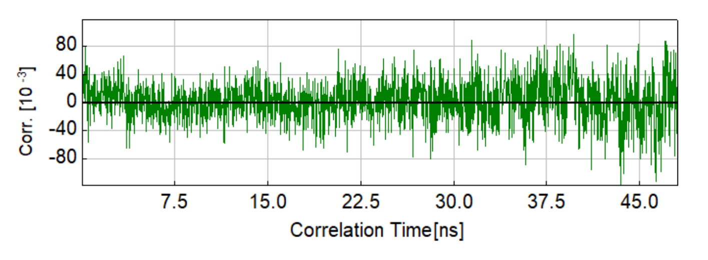

Fitted Parameters

Parameter	Value	Δ	δ
A_1[kCnts/Chnl]	2.494	±0.015	0.6%
τ_1[ns]	7.675	±0.012	0.2%
I_1[kCnts]	765.4	±2.2	0.3%
A_2[kCnts/Chnl]	4.119	±0.033	0.8%
τ_2[ns]	2.250	±0.013	0.6%
I_2[kCnts]	370.6	±2.0	0.5%
A_3[kCnts/Chnl]	3.728	±0.070	1.9%
τ_3[ns]	0.5216	±0.0058	1.1%
I_3[kCnts]	77.8	±2.2	2.8%
$Bkgr_{Dec}$[kCnts]	0.0278	±0.0003	0.9%
$Bkgr_{IRF}$[Cnts/Chnl]	2.741	±0.069	2.5%
$Shift_{IRF}$[ps]	19.98	±0.55	2.7%
A_{Scatt}[kCnts]	44.62	±0.72	1.6%
τ_{AvInt}[ns]	5.5601	±0.0074	0.1%
τ_{AvAmp}[ns]	2.935	±0.012	0.4%
λ_{Det}[nm]	650.0000	---	---

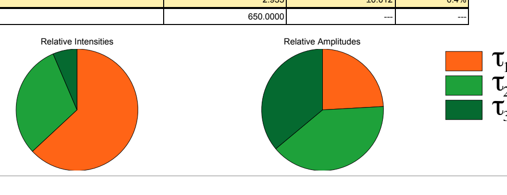

Data Set: 27 / 31

Decay: crv[27]; IRF: crv[0]

Fit

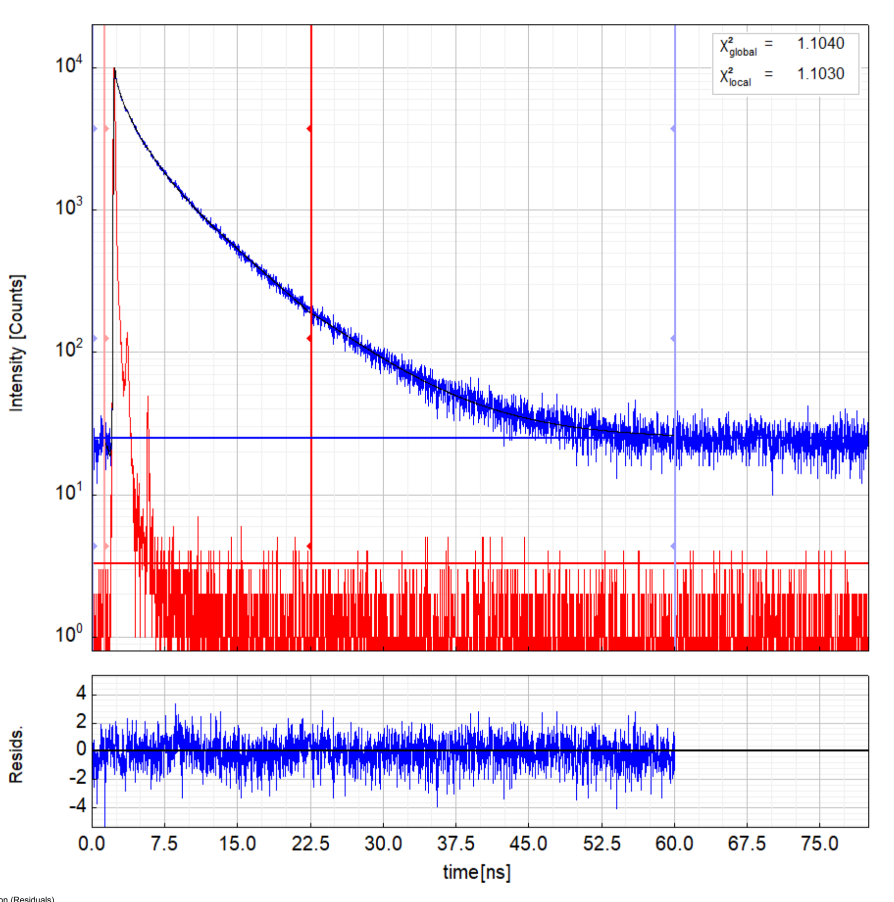

Autocorrelation (Residuals)

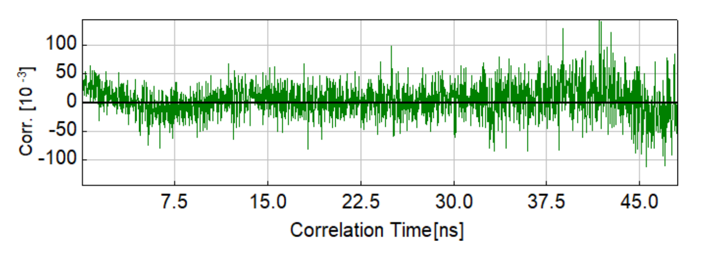

Fitted Parameters

Parameter	Value	Δ	δ
A_1[kCnts/Chnl]	2.710	±0.016	0.6%
τ_1[ns]	7.675	±0.012	0.2%
I_1[kCnts]	831.9	±3.2	0.4%
A_2[kCnts/Chnl]	4.252	±0.017	0.4%
τ_2[ns]	2.250	±0.013	0.6%
I_2[kCnts]	382.5	±2.7	0.7%
A_3[kCnts/Chnl]	3.534	±0.073	2.1%
τ_3[ns]	0.5216	±0.0058	1.1%
I_3[kCnts]	73.8	±1.1	1.4%
$Bkgr_{Dec}$[kCnts]	0.0248	±0.0002	0.5%
$Bkgr_{IRF}$[Cnts/Chnl]	3.26	±0.12	3.5%
$Shift_{IRF}$[ps]	19.70	±0.29	1.5%
A_{Scatt}[kCnts]	43.31	±0.52	1.2%
τ_{AvInt}[ns]	5.6543	±0.0095	0.2%
τ_{AvAmp}[ns]	3.069	±0.019	0.6%
λ_{Det}[nm]	660.0000	---	---

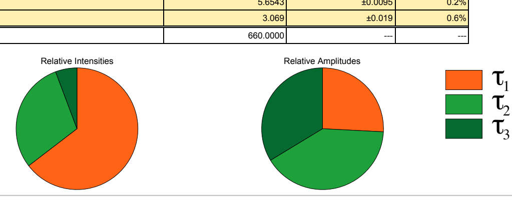

Data Set: 28 / 31

Decay: crv[28]; IRF: crv[0]

Fit

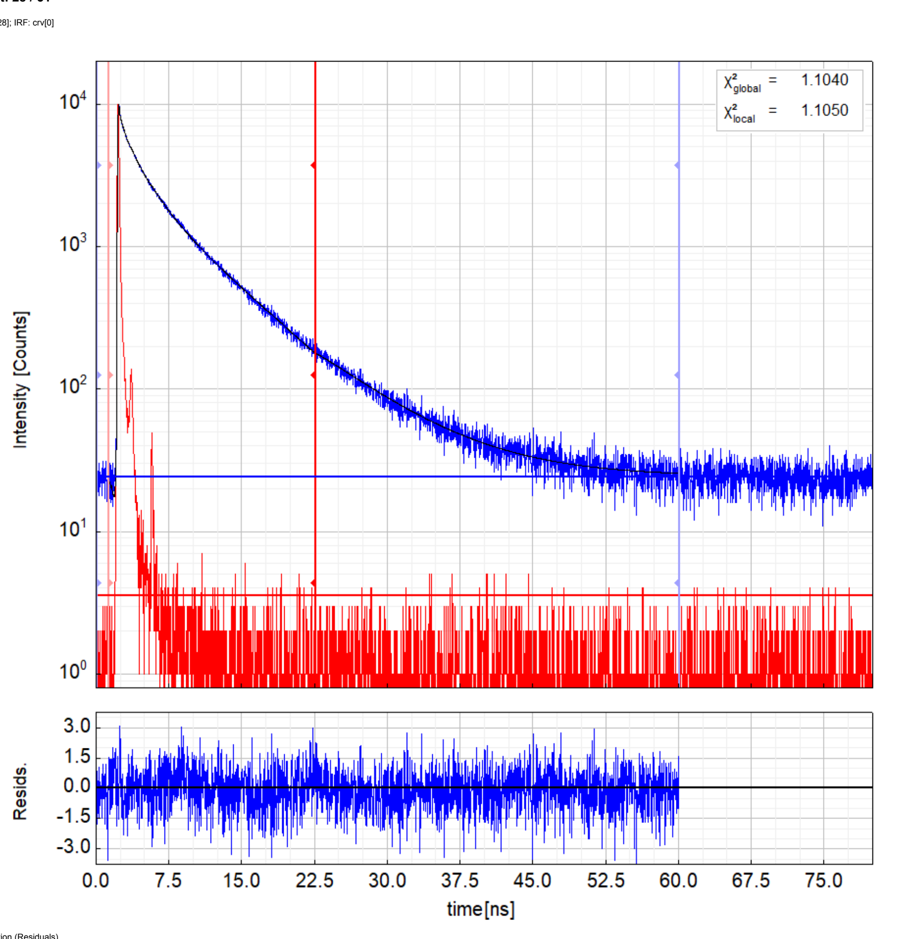

Autocorrelation (Residuals)

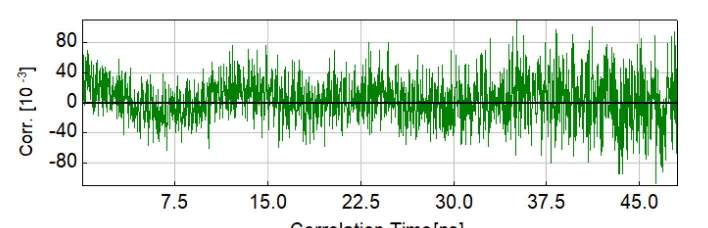

Fitted Parameters

Parameter	Value	Δ	δ
A_1[kCnts/Chnl]	2.642	±0.014	0.5%
τ_1[ns]	7.675	±0.012	0.2%
I_1[kCnts]	811.0	±2.3	0.3%
A_2[kCnts/Chnl]	4.295	±0.031	0.7%
τ_2[ns]	2.250	±0.013	0.6%
I_2[kCnts]	386.4	±3.2	0.8%
A_3[kCnts/Chnl]	3.481	±0.051	1.5%
τ_3[ns]	0.5216	±0.0058	1.1%
I_3[kCnts]	72.7	±1.6	2.1%
$Bkgr_{Dec}$[kCnts]	0.0244	±0.0002	0.7%
$Bkgr_{IRF}$[Cnts/Chnl]	3.529	±0.091	2.6%
$Shift_{IRF}$[ps]	20.57	±0.47	2.3%
A_{Scatt}[kCnts]	44.31	±0.40	0.9%
τ_{AvInt}[ns]	5.6152	±0.0041	0.1%
τ_{AvAmp}[ns]	3.048	±0.014	0.5%
λ_{Det}[nm]	670.0000	---	---

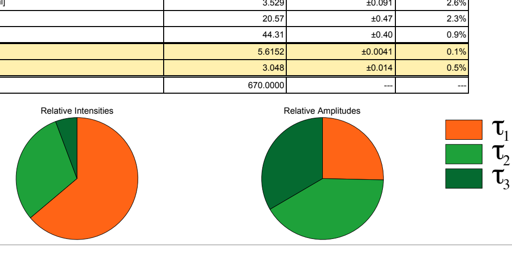

Data Set: 29 / 31

Decay: crv[29]; IRF: crv[0]

Fit

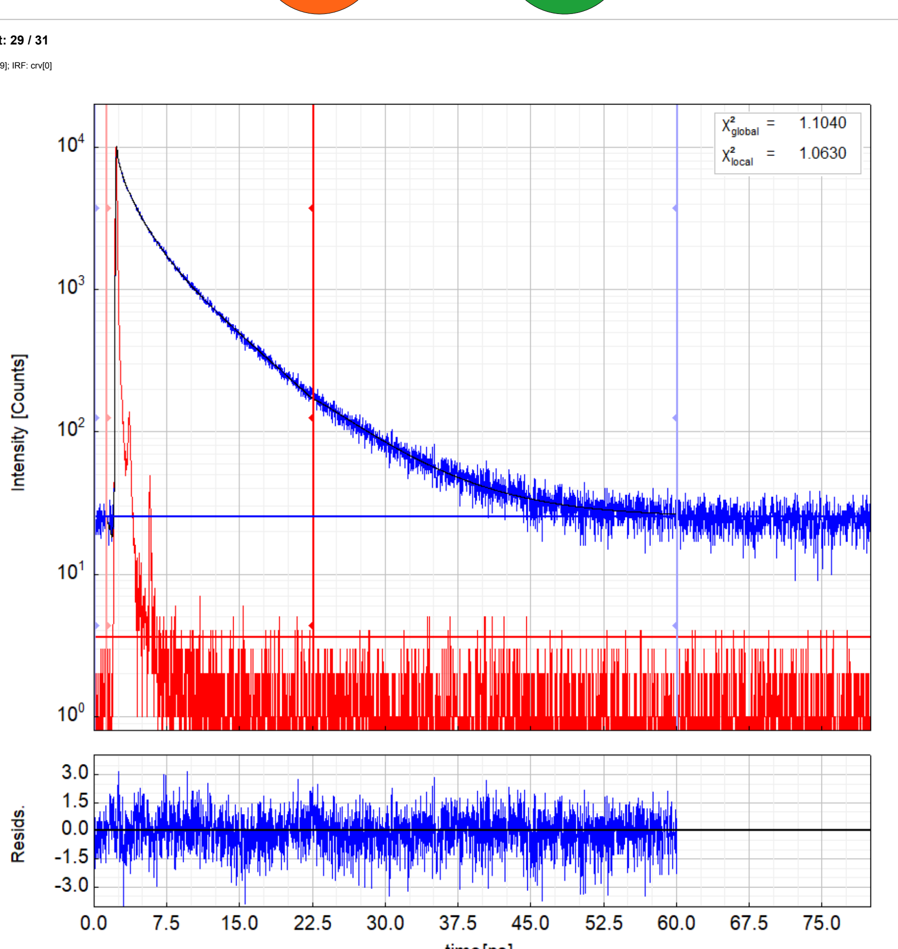

Autocorrelation (Residuals)

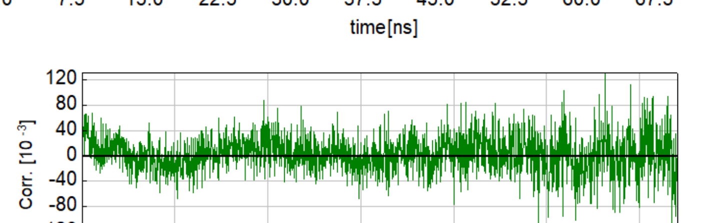

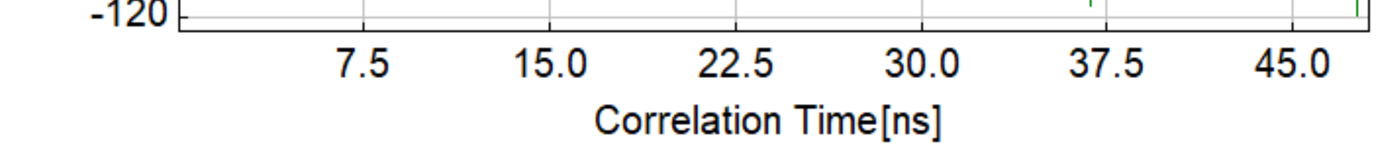

Fitted Parameters

Parameter	Value	Δ	δ
A_1[kCnts/Chnl]	2.509	±0.019	0.7%
τ_1[ns]	7.675	±0.012	0.2%
I_1[kCnts]	770.3	±5.0	0.6%
A_2[kCnts/Chnl]	4.315	±0.038	0.9%
τ_2[ns]	2.250	±0.013	0.6%
I_2[kCnts]	388.2	±3.9	1.0%
A_3[kCnts/Chnl]	3.592	±0.070	1.9%
τ_3[ns]	0.5216	±0.0058	1.1%
I_3[kCnts]	75.0	±1.2	1.5%
$Bkgr_{Dec}$[kCnts]	0.0253	±0.0002	0.5%
$Bkgr_{IRF}$[Cnts/Chnl]	3.60	±0.12	3.2%
$Shift_{IRF}$[ps]	20.68	±0.46	2.2%
A_{Scatt}[kCnts]	44.66	±0.52	1.2%
τ_{AvInt}[ns]	5.533	±0.011	0.2%
τ_{AvAmp}[ns]	2.961	±0.017	0.5%
λ_{Det}[nm]	680.0000	---	---

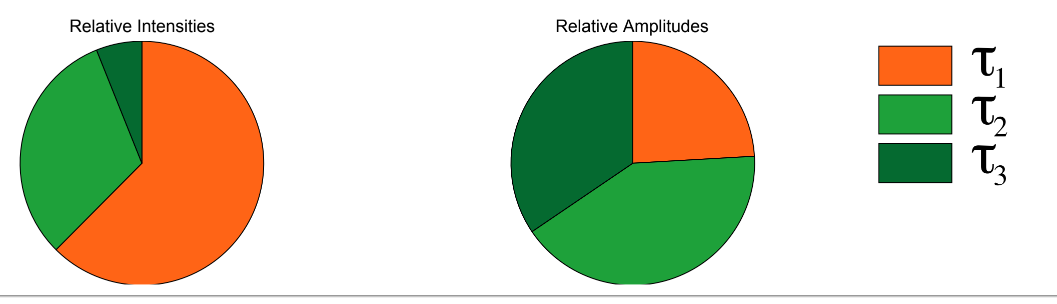

Data Set: 30 / 31

Decay: crv[30]; IRF: crv[0]

Fit

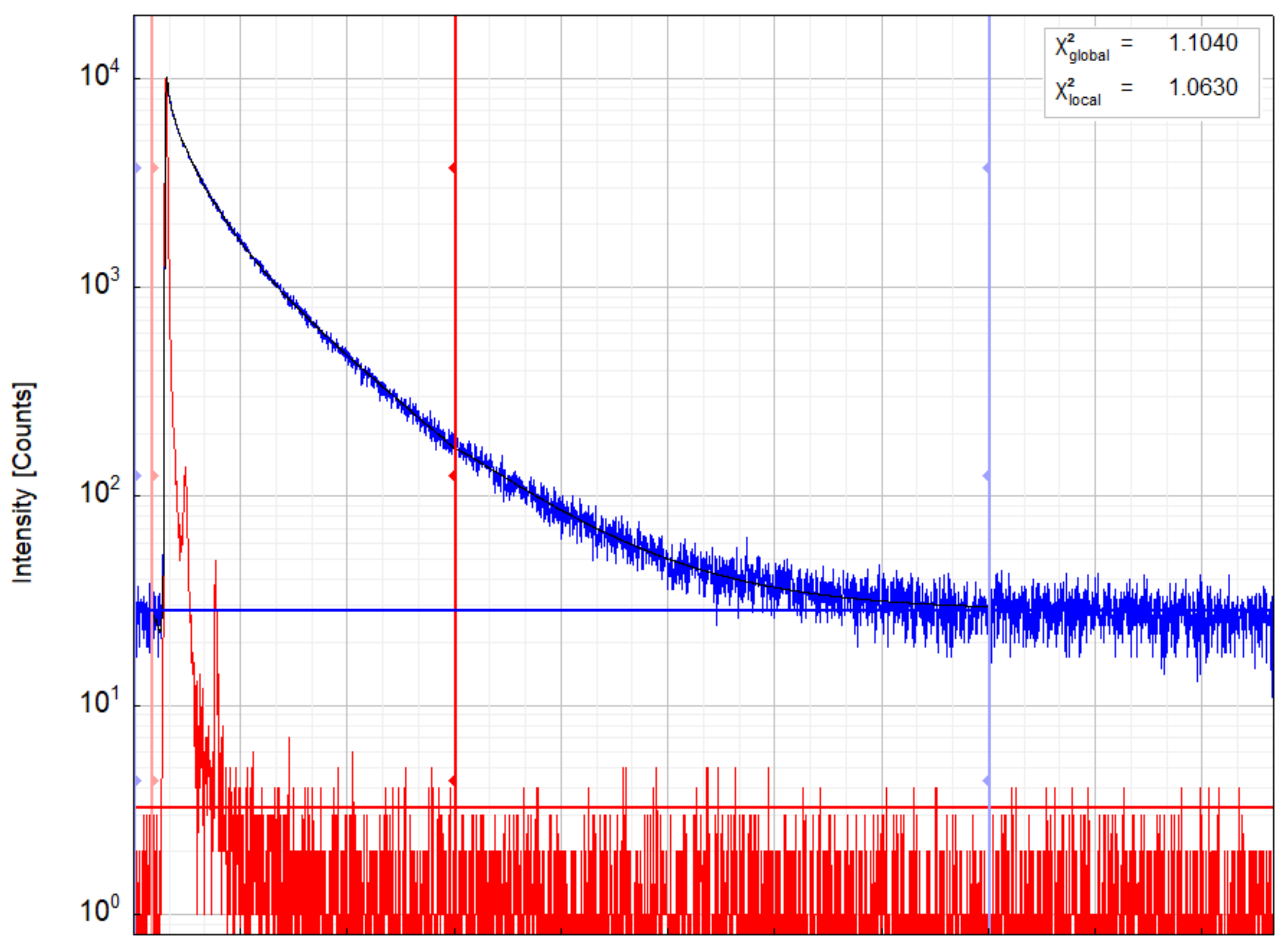

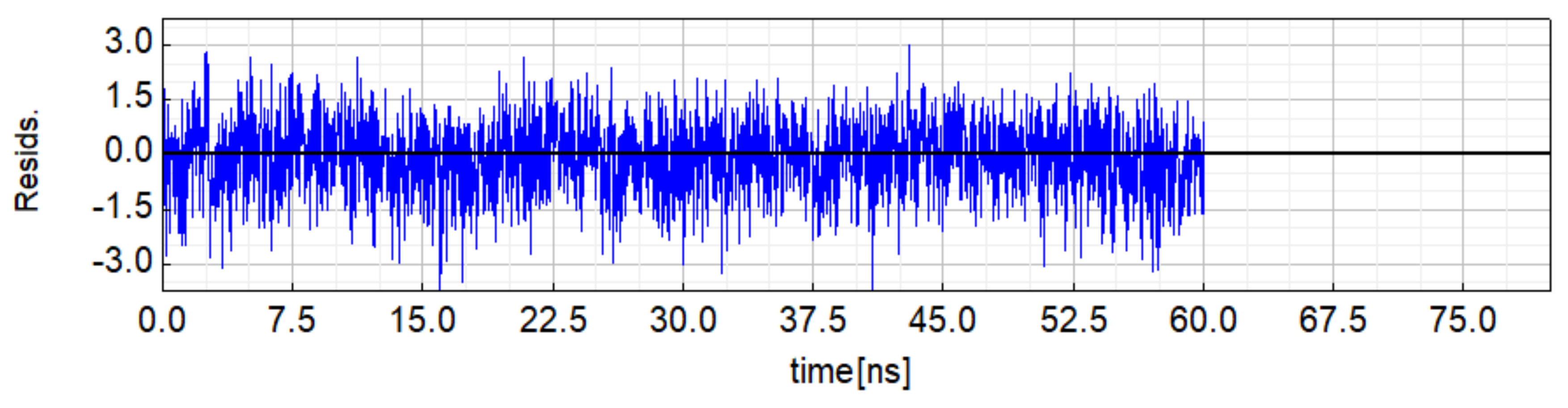

Autocorrelation (Residuals)

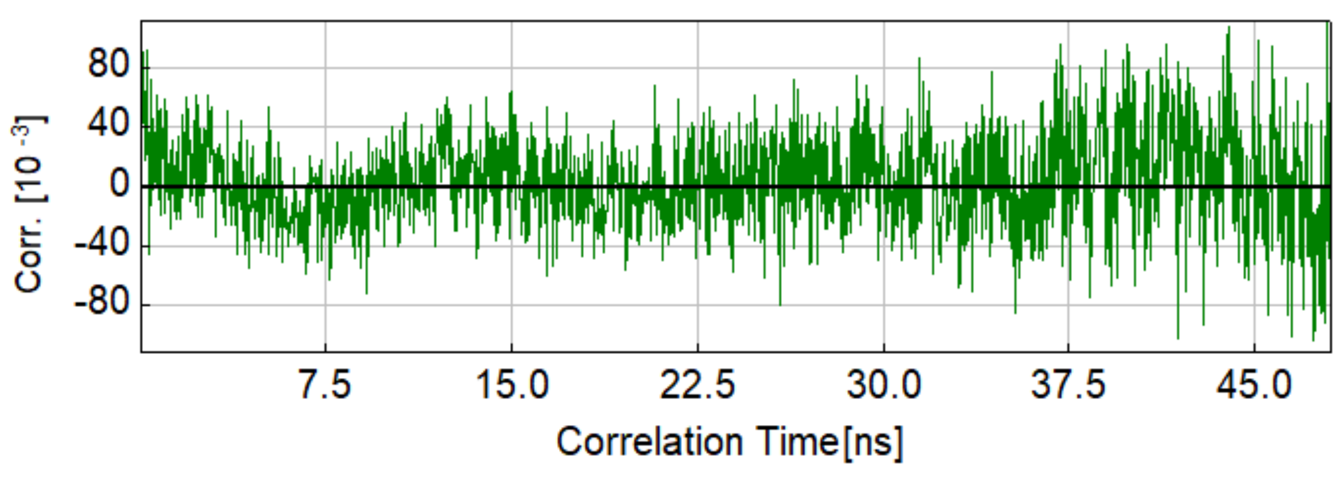

Fitted Parameters

Parameter	Value	Δ	δ
A_1[kCnts/Chnl]	2.370	±0.017	0.7%
τ_1[ns]	7.675	±0.012	0.2%
I_1[kCnts]	727.6	±4.1	0.6%
A_2[kCnts/Chnl]	4.305	±0.032	0.7%
τ_2[ns]	2.250	±0.013	0.6%
I_2[kCnts]	387.4	±2.3	0.6%
A_3[kCnts/Chnl]	3.56	±0.15	4.1%
τ_3[ns]	0.5216	±0.0058	1.1%
I_3[kCnts]	74.1	±3.8	5.1%
$Bkgr_{Dec}$[kCnts]	0.0285	±0.0002	0.6%
$Bkgr_{IRF}$[Cnts/Chnl]	3.248	±0.097	3.0%
$Shift_{IRF}$[ps]	21.51	±0.35	1.6%
A_{Scatt}[kCnts]	45.55	±0.51	1.1%
τ_{AvInt}[ns]	5.4618	±0.0084	0.2%
τ_{AvAmp}[ns]	2.907	±0.025	0.9%
λ_{Det}[nm]	690.0000	---	---

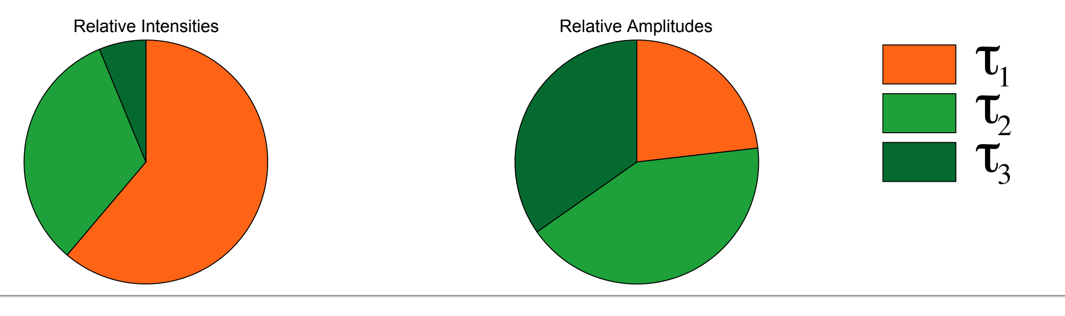

Data Set: 31 / 31

Decay: crv[31]; IRF: crv[0]

Fit

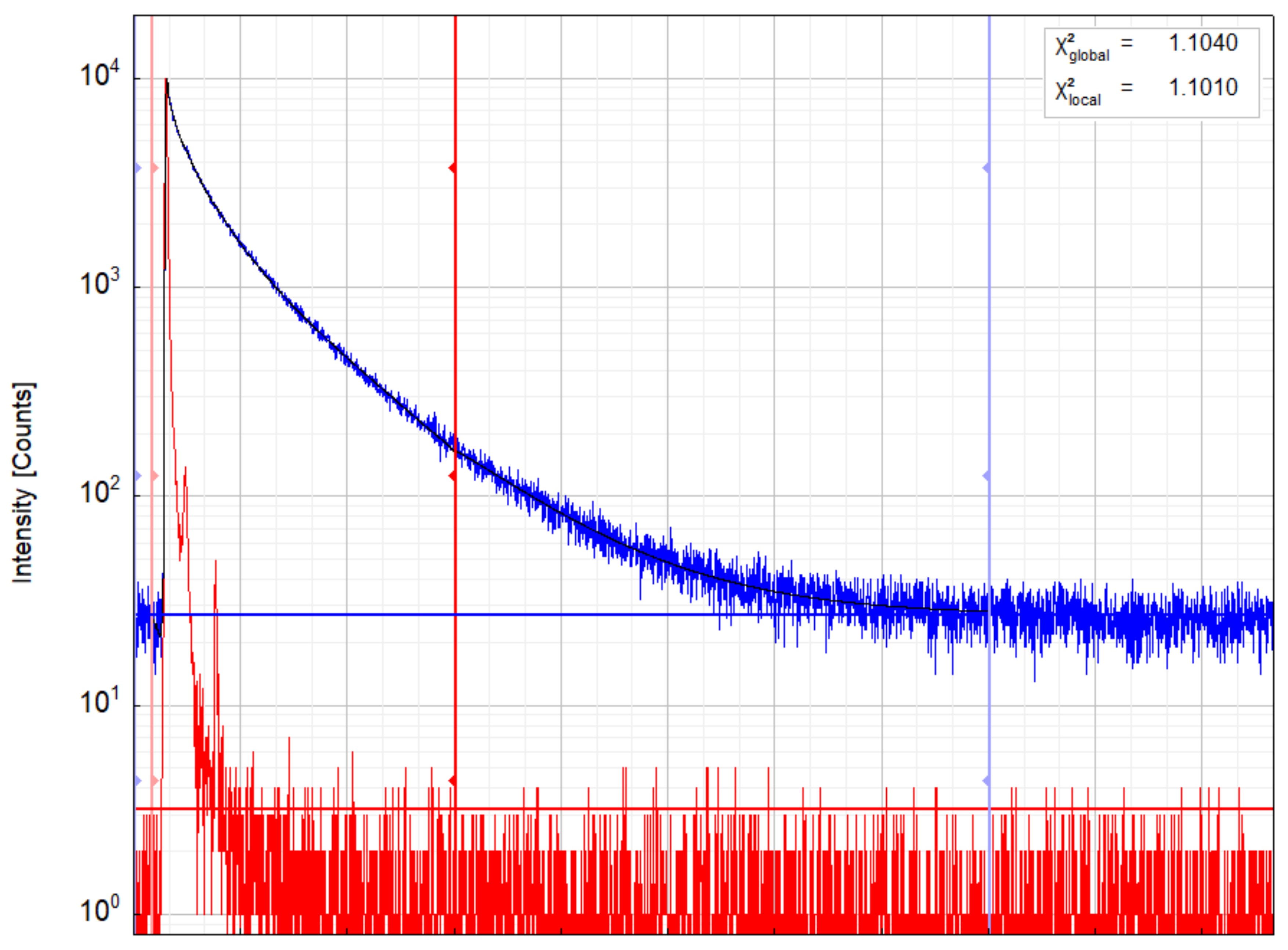

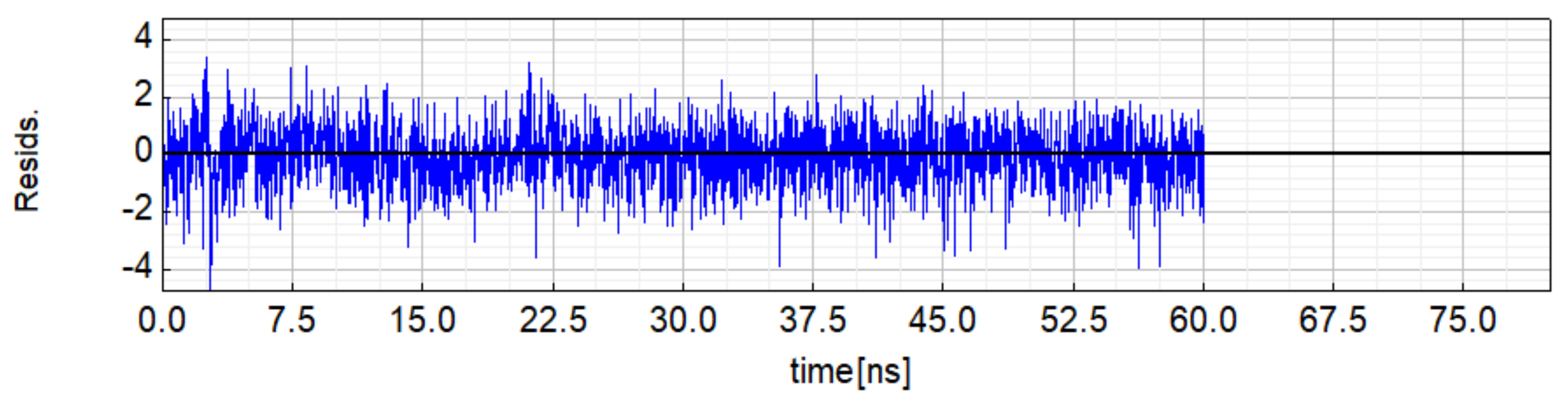

Autocorrelation (Residuals)

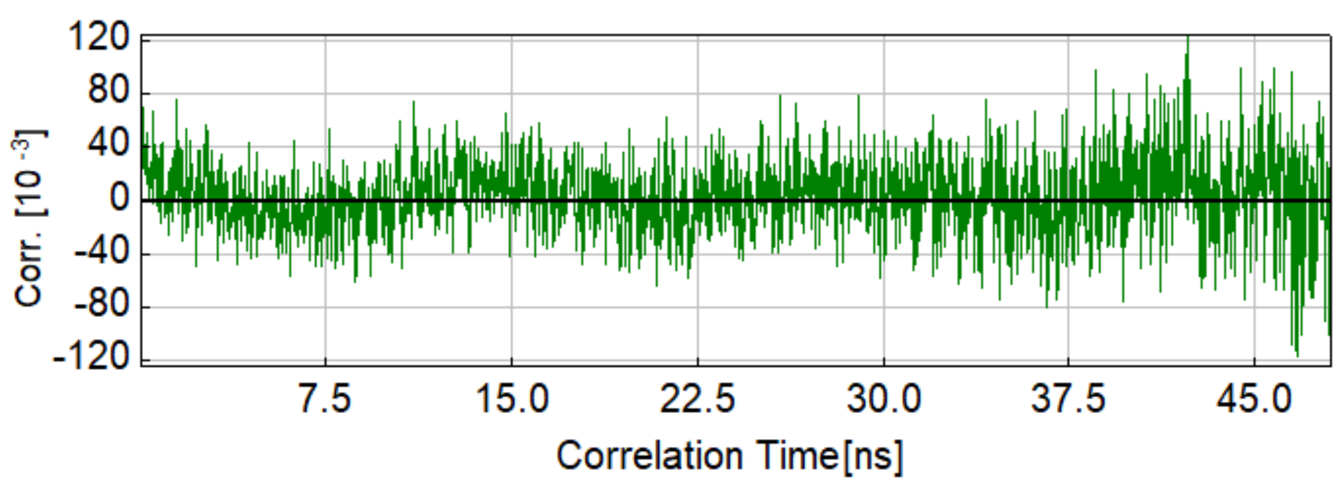

Fitted Parameters

Parameter	Value	Δ	δ
A_1[kCnts/Chnl]	2.300	±0.017	0.7%
τ_1[ns]	7.675	±0.012	0.2%
I_1[kCnts]	706.0	±3.5	0.5%
A_2[kCnts/Chnl]	4.263	±0.038	0.9%
τ_2[ns]	2.250	±0.013	0.6%
I_2[kCnts]	383.5	±3.6	0.9%
A_3[kCnts/Chnl]	3.555	±0.083	2.3%
τ_3[ns]	0.5216	±0.0058	1.1%
I_3[kCnts]	74.2	±2.6	3.5%
$Bkgr_{Dec}$[kCnts]	0.0271	±0.0002	0.7%
$Bkgr_{IRF}$[Cnts/Chnl]	3.16	±0.17	5.2%
$Shift_{IRF}$[ps]	21.83	±0.64	2.9%
A_{Scatt}[kCnts]	45.69	±0.76	1.7%
τ_{AvInt}[ns]	5.431	±0.017	0.3%
τ_{AvAmp}[ns]	2.876	±0.018	0.6%
λ_{Det}[nm]	700.0000	---	---

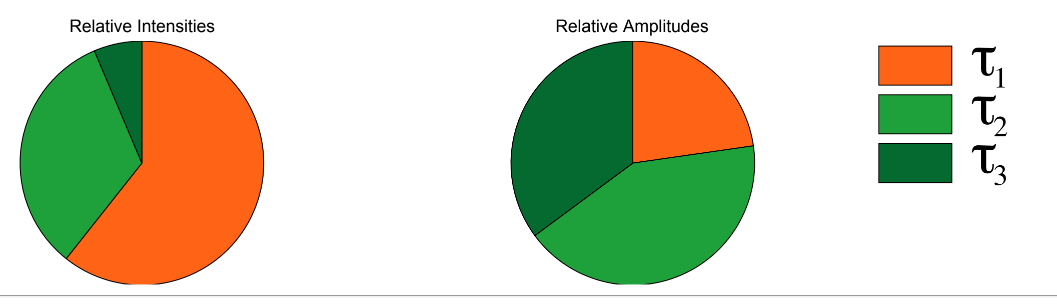

Parameter Plot

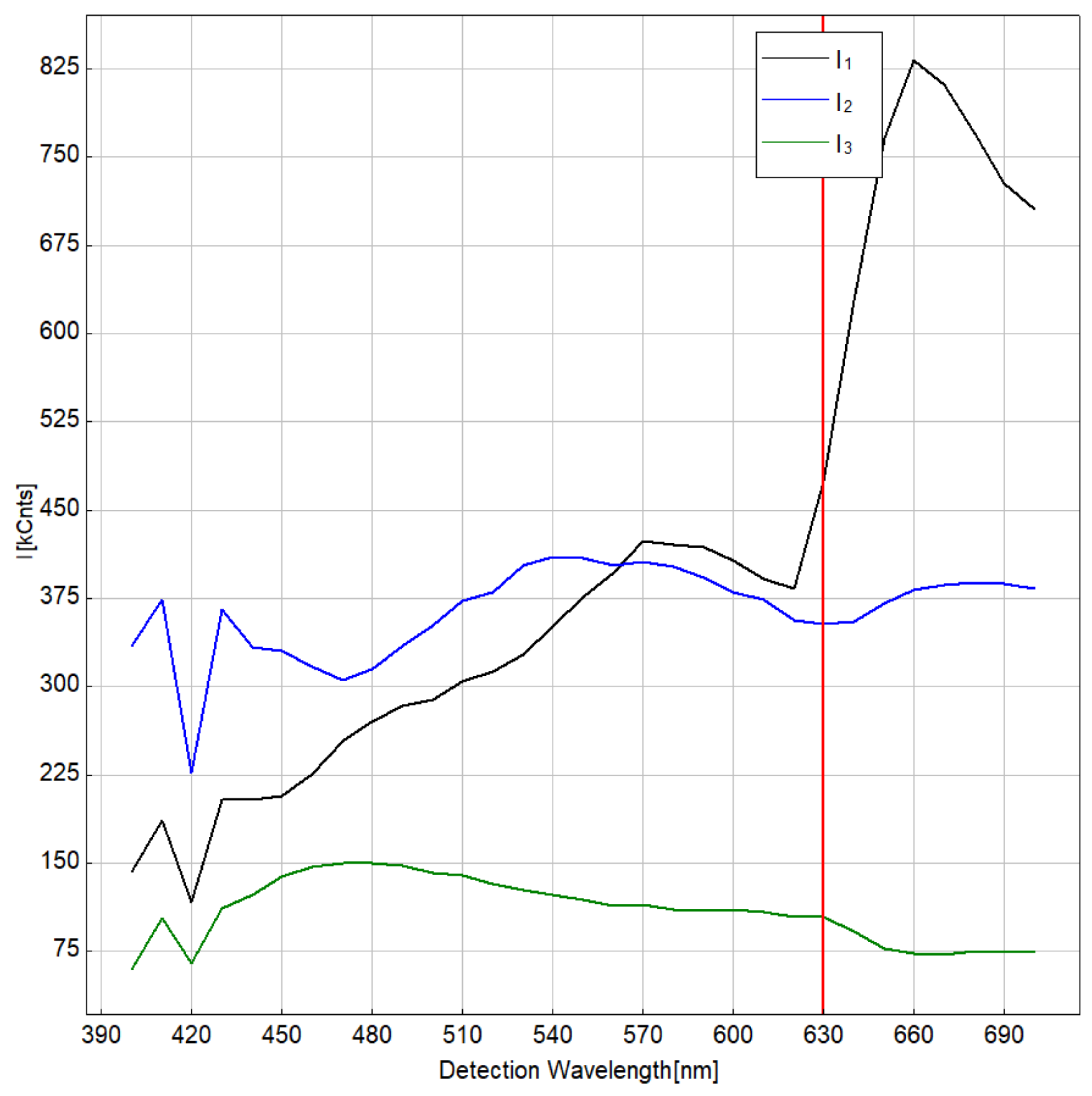

Global Parameters

Parameter	Value	Δ	δ
τ_1[ns]	7.675	±0.012	0.2%
τ_2[ns]	2.250	±0.013	0.6%
τ_3[ns]	0.5216	±0.0058	1.1%
