# Supplementary material for: A Porphyrin‐Based Organic Network Comprising Sustainable Carbon Dots for Photopolymerization
Source: Angew Chem Int Ed Engl. 2022 Sep 1;61(40):e202208180. doi: 10.1002/anie.202208180 (PMC9826160; doi:10.1002/anie.202208180)
Supplement: Supplementary file 3 — Supporting Information [file ANIE-61-0-s003.pdf]

EasyTau Report

3/11/2022 7:22:17 PM
F:\Paper Xiongfei\CD-1 DMSO-1 tau global.html

Container Curves

TRES+IRF_20201204_1943.etc: crv[0 - 29]

Container Properties

Measurement Context: Time Resolved Emission Scan
Detection Wavelength: 440.0 - 720.0 (Step: 10.0) nm
Summary:

Sample:	CD_X_1
Solvent:	Dimethyl sulfoxide
Excitation:	V pol 376±1nm with LDH-P-C-375
Detection:	M pol 440...720nm 10nm steps
	10000 peak counts
	grating 1200/500+
	detector UV-red [PMT]

Comment

Model: Exponential [Reconv.]

$$\mathrm{Dec}(t) = \left[\int_{-\infty}^{t} dt' \left[\mathrm{IRF}(t - Shift_{IRF}) - Bkgr_{IRF}\right]\left[\sum_{i=1}^{n_{Exp}} A_i \, e^{-\frac{t-t'}{\tau_i}} + A_{Scatt}\, \delta(t - t')\right]\right] + Bkgr_{Dec}$$

$$I_m = A_m \tau_m$$

$$A_{Rel\,m} = A_m \Big/ \sum_{\substack{i=1 \\ A_i > 0}}^{n_{Exp}} A_i \qquad I_{Rel\,m} = I_m \Big/ \sum_{\substack{i=1 \\ I_i > 0}}^{n_{Exp}} I_i$$

$$\tau_{Av\,Amp} = \sum_{\substack{i=1 \\ A_i > 0}}^{n_{Exp}} A_i \tau_i \Big/ \sum_{\substack{i=1 \\ A_i > 0}}^{n_{Exp}} A_i \qquad \tau_{Av\,Int} = \sum_{\substack{i=1 \\ I_i > 0}}^{n_{Exp}} I_i \tau_i \Big/ \sum_{\substack{i=1 \\ I_i > 0}}^{n_{Exp}} I_i$$

Data Set: 1 / 29

Decay: crv[1]; IRF: crv[0]

Fit

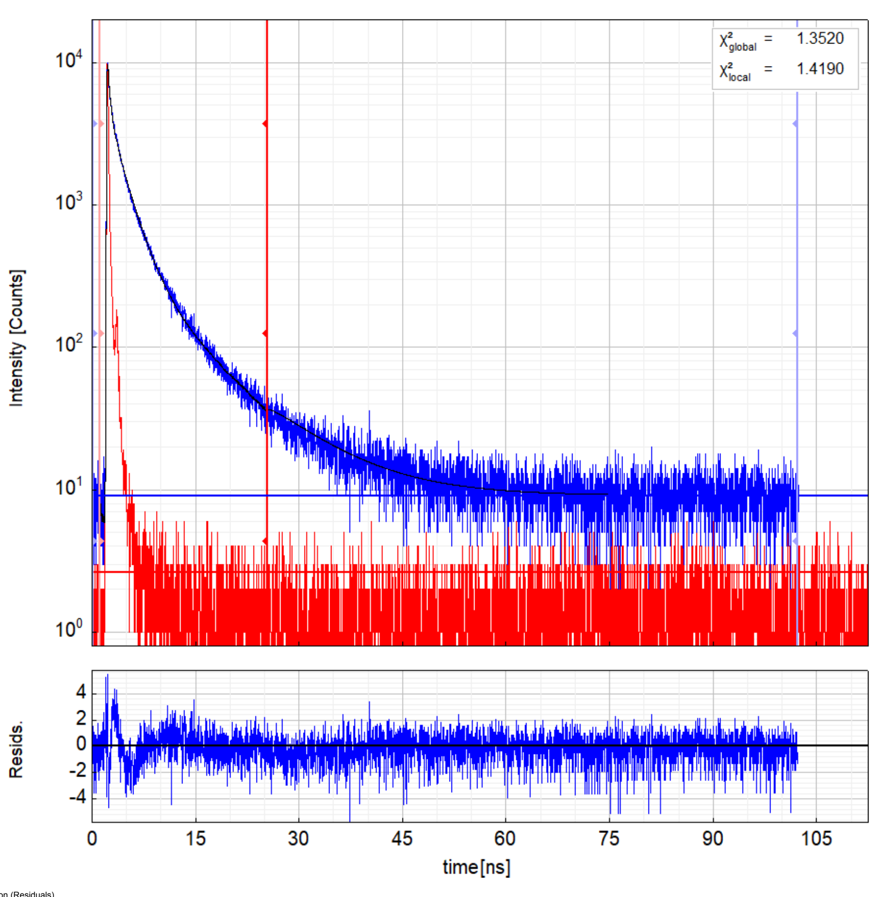

Autocorrelation (Residuals)

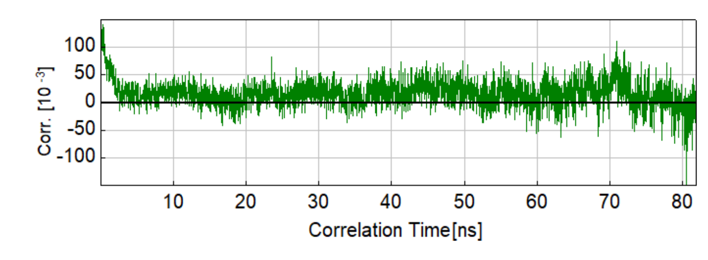

Fitted Parameters

Parameter	Value	Δ	δ
A_1[kCnts/Chnl]	3.95	±0.27	6.7%
τ_1[ns]	2.23	±0.14	5.9%
I_1[kCnts]	350.5	±4.8	1.3%
A_2[kCnts/Chnl]	0.415	±0.034	8.0%
τ_2[ns]	9.233	±0.054	0.6%
I_2[kCnts]	153	±12	7.5%
A_3[kCnts/Chnl]	6.68	±0.33	4.8%
τ_3[ns]	0.339	±0.056	16%
I_3[kCnts]	91	±13	13%
$Bkgr_{Dec}$[kCnts]	0.0092	±0.0001	1.0%
$Bkgr_{IRF}$[Cnts/Chnl]	2.62	±0.57	22%
$Shift_{IRF}$[ps]	-9.8	±1.8	18%
A_{Scatt}[kCnts]	60.5	±1.8	3.0%
τ_{AvInt}[ns]	3.741	±0.056	1.5%
τ_{AvAmp}[ns]	1.346	±0.061	4.5%
λ_{Det}[nm]	440.0000	---	---

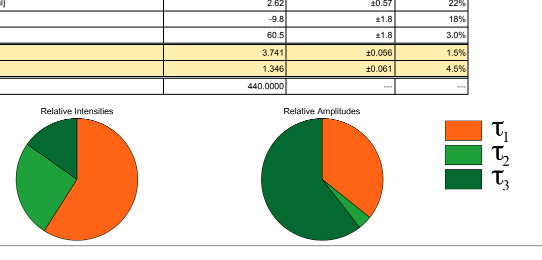

Data Set: 2 / 29

Decay: crv[2]; IRF: crv[0]

Fit

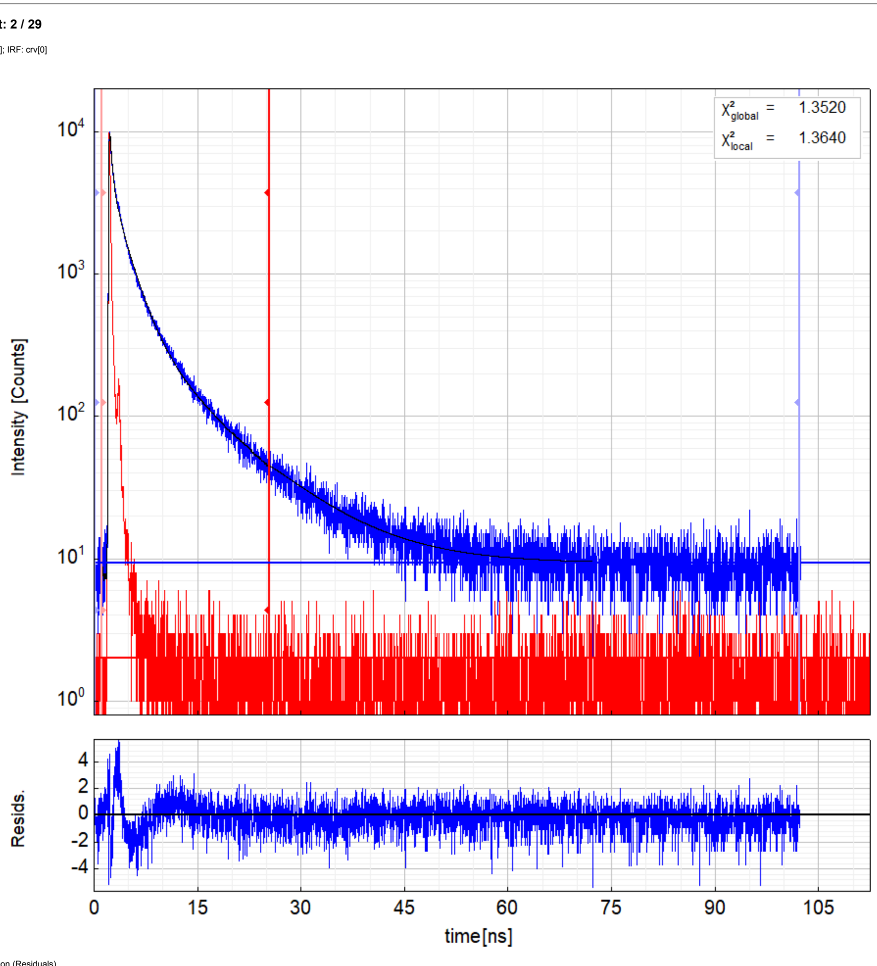

Autocorrelation (Residuals)

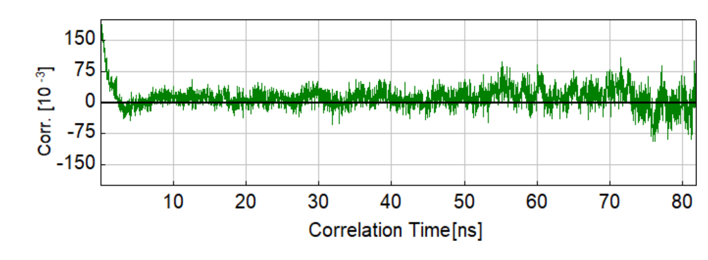

Fitted Parameters

Parameter	Value	Δ	δ
A_1[kCnts/Chnl]	3.69	±0.28	7.5%
τ_1[ns]	2.23	±0.14	5.9%
I_1[kCnts]	328.1	±8.9	2.7%
A_2[kCnts/Chnl]	0.477	±0.025	5.0%
τ_2[ns]	9.233	±0.054	0.6%
I_2[kCnts]	176.0	±8.5	4.8%
A_3[kCnts/Chnl]	8.15	±0.52	6.4%
τ_3[ns]	0.339	±0.056	16%
I_3[kCnts]	111	±13	11%
$Bkgr_{Dec}$[kCnts]	0.0094	±0.0001	0.7%
$Bkgr_{IRF}$[Cnts/Chnl]	2.02	±0.45	22%
$Shift_{IRF}$[ps]	-10.0	±2.0	20%
A_{Scatt}[kCnts]	54.5	±2.5	4.5%
τ_{AvInt}[ns]	3.892	±0.040	1.0%
τ_{AvAmp}[ns]	1.248	±0.065	5.1%
λ_{Det}[nm]	450.0000	---	---

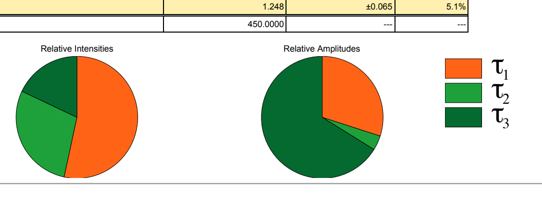

Data Set: 3 / 29

Decay: crv[3]; IRF: crv[0]

Fit

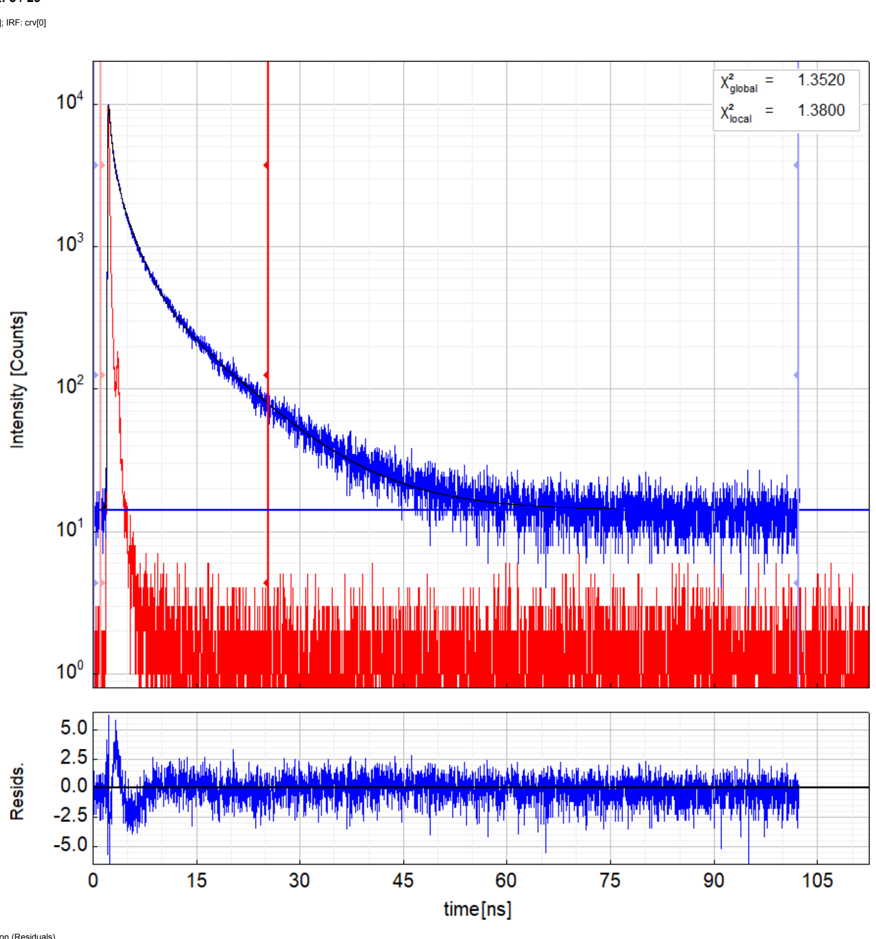

Autocorrelation (Residuals)

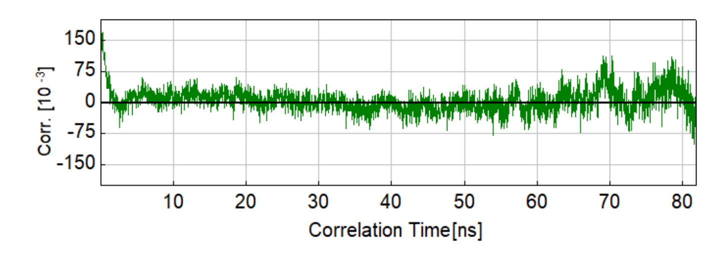

Fitted Parameters

Parameter	Value	Δ	δ
A_1[kCnts/Chnl]	3.43	±0.27	7.7%
τ_1[ns]	2.23	±0.14	5.9%
I_1[kCnts]	305.0	±8.1	2.6%
A_2[kCnts/Chnl]	0.759	±0.022	2.8%
τ_2[ns]	9.233	±0.054	0.6%
I_2[kCnts]	280.0	±6.9	2.5%
A_3[kCnts/Chnl]	8.72	±0.66	7.5%
τ_3[ns]	0.339	±0.056	16%
I_3[kCnts]	118.2	±9.9	8.3%
$Bkgr_{Dec}$[kCnts]	0.0142	±0.0001	0.5%
$Bkgr_{IRF}$[Cnts/Chnl]	0.75	±0.34	46%
$Shift_{IRF}$[ps]	-8.99	±0.94	10%
A_{Scatt}[kCnts]	49.2	±2.4	4.8%
τ_{AvInt}[ns]	4.698	±0.020	0.4%
τ_{AvAmp}[ns]	1.37	±0.11	7.5%
λ_{Det}[nm]	460.0000	---	---

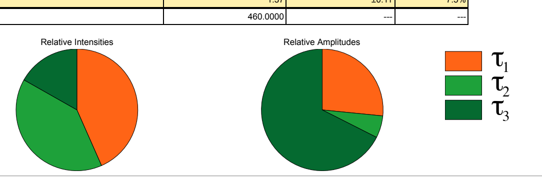

Data Set: 4 / 29

Decay: crv[4]; IRF: crv[0]

Fit

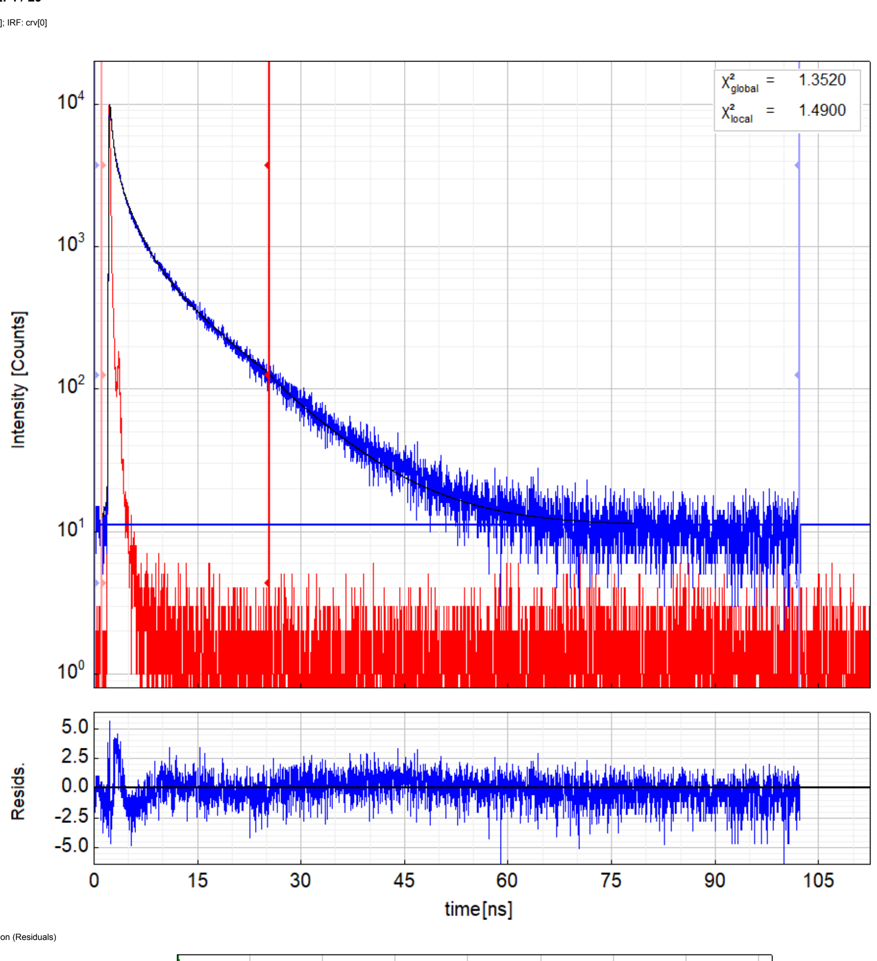

Autocorrelation (Residuals)

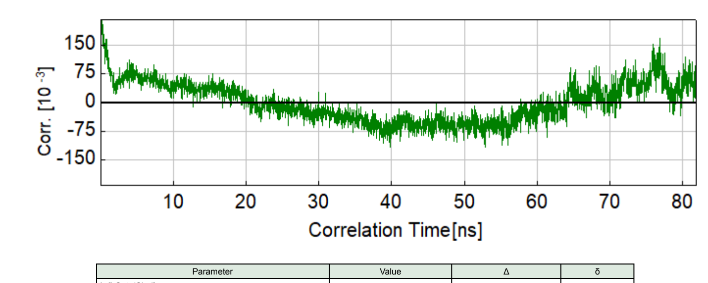

Fitted Parameters

Parameter	Value	Δ	δ
A_1[kCnts/Chnl]	3.35	±0.28	8.2%
τ_1[ns]	2.23	±0.14	5.9%
I_1[kCnts]	297.2	±8.8	2.9%
A_2[kCnts/Chnl]	1.241	±0.028	2.2%
τ_2[ns]	9.233	±0.054	0.6%
I_2[kCnts]	458.0	±5.9	1.3%
A_3[kCnts/Chnl]	9.07	±0.77	8.4%
τ_3[ns]	0.339	±0.056	16%
I_3[kCnts]	122.9	±9.2	7.5%
$Bkgr_{Dec}$[kCnts]	0.0111	±0.0001	0.6%
$Bkgr_{IRF}$[Cnts/Chnl]	-0.83	±0.17	21%
$Shift_{IRF}$[ps]	-8.8	±2.8	31%
A_{Scatt}[kCnts]	45.8	±4.2	9.0%
τ_{AvInt}[ns]	5.616	±0.047	0.8%
τ_{AvAmp}[ns]	1.61	±0.12	7.2%
λ_{Det}[nm]	470.0000	---	---

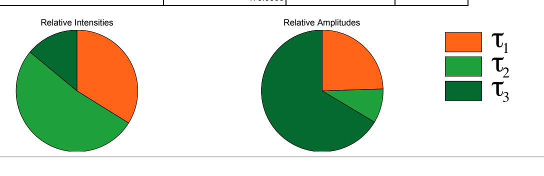

Data Set: 5 / 29

Decay: crv[5]; IRF: crv[0]

Fit

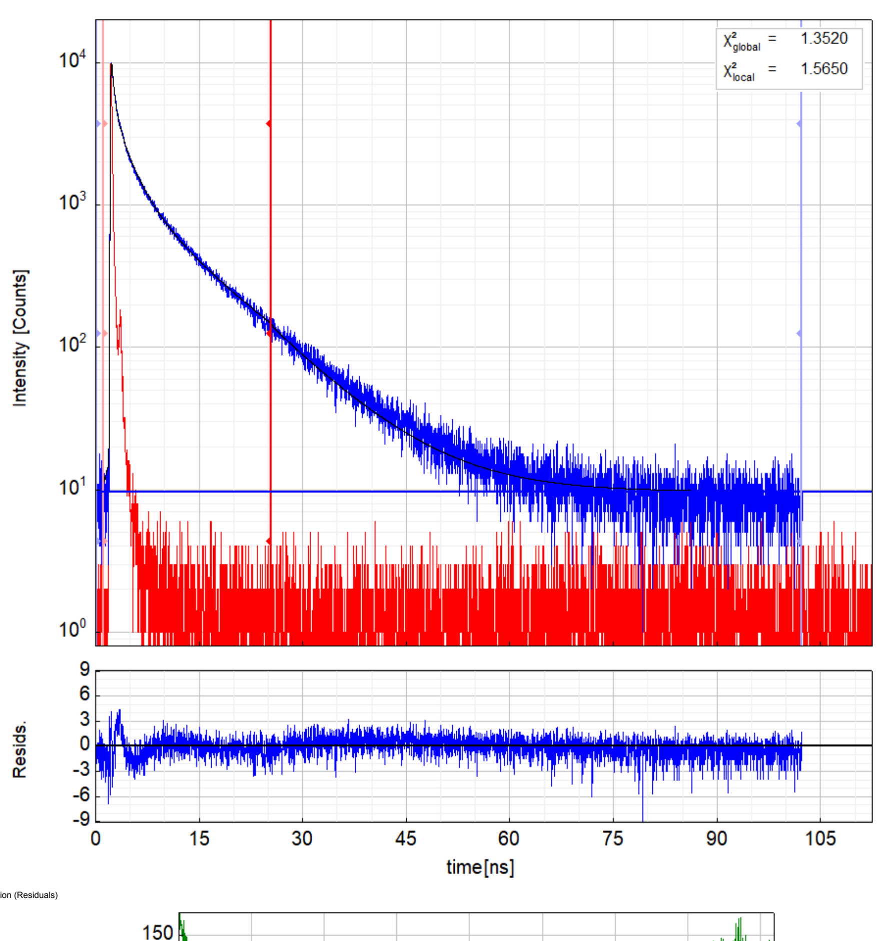

Autocorrelation (Residuals)

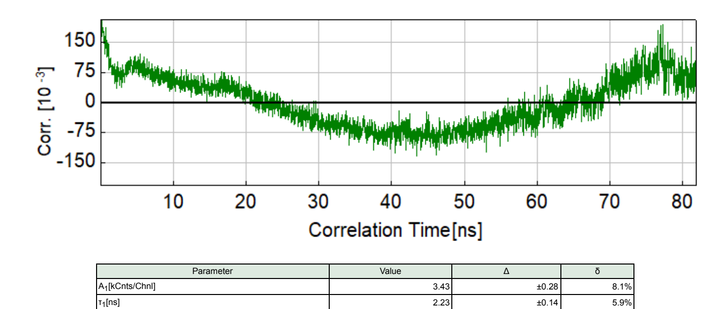

Fitted Parameters

Parameter	Value	Δ	δ
A_1[kCnts/Chnl]	3.43	±0.28	8.1%
τ_1[ns]	2.23	±0.14	5.9%
I_1[kCnts]	304.5	±8.4	2.7%
A_2[kCnts/Chnl]	1.462	±0.019	1.2%
τ_2[ns]	9.233	±0.054	0.6%
I_2[kCnts]	539.8	±4.8	0.9%
A_3[kCnts/Chnl]	9.03	±0.66	7.3%
τ_3[ns]	0.339	±0.056	16%
I_3[kCnts]	122.4	±9.3	7.6%
$Bkgr_{Dec}$[kCnts]	0.0097	±0.0001	0.6%
$Bkgr_{IRF}$[Cnts/Chnl]	-0.90	±0.17	18%
$Shift_{IRF}$[ps]	-9.9	±3.1	31%
A_{Scatt}[kCnts]	42.4	±4.6	11%
τ_{AvInt}[ns]	5.899	±0.036	0.6%
τ_{AvAmp}[ns]	1.74	±0.14	7.5%
λ_{Det}[nm]	480.0000	---	---

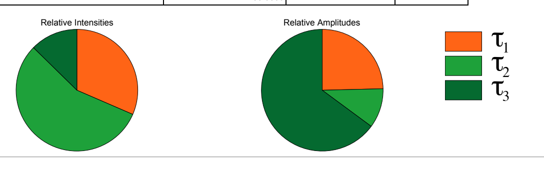

Data Set: 6 / 29

Decay: crv[6]; IRF: crv[0]

Fit

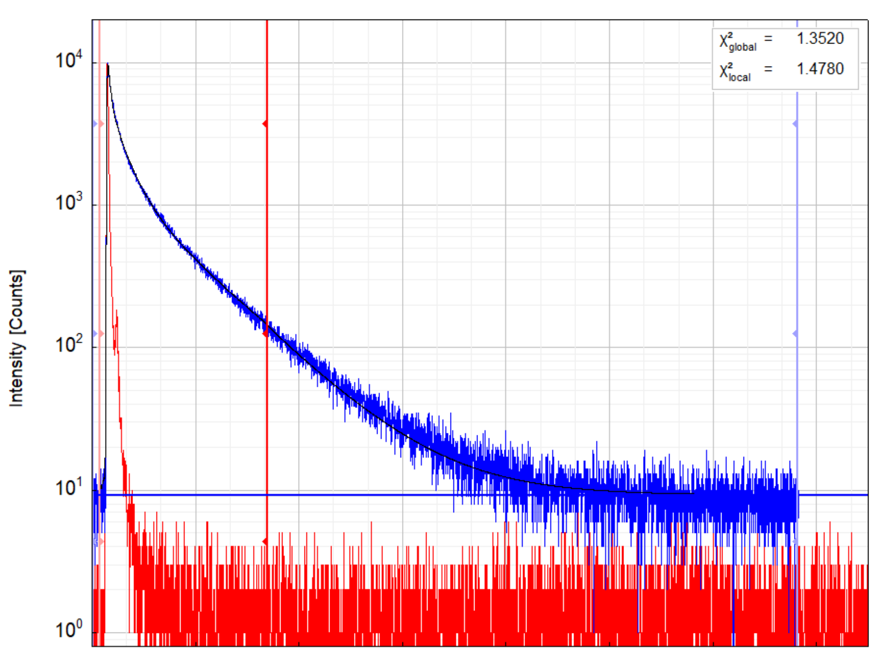

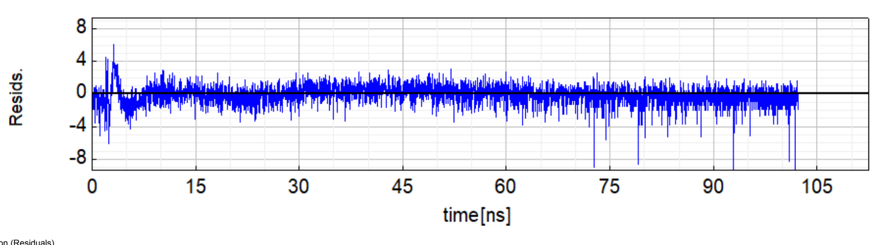

Autocorrelation (Residuals)

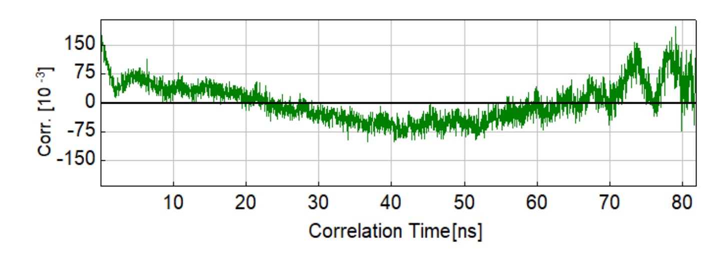

Fitted Parameters

Parameter	Value	Δ	δ
A_1[kCnts/Chnl]	3.79	±0.26	6.9%
$τ_1$[ns]	2.23	±0.14	5.9%
I_1[kCnts]	336.4	±8.6	2.6%
A_2[kCnts/Chnl]	1.519	±0.031	2.0%
$τ_2$[ns]	9.233	±0.054	0.6%
I_2[kCnts]	560.8	±7.8	1.4%
A_3[kCnts/Chnl]	8.42	±0.73	8.7%
$τ_3$[ns]	0.339	±0.056	16%
I_3[kCnts]	115	±11	8.9%
$Bkgr_{Dec}$[kCnts]	0.0093	±0.0001	0.9%
$Bkgr_{IRF}$[Cnts/Chnl]	-0.13	±0.13	101%
$Shift_{IRF}$[ps]	-9.2	±3.1	33%
A_{Scatt}[kCnts]	40.3	±4.8	12%
$τ_{AvInt}$[ns]	5.898	±0.038	0.6%
$τ_{AvAmp}$[ns]	1.85	±0.14	7.5%
$λ_{Det}$[nm]	490.0000	---	---

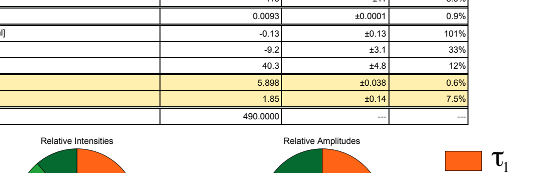

Data Set: 7 / 29

Decay: crv[7]; IRF: crv[0]

Fit

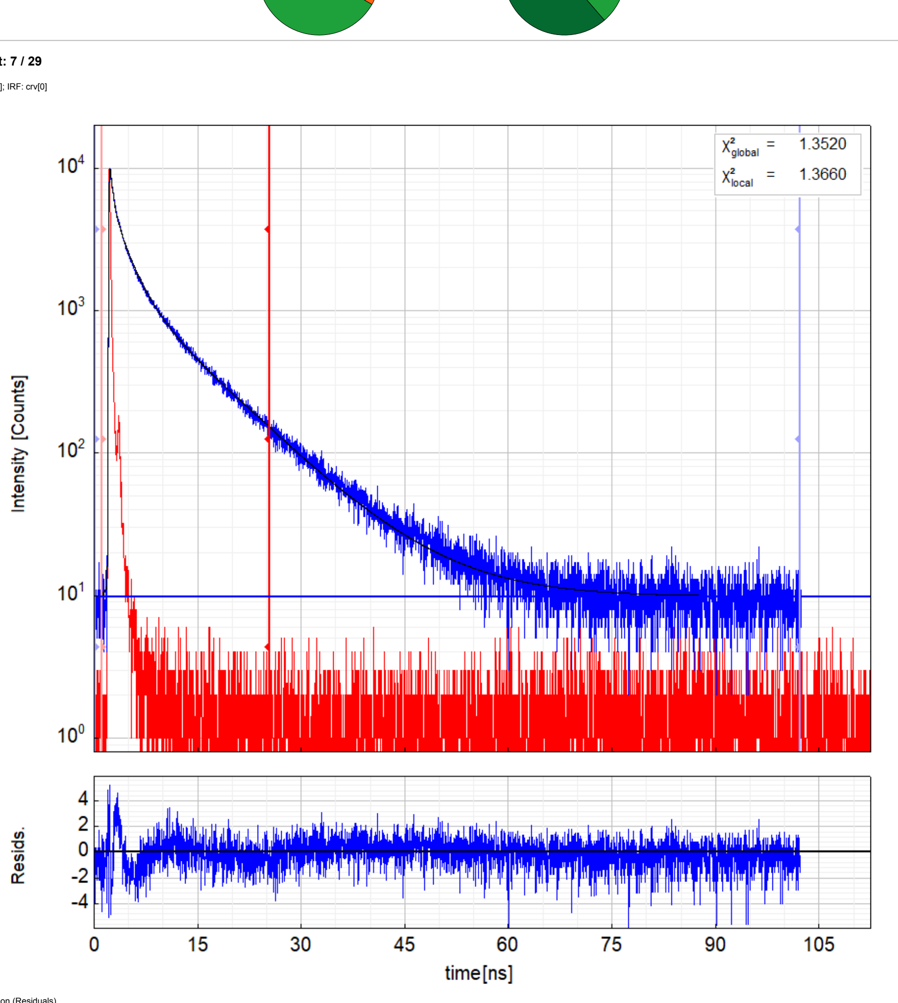

Autocorrelation (Residuals)

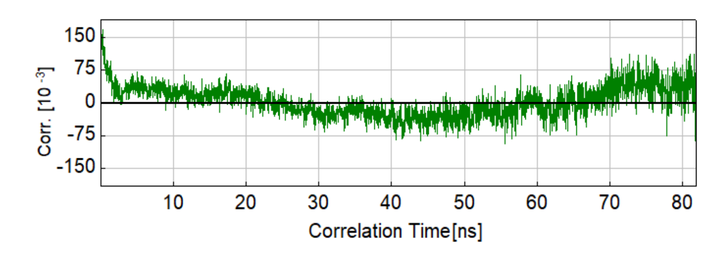

Fitted Parameters

Parameter	Value	Δ	δ
A_1[kCnts/Chnl]	4.27	±0.29	6.6%
$τ_1$[ns]	2.23	±0.14	5.9%
I_1[kCnts]	379.4	±5.3	1.4%
A_2[kCnts/Chnl]	1.674	±0.028	1.7%
$τ_2$[ns]	9.233	±0.054	0.6%
I_2[kCnts]	618.0	±8.8	1.4%
A_3[kCnts/Chnl]	8.55	±0.39	4.5%
$τ_3$[ns]	0.339	±0.056	16%
I_3[kCnts]	116	±13	11%
$Bkgr_{Dec}$[kCnts]	0.0099	±0.0001	0.5%
$Bkgr_{IRF}$[Cnts/Chnl]	0.53	±0.21	38%
$Shift_{IRF}$[ps]	-11.6	±2.2	18%
A_{Scatt}[kCnts]	36.2	±3.0	8.1%
$τ_{AvInt}$[ns]	5.9181	±0.0088	0.1%
$τ_{AvAmp}$[ns]	1.921	±0.092	4.8%
$λ_{Det}$[nm]	500.0000	---	---

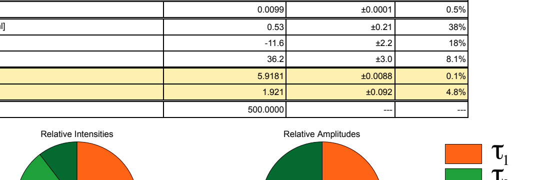

Data Set: 8 / 29

Decay: crv[8]; IRF: crv[0]

Fit

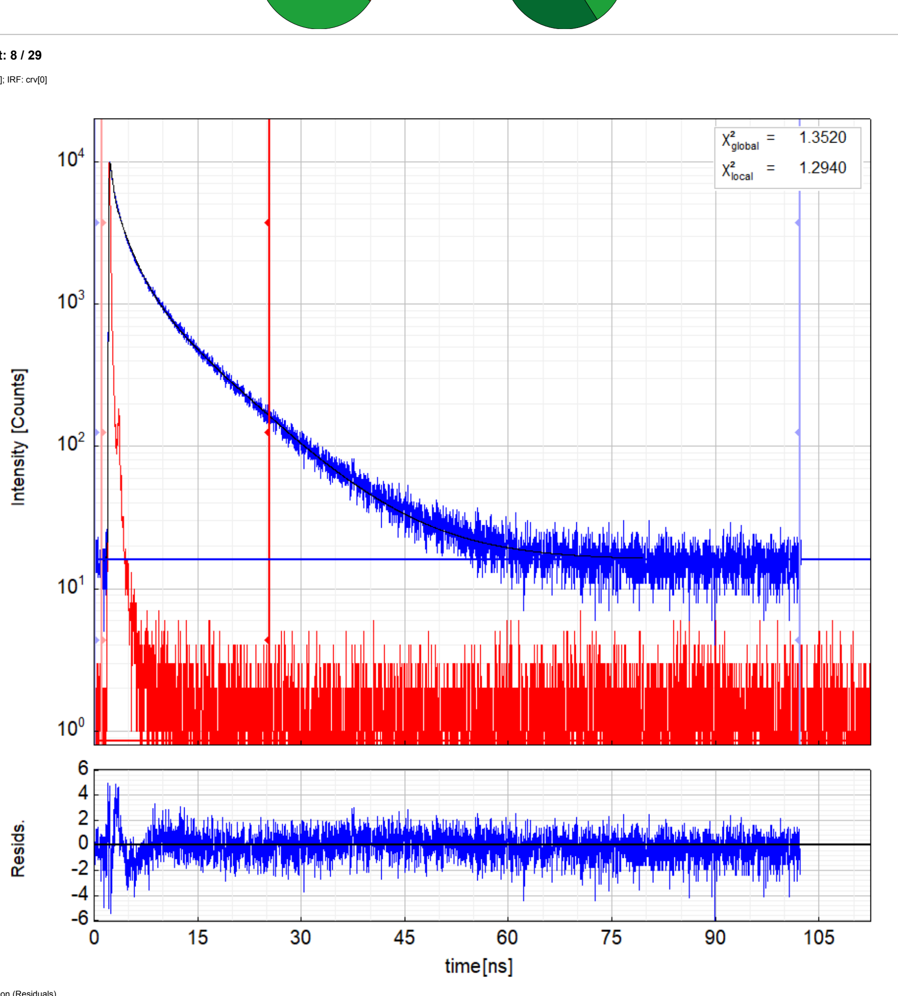

Autocorrelation (Residuals)

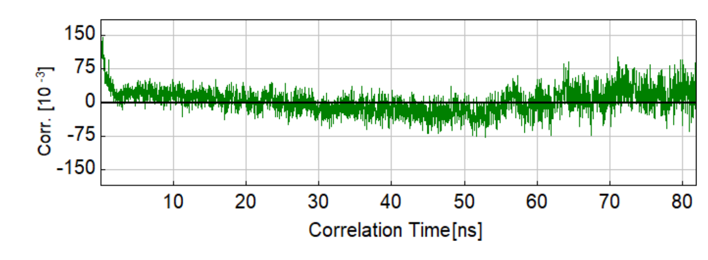

Fitted Parameters

Parameter	Value	Δ	δ
A_1[kCnts/Chnl]	4.60	±0.32	6.8%
$τ_1$[ns]	2.23	±0.14	5.9%
I_1[kCnts]	408.6	±6.7	1.6%
A_2[kCnts/Chnl]	1.761	±0.036	2.0%
$τ_2$[ns]	9.233	±0.054	0.6%
I_2[kCnts]	651	±11	1.6%
A_3[kCnts/Chnl]	8.23	±0.57	6.9%
$τ_3$[ns]	0.339	±0.056	16%
I_3[kCnts]	112	±11	9.4%
$Bkgr_{Dec}$[kCnts]	0.0160	±0.0002	0.7%
$Bkgr_{IRF}$[Cnts/Chnl]	0.85	±0.18	21%
$Shift_{IRF}$[ps]	-12.7	±3.1	24%
A_{Scatt}[kCnts]	31.9	±3.9	12%
$τ_{AvInt}$[ns]	5.938	±0.025	0.4%
$τ_{AvAmp}$[ns]	2.01	±0.11	5.4%
$λ_{Det}$[nm]	510.0000	---	---

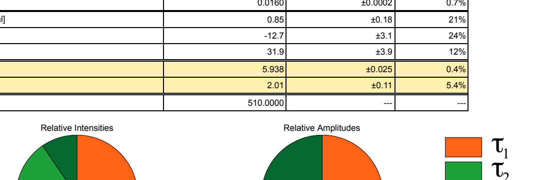

Data Set: 9 / 29

Decay: crv[9]; IRF: crv[0]

Fit

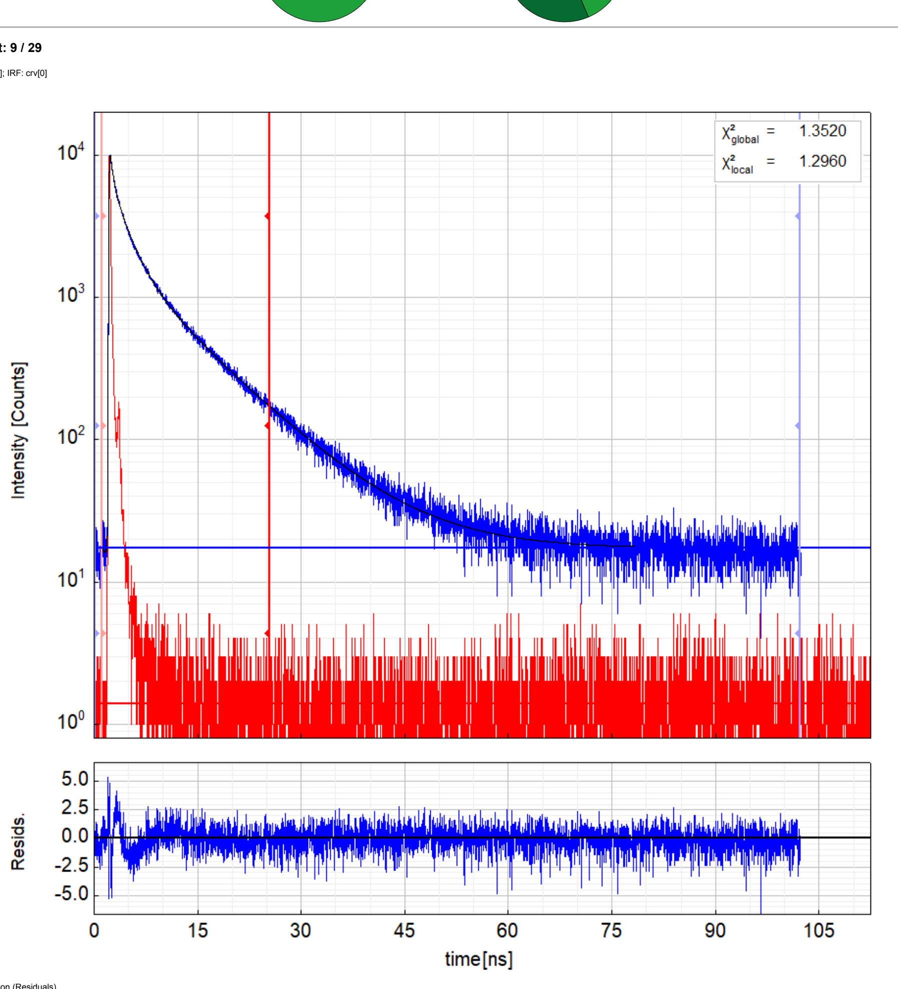

Autocorrelation (Residuals)

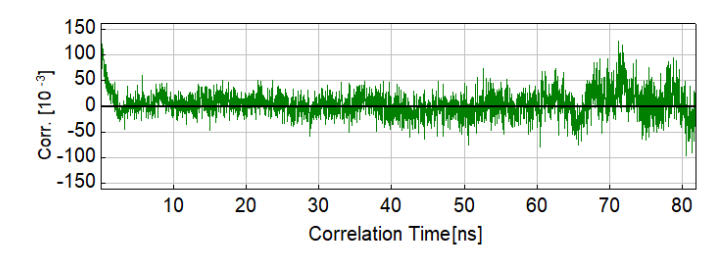

Fitted Parameters

Parameter	Value	Δ	δ
A_1[kCnts/Chnl]	4.96	±0.30	6.0%
$τ_1$[ns]	2.23	±0.14	5.9%
I_1[kCnts]	440.5	±5.5	1.2%
A_2[kCnts/Chnl]	1.907	±0.049	2.5%
$τ_2$[ns]	9.233	±0.054	0.6%
I_2[kCnts]	705	±15	2.0%
A_3[kCnts/Chnl]	8.12	±0.66	8.1%
$τ_3$[ns]	0.339	±0.056	16%
I_3[kCnts]	110	±12	10%
$Bkgr_{Dec}$[kCnts]	0.0173	±0.0001	0.4%
$Bkgr_{IRF}$[Cnts/Chnl]	1.40	±0.24	17%
$Shift_{IRF}$[ps]	-14.4	±4.2	29%
A_{Scatt}[kCnts]	28.8	±5.5	19%
$τ_{AvInt}$[ns]	5.992	±0.026	0.4%
$τ_{AvAmp}$[ns]	2.10	±0.13	5.7%
$λ_{Det}$[nm]	520.0000	---	---

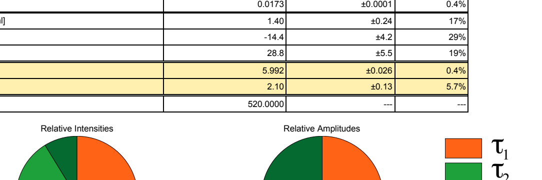

Data Set: 10 / 29

Decay: crv[10]; IRF: crv[0]

Fit

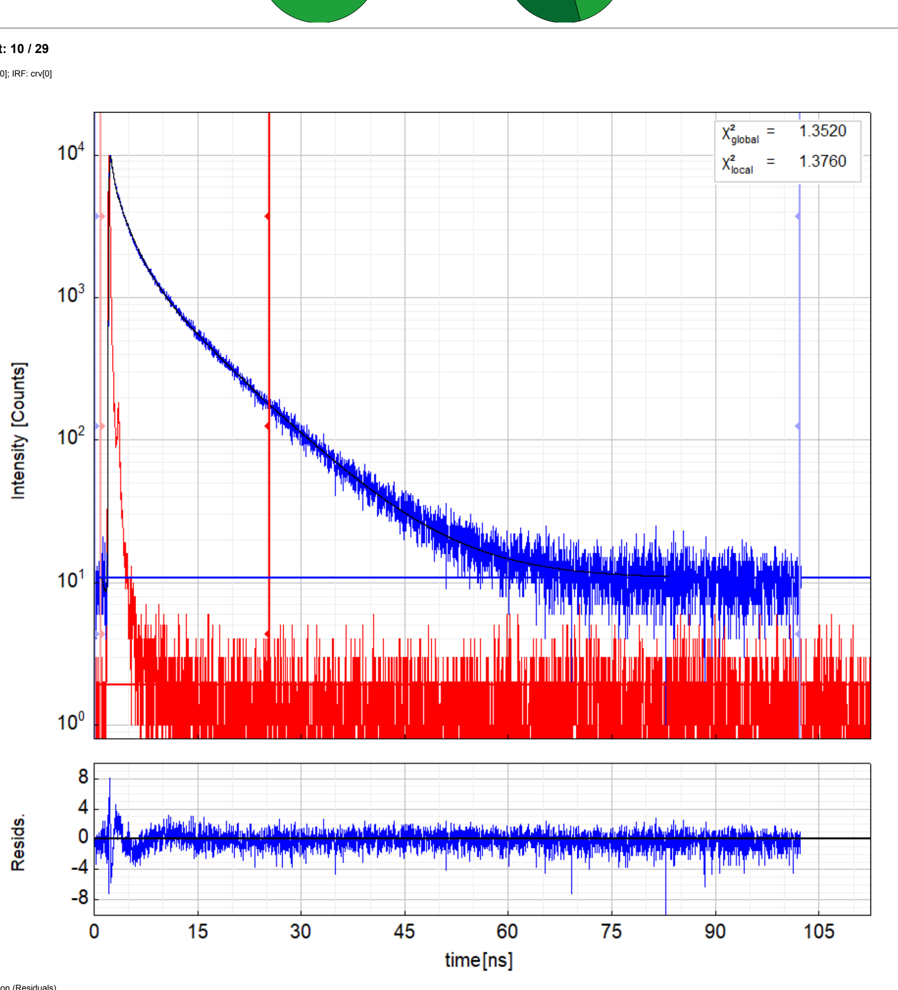

Autocorrelation (Residuals)

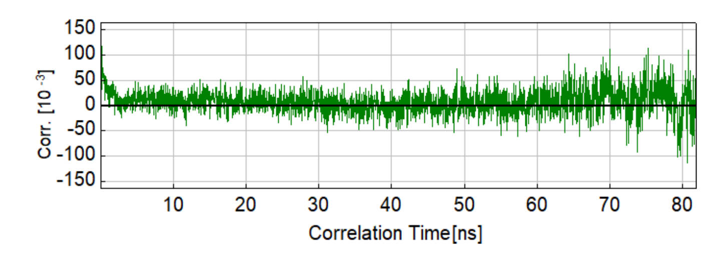

Fitted Parameters

Parameter	Value	Δ	δ
A_1[kCnts/Chnl]	5.32	±0.30	5.6%
$τ_1$[ns]	2.23	±0.14	5.9%
I_1[kCnts]	472.8	±5.8	1.2%
A_2[kCnts/Chnl]	2.126	±0.056	2.6%
$τ_2$[ns]	9.233	±0.054	0.6%
I_2[kCnts]	786	±16	1.9%
A_3[kCnts/Chnl]	10.24	±0.58	5.6%
$τ_3$[ns]	0.339	±0.056	16%
I_3[kCnts]	139	±16	11%
$Bkgr_{Dec}$[kCnts]	0.0108	±0.0002	1.6%
$Bkgr_{IRF}$[Cnts/Chnl]	1.91	±0.12	6.0%
$Shift_{IRF}$[ps]	-78.5	±6.4	8.0%
A_{Scatt}[kCnts]	-21.6	±1.8	8.2%
$τ_{AvInt}$[ns]	5.976	±0.013	0.2%
$τ_{AvAmp}$[ns]	1.98	±0.11	5.6%
$λ_{Det}$[nm]	530.0000	---	---

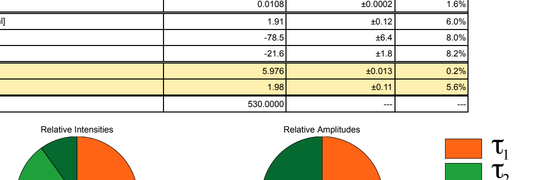

Data Set: 11 / 29

Decay: crv[11]; IRF: crv[0]

Fit

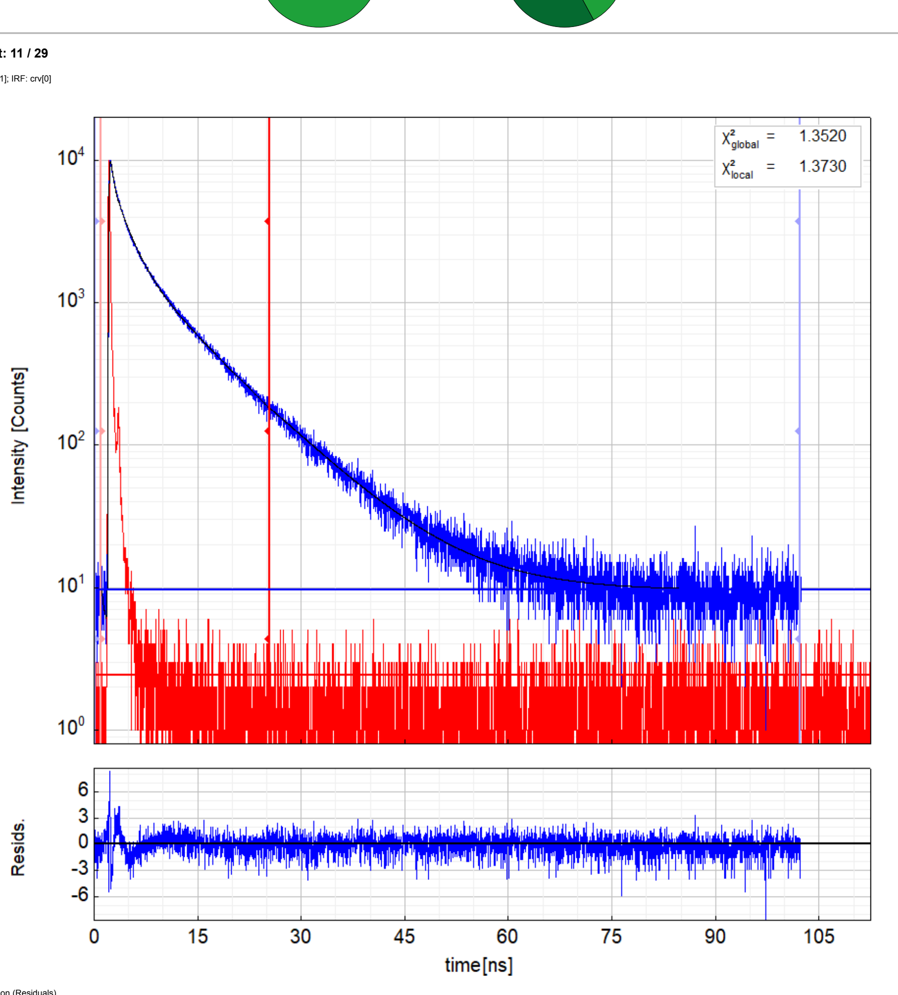

Autocorrelation (Residuals)

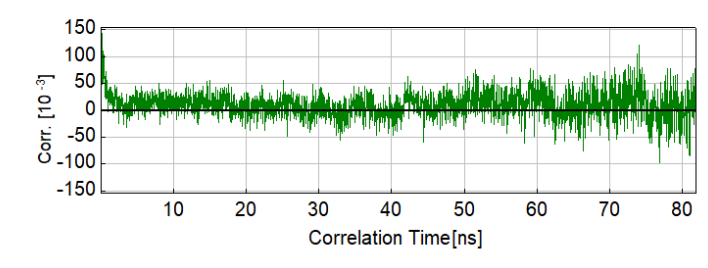

Fitted Parameters

Parameter	Value	Δ	δ
A_1[kCnts/Chnl]	5.40	±0.31	5.6%
$τ_1$[ns]	2.23	±0.14	5.9%
I_1[kCnts]	479.8	±5.2	1.1%
A_2[kCnts/Chnl]	2.284	±0.057	2.5%
$τ_2$[ns]	9.233	±0.054	0.6%
I_2[kCnts]	844	±16	1.8%
A_3[kCnts/Chnl]	9.69	±0.71	7.3%
$τ_3$[ns]	0.339	±0.056	16%
I_3[kCnts]	132	±12	8.6%
$Bkgr_{Dec}$[kCnts]	0.0096	±0.0001	0.8%
$Bkgr_{IRF}$[Cnts/Chnl]	2.43	±0.16	6.5%
$Shift_{IRF}$[ps]	-73.8	±4.9	6.6%
A_{Scatt}[kCnts]	-20.3	±1.7	8.2%
$τ_{AvInt}$[ns]	6.118	±0.027	0.4%
$τ_{AvAmp}$[ns]	2.10	±0.14	6.3%
$λ_{Det}$[nm]	540.0000	---	---

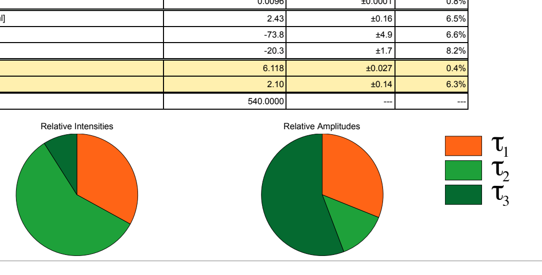

Data Set: 12 / 29

Decay: crv[12]; IRF: crv[0]

Fit

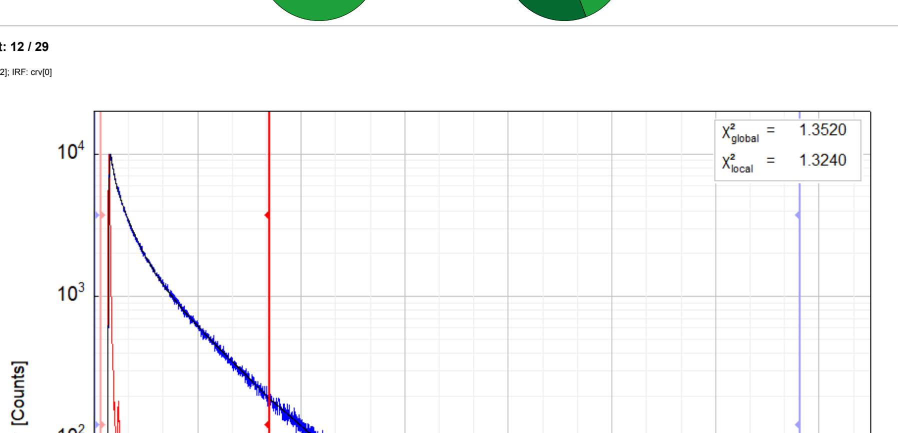

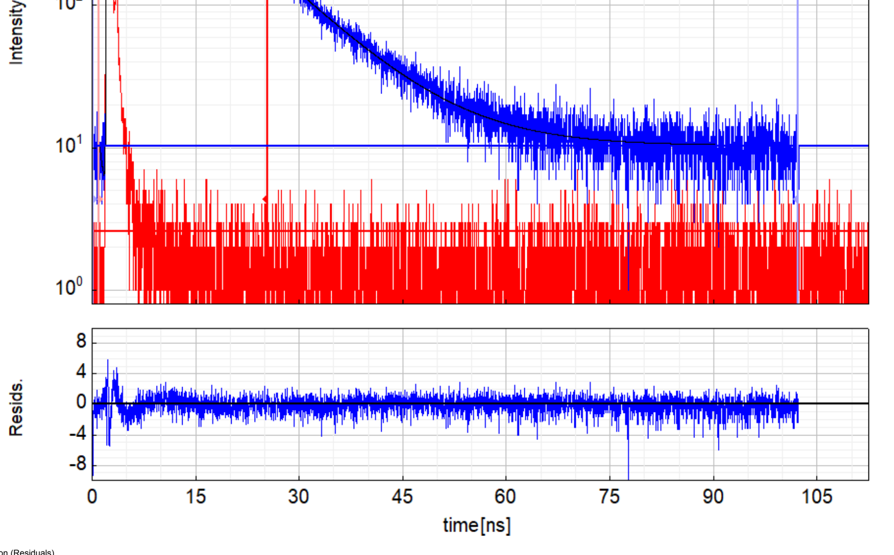

Autocorrelation (Residuals)

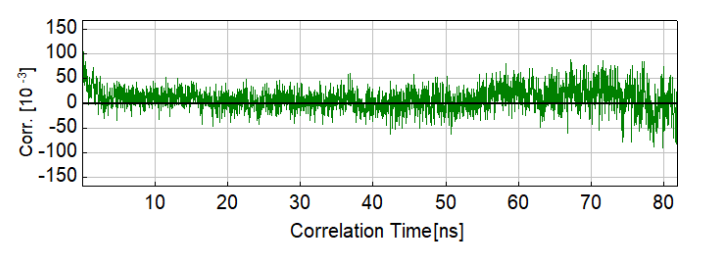

Fitted Parameters

Parameter	Value	Δ	δ
A_1[kCnts/Chnl]	5.60	±0.30	5.2%
τ_1[ns]	2.23	±0.14	5.9%
I_1[kCnts]	497.7	±4.9	1.0%
A_2[kCnts/Chnl]	2.416	±0.054	2.2%
τ_2[ns]	9.233	±0.054	0.6%
I_2[kCnts]	893	±14	1.5%
A_3[kCnts/Chnl]	9.36	±0.39	4.2%
τ_3[ns]	0.339	±0.056	16%
I_3[kCnts]	127	±17	13%
$Bkgr_{Dec}$[kCnts]	0.0104	±0.0001	0.9%
$Bkgr_{IRF}$[Cnts/Chnl]	2.58	±0.20	7.4%
$Shift_{IRF}$[ps]	-74.6	±4.0	5.4%
A_{Scatt}[kCnts]	-20.27	±0.75	3.7%
τ_{AvInt}[ns]	6.189	±0.016	0.2%
τ_{AvAmp}[ns]	2.184	±0.097	4.4%
λ_{Det}[nm]	550.0000	---	---

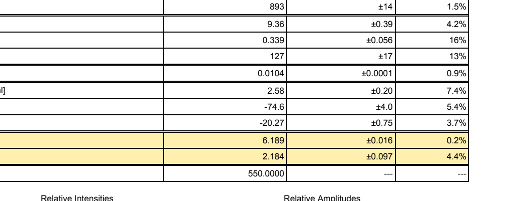

Data Set: 13 / 29

Decay: crv[13]; IRF: crv[0]

Fit

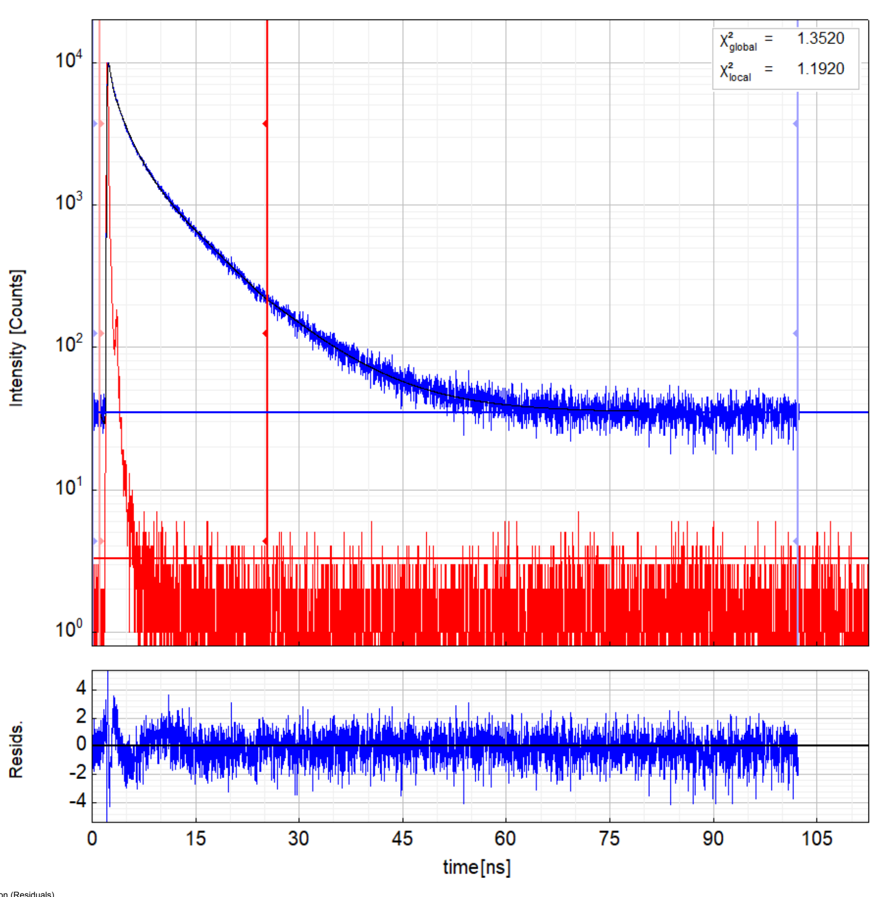

Autocorrelation (Residuals)

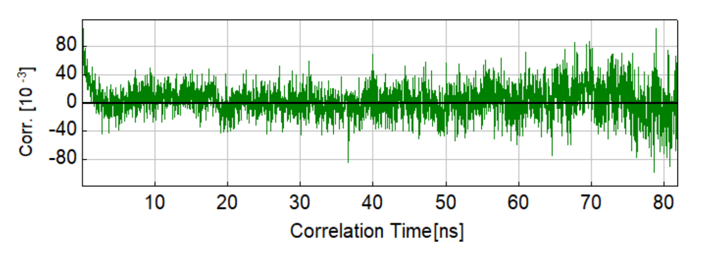

Fitted Parameters

Parameter	Value	Δ	δ
A_1[kCnts/Chnl]	5.44	±0.33	6.0%
τ_1[ns]	2.23	±0.14	5.9%
I_1[kCnts]	483.0	±2.6	0.5%
A_2[kCnts/Chnl]	2.501	±0.062	2.5%
τ_2[ns]	9.233	±0.054	0.6%
I_2[kCnts]	924	±18	1.9%
A_3[kCnts/Chnl]	7.92	±0.51	6.4%
τ_3[ns]	0.339	±0.056	16%
I_3[kCnts]	108	±13	11%
$Bkgr_{Dec}$[kCnts]	0.0351	±0.0002	0.5%
$Bkgr_{IRF}$[Cnts/Chnl]	3.31	±0.24	7.1%
$Shift_{IRF}$[ps]	-27	±11	36%
A_{Scatt}[kCnts]	10.8	±9.6	89%
τ_{AvInt}[ns]	6.366	±0.017	0.3%
τ_{AvAmp}[ns]	2.39	±0.14	5.7%
λ_{Det}[nm]	560.0000	---	---

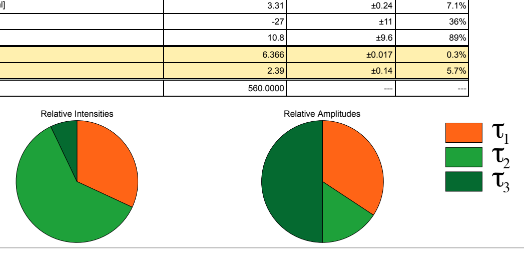

Data Set: 14 / 29

Decay: crv[14]; IRF: crv[0]

Fit

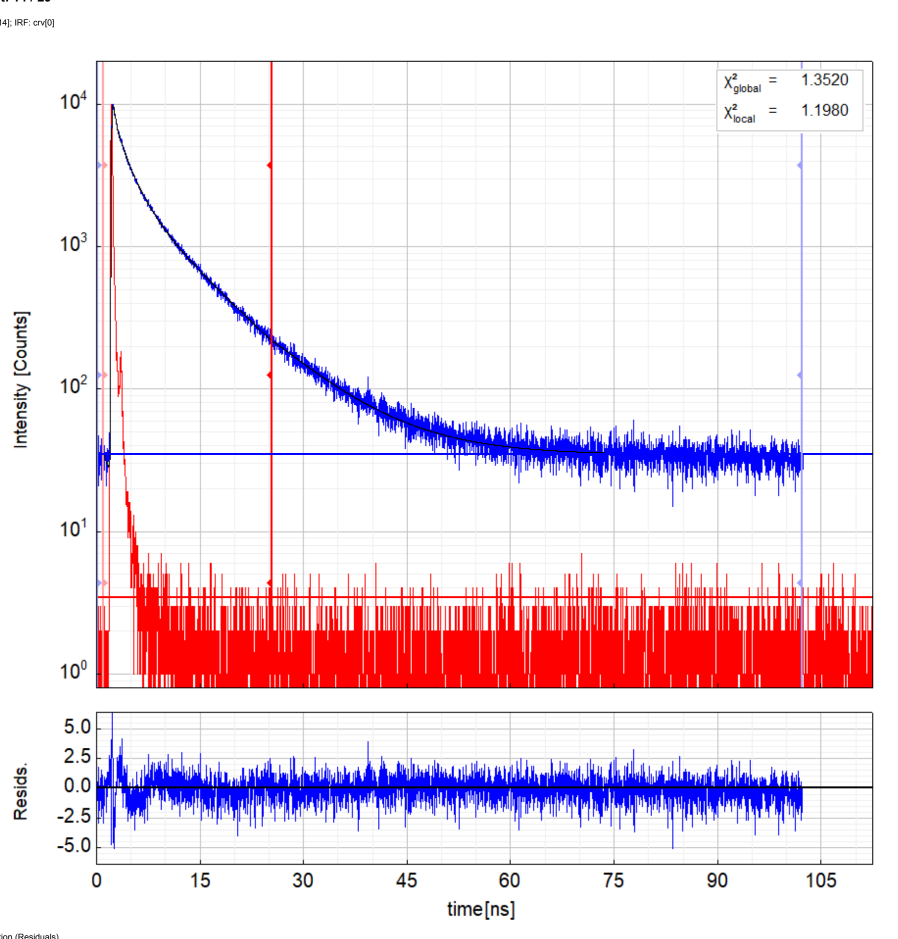

Autocorrelation (Residuals)

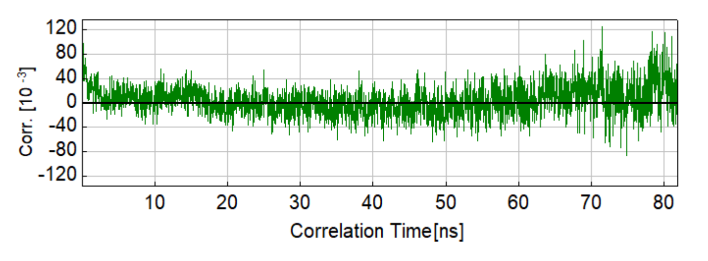

Fitted Parameters

Parameter	Value	Δ	δ
A_1[kCnts/Chnl]	5.47	±0.28	5.1%
τ_1[ns]	2.23	±0.14	5.9%
I_1[kCnts]	486.1	±4.4	0.9%
A_2[kCnts/Chnl]	2.590	±0.059	2.3%
τ_2[ns]	9.233	±0.054	0.6%
I_2[kCnts]	957	±17	1.7%
A_3[kCnts/Chnl]	9.06	±0.52	5.7%
τ_3[ns]	0.339	±0.056	16%
I_3[kCnts]	123	±14	11%
$Bkgr_{Dec}$[kCnts]	0.0346	±0.0002	0.5%
$Bkgr_{IRF}$[Cnts/Chnl]	3.43	±0.21	6.1%
$Shift_{IRF}$[ps]	-71.1	±5.7	7.9%
A_{Scatt}[kCnts]	-19.8	±1.9	9.3%
τ_{AvInt}[ns]	6.359	±0.012	0.2%
τ_{AvAmp}[ns]	2.29	±0.12	5.2%
λ_{Det}[nm]	570.0000	---	---

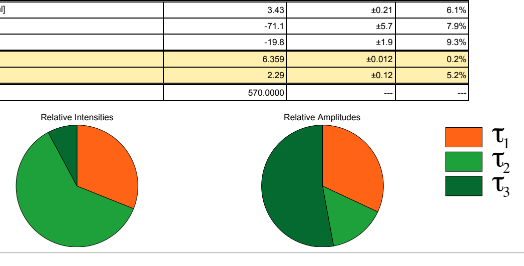

Data Set: 15 / 29

Decay: crv[15]; IRF: crv[0]

Fit

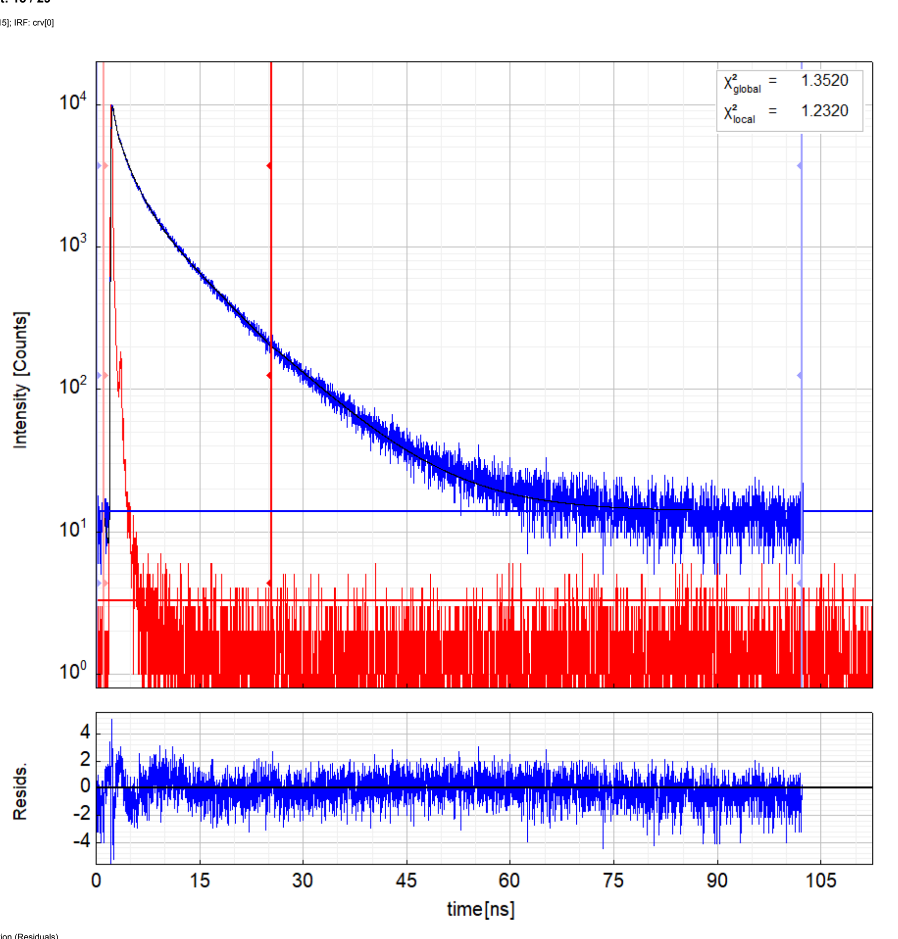

Autocorrelation (Residuals)

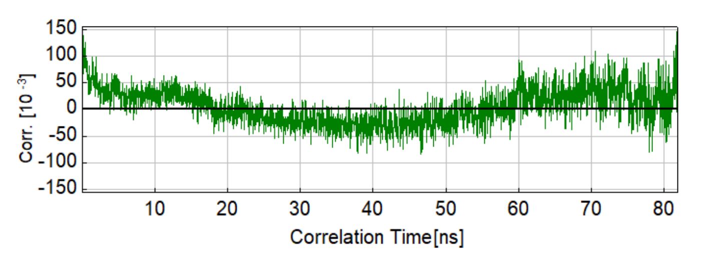

Fitted Parameters

Parameter	Value	Δ	δ
A_1[kCnts/Chnl]	5.29	±0.27	5.1%
τ_1[ns]	2.23	±0.14	5.9%
I_1[kCnts]	470.4	±3.1	0.7%
A_2[kCnts/Chnl]	2.583	±0.064	2.4%
τ_2[ns]	9.233	±0.054	0.6%
I_2[kCnts]	954	±18	1.9%
A_3[kCnts/Chnl]	7.59	±0.66	8.7%
τ_3[ns]	0.339	±0.056	16%
I_3[kCnts]	102.8	±8.5	8.3%
$Bkgr_{Dec}$[kCnts]	0.0140	±0.0002	0.9%
$Bkgr_{IRF}$[Cnts/Chnl]	3.28	±0.16	4.8%
$Shift_{IRF}$[ps]	-28	±12	41%
A_{Scatt}[kCnts]	11	±11	96%
τ_{AvInt}[ns]	6.475	±0.021	0.3%
τ_{AvAmp}[ns]	2.48	±0.18	6.9%
λ_{Det}[nm]	580.0000	---	---

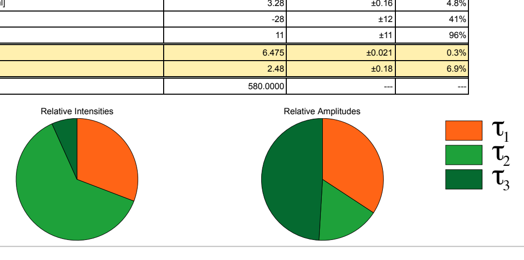

Data Set: 16 / 29

Decay: crv[16]; IRF: crv[0]

Fit

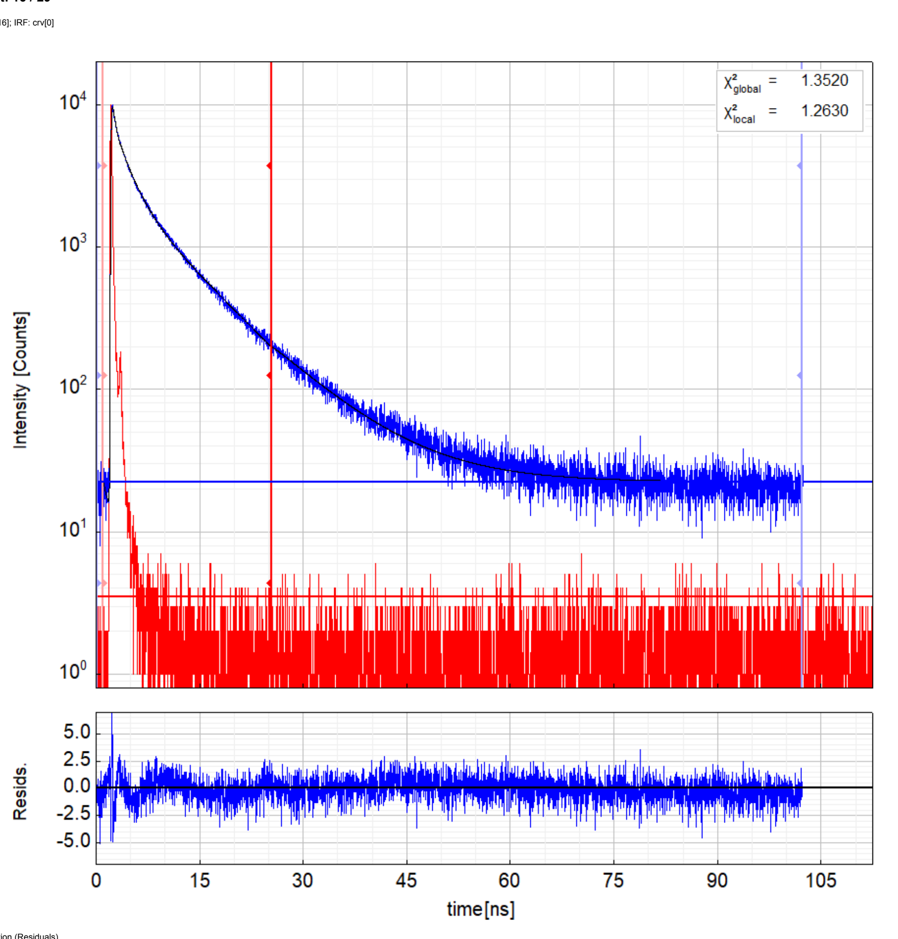

Autocorrelation (Residuals)

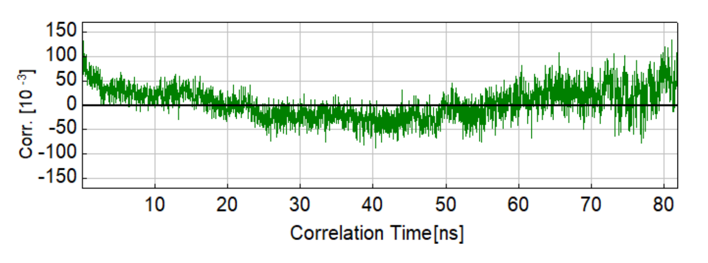

Fitted Parameters

Parameter	Value	Δ	δ
A_1[kCnts/Chnl]	5.23	±0.29	5.4%
τ_1[ns]	2.23	±0.14	5.9%
I_1[kCnts]	464.5	±2.5	0.5%
A_2[kCnts/Chnl]	2.510	±0.046	1.8%
τ_2[ns]	9.233	±0.054	0.6%
I_2[kCnts]	927	±13	1.4%
A_3[kCnts/Chnl]	8.95	±0.48	5.3%
τ_3[ns]	0.339	±0.056	16%
I_3[kCnts]	122	±14	11%
$Bkgr_{Dec}$[kCnts]	0.0225	±0.0002	0.8%
$Bkgr_{IRF}$[Cnts/Chnl]	3.51	±0.13	3.5%
$Shift_{IRF}$[ps]	-71.1	±5.9	8.3%
A_{Scatt}[kCnts]	-17.8	±1.1	5.9%
τ_{AvInt}[ns]	6.367	±0.014	0.2%
τ_{AvAmp}[ns]	2.27	±0.12	4.9%
λ_{Det}[nm]	590.0000	---	---

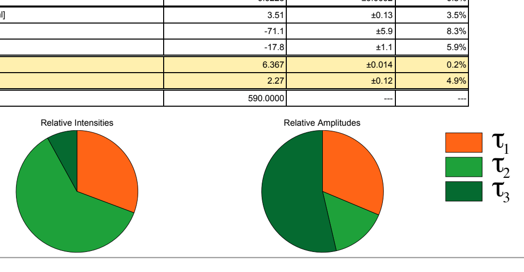

Data Set: 17 / 29

Decay: crv[17]; IRF: crv[0]

Fit

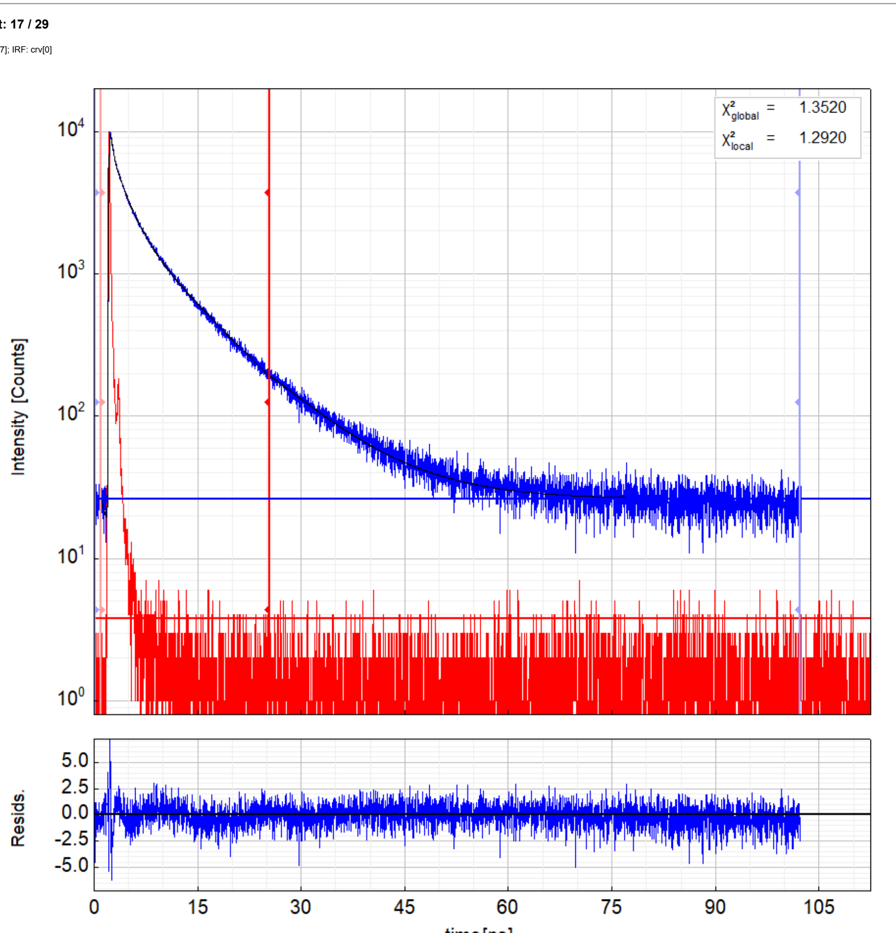

Autocorrelation (Residuals)

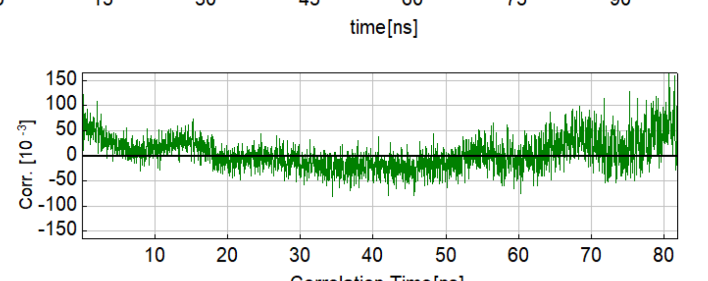

Fitted Parameters

Parameter	Value	Δ	δ
A_1[kCnts/Chnl]	5.15	±0.30	5.8%
τ_1[ns]	2.23	±0.14	5.9%
I_1[kCnts]	457.6	±6.1	1.3%
A_2[kCnts/Chnl]	2.391	±0.058	2.4%
τ_2[ns]	9.233	±0.054	0.6%
I_2[kCnts]	883	±17	1.8%
A_3[kCnts/Chnl]	9.26	±0.58	6.2%
τ_3[ns]	0.339	±0.056	16%
I_3[kCnts]	126	±15	11%
$Bkgr_{Dec}$[kCnts]	0.0261	±0.0002	0.4%
$Bkgr_{IRF}$[Cnts/Chnl]	3.79	±0.15	3.8%
$Shift_{IRF}$[ps]	-78.1	±6.8	8.6%
A_{Scatt}[kCnts]	-20.6	±3.9	18%
τ_{AvInt}[ns]	6.284	±0.015	0.2%
τ_{AvAmp}[ns]	2.19	±0.12	5.5%
λ_{Det}[nm]	600.0000	---	---

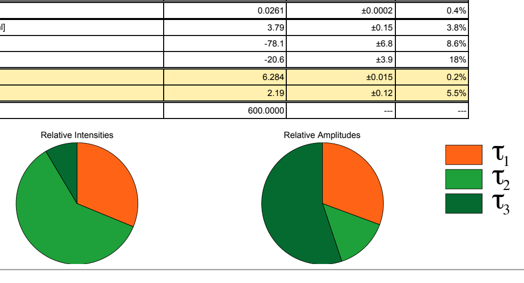

Data Set: 18 / 29

Decay: crv[18]; IRF: crv[0]

Fit

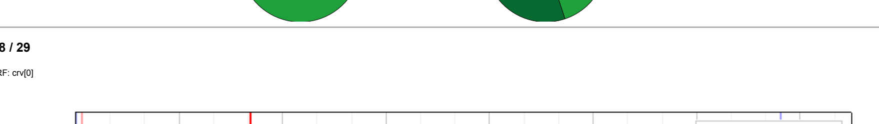

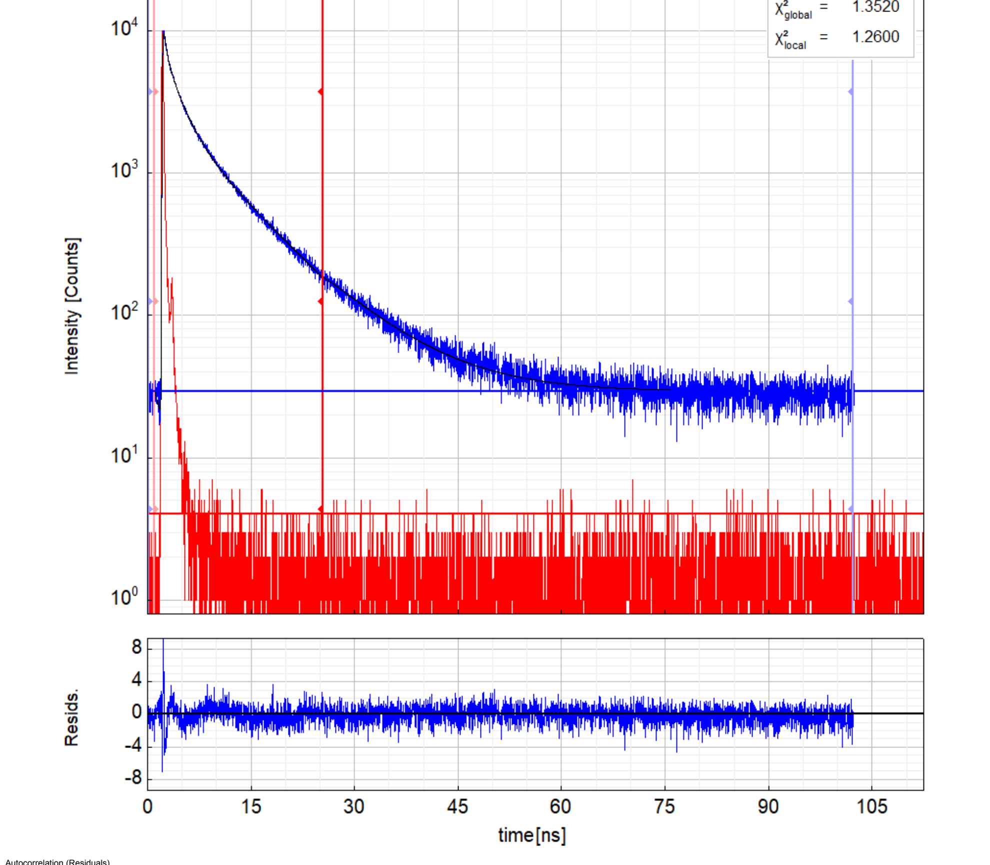

Autocorrelation (Residuals)

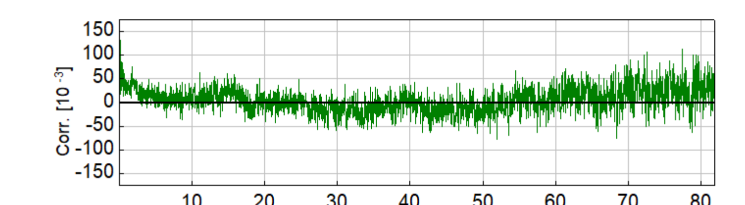

Fitted Parameters

Parameter	Value	Δ	δ
A_1[kCnts/Chnl]	5.03	±0.28	5.4%
τ_1[ns]	2.23	±0.14	5.9%
I_1[kCnts]	446.4	±3.6	0.8%
A_2[kCnts/Chnl]	2.298	±0.048	2.1%
τ_2[ns]	9.233	±0.054	0.6%
I_2[kCnts]	849	±15	1.7%
A_3[kCnts/Chnl]	9.59	±0.54	5.6%
τ_3[ns]	0.339	±0.056	16%
I_3[kCnts]	130	±14	11%
$Bkgr_{Dec}$[kCnts]	0.0292	±0.0001	0.3%
$Bkgr_{IRF}$[Cnts/Chnl]	4.04	±0.14	3.3%
$Shift_{IRF}$[ps]	-78	±14	17%
A_{Scatt}[kCnts]	-20.0	±4.0	20%
τ_{AvInt}[ns]	6.226	±0.012	0.2%
τ_{AvAmp}[ns]	2.11	±0.12	5.4%
λ_{Det}[nm]	610.0000	---	---

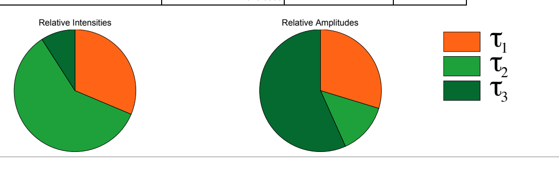

Data Set: 19 / 29

Decay: crv[19]; IRF: crv[0]

Fit

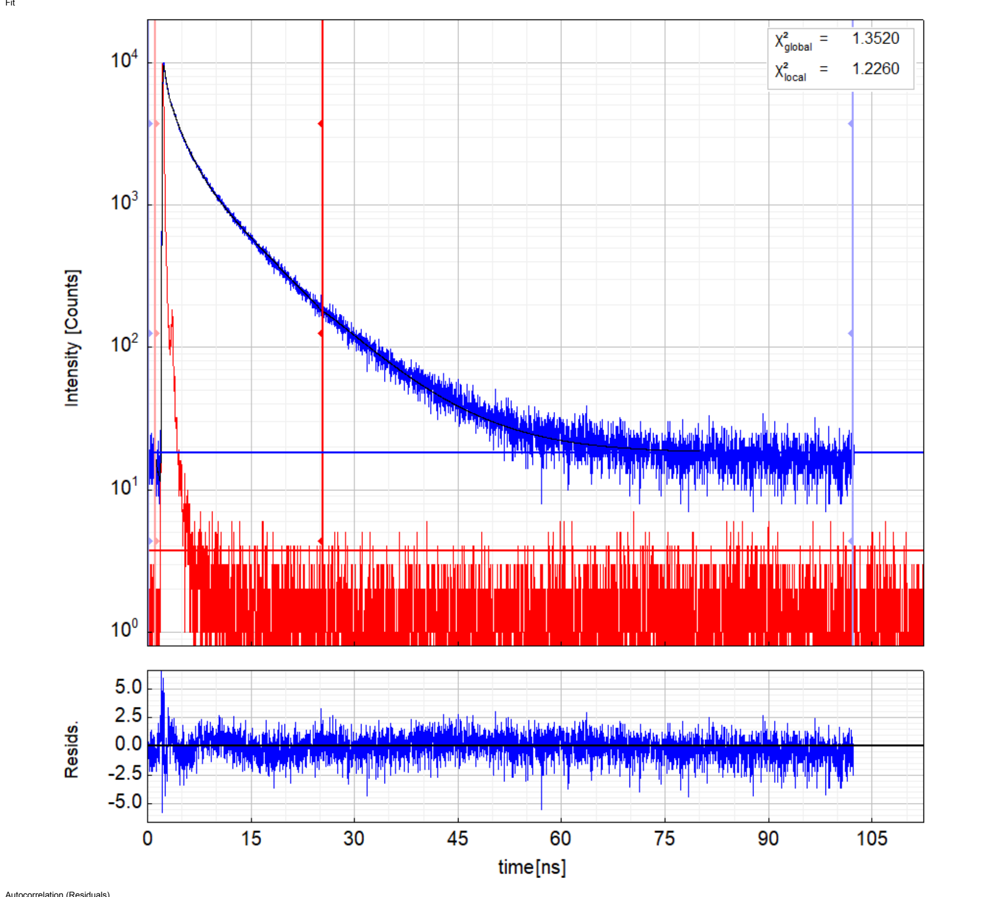

Autocorrelation (Residuals)

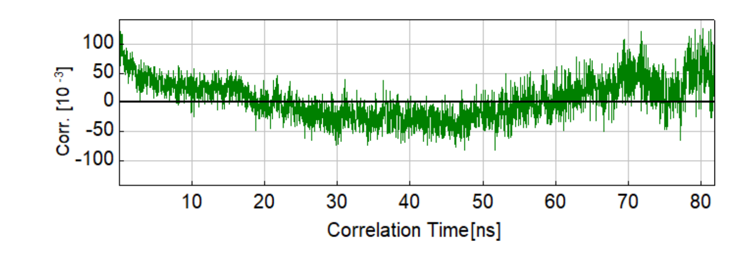

Fitted Parameters

Parameter	Value	Δ	δ
A_1[kCnts/Chnl]	4.83	±0.28	5.7%
τ_1[ns]	2.23	±0.14	5.9%
I_1[kCnts]	429.3	±3.2	0.7%
A_2[kCnts/Chnl]	2.306	±0.051	2.2%
τ_2[ns]	9.233	±0.054	0.6%
I_2[kCnts]	852	±15	1.8%
A_3[kCnts/Chnl]	7.00	±0.62	8.7%
τ_3[ns]	0.339	±0.056	16%
I_3[kCnts]	94.9	±7.7	8.0%
$Bkgr_{Dec}$[kCnts]	0.0182	±0.0001	0.4%
$Bkgr_{IRF}$[Cnts/Chnl]	3.74	±0.24	6.4%
$Shift_{IRF}$[ps]	-14.2	±4.3	30%
A_{Scatt}[kCnts]	28.5	±6.1	21%
τ_{AvInt}[ns]	6.432	±0.020	0.3%
τ_{AvAmp}[ns]	2.44	±0.15	5.9%
λ_{Det}[nm]	620.0000	---	---

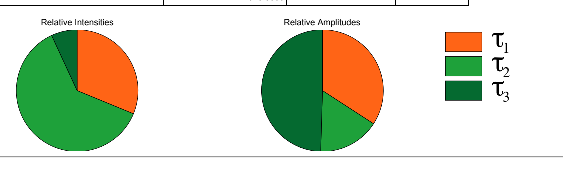

Data Set: 20 / 29

Decay: crv[20]; IRF: crv[0]

Fit

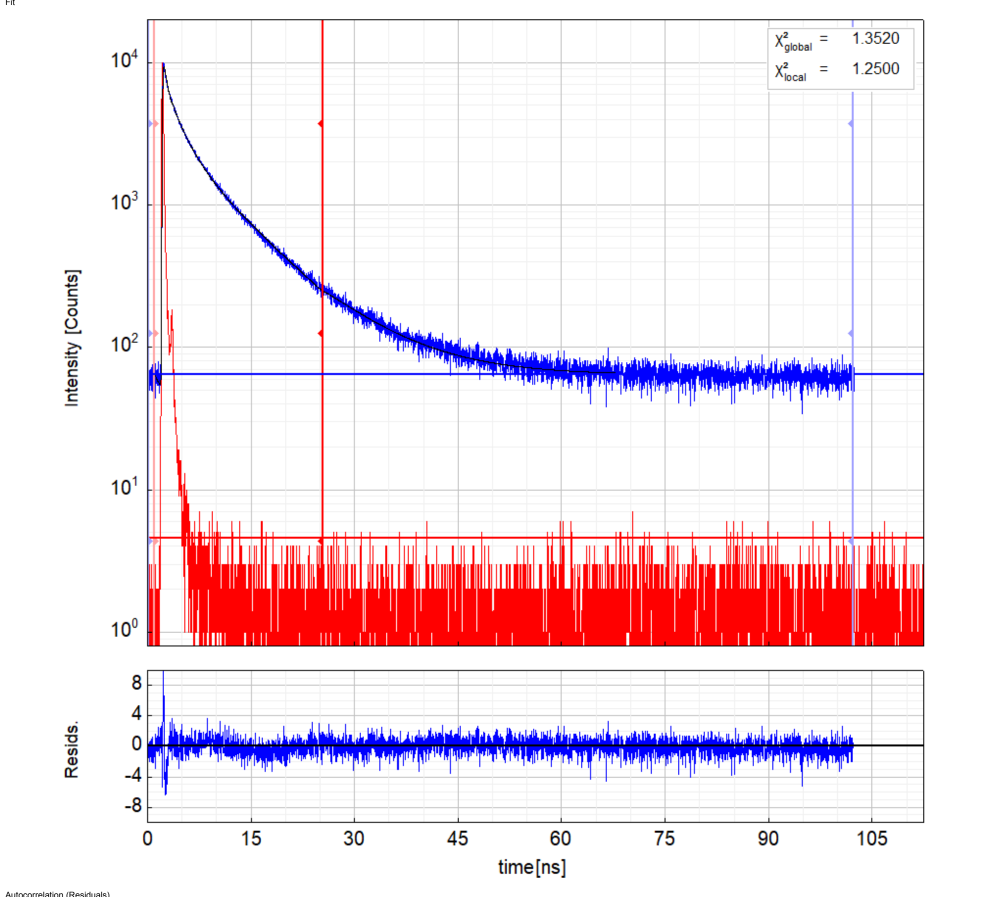

Autocorrelation (Residuals)

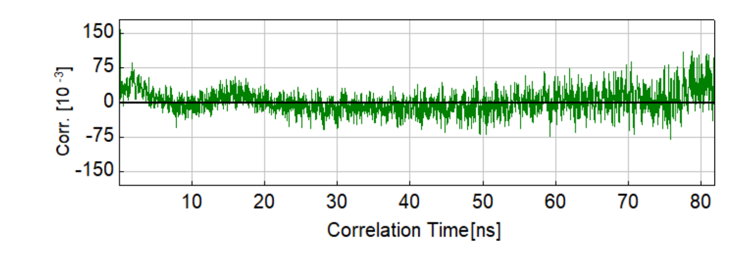

Fitted Parameters

Parameter	Value	Δ	δ
A_1[kCnts/Chnl]	4.80	±0.22	4.4%
τ_1[ns]	2.23	±0.14	5.9%
I_1[kCnts]	426.2	±6.5	1.5%
A_2[kCnts/Chnl]	2.775	±0.056	2.0%
τ_2[ns]	9.233	±0.054	0.6%
I_2[kCnts]	1 025	±15	1.4%
A_3[kCnts/Chnl]	8.58	±0.77	8.9%
τ_3[ns]	0.339	±0.056	16%
I_3[kCnts]	117	±12	10.0%
$Bkgr_{Dec}$[kCnts]	0.0641	±0.0003	0.3%
$Bkgr_{IRF}$[Cnts/Chnl]	4.61	±0.18	3.8%
$Shift_{IRF}$[ps]	-77.1	±4.1	5.3%
A_{Scatt}[kCnts]	-18.7	±1.1	5.6%
τ_{AvInt}[ns]	6.667	±0.015	0.2%
τ_{AvAmp}[ns]	2.43	±0.15	6.1%
λ_{Det}[nm]	630.0000	---	---

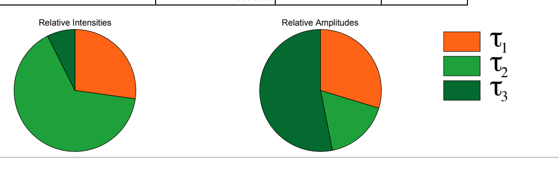

Data Set: 21 / 29

Decay: crv[21]; IRF: crv[0]

Fit

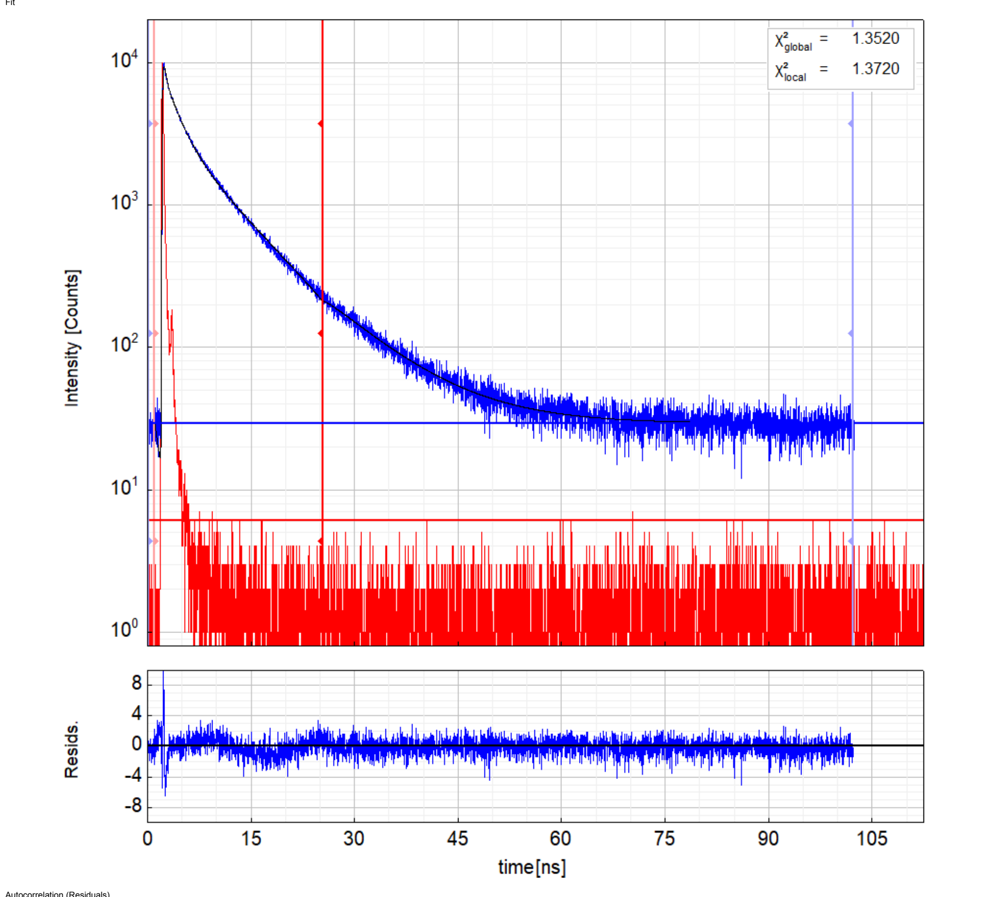

Autocorrelation (Residuals)

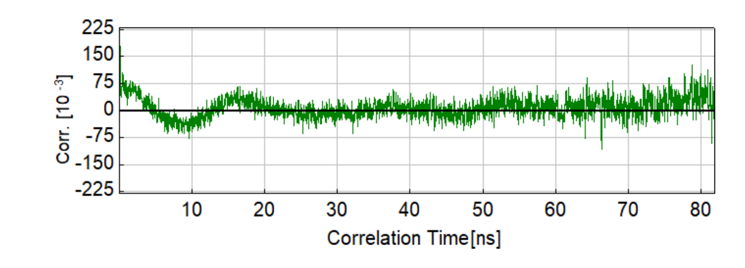

Fitted Parameters

Parameter	Value	Δ	δ
A_1[kCnts/Chnl]	5.24	±0.22	4.0%
τ_1[ns]	2.23	±0.14	5.9%
I_1[kCnts]	465.7	±9.1	1.9%
A_2[kCnts/Chnl]	3.049	±0.072	2.3%
τ_2[ns]	9.233	±0.054	0.6%
I_2[kCnts]	1 126	±18	1.6%
A_3[kCnts/Chnl]	7.39	±0.51	6.8%
τ_3[ns]	0.339	±0.056	16%
I_3[kCnts]	101	±14	13%
$Bkgr_{Dec}$[kCnts]	0.0293	±0.0001	0.2%
$Bkgr_{IRF}$[Cnts/Chnl]	6.07	±0.18	2.9%
$Shift_{IRF}$[ps]	-70	±13	18%
A_{Scatt}[kCnts]	-15.4	±4.5	29%
τ_{AvInt}[ns]	6.777	±0.017	0.3%
τ_{AvAmp}[ns]	2.70	±0.14	5.1%
λ_{Det}[nm]	640.0000	---	---

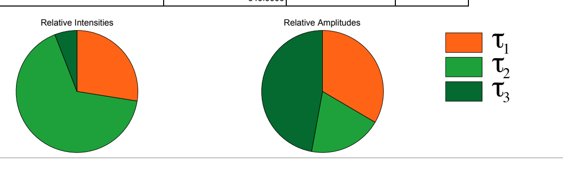

Data Set: 22 / 29

Decay: crv[22]; IRF: crv[0]

Fit

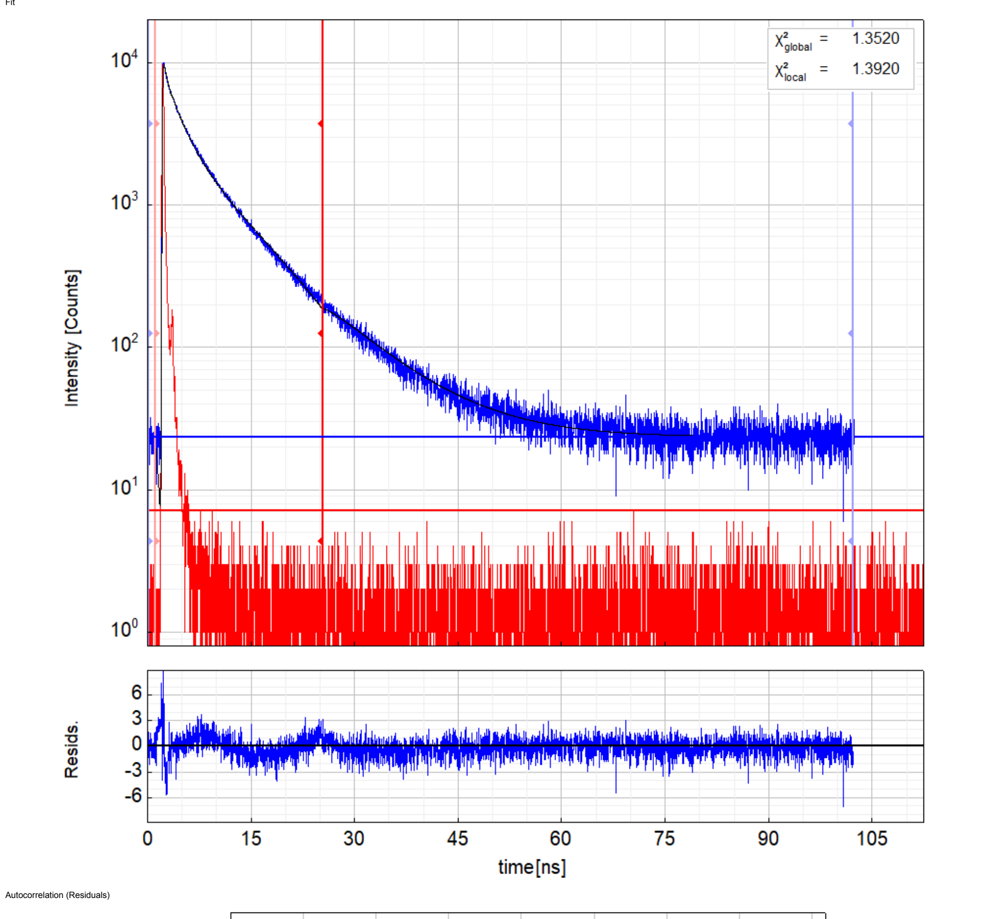

Autocorrelation (Residuals)

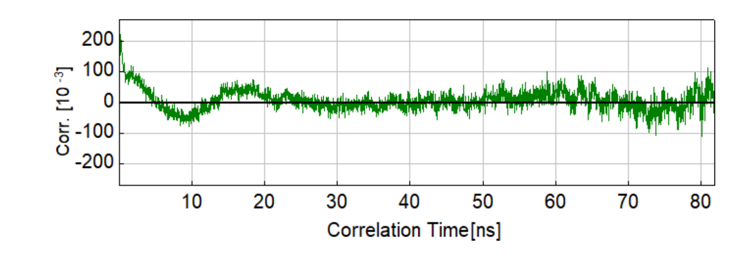

Fitted Parameters

Parameter	Value	Δ	δ
A_1[kCnts/Chnl]	5.661	±0.055	1.0%
τ_1[ns]	2.23	±0.14	5.9%
I_1[kCnts]	504	±21	4.1%
A_2[kCnts/Chnl]	2.98	±0.13	4.1%
τ_2[ns]	9.233	±0.054	0.6%
I_2[kCnts]	1 098	±38	3.4%
A_3[kCnts/Chnl]	5.7	±1.6	27%
τ_3[ns]	0.339	±0.056	16%
I_3[kCnts]	77	±13	16%
$Bkgr_{Dec}$[kCnts]	0.0234	±0.0003	1.0%
$Bkgr_{IRF}$[Cnts/Chnl]	7.09	±0.66	9.3%
$Shift_{IRF}$[ps]	-16.8	±9.0	53%
A_{Scatt}[kCnts]	19	±18	94%
τ_{AvInt}[ns]	6.7248	±0.0095	0.1%
τ_{AvAmp}[ns]	2.95	±0.35	12%
λ_{Det}[nm]	650.0000	---	---

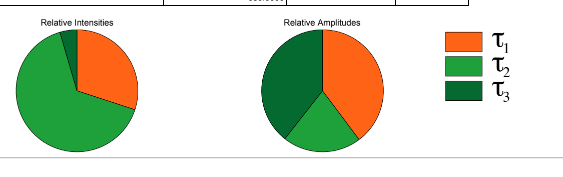

Data Set: 23 / 29

Decay: crv[23]; IRF: crv[0]

Fit

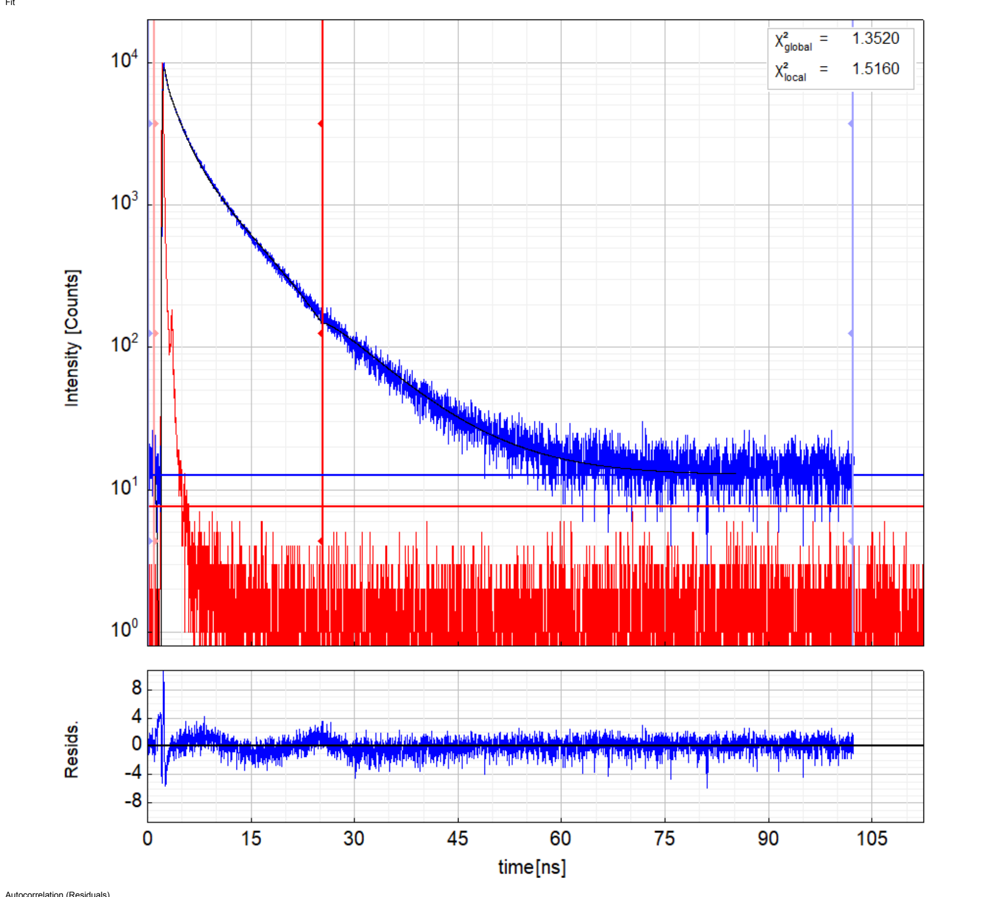

Autocorrelation (Residuals)

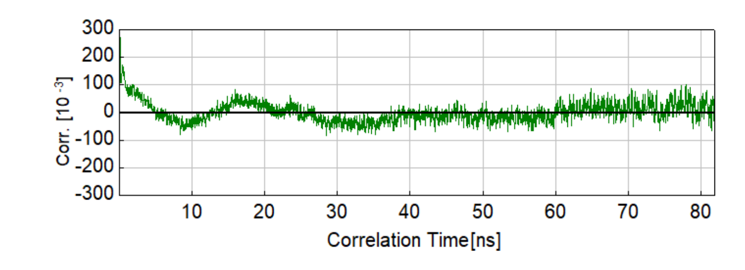

Fitted Parameters

Parameter	Value	Δ	δ
A_1[kCnts/Chnl]	5.66	±0.16	2.7%
τ_1[ns]	2.23	±0.14	5.9%
I_1[kCnts]	503	±12	2.3%
A_2[kCnts/Chnl]	2.623	±0.077	2.9%
τ_2[ns]	9.233	±0.054	0.6%
I_2[kCnts]	969	±27	2.7%
A_3[kCnts/Chnl]	6.94	±0.72	10%
τ_3[ns]	0.339	±0.056	16%
I_3[kCnts]	94	±14	14%
$Bkgr_{Dec}$[kCnts]	0.0127	±0.0003	1.7%
$Bkgr_{IRF}$[Cnts/Chnl]	7.57	±0.47	6.2%
$Shift_{IRF}$[ps]	-62	±18	28%

A_{Scatt}[kCnts]	-10.0	±8.2	82%
τ_{AvInt}[ns]	6.447	±0.050	0.8%
τ_{AvAmp}[ns]	2.58	±0.14	5.3%
λ_{Det}[nm]	660.0000	---	---

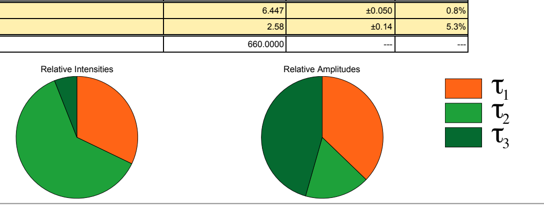

Data Set: 24 / 29

Decay: crv[24]; IRF: crv[0]

Fit

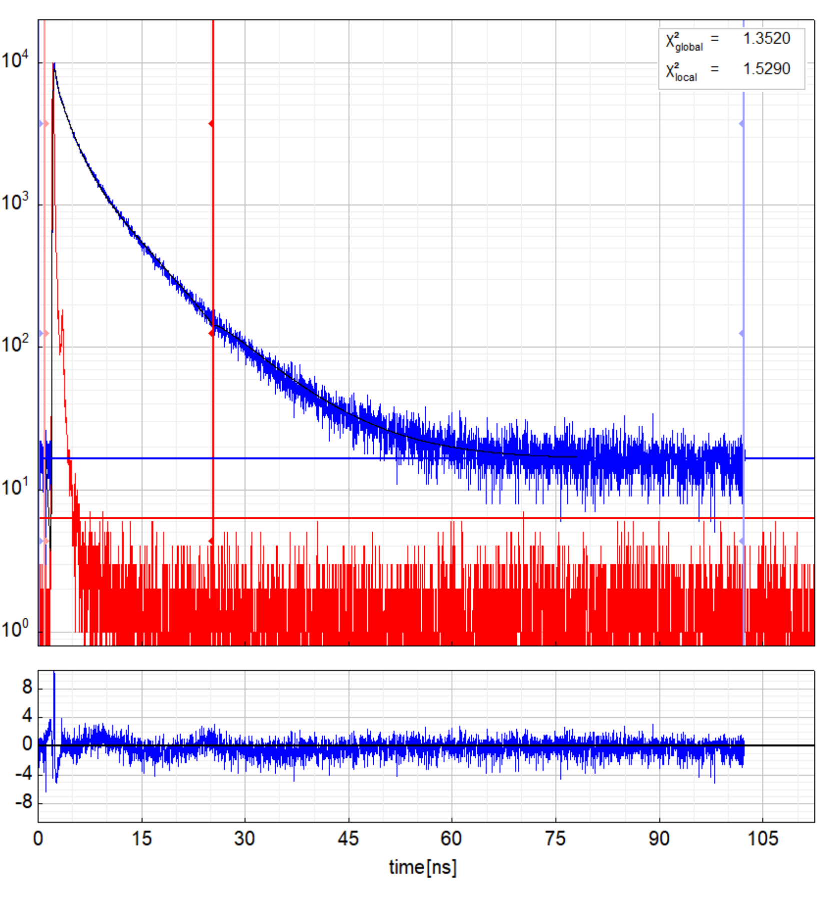

Autocorrelation (Residuals)

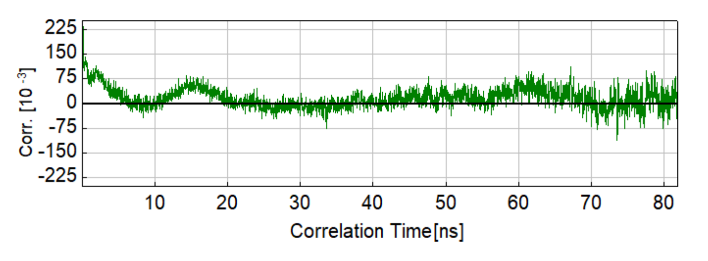

Fitted Parameters

Parameter	Value	Δ	δ
A_1[kCnts/Chnl]	5.57	±0.26	4.6%
τ_1[ns]	2.23	±0.14	5.9%
I_1[kCnts]	495.0	±7.0	1.4%
A_2[kCnts/Chnl]	2.268	±0.068	3.0%
τ_2[ns]	9.233	±0.054	0.6%
I_2[kCnts]	838	±23	2.7%
A_3[kCnts/Chnl]	7.64	±0.48	6.2%
τ_3[ns]	0.339	±0.056	16%
I_3[kCnts]	104	±13	12%
$Bkgr_{Dec}$[kCnts]	0.0164	±0.0002	1.2%
$Bkgr_{IRF}$[Cnts/Chnl]	6.27	±0.43	6.7%
$Shift_{IRF}$[ps]	-76.4	±5.4	7.0%
A_{Scatt}[kCnts]	-16.9	±1.5	8.7%
τ_{AvInt}[ns]	6.175	±0.035	0.6%
τ_{AvAmp}[ns]	2.33	±0.13	5.4%
λ_{Det}[nm]	670.0000	---	---

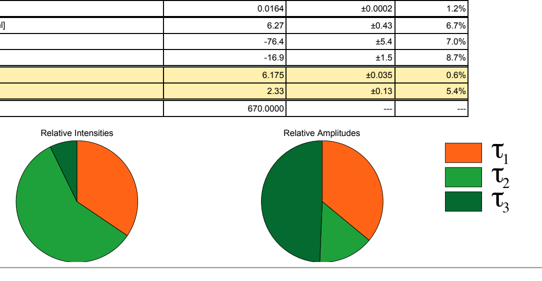

Data Set: 25 / 29

Decay: crv[25]; IRF: crv[0]

Fit

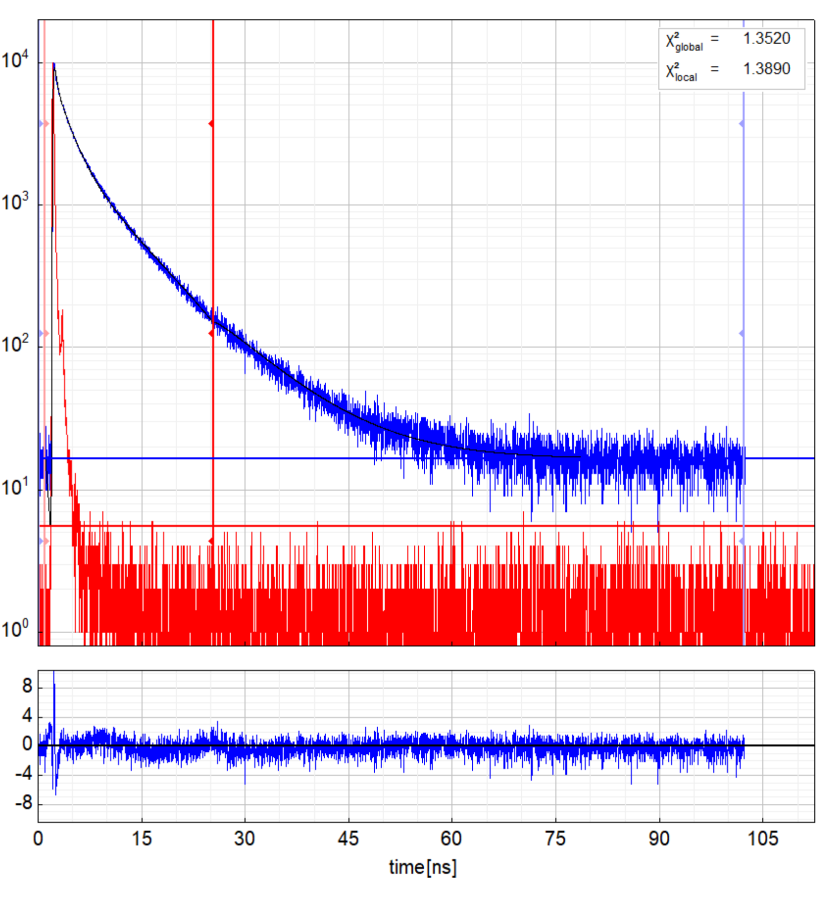

Autocorrelation (Residuals)

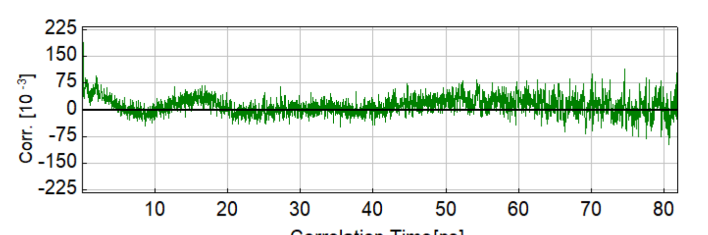

Fitted Parameters

Parameter	Value	Δ	δ
A_1[kCnts/Chnl]	5.37	±0.29	5.3%
τ_1[ns]	2.23	±0.14	5.9%
I_1[kCnts]	477.5	±5.3	1.1%
A_2[kCnts/Chnl]	2.241	±0.054	2.4%
τ_2[ns]	9.233	±0.054	0.6%
I_2[kCnts]	828	±13	1.5%
A_3[kCnts/Chnl]	8.26	±0.44	5.3%
τ_3[ns]	0.339	±0.056	16%
I_3[kCnts]	112	±16	14%
$Bkgr_{Dec}$[kCnts]	0.0165	±0.0002	0.8%
$Bkgr_{IRF}$[Cnts/Chnl]	5.53	±0.14	2.4%
$Shift_{IRF}$[ps]	-79.4	±6.2	7.8%
A_{Scatt}[kCnts]	-18.2	±2.5	13%
τ_{AvInt}[ns]	6.168	±0.020	0.3%
τ_{AvAmp}[ns]	2.232	±0.085	3.8%
λ_{Det}[nm]	680.0000	---	---

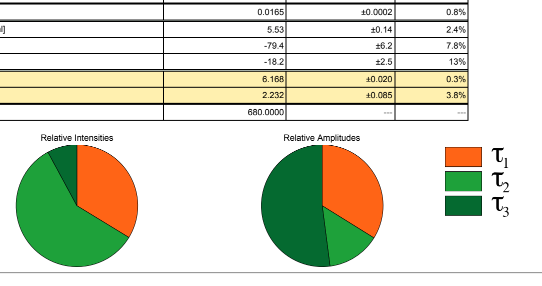

Data Set: 26 / 29

Decay: crv[26]; IRF: crv[0]

Fit

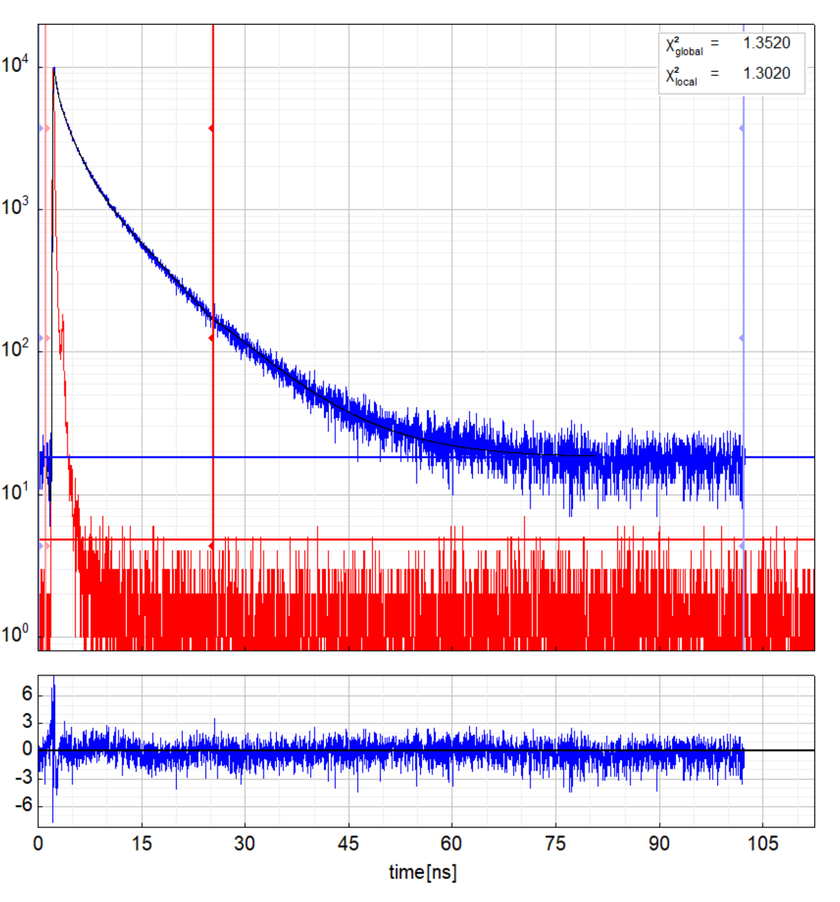

Autocorrelation (Residuals)

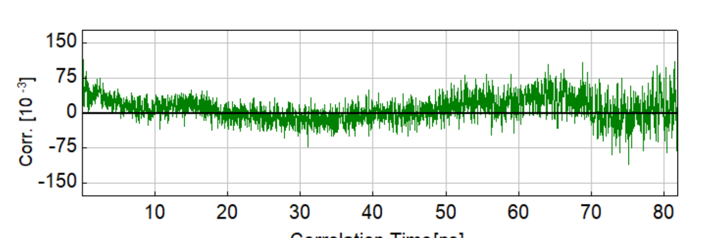

Fitted Parameters

Parameter	Value	Δ	δ
A_1[kCnts/Chnl]	5.05	±0.19	3.7%
τ_1[ns]	2.23	±0.14	5.9%
I_1[kCnts]	448.8	±6.2	1.4%
A_2[kCnts/Chnl]	2.310	±0.078	3.4%
τ_2[ns]	9.233	±0.054	0.6%
I_2[kCnts]	853	±24	2.8%
A_3[kCnts/Chnl]	6.62	±0.94	14%
τ_3[ns]	0.339	±0.056	16%
I_3[kCnts]	89.7	±8.0	8.8%
$Bkgr_{Dec}$[kCnts]	0.0182	±0.0001	0.3%
$Bkgr_{IRF}$[Cnts/Chnl]	4.81	±0.53	11%
$Shift_{IRF}$[ps]	-16.8	±5.3	31%
A_{Scatt}[kCnts]	24	±13	55%
τ_{AvInt}[ns]	6.399	±0.019	0.3%
τ_{AvAmp}[ns]	2.49	±0.20	8.0%
λ_{Det}[nm]	690.0000	---	---

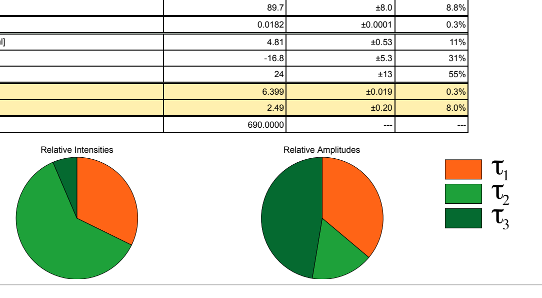

Data Set: 27 / 29

Decay: crv[27]; IRF: crv[0]

Fit

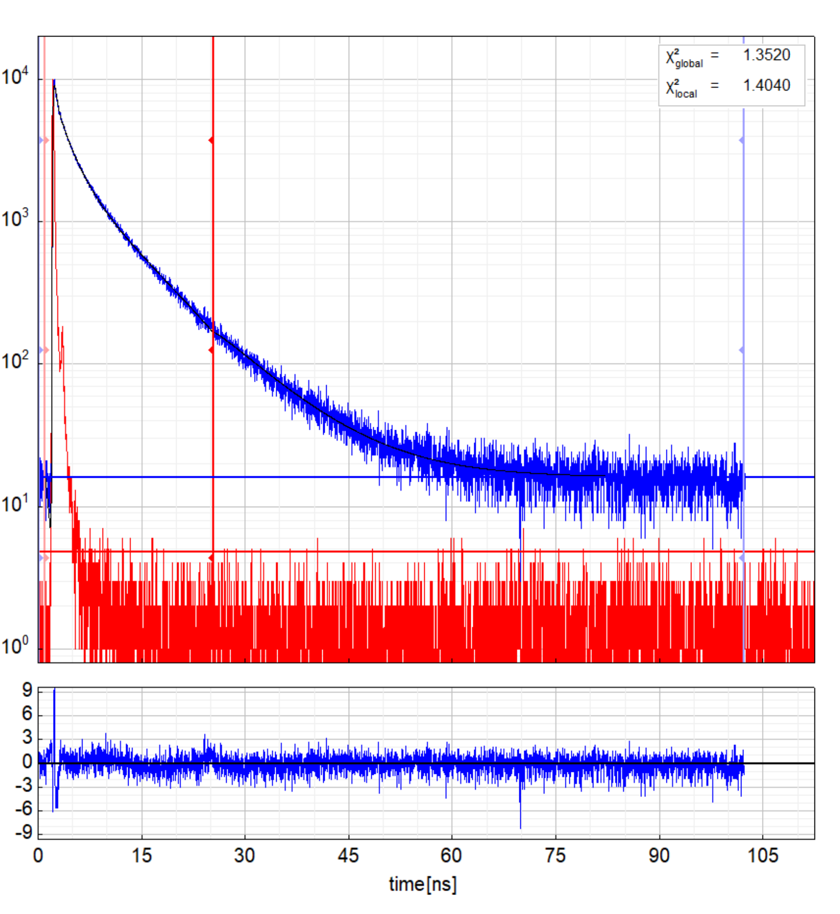

Autocorrelation (Residuals)

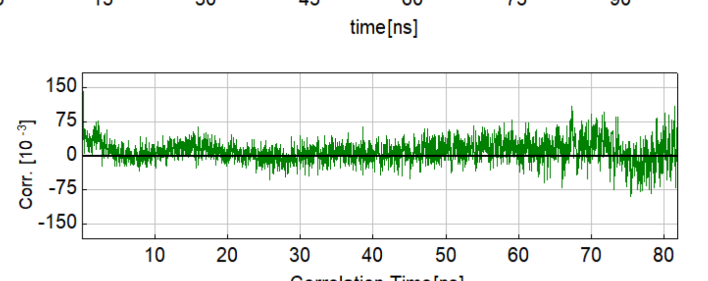

Fitted Parameters

Parameter	Value	Δ	δ
A_1[kCnts/Chnl]	4.93	±0.27	5.4%
τ_1[ns]	2.23	±0.14	5.9%
I_1[kCnts]	437.7	±5.5	1.3%
A_2[kCnts/Chnl]	2.355	±0.047	2.0%
τ_2[ns]	9.233	±0.054	0.6%
I_2[kCnts]	870	±13	1.4%
A_3[kCnts/Chnl]	8.62	±0.50	5.8%
τ_3[ns]	0.339	±0.056	16%
I_3[kCnts]	117	±14	12%
$Bkgr_{Dec}$[kCnts]	0.0161	±0.0001	0.6%
$Bkgr_{IRF}$[Cnts/Chnl]	4.81	±0.25	5.1%
$Shift_{IRF}$[ps]	-76.7	±8.5	11%
A_{Scatt}[kCnts]	-17.7	±1.8	9.8%
τ_{AvInt}[ns]	6.349	±0.014	0.2%
τ_{AvAmp}[ns]	2.24	±0.11	4.8%
λ_{Det}[nm]	700.0000	---	---

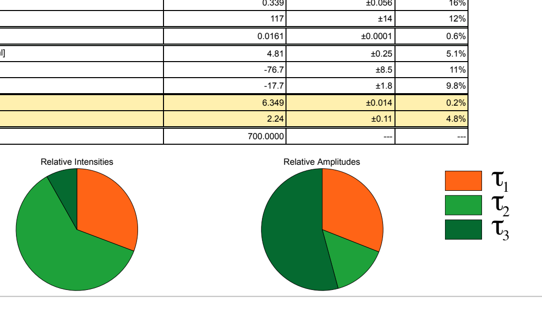

Data Set: 28 / 29

Decay: crv[28]; IRF: crv[0]

Fit

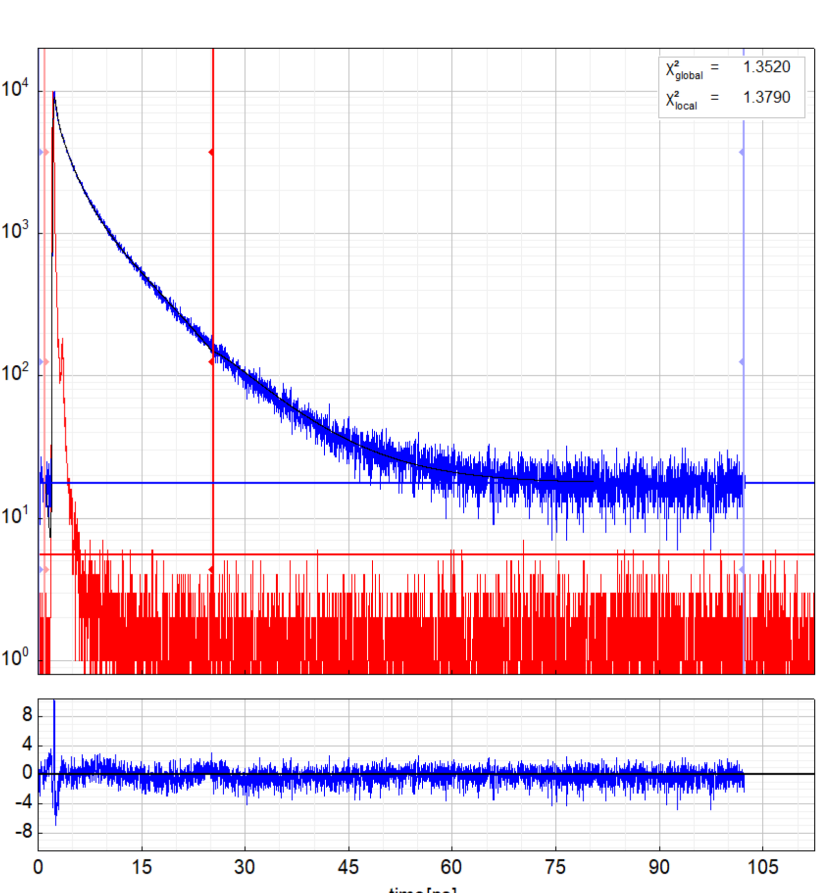

Autocorrelation (Residuals)

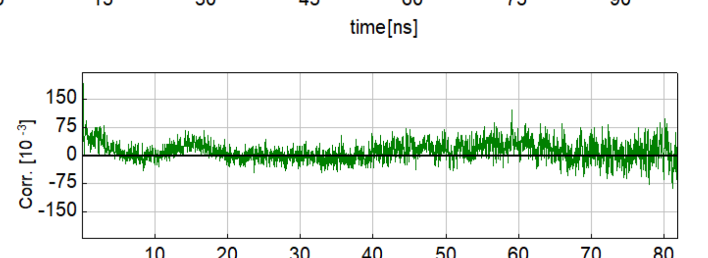

Fitted Parameters

Parameter	Value	Δ	δ
A_1[kCnts/Chnl]	4.92	±0.22	4.4%
τ_1[ns]	2.23	±0.14	5.9%
I_1[kCnts]	437.3	±7.2	1.6%
A_2[kCnts/Chnl]	2.152	±0.065	3.0%
τ_2[ns]	9.233	±0.054	0.6%
I_2[kCnts]	795	±20	2.5%
A_3[kCnts/Chnl]	8.64	±0.64	7.3%
τ_3[ns]	0.339	±0.056	16%
I_3[kCnts]	117	±16	13%
$Bkgr_{Dec}$[kCnts]	0.0177	±0.0002	0.6%
$Bkgr_{IRF}$[Cnts/Chnl]	5.50	±0.37	6.6%
$Shift_{IRF}$[ps]	-67	±15	21%
A_{Scatt}[kCnts]	-12.2	±5.2	43%
τ_{AvInt}[ns]	6.189	±0.035	0.6%
τ_{AvAmp}[ns]	2.15	±0.12	5.2%
λ_{Det}[nm]	710.0000	---	---

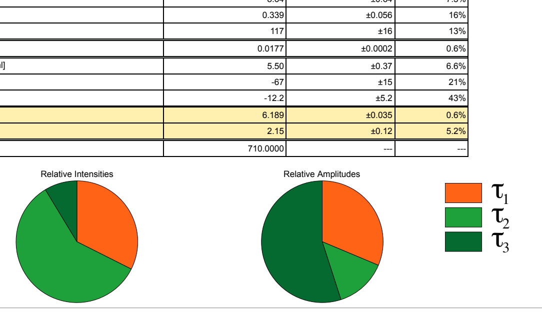

Data Set: 29 / 29

Decay: crv[29]; IRF: crv[0]

Fit

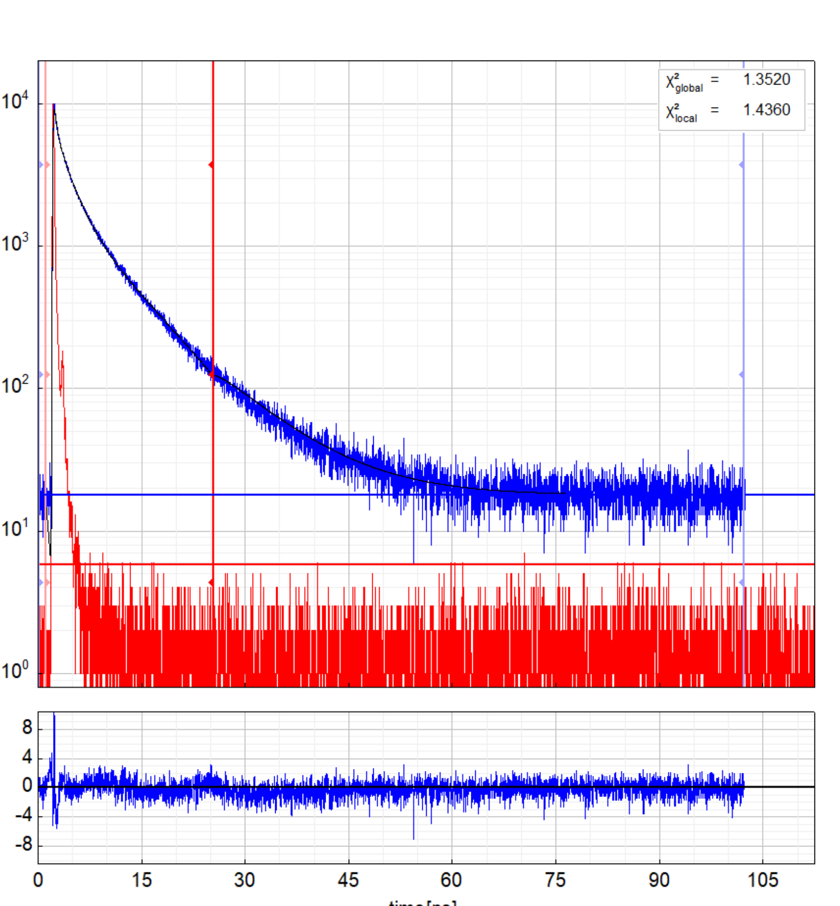

Autocorrelation (Residuals)

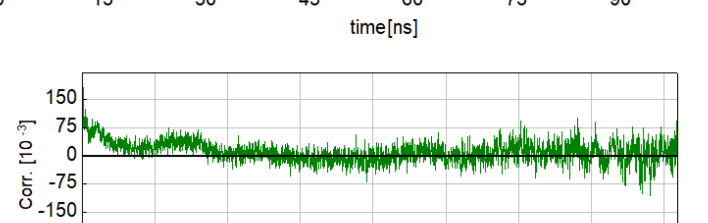

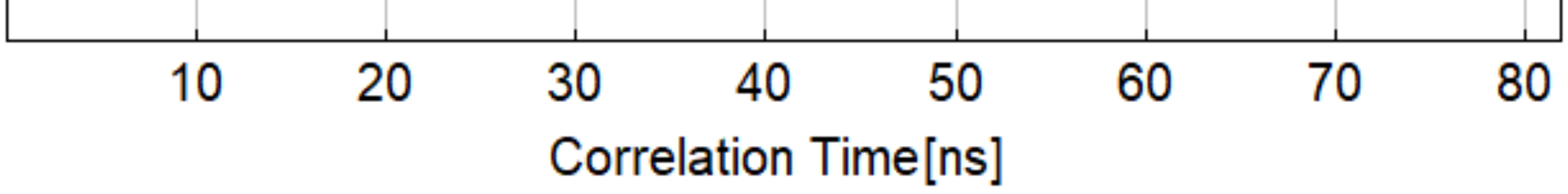

Fitted Parameters

Parameter	Value	Δ	δ
A_1[kCnts/Chnl]	4.93	±0.23	4.5%
τ_1[ns]	2.23	±0.14	5.9%
I_1[kCnts]	437.7	±4.8	1.1%
A_2[kCnts/Chnl]	1.838	±0.078	4.2%
τ_2[ns]	9.233	±0.054	0.6%
I_2[kCnts]	679	±25	3.6%
A_3[kCnts/Chnl]	7.7	±1.2	15%
τ_3[ns]	0.339	±0.056	16%
I_3[kCnts]	104.3	±5.3	5.0%
$Bkgr_{Dec}$[kCnts]	0.0178	±0.0002	0.6%
$Bkgr_{IRF}$[Cnts/Chnl]	5.80	±0.46	7.9%
$Shift_{IRF}$[ps]	-31	±16	50%
A_{Scatt}[kCnts]	15	±16	106%
τ_{AvInt}[ns]	5.959	±0.032	0.5%
τ_{AvAmp}[ns]	2.12	±0.21	9.7%
λ_{Det}[nm]	720.0000	---	---

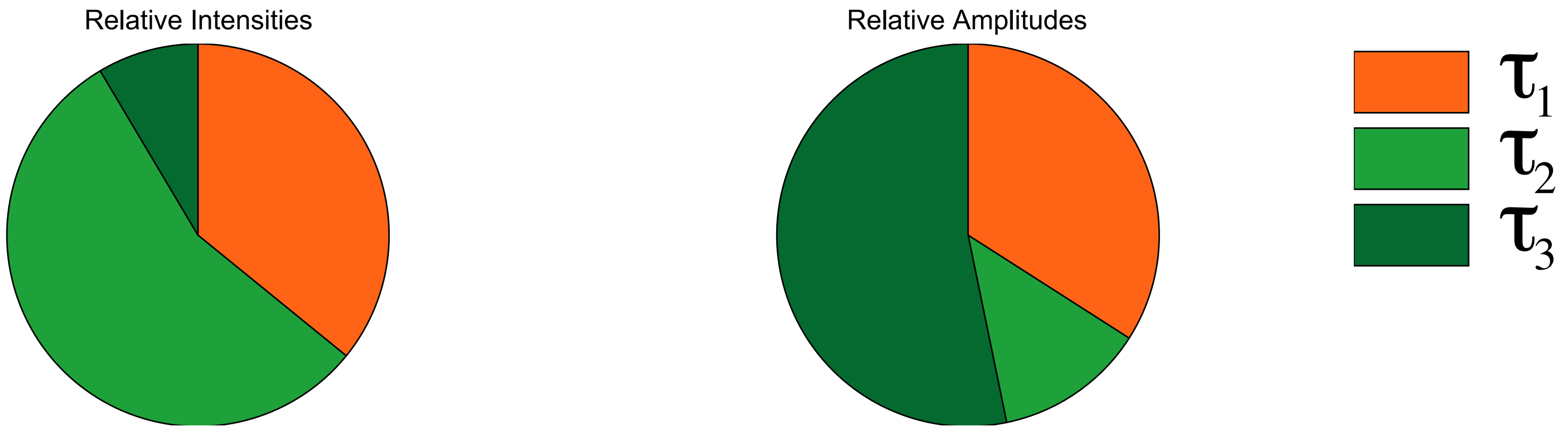

Parameter Plot

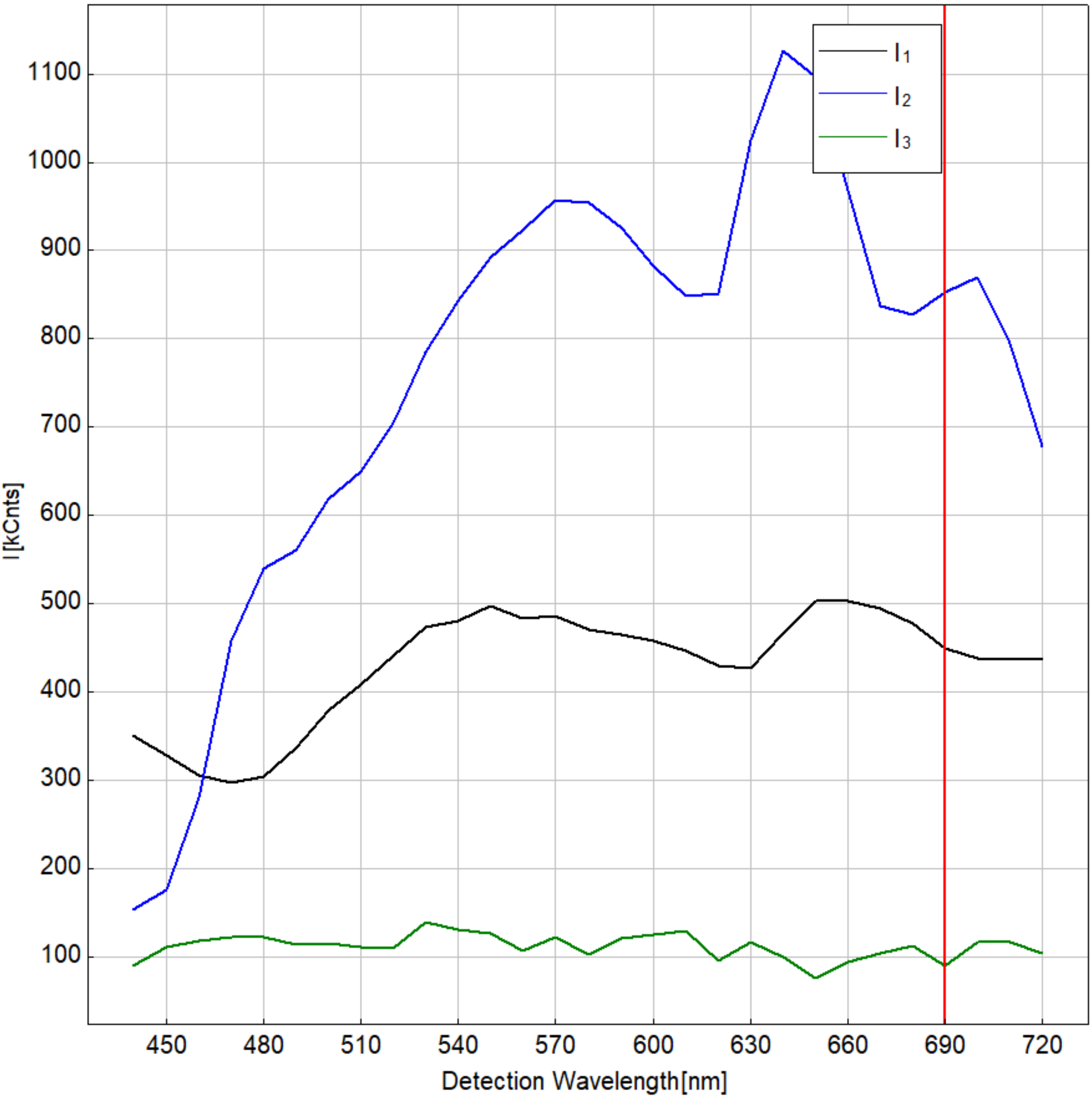

Global Parameters

Parameter	Value	Δ	δ
τ_1[ns]	2.23	±0.14	5.9%
τ_2[ns]	9.233	±0.054	0.6%
τ_3[ns]	0.339	±0.056	16%
