# Supplementary material for: A Porphyrin‐Based Organic Network Comprising Sustainable Carbon Dots for Photopolymerization
Source: Angew Chem Int Ed Engl. 2022 Sep 1;61(40):e202208180. doi: 10.1002/anie.202208180 (PMC9826160; doi:10.1002/anie.202208180)
Supplement: Supplementary file 4 — Supporting Information [file ANIE-61-0-s005.pdf]

EasyTau Report

3/11/2022 7:01:40 PM
F:\Paper Xiongfei\CD-1 DMSO tau global.html

PicoQuant

Container Curves

TRES+IRF_20201209_1730.etc: crv[0 - 28]

Container Properties

Measurement Context: Time Resolved Emission Scan
Detection Wavelength: 450.0 - 720.0 (Step: 10.0) nm
Summary:

Sample:	CD_X_1 tau sustractive
Solvent:	Dimethyl sulfoxide
Excitation:	V pol 376±1nm with LDH-P-C-375
Detection:	M pol 450...720nm 10nm steps<br>10000 peak counts<br>grating 1200/500-<br>detector UV-red [PMT]

Comment

Model: Exponential [Reconv.]

$$\mathrm{Dec}(t)=\left[\int_{-\infty}^{t} dt'\,[\mathrm{IRF}(t-Shift_{IRF})-Bkgr_{IRF}]\left[\sum_{i=1}^{n_{Exp}} A_i\, e^{-\frac{t-t'}{\tau_i}} + A_{Scatt}\,\delta(t-t')\right]\right]+Bkgr_{Dec}$$

$$I_m = A_m\tau_m$$

$$A_{Rel\,m}=A_m\Big/\sum_{\substack{i=1\\A_i>0}}^{n_{Exp}} A_i \qquad I_{Rel\,m}=I_m\Big/\sum_{\substack{i=1\\I_i>0}}^{n_{Exp}} I_i$$

$$\tau_{Av\,Amp}=\sum_{\substack{i=1\\A_i>0}}^{n_{Exp}} A_i\tau_i\Big/\sum_{\substack{i=1\\A_i>0}}^{n_{Exp}} A_i \qquad \tau_{Av\,Int}=\sum_{\substack{i=1\\I_i>0}}^{n_{Exp}} I_i\tau_i\Big/\sum_{\substack{i=1\\I_i>0}}^{n_{Exp}} I_i$$

Data Set: 1 / 28

Decay: crv[1]; IRF: crv[0]

Fit

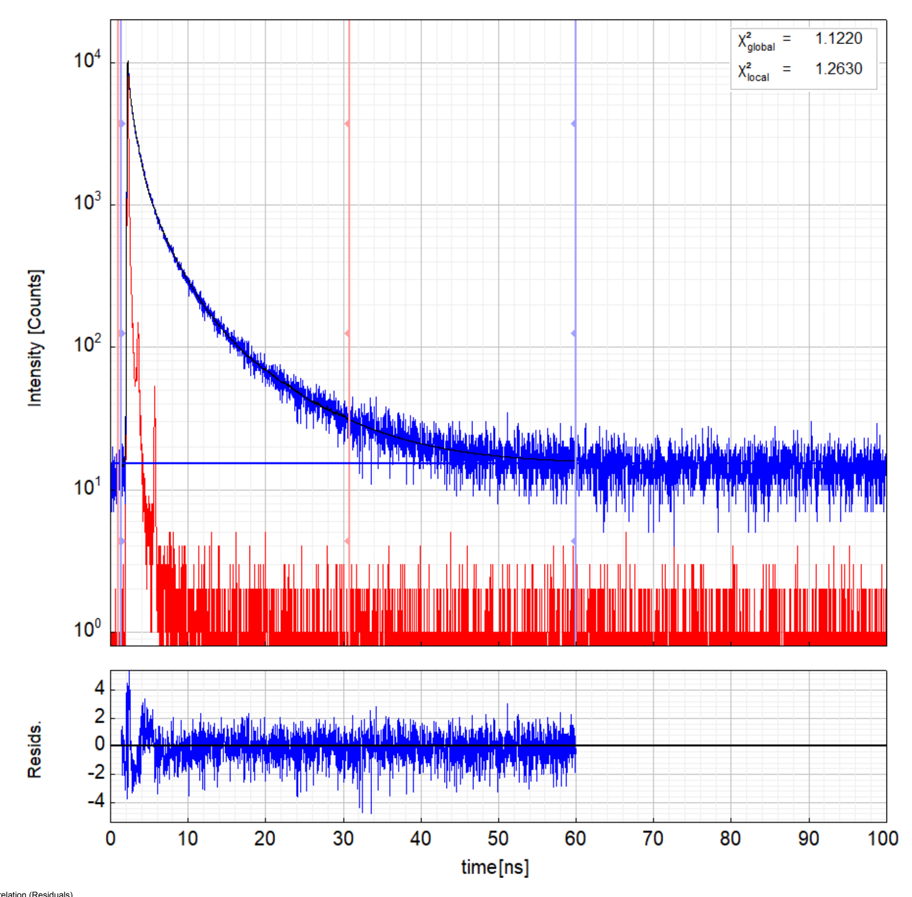

Autocorrelation (Residuals)

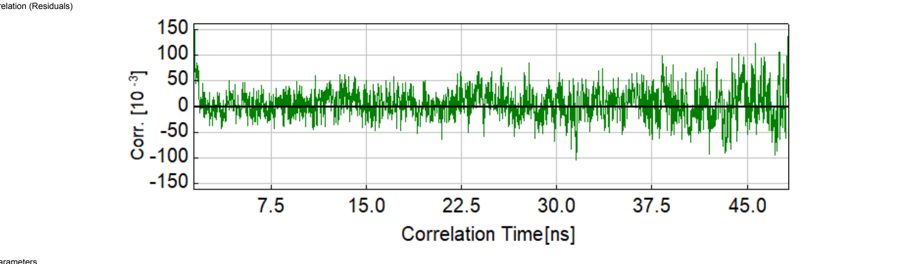

Fitted Parameters

Parameter	Value	Δ	δ
A_1[kCnts/Chnl]	0.3037	±0.0057	1.8%
τ_1[ns]	9.558	±0.017	0.2%
I_1[kCnts]	116.1	±2.2	1.8%
A_2[kCnts/Chnl]	6.233	±0.074	1.2%
τ_2[ns]	0.733	±0.012	1.6%
I_2[kCnts]	182.6	±3.8	2.1%
A_3[kCnts/Chnl]	1.967	±0.035	1.7%
τ_3[ns]	2.912	±0.033	1.1%
I_3[kCnts]	229.1	±3.6	1.6%
$Bkgr_{Dec}$[kCnts]	0.0152	±0.0002	1.1%
$Bkgr_{IRF}$[Cnts/Chnl]	0.565	±0.100	18%
$Shift_{IRF}$[ps]	11.1	±1.7	15%
A_{Scatt}[kCnts]	58.0	±2.3	3.9%
τ_{AvInt}[ns]	3.620	±0.013	0.3%
τ_{AvAmp}[ns]	1.552	±0.013	0.8%
λ_{Det}[nm]	450.0000	---	---

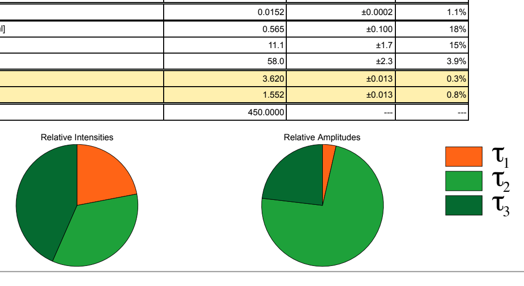

Data Set: 2 / 28

Decay: crv[2]; IRF: crv[0]

Fit

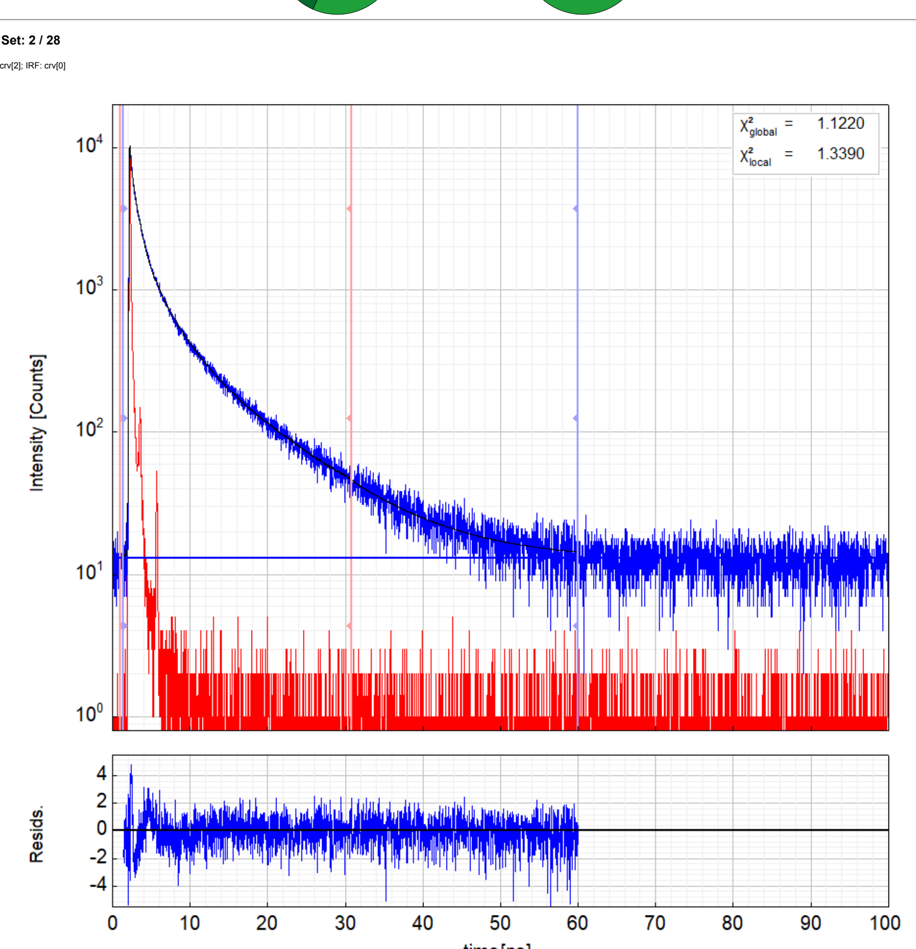

Autocorrelation (Residuals)

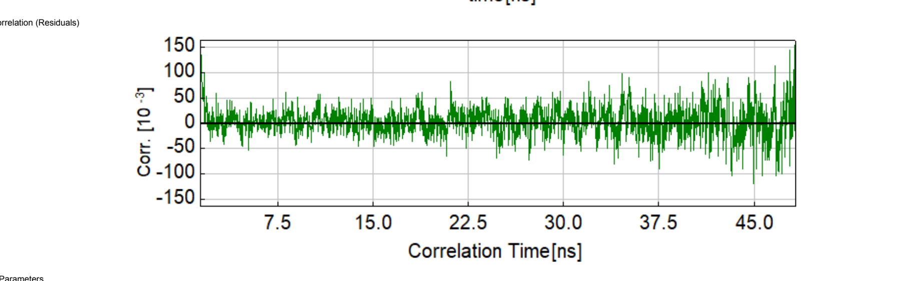

Fitted Parameters

Parameter	Value	Δ	δ
A_1[kCnts/Chnl]	0.5989	±0.0062	1.0%
τ_1[ns]	9.558	±0.017	0.2%
I_1[kCnts]	229.0	±2.2	0.9%
A_2[kCnts/Chnl]	6.79	±0.14	2.1%
τ_2[ns]	0.733	±0.012	1.6%
I_2[kCnts]	198.8	±3.9	2.0%
A_3[kCnts/Chnl]	1.918	±0.036	1.8%
τ_3[ns]	2.912	±0.033	1.1%
I_3[kCnts]	223.4	±2.9	1.3%
$Bkgr_{Dec}$[kCnts]	0.0130	±0.0002	1.5%
$Bkgr_{IRF}$[Cnts/Chnl]	0.249	±0.063	25%
$Shift_{IRF}$[ps]	9.6	±1.2	12%
A_{Scatt}[kCnts]	54.4	±1.4	2.5%
τ_{AvInt}[ns]	4.5830	±0.0097	0.2%
τ_{AvAmp}[ns]	1.750	±0.027	1.5%
λ_{Det}[nm]	460.0000	---	---

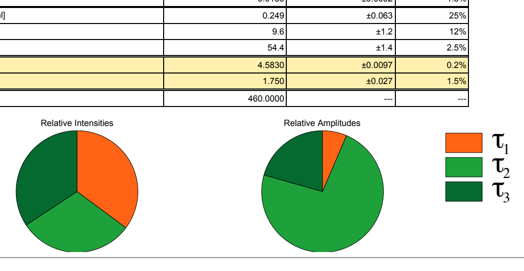

Data Set: 3 / 28

Decay: crv[3]; IRF: crv[0]

Fit

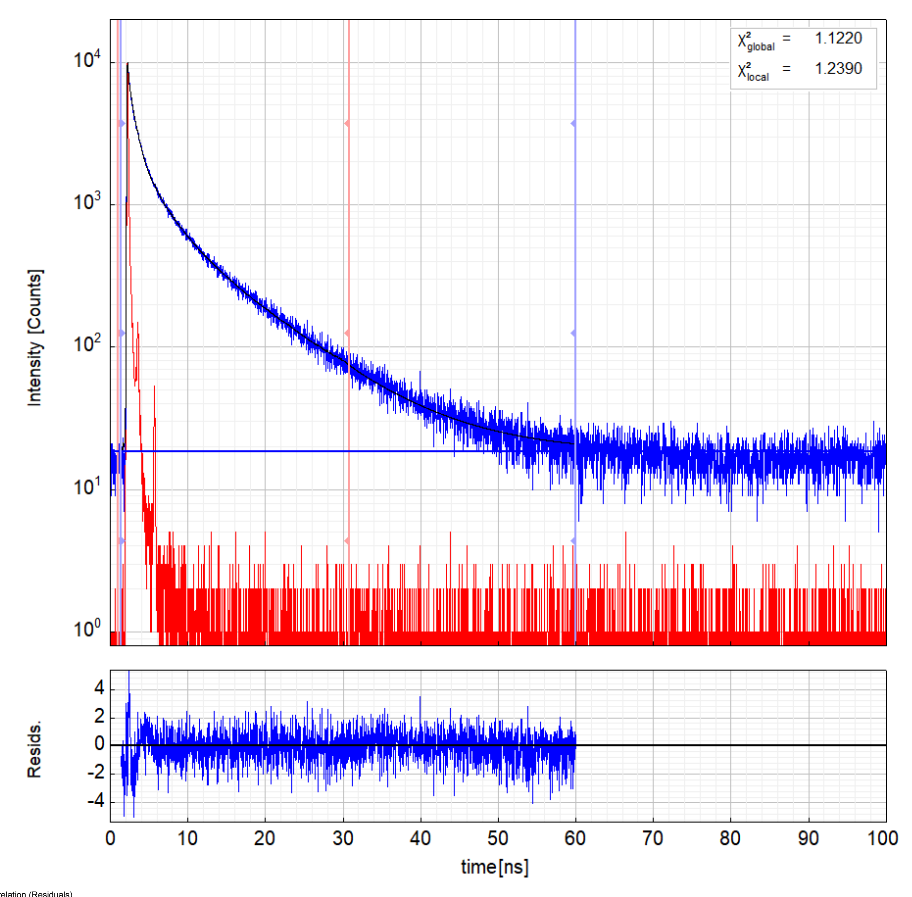

Autocorrelation (Residuals)

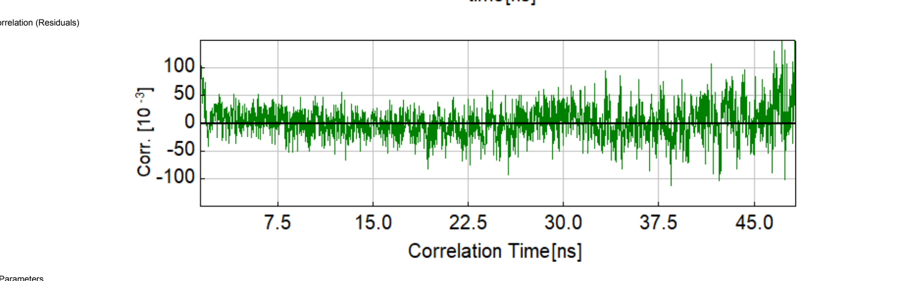

Fitted Parameters

Parameter	Value	Δ	δ
A_1[kCnts/Chnl]	0.9980	±0.0069	0.7%
τ_1[ns]	9.558	±0.017	0.2%
I_1[kCnts]	381.6	±2.5	0.6%
A_2[kCnts/Chnl]	6.530	±0.080	1.2%
τ_2[ns]	0.733	±0.012	1.6%
I_2[kCnts]	191.3	±3.1	1.6%
A_3[kCnts/Chnl]	1.812	±0.040	2.2%
τ_3[ns]	2.912	±0.033	1.1%
I_3[kCnts]	211.0	±2.7	1.2%
$Bkgr_{Dec}$[kCnts]	0.0184	±0.0002	0.6%
$Bkgr_{IRF}$[Cnts/Chnl]	-0.056	±0.088	156%
$Shift_{IRF}$[ps]	10.67	±0.66	6.1%
A_{Scatt}[kCnts]	51.4	±1.2	2.3%
τ_{AvInt}[ns]	5.616	±0.018	0.3%
τ_{AvAmp}[ns]	2.098	±0.024	1.1%
λ_{Det}[nm]	470.0000	---	---

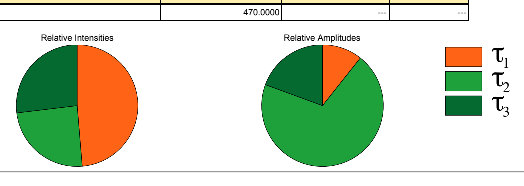

Data Set: 4 / 28

Decay: crv[4]; IRF: crv[0]

Fit

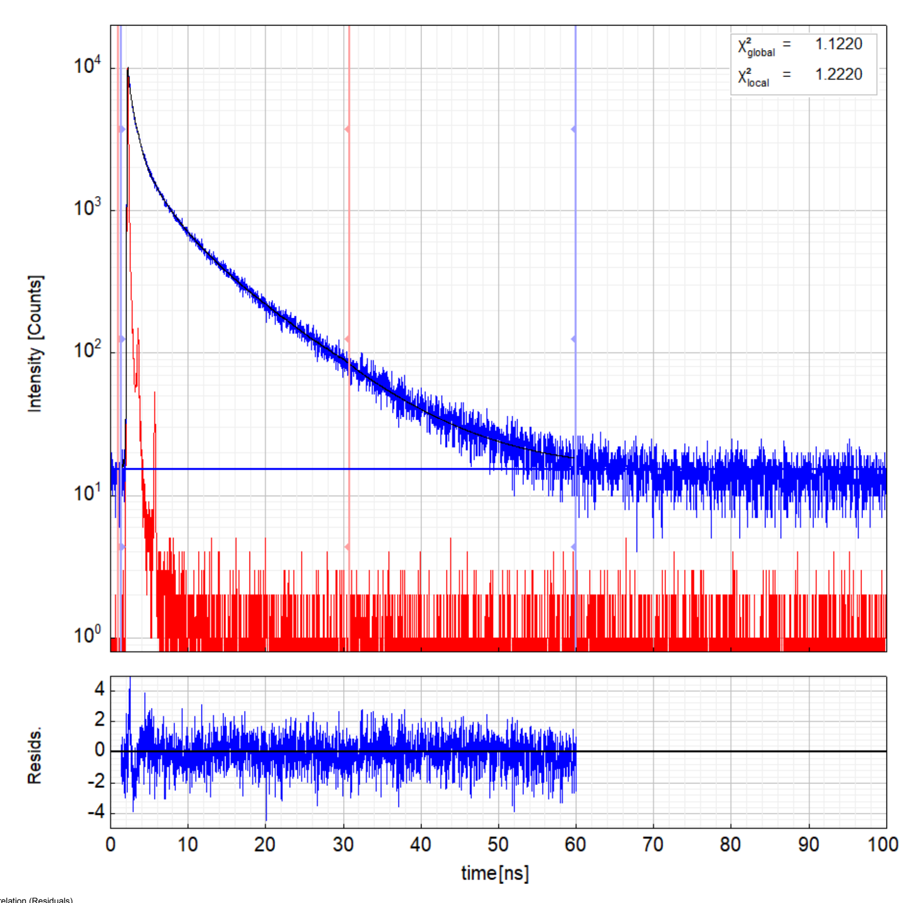

Autocorrelation (Residuals)

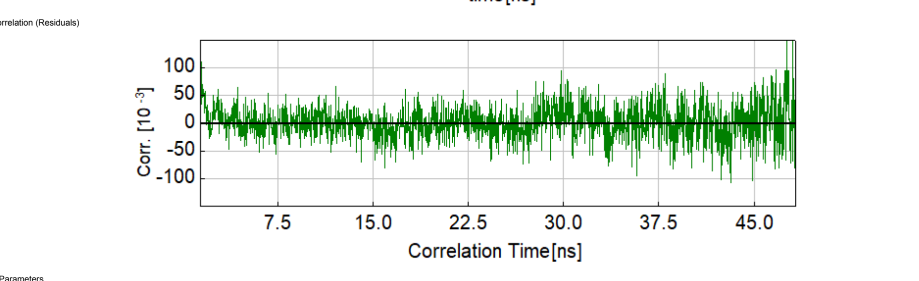

Fitted Parameters

Parameter	Value	Δ	δ
A_1[kCnts/Chnl]	1.2240	±0.0085	0.7%
τ_1[ns]	9.558	±0.017	0.2%
I_1[kCnts]	468.0	±2.8	0.6%
A_2[kCnts/Chnl]	6.63	±0.13	2.0%
τ_2[ns]	0.733	±0.012	1.6%
I_2[kCnts]	194.1	±3.1	1.6%
A_3[kCnts/Chnl]	1.933	±0.034	1.8%
τ_3[ns]	2.912	±0.033	1.1%
I_3[kCnts]	225.1	±3.4	1.5%
$Bkgr_{Dec}$[kCnts]	0.0152	±0.0004	2.0%
$Bkgr_{IRF}$[Cnts/Chnl]	-0.055	±0.050	90%
$Shift_{IRF}$[ps]	10.02	±0.69	6.9%
A_{Scatt}[kCnts]	50.11	±0.84	1.7%
τ_{AvInt}[ns]	5.941	±0.017	0.3%
τ_{AvAmp}[ns]	2.268	±0.025	1.1%
λ_{Det}[nm]	480.0000	---	---

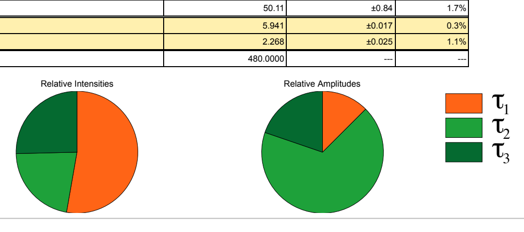

Data Set: 5 / 28

Decay: crv[5]; IRF: crv[0]

Fit

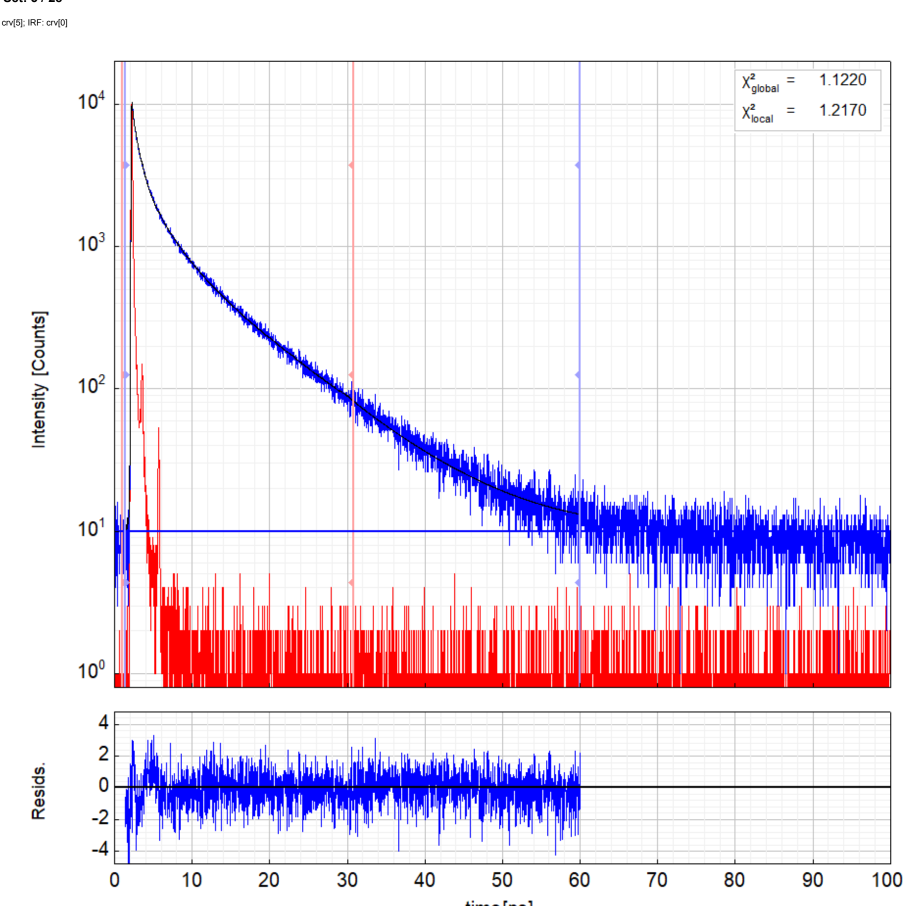

Autocorrelation (Residuals)

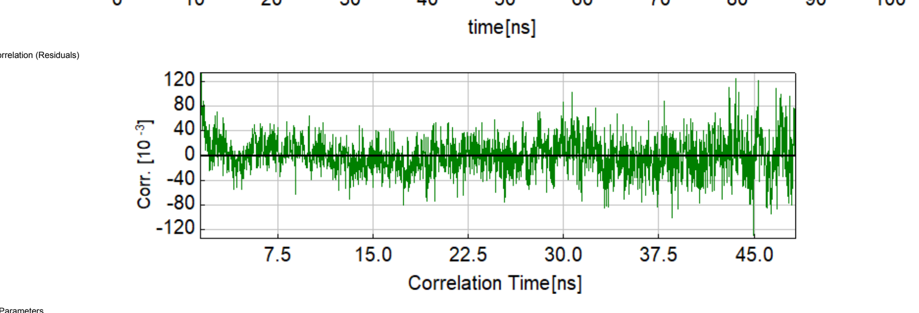

Fitted Parameters

Parameter	Value	Δ	δ
A_1[kCnts/Chnl]	1.3048	±0.0088	0.7%
τ_1[ns]	9.558	±0.017	0.2%
I_1[kCnts]	498.8	±2.6	0.5%
A_2[kCnts/Chnl]	6.720	±0.084	1.2%
τ_2[ns]	0.733	±0.012	1.6%
I_2[kCnts]	196.8	±2.6	1.3%
A_3[kCnts/Chnl]	2.375	±0.045	1.9%
τ_3[ns]	2.912	±0.033	1.1%
I_3[kCnts]	276.6	±4.2	1.5%
$Bkgr_{Dec}$[kCnts]	0.0099	±0.0002	1.6%
$Bkgr_{IRF}$[Cnts/Chnl]	0.059	±0.098	167%
$Shift_{IRF}$[ps]	8.9	±1.6	17%
A_{Scatt}[kCnts]	48.0	±1.8	3.6%
τ_{AvInt}[ns]	5.881	±0.021	0.3%
τ_{AvAmp}[ns]	2.338	±0.015	0.6%
λ_{Det}[nm]	490.0000	---	---

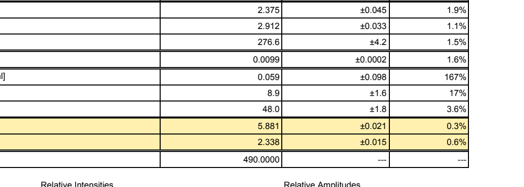

Data Set: 6 / 28

Decay: crv[6]; IRF: crv[0]

Fit

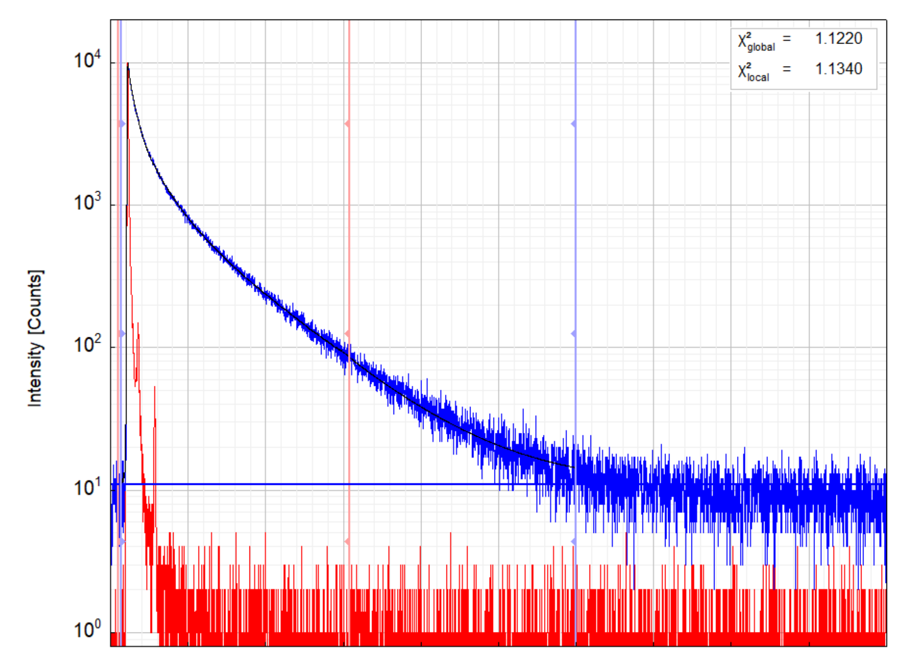

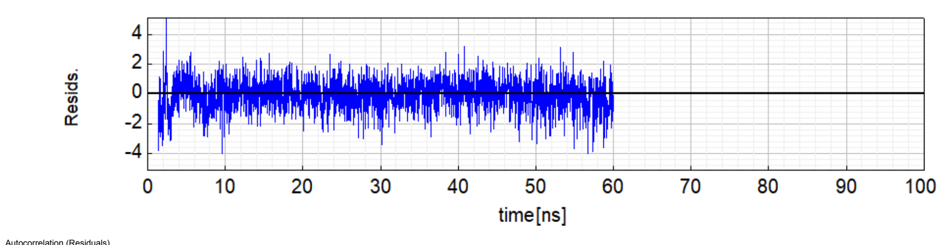

Autocorrelation (Residuals)

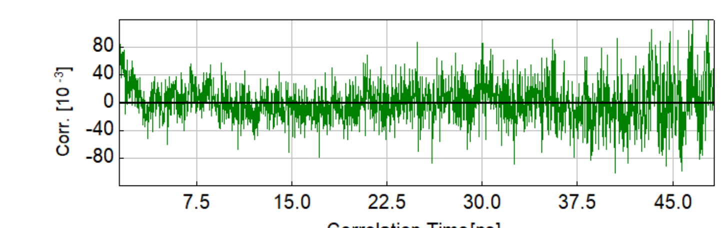

Fitted Parameters

Parameter	Value	Δ	δ
A_1[kCnts/Chnl]	1.355	±0.013	0.9%
τ_1[ns]	9.558	±0.017	0.2%
I_1[kCnts]	517.8	±4.2	0.8%
A_2[kCnts/Chnl]	6.226	±0.085	1.4%
τ_2[ns]	0.733	±0.012	1.6%
I_2[kCnts]	182.4	±3.5	1.9%
A_3[kCnts/Chnl]	2.698	±0.059	2.2%
τ_3[ns]	2.912	±0.033	1.1%
I_3[kCnts]	314.1	±4.5	1.4%
$Bkgr_{Dec}$[kCnts]	0.0110	±0.0002	1.0%
$Bkgr_{IRF}$[Cnts/Chnl]	0.114	±0.071	62%
$Shift_{IRF}$[ps]	9.58	±0.92	9.6%
A_{Scatt}[kCnts]	45.6	±1.2	2.4%
τ_{AvInt}[ns]	5.913	±0.014	0.2%
τ_{AvAmp}[ns]	2.468	±0.025	1.0%
λ_{Det}[nm]	500.0000	---	---

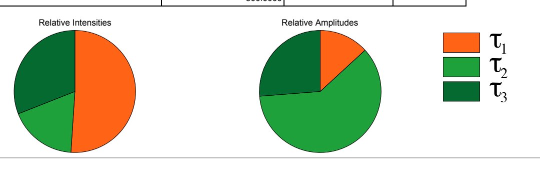

Data Set: 7 / 28

Decay: crv[7]; IRF: crv[0]

Fit

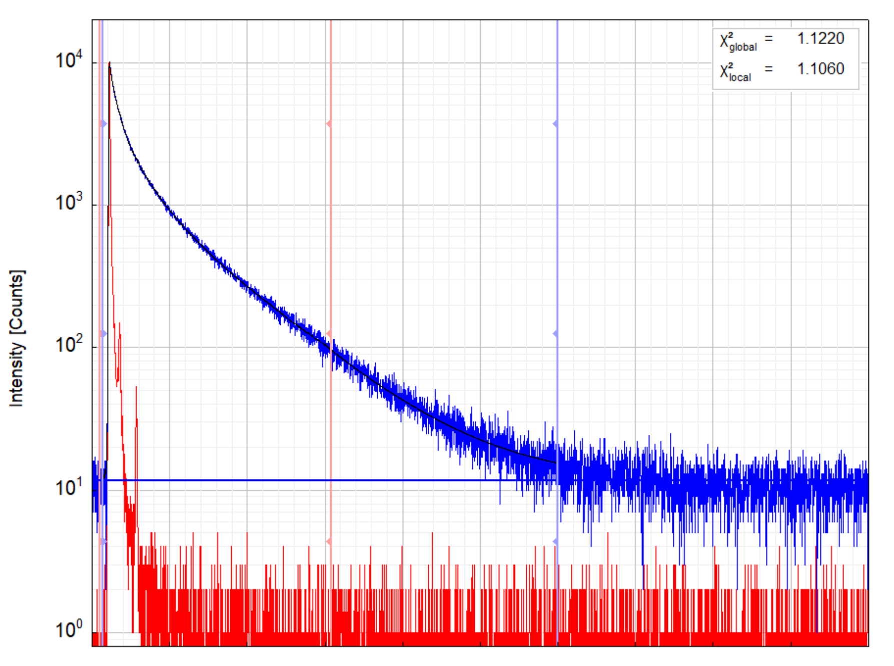

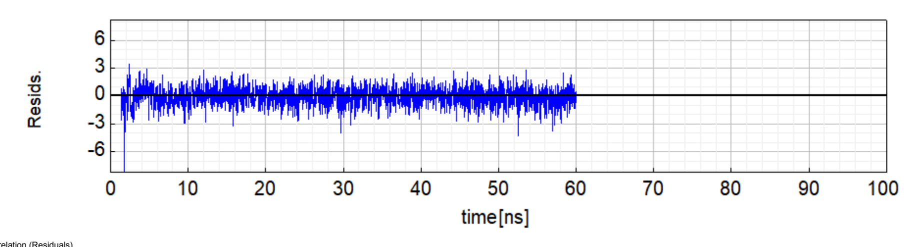

Autocorrelation (Residuals)

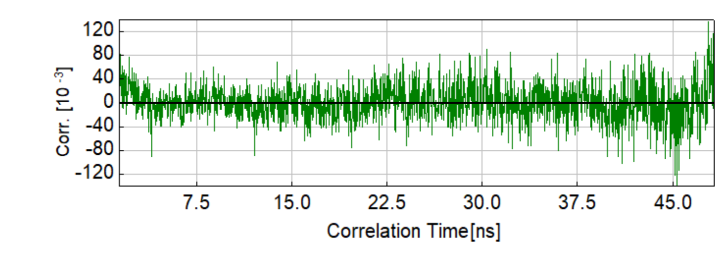

Fitted Parameters

Parameter	Value	Δ	δ
A_1[kCnts/Chnl]	1.541	±0.014	0.9%
τ_1[ns]	9.558	±0.017	0.2%
I_1[kCnts]	589.0	±4.2	0.7%
A_2[kCnts/Chnl]	6.203	±0.066	1.0%
τ_2[ns]	0.733	±0.012	1.6%
I_2[kCnts]	181.7	±3.8	2.0%
A_3[kCnts/Chnl]	3.039	±0.036	1.2%
τ_3[ns]	2.912	±0.033	1.1%
I_3[kCnts]	353.9	±2.5	0.7%
$Bkgr_{Dec}$[kCnts]	0.0117	±0.0004	2.7%
$Bkgr_{IRF}$[Cnts/Chnl]	0.10	±0.12	116%
$Shift_{IRF}$[ps]	9.39	±0.67	7.0%
A_{Scatt}[kCnts]	43.63	±0.88	2.0%
τ_{AvInt}[ns]	6.041	±0.014	0.2%
τ_{AvAmp}[ns]	2.608	±0.025	1.0%
λ_{Det}[nm]	510.0000	---	---

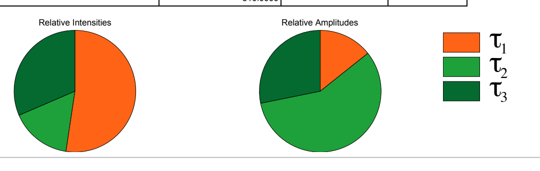

Data Set: 8 / 28

Decay: crv[8]; IRF: crv[0]

Fit

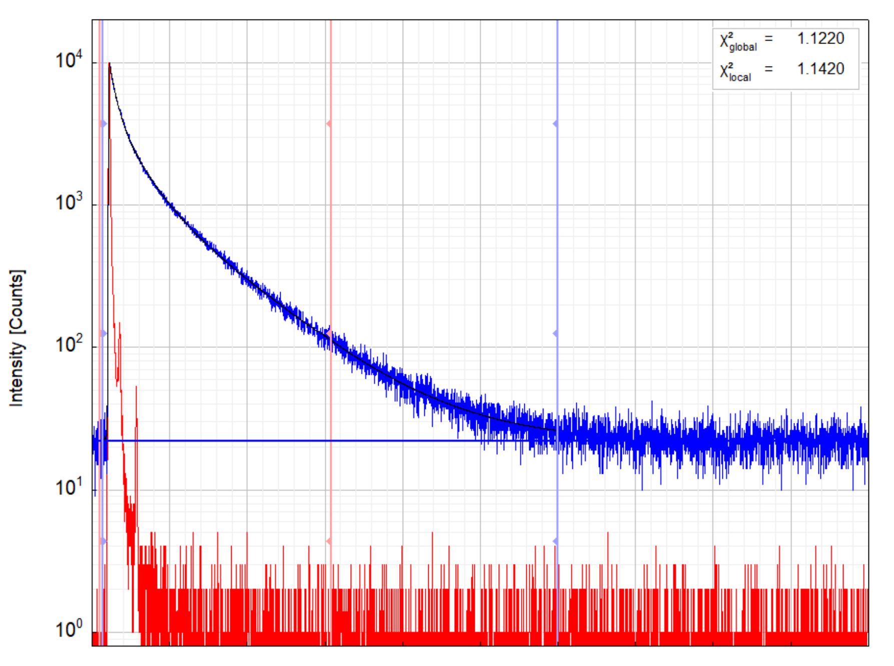

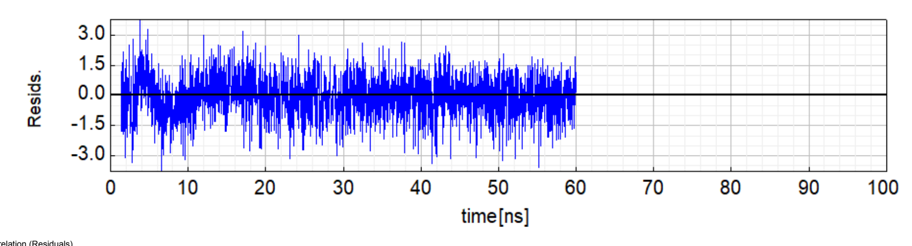

Autocorrelation (Residuals)

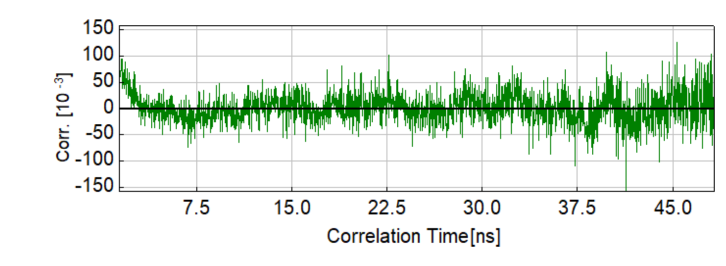

Fitted Parameters

Parameter	Value	Δ	δ
A_1[kCnts/Chnl]	1.6958	±0.0077	0.5%
τ_1[ns]	9.558	±0.017	0.2%
I_1[kCnts]	648.3	±2.6	0.4%
A_2[kCnts/Chnl]	6.111	±0.057	0.9%
τ_2[ns]	0.733	±0.012	1.6%
I_2[kCnts]	179.0	±4.7	2.6%
A_3[kCnts/Chnl]	3.267	±0.061	1.8%
τ_3[ns]	2.912	±0.033	1.1%
I_3[kCnts]	380.5	±2.7	0.7%
$Bkgr_{Dec}$[kCnts]	0.0220	±0.0002	0.8%
$Bkgr_{IRF}$[Cnts/Chnl]	0.333	±0.061	18%
$Shift_{IRF}$[ps]	7.98	±0.81	10%
A_{Scatt}[kCnts]	40.08	±0.47	1.2%
τ_{AvInt}[ns]	6.157	±0.016	0.3%
τ_{AvAmp}[ns]	2.7267	±0.0092	0.3%
λ_{Det}[nm]	520.0000	---	---

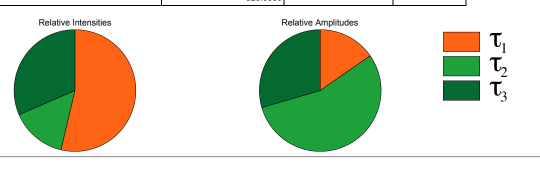

Data Set: 9 / 28

Decay: crv[9]; IRF: crv[0]

Fit

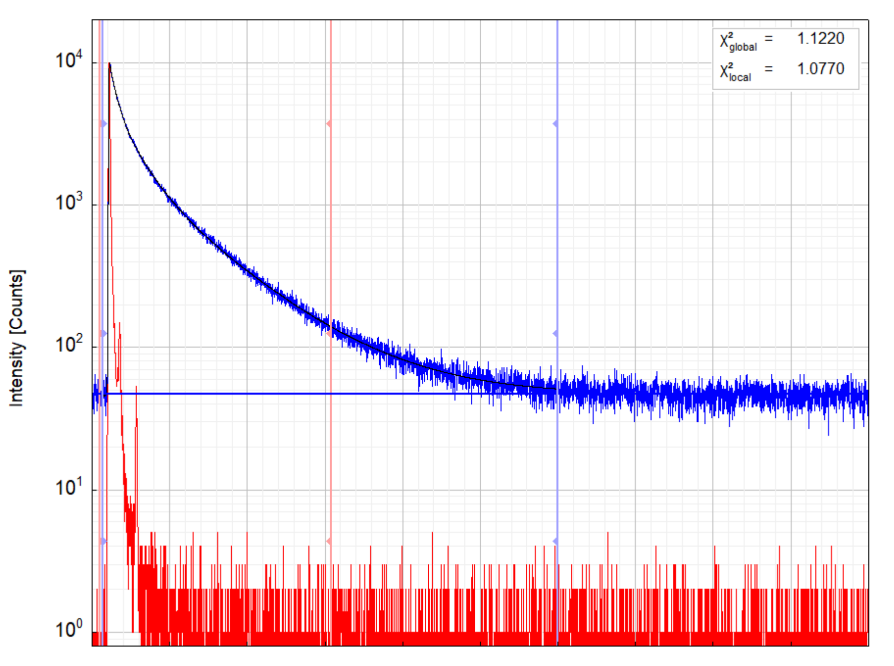

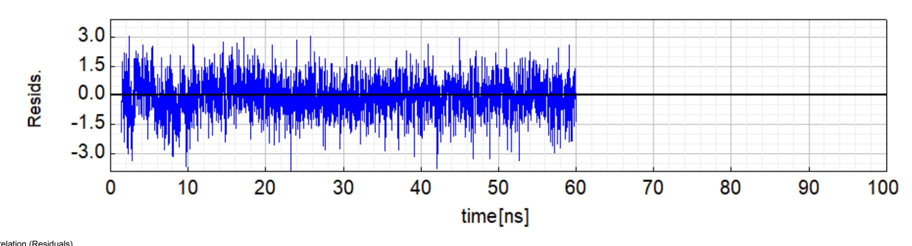

Autocorrelation (Residuals)

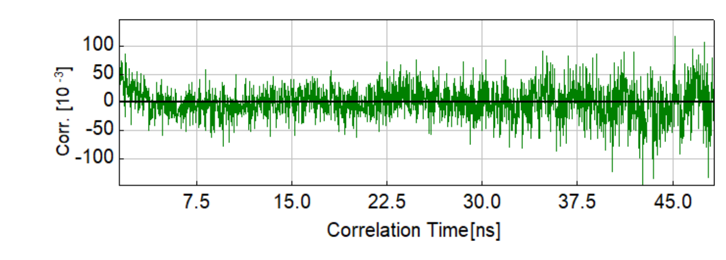

Fitted Parameters

Parameter	Value	Δ	δ
A_1[kCnts/Chnl]	1.846	±0.015	0.8%
τ_1[ns]	9.558	±0.017	0.2%
I_1[kCnts]	705.4	±5.8	0.8%
A_2[kCnts/Chnl]	5.818	±0.084	1.4%
τ_2[ns]	0.733	±0.012	1.6%
I_2[kCnts]	170.4	±2.3	1.3%
A_3[kCnts/Chnl]	3.565	±0.068	1.9%
τ_3[ns]	2.912	±0.033	1.1%
I_3[kCnts]	415.2	±4.2	1.0%
$Bkgr_{Dec}$[kCnts]	0.0468	±0.0005	0.9%
$Bkgr_{IRF}$[Cnts/Chnl]	0.731	±0.068	9.3%
$Shift_{IRF}$[ps]	7.27	±0.70	9.5%
A_{Scatt}[kCnts]	38.01	±0.73	1.9%
τ_{AvInt}[ns]	6.256	±0.014	0.2%
τ_{AvAmp}[ns]	2.875	±0.013	0.4%
λ_{Det}[nm]	530.0000	---	---

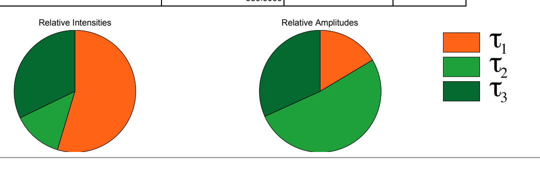

Data Set: 10 / 28

Decay: crv[10]; IRF: crv[0]

Fit

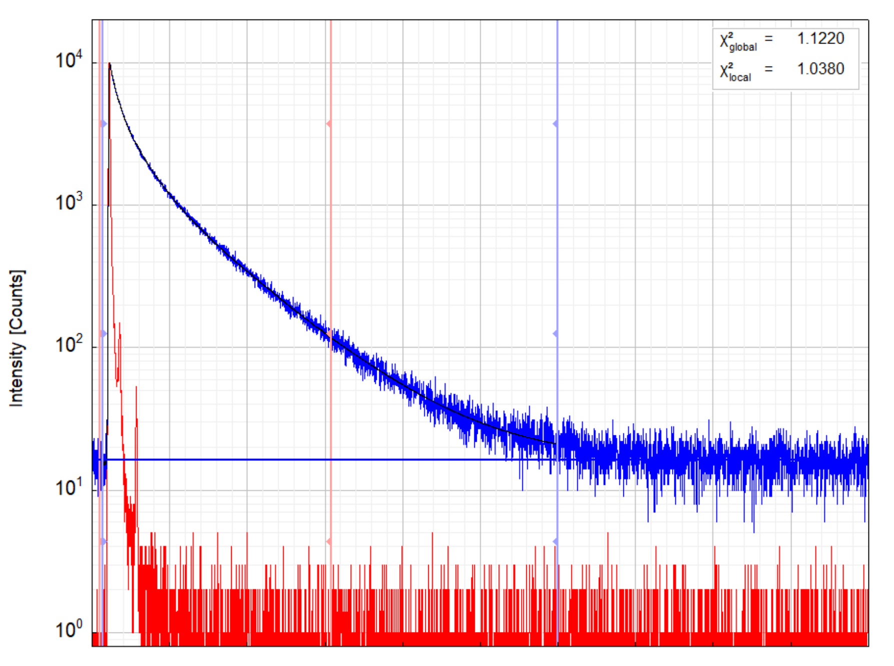

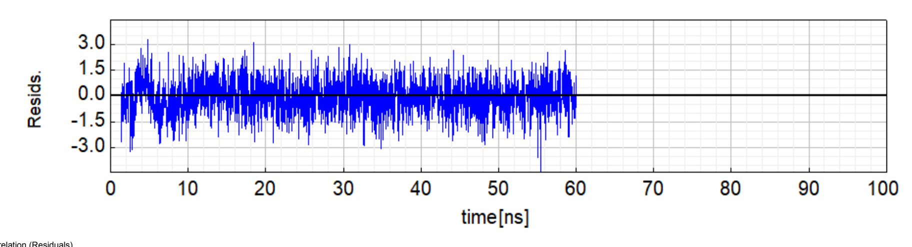

Autocorrelation (Residuals)

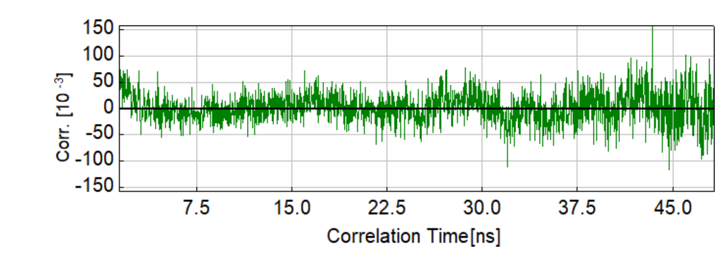

Fitted Parameters

Parameter	Value	Δ	δ
A_1[kCnts/Chnl]	2.027	±0.013	0.6%
τ_1[ns]	9.558	±0.017	0.2%
I_1[kCnts]	774.7	±3.8	0.5%
A_2[kCnts/Chnl]	5.653	±0.083	1.5%
τ_2[ns]	0.733	±0.012	1.6%
I_2[kCnts]	165.6	±4.5	2.7%
A_3[kCnts/Chnl]	3.819	±0.073	1.9%
τ_3[ns]	2.912	±0.033	1.1%
I_3[kCnts]	444.7	±4.6	1.0%
$Bkgr_{Dec}$[kCnts]	0.0163	±0.0002	1.0%
$Bkgr_{IRF}$[Cnts/Chnl]	0.787	±0.072	9.0%
$Shift_{IRF}$[ps]	6.96	±0.56	7.9%
A_{Scatt}[kCnts]	36.69	±0.75	2.0%
τ_{AvInt}[ns]	6.369	±0.014	0.2%
τ_{AvAmp}[ns]	3.012	±0.023	0.8%
λ_{Det}[nm]	540.0000	---	---

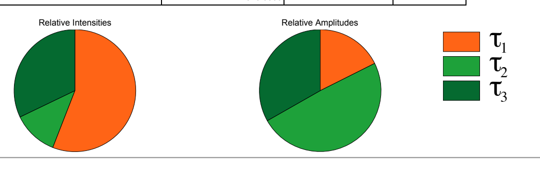

Data Set: 11 / 28

Decay: crv[11]; IRF: crv[0]

Fit

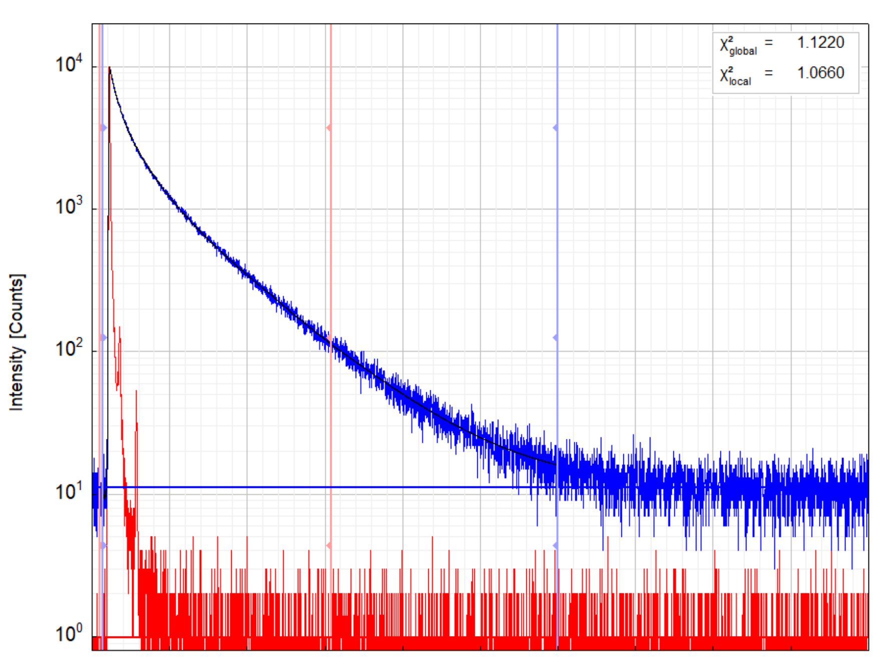

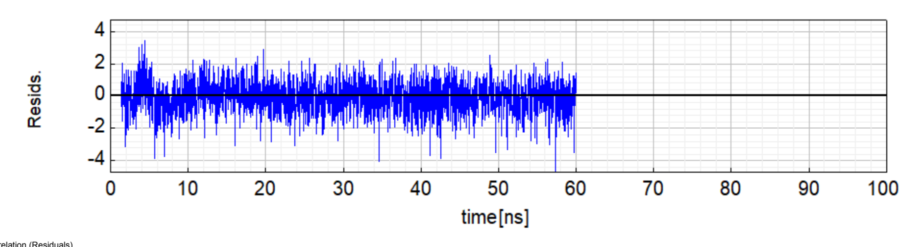

Autocorrelation (Residuals)

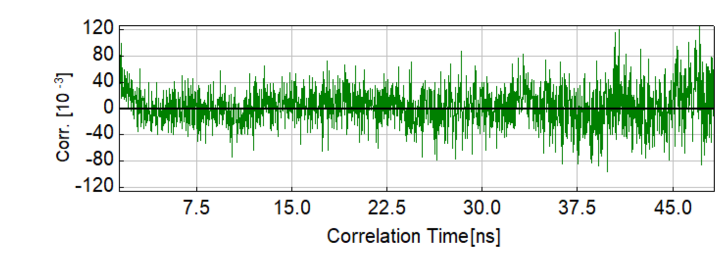

Fitted Parameters

Parameter	Value	Δ	δ
A_1[kCnts/Chnl]	2.0963	±0.0087	0.4%
τ_1[ns]	9.558	±0.017	0.2%
I_1[kCnts]	801.4	±2.8	0.3%
A_2[kCnts/Chnl]	5.522	±0.053	0.9%
τ_2[ns]	0.733	±0.012	1.6%
I_2[kCnts]	161.7	±4.0	2.5%
A_3[kCnts/Chnl]	3.849	±0.061	1.6%
τ_3[ns]	2.912	±0.033	1.1%
I_3[kCnts]	448.2	±4.3	1.0%
$Bkgr_{Dec}$[kCnts]	0.0112	±0.0002	1.3%
$Bkgr_{IRF}$[Cnts/Chnl]	0.990	±0.062	6.2%
$Shift_{IRF}$[ps]	8.96	±0.93	10%
A_{Scatt}[kCnts]	36.38	±0.77	2.1%
τ_{AvInt}[ns]	6.4356	±0.0077	0.1%
τ_{AvAmp}[ns]	3.0770	±0.0083	0.3%
λ_{Det}[nm]	550.0000	---	---

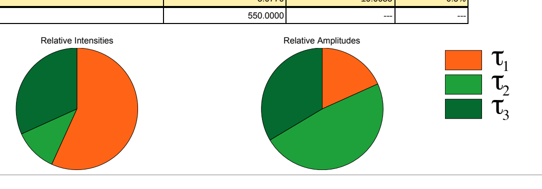

Data Set: 12 / 28

Decay: crv[12]; IRF: crv[0]

Fit

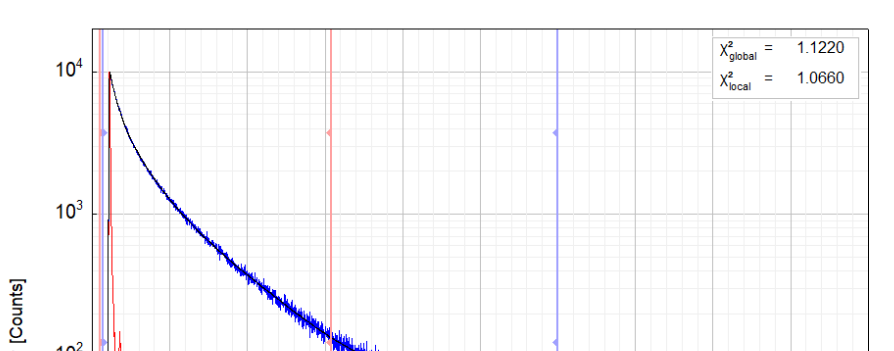

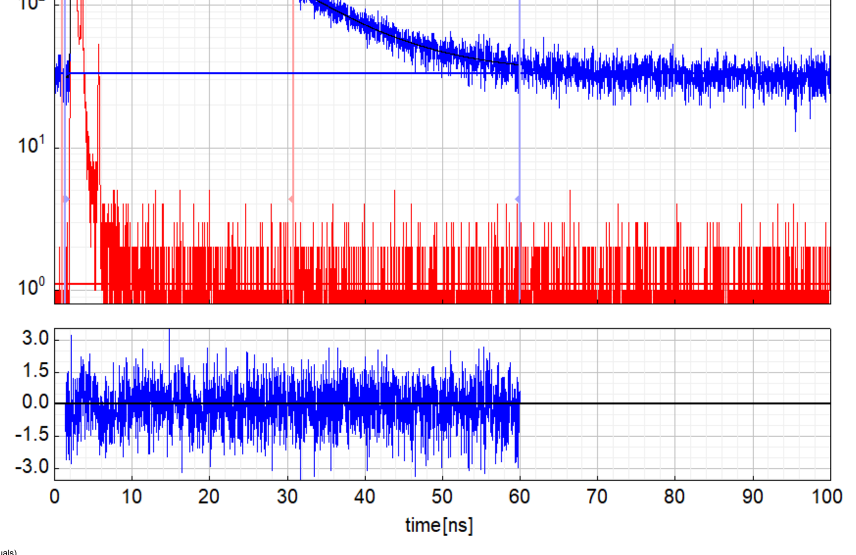

Autocorrelation (Residuals)

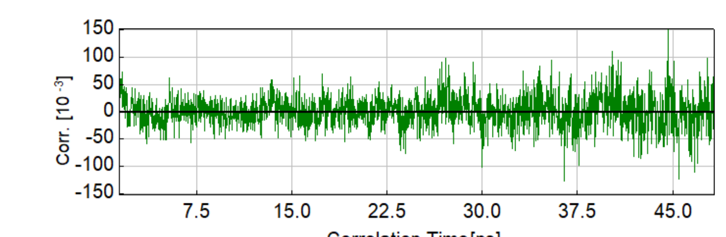

Fitted Parameters

Parameter	Value	Δ	δ
A_1[kCnts/Chnl]	2.148	±0.022	1.0%
τ_1[ns]	9.558	±0.017	0.2%
I_1[kCnts]	820.9	±4.7	0.6%
A_2[kCnts/Chnl]	5.251	±0.077	1.5%
τ_2[ns]	0.733	±0.012	1.6%
I_2[kCnts]	153.8	±3.0	1.9%
A_3[kCnts/Chnl]	3.957	±0.028	0.7%
τ_3[ns]	2.912	±0.033	1.1%
I_3[kCnts]	460.8	±4.1	0.9%
$Bkgr_{Dec}$[kCnts]	0.0332	±0.0003	0.8%
$Bkgr_{IRF}$[Cnts/Chnl]	1.106	±0.068	6.1%
$Shift_{IRF}$[ps]	9.8	±1.2	12%
A_{Scatt}[kCnts]	37.37	±0.68	1.8%
τ_{AvInt}[ns]	6.479	±0.011	0.2%
τ_{AvAmp}[ns]	3.161	±0.014	0.4%
λ_{Det}[nm]	560.0000	---	---

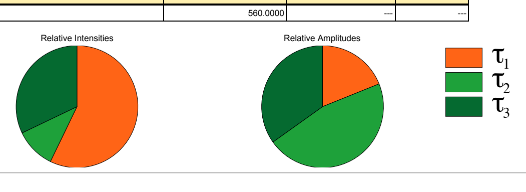

Data Set: 13 / 28

Decay: crv[13]; IRF: crv[0]

Fit

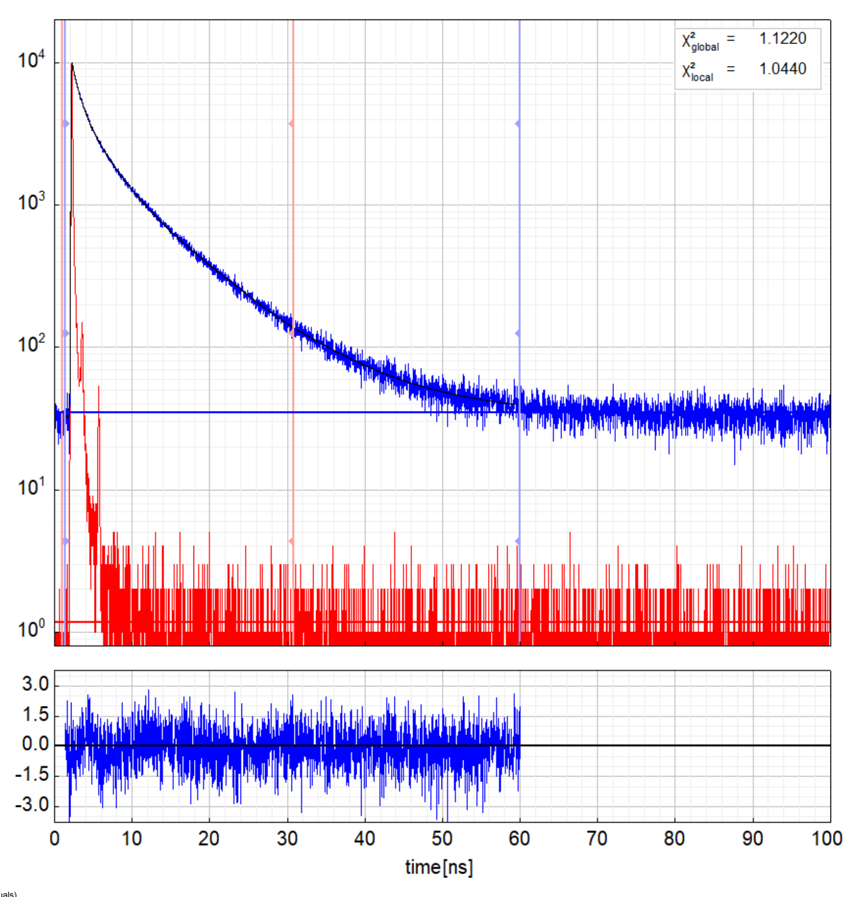

Autocorrelation (Residuals)

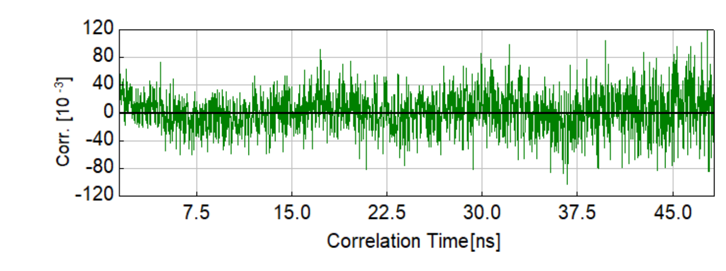

Fitted Parameters

Parameter	Value	Δ	δ
A_1[kCnts/Chnl]	2.179	±0.016	0.7%
τ_1[ns]	9.558	±0.017	0.2%
I_1[kCnts]	832.8	±4.8	0.6%
A_2[kCnts/Chnl]	5.155	±0.057	1.1%
τ_2[ns]	0.733	±0.012	1.6%
I_2[kCnts]	151.0	±3.1	2.0%
A_3[kCnts/Chnl]	3.918	±0.054	1.4%
τ_3[ns]	2.912	±0.033	1.1%
I_3[kCnts]	456.3	±3.2	0.7%
$Bkgr_{Dec}$[kCnts]	0.0346	±0.0004	1.1%
$Bkgr_{IRF}$[Cnts/Chnl]	1.177	±0.056	4.7%
$Shift_{IRF}$[ps]	10.29	±0.79	7.7%
A_{Scatt}[kCnts]	37.45	±0.63	1.7%
τ_{AvInt}[ns]	6.527	±0.012	0.2%
τ_{AvAmp}[ns]	3.200	±0.012	0.4%
λ_{Det}[nm]	570.0000	---	---

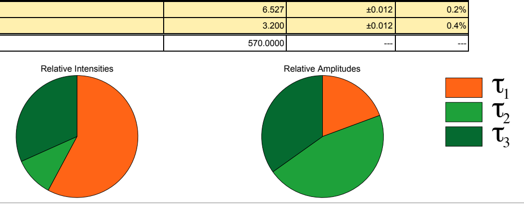

Data Set: 14 / 28

Decay: crv[14]; IRF: crv[0]

Fit

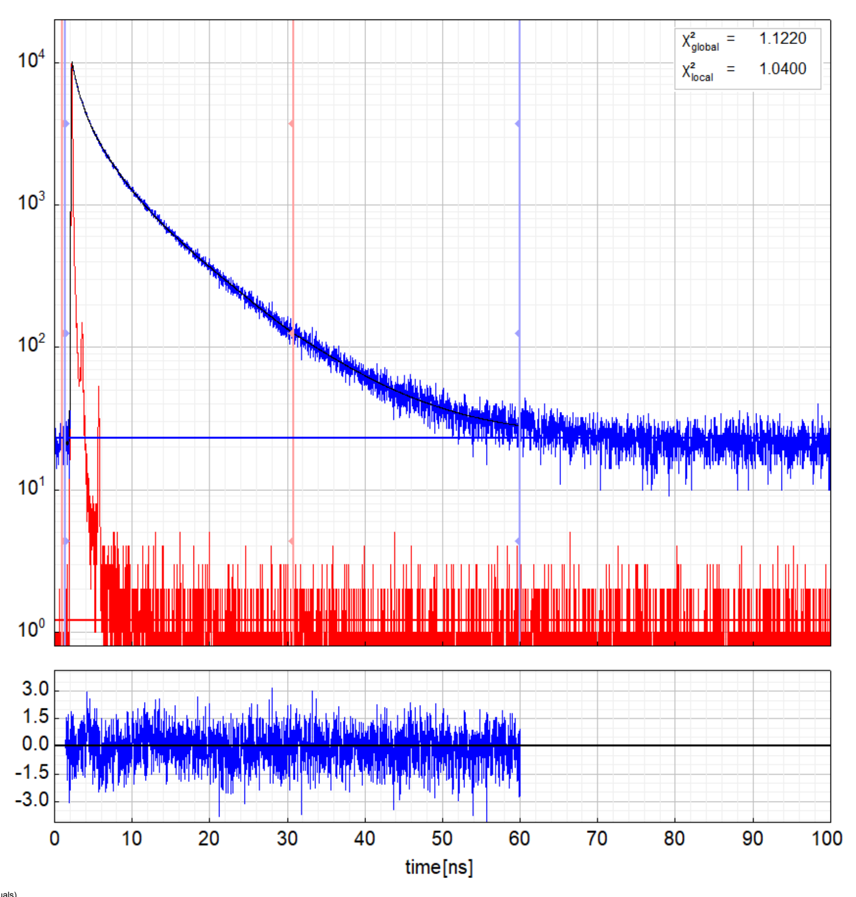

Autocorrelation (Residuals)

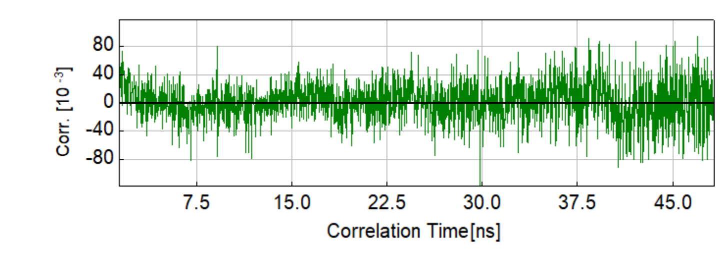

Fitted Parameters

Parameter	Value	Δ	δ
A_1[kCnts/Chnl]	2.178	±0.014	0.6%
τ_1[ns]	9.558	±0.017	0.2%
I_1[kCnts]	832.5	±4.6	0.5%
A_2[kCnts/Chnl]	5.099	±0.028	0.5%
τ_2[ns]	0.733	±0.012	1.6%
I_2[kCnts]	149.4	±3.0	2.0%
A_3[kCnts/Chnl]	3.955	±0.040	1.0%
τ_3[ns]	2.912	±0.033	1.1%
I_3[kCnts]	460.6	±4.0	0.9%
$Bkgr_{Dec}$[kCnts]	0.0232	±0.0003	1.0%
$Bkgr_{IRF}$[Cnts/Chnl]	1.206	±0.070	5.8%
$Shift_{IRF}$[ps]	11.27	±0.57	5.0%
A_{Scatt}[kCnts]	39.93	±0.51	1.3%
τ_{AvInt}[ns]	6.522	±0.013	0.2%
τ_{AvAmp}[ns]	3.211	±0.011	0.3%
λ_{Det}[nm]	580.0000	---	---

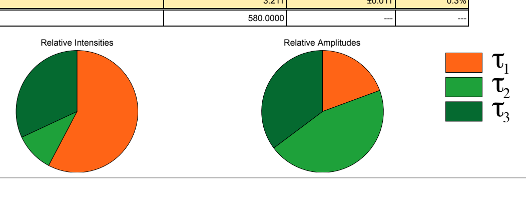

Data Set: 15 / 28

Decay: crv[15]; IRF: crv[0]

Fit

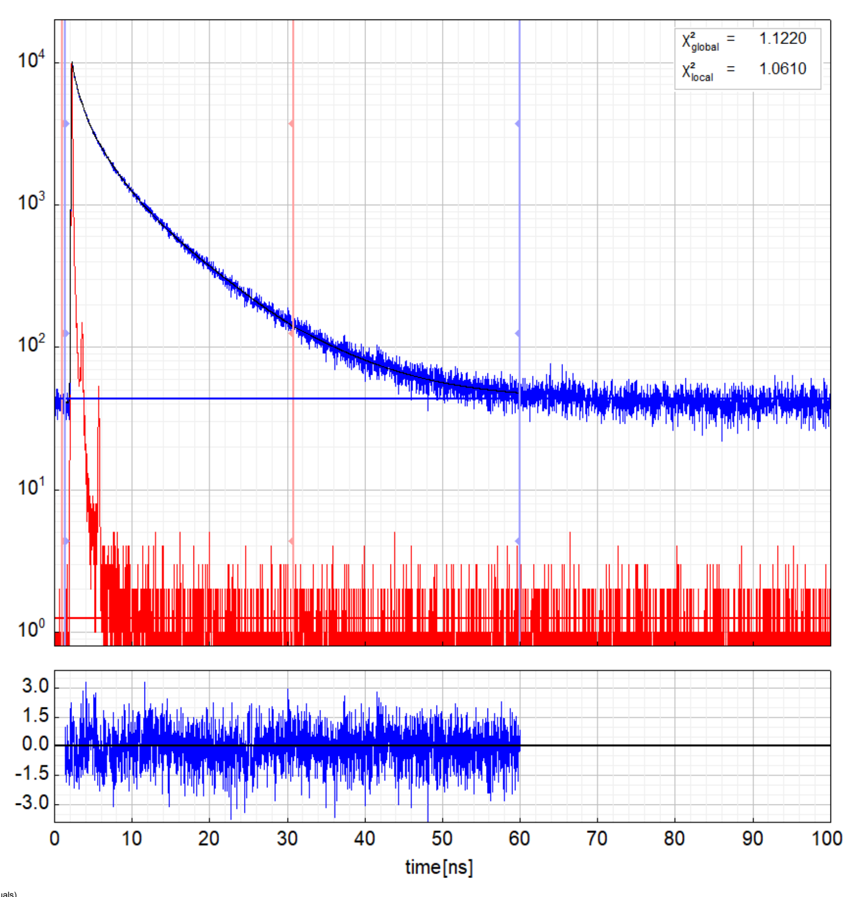

Autocorrelation (Residuals)

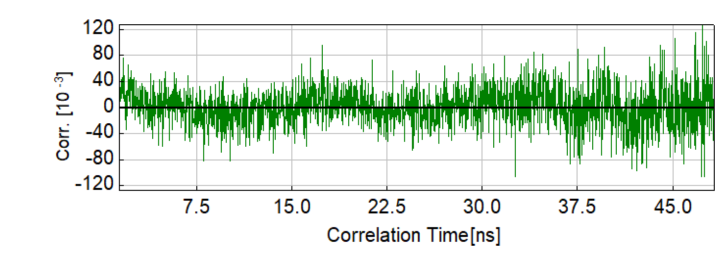

Fitted Parameters

Parameter	Value	Δ	δ
A_1[kCnts/Chnl]	2.088	±0.018	0.8%
τ_1[ns]	9.558	±0.017	0.2%
I_1[kCnts]	797.9	±4.6	0.6%
A_2[kCnts/Chnl]	4.958	±0.082	1.6%
τ_2[ns]	0.733	±0.012	1.6%
I_2[kCnts]	145.2	±4.6	3.2%
A_3[kCnts/Chnl]	3.888	±0.056	1.4%
τ_3[ns]	2.912	±0.033	1.1%
I_3[kCnts]	452.7	±3.7	0.8%
$Bkgr_{Dec}$[kCnts]	0.0432	±0.0003	0.6%
$Bkgr_{IRF}$[Cnts/Chnl]	1.242	±0.070	5.6%
$Shift_{IRF}$[ps]	11.31	±0.59	5.1%
A_{Scatt}[kCnts]	41.26	±0.35	0.8%
τ_{AvInt}[ns]	6.484	±0.013	0.2%
τ_{AvAmp}[ns]	3.193	±0.013	0.4%
λ_{Det}[nm]	590.0000	---	---

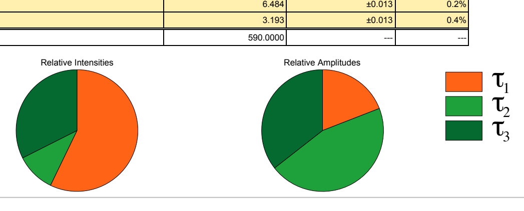

Data Set: 16 / 28

Decay: crv[16]; IRF: crv[0]

Fit

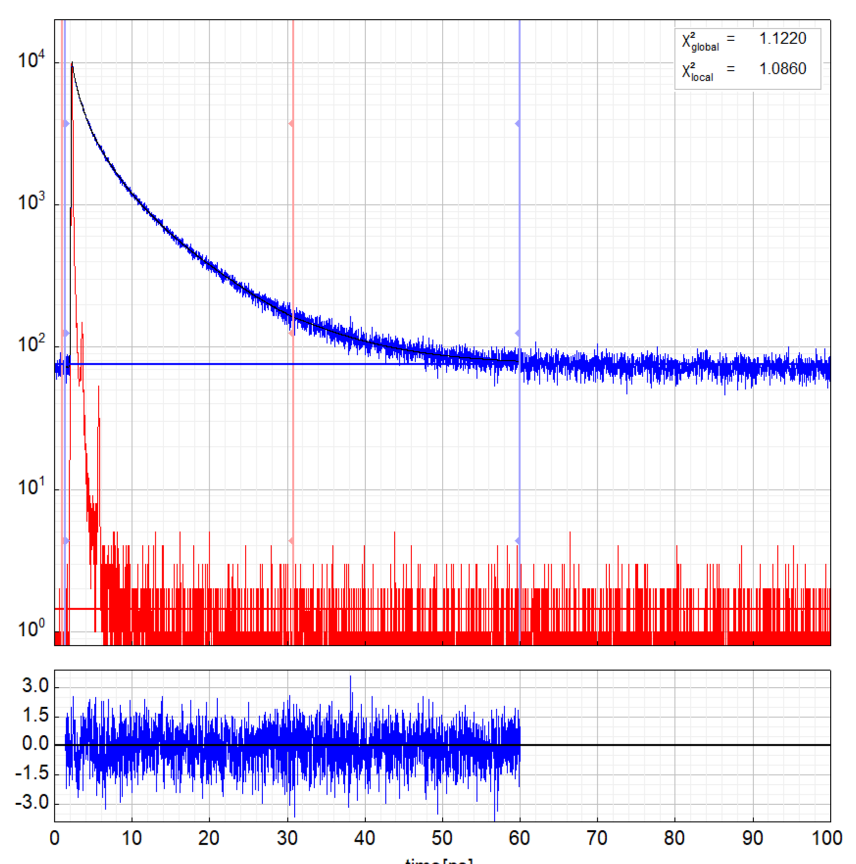

Autocorrelation (Residuals)

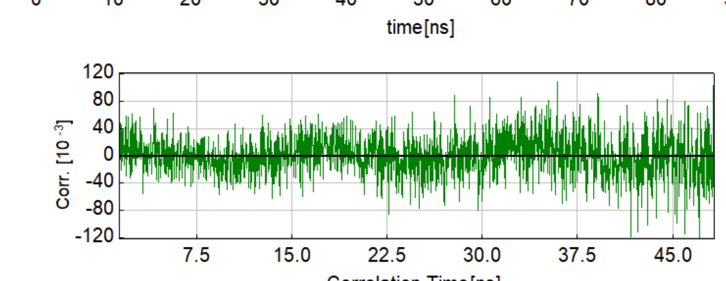

Fitted Parameters

Parameter	Value	Δ	δ
A_1[kCnts/Chnl]	1.936	±0.018	0.9%
τ_1[ns]	9.558	±0.017	0.2%
I_1[kCnts]	740.1	±5.6	0.7%
A_2[kCnts/Chnl]	4.965	±0.047	0.9%
τ_2[ns]	0.733	±0.012	1.6%
I_2[kCnts]	145.4	±3.7	2.5%
A_3[kCnts/Chnl]	3.779	±0.039	1.0%
τ_3[ns]	2.912	±0.033	1.1%
I_3[kCnts]	440.1	±2.6	0.6%
$Bkgr_{Dec}$[kCnts]	0.0753	±0.0005	0.5%
$Bkgr_{IRF}$[Cnts/Chnl]	1.435	±0.076	5.3%
$Shift_{IRF}$[ps]	12.27	±0.56	4.5%
A_{Scatt}[kCnts]	43.46	±0.58	1.3%
τ_{AvInt}[ns]	6.383	±0.011	0.2%
τ_{AvAmp}[ns]	3.104	±0.017	0.5%
λ_{Det}[nm]	600.0000	---	---

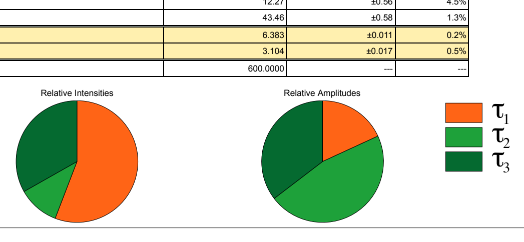

Data Set: 17 / 28

Decay: crv[17]; IRF: crv[0]

Fit

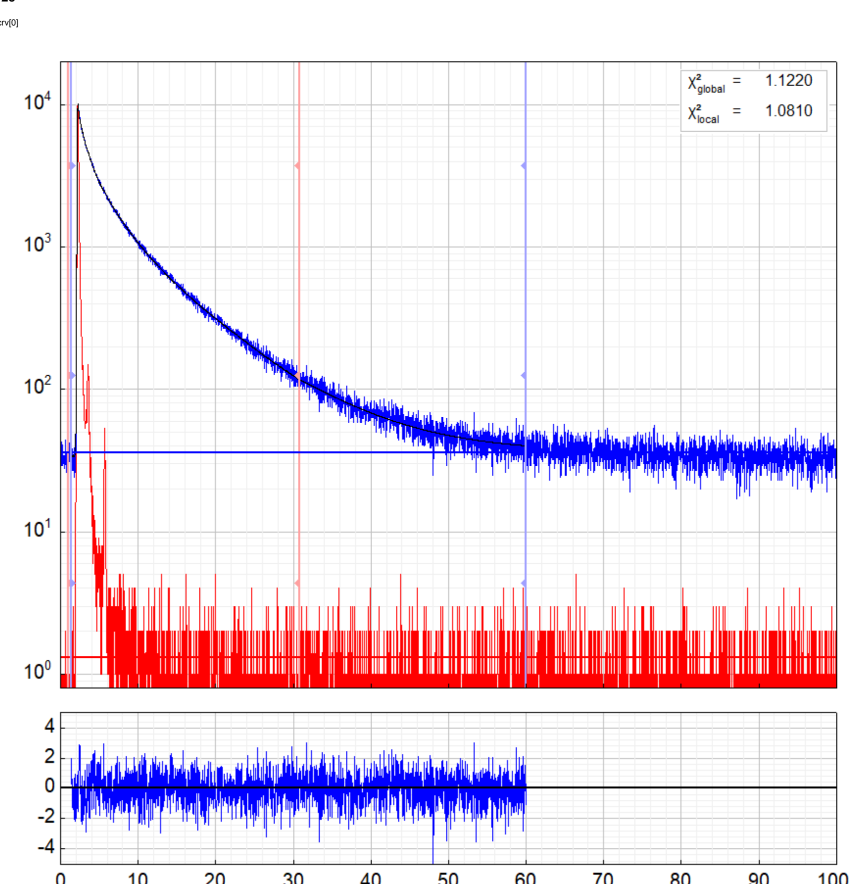

Autocorrelation (Residuals)

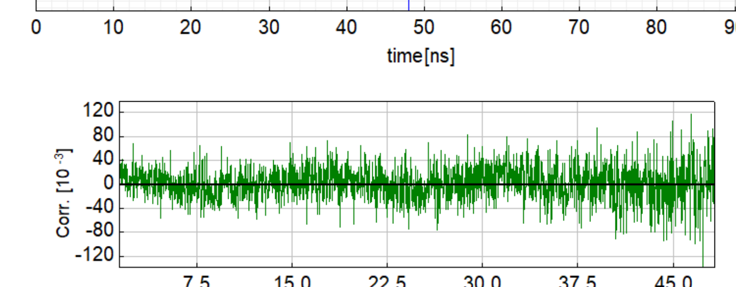

Fitted Parameters

Parameter	Value	Δ	δ
A_1[kCnts/Chnl]	1.771	±0.012	0.6%
τ_1[ns]	9.558	±0.017	0.2%
I_1[kCnts]	676.8	±3.5	0.5%
A_2[kCnts/Chnl]	4.803	±0.058	1.2%
τ_2[ns]	0.733	±0.012	1.6%
I_2[kCnts]	140.7	±3.5	2.5%
A_3[kCnts/Chnl]	3.525	±0.032	0.9%
τ_3[ns]	2.912	±0.033	1.1%
I_3[kCnts]	410.5	±2.9	0.7%
$Bkgr_{Dec}$[kCnts]	0.0362	±0.0003	0.8%
$Bkgr_{IRF}$[Cnts/Chnl]	1.313	±0.082	6.2%
$Shift_{IRF}$[ps]	14.21	±0.54	3.8%
A_{Scatt}[kCnts]	46.63	±0.35	0.7%
τ_{AvInt}[ns]	6.326	±0.023	0.4%
τ_{AvAmp}[ns]	3.041	±0.016	0.5%
λ_{Det}[nm]	610.0000	---	---

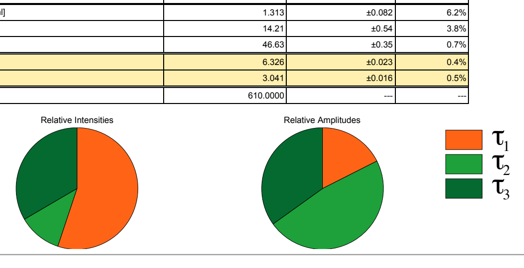

Data Set: 18 / 28

Decay: crv[18]; IRF: crv[0]

Fit

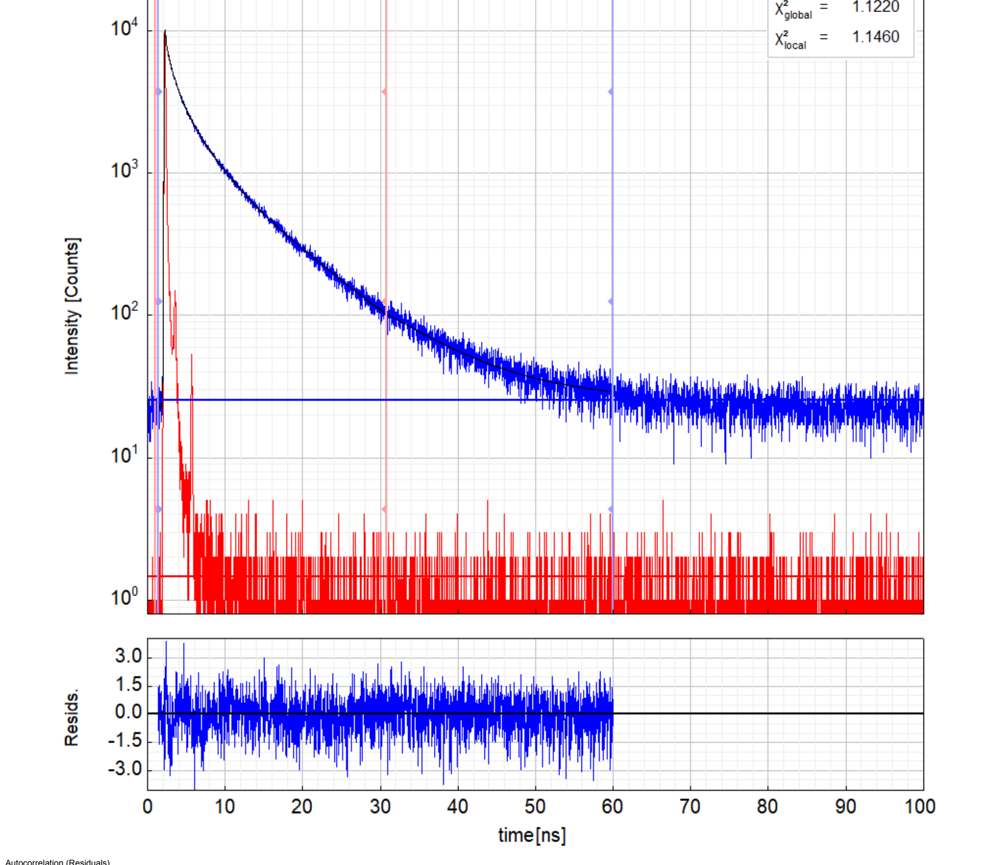

Autocorrelation (Residuals)

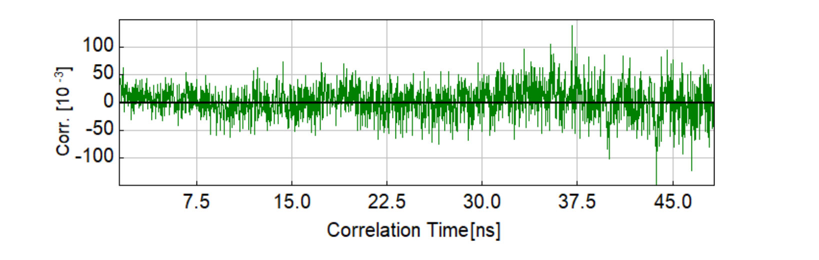

Fitted Parameters

Parameter	Value	Δ	δ
A_1[kCnts/Chnl]	1.723	±0.015	0.9%
$τ_1$[ns]	9.558	±0.017	0.2%
I_1[kCnts]	658.5	±3.7	0.6%
A_2[kCnts/Chnl]	4.743	±0.061	1.3%
$τ_2$[ns]	0.733	±0.012	1.6%
I_2[kCnts]	138.9	±3.8	2.7%
A_3[kCnts/Chnl]	3.425	±0.030	0.9%
$τ_3$[ns]	2.912	±0.033	1.1%
I_3[kCnts]	398.9	±2.9	0.7%
$Bkgr_{Dec}$[kCnts]	0.0253	±0.0003	0.9%
$Bkgr_{IRF}$[Cnts/Chnl]	1.461	±0.077	5.2%
$Shift_{IRF}$[ps]	15.21	±0.56	3.6%
A_{Scatt}[kCnts]	48.40	±0.84	1.7%
$τ_{AvInt}$[ns]	6.317	±0.014	0.2%
$τ_{AvAmp}$[ns]	3.024	±0.020	0.6%
$λ_{Det}$[nm]	620.0000	---	---

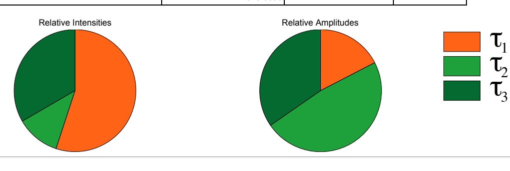

Data Set: 19 / 28

Decay: crv[19]; IRF: crv[0]

Fit

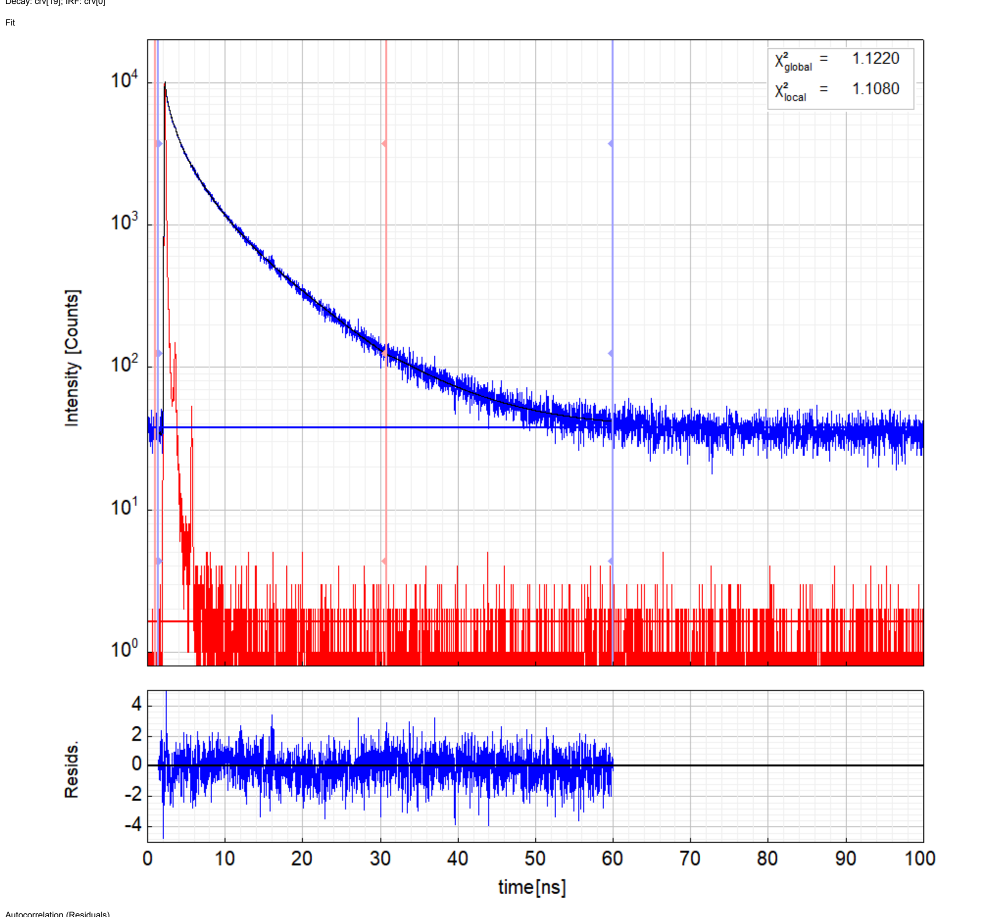

Autocorrelation (Residuals)

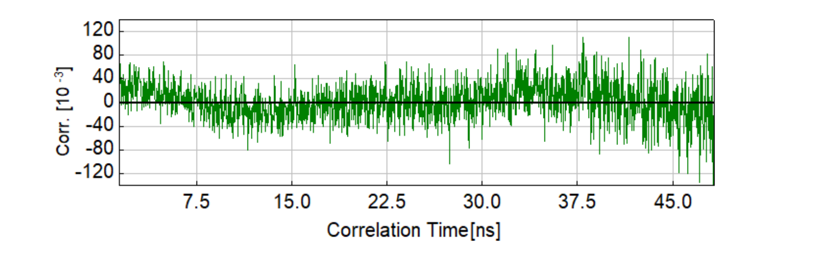

Fitted Parameters

Parameter	Value	Δ	δ
A_1[kCnts/Chnl]	1.980	±0.012	0.6%
$τ_1$[ns]	9.558	±0.017	0.2%
I_1[kCnts]	756.8	±2.7	0.3%
A_2[kCnts/Chnl]	4.148	±0.057	1.4%
$τ_2$[ns]	0.733	±0.012	1.6%
I_2[kCnts]	121.5	±4.2	3.4%
A_3[kCnts/Chnl]	3.696	±0.046	1.2%
$τ_3$[ns]	2.912	±0.033	1.1%
I_3[kCnts]	430.4	±1.7	0.4%
$Bkgr_{Dec}$[kCnts]	0.0374	±0.0004	1.0%
$Bkgr_{IRF}$[Cnts/Chnl]	1.64	±0.13	7.6%
$Shift_{IRF}$[ps]	16.50	±0.55	3.3%
A_{Scatt}[kCnts]	48.30	±0.56	1.1%
$τ_{AvInt}$[ns]	6.553	±0.017	0.2%
$τ_{AvAmp}$[ns]	3.331	±0.015	0.4%
$λ_{Det}$[nm]	630.0000	---	---

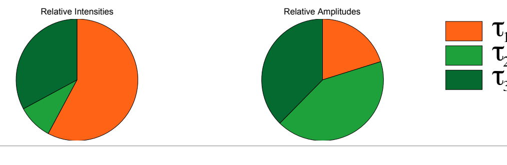

Data Set: 20 / 28

Decay: crv[20]; IRF: crv[0]

Fit

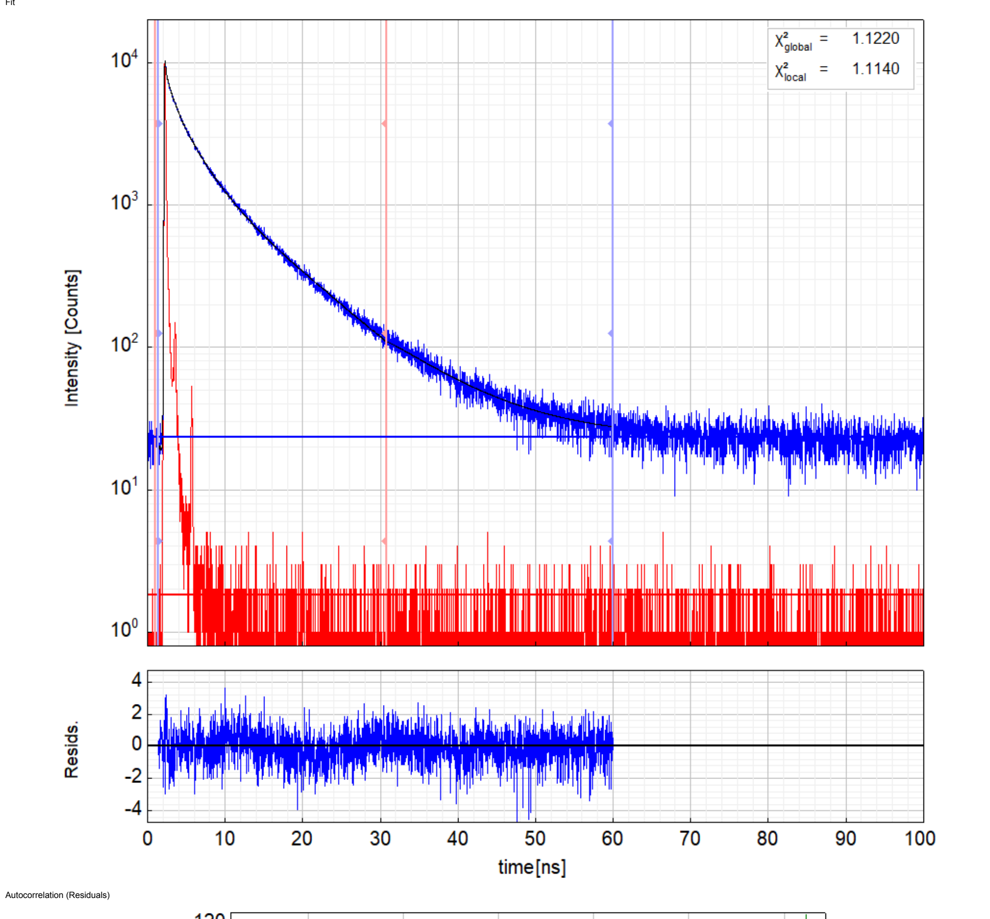

Autocorrelation (Residuals)

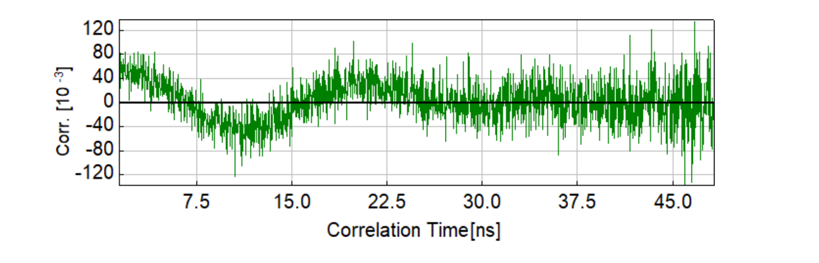

Fitted Parameters

Parameter	Value	Δ	δ
A_1[kCnts/Chnl]	2.067	±0.021	1.0%
$τ_1$[ns]	9.558	±0.017	0.2%
I_1[kCnts]	790.0	±6.7	0.8%
A_2[kCnts/Chnl]	3.587	±0.060	1.7%
$τ_2$[ns]	0.733	±0.012	1.6%
I_2[kCnts]	105.1	±1.6	1.5%
A_3[kCnts/Chnl]	4.382	±0.022	0.5%
$τ_3$[ns]	2.912	±0.033	1.1%
I_3[kCnts]	510.3	±4.8	0.9%
$Bkgr_{Dec}$[kCnts]	0.0233	±0.0003	1.2%
$Bkgr_{IRF}$[Cnts/Chnl]	1.82	±0.12	6.5%
$Shift_{IRF}$[ps]	17.79	±0.79	4.4%
A_{Scatt}[kCnts]	48.4	±1.2	2.3%
$τ_{AvInt}$[ns]	6.485	±0.018	0.3%
$τ_{AvAmp}$[ns]	3.501	±0.012	0.3%
$λ_{Det}$[nm]	640.0000	---	---

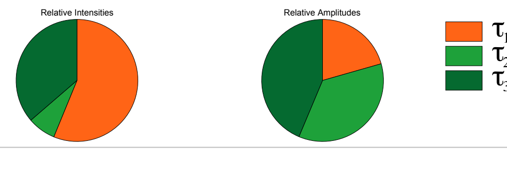

Data Set: 21 / 28

Decay: crv[21]; IRF: crv[0]

Fit

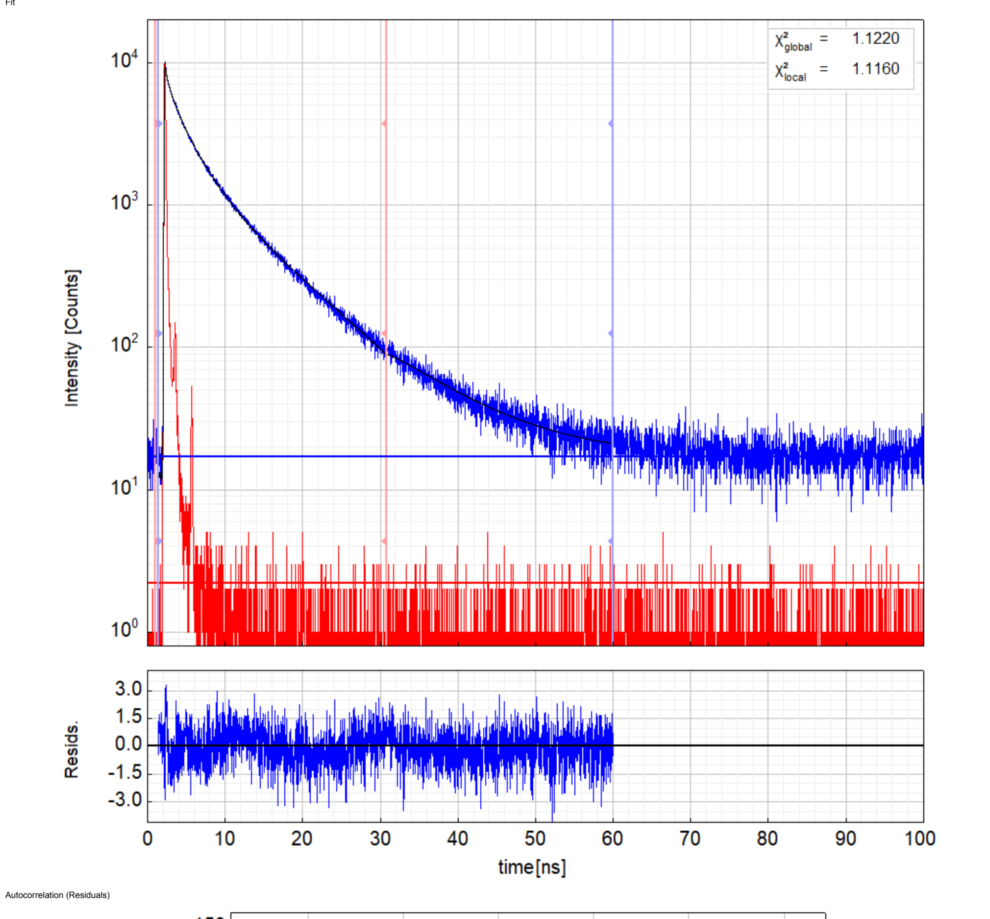

Autocorrelation (Residuals)

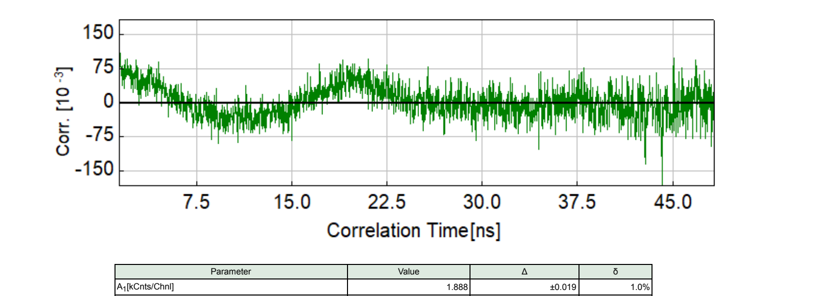

Fitted Parameters

Parameter	Value	Δ	δ
A_1[kCnts/Chnl]	1.888	±0.019	1.0%
$τ_1$[ns]	9.558	±0.017	0.2%
I_1[kCnts]	721.7	±5.0	0.7%
A_2[kCnts/Chnl]	3.300	±0.052	1.5%
$τ_2$[ns]	0.733	±0.012	1.6%
I_2[kCnts]	96.7	±2.5	2.5%
A_3[kCnts/Chnl]	4.656	±0.040	0.8%
$τ_3$[ns]	2.912	±0.033	1.1%
I_3[kCnts]	542.2	±4.0	0.7%
$Bkgr_{Dec}$[kCnts]	0.0172	±0.0002	1.1%
$Bkgr_{IRF}$[Cnts/Chnl]	2.197	±0.076	3.4%
$Shift_{IRF}$[ps]	18.02	±0.76	4.2%
A_{Scatt}[kCnts]	47.4	±1.2	2.4%
$τ_{AvInt}$[ns]	6.2821	±0.0060	0.1%
$τ_{AvAmp}$[ns]	3.456	±0.015	0.4%
$λ_{Det}$[nm]	650.0000	---	---

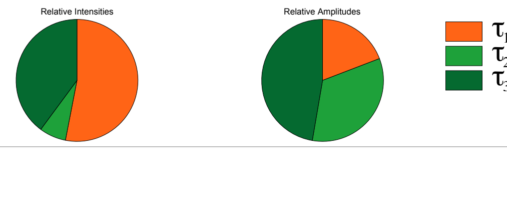

Data Set: 22 / 28

Decay: crv[22]; IRF: crv[0]

Fit

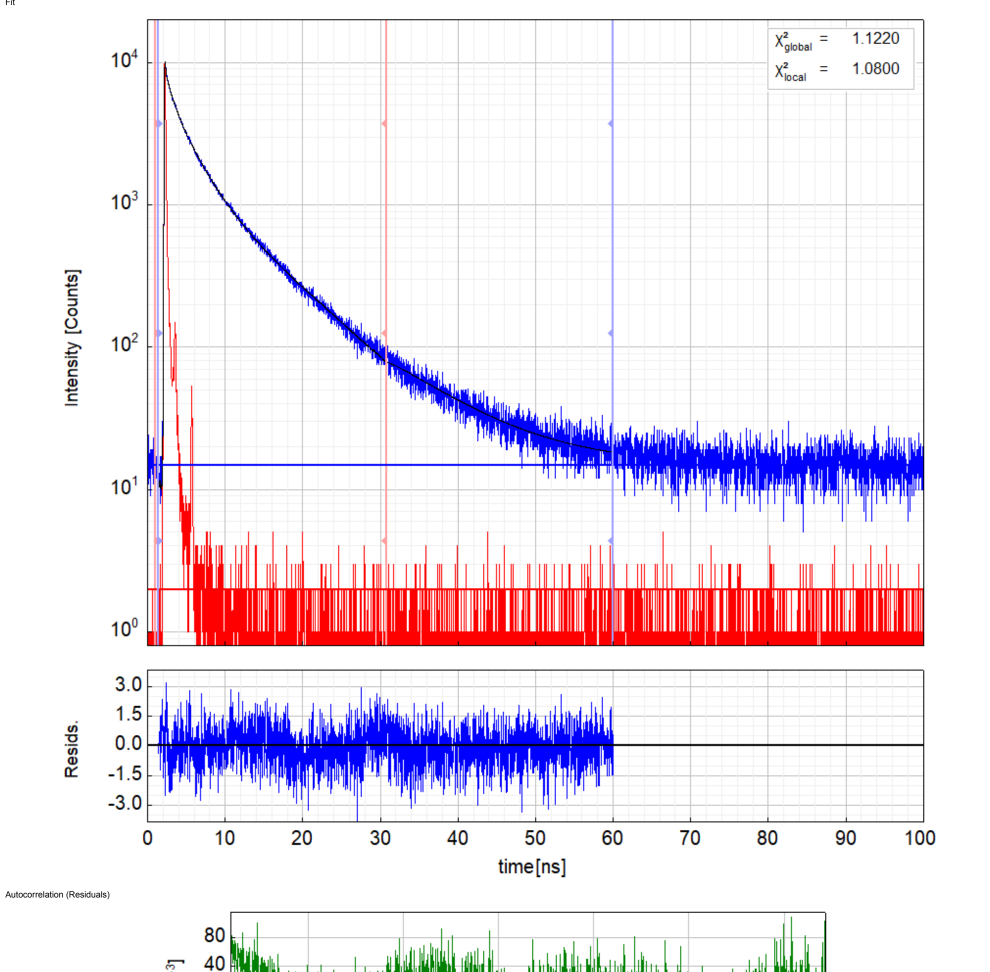

Autocorrelation (Residuals)

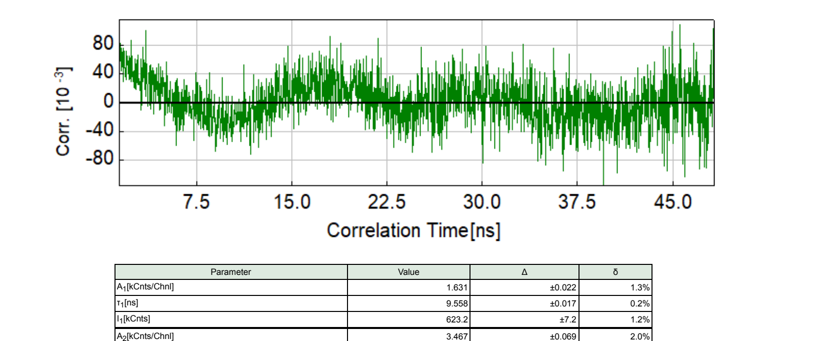

Fitted Parameters

Parameter	Value	Δ	δ
A_1[kCnts/Chnl]	1.631	±0.022	1.3%
$τ_1$[ns]	9.558	±0.017	0.2%
I_1[kCnts]	623.2	±7.2	1.2%
A_2[kCnts/Chnl]	3.467	±0.069	2.0%
$τ_2$[ns]	0.733	±0.012	1.6%
I_2[kCnts]	101.6	±2.9	2.8%
A_3[kCnts/Chnl]	4.735	±0.060	1.3%
$τ_3$[ns]	2.912	±0.033	1.1%
I_3[kCnts]	551.4	±4.1	0.7%
$Bkgr_{Dec}$[kCnts]	0.0148	±0.0002	0.7%
$Bkgr_{IRF}$[Cnts/Chnl]	1.987	±0.084	4.2%
$Shift_{IRF}$[ps]	19.03	±0.77	4.0%
A_{Scatt}[kCnts]	48.8	±2.2	4.5%
$τ_{AvInt}$[ns]	5.984	±0.013	0.2%
$τ_{AvAmp}$[ns]	3.246	±0.018	0.6%
$λ_{Det}$[nm]	660.0000	---	---

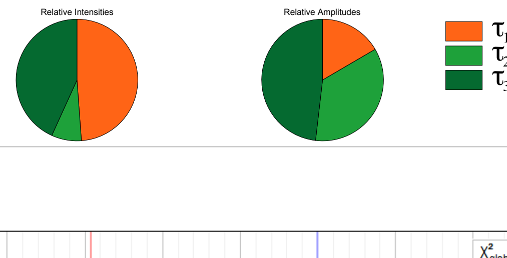

Data Set: 23 / 28

Decay: crv[23]; IRF: crv[0]

Fit

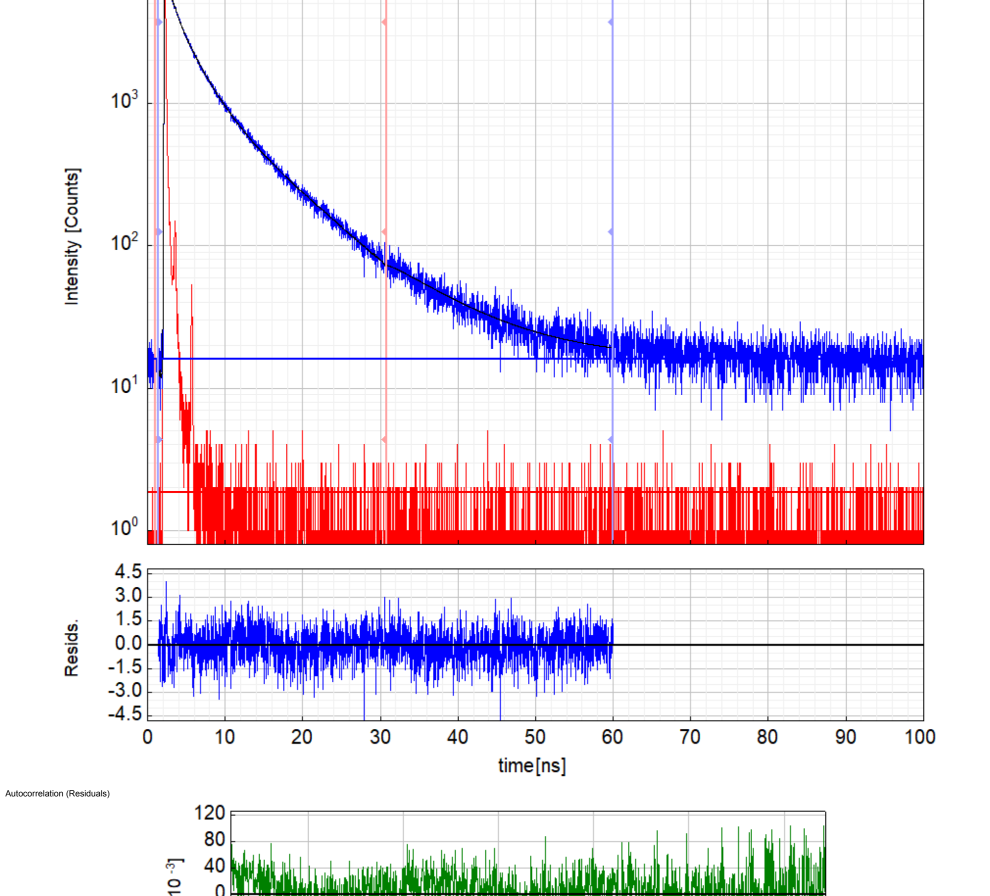

Autocorrelation (Residuals)

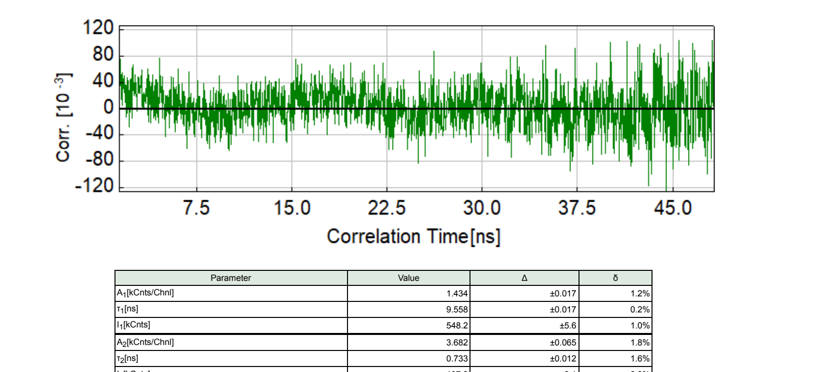

Fitted Parameters

Parameter	Value	Δ	δ
A_1[kCnts/Chnl]	1.434	±0.017	1.2%
$τ_1$[ns]	9.558	±0.017	0.2%
I_1[kCnts]	548.2	±5.6	1.0%
A_2[kCnts/Chnl]	3.682	±0.065	1.8%
$τ_2$[ns]	0.733	±0.012	1.6%
I_2[kCnts]	107.9	±3.1	2.8%
A_3[kCnts/Chnl]	4.411	±0.037	0.8%
$τ_3$[ns]	2.912	±0.033	1.1%
I_3[kCnts]	513.7	±1.7	0.3%
$Bkgr_{Dec}$[kCnts]	0.0162	±0.0003	1.4%
$Bkgr_{IRF}$[Cnts/Chnl]	1.861	±0.089	4.7%
$Shift_{IRF}$[ps]	19.58	±0.70	3.6%
[illegible]	[illegible]	[illegible]	[illegible]

Parameter	Value	Δ	δ
A_{Scatt}[kCnts]	50.3	±1.1	2.2%
τ_{AvInt}[ns]	5.826	±0.012	0.2%
τ_{AvAmp}[ns]	3.070	±0.016	0.5%
λ_{Det}[nm]	670.0000	---	---

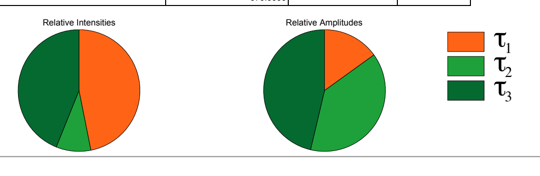

Data Set: 24 / 28

Decay: crv[24]; IRF: crv[0]

Fit

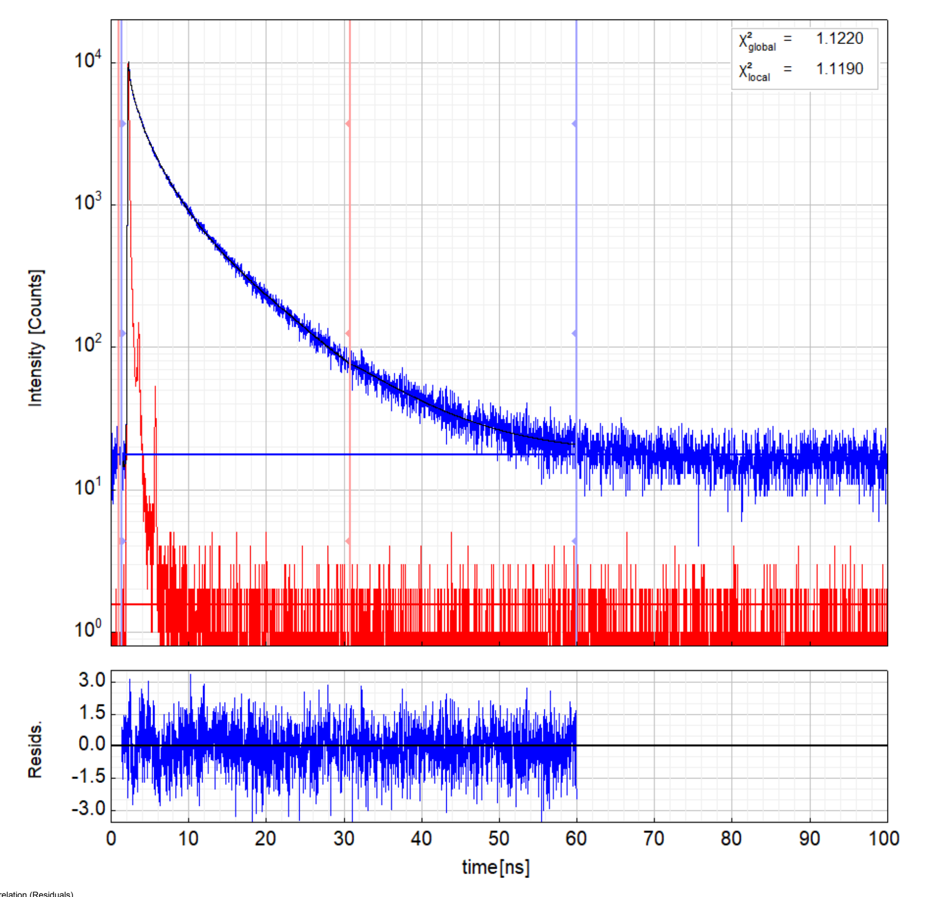

Autocorrelation (Residuals)

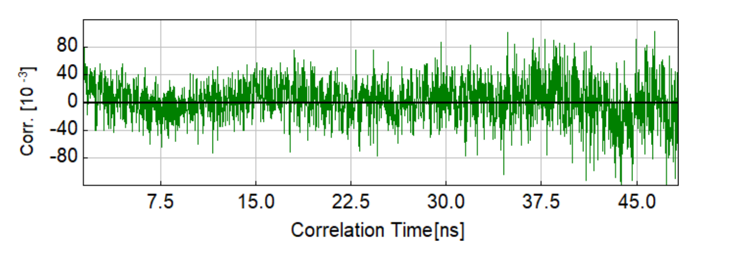

Fitted Parameters

Parameter	Value	Δ	δ
A_1[kCnts/Chnl]	1.376	±0.017	1.2%
τ_1[ns]	9.558	±0.017	0.2%
I_1[kCnts]	525.7	±5.4	1.0%
A_2[kCnts/Chnl]	3.94	±0.12	2.9%
τ_2[ns]	0.733	±0.012	1.6%
I_2[kCnts]	115.2	±3.3	2.9%
A_3[kCnts/Chnl]	4.005	±0.054	1.3%
τ_3[ns]	2.912	±0.033	1.1%
I_3[kCnts]	466.4	±2.8	0.6%
$Bkgr_{Dec}$[kCnts]	0.0177	±0.0002	0.6%
$Bkgr_{IRF}$[Cnts/Chnl]	1.562	±0.054	3.4%
$Shift_{IRF}$[ps]	19.75	±0.57	2.9%
A_{Scatt}[kCnts]	50.78	±0.64	1.3%
τ_{AvInt}[ns]	5.8404	±0.0087	0.1%
τ_{AvAmp}[ns]	2.973	±0.020	0.6%
λ_{Det}[nm]	680.0000	---	---

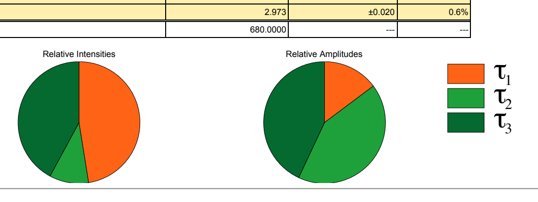

Data Set: 25 / 28

Decay: crv[25]; IRF: crv[0]

Fit

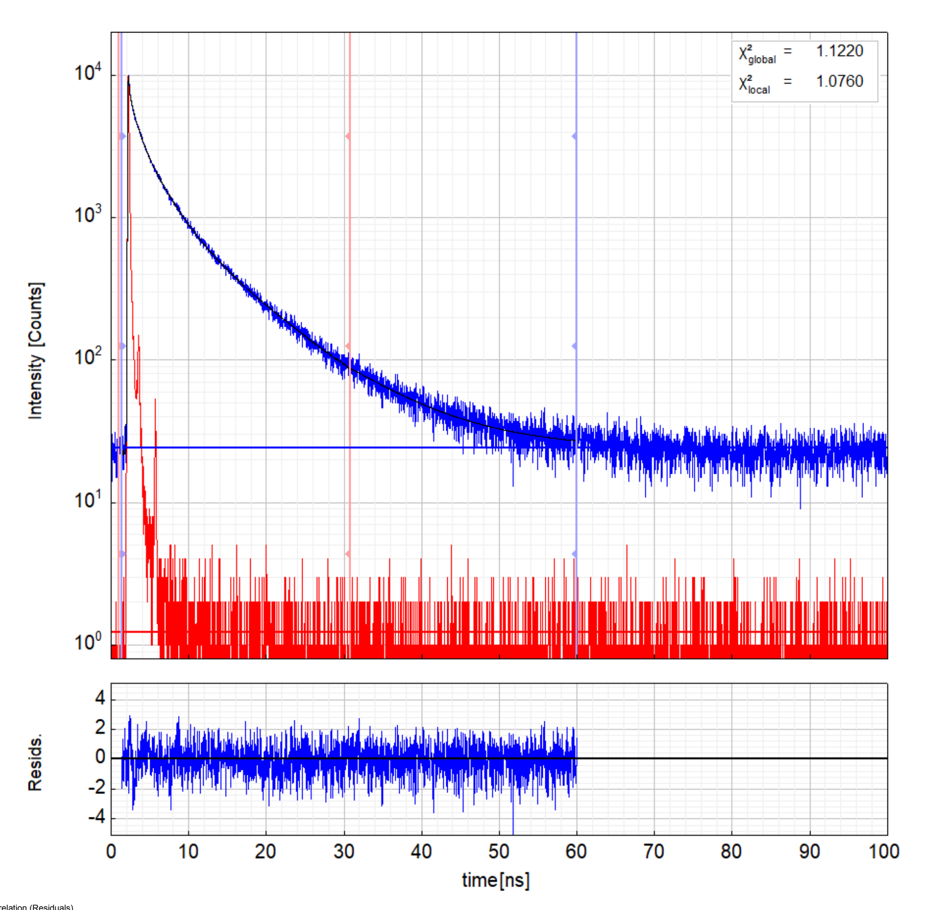

Autocorrelation (Residuals)

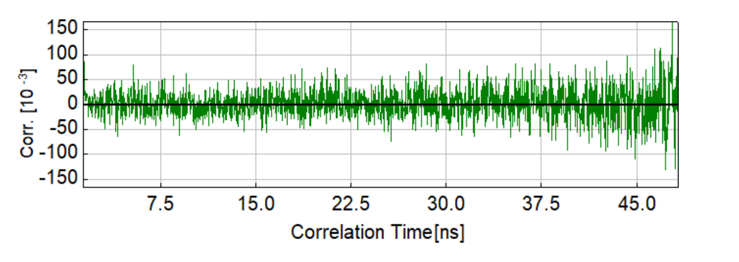

Fitted Parameters

Parameter	Value	Δ	δ
A_1[kCnts/Chnl]	1.374	±0.012	0.8%
τ_1[ns]	9.558	±0.017	0.2%
I_1[kCnts]	525.1	±3.7	0.7%
A_2[kCnts/Chnl]	3.865	±0.031	0.8%
τ_2[ns]	0.733	±0.012	1.6%
I_2[kCnts]	113.2	±2.8	2.4%
A_3[kCnts/Chnl]	3.641	±0.039	1.1%
τ_3[ns]	2.912	±0.033	1.1%
I_3[kCnts]	424.1	±1.2	0.3%
$Bkgr_{Dec}$[kCnts]	0.0240	±0.0003	0.9%
$Bkgr_{IRF}$[Cnts/Chnl]	1.22	±0.11	8.5%
$Shift_{IRF}$[ps]	21.18	±0.70	3.3%
A_{Scatt}[kCnts]	50.76	±0.77	1.5%
τ_{AvInt}[ns]	5.965	±0.019	0.3%
τ_{AvAmp}[ns]	2.992	±0.014	0.5%
λ_{Det}[nm]	690.0000	---	---

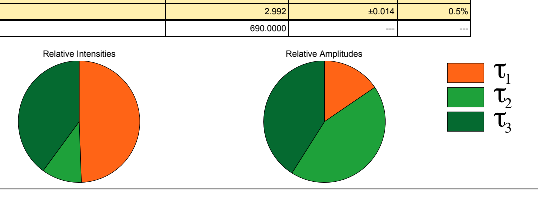

Data Set: 26 / 28

Decay: crv[26]; IRF: crv[0]

Fit

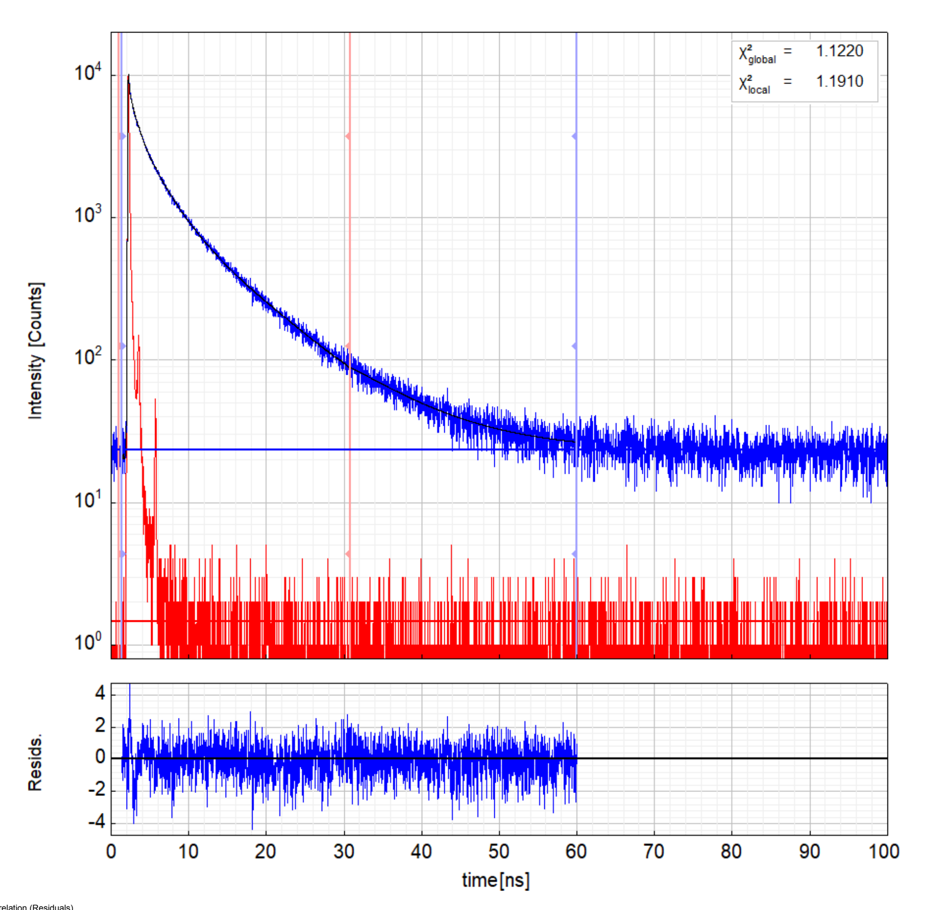

Autocorrelation (Residuals)

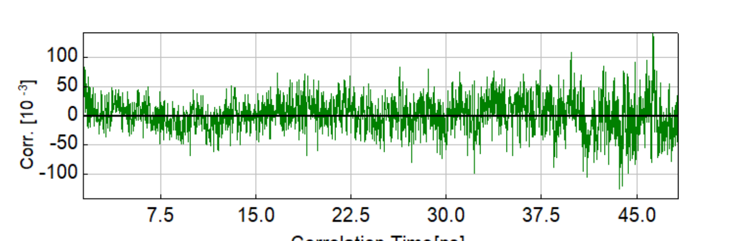

Fitted Parameters

Parameter	Value	Δ	δ
A_1[kCnts/Chnl]	1.482	±0.017	1.1%
τ_1[ns]	9.558	±0.017	0.2%
I_1[kCnts]	566.2	±6.4	1.1%
A_2[kCnts/Chnl]	3.933	±0.096	2.4%
τ_2[ns]	0.733	±0.012	1.6%
I_2[kCnts]	115.2	±2.8	2.4%
A_3[kCnts/Chnl]	3.682	±0.032	0.8%
τ_3[ns]	2.912	±0.033	1.1%
I_3[kCnts]	428.8	±2.7	0.6%
$Bkgr_{Dec}$[kCnts]	0.0233	±0.0004	1.7%
$Bkgr_{IRF}$[Cnts/Chnl]	1.469	±0.094	6.4%
$Shift_{IRF}$[ps]	21.79	±0.69	3.2%
A_{Scatt}[kCnts]	52.2	±1.4	2.5%
τ_{AvInt}[ns]	6.075	±0.014	0.2%
τ_{AvAmp}[ns]	3.052	±0.025	0.8%
λ_{Det}[nm]	700.0000	---	---

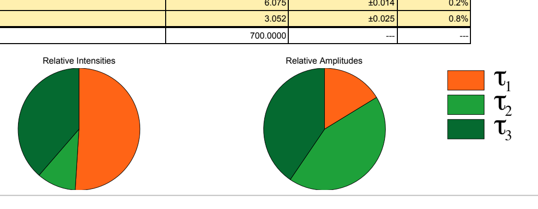

Data Set: 27 / 28

Decay: crv[27]; IRF: crv[0]

Fit

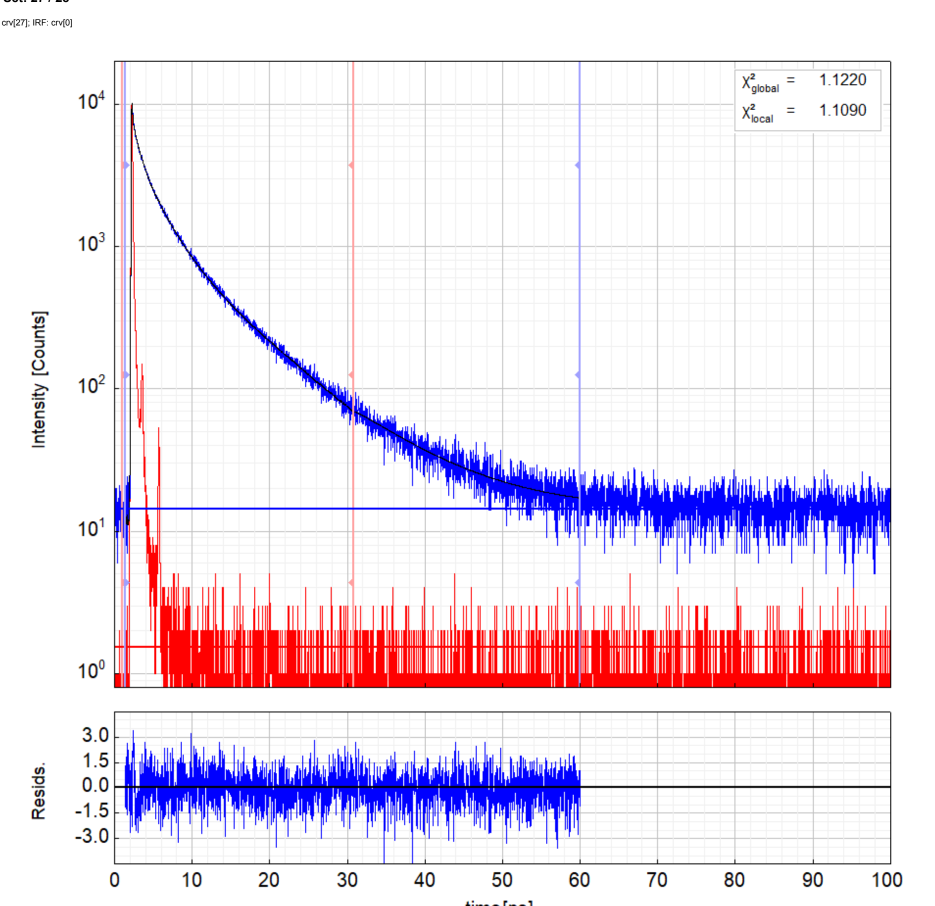

Autocorrelation (Residuals)

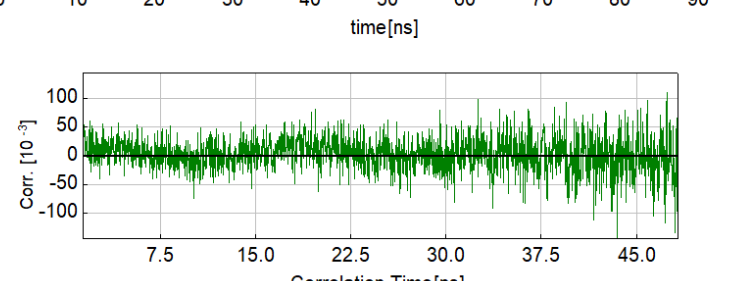

Fitted Parameters

Parameter	Value	Δ	δ
A_1[kCnts/Chnl]	1.287	±0.012	0.9%
τ_1[ns]	9.558	±0.017	0.2%
I_1[kCnts]	491.7	±3.2	0.6%
A_2[kCnts/Chnl]	3.893	±0.082	2.1%
τ_2[ns]	0.733	±0.012	1.6%
I_2[kCnts]	114.1	±3.7	3.2%
A_3[kCnts/Chnl]	3.603	±0.038	1.0%
τ_3[ns]	2.912	±0.033	1.1%
I_3[kCnts]	419.6	±1.6	0.4%
$Bkgr_{Dec}$[kCnts]	0.0143	±0.0003	1.5%
$Bkgr_{IRF}$[Cnts/Chnl]	1.523	±0.093	6.1%
$Shift_{IRF}$[ps]	22.93	±0.80	3.5%
A_{Scatt}[kCnts]	53.41	±0.51	1.0%
τ_{AvInt}[ns]	5.857	±0.016	0.3%
τ_{AvAmp}[ns]	2.919	±0.017	0.6%
λ_{Det}[nm]	710.0000	---	---

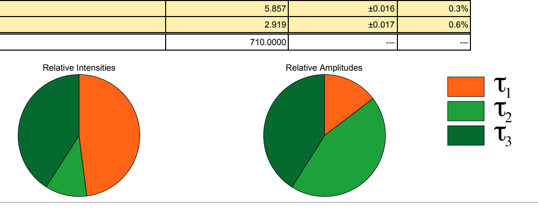

Data Set: 28 / 28

Decay: crv[28]; IRF: crv[0]

Fit

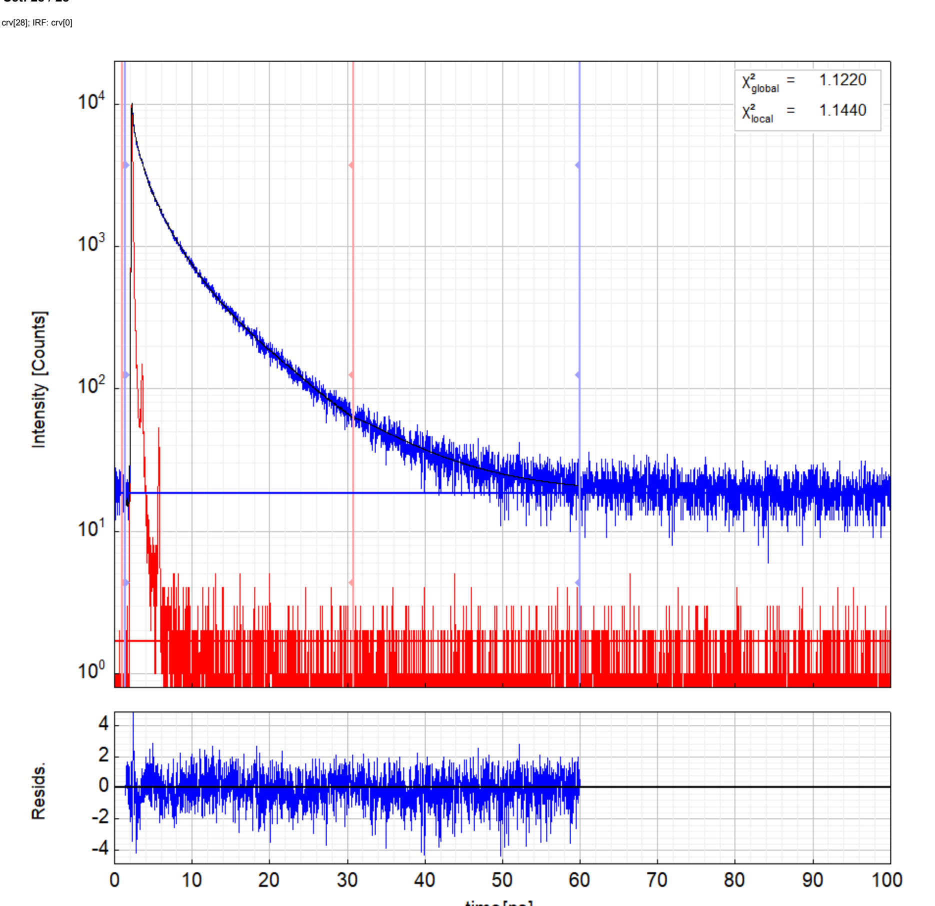

Autocorrelation (Residuals)

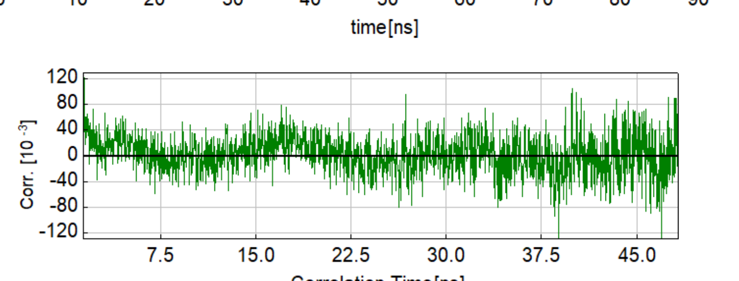

Fitted Parameters

Parameter	Value	Δ	δ
A_1[kCnts/Chnl]	1.0802	±0.0073	0.7%
τ_1[ns]	9.558	±0.017	0.2%
I_1[kCnts]	413.0	±2.2	0.5%
A_2[kCnts/Chnl]	4.027	±0.079	1.9%
τ_2[ns]	0.733	±0.012	1.6%
I_2[kCnts]	118.0	±3.4	2.8%
A_3[kCnts/Chnl]	3.513	±0.050	1.4%
τ_3[ns]	2.912	±0.033	1.1%
I_3[kCnts]	409.1	±2.5	0.6%
$Bkgr_{Dec}$[kCnts]	0.0186	±0.0002	1.0%
$Bkgr_{IRF}$[Cnts/Chnl]	1.690	±0.043	2.5%
$Shift_{IRF}$[ps]	22.62	±0.60	2.6%
A_{Scatt}[kCnts]	55.55	±0.37	0.7%
τ_{AvInt}[ns]	5.5579	±0.0082	0.1%
τ_{AvAmp}[ns]	2.727	±0.022	0.8%
λ_{Det}[nm]	720.0000	---	---

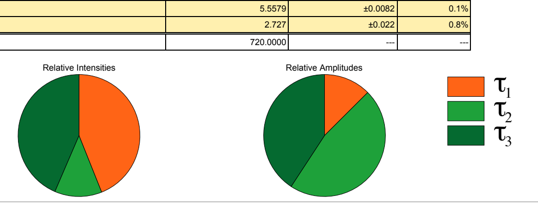

Parameter Plot

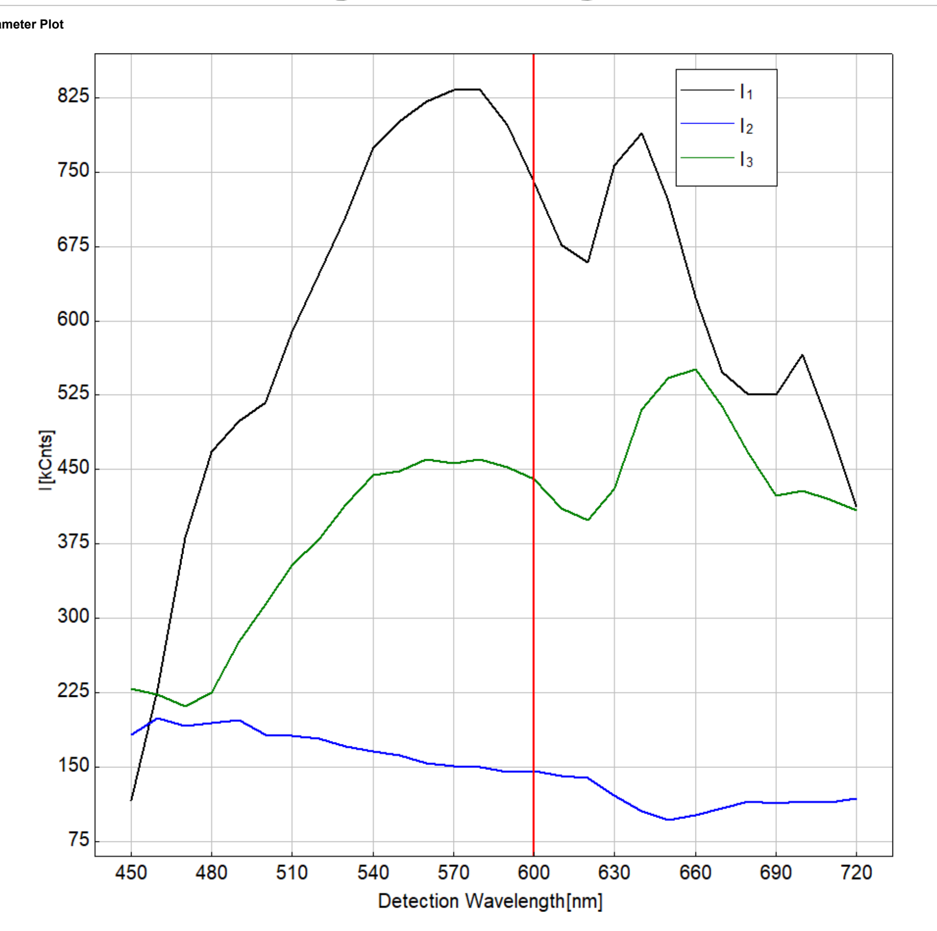

Global Parameters

Parameter	Value	Δ	δ
τ_1[ns]	9.558	±0.017	0.2%
τ_2[ns]	0.733	±0.012	1.6%
τ_3[ns]	2.912	±0.033	1.1%
