# Supplementary material for: A Porphyrin‐Based Organic Network Comprising Sustainable Carbon Dots for Photopolymerization
Source: Angew Chem Int Ed Engl. 2022 Sep 1;61(40):e202208180. doi: 10.1002/anie.202208180 (PMC9826160; doi:10.1002/anie.202208180)
Supplement: Supplementary file 5 — Supporting Information [file ANIE-61-0-s004.pdf]

EasyTau Report

3/11/2022 7:28:11 PM
F:\Paper Xiongfei\CD-1 EtOH tau global.html

Container Curves

TRES+IRF_20201222_1453.etc: crv[0 - 33]

Container Properties

Measurement Context: Time Resolved Emission Scan
Detection Wavelength: 400.0 - 720.0 (Step: 10.0) nm
Summary:

Sample:	CD_X_1
Solvent:	Ethanol
Excitation:	V pol 376±1nm with LDH-P-C-375
Detection:	M pol 400...720nm 10nm steps<br>10000 peak counts<br>grating 1200/500-<br>detector UV-red [PMT]

Comment

Model: Exponential [Reconv.]

$$\mathrm{Dec}(t)=\left[\int_{-\infty}^{t} dt'\left[\mathrm{IRF}(t-Shift_{IRF})-Bkgr_{IRF}\right]\left[\sum_{i=1}^{n_{Exp}} A_i\, e^{-\frac{t-t'}{\tau_i}}+A_{Scatt}\,\delta(t-t')\right]\right]+Bkgr_{Dec}$$

$$I_m = A_m\tau_m$$

$$A_{Rel\,m}=A_m \Big/ \sum_{\substack{i=1\\A_i>0}}^{n_{Exp}} A_i \qquad I_{Rel\,m}=I_m \Big/ \sum_{\substack{i=1\\I_i>0}}^{n_{Exp}} I_i$$

$$\tau_{Av\,Amp}=\sum_{\substack{i=1\\A_i>0}}^{n_{Exp}} A_i\tau_i \Big/ \sum_{\substack{i=1\\A_i>0}}^{n_{Exp}} A_i \qquad \tau_{Av\,Int}=\sum_{\substack{i=1\\I_i>0}}^{n_{Exp}} I_i\tau_i \Big/ \sum_{\substack{i=1\\I_i>0}}^{n_{Exp}} I_i$$

Data Set: 1 / 33

Decay: crv[1]; IRF: crv[0]

Fit

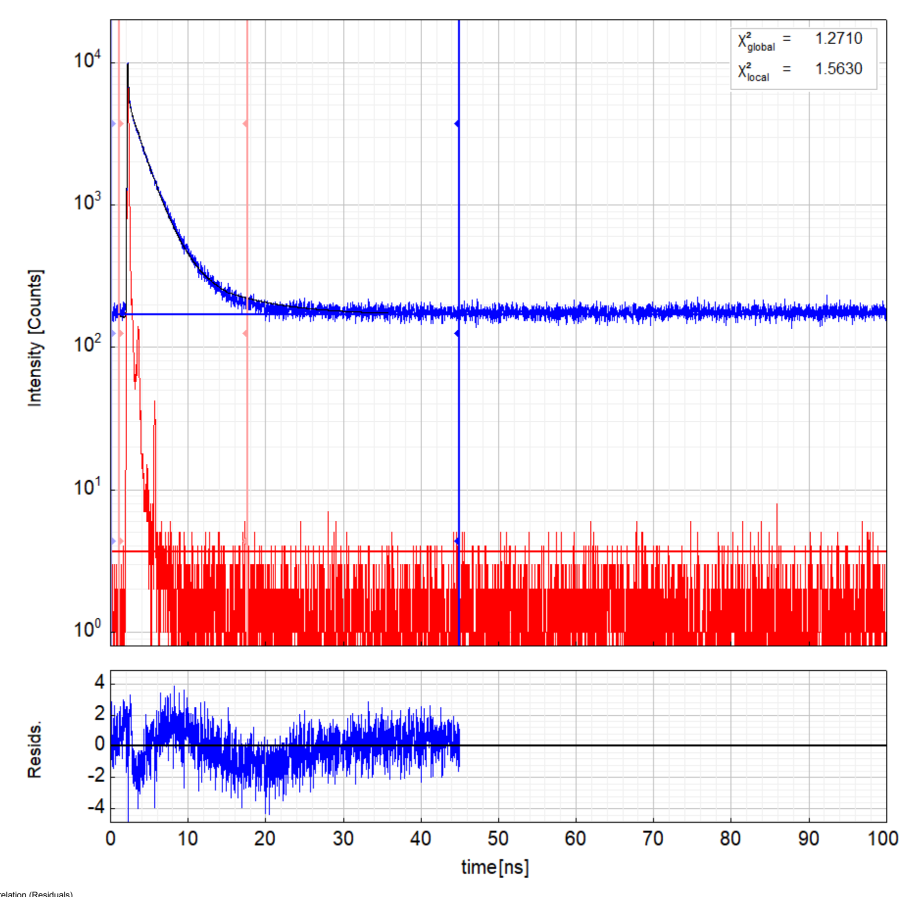

Autocorrelation (Residuals)

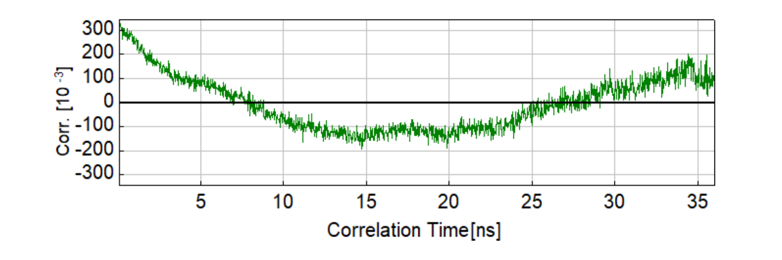

Fitted Parameters

Parameter	Value	Δ	δ
A_1[kCnts/Chnl]	0.35	±0.11	31%
τ_1[ns]	0.460	±0.019	4.0%
I_1[kCnts]	6.3	±2.3	36%
A_2[kCnts/Chnl]	4.939	±0.057	1.1%
τ_2[ns]	2.220	±0.021	0.9%
I_2[kCnts]	438.5	±6.3	1.4%
A_3[kCnts/Chnl]	0.386	±0.042	11%
τ_3[ns]	7.772	±0.017	0.2%
I_3[kCnts]	120	±13	11%
$Bkgr_{Dec}$[kCnts]	0.1687	±0.0011	0.6%
$Bkgr_{IRF}$[Cnts/Chnl]	3.7	±1.2	32%
$Shift_{IRF}$[ps]	-11.42	±0.62	5.4%
A_{Scatt}[kCnts]	62.46	±0.94	1.5%
τ_{AvInt}[ns]	3.379	±0.092	2.7%
τ_{AvAmp}[ns]	2.492	±0.036	1.4%
λ_{Det}[nm]	400.0000	---	---

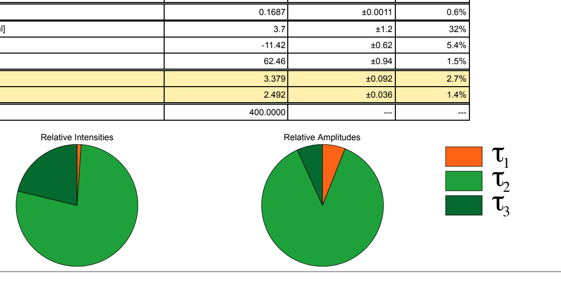

Data Set: 2 / 33

Decay: crv[2]; IRF: crv[0]

Fit

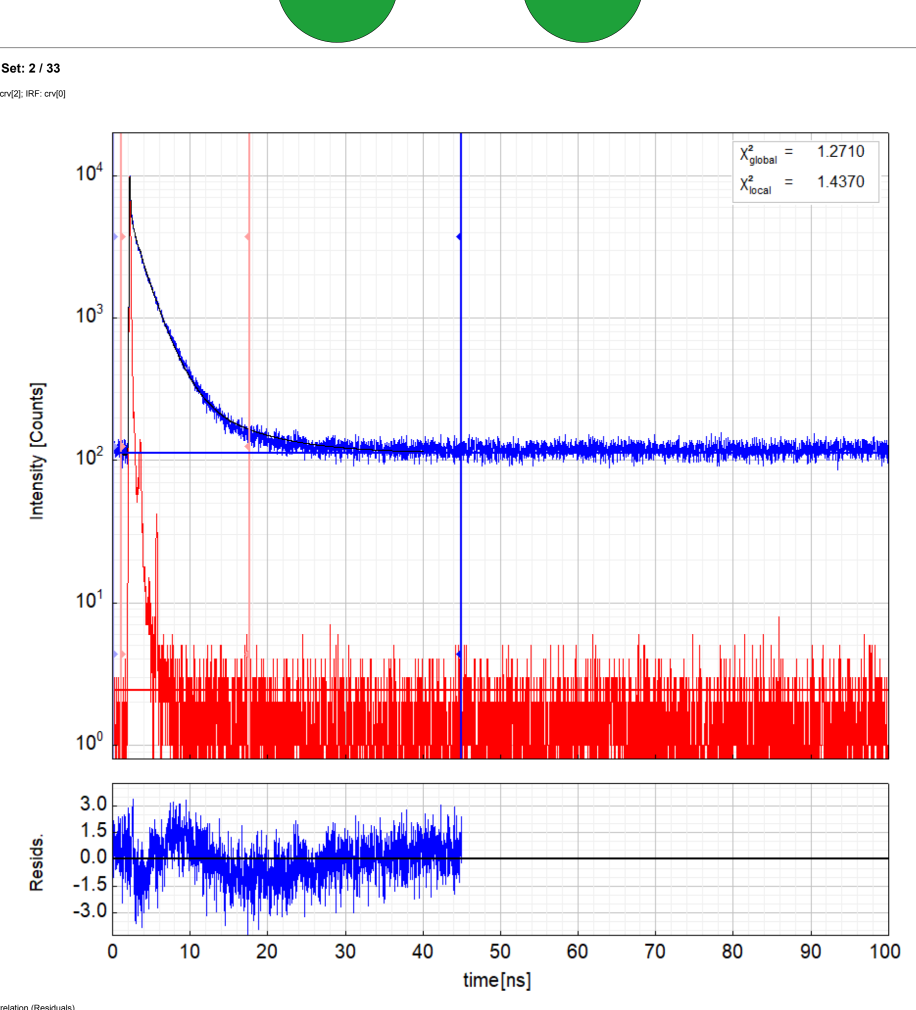

Autocorrelation (Residuals)

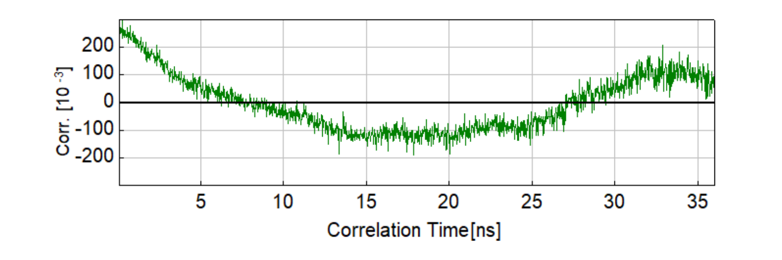

Fitted Parameters

Parameter	Value	Δ	δ
A_1[kCnts/Chnl]	1.129	±0.083	7.3%
τ_1[ns]	0.460	±0.019	4.0%
I_1[kCnts]	20.8	±1.9	8.8%
A_2[kCnts/Chnl]	4.383	±0.050	1.1%
τ_2[ns]	2.220	±0.021	0.9%
I_2[kCnts]	389.2	±4.3	1.1%
A_3[kCnts/Chnl]	0.364	±0.026	7.1%
τ_3[ns]	7.772	±0.017	0.2%
I_3[kCnts]	113.1	±7.8	6.8%
$Bkgr_{Dec}$[kCnts]	0.1123	±0.0006	0.5%
$Bkgr_{IRF}$[Cnts/Chnl]	2.44	±0.79	32%
$Shift_{IRF}$[ps]	-10.04	±0.73	7.2%
A_{Scatt}[kCnts]	62.9	±1.2	1.8%
τ_{AvInt}[ns]	3.351	±0.063	1.9%
τ_{AvAmp}[ns]	2.226	±0.019	0.8%
λ_{Det}[nm]	410.0000	---	---

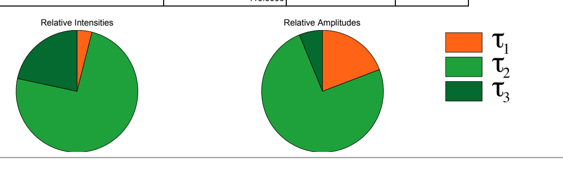

Data Set: 3 / 33

Decay: crv[3]; IRF: crv[0]

Fit

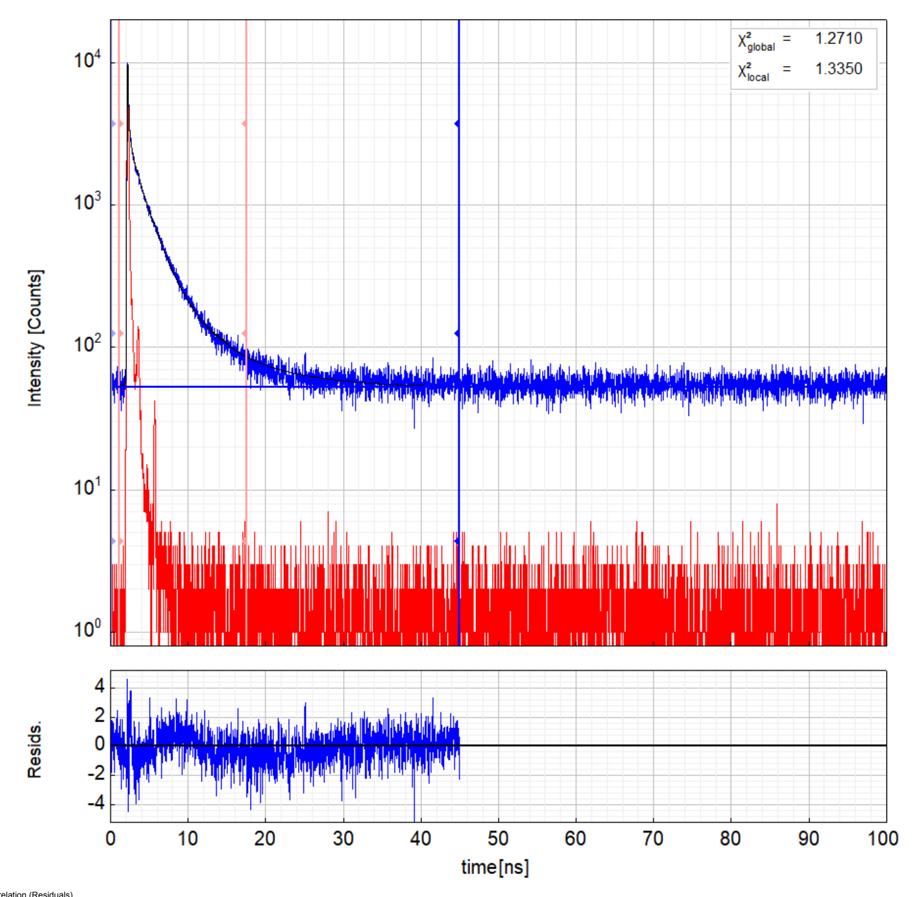

Autocorrelation (Residuals)

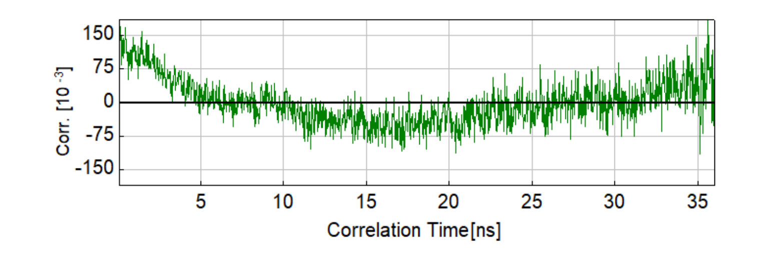

Fitted Parameters

Parameter	Value	Δ	δ
A_1[kCnts/Chnl]	1.02	±0.15	14%
τ_1[ns]	0.460	±0.019	4.0%
I_1[kCnts]	18.8	±2.4	12%
A_2[kCnts/Chnl]	2.379	±0.035	1.4%
τ_2[ns]	2.220	±0.021	0.9%
I_2[kCnts]	211.2	±2.4	1.1%
A_3[kCnts/Chnl]	0.2064	±0.0059	2.8%
τ_3[ns]	7.772	±0.017	0.2%
I_3[kCnts]	64.2	±1.9	2.9%
$Bkgr_{Dec}$[kCnts]	0.0522	±0.0003	0.4%
$Bkgr_{IRF}$[Cnts/Chnl]	-0.17	±0.59	362%
$Shift_{IRF}$[ps]	-18.0	±1.1	5.6%
A_{Scatt}[kCnts]	70.1	±1.4	2.0%
τ_{AvInt}[ns]	3.319	±0.044	1.3%
τ_{AvAmp}[ns]	2.041	±0.032	1.5%
λ_{Det}[nm]	420.0000	---	---

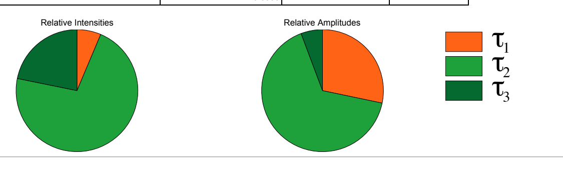

Data Set: 4 / 33

Decay: crv[4]; IRF: crv[0]

Fit

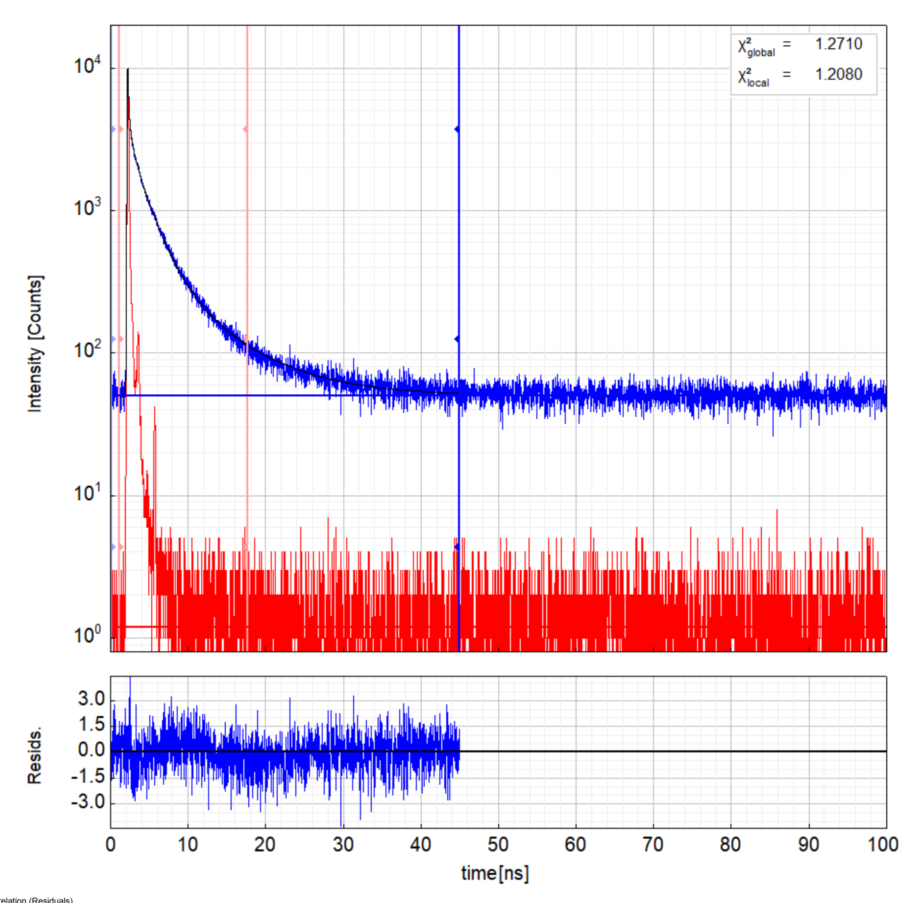

Autocorrelation (Residuals)

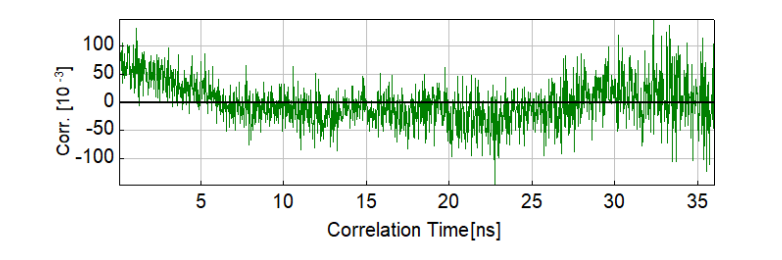

Fitted Parameters

Parameter	Value	Δ	δ
A_1[kCnts/Chnl]	2.216	±0.072	3.2%
τ_1[ns]	0.460	±0.019	4.0%
I_1[kCnts]	40.8	±2.5	6.0%
A_2[kCnts/Chnl]	2.562	±0.035	1.3%
τ_2[ns]	2.220	±0.021	0.9%
I_2[kCnts]	227.5	±1.9	0.8%
A_3[kCnts/Chnl]	0.435	±0.014	3.2%
τ_3[ns]	7.772	±0.017	0.2%
I_3[kCnts]	135.0	±4.1	3.0%
$Bkgr_{Dec}$[kCnts]	0.0504	±0.0004	0.7%
$Bkgr_{IRF}$[Cnts/Chnl]	1.20	±0.47	39%
$Shift_{IRF}$[ps]	-7.97	±0.54	6.7%
A_{Scatt}[kCnts]	67.94	±0.60	0.9%
τ_{AvInt}[ns]	3.901	±0.032	0.8%
τ_{AvAmp}[ns]	1.935	±0.029	1.5%
λ_{Det}[nm]	430.0000	---	---

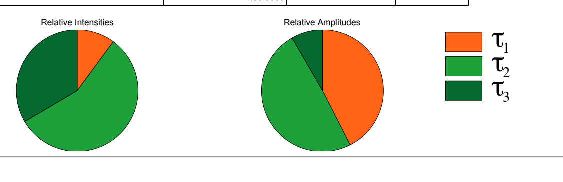

Data Set: 5 / 33

Decay: crv[5]; IRF: crv[0]

Fit

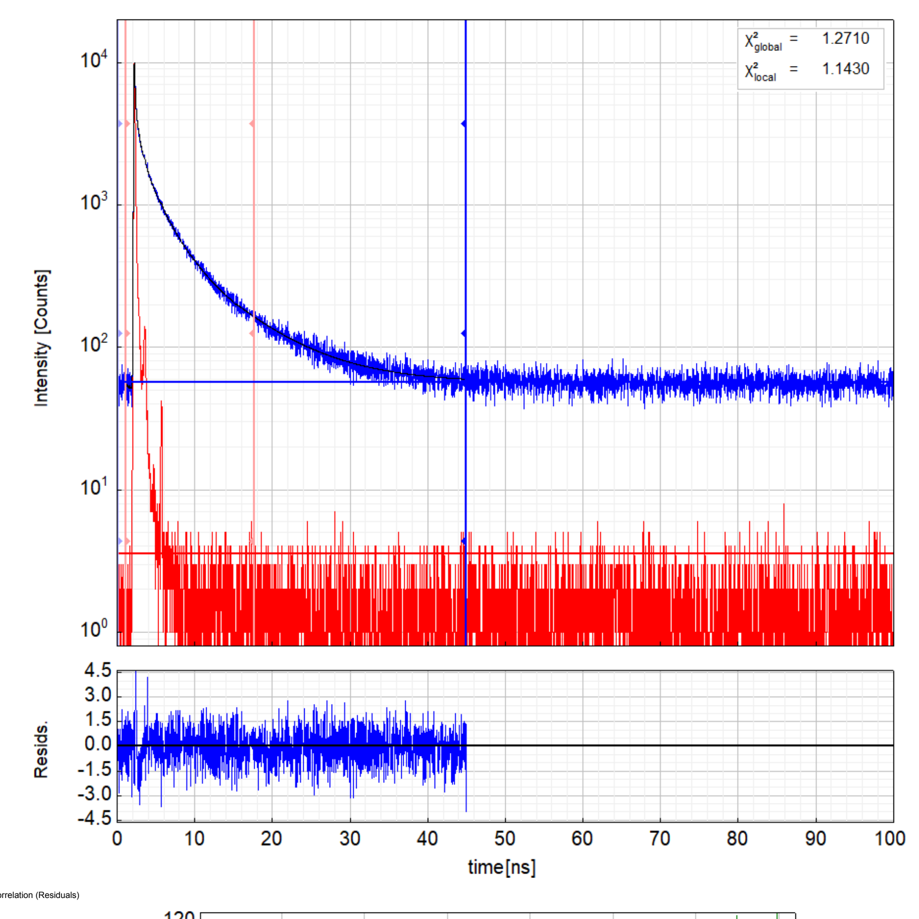

Autocorrelation (Residuals)

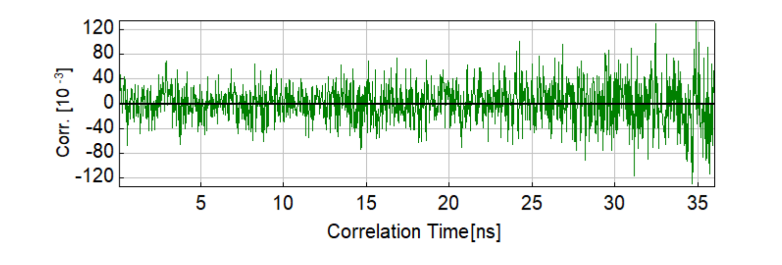

Fitted Parameters

Parameter	Value	Δ	δ
A_1[kCnts/Chnl]	3.15	±0.15	4.6%
τ_1[ns]	0.460	±0.019	4.0%
I_1[kCnts]	58.0	±2.3	3.9%
A_2[kCnts/Chnl]	1.990	±0.023	1.1%
τ_2[ns]	2.220	±0.021	0.9%
I_2[kCnts]	176.7	±2.0	1.1%
A_3[kCnts/Chnl]	0.801	±0.016	1.9%
τ_3[ns]	7.772	±0.017	0.2%
I_3[kCnts]	248.8	±4.8	1.9%
$Bkgr_{Dec}$[kCnts]	0.0567	±0.0004	0.6%
$Bkgr_{IRF}$[Cnts/Chnl]	3.53	±0.71	20%
$Shift_{IRF}$[ps]	-3.61	±0.24	6.5%
A_{Scatt}[kCnts]	65.00	±0.52	0.8%
τ_{AvInt}[ns]	4.867	±0.026	0.5%
τ_{AvAmp}[ns]	2.035	±0.055	2.7%
λ_{Det}[nm]	440.0000	---	---

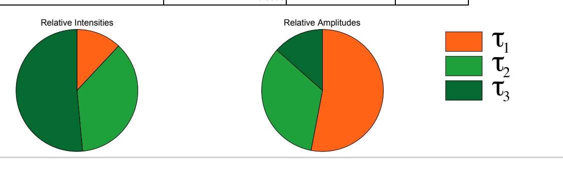

Data Set: 6 / 33

Decay: crv[6]; IRF: crv[0]

Fit

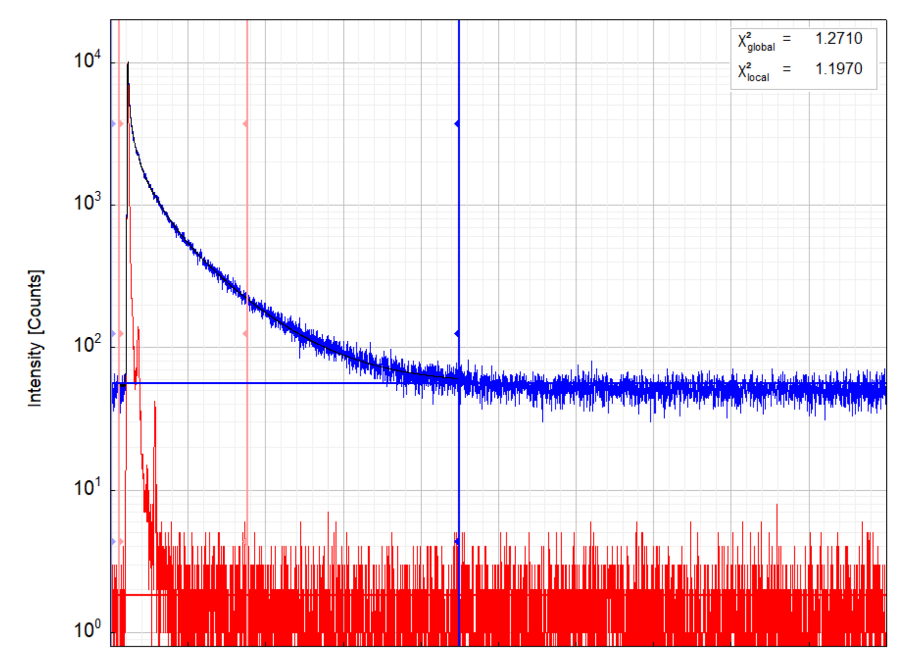

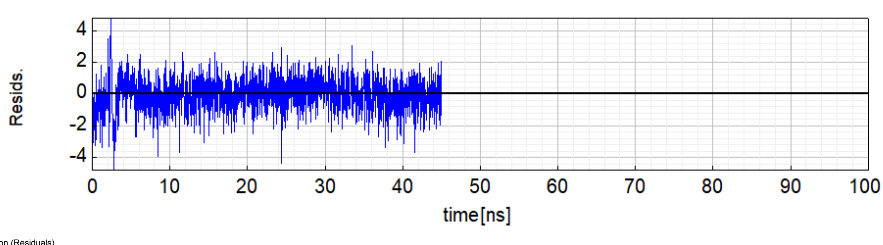

Autocorrelation (Residuals)

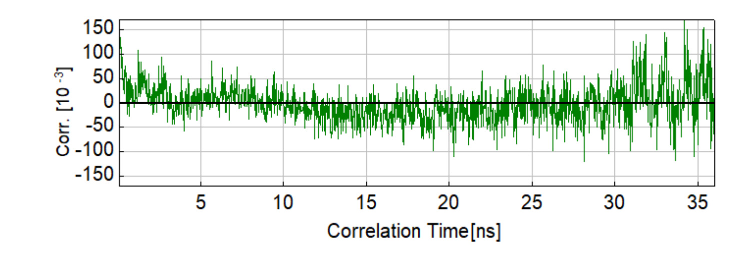

Fitted Parameters

Parameter	Value	Δ	δ
A_1[kCnts/Chnl]	3.65	±0.20	5.5%
τ_1[ns]	0.460	±0.019	4.0%
I_1[kCnts]	67.1	±3.4	5.0%
A_2[kCnts/Chnl]	1.597	±0.033	2.0%
τ_2[ns]	2.220	±0.021	0.9%
I_2[kCnts]	141.8	±2.9	2.0%
A_3[kCnts/Chnl]	1.180	±0.014	1.2%
τ_3[ns]	7.772	±0.017	0.2%
I_3[kCnts]	366.8	±3.8	1.0%
$Bkgr_{Dec}$[kCnts]	0.0557	±0.0002	0.4%
$Bkgr_{IRF}$[Cnts/Chnl]	1.82	±0.37	20%
$Shift_{IRF}$[ps]	-2.06	±0.60	29%
A_{Scatt}[kCnts]	63.7	±1.9	2.8%
τ_{AvInt}[ns]	5.553	±0.023	0.4%
τ_{AvAmp}[ns]	2.241	±0.055	2.4%
λ_{Det}[nm]	450.0000	---	---

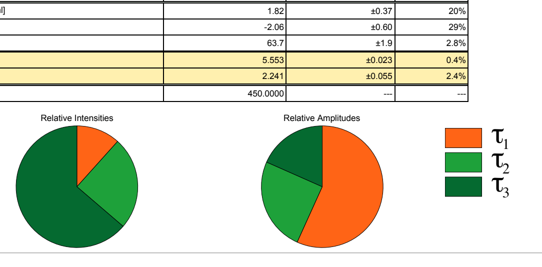

Data Set: 7 / 33

Decay: crv[7]; IRF: crv[0]

Fit

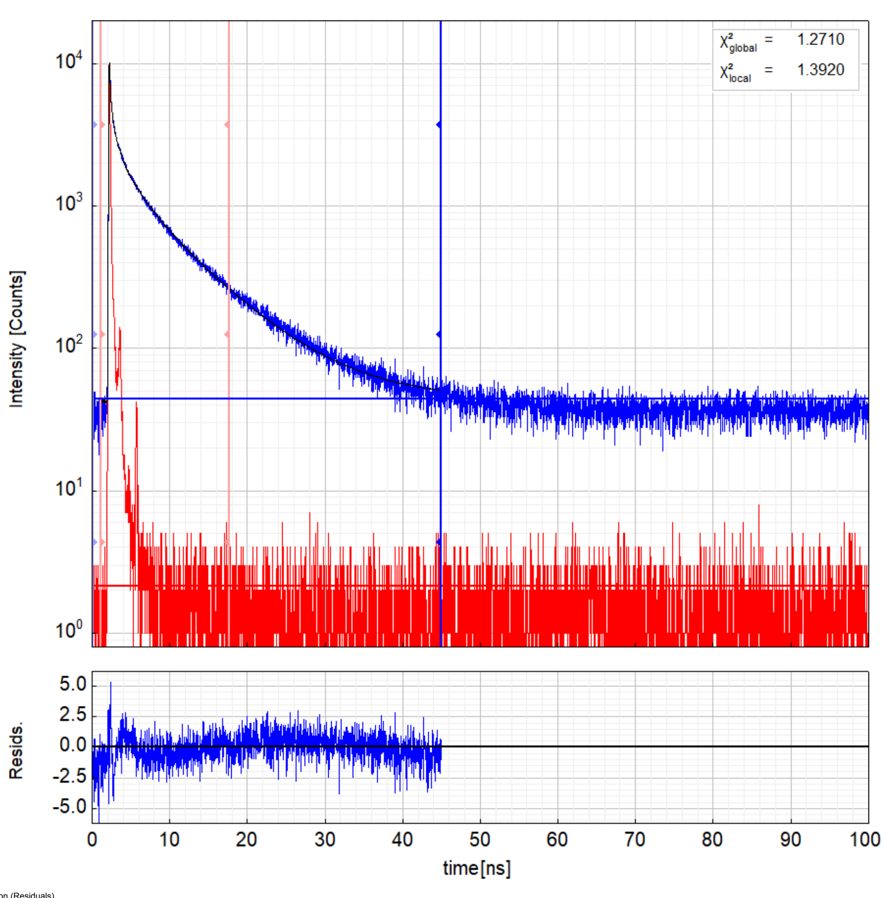

Autocorrelation (Residuals)

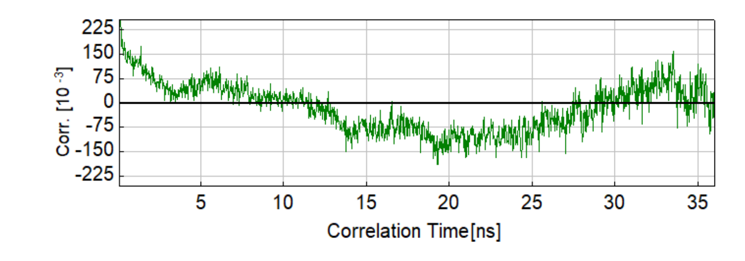

Fitted Parameters

Parameter	Value	Δ	δ
A_1[kCnts/Chnl]	4.13	±0.19	4.4%
τ_1[ns]	0.460	±0.019	4.0%
I_1[kCnts]	76.0	±2.8	3.6%
A_2[kCnts/Chnl]	1.412	±0.037	2.6%
τ_2[ns]	2.220	±0.021	0.9%
I_2[kCnts]	125.4	±3.5	2.7%
A_3[kCnts/Chnl]	1.587	±0.012	0.7%
τ_3[ns]	7.772	±0.017	0.2%
I_3[kCnts]	493.2	±4.6	0.9%
$Bkgr_{Dec}$[kCnts]	0.0444	±0.0003	0.6%
$Bkgr_{IRF}$[Cnts/Chnl]	2.13	±0.45	21%
$Shift_{IRF}$[ps]	-1.8	±1.1	61%
A_{Scatt}[kCnts]	61.2	±1.2	1.8%
τ_{AvInt}[ns]	5.970	±0.035	0.6%
τ_{AvAmp}[ns]	2.436	±0.045	1.8%
λ_{Det}[nm]	460.0000	---	---

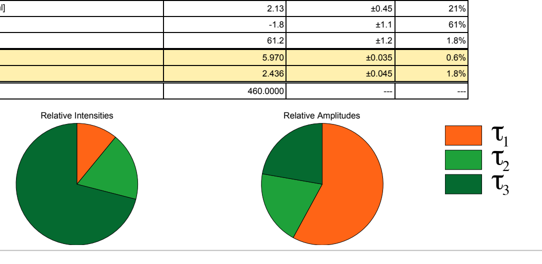

Data Set: 8 / 33

Decay: crv[8]; IRF: crv[0]

Fit

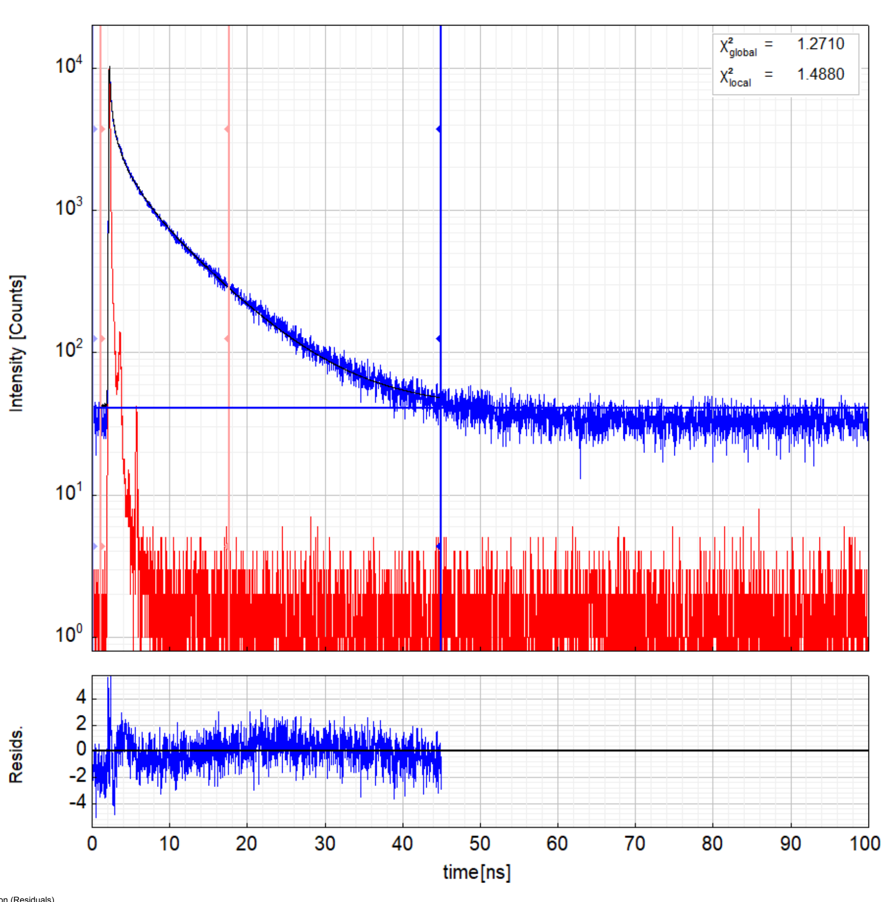

Autocorrelation (Residuals)

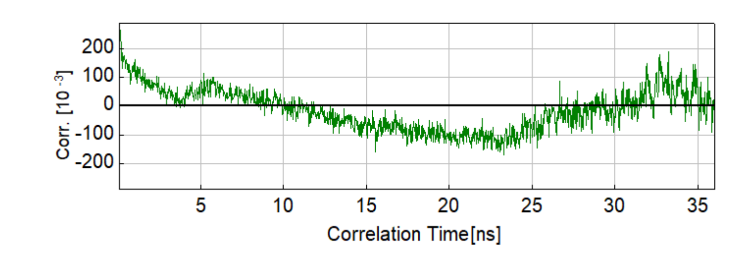

Fitted Parameters

Parameter	Value	Δ	δ
A_1[kCnts/Chnl]	4.41	±0.20	4.5%
τ_1[ns]	0.460	±0.019	4.0%
I_1[kCnts]	81.0	±3.0	3.6%
A_2[kCnts/Chnl]	1.654	±0.058	3.5%
τ_2[ns]	2.220	±0.021	0.9%
I_2[kCnts]	146.9	±4.2	2.8%
A_3[kCnts/Chnl]	1.712	±0.018	1.0%
τ_3[ns]	7.772	±0.017	0.2%
I_3[kCnts]	532.1	±5.6	1.0%
$Bkgr_{Dec}$[kCnts]	0.0411	±0.0004	0.8%
$Bkgr_{IRF}$[Cnts/Chnl]	0.41	±0.71	175%
$Shift_{IRF}$[ps]	0.9	±3.5	392%
A_{Scatt}[kCnts]	60.0	±2.0	3.3%
τ_{AvInt}[ns]	5.920	±0.035	0.6%
τ_{AvAmp}[ns]	2.446	±0.060	2.4%
λ_{Det}[nm]	470.0000	---	---

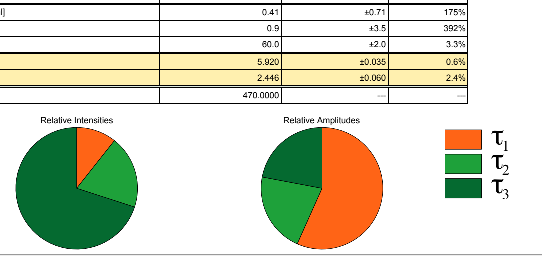

Data Set: 9 / 33

Decay: crv[9]; IRF: crv[0]

Fit

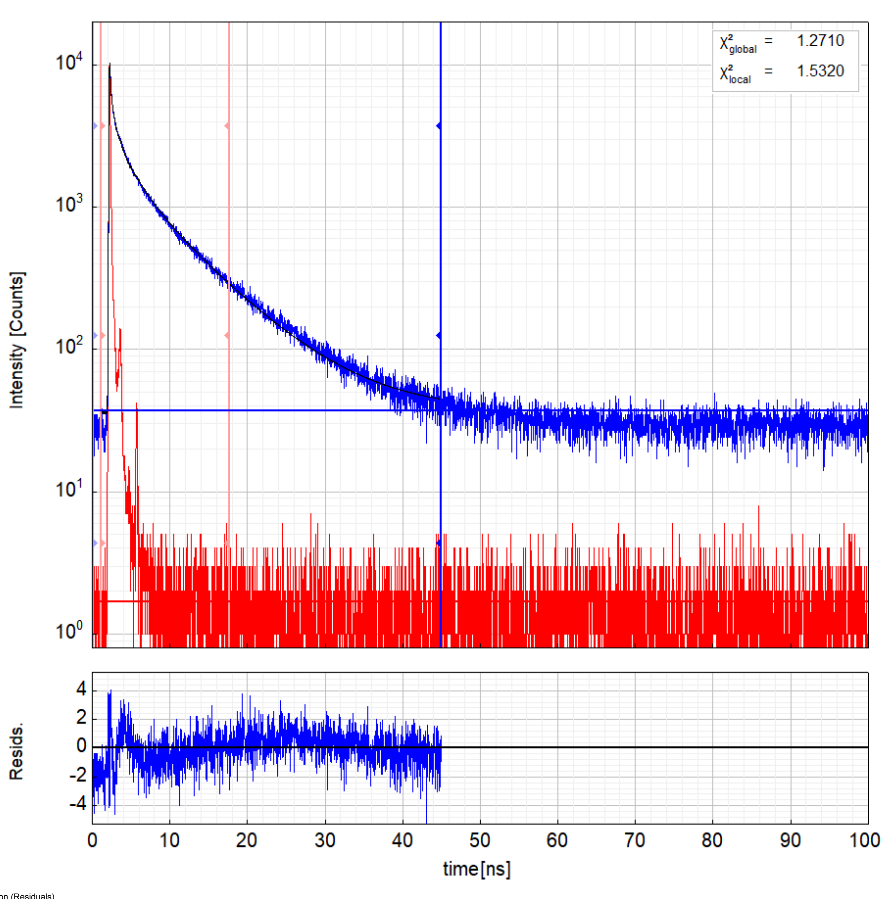

Autocorrelation (Residuals)

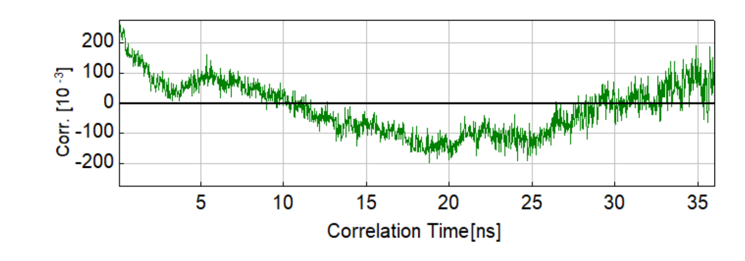

Fitted Parameters

Parameter	Value	Δ	δ
A_1[kCnts/Chnl]	4.47	±0.22	4.7%
τ_1[ns]	0.460	±0.019	4.0%
I_1[kCnts]	82.1	±1.2	1.4%
A_2[kCnts/Chnl]	1.902	±0.042	2.2%
τ_2[ns]	2.220	±0.021	0.9%
I_2[kCnts]	168.9	±3.1	1.8%
A_3[kCnts/Chnl]	1.8196	±0.0078	0.4%
τ_3[ns]	7.772	±0.017	0.2%
I_3[kCnts]	565.6	±2.7	0.5%
$Bkgr_{Dec}$[kCnts]	0.0374	±0.0004	0.9%
$Bkgr_{IRF}$[Cnts/Chnl]	1.70	±0.16	9.0%
$Shift_{IRF}$[ps]	1.4	±1.6	115%
A_{Scatt}[kCnts]	57.9	±2.0	3.3%
τ_{AvInt}[ns]	5.889	±0.021	0.4%
τ_{AvAmp}[ns]	2.495	±0.082	3.3%
λ_{Det}[nm]	480.0000	---	---

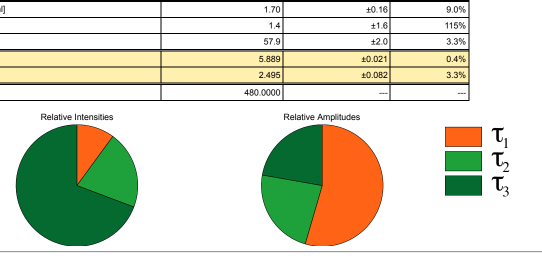

Data Set: 10 / 33

Decay: crv[10]; IRF: crv[0]

Fit

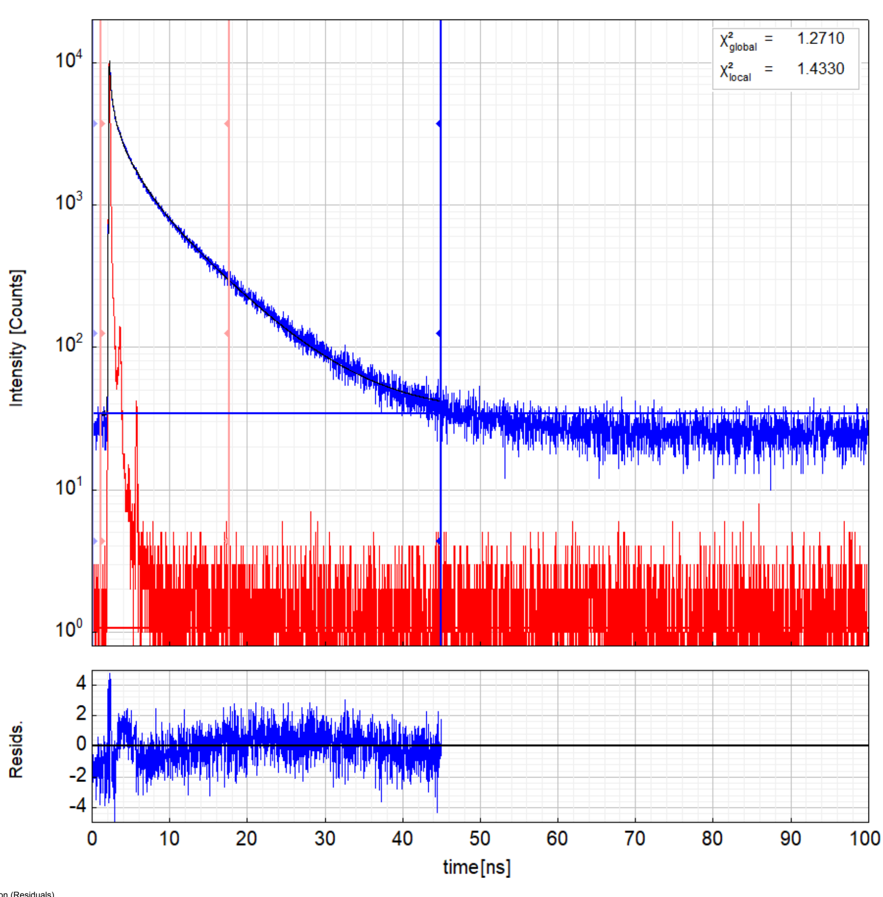

Autocorrelation (Residuals)

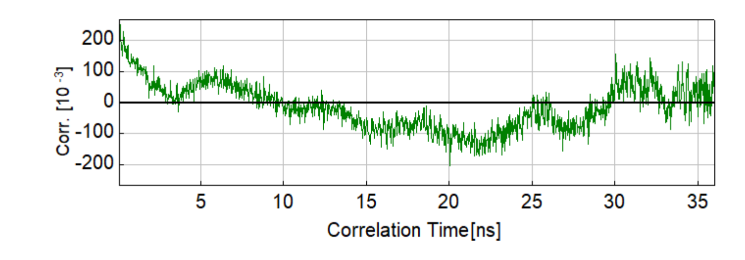

Fitted Parameters

Parameter	Value	Δ	δ
A_1[kCnts/Chnl]	4.35	±0.15	3.4%
τ_1[ns]	0.460	±0.019	4.0%
I_1[kCnts]	79.9	±2.3	2.8%
A_2[kCnts/Chnl]	2.272	±0.065	2.8%
τ_2[ns]	2.220	±0.021	0.9%
I_2[kCnts]	201.8	±4.8	2.4%
A_3[kCnts/Chnl]	1.860	±0.012	0.6%
τ_3[ns]	7.772	±0.017	0.2%
I_3[kCnts]	578.0	±2.9	0.5%
$Bkgr_{Dec}$[kCnts]	0.0342	±0.0003	0.7%
$Bkgr_{IRF}$[Cnts/Chnl]	1.06	±0.37	35%
$Shift_{IRF}$[ps]	2.33	±0.94	40%
A_{Scatt}[kCnts]	56.2	±1.2	2.0%
τ_{AvInt}[ns]	5.789	±0.029	0.5%
τ_{AvAmp}[ns]	2.536	±0.050	2.0%
λ_{Det}[nm]	490.0000	---	---

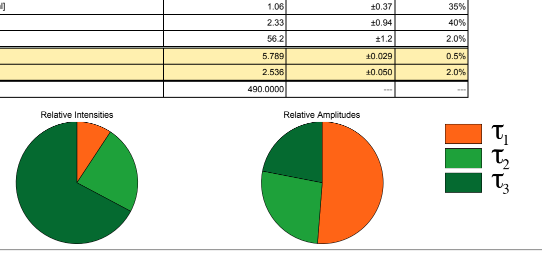

Data Set: 11 / 33

Decay: crv[11]; IRF: crv[0]

Fit

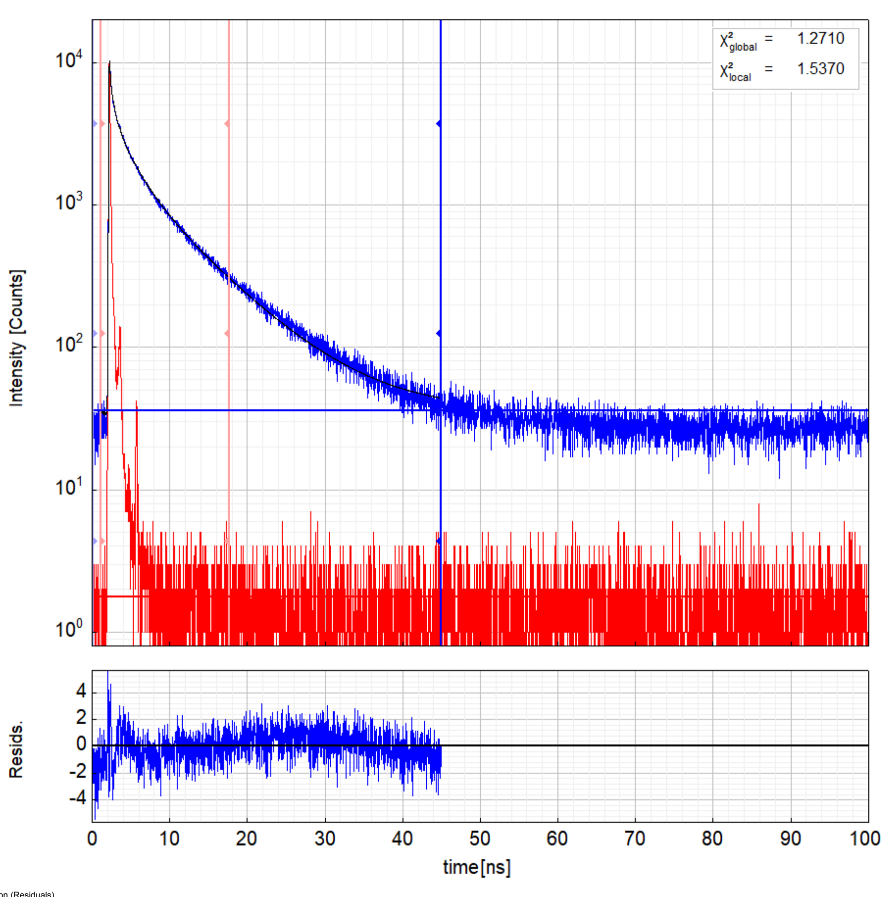

Autocorrelation (Residuals)

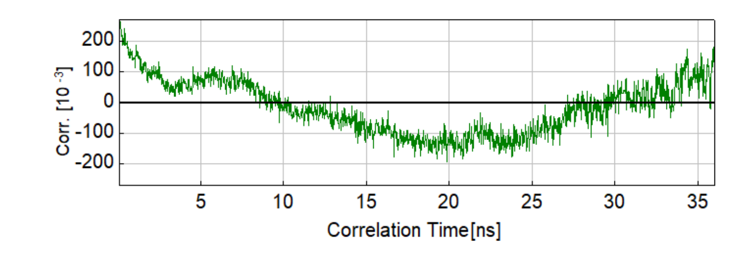

Fitted Parameters

Parameter	Value	Δ	δ
A_1[kCnts/Chnl]	4.69	±0.15	3.1%
τ_1[ns]	0.460	±0.019	4.0%
I_1[kCnts]	86.2	±2.9	3.3%
A_2[kCnts/Chnl]	2.590	±0.042	1.6%
τ_2[ns]	2.220	±0.021	0.9%
I_2[kCnts]	230.0	±3.3	1.4%
A_3[kCnts/Chnl]	1.962	±0.020	1.0%
τ_3[ns]	7.772	±0.017	0.2%
I_3[kCnts]	609.7	±5.6	0.9%
$Bkgr_{Dec}$[kCnts]	0.0361	±0.0003	0.8%
$Bkgr_{IRF}$[Cnts/Chnl]	1.76	±0.40	22%
$Shift_{IRF}$[ps]	0.5	±1.2	256%
A_{Scatt}[kCnts]	52.2	±1.9	3.6%
τ_{AvInt}[ns]	5.712	±0.031	0.5%
τ_{AvAmp}[ns]	2.507	±0.057	2.3%
λ_{Det}[nm]	500.0000	---	---

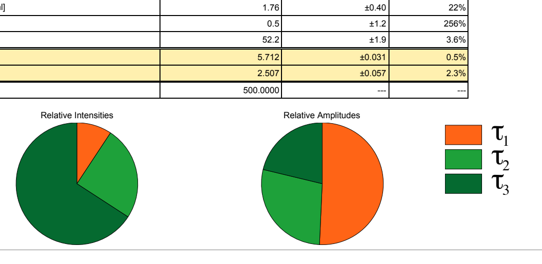

Data Set: 12 / 33

Decay: crv[12]; IRF: crv[0]

Fit

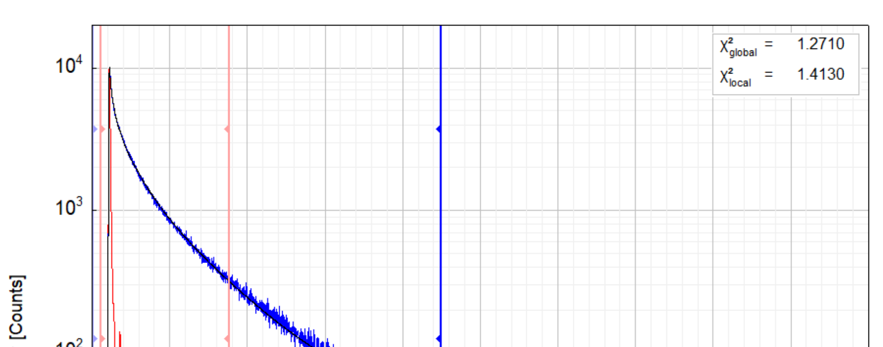

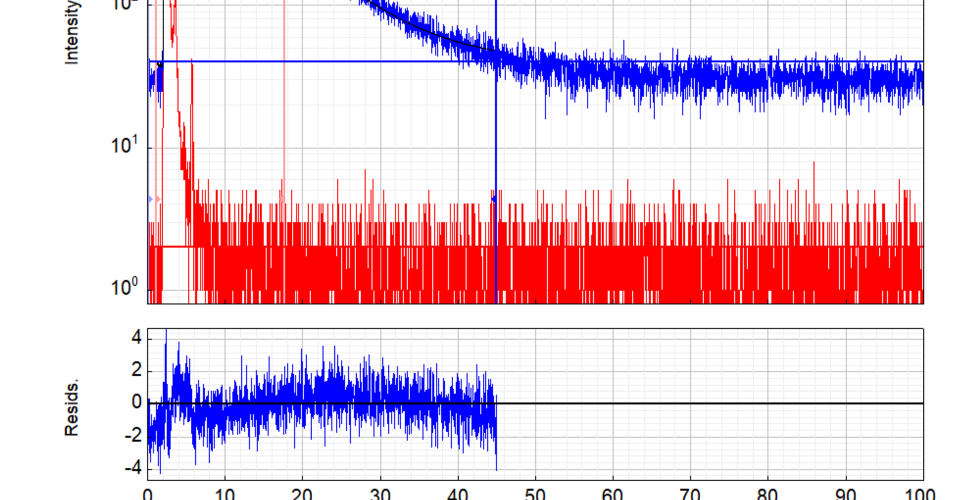

Autocorrelation (Residuals)

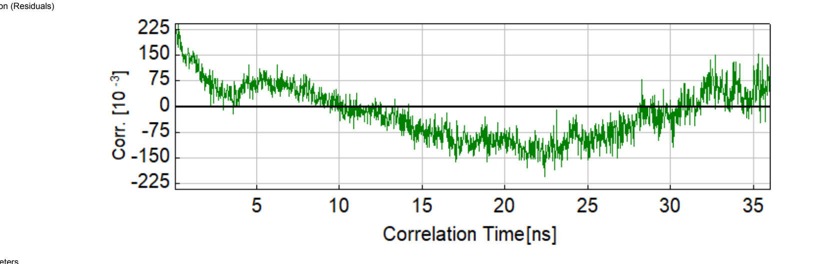

Fitted Parameters

Parameter	Value	Δ	δ
A_1[kCnts/Chnl]	4.802	±0.099	2.1%
τ_1[ns]	0.460	±0.019	4.0%
I_1[kCnts]	88.4	±3.2	3.6%
A_2[kCnts/Chnl]	2.846	±0.044	1.5%
τ_2[ns]	2.220	±0.021	0.9%
I_2[kCnts]	252.8	±3.1	1.2%
A_3[kCnts/Chnl]	2.012	±0.020	1.0%
τ_3[ns]	7.772	±0.017	0.2%
I_3[kCnts]	625.3	±5.3	0.8%
$Bkgr_{Dec}$[kCnts]	0.0399	±0.0003	0.6%
$Bkgr_{IRF}$[Cnts/Chnl]	2.03	±0.38	19%
$Shift_{IRF}$[ps]	-1.2	±3.4	302%
A_{Scatt}[kCnts]	49.1	±2.9	5.7%
τ_{AvInt}[ns]	5.652	±0.018	0.3%
τ_{AvAmp}[ns]	2.501	±0.068	2.7%
λ_{Det}[nm]	510.0000	---	---

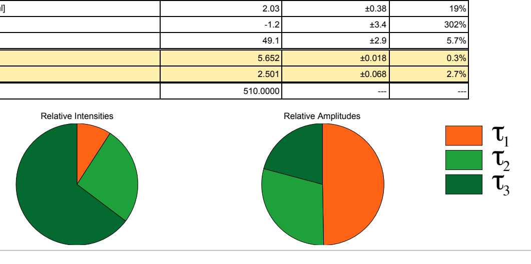

Data Set: 13 / 33

Decay: crv[13]; IRF: crv[0]

Fit

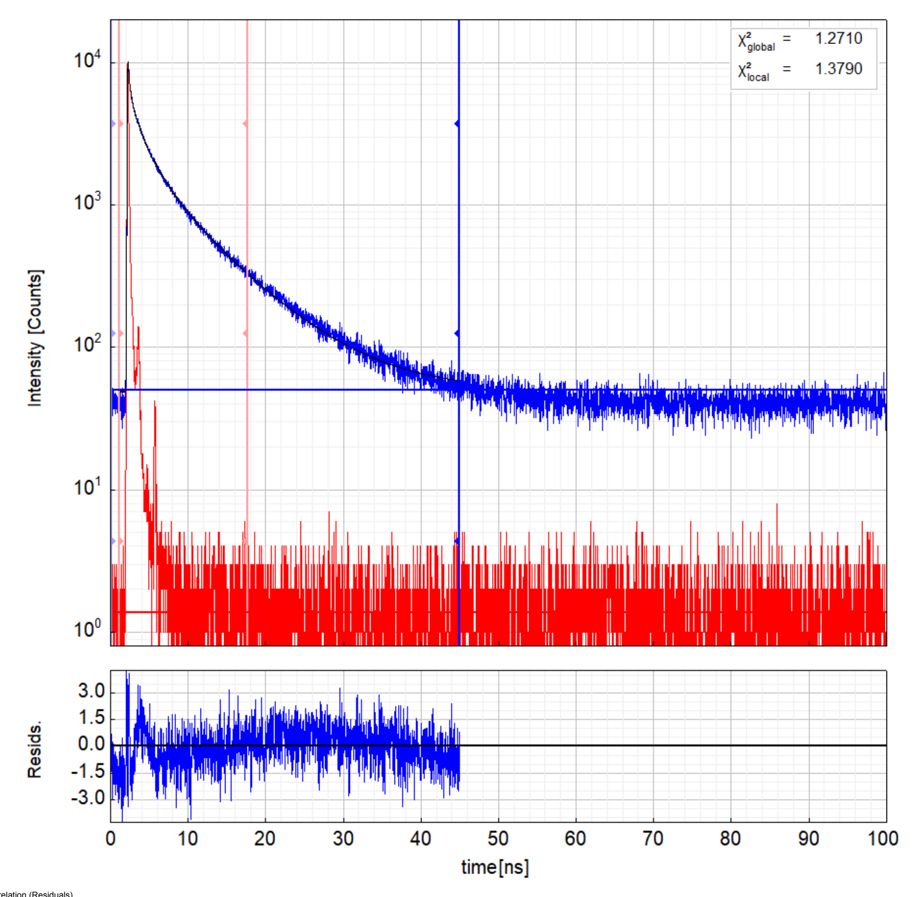

Autocorrelation (Residuals)

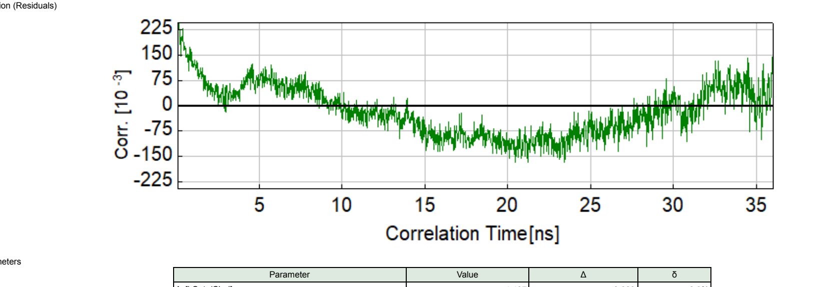

Fitted Parameters

Parameter	Value	Δ	δ
A_1[kCnts/Chnl]	4.437	±0.089	2.0%
τ_1[ns]	0.460	±0.019	4.0%
I_1[kCnts]	81.6	±2.7	3.2%
A_2[kCnts/Chnl]	3.045	±0.098	3.2%
τ_2[ns]	2.220	±0.021	0.9%
I_2[kCnts]	270.4	±5.2	1.9%
A_3[kCnts/Chnl]	2.005	±0.016	0.8%
τ_3[ns]	7.772	±0.017	0.2%
I_3[kCnts]	623.1	±4.9	0.8%
$Bkgr_{Dec}$[kCnts]	0.0501	±0.0003	0.6%
$Bkgr_{IRF}$[Cnts/Chnl]	1.38	±0.39	28%
$Shift_{IRF}$[ps]	2.3	±1.9	84%
A_{Scatt}[kCnts]	50.1	±1.9	3.8%
τ_{AvInt}[ns]	5.620	±0.023	0.4%
τ_{AvAmp}[ns]	2.570	±0.039	1.5%
λ_{Det}[nm]	520.0000	---	---

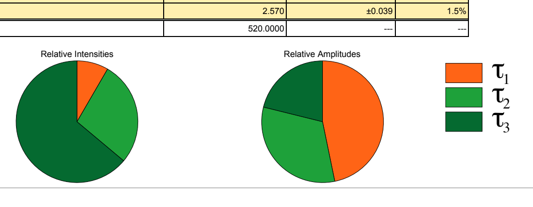

Data Set: 14 / 33

Decay: crv[14]; IRF: crv[0]

Fit

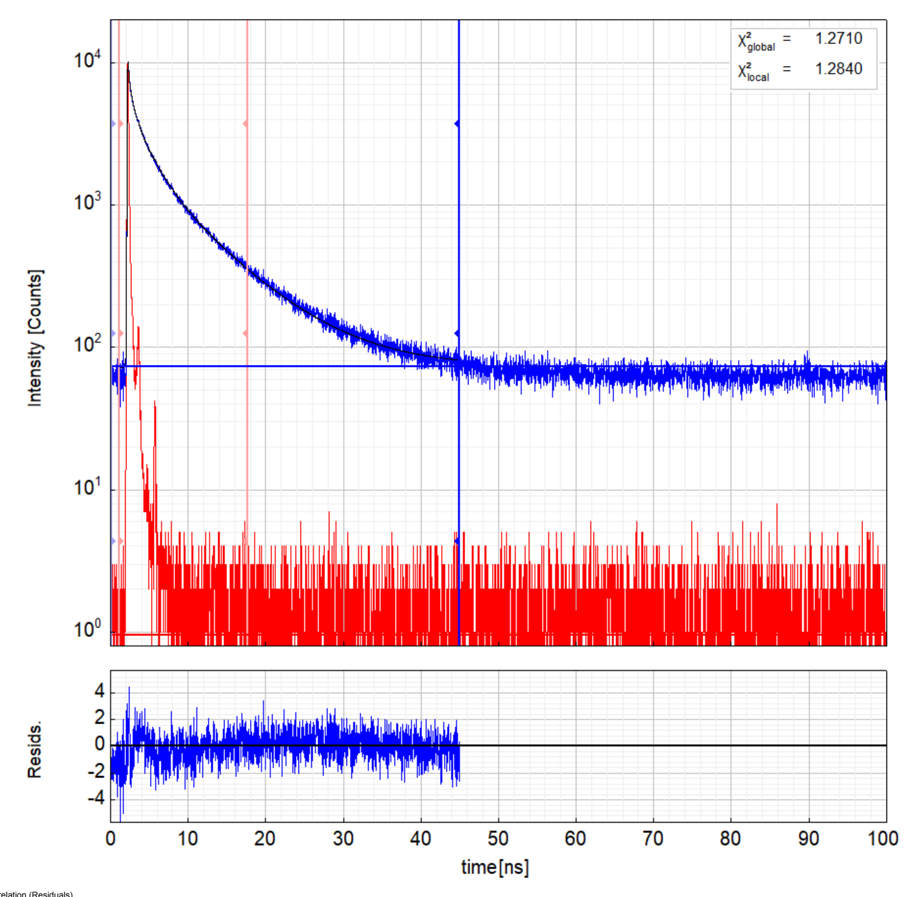

Autocorrelation (Residuals)

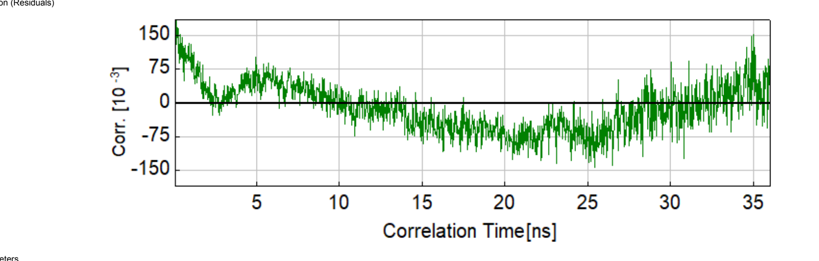

Fitted Parameters

Parameter	Value	Δ	δ
A_1[kCnts/Chnl]	4.52	±0.13	2.7%
τ_1[ns]	0.460	±0.019	4.0%
I_1[kCnts]	83.0	±3.5	4.1%
A_2[kCnts/Chnl]	3.184	±0.058	1.8%
τ_2[ns]	2.220	±0.021	0.9%
I_2[kCnts]	282.8	±4.0	1.4%
A_3[kCnts/Chnl]	1.995	±0.025	1.2%
τ_3[ns]	7.772	±0.017	0.2%
I_3[kCnts]	620.1	±6.4	1.0%
$Bkgr_{Dec}$[kCnts]	0.0730	±0.0005	0.6%
$Bkgr_{IRF}$[Cnts/Chnl]	0.96	±0.30	31%
$Shift_{IRF}$[ps]	4.5	±1.2	26%
A_{Scatt}[kCnts]	48.9	±1.3	2.6%
τ_{AvInt}[ns]	5.564	±0.023	0.4%
τ_{AvAmp}[ns]	2.544	±0.040	1.6%
λ_{Det}[nm]	530.0000	---	---

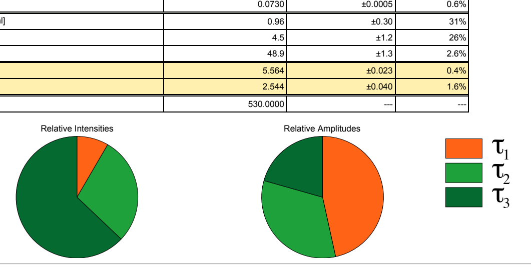

Data Set: 15 / 33

Decay: crv[15]; IRF: crv[0]

Fit

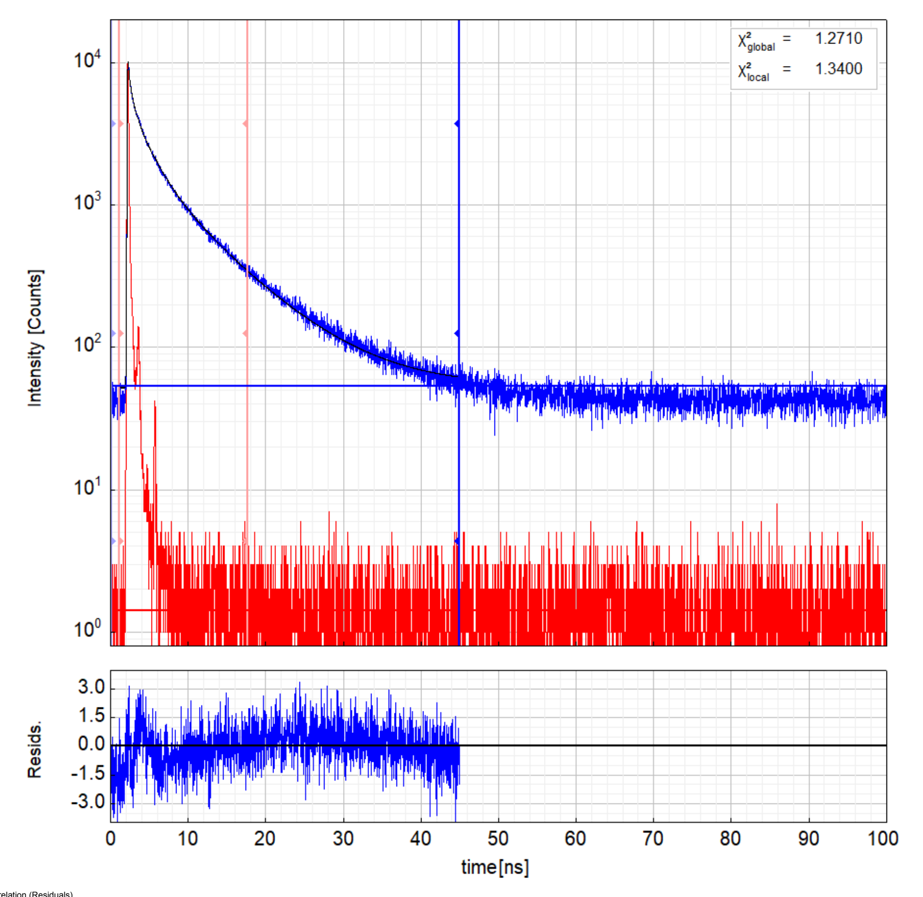

Autocorrelation (Residuals)

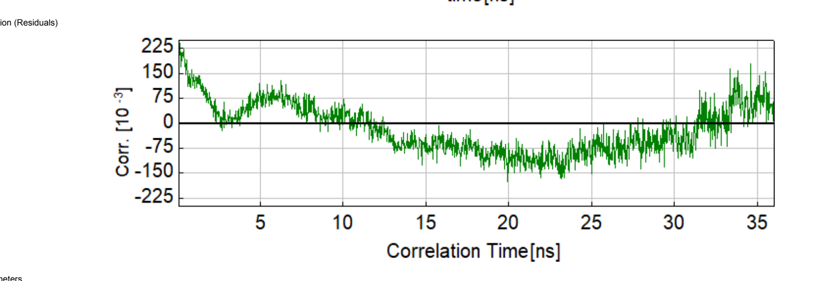

Fitted Parameters

Parameter	Value	Δ	δ
A_1[kCnts/Chnl]	4.72	±0.16	3.2%
τ_1[ns]	0.460	±0.019	4.0%
I_1[kCnts]	86.7	±1.9	2.1%
A_2[kCnts/Chnl]	3.411	±0.066	1.9%
τ_2[ns]	2.220	±0.021	0.9%
I_2[kCnts]	302.9	±4.4	1.4%
A_3[kCnts/Chnl]	2.080	±0.023	1.1%
τ_3[ns]	7.772	±0.017	0.2%
I_3[kCnts]	646.5	±5.7	0.9%
$Bkgr_{Dec}$[kCnts]	0.0533	±0.0003	0.5%
$Bkgr_{IRF}$[Cnts/Chnl]	1.42	±0.37	26%
$Shift_{IRF}$[ps]	3.1	±1.3	40%
A_{Scatt}[kCnts]	47.4	±1.1	2.2%
τ_{AvInt}[ns]	5.537	±0.014	0.2%
τ_{AvAmp}[ns]	2.539	±0.039	1.5%
λ_{Det}[nm]	540.0000	---	---

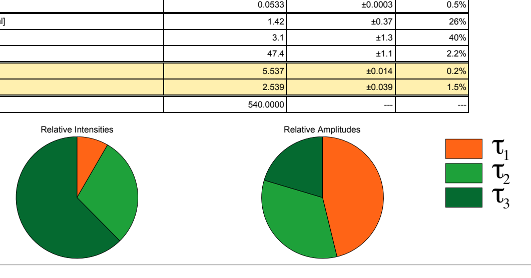

Data Set: 16 / 33

Decay: crv[16]; IRF: crv[0]

Fit

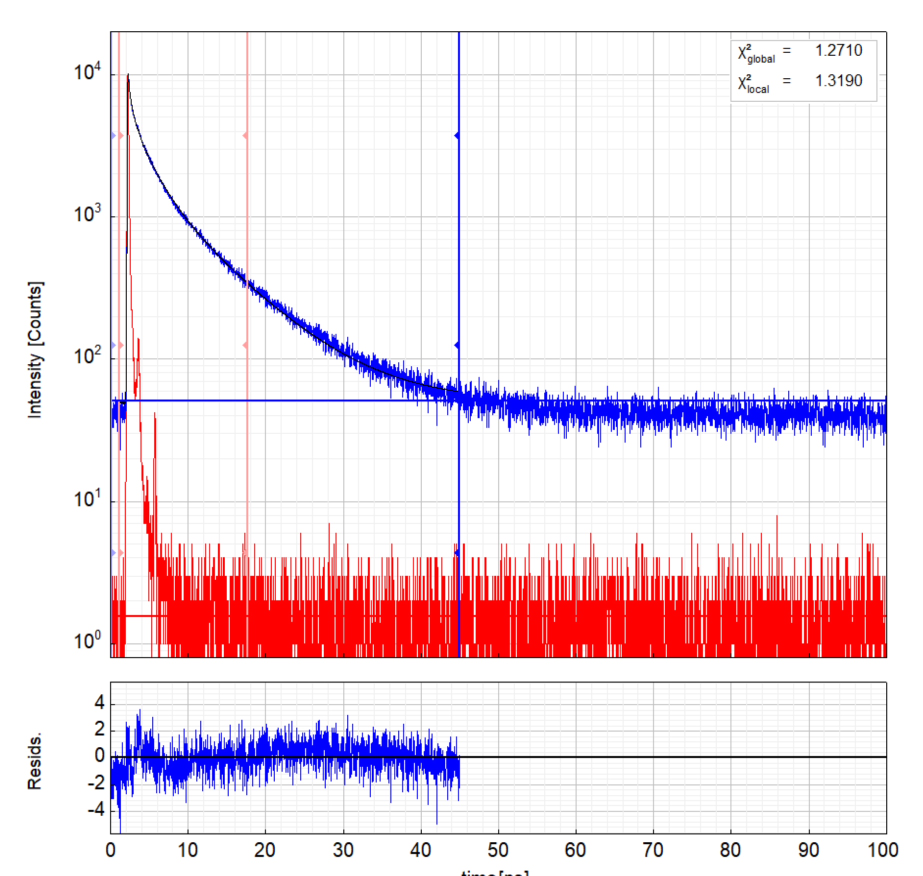

Autocorrelation (Residuals)

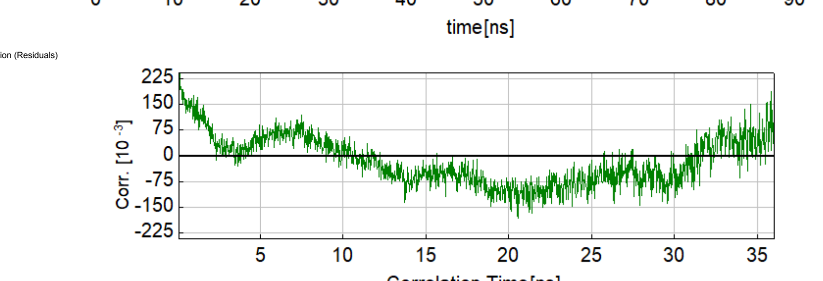

Fitted Parameters

Parameter	Value	Δ	δ
A_1[kCnts/Chnl]	4.504	±0.073	1.6%
τ_1[ns]	0.460	±0.019	4.0%
I_1[kCnts]	82.9	±3.1	3.6%
A_2[kCnts/Chnl]	3.487	±0.057	1.6%
τ_2[ns]	2.220	±0.021	0.9%
I_2[kCnts]	309.6	±3.4	1.1%
A_3[kCnts/Chnl]	2.090	±0.013	0.6%
τ_3[ns]	7.772	±0.017	0.2%
I_3[kCnts]	649.5	±4.1	0.6%
$Bkgr_{Dec}$[kCnts]	0.0506	±0.0005	0.9%
$Bkgr_{IRF}$[Cnts/Chnl]	1.55	±0.65	41%
$Shift_{IRF}$[ps]	4.9	±1.2	24%
A_{Scatt}[kCnts]	47.5	±1.1	2.3%
τ_{AvInt}[ns]	5.541	±0.030	0.5%
τ_{AvAmp}[ns]	2.585	±0.031	1.2%
λ_{Det}[nm]	550.0000	---	---

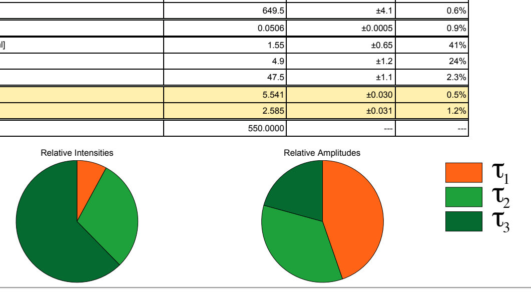

Data Set: 17 / 33

Decay: crv[17]; IRF: crv[0]

Fit

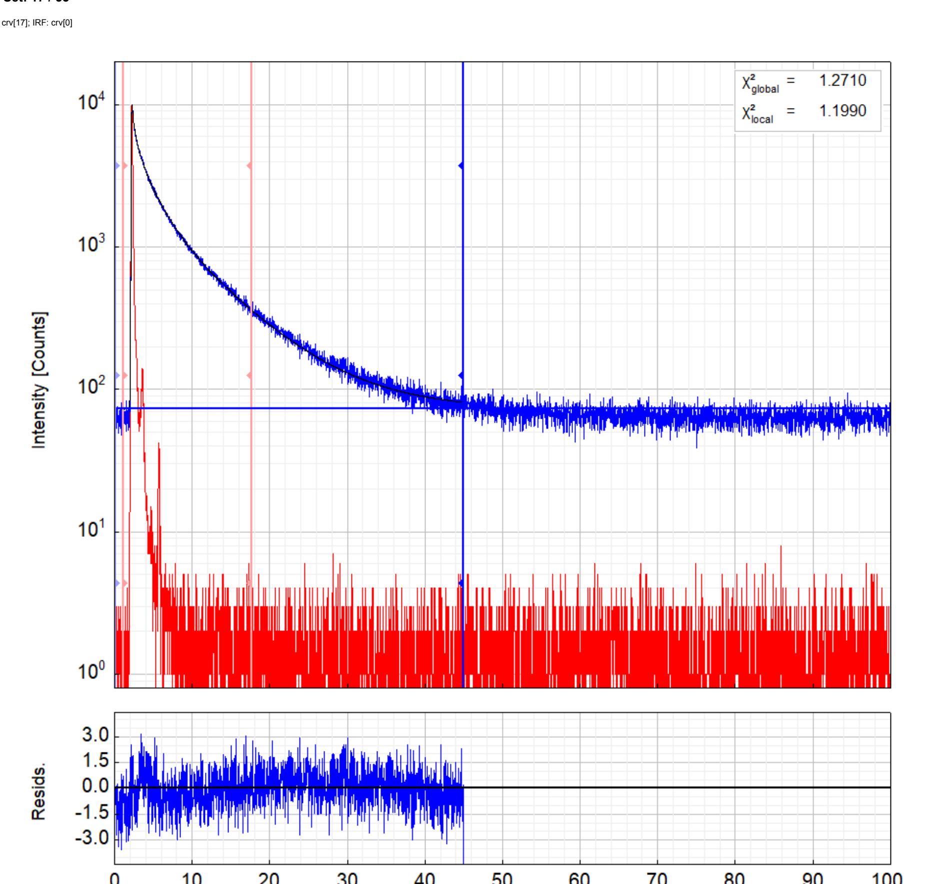

Autocorrelation (Residuals)

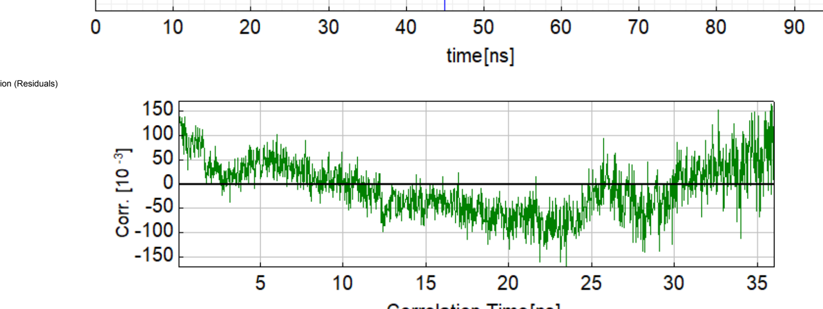

Fitted Parameters

Parameter	Value	Δ	δ
A_1[kCnts/Chnl]	4.468	±0.060	1.3%
τ_1[ns]	0.460	±0.019	4.0%
I_1[kCnts]	82.2	±3.7	4.4%
A_2[kCnts/Chnl]	3.523	±0.072	2.0%
τ_2[ns]	2.220	±0.021	0.9%
I_2[kCnts]	312.9	±4.7	1.5%
A_3[kCnts/Chnl]	2.022	±0.019	0.9%
τ_3[ns]	7.772	±0.017	0.2%
I_3[kCnts]	628.5	±6.0	0.9%
$Bkgr_{Dec}$[kCnts]	0.0728	±0.0005	0.7%
$Bkgr_{IRF}$[Cnts/Chnl]	0.64	±0.56	88%
$Shift_{IRF}$[ps]	4.8	±1.1	23%
A_{Scatt}[kCnts]	46.7	±1.1	2.3%
τ_{AvInt}[ns]	5.488	±0.021	0.4%
τ_{AvAmp}[ns]	2.556	±0.033	1.3%
λ_{Det}[nm]	560.0000	---	---

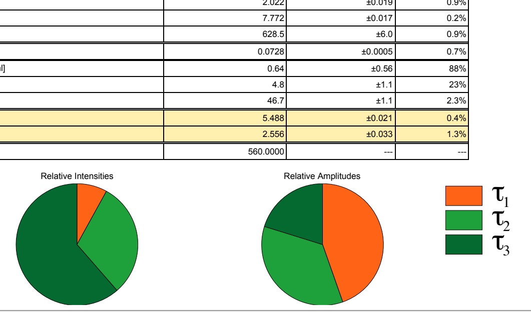

Data Set: 18 / 33

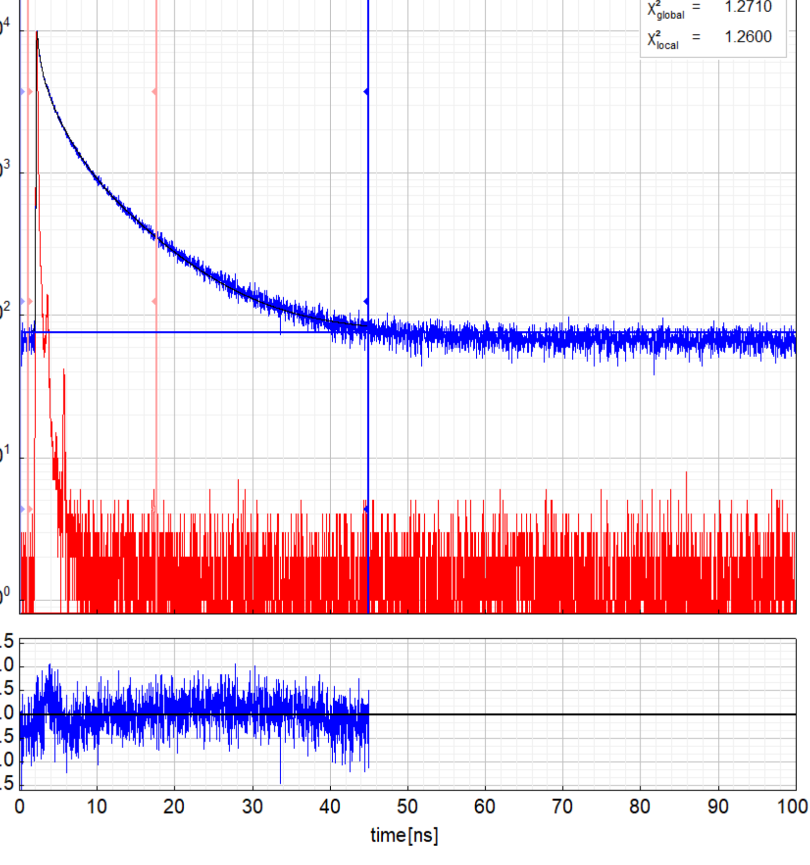

Autocorrelation (Residuals)

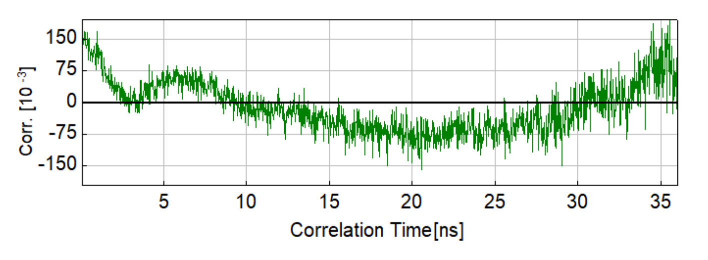

Fitted Parameters

Parameter	Value	Δ	δ
A_1[kCnts/Chnl]	4.56	±0.13	2.7%
τ_1[ns]	0.460	±0.019	4.0%
I_1[kCnts]	83.8	±3.2	3.7%
A_2[kCnts/Chnl]	3.444	±0.053	1.5%
τ_2[ns]	2.220	±0.021	0.9%
I_2[kCnts]	305.8	±2.6	0.8%
A_3[kCnts/Chnl]	1.939	±0.017	0.9%
τ_3[ns]	7.772	±0.017	0.2%
I_3[kCnts]	602.5	±3.8	0.6%
$Bkgr_{Dec}$[kCnts]	0.0760	±0.0005	0.6%
$Bkgr_{IRF}$[Cnts/Chnl]	0.81	±0.32	39%
$Shift_{IRF}$[ps]	5.0	±1.5	29%
A_{Scatt}[kCnts]	45.6	±1.1	2.4%
τ_{AvInt}[ns]	5.444	±0.014	0.3%
τ_{AvAmp}[ns]	2.497	±0.032	1.2%
λ_{Det}[nm]	570.0000	---	---

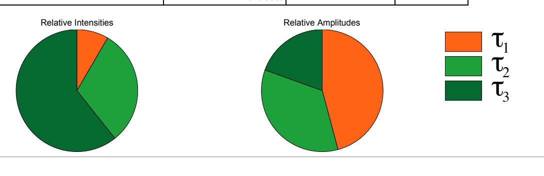

Data Set: 19 / 33

Decay: crv[19]; IRF: crv[0]

Fit

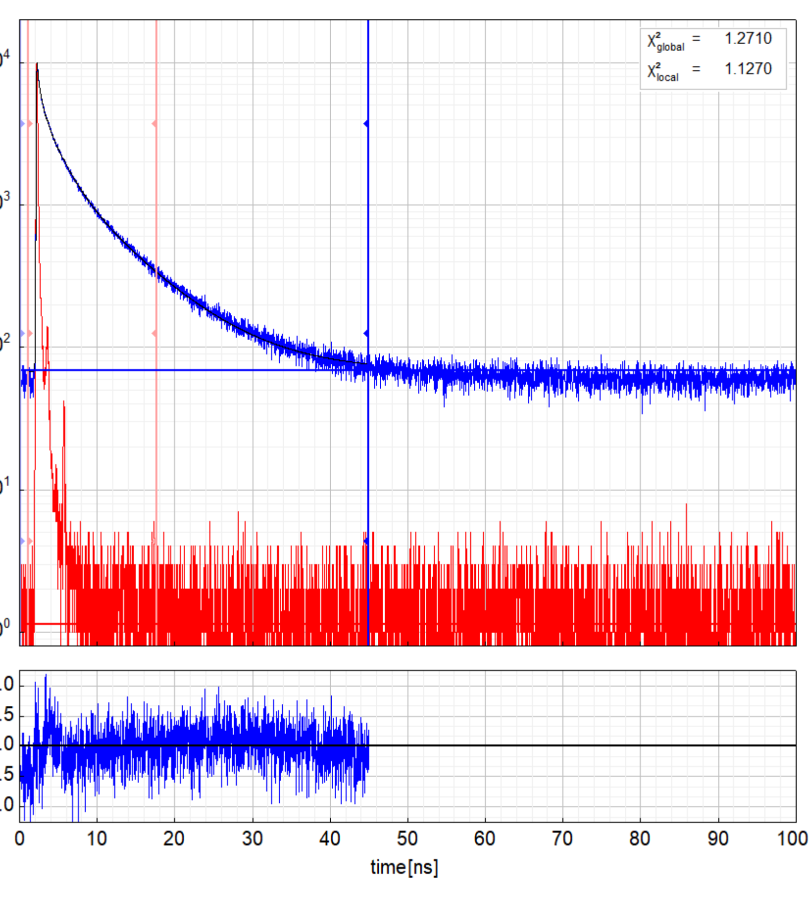

Autocorrelation (Residuals)

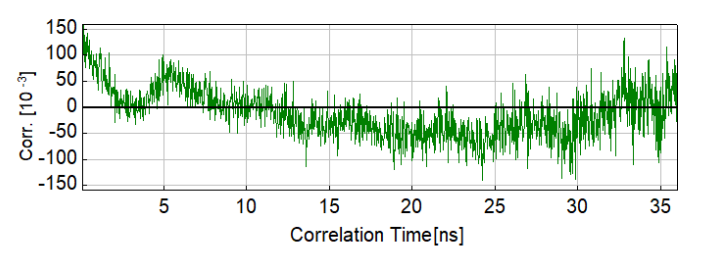

Fitted Parameters

Parameter	Value	Δ	δ
A_1[kCnts/Chnl]	4.60	±0.13	2.6%
τ_1[ns]	0.460	±0.019	4.0%
I_1[kCnts]	84.6	±3.0	3.5%
A_2[kCnts/Chnl]	3.502	±0.054	1.5%
τ_2[ns]	2.220	±0.021	0.9%
I_2[kCnts]	310.9	±4.2	1.3%
A_3[kCnts/Chnl]	1.906	±0.030	1.5%
τ_3[ns]	7.772	±0.017	0.2%
I_3[kCnts]	592.3	±9.9	1.7%
$Bkgr_{Dec}$[kCnts]	0.0684	±0.0003	0.4%
$Bkgr_{IRF}$[Cnts/Chnl]	1.15	±0.80	70%
$Shift_{IRF}$[ps]	5.1	±1.1	21%
A_{Scatt}[kCnts]	46.7	±1.8	3.8%
τ_{AvInt}[ns]	5.398	±0.027	0.5%
τ_{AvAmp}[ns]	2.469	±0.036	1.4%
λ_{Det}[nm]	580.0000	---	---

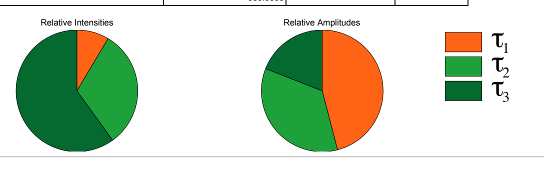

Data Set: 20 / 33

Decay: crv[20]; IRF: crv[0]

Fit

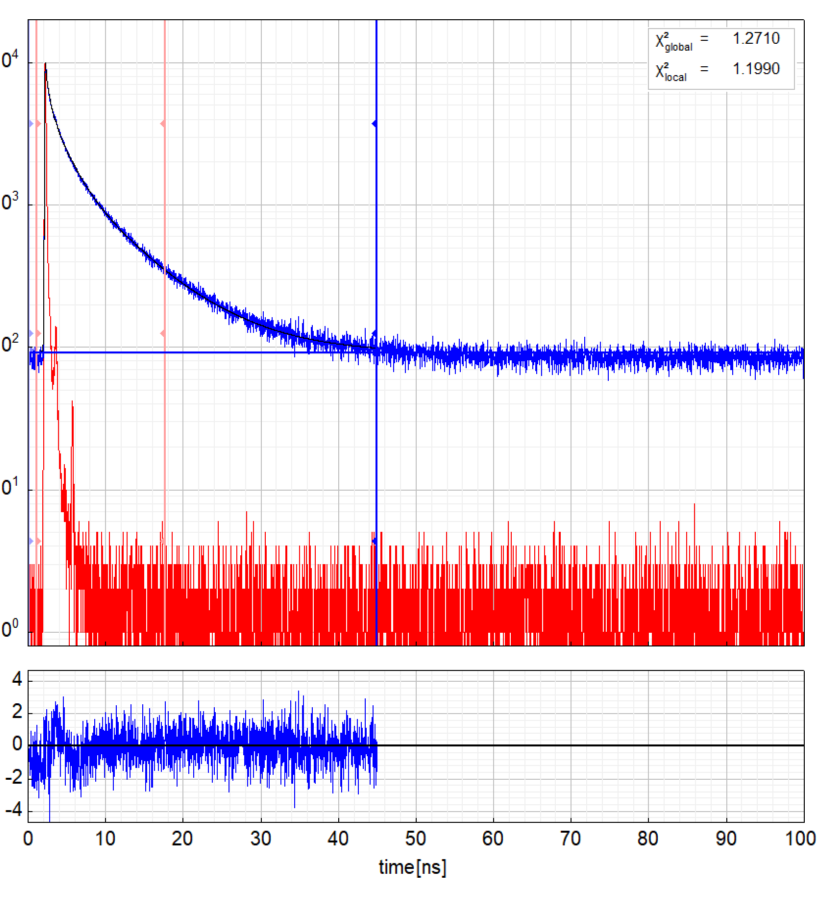

Autocorrelation (Residuals)

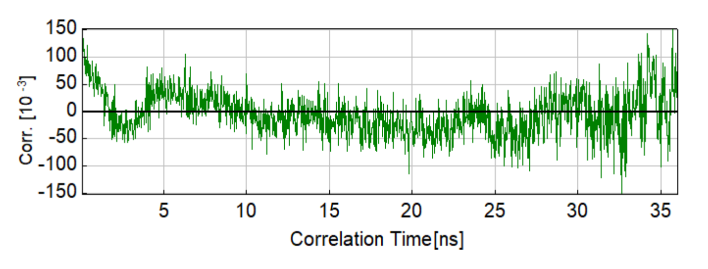

Fitted Parameters

Parameter	Value	Δ	δ
A_1[kCnts/Chnl]	4.743	±0.072	1.5%
τ_1[ns]	0.460	±0.019	4.0%
I_1[kCnts]	87.3	±3.5	4.0%
A_2[kCnts/Chnl]	3.479	±0.096	2.7%
τ_2[ns]	2.220	±0.021	0.9%
I_2[kCnts]	308.9	±6.0	1.9%
A_3[kCnts/Chnl]	1.787	±0.020	1.1%
τ_3[ns]	7.772	±0.017	0.2%
I_3[kCnts]	555.5	±4.7	0.8%
$Bkgr_{Dec}$[kCnts]	0.0916	±0.0004	0.4%
$Bkgr_{IRF}$[Cnts/Chnl]	0.15	±0.29	198%
$Shift_{IRF}$[ps]	6.49	±0.94	14%
A_{Scatt}[kCnts]	46.2	±1.3	2.7%
τ_{AvInt}[ns]	5.299	±0.015	0.3%
τ_{AvAmp}[ns]	2.377	±0.029	1.2%
λ_{Det}[nm]	590.0000	---	---

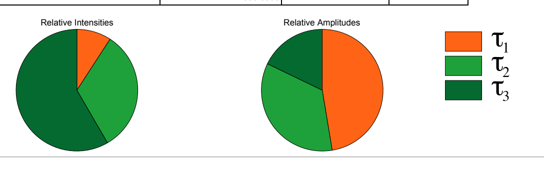

Data Set: 21 / 33

Decay: crv[21]; IRF: crv[0]

Fit

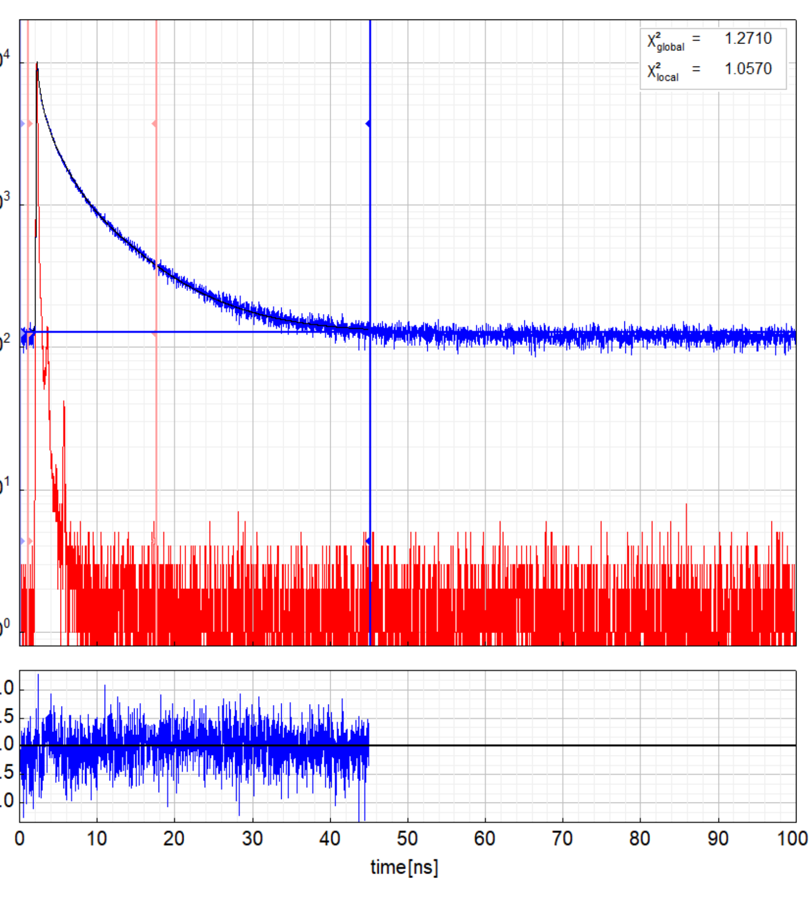

Autocorrelation (Residuals)

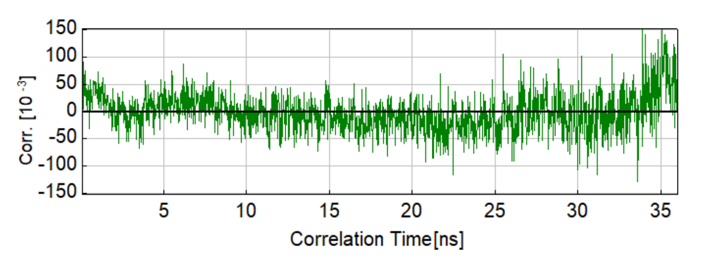

Fitted Parameters

Parameter	Value	Δ	δ
A_1[kCnts/Chnl]	4.870	±0.080	1.6%
τ_1[ns]	0.460	±0.019	4.0%
I_1[kCnts]	89.6	±4.6	5.1%
A_2[kCnts/Chnl]	3.476	±0.096	2.8%
τ_2[ns]	2.220	±0.021	0.9%
I_2[kCnts]	308.7	±6.7	2.2%
A_3[kCnts/Chnl]	1.688	±0.016	0.9%
τ_3[ns]	7.772	±0.017	0.2%
I_3[kCnts]	524.5	±5.8	1.1%
$Bkgr_{Dec}$[kCnts]	0.1270	±0.0005	0.4%
$Bkgr_{IRF}$[Cnts/Chnl]	-0.60	±0.51	85%
$Shift_{IRF}$[ps]	7.91	±0.56	7.1%
A_{Scatt}[kCnts]	47.9	±1.1	2.1%
τ_{AvInt}[ns]	5.205	±0.028	0.5%
τ_{AvAmp}[ns]	2.300	±0.033	1.4%
λ_{Det}[nm]	600.0000	---	---

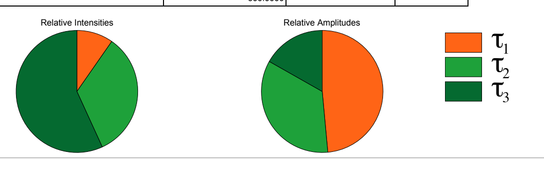

Data Set: 22 / 33

Decay: crv[22]; IRF: crv[0]

Fit

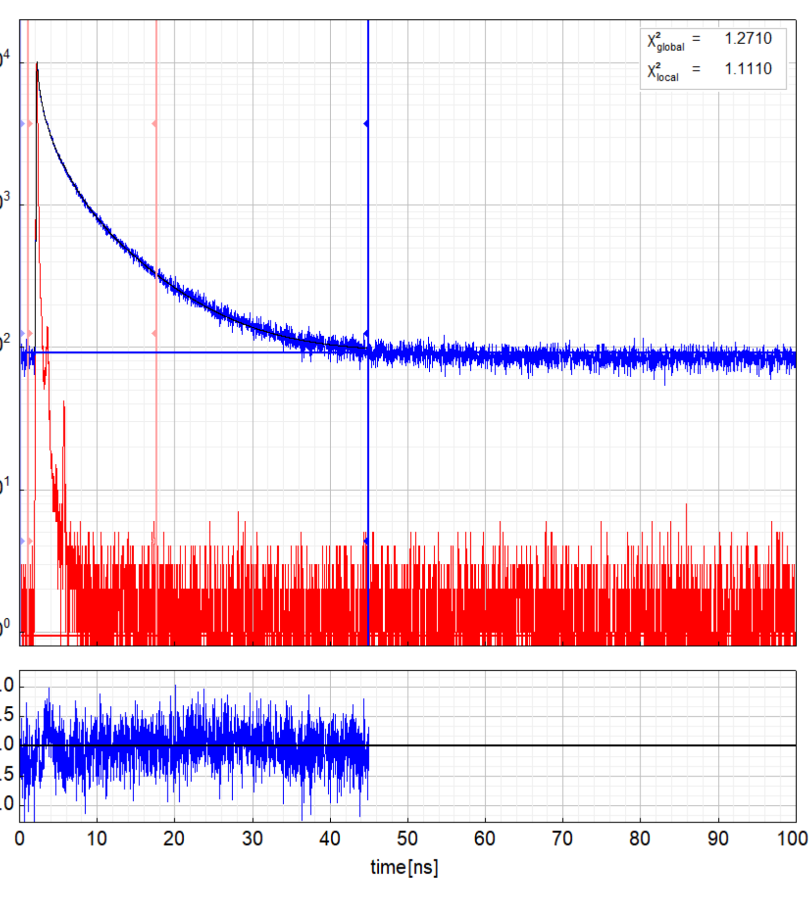

Autocorrelation (Residuals)

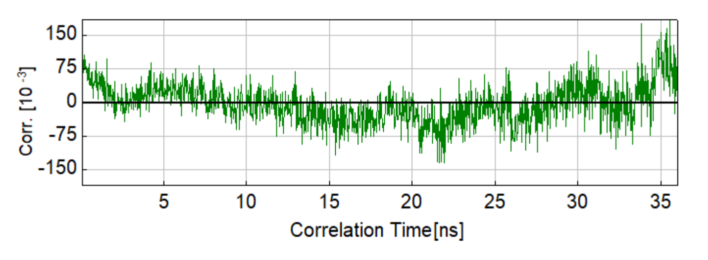

Fitted Parameters

Parameter	Value	Δ	δ
A_1[kCnts/Chnl]	4.95	±0.11	2.1%
τ_1[ns]	0.460	±0.019	4.0%
I_1[kCnts]	90.9	±3.1	3.4%
A_2[kCnts/Chnl]	3.296	±0.076	2.3%
τ_2[ns]	2.220	±0.021	0.9%
I_2[kCnts]	292.7	±4.3	1.4%
A_3[kCnts/Chnl]	1.643	±0.022	1.3%
τ_3[ns]	7.772	±0.017	0.2%
I_3[kCnts]	510.6	±6.6	1.3%
$Bkgr_{Dec}$[kCnts]	0.0916	±0.0004	0.4%
$Bkgr_{IRF}$[Cnts/Chnl]	0.94	±0.35	37%
$Shift_{IRF}$[ps]	7.99	±0.62	7.7%
A_{Scatt}[kCnts]	48.26	±0.97	2.0%
τ_{AvInt}[ns]	5.212	±0.023	0.4%
τ_{AvAmp}[ns]	2.263	±0.033	1.4%
λ_{Det}[nm]	610.0000	---	---

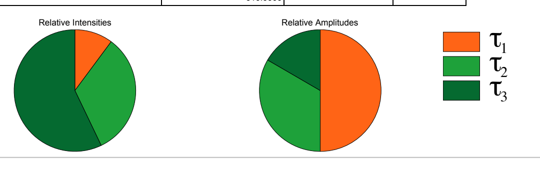

Data Set: 23 / 33

Decay: crv[23]; IRF: crv[0]

Fit

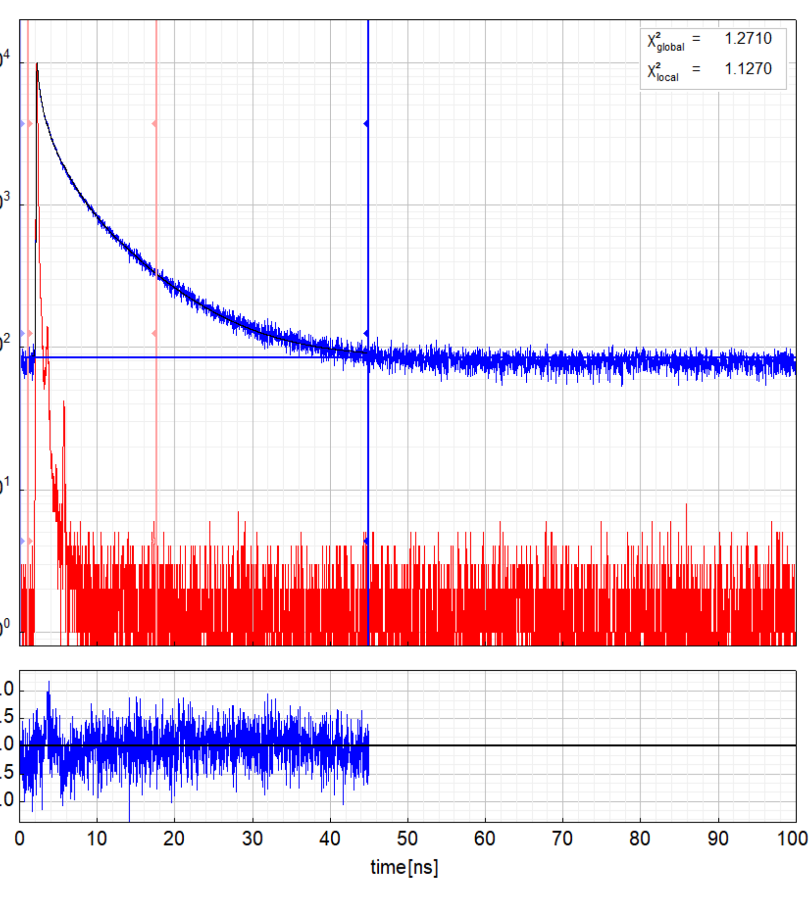

Autocorrelation (Residuals)

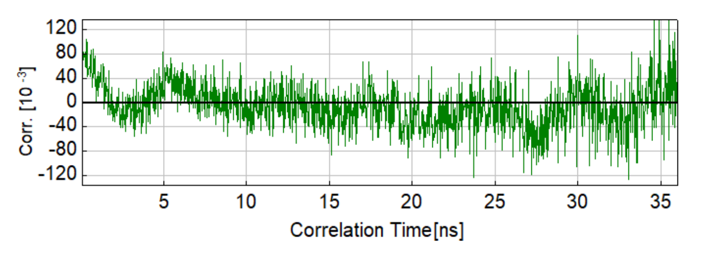

Fitted Parameters

Parameter	Value	Δ	δ
A_1[kCnts/Chnl]	5.12	±0.12	2.2%
τ_1[ns]	0.460	±0.019	4.0%
I_1[kCnts]	94.1	±1.9	2.0%
A_2[kCnts/Chnl]	3.163	±0.059	1.9%
τ_2[ns]	2.220	±0.021	0.9%
I_2[kCnts]	280.9	±3.6	1.3%
A_3[kCnts/Chnl]	1.689	±0.013	0.7%
τ_3[ns]	7.772	±0.017	0.2%
I_3[kCnts]	525.0	±4.0	0.8%
$Bkgr_{Dec}$[kCnts]	0.0844	±0.0003	0.3%
$Bkgr_{IRF}$[Cnts/Chnl]	0.22	±0.37	171%
$Shift_{IRF}$[ps]	8.3	±1.2	14%

A_{Scatt}[kCnts]	47.80	±0.95	2.0%
τ_{AvInt}[ns]	5.275	±0.021	0.4%
τ_{AvAmp}[ns]	2.258	±0.030	1.3%
λ_{Det}[nm]	620.0000	---	---

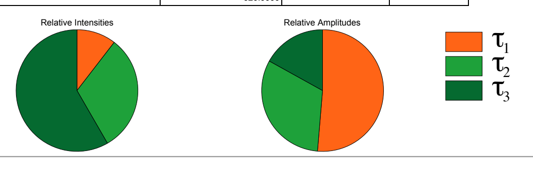

Data Set: 24 / 33

Decay: crv[24]; IRF: crv[0]

Fit

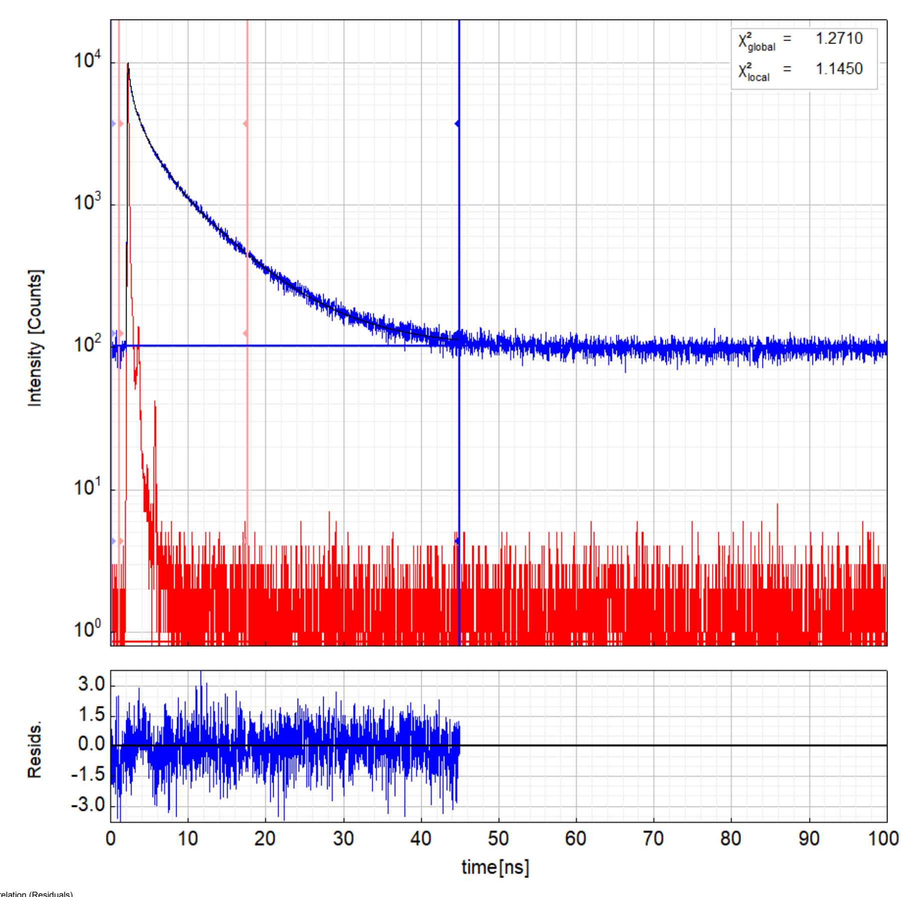

Autocorrelation (Residuals)

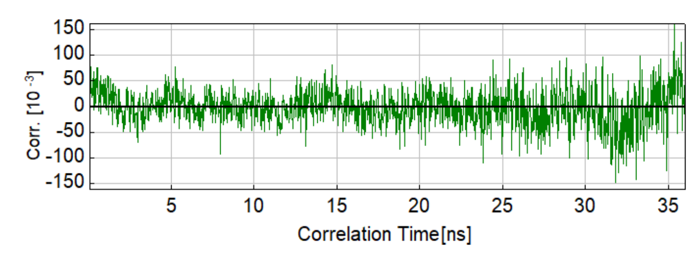

Fitted Parameters

Parameter	Value	Δ	δ
A_1[kCnts/Chnl]	4.54	±0.12	2.6%
τ_1[ns]	0.460	±0.019	4.0%
I_1[kCnts]	83.5	±3.3	3.9%
A_2[kCnts/Chnl]	3.120	±0.038	1.2%
τ_2[ns]	2.220	±0.021	0.9%
I_2[kCnts]	277.1	±2.5	0.9%
A_3[kCnts/Chnl]	2.453	±0.027	1.1%
τ_3[ns]	7.772	±0.017	0.2%
I_3[kCnts]	762.3	±6.7	0.9%
$Bkgr_{Dec}$[kCnts]	0.1027	±0.0004	0.3%
$Bkgr_{IRF}$[Cnts/Chnl]	0.86	±0.47	55%
$Shift_{IRF}$[ps]	9.88	±0.58	5.8%
A_{Scatt}[kCnts]	45.62	±0.59	1.3%
τ_{AvInt}[ns]	5.858	±0.013	0.2%
τ_{AvAmp}[ns]	2.777	±0.035	1.2%
λ_{Det}[nm]	630.0000	---	---

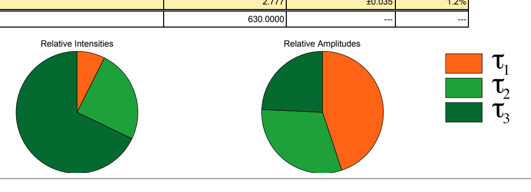

Data Set: 25 / 33

Decay: crv[25]; IRF: crv[0]

Fit

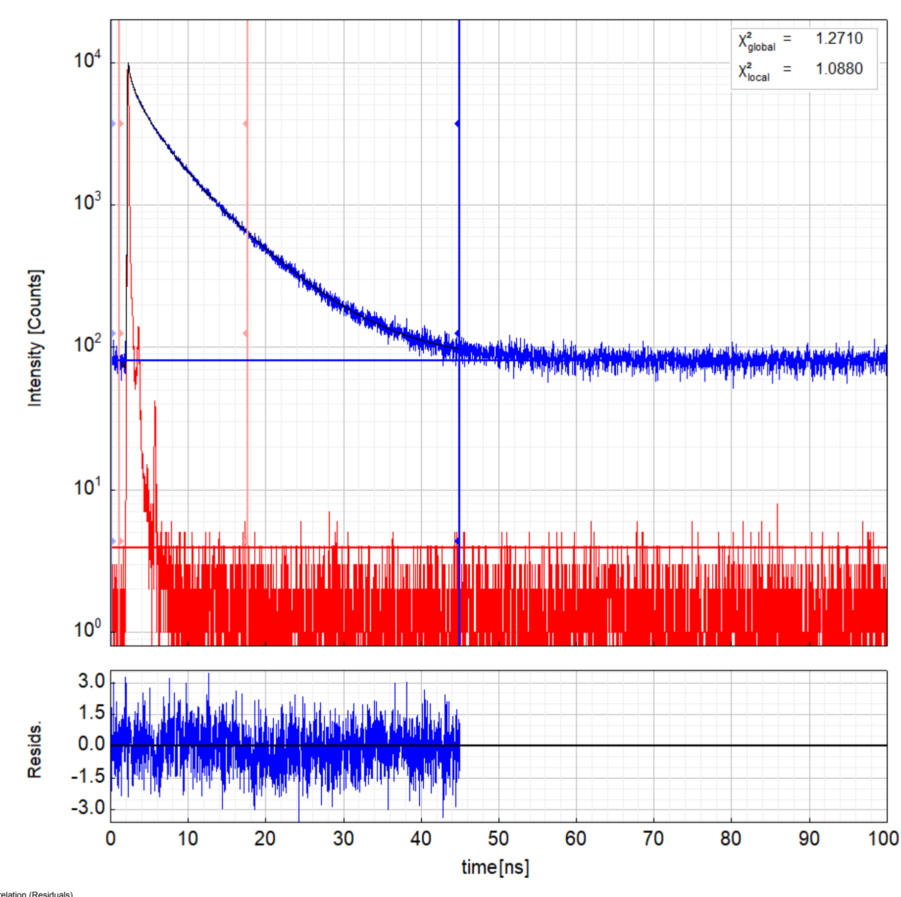

Autocorrelation (Residuals)

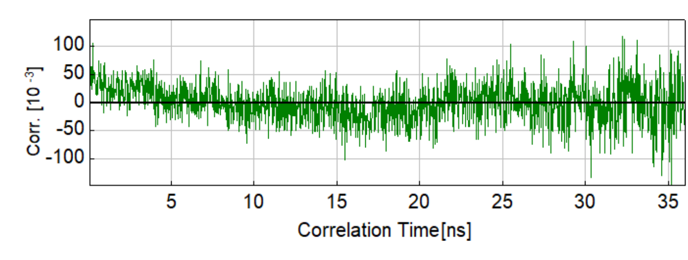

Fitted Parameters

Parameter	Value	Δ	δ
A_1[kCnts/Chnl]	3.083	±0.077	2.5%
τ_1[ns]	0.460	±0.019	4.0%
I_1[kCnts]	56.70	±0.98	1.7%
A_2[kCnts/Chnl]	3.302	±0.067	2.0%
τ_2[ns]	2.220	±0.021	0.9%
I_2[kCnts]	293.2	±3.4	1.2%
A_3[kCnts/Chnl]	4.202	±0.015	0.4%
τ_3[ns]	7.772	±0.017	0.2%
I_3[kCnts]	1 306.0	±4.8	0.4%
$Bkgr_{Dec}$[kCnts]	0.0810	±0.0005	0.5%
$Bkgr_{IRF}$[Cnts/Chnl]	3.93	±0.33	8.3%
$Shift_{IRF}$[ps]	12.28	±0.49	4.0%
A_{Scatt}[kCnts]	42.10	±0.90	2.1%
τ_{AvInt}[ns]	6.5379	±0.0087	0.1%
τ_{AvAmp}[ns]	3.911	±0.030	0.7%
λ_{Det}[nm]	640.0000	---	---

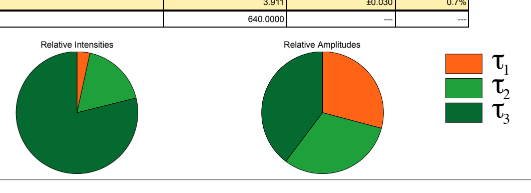

Data Set: 26 / 33

Decay: crv[26]; IRF: crv[0]

Fit

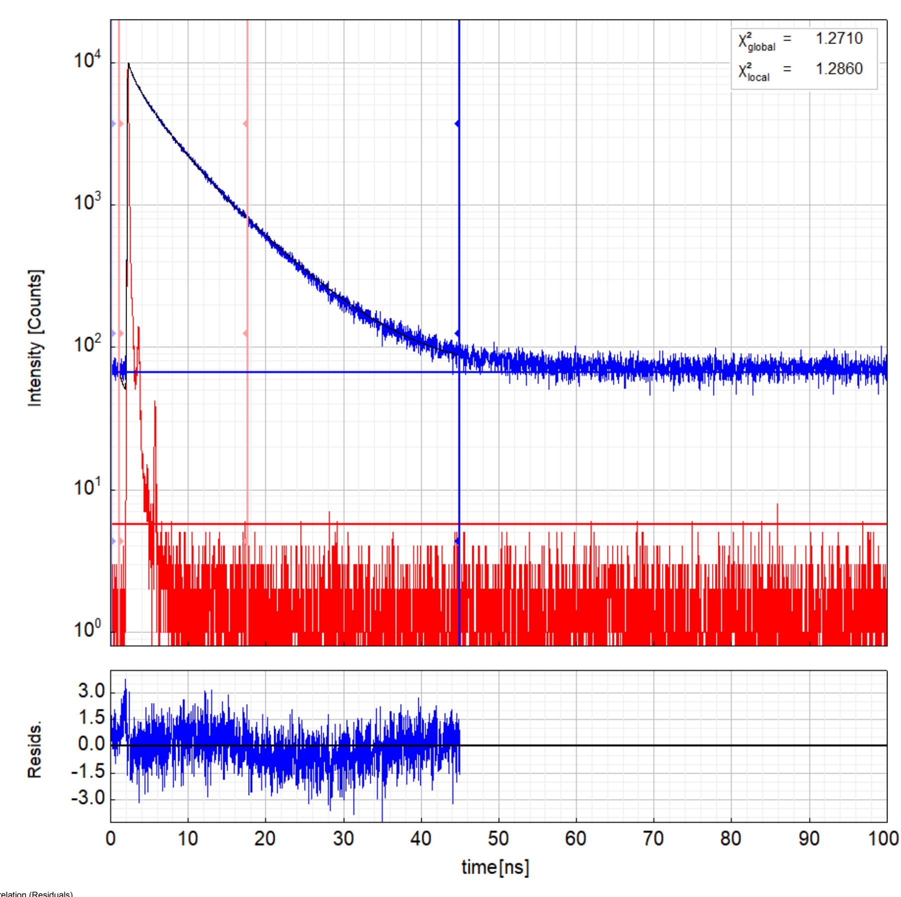

Autocorrelation (Residuals)

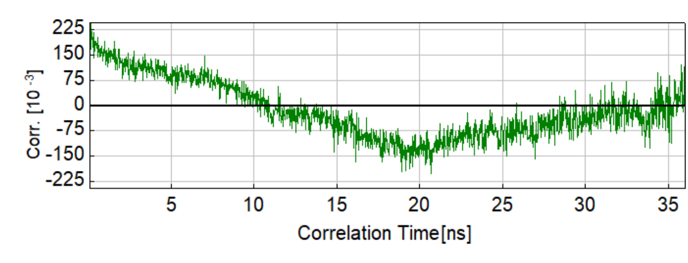

Fitted Parameters

Parameter	Value	Δ	δ
A_1[kCnts/Chnl]	2.133	±0.025	1.2%
τ_1[ns]	0.460	±0.019	4.0%
I_1[kCnts]	39.3	±2.1	5.1%
A_2[kCnts/Chnl]	3.466	±0.063	1.8%
τ_2[ns]	2.220	±0.021	0.9%
I_2[kCnts]	307.8	±5.0	1.6%
A_3[kCnts/Chnl]	5.705	±0.023	0.4%
τ_3[ns]	7.772	±0.017	0.2%
I_3[kCnts]	1 773.2	±5.8	0.3%
$Bkgr_{Dec}$[kCnts]	0.0663	±0.0004	0.5%
$Bkgr_{IRF}$[Cnts/Chnl]	5.67	±0.20	3.4%
$Shift_{IRF}$[ps]	11.01	±0.64	5.8%
A_{Scatt}[kCnts]	36.29	±0.60	1.6%
τ_{AvInt}[ns]	6.831	±0.014	0.2%
τ_{AvAmp}[ns]	4.690	±0.017	0.4%
λ_{Det}[nm]	650.0000	---	---

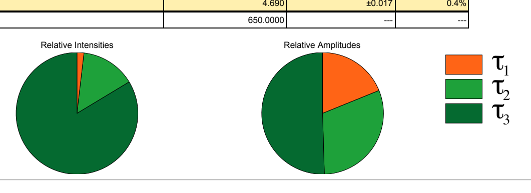

Data Set: 27 / 33

Decay: crv[27]; IRF: crv[0]

Fit

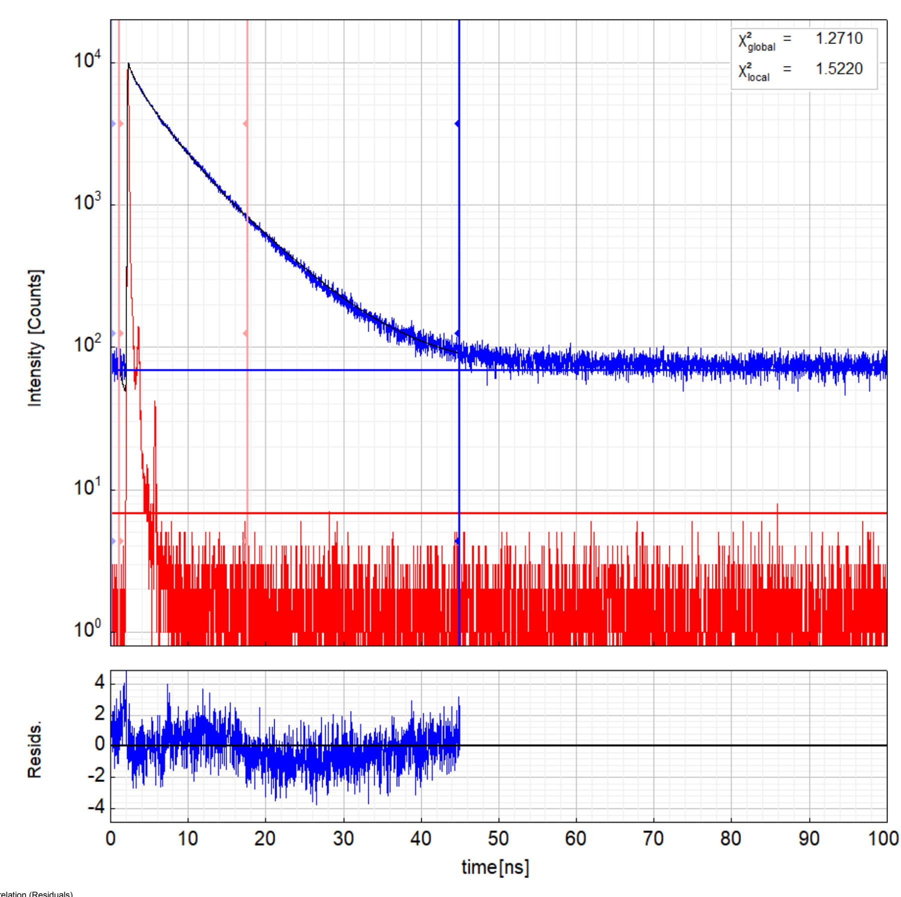

Autocorrelation (Residuals)

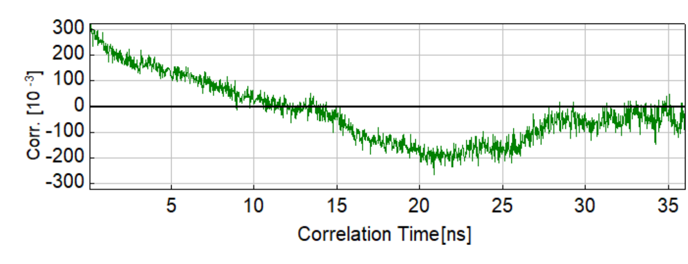

Fitted Parameters

Parameter	Value	Δ	δ
A_1[kCnts/Chnl]	1.74	±0.11	6.3%
τ_1[ns]	0.460	±0.019	4.0%
I_1[kCnts]	31.9	±2.1	6.6%
A_2[kCnts/Chnl]	3.434	±0.082	2.4%
τ_2[ns]	2.220	±0.021	0.9%
I_2[kCnts]	305	±13	4.1%
A_3[kCnts/Chnl]	5.955	±0.073	1.2%
τ_3[ns]	7.772	±0.017	0.2%
I_3[kCnts]	1 851	±21	1.1%
$Bkgr_{Dec}$[kCnts]	0.0687	±0.0008	1.1%
$Bkgr_{IRF}$[Cnts/Chnl]	6.77	±0.50	7.3%
$Shift_{IRF}$[ps]	12.90	±0.69	5.3%
A_{Scatt}[kCnts]	35.81	±0.71	2.0%
τ_{AvInt}[ns]	6.892	±0.018	0.3%
τ_{AvAmp}[ns]	4.919	±0.032	0.6%
λ_{Det}[nm]	660.0000	---	---

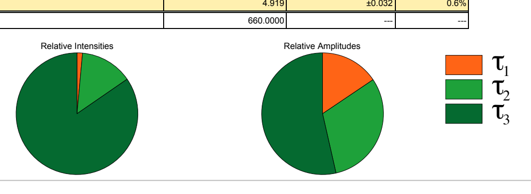

Data Set: 28 / 33

Decay: crv[28]; IRF: crv[0]

Fit

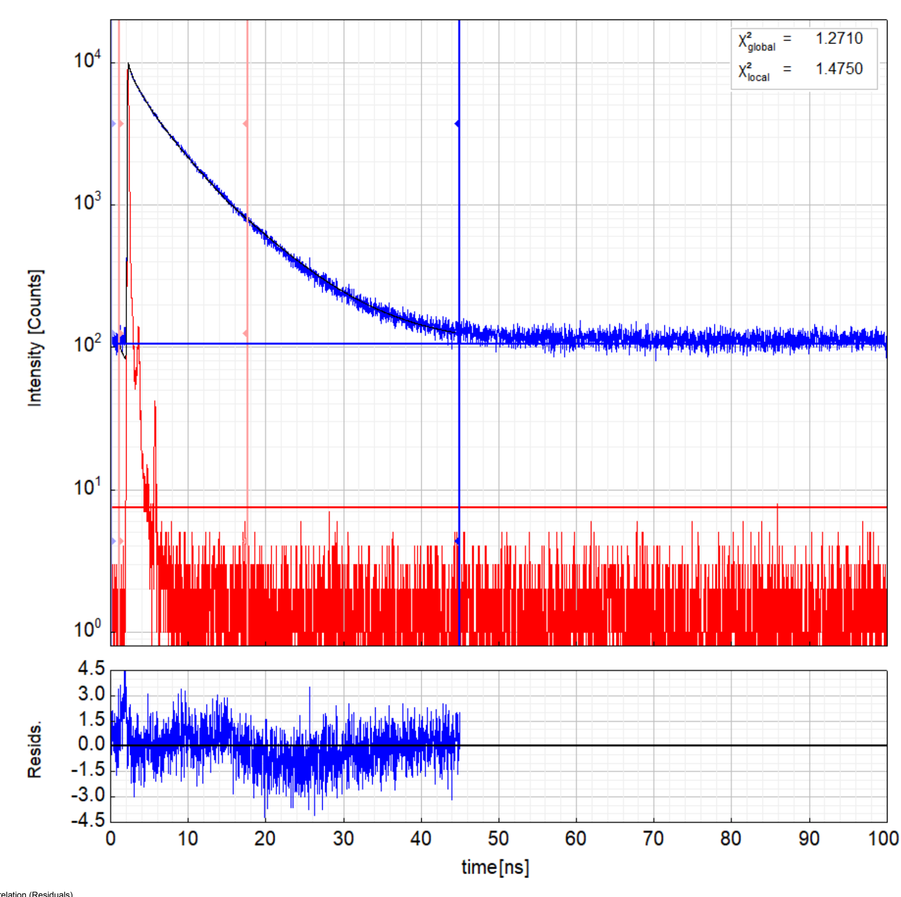

Autocorrelation (Residuals)

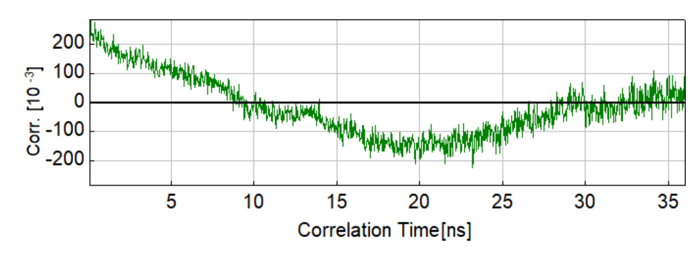

Fitted Parameters

Parameter	Value	Δ	δ
A_1[kCnts/Chnl]	2.14	±0.14	6.4%
τ_1[ns]	0.460	±0.019	4.0%
I_1[kCnts]	39.3	±1.9	4.8%
A_2[kCnts/Chnl]	3.389	±0.058	1.7%
τ_2[ns]	2.220	±0.021	0.9%
I_2[kCnts]	301	±11	3.4%
A_3[kCnts/Chnl]	5.519	±0.039	0.7%
τ_3[ns]	7.772	±0.017	0.2%
I_3[kCnts]	1 716	±13	0.7%
$Bkgr_{Dec}$[kCnts]	0.1049	±0.0006	0.6%
$Bkgr_{IRF}$[Cnts/Chnl]	7.46	±0.36	4.7%
$Shift_{IRF}$[ps]	13.9	±1.2	8.2%
A_{Scatt}[kCnts]	37.03	±0.93	2.5%
τ_{AvInt}[ns]	6.820	±0.018	0.3%
τ_{AvAmp}[ns]	4.656	±0.041	0.9%
λ_{Det}[nm]	670.0000	---	---

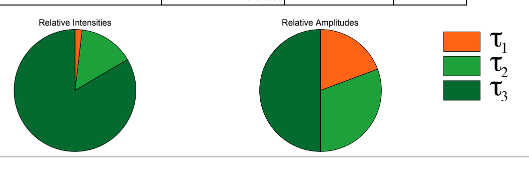

Data Set: 29 / 33

Decay: crv[29]; IRF: crv[0]

Fit

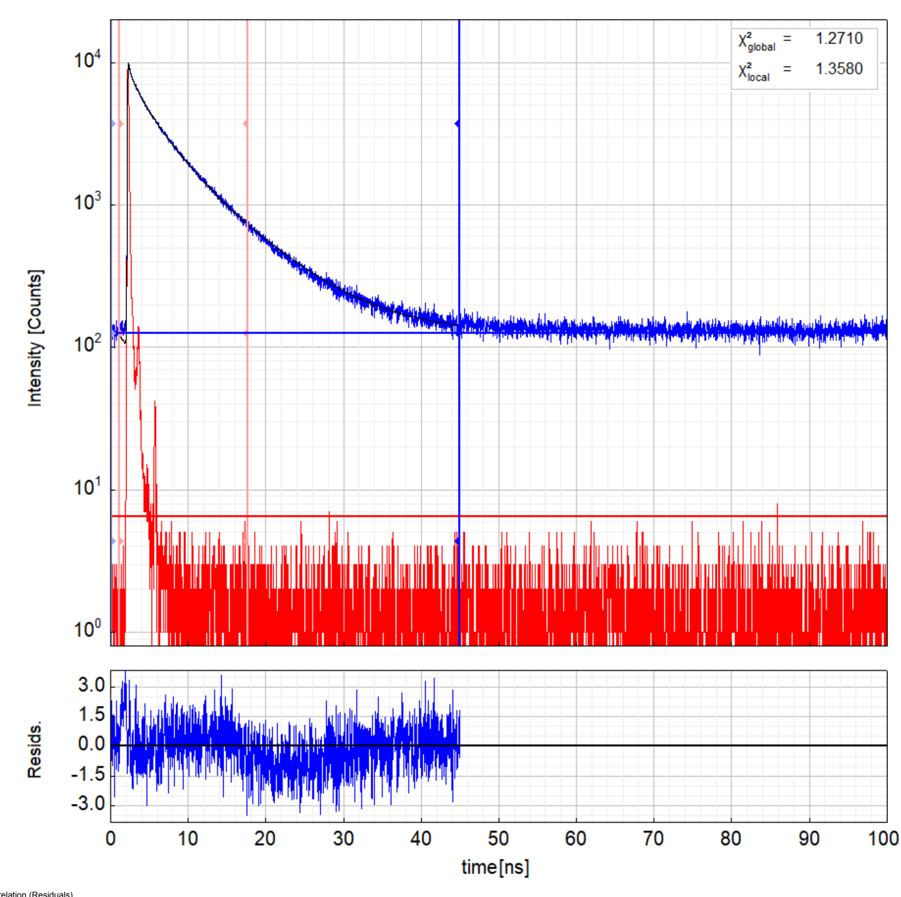

Autocorrelation (Residuals)

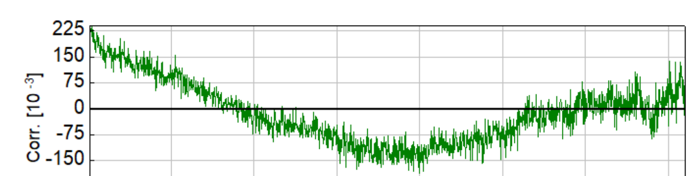

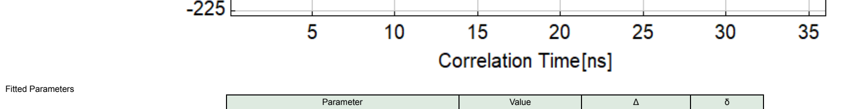

Fitted Parameters

Parameter	Value	Δ	δ
A_1[kCnts/Chnl]	2.33	±0.11	4.4%
τ_1[ns]	0.460	±0.019	4.0%
I_1[kCnts]	42.8	±2.2	5.1%
A_2[kCnts/Chnl]	3.717	±0.070	1.9%
τ_2[ns]	2.220	±0.021	0.9%
I_2[kCnts]	330.1	±7.1	2.1%
A_3[kCnts/Chnl]	4.807	±0.037	0.8%
τ_3[ns]	7.772	±0.017	0.2%
I_3[kCnts]	1 494.0	±8.4	0.6%
$Bkgr_{Dec}$[kCnts]	0.1246	±0.0005	0.4%
$Bkgr_{IRF}$[Cnts/Chnl]	6.43	±0.33	5.1%
$Shift_{IRF}$[ps]	15.26	±0.51	3.3%
A_{Scatt}[kCnts]	39.05	±0.94	2.4%
τ_{AvInt}[ns]	6.6222	±0.0071	0.1%
τ_{AvAmp}[ns]	4.302	±0.022	0.5%
λ_{Det}[nm]	680.0000	---	---

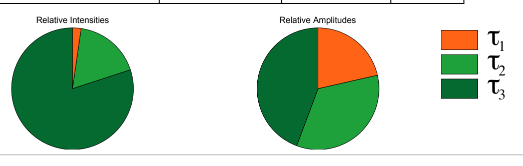

Data Set: 30 / 33

Decay: crv[30]; IRF: crv[0]

Fit

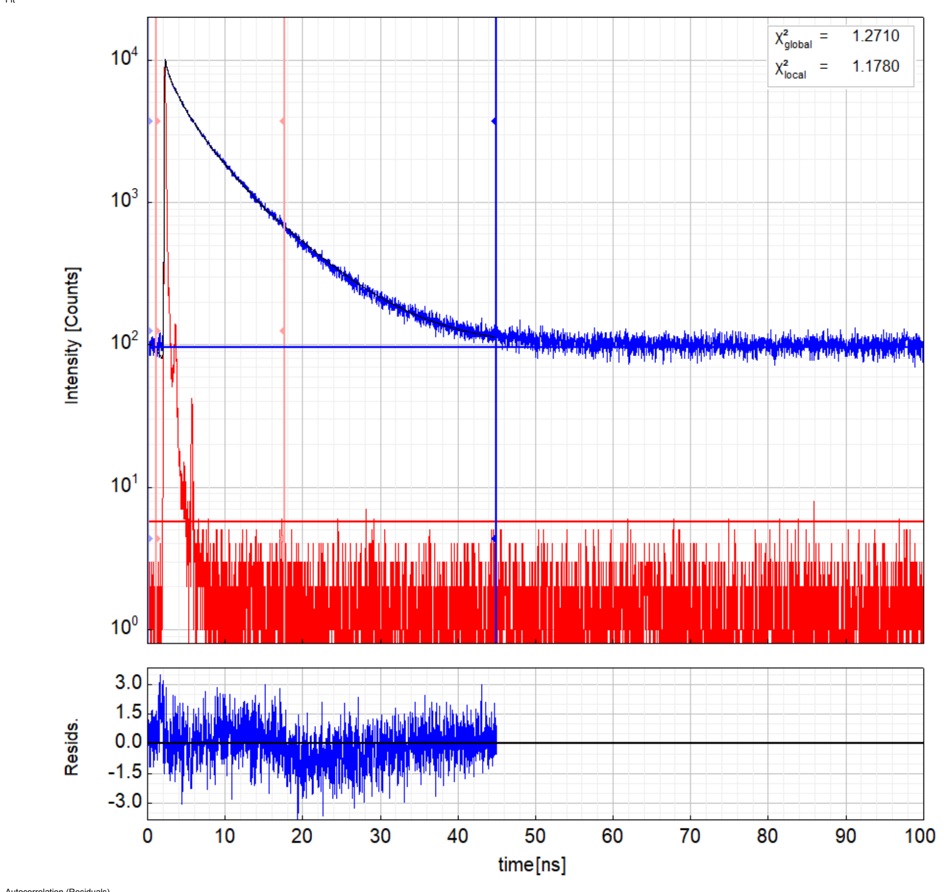

Autocorrelation (Residuals)

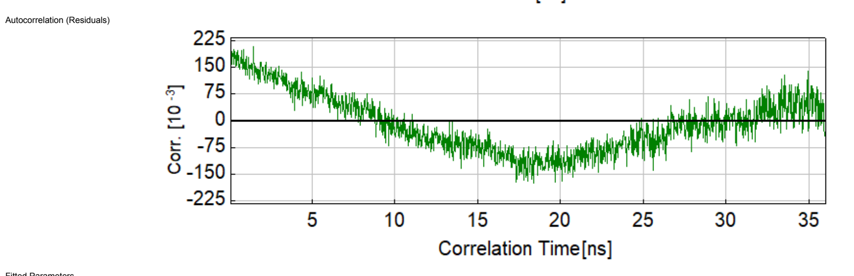

Fitted Parameters

Parameter	Value	Δ	δ
A_1[kCnts/Chnl]	2.528	±0.098	3.9%
τ_1[ns]	0.460	±0.019	4.0%
I_1[kCnts]	46.5	±2.0	4.1%
A_2[kCnts/Chnl]	3.983	±0.063	1.6%
τ_2[ns]	2.220	±0.021	0.9%
I_2[kCnts]	353.7	±8.3	2.3%
A_3[kCnts/Chnl]	4.565	±0.038	0.8%
τ_3[ns]	7.772	±0.017	0.2%
I_3[kCnts]	1 420	±15	1.0%
$Bkgr_{Dec}$[kCnts]	0.0956	±0.0007	0.7%
$Bkgr_{IRF}$[Cnts/Chnl]	5.75	±0.63	11%
$Shift_{IRF}$[ps]	15.80	±0.29	1.8%
A_{Scatt}[kCnts]	38.94	±0.27	0.7%
τ_{AvInt}[ns]	6.506	±0.027	0.4%
τ_{AvAmp}[ns]	4.107	±0.038	0.9%
λ_{Det}[nm]	690.0000	---	---

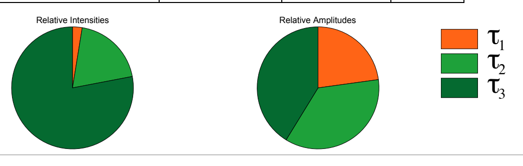

Data Set: 31 / 33

Decay: crv[31]; IRF: crv[0]

Fit

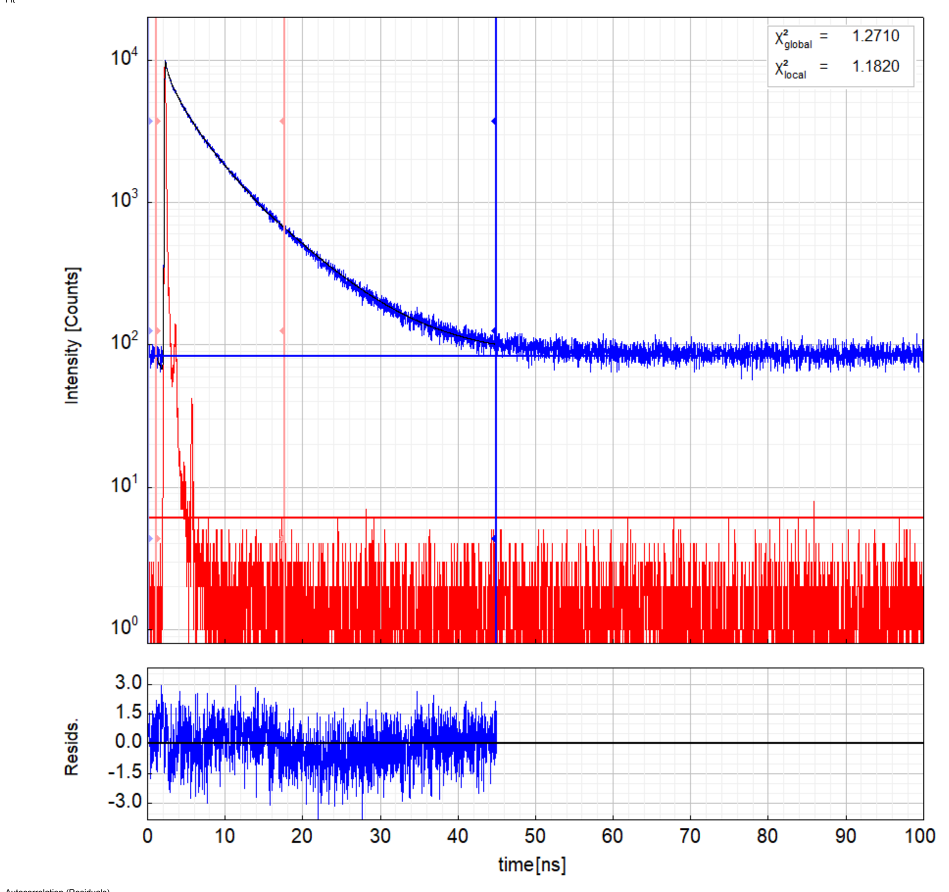

Autocorrelation (Residuals)

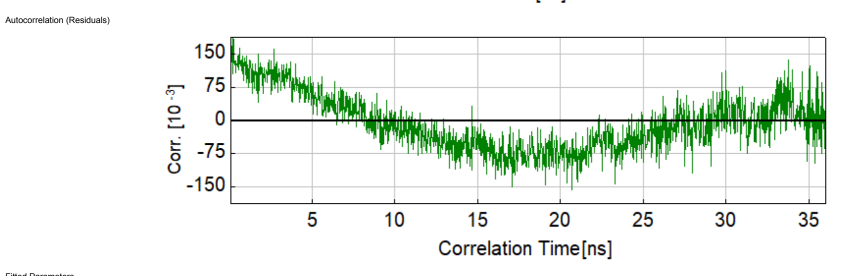

Fitted Parameters

Parameter	Value	Δ	δ
A_1[kCnts/Chnl]	2.61	±0.11	4.1%
τ_1[ns]	0.460	±0.019	4.0%
I_1[kCnts]	48.0	±2.2	4.4%
A_2[kCnts/Chnl]	3.743	±0.077	2.0%
τ_2[ns]	2.220	±0.021	0.9%
I_2[kCnts]	332.4	±6.4	1.9%
A_3[kCnts/Chnl]	4.533	±0.038	0.8%
τ_3[ns]	7.772	±0.017	0.2%
I_3[kCnts]	1 409	±12	0.8%
$Bkgr_{Dec}$[kCnts]	0.0834	±0.0004	0.4%
$Bkgr_{IRF}$[Cnts/Chnl]	6.09	±0.46	7.4%
$Shift_{IRF}$[ps]	16.6	±1.1	6.1%
A_{Scatt}[kCnts]	37.47	±0.90	2.4%
τ_{AvInt}[ns]	6.544	±0.023	0.3%
τ_{AvAmp}[ns]	4.110	±0.036	0.9%
λ_{Det}[nm]	700.0000	---	---

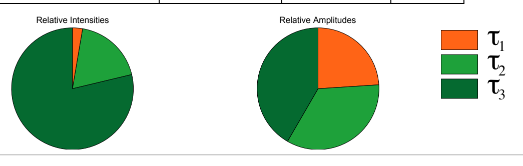

Data Set: 32 / 33

Decay: crv[32]; IRF: crv[0]

Fit

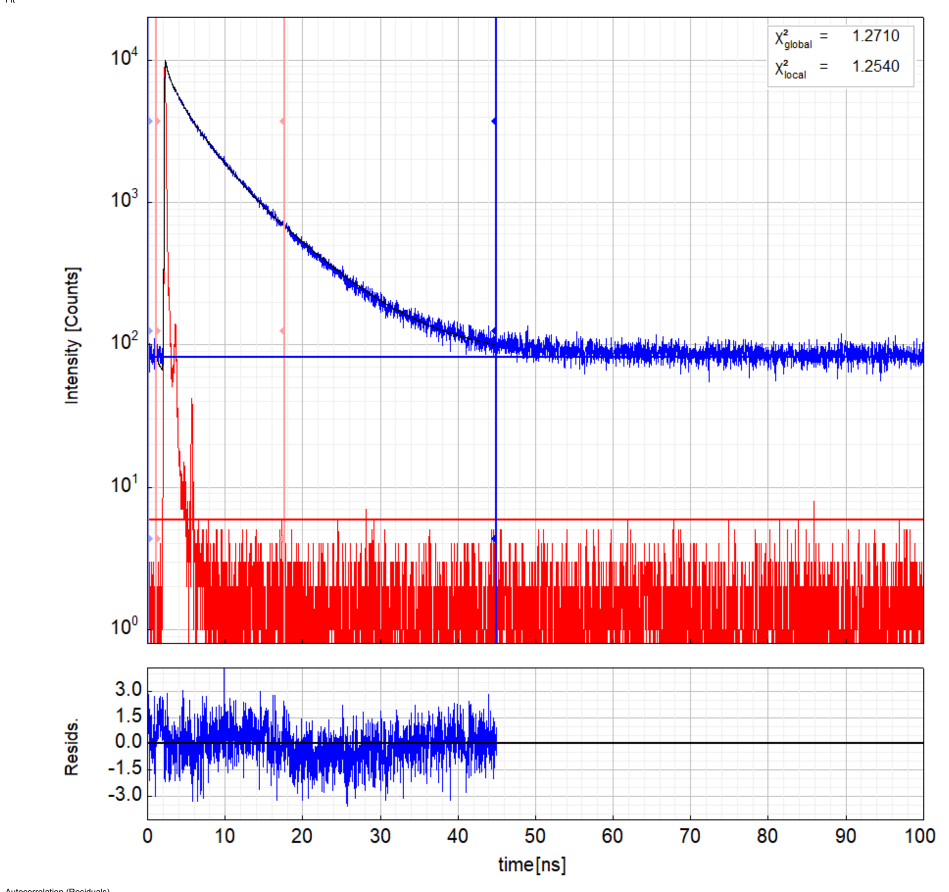

Autocorrelation (Residuals)

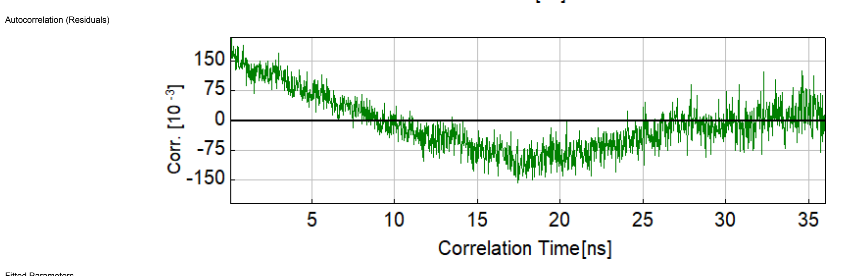

Fitted Parameters

Parameter	Value	Δ	δ
A_1[kCnts/Chnl]	2.623	±0.081	3.1%
τ_1[ns]	0.460	±0.019	4.0%
I_1[kCnts]	48.3	±2.5	5.1%
A_2[kCnts/Chnl]	3.728	±0.080	2.1%
τ_2[ns]	2.220	±0.021	0.9%
I_2[kCnts]	331.1	±7.1	2.1%
A_3[kCnts/Chnl]	4.693	±0.043	0.9%
τ_3[ns]	7.772	±0.017	0.2%
I_3[kCnts]	1 459	±11	0.7%
$Bkgr_{Dec}$[kCnts]	0.0823	±0.0004	0.5%
$Bkgr_{IRF}$[Cnts/Chnl]	5.86	±0.40	6.7%
$Shift_{IRF}$[ps]	17.60	±0.45	2.5%
A_{Scatt}[kCnts]	37.53	±0.80	2.1%
τ_{AvInt}[ns]	6.580	±0.014	0.2%
τ_{AvAmp}[ns]	4.162	±0.032	0.8%
λ_{Det}[nm]	710.0000	---	---

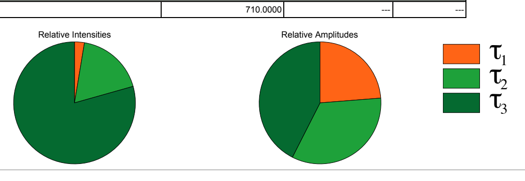

Data Set: 33 / 33

Decay: crv[33]; IRF: crv[0]

Fit

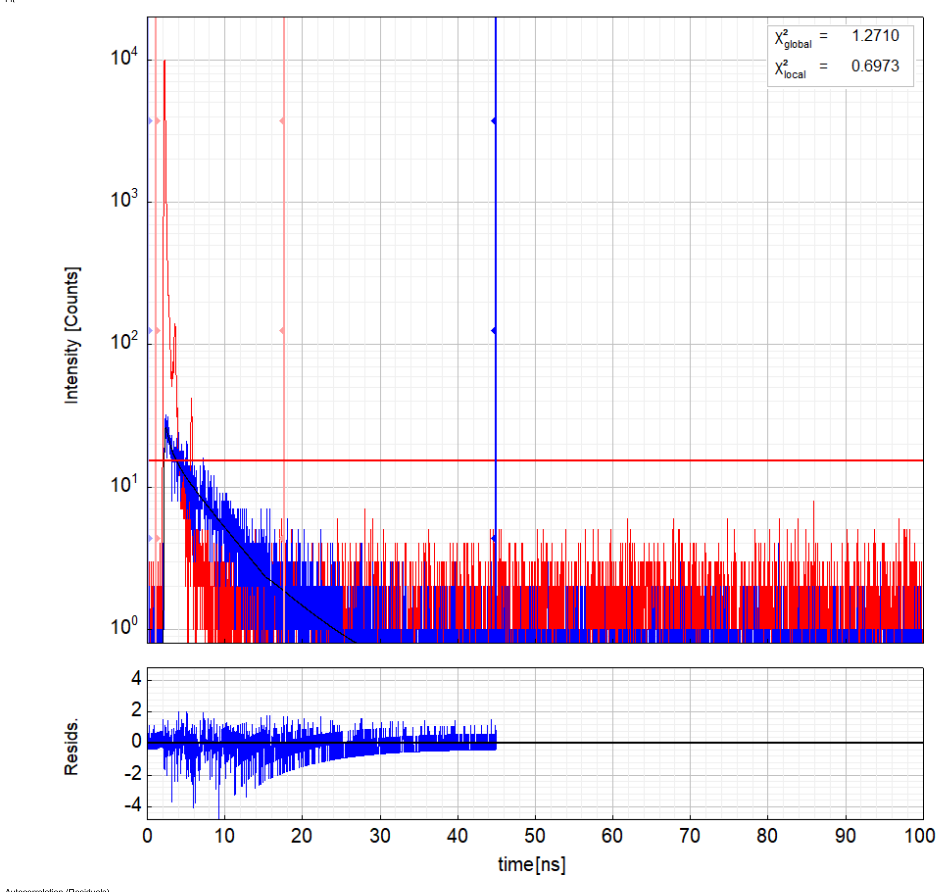

Autocorrelation (Residuals)

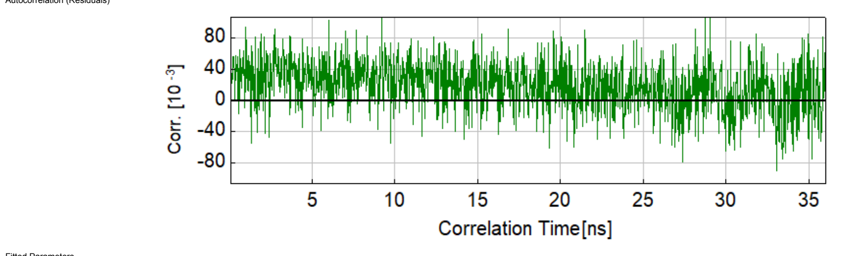

Fitted Parameters

Parameter	Value	Δ	δ
A_1[kCnts/Chnl]	0.0156	±0.0043	27%
τ_1[ns]	0.460	±0.019	4.0%
I_1[kCnts]	0.286	±0.065	23%
A_2[kCnts/Chnl]	0.0061	±0.0011	18%
τ_2[ns]	2.220	±0.021	0.9%
I_2[kCnts]	0.54	±0.11	19%
A_3[kCnts/Chnl]	0.0143	±0.0003	1.7%
τ_3[ns]	7.772	±0.017	0.2%
I_3[kCnts]	4.436	±0.073	1.6%
$Bkgr_{Dec}$[kCnts]	0.0004	±0.0001	8.6%
$Bkgr_{IRF}$[Cnts/Chnl]	15.2	±2.1	14%
$Shift_{IRF}$[ps]	-9	±37	390%
A_{Scatt}[kCnts]	0.025	±0.073	298%
τ_{AvInt}[ns]	6.805	±0.081	1.2%
τ_{AvAmp}[ns]	3.67	±0.36	9.8%
λ_{Det}[nm]	720.0000	---	---

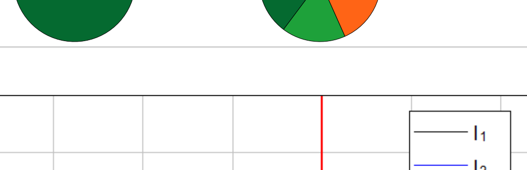

Parameter Plot

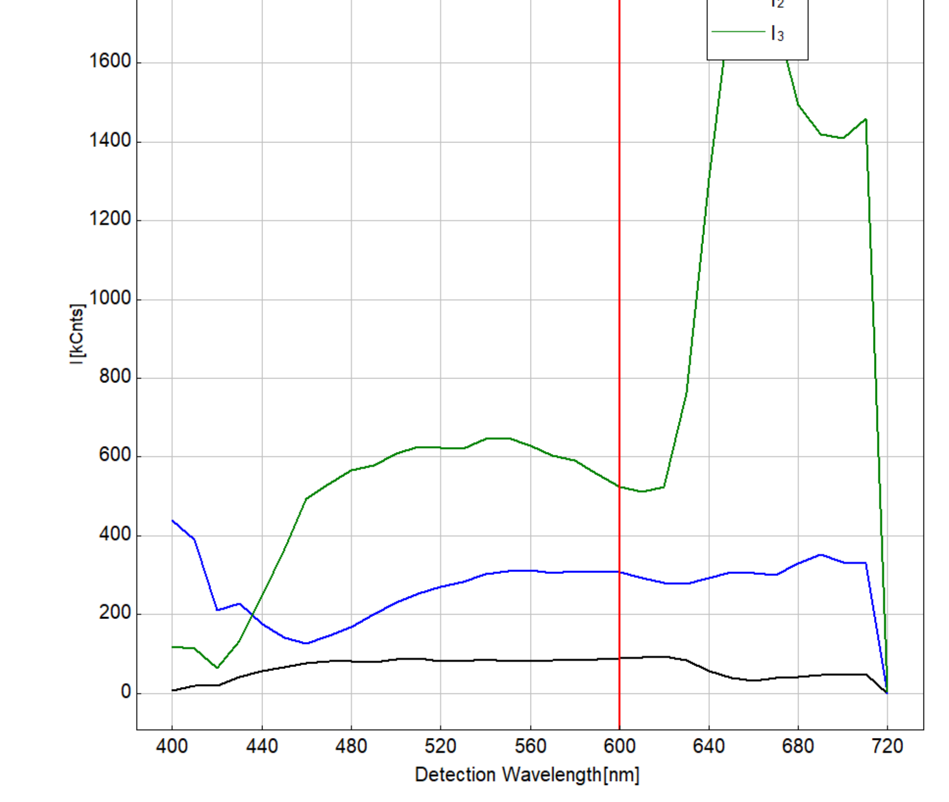

Global Parameters

Parameter	Value	Δ	δ
τ_1[ns]	0.460	±0.019	4.0%
τ_2[ns]	2.220	±0.021	0.9%
τ_3[ns]	7.772	±0.017	0.2%
