# Supplementary material for: A Porphyrin‐Based Organic Network Comprising Sustainable Carbon Dots for Photopolymerization
Source: Angew Chem Int Ed Engl. 2022 Sep 1;61(40):e202208180. doi: 10.1002/anie.202208180 (PMC9826160; doi:10.1002/anie.202208180)
Supplement: Supplementary file 6 — Supporting Information [file ANIE-61-0-s001.pdf]

EasyTau Report

3/11/2022 7:47:16 PM
F:\Paper Xiongfei\CD-1 MeOH tau global.html

Container Curves

TRES+IRF_20201222_1636.etc: crv[0 - 31]

Container Properties

Measurement Context:	Time Resolved Emission Scan
Detection Wavelength:	400.0 - 700.0 (Step: 10.0) nm
Summary:	
Sample:	CD_X_1
Solvent:	Methanol
Excitation:	V pol 376±1nm with LDH-P-C-375
Detection:	M pol 400...700nm 10nm steps<br>10000 peak counts<br>grating 1200/500-<br>detector UV-red [PMT]

Comment

Model: Exponential [Reconv.]

$$\mathrm{Dec}(t)=\left[\int_{-\infty}^{t} dt'\,[\mathrm{IRF}(t-Shift_{IRF})-Bkgr_{IRF}]\left[\sum_{i=1}^{n_{Exp}} A_i\, e^{-\frac{t-t'}{\tau_i}} + A_{Scatt}\,\delta(t-t')\right]\right]+Bkgr_{Dec}$$

$$I_m = A_m \tau_m$$

$$A_{Rel\,m} = A_m \Big/ \sum_{\substack{i=1\\A_i>0}}^{n_{Exp}} A_i \qquad I_{Rel\,m} = I_m \Big/ \sum_{\substack{i=1\\I_i>0}}^{n_{Exp}} I_i$$

$$\tau_{Av\,Amp} = \sum_{\substack{i=1\\A_i>0}}^{n_{Exp}} A_i\tau_i \Big/ \sum_{\substack{i=1\\A_i>0}}^{n_{Exp}} A_i \qquad \tau_{Av\,Int} = \sum_{\substack{i=1\\I_i>0}}^{n_{Exp}} I_i\tau_i \Big/ \sum_{\substack{i=1\\I_i>0}}^{n_{Exp}} I_i$$

Data Set: 1 / 31

Decay: crv[1]; IRF: crv[0]

Fit

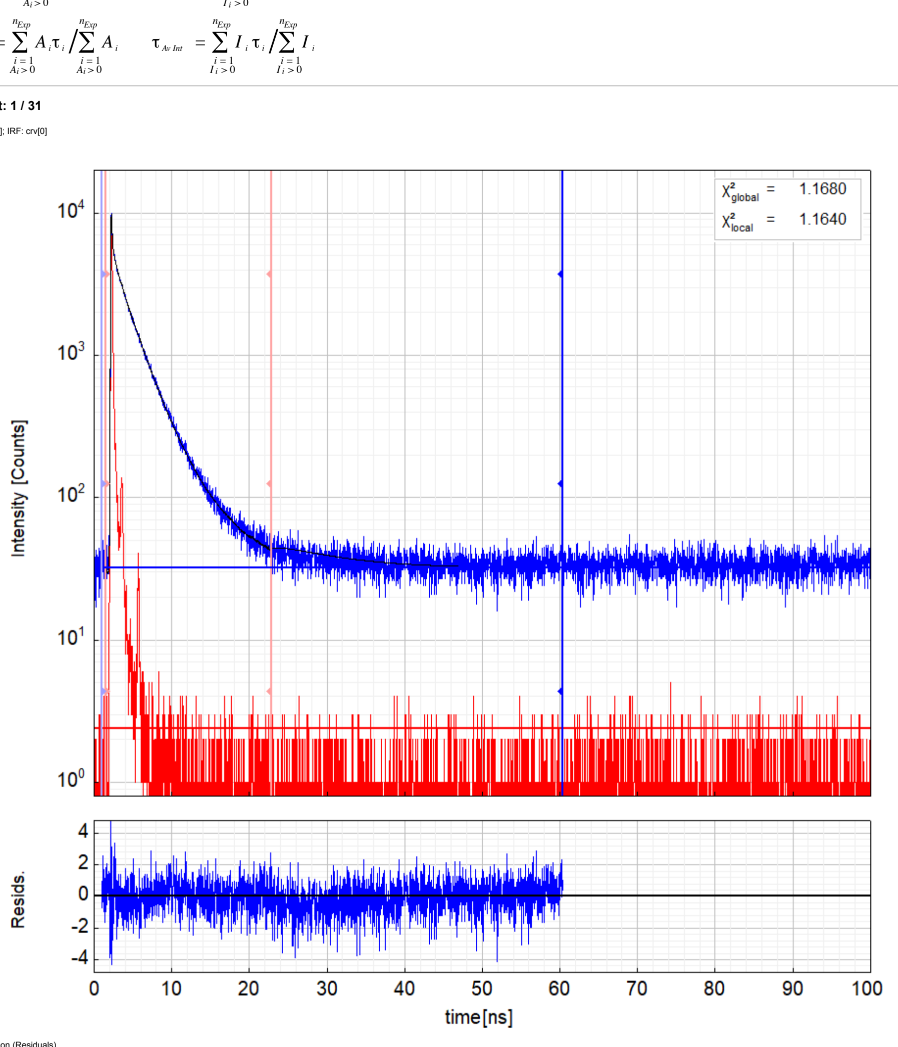

Autocorrelation (Residuals)

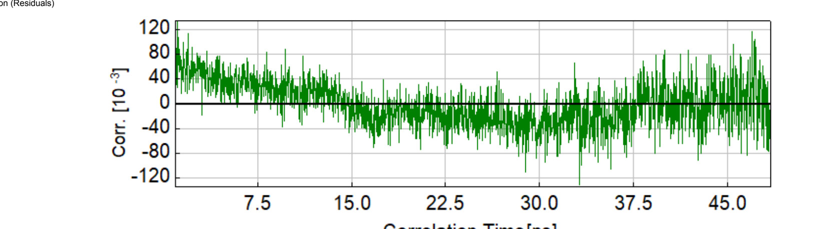

Fitted Parameters

Parameter	Value	Δ	δ
A_1[kCnts/Chnl]	4.557	±0.038	0.8%
τ_1[ns]	2.505	±0.013	0.5%
I_1[kCnts]	456.6	±1.6	0.3%
A_2[kCnts/Chnl]	0.2643	±0.0062	2.3%
τ_2[ns]	8.0397	±0.0067	0.1%
I_2[kCnts]	85.0	±2.3	2.6%
A_3[kCnts/Chnl]	1.77	±0.12	6.7%
τ_3[ns]	0.5435	±0.0070	1.3%
I_3[kCnts]	38.5	±3.2	8.2%
$Bkgr_{Dec}$[kCnts]	0.0320	±0.0002	0.6%
$Bkgr_{IRF}$[Cnts/Chnl]	2.40	±0.13	5.3%
$Shift_{IRF}$[ps]	-9.7	±2.6	26%
A_{Scatt}[kCnts]	59.9	±1.6	2.6%
τ_{AvInt}[ns]	3.186	±0.017	0.5%
τ_{AvAmp}[ns]	2.201	±0.020	0.9%
λ_{Det}[nm]	400.0000	---	---

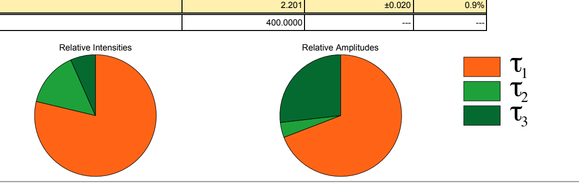

Data Set: 2 / 31

Decay: crv[2]; IRF: crv[0]

Fit

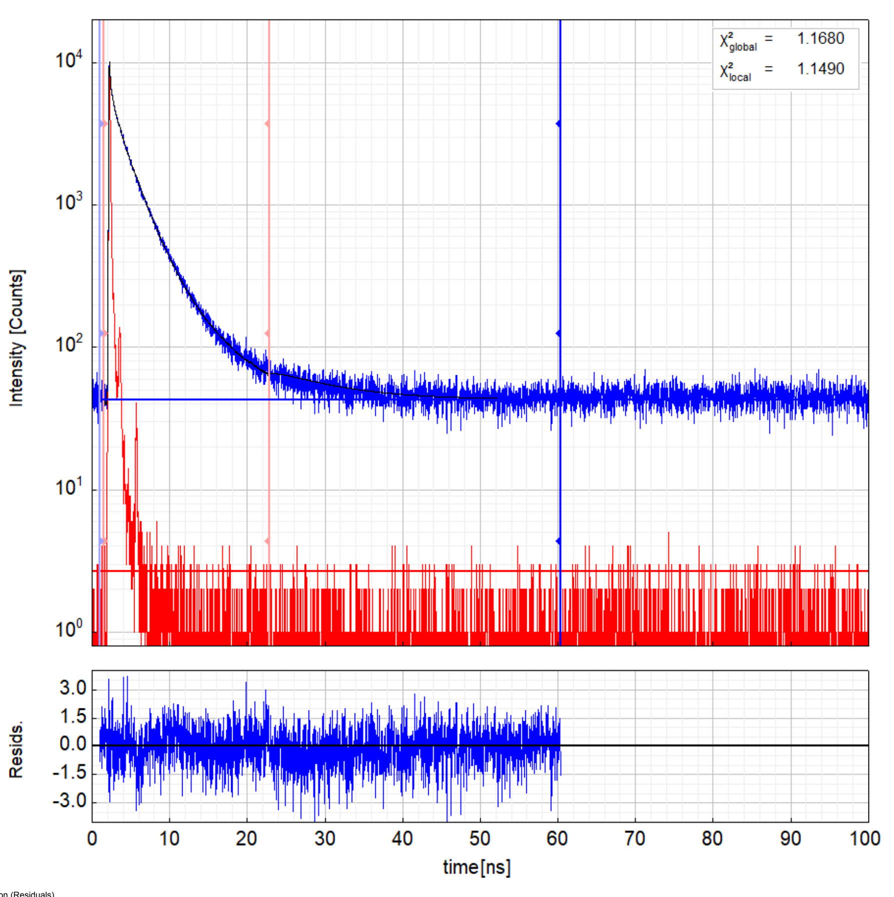

Autocorrelation (Residuals)

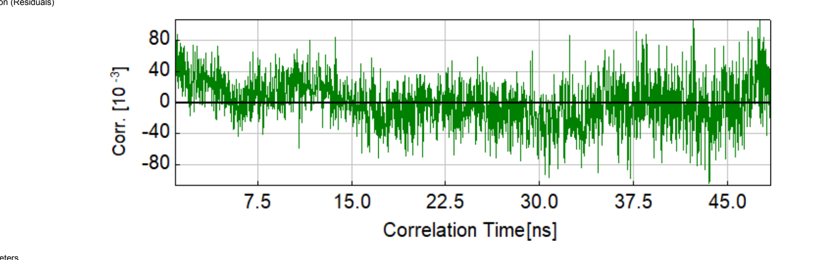

Fitted Parameters

Parameter	Value	Δ	δ
A_1[kCnts/Chnl]	4.764	±0.038	0.8%
τ_1[ns]	2.505	±0.013	0.5%
I_1[kCnts]	477.3	±4.1	0.9%
A_2[kCnts/Chnl]	0.446	±0.017	3.7%
τ_2[ns]	8.0397	±0.0067	0.1%
I_2[kCnts]	143.2	±5.2	3.6%
A_3[kCnts/Chnl]	2.854	±0.082	2.8%
τ_3[ns]	0.5435	±0.0070	1.3%
I_3[kCnts]	62.1	±2.1	3.3%
$Bkgr_{Dec}$[kCnts]	0.0430	±0.0003	0.5%
$Bkgr_{IRF}$[Cnts/Chnl]	2.68	±0.22	7.9%
$Shift_{IRF}$[ps]	-5.76	±0.80	14%
A_{Scatt}[kCnts]	55.43	±0.76	1.4%
τ_{AvInt}[ns]	3.488	±0.031	0.9%
τ_{AvAmp}[ns]	2.117	±0.015	0.7%
λ_{Det}[nm]	410.0000	---	---

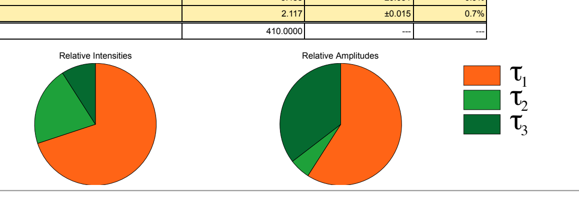

Data Set: 3 / 31

Decay: crv[3]; IRF: crv[0]

Fit

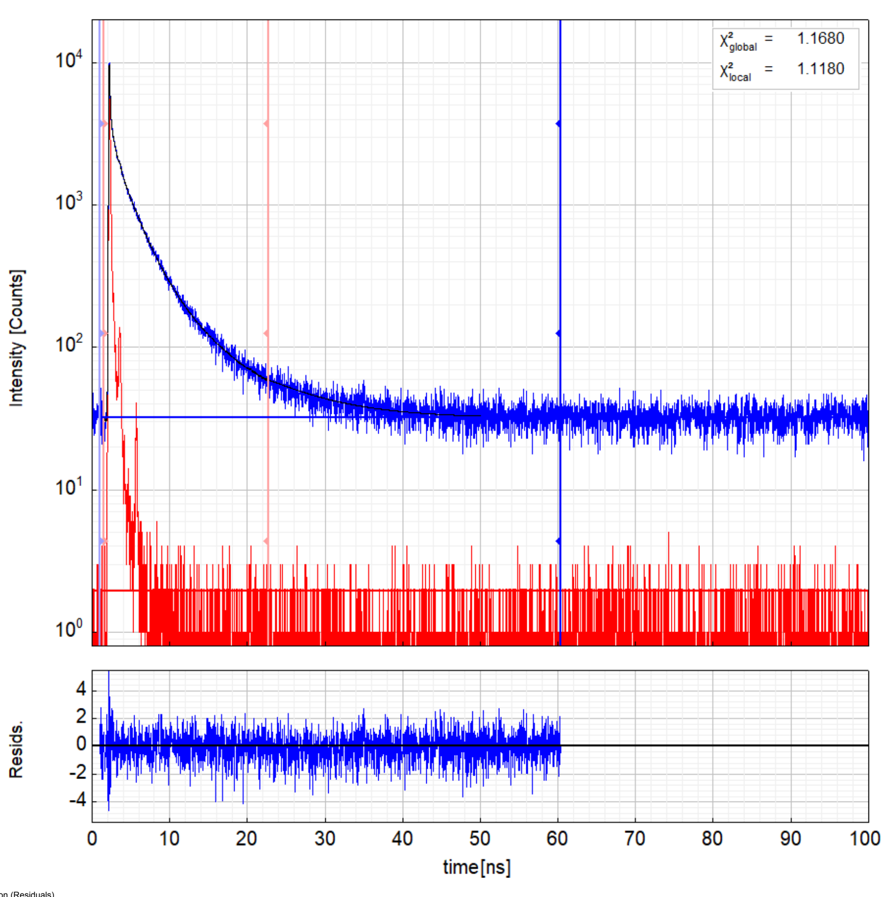

Autocorrelation (Residuals)

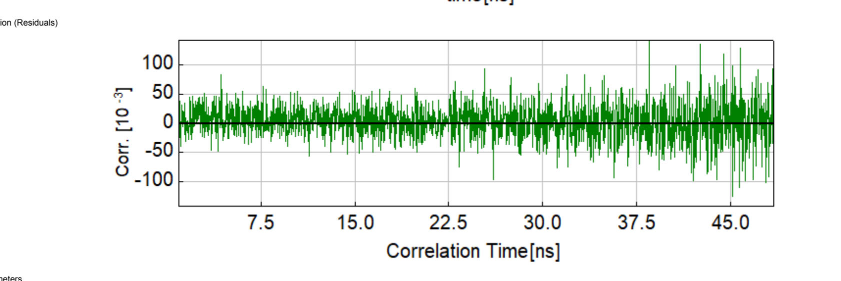

Fitted Parameters

Parameter	Value	Δ	δ
A_1[kCnts/Chnl]	2.290	±0.018	0.8%
τ_1[ns]	2.505	±0.013	0.5%
I_1[kCnts]	229.5	±1.4	0.6%
A_2[kCnts/Chnl]	0.3956	±0.0042	1.1%
τ_2[ns]	8.0397	±0.0067	0.1%
I_2[kCnts]	127.2	±1.3	1.0%
A_3[kCnts/Chnl]	1.856	±0.081	4.3%
τ_3[ns]	0.5435	±0.0070	1.3%
I_3[kCnts]	40.4	±1.9	4.6%
$Bkgr_{Dec}$[kCnts]	0.0320	±0.0003	0.7%
$Bkgr_{IRF}$[Cnts/Chnl]	1.96	±0.11	5.4%
$Shift_{IRF}$[ps]	-13.91	±0.34	2.4%
A_{Scatt}[kCnts]	67.33	±0.87	1.3%
τ_{AvInt}[ns]	4.079	±0.017	0.4%
τ_{AvAmp}[ns]	2.186	±0.026	1.1%
λ_{Det}[nm]	420.0000	---	---

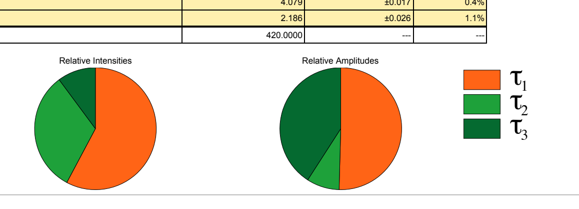

Data Set: 4 / 31

Decay: crv[4]; IRF: crv[0]

Fit

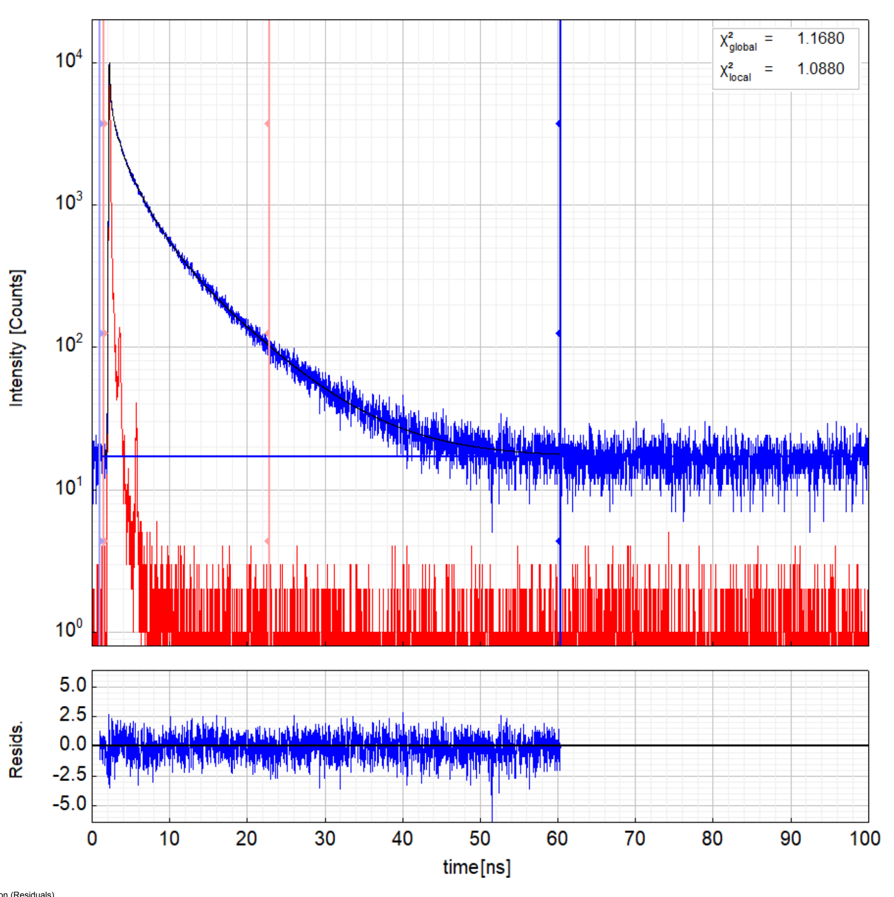

Autocorrelation (Residuals)

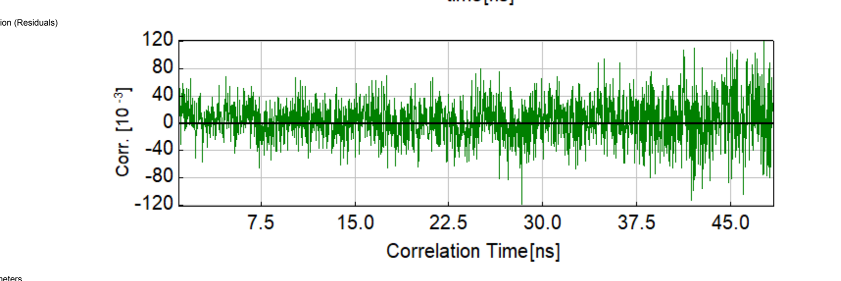

Fitted Parameters

Parameter	Value	Δ	δ
A_1[kCnts/Chnl]	2.525	±0.025	1.0%
τ_1[ns]	2.505	±0.013	0.5%
I_1[kCnts]	253.0	±2.1	0.8%
A_2[kCnts/Chnl]	1.0815	±0.0086	0.8%
τ_2[ns]	8.0397	±0.0067	0.1%
I_2[kCnts]	347.8	±2.8	0.8%
A_3[kCnts/Chnl]	2.872	±0.045	1.5%
τ_3[ns]	0.5435	±0.0070	1.3%
I_3[kCnts]	62.42	±0.91	1.4%
$Bkgr_{Dec}$[kCnts]	0.0170	±0.0002	1.0%
$Bkgr_{IRF}$[Cnts/Chnl]	0.74	±0.16	21%
$Shift_{IRF}$[ps]	-8.1	±1.2	14%
A_{Scatt}[kCnts]	62.39	±0.51	0.8%
τ_{AvInt}[ns]	5.223	±0.017	0.3%
τ_{AvAmp}[ns]	2.560	±0.013	0.5%
λ_{Det}[nm]	430.0000	---	---

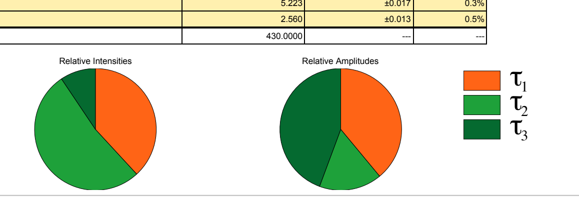

Data Set: 5 / 31

Decay: crv[5]; IRF: crv[0]

Fit

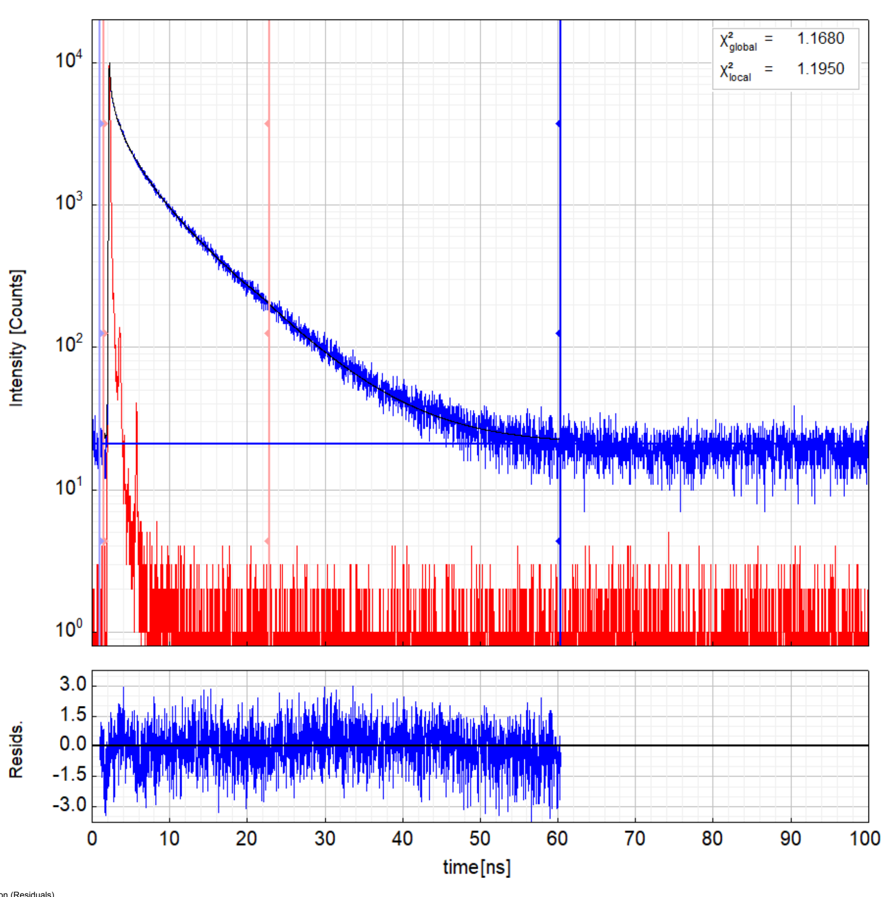

Autocorrelation (Residuals)

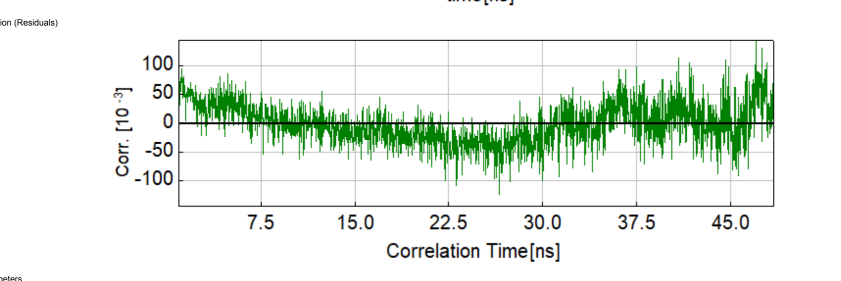

Fitted Parameters

Parameter	Value	Δ	δ
A_1[kCnts/Chnl]	2.475	±0.028	1.1%
τ_1[ns]	2.505	±0.013	0.5%
I_1[kCnts]	248.0	±2.2	0.9%
A_2[kCnts/Chnl]	2.106	±0.012	0.6%
τ_2[ns]	8.0397	±0.0067	0.1%
I_2[kCnts]	677.3	±2.5	0.4%
A_3[kCnts/Chnl]	3.288	±0.045	1.3%
τ_3[ns]	0.5435	±0.0070	1.3%
I_3[kCnts]	71.5	±1.8	2.4%
$Bkgr_{Dec}$[kCnts]	0.0209	±0.0002	0.8%
$Bkgr_{IRF}$[Cnts/Chnl]	-0.67	±0.12	18%
$Shift_{IRF}$[ps]	-2.97	±0.30	9.9%
A_{Scatt}[kCnts]	55.25	±0.41	0.7%
τ_{AvInt}[ns]	6.1253	±0.0097	0.2%
τ_{AvAmp}[ns]	3.1668	±0.0066	0.2%
λ_{Det}[nm]	440.0000	---	---

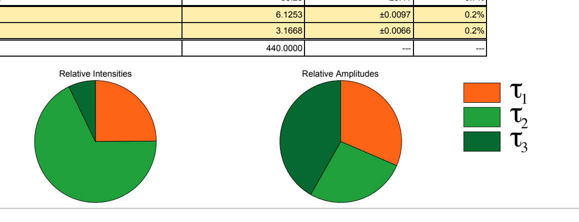

Data Set: 6 / 31

Decay: crv[6]; IRF: crv[0]

Fit

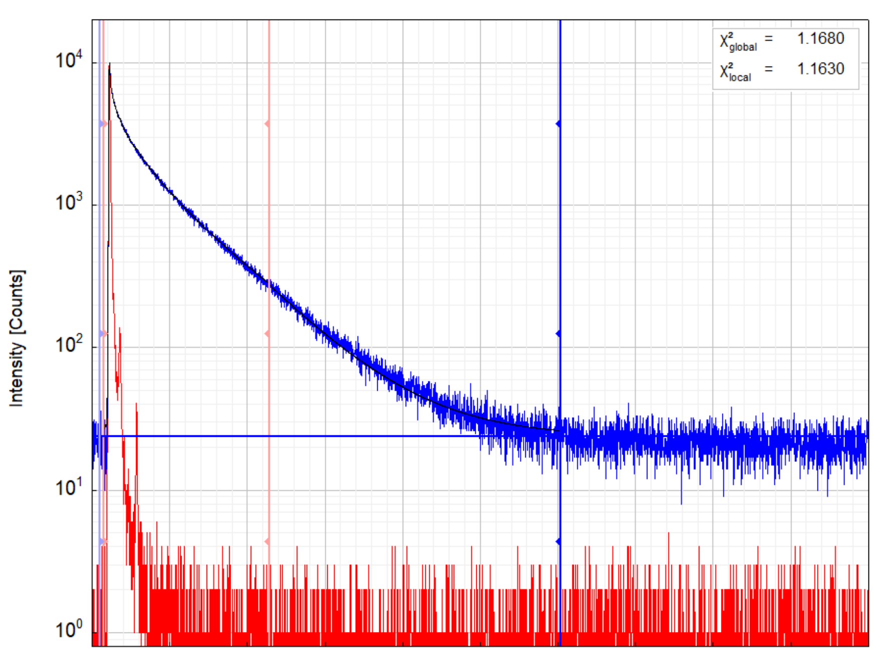

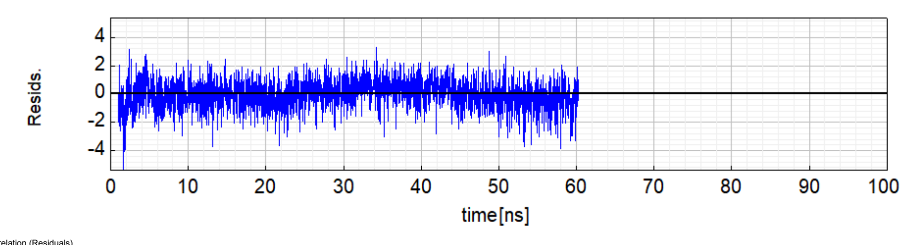

Autocorrelation (Residuals)

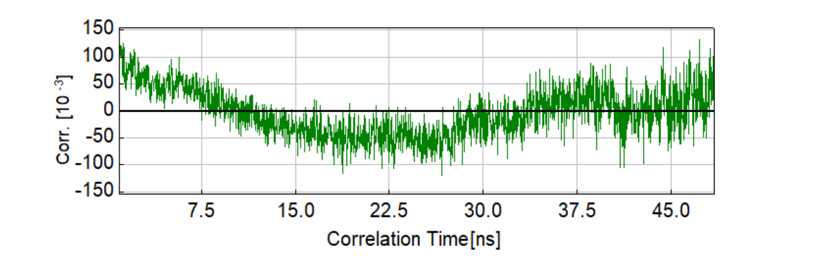

Fitted Parameters

Parameter	Value	Δ	δ
A_1[kCnts/Chnl]	2.085	±0.036	1.7%
τ_1[ns]	2.505	±0.013	0.5%
I_1[kCnts]	208.9	±3.6	1.7%
A_2[kCnts/Chnl]	2.863	±0.013	0.4%
τ_2[ns]	8.0397	±0.0067	0.1%
I_2[kCnts]	920.6	±5.5	0.6%
A_3[kCnts/Chnl]	3.315	±0.050	1.5%
τ_3[ns]	0.5435	±0.0070	1.3%
I_3[kCnts]	72.1	±1.6	2.1%
$Bkgr_{Dec}$[kCnts]	0.0238	±0.0003	1.2%
$Bkgr_{IRF}$[Cnts/Chnl]	-1.52	±0.21	14%
$Shift_{IRF}$[ps]	-1.47	±0.32	22%
A_{Scatt}[kCnts]	53.43	±0.44	0.8%
τ_{AvInt}[ns]	6.628	±0.017	0.2%
τ_{AvAmp}[ns]	3.636	±0.029	0.8%
λ_{Det}[nm]	450.0000	---	---

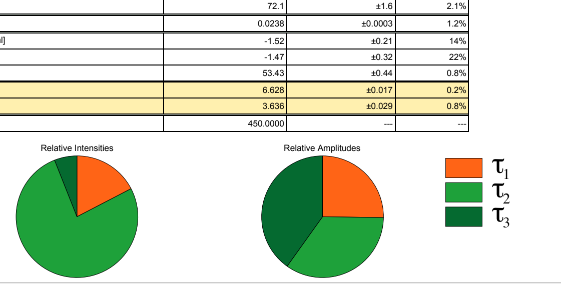

Data Set: 7 / 31

Decay: crv[7]; IRF: crv[0]

Fit

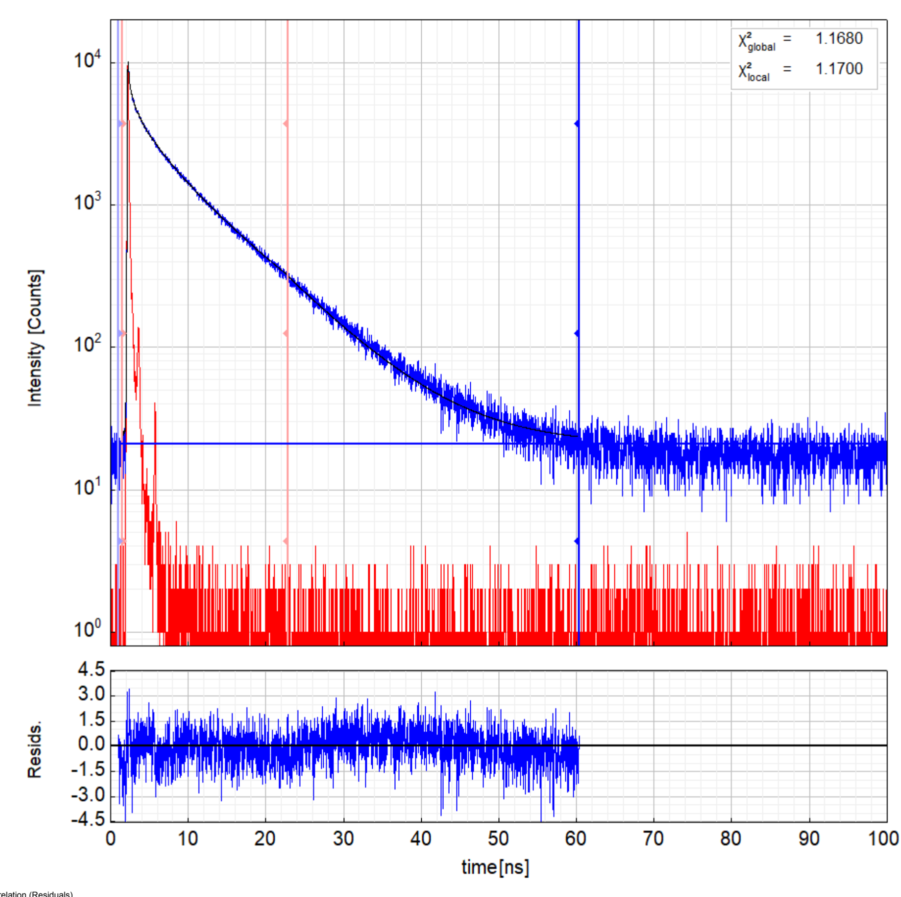

Autocorrelation (Residuals)

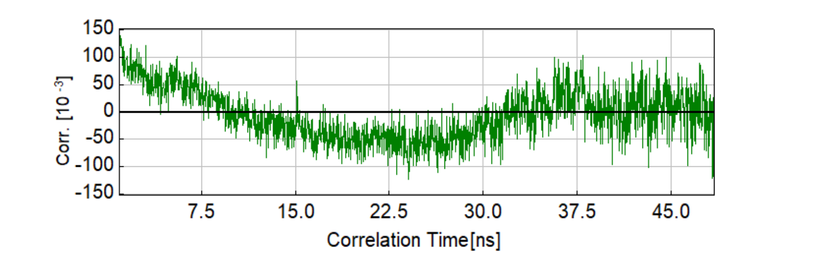

Fitted Parameters

Parameter	Value	Δ	δ
A_1[kCnts/Chnl]	2.000	±0.036	1.8%
τ_1[ns]	2.505	±0.013	0.5%
I_1[kCnts]	200.4	±4.1	2.0%
A_2[kCnts/Chnl]	3.3418	±0.0097	0.3%
τ_2[ns]	8.0397	±0.0067	0.1%
I_2[kCnts]	1 074.7	±2.3	0.2%
A_3[kCnts/Chnl]	3.382	±0.083	2.4%
τ_3[ns]	0.5435	±0.0070	1.3%
I_3[kCnts]	73.6	±2.5	3.3%
$Bkgr_{Dec}$[kCnts]	0.0209	±0.0004	1.5%
$Bkgr_{IRF}$[Cnts/Chnl]	-1.692	±0.086	5.1%
$Shift_{IRF}$[ps]	0.8	±1.3	171%
A_{Scatt}[kCnts]	53.2	±1.3	2.3%
τ_{AvInt}[ns]	6.8088	±0.0075	0.1%
τ_{AvAmp}[ns]	3.866	±0.028	0.7%
λ_{Det}[nm]	460.0000	---	---

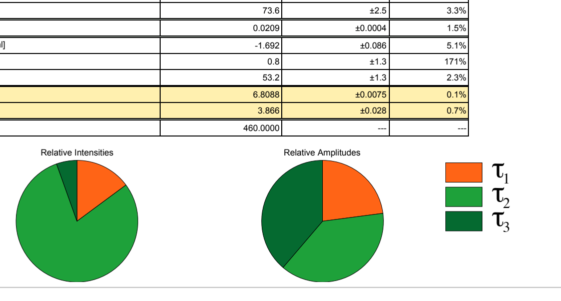

Data Set: 8 / 31

Decay: crv[8]; IRF: crv[0]

Fit

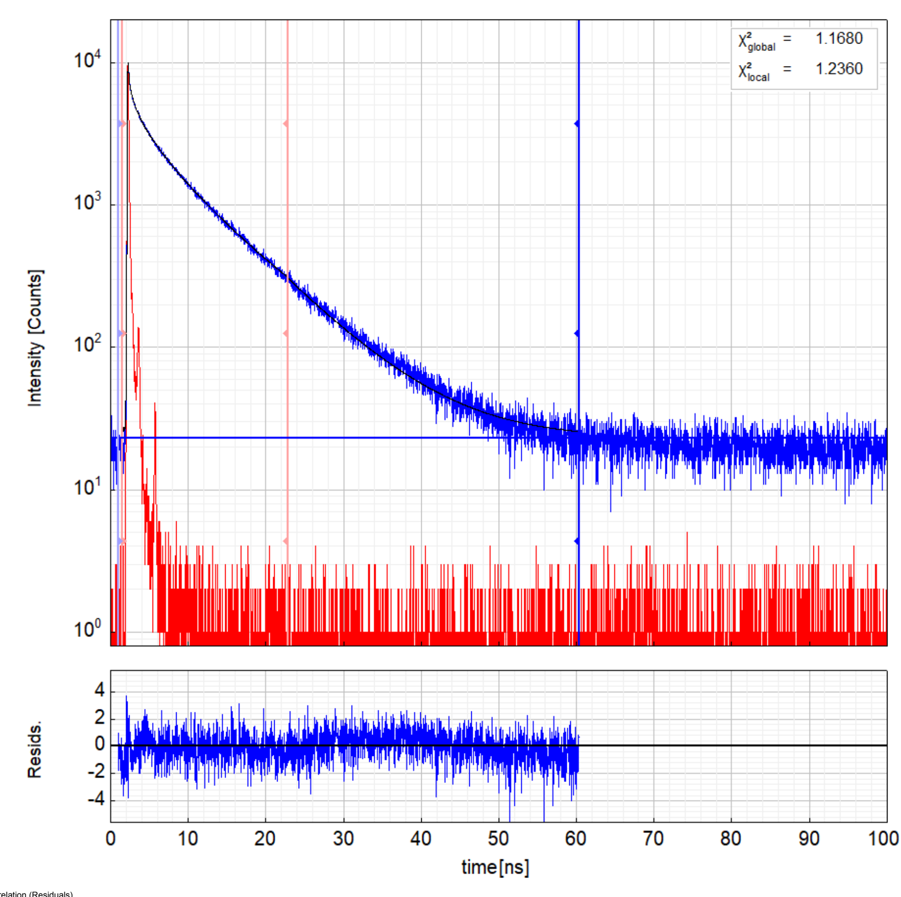

Autocorrelation (Residuals)

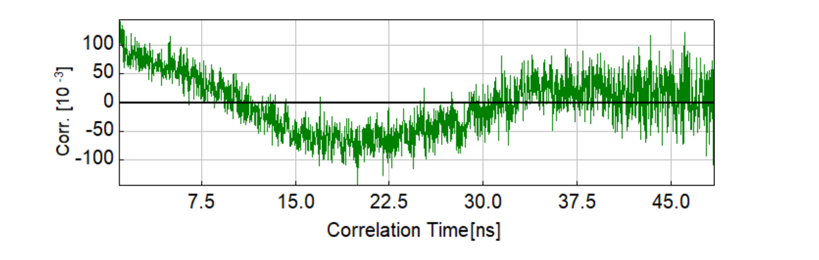

Fitted Parameters

Parameter	Value	Δ	δ
A_1[kCnts/Chnl]	2.200	±0.037	1.7%
τ_1[ns]	2.505	±0.013	0.5%
I_1[kCnts]	220.5	±2.7	1.2%
A_2[kCnts/Chnl]	3.253	±0.012	0.4%
τ_2[ns]	8.0397	±0.0067	0.1%
I_2[kCnts]	1 045.9	±4.2	0.4%
A_3[kCnts/Chnl]	3.154	±0.091	2.9%
τ_3[ns]	0.5435	±0.0070	1.3%
I_3[kCnts]	68.6	±2.5	3.6%
$Bkgr_{Dec}$[kCnts]	0.0230	±0.0002	0.5%
$Bkgr_{IRF}$[Cnts/Chnl]	-1.33	±0.21	15%
$Shift_{IRF}$[ps]	0.8	±1.3	165%
A_{Scatt}[kCnts]	51.3	±1.9	3.6%
τ_{AvInt}[ns]	6.741	±0.014	0.2%
τ_{AvAmp}[ns]	3.878	±0.032	0.8%
λ_{Det}[nm]	470.0000	---	---

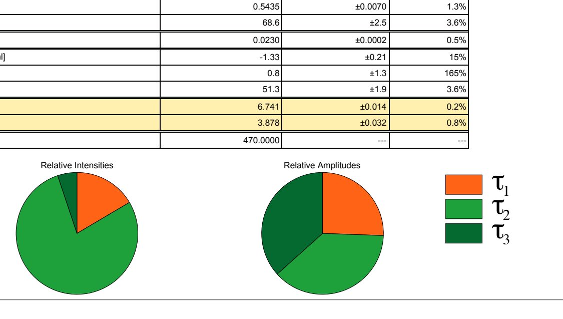

Data Set: 9 / 31

Decay: crv[9]; IRF: crv[0]

Fit

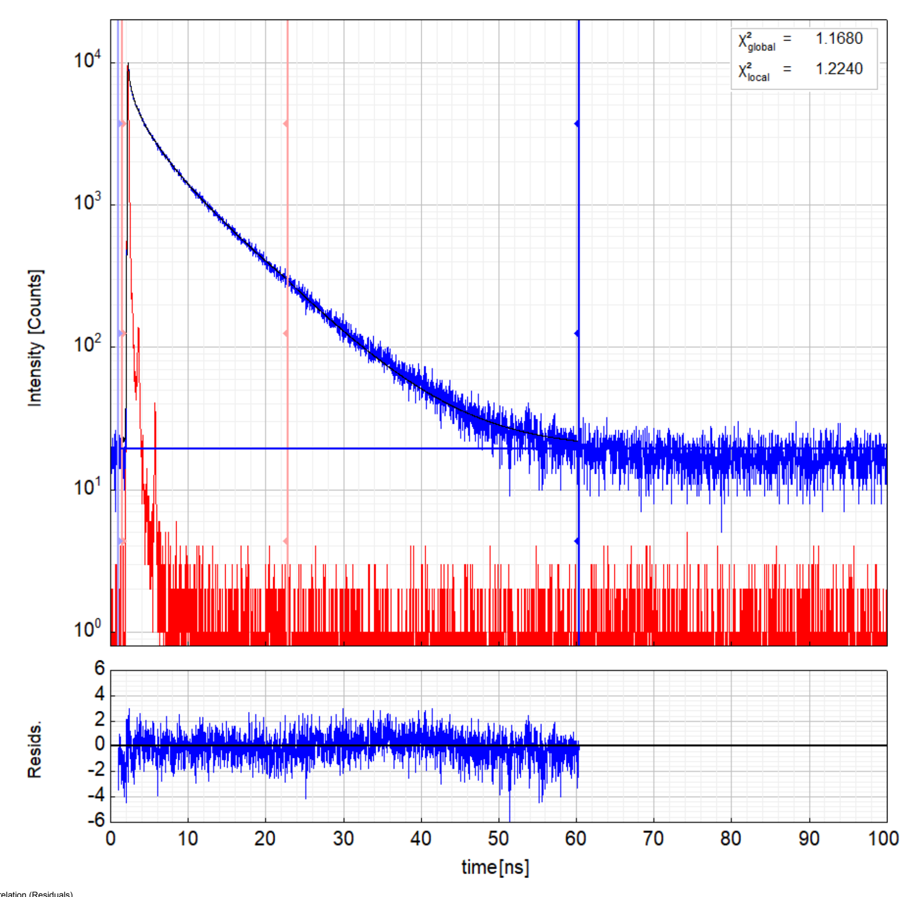

Autocorrelation (Residuals)

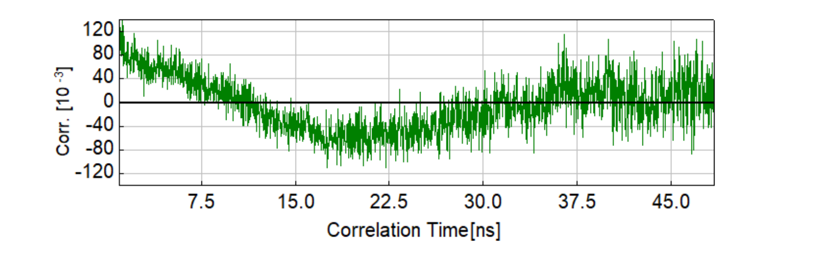

Fitted Parameters

Parameter	Value	Δ	δ
A_1[kCnts/Chnl]	2.574	±0.028	1.1%
τ_1[ns]	2.505	±0.013	0.5%
I_1[kCnts]	257.9	±2.0	0.7%
A_2[kCnts/Chnl]	3.2222	±0.0096	0.3%
τ_2[ns]	8.0397	±0.0067	0.1%
I_2[kCnts]	1 036.2	±3.4	0.3%
A_3[kCnts/Chnl]	3.484	±0.053	1.5%
τ_3[ns]	0.5435	±0.0070	1.3%
I_3[kCnts]	75.8	±2.0	2.6%
$Bkgr_{Dec}$[kCnts]	0.0194	±0.0003	1.1%
$Bkgr_{IRF}$[Cnts/Chnl]	-0.87	±0.12	14%
$Shift_{IRF}$[ps]	0.63	±0.74	117%
A_{Scatt}[kCnts]	49.4	±1.2	2.3%
τ_{AvInt}[ns]	6.584	±0.014	0.2%
τ_{AvAmp}[ns]	3.691	±0.016	0.4%
λ_{Det}[nm]	480.0000	---	---

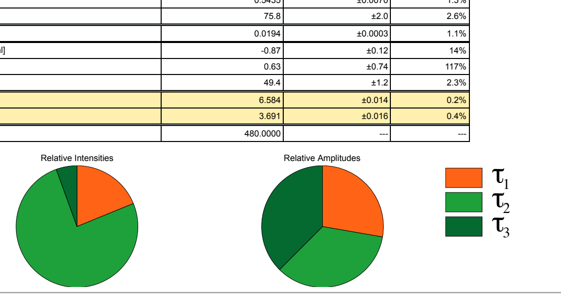

Data Set: 10 / 31

Decay: crv[10]; IRF: crv[0]

Fit

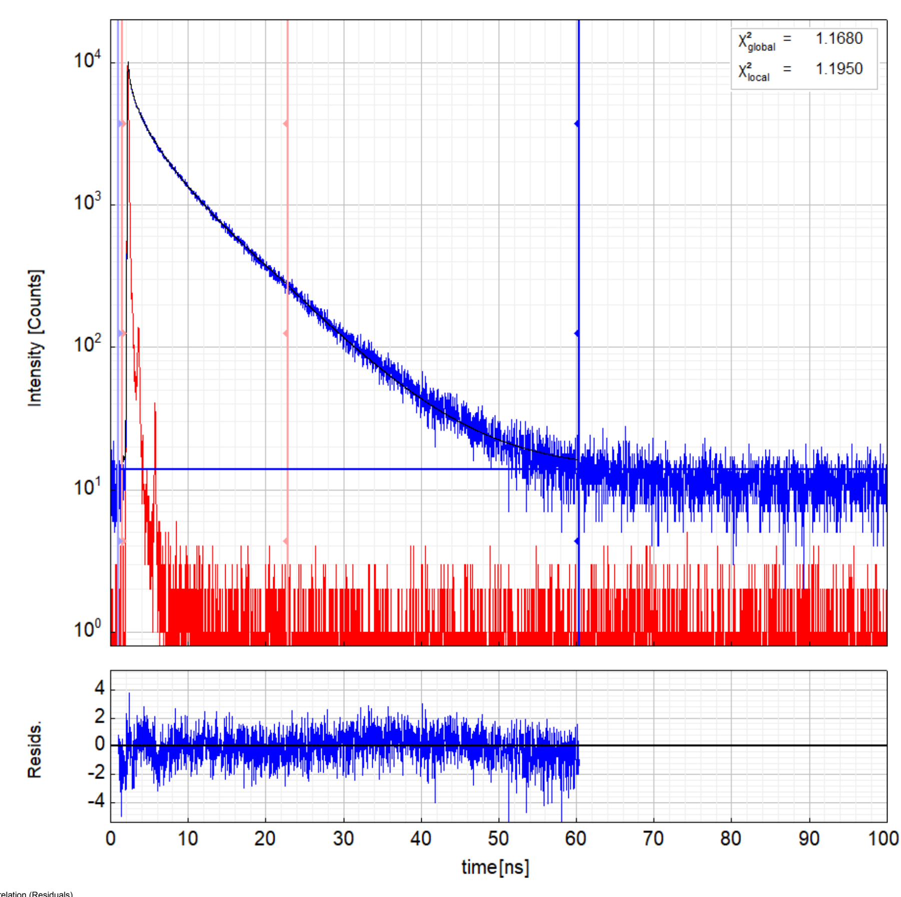

Autocorrelation (Residuals)

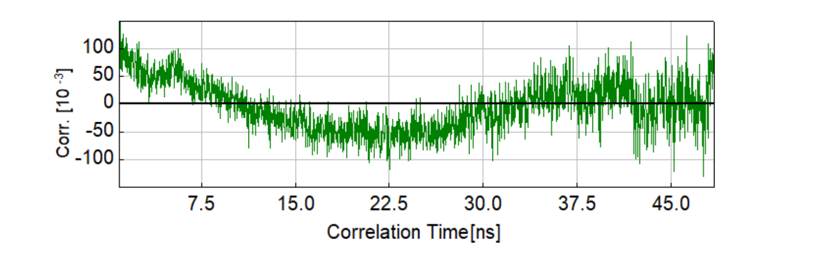

Fitted Parameters

Parameter	Value	Δ	δ
A_1[kCnts/Chnl]	2.975	±0.052	1.7%
τ_1[ns]	2.505	±0.013	0.5%
I_1[kCnts]	298.1	±6.0	2.0%
A_2[kCnts/Chnl]	3.053	±0.015	0.5%
τ_2[ns]	8.0397	±0.0067	0.1%
I_2[kCnts]	981.6	±5.9	0.6%
A_3[kCnts/Chnl]	3.55	±0.17	4.7%
τ_3[ns]	0.5435	±0.0070	1.3%
I_3[kCnts]	77.1	±3.7	4.8%
$Bkgr_{Dec}$[kCnts]	0.0139	±0.0002	1.1%
$Bkgr_{IRF}$[Cnts/Chnl]	-0.59	±0.18	30%
$Shift_{IRF}$[ps]	2.3	±1.1	45%
A_{Scatt}[kCnts]	48.78	±0.60	1.2%
τ_{AvInt}[ns]	6.3979	±0.0082	0.1%
τ_{AvAmp}[ns]	3.544	±0.031	0.9%
λ_{Det}[nm]	490.0000	---	---

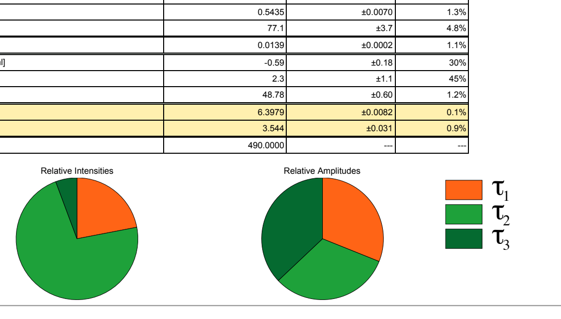

Data Set: 11 / 31

Decay: crv[11]; IRF: crv[0]

Fit

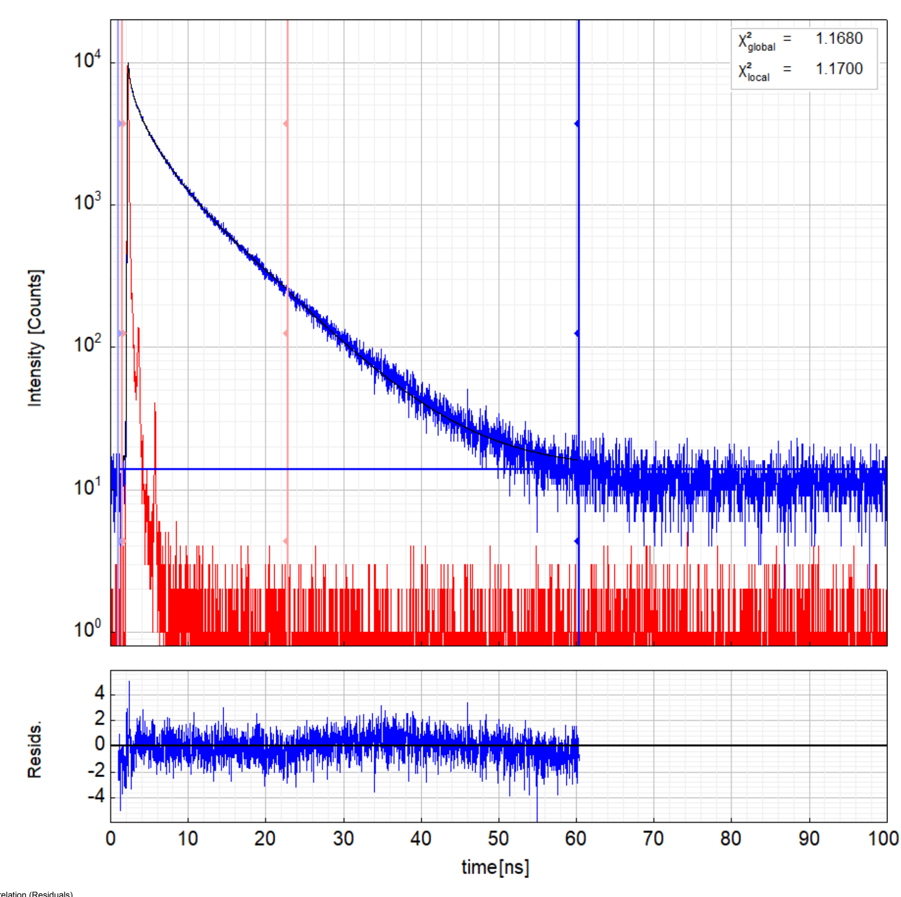

Autocorrelation (Residuals)

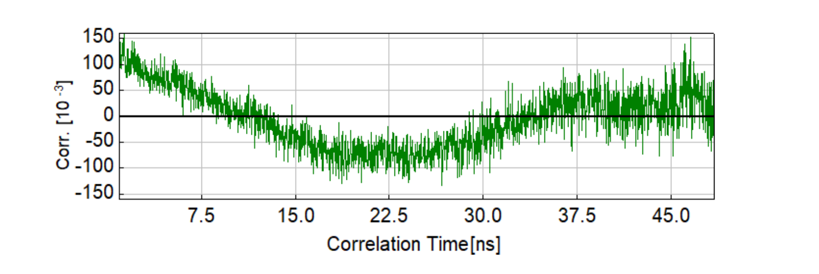

Fitted Parameters

Parameter	Value	Δ	δ
A_1[kCnts/Chnl]	3.356	±0.021	0.6%
τ_1[ns]	2.505	±0.013	0.5%
I_1[kCnts]	336.3	±2.8	0.8%
A_2[kCnts/Chnl]	2.7948	±0.0082	0.3%
τ_2[ns]	8.0397	±0.0067	0.1%
I_2[kCnts]	898.8	±4.2	0.5%
A_3[kCnts/Chnl]	3.560	±0.037	1.0%
τ_3[ns]	0.5435	±0.0070	1.3%
I_3[kCnts]	77.4	±1.2	1.4%
$Bkgr_{Dec}$[kCnts]	0.0140	±0.0002	1.3%
$Bkgr_{IRF}$[Cnts/Chnl]	-0.60	±0.11	18%
$Shift_{IRF}$[ps]	3.3	±1.1	31%
A_{Scatt}[kCnts]	47.23	±0.64	1.3%
τ_{AvInt}[ns]	6.180	±0.011	0.2%
τ_{AvAmp}[ns]	3.3790	±0.0092	0.3%
λ_{Det}[nm]	500.0000	---	---

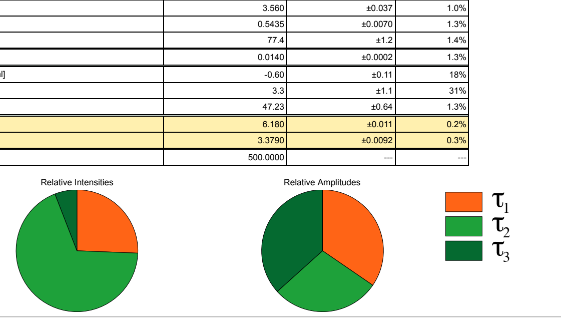

Data Set: 12 / 31

Decay: crv[12]; IRF: crv[0]

Fit

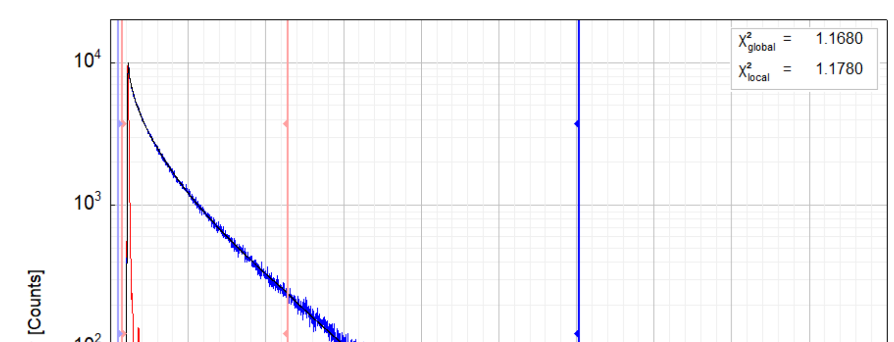

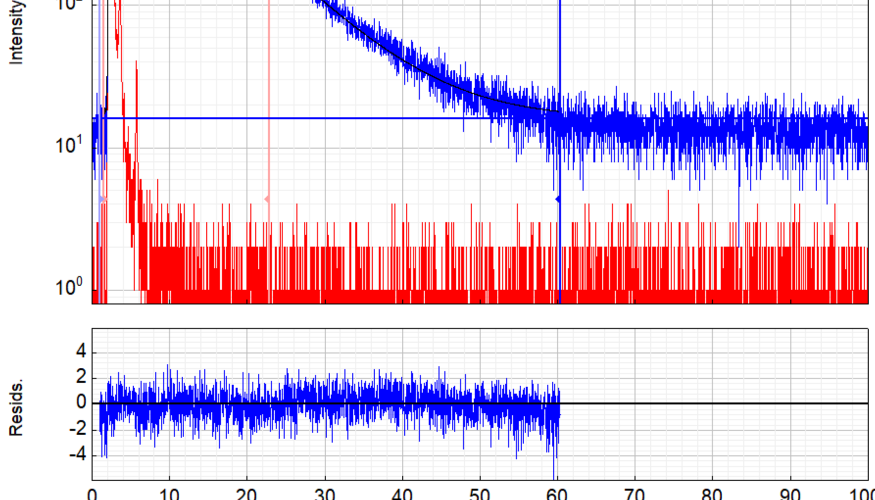

Autocorrelation (Residuals)

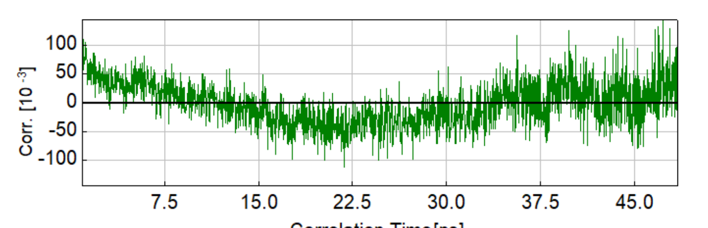

Fitted Parameters

Parameter	Value	Δ	δ
A_1[kCnts/Chnl]	3.592	±0.037	1.0%
τ_1[ns]	2.505	±0.013	0.5%
I_1[kCnts]	359.9	±3.0	0.8%
A_2[kCnts/Chnl]	2.650	±0.015	0.6%
τ_2[ns]	8.0397	±0.0067	0.1%
I_2[kCnts]	852.0	±4.2	0.5%
A_3[kCnts/Chnl]	3.640	±0.079	2.1%
τ_3[ns]	0.5435	±0.0070	1.3%
I_3[kCnts]	79.2	±1.3	1.5%
$Bkgr_{Dec}$[kCnts]	0.0159	±0.0004	2.0%
$Bkgr_{IRF}$[Cnts/Chnl]	-0.34	±0.14	39%
$Shift_{IRF}$[ps]	3.08	±0.82	26%
A_{Scatt}[kCnts]	46.44	±0.53	1.1%
τ_{AvInt}[ns]	6.038	±0.016	0.3%
τ_{AvAmp}[ns]	3.267	±0.019	0.6%
λ_{Det}[nm]	510.0000	---	---

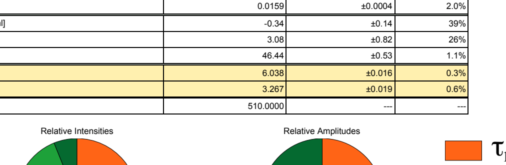

Data Set: 13 / 31

Decay: crv[13]; IRF: crv[0]

Fit

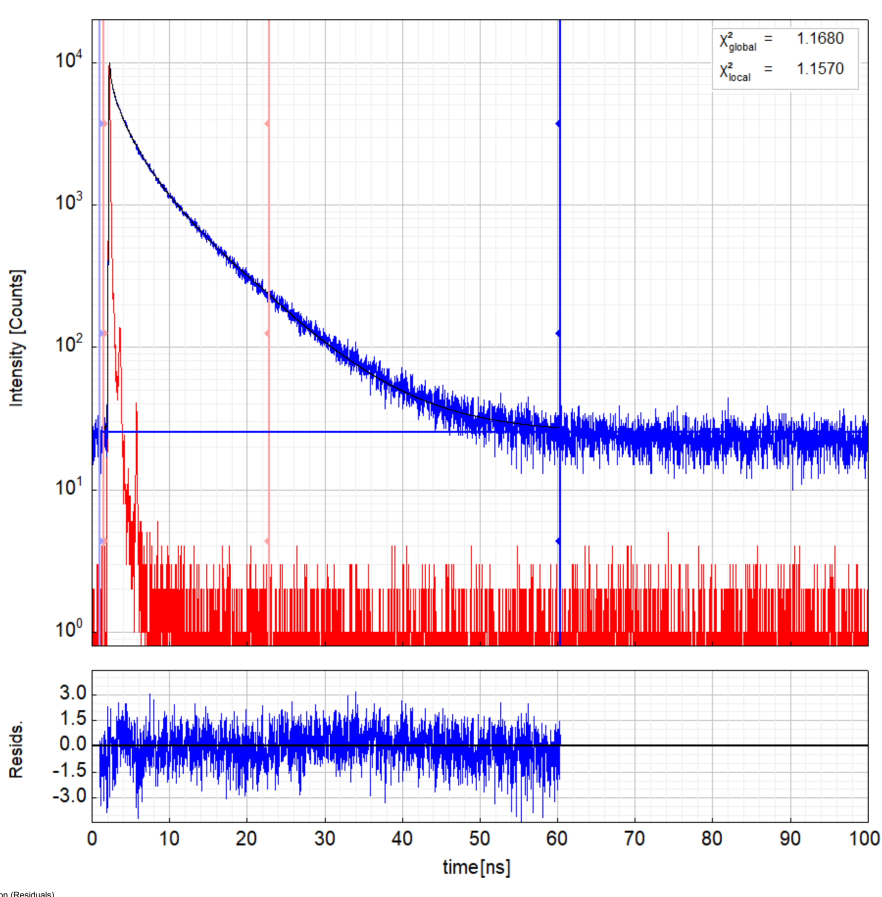

Autocorrelation (Residuals)

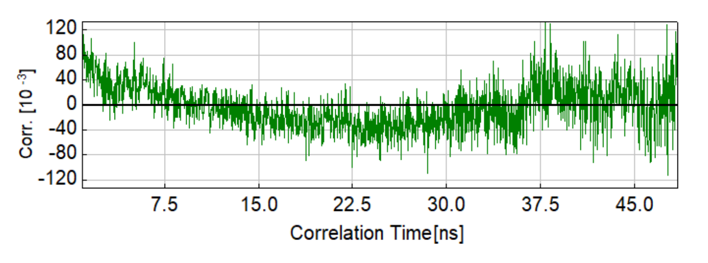

Fitted Parameters

Parameter	Value	Δ	δ
A_1[kCnts/Chnl]	3.636	±0.019	0.5%
τ_1[ns]	2.505	±0.013	0.5%
I_1[kCnts]	364.3	±2.7	0.7%
A_2[kCnts/Chnl]	2.499	±0.014	0.5%
τ_2[ns]	8.0397	±0.0067	0.1%
I_2[kCnts]	803.5	±4.2	0.5%
A_3[kCnts/Chnl]	3.522	±0.059	1.7%
τ_3[ns]	0.5435	±0.0070	1.3%
I_3[kCnts]	76.6	±1.3	1.6%
$Bkgr_{Dec}$[kCnts]	0.0254	±0.0003	0.8%
$Bkgr_{IRF}$[Cnts/Chnl]	-0.17	±0.15	89%
$Shift_{IRF}$[ps]	5.56	±0.33	5.9%
A_{Scatt}[kCnts]	46.65	±0.63	1.3%
τ_{AvInt}[ns]	5.959	±0.011	0.2%
τ_{AvAmp}[ns]	3.222	±0.013	0.4%
λ_{Det}[nm]	520.0000	---	---

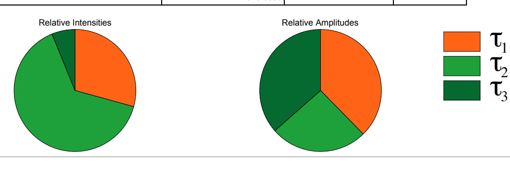

Data Set: 14 / 31

Decay: crv[14]; IRF: crv[0]

Fit

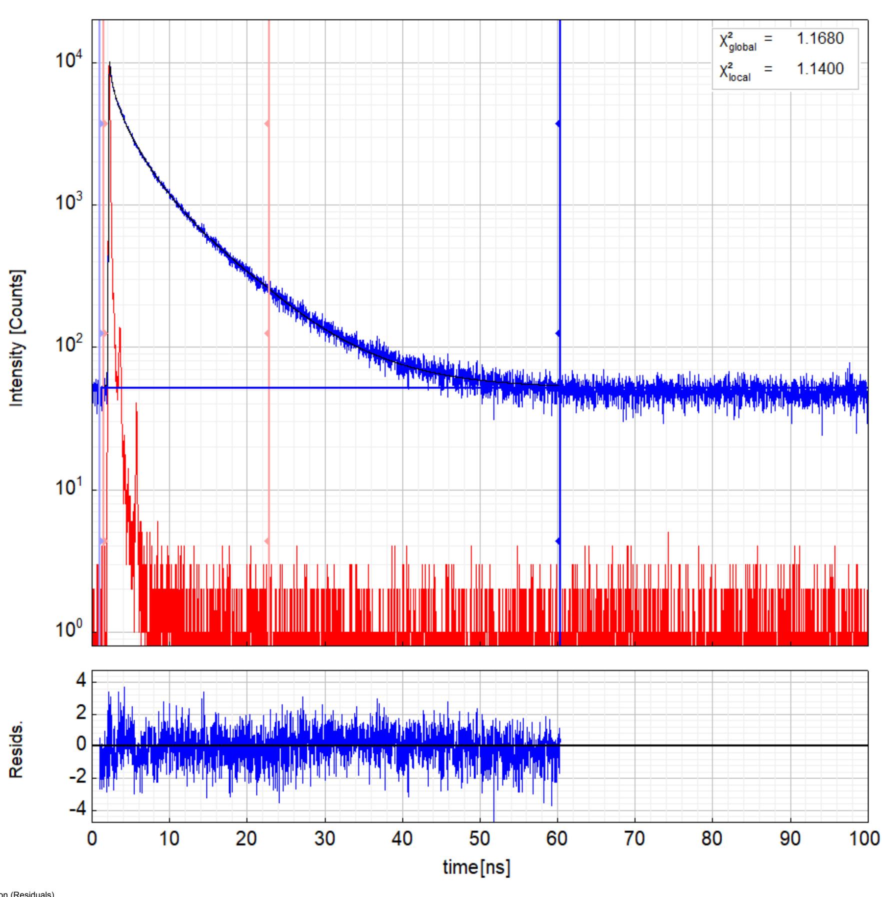

Autocorrelation (Residuals)

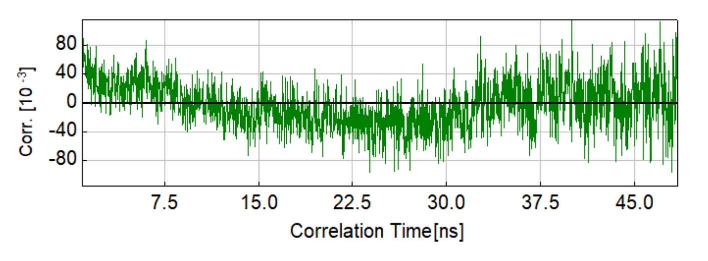

Fitted Parameters

Parameter	Value	Δ	δ
A_1[kCnts/Chnl]	3.792	±0.055	1.4%
τ_1[ns]	2.505	±0.013	0.5%
I_1[kCnts]	379.9	±4.7	1.2%
A_2[kCnts/Chnl]	2.453	±0.015	0.6%
τ_2[ns]	8.0397	±0.0067	0.1%
I_2[kCnts]	788.9	±4.9	0.6%
A_3[kCnts/Chnl]	3.77	±0.11	2.9%
τ_3[ns]	0.5435	±0.0070	1.3%
I_3[kCnts]	81.9	±2.6	3.1%
$Bkgr_{Dec}$[kCnts]	0.0520	±0.0003	0.5%
$Bkgr_{IRF}$[Cnts/Chnl]	-0.21	±0.16	73%
$Shift_{IRF}$[ps]	6.6	±1.1	16%
A_{Scatt}[kCnts]	46.72	±0.48	1.0%
τ_{AvInt}[ns]	5.8677	±0.0065	0.1%
τ_{AvAmp}[ns]	3.123	±0.026	0.8%
λ_{Det}[nm]	530.0000	---	---

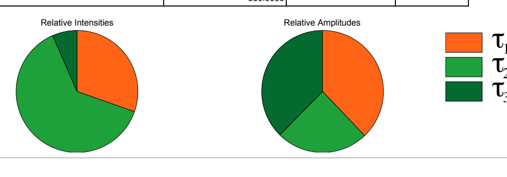

Data Set: 15 / 31

Decay: crv[15]; IRF: crv[0]

Fit

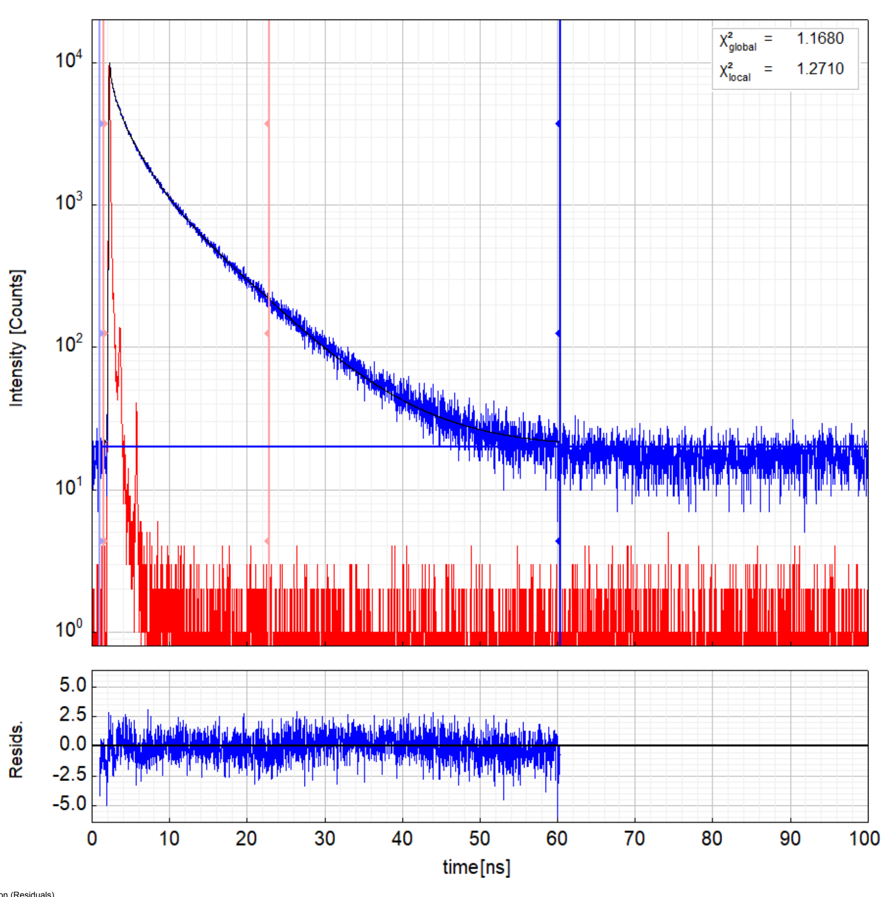

Autocorrelation (Residuals)

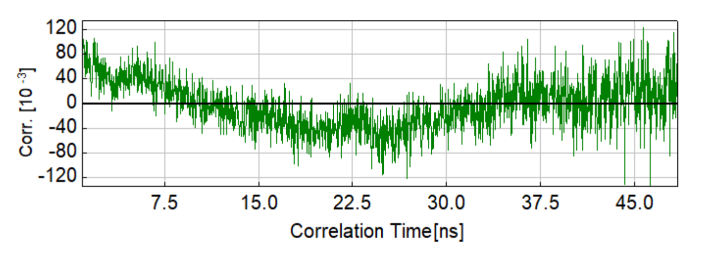

Fitted Parameters

Parameter	Value	Δ	δ
A_1[kCnts/Chnl]	3.755	±0.032	0.8%
τ_1[ns]	2.505	±0.013	0.5%
I_1[kCnts]	376.3	±2.2	0.6%
A_2[kCnts/Chnl]	2.376	±0.012	0.5%
τ_2[ns]	8.0397	±0.0067	0.1%
I_2[kCnts]	764.1	±4.2	0.5%
A_3[kCnts/Chnl]	3.829	±0.093	2.4%
τ_3[ns]	0.5435	±0.0070	1.3%
I_3[kCnts]	83.3	±1.8	2.1%
$Bkgr_{Dec}$[kCnts]	0.0199	±0.0003	1.3%
$Bkgr_{IRF}$[Cnts/Chnl]	-0.11	±0.12	104%
$Shift_{IRF}$[ps]	7.53	±0.74	9.7%
A_{Scatt}[kCnts]	45.94	±0.50	1.1%
τ_{AvInt}[ns]	5.828	±0.015	0.3%
τ_{AvAmp}[ns]	3.072	±0.023	0.7%
λ_{Det}[nm]	540.0000	---	---

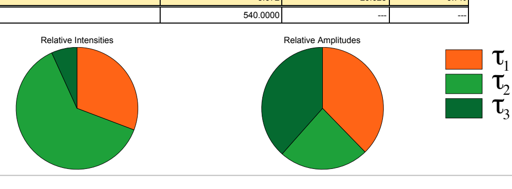

Data Set: 16 / 31

Decay: crv[16]; IRF: crv[0]

Fit

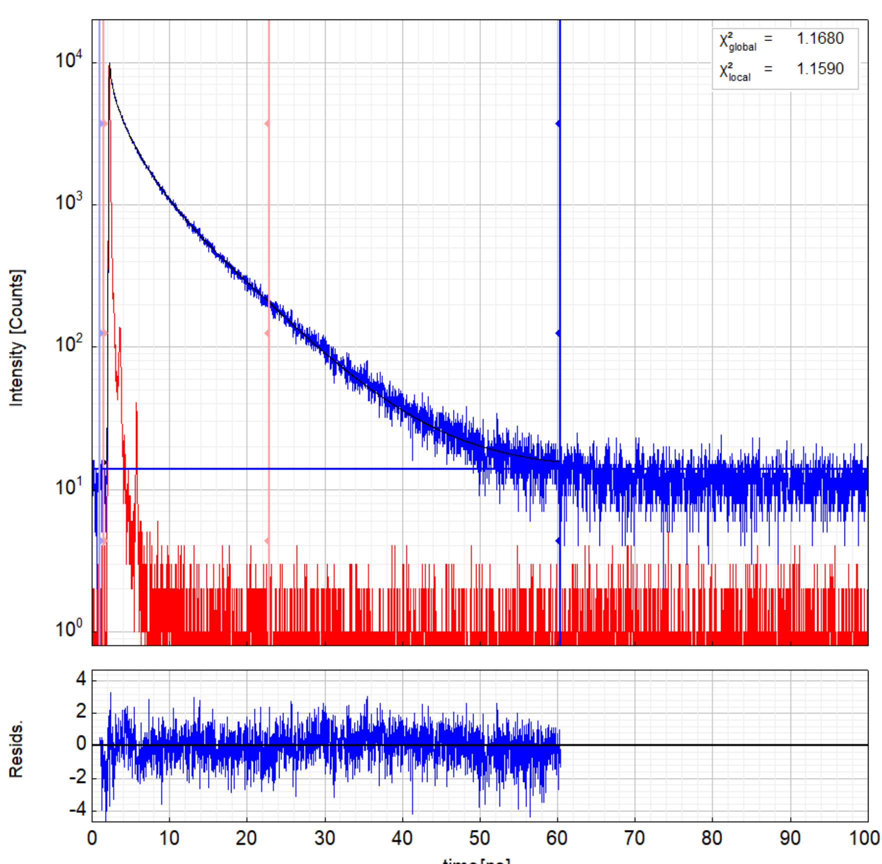

Autocorrelation (Residuals)

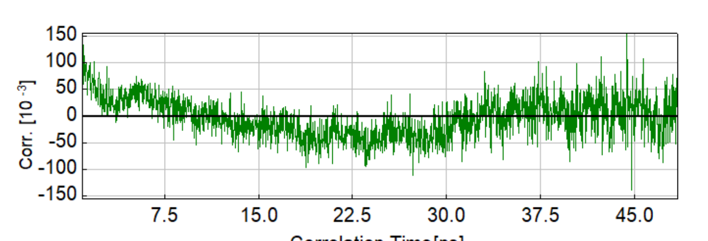

Fitted Parameters

Parameter	Value	Δ	δ
A_1[kCnts/Chnl]	3.640	±0.027	0.7%
τ_1[ns]	2.505	±0.013	0.5%
I_1[kCnts]	364.7	±3.3	0.9%
A_2[kCnts/Chnl]	2.3325	±0.0070	0.3%
τ_2[ns]	8.0397	±0.0067	0.1%
I_2[kCnts]	750.1	±3.6	0.5%
A_3[kCnts/Chnl]	3.799	±0.098	2.6%
τ_3[ns]	0.5435	±0.0070	1.3%
I_3[kCnts]	82.6	±2.5	3.0%
$Bkgr_{Dec}$[kCnts]	0.0139	±0.0002	1.4%
$Bkgr_{IRF}$[Cnts/Chnl]	0.04	±0.13	358%
$Shift_{IRF}$[ps]	7.95	±0.25	3.1%
A_{Scatt}[kCnts]	45.70	±0.51	1.1%
τ_{AvInt}[ns]	5.837	±0.012	0.2%
τ_{AvAmp}[ns]	3.064	±0.024	0.8%
λ_{Det}[nm]	550.0000	---	---

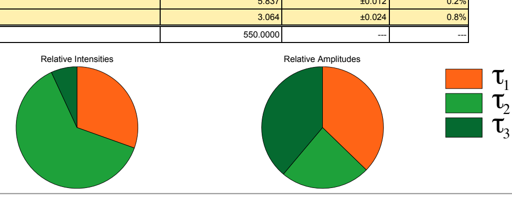

Data Set: 17 / 31

Decay: crv[17]; IRF: crv[0]

Fit

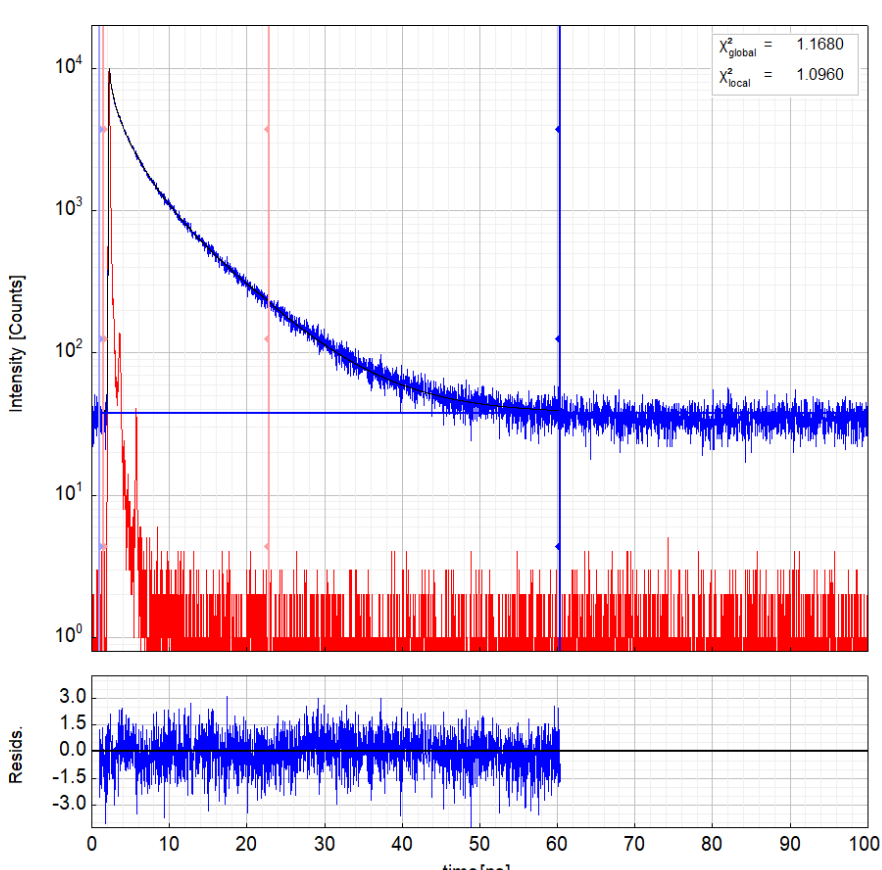

Autocorrelation (Residuals)

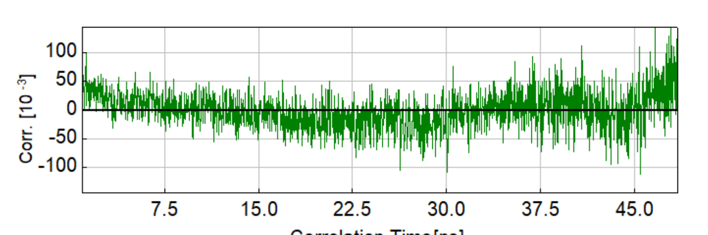

Fitted Parameters

Parameter	Value	Δ	δ
A_1[kCnts/Chnl]	3.633	±0.043	1.2%
τ_1[ns]	2.505	±0.013	0.5%
I_1[kCnts]	364.0	±2.1	0.6%
A_2[kCnts/Chnl]	2.298	±0.011	0.5%
τ_2[ns]	8.0397	±0.0067	0.1%
I_2[kCnts]	738.9	±3.2	0.4%
A_3[kCnts/Chnl]	3.946	±0.026	0.6%
τ_3[ns]	0.5435	±0.0070	1.3%
I_3[kCnts]	85.8	±1.7	2.0%
$Bkgr_{Dec}$[kCnts]	0.0377	±0.0002	0.5%
$Bkgr_{IRF}$[Cnts/Chnl]	0.006	±0.081	1489%
$Shift_{IRF}$[ps]	8.67	±0.57	6.5%
A_{Scatt}[kCnts]	45.74	±0.51	1.1%
τ_{AvInt}[ns]	5.804	±0.012	0.2%
τ_{AvAmp}[ns]	3.009	±0.012	0.4%
λ_{Det}[nm]	560.0000	---	---

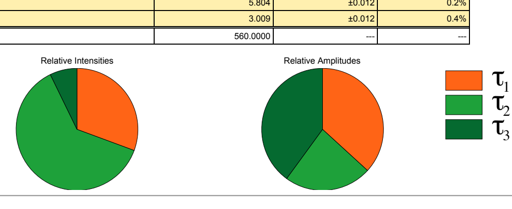

Data Set: 18 / 31

Decay: crv[18]; IRF: crv[0]

Fit

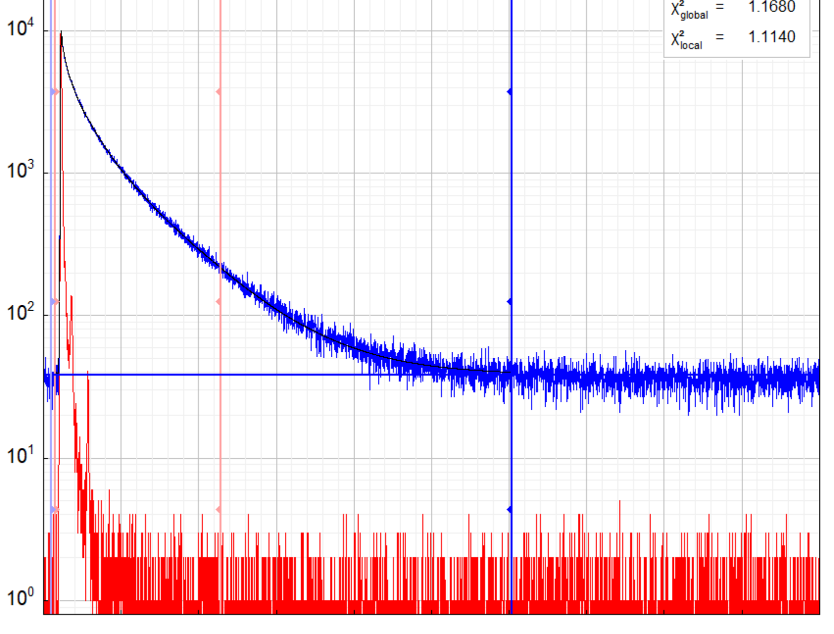

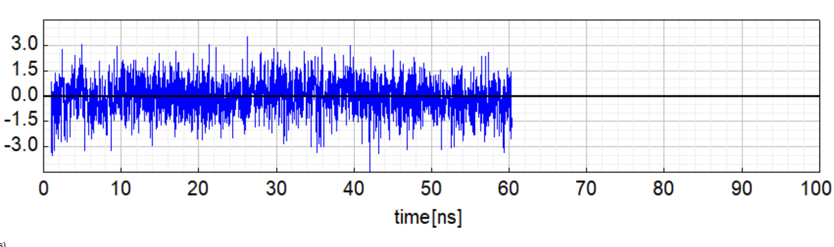

Autocorrelation (Residuals)

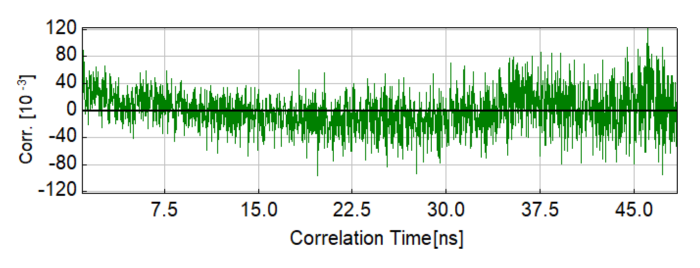

Fitted Parameters

Parameter	Value	Δ	δ
A_1[kCnts/Chnl]	3.493	±0.045	1.3%
τ_1[ns]	2.505	±0.013	0.5%
I_1[kCnts]	350.0	±2.8	0.8%
A_2[kCnts/Chnl]	2.226	±0.011	0.5%
τ_2[ns]	8.0397	±0.0067	0.1%
I_2[kCnts]	715.7	±2.4	0.3%
A_3[kCnts/Chnl]	4.150	±0.078	1.9%
τ_3[ns]	0.5435	±0.0070	1.3%
I_3[kCnts]	90.2	±3.0	3.2%
$Bkgr_{Dec}$[kCnts]	0.0385	±0.0003	0.6%
$Bkgr_{IRF}$[Cnts/Chnl]	0.512	±0.081	16%
$Shift_{IRF}$[ps]	9.09	±0.77	8.5%
A_{Scatt}[kCnts]	45.5	±1.3	2.8%
τ_{AvInt}[ns]	5.779	±0.011	0.2%
τ_{AvAmp}[ns]	2.929	±0.022	0.7%
λ_{Det}[nm]	570.0000	---	---

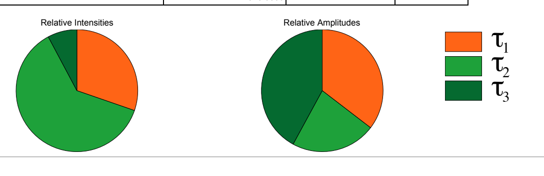

Data Set: 19 / 31

Decay: crv[19]; IRF: crv[0]

Fit

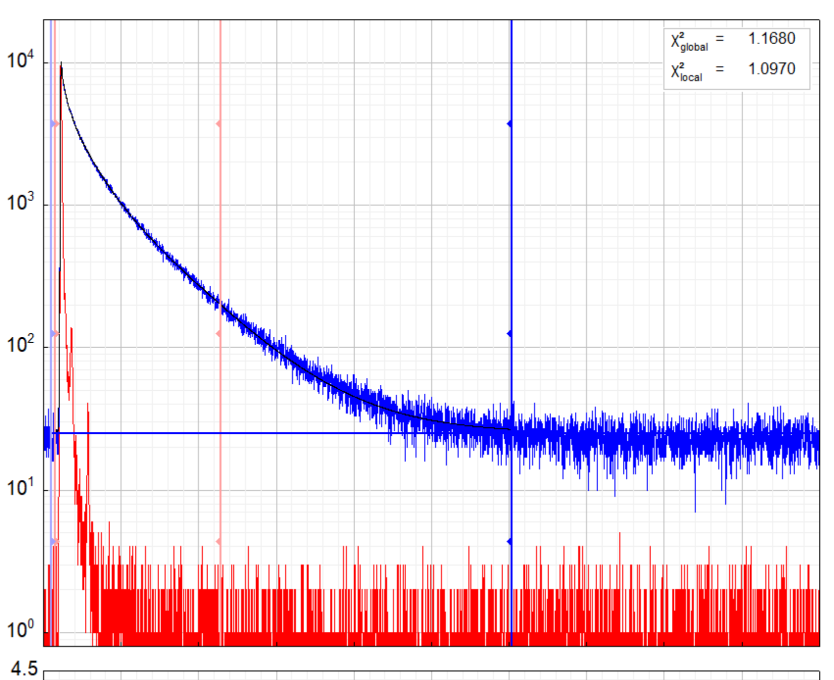

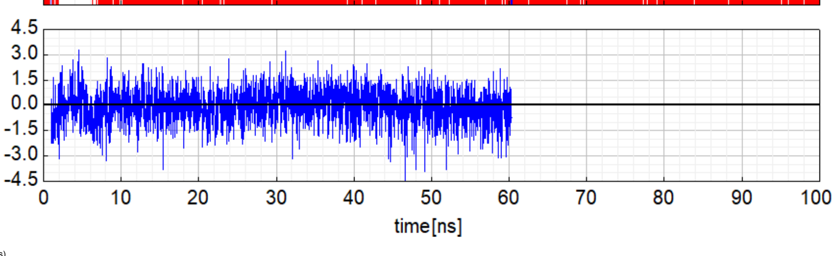

Autocorrelation (Residuals)

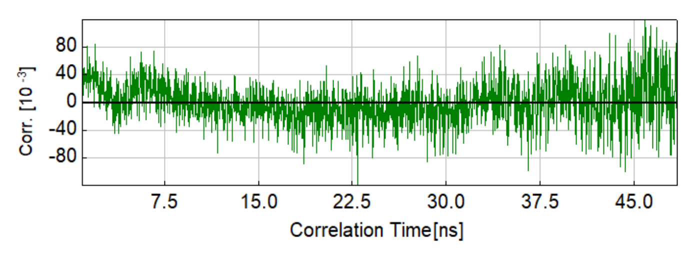

Fitted Parameters

Parameter	Value	Δ	δ
A_1[kCnts/Chnl]	3.463	±0.047	1.4%
τ_1[ns]	2.505	±0.013	0.5%
I_1[kCnts]	347.0	±3.8	1.1%
A_2[kCnts/Chnl]	2.178	±0.011	0.5%
τ_2[ns]	8.0397	±0.0067	0.1%
I_2[kCnts]	700.3	±3.1	0.4%
A_3[kCnts/Chnl]	4.265	±0.060	1.4%
τ_3[ns]	0.5435	±0.0070	1.3%
I_3[kCnts]	92.8	±1.2	1.2%
$Bkgr_{Dec}$[kCnts]	0.0251	±0.0003	1.1%
$Bkgr_{IRF}$[Cnts/Chnl]	0.49	±0.16	33%
$Shift_{IRF}$[ps]	8.92	±0.30	3.3%
A_{Scatt}[kCnts]	47.23	±0.75	1.6%
τ_{AvInt}[ns]	5.746	±0.015	0.3%
τ_{AvAmp}[ns]	2.8773	±0.0048	0.2%
λ_{Det}[nm]	580.0000	---	---

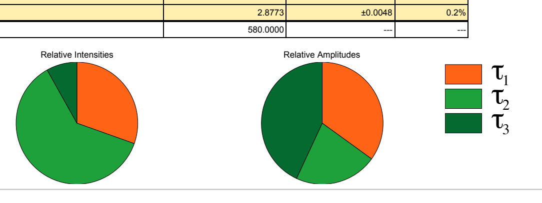

Data Set: 20 / 31

Decay: crv[20]; IRF: crv[0]

Fit

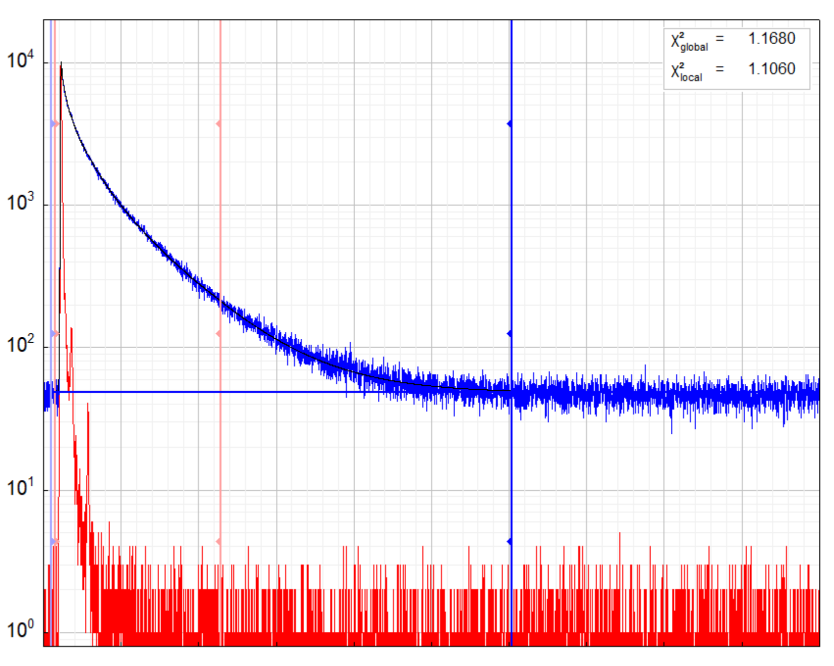

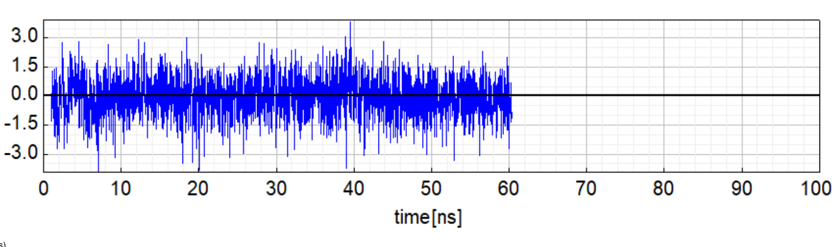

Autocorrelation (Residuals)

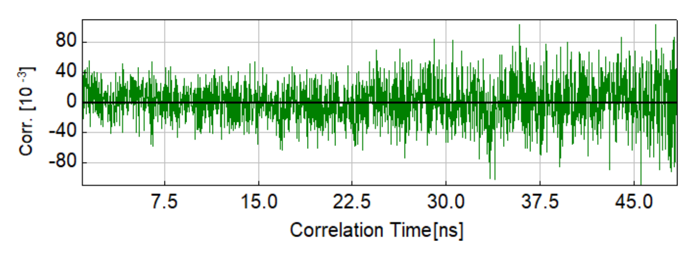

Fitted Parameters

Parameter	Value	Δ	δ
A_1[kCnts/Chnl]	3.295	±0.024	0.7%
τ_1[ns]	2.505	±0.013	0.5%
I_1[kCnts]	330.2	±2.6	0.8%
A_2[kCnts/Chnl]	2.057	±0.012	0.6%
τ_2[ns]	8.0397	±0.0067	0.1%
I_2[kCnts]	661.3	±3.7	0.5%
A_3[kCnts/Chnl]	4.394	±0.030	0.7%
τ_3[ns]	0.5435	±0.0070	1.3%
I_3[kCnts]	95.6	±1.2	1.2%
$Bkgr_{Dec}$[kCnts]	0.0483	±0.0004	0.7%
$Bkgr_{IRF}$[Cnts/Chnl]	0.626	±0.066	10%
$Shift_{IRF}$[ps]	10.12	±0.49	4.8%
A_{Scatt}[kCnts]	48.10	±0.33	0.7%
τ_{AvInt}[ns]	5.700	±0.018	0.3%
τ_{AvAmp}[ns]	2.7885	±0.0085	0.3%
λ_{Det}[nm]	590.0000	---	---

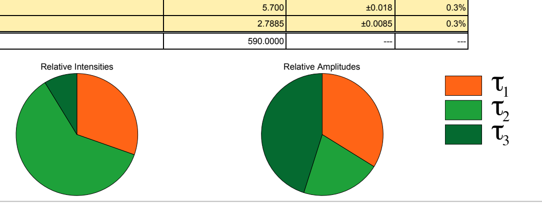

Data Set: 21 / 31

Decay: crv[21]; IRF: crv[0]

Fit

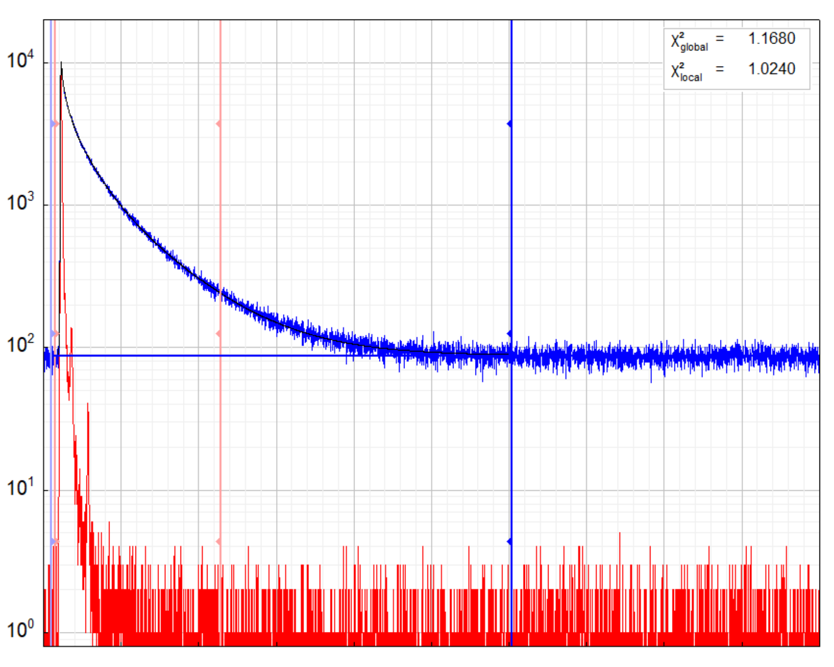

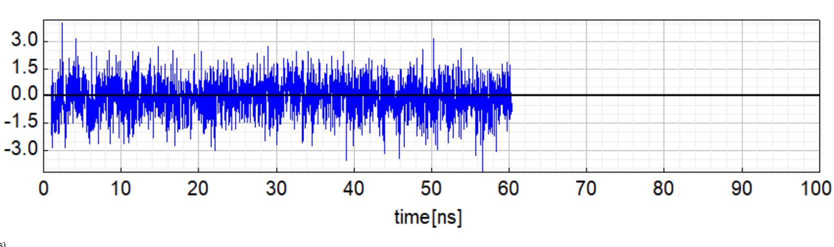

Autocorrelation (Residuals)

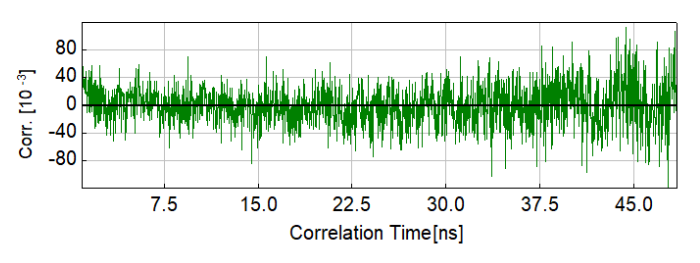

Fitted Parameters

Parameter	Value	Δ	δ
A_1[kCnts/Chnl]	3.214	±0.035	1.1%
τ_1[ns]	2.505	±0.013	0.5%
I_1[kCnts]	322.1	±3.3	1.0%
A_2[kCnts/Chnl]	1.945	±0.016	0.8%
τ_2[ns]	8.0397	±0.0067	0.1%
I_2[kCnts]	625.3	±5.0	0.8%
A_3[kCnts/Chnl]	4.40	±0.11	2.3%
τ_3[ns]	0.5435	±0.0070	1.3%
I_3[kCnts]	95.5	±3.4	3.5%
$Bkgr_{Dec}$[kCnts]	0.0878	±0.0003	0.3%
$Bkgr_{IRF}$[Cnts/Chnl]	0.76	±0.19	25%
$Shift_{IRF}$[ps]	10.93	±0.86	7.9%
A_{Scatt}[kCnts]	49.16	±0.52	1.0%
τ_{AvInt}[ns]	5.645	±0.013	0.2%
τ_{AvAmp}[ns]	2.730	±0.023	0.8%
λ_{Det}[nm]	600.0000	---	---

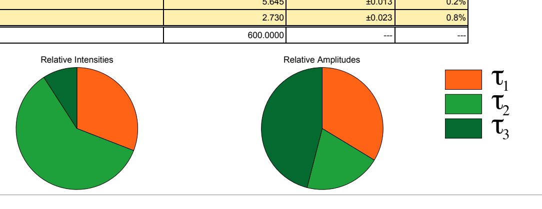

Data Set: 22 / 31

Decay: crv[22]; IRF: crv[0]

Fit

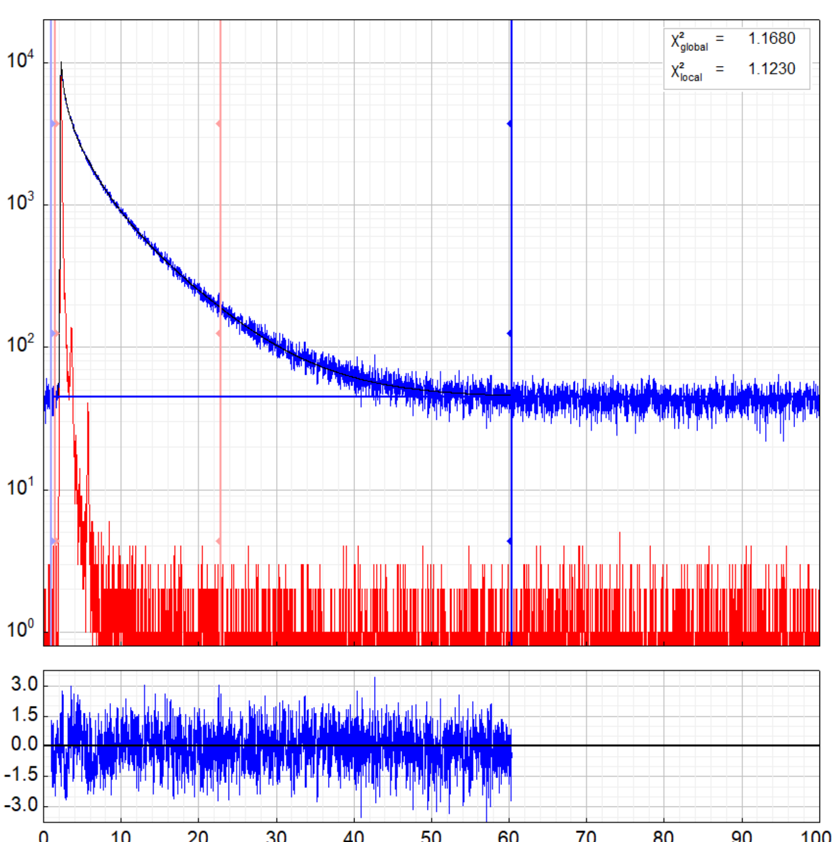

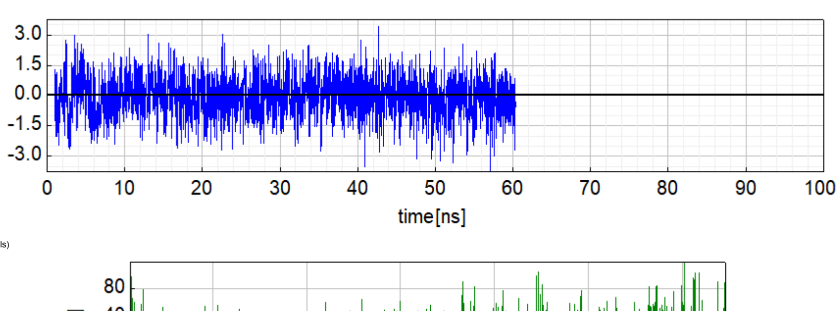

Autocorrelation (Residuals)

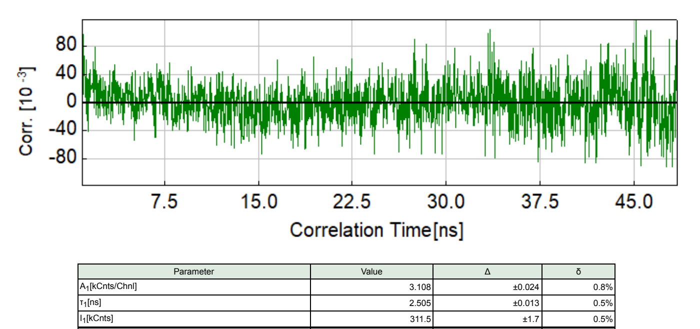

Fitted Parameters

Parameter	Value	Δ	δ
A_1[kCnts/Chnl]	3.108	±0.024	0.8%
τ_1[ns]	2.505	±0.013	0.5%
I_1[kCnts]	311.5	±1.7	0.5%
A_2[kCnts/Chnl]	1.8431	±0.0084	0.5%
τ_2[ns]	8.0397	±0.0067	0.1%
I_2[kCnts]	592.8	±2.5	0.4%
A_3[kCnts/Chnl]	4.598	±0.063	1.4%
τ_3[ns]	0.5435	±0.0070	1.3%
I_3[kCnts]	100.0	±1.5	1.5%
$Bkgr_{Dec}$[kCnts]	0.0446	±0.0004	0.9%
$Bkgr_{IRF}$[Cnts/Chnl]	0.79	±0.11	13%
$Shift_{IRF}$[ps]	10.74	±0.38	3.4%
A_{Scatt}[kCnts]	50.09	±0.48	0.9%
τ_{AvInt}[ns]	5.5769	±0.0066	0.1%
τ_{AvAmp}[ns]	2.629	±0.017	0.6%
λ_{Det}[nm]	610.0000	---	---

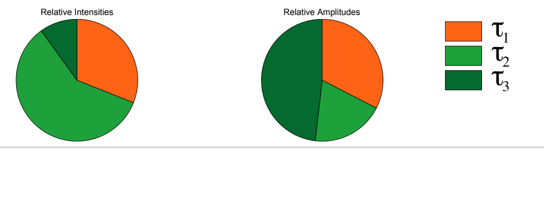

Data Set: 23 / 31

Decay: crv[23]; IRF: crv[0]

Fit

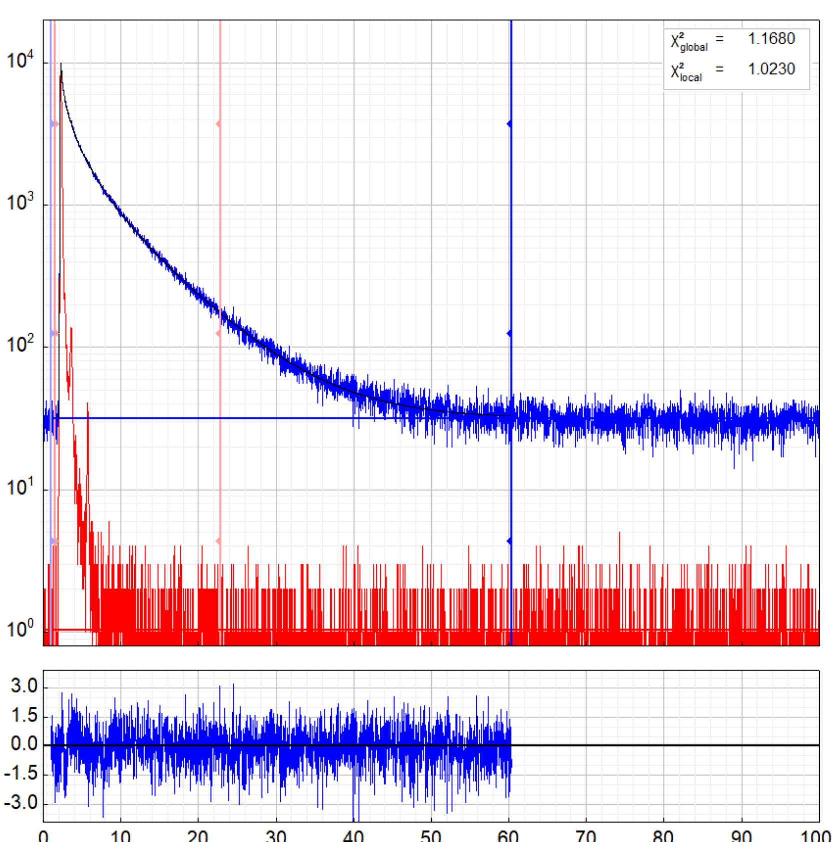

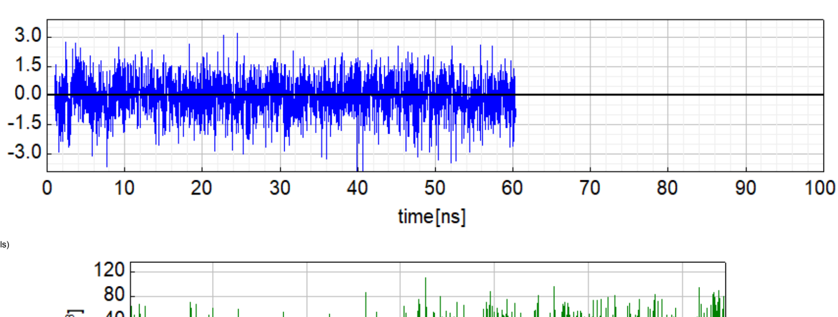

Autocorrelation (Residuals)

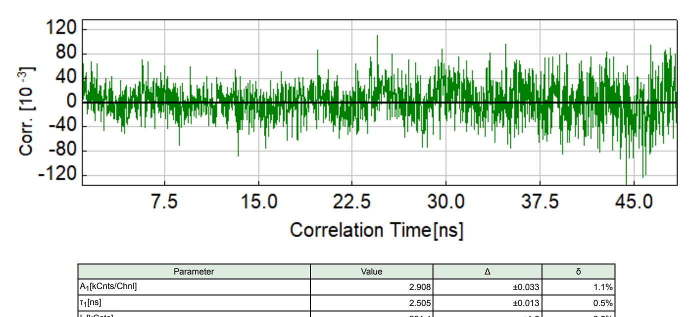

Fitted Parameters

Parameter	Value	Δ	δ
A_1[kCnts/Chnl]	2.908	±0.033	1.1%
τ_1[ns]	2.505	±0.013	0.5%
I_1[kCnts]	291.4	±1.6	0.5%
A_2[kCnts/Chnl]	1.8478	±0.0075	0.4%
τ_2[ns]	8.0397	±0.0067	0.1%
I_2[kCnts]	594.2	±2.1	0.3%
A_3[kCnts/Chnl]	4.470	±0.046	1.0%
τ_3[ns]	0.5435	±0.0070	1.3%
I_3[kCnts]	97.2	±1.7	1.7%
$Bkgr_{Dec}$[kCnts]	0.0316	±0.0004	1.0%
$Bkgr_{IRF}$[Cnts/Chnl]	1.04	±0.15	14%
$Shift_{IRF}$[ps]	12.85	±0.43	3.3%

A_{Scatt}[kCnts]	50.30	±0.24	0.5%
τ_{AvInt}[ns]	5.6577	±0.0100	0.2%
τ_{AvAmp}[ns]	2.664	±0.012	0.4%
λ_{Det}[nm]	620.0000	---	---

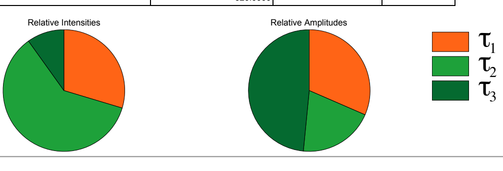

Data Set: 24 / 31

Decay: crv[24]; IRF: crv[0]

Fit

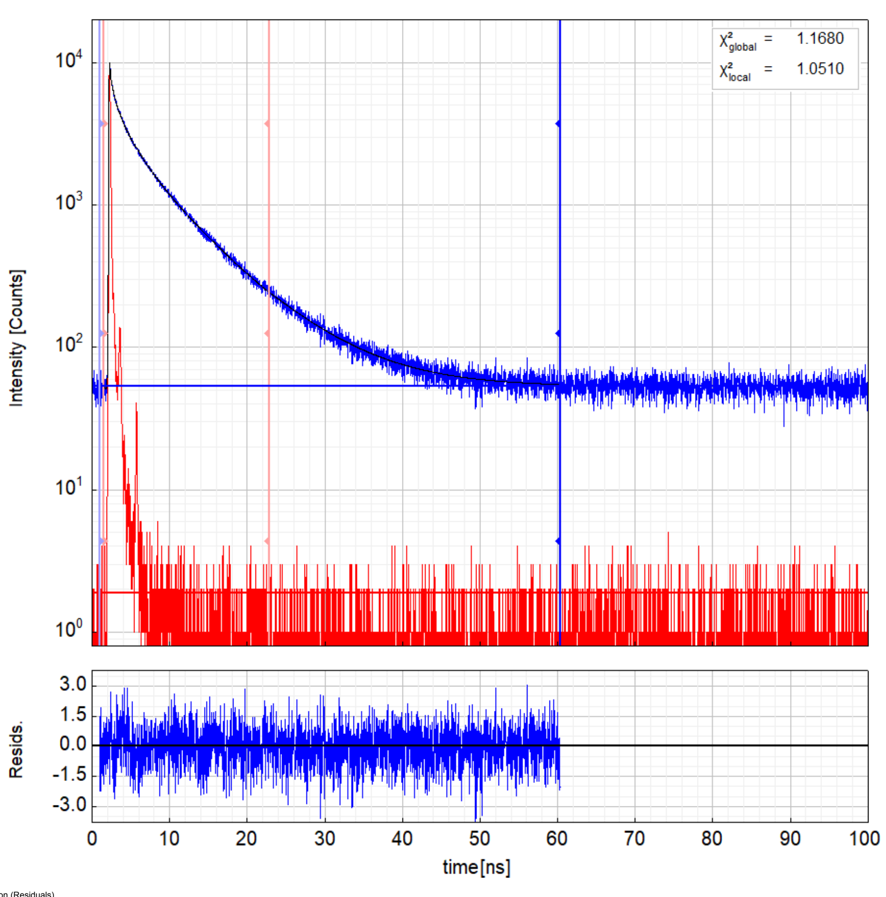

Autocorrelation (Residuals)

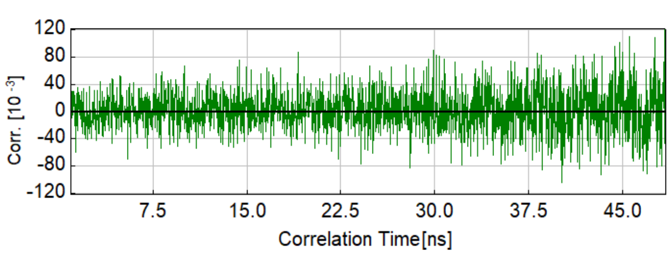

Fitted Parameters

Parameter	Value	Δ	δ
A_1[kCnts/Chnl]	2.943	±0.021	0.7%
τ_1[ns]	2.505	±0.013	0.5%
I_1[kCnts]	294.8	±2.8	0.9%
A_2[kCnts/Chnl]	2.610	±0.011	0.4%
τ_2[ns]	8.0397	±0.0067	0.1%
I_2[kCnts]	839.2	±3.6	0.4%
A_3[kCnts/Chnl]	4.011	±0.087	2.2%
τ_3[ns]	0.5435	±0.0070	1.3%
I_3[kCnts]	87.2	±1.6	1.8%
$Bkgr_{Dec}$[kCnts]	0.0531	±0.0002	0.3%
$Bkgr_{IRF}$[Cnts/Chnl]	1.90	±0.14	6.9%
$Shift_{IRF}$[ps]	12.89	±0.70	5.4%
A_{Scatt}[kCnts]	47.00	±0.53	1.1%
τ_{AvInt}[ns]	6.169	±0.013	0.2%
τ_{AvAmp}[ns]	3.193	±0.020	0.6%
λ_{Det}[nm]	630.0000	---	---

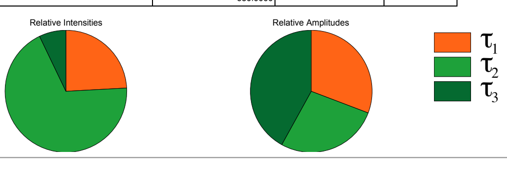

Data Set: 25 / 31

Decay: crv[25]; IRF: crv[0]

Fit

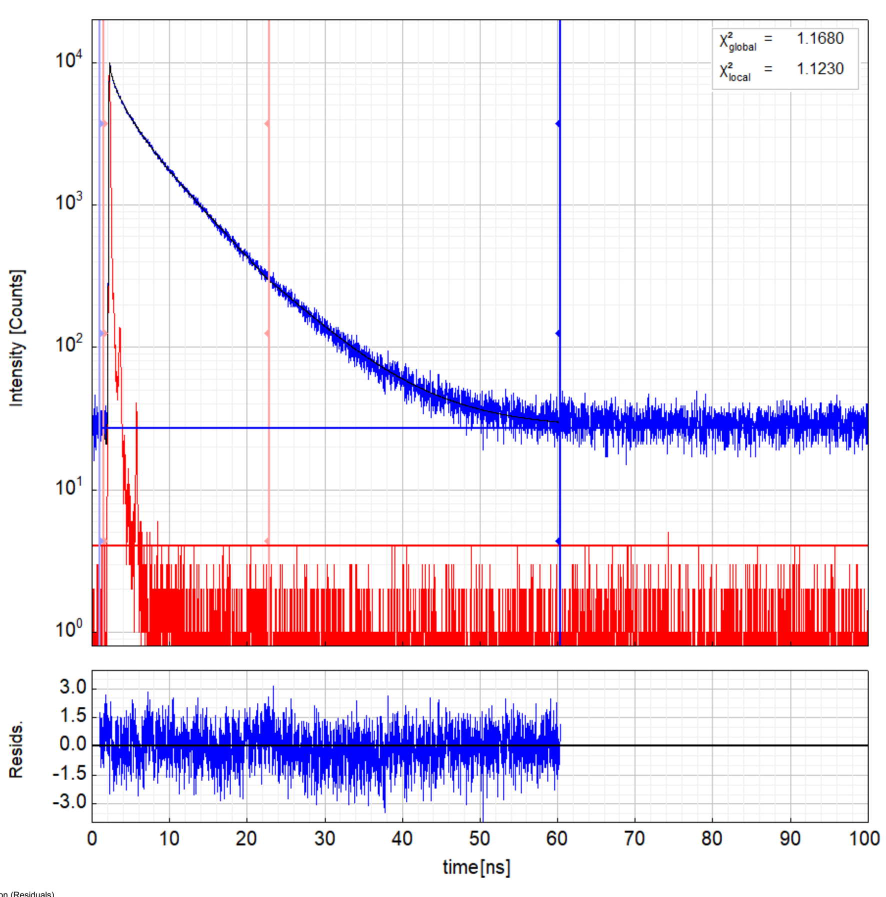

Autocorrelation (Residuals)

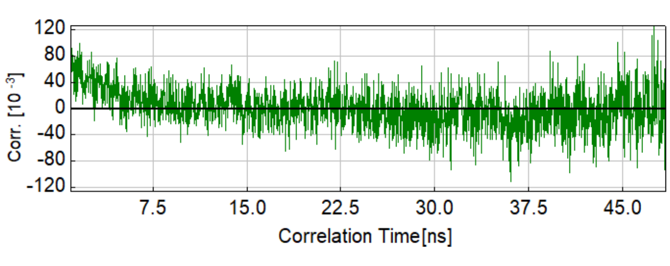

Fitted Parameters

Parameter	Value	Δ	δ
A_1[kCnts/Chnl]	3.271	±0.030	0.9%
τ_1[ns]	2.505	±0.013	0.5%
I_1[kCnts]	327.7	±2.2	0.6%
A_2[kCnts/Chnl]	4.1844	±0.0072	0.2%
τ_2[ns]	8.0397	±0.0067	0.1%
I_2[kCnts]	1 345.7	±5.5	0.4%
A_3[kCnts/Chnl]	2.969	±0.073	2.5%
τ_3[ns]	0.5435	±0.0070	1.3%
I_3[kCnts]	64.6	±1.4	2.1%
$Bkgr_{Dec}$[kCnts]	0.0272	±0.0002	0.5%
$Bkgr_{IRF}$[Cnts/Chnl]	4.01	±0.16	3.8%
$Shift_{IRF}$[ps]	12.82	±0.62	4.8%
A_{Scatt}[kCnts]	41.93	±0.95	2.3%
τ_{AvInt}[ns]	6.718	±0.013	0.2%
τ_{AvAmp}[ns]	4.169	±0.031	0.7%
λ_{Det}[nm]	640.0000	---	---

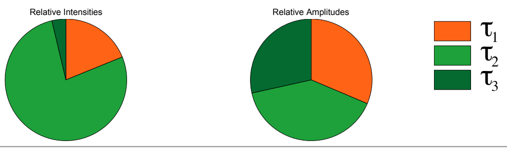

Data Set: 26 / 31

Decay: crv[26]; IRF: crv[0]

Fit

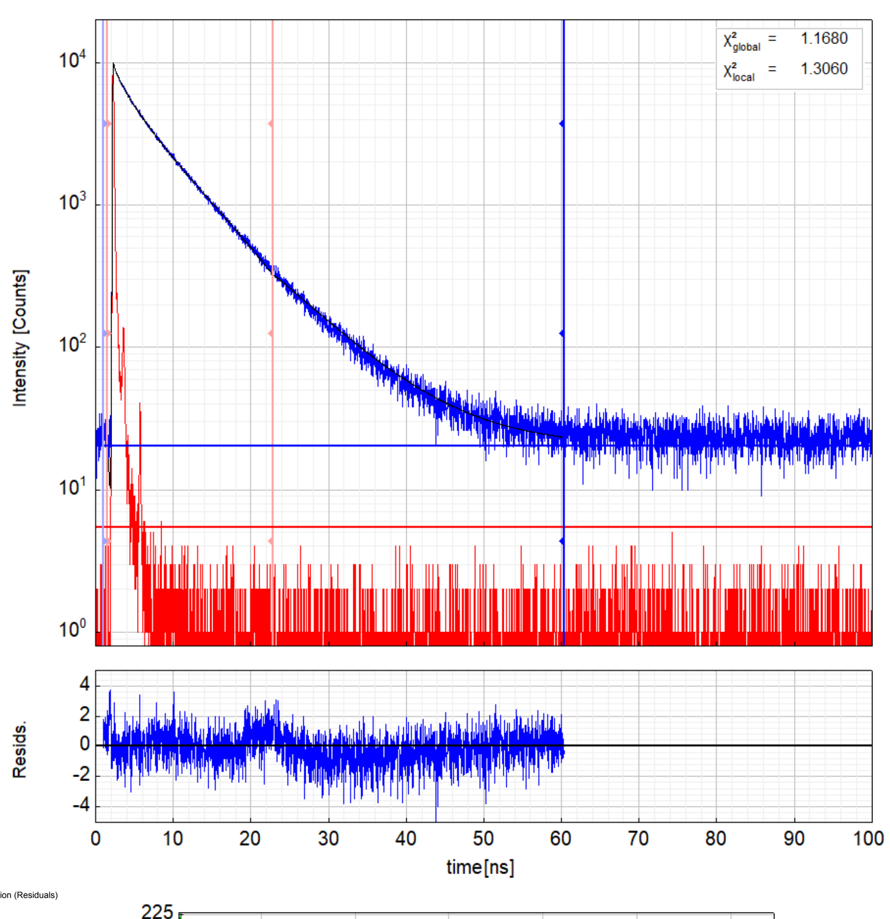

Autocorrelation (Residuals)

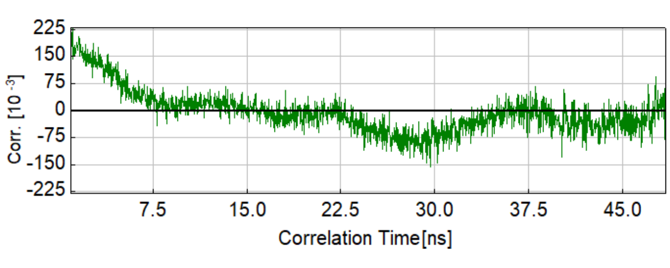

Fitted Parameters

Parameter	Value	Δ	δ
A_1[kCnts/Chnl]	3.690	±0.037	1.0%
τ_1[ns]	2.505	±0.013	0.5%
I_1[kCnts]	369.8	±4.6	1.2%
A_2[kCnts/Chnl]	5.290	±0.015	0.3%
τ_2[ns]	8.0397	±0.0067	0.1%
I_2[kCnts]	1 701.2	±4.9	0.3%
A_3[kCnts/Chnl]	1.913	±0.068	3.5%
τ_3[ns]	0.5435	±0.0070	1.3%
I_3[kCnts]	41.6	±1.2	2.8%
$Bkgr_{Dec}$[kCnts]	0.0204	±0.0003	1.5%
$Bkgr_{IRF}$[Cnts/Chnl]	5.47	±0.11	1.9%
$Shift_{IRF}$[ps]	14.50	±0.63	4.3%
A_{Scatt}[kCnts]	38.75	±0.74	1.9%
τ_{AvInt}[ns]	6.9234	±0.0085	0.1%
τ_{AvAmp}[ns]	4.849	±0.017	0.3%
λ_{Det}[nm]	650.0000	---	---

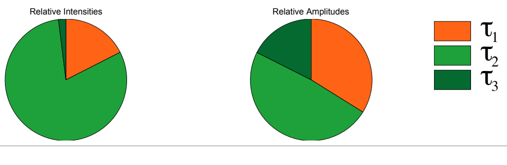

Data Set: 27 / 31

Decay: crv[27]; IRF: crv[0]

Fit

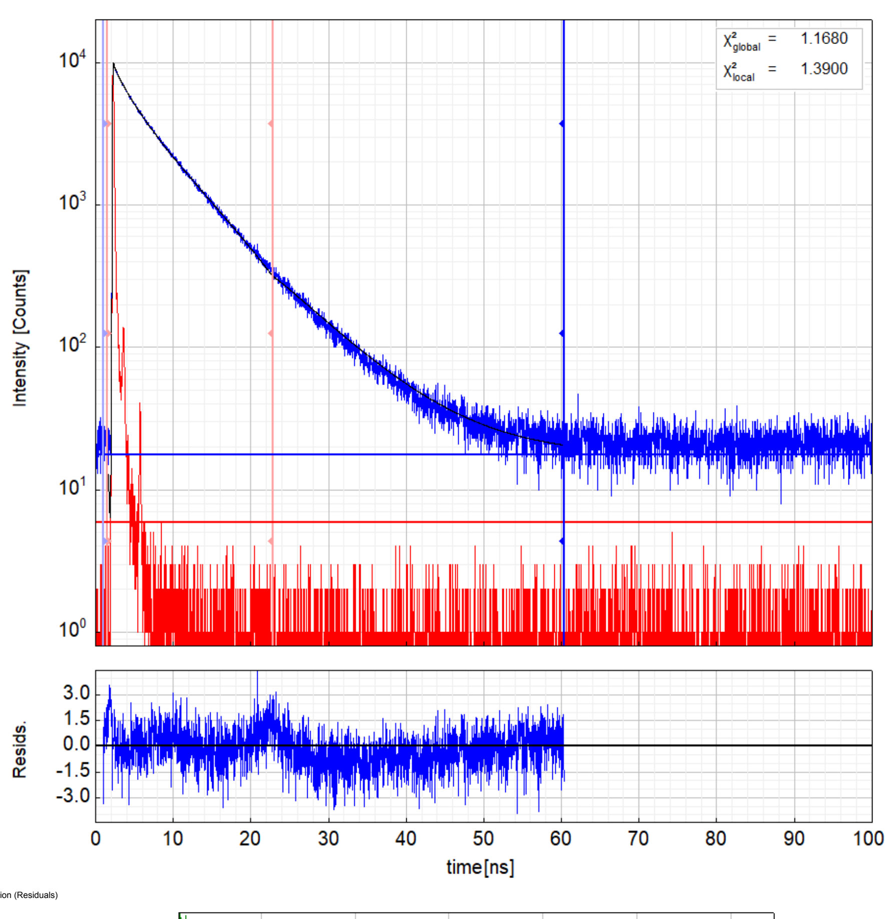

Autocorrelation (Residuals)

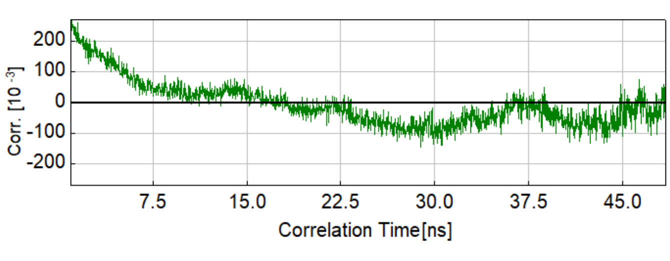

Fitted Parameters

Parameter	Value	Δ	δ
A_1[kCnts/Chnl]	3.921	±0.023	0.6%
τ_1[ns]	2.505	±0.013	0.5%
I_1[kCnts]	392.8	±2.9	0.7%
A_2[kCnts/Chnl]	5.373	±0.015	0.3%
τ_2[ns]	8.0397	±0.0067	0.1%
I_2[kCnts]	1 727.9	±6.1	0.4%
A_3[kCnts/Chnl]	1.753	±0.084	4.8%
τ_3[ns]	0.5435	±0.0070	1.3%
I_3[kCnts]	38.2	±1.3	3.3%
$Bkgr_{Dec}$[kCnts]	0.0176	±0.0004	1.8%
$Bkgr_{IRF}$[Cnts/Chnl]	5.906	±0.094	1.6%
$Shift_{IRF}$[ps]	14.40	±0.79	5.5%
A_{Scatt}[kCnts]	36.94	±0.42	1.1%
τ_{AvInt}[ns]	6.9003	±0.0077	0.1%
τ_{AvAmp}[ns]	4.886	±0.027	0.5%
λ_{Det}[nm]	660.0000	---	---

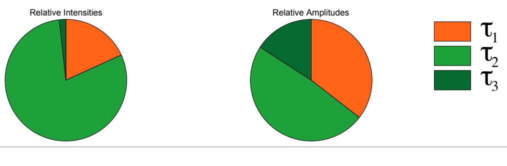

Data Set: 28 / 31

Decay: crv[28]; IRF: crv[0]

Fit

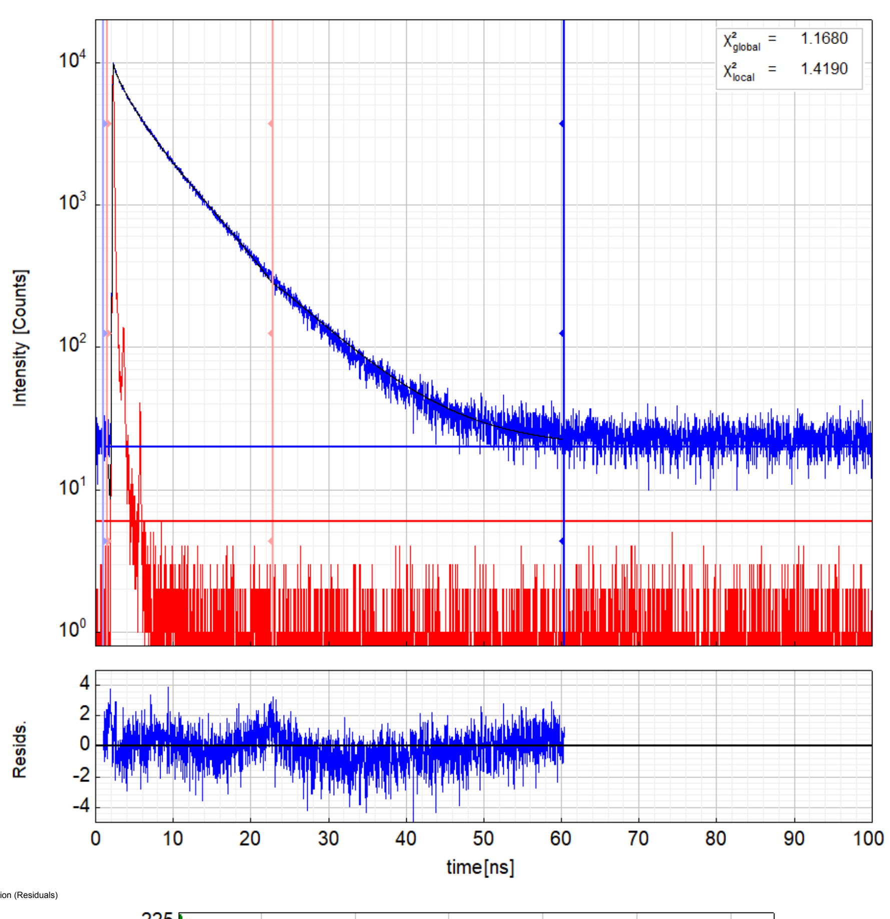

Autocorrelation (Residuals)

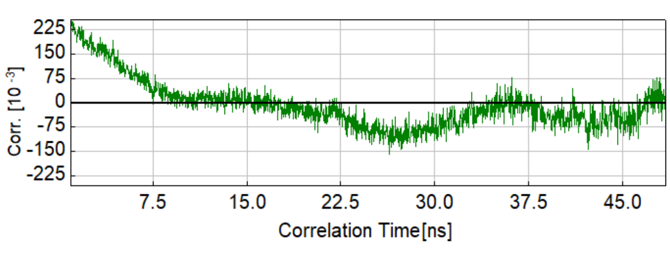

Fitted Parameters

Parameter	Value	Δ	δ
A_1[kCnts/Chnl]	4.064	±0.032	0.8%
τ_1[ns]	2.505	±0.013	0.5%
I_1[kCnts]	407.2	±3.0	0.7%
A_2[kCnts/Chnl]	4.818	±0.018	0.4%
τ_2[ns]	8.0397	±0.0067	0.1%
I_2[kCnts]	1 549.3	±5.6	0.4%
A_3[kCnts/Chnl]	1.887	±0.060	3.2%
τ_3[ns]	0.5435	±0.0070	1.3%
I_3[kCnts]	41.1	±1.9	4.5%
$Bkgr_{Dec}$[kCnts]	0.0200	±0.0004	1.9%
$Bkgr_{IRF}$[Cnts/Chnl]	6.031	±0.086	1.4%
$Shift_{IRF}$[ps]	14.42	±0.62	4.2%
A_{Scatt}[kCnts]	38.12	±0.45	1.2%
τ_{AvInt}[ns]	6.7576	±0.0087	0.1%
τ_{AvAmp}[ns]	4.638	±0.017	0.4%
λ_{Det}[nm]	670.0000	---	---

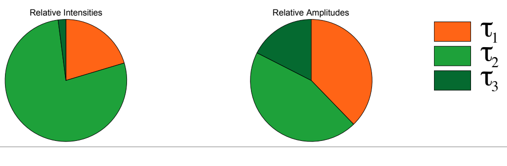

Data Set: 29 / 31

Decay: crv[29]; IRF: crv[0]

Fit

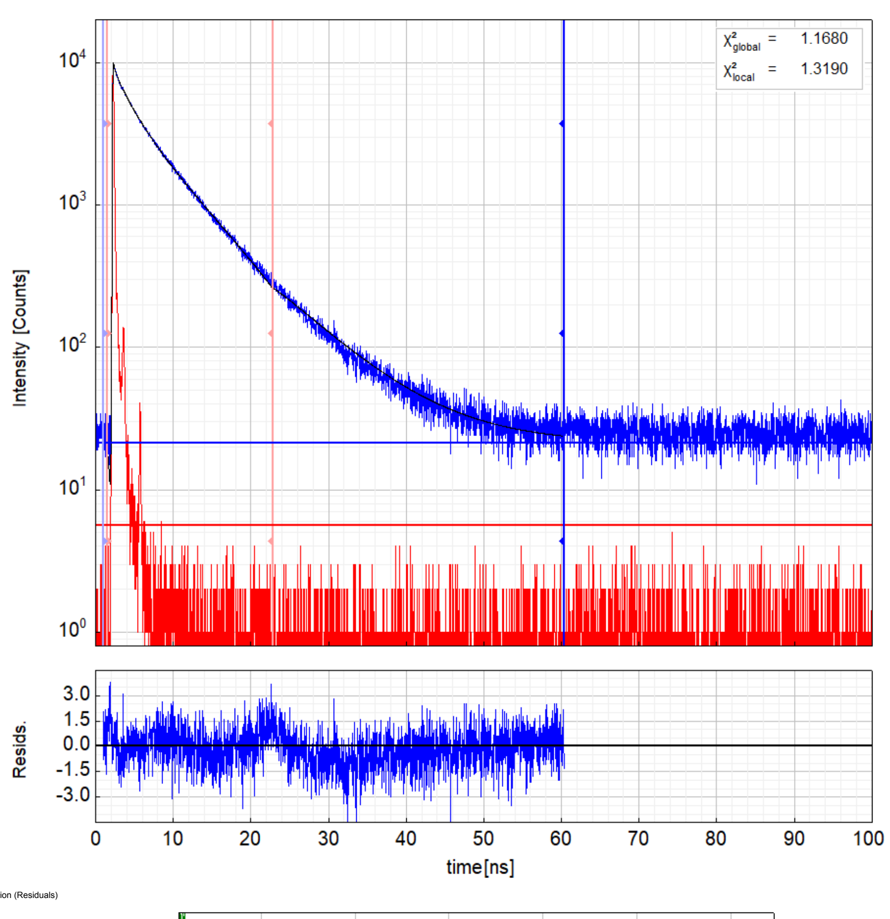

Autocorrelation (Residuals)

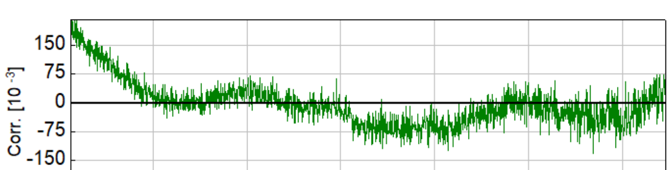

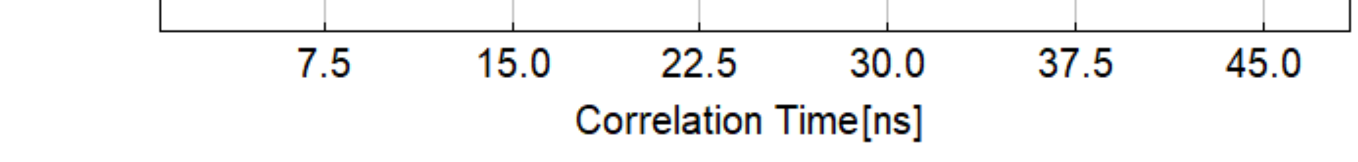

Fitted Parameters

Parameter	Value	Δ	δ
A_1[kCnts/Chnl]	4.403	±0.033	0.7%
$τ_1$[ns]	2.505	±0.013	0.5%
I_1[kCnts]	441.2	±3.9	0.9%
A_2[kCnts/Chnl]	4.351	±0.021	0.5%
$τ_2$[ns]	8.0397	±0.0067	0.1%
I_2[kCnts]	1 399.2	±6.6	0.5%
A_3[kCnts/Chnl]	2.077	±0.057	2.7%
$τ_3$[ns]	0.5435	±0.0070	1.3%
I_3[kCnts]	45.2	±1.3	2.8%
$Bkgr_{Dec}$[kCnts]	0.0214	±0.0003	1.1%
$Bkgr_{IRF}$[Cnts/Chnl]	5.65	±0.17	2.9%
$Shift_{IRF}$[ps]	15.18	±0.43	2.8%
A_{Scatt}[kCnts]	39.47	±0.46	1.1%
$τ_{AvInt}$[ns]	6.566	±0.016	0.2%
$τ_{AvAmp}$[ns]	4.353	±0.012	0.3%
$λ_{Det}$[nm]	680.0000	---	---

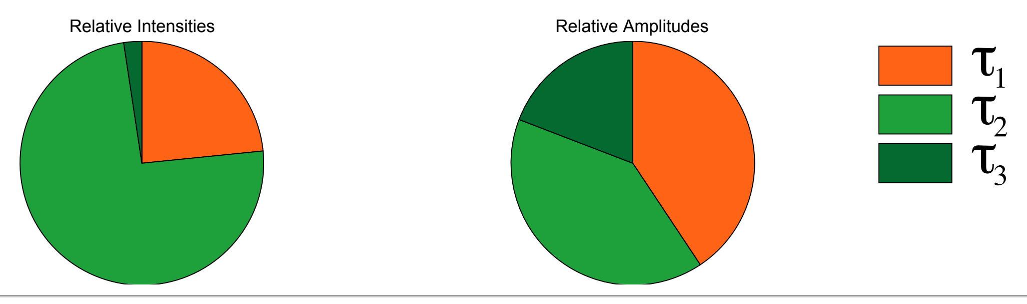

Data Set: 30 / 31

Decay: crv[30]; IRF: crv[0]

Fit

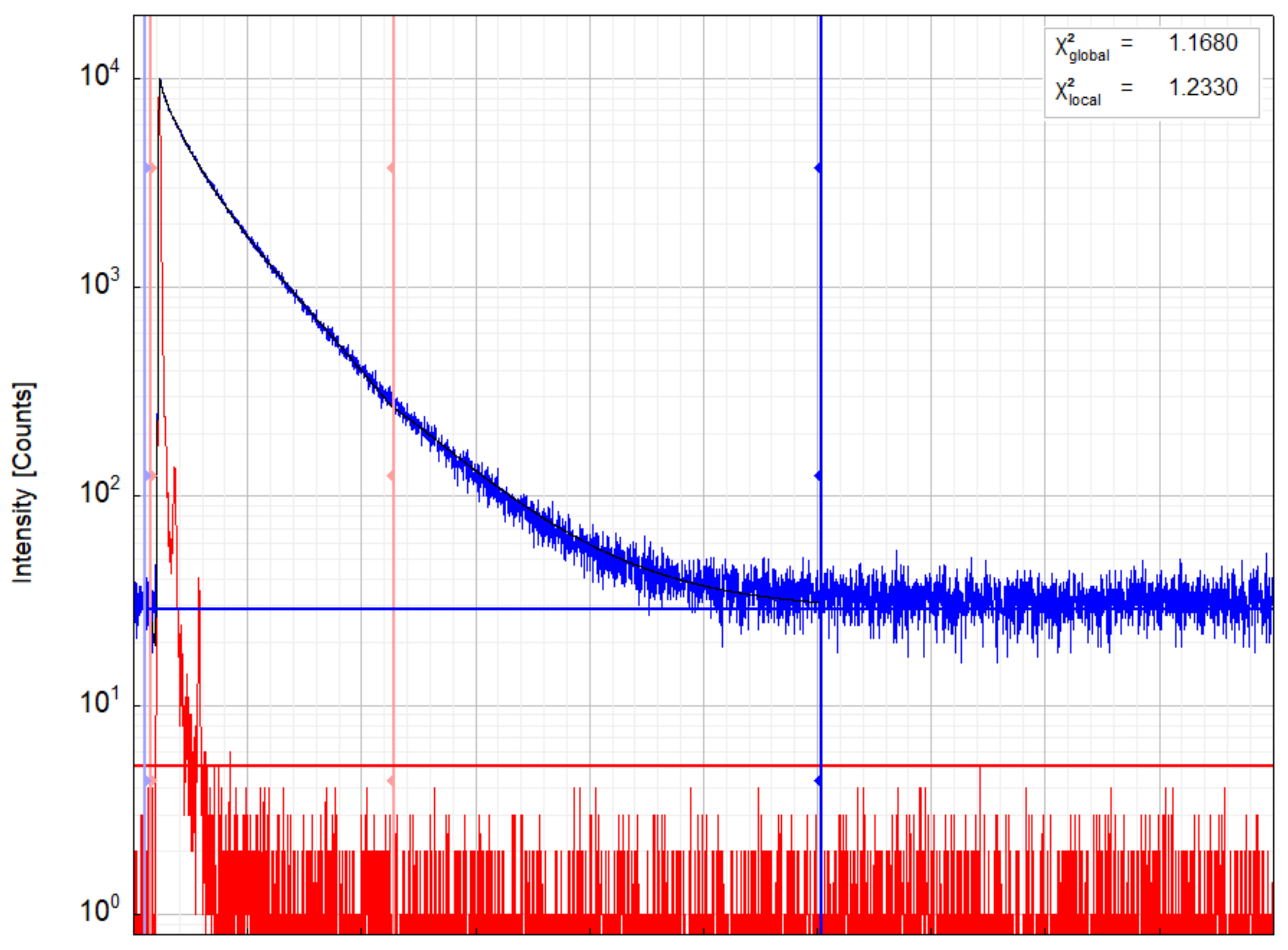

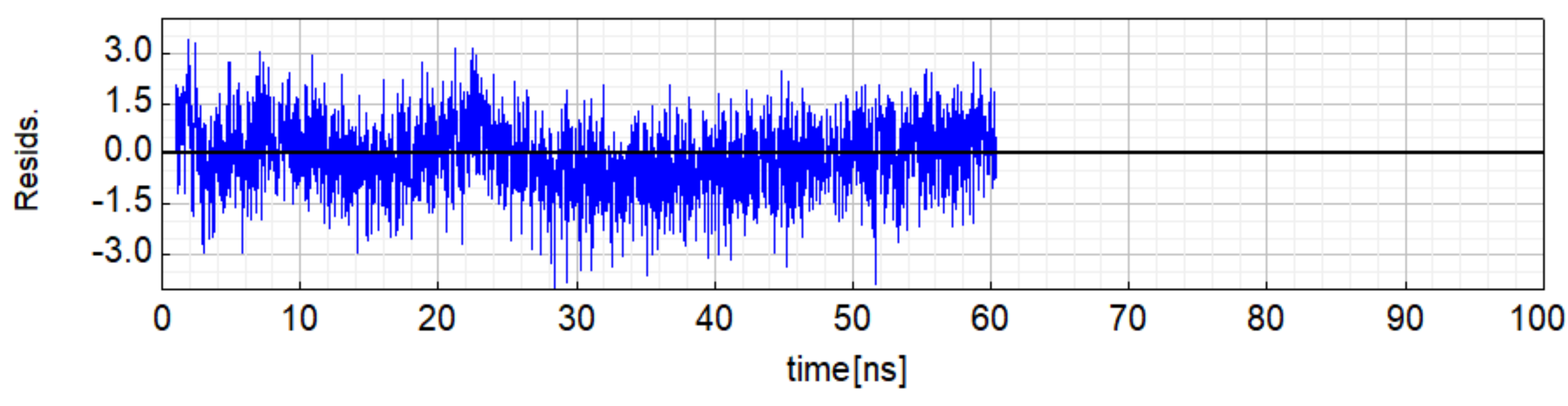

Autocorrelation (Residuals)

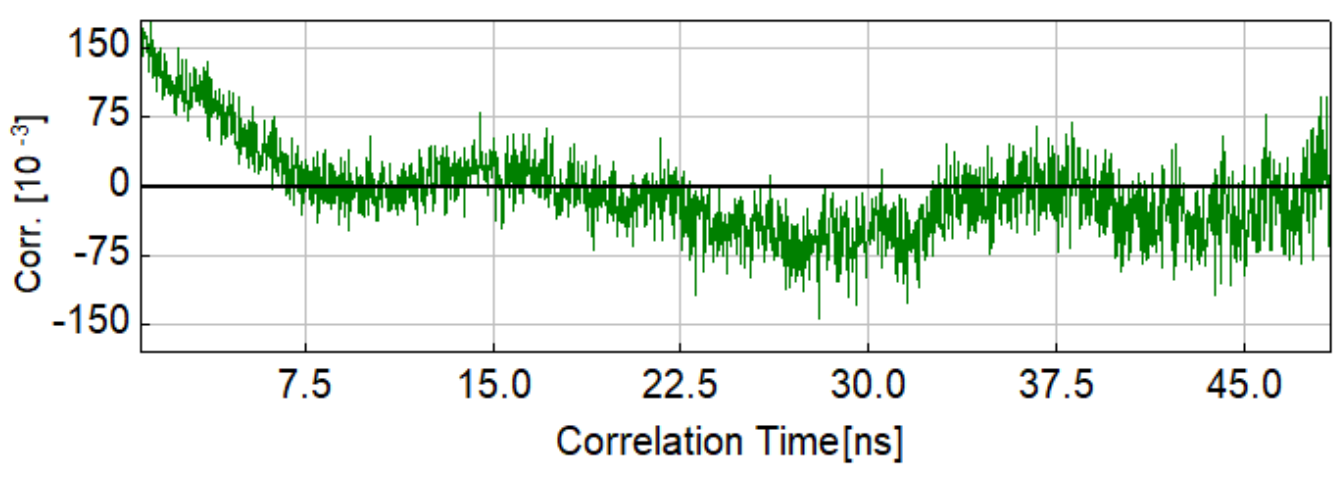

Fitted Parameters

Parameter	Value	Δ	δ
A_1[kCnts/Chnl]	4.577	±0.029	0.6%
$τ_1$[ns]	2.505	±0.013	0.5%
I_1[kCnts]	458.6	±3.5	0.8%
A_2[kCnts/Chnl]	4.094	±0.012	0.3%
$τ_2$[ns]	8.0397	±0.0067	0.1%
I_2[kCnts]	1 316.5	±3.9	0.3%
A_3[kCnts/Chnl]	2.151	±0.077	3.5%
$τ_3$[ns]	0.5435	±0.0070	1.3%
I_3[kCnts]	46.8	±2.1	4.3%
$Bkgr_{Dec}$[kCnts]	0.0286	±0.0003	0.8%
$Bkgr_{IRF}$[Cnts/Chnl]	5.09	±0.11	2.0%
$Shift_{IRF}$[ps]	15.65	±0.64	4.1%
A_{Scatt}[kCnts]	39.29	±0.97	2.5%
$τ_{AvInt}$[ns]	6.455	±0.014	0.2%
$τ_{AvAmp}$[ns]	4.209	±0.035	0.8%
$λ_{Det}$[nm]	690.0000	---	---

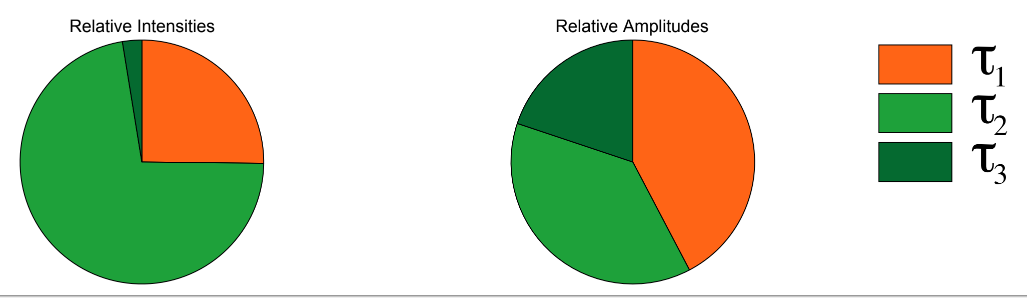

Data Set: 31 / 31

Decay: crv[31]; IRF: crv[0]

Fit

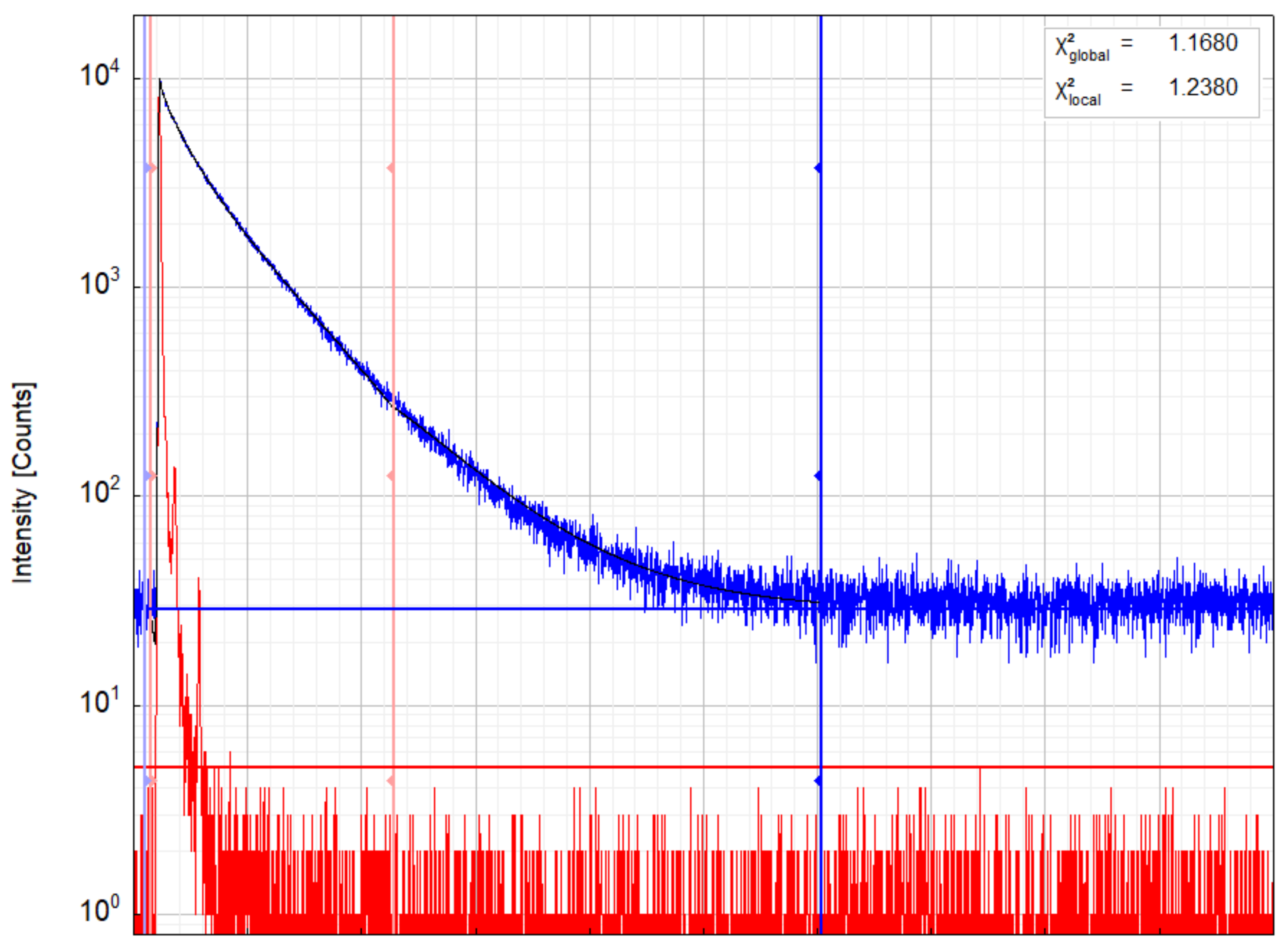

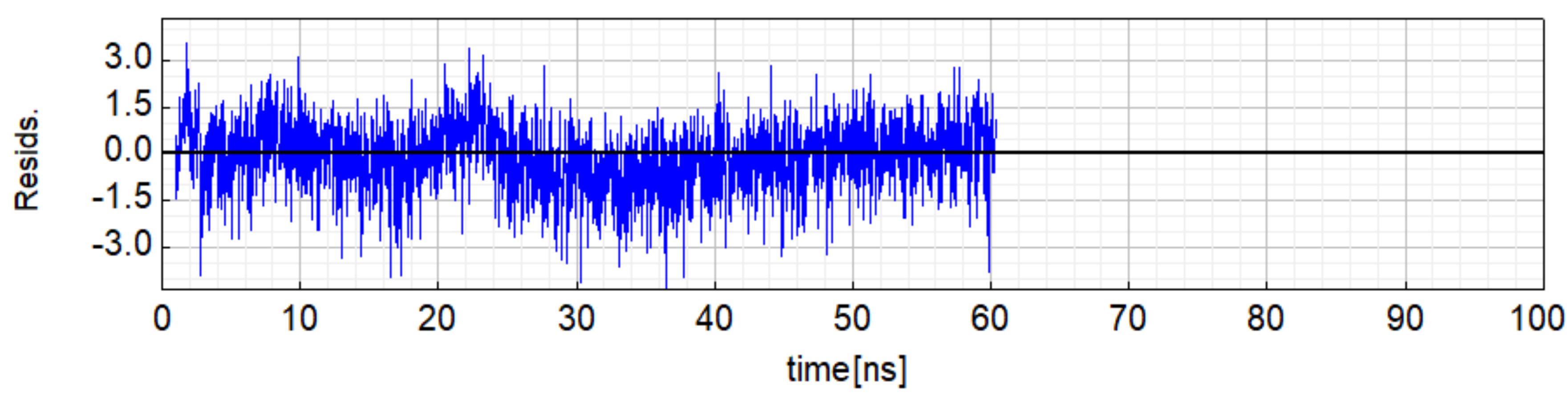

Autocorrelation (Residuals)

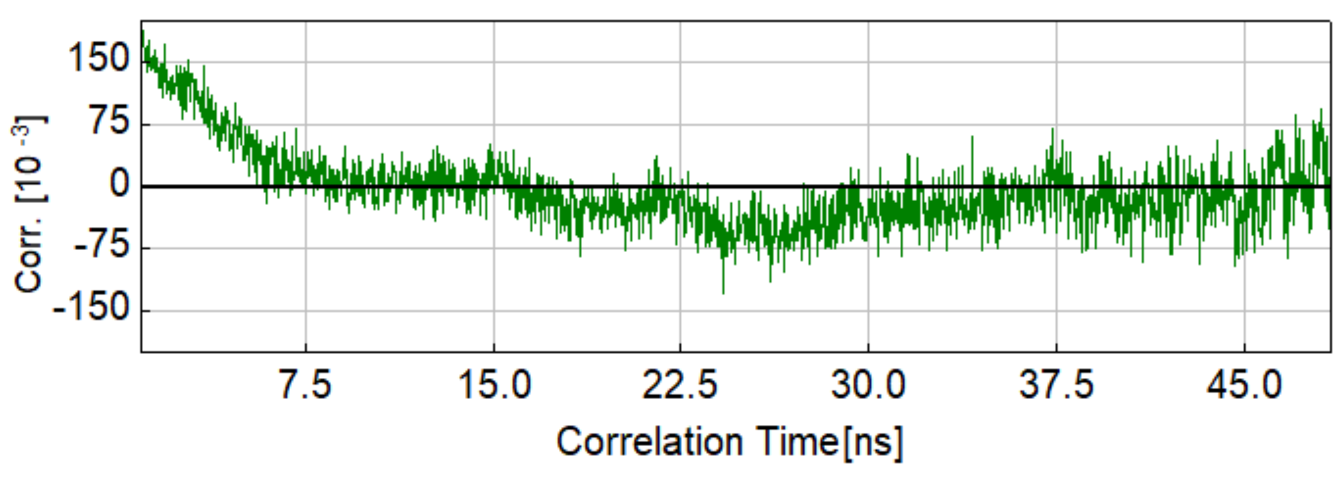

Fitted Parameters

Parameter	Value	Δ	δ
A_1[kCnts/Chnl]	4.458	±0.025	0.5%
$τ_1$[ns]	2.505	±0.013	0.5%
I_1[kCnts]	446.7	±4.2	0.9%
A_2[kCnts/Chnl]	4.106	±0.015	0.4%
$τ_2$[ns]	8.0397	±0.0067	0.1%
I_2[kCnts]	1 320.3	±4.4	0.3%
A_3[kCnts/Chnl]	2.059	±0.075	3.6%
$τ_3$[ns]	0.5435	±0.0070	1.3%
I_3[kCnts]	44.8	±1.6	3.5%
$Bkgr_{Dec}$[kCnts]	0.0287	±0.0003	0.9%
$Bkgr_{IRF}$[Cnts/Chnl]	5.03	±0.15	3.0%
$Shift_{IRF}$[ps]	17.59	±0.60	3.4%
A_{Scatt}[kCnts]	39.58	±0.76	1.9%
$τ_{AvInt}$[ns]	6.490	±0.012	0.2%
$τ_{AvAmp}$[ns]	4.264	±0.025	0.6%
$λ_{Det}$[nm]	700.0000	---	---

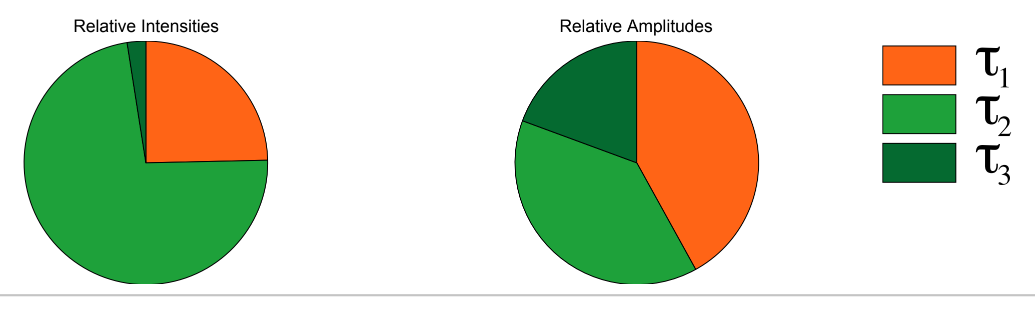

Parameter Plot

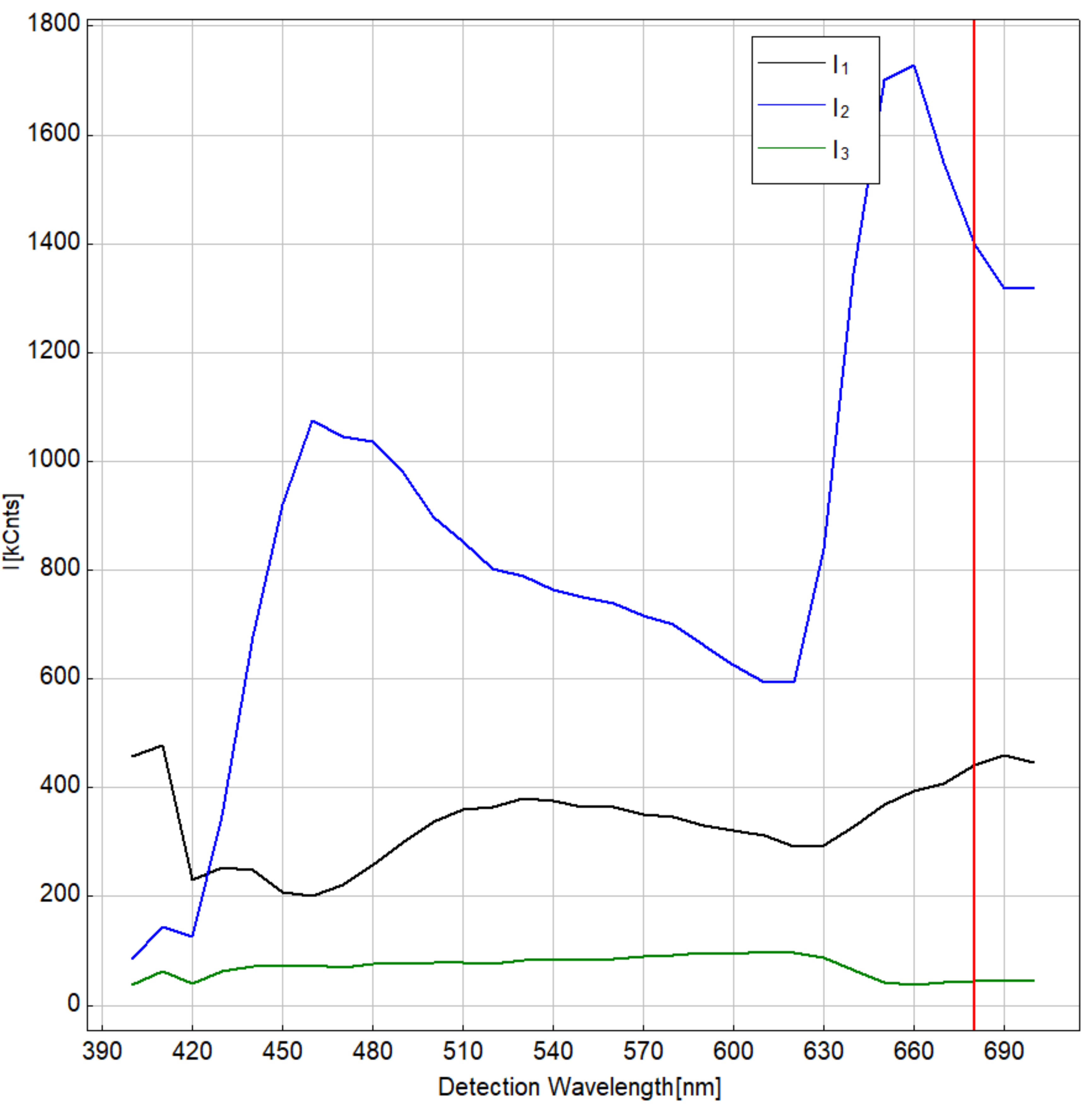

Global Parameters

Parameter	Value	Δ	δ
$τ_1$[ns]	2.505	±0.013	0.5%
$τ_2$[ns]	8.0397	±0.0067	0.1%
$τ_3$[ns]	0.5435	±0.0070	1.3%
