# Supplementary material for: A Porphyrin‐Based Organic Network Comprising Sustainable Carbon Dots for Photopolymerization
Source: Angew Chem Int Ed Engl. 2022 Sep 1;61(40):e202208180. doi: 10.1002/anie.202208180 (PMC9826160; doi:10.1002/anie.202208180)
Supplement: Supplementary file 7 — Supporting Information [file ANIE-61-0-s008.pdf]

EasyTau Report

3/11/2022 7:51:21 PM
F:\Paper Xiongfei\CD-1 THF tau global.html

Container Curves

TRES+IRF_20201222_1824.etc: crv[0 - 16]

Container Properties

Measurement Context: Time Resolved Emission Scan
Detection Wavelength: 400.0 - 700.0 (Step: 20.0) nm
Summary:

Sample:	CD_X_1
Solvent:	Tetrahydrofuran
Excitation:	V pol 376±1nm with LDH-P-C-375
Detection:	M pol 400...700nm 20nm steps<br>10000 peak counts<br>grating 1200/500-<br>detector UV-red [PMT]

Comment

Model: Exponential [Reconv.]

$$Dec(t) = \left[\int_{-\infty}^{t} dt' \left[IRF(t - Shift_{IRF}) - Bkgr_{IRF}\right]\left[\sum_{i=1}^{n_{Exp}} A_i e^{-\frac{t-t'}{\tau_i}} + A_{Scatt}\,\delta(t-t')\right]\right] + Bkgr_{Dec}$$

$$I_m = A_m \tau_m$$

$$A_{Rel\,m} = A_m \Big/ \sum_{\substack{i=1 \\ A_i>0}}^{n_{Exp}} A_i \qquad I_{Rel\,m} = I_m \Big/ \sum_{\substack{i=1 \\ I_i>0}}^{n_{Exp}} I_i$$

$$\tau_{Av\,Amp} = \sum_{\substack{i=1 \\ A_i>0}}^{n_{Exp}} A_i \tau_i \Big/ \sum_{\substack{i=1 \\ A_i>0}}^{n_{Exp}} A_i \qquad \tau_{Av\,Int} = \sum_{\substack{i=1 \\ I_i>0}}^{n_{Exp}} I_i \tau_i \Big/ \sum_{\substack{i=1 \\ I_i>0}}^{n_{Exp}} I_i$$

Data Set: 1 / 16

Decay: crv[1]; IRF: crv[0]

Fit

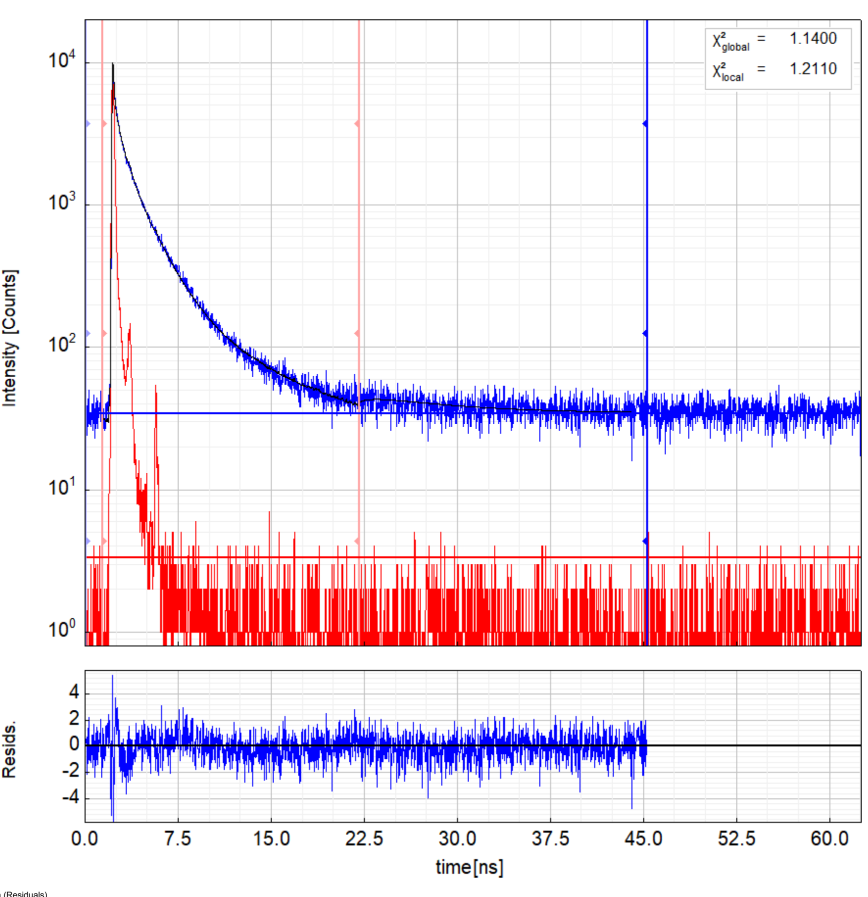

Autocorrelation (Residuals)

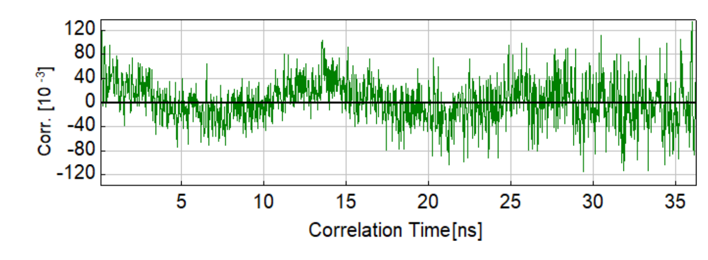

Fitted Parameters

Parameter	Value	Δ	δ
A_1[kCnts/Chnl]	0.2478	±0.0084	3.4%
τ_1[ns]	7.166	±0.030	0.4%
I_1[kCnts]	71.1	±2.1	2.9%
A_2[kCnts/Chnl]	2.521	±0.070	2.8%
τ_2[ns]	1.943	±0.028	1.4%
I_2[kCnts]	195.9	±2.1	1.1%
A_3[kCnts/Chnl]	3.30	±0.11	3.3%
τ_3[ns]	0.534	±0.025	4.6%
I_3[kCnts]	70.20	±1.00	1.4%
$Bkgr_{Dec}$[kCnts]	0.0344	±0.0002	0.4%
$Bkgr_{IRF}$[Cnts/Chnl]	3.34	±0.17	4.8%
$Shift_{IRF}$[ps]	-10.1	±1.3	13%
A_{Scatt}[kCnts]	63.9	±2.2	3.4%
τ_{AvInt}[ns]	2.750	±0.014	0.5%
τ_{AvAmp}[ns]	1.391	±0.028	2.0%
λ_{Det}[nm]	400.0000	---	---

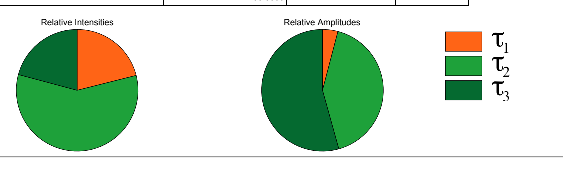

Data Set: 2 / 16

Decay: crv[2]; IRF: crv[0]

Fit

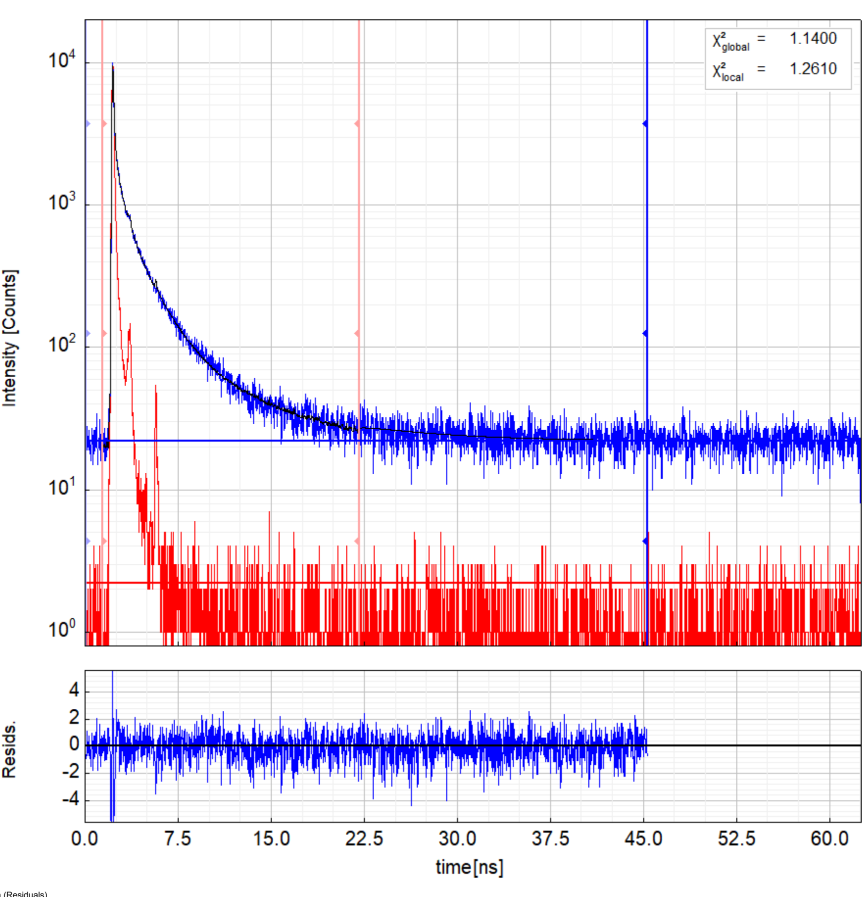

Autocorrelation (Residuals)

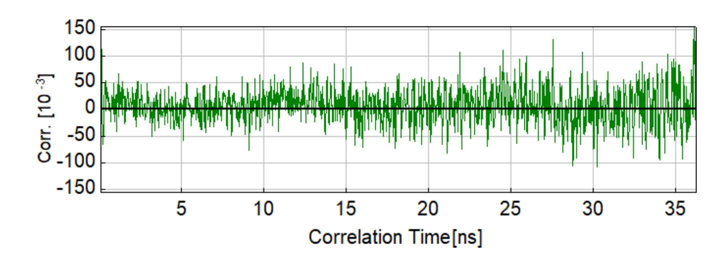

Fitted Parameters

Parameter	Value	Δ	δ
A_1[kCnts/Chnl]	0.1107	±0.0040	3.6%
τ_1[ns]	7.166	±0.030	0.4%
I_1[kCnts]	31.8	±1.1	3.5%
A_2[kCnts/Chnl]	0.927	±0.027	2.8%
τ_2[ns]	1.943	±0.028	1.4%
I_2[kCnts]	72.0	±1.2	1.6%
A_3[kCnts/Chnl]	1.577	±0.049	3.1%
τ_3[ns]	0.534	±0.025	4.6%
I_3[kCnts]	33.7	±1.1	3.2%
$Bkgr_{Dec}$[kCnts]	0.0221	±0.0002	0.7%
$Bkgr_{IRF}$[Cnts/Chnl]	2.21	±0.16	7.2%
$Shift_{IRF}$[ps]	-18.16	±0.92	5.0%
A_{Scatt}[kCnts]	72.9	±1.7	2.2%
τ_{AvInt}[ns]	2.804	±0.016	0.6%
τ_{AvAmp}[ns]	1.314	±0.026	1.9%
λ_{Det}[nm]	420.0000	---	---

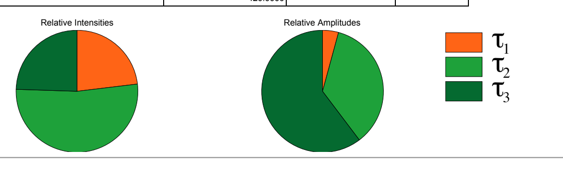

Data Set: 3 / 16

Decay: crv[3]; IRF: crv[0]

Fit

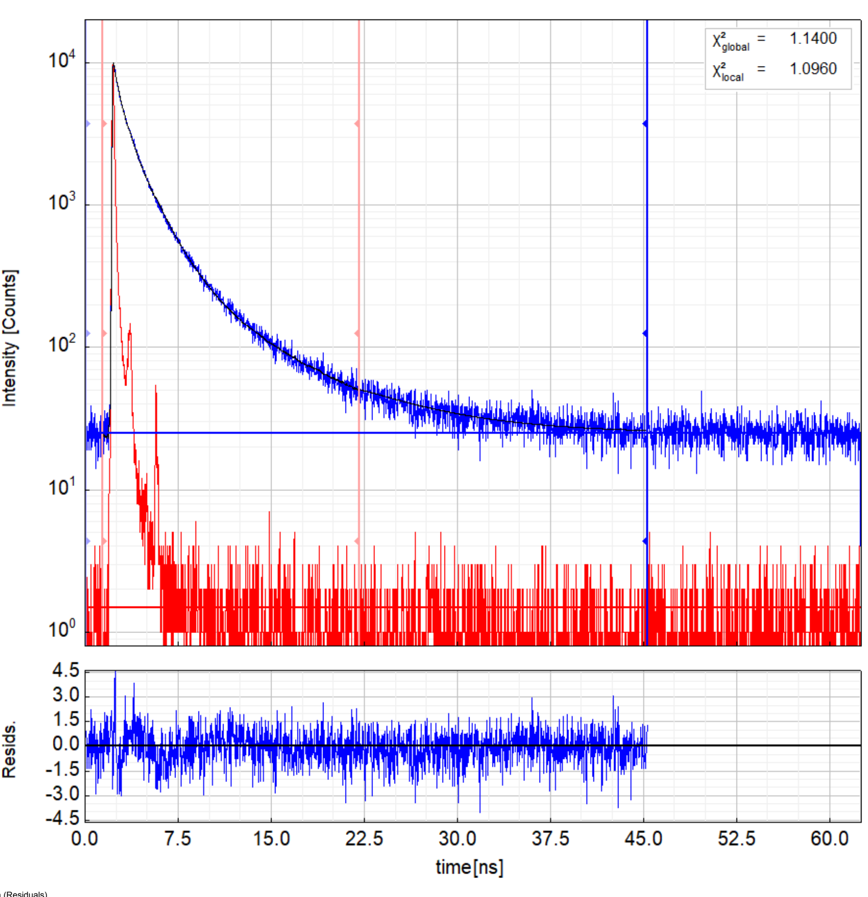

Autocorrelation (Residuals)

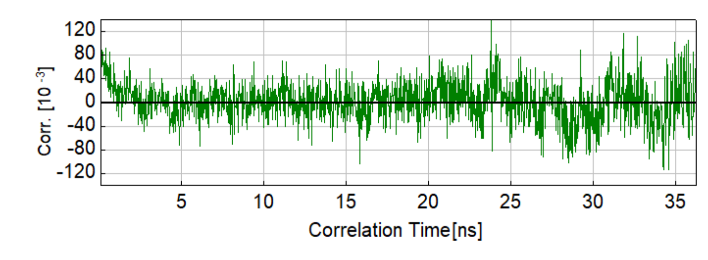

Fitted Parameters

Parameter	Value	Δ	δ
A_1[kCnts/Chnl]	0.453	±0.013	2.7%
τ_1[ns]	7.166	±0.030	0.4%
I_1[kCnts]	129.7	±3.0	2.2%
A_2[kCnts/Chnl]	4.43	±0.11	2.3%
τ_2[ns]	1.943	±0.028	1.4%
I_2[kCnts]	343.9	±3.4	1.0%
A_3[kCnts/Chnl]	6.37	±0.13	2.0%
τ_3[ns]	0.534	±0.025	4.6%
I_3[kCnts]	135.7	±4.1	3.0%
$Bkgr_{Dec}$[kCnts]	0.0250	±0.0003	1.0%
$Bkgr_{IRF}$[Cnts/Chnl]	1.49	±0.13	8.4%
$Shift_{IRF}$[ps]	4.32	±0.90	21%
A_{Scatt}[kCnts]	40.2	±1.5	3.5%
τ_{AvInt}[ns]	2.741	±0.011	0.4%
τ_{AvAmp}[ns]	1.355	±0.024	1.8%
λ_{Det}[nm]	440.0000	---	---

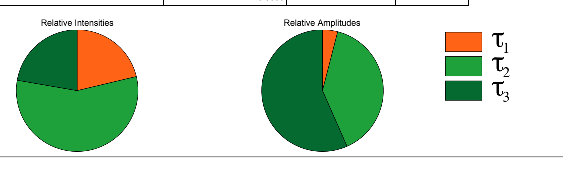

Data Set: 4 / 16

Decay: crv[4]; IRF: crv[0]

Fit

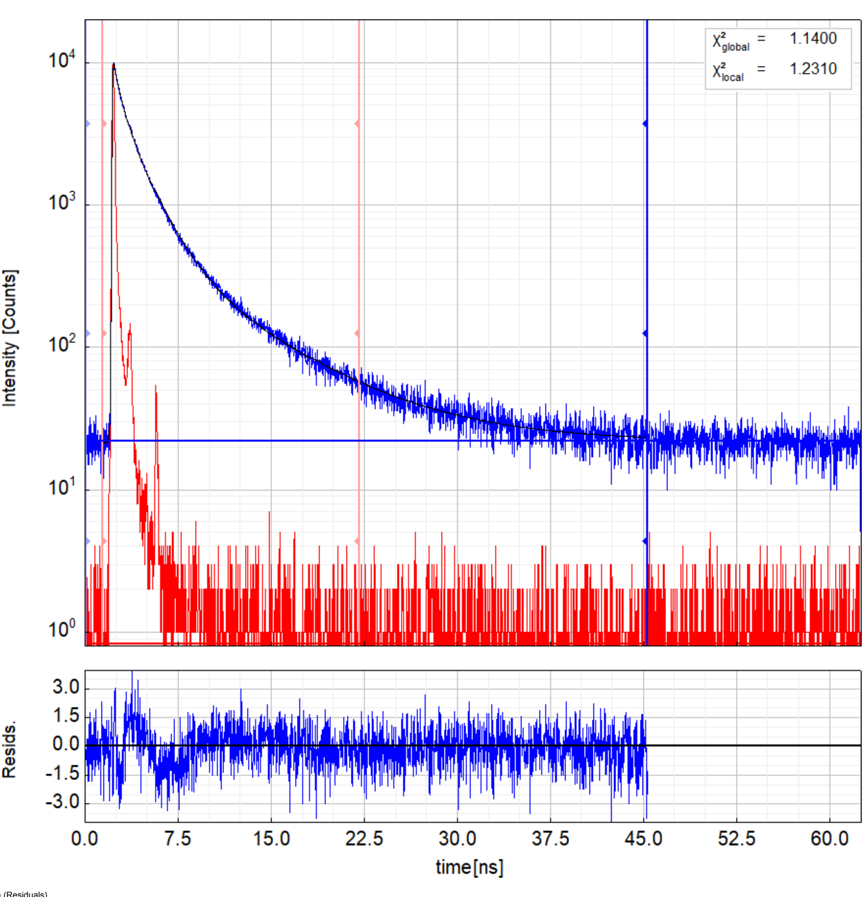

Autocorrelation (Residuals)

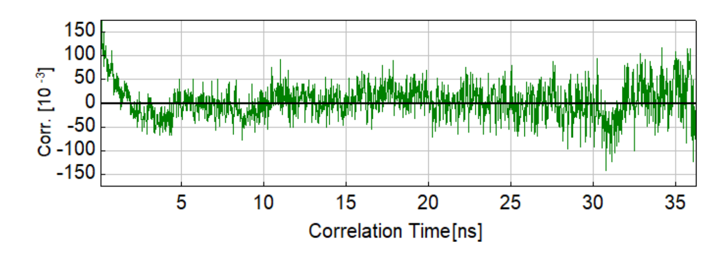

Fitted Parameters

Parameter	Value	Δ	δ
A_1[kCnts/Chnl]	0.5378	±0.0097	1.8%
τ_1[ns]	7.166	±0.030	0.4%
I_1[kCnts]	154.2	±2.6	1.6%
A_2[kCnts/Chnl]	4.73	±0.15	3.1%
τ_2[ns]	1.943	±0.028	1.4%
I_2[kCnts]	367.4	±4.9	1.3%
A_3[kCnts/Chnl]	6.26	±0.12	1.8%
τ_3[ns]	0.534	±0.025	4.6%
I_3[kCnts]	133.4	±5.4	4.0%
$Bkgr_{Dec}$[kCnts]	0.0221	±0.0003	1.2%
$Bkgr_{IRF}$[Cnts/Chnl]	0.829	±0.098	12%
$Shift_{IRF}$[ps]	9.52	±0.88	9.2%
A_{Scatt}[kCnts]	38.06	±0.78	2.0%
τ_{AvInt}[ns]	2.885	±0.015	0.5%
τ_{AvAmp}[ns]	1.421	±0.022	1.5%
λ_{Det}[nm]	460.0000	---	---

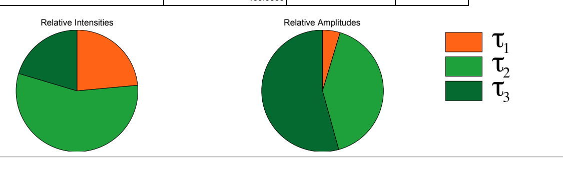

Data Set: 5 / 16

Decay: crv[5]; IRF: crv[0]

Fit

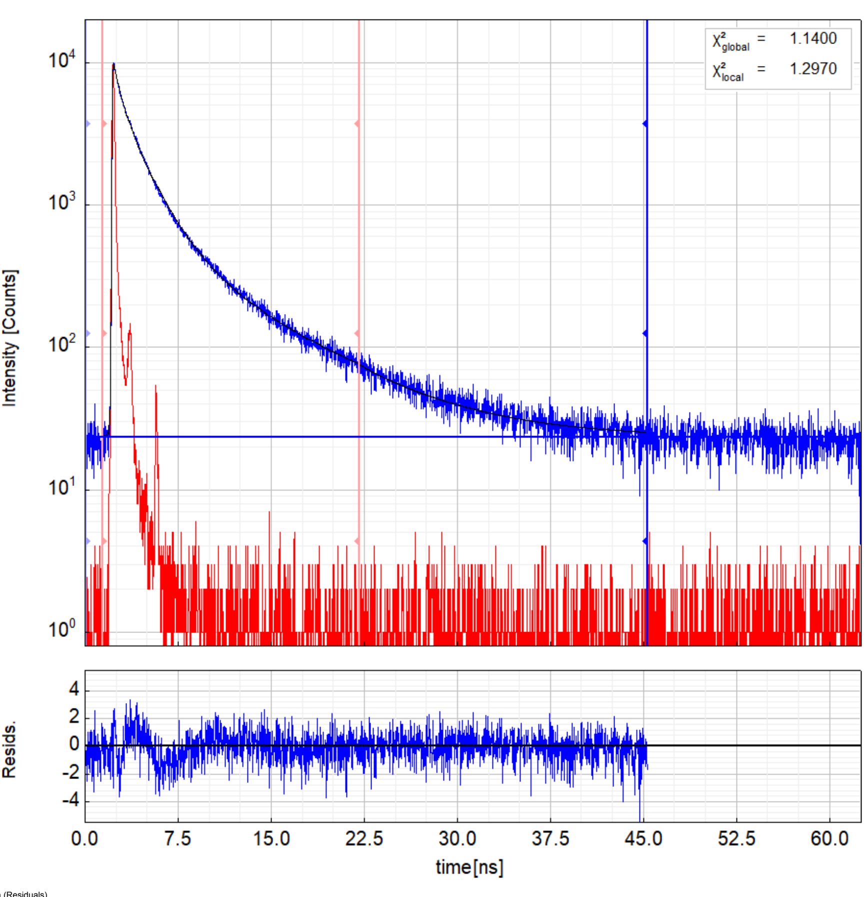

Autocorrelation (Residuals)

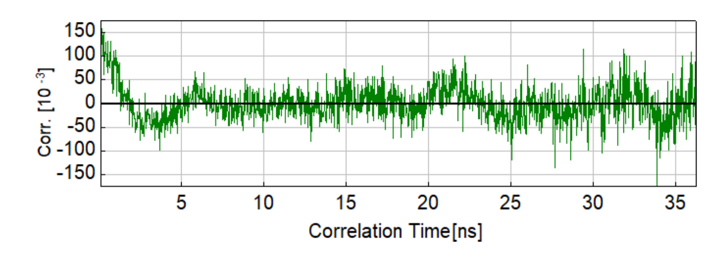

Fitted Parameters

Parameter	Value	Δ	δ
A_1[kCnts/Chnl]	0.7340	±0.0068	0.9%
τ_1[ns]	7.166	±0.030	0.4%
I_1[kCnts]	210.4	±2.7	1.3%
A_2[kCnts/Chnl]	5.04	±0.13	2.5%
τ_2[ns]	1.943	±0.028	1.4%
I_2[kCnts]	391.4	±5.2	1.3%
A_3[kCnts/Chnl]	5.68	±0.12	2.1%
τ_3[ns]	0.534	±0.025	4.6%
I_3[kCnts]	121.1	±6.9	5.7%
$Bkgr_{Dec}$[kCnts]	0.0235	±0.0003	1.2%
$Bkgr_{IRF}$[Cnts/Chnl]	0.28	±0.12	42%
$Shift_{IRF}$[ps]	10.0	±1.1	11%
A_{Scatt}[kCnts]	36.7	±1.2	3.2%
τ_{AvInt}[ns]	3.227	±0.013	0.4%
τ_{AvAmp}[ns]	1.579	±0.016	1.0%
λ_{Det}[nm]	480.0000	---	---

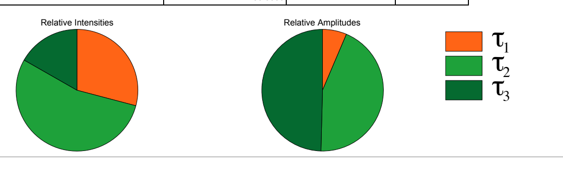

Data Set: 6 / 16

Decay: crv[6]; IRF: crv[0]

Fit

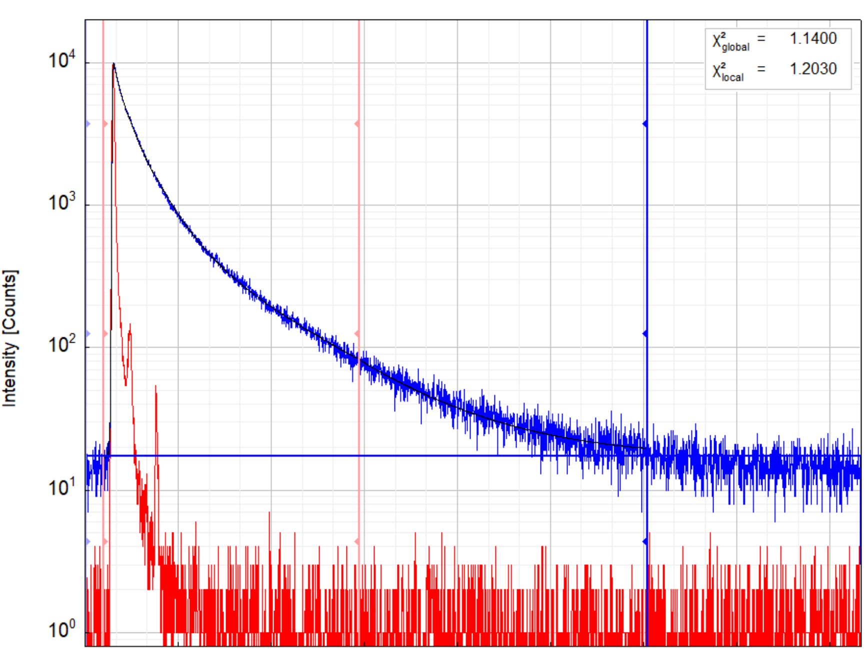

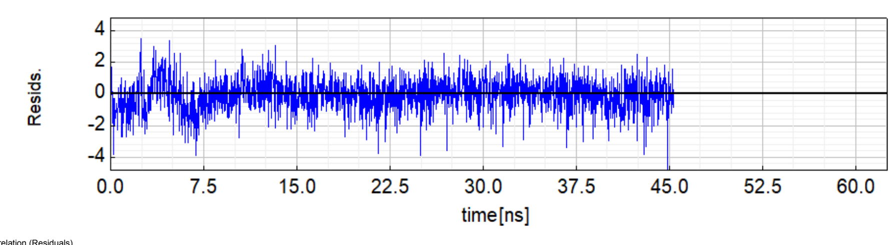

Autocorrelation (Residuals)

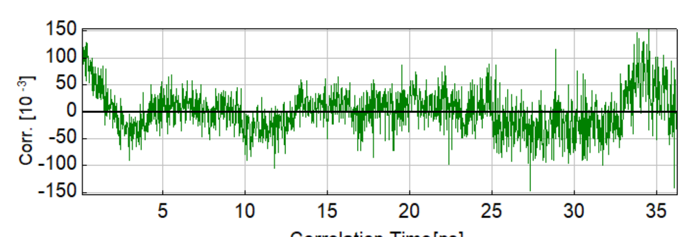

Fitted Parameters

Parameter	Value	Δ	δ
A_1[kCnts/Chnl]	0.953	±0.018	1.8%
τ_1[ns]	7.166	±0.030	0.4%
I_1[kCnts]	273.1	±4.3	1.6%
A_2[kCnts/Chnl]	5.27	±0.14	2.6%
τ_2[ns]	1.943	±0.028	1.4%
I_2[kCnts]	409.3	±5.1	1.2%
A_3[kCnts/Chnl]	5.291	±0.080	1.5%
τ_3[ns]	0.534	±0.025	4.6%
I_3[kCnts]	112.9	±7.6	6.7%
$Bkgr_{Dec}$[kCnts]	0.0173	±0.0002	0.8%
$Bkgr_{IRF}$[Cnts/Chnl]	0.47	±0.13	27%
$Shift_{IRF}$[ps]	12.60	±0.82	6.4%
A_{Scatt}[kCnts]	37.42	±0.83	2.2%
τ_{AvInt}[ns]	3.537	±0.013	0.4%
τ_{AvAmp}[ns]	1.7269	±0.0094	0.5%
λ_{Det}[nm]	500.0000	---	---

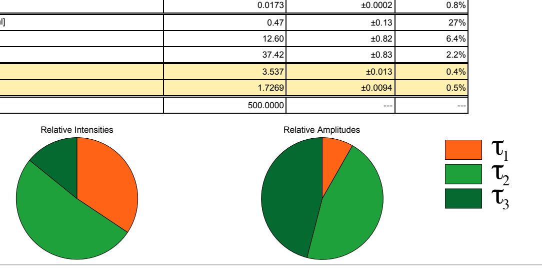

Data Set: 7 / 16

Decay: crv[7]; IRF: crv[0]

Fit

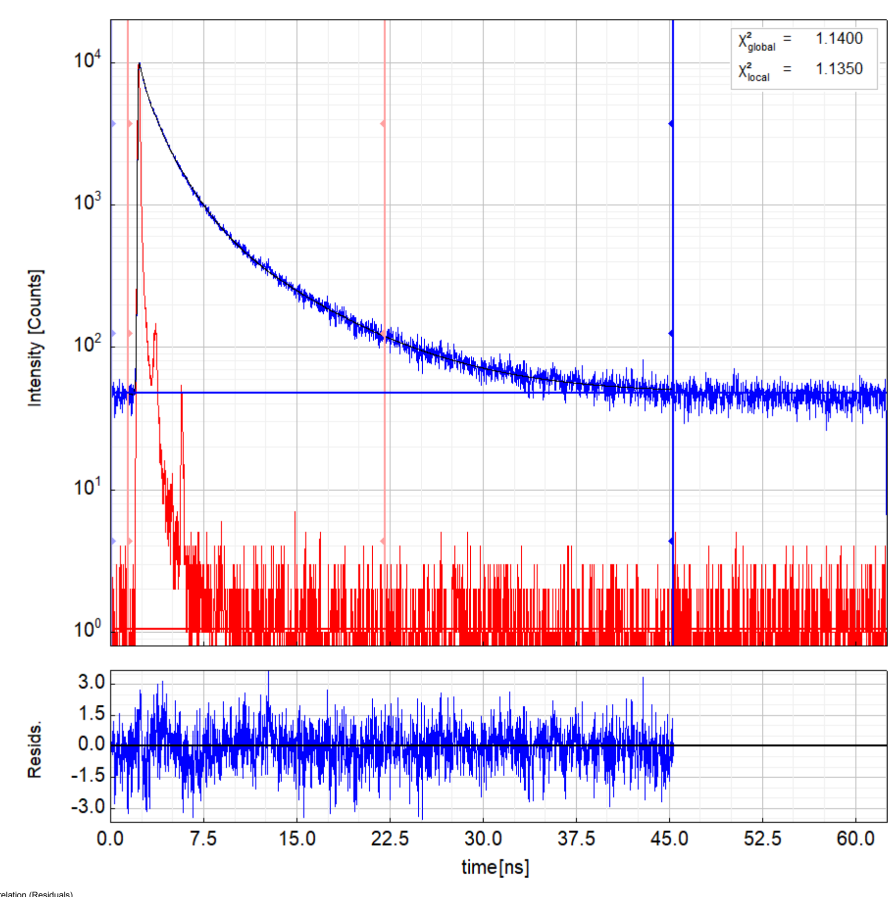

Autocorrelation (Residuals)

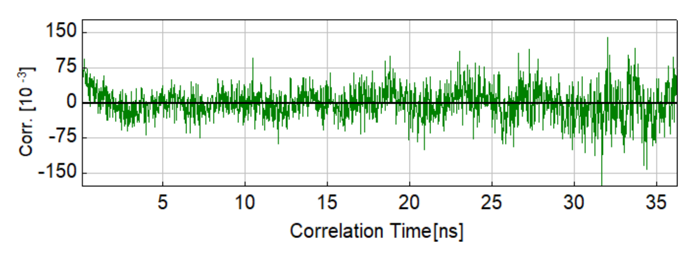

Fitted Parameters

Parameter	Value	Δ	δ
A_1[kCnts/Chnl]	1.133	±0.019	1.6%
τ_1[ns]	7.166	±0.030	0.4%
I_1[kCnts]	324.7	±3.8	1.1%
A_2[kCnts/Chnl]	5.54	±0.13	2.3%
τ_2[ns]	1.943	±0.028	1.4%
I_2[kCnts]	429.9	±3.5	0.8%
A_3[kCnts/Chnl]	4.82	±0.15	2.9%
τ_3[ns]	0.534	±0.025	4.6%
I_3[kCnts]	102.7	±5.5	5.3%
$Bkgr_{Dec}$[kCnts]	0.0478	±0.0002	0.3%
$Bkgr_{IRF}$[Cnts/Chnl]	1.042	±0.080	7.6%
$Shift_{IRF}$[ps]	11.05	±0.69	6.2%
A_{Scatt}[kCnts]	37.05	±0.86	2.3%
τ_{AvInt}[ns]	3.7518	±0.0043	0.1%
τ_{AvAmp}[ns]	1.867	±0.019	1.0%
λ_{Det}[nm]	520.0000	---	---

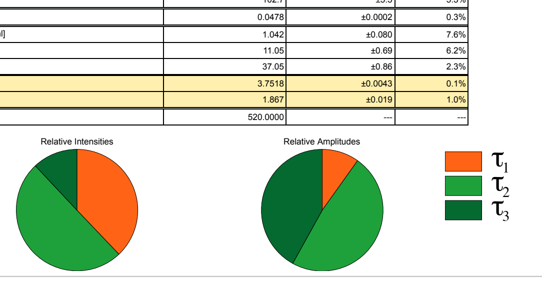

Data Set: 8 / 16

Decay: crv[8]; IRF: crv[0]

Fit

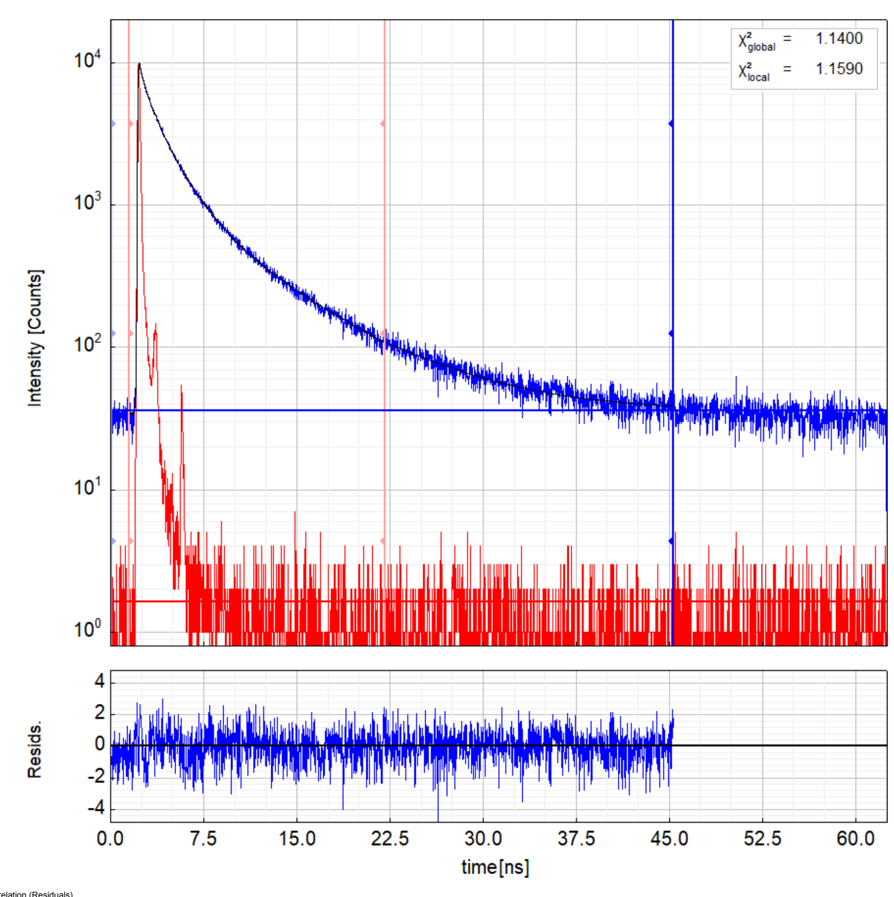

Autocorrelation (Residuals)

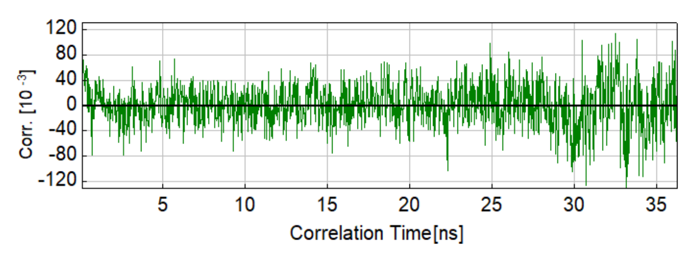

Fitted Parameters

Parameter	Value	Δ	δ
A_1[kCnts/Chnl]	1.241	±0.017	1.4%
τ_1[ns]	7.166	±0.030	0.4%
I_1[kCnts]	355.7	±4.4	1.2%
A_2[kCnts/Chnl]	5.51	±0.14	2.5%
τ_2[ns]	1.943	±0.028	1.4%
I_2[kCnts]	427.5	±3.9	0.9%
A_3[kCnts/Chnl]	4.402	±0.076	1.7%
τ_3[ns]	0.534	±0.025	4.6%
I_3[kCnts]	93.9	±6.9	7.3%
$Bkgr_{Dec}$[kCnts]	0.0357	±0.0003	0.7%
$Bkgr_{IRF}$[Cnts/Chnl]	1.63	±0.15	8.6%
$Shift_{IRF}$[ps]	12.88	±0.67	5.2%
A_{Scatt}[kCnts]	38.44	±0.67	1.7%
τ_{AvInt}[ns]	3.910	±0.011	0.3%
τ_{AvAmp}[ns]	1.968	±0.014	0.7%
λ_{Det}[nm]	540.0000	---	---

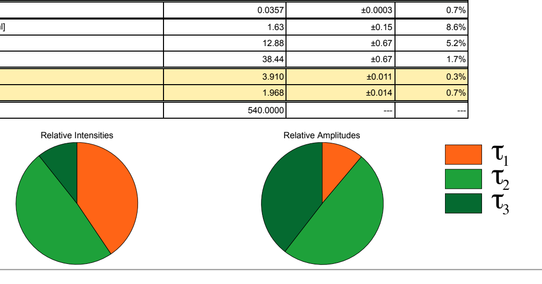

Data Set: 9 / 16

Decay: crv[9]; IRF: crv[0]

Fit

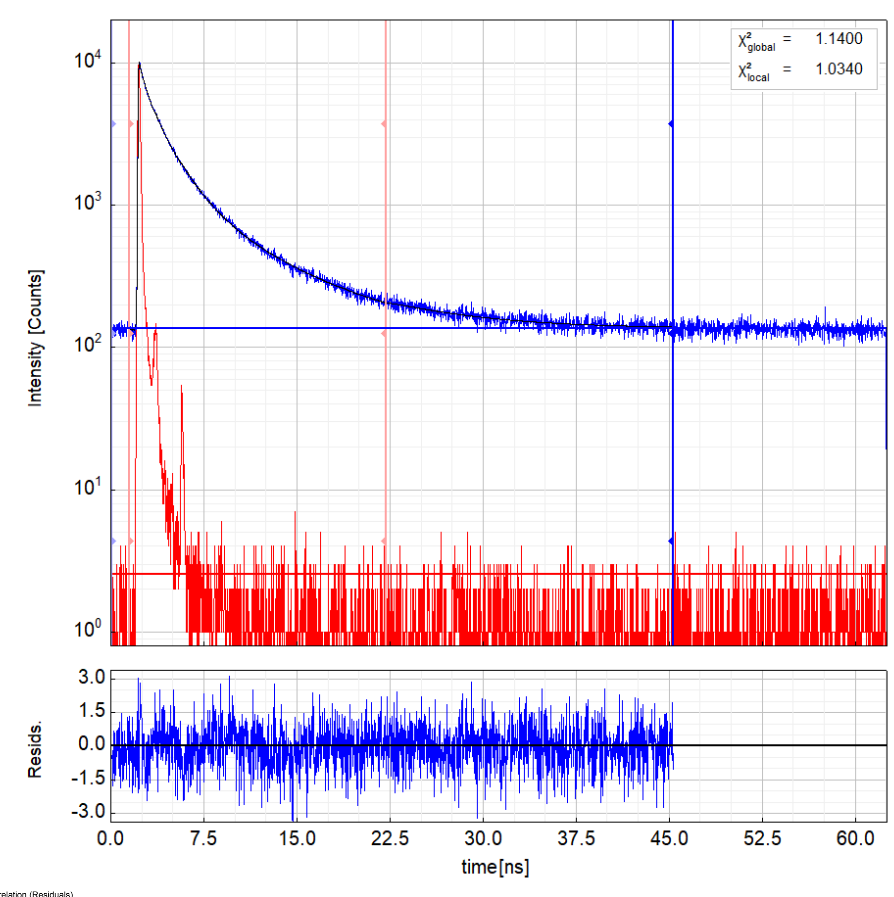

Autocorrelation (Residuals)

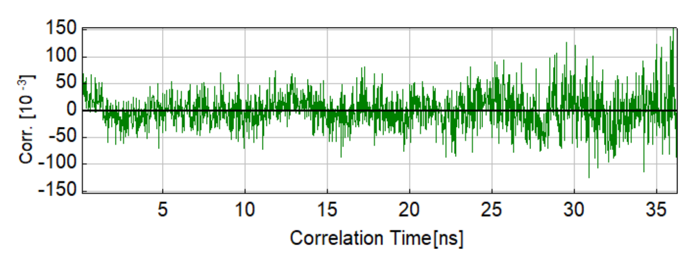

Fitted Parameters

Parameter	Value	Δ	δ
A_1[kCnts/Chnl]	1.356	±0.016	1.2%
τ_1[ns]	7.166	±0.030	0.4%
I_1[kCnts]	388.7	±3.6	0.9%
A_2[kCnts/Chnl]	5.39	±0.11	2.0%
τ_2[ns]	1.943	±0.028	1.4%
I_2[kCnts]	418.3	±3.6	0.8%
A_3[kCnts/Chnl]	4.31	±0.14	3.0%
τ_3[ns]	0.534	±0.025	4.6%
I_3[kCnts]	91.8	±4.6	5.0%
$Bkgr_{Dec}$[kCnts]	0.1361	±0.0005	0.3%
$Bkgr_{IRF}$[Cnts/Chnl]	2.531	±0.099	3.9%
$Shift_{IRF}$[ps]	13.16	±0.78	5.9%
A_{Scatt}[kCnts]	39.70	±0.93	2.3%
τ_{AvInt}[ns]	4.058	±0.017	0.4%
τ_{AvAmp}[ns]	2.035	±0.023	1.1%
λ_{Det}[nm]	560.0000	---	---

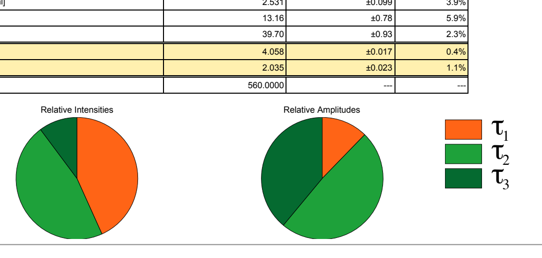

Data Set: 10 / 16

Decay: crv[10]; IRF: crv[0]

Fit

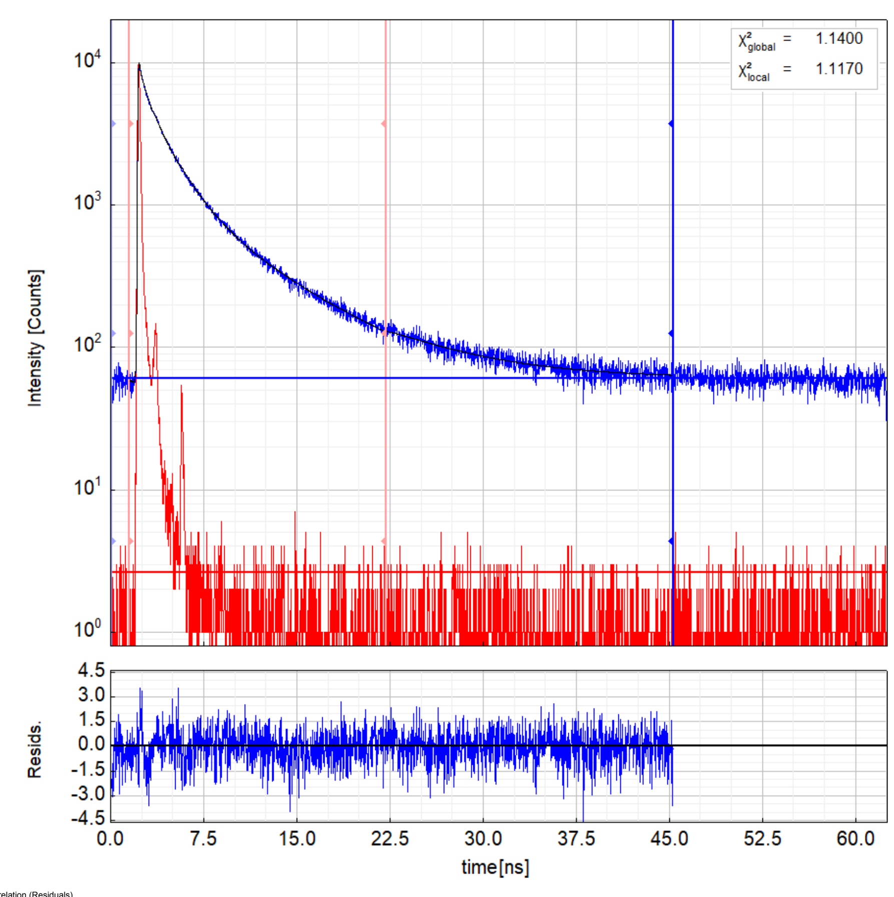

Autocorrelation (Residuals)

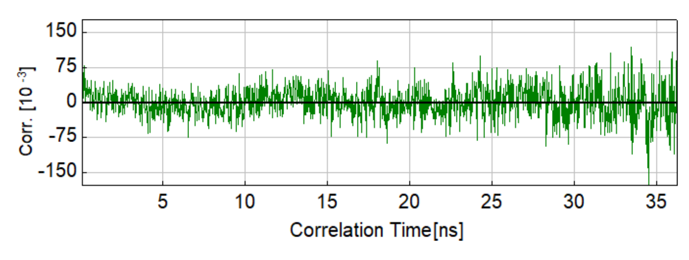

Fitted Parameters

Parameter	Value	Δ	δ
A_1[kCnts/Chnl]	1.347	±0.023	1.7%
τ_1[ns]	7.166	±0.030	0.4%
I_1[kCnts]	386.0	±4.5	1.2%
A_2[kCnts/Chnl]	5.17	±0.14	2.5%
τ_2[ns]	1.943	±0.028	1.4%
I_2[kCnts]	401.6	±3.6	0.9%
A_3[kCnts/Chnl]	4.03	±0.12	2.8%
τ_3[ns]	0.534	±0.025	4.6%
I_3[kCnts]	85.9	±5.7	6.6%
$Bkgr_{Dec}$[kCnts]	0.0608	±0.0004	0.5%
$Bkgr_{IRF}$[Cnts/Chnl]	2.64	±0.11	4.1%
$Shift_{IRF}$[ps]	14.6	±1.2	8.0%
A_{Scatt}[kCnts]	42.6	±1.4	3.1%
τ_{AvInt}[ns]	4.113	±0.012	0.3%
τ_{AvAmp}[ns]	2.072	±0.022	1.0%
λ_{Det}[nm]	580.0000	---	---

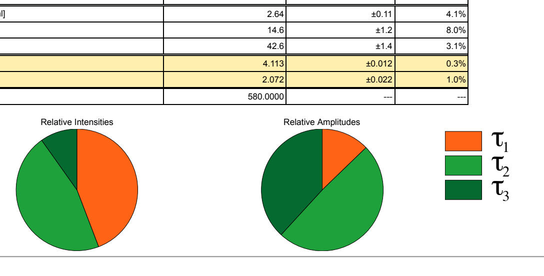

Data Set: 11 / 16

Decay: crv[11]; IRF: crv[0]

Fit

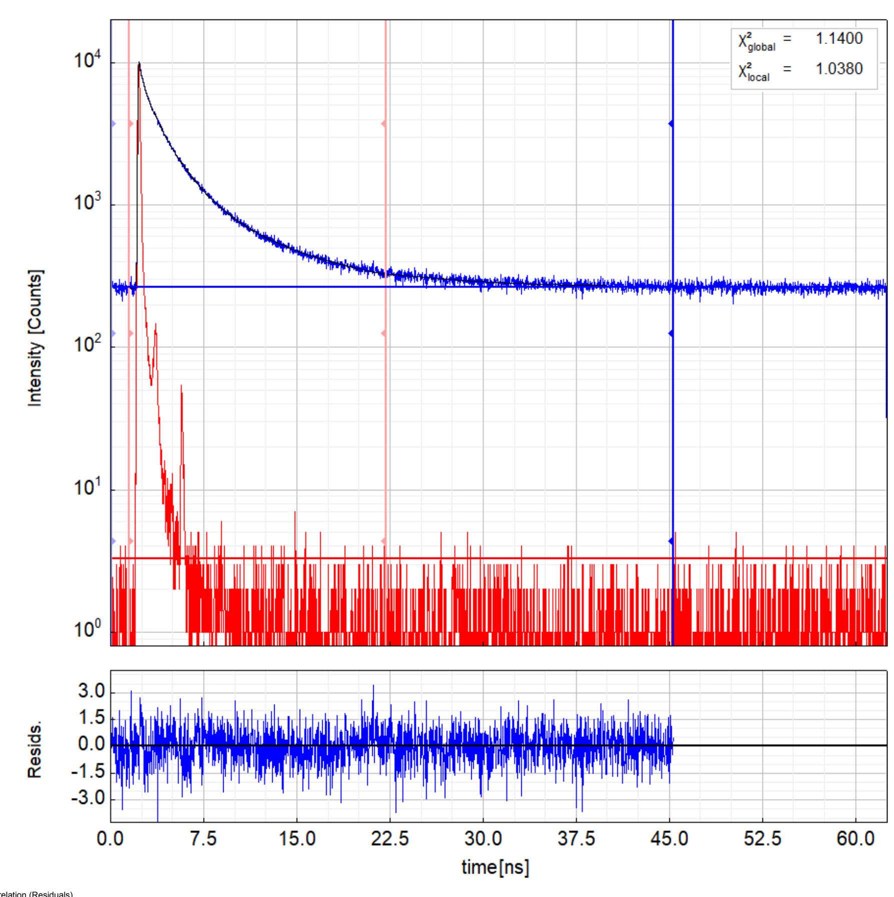

Autocorrelation (Residuals)

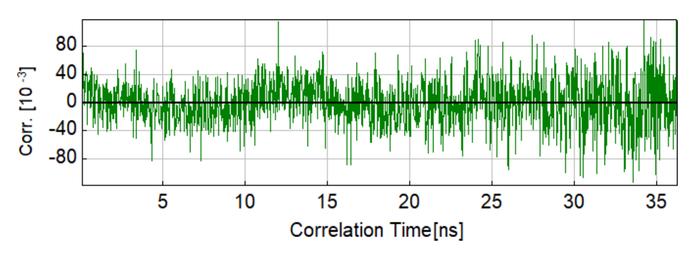

Fitted Parameters

Parameter	Value	Δ	δ
A_1[kCnts/Chnl]	1.328	±0.024	1.8%
τ_1[ns]	7.166	±0.030	0.4%
I_1[kCnts]	380.6	±5.1	1.3%
A_2[kCnts/Chnl]	5.03	±0.13	2.4%
τ_2[ns]	1.943	±0.028	1.4%
I_2[kCnts]	390.2	±4.1	1.0%
A_3[kCnts/Chnl]	3.915	±0.072	1.8%
τ_3[ns]	0.534	±0.025	4.6%
I_3[kCnts]	83.5	±5.5	6.5%
$Bkgr_{Dec}$[kCnts]	0.2645	±0.0004	0.1%
$Bkgr_{IRF}$[Cnts/Chnl]	3.26	±0.14	4.2%
$Shift_{IRF}$[ps]	14.53	±0.27	1.8%
A_{Scatt}[kCnts]	43.81	±0.64	1.4%
τ_{AvInt}[ns]	4.132	±0.012	0.3%
τ_{AvAmp}[ns]	2.081	±0.019	0.9%
λ_{Det}[nm]	600.0000	---	---

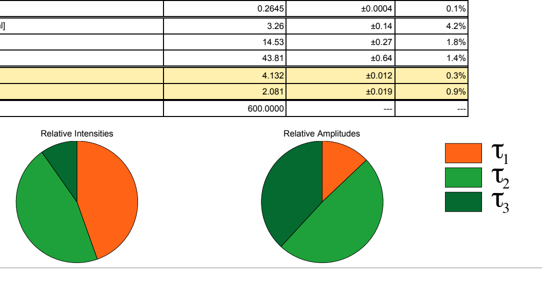

Data Set: 12 / 16

Decay: crv[12]; IRF: crv[0]

Fit

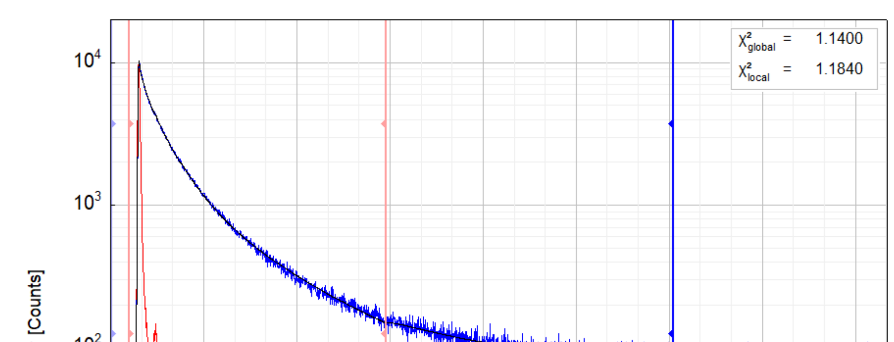

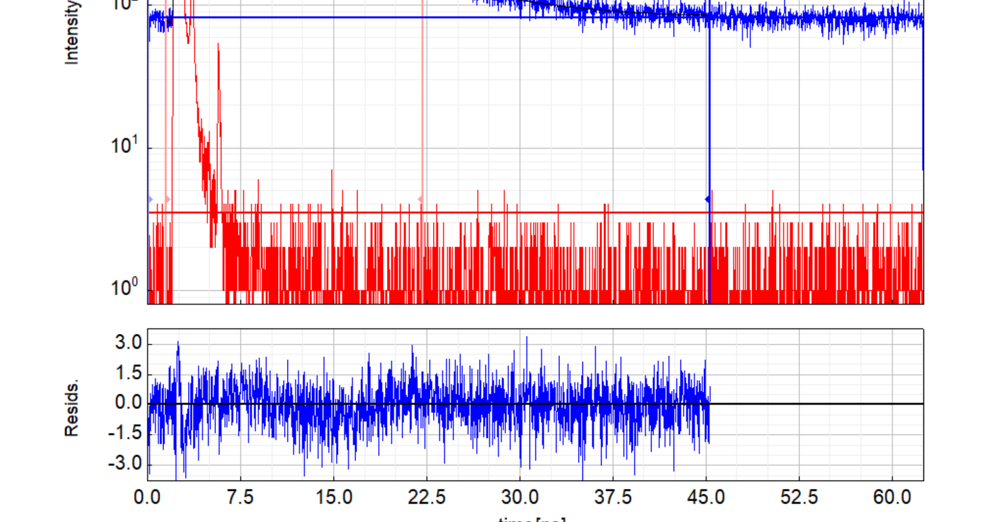

Autocorrelation (Residuals)

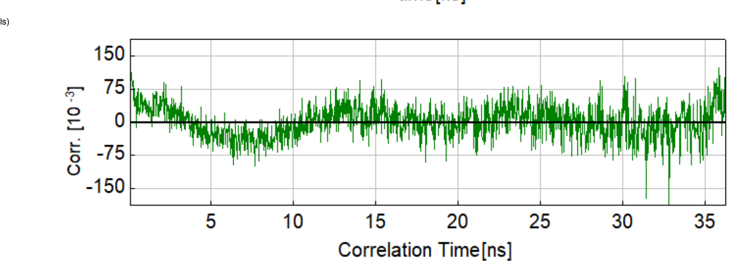

Fitted Parameters

Parameter	Value	Δ	δ
A_1[kCnts/Chnl]	1.496	±0.020	1.3%
τ_1[ns]	7.166	±0.030	0.4%
I_1[kCnts]	428.7	±5.0	1.2%
A_2[kCnts/Chnl]	5.249	±0.096	1.8%
τ_2[ns]	1.943	±0.028	1.4%
I_2[kCnts]	407.8	±2.3	0.6%
A_3[kCnts/Chnl]	3.50	±0.17	4.6%
τ_3[ns]	0.534	±0.025	4.6%
I_3[kCnts]	74.5	±5.8	7.8%
$Bkgr_{Dec}$[kCnts]	0.0817	±0.0006	0.7%
$Bkgr_{IRF}$[Cnts/Chnl]	3.48	±0.21	5.9%
$Shift_{IRF}$[ps]	13.68	±0.93	6.7%
A_{Scatt}[kCnts]	45.8	±1.2	2.4%
τ_{AvInt}[ns]	4.286	±0.014	0.3%
τ_{AvAmp}[ns]	2.225	±0.013	0.6%
λ_{Det}[nm]	620.0000	---	---

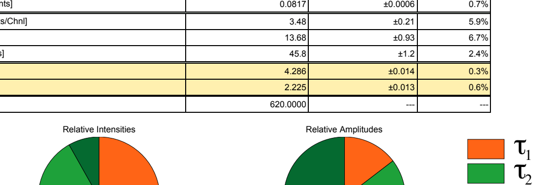

Data Set: 13 / 16

Decay: crv[13]; IRF: crv[0]

Fit

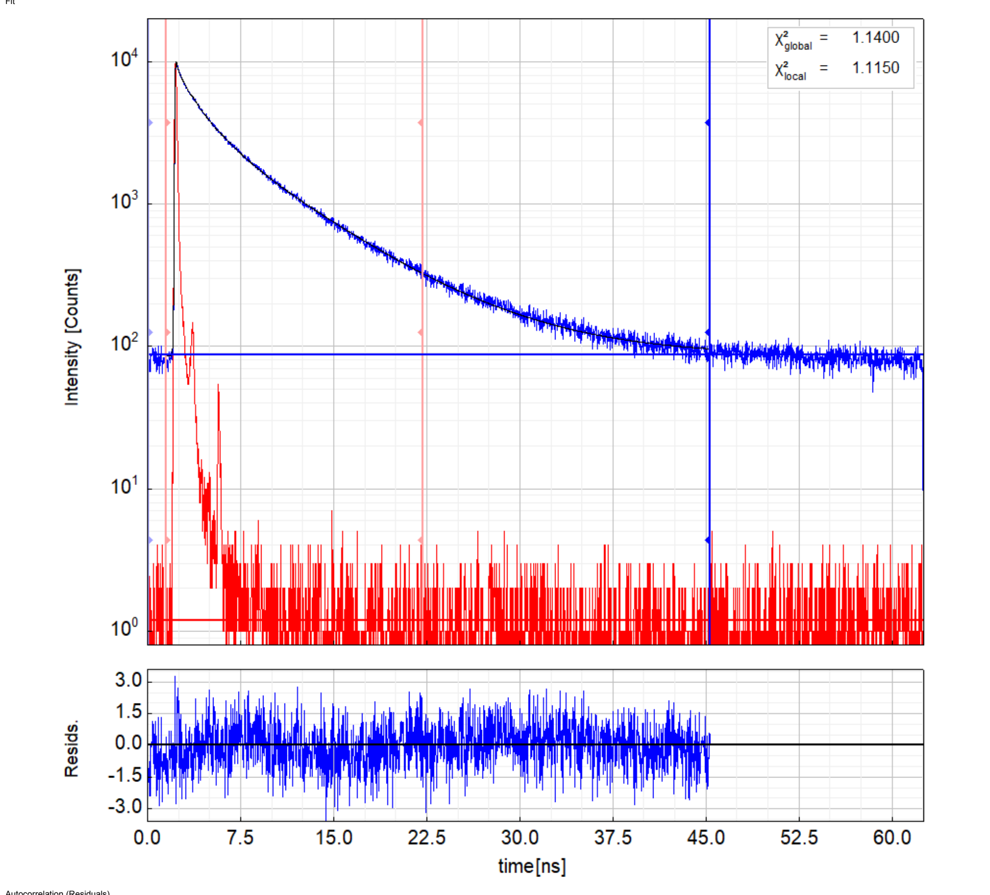

Autocorrelation (Residuals)

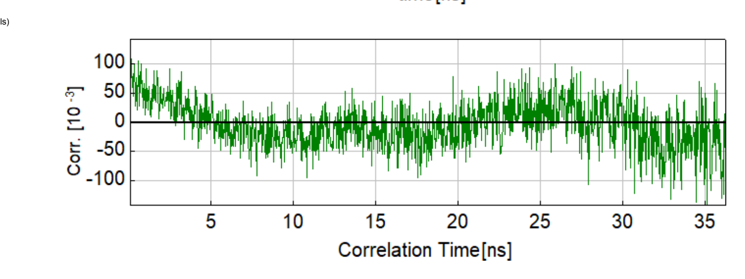

Fitted Parameters

Parameter	Value	Δ	δ
A_1[kCnts/Chnl]	3.837	±0.022	0.6%
τ_1[ns]	7.166	±0.030	0.4%
I_1[kCnts]	1 099.8	±3.0	0.3%
A_2[kCnts/Chnl]	4.415	±0.091	2.0%
τ_2[ns]	1.943	±0.028	1.4%
I_2[kCnts]	343.0	±3.3	0.9%
A_3[kCnts/Chnl]	2.403	±0.088	3.6%
τ_3[ns]	0.534	±0.025	4.6%
I_3[kCnts]	51.3	±4.0	7.7%
$Bkgr_{Dec}$[kCnts]	0.0867	±0.0004	0.4%
$Bkgr_{IRF}$[Cnts/Chnl]	1.188	±0.080	6.7%
$Shift_{IRF}$[ps]	15.74	±0.72	4.5%
A_{Scatt}[kCnts]	40.34	±0.97	2.4%
τ_{AvInt}[ns]	5.740	±0.013	0.2%
τ_{AvAmp}[ns]	3.506	±0.017	0.5%
λ_{Det}[nm]	640.0000	---	---

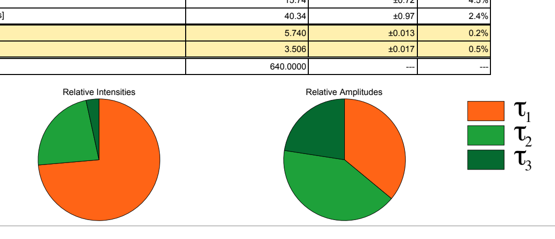

Data Set: 14 / 16

Decay: crv[14]; IRF: crv[0]

Fit

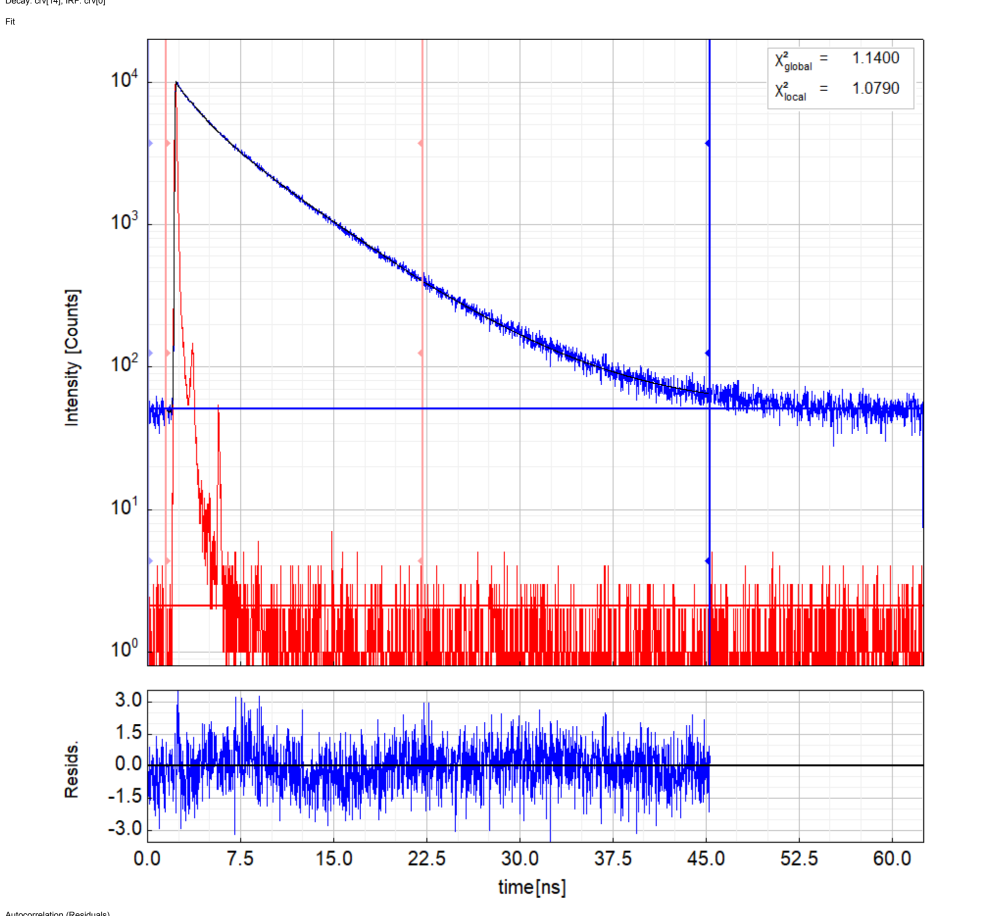

Autocorrelation (Residuals)

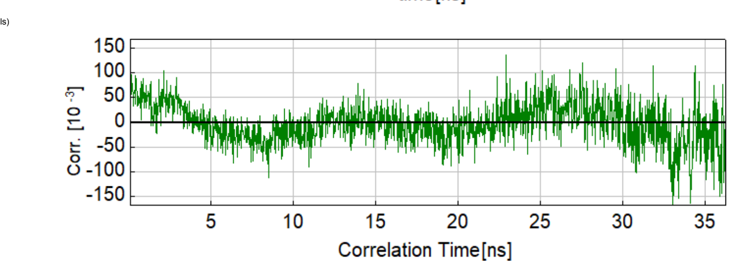

Fitted Parameters

Parameter	Value	Δ	δ
A_1[kCnts/Chnl]	5.951	±0.033	0.5%
τ_1[ns]	7.166	±0.030	0.4%
I_1[kCnts]	1 705.7	±5.5	0.3%
A_2[kCnts/Chnl]	4.196	±0.061	1.4%
τ_2[ns]	1.943	±0.028	1.4%
I_2[kCnts]	326.0	±3.4	1.0%
A_3[kCnts/Chnl]	0.919	±0.088	9.5%
τ_3[ns]	0.534	±0.025	4.6%
I_3[kCnts]	19.6	±2.7	13%
$Bkgr_{Dec}$[kCnts]	0.0511	±0.0003	0.5%
$Bkgr_{IRF}$[Cnts/Chnl]	2.109	±0.094	4.4%
$Shift_{IRF}$[ps]	16.88	±0.74	4.4%
A_{Scatt}[kCnts]	36.1	±1.1	3.1%
τ_{AvInt}[ns]	6.273	±0.019	0.3%
τ_{AvAmp}[ns]	4.635	±0.024	0.5%
λ_{Det}[nm]	660.0000	---	---

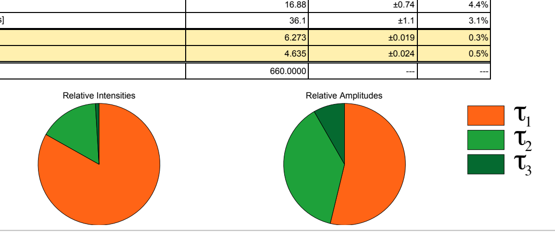

Data Set: 15 / 16

Decay: crv[15]; IRF: crv[0]

Fit

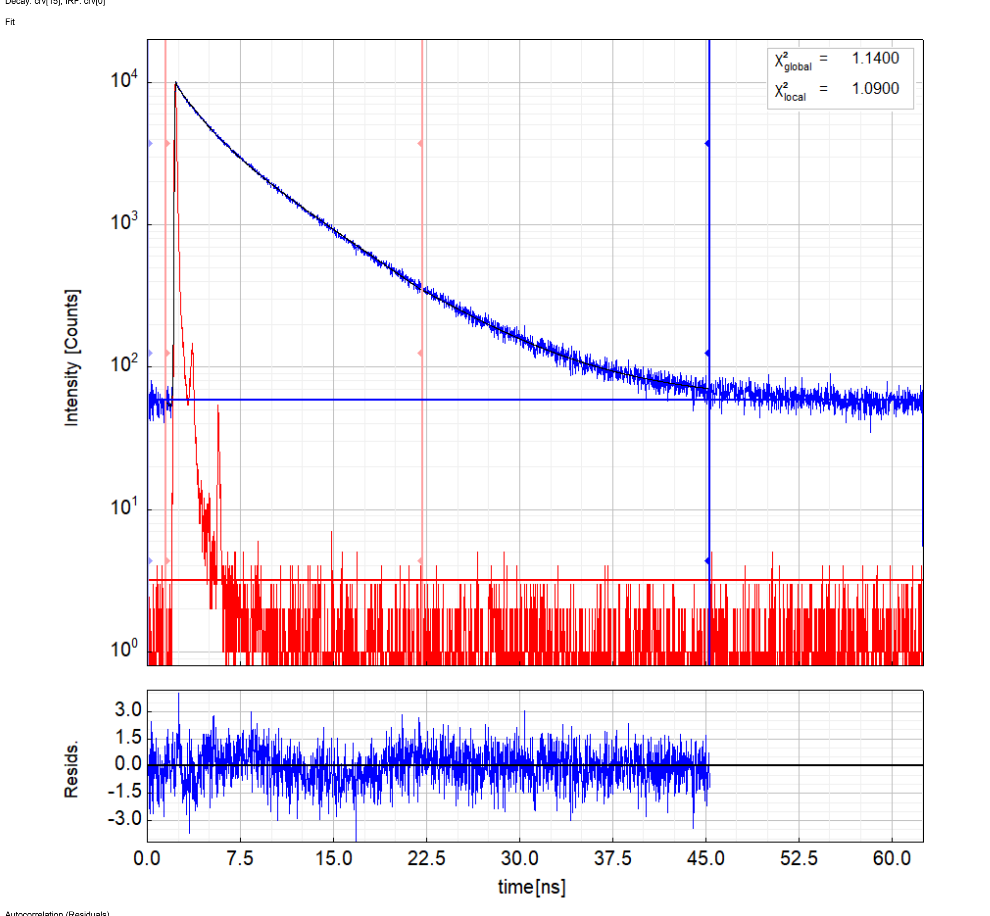

Autocorrelation (Residuals)

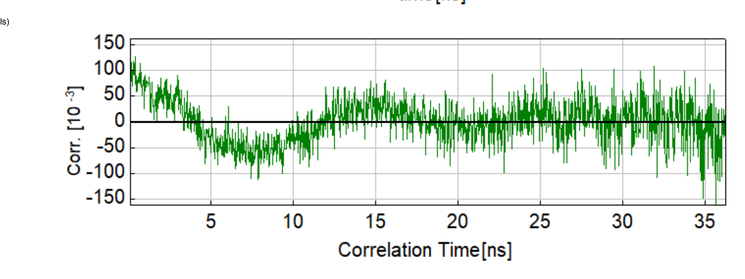

Fitted Parameters

Parameter	Value	Δ	δ
A_1[kCnts/Chnl]	5.311	±0.048	0.9%
τ_1[ns]	7.166	±0.030	0.4%
I_1[kCnts]	1 522.1	±7.8	0.5%
A_2[kCnts/Chnl]	4.641	±0.090	1.9%
τ_2[ns]	1.943	±0.028	1.4%
I_2[kCnts]	360.5	±8.7	2.4%
A_3[kCnts/Chnl]	0.87	±0.12	13%
τ_3[ns]	0.534	±0.025	4.6%
I_3[kCnts]	18.5	±2.0	10%
$Bkgr_{Dec}$[kCnts]	0.0589	±0.0004	0.6%
$Bkgr_{IRF}$[Cnts/Chnl]	3.20	±0.17	5.1%
$Shift_{IRF}$[ps]	18.24	±0.36	2.0%
A_{Scatt}[kCnts]	38.04	±0.54	1.4%
τ_{AvInt}[ns]	6.111	±0.015	0.2%
τ_{AvAmp}[ns]	4.395	±0.022	0.5%
λ_{Det}[nm]	680.0000	---	---

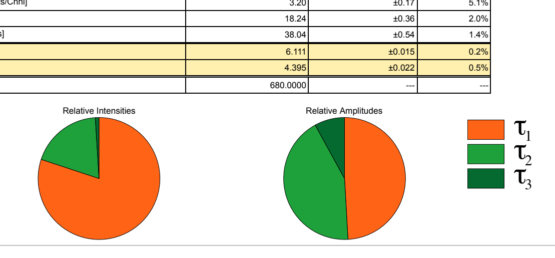

Data Set: 16 / 16

Decay: crv[16]; IRF: crv[0]

Fit

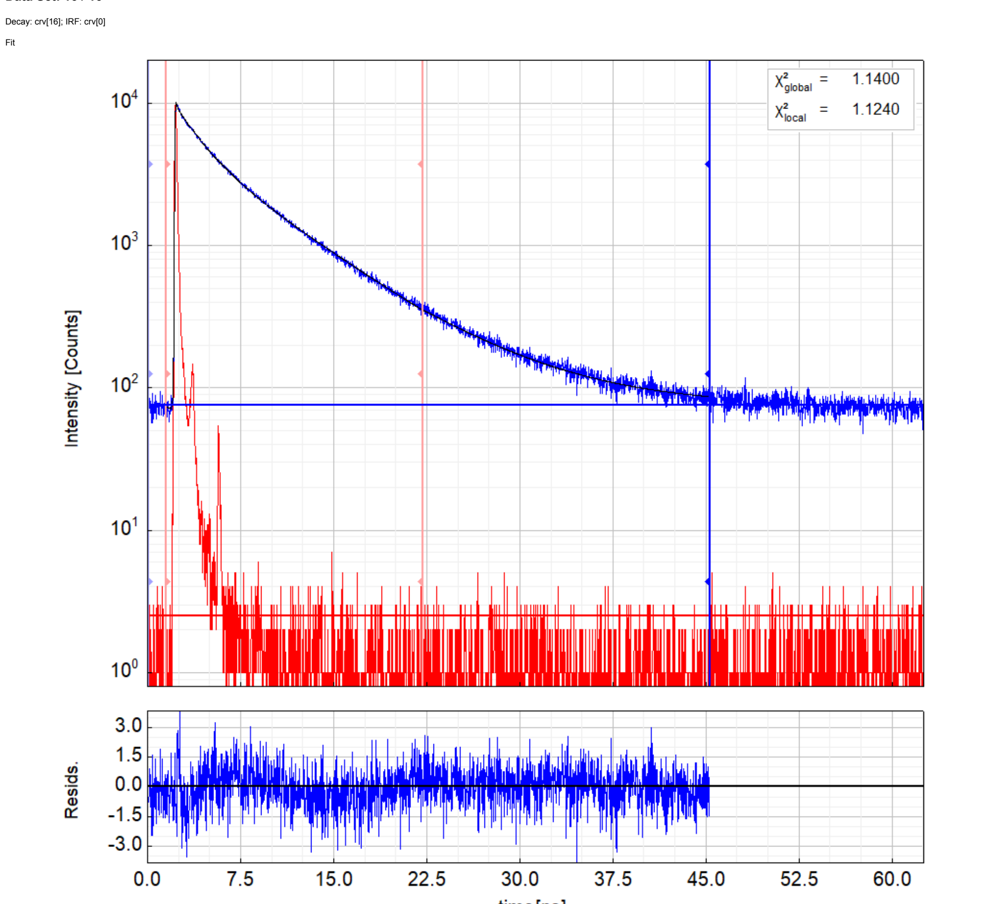

Autocorrelation (Residuals)

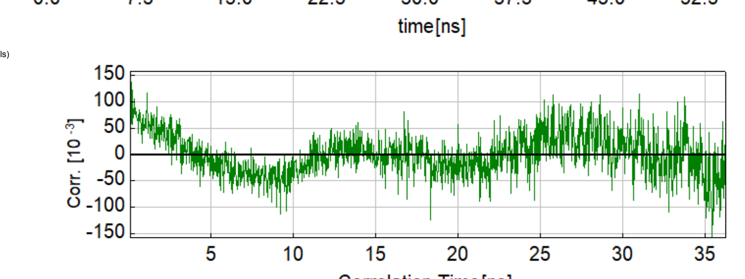

Fitted Parameters

Parameter	Value	Δ	δ
A_1[kCnts/Chnl]	4.881	±0.039	0.8%
τ_1[ns]	7.166	±0.030	0.4%
I_1[kCnts]	1 399.0	±6.1	0.4%
A_2[kCnts/Chnl]	4.695	±0.030	0.6%
τ_2[ns]	1.943	±0.028	1.4%
I_2[kCnts]	364.8	±5.9	1.6%
A_3[kCnts/Chnl]	1.13	±0.11	9.5%
τ_3[ns]	0.534	±0.025	4.6%
I_3[kCnts]	24.1	±3.4	14%
$Bkgr_{Dec}$[kCnts]	0.0756	±0.0005	0.6%
$Bkgr_{IRF}$[Cnts/Chnl]	2.53	±0.17	6.7%
$Shift_{IRF}$[ps]	18.55	±0.54	2.9%
A_{Scatt}[kCnts]	39.31	±0.81	2.0%
τ_{AvInt}[ns]	6.011	±0.012	0.2%
τ_{AvAmp}[ns]	4.176	±0.030	0.7%
λ_{Det}[nm]	700.0000	---	---

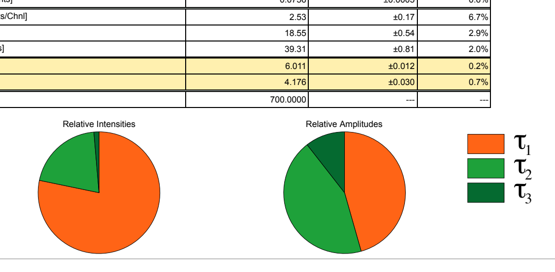

Parameter Plot

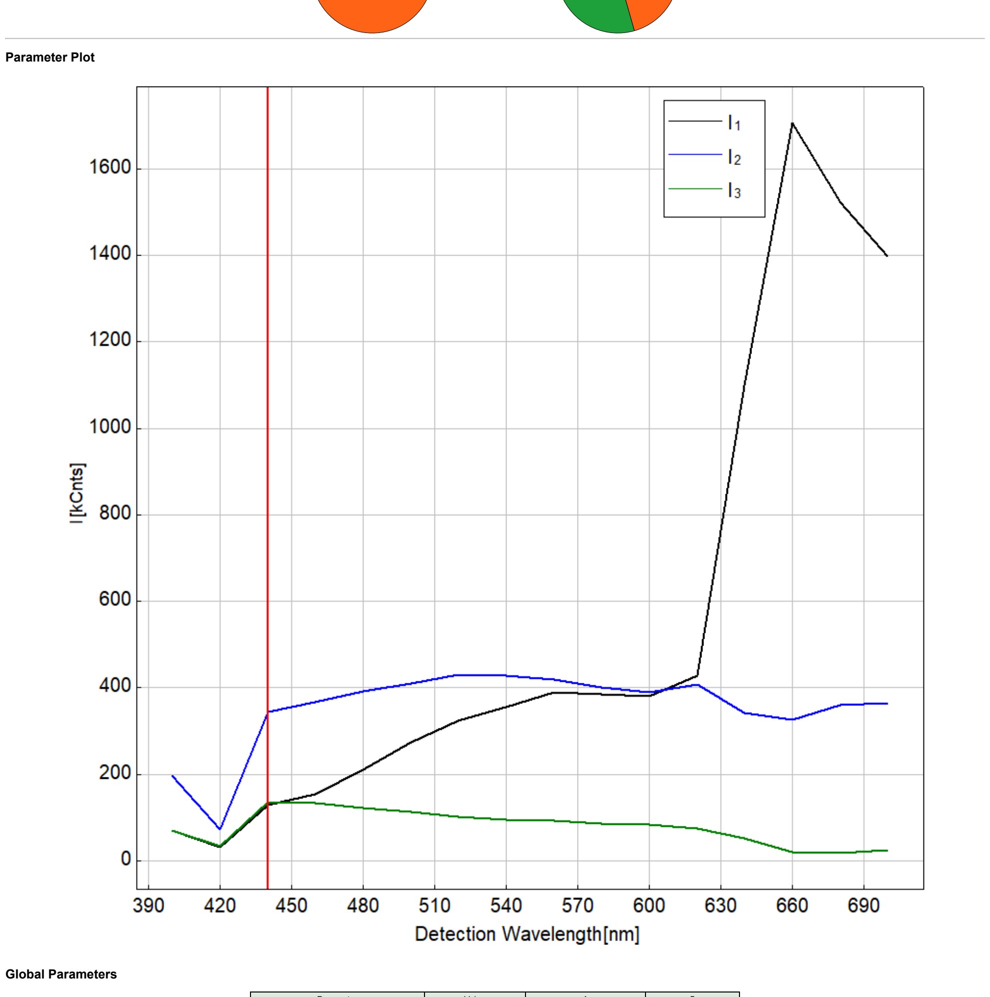

Global Parameters

Parameter	Value	Δ	δ
τ_1[ns]	7.166	±0.030	0.4%
τ_2[ns]	1.943	±0.028	1.4%
τ_3[ns]	0.534	±0.025	4.6%
