# Supplementary material for: A Porphyrin‐Based Organic Network Comprising Sustainable Carbon Dots for Photopolymerization
Source: Angew Chem Int Ed Engl. 2022 Sep 1;61(40):e202208180. doi: 10.1002/anie.202208180 (PMC9826160; doi:10.1002/anie.202208180)
Supplement: Supplementary file 8 — Supporting Information [file ANIE-61-0-s006.pdf]

Angewandte Chemie
Eine Zeitschrift der Gesellschaft Deutscher Chemiker

Supporting Information

A Porphyrin-Based Organic Network Comprising Sustainable Carbon Dots for Photopolymerization

X. Luo, J. Wan, N. Meckbach, V. Strehmel, S. Li, Z. Chen, B. Strehmel**

1. Experimental Procedures and methods

1.1 Materials

Furfural aldehyde, Dichloromethane (DCM), Di(1H-pyrrol-2-yl)methane and 2,3-Dichloro-5,6-dicyano-*p*-benzoquinone (DDQ) were received from Sigma-Aldrich. Methyl methacrylate (MMA) and styrene (styrene) were purchased from Sigma Aldrich. The inhibitor was removed from both monomers prior to use. Dimethyl sulfoxide (DMSO, anhydrous, 99.8%), Copper(II)bromide ($CuBr_2$), tris(2-pyridylmethyl) amine (TPMA, 98%), ethyl α-bromophenylacetate (EBPA, 97%) and methanol (99.9%) were obtained from Sigma-Aldrich. All these reagents were used as received unless otherwise mentioned. All other chemicals needed for the experiments were received from Sigma-Aldrich as well and used as received.

1.2 Synthetic method and photopolymerization

1.2.1 Synthesis of F-CDs and P-CDs

Furfural aldehyde (2.5 mL) and ethanol (25 mL) were treated using hydrothermal method at 180°C for 8 h following a procedure from the literature[1]. After that, carbon nanodots (F-**CD**s) powders were obtained via freeze drying. F-**CD**s (3 mL) were then mixed with Di(1H-pyrrol-2-yl)methane (0.5 g) in DCM as solvent (5 mL). It followed stirring under N_2 atmosphere for 3h after adding 0.5 mL trifluoracetic acid. Then, 0.6g DDQ (2,3-dichloro-5,6-dicyano-*p*-benzoquinone.) in 10 mL DCM was added to continue stirring for additional 4h. After this time, 30 mL saturated sodium hydroxide solution and large amounts of ethanol were added to precipitate the products overnight. The porphyrin-based carbon nanodots (P-**CD**s) were obtained by washing the precipitation with deionization water.

1.2.2 Photo-ATRP Procedure

Photo-ATRP procedure was described previously[2,3]. Typically, Schlenk flask with a magnetic stirrer was degassed by four Freeze-Pump-Thaw cycles. After that, P-CDs solution (1mg mL^{-1}, 3 mL, DMSO) was mixed with MMA (1 mL), $CuBr_2$ solution (5.20 µL, 180 mM, DMSO), TPMA solution (15.6 µL, 270 mM, DMSO) and ethyl α-bromophenylacetate (5.5 µL). The mixture was irradiated at 405 nm during stirring. Exposure was stopped and polymers were precipitated in cold methanol. Thus, one received a mixture comprising polymer and P-CDs in the filter. After

drying the isolated material in air, it was dissolved again in THF during stirring. Then, the P-CDs were removed by centrifugation at 5000 rpm for 10min. The liquid supernatant was put into cold methanol again to precipitate the polymers again. The collected polymers were dried in the vacuum oven at 50℃ for 24h. This was repeated until H-NMR spectra showed no appearance of residual monomer.

The use of surfactants in the photoinduced polymerization was carried out in a similar way. Specifically, 7 mg (8 mM) and 21 mg (24 mM) sodium dodecylsulfate (SDS) were dissolved in 3 mL DMSO, respectively. Then, 3 mg P-**CD**s were added and dispersed by sonification for 10 mins. After that, the mixture of the SDS and P-**CD**s was obtained. The next steps were the same as *vide supra* disclosed.

1.3 Characterization

1.3.1 Fluorescence measurements

The regular fluorescence set up (FluoTime 300 from PICOQUANT) was carried for recording fluorescence emission λ_{max}, to take decay time τ_f, and quantum yield Φ_f with the excitation from diode laser LDH-P-C-375 emitting at 376 nm. Time correlated single photon counting was the technique to take the emission decay. This instrument from PICOQUANT provides the opportunity to measure decay between 25 ps and several milliseconds. The instrument was equipped with high resolution emission double monochromator operated in subtractive mode (2x 300 mm focal length, UV/VIS/IR grating pair 1st stage: 600 l / mm blaze 1250 nm and 1200 l / mm blaze 500 nm, dispersion: 4.5 nm / mm, UV / VIS grid pair 2nd stage: 1200 l / mm blaze 500 nm 1200 l / mm blaze 500 nm (-), 2.7 nm / mm (-), PMA-C 192-M Cooled photomultiplier) for the emission side. Excitation proceeded by using a Computer controlled diode laser driver for picosecond pulses (196 kHz and 80 MHz, external trigger input). EasyTau software and laser control software controlled the system. This also facilitated to calculate decay time by the software by iterative convolution. An appropriate scatter (Ludox in water) was taken to record the instrumental response function of the system. Time resolution of the instrument is >25 ps. For preparation, the sample was dissolved in methanol (spectroscopic grade) to get the absorption of extinction of < 0.1 at 677 nm. The EasyTau Software also operated to calculate the decay time by iterative convolution between the instrumental response and the sample. The EasyTau Software also contained Global Analysis tool. Here, the decay was measured at different wavelengths. Data obtained were linked to a global analysis data set. The number of

contributing decays were kept to a minimum so that a reasonable global χ^2 was obtained for the linked components.
The system can be also operated in *cw*-mode to record spectra in additive mode (monochromator: blaze 500 nm (+), dispersion: 1,4 (+)). This was used to take the excitation spectra.
The solutions investigated had a concentration of 3 g in 10 mL solvent.

1.3.2 UV-Visible spectroscopy

UV-Vis spectra were recorded by Agilent 8453 UV-Vis Spectrometer.

1.3.3 Fourier transform-infrared (FT-IR) spectroscopy

FT-IR spectra were recorded using a Fourier FT-IR spectrometer (Perkin Elmer Inc., Waltham, MA, USA) in ATR mode.

1.3.4 Transmission Electron Microscopy (TEM)

TEM was conducted using a JEM 2100 transmission electron microscope (JEOL Ltd., Tokyo, Japan), at an accelerating voltage of 200 kV. Ceshigo research service pursued the experiments.

1.3.5 X-ray photoelectron spectroscopy (XPS)

X-ray photoelectron spectroscopy (XPS) was performed using a ESCALAB 250Xi thermal spectrometer (ThermoFisher Scientific, Waltham, MA, USA) with a monochromatic Al Kα X-ray source. Ceshigo research service pursued the experiments.

1.3.6 Cyclic voltammetry (CV)

Electrochemical experiments were performed with an CHI660E potentiostat (Chenhua Company, Shanghai, China) in a conventional three-electrode cell. The electrode assembly consists of a platinum wire as the counter electrode, a standard calomel electrode(SCE) as the reference electrode, and a glass carbon (GC) electrode as the working electrode. The measurement buffer contains 0.2 M Na_2SO_4 and 5 mM $[Fe(CN)_6]^{4-/3-}$ as redox indicator. Cyclic voltammetry (CV) was recorded in the range from −1 to 1 V with a scan rate of 0.01 V s^{-1}. To prepare the samples, 20mg CDs was dissolved in 1 mL ethanol, then coating in the glass carbon

electrode and drying at 60°C on the oven. A CHI660E electrochemistry workstation (Chenhua Company, Shanghai, China) was used for the instruments.

1.3.7 Photocurrent measurement

Photocurrent was performed using CHI660E potentiostat (Chenhua Company, Shanghai, China). P-CDs solution (800 uL, 1 mg mL^{-1}, DMSO) was mixed with naphthol (100 uL) assisted by sonification for 30 min. After that, the mixture was dropped on the ITO coated glass and dried in the air for 24 h for the measurement. The coated ITO glass worked as the working electrode and Na_2SO_4 (0.5 M) was used as buffer. The photocurrent was recorded under 405-nm-LED irradiation.

1.3.9 Gel Permeation Chromatography (GPC)

Gel Permeation Chromatography (GPC) measurement was performed at GPC Viscotek 270 max using TGuard Col 10 x 4.6 mm and two T6000M General Mixed 3000 x 7.8 mm columns, a column temperature of 35°C, an RI detector, and tetrahydrofuran (THF) as an eluent at a flow rate of 1 mL min^{-1}.

1.3.10 NMR Spectroscopy

All ^{1}H-NMR-measurements were performed at Fourier 300 from Bruker. Around 10 mg sample were dissolved in 0.7 mL deuterated solvent for the measurement. This was pursued for all polymers before taking GPC data to ensure the absence of low molecular weight compounds.

2 Complementary Results

2.1 Characterization of P-CDs

2.1.1 FT-IR of F-CDs and P-CDs

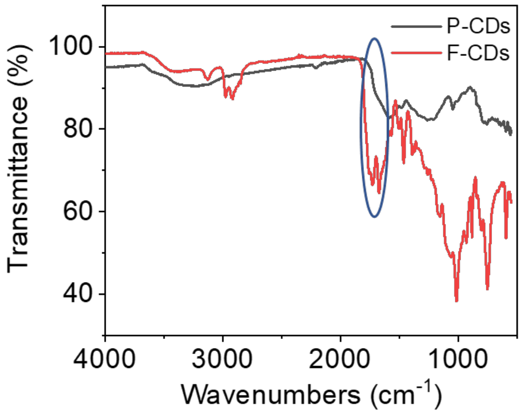

Figure S1. FT-IR spectra of F-**CDs** and P-**CDs** (Signals at around 1720 cm^{-1} belong to aldehyde moieties) obviously decreased after converting F-CDs to P-CDs, suggesting aldehyde moieties of F-CDs were well reacted in Alder-Longo reaction).

2.1.2 XRD pattern of P-CDs

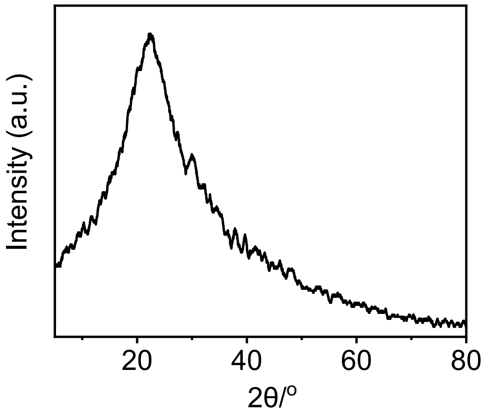

Figure S2. XRD pattern of P-**CDs**. A characteristic diffraction peak was observed around 22°, which is a typical signal resulting from carbon dots and similar to those of reported results.[reference is missing]

2.1.3 UV-Vis absorbance of F-CDs and P-CDs

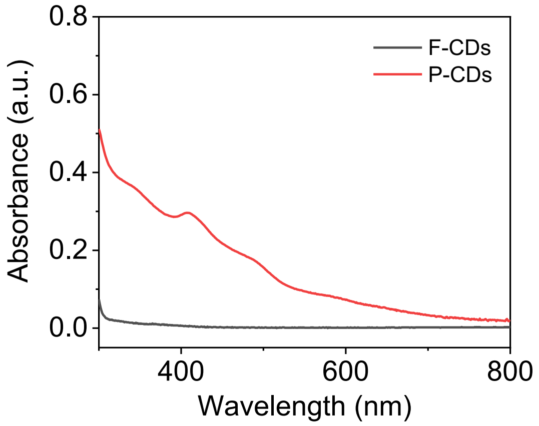

Figure S3. UV-Vis spectrum of F-**CD**s and P-**CD**s in DMSO solution (0.02 mg mL^{-1}). P-**CD**s showed much more absorbance in the visible region than F-**CD**s, indicating that it could efficiently utilize visible light

2.1.4 Cyclic voltametric (CV) spectra of P-CDs

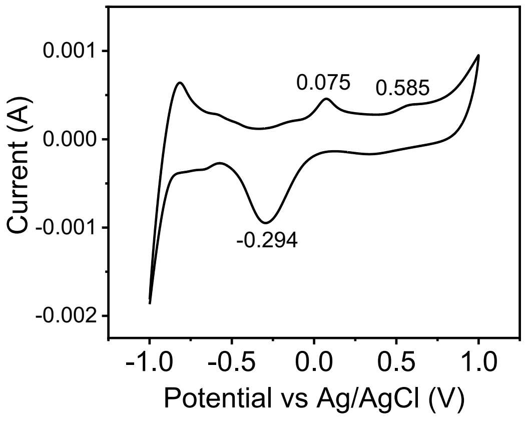

Figure S4. CV spectra of P-**CD**s. (P–**CD**s had an oxidation potential (Eox) of 0.58 V and reduction potential (E_{red}) of –0.29 V)

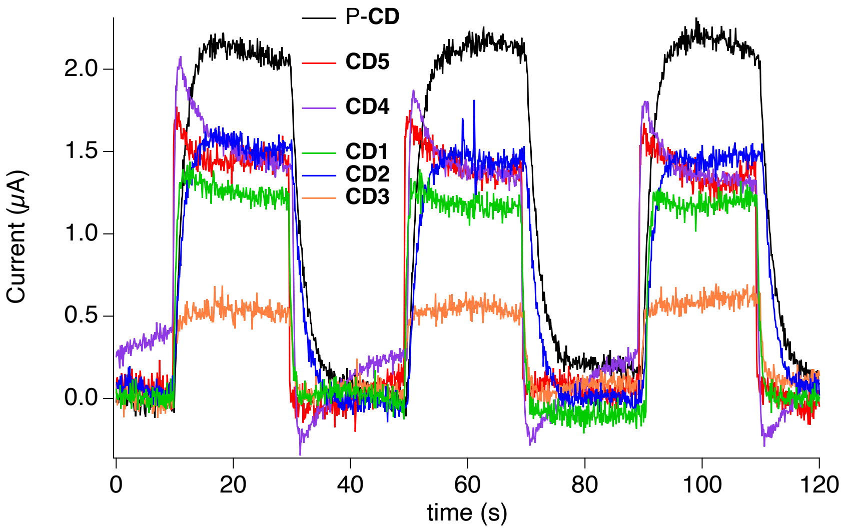

Figure S5. Photocurrent experiments of different P-**CD**s (**CD**-1: lacaic acid based, **CD**-2: lacaic acid based with amine, **CD**-3: lacaic acid based with increased amine, **CD**-4: sodium alginate, **CD**-5: citric acid). **CD**-1 – **CD**-5 were previously disclosed[2,3].

2.1.5 ^{1}H-NMR spectra of P-CDs before and after the reaction

^{1}H-NMR Investigation of the solid material separated from centrifugate resulted in NMR signals assigned to PMMA. The solid material isolated was washed with solvent several times, isolated, and dried at 40°C in vacuum oven. While the neat P-**CD**s exhibit no additional organic matter of the solid particle in a), Figure S6 b) exhibits PMMA signals that shall be assigned to grafted material. Otherwise, the polymer should be washed out if no grafting proceeded.

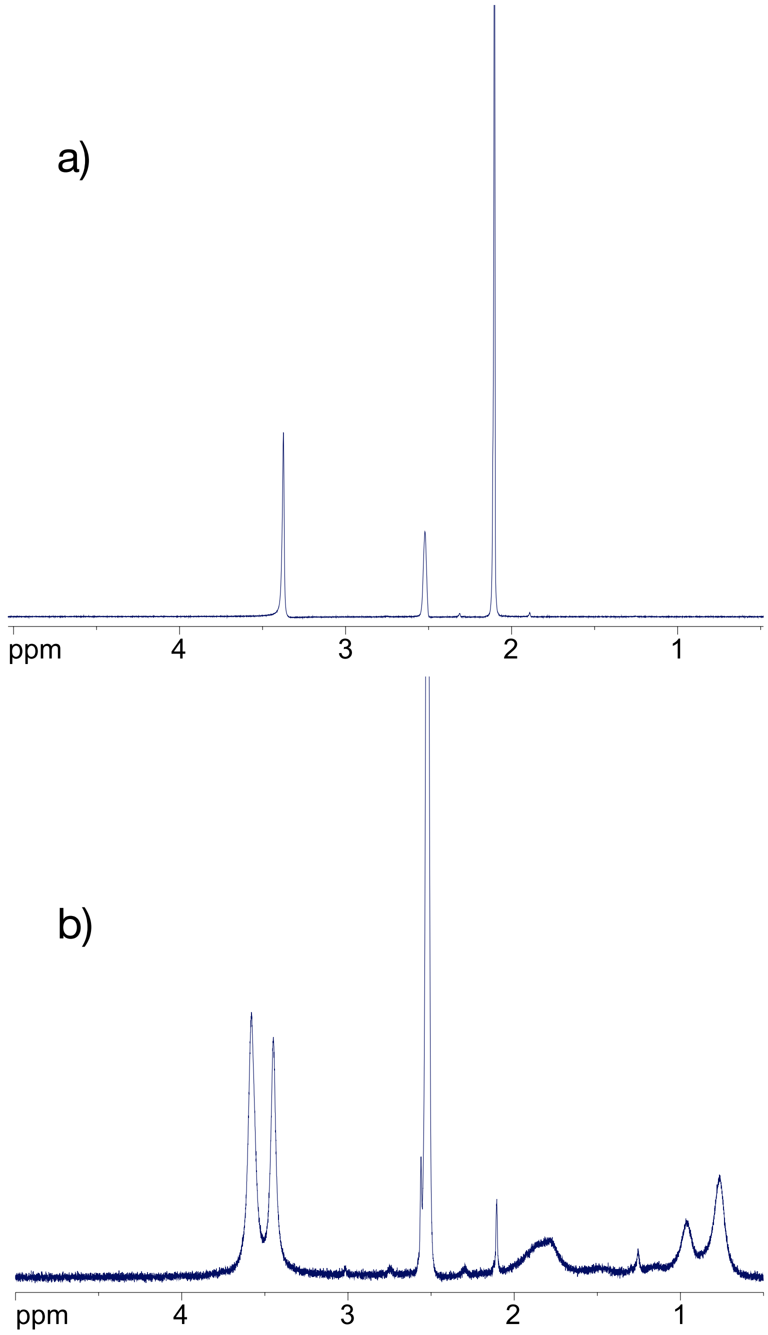

Figure S6. ^{1}HNMR spectra of (a) P-**CD**s and (b) isolated P-**CD**s after polymerization showing PMMA signals.

2.2 Photo ATRP

2.1 On-off experiments

Temporal control in ATRP of MMA was performed by intermittent switching the light on/off. Polymer was obtained upon irradiation for 12 h and no polymerization occurred in the dark in the first cycle. However, no polymer formation was found after the second cycle, which was confirmed by several experiments no matter if the light source was OFF for a short or long time. Blocking of reactive centers by grafted polymer may be one explanation.

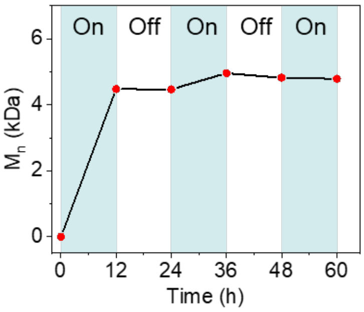

Figure S7. Temporal control in ATRP of MMA by switching on and off the light. Reaction conditions: [MMA]:[I]: [Met]:[TPMA]=300:1:0.03:0.135, conducted in 75% DMSO, $m_{P\text{-}CDs}$=3 mg and irradiated under 405 nm LED (60 mW×cm^{-2})

2.2. Free radical photopolymerization with iodonium salt

Photo-DSC[4] was pursued using the experimental conditions of an earlier contribution[3]. The iodonium salt used was bis[4-(*t*-butyl)phenyl] iodonium tetra(nonafluoro-*t*-butoxy)aluminate available as S 2617 from FEWChemicals GmbH. The concentration on P-CD was 0.6067 mg/mL while S 2617 was loaded with an amount of 15 mM.

2.3. Results of global analysis obtained in DMSO at different wavelengths

Results of Global analysis can be found in additional files submitted.

EasyTau Report

3/11/2022 7:09:02 PM
F:\Paper Xiongfei\CD-1 CH3CN tau global.html

PicoQuant

Container Curves

TRES+IRF_20201222_1747.etc: crv[0 - 11]

Container Properties

Measurement Context: Time Resolved Emission Scan
Detection Wavelength: 450.0 - 650.0 (Step: 20.0) nm
Summary:

Sample:	CD_X_1
Solvent:	Acetonitrile
Excitation:	V pol 376±1nm with LDH-P-C-375
Detection:	M pol 450...650nm 20nm steps<br>10000 peak counts<br>grating 1200/500-<br>detector UV-red [PMT]

Comment

Model: Exponential [Reconv.]

$$\mathrm{Dec}(t)=\left[\int_{-\infty}^{t} dt' \left[\mathrm{IRF}(t-Shift_{IRF})-Bkgr_{IRF}\right]\left[\sum_{i=1}^{n_{Exp}} A_i e^{-\frac{t-t'}{\tau_i}} + A_{Scatt}\,\delta(t-t')\right]\right]+Bkgr_{Dec}$$

$$I_m = A_m \tau_m$$

$$A_{Rel\,m} = A_m \Big/ \sum_{\substack{i=1\\A_i>0}}^{n_{Exp}} A_i \qquad I_{Rel\,m} = I_m \Big/ \sum_{\substack{i=1\\I_i>0}}^{n_{Exp}} I_i$$

$$\tau_{Av\,Amp} = \sum_{\substack{i=1\\A_i>0}}^{n_{Exp}} A_i \tau_i \Big/ \sum_{\substack{i=1\\A_i>0}}^{n_{Exp}} A_i \qquad \tau_{Av\,Int} = \sum_{\substack{i=1\\I_i>0}}^{n_{Exp}} I_i \tau_i \Big/ \sum_{\substack{i=1\\I_i>0}}^{n_{Exp}} I_i$$

Data Set: 1 / 11

Decay: crv[1]; IRF: crv[0]

Fit

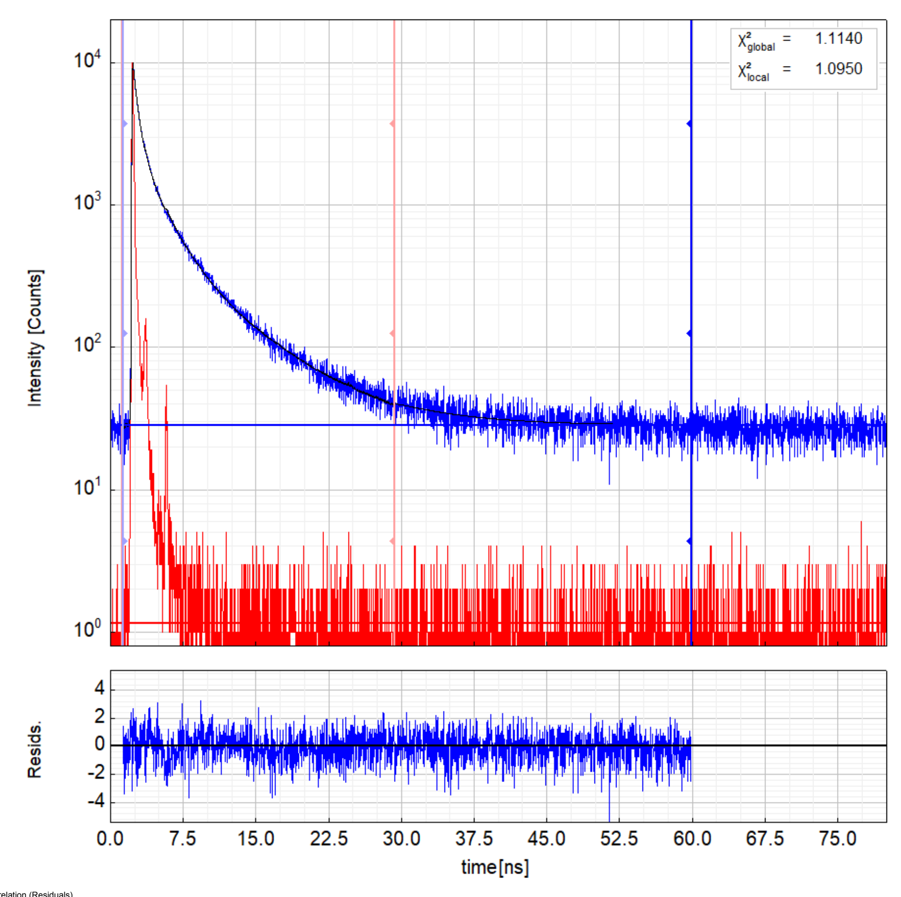

Autocorrelation (Residuals)

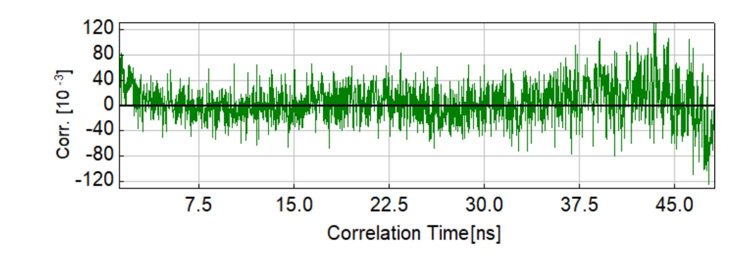

Fitted Parameters

Parameter	Value	Δ	δ
A_1[kCnts/Chnl]	2.279	±0.019	0.8%
τ_1[ns]	2.215	±0.035	1.6%
I_1[kCnts]	201.9	±2.1	1.0%
A_2[kCnts/Chnl]	8.92	±0.12	1.3%
τ_2[ns]	0.4455	±0.0055	1.2%
I_2[kCnts]	158.8	±1.7	1.0%
A_3[kCnts/Chnl]	0.613	±0.011	1.6%
τ_3[ns]	7.088	±0.041	0.6%
I_3[kCnts]	173.7	±2.3	1.3%
$Bkgr_{Dec}$[kCnts]	0.0285	±0.0002	0.6%
$Bkgr_{IRF}$[Cnts/Chnl]	1.147	±0.083	7.2%
$Shift_{IRF}$[ps]	2.4	±1.8	74%
A_{Scatt}[kCnts]	40.5	±1.2	2.9%
τ_{AvInt}[ns]	3.273	±0.015	0.4%
τ_{AvAmp}[ns]	1.1318	±0.0072	0.6%

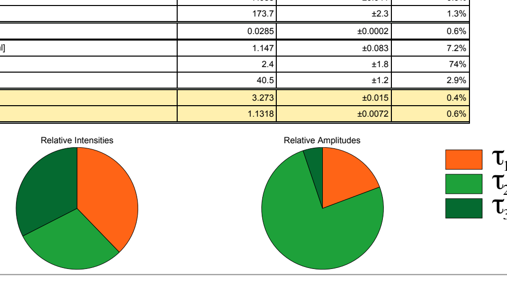

Data Set: 2 / 11

Decay: crv[2]; IRF: crv[0]

Fit

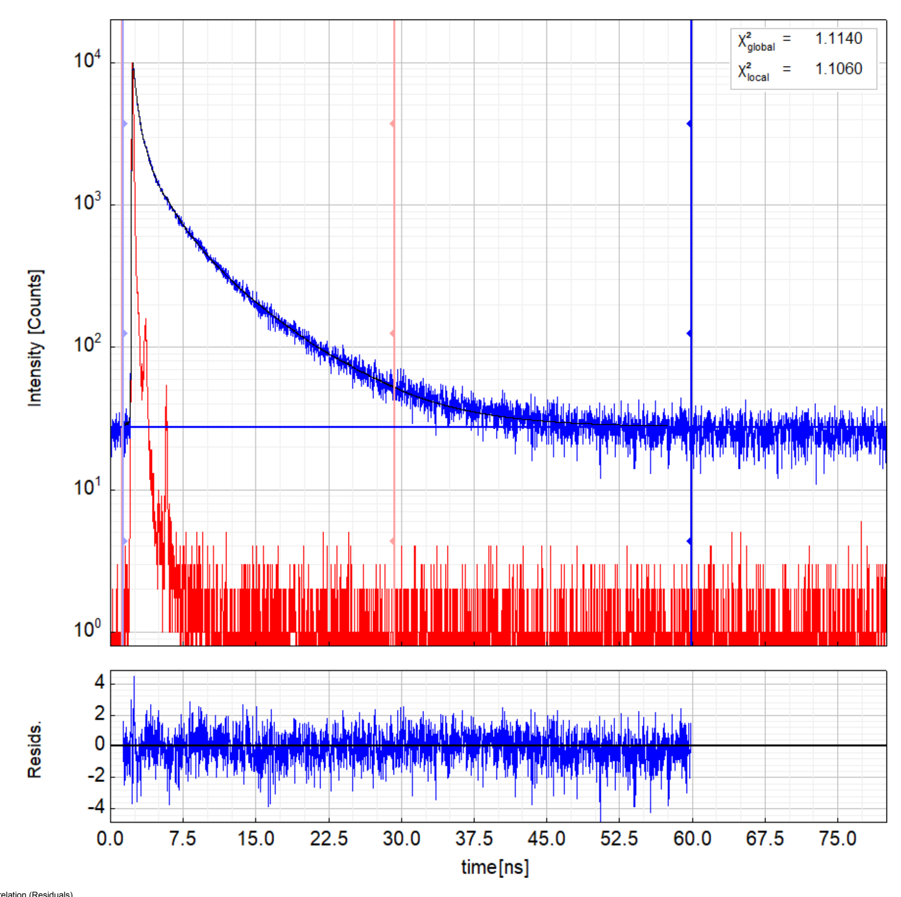

Autocorrelation (Residuals)

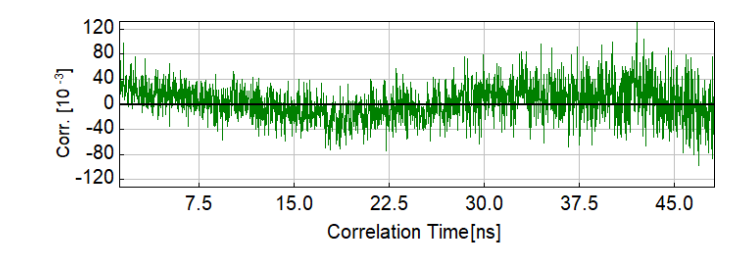

Fitted Parameters

Parameter	Value	Δ	δ
A_1[kCnts/Chnl]	2.051	±0.026	1.2%
τ_1[ns]	2.215	±0.035	1.6%
I_1[kCnts]	181.7	±1.6	0.8%
A_2[kCnts/Chnl]	8.63	±0.12	1.4%
τ_2[ns]	0.4455	±0.0055	1.2%
I_2[kCnts]	153.7	±1.9	1.2%
A_3[kCnts/Chnl]	1.018	±0.013	1.3%
τ_3[ns]	7.088	±0.041	0.6%
I_3[kCnts]	288.4	±2.0	0.7%
$Bkgr_{Dec}$[kCnts]	0.0277	±0.0002	0.7%
$Bkgr_{IRF}$[Cnts/Chnl]	0.49	±0.11	22%
$Shift_{IRF}$[ps]	6.63	±0.91	14%
A_{Scatt}[kCnts]	41.1	±1.2	2.9%
τ_{AvInt}[ns]	4.032	±0.023	0.6%
τ_{AvAmp}[ns]	1.3334	±0.0076	0.6%

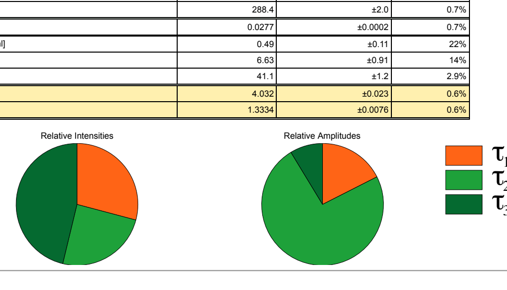

Data Set: 3 / 11

Decay: crv[3]; IRF: crv[0]

Fit

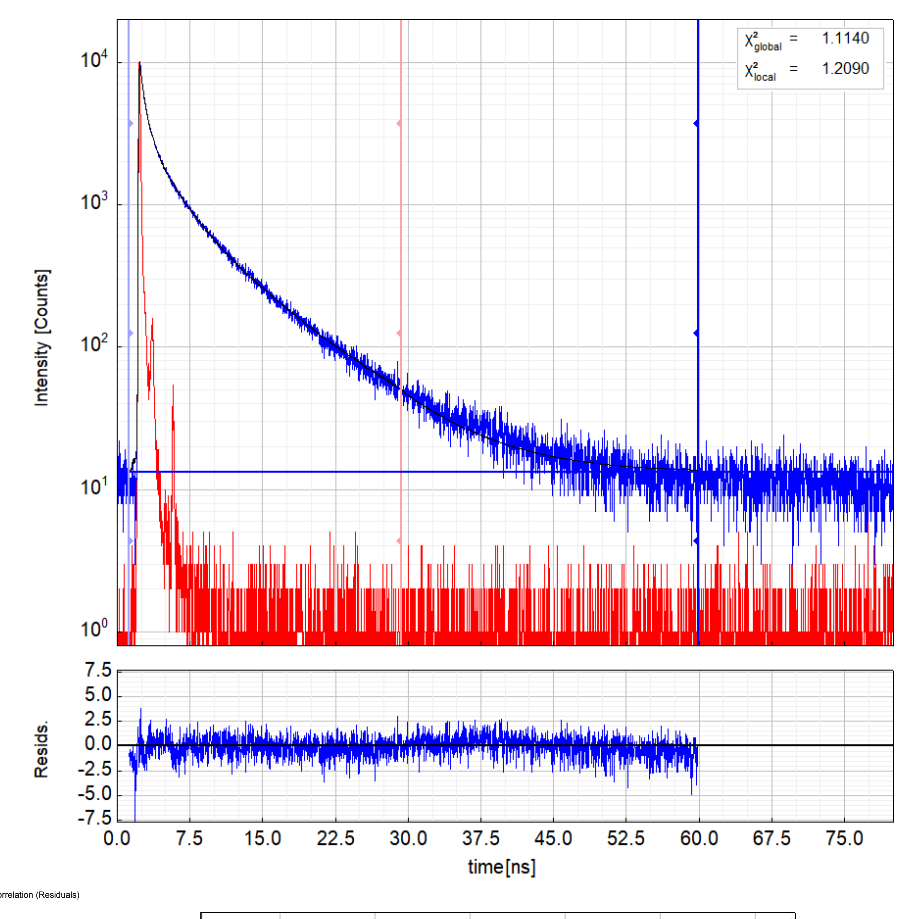

Autocorrelation (Residuals)

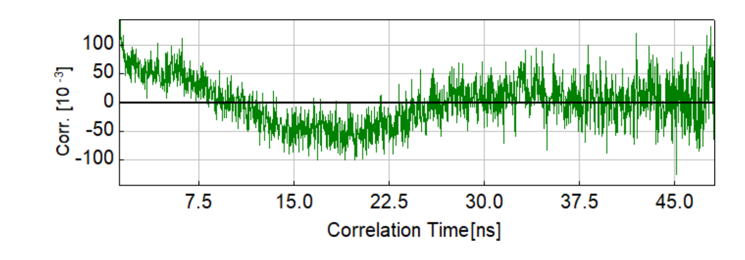

Fitted Parameters

Parameter	Value	Δ	δ
A_1[kCnts/Chnl]	2.453	±0.035	1.4%
τ_1[ns]	2.215	±0.035	1.6%
I_1[kCnts]	217.26	±0.92	0.4%
A_2[kCnts/Chnl]	8.332	±0.081	1.0%
τ_2[ns]	0.4455	±0.0055	1.2%
I_2[kCnts]	148.5	±2.4	1.6%
A_3[kCnts/Chnl]	1.382	±0.014	1.0%
τ_3[ns]	7.088	±0.041	0.6%
I_3[kCnts]	391.6	±2.5	0.6%
$Bkgr_{Dec}$[kCnts]	0.0133	±0.0002	1.0%
$Bkgr_{IRF}$[Cnts/Chnl]	0.03	±0.13	483%
$Shift_{IRF}$[ps]	6.6	±1.1	16%
A_{Scatt}[kCnts]	38.91	±0.73	1.9%
τ_{AvInt}[ns]	4.388	±0.018	0.4%
τ_{AvAmp}[ns]	1.5562	±0.0099	0.6%

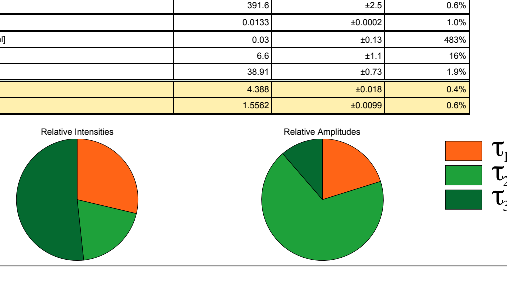

Data Set: 4 / 11

Decay: crv[4]; IRF: crv[0]

Fit

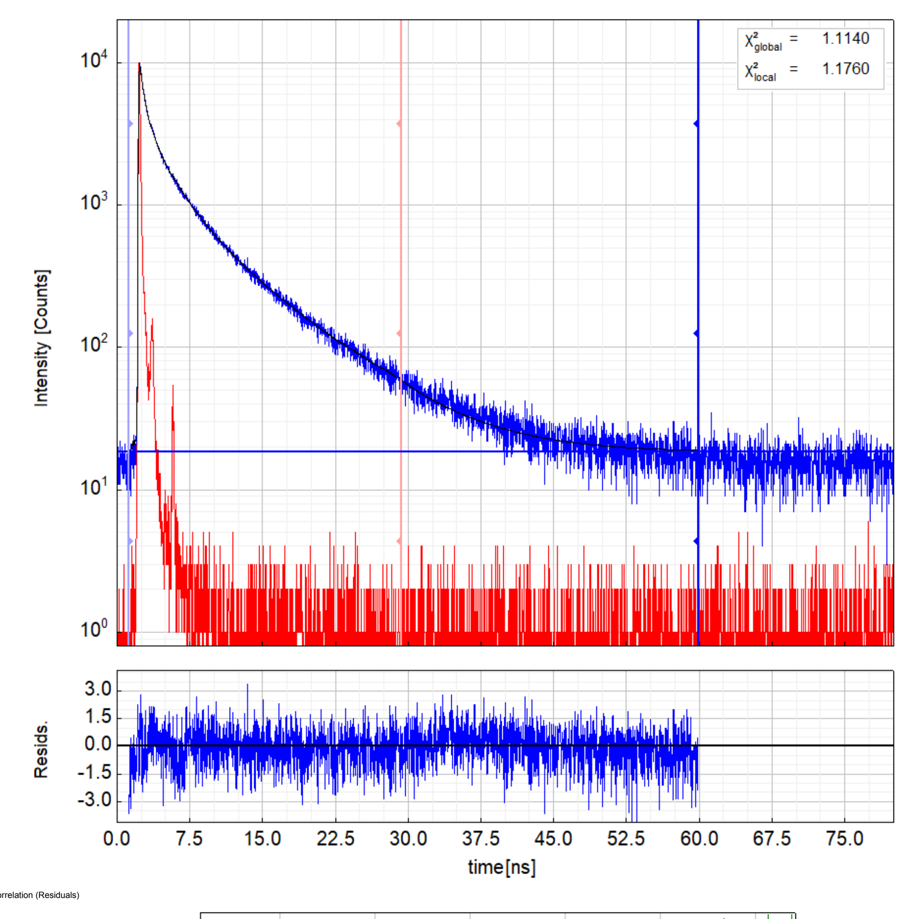

Autocorrelation (Residuals)

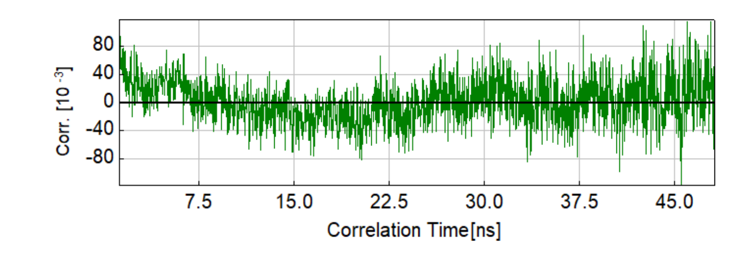

Fitted Parameters

Parameter	Value	Δ	δ
A_1[kCnts/Chnl]	2.950	±0.044	1.5%
τ_1[ns]	2.215	±0.035	1.6%
I_1[kCnts]	261.3	±1.7	0.6%
A_2[kCnts/Chnl]	7.735	±0.066	0.9%
τ_2[ns]	0.4455	±0.0055	1.2%
I_2[kCnts]	137.8	±2.9	2.1%
A_3[kCnts/Chnl]	1.481	±0.020	1.3%
τ_3[ns]	7.088	±0.041	0.6%
I_3[kCnts]	419.7	±2.4	0.6%
$Bkgr_{Dec}$[kCnts]	0.0184	±0.0002	0.9%
$Bkgr_{IRF}$[Cnts/Chnl]	-0.092	±0.097	105%
$Shift_{IRF}$[ps]	6.70	±0.83	12%
A_{Scatt}[kCnts]	37.44	±0.97	2.6%
τ_{AvInt}[ns]	4.415	±0.019	0.4%
τ_{AvAmp}[ns]	1.6826	±0.0086	0.5%

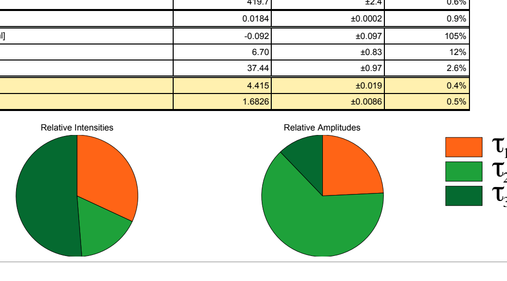

Data Set: 5 / 11

Decay: crv[5]; IRF: crv[0]

Fit

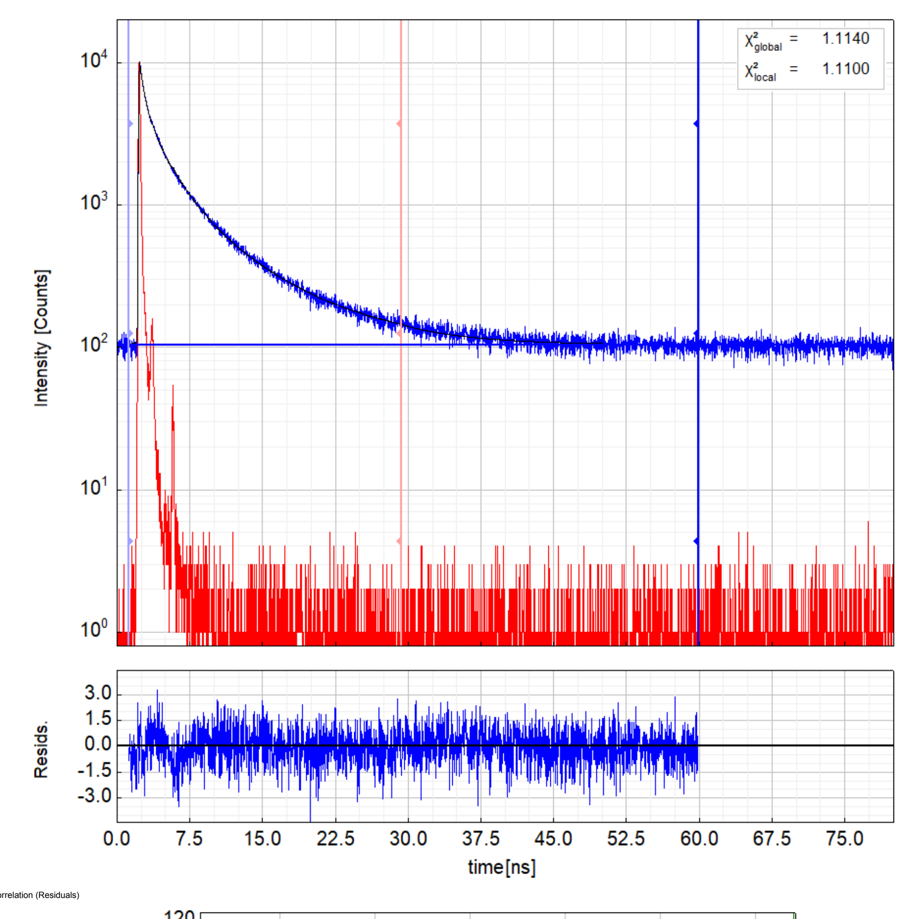

Autocorrelation (Residuals)

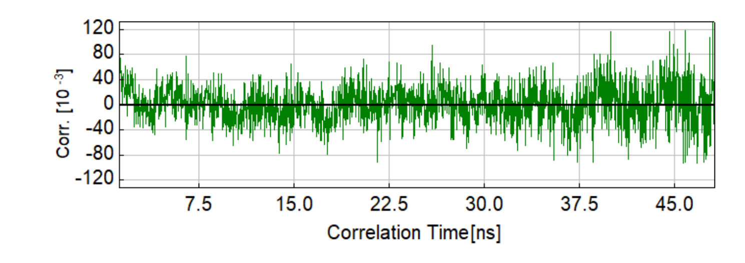

Fitted Parameters

Parameter	Value	Δ	δ
A_1[kCnts/Chnl]	3.533	±0.030	0.8%
τ_1[ns]	2.215	±0.035	1.6%
I_1[kCnts]	313.0	±1.5	0.5%
A_2[kCnts/Chnl]	7.027	±0.069	1.0%
τ_2[ns]	0.4455	±0.0055	1.2%
I_2[kCnts]	125.2	±1.7	1.3%
A_3[kCnts/Chnl]	1.485	±0.023	1.5%
τ_3[ns]	7.088	±0.041	0.6%
I_3[kCnts]	421.0	±4.1	1.0%
$Bkgr_{Dec}$[kCnts]	0.1043	±0.0004	0.3%
$Bkgr_{IRF}$[Cnts/Chnl]	-0.019	±0.057	308%
$Shift_{IRF}$[ps]	8.46	±0.64	7.5%
A_{Scatt}[kCnts]	37.31	±0.47	1.3%
τ_{AvInt}[ns]	4.345	±0.015	0.3%
τ_{AvAmp}[ns]	1.7832	±0.0056	0.3%

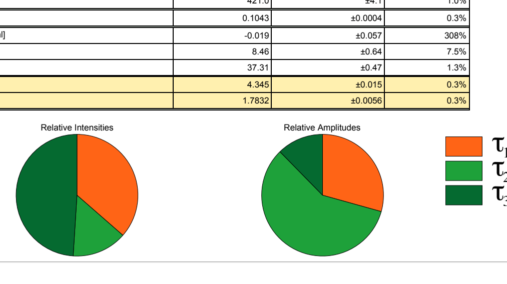

Data Set: 6 / 11

Decay: crv[6]; IRF: crv[0]

Fit

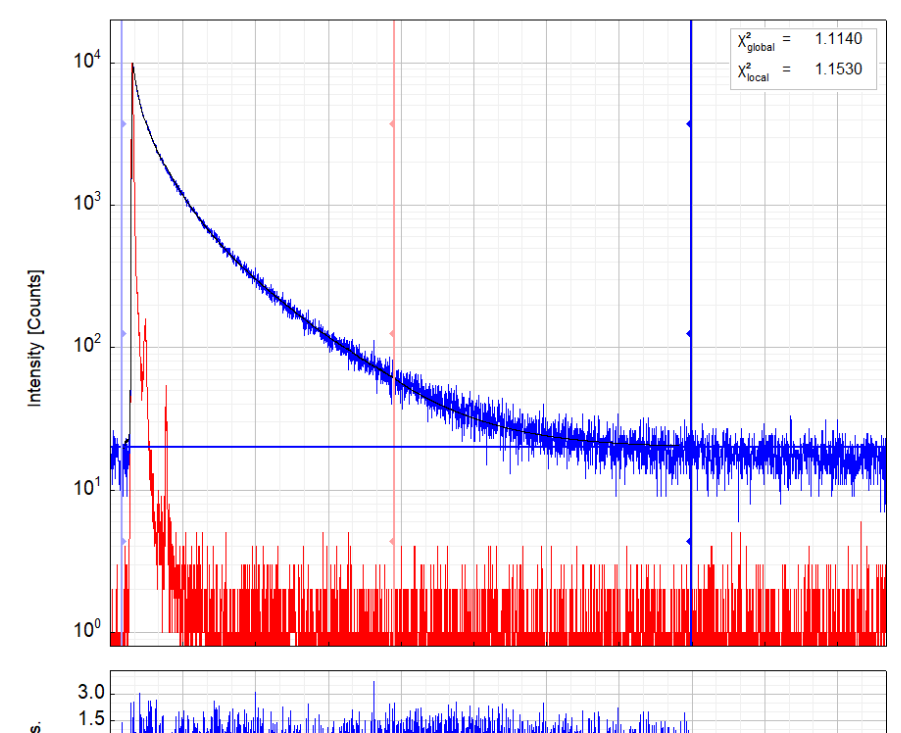

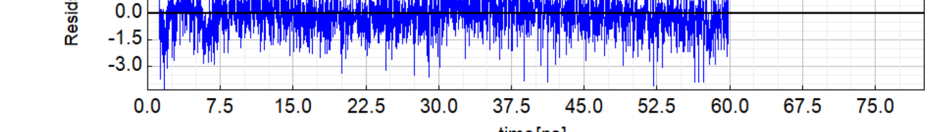

Autocorrelation (Residuals)

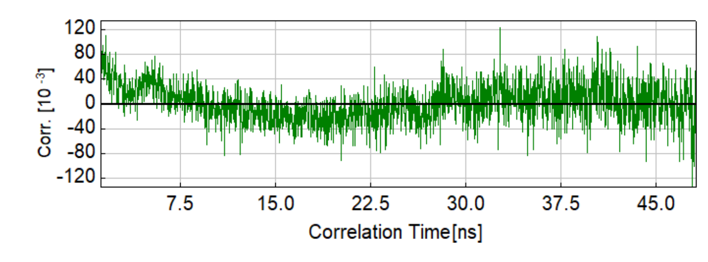

Fitted Parameters

Parameter	Value	Δ	δ
A_1[kCnts/Chnl]	3.948	±0.034	0.9%
$τ_1$[ns]	2.215	±0.035	1.6%
I_1[kCnts]	349.7	±1.5	0.4%
A_2[kCnts/Chnl]	6.414	±0.072	1.1%
$τ_2$[ns]	0.4455	±0.0055	1.2%
I_2[kCnts]	114.3	±2.0	1.7%
A_3[kCnts/Chnl]	1.566	±0.022	1.4%
$τ_3$[ns]	7.088	±0.041	0.6%
I_3[kCnts]	444.0	±3.4	0.8%
$Bkgr_{Dec}$[kCnts]	0.0199	±0.0003	1.1%
$Bkgr_{IRF}$[Cnts/Chnl]	0.187	±0.058	31%
$Shift_{IRF}$[ps]	9.63	±0.57	5.9%
A_{Scatt}[kCnts]	38.00	±0.63	1.6%
$τ_{AvInt}$[ns]	4.375	±0.012	0.3%
$τ_{AvAmp}$[ns]	1.9031	±0.0093	0.5%

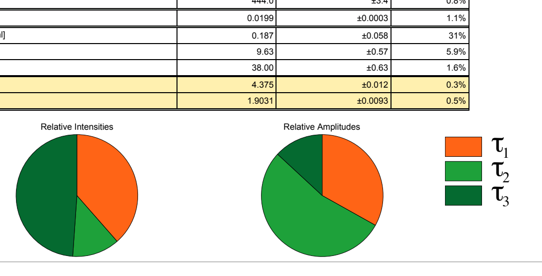

Data Set: 7 / 11

Decay: crv[7]; IRF: crv[0]

Fit

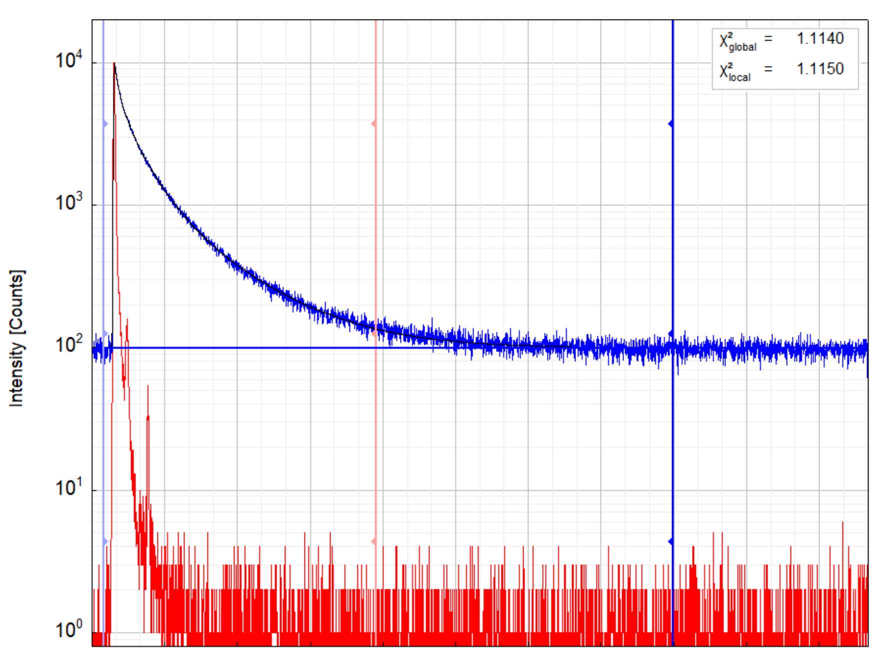

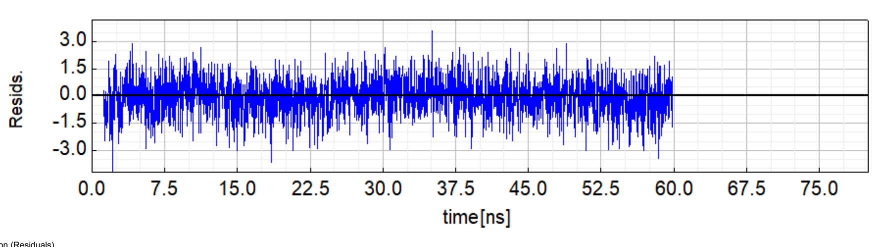

Autocorrelation (Residuals)

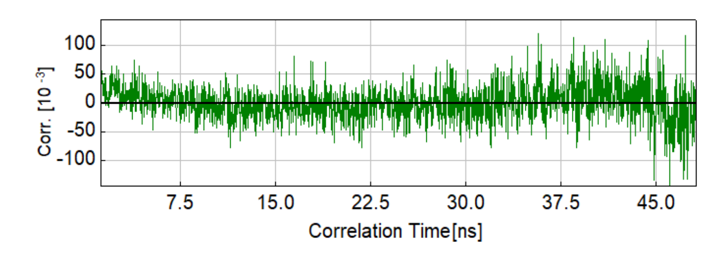

Fitted Parameters

Parameter	Value	Δ	δ
A_1[kCnts/Chnl]	4.138	±0.030	0.7%
$τ_1$[ns]	2.215	±0.035	1.6%
I_1[kCnts]	366.6	±3.0	0.8%
A_2[kCnts/Chnl]	5.908	±0.048	0.8%
$τ_2$[ns]	0.4455	±0.0055	1.2%
I_2[kCnts]	105.3	±1.6	1.5%
A_3[kCnts/Chnl]	1.570	±0.030	1.9%
$τ_3$[ns]	7.088	±0.041	0.6%
I_3[kCnts]	444.9	±5.9	1.3%
$Bkgr_{Dec}$[kCnts]	0.0993	±0.0004	0.4%
$Bkgr_{IRF}$[Cnts/Chnl]	0.694	±0.043	6.2%
$Shift_{IRF}$[ps]	11.81	±0.53	4.5%
A_{Scatt}[kCnts]	38.09	±0.54	1.4%
$τ_{AvInt}$[ns]	4.377	±0.015	0.3%
$τ_{AvAmp}$[ns]	1.974	±0.012	0.6%

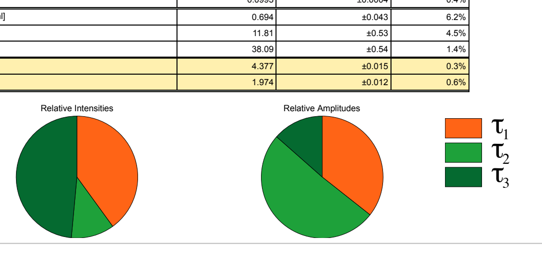

Data Set: 8 / 11

Decay: crv[8]; IRF: crv[0]

Fit

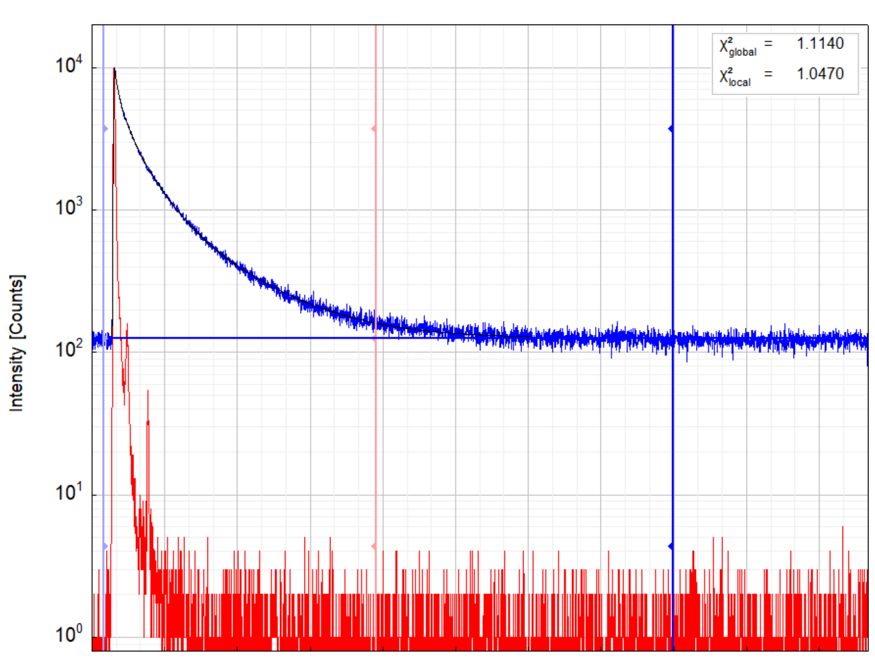

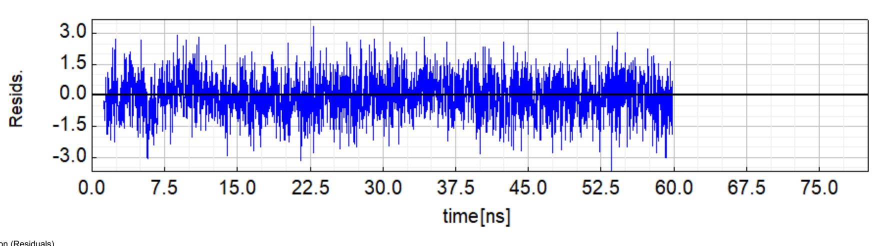

Autocorrelation (Residuals)

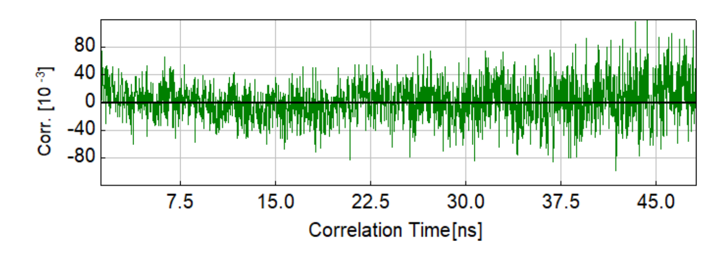

Fitted Parameters

Parameter	Value	Δ	δ
A_1[kCnts/Chnl]	4.124	±0.012	0.3%
$τ_1$[ns]	2.215	±0.035	1.6%
I_1[kCnts]	365.3	±2.0	0.5%
A_2[kCnts/Chnl]	5.786	±0.071	1.2%
$τ_2$[ns]	0.4455	±0.0055	1.2%
I_2[kCnts]	103.1	±1.6	1.5%
A_3[kCnts/Chnl]	1.588	±0.024	1.5%
$τ_3$[ns]	7.088	±0.041	0.6%
I_3[kCnts]	450.2	±6.1	1.4%
$Bkgr_{Dec}$[kCnts]	0.1249	±0.0004	0.3%
$Bkgr_{IRF}$[Cnts/Chnl]	0.80	±0.15	19%
$Shift_{IRF}$[ps]	12.62	±0.46	3.6%
A_{Scatt}[kCnts]	39.27	±0.43	1.1%
$τ_{AvInt}$[ns]	4.405	±0.018	0.4%
$τ_{AvAmp}$[ns]	1.9974	±0.0100	0.5%

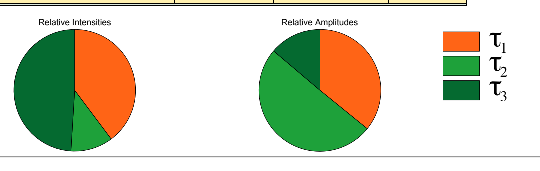

Data Set: 9 / 11

Decay: crv[9]; IRF: crv[0]

Fit

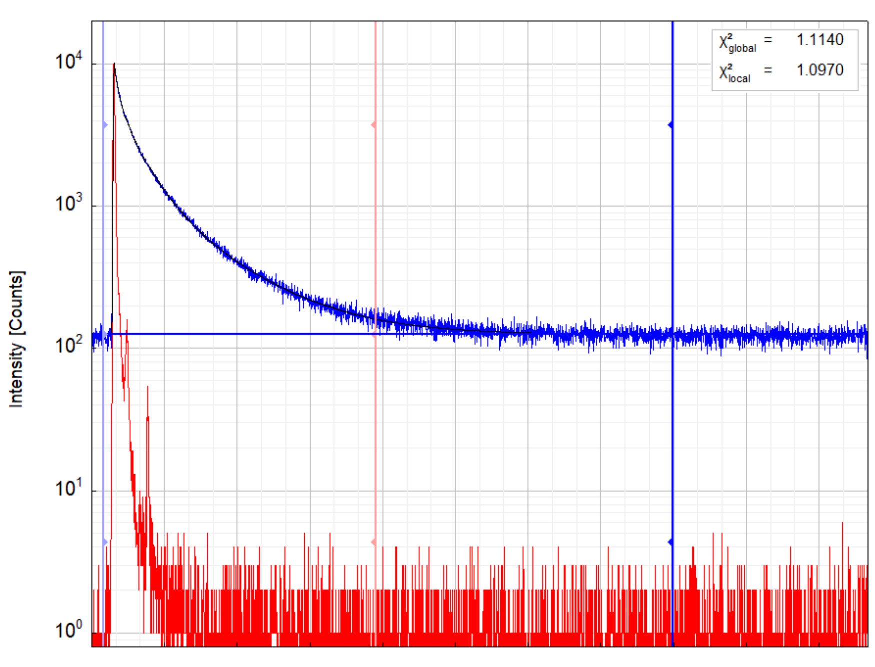

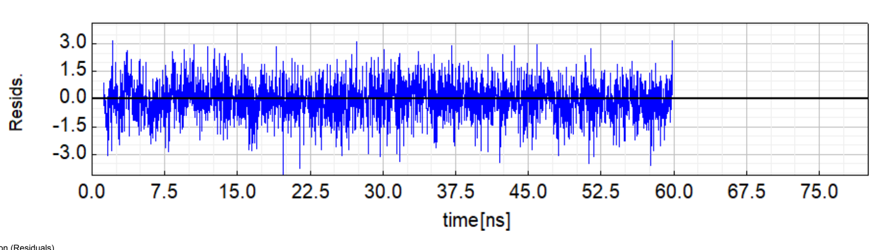

Autocorrelation (Residuals)

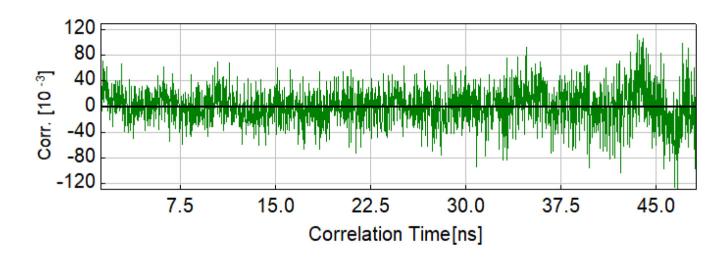

Fitted Parameters

Parameter	Value	Δ	δ
A_1[kCnts/Chnl]	3.955	±0.031	0.8%
$τ_1$[ns]	2.215	±0.035	1.6%
I_1[kCnts]	350.3	±2.9	0.8%
A_2[kCnts/Chnl]	5.691	±0.087	1.5%
$τ_2$[ns]	0.4455	±0.0055	1.2%
I_2[kCnts]	101.4	±2.8	2.7%
A_3[kCnts/Chnl]	1.5952	±0.0094	0.6%
$τ_3$[ns]	7.088	±0.041	0.6%
I_3[kCnts]	452.3	±1.9	0.4%
$Bkgr_{Dec}$[kCnts]	0.1248	±0.0004	0.3%
$Bkgr_{IRF}$[Cnts/Chnl]	0.793	±0.087	11%
$Shift_{IRF}$[ps]	13.67	±0.54	3.9%
A_{Scatt}[kCnts]	41.11	±0.56	1.4%
$τ_{AvInt}$[ns]	4.454	±0.014	0.3%
$τ_{AvAmp}$[ns]	2.0105	±0.0096	0.5%

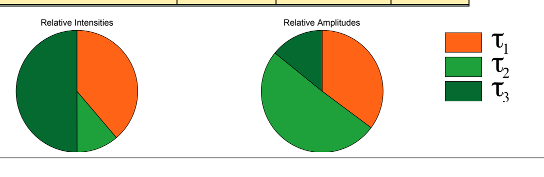

Data Set: 10 / 11

Decay: crv[10]; IRF: crv[0]

Fit

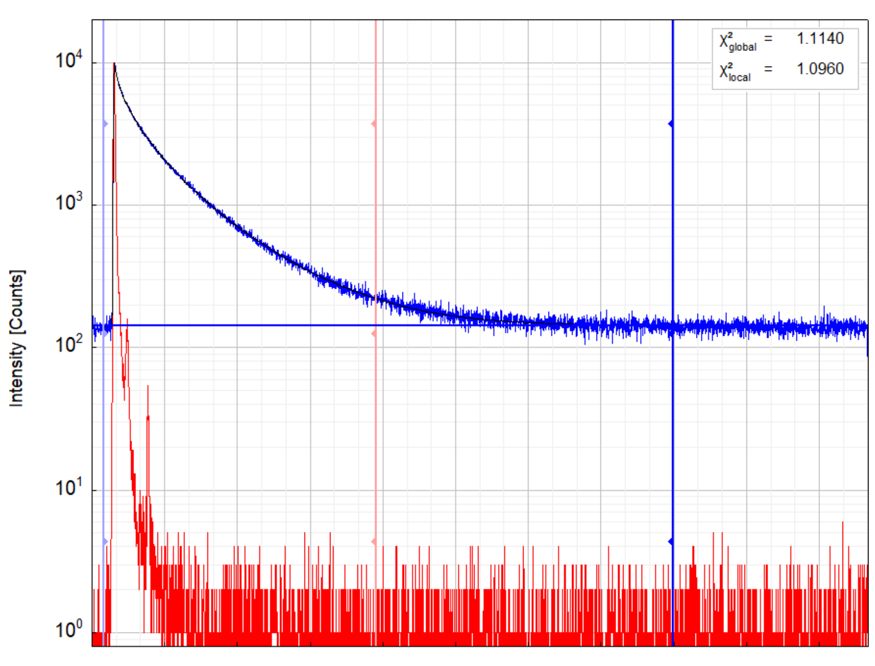

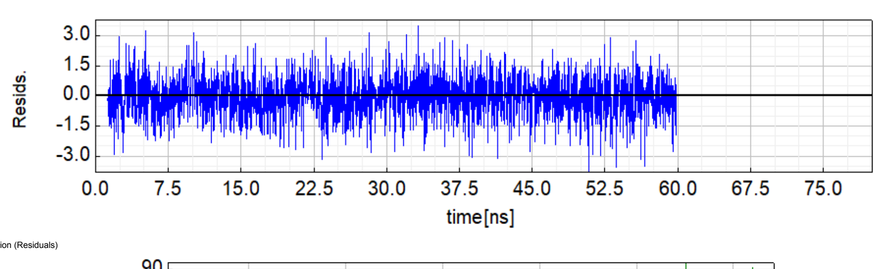

Autocorrelation (Residuals)

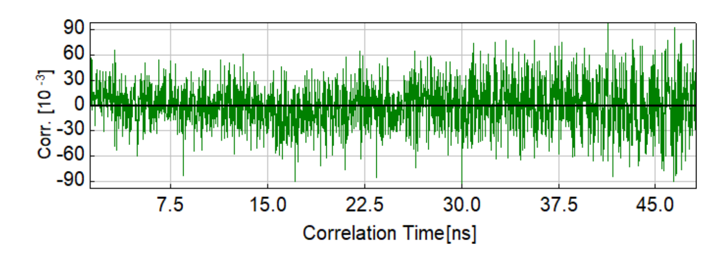

Fitted Parameters

Parameter	Value	Δ	δ
A_1[kCnts/Chnl]	3.577	±0.035	1.0%
$τ_1$[ns]	2.215	±0.035	1.6%
I_1[kCnts]	316.8	±5.3	1.7%
A_2[kCnts/Chnl]	4.211	±0.079	1.9%
$τ_2$[ns]	0.4455	±0.0055	1.2%
I_2[kCnts]	75.02	±0.49	0.6%
A_3[kCnts/Chnl]	3.274	±0.036	1.1%
$τ_3$[ns]	7.088	±0.041	0.6%
I_3[kCnts]	928.0	±5.4	0.6%
$Bkgr_{Dec}$[kCnts]	0.1415	±0.0007	0.5%
$Bkgr_{IRF}$[Cnts/Chnl]	0.34	±0.16	45%
$Shift_{IRF}$[ps]	14.62	±0.60	4.0%
A_{Scatt}[kCnts]	38.79	±0.71	1.8%
$τ_{AvInt}$[ns]	5.541	±0.020	0.4%
$τ_{AvAmp}$[ns]	2.984	±0.019	0.6%

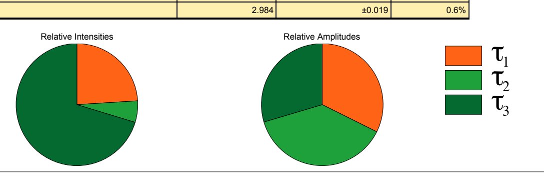

Data Set: 11 / 11

Decay: crv[11]; IRF: crv[0]

Fit

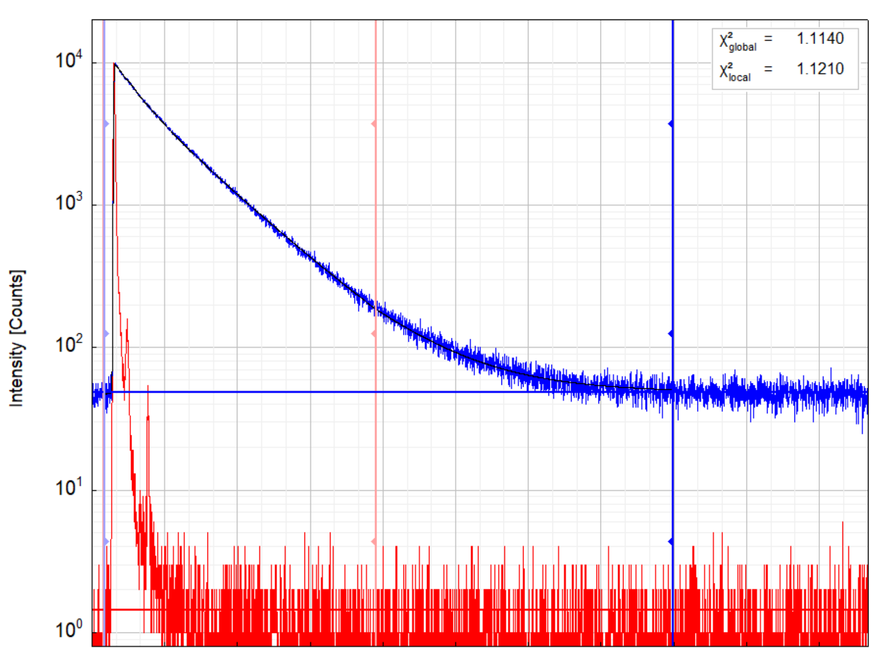

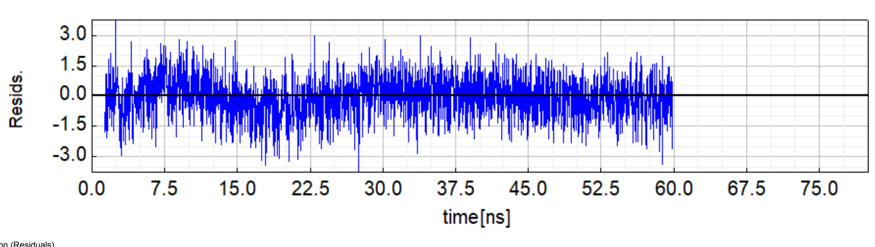

Autocorrelation (Residuals)

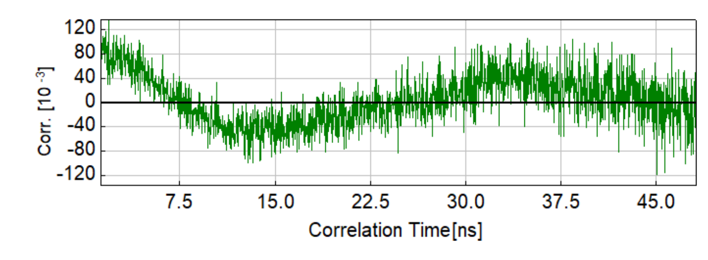

Fitted Parameters

Parameter	Value	Δ	δ
A_1[kCnts/Chnl]	3.60	±0.12	3.3%
$τ_1$[ns]	2.215	±0.035	1.6%
I_1[kCnts]	319	±11	3.2%
A_2[kCnts/Chnl]	0.912	±0.056	6.1%
$τ_2$[ns]	0.4455	±0.0055	1.2%
I_2[kCnts]	16.25	±0.96	5.9%
A_3[kCnts/Chnl]	6.825	±0.066	1.0%
$τ_3$[ns]	7.088	±0.041	0.6%
I_3[kCnts]	1 934.7	±8.8	0.5%
$Bkgr_{Dec}$[kCnts]	0.0489	±0.0003	0.4%
$Bkgr_{IRF}$[Cnts/Chnl]	1.440	±0.062	4.3%
$Shift_{IRF}$[ps]	17.98	±0.40	2.2%
A_{Scatt}[kCnts]	30.55	±0.28	0.9%
$τ_{AvInt}$[ns]	6.356	±0.016	0.3%
$τ_{AvAmp}$[ns]	5.007	±0.026	0.5%

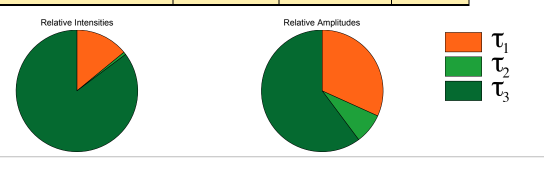

Parameter Plot

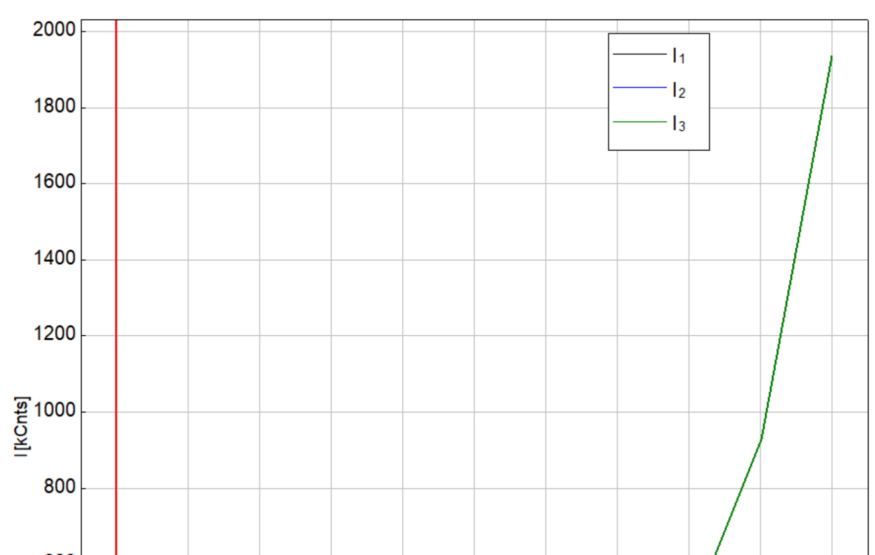

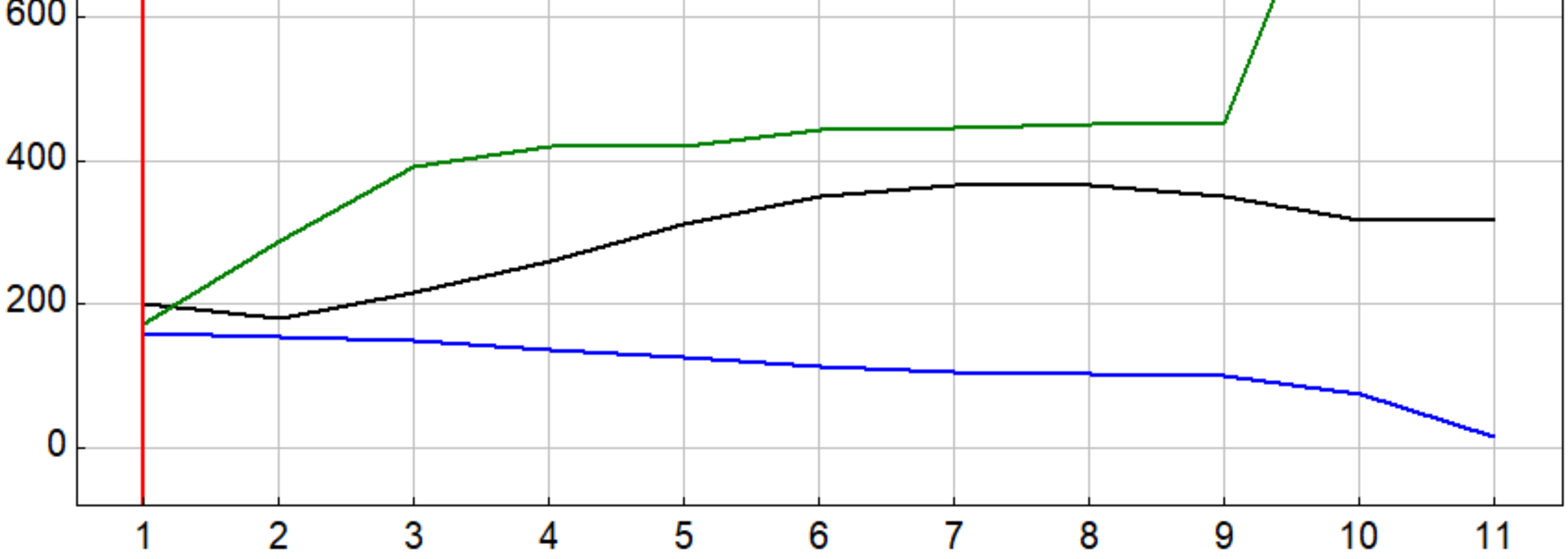

Global Parameters

Parameter	Value	Δ	δ
τ_1[ns]	2.215	±0.035	1.6%
τ_2[ns]	0.4455	±0.0055	1.2%
τ_3[ns]	7.088	±0.041	0.6%

EasyTau Report

3/11/2022 7:15:42 PM
F:\Paper Xiongfei\CD-1 DMF tau global.html

Container Curves

TRES+IRF_20201222_1558.etc: crv[0 - 31]

Container Properties

Measurement Context: Time Resolved Emission Scan
Detection Wavelength: 400.0 - 700.0 (Step: 10.0) nm
Summary:

Sample:	CD_X_1
Solvent:	Dimethyl formamide
Excitation:	V pol 376±1nm with LDH-P-C-375
Detection:	M pol 400...700nm 10nm steps<br>10000 peak counts<br>grating 1200/500-<br>detector UV-red [PMT]

Comment

Model: Exponential [Reconv.]

$$\mathrm{Dec}(t)=\left[\int_{-\infty}^{t} dt'\,[\mathrm{IRF}(t-Shift_{IRF})-Bkgr_{IRF}]\left[\sum_{i=1}^{n_{Exp}} A_i\, e^{-\frac{t-t'}{\tau_i}}+A_{Scatt}\,\delta(t-t')\right]\right]+Bkgr_{Dec}$$

$$I_m = A_m \tau_m$$

$$A_{Rel\,m}=A_m \Big/ \sum_{\substack{i=1\\A_i>0}}^{n_{Exp}} A_i \qquad I_{Rel\,m}=I_m \Big/ \sum_{\substack{i=1\\I_i>0}}^{n_{Exp}} I_i$$

$$\tau_{Av\,Amp}=\sum_{\substack{i=1\\A_i>0}}^{n_{Exp}} A_i\tau_i \Big/ \sum_{\substack{i=1\\A_i>0}}^{n_{Exp}} A_i \qquad \tau_{Av\,Int}=\sum_{\substack{i=1\\I_i>0}}^{n_{Exp}} I_i\tau_i \Big/ \sum_{\substack{i=1\\I_i>0}}^{n_{Exp}} I_i$$

Data Set: 1 / 31

Decay: crv[1]; IRF: crv[0]

Fit

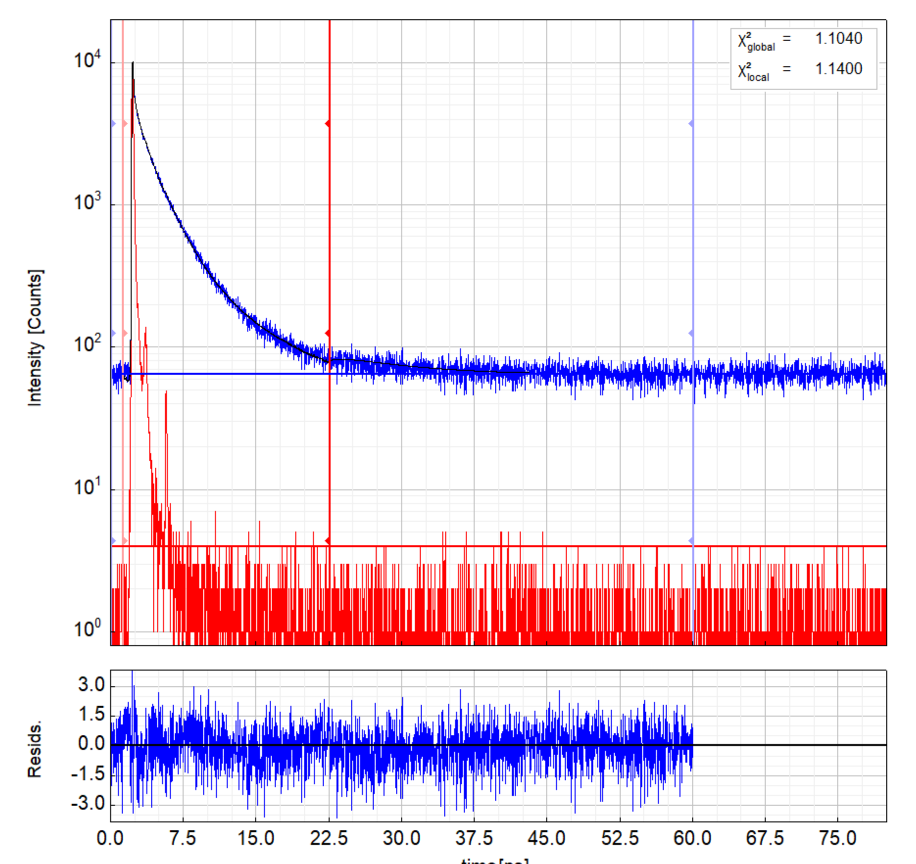

Autocorrelation (Residuals)

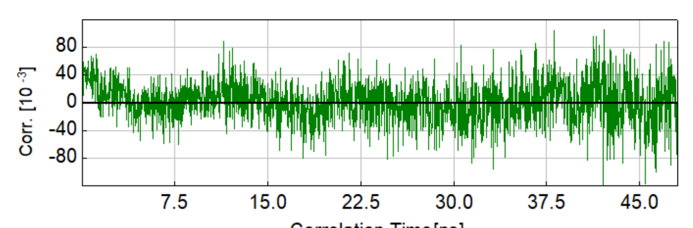

Fitted Parameters

Parameter	Value	Δ	δ
A_1[kCnts/Chnl]	0.4647	±0.0097	2.1%
τ_1[ns]	7.675	±0.012	0.2%
I_1[kCnts]	142.7	±3.0	2.1%
A_2[kCnts/Chnl]	3.716	±0.025	0.7%
τ_2[ns]	2.250	±0.013	0.6%
I_2[kCnts]	334.3	±2.1	0.6%
A_3[kCnts/Chnl]	2.876	±0.095	3.3%
τ_3[ns]	0.5216	±0.0058	1.1%
I_3[kCnts]	60.0	±2.7	4.4%
$Bkgr_{Dec}$[kCnts]	0.0641	±0.0004	0.5%
$Bkgr_{IRF}$[Cnts/Chnl]	3.95	±0.16	3.9%
$Shift_{IRF}$[ps]	-2.82	±0.76	27%
A_{Scatt}[kCnts]	60.9	±1.7	2.8%
τ_{AvInt}[ns]	3.498	±0.021	0.6%
τ_{AvAmp}[ns]	1.903	±0.021	1.1%
λ_{Det}[nm]	400.0000	---	---

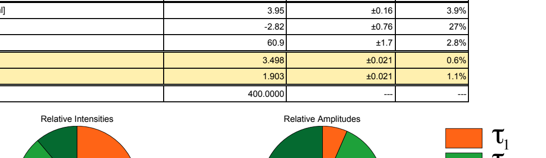

Data Set: 2 / 31

Decay: crv[2]; IRF: crv[0]

Fit

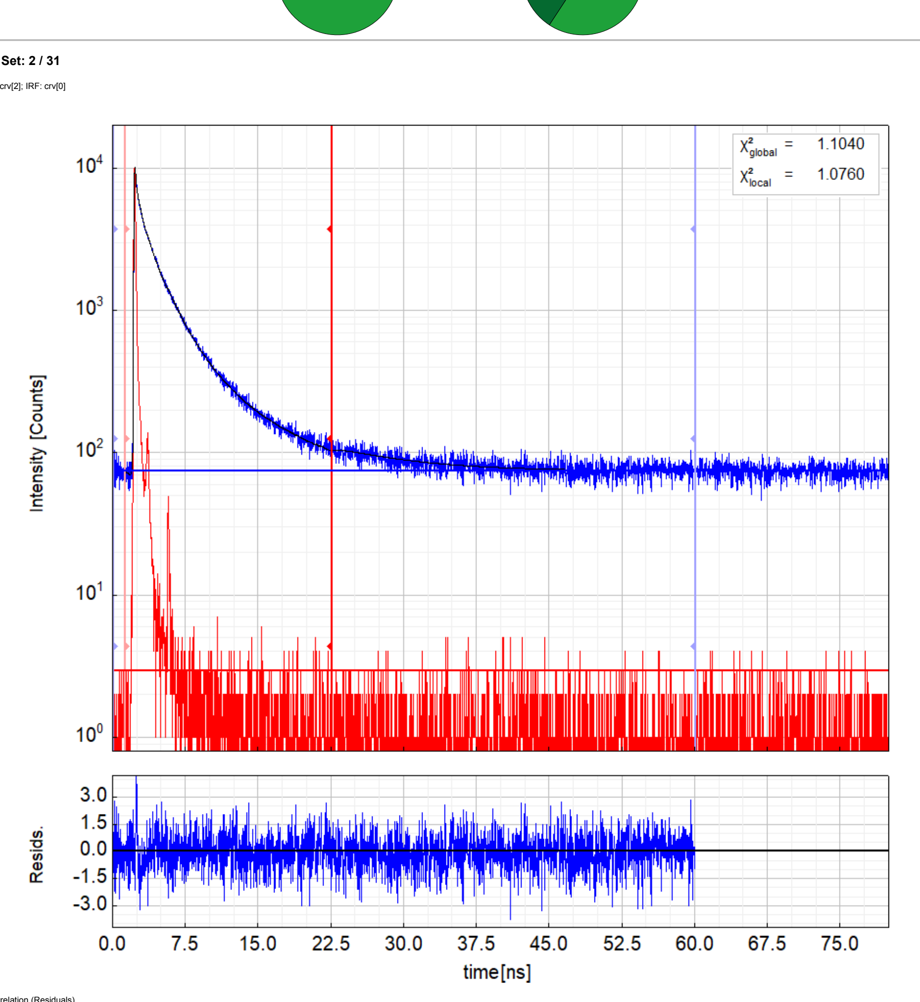

Autocorrelation (Residuals)

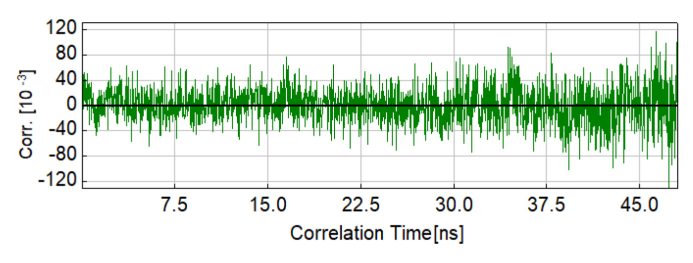

Fitted Parameters

Parameter	Value	Δ	δ
A_1[kCnts/Chnl]	0.6079	±0.0091	1.5%
τ_1[ns]	7.675	±0.012	0.2%
I_1[kCnts]	186.6	±2.7	1.4%
A_2[kCnts/Chnl]	4.154	±0.032	0.7%
τ_2[ns]	2.250	±0.013	0.6%
I_2[kCnts]	373.7	±1.5	0.4%
A_3[kCnts/Chnl]	4.95	±0.13	2.5%
τ_3[ns]	0.5216	±0.0058	1.1%
I_3[kCnts]	103.3	±2.1	2.0%
$Bkgr_{Dec}$[kCnts]	0.0745	±0.0003	0.3%
$Bkgr_{IRF}$[Cnts/Chnl]	2.95	±0.13	4.2%
$Shift_{IRF}$[ps]	11.3	±1.3	11%
A_{Scatt}[kCnts]	50.3	±1.4	2.6%
τ_{AvInt}[ns]	3.507	±0.015	0.4%
τ_{AvAmp}[ns]	1.709	±0.017	1.0%
λ_{Det}[nm]	410.0000	---	---

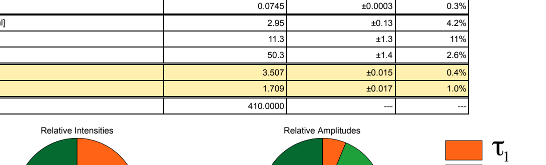

Data Set: 3 / 31

Decay: crv[3]; IRF: crv[0]

Fit

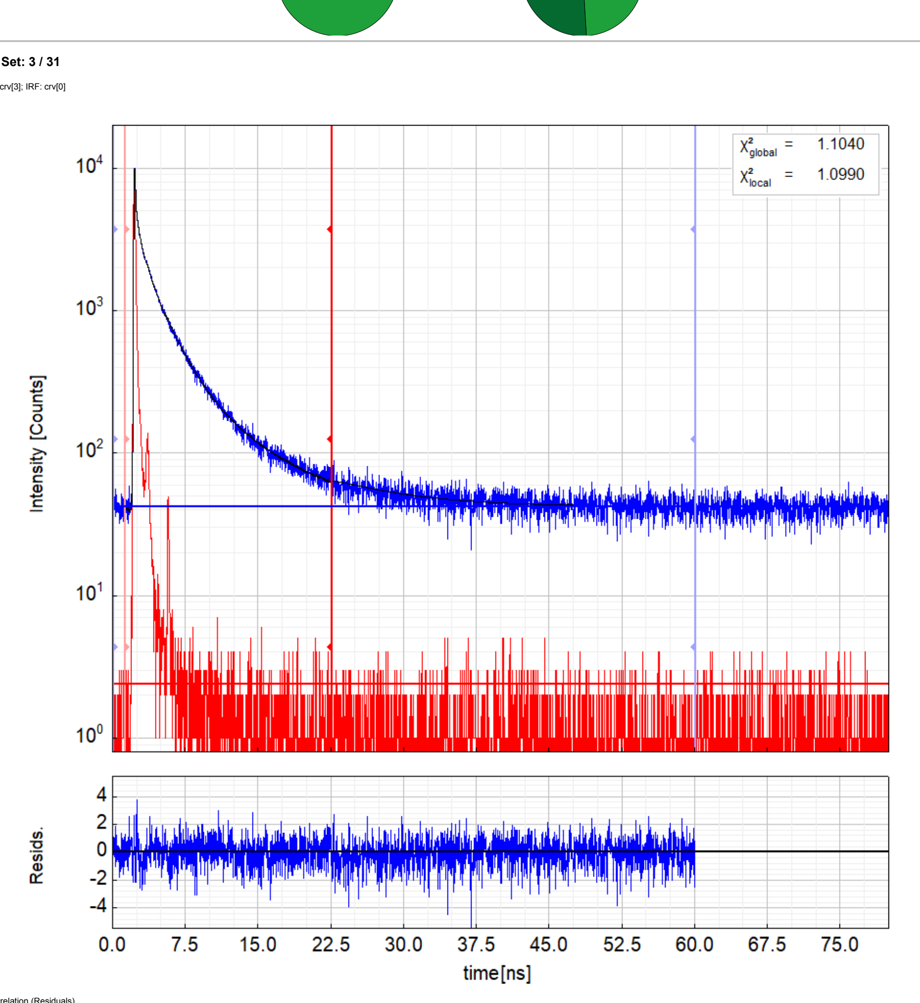

Autocorrelation (Residuals)

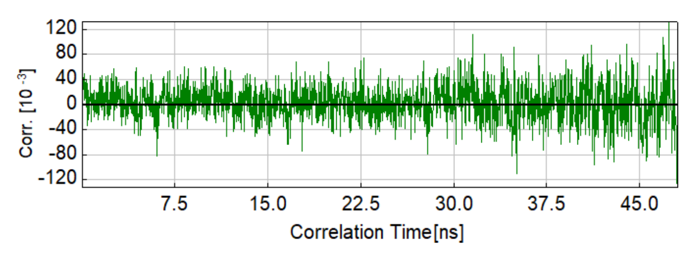

Fitted Parameters

Parameter	Value	Δ	δ
A_1[kCnts/Chnl]	0.3806	±0.0063	1.6%
τ_1[ns]	7.675	±0.012	0.2%
I_1[kCnts]	116.9	±1.8	1.5%
A_2[kCnts/Chnl]	2.519	±0.022	0.8%
τ_2[ns]	2.250	±0.013	0.6%
I_2[kCnts]	226.6	±1.7	0.7%
A_3[kCnts/Chnl]	3.115	±0.084	2.7%
τ_3[ns]	0.5216	±0.0058	1.1%
I_3[kCnts]	65.0	±2.1	3.2%
$Bkgr_{Dec}$[kCnts]	0.0421	±0.0002	0.4%
$Bkgr_{IRF}$[Cnts/Chnl]	2.39	±0.18	7.1%
$Shift_{IRF}$[ps]	-4.29	±0.63	15%
A_{Scatt}[kCnts]	65.06	±0.87	1.3%
τ_{AvInt}[ns]	3.527	±0.024	0.7%
τ_{AvAmp}[ns]	1.698	±0.014	0.8%
λ_{Det}[nm]	420.0000	---	---

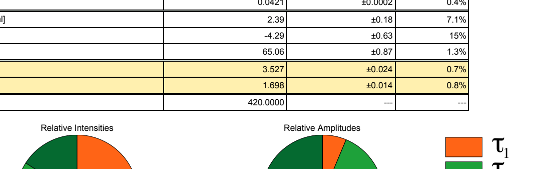

Data Set: 4 / 31

Decay: crv[4]; IRF: crv[0]

Fit

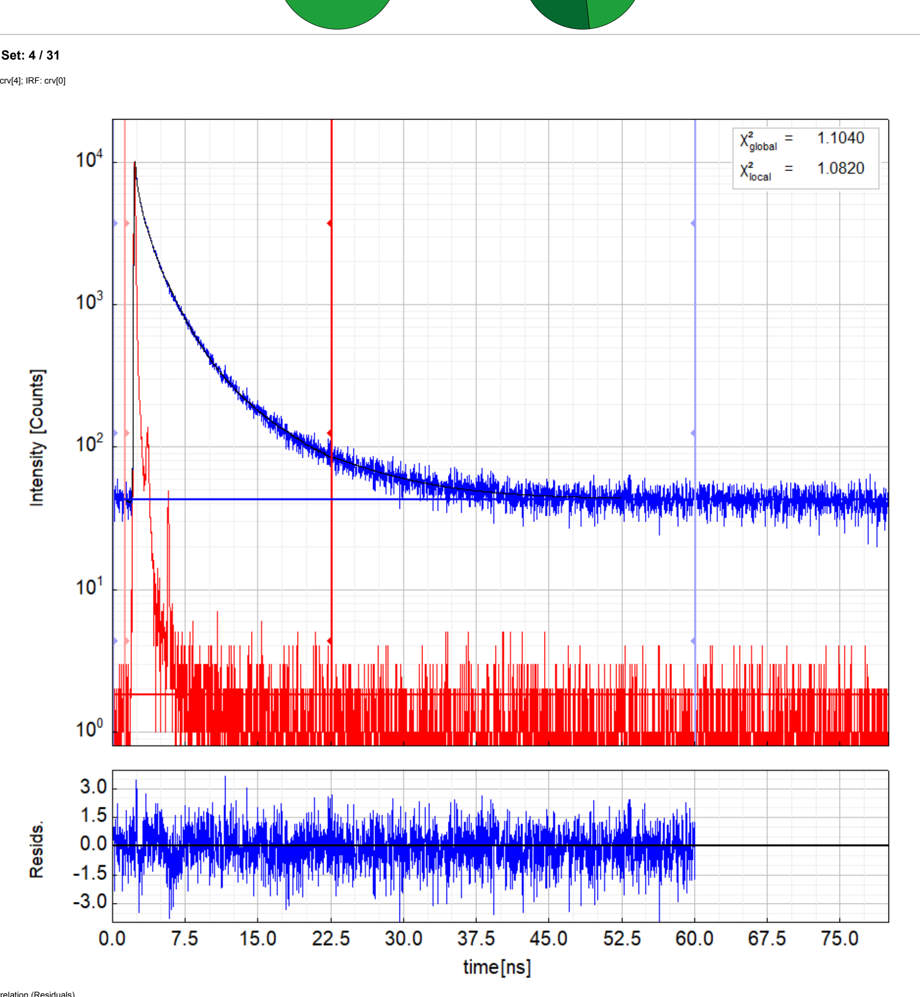

Autocorrelation (Residuals)

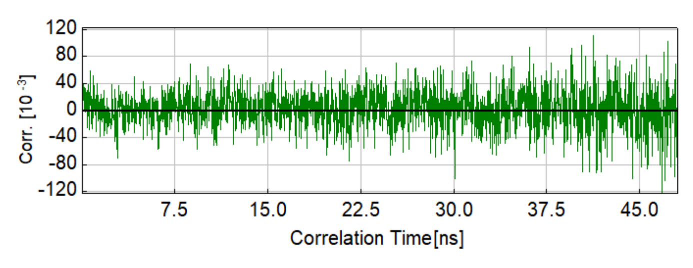

Fitted Parameters

Parameter	Value	Δ	δ
A_1[kCnts/Chnl]	0.6642	±0.0080	1.2%
τ_1[ns]	7.675	±0.012	0.2%
I_1[kCnts]	203.9	±2.6	1.2%
A_2[kCnts/Chnl]	4.066	±0.038	0.9%
τ_2[ns]	2.250	±0.013	0.6%
I_2[kCnts]	365.9	±1.2	0.3%
A_3[kCnts/Chnl]	5.340	±0.060	1.1%
τ_3[ns]	0.5216	±0.0058	1.1%
I_3[kCnts]	111.4	±2.0	1.7%
$Bkgr_{Dec}$[kCnts]	0.0429	±0.0002	0.4%
$Bkgr_{IRF}$[Cnts/Chnl]	1.83	±0.17	9.2%
$Shift_{IRF}$[ps]	10.72	±0.95	8.8%
A_{Scatt}[kCnts]	49.0	±1.1	2.2%
τ_{AvInt}[ns]	3.591	±0.018	0.5%
τ_{AvAmp}[ns]	1.692	±0.014	0.8%
λ_{Det}[nm]	430.0000	---	---

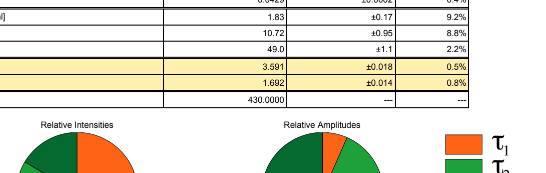

Data Set: 5 / 31

Decay: crv[5]; IRF: crv[0]

Fit

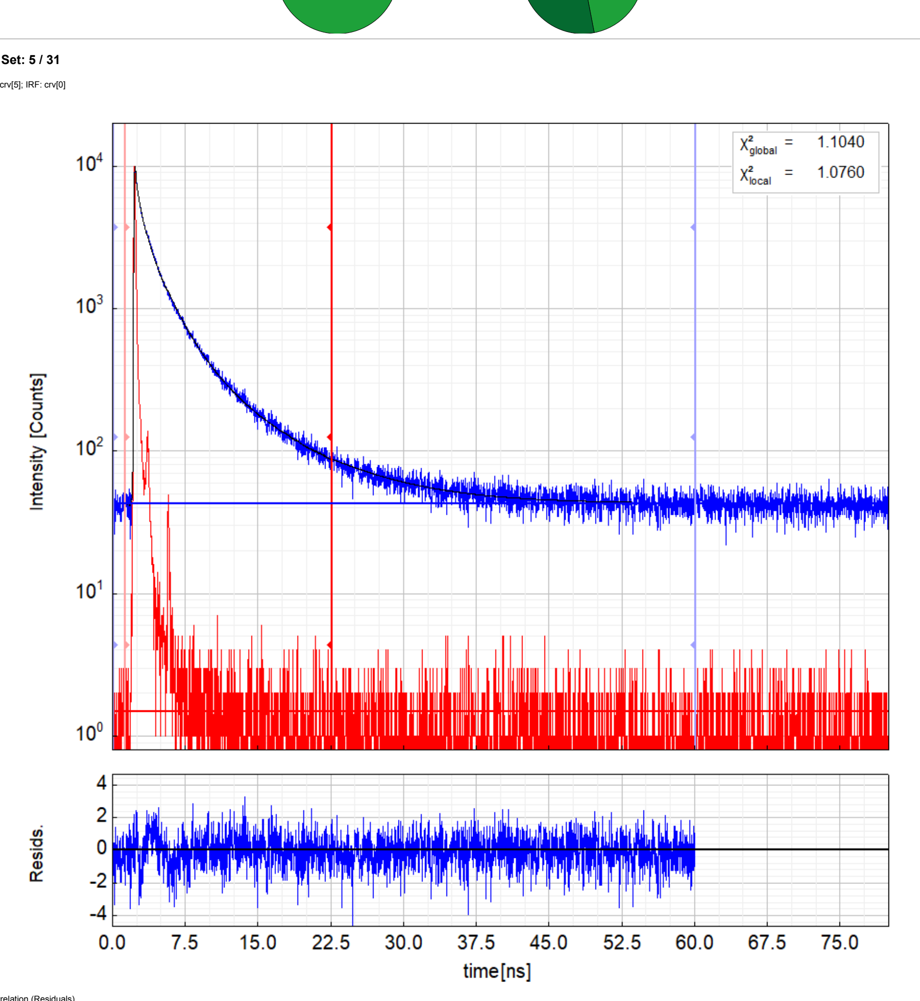

Autocorrelation (Residuals)

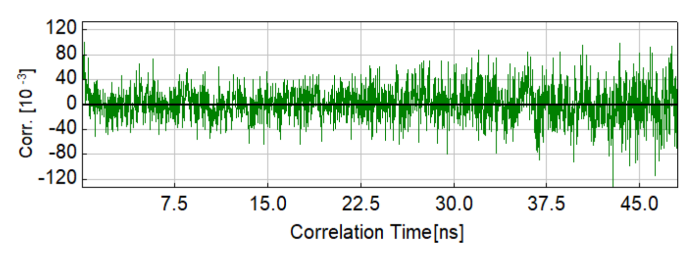

Fitted Parameters

Parameter	Value	Δ	δ
A_1[kCnts/Chnl]	0.6630	±0.0046	0.7%
τ_1[ns]	7.675	±0.012	0.2%
I_1[kCnts]	203.6	±1.6	0.7%
A_2[kCnts/Chnl]	3.702	±0.035	0.9%
τ_2[ns]	2.250	±0.013	0.6%
I_2[kCnts]	333.0	±2.3	0.7%
A_3[kCnts/Chnl]	5.916	±0.065	1.1%
τ_3[ns]	0.5216	±0.0058	1.1%
I_3[kCnts]	123.5	±1.8	1.4%
$Bkgr_{Dec}$[kCnts]	0.0430	±0.0003	0.6%
$Bkgr_{IRF}$[Cnts/Chnl]	1.481	±0.084	5.7%
$Shift_{IRF}$[ps]	11.4	±1.1	8.8%
A_{Scatt}[kCnts]	46.19	±0.75	1.6%
τ_{AvInt}[ns]	3.600	±0.013	0.4%
τ_{AvAmp}[ns]	1.6051	±0.0086	0.5%
λ_{Det}[nm]	440.0000	---	---

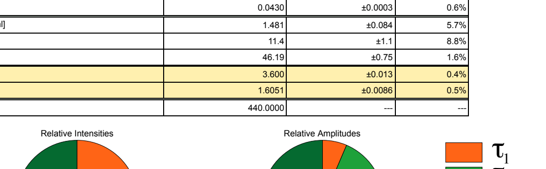

Data Set: 6 / 31

Decay: crv[6]; IRF: crv[0]

Fit

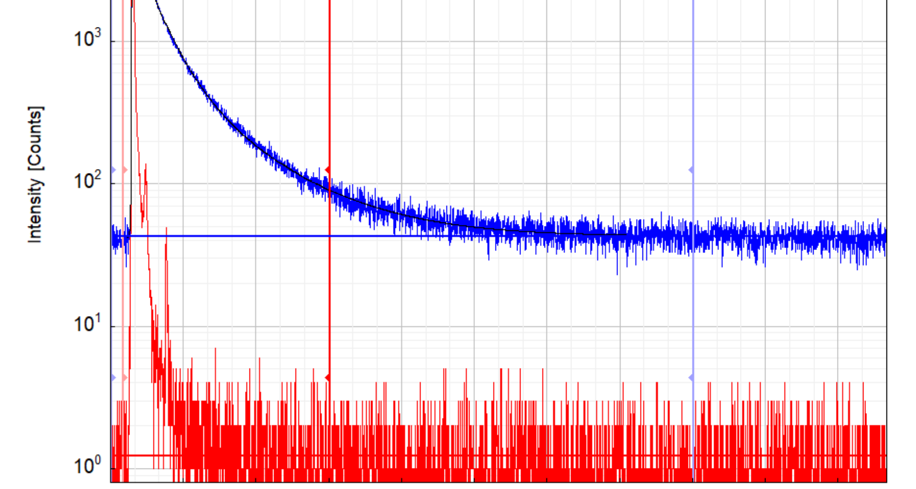

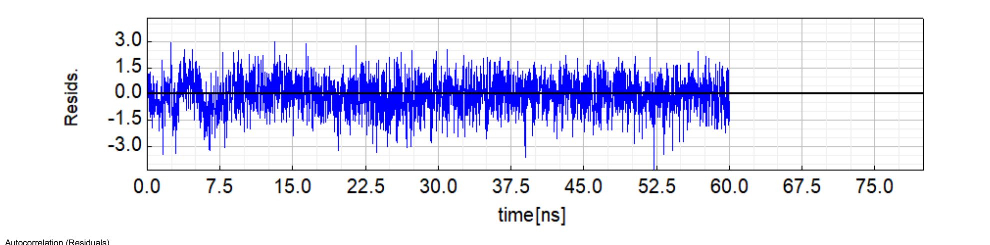

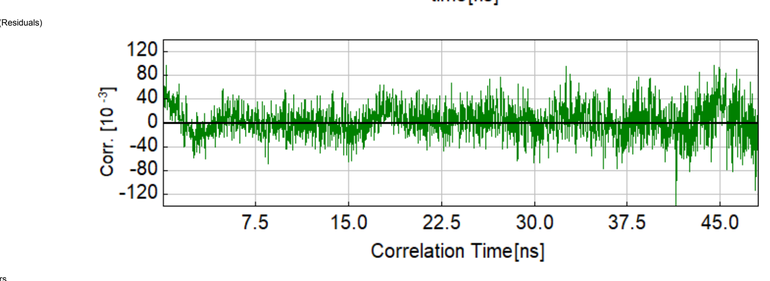

Parameter	Value	Δ	δ
A_1[kCnts/Chnl]	0.6730	±0.0092	1.4%
τ_1[ns]	7.675	±0.012	0.2%
I_1[kCnts]	206.7	±3.1	1.5%
A_2[kCnts/Chnl]	3.672	±0.042	1.1%
τ_2[ns]	2.250	±0.013	0.6%
I_2[kCnts]	330.3	±2.3	0.7%
A_3[kCnts/Chnl]	6.647	±0.054	0.8%
τ_3[ns]	0.5216	±0.0058	1.1%
I_3[kCnts]	138.7	±1.8	1.3%
$Bkgr_{Dec}$[kCnts]	0.0429	±0.0002	0.4%
$Bkgr_{IRF}$[Cnts/Chnl]	1.24	±0.15	12%
$Shift_{IRF}$[ps]	11.42	±0.41	3.6%
A_{Scatt}[kCnts]	43.50	±0.45	1.0%
τ_{AvInt}[ns]	3.554	±0.022	0.6%
τ_{AvAmp}[ns]	1.5367	±0.0053	0.3%
λ_{Det}[nm]	450.0000	---	---

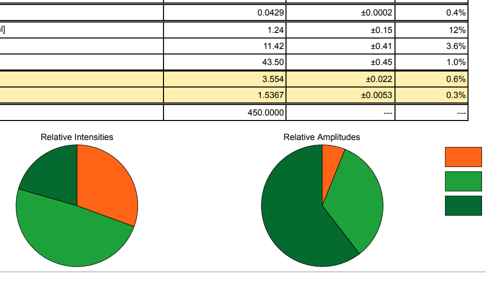

Data Set: 7 / 31

Decay: crv[7]; IRF: crv[0]

Fit

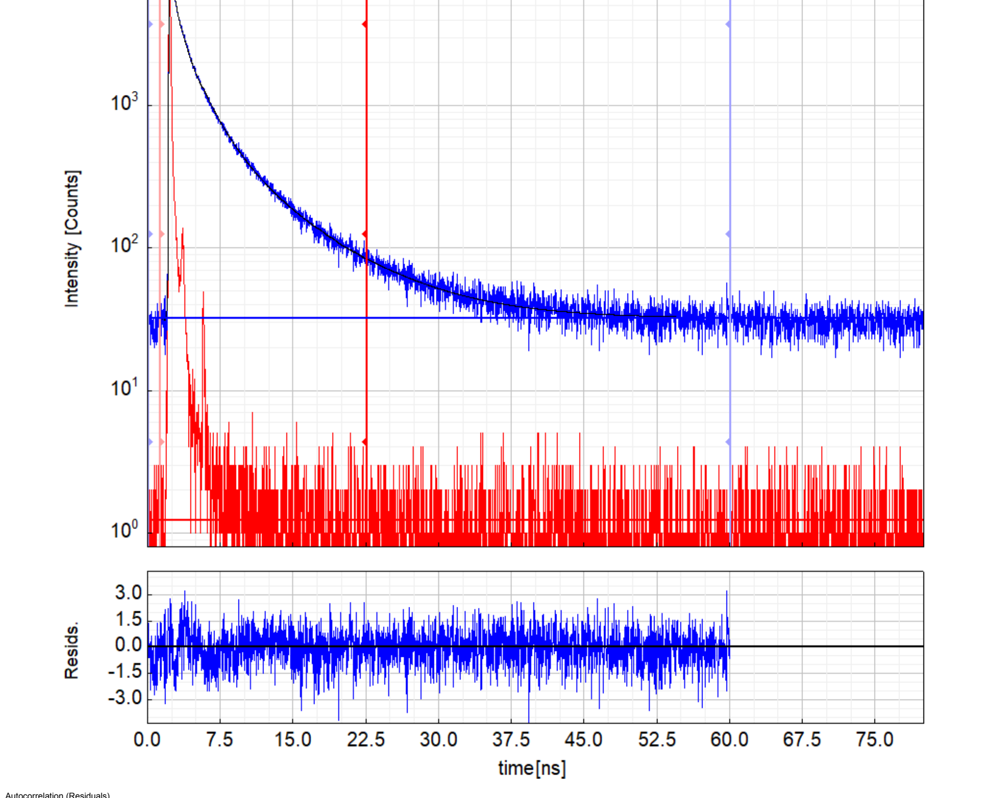

Autocorrelation (Residuals)

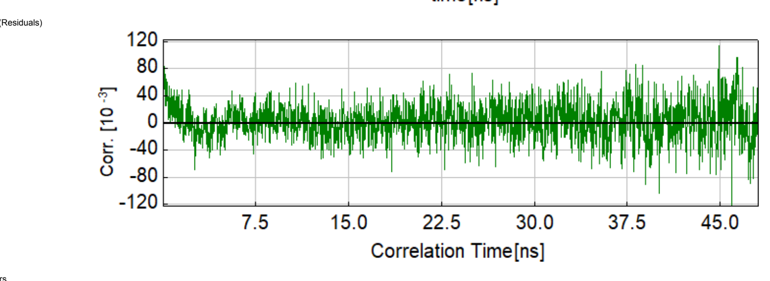

Fitted Parameters

Parameter	Value	Δ	δ
A_1[kCnts/Chnl]	0.7366	±0.0082	1.1%
τ_1[ns]	7.675	±0.012	0.2%
I_1[kCnts]	226.2	±2.5	1.1%
A_2[kCnts/Chnl]	3.522	±0.019	0.5%
τ_2[ns]	2.250	±0.013	0.6%
I_2[kCnts]	316.9	±2.1	0.6%
A_3[kCnts/Chnl]	7.014	±0.069	1.0%
τ_3[ns]	0.5216	±0.0058	1.1%
I_3[kCnts]	146.4	±2.6	1.8%
$Bkgr_{Dec}$[kCnts]	0.0321	±0.0002	0.4%
$Bkgr_{IRF}$[Cnts/Chnl]	1.24	±0.14	11%
$Shift_{IRF}$[ps]	11.17	±0.58	5.2%
A_{Scatt}[kCnts]	40.85	±0.89	2.2%
τ_{AvInt}[ns]	3.663	±0.016	0.4%
τ_{AvAmp}[ns]	1.5289	±0.0069	0.4%
λ_{Det}[nm]	460.0000	---	---

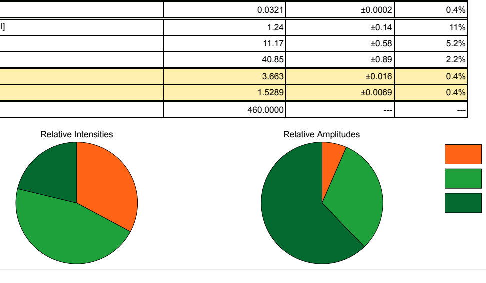

Data Set: 8 / 31

Decay: crv[8]; IRF: crv[0]

Fit

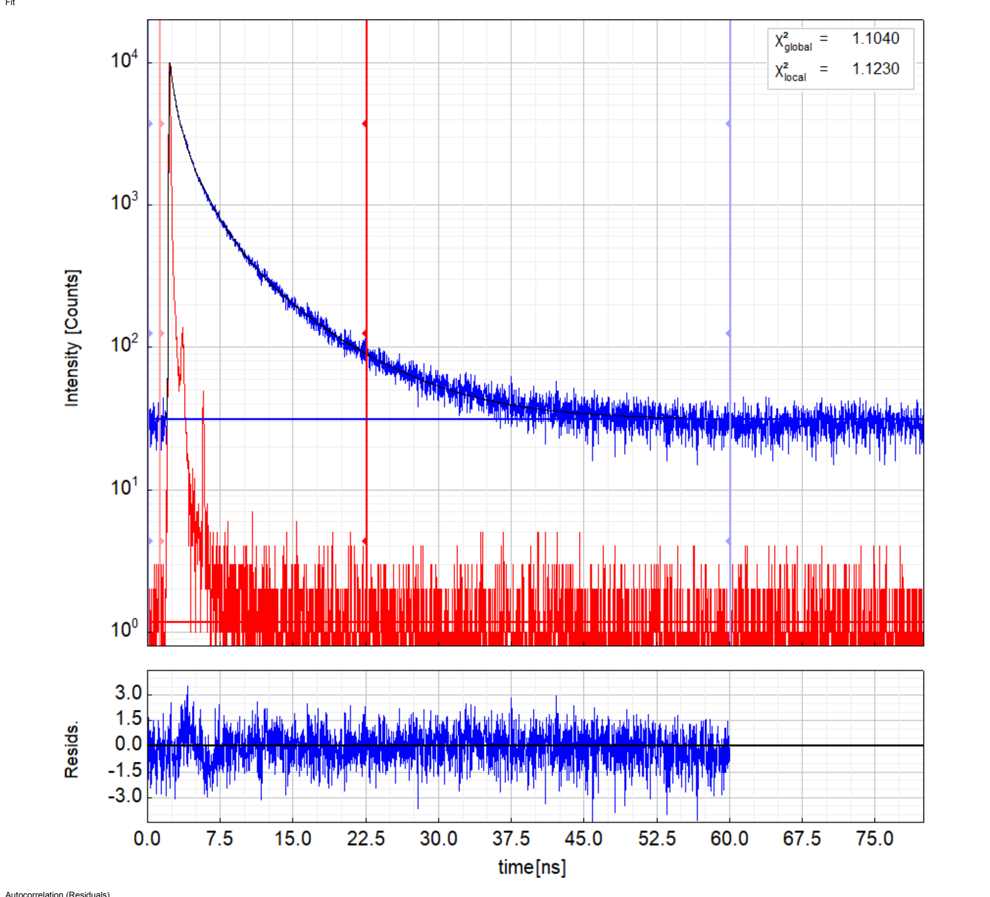

Autocorrelation (Residuals)

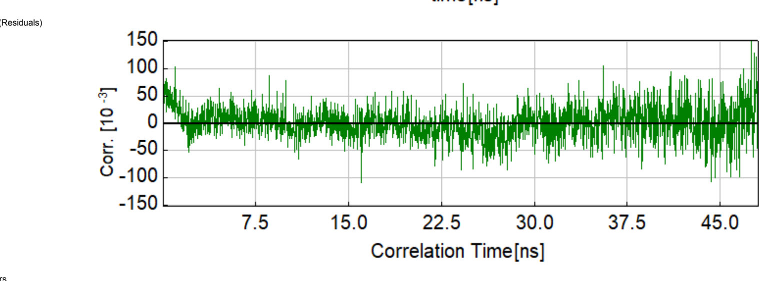

Fitted Parameters

Parameter	Value	Δ	δ
A_1[kCnts/Chnl]	0.8249	±0.0079	0.9%
τ_1[ns]	7.675	±0.012	0.2%
I_1[kCnts]	253.3	±2.1	0.8%
A_2[kCnts/Chnl]	3.395	±0.025	0.7%
τ_2[ns]	2.250	±0.013	0.6%
I_2[kCnts]	305.4	±2.0	0.7%
A_3[kCnts/Chnl]	7.174	±0.085	1.2%
τ_3[ns]	0.5216	±0.0058	1.1%
I_3[kCnts]	149.7	±1.9	1.2%
$Bkgr_{Dec}$[kCnts]	0.0311	±0.0003	0.7%
$Bkgr_{IRF}$[Cnts/Chnl]	1.178	±0.063	5.3%
$Shift_{IRF}$[ps]	10.03	±0.87	8.6%
A_{Scatt}[kCnts]	39.1	±1.4	3.5%
τ_{AvInt}[ns]	3.825	±0.013	0.3%
τ_{AvAmp}[ns]	1.555	±0.013	0.8%
λ_{Det}[nm]	470.0000	---	---

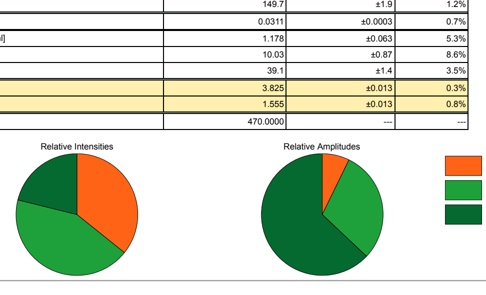

Data Set: 9 / 31

Decay: crv[9]; IRF: crv[0]

Fit

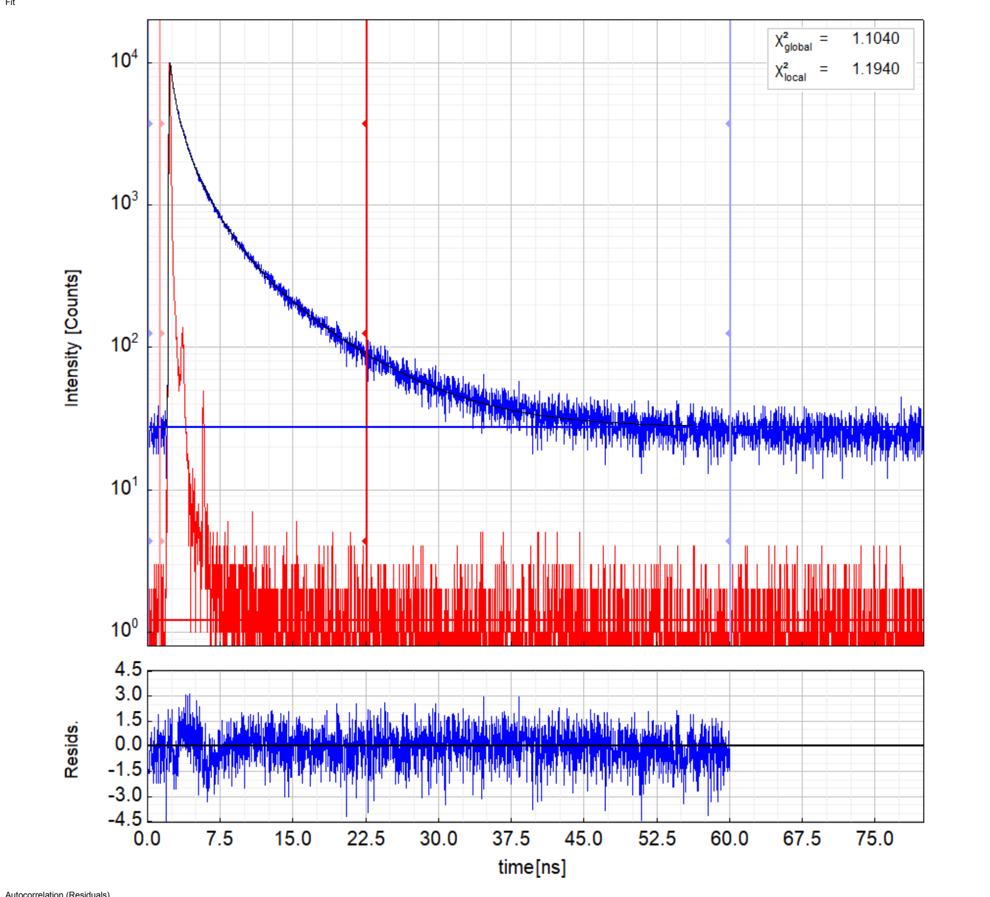

Autocorrelation (Residuals)

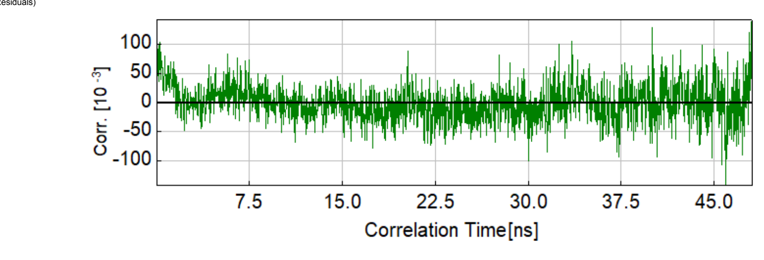

Fitted Parameters

Parameter	Value	Δ	δ
A_1[kCnts/Chnl]	0.881	±0.011	1.2%
τ_1[ns]	7.675	±0.012	0.2%
I_1[kCnts]	270.2	±3.0	1.1%
A_2[kCnts/Chnl]	3.496	±0.026	0.7%
τ_2[ns]	2.250	±0.013	0.6%
I_2[kCnts]	314.6	±1.8	0.6%
A_3[kCnts/Chnl]	7.184	±0.071	1.0%
τ_3[ns]	0.5216	±0.0058	1.1%
I_3[kCnts]	149.9	±1.7	1.1%
$Bkgr_{Dec}$[kCnts]	0.0273	±0.0002	0.4%
$Bkgr_{IRF}$[Cnts/Chnl]	1.21	±0.16	13%
$Shift_{IRF}$[ps]	10.8	±1.1	9.8%
A_{Scatt}[kCnts]	39.0	±1.4	3.5%
τ_{AvInt}[ns]	3.893	±0.013	0.3%
τ_{AvAmp}[ns]	1.589	±0.011	0.7%
λ_{Det}[nm]	480.0000	---	---

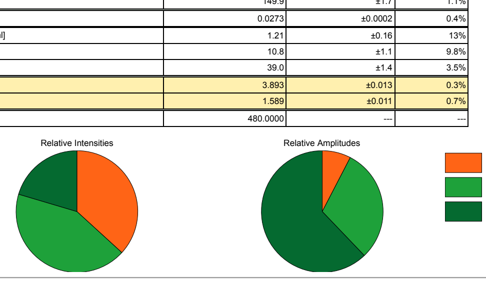

Data Set: 10 / 31

Decay: crv[10]; IRF: crv[0]

Fit

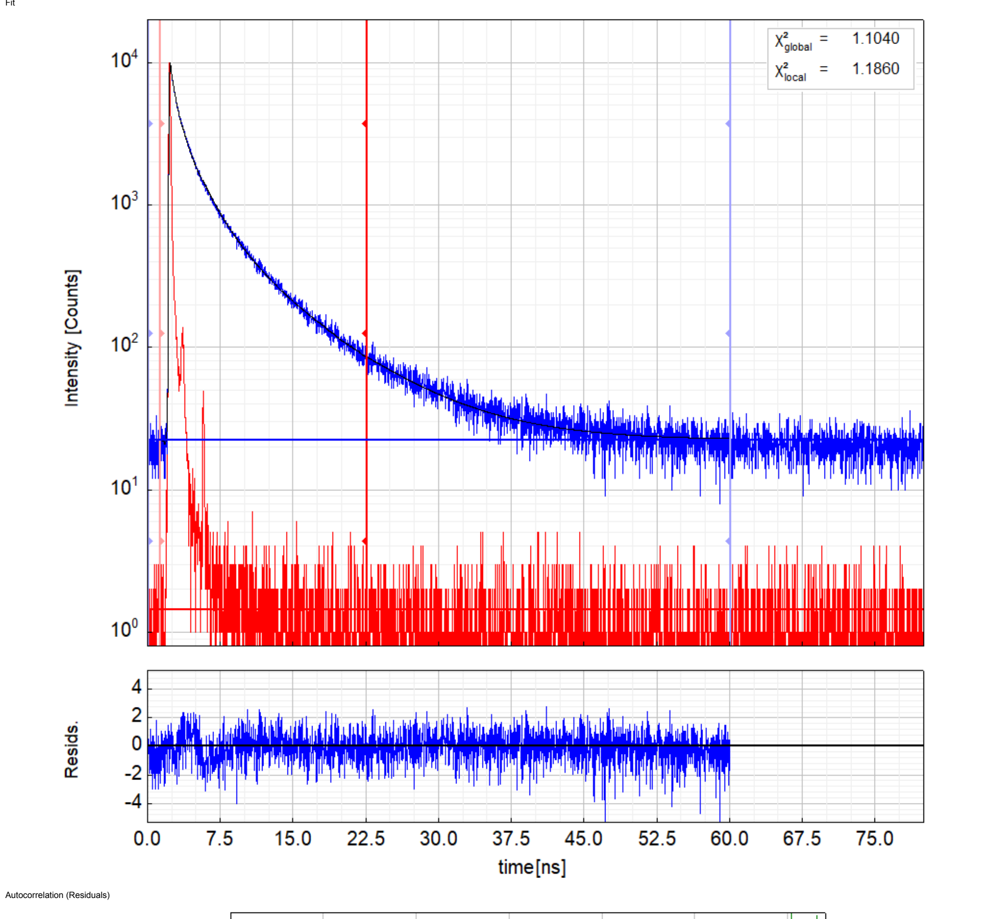

Autocorrelation (Residuals)

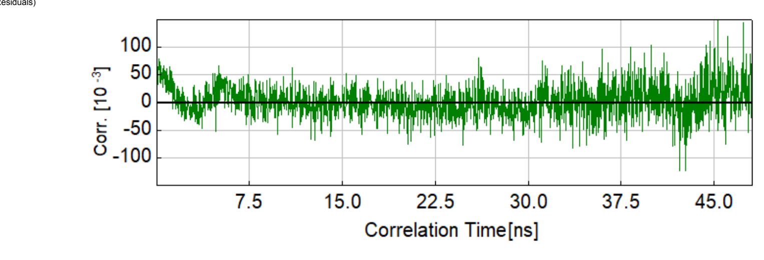

Fitted Parameters

Parameter	Value	Δ	δ
A_1[kCnts/Chnl]	0.9228	±0.0067	0.7%
τ_1[ns]	7.675	±0.012	0.2%
I_1[kCnts]	283.3	±2.1	0.7%
A_2[kCnts/Chnl]	3.714	±0.053	1.4%
τ_2[ns]	2.250	±0.013	0.6%
I_2[kCnts]	334.2	±3.3	1.0%
A_3[kCnts/Chnl]	7.096	±0.058	0.8%
τ_3[ns]	0.5216	±0.0058	1.1%
I_3[kCnts]	148.1	±2.2	1.5%
$Bkgr_{Dec}$[kCnts]	0.0224	±0.0001	0.4%
$Bkgr_{IRF}$[Cnts/Chnl]	1.45	±0.15	10%
$Shift_{IRF}$[ps]	10.64	±0.59	5.5%
A_{Scatt}[kCnts]	37.83	±0.68	1.8%
τ_{AvInt}[ns]	3.924	±0.020	0.5%
τ_{AvAmp}[ns]	1.6312	±0.0070	0.4%
λ_{Det}[nm]	490.0000	---	---

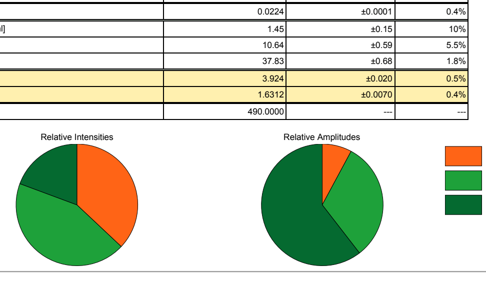

Data Set: 11 / 31

Decay: crv[11]; IRF: crv[0]

Fit

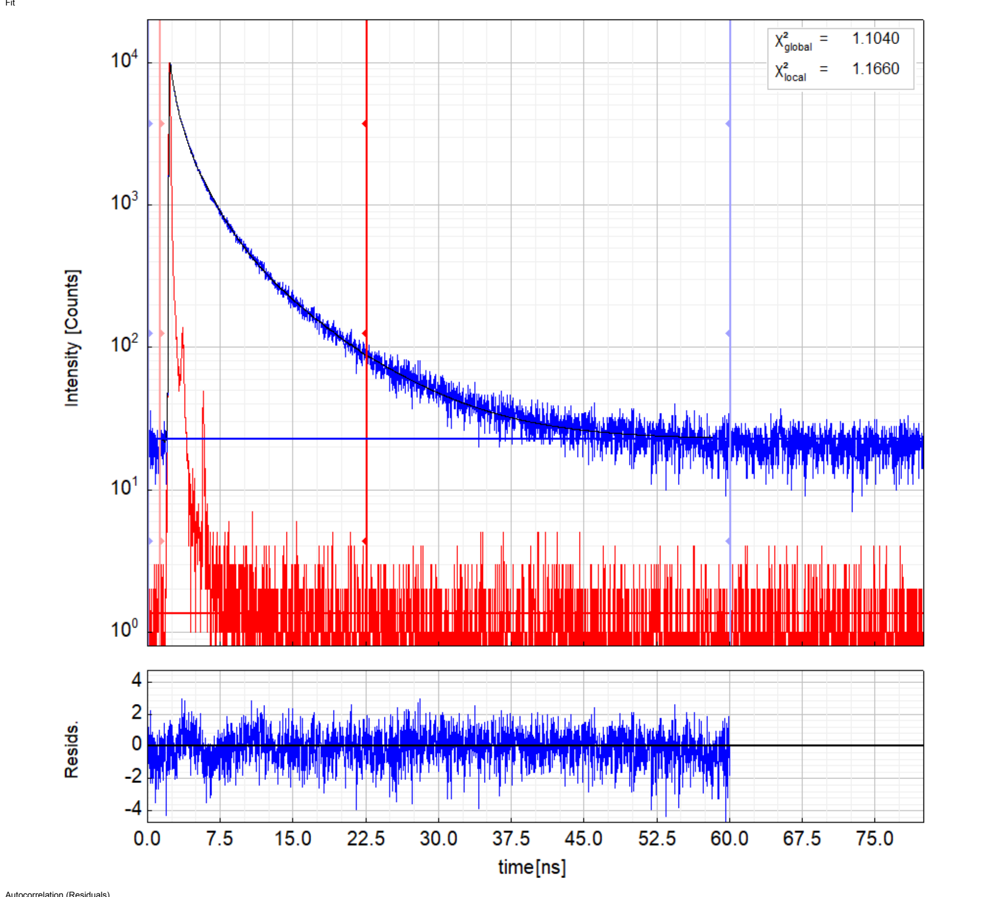

Autocorrelation (Residuals)

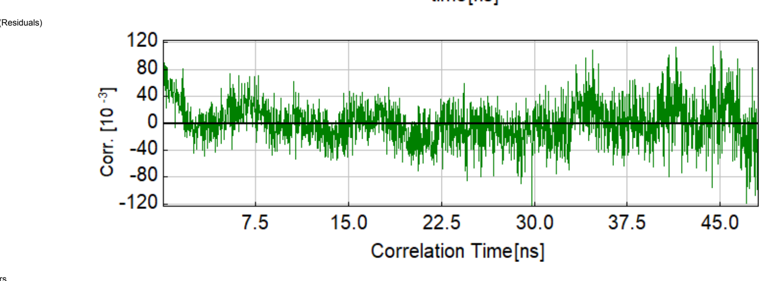

Fitted Parameters

Parameter	Value	Δ	δ
A_1[kCnts/Chnl]	0.9403	±0.0045	0.5%
τ_1[ns]	7.675	±0.012	0.2%
I_1[kCnts]	288.65	±0.96	0.3%
A_2[kCnts/Chnl]	3.912	±0.032	0.8%
τ_2[ns]	2.250	±0.013	0.6%
I_2[kCnts]	351.9	±2.8	0.8%
A_3[kCnts/Chnl]	6.794	±0.047	0.7%
τ_3[ns]	0.5216	±0.0058	1.1%
I_3[kCnts]	141.8	±1.8	1.2%
$Bkgr_{Dec}$[kCnts]	0.0226	±0.0002	0.8%
$Bkgr_{IRF}$[Cnts/Chnl]	1.348	±0.086	6.4%
$Shift_{IRF}$[ps]	11.72	±0.73	6.2%
A_{Scatt}[kCnts]	38.45	±0.47	1.2%
τ_{AvInt}[ns]	3.9382	±0.0049	0.1%
τ_{AvAmp}[ns]	1.6795	±0.0067	0.4%
λ_{Det}[nm]	500.0000	---	---

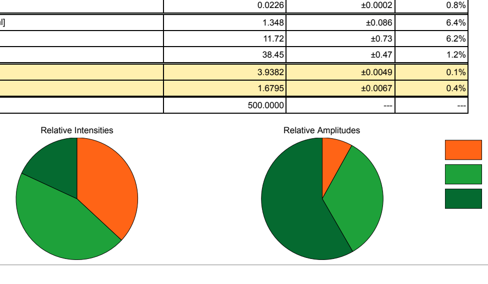

Data Set: 12 / 31

Decay: crv[12]; IRF: crv[0]

Fit

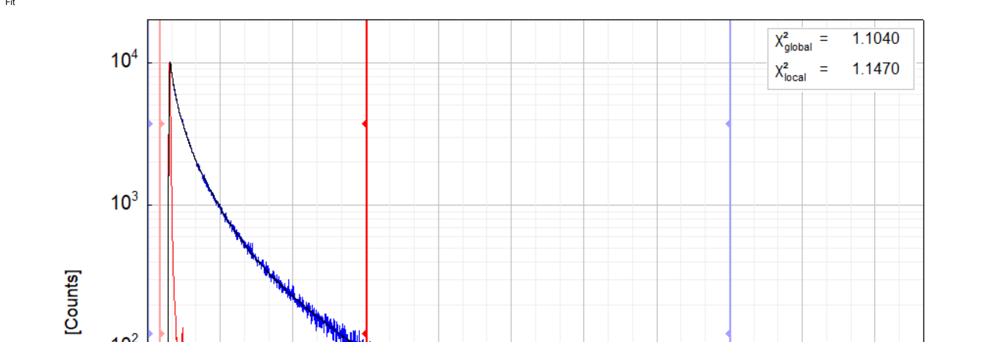

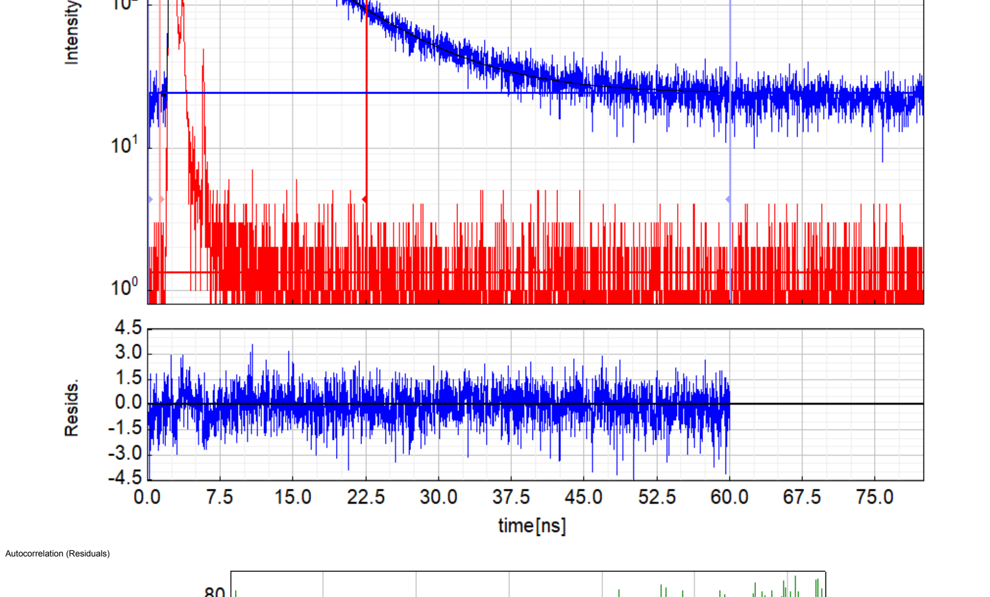

Autocorrelation (Residuals)

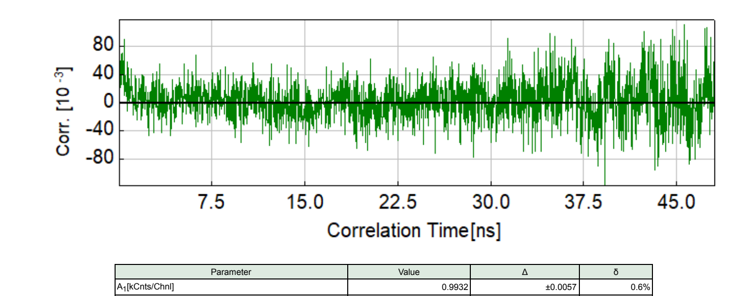

Fitted Parameters

Parameter	Value	Δ	δ
A_1[kCnts/Chnl]	0.9932	±0.0057	0.6%
τ_1[ns]	7.675	±0.012	0.2%
I_1[kCnts]	305.0	±1.9	0.6%
A_2[kCnts/Chnl]	4.149	±0.047	1.1%
τ_2[ns]	2.250	±0.013	0.6%
I_2[kCnts]	373.3	±2.5	0.7%
A_3[kCnts/Chnl]	6.703	±0.064	1.0%
τ_3[ns]	0.5216	±0.0058	1.1%
I_3[kCnts]	139.9	±2.1	1.5%
$Bkgr_{Dec}$[kCnts]	0.0242	±0.0002	0.5%
$Bkgr_{IRF}$[Cnts/Chnl]	1.32	±0.13	9.5%
$Shift_{IRF}$[ps]	11.69	±0.60	5.1%
A_{Scatt}[kCnts]	39.21	±0.50	1.3%
τ_{AvInt}[ns]	3.977	±0.012	0.3%
τ_{AvAmp}[ns]	1.727	±0.011	0.6%
λ_{Det}[nm]	510.0000	---	---

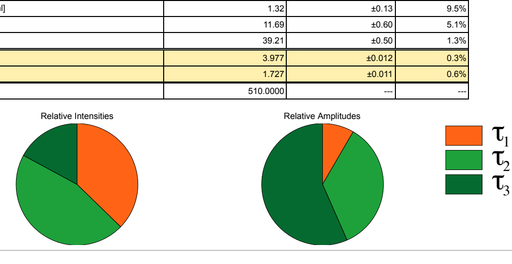

Data Set: 13 / 31

Decay: crv[13]; IRF: crv[0]

Fit

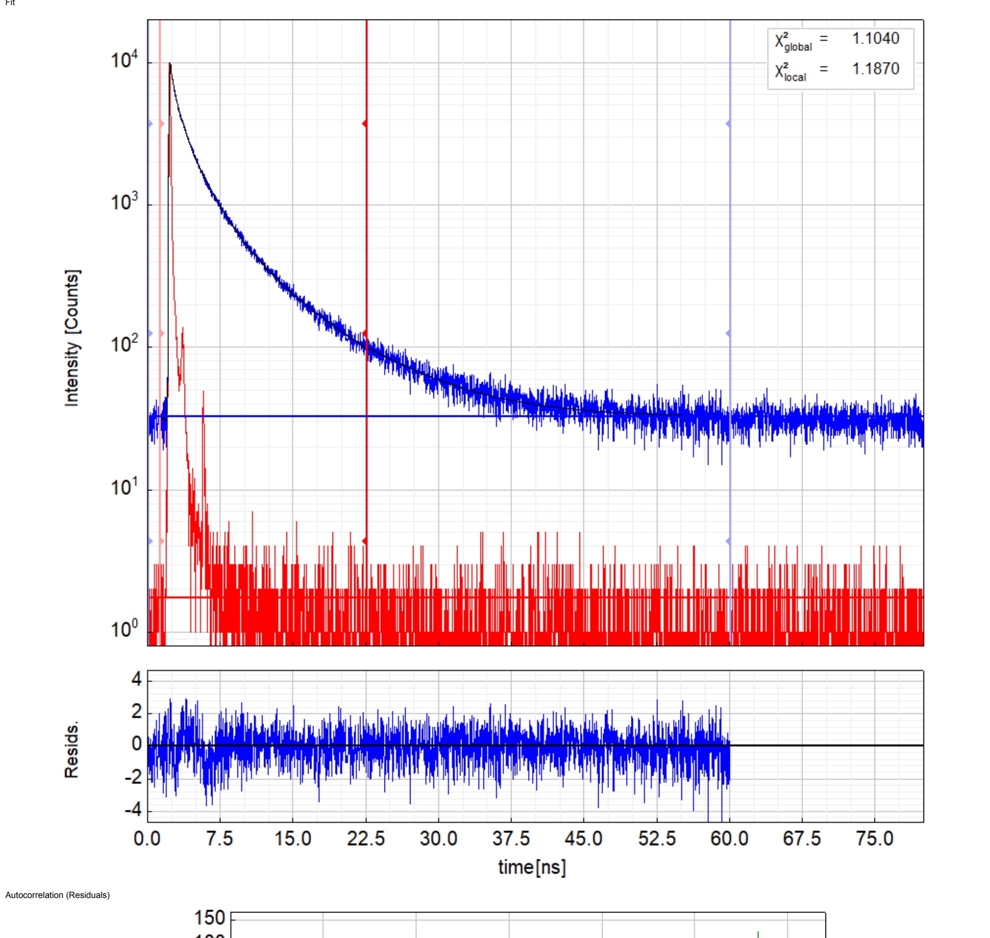

Autocorrelation (Residuals)

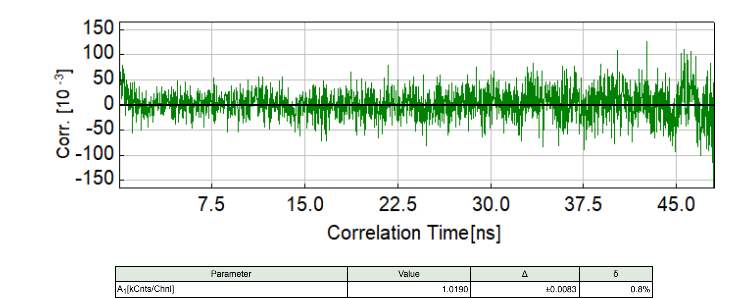

Fitted Parameters

Parameter	Value	Δ	δ
A_1[kCnts/Chnl]	1.0190	±0.0083	0.8%
τ_1[ns]	7.675	±0.012	0.2%
I_1[kCnts]	312.9	±2.5	0.8%
A_2[kCnts/Chnl]	4.220	±0.044	1.0%
τ_2[ns]	2.250	±0.013	0.6%
I_2[kCnts]	379.6	±2.1	0.5%
A_3[kCnts/Chnl]	6.320	±0.069	1.1%
τ_3[ns]	0.5216	±0.0058	1.1%
I_3[kCnts]	131.9	±2.1	1.6%
$Bkgr_{Dec}$[kCnts]	0.0324	±0.0002	0.4%
$Bkgr_{IRF}$[Cnts/Chnl]	1.745	±0.096	5.5%
$Shift_{IRF}$[ps]	11.56	±0.84	7.2%
A_{Scatt}[kCnts]	37.66	±0.78	2.1%
τ_{AvInt}[ns]	4.033	±0.020	0.5%
τ_{AvAmp}[ns]	1.783	±0.011	0.6%
λ_{Det}[nm]	520.0000	---	---

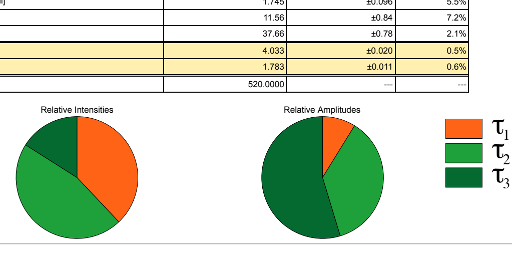

Data Set: 14 / 31

Decay: crv[14]; IRF: crv[0]

Fit

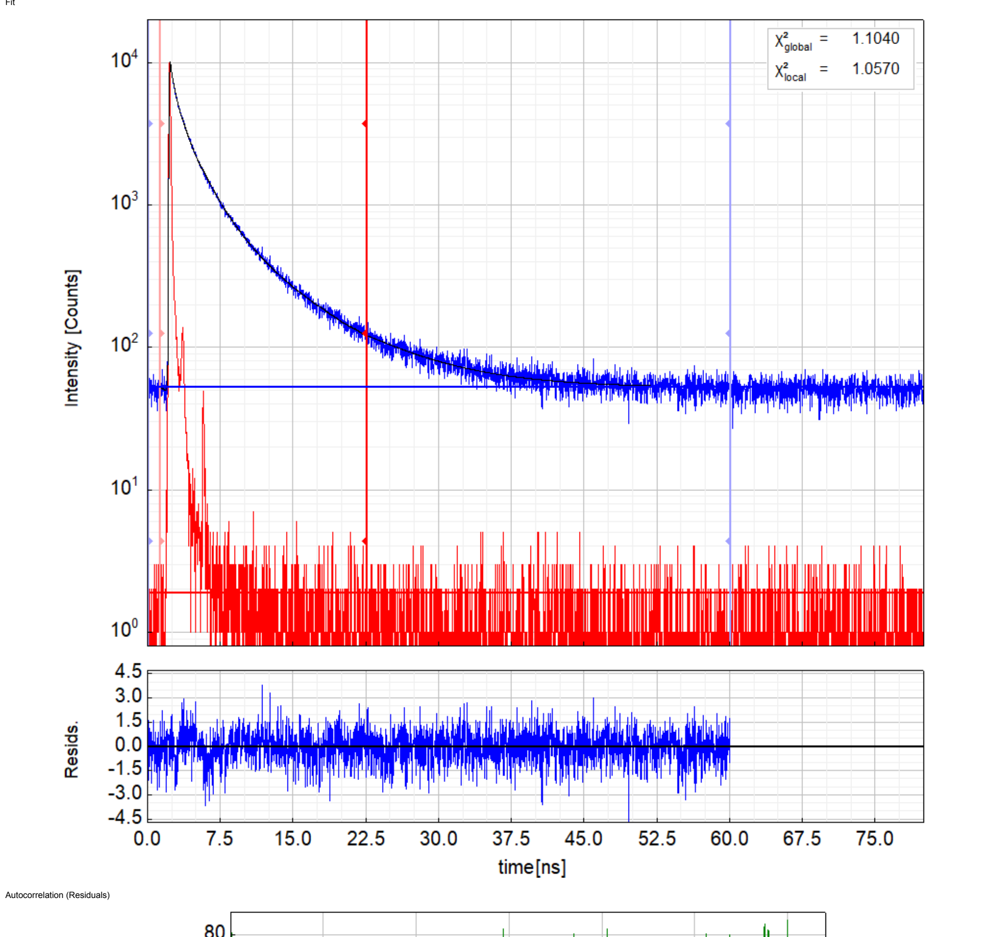

Autocorrelation (Residuals)

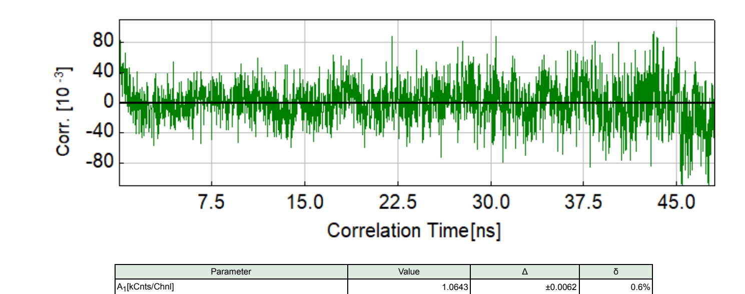

Fitted Parameters

Parameter	Value	Δ	δ
A_1[kCnts/Chnl]	1.0643	±0.0062	0.6%
τ_1[ns]	7.675	±0.012	0.2%
I_1[kCnts]	326.8	±2.1	0.6%
A_2[kCnts/Chnl]	4.474	±0.062	1.4%
τ_2[ns]	2.250	±0.013	0.6%
I_2[kCnts]	402.5	±3.7	0.9%
A_3[kCnts/Chnl]	6.117	±0.043	0.7%
τ_3[ns]	0.5216	±0.0058	1.1%
I_3[kCnts]	127.7	±1.2	0.9%
$Bkgr_{Dec}$[kCnts]	0.0522	±0.0003	0.5%
$Bkgr_{IRF}$[Cnts/Chnl]	1.89	±0.13	6.4%
$Shift_{IRF}$[ps]	13.60	±0.42	3.1%
A_{Scatt}[kCnts]	38.50	±0.59	1.5%
τ_{AvInt}[ns]	4.061	±0.013	0.3%
τ_{AvAmp}[ns]	1.8381	±0.0099	0.5%
λ_{Det}[nm]	530.0000	---	---

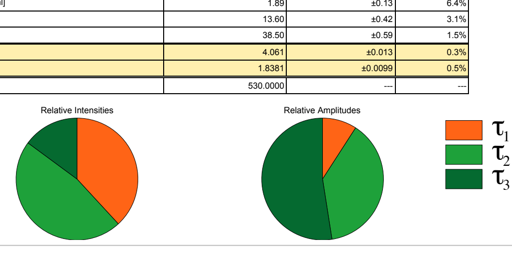

Data Set: 15 / 31

Decay: crv[15]; IRF: crv[0]

Fit

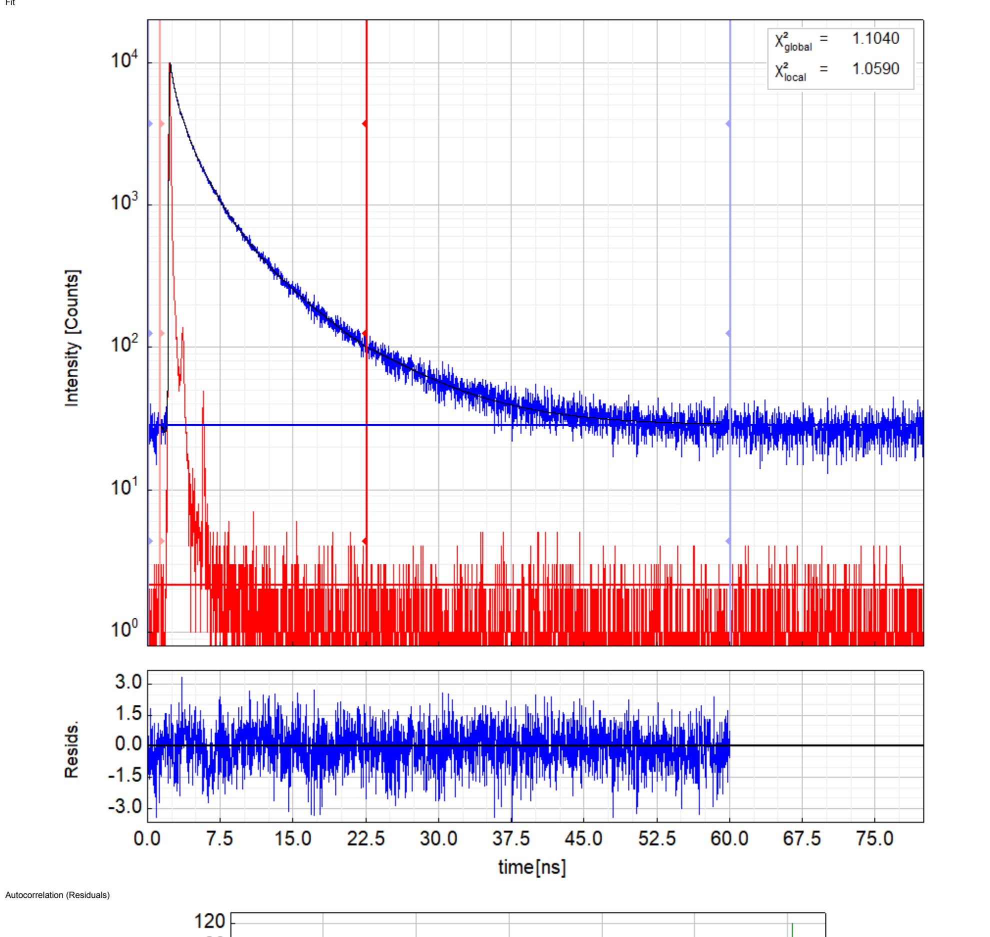

Autocorrelation (Residuals)

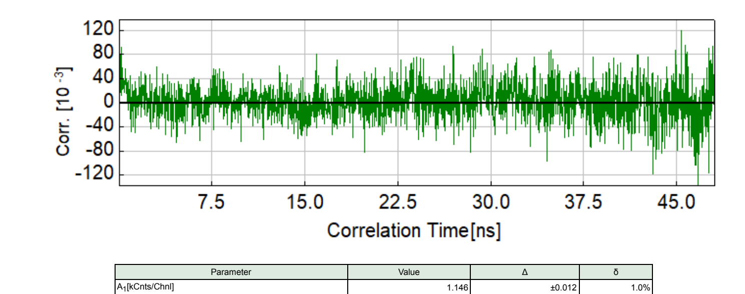

Fitted Parameters

Parameter	Value	Δ	δ
A_1[kCnts/Chnl]	1.146	±0.012	1.0%
τ_1[ns]	7.675	±0.012	0.2%
I_1[kCnts]	351.6	±3.9	1.1%
A_2[kCnts/Chnl]	4.563	±0.040	0.9%
τ_2[ns]	2.250	±0.013	0.6%
I_2[kCnts]	410.5	±3.0	0.7%
A_3[kCnts/Chnl]	5.913	±0.089	1.5%
τ_3[ns]	0.5216	±0.0058	1.1%
I_3[kCnts]	123.4	±2.7	2.2%
$Bkgr_{Dec}$[kCnts]	0.0284	±0.0002	0.5%
$Bkgr_{IRF}$[Cnts/Chnl]	2.13	±0.11	5.2%
$Shift_{IRF}$[ps]	13.62	±0.59	4.3%
A_{Scatt}[kCnts]	37.91	±0.75	2.0%
τ_{AvInt}[ns]	4.163	±0.013	0.3%
τ_{AvAmp}[ns]	1.9049	±0.0076	0.4%
λ_{Det}[nm]	540.0000	---	---

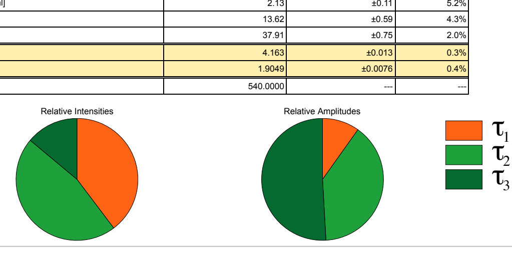

Data Set: 16 / 31

Decay: crv[16]; IRF: crv[0]

Fit

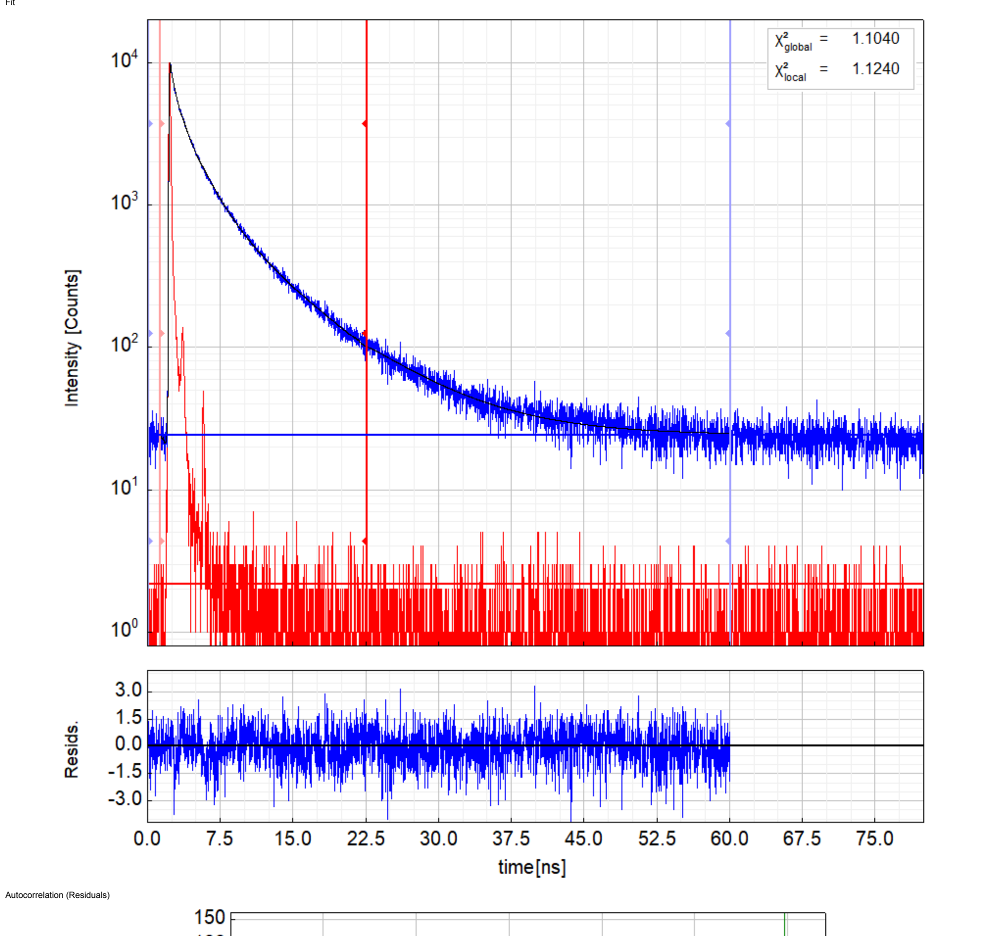

Autocorrelation (Residuals)

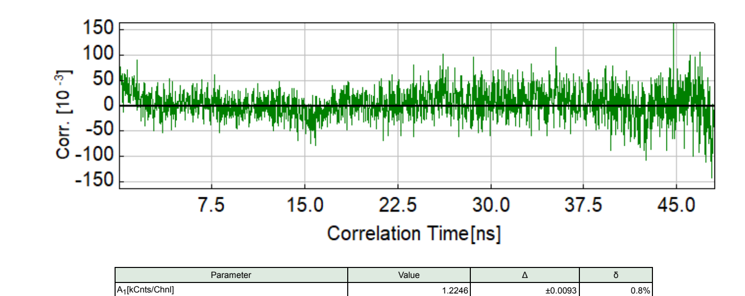

Fitted Parameters

Parameter	Value	Δ	δ
A_1[kCnts/Chnl]	1.2246	±0.0093	0.8%
τ_1[ns]	7.675	±0.012	0.2%
I_1[kCnts]	376.0	±2.1	0.6%
A_2[kCnts/Chnl]	4.545	±0.038	0.8%
τ_2[ns]	2.250	±0.013	0.6%
I_2[kCnts]	408.9	±1.6	0.4%
A_3[kCnts/Chnl]	5.708	±0.052	0.9%
τ_3[ns]	0.5216	±0.0058	1.1%
I_3[kCnts]	119.1	±2.7	2.2%
$Bkgr_{Dec}$[kCnts]	0.0243	±0.0002	0.8%
$Bkgr_{IRF}$[Cnts/Chnl]	2.164	±0.094	4.3%
$Shift_{IRF}$[ps]	14.56	±0.44	3.0%
A_{Scatt}[kCnts]	38.54	±0.43	1.1%
τ_{AvInt}[ns]	4.279	±0.011	0.3%
τ_{AvAmp}[ns]	1.9689	±0.0089	0.4%
λ_{Det}[nm]	550.0000	---	---

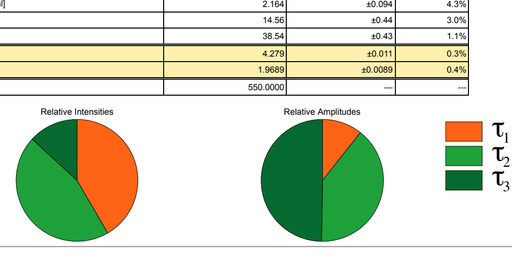

Data Set: 17 / 31

Decay: crv[17]; IRF: crv[0]

Fit

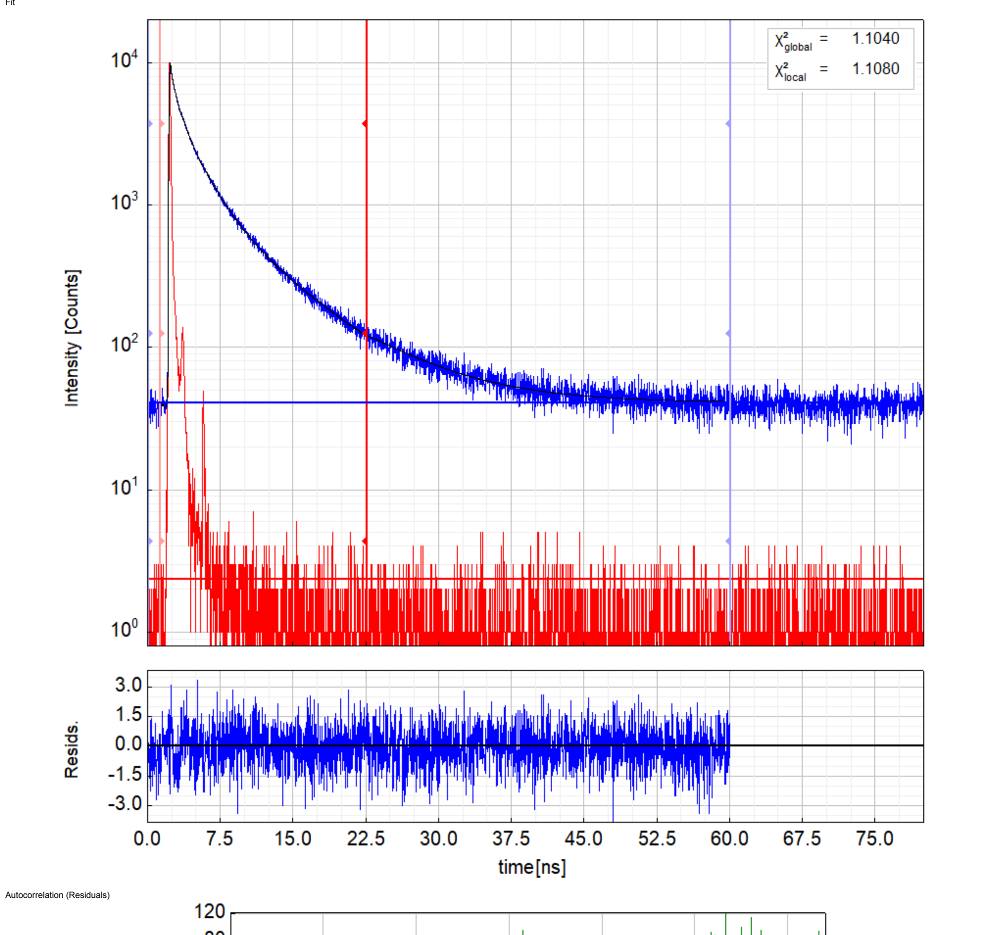

Autocorrelation (Residuals)

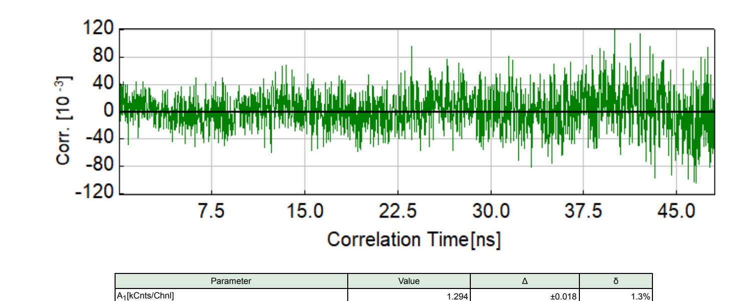

Fitted Parameters

Parameter	Value	Δ	δ
A_1[kCnts/Chnl]	1.294	±0.018	1.3%
τ_1[ns]	7.675	±0.012	0.2%
I_1[kCnts]	397.2	±5.0	1.2%
A_2[kCnts/Chnl]	4.475	±0.048	1.1%
τ_2[ns]	2.250	±0.013	0.6%
I_2[kCnts]	402.6	±4.5	1.1%
A_3[kCnts/Chnl]	5.45	±0.11	2.0%
τ_3[ns]	0.5216	±0.0058	1.1%
I_3[kCnts]	113.6	±1.9	1.6%
$Bkgr_{Dec}$[kCnts]	0.0410	±0.0003	0.5%
$Bkgr_{IRF}$[Cnts/Chnl]	2.36	±0.19	7.8%
$Shift_{IRF}$[ps]	14.58	±0.79	5.4%
A_{Scatt}[kCnts]	39.40	±0.96	2.4%
τ_{AvInt}[ns]	4.394	±0.019	0.4%
τ_{AvAmp}[ns]	2.037	±0.014	0.6%
λ_{Det}[nm]	560.0000	---	---

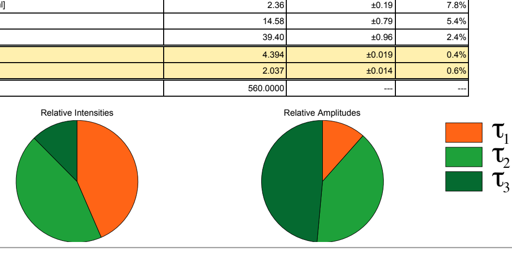

Data Set: 18 / 31

Decay: crv[18]; IRF: crv[0]

Fit

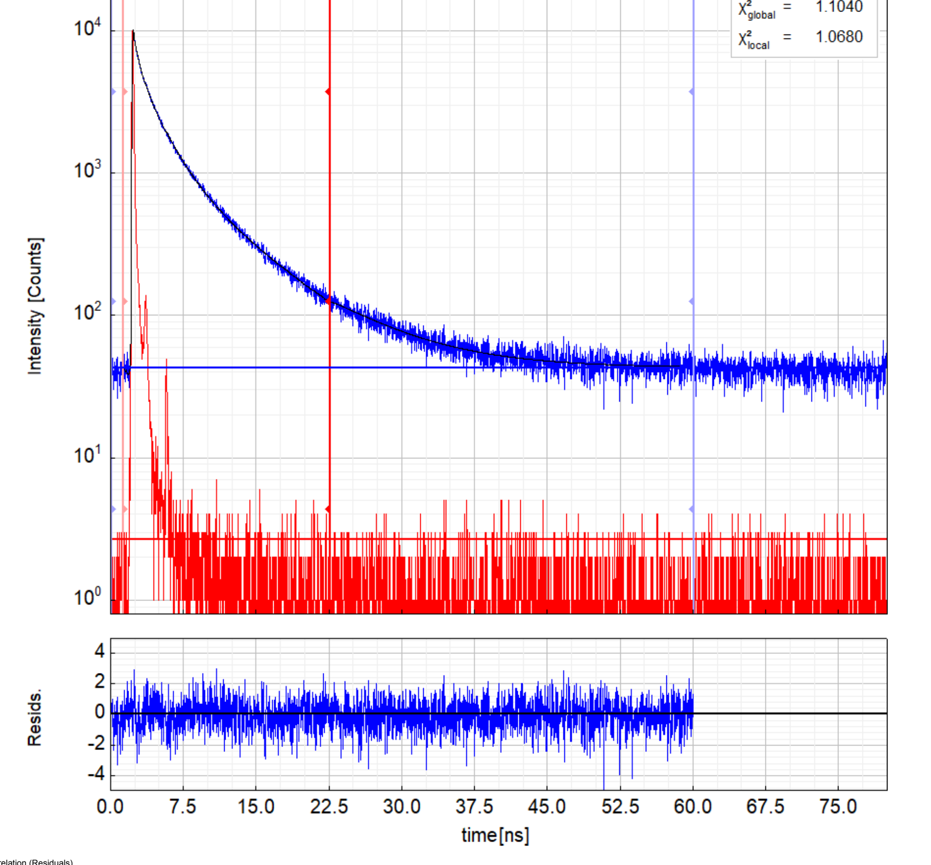

Autocorrelation (Residuals)

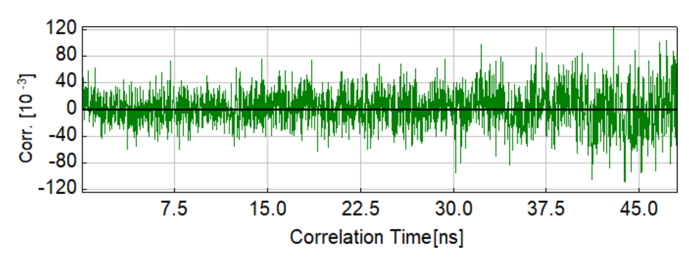

Fitted Parameters

Parameter	Value	Δ	δ
A_1[kCnts/Chnl]	1.381	±0.017	1.2%
$τ_1$[ns]	7.675	±0.012	0.2%
I_1[kCnts]	424.0	±4.5	1.1%
A_2[kCnts/Chnl]	4.514	±0.024	0.5%
$τ_2$[ns]	2.250	±0.013	0.6%
I_2[kCnts]	406.1	±4.6	1.1%
A_3[kCnts/Chnl]	5.489	±0.072	1.3%
$τ_3$[ns]	0.5216	±0.0058	1.1%
I_3[kCnts]	114.6	±1.9	1.6%
$Bkgr_{Dec}$[kCnts]	0.0430	±0.0002	0.4%
$Bkgr_{IRF}$[Cnts/Chnl]	2.69	±0.21	7.5%
$Shift_{IRF}$[ps]	15.46	±0.56	3.6%
A_{Scatt}[kCnts]	40.12	±0.48	1.2%
$τ_{AvInt}$[ns]	4.475	±0.019	0.4%
$τ_{AvAmp}$[ns]	2.0744	±0.0048	0.2%
$λ_{Det}$[nm]	570.0000	---	---

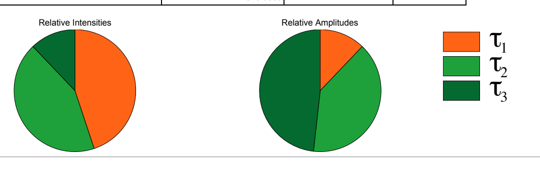

Data Set: 19 / 31

Decay: crv[19]; IRF: crv[0]

Fit

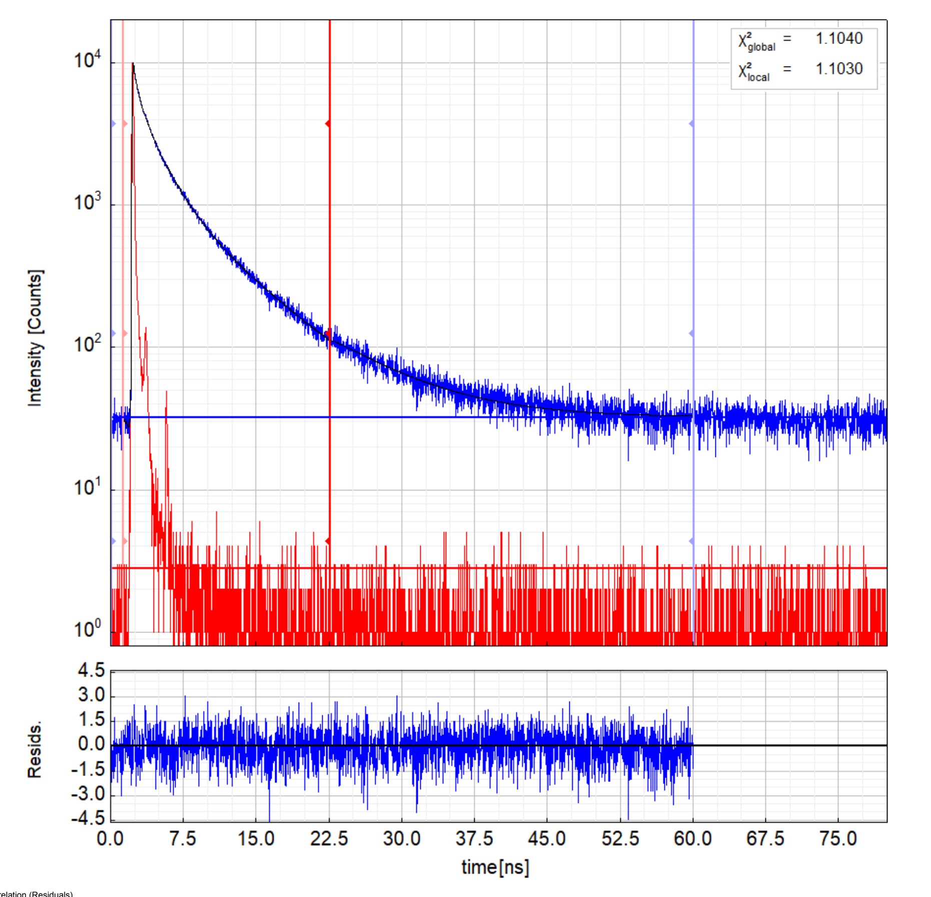

Autocorrelation (Residuals)

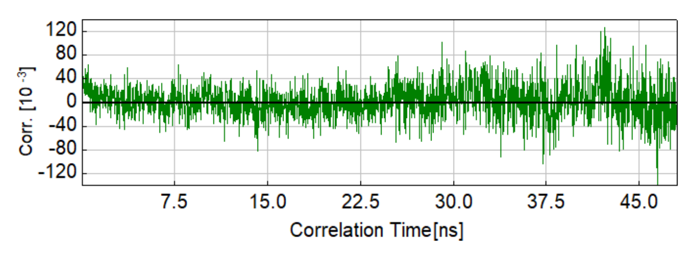

Fitted Parameters

Parameter	Value	Δ	δ
A_1[kCnts/Chnl]	1.371	±0.018	1.3%
$τ_1$[ns]	7.675	±0.012	0.2%
I_1[kCnts]	420.8	±4.6	1.1%
A_2[kCnts/Chnl]	4.467	±0.024	0.5%
$τ_2$[ns]	2.250	±0.013	0.6%
I_2[kCnts]	401.9	±1.7	0.4%
A_3[kCnts/Chnl]	5.317	±0.084	1.6%
$τ_3$[ns]	0.5216	±0.0058	1.1%
I_3[kCnts]	111.0	±1.5	1.3%
$Bkgr_{Dec}$[kCnts]	0.0322	±0.0002	0.4%
$Bkgr_{IRF}$[Cnts/Chnl]	2.80	±0.12	4.1%
$Shift_{IRF}$[ps]	16.32	±0.99	6.1%
A_{Scatt}[kCnts]	41.15	±0.84	2.0%
$τ_{AvInt}$[ns]	4.490	±0.014	0.3%
$τ_{AvAmp}$[ns]	2.0924	±0.0093	0.4%
$λ_{Det}$[nm]	580.0000	---	---

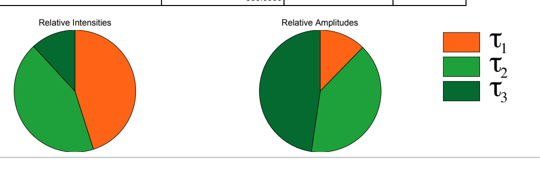

Data Set: 20 / 31

Decay: crv[20]; IRF: crv[0]

Fit

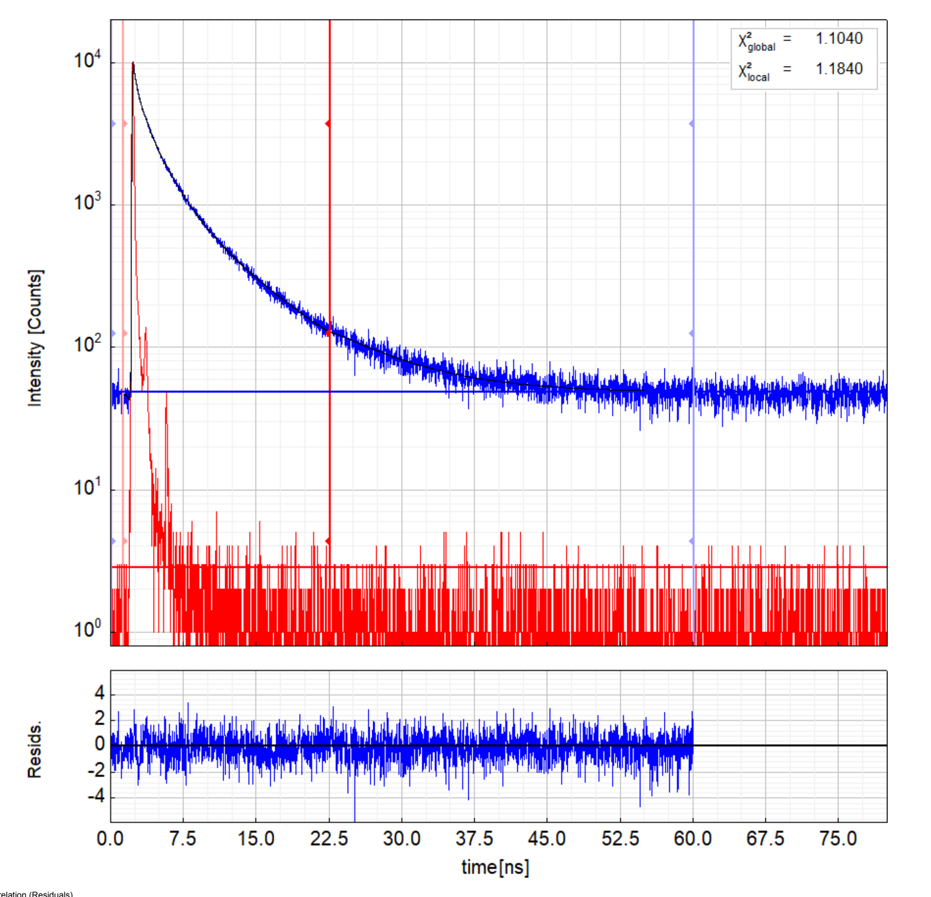

Autocorrelation (Residuals)

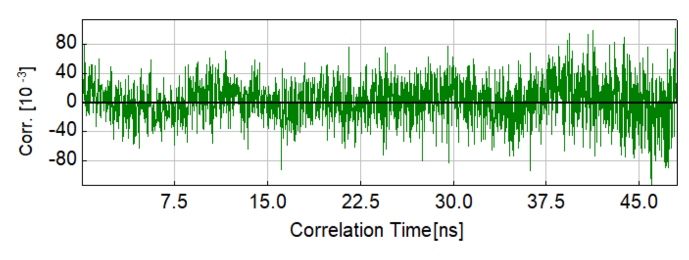

Fitted Parameters

Parameter	Value	Δ	δ
A_1[kCnts/Chnl]	1.3639	±0.0037	0.3%
$τ_1$[ns]	7.675	±0.012	0.2%
I_1[kCnts]	418.7	±1.5	0.3%
A_2[kCnts/Chnl]	4.364	±0.051	1.2%
$τ_2$[ns]	2.250	±0.013	0.6%
I_2[kCnts]	392.6	±2.8	0.7%
A_3[kCnts/Chnl]	5.268	±0.081	1.5%
$τ_3$[ns]	0.5216	±0.0058	1.1%
I_3[kCnts]	109.9	±1.5	1.3%
$Bkgr_{Dec}$[kCnts]	0.0484	±0.0002	0.3%
$Bkgr_{IRF}$[Cnts/Chnl]	2.85	±0.11	3.6%
$Shift_{IRF}$[ps]	16.78	±0.17	1.0%
A_{Scatt}[kCnts]	42.64	±0.30	0.7%
$τ_{AvInt}$[ns]	4.510	±0.016	0.3%
$τ_{AvAmp}$[ns]	2.095	±0.013	0.6%
$λ_{Det}$[nm]	590.0000	---	---

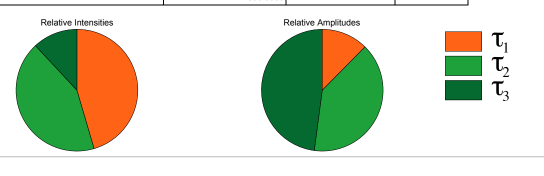

Data Set: 21 / 31

Decay: crv[21]; IRF: crv[0]

Fit

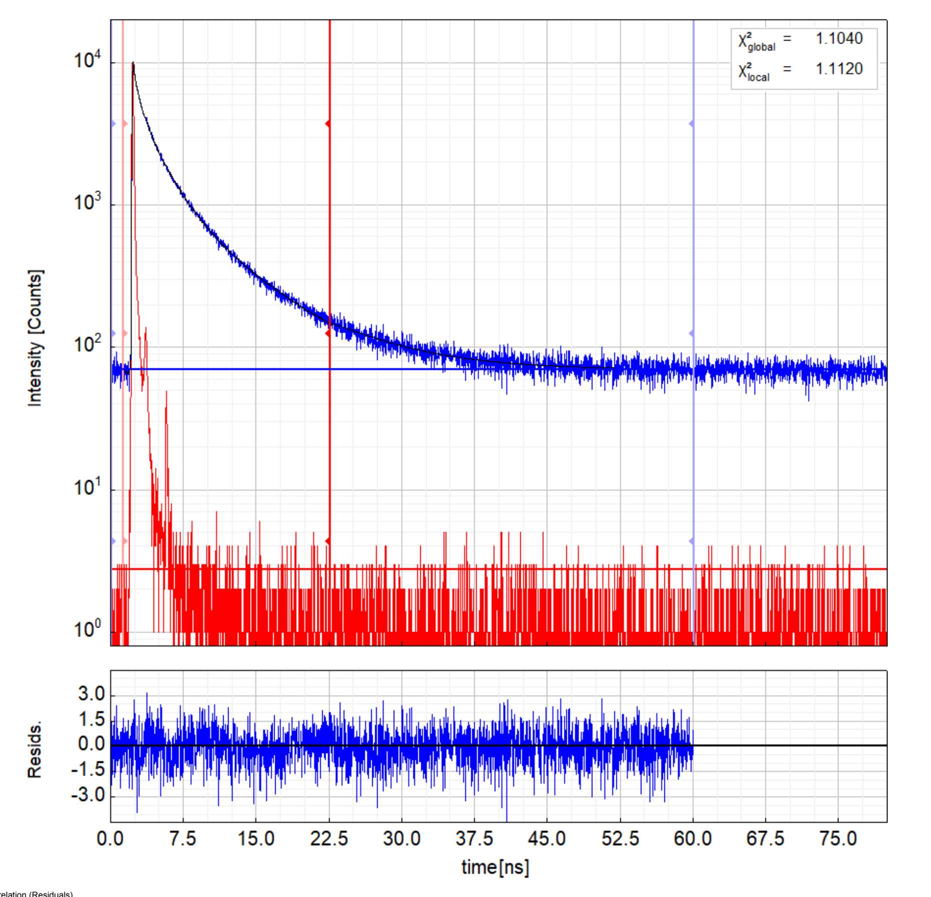

Autocorrelation (Residuals)

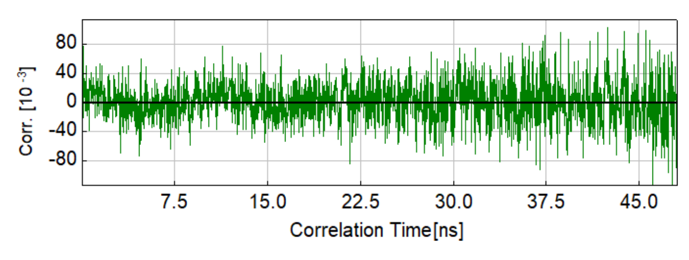

Fitted Parameters

Parameter	Value	Δ	δ
A_1[kCnts/Chnl]	1.3253	±0.0074	0.6%
$τ_1$[ns]	7.675	±0.012	0.2%
I_1[kCnts]	406.9	±2.2	0.5%
A_2[kCnts/Chnl]	4.228	±0.025	0.6%
$τ_2$[ns]	2.250	±0.013	0.6%
I_2[kCnts]	380.38	±0.99	0.3%
A_3[kCnts/Chnl]	5.275	±0.065	1.2%
$τ_3$[ns]	0.5216	±0.0058	1.1%
I_3[kCnts]	110.05	±0.70	0.6%
$Bkgr_{Dec}$[kCnts]	0.0696	±0.0002	0.2%
$Bkgr_{IRF}$[Cnts/Chnl]	2.742	±0.097	3.5%
$Shift_{IRF}$[ps]	16.85	±0.31	1.8%
A_{Scatt}[kCnts]	43.27	±0.82	1.9%
$τ_{AvInt}$[ns]	4.498	±0.013	0.3%
$τ_{AvAmp}$[ns]	2.072	±0.017	0.8%
$λ_{Det}$[nm]	600.0000	---	---

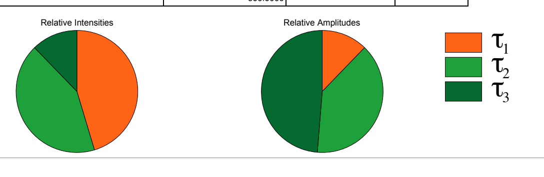

Data Set: 22 / 31

Decay: crv[22]; IRF: crv[0]

Fit

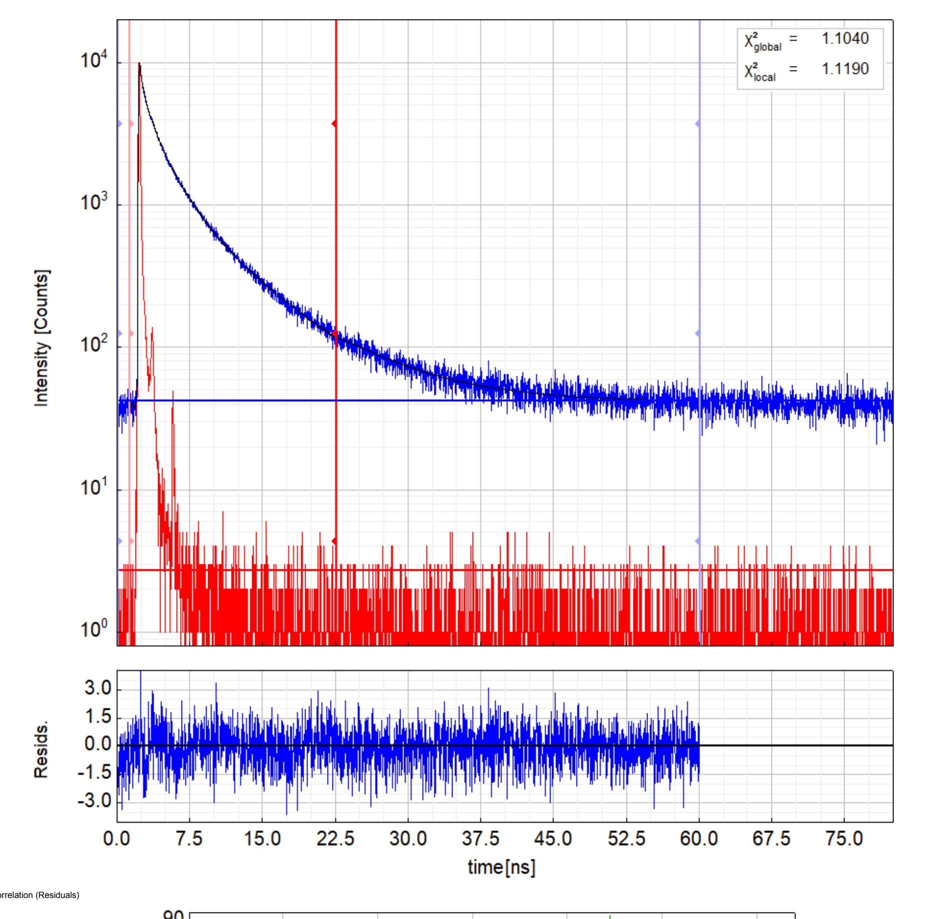

Autocorrelation (Residuals)

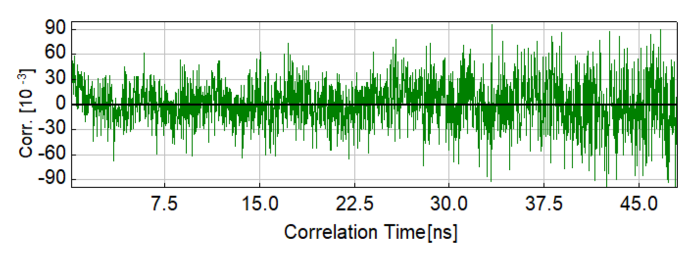

Fitted Parameters

Parameter	Value	Δ	δ
A_1[kCnts/Chnl]	1.2764	±0.0084	0.7%
$τ_1$[ns]	7.675	±0.012	0.2%
I_1[kCnts]	391.9	±2.3	0.6%
A_2[kCnts/Chnl]	4.155	±0.040	0.9%
$τ_2$[ns]	2.250	±0.013	0.6%
I_2[kCnts]	373.8	±2.0	0.5%
A_3[kCnts/Chnl]	5.185	±0.079	1.5%
$τ_3$[ns]	0.5216	±0.0058	1.1%
I_3[kCnts]	108.2	±2.5	2.2%
$Bkgr_{Dec}$[kCnts]	0.0418	±0.0002	0.5%
$Bkgr_{IRF}$[Cnts/Chnl]	2.73	±0.11	3.9%
$Shift_{IRF}$[ps]	17.43	±0.31	1.8%
A_{Scatt}[kCnts]	44.58	±0.68	1.5%
$τ_{AvInt}$[ns]	4.469	±0.013	0.3%
$τ_{AvAmp}$[ns]	2.058	±0.011	0.5%
$λ_{Det}$[nm]	610.0000	---	---

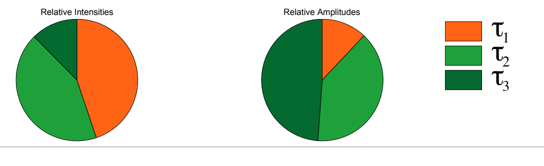

Data Set: 23 / 31

Decay: crv[23]; IRF: crv[0]

Fit

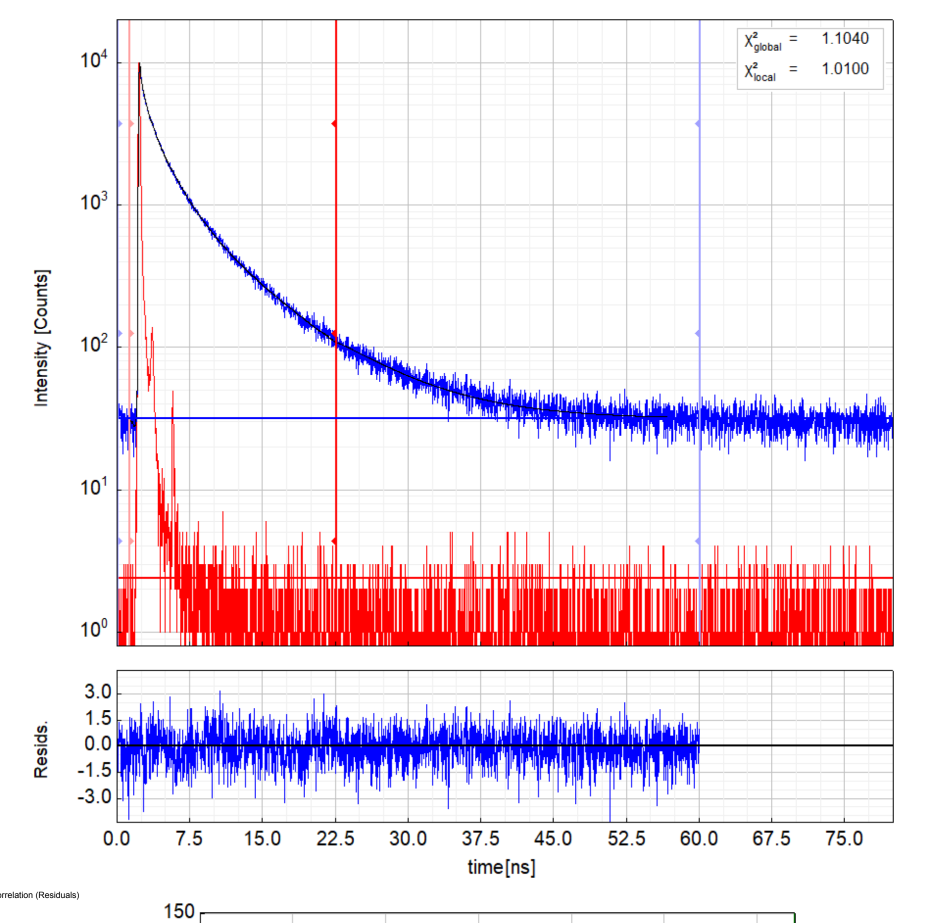

Autocorrelation (Residuals)

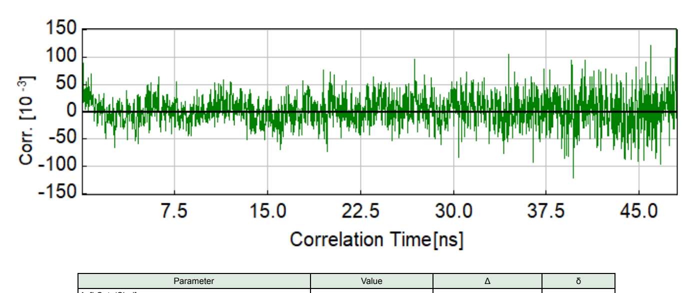

Fitted Parameters

Parameter	Value	Δ	δ
A_1[kCnts/Chnl]	1.2491	±0.0041	0.3%
$τ_1$[ns]	7.675	±0.012	0.2%
I_1[kCnts]	383.5	±1.8	0.5%
A_2[kCnts/Chnl]	3.956	±0.023	0.6%
$τ_2$[ns]	2.250	±0.013	0.6%
I_2[kCnts]	355.9	±1.3	0.4%
A_3[kCnts/Chnl]	4.978	±0.081	1.6%
$τ_3$[ns]	0.5216	±0.0058	1.1%
I_3[kCnts]	103.9	±2.7	2.5%
$Bkgr_{Dec}$[kCnts]	0.0314	±0.0002	0.4%
$Bkgr_{IRF}$[Cnts/Chnl]	2.39	±0.12	4.8%
$Shift_{IRF}$[ps]	19.11	±0.53	2.7%

A_{Scatt}[kCnts]	45.60	±0.85	1.9%
τ_{AvInt}[ns]	4.505	±0.013	0.3%
τ_{AvAmp}[ns]	2.071	±0.016	0.8%
λ_{Det}[nm]	620.0000	---	---

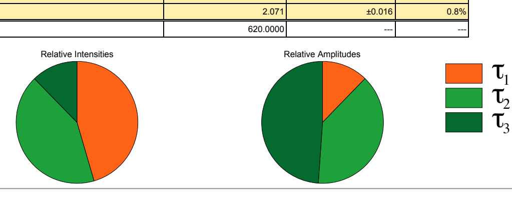

Data Set: 24 / 31

Decay: crv[24]; IRF: crv[0]

Fit

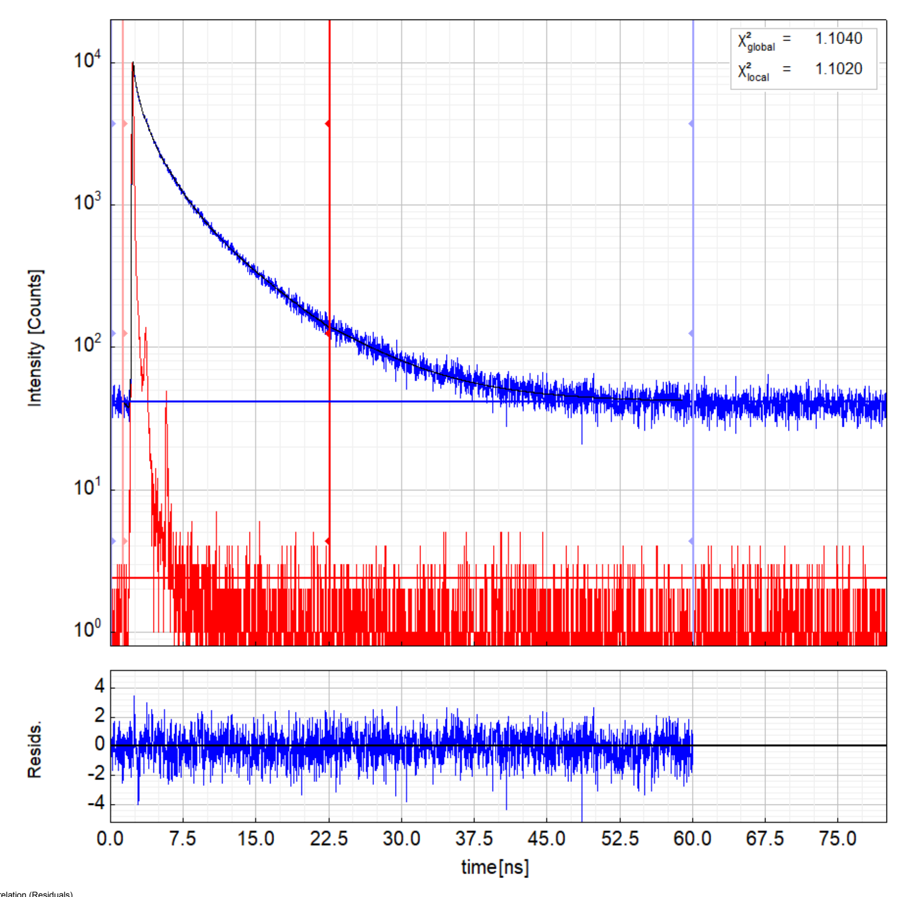

Autocorrelation (Residuals)

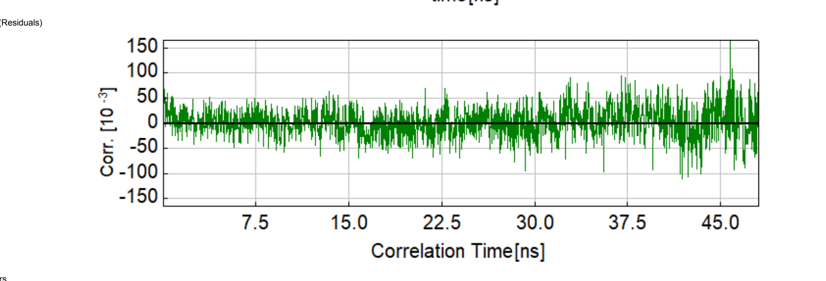

Fitted Parameters

Parameter	Value	Δ	δ
A_1[kCnts/Chnl]	1.547	±0.018	1.1%
τ_1[ns]	7.675	±0.012	0.2%
I_1[kCnts]	474.7	±4.1	0.9%
A_2[kCnts/Chnl]	3.920	±0.031	0.8%
τ_2[ns]	2.250	±0.013	0.6%
I_2[kCnts]	352.7	±2.7	0.8%
A_3[kCnts/Chnl]	4.989	±0.084	1.7%
τ_3[ns]	0.5216	±0.0058	1.1%
I_3[kCnts]	104.1	±1.8	1.6%
$Bkgr_{Dec}$[kCnts]	0.0415	±0.0003	0.6%
$Bkgr_{IRF}$[Cnts/Chnl]	2.38	±0.13	5.2%
$Shift_{IRF}$[ps]	19.13	±0.58	3.0%
A_{Scatt}[kCnts]	45.79	±0.85	1.8%
τ_{AvInt}[ns]	4.822	±0.011	0.2%
τ_{AvAmp}[ns]	2.228	±0.013	0.6%
λ_{Det}[nm]	630.0000	---	---

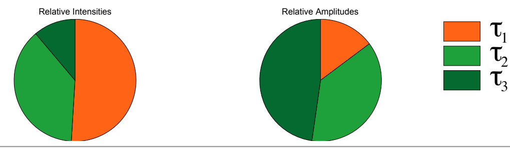

Data Set: 25 / 31

Decay: crv[25]; IRF: crv[0]

Fit

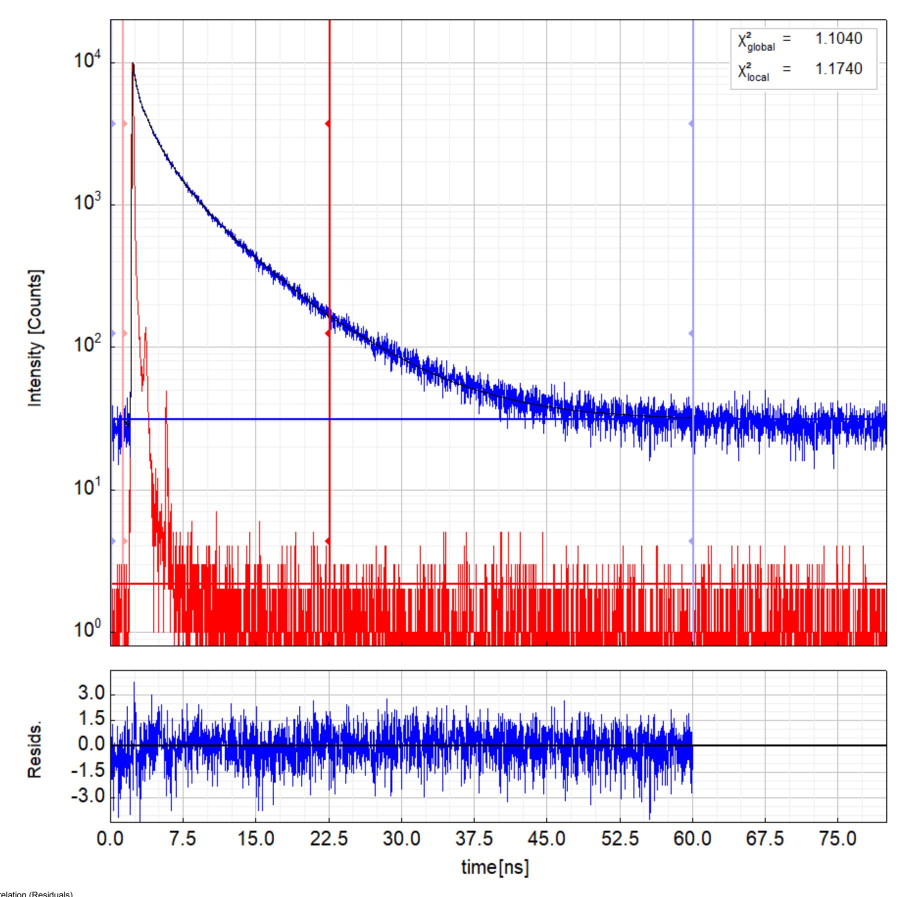

Autocorrelation (Residuals)

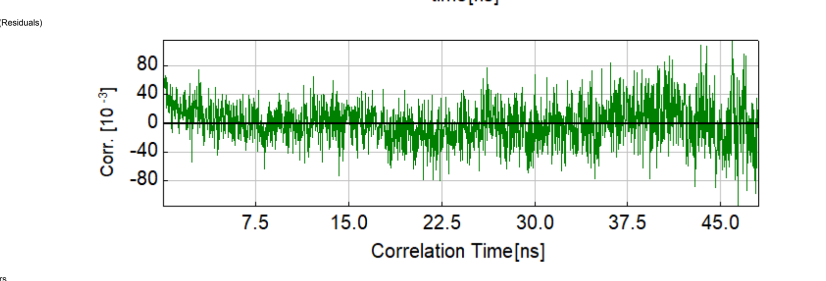

Fitted Parameters

Parameter	Value	Δ	δ
A_1[kCnts/Chnl]	2.044	±0.013	0.6%
τ_1[ns]	7.675	±0.012	0.2%
I_1[kCnts]	627.5	±3.7	0.6%
A_2[kCnts/Chnl]	3.949	±0.039	1.0%
τ_2[ns]	2.250	±0.013	0.6%
I_2[kCnts]	355.3	±3.4	0.9%
A_3[kCnts/Chnl]	4.426	±0.075	1.7%
τ_3[ns]	0.5216	±0.0058	1.1%
I_3[kCnts]	92.4	±2.9	3.1%
$Bkgr_{Dec}$[kCnts]	0.0310	±0.0002	0.4%
$Bkgr_{IRF}$[Cnts/Chnl]	2.17	±0.18	7.9%
$Shift_{IRF}$[ps]	19.96	±0.40	2.0%
A_{Scatt}[kCnts]	44.64	±0.69	1.5%
τ_{AvInt}[ns]	5.2677	±0.0092	0.2%
τ_{AvAmp}[ns]	2.5798	±0.0097	0.4%
λ_{Det}[nm]	640.0000	---	---

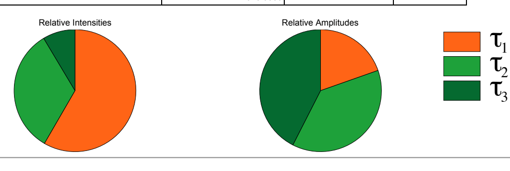

Data Set: 26 / 31

Decay: crv[26]; IRF: crv[0]

Fit

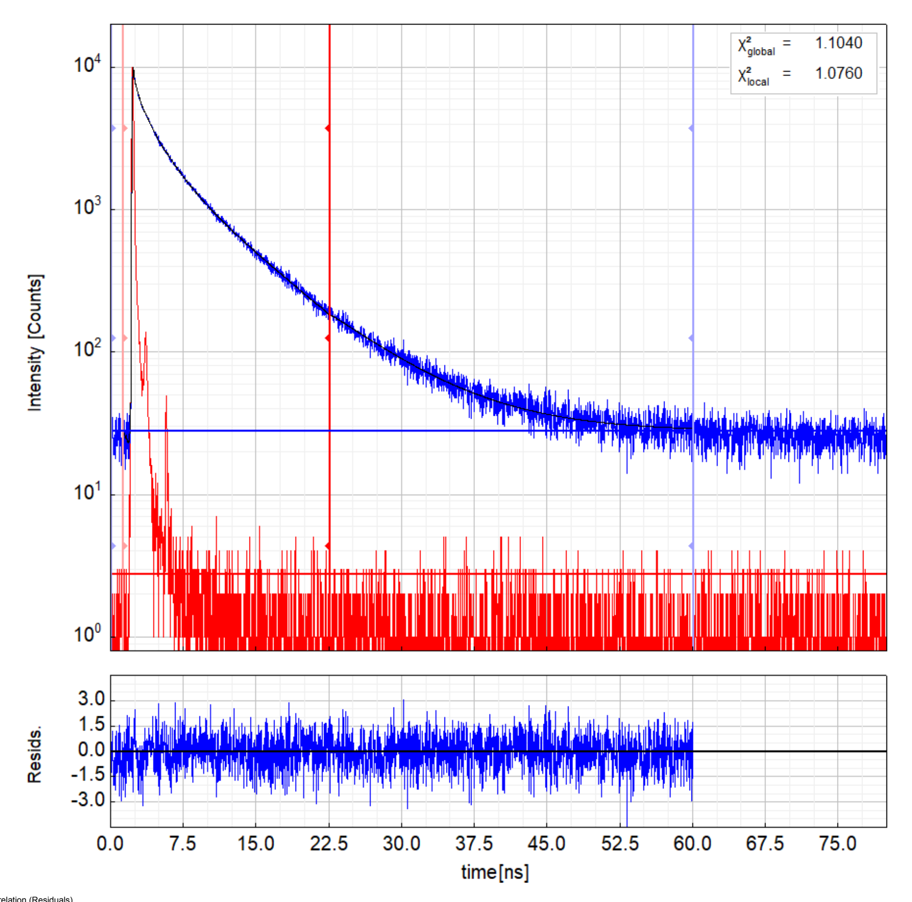

Autocorrelation (Residuals)

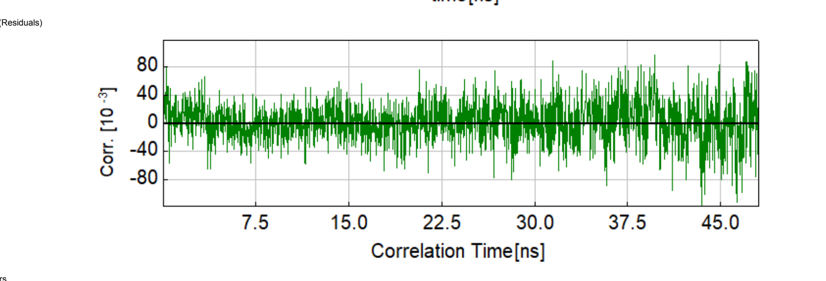

Fitted Parameters

Parameter	Value	Δ	δ
A_1[kCnts/Chnl]	2.494	±0.015	0.6%
τ_1[ns]	7.675	±0.012	0.2%
I_1[kCnts]	765.4	±2.2	0.3%
A_2[kCnts/Chnl]	4.119	±0.033	0.8%
τ_2[ns]	2.250	±0.013	0.6%
I_2[kCnts]	370.6	±2.0	0.5%
A_3[kCnts/Chnl]	3.728	±0.070	1.9%
τ_3[ns]	0.5216	±0.0058	1.1%
I_3[kCnts]	77.8	±2.2	2.8%
$Bkgr_{Dec}$[kCnts]	0.0278	±0.0003	0.9%
$Bkgr_{IRF}$[Cnts/Chnl]	2.741	±0.069	2.5%
$Shift_{IRF}$[ps]	19.98	±0.55	2.7%
A_{Scatt}[kCnts]	44.62	±0.72	1.6%
τ_{AvInt}[ns]	5.5601	±0.0074	0.1%
τ_{AvAmp}[ns]	2.935	±0.012	0.4%
λ_{Det}[nm]	650.0000	---	---

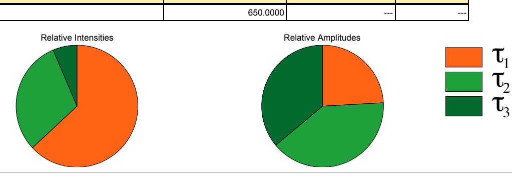

Data Set: 27 / 31

Decay: crv[27]; IRF: crv[0]

Fit

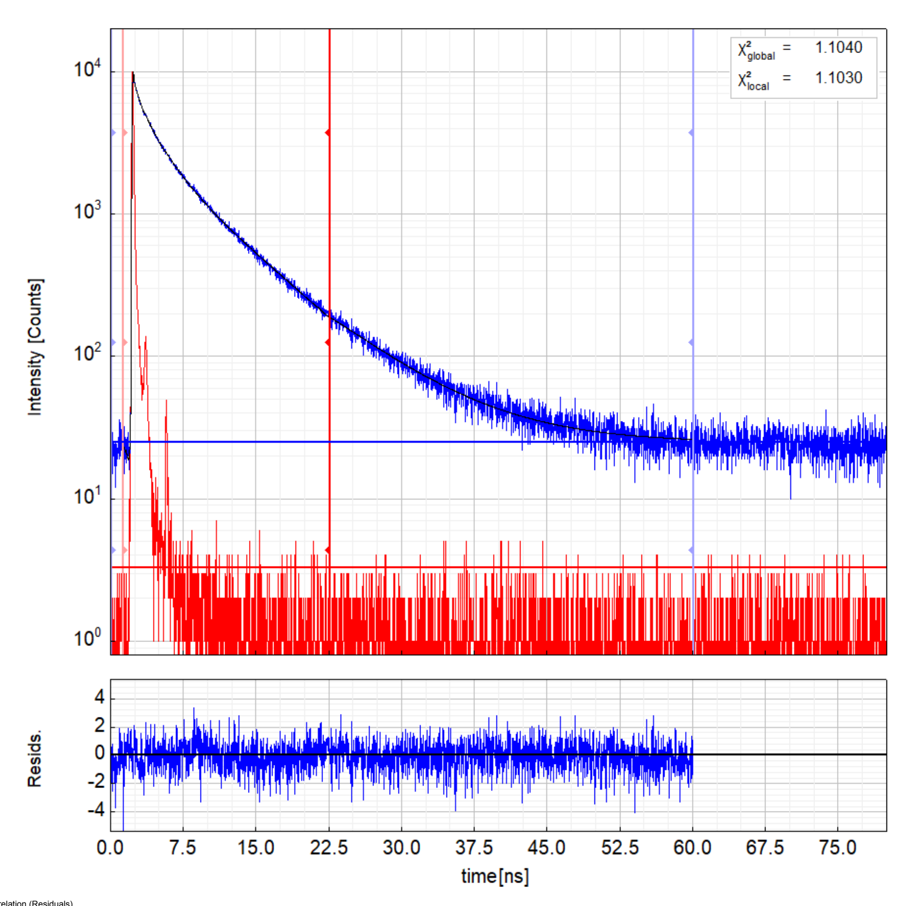

Autocorrelation (Residuals)

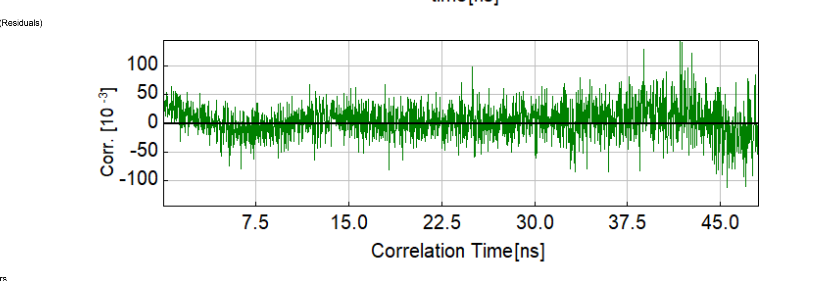

Fitted Parameters

Parameter	Value	Δ	δ
A_1[kCnts/Chnl]	2.710	±0.016	0.6%
τ_1[ns]	7.675	±0.012	0.2%
I_1[kCnts]	831.9	±3.2	0.4%
A_2[kCnts/Chnl]	4.252	±0.017	0.4%
τ_2[ns]	2.250	±0.013	0.6%
I_2[kCnts]	382.5	±2.7	0.7%
A_3[kCnts/Chnl]	3.534	±0.073	2.1%
τ_3[ns]	0.5216	±0.0058	1.1%
I_3[kCnts]	73.8	±1.1	1.4%
$Bkgr_{Dec}$[kCnts]	0.0248	±0.0002	0.5%
$Bkgr_{IRF}$[Cnts/Chnl]	3.26	±0.12	3.5%
$Shift_{IRF}$[ps]	19.70	±0.29	1.5%
A_{Scatt}[kCnts]	43.31	±0.52	1.2%
τ_{AvInt}[ns]	5.6543	±0.0095	0.2%
τ_{AvAmp}[ns]	3.069	±0.019	0.6%
λ_{Det}[nm]	660.0000	---	---

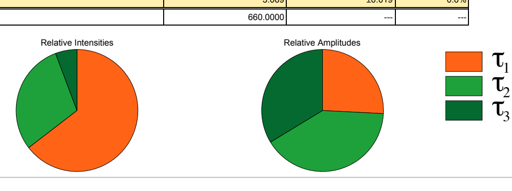

Data Set: 28 / 31

Decay: crv[28]; IRF: crv[0]

Fit

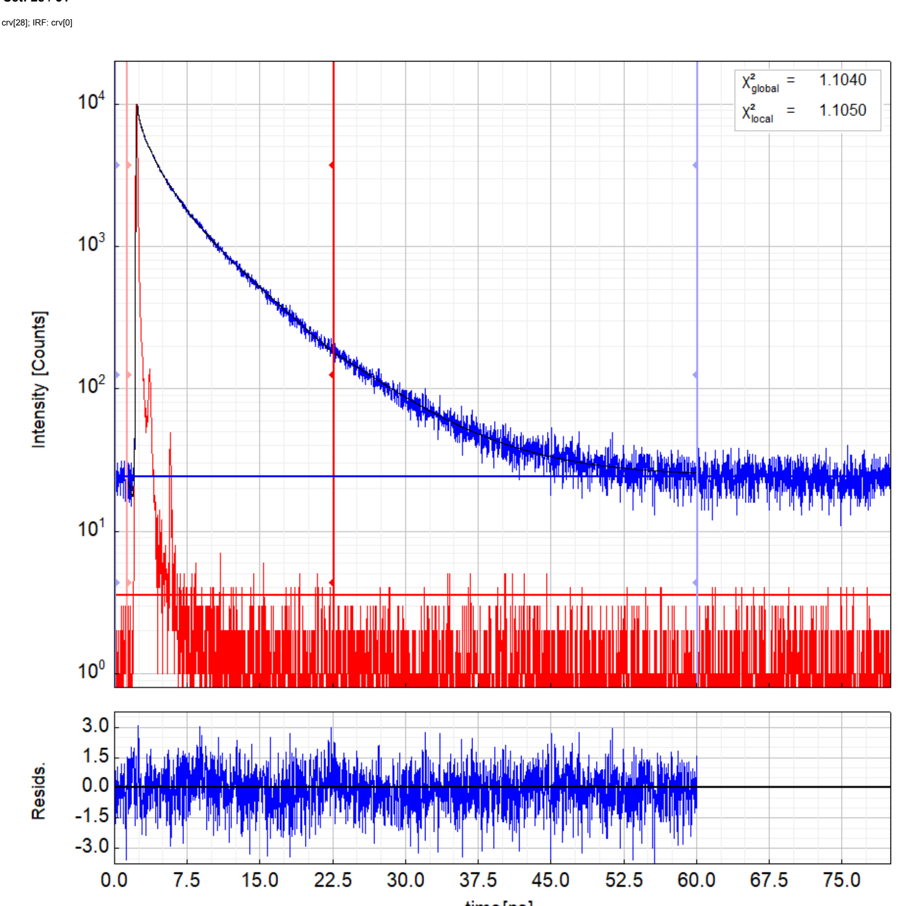

Autocorrelation (Residuals)

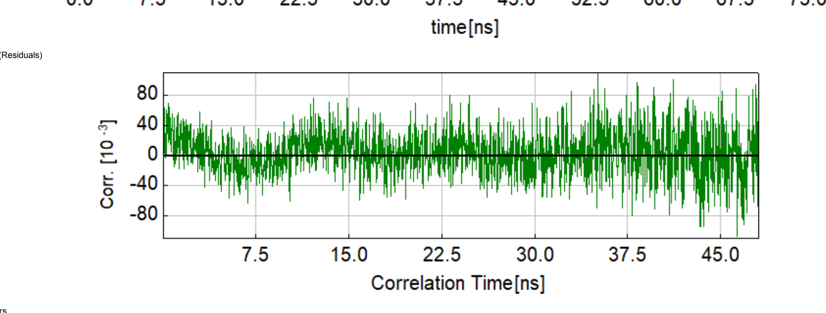

Fitted Parameters

Parameter	Value	Δ	δ
A_1[kCnts/Chnl]	2.642	±0.014	0.5%
τ_1[ns]	7.675	±0.012	0.2%
I_1[kCnts]	811.0	±2.3	0.3%
A_2[kCnts/Chnl]	4.295	±0.031	0.7%
τ_2[ns]	2.250	±0.013	0.6%
I_2[kCnts]	386.4	±3.2	0.8%
A_3[kCnts/Chnl]	3.481	±0.051	1.5%
τ_3[ns]	0.5216	±0.0058	1.1%
I_3[kCnts]	72.7	±1.6	2.1%
$Bkgr_{Dec}$[kCnts]	0.0244	±0.0002	0.7%
$Bkgr_{IRF}$[Cnts/Chnl]	3.529	±0.091	2.6%
$Shift_{IRF}$[ps]	20.57	±0.47	2.3%
A_{Scatt}[kCnts]	44.31	±0.40	0.9%
τ_{AvInt}[ns]	5.6152	±0.0041	0.1%
τ_{AvAmp}[ns]	3.048	±0.014	0.5%
λ_{Det}[nm]	670.0000	---	---

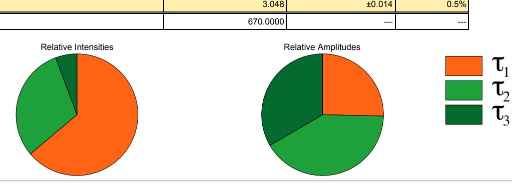

Data Set: 29 / 31

Decay: crv[29]; IRF: crv[0]

Fit

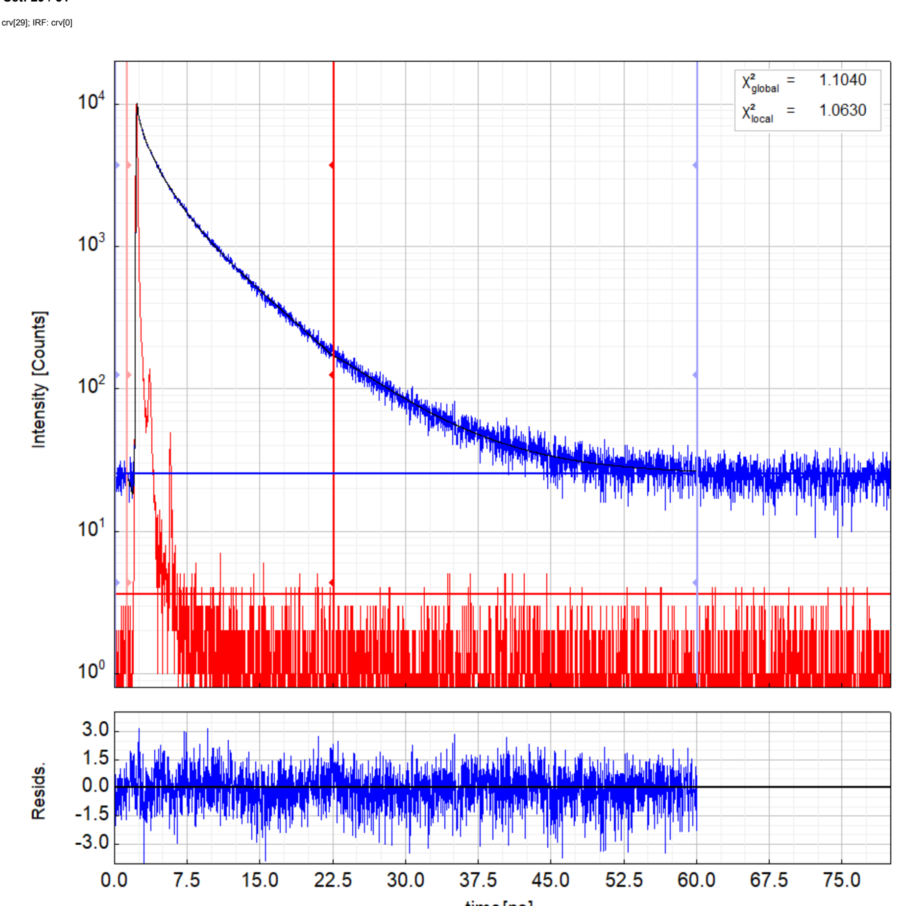

Autocorrelation (Residuals)

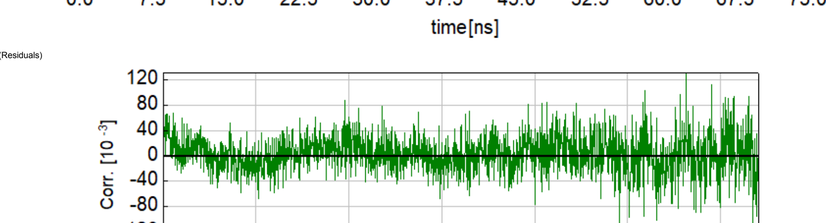

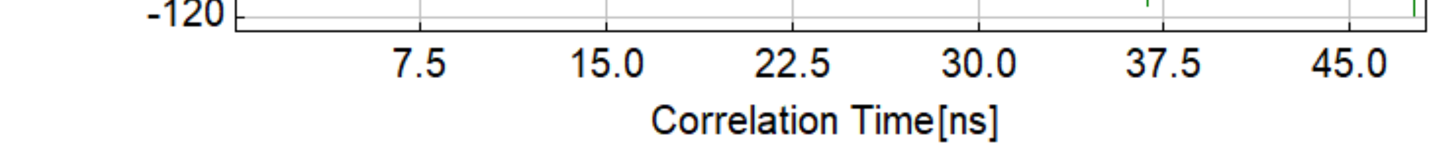

Fitted Parameters

Parameter	Value	Δ	δ
A_1[kCnts/Chnl]	2.509	±0.019	0.7%
τ_1[ns]	7.675	±0.012	0.2%
I_1[kCnts]	770.3	±5.0	0.6%
A_2[kCnts/Chnl]	4.315	±0.038	0.9%
τ_2[ns]	2.250	±0.013	0.6%
I_2[kCnts]	388.2	±3.9	1.0%
A_3[kCnts/Chnl]	3.592	±0.070	1.9%
τ_3[ns]	0.5216	±0.0058	1.1%
I_3[kCnts]	75.0	±1.2	1.5%
$Bkgr_{Dec}$[kCnts]	0.0253	±0.0002	0.5%
$Bkgr_{IRF}$[Cnts/Chnl]	3.60	±0.12	3.2%
$Shift_{IRF}$[ps]	20.68	±0.46	2.2%
A_{Scatt}[kCnts]	44.66	±0.52	1.2%
τ_{AvInt}[ns]	5.533	±0.011	0.2%
τ_{AvAmp}[ns]	2.961	±0.017	0.5%
λ_{Det}[nm]	680.0000	---	---

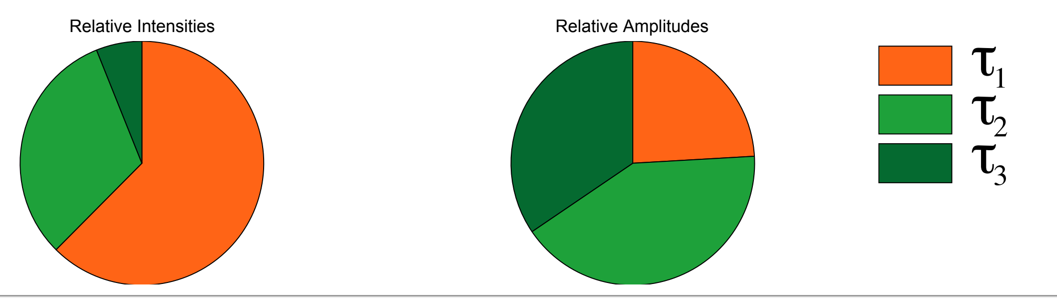

Data Set: 30 / 31

Decay: crv[30]; IRF: crv[0]

Fit

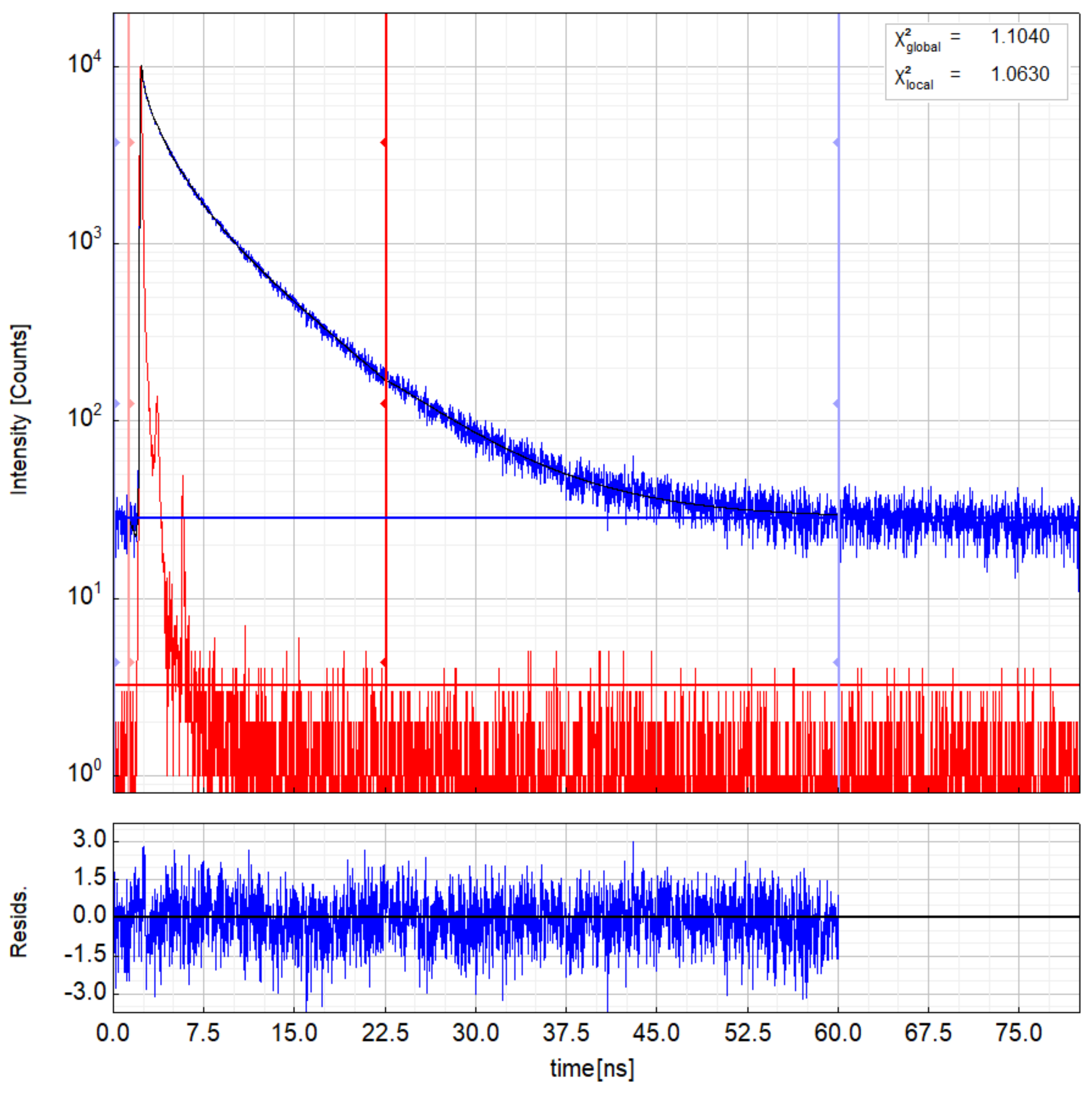

Autocorrelation (Residuals)

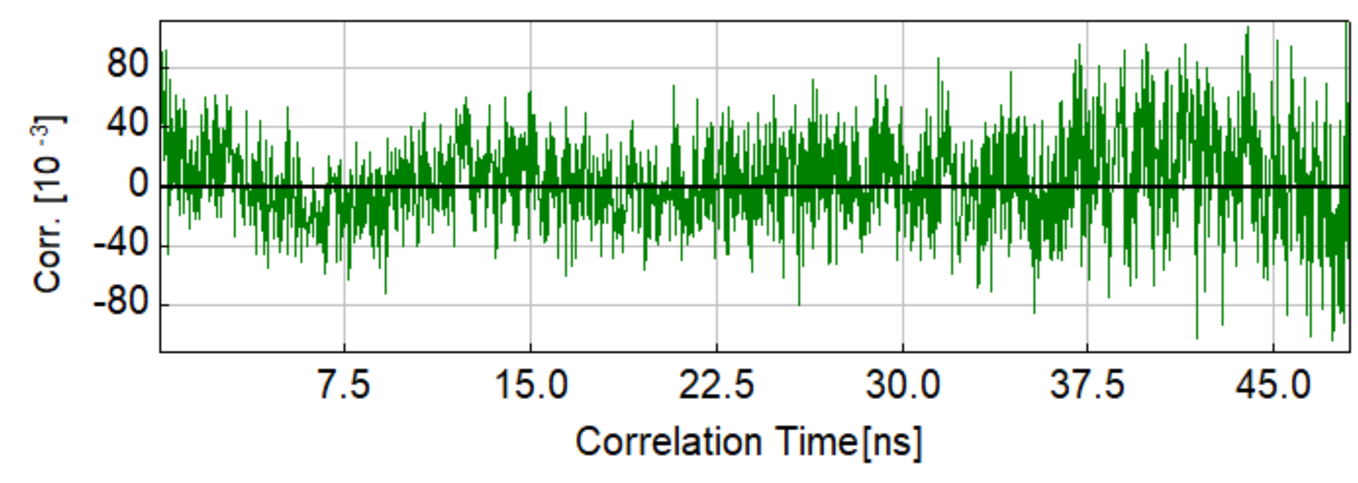

Fitted Parameters

Parameter	Value	Δ	δ
A_1[kCnts/Chnl]	2.370	±0.017	0.7%
τ_1[ns]	7.675	±0.012	0.2%
I_1[kCnts]	727.6	±4.1	0.6%
A_2[kCnts/Chnl]	4.305	±0.032	0.7%
τ_2[ns]	2.250	±0.013	0.6%
I_2[kCnts]	387.4	±2.3	0.6%
A_3[kCnts/Chnl]	3.56	±0.15	4.1%
τ_3[ns]	0.5216	±0.0058	1.1%
I_3[kCnts]	74.1	±3.8	5.1%
$Bkgr_{Dec}$[kCnts]	0.0285	±0.0002	0.6%
$Bkgr_{IRF}$[Cnts/Chnl]	3.248	±0.097	3.0%
$Shift_{IRF}$[ps]	21.51	±0.35	1.6%
A_{Scatt}[kCnts]	45.55	±0.51	1.1%
τ_{AvInt}[ns]	5.4618	±0.0084	0.2%
τ_{AvAmp}[ns]	2.907	±0.025	0.9%
λ_{Det}[nm]	690.0000	---	---

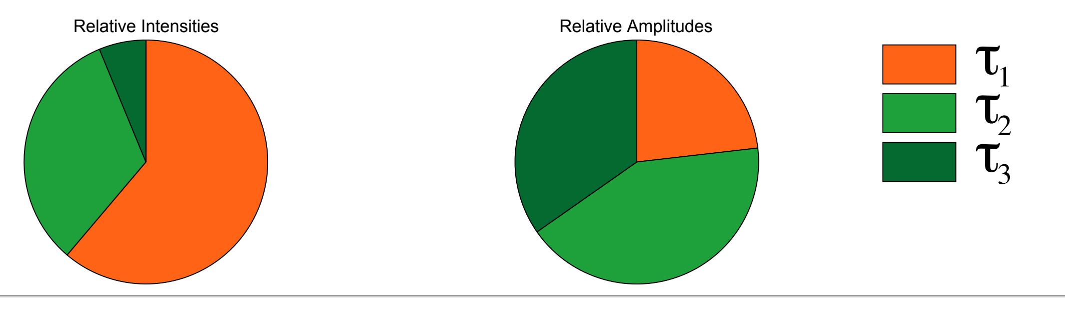

Data Set: 31 / 31

Decay: crv[31]; IRF: crv[0]

Fit

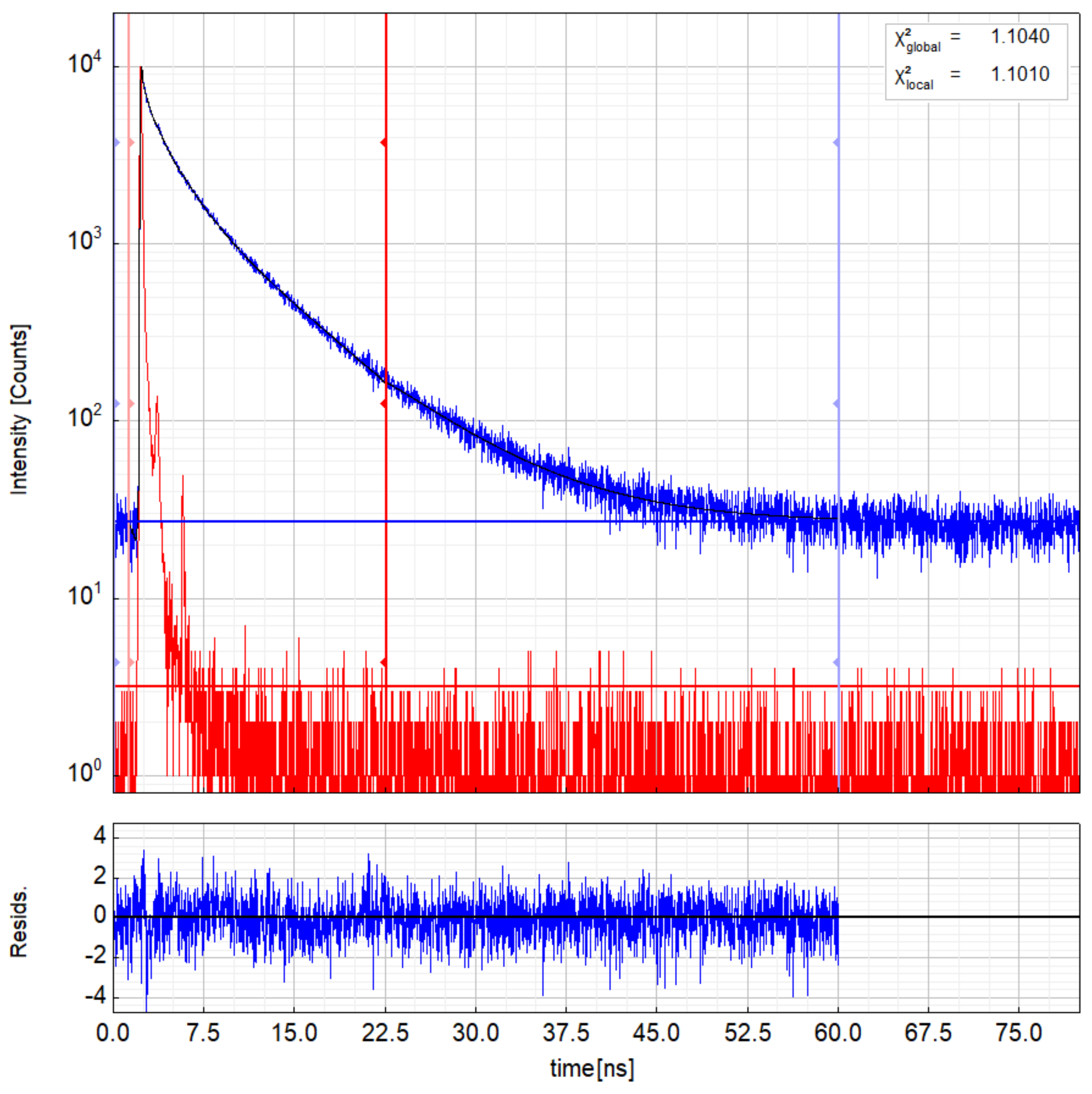

Autocorrelation (Residuals)

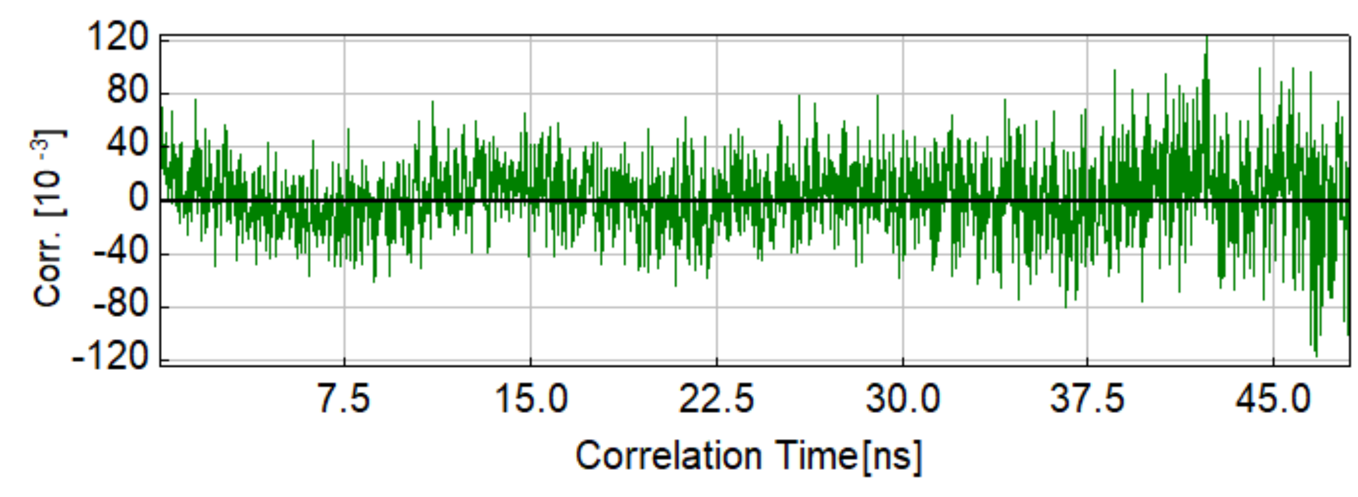

Fitted Parameters

Parameter	Value	Δ	δ
A_1[kCnts/Chnl]	2.300	±0.017	0.7%
τ_1[ns]	7.675	±0.012	0.2%
I_1[kCnts]	706.0	±3.5	0.5%
A_2[kCnts/Chnl]	4.263	±0.038	0.9%
τ_2[ns]	2.250	±0.013	0.6%
I_2[kCnts]	383.5	±3.6	0.9%
A_3[kCnts/Chnl]	3.555	±0.083	2.3%
τ_3[ns]	0.5216	±0.0058	1.1%
I_3[kCnts]	74.2	±2.6	3.5%
$Bkgr_{Dec}$[kCnts]	0.0271	±0.0002	0.7%
$Bkgr_{IRF}$[Cnts/Chnl]	3.16	±0.17	5.2%
$Shift_{IRF}$[ps]	21.83	±0.64	2.9%
A_{Scatt}[kCnts]	45.69	±0.76	1.7%
τ_{AvInt}[ns]	5.431	±0.017	0.3%
τ_{AvAmp}[ns]	2.876	±0.018	0.6%
λ_{Det}[nm]	700.0000	---	---

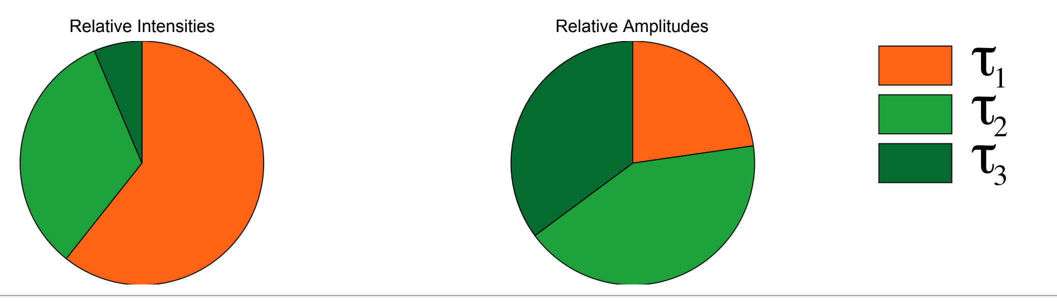

Parameter Plot

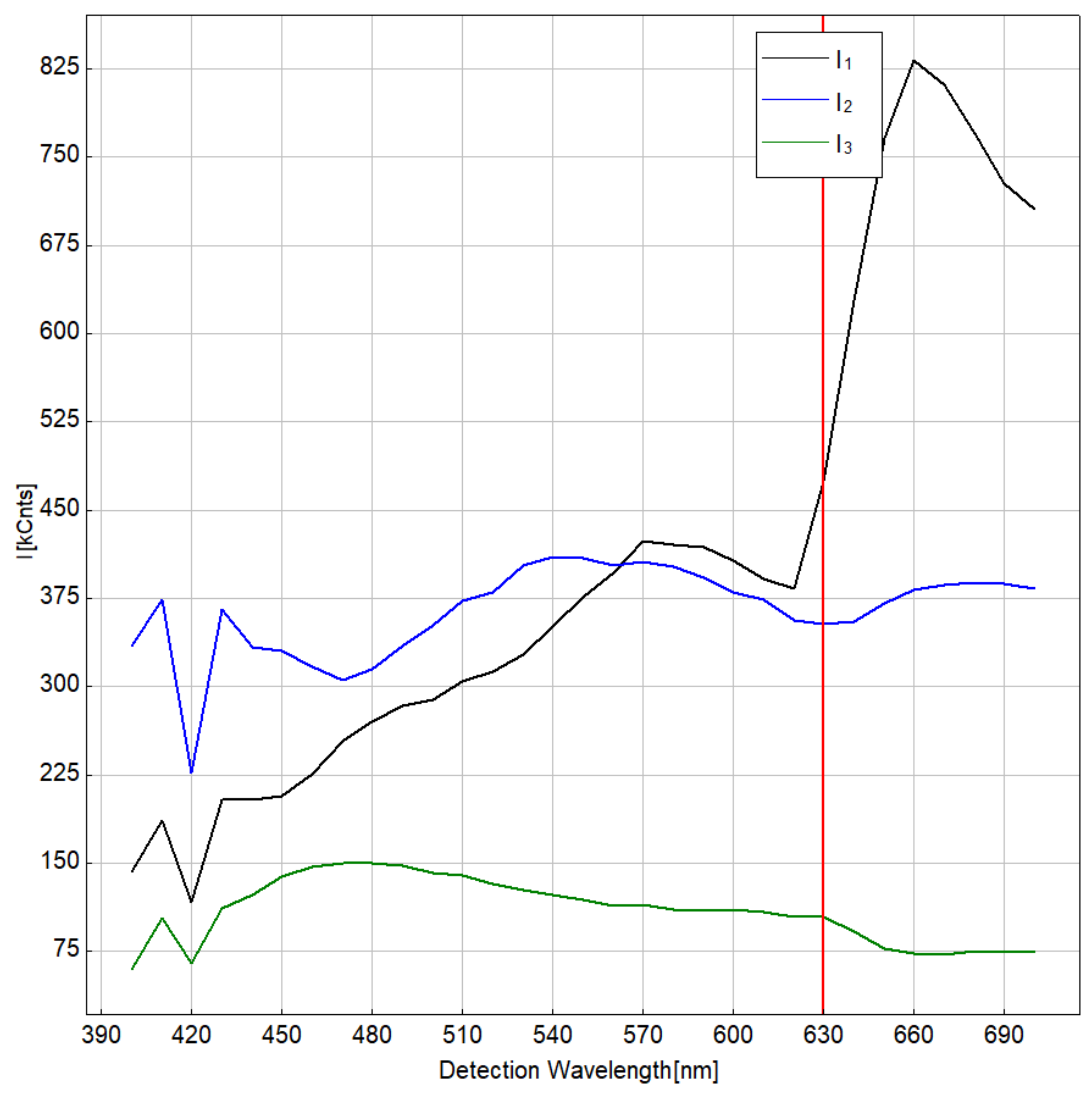

Global Parameters

Parameter	Value	Δ	δ
τ_1[ns]	7.675	±0.012	0.2%
τ_2[ns]	2.250	±0.013	0.6%
τ_3[ns]	0.5216	±0.0058	1.1%

EasyTau Report

3/11/2022 7:01:40 PM
F:\Paper Xiongfei\CD-1 DMSO tau global.html

Container Curves

TRES+IRF_20201209_1730.etc: crv[0 - 28]

Container Properties

Measurement Context: Time Resolved Emission Scan
Detection Wavelength: 450.0 - 720.0 (Step: 10.0) nm
Summary:

Sample:	CD_X_1 tau sustractive
Solvent:	Dimethyl sulfoxide
Excitation:	V pol 376±1nm with LDH-P-C-375
Detection:	M pol 450...720nm 10nm steps<br>10000 peak counts<br>grating 1200/500-<br>detector UV-red [PMT]

Comment

Model: Exponential [Reconv.]

$$\mathrm{Dec}(t)=\left[\int_{-\infty}^{t} dt'\,[\mathrm{IRF}(t-Shift_{IRF})-Bkgr_{IRF}]\left[\sum_{i=1}^{n_{Exp}} A_i\, e^{-\frac{t-t'}{\tau_i}}+A_{Scatt}\,\delta(t-t')\right]\right]+Bkgr_{Dec}$$

$$I_m = A_m \tau_m$$

$$A_{Rel\,m} = A_m \Big/ \sum_{\substack{i=1\\A_i>0}}^{n_{Exp}} A_i \qquad I_{Rel\,m} = I_m \Big/ \sum_{\substack{i=1\\I_i>0}}^{n_{Exp}} I_i$$

$$\tau_{Av\,Amp} = \sum_{\substack{i=1\\A_i>0}}^{n_{Exp}} A_i \tau_i \Big/ \sum_{\substack{i=1\\A_i>0}}^{n_{Exp}} A_i \qquad \tau_{Av\,Int} = \sum_{\substack{i=1\\I_i>0}}^{n_{Exp}} I_i \tau_i \Big/ \sum_{\substack{i=1\\I_i>0}}^{n_{Exp}} I_i$$

Data Set: 1 / 28

Decay: crv[1]; IRF: crv[0]

Fit

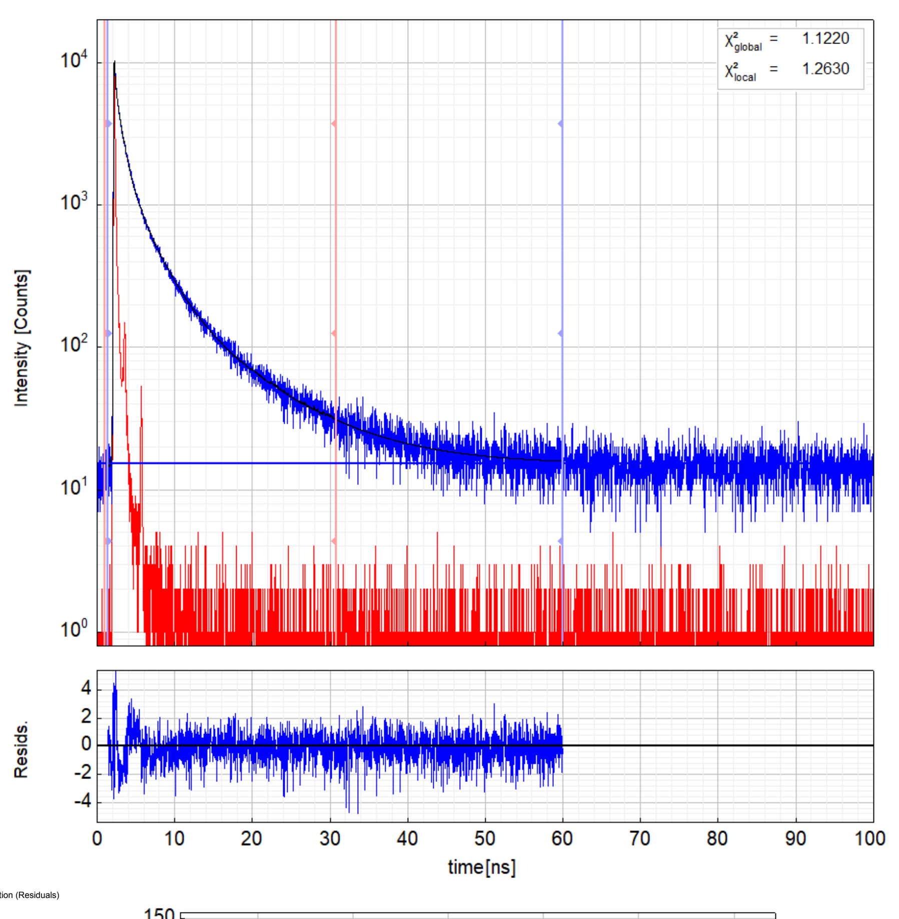

Autocorrelation (Residuals)

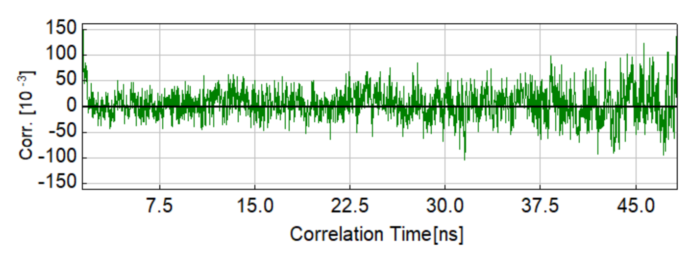

Fitted Parameters

Parameter	Value	Δ	δ
A_1[kCnts/Chnl]	0.3037	±0.0057	1.8%
τ_1[ns]	9.558	±0.017	0.2%
I_1[kCnts]	116.1	±2.2	1.8%
A_2[kCnts/Chnl]	6.233	±0.074	1.2%
τ_2[ns]	0.733	±0.012	1.6%
I_2[kCnts]	182.6	±3.8	2.1%
A_3[kCnts/Chnl]	1.967	±0.035	1.7%
τ_3[ns]	2.912	±0.033	1.1%
I_3[kCnts]	229.1	±3.6	1.6%
$Bkgr_{Dec}$[kCnts]	0.0152	±0.0002	1.1%
$Bkgr_{IRF}$[Cnts/Chnl]	0.565	±0.100	18%
$Shift_{IRF}$[ps]	11.1	±1.7	15%
A_{Scatt}[kCnts]	58.0	±2.3	3.9%
τ_{AvInt}[ns]	3.620	±0.013	0.3%
τ_{AvAmp}[ns]	1.552	±0.013	0.8%
λ_{Det}[nm]	450.0000	---	---

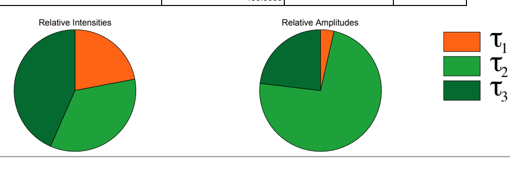

Data Set: 2 / 28

Decay: crv[2]; IRF: crv[0]

Fit

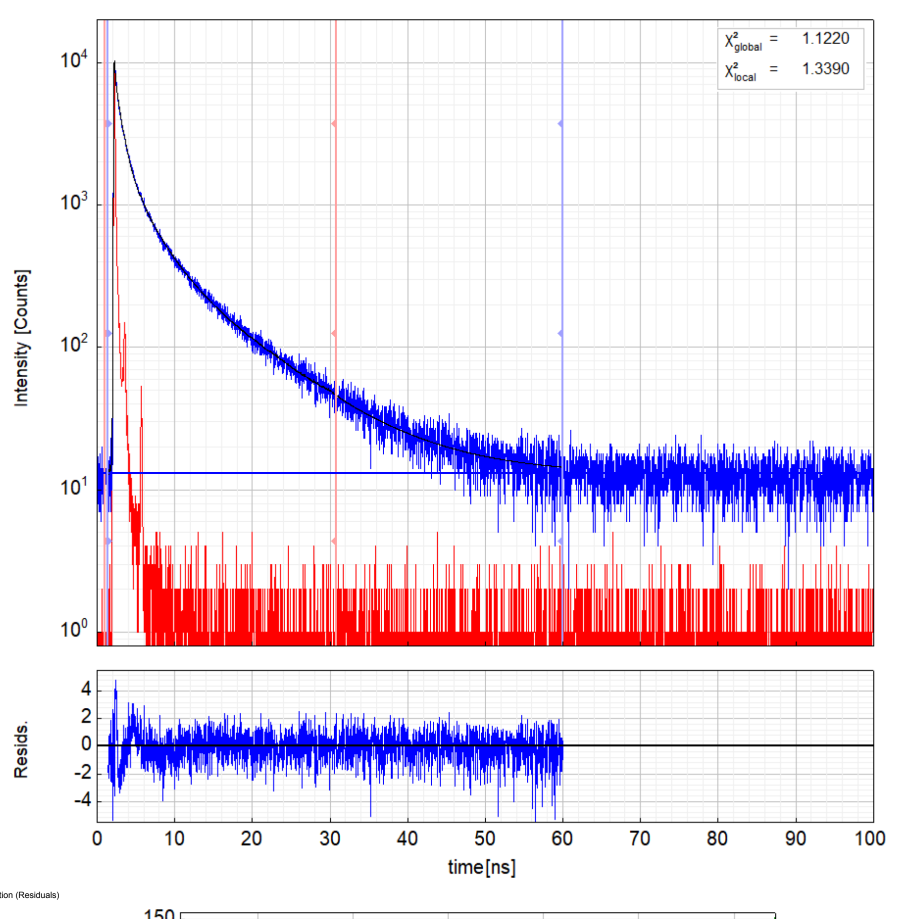

Autocorrelation (Residuals)

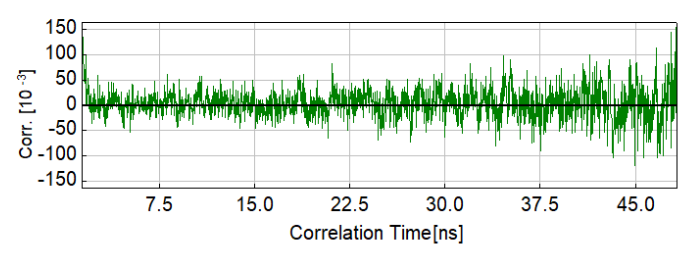

Fitted Parameters

Parameter	Value	Δ	δ
A_1[kCnts/Chnl]	0.5989	±0.0062	1.0%
τ_1[ns]	9.558	±0.017	0.2%
I_1[kCnts]	229.0	±2.2	0.9%
A_2[kCnts/Chnl]	6.79	±0.14	2.1%
τ_2[ns]	0.733	±0.012	1.6%
I_2[kCnts]	198.8	±3.9	2.0%
A_3[kCnts/Chnl]	1.918	±0.036	1.8%
τ_3[ns]	2.912	±0.033	1.1%
I_3[kCnts]	223.4	±2.9	1.3%
$Bkgr_{Dec}$[kCnts]	0.0130	±0.0002	1.5%
$Bkgr_{IRF}$[Cnts/Chnl]	0.249	±0.063	25%
$Shift_{IRF}$[ps]	9.6	±1.2	12%
A_{Scatt}[kCnts]	54.4	±1.4	2.5%
τ_{AvInt}[ns]	4.5830	±0.0097	0.2%
τ_{AvAmp}[ns]	1.750	±0.027	1.5%
λ_{Det}[nm]	460.0000	---	---

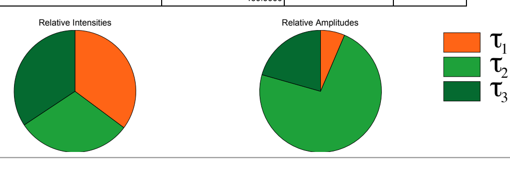

Data Set: 3 / 28

Decay: crv[3]; IRF: crv[0]

Fit

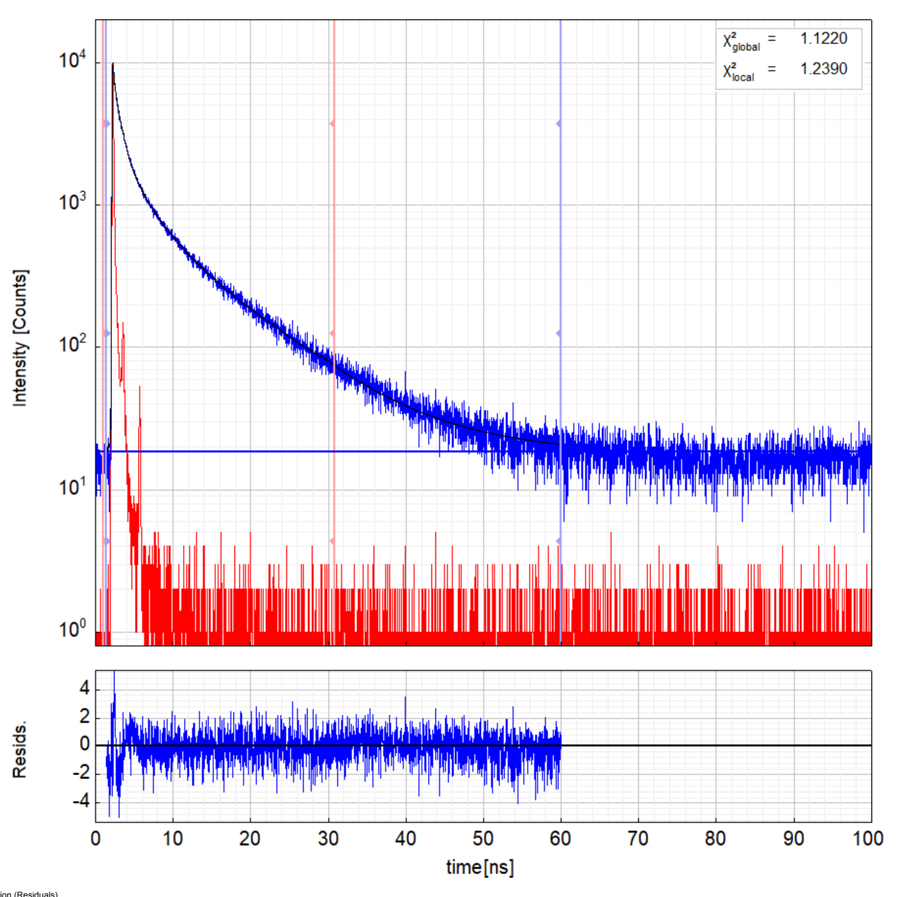

Autocorrelation (Residuals)

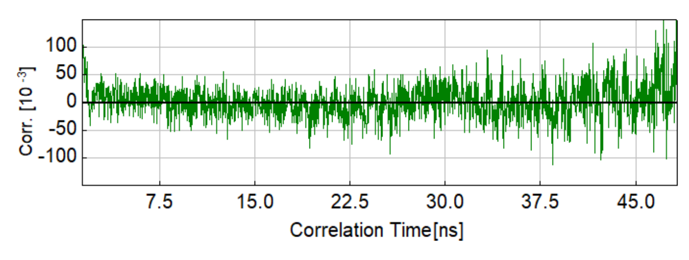

Fitted Parameters

Parameter	Value	Δ	δ
A_1[kCnts/Chnl]	0.9980	±0.0069	0.7%
τ_1[ns]	9.558	±0.017	0.2%
I_1[kCnts]	381.6	±2.5	0.6%
A_2[kCnts/Chnl]	6.530	±0.080	1.2%
τ_2[ns]	0.733	±0.012	1.6%
I_2[kCnts]	191.3	±3.1	1.6%
A_3[kCnts/Chnl]	1.812	±0.040	2.2%
τ_3[ns]	2.912	±0.033	1.1%
I_3[kCnts]	211.0	±2.7	1.2%
$Bkgr_{Dec}$[kCnts]	0.0184	±0.0002	0.6%
$Bkgr_{IRF}$[Cnts/Chnl]	-0.056	±0.088	156%
$Shift_{IRF}$[ps]	10.67	±0.66	6.1%
A_{Scatt}[kCnts]	51.4	±1.2	2.3%
τ_{AvInt}[ns]	5.616	±0.018	0.3%
τ_{AvAmp}[ns]	2.098	±0.024	1.1%
λ_{Det}[nm]	470.0000	---	---

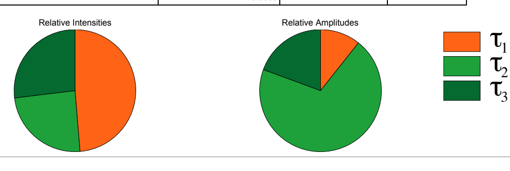

Data Set: 4 / 28

Decay: crv[4]; IRF: crv[0]

Fit

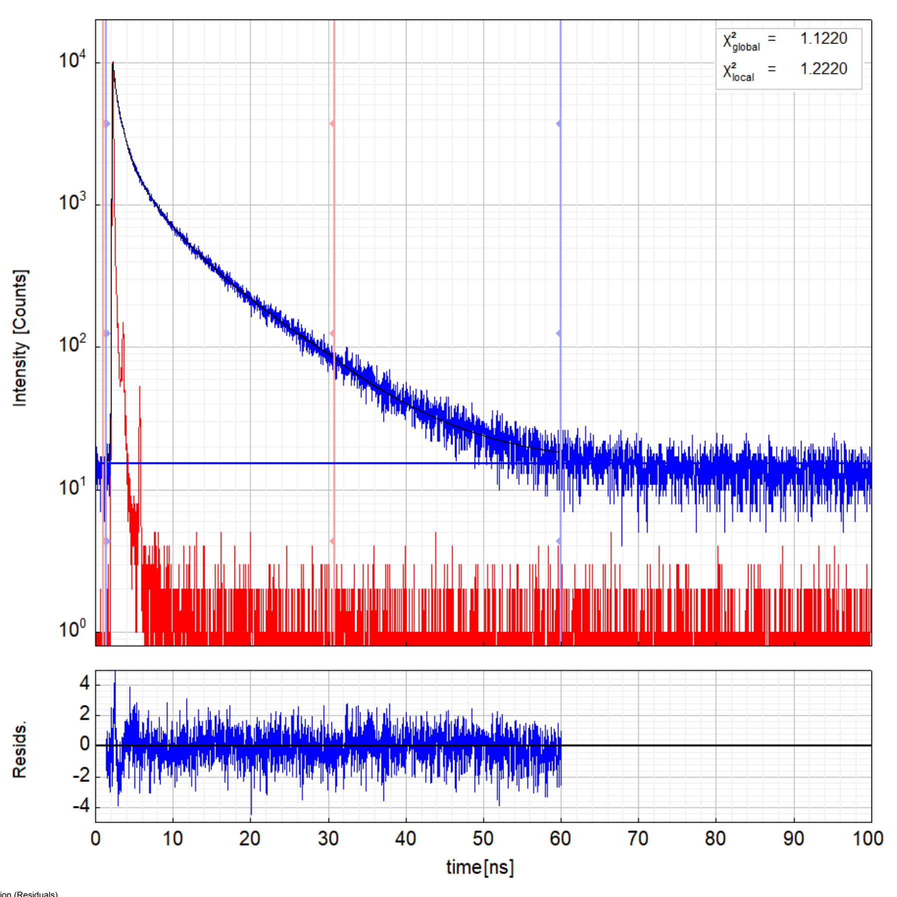

Autocorrelation (Residuals)

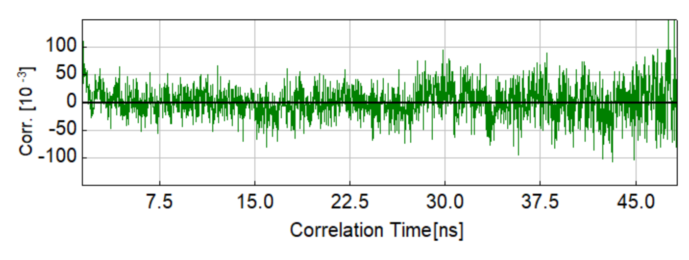

Fitted Parameters

Parameter	Value	Δ	δ
A_1[kCnts/Chnl]	1.2240	±0.0085	0.7%
τ_1[ns]	9.558	±0.017	0.2%
I_1[kCnts]	468.0	±2.8	0.6%
A_2[kCnts/Chnl]	6.63	±0.13	2.0%
τ_2[ns]	0.733	±0.012	1.6%
I_2[kCnts]	194.1	±3.1	1.6%
A_3[kCnts/Chnl]	1.933	±0.034	1.8%
τ_3[ns]	2.912	±0.033	1.1%
I_3[kCnts]	225.1	±3.4	1.5%
$Bkgr_{Dec}$[kCnts]	0.0152	±0.0004	2.0%
$Bkgr_{IRF}$[Cnts/Chnl]	-0.055	±0.050	90%
$Shift_{IRF}$[ps]	10.02	±0.69	6.9%
A_{Scatt}[kCnts]	50.11	±0.84	1.7%
τ_{AvInt}[ns]	5.941	±0.017	0.3%
τ_{AvAmp}[ns]	2.268	±0.025	1.1%
λ_{Det}[nm]	480.0000	---	---

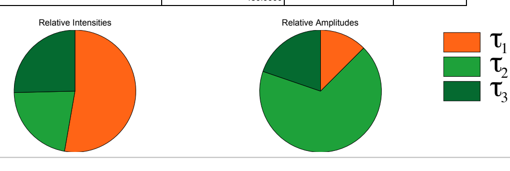

Data Set: 5 / 28

Decay: crv[5]; IRF: crv[0]

Fit

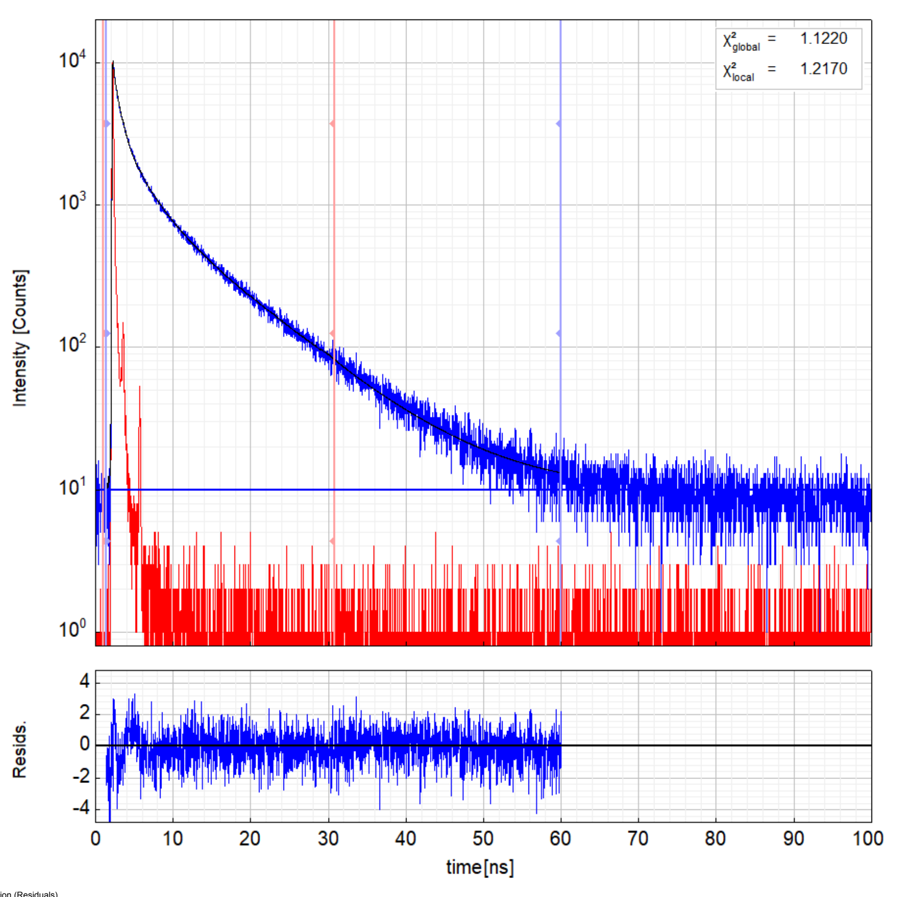

Autocorrelation (Residuals)

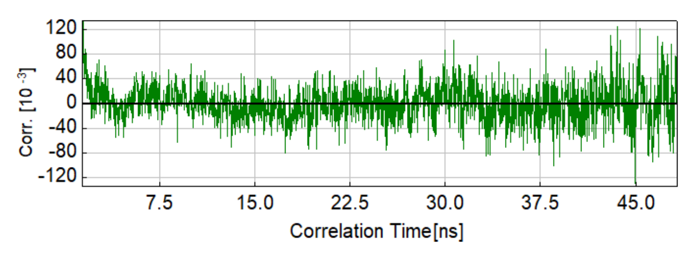

Fitted Parameters

Parameter	Value	Δ	δ
A_1[kCnts/Chnl]	1.3048	±0.0088	0.7%
τ_1[ns]	9.558	±0.017	0.2%
I_1[kCnts]	498.8	±2.6	0.5%
A_2[kCnts/Chnl]	6.720	±0.084	1.2%
τ_2[ns]	0.733	±0.012	1.6%
I_2[kCnts]	196.8	±2.6	1.3%
A_3[kCnts/Chnl]	2.375	±0.045	1.9%
τ_3[ns]	2.912	±0.033	1.1%
I_3[kCnts]	276.6	±4.2	1.5%
$Bkgr_{Dec}$[kCnts]	0.0099	±0.0002	1.6%
$Bkgr_{IRF}$[Cnts/Chnl]	0.059	±0.098	167%
$Shift_{IRF}$[ps]	8.9	±1.6	17%
A_{Scatt}[kCnts]	48.0	±1.8	3.6%
τ_{AvInt}[ns]	5.881	±0.021	0.3%
τ_{AvAmp}[ns]	2.338	±0.015	0.6%
λ_{Det}[nm]	490.0000	---	---

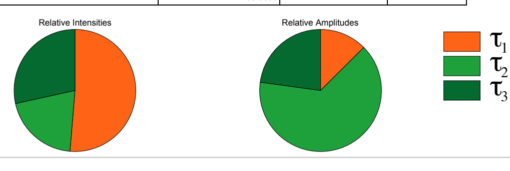

Data Set: 6 / 28

Decay: crv[6]; IRF: crv[0]

Fit

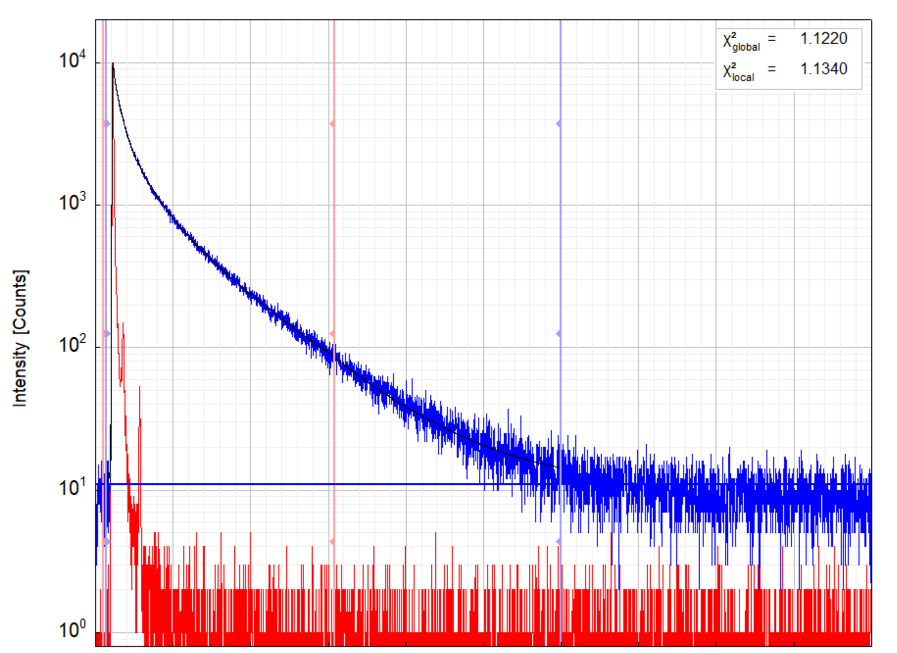

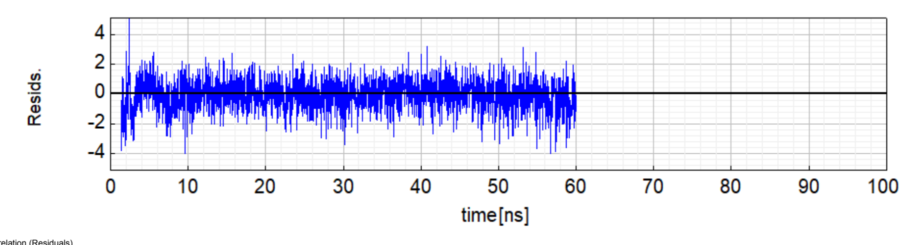

Autocorrelation (Residuals)

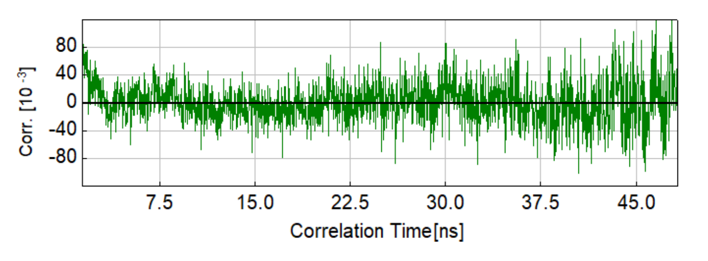

Fitted Parameters

Parameter	Value	Δ	δ
A_1[kCnts/Chnl]	1.355	±0.013	0.9%
τ_1[ns]	9.558	±0.017	0.2%
I_1[kCnts]	517.8	±4.2	0.8%
A_2[kCnts/Chnl]	6.226	±0.085	1.4%
τ_2[ns]	0.733	±0.012	1.6%
I_2[kCnts]	182.4	±3.5	1.9%
A_3[kCnts/Chnl]	2.698	±0.059	2.2%
τ_3[ns]	2.912	±0.033	1.1%
I_3[kCnts]	314.1	±4.5	1.4%
$Bkgr_{Dec}$[kCnts]	0.0110	±0.0002	1.0%
$Bkgr_{IRF}$[Cnts/Chnl]	0.114	±0.071	62%
$Shift_{IRF}$[ps]	9.58	±0.92	9.6%
A_{Scatt}[kCnts]	45.6	±1.2	2.4%
τ_{AvInt}[ns]	5.913	±0.014	0.2%
τ_{AvAmp}[ns]	2.468	±0.025	1.0%
λ_{Det}[nm]	500.0000	---	---

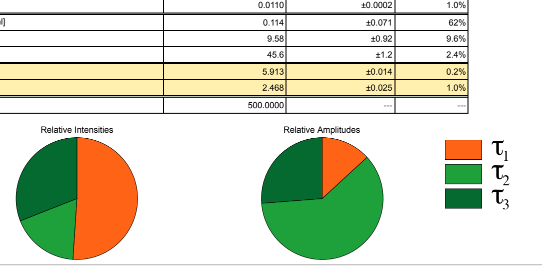

Data Set: 7 / 28

Decay: crv[7]; IRF: crv[0]

Fit

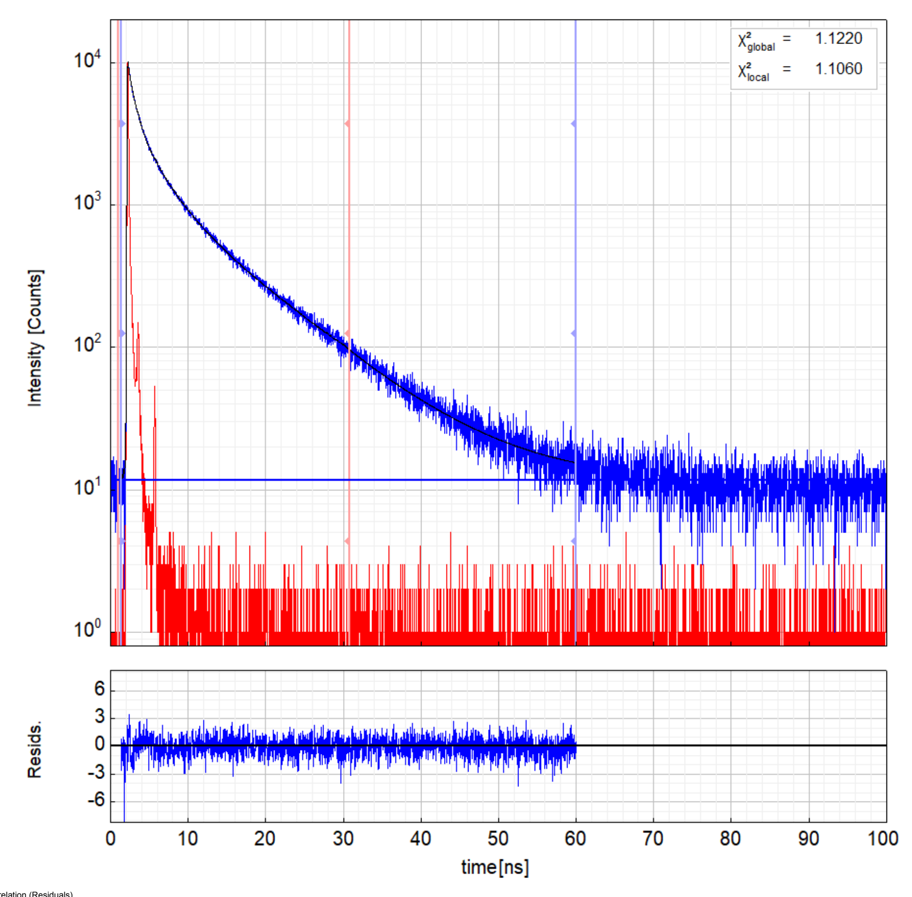

Autocorrelation (Residuals)

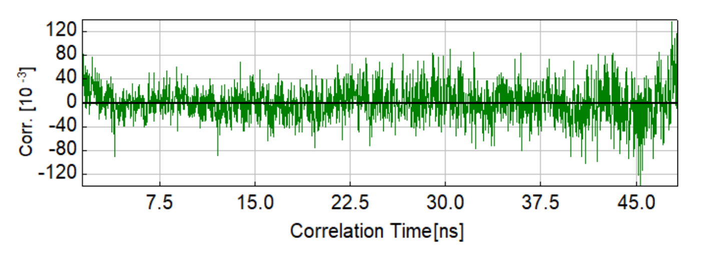

Fitted Parameters

Parameter	Value	Δ	δ
A_1[kCnts/Chnl]	1.541	±0.014	0.9%
τ_1[ns]	9.558	±0.017	0.2%
I_1[kCnts]	589.0	±4.2	0.7%
A_2[kCnts/Chnl]	6.203	±0.066	1.0%
τ_2[ns]	0.733	±0.012	1.6%
I_2[kCnts]	181.7	±3.8	2.0%
A_3[kCnts/Chnl]	3.039	±0.036	1.2%
τ_3[ns]	2.912	±0.033	1.1%
I_3[kCnts]	353.9	±2.5	0.7%
$Bkgr_{Dec}$[kCnts]	0.0117	±0.0004	2.7%
$Bkgr_{IRF}$[Cnts/Chnl]	0.10	±0.12	116%
$Shift_{IRF}$[ps]	9.39	±0.67	7.0%
A_{Scatt}[kCnts]	43.63	±0.88	2.0%
τ_{AvInt}[ns]	6.041	±0.014	0.2%
τ_{AvAmp}[ns]	2.608	±0.025	1.0%
λ_{Det}[nm]	510.0000	---	---

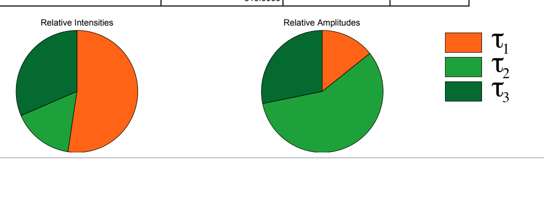

Data Set: 8 / 28

Decay: crv[8]; IRF: crv[0]

Fit

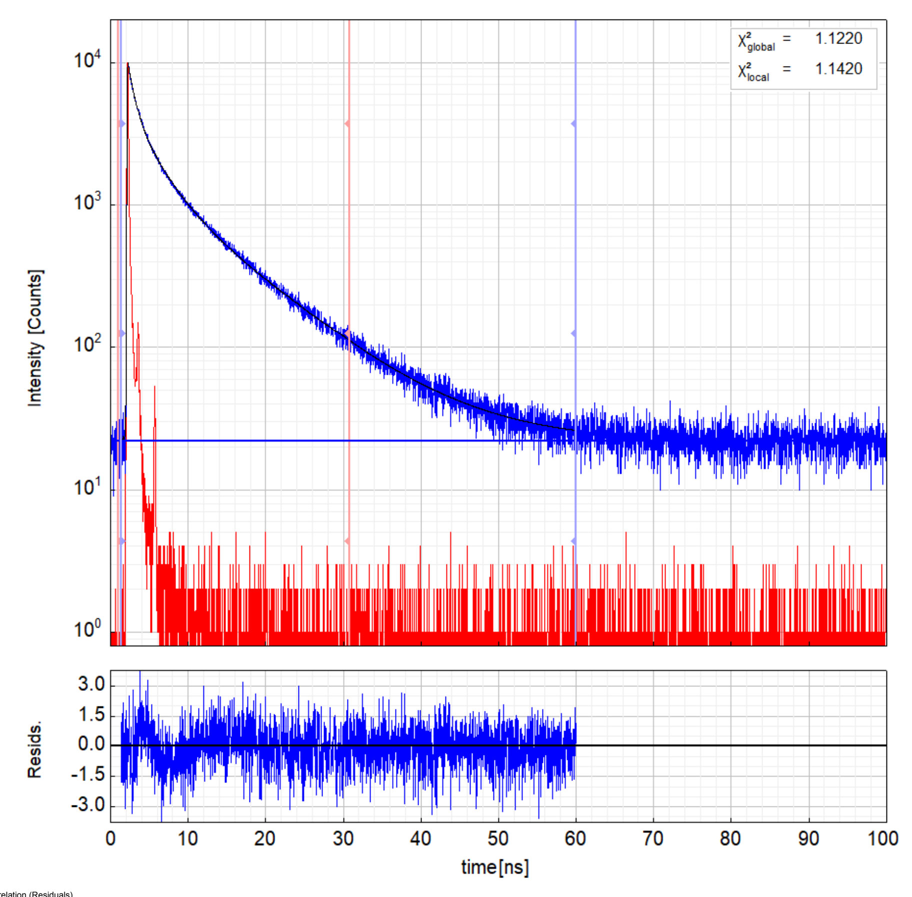

Autocorrelation (Residuals)

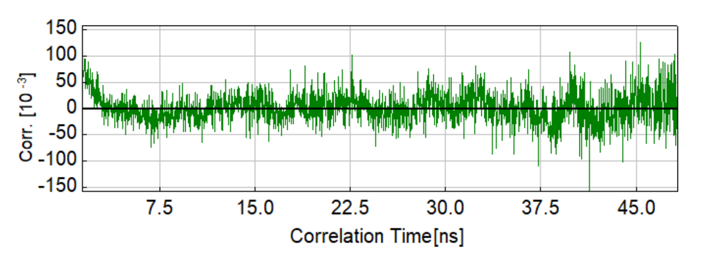

Fitted Parameters

Parameter	Value	Δ	δ
A_1[kCnts/Chnl]	1.6958	±0.0077	0.5%
τ_1[ns]	9.558	±0.017	0.2%
I_1[kCnts]	648.3	±2.6	0.4%
A_2[kCnts/Chnl]	6.111	±0.057	0.9%
τ_2[ns]	0.733	±0.012	1.6%
I_2[kCnts]	179.0	±4.7	2.6%
A_3[kCnts/Chnl]	3.267	±0.061	1.8%
τ_3[ns]	2.912	±0.033	1.1%
I_3[kCnts]	380.5	±2.7	0.7%
$Bkgr_{Dec}$[kCnts]	0.0220	±0.0002	0.8%
$Bkgr_{IRF}$[Cnts/Chnl]	0.333	±0.061	18%
$Shift_{IRF}$[ps]	7.98	±0.81	10%
A_{Scatt}[kCnts]	40.08	±0.47	1.2%
τ_{AvInt}[ns]	6.157	±0.016	0.3%
τ_{AvAmp}[ns]	2.7267	±0.0092	0.3%
λ_{Det}[nm]	520.0000	---	---

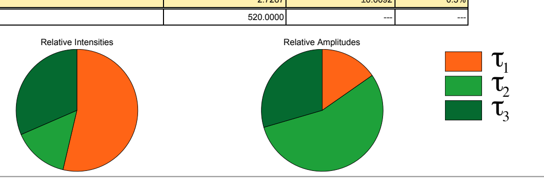

Data Set: 9 / 28

Decay: crv[9]; IRF: crv[0]

Fit

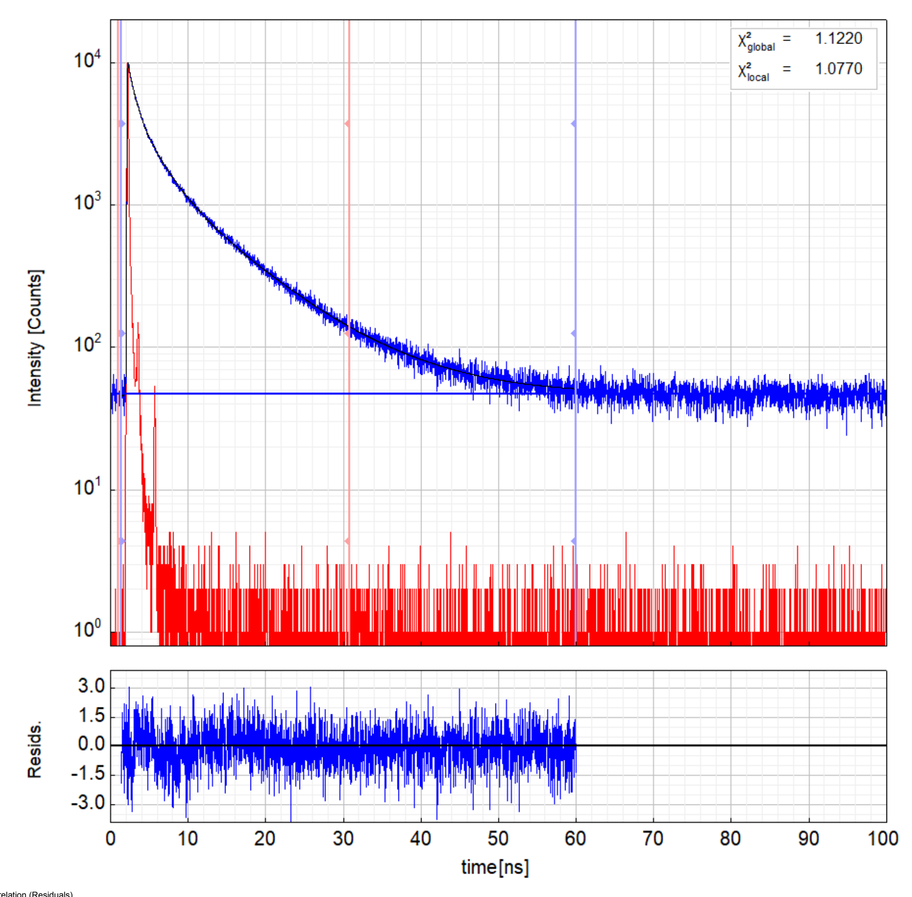

Autocorrelation (Residuals)

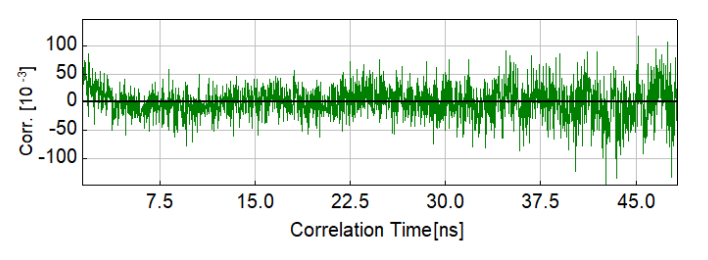

Fitted Parameters

Parameter	Value	Δ	δ
A_1[kCnts/Chnl]	1.846	±0.015	0.8%
τ_1[ns]	9.558	±0.017	0.2%
I_1[kCnts]	705.4	±5.8	0.8%
A_2[kCnts/Chnl]	5.818	±0.084	1.4%
τ_2[ns]	0.733	±0.012	1.6%
I_2[kCnts]	170.4	±2.3	1.3%
A_3[kCnts/Chnl]	3.565	±0.068	1.9%
τ_3[ns]	2.912	±0.033	1.1%
I_3[kCnts]	415.2	±4.2	1.0%
$Bkgr_{Dec}$[kCnts]	0.0468	±0.0005	0.9%
$Bkgr_{IRF}$[Cnts/Chnl]	0.731	±0.068	9.3%
$Shift_{IRF}$[ps]	7.27	±0.70	9.5%
A_{Scatt}[kCnts]	38.01	±0.73	1.9%
τ_{AvInt}[ns]	6.256	±0.014	0.2%
τ_{AvAmp}[ns]	2.875	±0.013	0.4%
λ_{Det}[nm]	530.0000	---	---

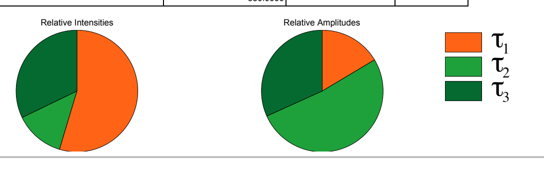

Data Set: 10 / 28

Decay: crv[10]; IRF: crv[0]

Fit

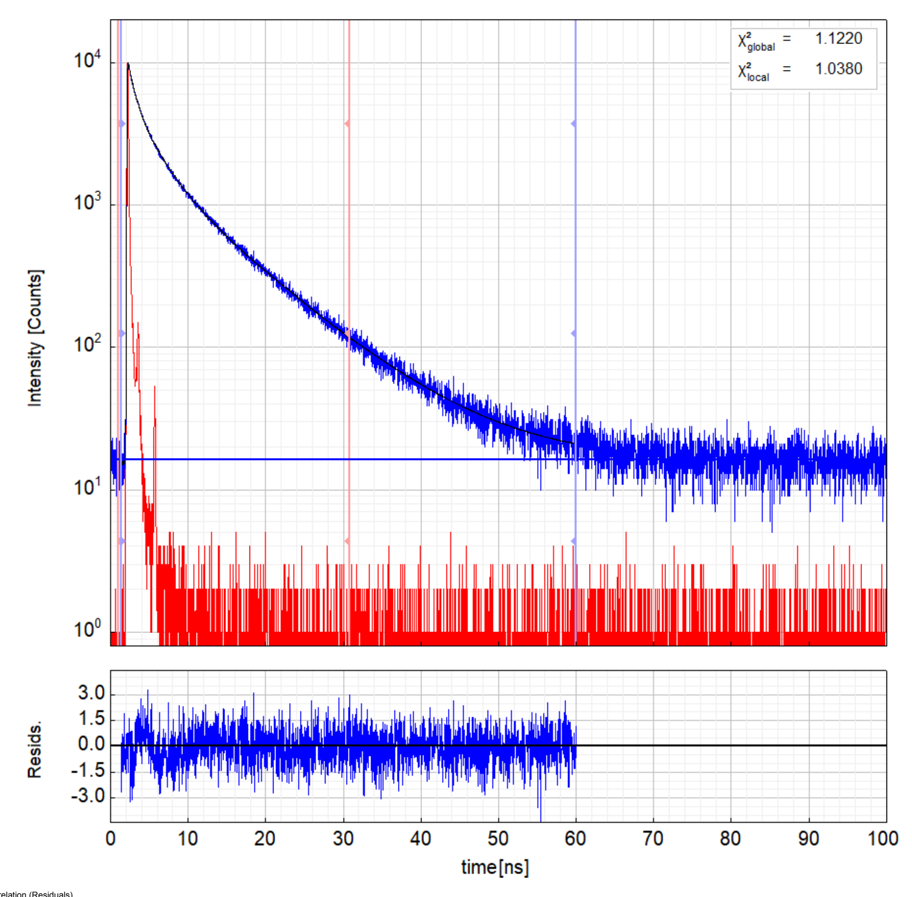

Autocorrelation (Residuals)

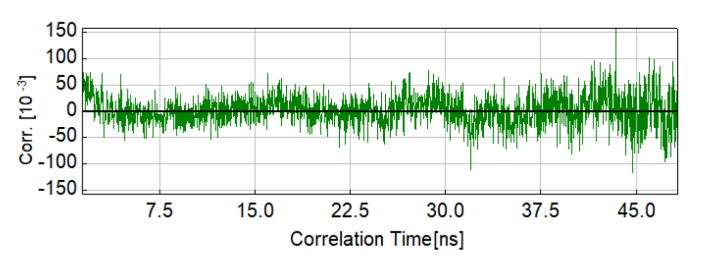

Fitted Parameters

Parameter	Value	Δ	δ
A_1[kCnts/Chnl]	2.027	±0.013	0.6%
τ_1[ns]	9.558	±0.017	0.2%
I_1[kCnts]	774.7	±3.8	0.5%
A_2[kCnts/Chnl]	5.653	±0.083	1.5%
τ_2[ns]	0.733	±0.012	1.6%
I_2[kCnts]	165.6	±4.5	2.7%
A_3[kCnts/Chnl]	3.819	±0.073	1.9%
τ_3[ns]	2.912	±0.033	1.1%
I_3[kCnts]	444.7	±4.6	1.0%
$Bkgr_{Dec}$[kCnts]	0.0163	±0.0002	1.0%
$Bkgr_{IRF}$[Cnts/Chnl]	0.787	±0.072	9.0%
$Shift_{IRF}$[ps]	6.96	±0.56	7.9%
A_{Scatt}[kCnts]	36.69	±0.75	2.0%
τ_{AvInt}[ns]	6.369	±0.014	0.2%
τ_{AvAmp}[ns]	3.012	±0.023	0.8%
λ_{Det}[nm]	540.0000	---	---

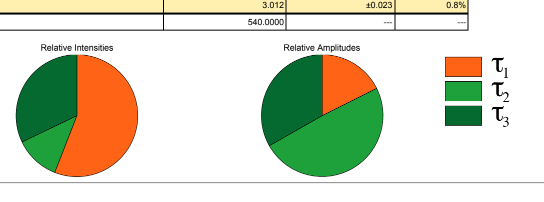

Data Set: 11 / 28

Decay: crv[11]; IRF: crv[0]

Fit

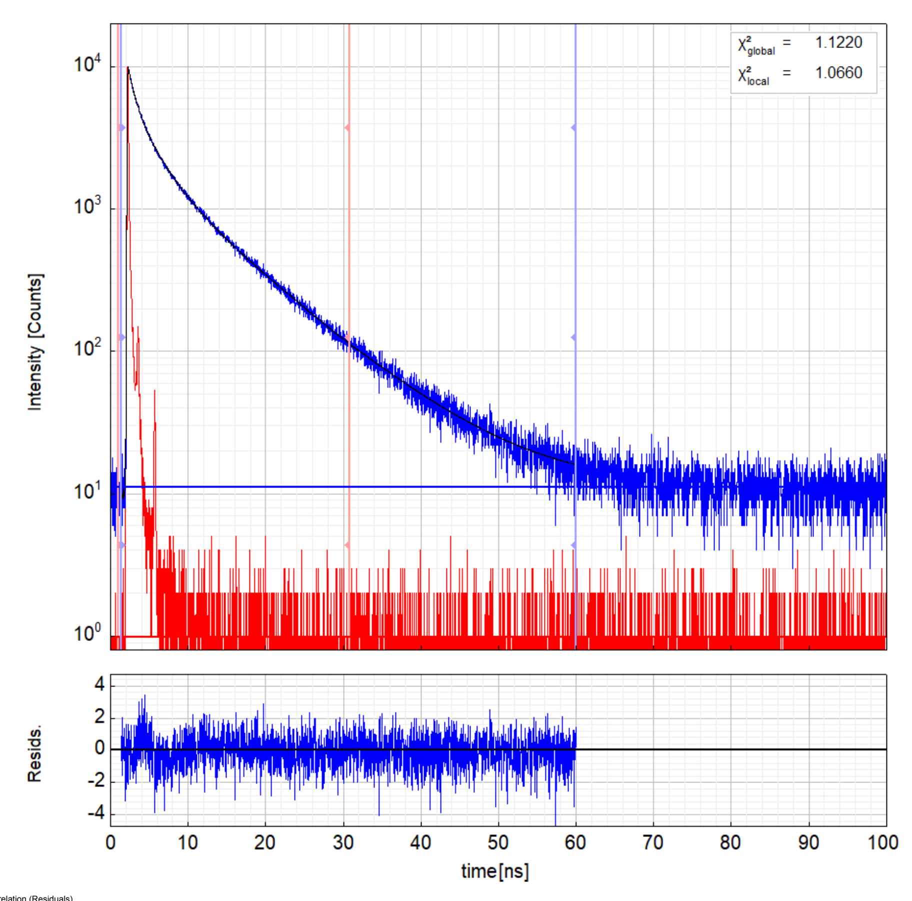

Autocorrelation (Residuals)

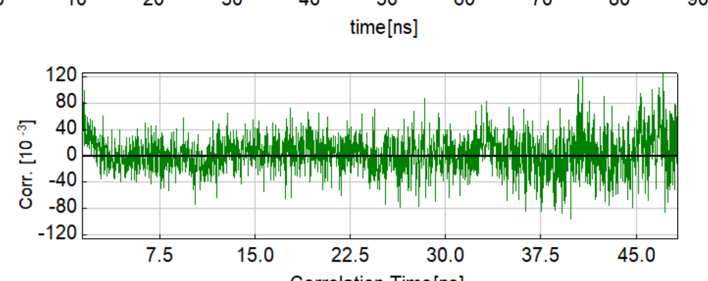

Fitted Parameters

Parameter	Value	Δ	δ
A_1[kCnts/Chnl]	2.0963	±0.0087	0.4%
τ_1[ns]	9.558	±0.017	0.2%
I_1[kCnts]	801.4	±2.8	0.3%
A_2[kCnts/Chnl]	5.522	±0.053	0.9%
τ_2[ns]	0.733	±0.012	1.6%
I_2[kCnts]	161.7	±4.0	2.5%
A_3[kCnts/Chnl]	3.849	±0.061	1.6%
τ_3[ns]	2.912	±0.033	1.1%
I_3[kCnts]	448.2	±4.3	1.0%
$Bkgr_{Dec}$[kCnts]	0.0112	±0.0002	1.3%
$Bkgr_{IRF}$[Cnts/Chnl]	0.990	±0.062	6.2%
$Shift_{IRF}$[ps]	8.96	±0.93	10%
A_{Scatt}[kCnts]	36.38	±0.77	2.1%
τ_{AvInt}[ns]	6.4356	±0.0077	0.1%
τ_{AvAmp}[ns]	3.0770	±0.0083	0.3%
λ_{Det}[nm]	550.0000	---	---

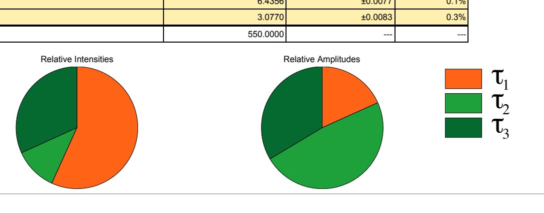

Data Set: 12 / 28

Decay: crv[12]; IRF: crv[0]

Fit

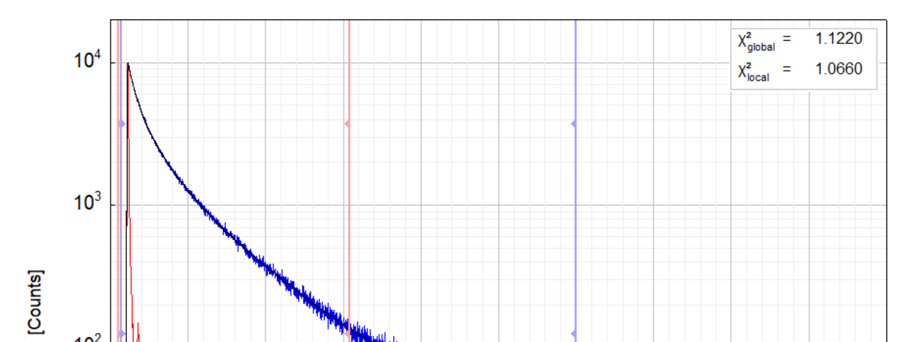

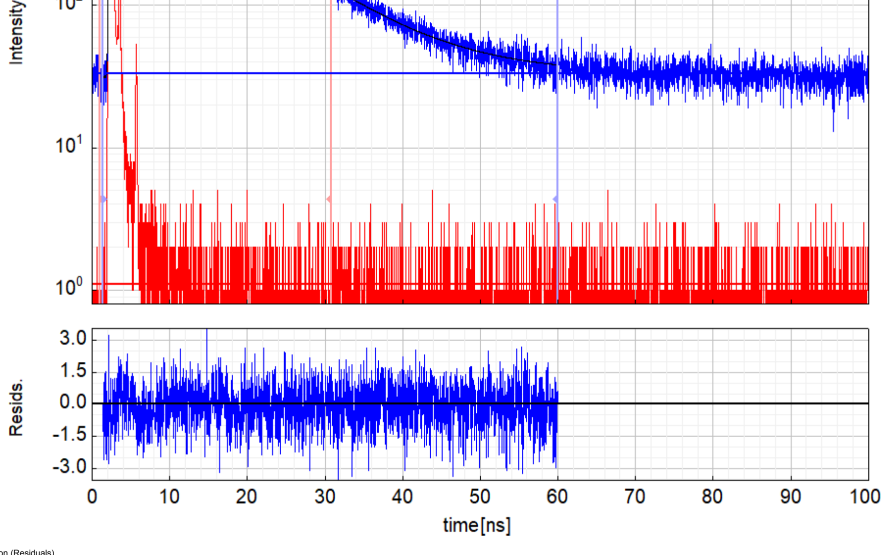

Autocorrelation (Residuals)

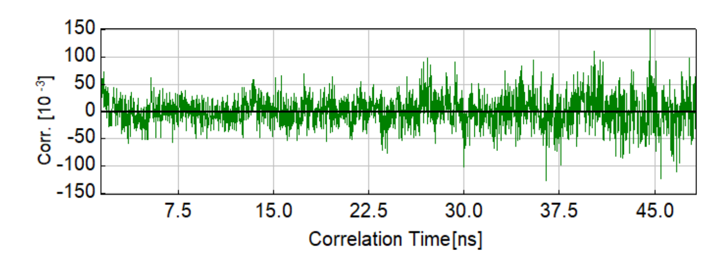

Fitted Parameters

Parameter	Value	Δ	δ
A_1[kCnts/Chnl]	2.148	±0.022	1.0%
τ_1[ns]	9.558	±0.017	0.2%
I_1[kCnts]	820.9	±4.7	0.6%
A_2[kCnts/Chnl]	5.251	±0.077	1.5%
τ_2[ns]	0.733	±0.012	1.6%
I_2[kCnts]	153.8	±3.0	1.9%
A_3[kCnts/Chnl]	3.957	±0.028	0.7%
τ_3[ns]	2.912	±0.033	1.1%
I_3[kCnts]	460.8	±4.1	0.9%
$Bkgr_{Dec}$[kCnts]	0.0332	±0.0003	0.8%
$Bkgr_{IRF}$[Cnts/Chnl]	1.106	±0.068	6.1%
$Shift_{IRF}$[ps]	9.8	±1.2	12%
A_{Scatt}[kCnts]	37.37	±0.68	1.8%
τ_{AvInt}[ns]	6.479	±0.011	0.2%
τ_{AvAmp}[ns]	3.161	±0.014	0.4%
λ_{Det}[nm]	560.0000	---	---

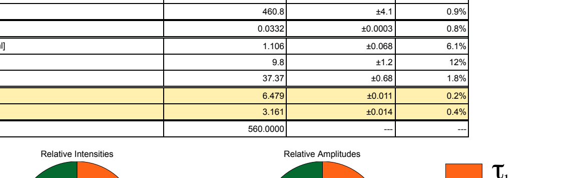

Data Set: 13 / 28

Decay: crv[13]; IRF: crv[0]

Fit

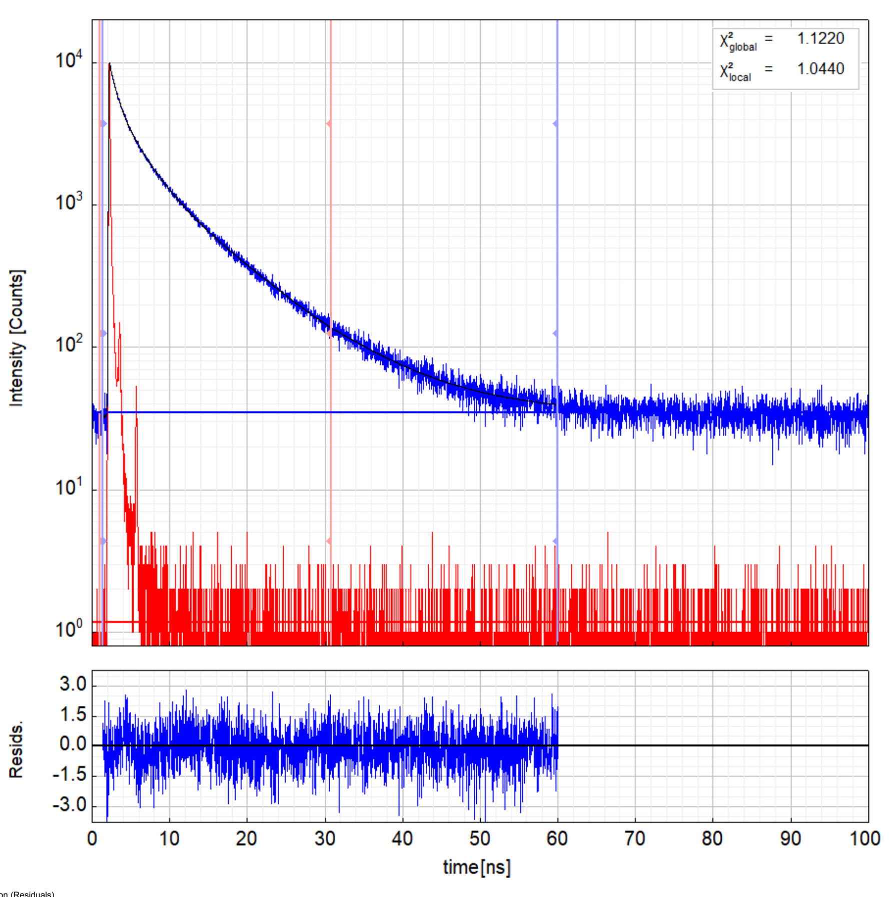

Autocorrelation (Residuals)

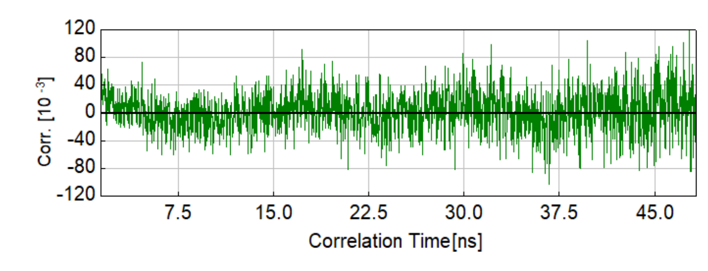

Fitted Parameters

Parameter	Value	Δ	δ
A_1[kCnts/Chnl]	2.179	±0.016	0.7%
τ_1[ns]	9.558	±0.017	0.2%
I_1[kCnts]	832.8	±4.8	0.6%
A_2[kCnts/Chnl]	5.155	±0.057	1.1%
τ_2[ns]	0.733	±0.012	1.6%
I_2[kCnts]	151.0	±3.1	2.0%
A_3[kCnts/Chnl]	3.918	±0.054	1.4%
τ_3[ns]	2.912	±0.033	1.1%
I_3[kCnts]	456.3	±3.2	0.7%
$Bkgr_{Dec}$[kCnts]	0.0346	±0.0004	1.1%
$Bkgr_{IRF}$[Cnts/Chnl]	1.177	±0.056	4.7%
$Shift_{IRF}$[ps]	10.29	±0.79	7.7%
A_{Scatt}[kCnts]	37.45	±0.63	1.7%
τ_{AvInt}[ns]	6.527	±0.012	0.2%
τ_{AvAmp}[ns]	3.200	±0.012	0.4%
λ_{Det}[nm]	570.0000	---	---

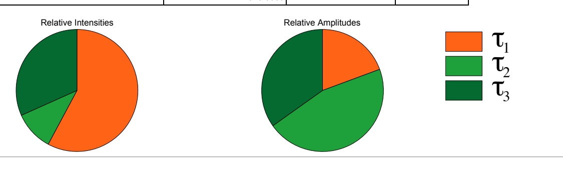

Data Set: 14 / 28

Decay: crv[14]; IRF: crv[0]

Fit

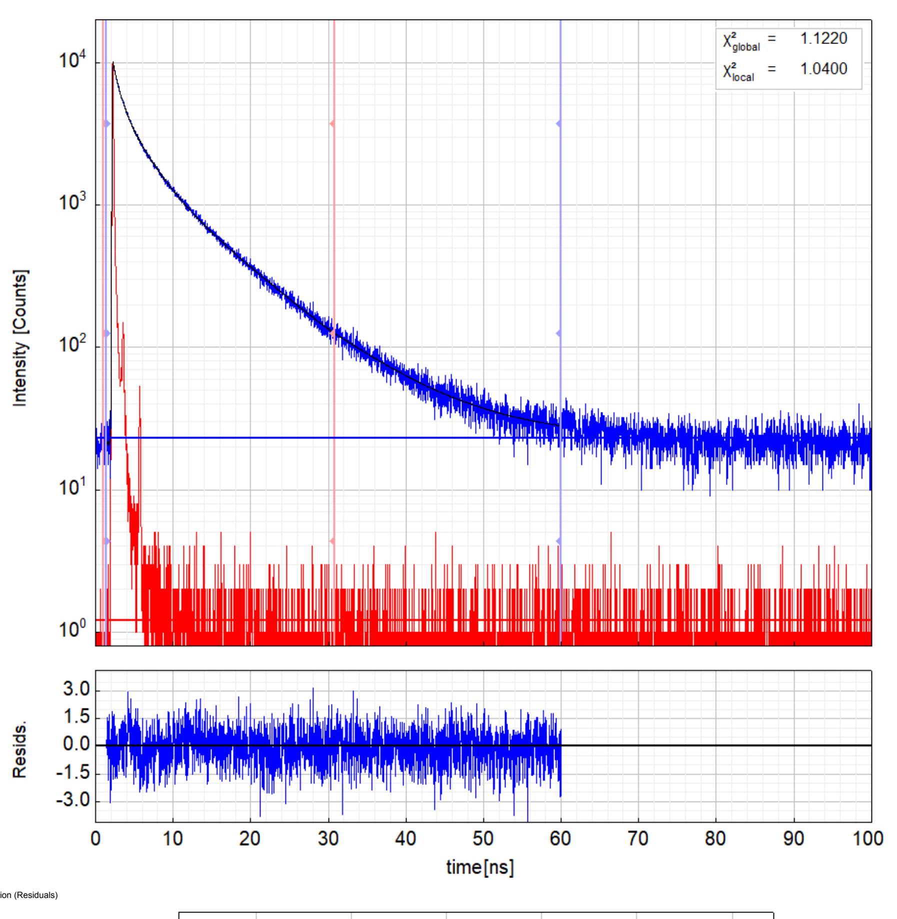

Autocorrelation (Residuals)

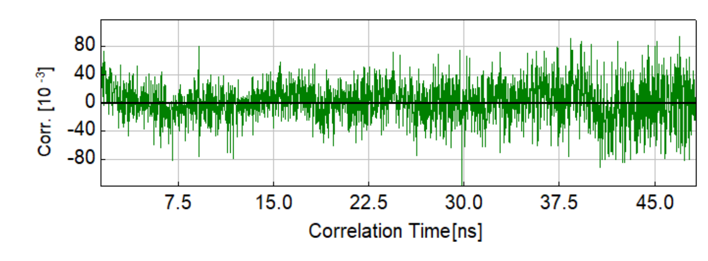

Fitted Parameters

Parameter	Value	Δ	δ
A_1[kCnts/Chnl]	2.178	±0.014	0.6%
τ_1[ns]	9.558	±0.017	0.2%
I_1[kCnts]	832.5	±4.6	0.5%
A_2[kCnts/Chnl]	5.099	±0.028	0.5%
τ_2[ns]	0.733	±0.012	1.6%
I_2[kCnts]	149.4	±3.0	2.0%
A_3[kCnts/Chnl]	3.955	±0.040	1.0%
τ_3[ns]	2.912	±0.033	1.1%
I_3[kCnts]	460.6	±4.0	0.9%
$Bkgr_{Dec}$[kCnts]	0.0232	±0.0003	1.0%
$Bkgr_{IRF}$[Cnts/Chnl]	1.206	±0.070	5.8%
$Shift_{IRF}$[ps]	11.27	±0.57	5.0%
A_{Scatt}[kCnts]	39.93	±0.51	1.3%
τ_{AvInt}[ns]	6.522	±0.013	0.2%
τ_{AvAmp}[ns]	3.211	±0.011	0.3%
λ_{Det}[nm]	580.0000	---	---

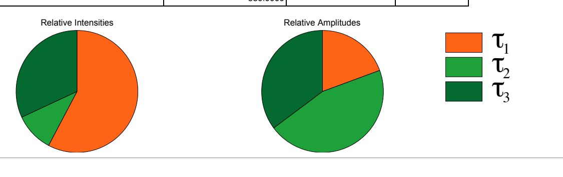

Data Set: 15 / 28

Decay: crv[15]; IRF: crv[0]

Fit

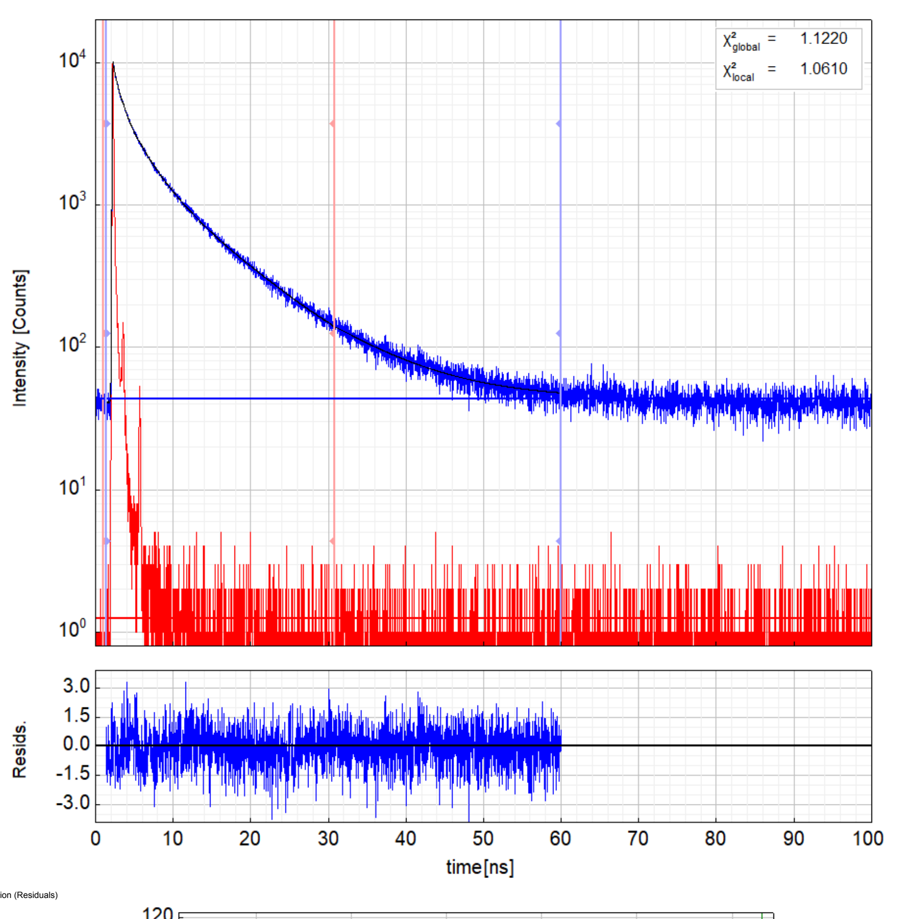

Autocorrelation (Residuals)

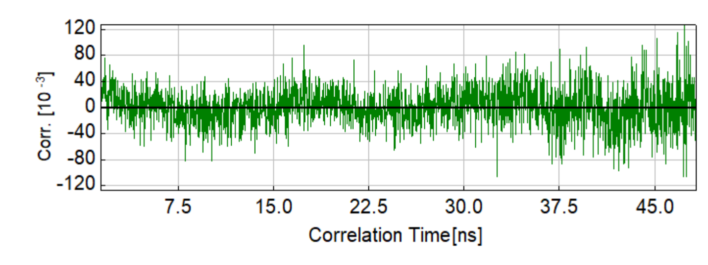

Fitted Parameters

Parameter	Value	Δ	δ
A_1[kCnts/Chnl]	2.088	±0.018	0.8%
τ_1[ns]	9.558	±0.017	0.2%
I_1[kCnts]	797.9	±4.6	0.6%
A_2[kCnts/Chnl]	4.958	±0.082	1.6%
τ_2[ns]	0.733	±0.012	1.6%
I_2[kCnts]	145.2	±4.6	3.2%
A_3[kCnts/Chnl]	3.888	±0.056	1.4%
τ_3[ns]	2.912	±0.033	1.1%
I_3[kCnts]	452.7	±3.7	0.8%
$Bkgr_{Dec}$[kCnts]	0.0432	±0.0003	0.6%
$Bkgr_{IRF}$[Cnts/Chnl]	1.242	±0.070	5.6%
$Shift_{IRF}$[ps]	11.31	±0.59	5.1%
A_{Scatt}[kCnts]	41.26	±0.35	0.8%
τ_{AvInt}[ns]	6.484	±0.013	0.2%
τ_{AvAmp}[ns]	3.193	±0.013	0.4%
λ_{Det}[nm]	590.0000	---	---

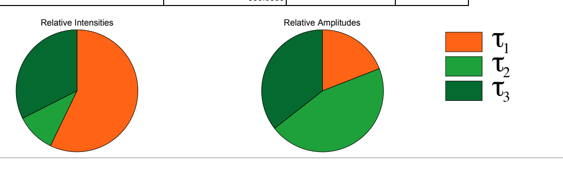

Data Set: 16 / 28

Decay: crv[16]; IRF: crv[0]

Fit

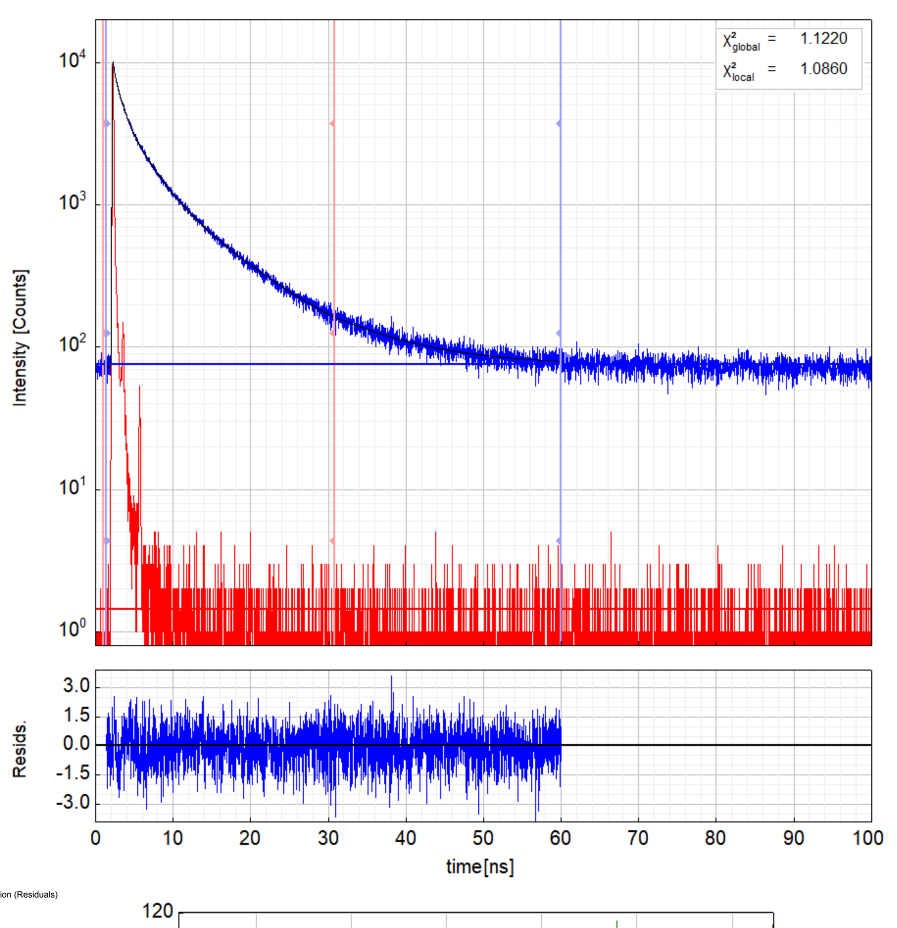

Autocorrelation (Residuals)

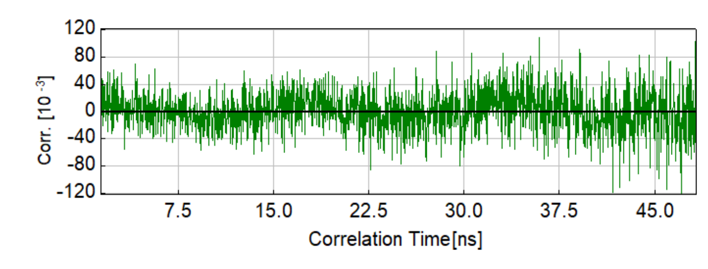

Fitted Parameters

Parameter	Value	Δ	δ
A_1[kCnts/Chnl]	1.936	±0.018	0.9%
τ_1[ns]	9.558	±0.017	0.2%
I_1[kCnts]	740.1	±5.6	0.7%
A_2[kCnts/Chnl]	4.965	±0.047	0.9%
τ_2[ns]	0.733	±0.012	1.6%
I_2[kCnts]	145.4	±3.7	2.5%
A_3[kCnts/Chnl]	3.779	±0.039	1.0%
τ_3[ns]	2.912	±0.033	1.1%
I_3[kCnts]	440.1	±2.6	0.6%
$Bkgr_{Dec}$[kCnts]	0.0753	±0.0005	0.5%
$Bkgr_{IRF}$[Cnts/Chnl]	1.435	±0.076	5.3%
$Shift_{IRF}$[ps]	12.27	±0.56	4.5%
A_{Scatt}[kCnts]	43.46	±0.58	1.3%
τ_{AvInt}[ns]	6.383	±0.011	0.2%
τ_{AvAmp}[ns]	3.104	±0.017	0.5%
λ_{Det}[nm]	600.0000	---	---

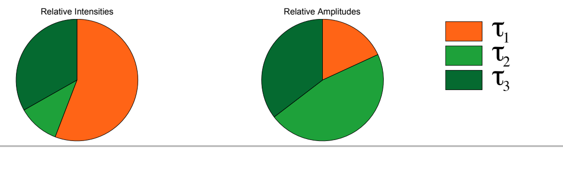

Data Set: 17 / 28

Decay: crv[17]; IRF: crv[0]

Fit

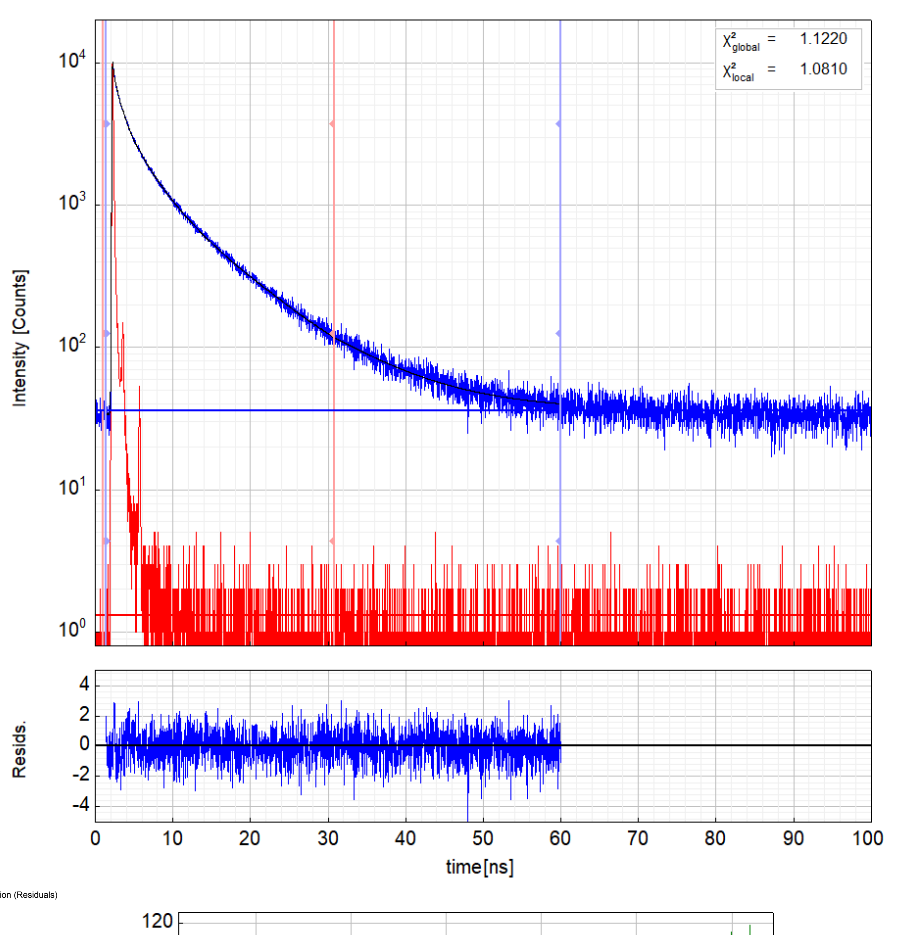

Autocorrelation (Residuals)

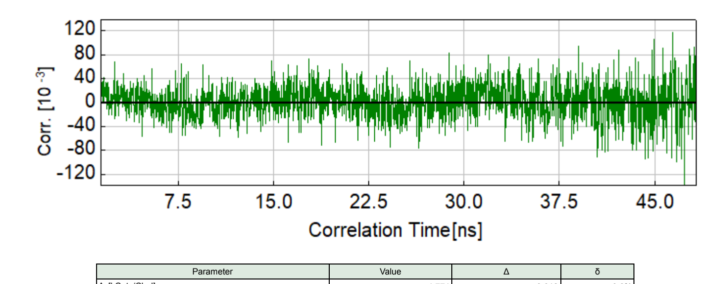

Fitted Parameters

Parameter	Value	Δ	δ
A_1[kCnts/Chnl]	1.771	±0.012	0.6%
τ_1[ns]	9.558	±0.017	0.2%
I_1[kCnts]	676.8	±3.5	0.5%
A_2[kCnts/Chnl]	4.803	±0.058	1.2%
τ_2[ns]	0.733	±0.012	1.6%
I_2[kCnts]	140.7	±3.5	2.5%
A_3[kCnts/Chnl]	3.525	±0.032	0.9%
τ_3[ns]	2.912	±0.033	1.1%
I_3[kCnts]	410.5	±2.9	0.7%
$Bkgr_{Dec}$[kCnts]	0.0362	±0.0003	0.8%
$Bkgr_{IRF}$[Cnts/Chnl]	1.313	±0.082	6.2%
$Shift_{IRF}$[ps]	14.21	±0.54	3.8%
A_{Scatt}[kCnts]	46.63	±0.35	0.7%
τ_{AvInt}[ns]	6.326	±0.023	0.4%
τ_{AvAmp}[ns]	3.041	±0.016	0.5%
λ_{Det}[nm]	610.0000	---	---

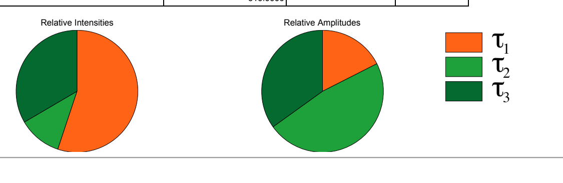

Data Set: 18 / 28

Decay: crv[18]; IRF: crv[0]

Fit

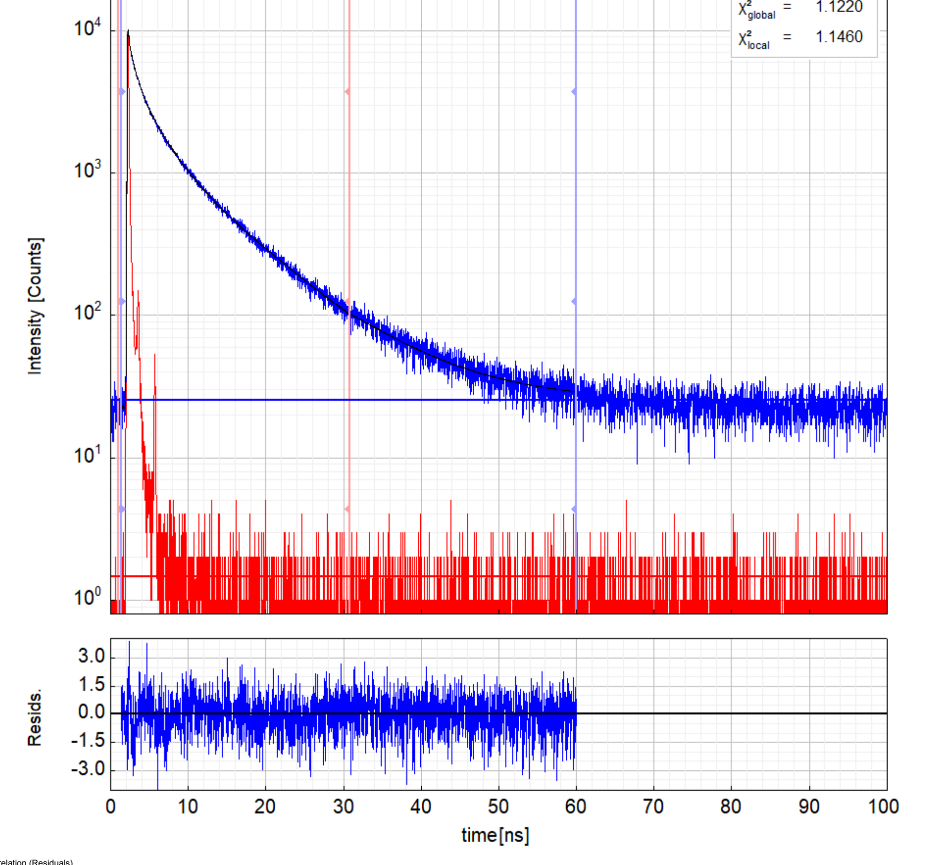

Autocorrelation (Residuals)

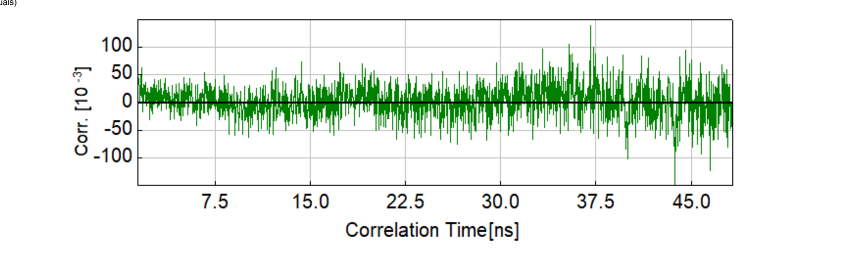

Fitted Parameters

Parameter	Value	Δ	δ
A_1[kCnts/Chnl]	1.723	±0.015	0.9%
τ_1[ns]	9.558	±0.017	0.2%
I_1[kCnts]	658.5	±3.7	0.6%
A_2[kCnts/Chnl]	4.743	±0.061	1.3%
τ_2[ns]	0.733	±0.012	1.6%
I_2[kCnts]	138.9	±3.8	2.7%
A_3[kCnts/Chnl]	3.425	±0.030	0.9%
τ_3[ns]	2.912	±0.033	1.1%
I_3[kCnts]	398.9	±2.9	0.7%
$Bkgr_{Dec}$[kCnts]	0.0253	±0.0003	0.9%
$Bkgr_{IRF}$[Cnts/Chnl]	1.461	±0.077	5.2%
$Shift_{IRF}$[ps]	15.21	±0.56	3.6%
A_{Scatt}[kCnts]	48.40	±0.84	1.7%
τ_{AvInt}[ns]	6.317	±0.014	0.2%
τ_{AvAmp}[ns]	3.024	±0.020	0.6%
λ_{Det}[nm]	620.0000	---	---

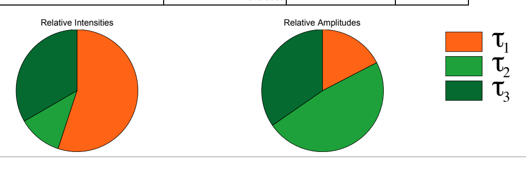

Data Set: 19 / 28

Decay: crv[19]; IRF: crv[0]

Fit

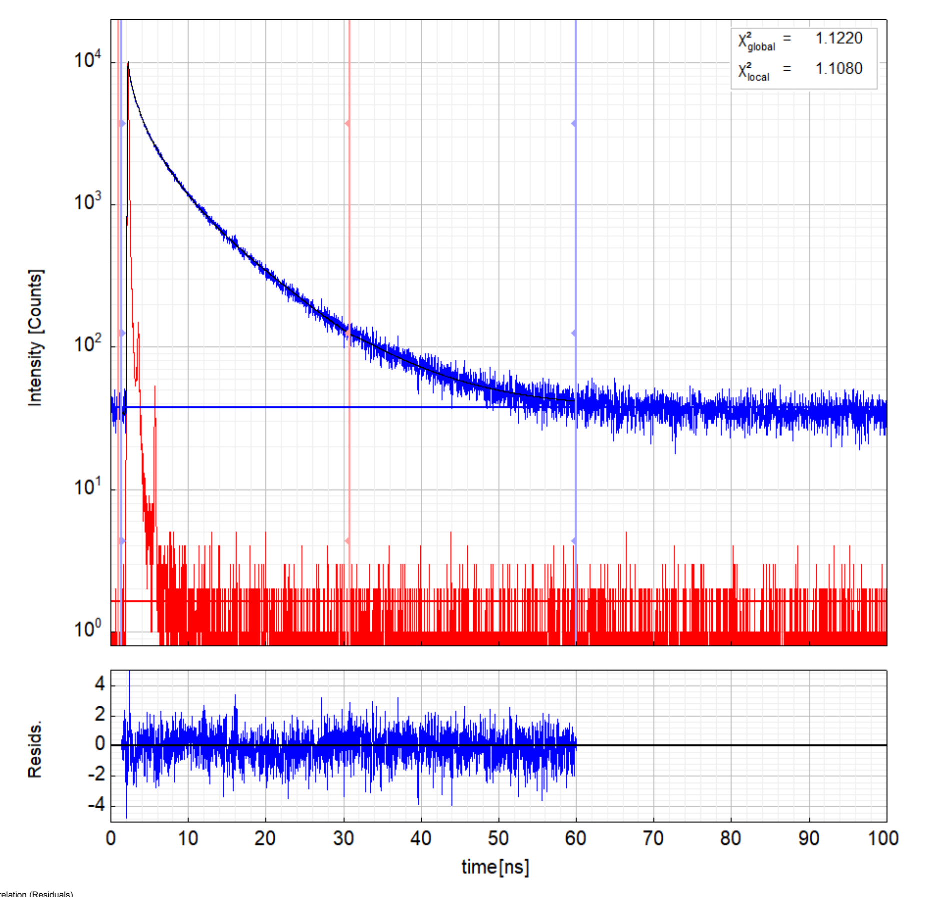

Autocorrelation (Residuals)

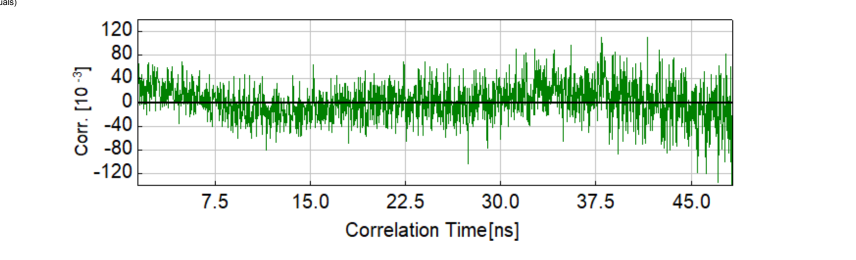

Fitted Parameters

Parameter	Value	Δ	δ
A_1[kCnts/Chnl]	1.980	±0.012	0.6%
τ_1[ns]	9.558	±0.017	0.2%
I_1[kCnts]	756.8	±2.7	0.3%
A_2[kCnts/Chnl]	4.148	±0.057	1.4%
τ_2[ns]	0.733	±0.012	1.6%
I_2[kCnts]	121.5	±4.2	3.4%
A_3[kCnts/Chnl]	3.696	±0.046	1.2%
τ_3[ns]	2.912	±0.033	1.1%
I_3[kCnts]	430.4	±1.7	0.4%
$Bkgr_{Dec}$[kCnts]	0.0374	±0.0004	1.0%
$Bkgr_{IRF}$[Cnts/Chnl]	1.64	±0.13	7.6%
$Shift_{IRF}$[ps]	16.50	±0.55	3.3%
A_{Scatt}[kCnts]	48.30	±0.56	1.1%
τ_{AvInt}[ns]	6.553	±0.017	0.2%
τ_{AvAmp}[ns]	3.331	±0.015	0.4%
λ_{Det}[nm]	630.0000	---	---

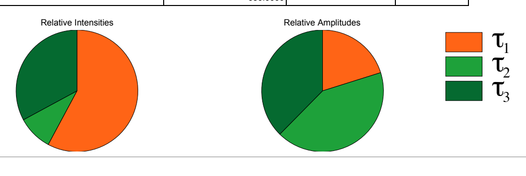

Data Set: 20 / 28

Decay: crv[20]; IRF: crv[0]

Fit

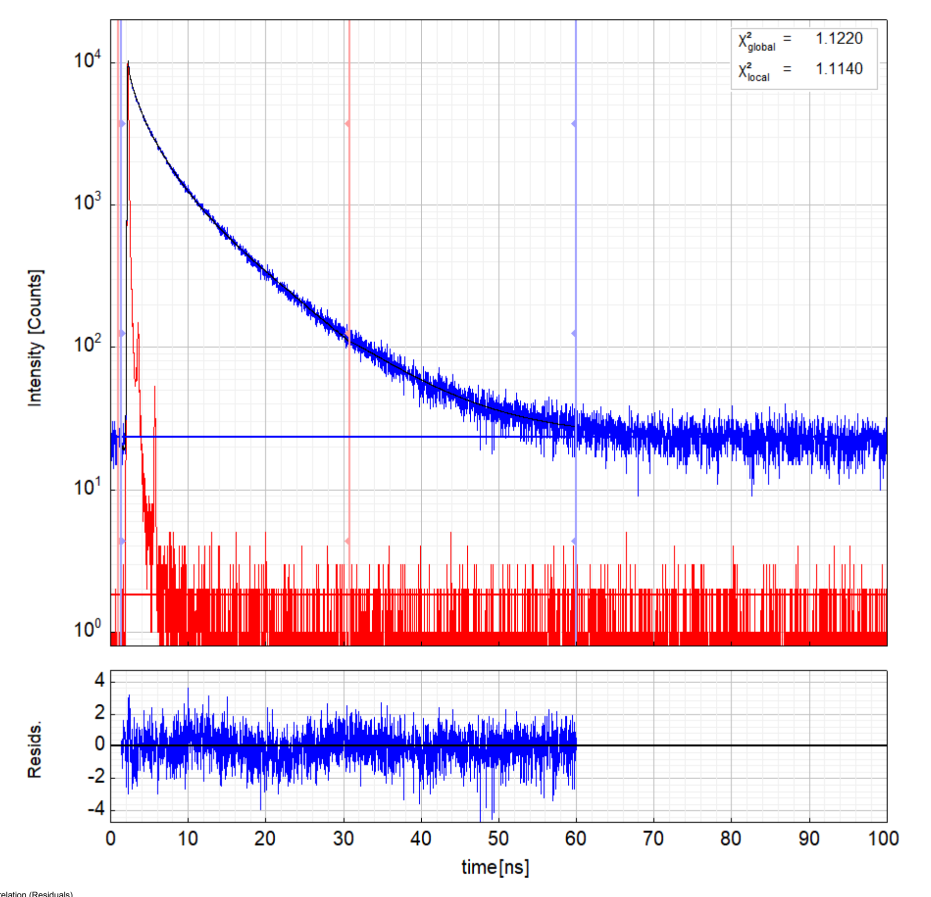

Autocorrelation (Residuals)

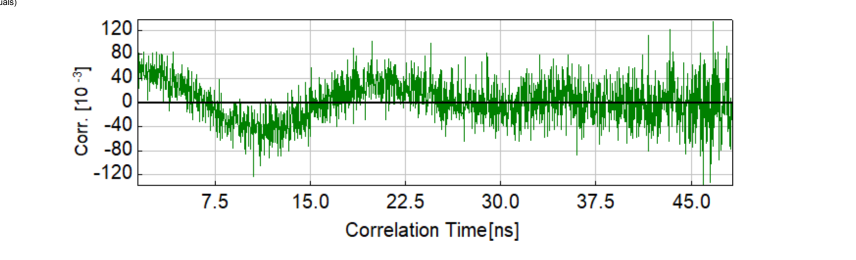

Fitted Parameters

Parameter	Value	Δ	δ
A_1[kCnts/Chnl]	2.067	±0.021	1.0%
τ_1[ns]	9.558	±0.017	0.2%
I_1[kCnts]	790.0	±6.7	0.8%
A_2[kCnts/Chnl]	3.587	±0.060	1.7%
τ_2[ns]	0.733	±0.012	1.6%
I_2[kCnts]	105.1	±1.6	1.5%
A_3[kCnts/Chnl]	4.382	±0.022	0.5%
τ_3[ns]	2.912	±0.033	1.1%
I_3[kCnts]	510.3	±4.8	0.9%
$Bkgr_{Dec}$[kCnts]	0.0233	±0.0003	1.2%
$Bkgr_{IRF}$[Cnts/Chnl]	1.82	±0.12	6.5%
$Shift_{IRF}$[ps]	17.79	±0.79	4.4%
A_{Scatt}[kCnts]	48.4	±1.2	2.3%
τ_{AvInt}[ns]	6.485	±0.018	0.3%
τ_{AvAmp}[ns]	3.501	±0.012	0.3%
λ_{Det}[nm]	640.0000	---	---

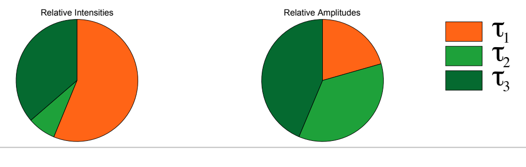

Data Set: 21 / 28

Decay: crv[21]; IRF: crv[0]

Fit

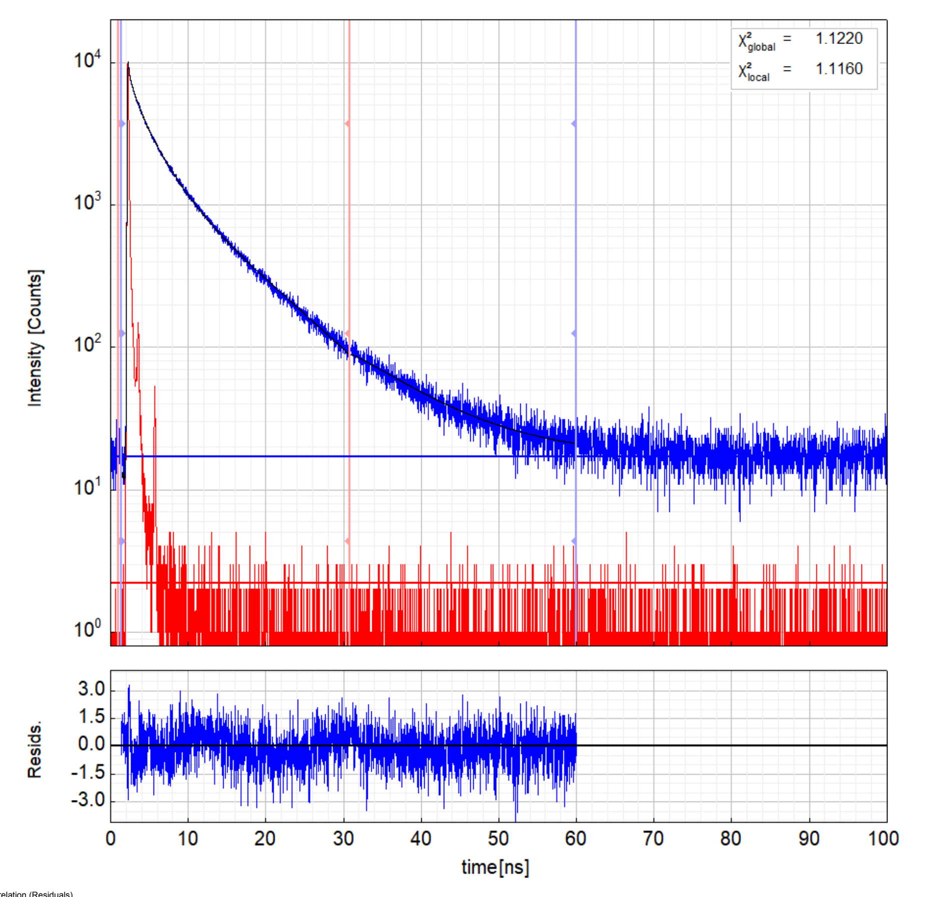

Autocorrelation (Residuals)

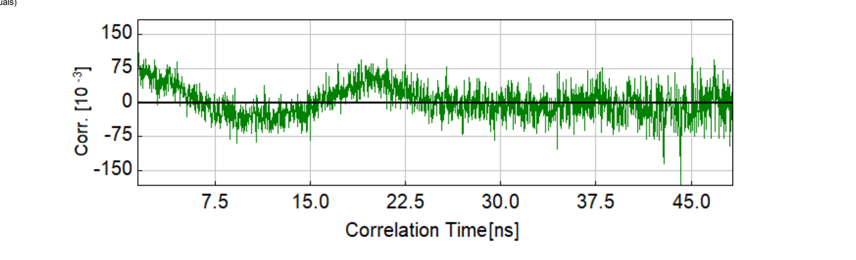

Fitted Parameters

Parameter	Value	Δ	δ
A_1[kCnts/Chnl]	1.888	±0.019	1.0%
τ_1[ns]	9.558	±0.017	0.2%
I_1[kCnts]	721.7	±5.0	0.7%
A_2[kCnts/Chnl]	3.300	±0.052	1.5%
τ_2[ns]	0.733	±0.012	1.6%
I_2[kCnts]	96.7	±2.5	2.5%
A_3[kCnts/Chnl]	4.656	±0.040	0.8%
τ_3[ns]	2.912	±0.033	1.1%
I_3[kCnts]	542.2	±4.0	0.7%
$Bkgr_{Dec}$[kCnts]	0.0172	±0.0002	1.1%
$Bkgr_{IRF}$[Cnts/Chnl]	2.197	±0.076	3.4%
$Shift_{IRF}$[ps]	18.02	±0.76	4.2%
A_{Scatt}[kCnts]	47.4	±1.2	2.4%
τ_{AvInt}[ns]	6.2821	±0.0060	0.1%
τ_{AvAmp}[ns]	3.456	±0.015	0.4%
λ_{Det}[nm]	650.0000	---	---

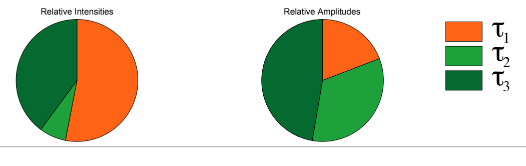

Data Set: 22 / 28

Decay: crv[22]; IRF: crv[0]

Fit

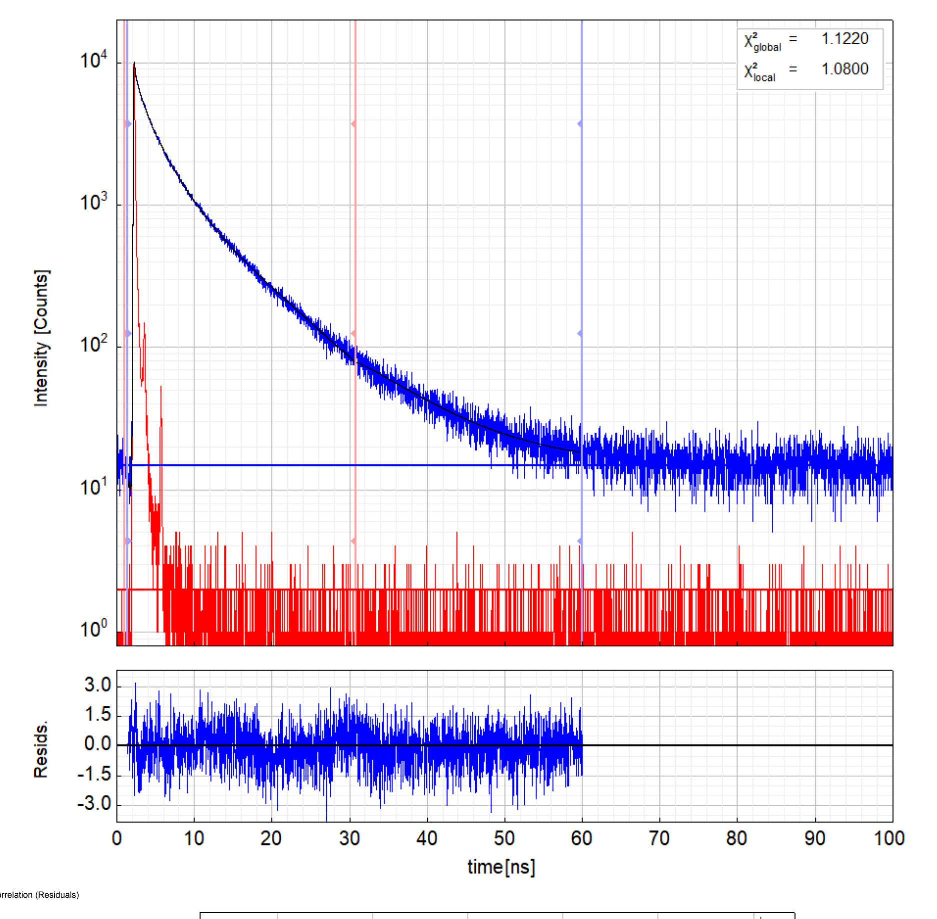

Autocorrelation (Residuals)

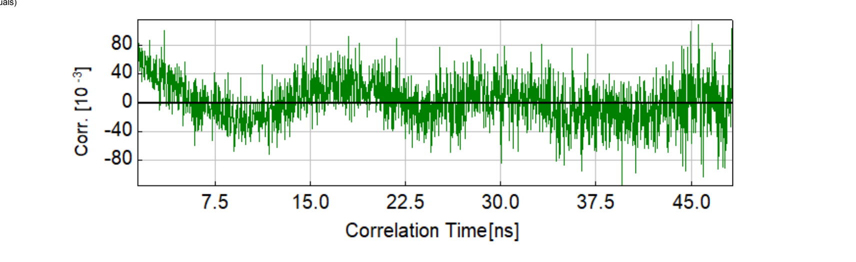

Fitted Parameters

Parameter	Value	Δ	δ
A_1[kCnts/Chnl]	1.631	±0.022	1.3%
τ_1[ns]	9.558	±0.017	0.2%
I_1[kCnts]	623.2	±7.2	1.2%
A_2[kCnts/Chnl]	3.467	±0.069	2.0%
τ_2[ns]	0.733	±0.012	1.6%
I_2[kCnts]	101.6	±2.9	2.8%
A_3[kCnts/Chnl]	4.735	±0.060	1.3%
τ_3[ns]	2.912	±0.033	1.1%
I_3[kCnts]	551.4	±4.1	0.7%
$Bkgr_{Dec}$[kCnts]	0.0148	±0.0002	0.7%
$Bkgr_{IRF}$[Cnts/Chnl]	1.987	±0.084	4.2%
$Shift_{IRF}$[ps]	19.03	±0.77	4.0%
A_{Scatt}[kCnts]	48.8	±2.2	4.5%
τ_{AvInt}[ns]	5.984	±0.013	0.2%
τ_{AvAmp}[ns]	3.246	±0.018	0.6%
λ_{Det}[nm]	660.0000	---	---

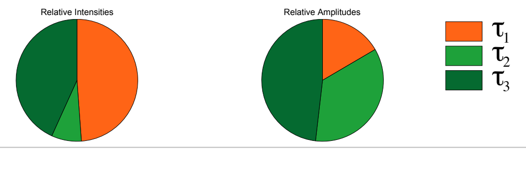

Data Set: 23 / 28

Decay: crv[23]; IRF: crv[0]

Fit

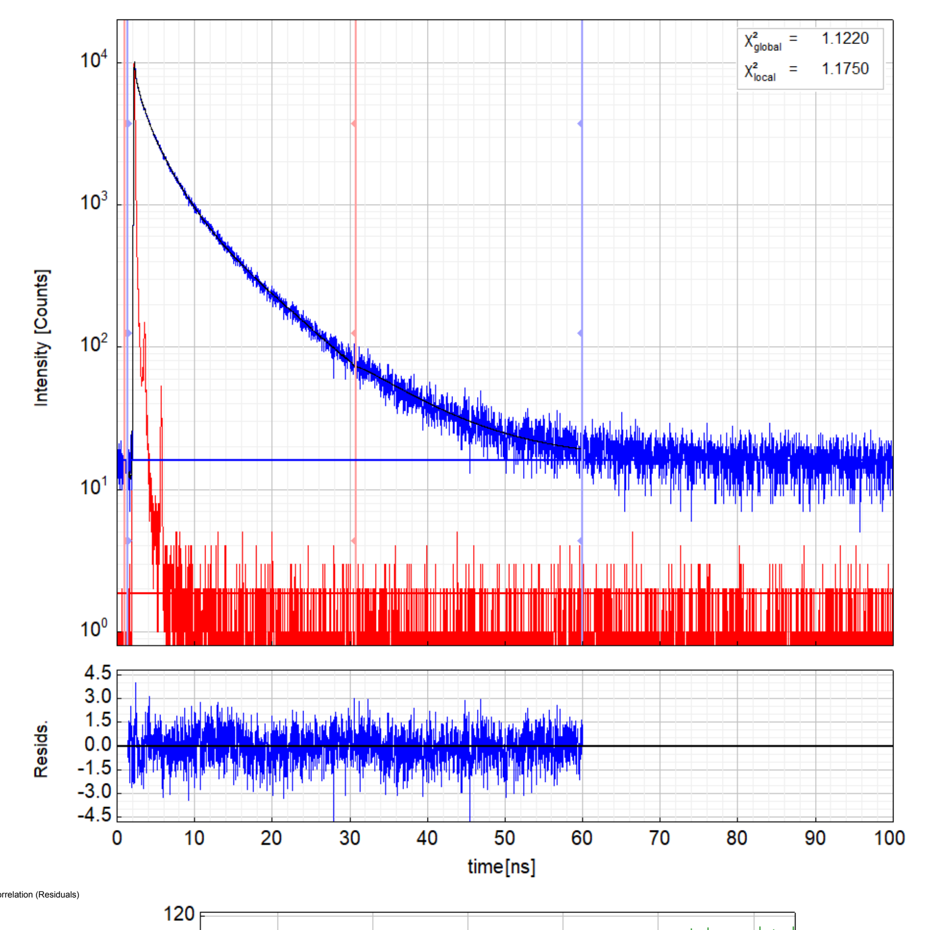

Autocorrelation (Residuals)

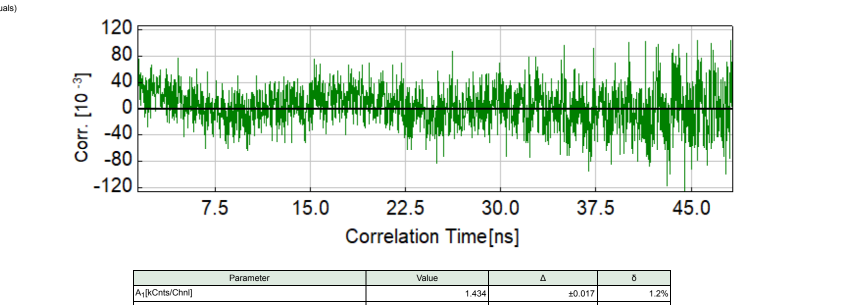

Fitted Parameters

Parameter	Value	Δ	δ
A_1[kCnts/Chnl]	1.434	±0.017	1.2%
τ_1[ns]	9.558	±0.017	0.2%
I_1[kCnts]	548.2	±5.6	1.0%
A_2[kCnts/Chnl]	3.682	±0.065	1.8%
τ_2[ns]	0.733	±0.012	1.6%
I_2[kCnts]	107.9	±3.1	2.8%
A_3[kCnts/Chnl]	4.411	±0.037	0.8%
τ_3[ns]	2.912	±0.033	1.1%
I_3[kCnts]	513.7	±1.7	0.3%
$Bkgr_{Dec}$[kCnts]	0.0162	±0.0003	1.4%
$Bkgr_{IRF}$[Cnts/Chnl]	1.861	±0.089	4.7%
$Shift_{IRF}$[ps]	19.58	±0.70	3.6%

Parameter	Value	Δ	δ
A_{Scatt}[kCnts]	50.3	±1.1	2.2%
τ_{AvInt}[ns]	5.826	±0.012	0.2%
τ_{AvAmp}[ns]	3.070	±0.016	0.5%
λ_{Det}[nm]	670.0000	---	---

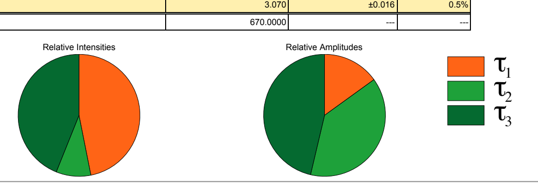

Data Set: 24 / 28

Decay: crv[24]; IRF: crv[0]

Fit

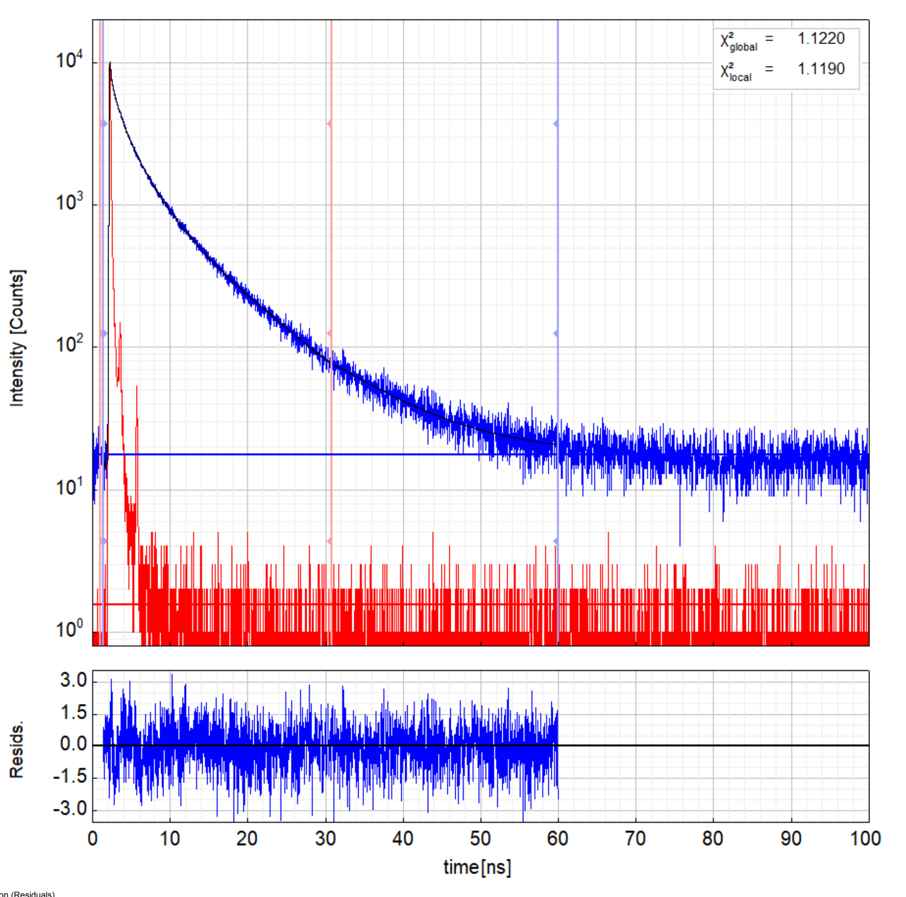

Autocorrelation (Residuals)

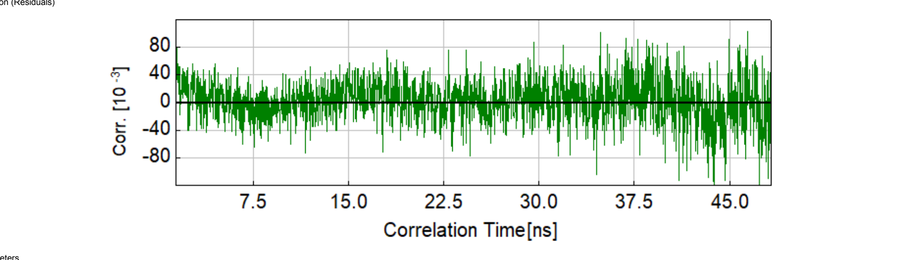

Fitted Parameters

Parameter	Value	Δ	δ
A_1[kCnts/Chnl]	1.376	±0.017	1.2%
τ_1[ns]	9.558	±0.017	0.2%
I_1[kCnts]	525.7	±5.4	1.0%
A_2[kCnts/Chnl]	3.94	±0.12	2.9%
τ_2[ns]	0.733	±0.012	1.6%
I_2[kCnts]	115.2	±3.3	2.9%
A_3[kCnts/Chnl]	4.005	±0.054	1.3%
τ_3[ns]	2.912	±0.033	1.1%
I_3[kCnts]	466.4	±2.8	0.6%
$Bkgr_{Dec}$[kCnts]	0.0177	±0.0002	0.6%
$Bkgr_{IRF}$[Cnts/Chnl]	1.562	±0.054	3.4%
$Shift_{IRF}$[ps]	19.75	±0.57	2.9%
A_{Scatt}[kCnts]	50.78	±0.64	1.3%
τ_{AvInt}[ns]	5.8404	±0.0087	0.1%
τ_{AvAmp}[ns]	2.973	±0.020	0.6%
λ_{Det}[nm]	680.0000	---	---

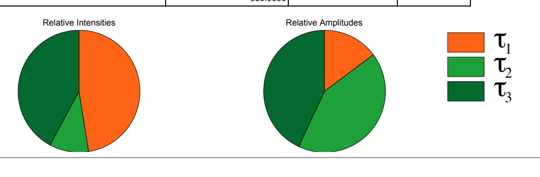

Data Set: 25 / 28

Decay: crv[25]; IRF: crv[0]

Fit

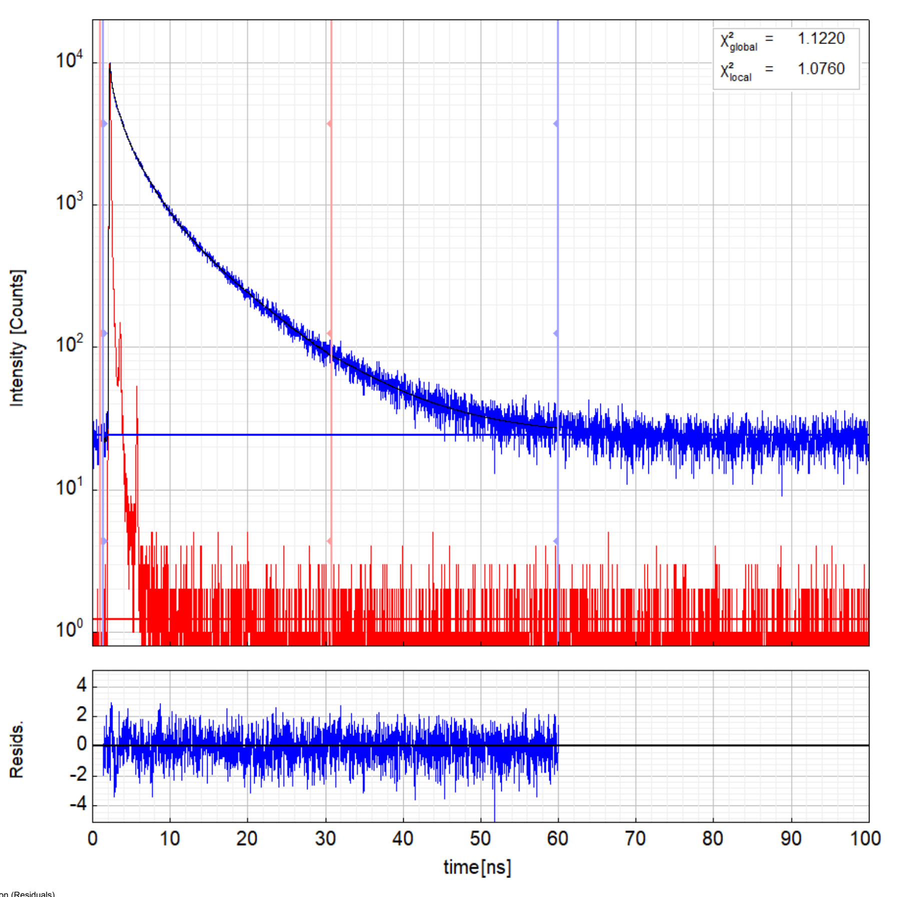

Autocorrelation (Residuals)

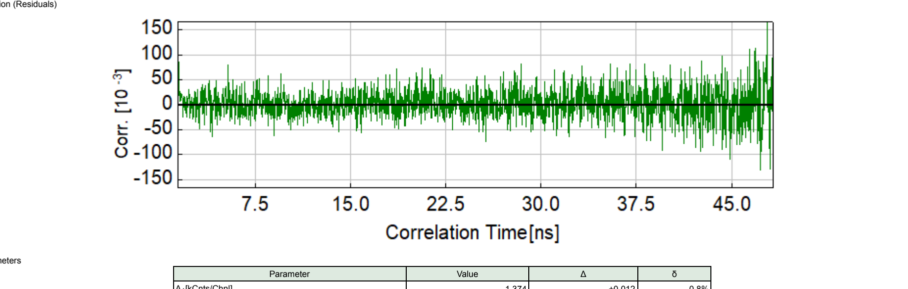

Fitted Parameters

Parameter	Value	Δ	δ
A_1[kCnts/Chnl]	1.374	±0.012	0.8%
τ_1[ns]	9.558	±0.017	0.2%
I_1[kCnts]	525.1	±3.7	0.7%
A_2[kCnts/Chnl]	3.865	±0.031	0.8%
τ_2[ns]	0.733	±0.012	1.6%
I_2[kCnts]	113.2	±2.8	2.4%
A_3[kCnts/Chnl]	3.641	±0.039	1.1%
τ_3[ns]	2.912	±0.033	1.1%
I_3[kCnts]	424.1	±1.2	0.3%
$Bkgr_{Dec}$[kCnts]	0.0240	±0.0003	0.9%
$Bkgr_{IRF}$[Cnts/Chnl]	1.22	±0.11	8.5%
$Shift_{IRF}$[ps]	21.18	±0.70	3.3%
A_{Scatt}[kCnts]	50.76	±0.77	1.5%
τ_{AvInt}[ns]	5.965	±0.019	0.3%
τ_{AvAmp}[ns]	2.992	±0.014	0.5%
λ_{Det}[nm]	690.0000	---	---

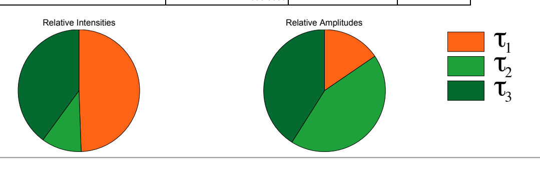

Data Set: 26 / 28

Decay: crv[26]; IRF: crv[0]

Fit

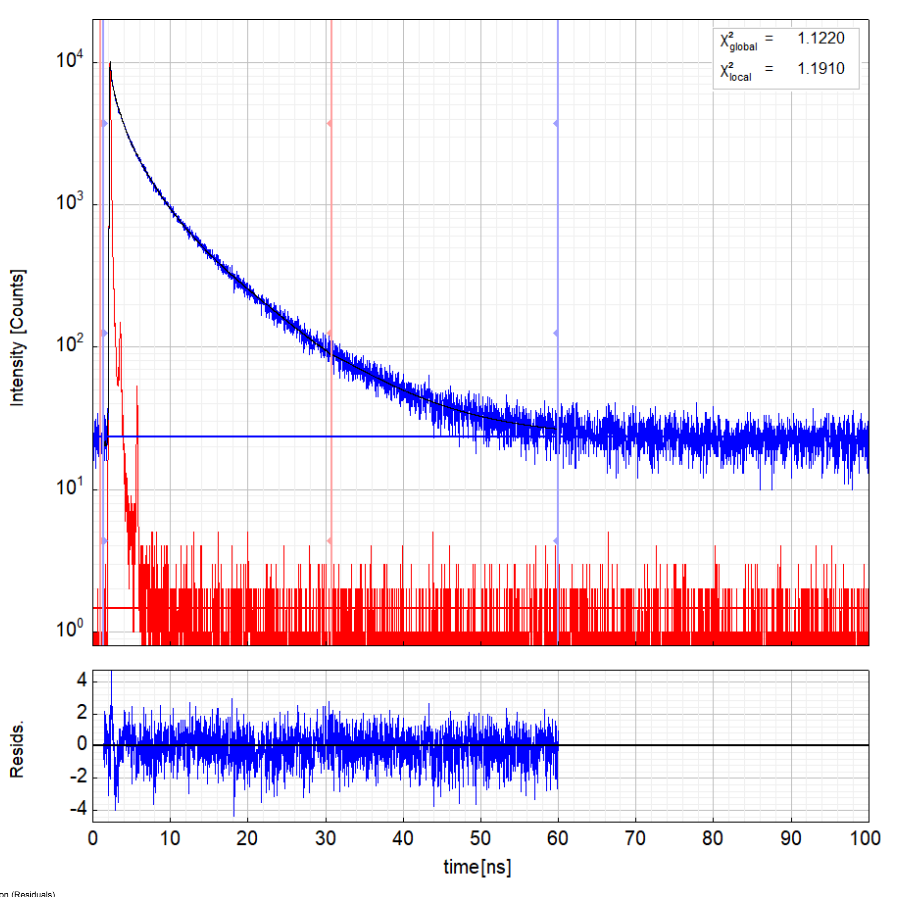

Autocorrelation (Residuals)

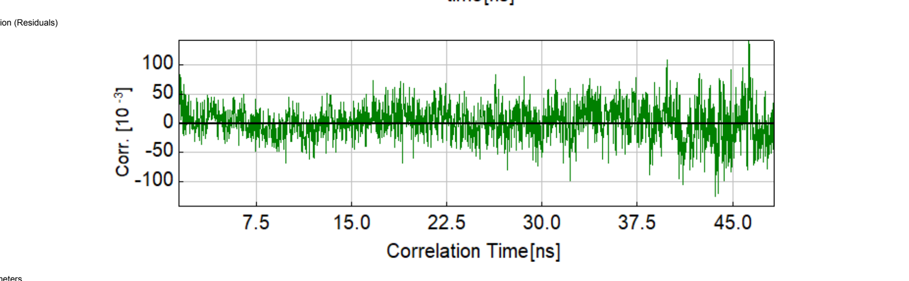

Fitted Parameters

Parameter	Value	Δ	δ
A_1[kCnts/Chnl]	1.482	±0.017	1.1%
τ_1[ns]	9.558	±0.017	0.2%
I_1[kCnts]	566.2	±6.4	1.1%
A_2[kCnts/Chnl]	3.933	±0.096	2.4%
τ_2[ns]	0.733	±0.012	1.6%
I_2[kCnts]	115.2	±2.8	2.4%
A_3[kCnts/Chnl]	3.682	±0.032	0.8%
τ_3[ns]	2.912	±0.033	1.1%
I_3[kCnts]	428.8	±2.7	0.6%
$Bkgr_{Dec}$[kCnts]	0.0233	±0.0004	1.7%
$Bkgr_{IRF}$[Cnts/Chnl]	1.469	±0.094	6.4%
$Shift_{IRF}$[ps]	21.79	±0.69	3.2%
A_{Scatt}[kCnts]	52.2	±1.4	2.5%
τ_{AvInt}[ns]	6.075	±0.014	0.2%
τ_{AvAmp}[ns]	3.052	±0.025	0.8%
λ_{Det}[nm]	700.0000	---	---

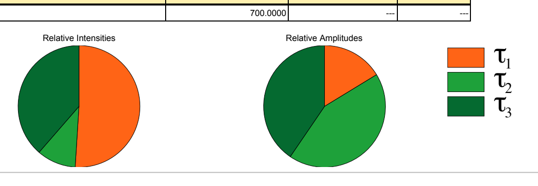

Data Set: 27 / 28

Decay: crv[27]; IRF: crv[0]

Fit

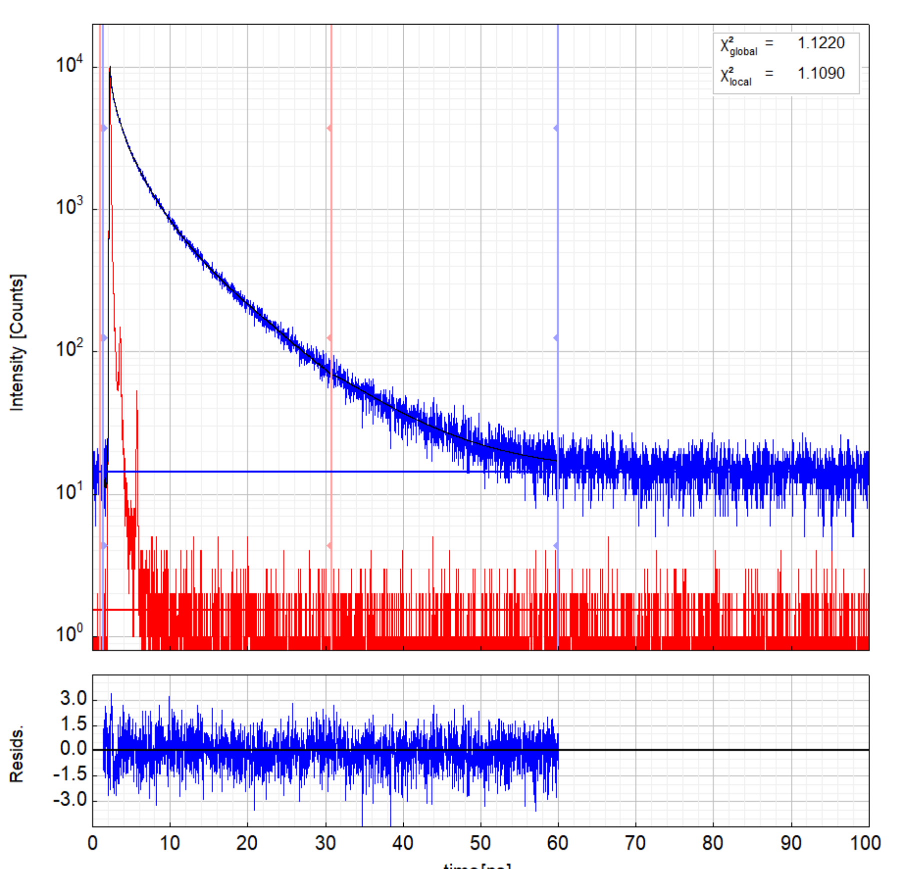

Autocorrelation (Residuals)

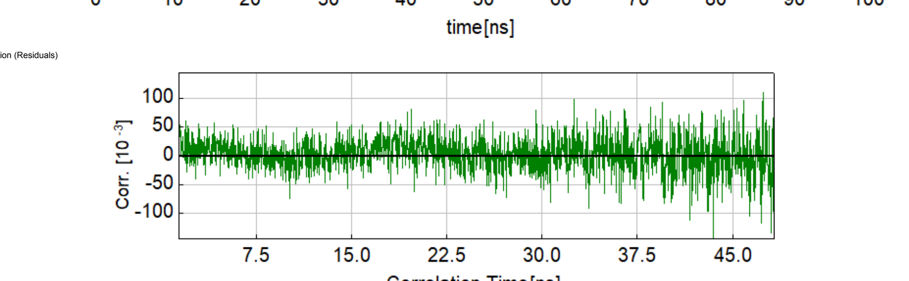

Fitted Parameters

Parameter	Value	Δ	δ
A_1[kCnts/Chnl]	1.287	±0.012	0.9%
τ_1[ns]	9.558	±0.017	0.2%
I_1[kCnts]	491.7	±3.2	0.6%
A_2[kCnts/Chnl]	3.893	±0.082	2.1%
τ_2[ns]	0.733	±0.012	1.6%
I_2[kCnts]	114.1	±3.7	3.2%
A_3[kCnts/Chnl]	3.603	±0.038	1.0%
τ_3[ns]	2.912	±0.033	1.1%
I_3[kCnts]	419.6	±1.6	0.4%
$Bkgr_{Dec}$[kCnts]	0.0143	±0.0003	1.5%
$Bkgr_{IRF}$[Cnts/Chnl]	1.523	±0.093	6.1%
$Shift_{IRF}$[ps]	22.93	±0.80	3.5%
A_{Scatt}[kCnts]	53.41	±0.51	1.0%
τ_{AvInt}[ns]	5.857	±0.016	0.3%
τ_{AvAmp}[ns]	2.919	±0.017	0.6%
λ_{Det}[nm]	710.0000	---	---

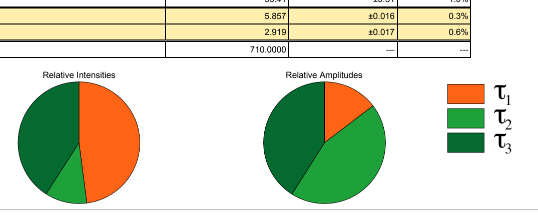

Data Set: 28 / 28

Decay: crv[28]; IRF: crv[0]

Fit

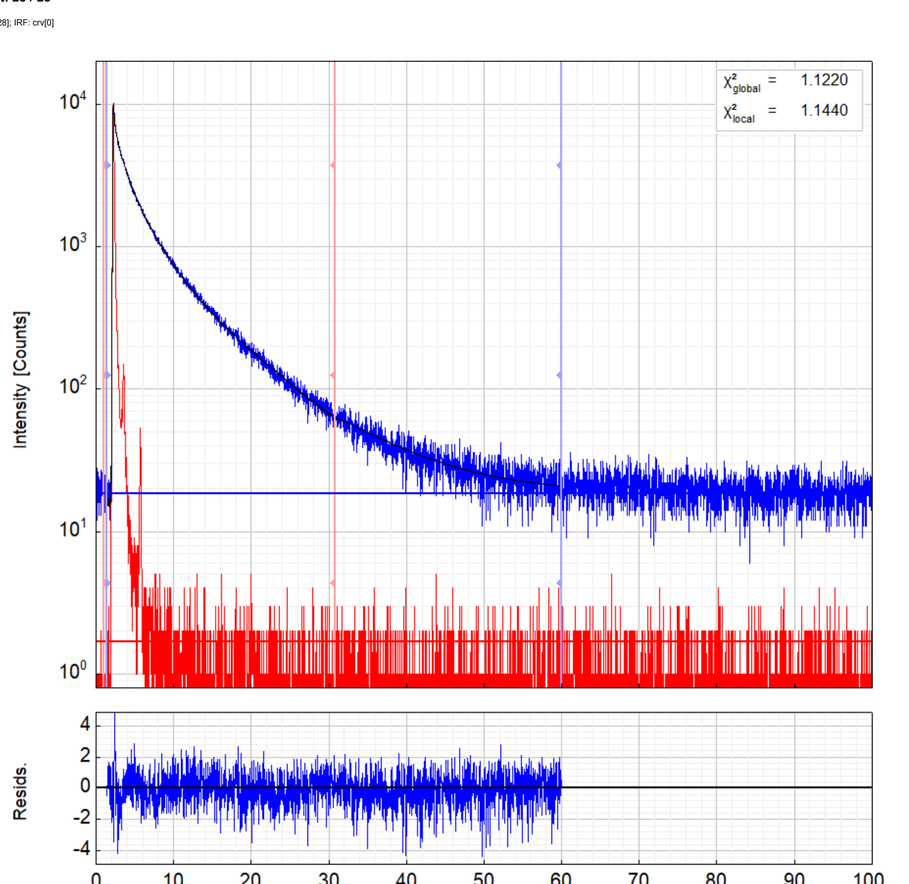

Autocorrelation (Residuals)

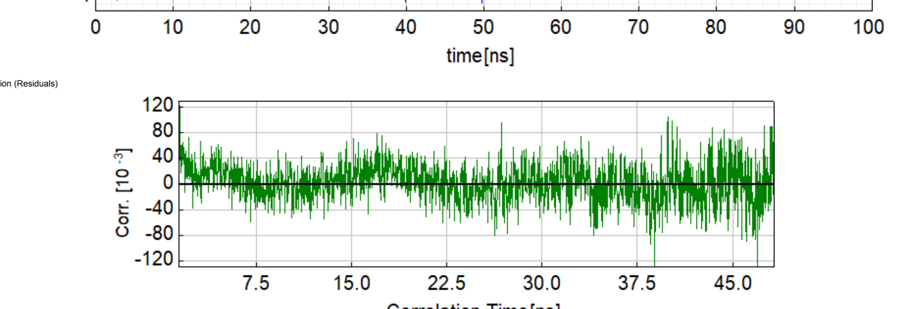

Fitted Parameters

Parameter	Value	Δ	δ
A_1[kCnts/Chnl]	1.0802	±0.0073	0.7%
τ_1[ns]	9.558	±0.017	0.2%
I_1[kCnts]	413.0	±2.2	0.5%
A_2[kCnts/Chnl]	4.027	±0.079	1.9%
τ_2[ns]	0.733	±0.012	1.6%
I_2[kCnts]	118.0	±3.4	2.8%
A_3[kCnts/Chnl]	3.513	±0.050	1.4%
τ_3[ns]	2.912	±0.033	1.1%
I_3[kCnts]	409.1	±2.5	0.6%
$Bkgr_{Dec}$[kCnts]	0.0186	±0.0002	1.0%
$Bkgr_{IRF}$[Cnts/Chnl]	1.690	±0.043	2.5%
$Shift_{IRF}$[ps]	22.62	±0.60	2.6%
A_{Scatt}[kCnts]	55.55	±0.37	0.7%
τ_{AvInt}[ns]	5.5579	±0.0082	0.1%
τ_{AvAmp}[ns]	2.727	±0.022	0.8%
λ_{Det}[nm]	720.0000	---	---

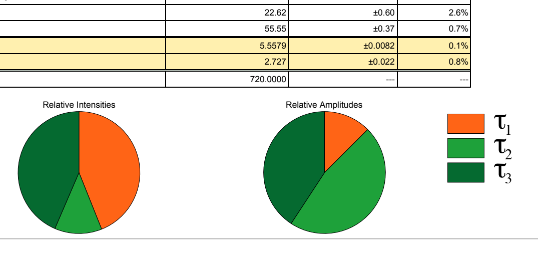

Parameter Plot

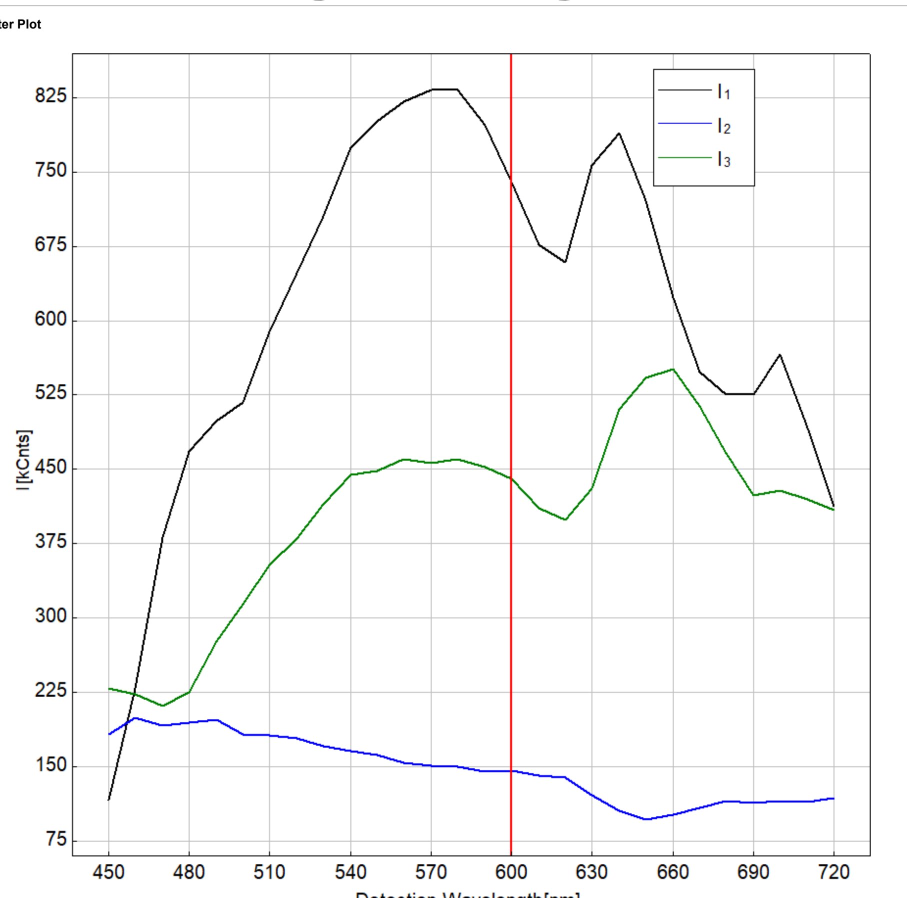

Global Parameters

Parameter	Value	Δ	δ
τ_1[ns]	9.558	±0.017	0.2%
τ_2[ns]	0.733	±0.012	1.6%
τ_3[ns]	2.912	±0.033	1.1%

EasyTau Report

3/11/2022 7:28:11 PM
F:\Paper Xiongfei\CD-1 EtOH tau global.html

Container Curves

TRES+IRF_20201222_1453.etc: crv[0 - 33]

Container Properties

Measurement Context: Time Resolved Emission Scan
Detection Wavelength: 400.0 - 720.0 (Step: 10.0) nm
Summary:

Sample:	CD_X_1
Solvent:	Ethanol
Excitation:	V pol 376±1nm with LDH-P-C-375
Detection:	M pol 400...720nm 10nm steps<br>10000 peak counts<br>grating 1200/500-<br>detector UV-red [PMT]

Comment

Model: Exponential [Reconv.]

$$\mathrm{Dec}(t)=\left[\int_{-\infty}^{t} dt'\,[\mathrm{IRF}(t-Shift_{IRF})-Bkgr_{IRF}]\left[\sum_{i=1}^{n_{Exp}} A_i\, e^{-\frac{t-t'}{\tau_i}}+A_{Scatt}\,\delta(t-t')\right]\right]+Bkgr_{Dec}$$

$$I_m = A_m \tau_m$$

$$A_{Rel\,m}=A_m \Big/ \sum_{\substack{i=1\\A_i>0}}^{n_{Exp}} A_i \qquad I_{Rel\,m}=I_m \Big/ \sum_{\substack{i=1\\I_i>0}}^{n_{Exp}} I_i$$

$$\tau_{Av\,Amp}=\sum_{\substack{i=1\\A_i>0}}^{n_{Exp}} A_i\tau_i \Big/ \sum_{\substack{i=1\\A_i>0}}^{n_{Exp}} A_i \qquad \tau_{Av\,Int}=\sum_{\substack{i=1\\I_i>0}}^{n_{Exp}} I_i\tau_i \Big/ \sum_{\substack{i=1\\I_i>0}}^{n_{Exp}} I_i$$

Data Set: 1 / 33

Decay: crv[1]; IRF: crv[0]

Fit

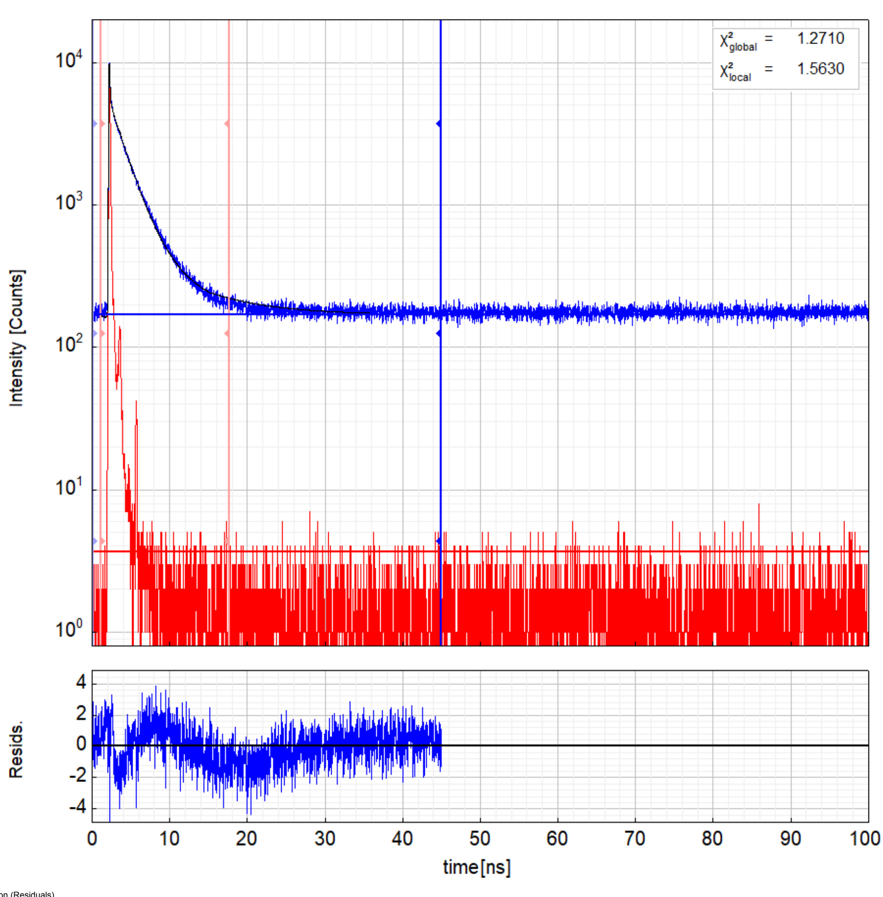

Autocorrelation (Residuals)

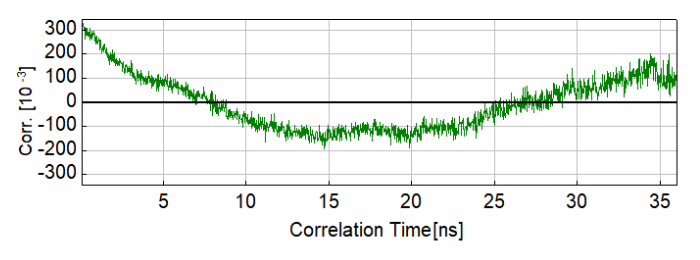

Fitted Parameters

Parameter	Value	Δ	δ
A_1[kCnts/Chnl]	0.35	±0.11	31%
τ_1[ns]	0.460	±0.019	4.0%
I_1[kCnts]	6.3	±2.3	36%
A_2[kCnts/Chnl]	4.939	±0.057	1.1%
τ_2[ns]	2.220	±0.021	0.9%
I_2[kCnts]	438.5	±6.3	1.4%
A_3[kCnts/Chnl]	0.386	±0.042	11%
τ_3[ns]	7.772	±0.017	0.2%
I_3[kCnts]	120	±13	11%
$Bkgr_{Dec}$[kCnts]	0.1687	±0.0011	0.6%
$Bkgr_{IRF}$[Cnts/Chnl]	3.7	±1.2	32%
$Shift_{IRF}$[ps]	-11.42	±0.62	5.4%
A_{Scatt}[kCnts]	62.46	±0.94	1.5%
τ_{AvInt}[ns]	3.379	±0.092	2.7%
τ_{AvAmp}[ns]	2.492	±0.036	1.4%
λ_{Det}[nm]	400.0000	---	---

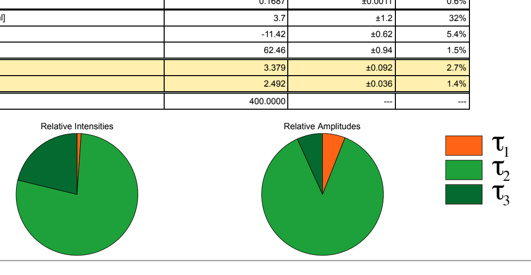

Data Set: 2 / 33

Decay: crv[2]; IRF: crv[0]

Fit

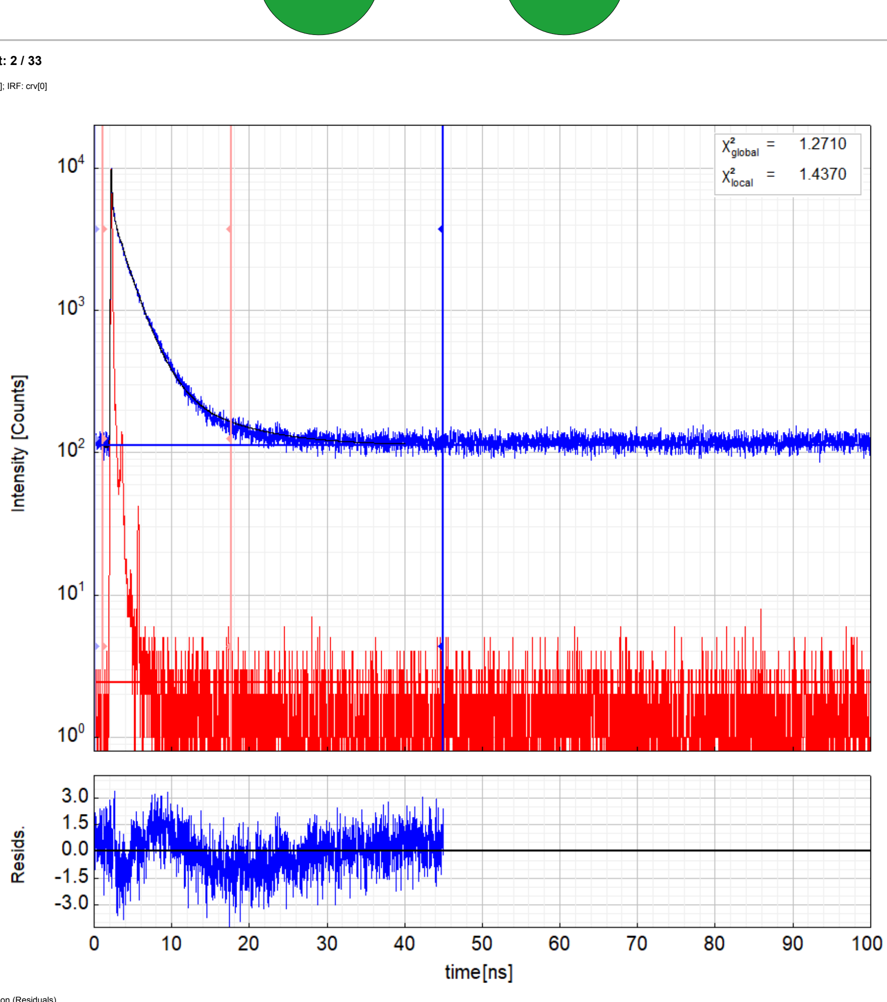

Autocorrelation (Residuals)

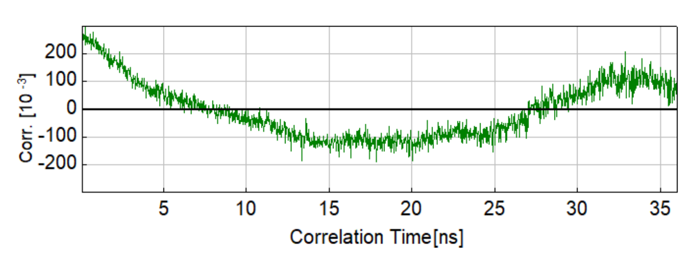

Fitted Parameters

Parameter	Value	Δ	δ
A_1[kCnts/Chnl]	1.129	±0.083	7.3%
τ_1[ns]	0.460	±0.019	4.0%
I_1[kCnts]	20.8	±1.9	8.8%
A_2[kCnts/Chnl]	4.383	±0.050	1.1%
τ_2[ns]	2.220	±0.021	0.9%
I_2[kCnts]	389.2	±4.3	1.1%
A_3[kCnts/Chnl]	0.364	±0.026	7.1%
τ_3[ns]	7.772	±0.017	0.2%
I_3[kCnts]	113.1	±7.8	6.8%
$Bkgr_{Dec}$[kCnts]	0.1123	±0.0006	0.5%
$Bkgr_{IRF}$[Cnts/Chnl]	2.44	±0.79	32%
$Shift_{IRF}$[ps]	-10.04	±0.73	7.2%
A_{Scatt}[kCnts]	62.9	±1.2	1.8%
τ_{AvInt}[ns]	3.351	±0.063	1.9%
τ_{AvAmp}[ns]	2.226	±0.019	0.8%
λ_{Det}[nm]	410.0000	---	---

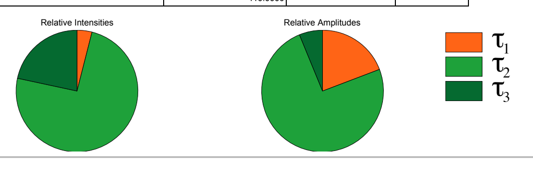

Data Set: 3 / 33

Decay: crv[3]; IRF: crv[0]

Fit

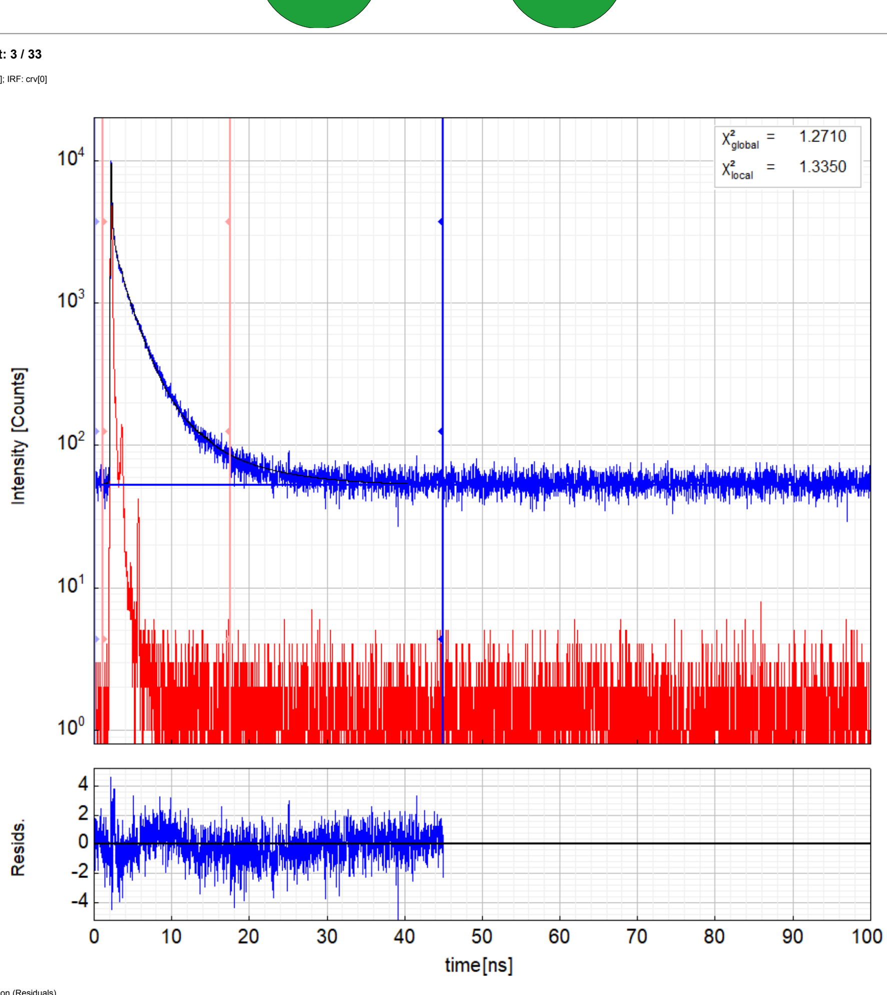

Autocorrelation (Residuals)

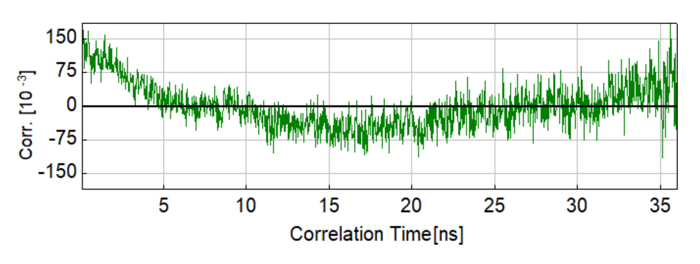

Fitted Parameters

Parameter	Value	Δ	δ
A_1[kCnts/Chnl]	1.02	±0.15	14%
τ_1[ns]	0.460	±0.019	4.0%
I_1[kCnts]	18.8	±2.4	12%
A_2[kCnts/Chnl]	2.379	±0.035	1.4%
τ_2[ns]	2.220	±0.021	0.9%
I_2[kCnts]	211.2	±2.4	1.1%
A_3[kCnts/Chnl]	0.2064	±0.0059	2.8%
τ_3[ns]	7.772	±0.017	0.2%
I_3[kCnts]	64.2	±1.9	2.9%
$Bkgr_{Dec}$[kCnts]	0.0522	±0.0003	0.4%
$Bkgr_{IRF}$[Cnts/Chnl]	-0.17	±0.59	362%
$Shift_{IRF}$[ps]	-18.0	±1.1	5.6%
A_{Scatt}[kCnts]	70.1	±1.4	2.0%
τ_{AvInt}[ns]	3.319	±0.044	1.3%
τ_{AvAmp}[ns]	2.041	±0.032	1.5%
λ_{Det}[nm]	420.0000	---	---

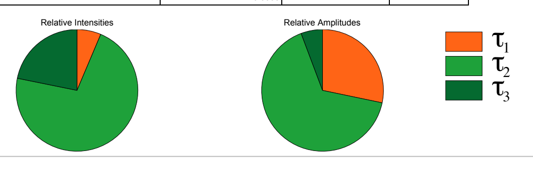

Data Set: 4 / 33

Decay: crv[4]; IRF: crv[0]

Fit

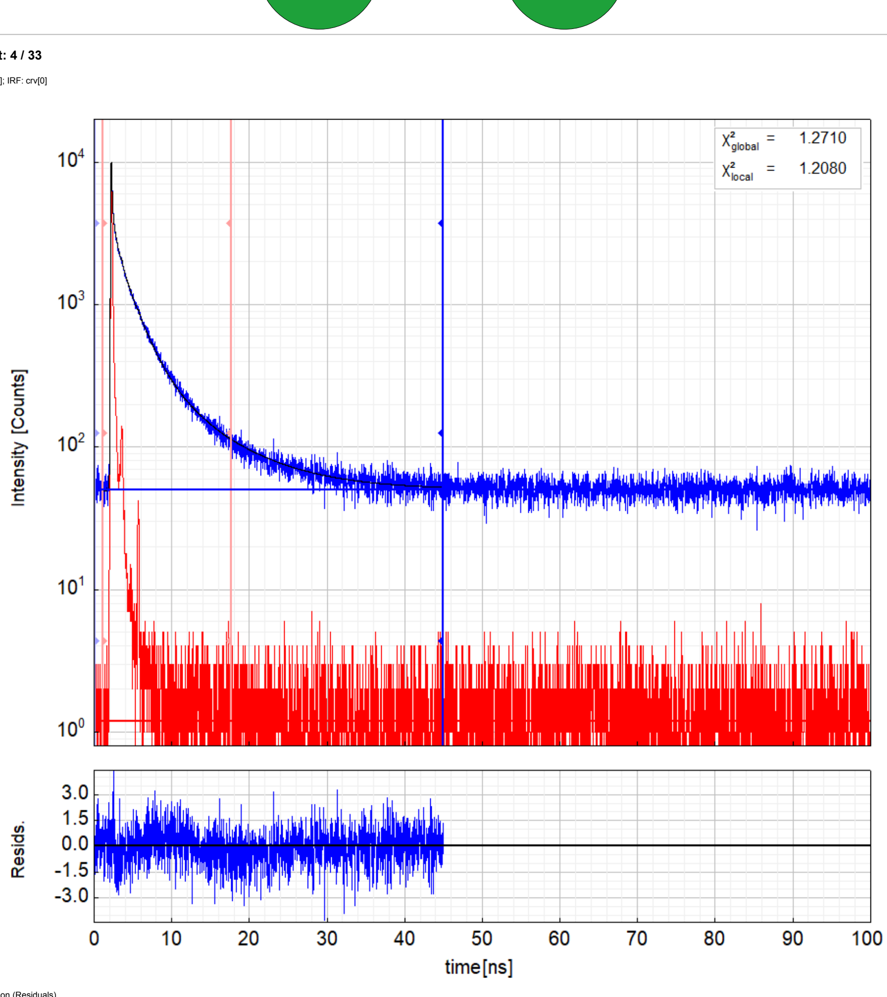

Autocorrelation (Residuals)

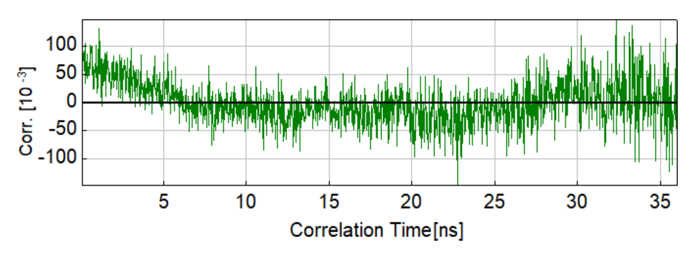

Fitted Parameters

Parameter	Value	Δ	δ
A_1[kCnts/Chnl]	2.216	±0.072	3.2%
τ_1[ns]	0.460	±0.019	4.0%
I_1[kCnts]	40.8	±2.5	6.0%
A_2[kCnts/Chnl]	2.562	±0.035	1.3%
τ_2[ns]	2.220	±0.021	0.9%
I_2[kCnts]	227.5	±1.9	0.8%
A_3[kCnts/Chnl]	0.435	±0.014	3.2%
τ_3[ns]	7.772	±0.017	0.2%
I_3[kCnts]	135.0	±4.1	3.0%
$Bkgr_{Dec}$[kCnts]	0.0504	±0.0004	0.7%
$Bkgr_{IRF}$[Cnts/Chnl]	1.20	±0.47	39%
$Shift_{IRF}$[ps]	-7.97	±0.54	6.7%
A_{Scatt}[kCnts]	67.94	±0.60	0.9%
τ_{AvInt}[ns]	3.901	±0.032	0.8%
τ_{AvAmp}[ns]	1.935	±0.029	1.5%
λ_{Det}[nm]	430.0000	---	---

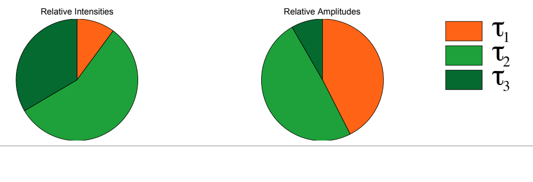

Data Set: 5 / 33

Decay: crv[5]; IRF: crv[0]

Fit

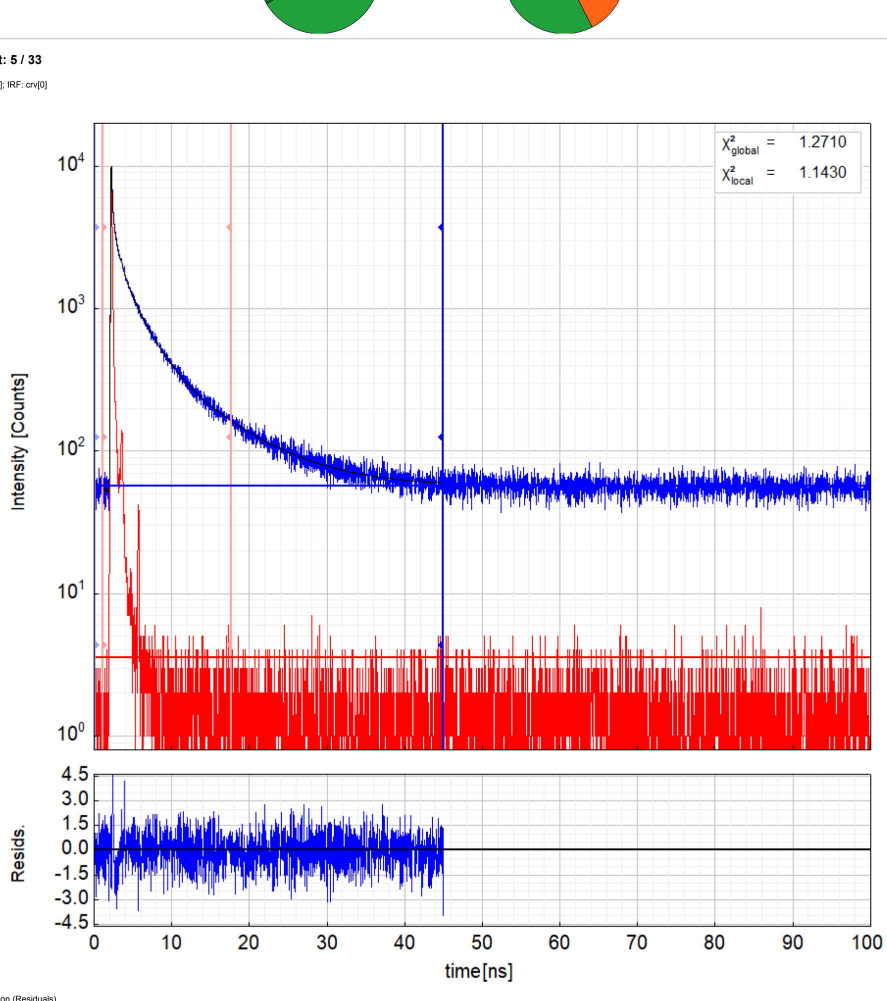

Autocorrelation (Residuals)

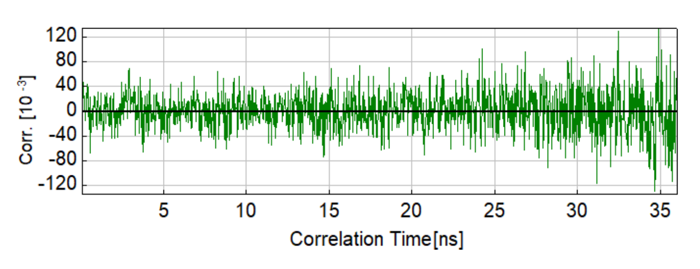

Fitted Parameters

Parameter	Value	Δ	δ
A_1[kCnts/Chnl]	3.15	±0.15	4.6%
τ_1[ns]	0.460	±0.019	4.0%
I_1[kCnts]	58.0	±2.3	3.9%
A_2[kCnts/Chnl]	1.990	±0.023	1.1%
τ_2[ns]	2.220	±0.021	0.9%
I_2[kCnts]	176.7	±2.0	1.1%
A_3[kCnts/Chnl]	0.801	±0.016	1.9%
τ_3[ns]	7.772	±0.017	0.2%
I_3[kCnts]	248.8	±4.8	1.9%
$Bkgr_{Dec}$[kCnts]	0.0567	±0.0004	0.6%
$Bkgr_{IRF}$[Cnts/Chnl]	3.53	±0.71	20%
$Shift_{IRF}$[ps]	-3.61	±0.24	6.5%
A_{Scatt}[kCnts]	65.00	±0.52	0.8%
τ_{AvInt}[ns]	4.867	±0.026	0.5%
τ_{AvAmp}[ns]	2.035	±0.055	2.7%
λ_{Det}[nm]	440.0000	---	---

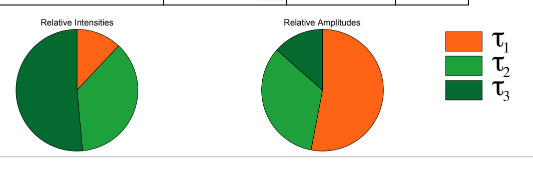

Data Set: 6 / 33

Decay: crv[6]; IRF: crv[0]

Fit

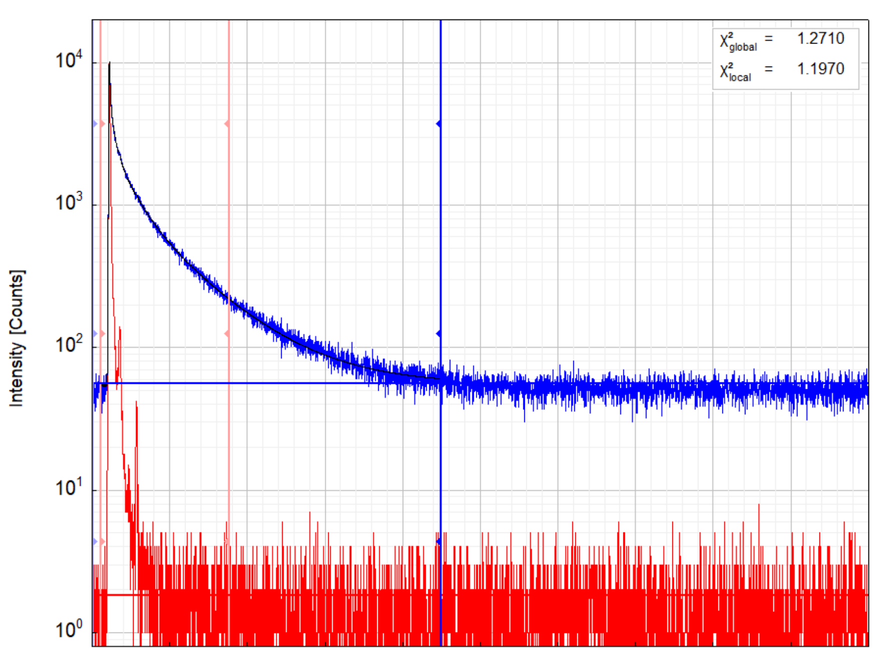

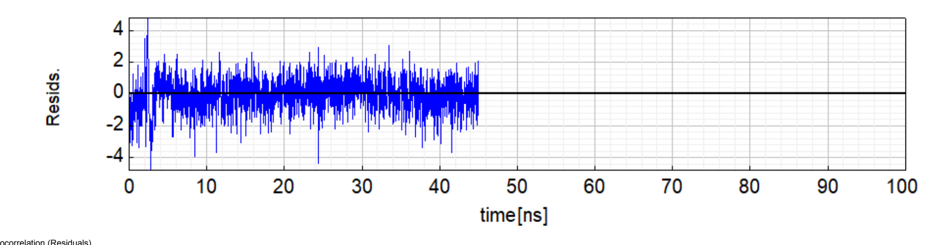

Autocorrelation (Residuals)

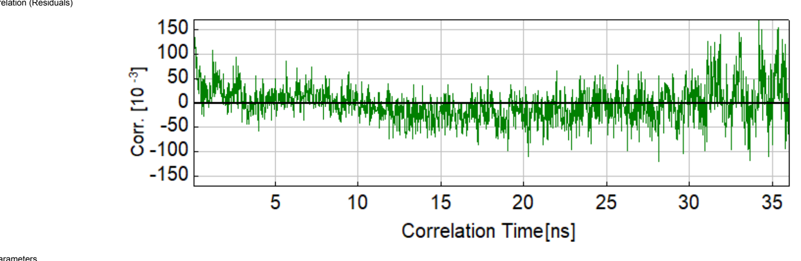

Fitted Parameters

Parameter	Value	Δ	δ
A_1[kCnts/Chnl]	3.65	±0.20	5.5%
$τ_1$[ns]	0.460	±0.019	4.0%
I_1[kCnts]	67.1	±3.4	5.0%
A_2[kCnts/Chnl]	1.597	±0.033	2.0%
$τ_2$[ns]	2.220	±0.021	0.9%
I_2[kCnts]	141.8	±2.9	2.0%
A_3[kCnts/Chnl]	1.180	±0.014	1.2%
$τ_3$[ns]	7.772	±0.017	0.2%
I_3[kCnts]	366.8	±3.8	1.0%
$Bkgr_{Dec}$[kCnts]	0.0557	±0.0002	0.4%
$Bkgr_{IRF}$[Cnts/Chnl]	1.82	±0.37	20%
$Shift_{IRF}$[ps]	-2.06	±0.60	29%
A_{Scatt}[kCnts]	63.7	±1.9	2.8%
$τ_{AvInt}$[ns]	5.553	±0.023	0.4%
$τ_{AvAmp}$[ns]	2.241	±0.055	2.4%
$λ_{Det}$[nm]	450.0000	---	---

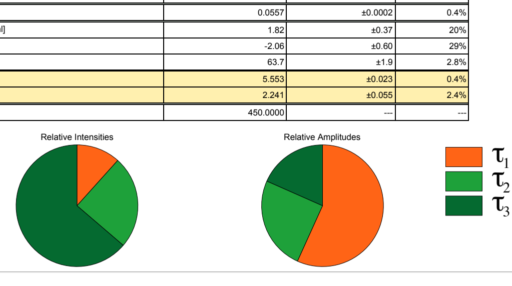

Data Set: 7 / 33

Decay: crv[7]; IRF: crv[0]

Fit

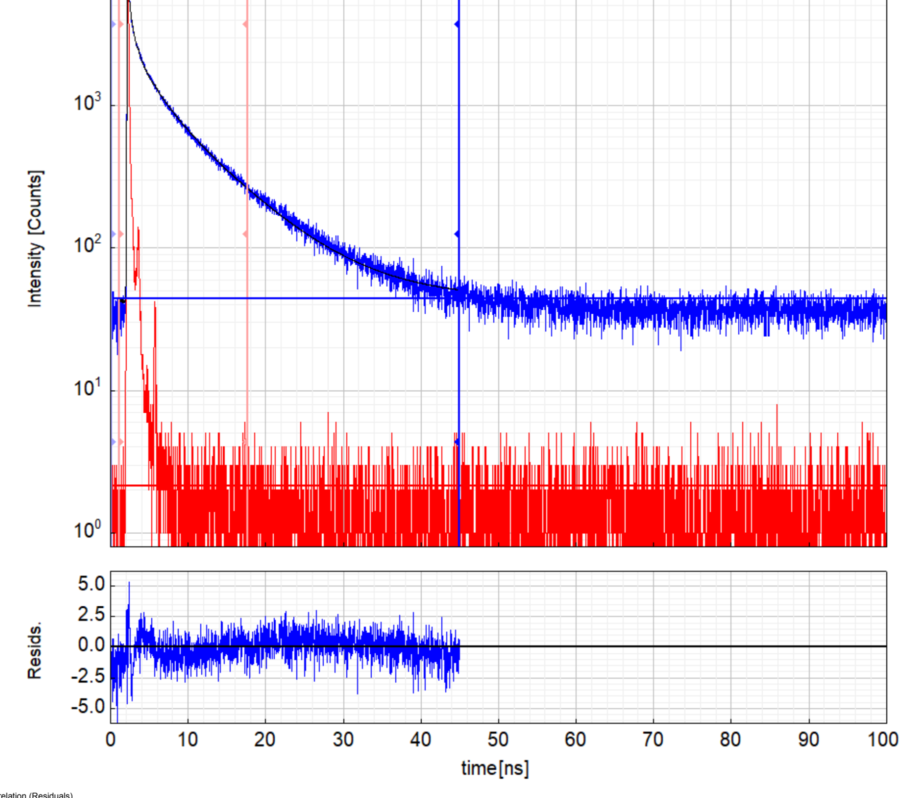

Autocorrelation (Residuals)

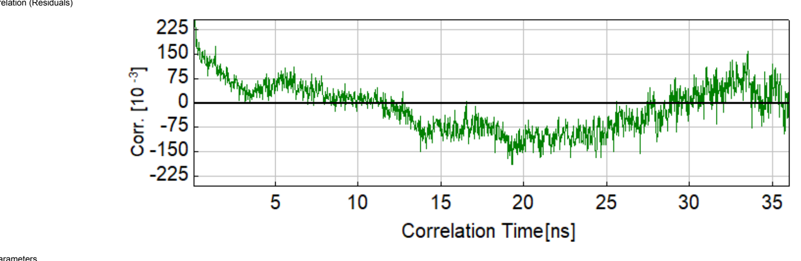

Fitted Parameters

Parameter	Value	Δ	δ
A_1[kCnts/Chnl]	4.13	±0.19	4.4%
$τ_1$[ns]	0.460	±0.019	4.0%
I_1[kCnts]	76.0	±2.8	3.6%
A_2[kCnts/Chnl]	1.412	±0.037	2.6%
$τ_2$[ns]	2.220	±0.021	0.9%
I_2[kCnts]	125.4	±3.5	2.7%
A_3[kCnts/Chnl]	1.587	±0.012	0.7%
$τ_3$[ns]	7.772	±0.017	0.2%
I_3[kCnts]	493.2	±4.6	0.9%
$Bkgr_{Dec}$[kCnts]	0.0444	±0.0003	0.6%
$Bkgr_{IRF}$[Cnts/Chnl]	2.13	±0.45	21%
$Shift_{IRF}$[ps]	-1.8	±1.1	61%
A_{Scatt}[kCnts]	61.2	±1.2	1.8%
$τ_{AvInt}$[ns]	5.970	±0.035	0.6%
$τ_{AvAmp}$[ns]	2.436	±0.045	1.8%
$λ_{Det}$[nm]	460.0000	---	---

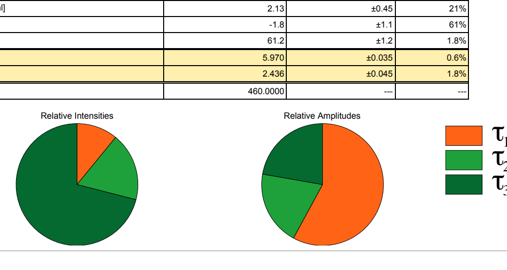

Data Set: 8 / 33

Decay: crv[8]; IRF: crv[0]

Fit

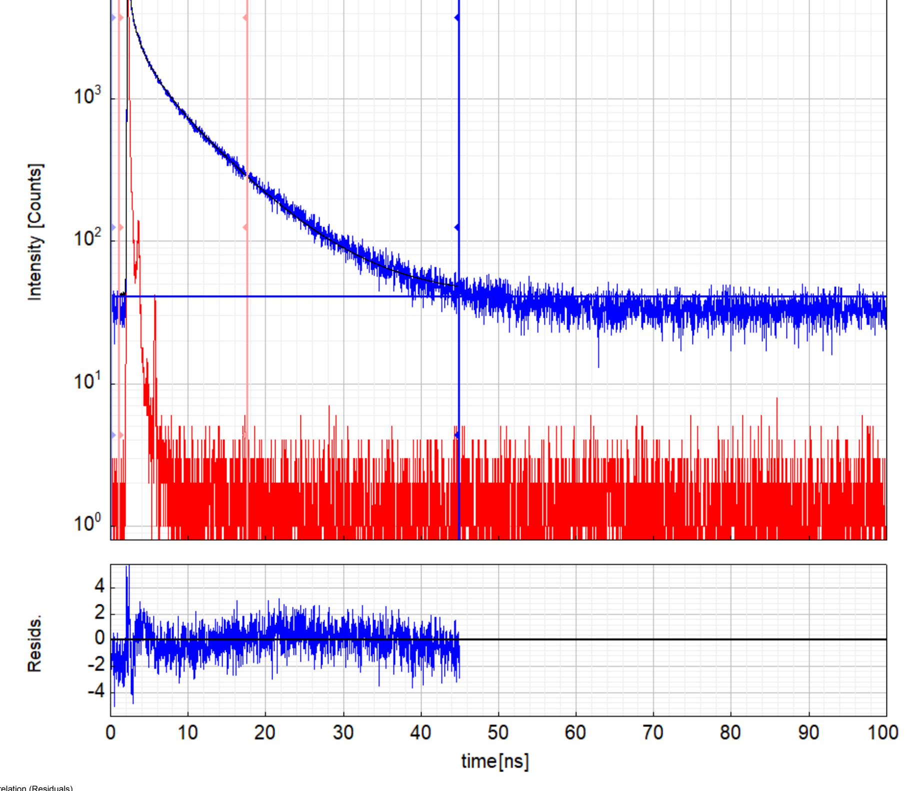

Autocorrelation (Residuals)

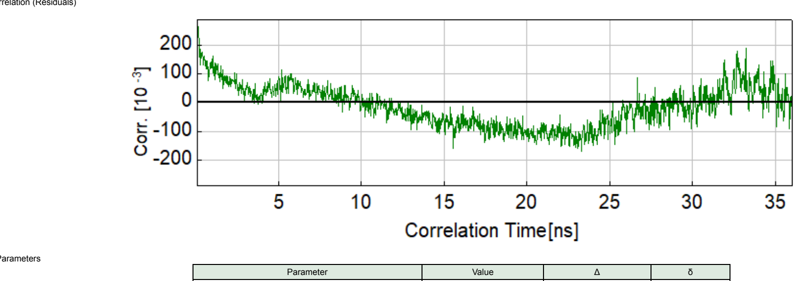

Fitted Parameters

Parameter	Value	Δ	δ
A_1[kCnts/Chnl]	4.41	±0.20	4.5%
$τ_1$[ns]	0.460	±0.019	4.0%
I_1[kCnts]	81.0	±3.0	3.6%
A_2[kCnts/Chnl]	1.654	±0.058	3.5%
$τ_2$[ns]	2.220	±0.021	0.9%
I_2[kCnts]	146.9	±4.2	2.8%
A_3[kCnts/Chnl]	1.712	±0.018	1.0%
$τ_3$[ns]	7.772	±0.017	0.2%
I_3[kCnts]	532.1	±5.6	1.0%
$Bkgr_{Dec}$[kCnts]	0.0411	±0.0004	0.8%
$Bkgr_{IRF}$[Cnts/Chnl]	0.41	±0.71	175%
$Shift_{IRF}$[ps]	0.9	±3.5	392%
A_{Scatt}[kCnts]	60.0	±2.0	3.3%
$τ_{AvInt}$[ns]	5.920	±0.035	0.6%
$τ_{AvAmp}$[ns]	2.446	±0.060	2.4%
$λ_{Det}$[nm]	470.0000	---	---

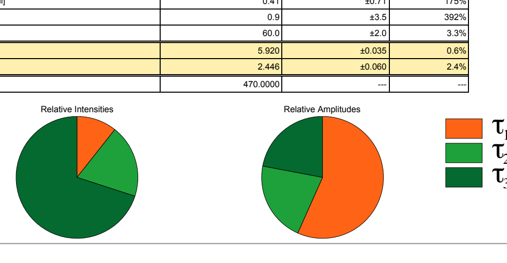

Data Set: 9 / 33

Decay: crv[9]; IRF: crv[0]

Fit

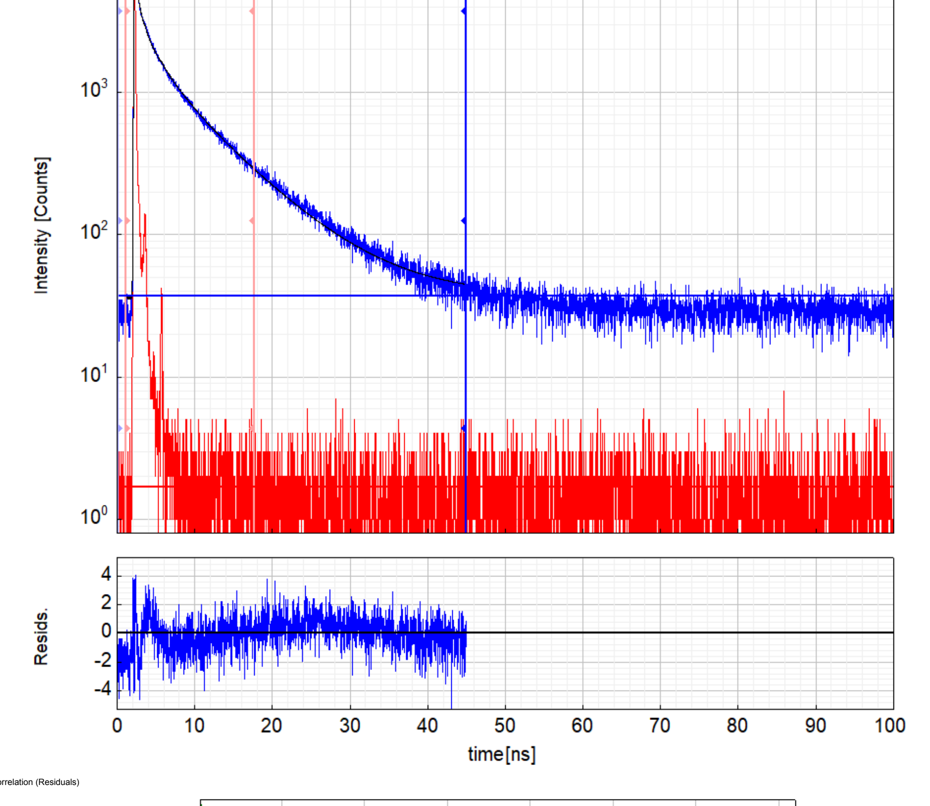

Autocorrelation (Residuals)

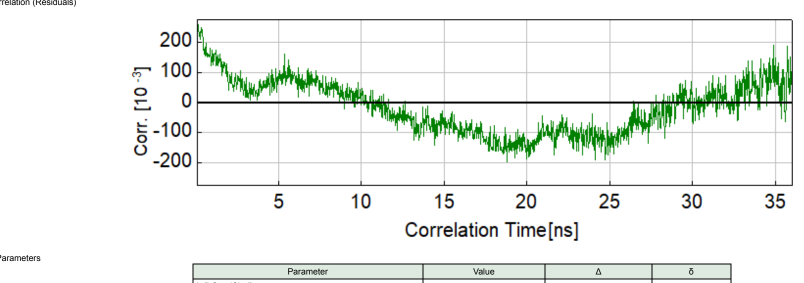

Fitted Parameters

Parameter	Value	Δ	δ
A_1[kCnts/Chnl]	4.47	±0.22	4.7%
$τ_1$[ns]	0.460	±0.019	4.0%
I_1[kCnts]	82.1	±1.2	1.4%
A_2[kCnts/Chnl]	1.902	±0.042	2.2%
$τ_2$[ns]	2.220	±0.021	0.9%
I_2[kCnts]	168.9	±3.1	1.8%
A_3[kCnts/Chnl]	1.8196	±0.0078	0.4%
$τ_3$[ns]	7.772	±0.017	0.2%
I_3[kCnts]	565.6	±2.7	0.5%
$Bkgr_{Dec}$[kCnts]	0.0374	±0.0004	0.9%
$Bkgr_{IRF}$[Cnts/Chnl]	1.70	±0.16	9.0%
$Shift_{IRF}$[ps]	1.4	±1.6	115%
A_{Scatt}[kCnts]	57.9	±2.0	3.3%
$τ_{AvInt}$[ns]	5.889	±0.021	0.4%
$τ_{AvAmp}$[ns]	2.495	±0.082	3.3%
$λ_{Det}$[nm]	480.0000	---	---

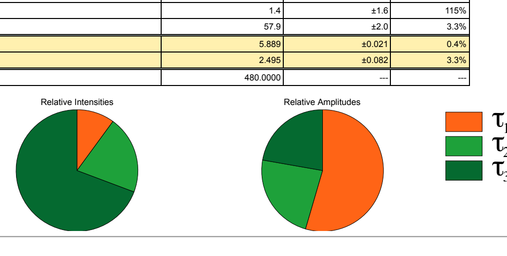

Data Set: 10 / 33

Decay: crv[10]; IRF: crv[0]

Fit

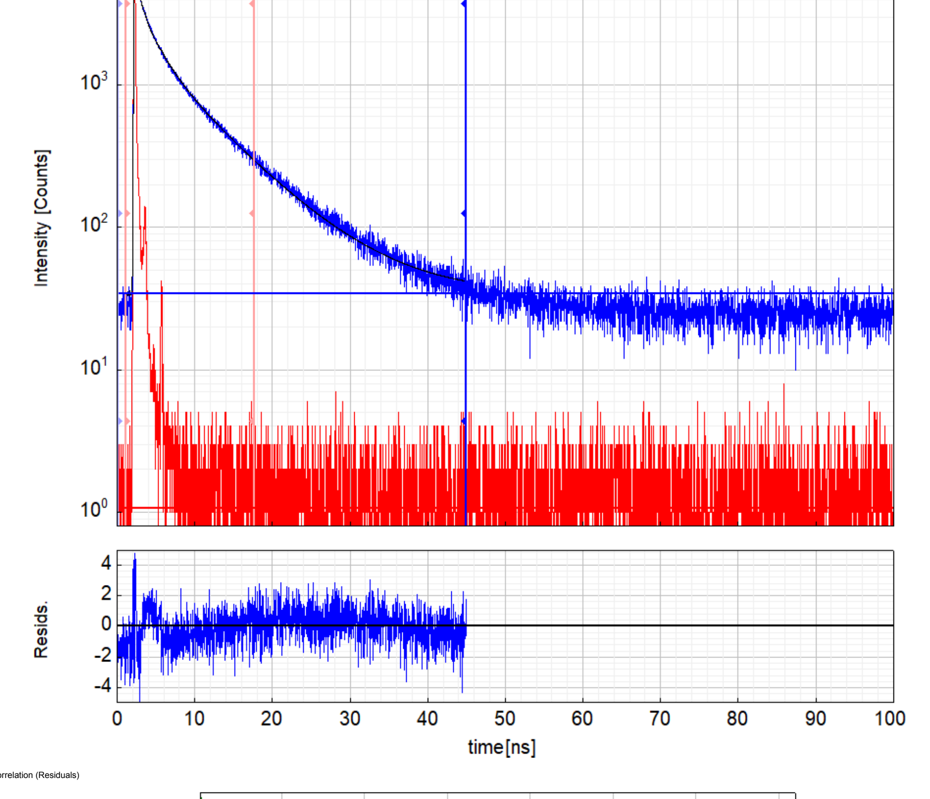

Autocorrelation (Residuals)

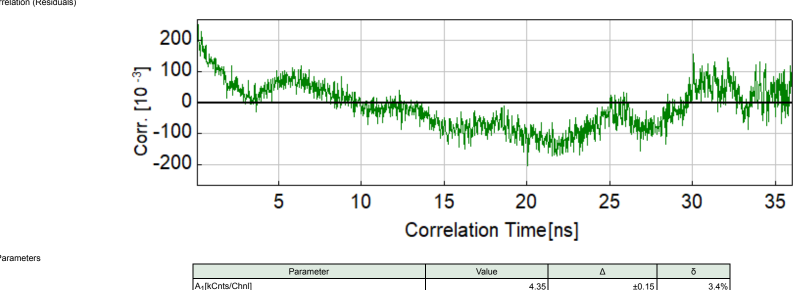

Fitted Parameters

Parameter	Value	Δ	δ
A_1[kCnts/Chnl]	4.35	±0.15	3.4%
$τ_1$[ns]	0.460	±0.019	4.0%
I_1[kCnts]	79.9	±2.3	2.8%
A_2[kCnts/Chnl]	2.272	±0.065	2.8%
$τ_2$[ns]	2.220	±0.021	0.9%
I_2[kCnts]	201.8	±4.8	2.4%
A_3[kCnts/Chnl]	1.860	±0.012	0.6%
$τ_3$[ns]	7.772	±0.017	0.2%
I_3[kCnts]	578.0	±2.9	0.5%
$Bkgr_{Dec}$[kCnts]	0.0342	±0.0003	0.7%
$Bkgr_{IRF}$[Cnts/Chnl]	1.06	±0.37	35%
$Shift_{IRF}$[ps]	2.33	±0.94	40%
A_{Scatt}[kCnts]	56.2	±1.2	2.0%
$τ_{AvInt}$[ns]	5.789	±0.029	0.5%
$τ_{AvAmp}$[ns]	2.536	±0.050	2.0%
$λ_{Det}$[nm]	490.0000	---	---

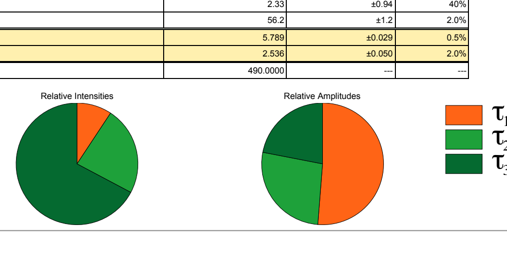

Data Set: 11 / 33

Decay: crv[11]; IRF: crv[0]

Fit

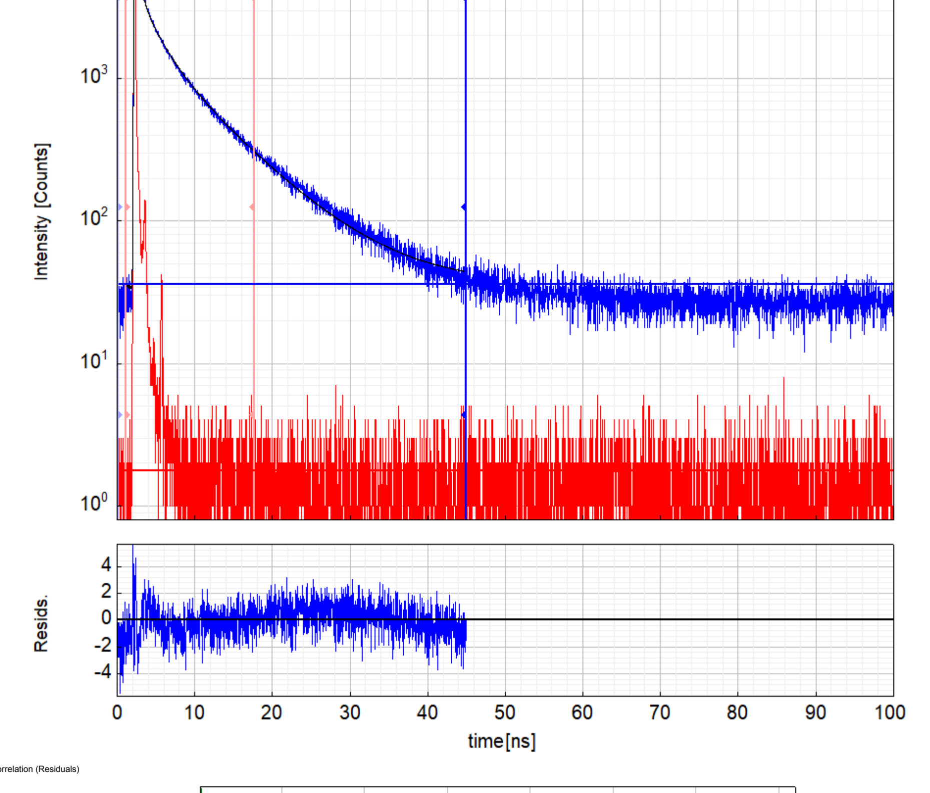

Autocorrelation (Residuals)

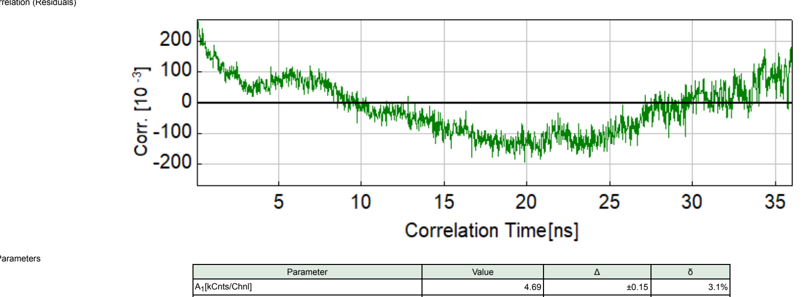

Fitted Parameters

Parameter	Value	Δ	δ
A_1[kCnts/Chnl]	4.69	±0.15	3.1%
$τ_1$[ns]	0.460	±0.019	4.0%
I_1[kCnts]	86.2	±2.9	3.3%
A_2[kCnts/Chnl]	2.590	±0.042	1.6%
$τ_2$[ns]	2.220	±0.021	0.9%
I_2[kCnts]	230.0	±3.3	1.4%
A_3[kCnts/Chnl]	1.962	±0.020	1.0%
$τ_3$[ns]	7.772	±0.017	0.2%
I_3[kCnts]	609.7	±5.6	0.9%
$Bkgr_{Dec}$[kCnts]	0.0361	±0.0003	0.8%
$Bkgr_{IRF}$[Cnts/Chnl]	1.76	±0.40	22%
$Shift_{IRF}$[ps]	0.5	±1.2	256%
A_{Scatt}[kCnts]	52.2	±1.9	3.6%
$τ_{AvInt}$[ns]	5.712	±0.031	0.5%
$τ_{AvAmp}$[ns]	2.507	±0.057	2.3%
$λ_{Det}$[nm]	500.0000	---	---

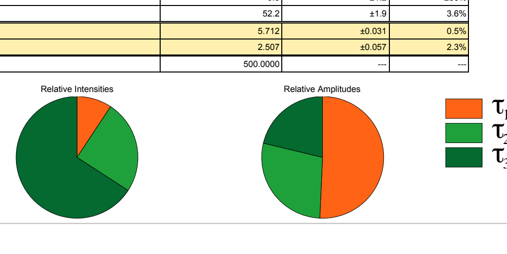

Data Set: 12 / 33

Decay: crv[12]; IRF: crv[0]

Fit

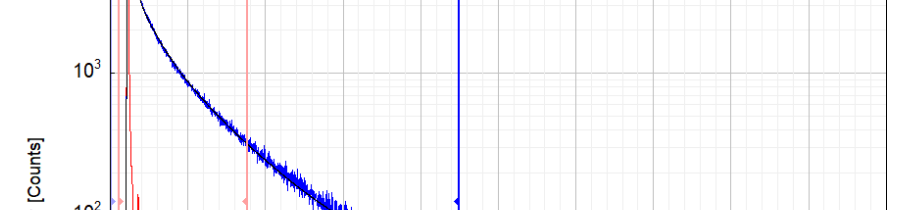

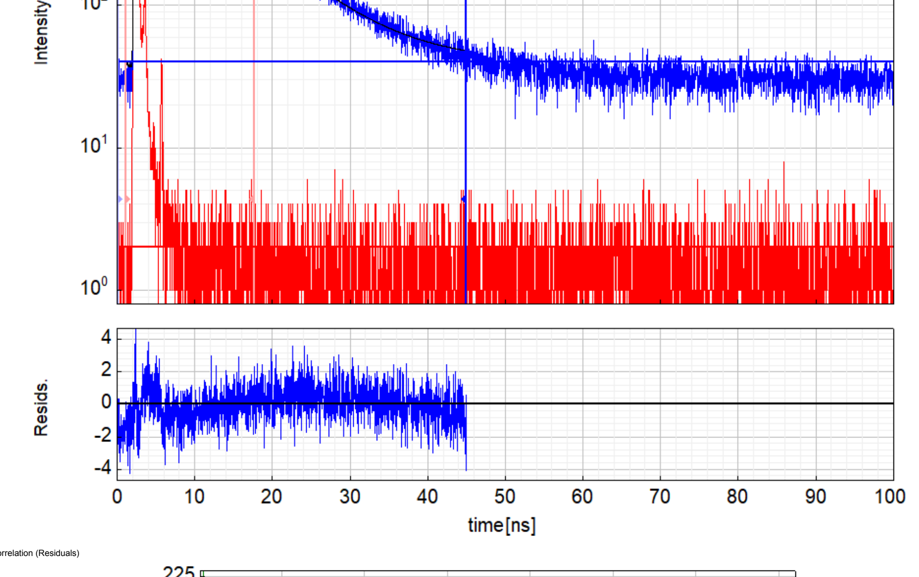

Autocorrelation (Residuals)

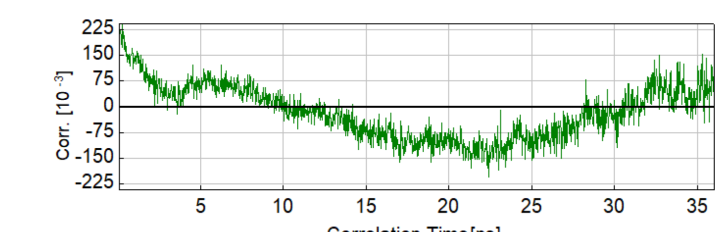

Fitted Parameters

Parameter	Value	Δ	δ
A_1[kCnts/Chnl]	4.802	±0.099	2.1%
τ_1[ns]	0.460	±0.019	4.0%
I_1[kCnts]	88.4	±3.2	3.6%
A_2[kCnts/Chnl]	2.846	±0.044	1.5%
τ_2[ns]	2.220	±0.021	0.9%
I_2[kCnts]	252.8	±3.1	1.2%
A_3[kCnts/Chnl]	2.012	±0.020	1.0%
τ_3[ns]	7.772	±0.017	0.2%
I_3[kCnts]	625.3	±5.3	0.8%
$Bkgr_{Dec}$[kCnts]	0.0399	±0.0003	0.6%
$Bkgr_{IRF}$[Cnts/Chnl]	2.03	±0.38	19%
$Shift_{IRF}$[ps]	-1.2	±3.4	302%
A_{Scatt}[kCnts]	49.1	±2.9	5.7%
τ_{AvInt}[ns]	5.652	±0.018	0.3%
τ_{AvAmp}[ns]	2.501	±0.068	2.7%
λ_{Det}[nm]	510.0000	---	---

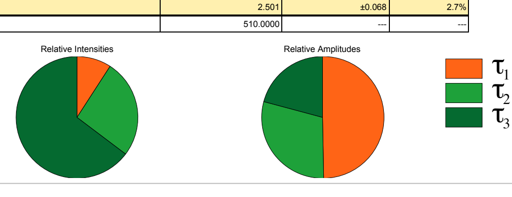

Data Set: 13 / 33

Decay: crv[13]; IRF: crv[0]

Fit

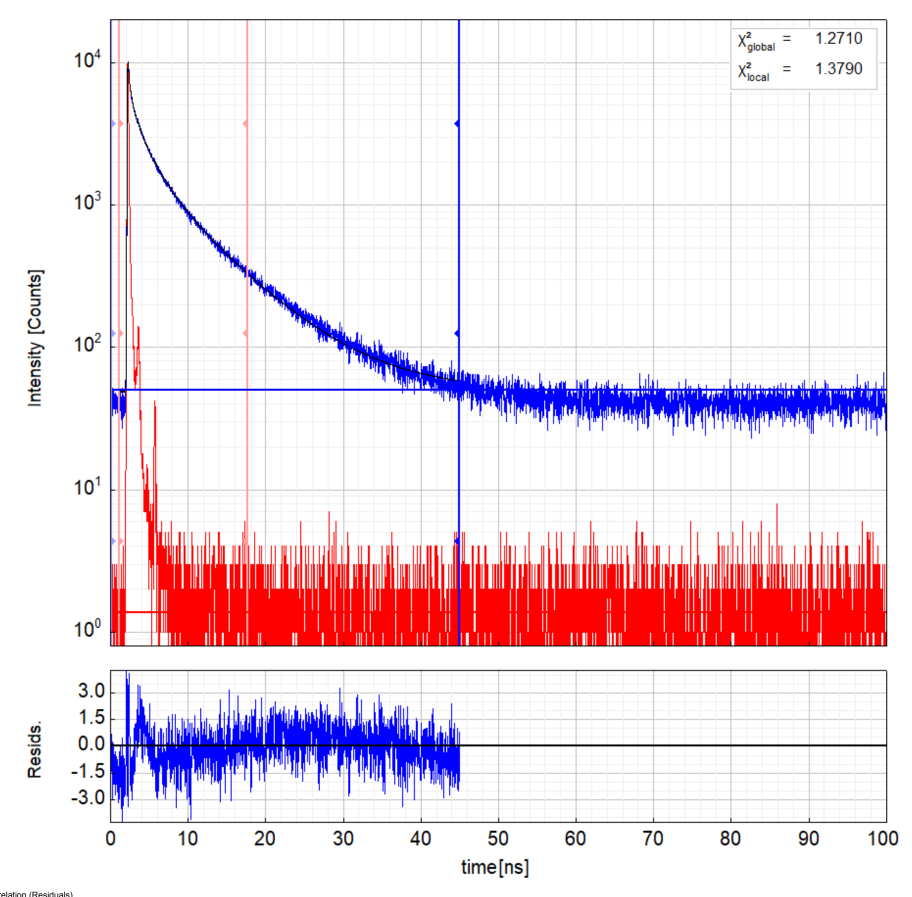

Autocorrelation (Residuals)

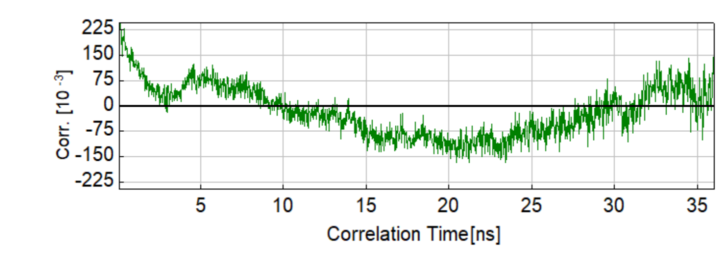

Fitted Parameters

Parameter	Value	Δ	δ
A_1[kCnts/Chnl]	4.437	±0.089	2.0%
τ_1[ns]	0.460	±0.019	4.0%
I_1[kCnts]	81.6	±2.7	3.2%
A_2[kCnts/Chnl]	3.045	±0.098	3.2%
τ_2[ns]	2.220	±0.021	0.9%
I_2[kCnts]	270.4	±5.2	1.9%
A_3[kCnts/Chnl]	2.005	±0.016	0.8%
τ_3[ns]	7.772	±0.017	0.2%
I_3[kCnts]	623.1	±4.9	0.8%
$Bkgr_{Dec}$[kCnts]	0.0501	±0.0003	0.6%
$Bkgr_{IRF}$[Cnts/Chnl]	1.38	±0.39	28%
$Shift_{IRF}$[ps]	2.3	±1.9	84%
A_{Scatt}[kCnts]	50.1	±1.9	3.8%
τ_{AvInt}[ns]	5.620	±0.023	0.4%
τ_{AvAmp}[ns]	2.570	±0.039	1.5%
λ_{Det}[nm]	520.0000	---	---

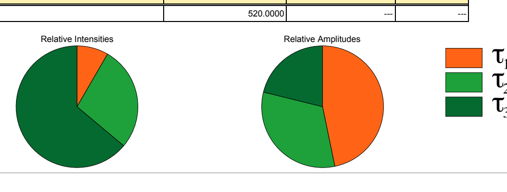

Data Set: 14 / 33

Decay: crv[14]; IRF: crv[0]

Fit

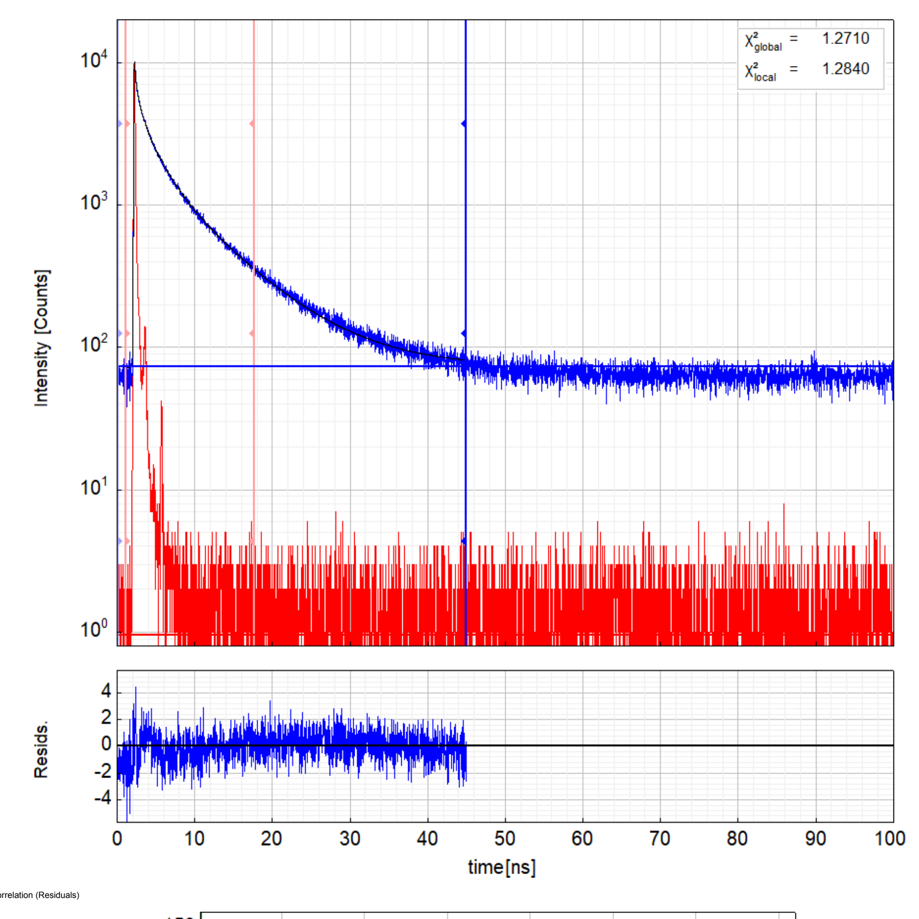

Autocorrelation (Residuals)

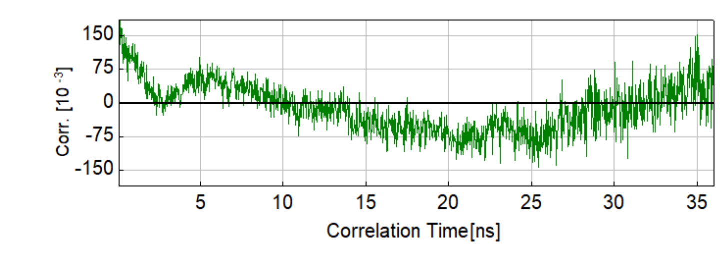

Fitted Parameters

Parameter	Value	Δ	δ
A_1[kCnts/Chnl]	4.52	±0.13	2.7%
τ_1[ns]	0.460	±0.019	4.0%
I_1[kCnts]	83.0	±3.5	4.1%
A_2[kCnts/Chnl]	3.184	±0.058	1.8%
τ_2[ns]	2.220	±0.021	0.9%
I_2[kCnts]	282.8	±4.0	1.4%
A_3[kCnts/Chnl]	1.995	±0.025	1.2%
τ_3[ns]	7.772	±0.017	0.2%
I_3[kCnts]	620.1	±6.4	1.0%
$Bkgr_{Dec}$[kCnts]	0.0730	±0.0005	0.6%
$Bkgr_{IRF}$[Cnts/Chnl]	0.96	±0.30	31%
$Shift_{IRF}$[ps]	4.5	±1.2	26%
A_{Scatt}[kCnts]	48.9	±1.3	2.6%
τ_{AvInt}[ns]	5.564	±0.023	0.4%
τ_{AvAmp}[ns]	2.544	±0.040	1.6%
λ_{Det}[nm]	530.0000	---	---

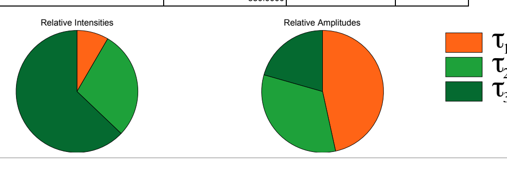

Data Set: 15 / 33

Decay: crv[15]; IRF: crv[0]

Fit

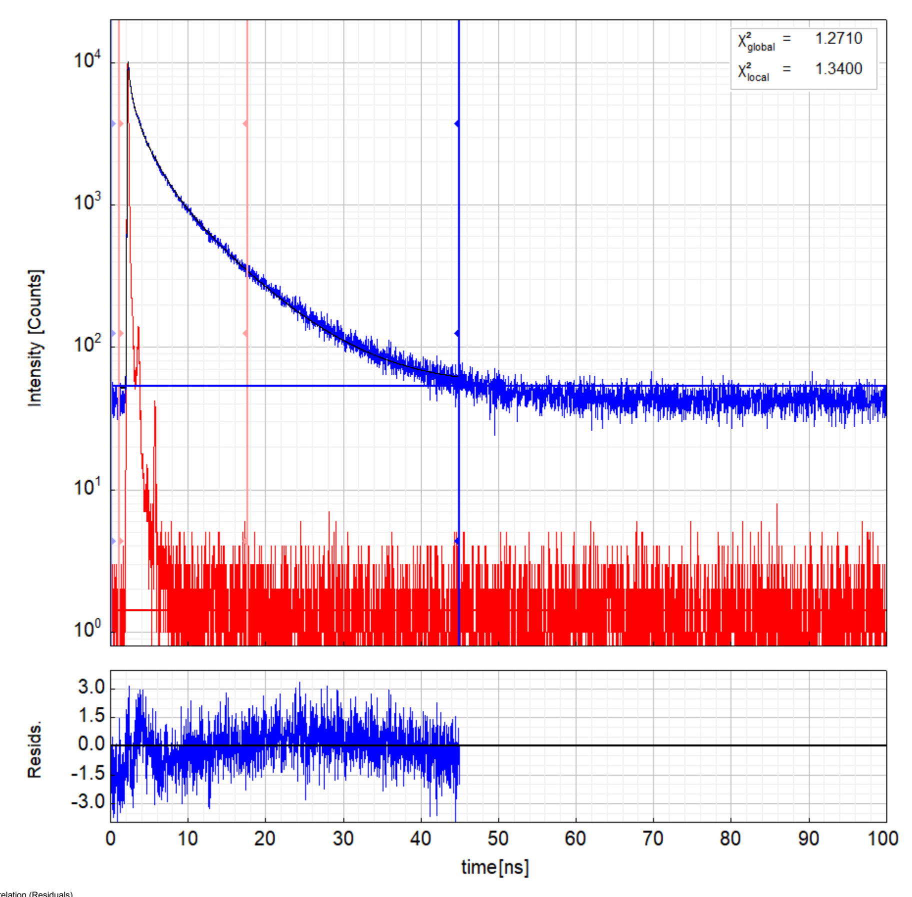

Autocorrelation (Residuals)

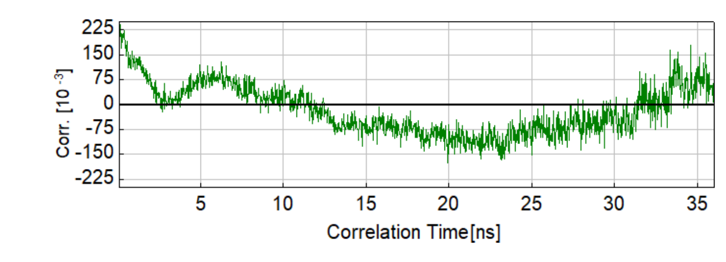

Fitted Parameters

Parameter	Value	Δ	δ
A_1[kCnts/Chnl]	4.72	±0.16	3.2%
τ_1[ns]	0.460	±0.019	4.0%
I_1[kCnts]	86.7	±1.9	2.1%
A_2[kCnts/Chnl]	3.411	±0.066	1.9%
τ_2[ns]	2.220	±0.021	0.9%
I_2[kCnts]	302.9	±4.4	1.4%
A_3[kCnts/Chnl]	2.080	±0.023	1.1%
τ_3[ns]	7.772	±0.017	0.2%
I_3[kCnts]	646.5	±5.7	0.9%
$Bkgr_{Dec}$[kCnts]	0.0533	±0.0003	0.5%
$Bkgr_{IRF}$[Cnts/Chnl]	1.42	±0.37	26%
$Shift_{IRF}$[ps]	3.1	±1.3	40%
A_{Scatt}[kCnts]	47.4	±1.1	2.2%
τ_{AvInt}[ns]	5.537	±0.014	0.2%
τ_{AvAmp}[ns]	2.539	±0.039	1.5%
λ_{Det}[nm]	540.0000	---	---

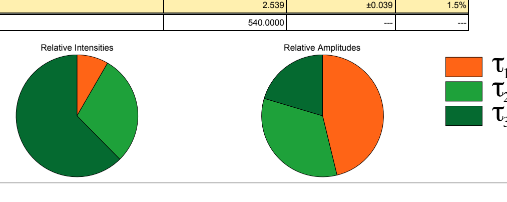

Data Set: 16 / 33

Decay: crv[16]; IRF: crv[0]

Fit

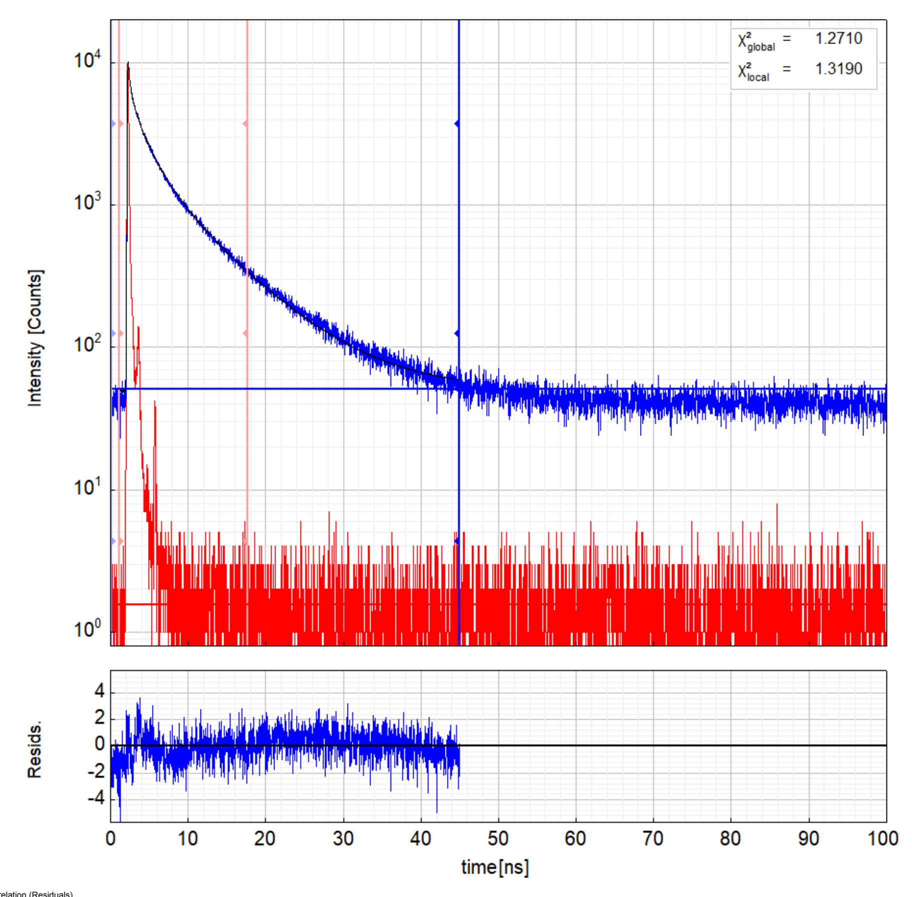

Autocorrelation (Residuals)

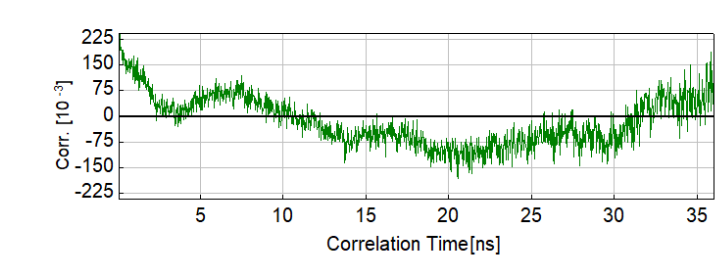

Fitted Parameters

Parameter	Value	Δ	δ
A_1[kCnts/Chnl]	4.504	±0.073	1.6%
τ_1[ns]	0.460	±0.019	4.0%
I_1[kCnts]	82.9	±3.1	3.6%
A_2[kCnts/Chnl]	3.487	±0.057	1.6%
τ_2[ns]	2.220	±0.021	0.9%
I_2[kCnts]	309.6	±3.4	1.1%
A_3[kCnts/Chnl]	2.090	±0.013	0.6%
τ_3[ns]	7.772	±0.017	0.2%
I_3[kCnts]	649.5	±4.1	0.6%
$Bkgr_{Dec}$[kCnts]	0.0506	±0.0005	0.9%
$Bkgr_{IRF}$[Cnts/Chnl]	1.55	±0.65	41%
$Shift_{IRF}$[ps]	4.9	±1.2	24%
A_{Scatt}[kCnts]	47.5	±1.1	2.3%
τ_{AvInt}[ns]	5.541	±0.030	0.5%
τ_{AvAmp}[ns]	2.585	±0.031	1.2%
λ_{Det}[nm]	550.0000	---	---

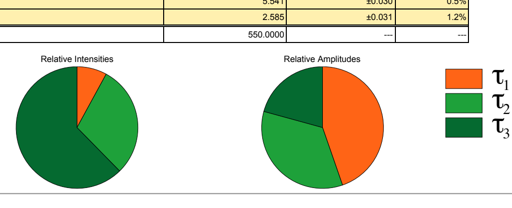

Data Set: 17 / 33

Decay: crv[17]; IRF: crv[0]

Fit

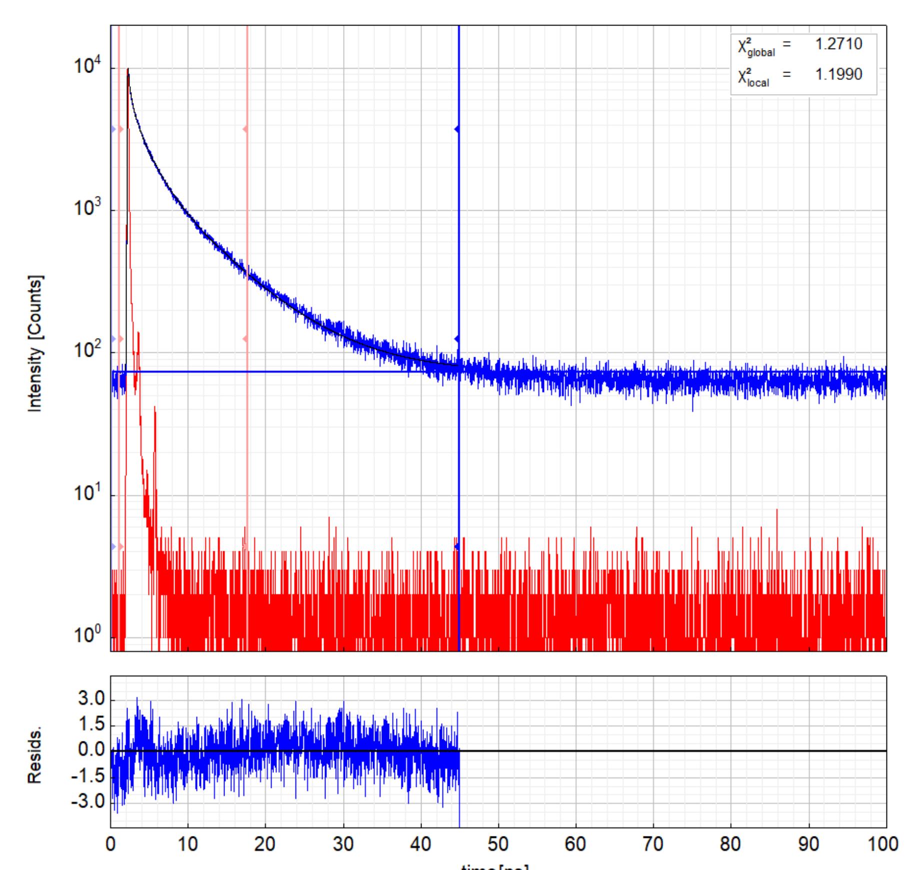

Autocorrelation (Residuals)

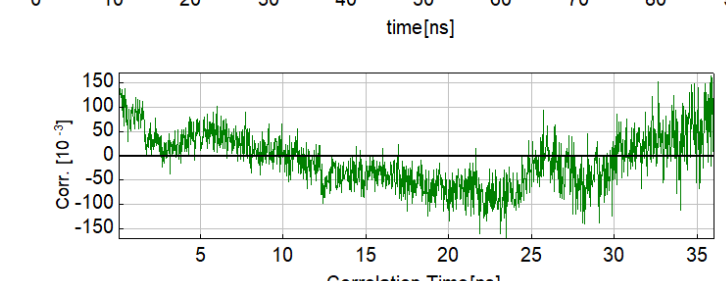

Fitted Parameters

Parameter	Value	Δ	δ
A_1[kCnts/Chnl]	4.468	±0.060	1.3%
τ_1[ns]	0.460	±0.019	4.0%
I_1[kCnts]	82.2	±3.7	4.4%
A_2[kCnts/Chnl]	3.523	±0.072	2.0%
τ_2[ns]	2.220	±0.021	0.9%
I_2[kCnts]	312.9	±4.7	1.5%
A_3[kCnts/Chnl]	2.022	±0.019	0.9%
τ_3[ns]	7.772	±0.017	0.2%
I_3[kCnts]	628.5	±6.0	0.9%
$Bkgr_{Dec}$[kCnts]	0.0728	±0.0005	0.7%
$Bkgr_{IRF}$[Cnts/Chnl]	0.64	±0.56	88%
$Shift_{IRF}$[ps]	4.8	±1.1	23%
A_{Scatt}[kCnts]	46.7	±1.1	2.3%
τ_{AvInt}[ns]	5.488	±0.021	0.4%
τ_{AvAmp}[ns]	2.556	±0.033	1.3%
λ_{Det}[nm]	560.0000	---	---

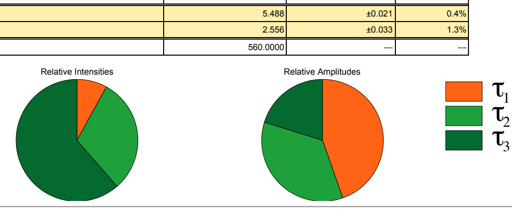

Data Set: 18 / 33

Decay: crv[18]; IRF: crv[0]

Fit

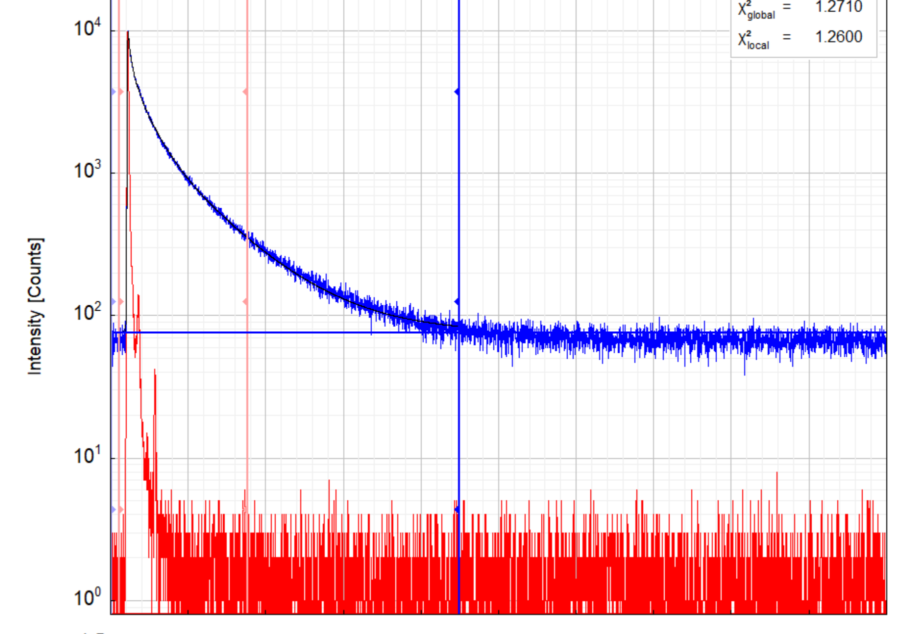

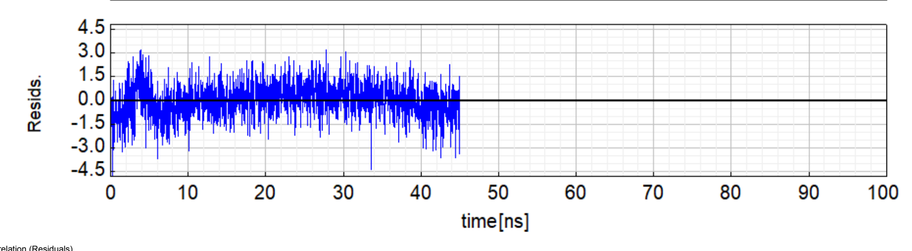

Autocorrelation (Residuals)

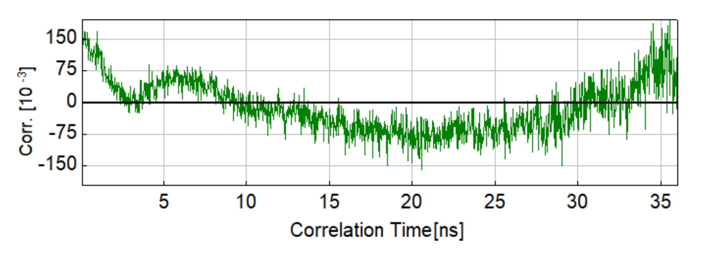

Fitted Parameters

Parameter	Value	Δ	δ
A_1[kCnts/Chnl]	4.56	±0.13	2.7%
τ_1[ns]	0.460	±0.019	4.0%
I_1[kCnts]	83.8	±3.2	3.7%
A_2[kCnts/Chnl]	3.444	±0.053	1.5%
τ_2[ns]	2.220	±0.021	0.9%
I_2[kCnts]	305.8	±2.6	0.8%
A_3[kCnts/Chnl]	1.939	±0.017	0.9%
τ_3[ns]	7.772	±0.017	0.2%
I_3[kCnts]	602.5	±3.8	0.6%
$Bkgr_{Dec}$[kCnts]	0.0760	±0.0005	0.6%
$Bkgr_{IRF}$[Cnts/Chnl]	0.81	±0.32	39%
$Shift_{IRF}$[ps]	5.0	±1.5	29%
A_{Scatt}[kCnts]	45.6	±1.1	2.4%
τ_{AvInt}[ns]	5.444	±0.014	0.3%
τ_{AvAmp}[ns]	2.497	±0.032	1.2%
λ_{Det}[nm]	570.0000	---	---

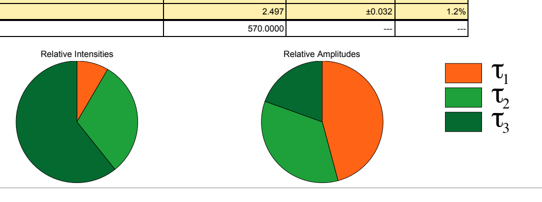

Data Set: 19 / 33

Decay: crv[19]; IRF: crv[0]

Fit

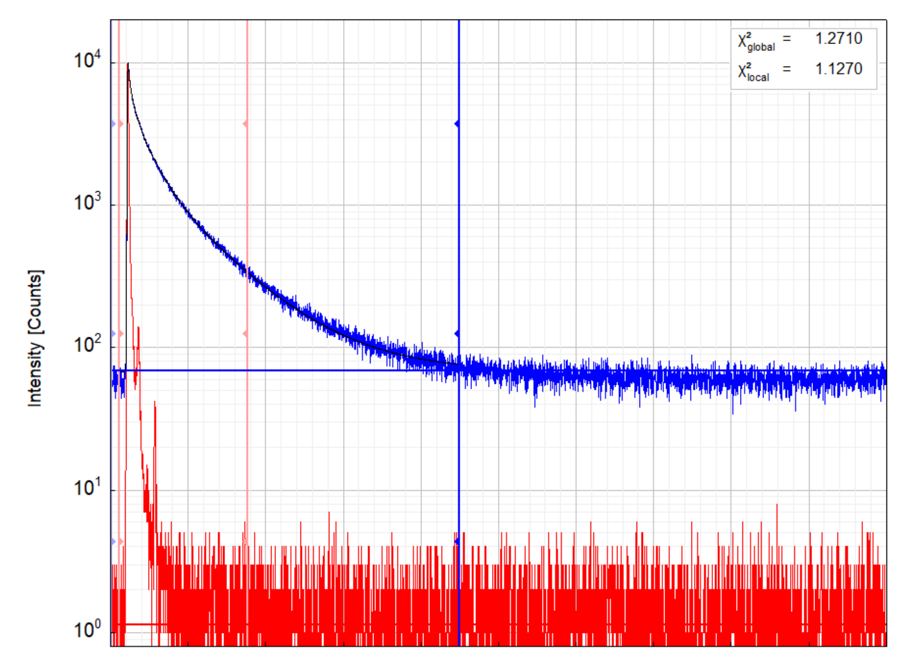

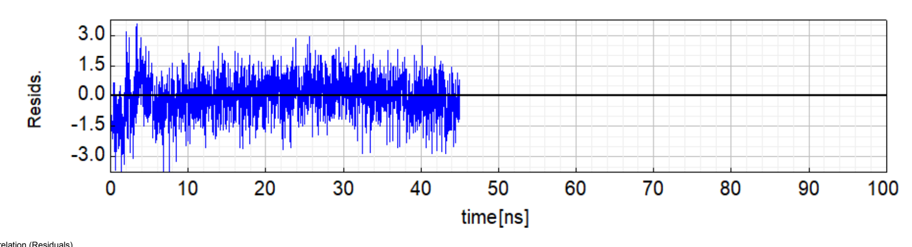

Autocorrelation (Residuals)

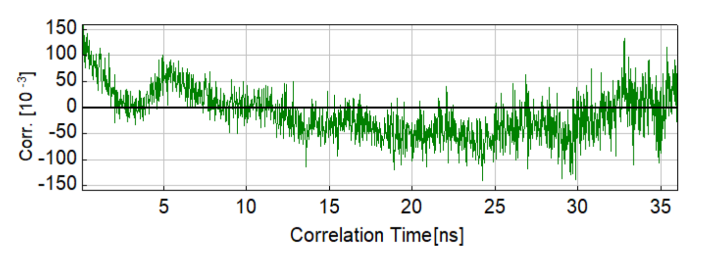

Fitted Parameters

Parameter	Value	Δ	δ
A_1[kCnts/Chnl]	4.60	±0.13	2.6%
τ_1[ns]	0.460	±0.019	4.0%
I_1[kCnts]	84.6	±3.0	3.5%
A_2[kCnts/Chnl]	3.502	±0.054	1.5%
τ_2[ns]	2.220	±0.021	0.9%
I_2[kCnts]	310.9	±4.2	1.3%
A_3[kCnts/Chnl]	1.906	±0.030	1.5%
τ_3[ns]	7.772	±0.017	0.2%
I_3[kCnts]	592.3	±9.9	1.7%
$Bkgr_{Dec}$[kCnts]	0.0684	±0.0003	0.4%
$Bkgr_{IRF}$[Cnts/Chnl]	1.15	±0.80	70%
$Shift_{IRF}$[ps]	5.1	±1.1	21%
A_{Scatt}[kCnts]	46.7	±1.8	3.8%
τ_{AvInt}[ns]	5.398	±0.027	0.5%
τ_{AvAmp}[ns]	2.469	±0.036	1.4%
λ_{Det}[nm]	580.0000	---	---

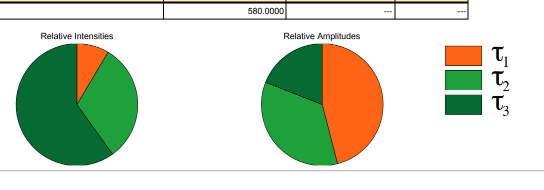

Data Set: 20 / 33

Decay: crv[20]; IRF: crv[0]

Fit

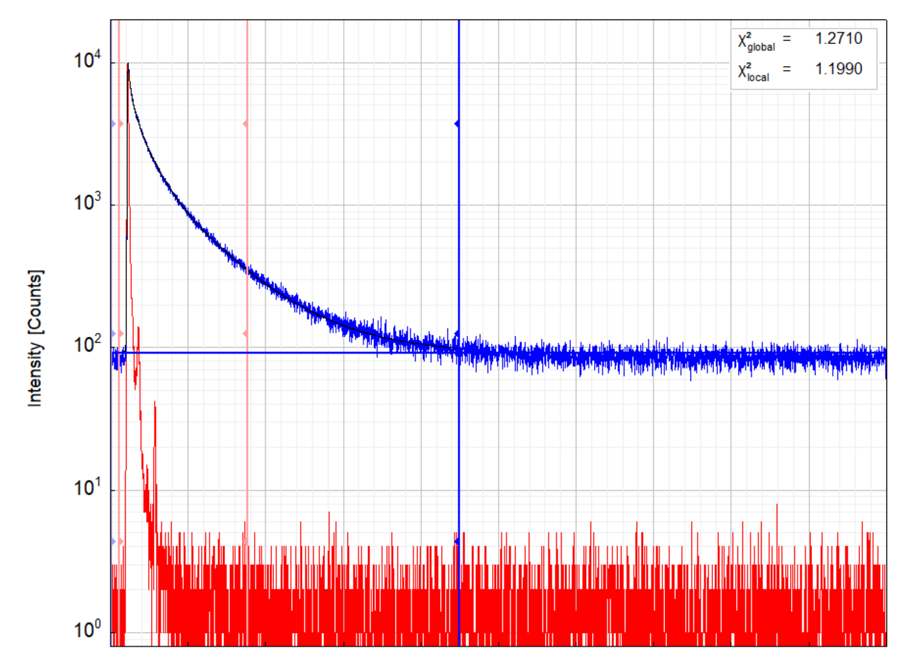

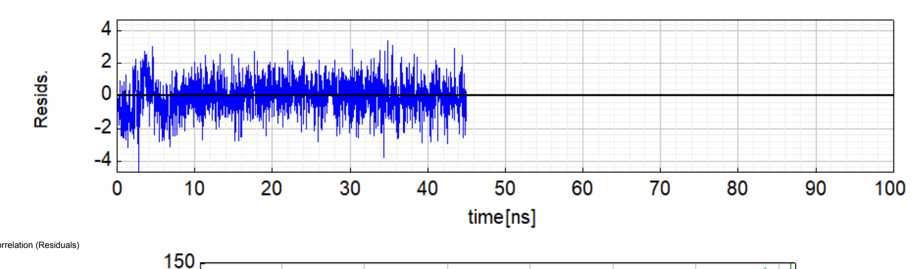

Autocorrelation (Residuals)

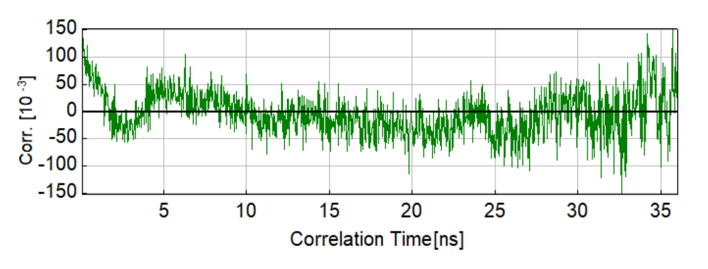

Fitted Parameters

Parameter	Value	Δ	δ
A_1[kCnts/Chnl]	4.743	±0.072	1.5%
τ_1[ns]	0.460	±0.019	4.0%
I_1[kCnts]	87.3	±3.5	4.0%
A_2[kCnts/Chnl]	3.479	±0.096	2.7%
τ_2[ns]	2.220	±0.021	0.9%
I_2[kCnts]	308.9	±6.0	1.9%
A_3[kCnts/Chnl]	1.787	±0.020	1.1%
τ_3[ns]	7.772	±0.017	0.2%
I_3[kCnts]	555.5	±4.7	0.8%
$Bkgr_{Dec}$[kCnts]	0.0916	±0.0004	0.4%
$Bkgr_{IRF}$[Cnts/Chnl]	0.15	±0.29	198%
$Shift_{IRF}$[ps]	6.49	±0.94	14%
A_{Scatt}[kCnts]	46.2	±1.3	2.7%
τ_{AvInt}[ns]	5.299	±0.015	0.3%
τ_{AvAmp}[ns]	2.377	±0.029	1.2%
λ_{Det}[nm]	590.0000	---	---

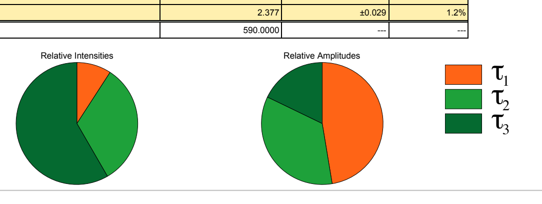

Data Set: 21 / 33

Decay: crv[21]; IRF: crv[0]

Fit

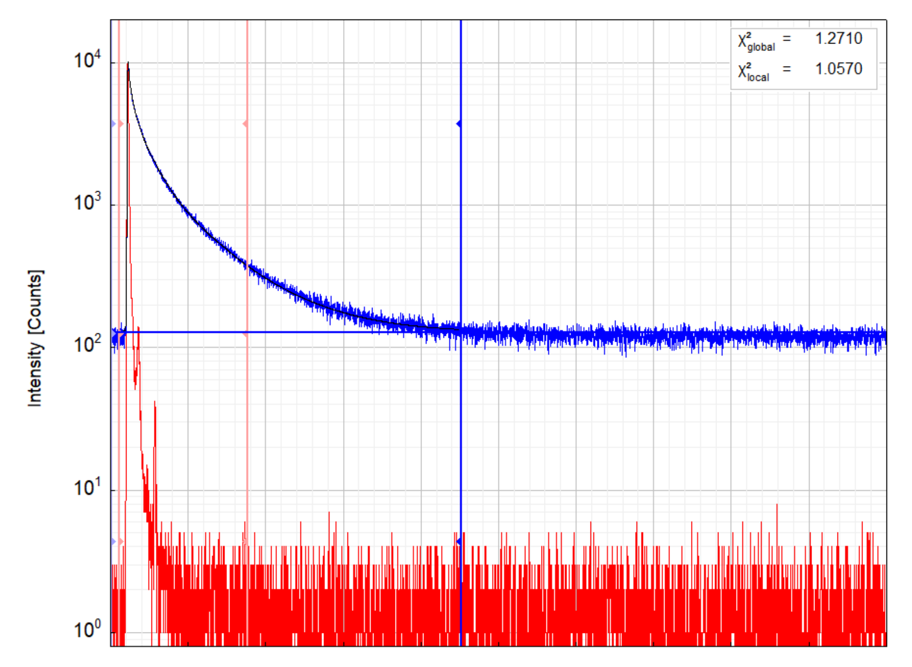

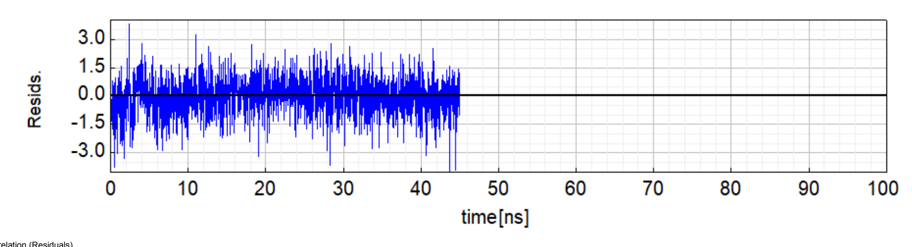

Autocorrelation (Residuals)

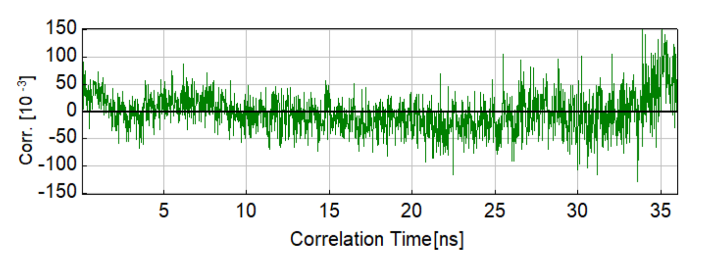

Fitted Parameters

Parameter	Value	Δ	δ
A_1[kCnts/Chnl]	4.870	±0.080	1.6%
τ_1[ns]	0.460	±0.019	4.0%
I_1[kCnts]	89.6	±4.6	5.1%
A_2[kCnts/Chnl]	3.476	±0.096	2.8%
τ_2[ns]	2.220	±0.021	0.9%
I_2[kCnts]	308.7	±6.7	2.2%
A_3[kCnts/Chnl]	1.688	±0.016	0.9%
τ_3[ns]	7.772	±0.017	0.2%
I_3[kCnts]	524.5	±5.8	1.1%
$Bkgr_{Dec}$[kCnts]	0.1270	±0.0005	0.4%
$Bkgr_{IRF}$[Cnts/Chnl]	-0.60	±0.51	85%
$Shift_{IRF}$[ps]	7.91	±0.56	7.1%
A_{Scatt}[kCnts]	47.9	±1.1	2.1%
τ_{AvInt}[ns]	5.205	±0.028	0.5%
τ_{AvAmp}[ns]	2.300	±0.033	1.4%
λ_{Det}[nm]	600.0000	---	---

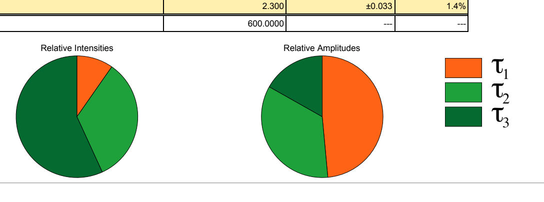

Data Set: 22 / 33

Decay: crv[22]; IRF: crv[0]

Fit

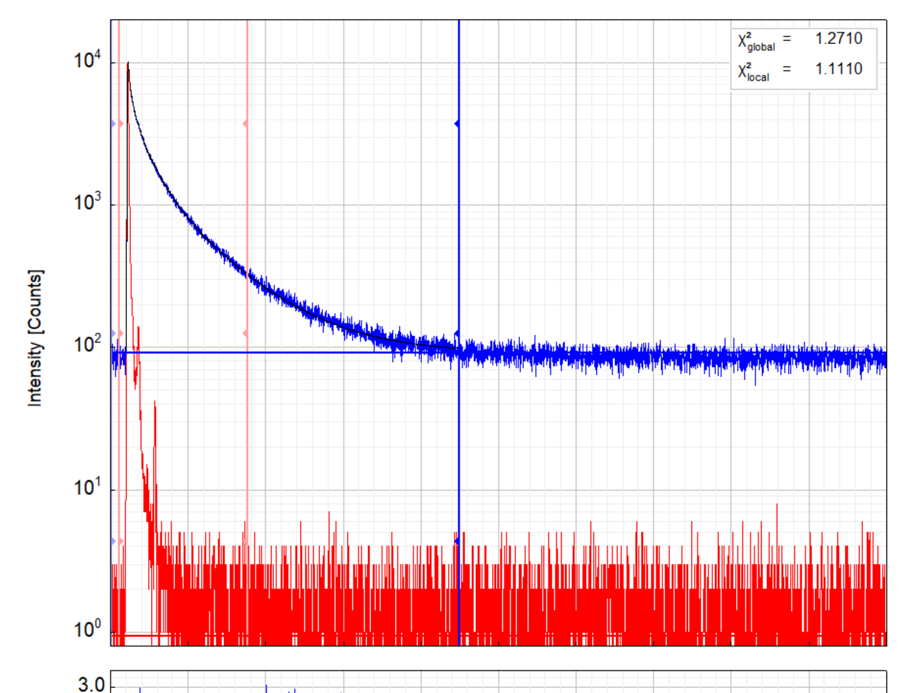

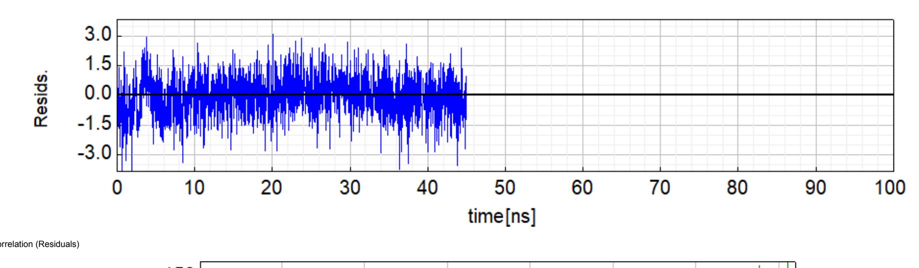

Autocorrelation (Residuals)

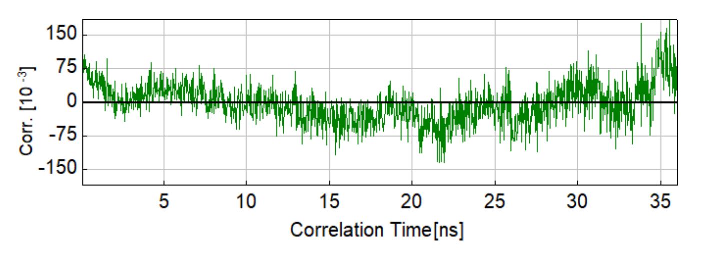

Fitted Parameters

Parameter	Value	Δ	δ
A_1[kCnts/Chnl]	4.95	±0.11	2.1%
τ_1[ns]	0.460	±0.019	4.0%
I_1[kCnts]	90.9	±3.1	3.4%
A_2[kCnts/Chnl]	3.296	±0.076	2.3%
τ_2[ns]	2.220	±0.021	0.9%
I_2[kCnts]	292.7	±4.3	1.4%
A_3[kCnts/Chnl]	1.643	±0.022	1.3%
τ_3[ns]	7.772	±0.017	0.2%
I_3[kCnts]	510.6	±6.6	1.3%
$Bkgr_{Dec}$[kCnts]	0.0916	±0.0004	0.4%
$Bkgr_{IRF}$[Cnts/Chnl]	0.94	±0.35	37%
$Shift_{IRF}$[ps]	7.99	±0.62	7.7%
A_{Scatt}[kCnts]	48.26	±0.97	2.0%
τ_{AvInt}[ns]	5.212	±0.023	0.4%
τ_{AvAmp}[ns]	2.263	±0.033	1.4%
λ_{Det}[nm]	610.0000	---	---

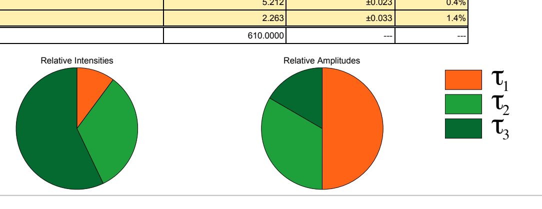

Data Set: 23 / 33

Decay: crv[23]; IRF: crv[0]

Fit

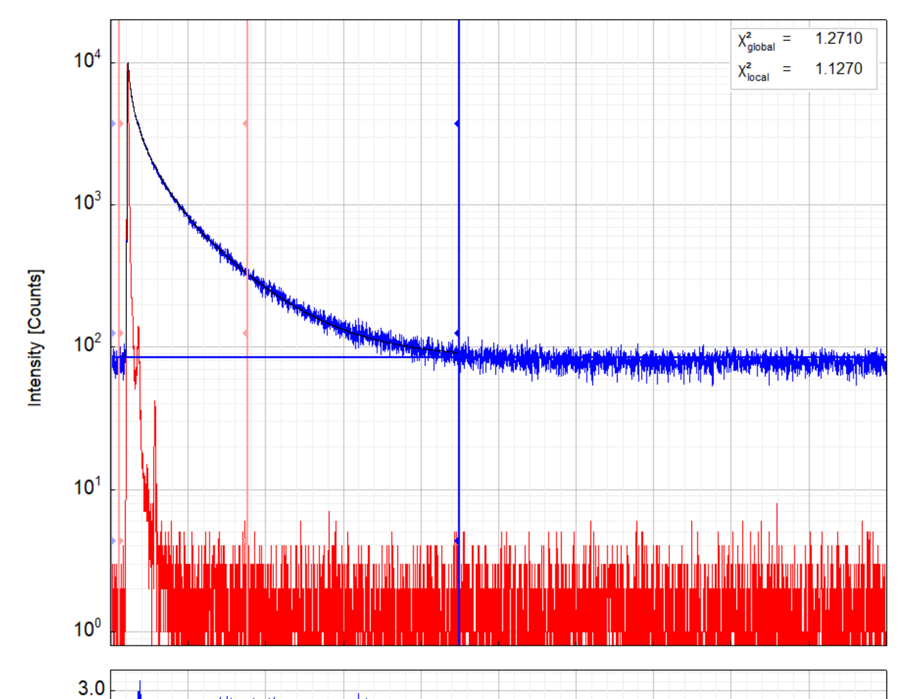

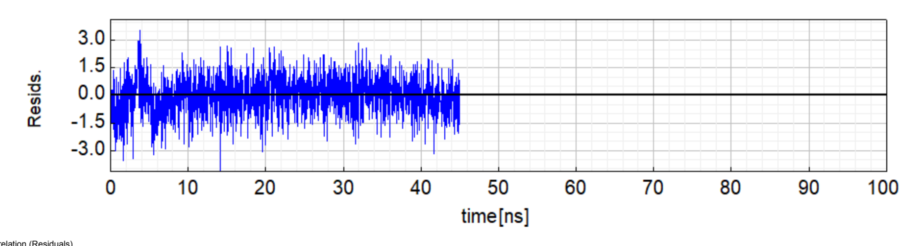

Autocorrelation (Residuals)

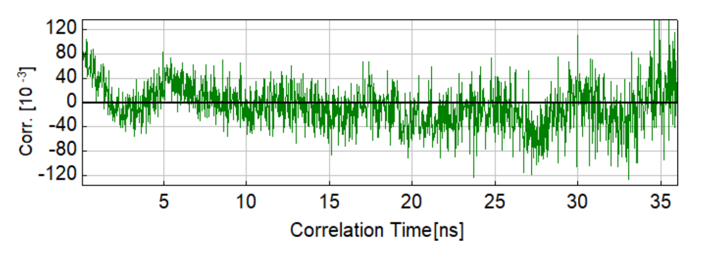

Fitted Parameters

Parameter	Value	Δ	δ
A_1[kCnts/Chnl]	5.12	±0.12	2.2%
τ_1[ns]	0.460	±0.019	4.0%
I_1[kCnts]	94.1	±1.9	2.0%
A_2[kCnts/Chnl]	3.163	±0.059	1.9%
τ_2[ns]	2.220	±0.021	0.9%
I_2[kCnts]	280.9	±3.6	1.3%
A_3[kCnts/Chnl]	1.689	±0.013	0.7%
τ_3[ns]	7.772	±0.017	0.2%
I_3[kCnts]	525.0	±4.0	0.8%
$Bkgr_{Dec}$[kCnts]	0.0844	±0.0003	0.3%
$Bkgr_{IRF}$[Cnts/Chnl]	0.22	±0.37	171%
$Shift_{IRF}$[ps]	8.3	±1.2	14%

A_{Scatt}[kCnts]	47.80	±0.95	2.0%
τ_{AvInt}[ns]	5.275	±0.021	0.4%
τ_{AvAmp}[ns]	2.258	±0.030	1.3%
λ_{Det}[nm]	620.0000	---	---

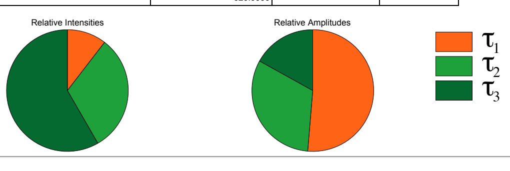

Data Set: 24 / 33

Decay: crv[24]; IRF: crv[0]

Fit

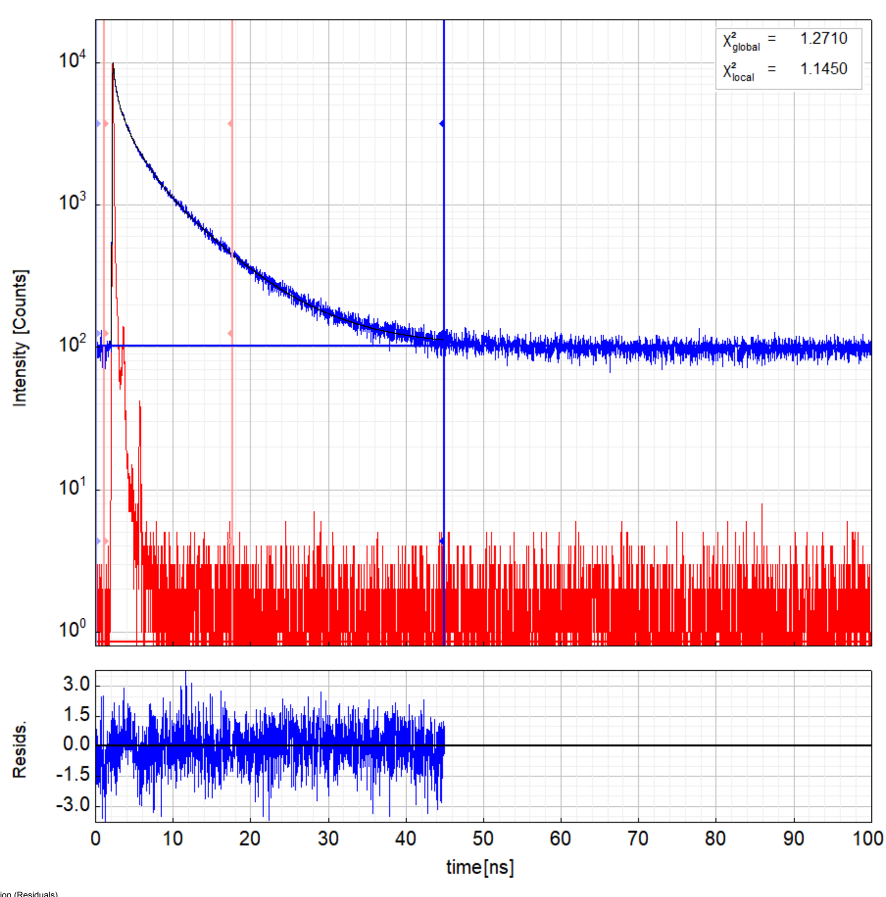

Autocorrelation (Residuals)

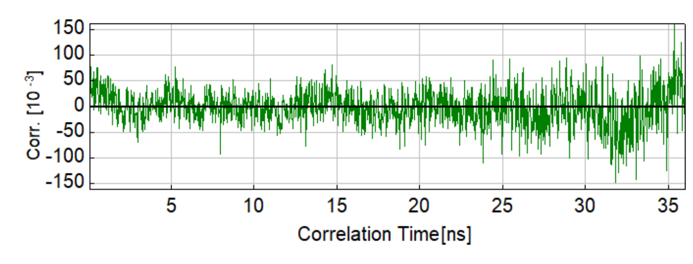

Fitted Parameters

Parameter	Value	Δ	δ
A_1[kCnts/Chnl]	4.54	±0.12	2.6%
τ_1[ns]	0.460	±0.019	4.0%
I_1[kCnts]	83.5	±3.3	3.9%
A_2[kCnts/Chnl]	3.120	±0.038	1.2%
τ_2[ns]	2.220	±0.021	0.9%
I_2[kCnts]	277.1	±2.5	0.9%
A_3[kCnts/Chnl]	2.453	±0.027	1.1%
τ_3[ns]	7.772	±0.017	0.2%
I_3[kCnts]	762.3	±6.7	0.9%
$Bkgr_{Dec}$[kCnts]	0.1027	±0.0004	0.3%
$Bkgr_{IRF}$[Cnts/Chnl]	0.86	±0.47	55%
$Shift_{IRF}$[ps]	9.88	±0.58	5.8%
A_{Scatt}[kCnts]	45.62	±0.59	1.3%
τ_{AvInt}[ns]	5.858	±0.013	0.2%
τ_{AvAmp}[ns]	2.777	±0.035	1.2%
λ_{Det}[nm]	630.0000	---	---

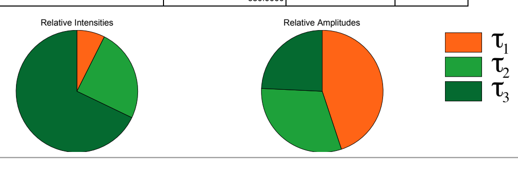

Data Set: 25 / 33

Decay: crv[25]; IRF: crv[0]

Fit

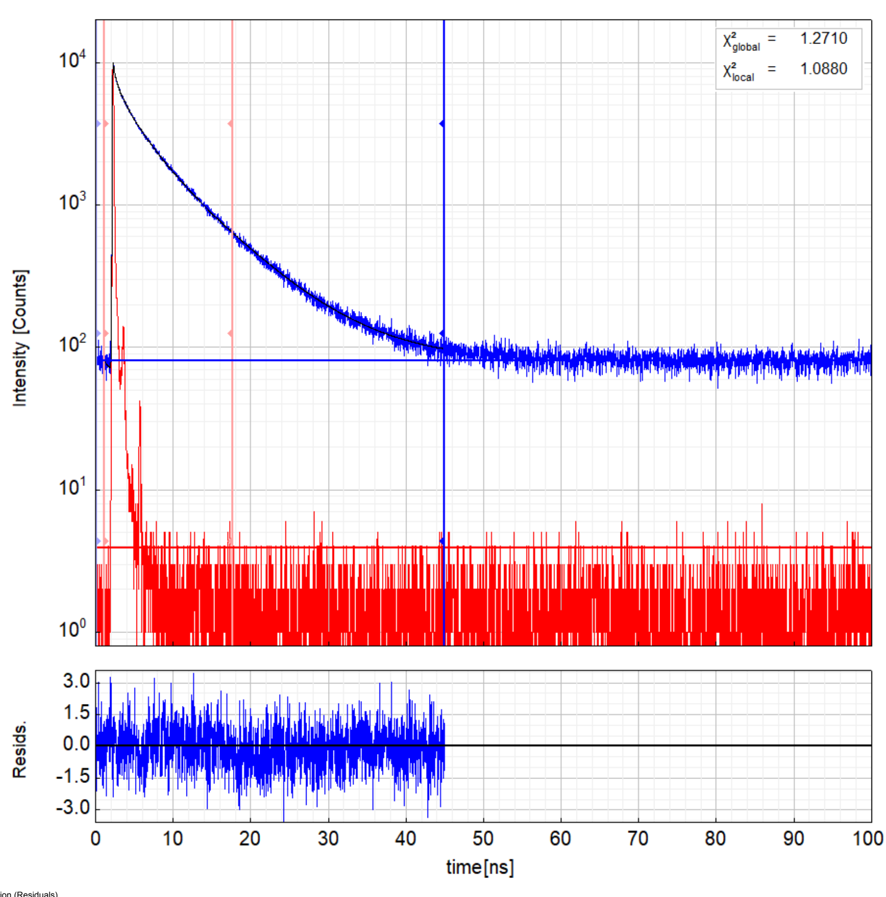

Autocorrelation (Residuals)

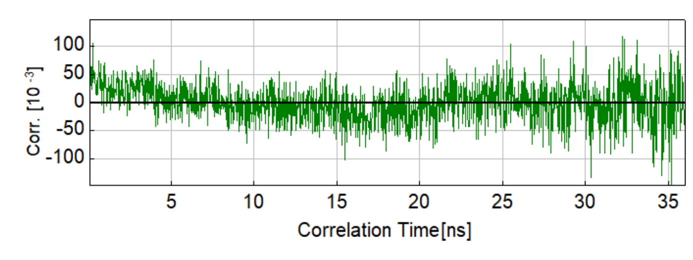

Fitted Parameters

Parameter	Value	Δ	δ
A_1[kCnts/Chnl]	3.083	±0.077	2.5%
τ_1[ns]	0.460	±0.019	4.0%
I_1[kCnts]	56.70	±0.98	1.7%
A_2[kCnts/Chnl]	3.302	±0.067	2.0%
τ_2[ns]	2.220	±0.021	0.9%
I_2[kCnts]	293.2	±3.4	1.2%
A_3[kCnts/Chnl]	4.202	±0.015	0.4%
τ_3[ns]	7.772	±0.017	0.2%
I_3[kCnts]	1 306.0	±4.8	0.4%
$Bkgr_{Dec}$[kCnts]	0.0810	±0.0005	0.5%
$Bkgr_{IRF}$[Cnts/Chnl]	3.93	±0.33	8.3%
$Shift_{IRF}$[ps]	12.28	±0.49	4.0%
A_{Scatt}[kCnts]	42.10	±0.90	2.1%
τ_{AvInt}[ns]	6.5379	±0.0087	0.1%
τ_{AvAmp}[ns]	3.911	±0.030	0.7%
λ_{Det}[nm]	640.0000	---	---

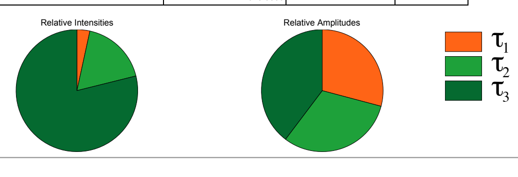

Data Set: 26 / 33

Decay: crv[26]; IRF: crv[0]

Fit

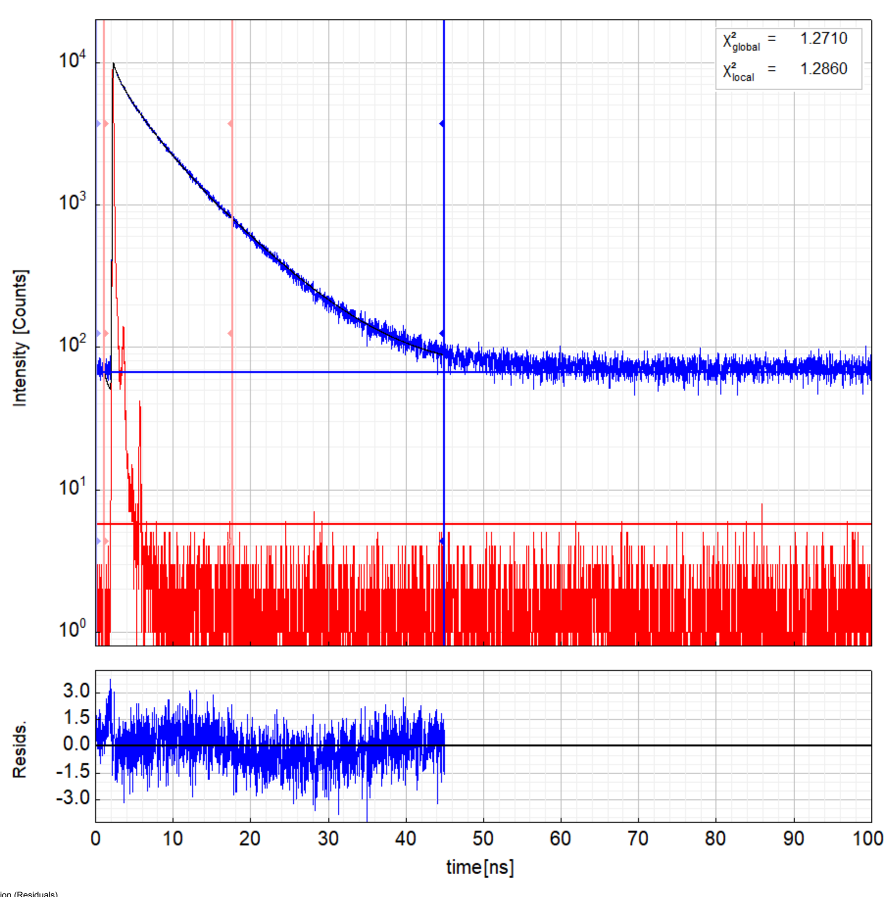

Autocorrelation (Residuals)

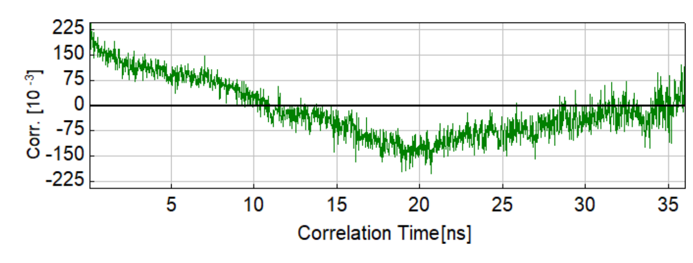

Fitted Parameters

Parameter	Value	Δ	δ
A_1[kCnts/Chnl]	2.133	±0.025	1.2%
τ_1[ns]	0.460	±0.019	4.0%
I_1[kCnts]	39.3	±2.1	5.1%
A_2[kCnts/Chnl]	3.466	±0.063	1.8%
τ_2[ns]	2.220	±0.021	0.9%
I_2[kCnts]	307.8	±5.0	1.6%
A_3[kCnts/Chnl]	5.705	±0.023	0.4%
τ_3[ns]	7.772	±0.017	0.2%
I_3[kCnts]	1 773.2	±5.8	0.3%
$Bkgr_{Dec}$[kCnts]	0.0663	±0.0004	0.5%
$Bkgr_{IRF}$[Cnts/Chnl]	5.67	±0.20	3.4%
$Shift_{IRF}$[ps]	11.01	±0.64	5.8%
A_{Scatt}[kCnts]	36.29	±0.60	1.6%
τ_{AvInt}[ns]	6.831	±0.014	0.2%
τ_{AvAmp}[ns]	4.690	±0.017	0.4%
λ_{Det}[nm]	650.0000	---	---

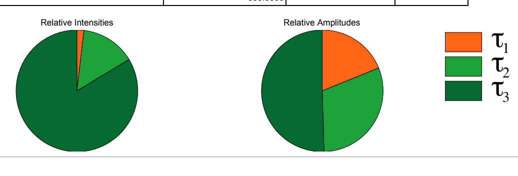

Data Set: 27 / 33

Decay: crv[27]; IRF: crv[0]

Fit

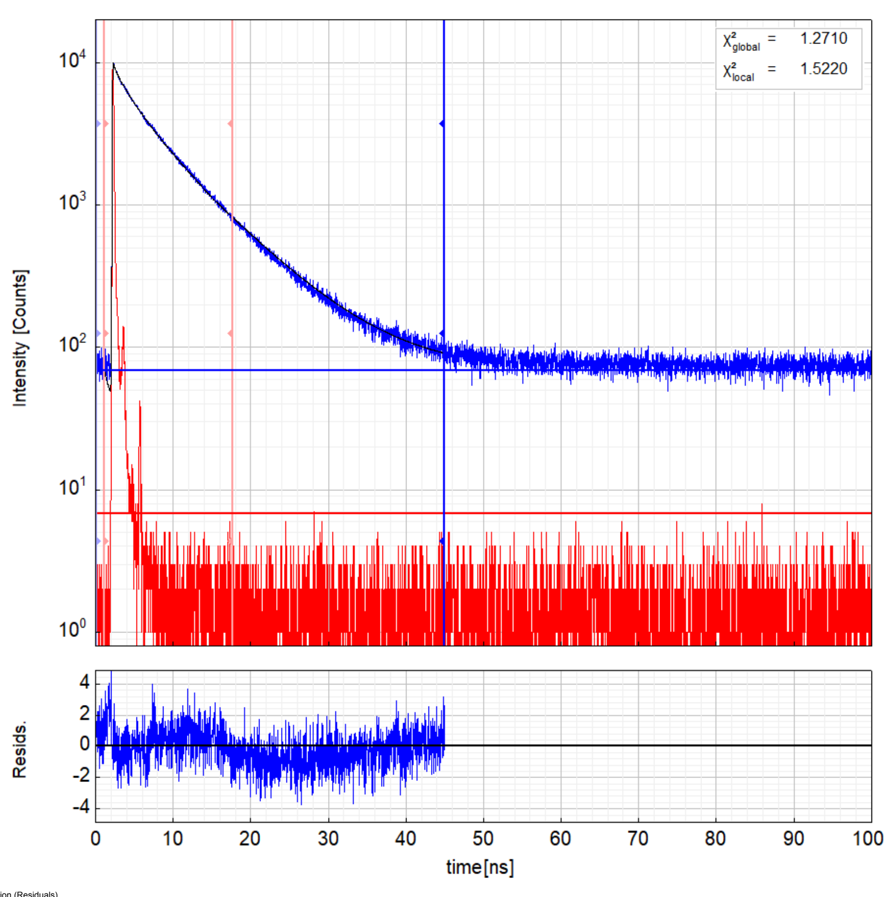

Autocorrelation (Residuals)

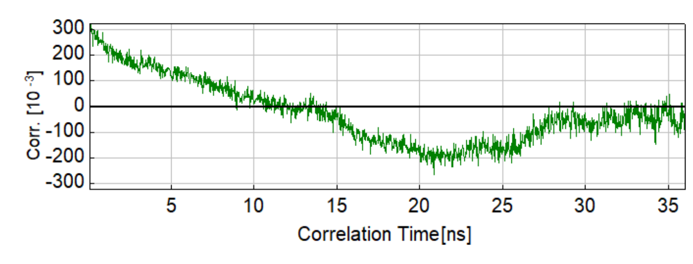

Fitted Parameters

Parameter	Value	Δ	δ
A_1[kCnts/Chnl]	1.74	±0.11	6.3%
τ_1[ns]	0.460	±0.019	4.0%
I_1[kCnts]	31.9	±2.1	6.6%
A_2[kCnts/Chnl]	3.434	±0.082	2.4%
τ_2[ns]	2.220	±0.021	0.9%
I_2[kCnts]	305	±13	4.1%
A_3[kCnts/Chnl]	5.955	±0.073	1.2%
τ_3[ns]	7.772	±0.017	0.2%
I_3[kCnts]	1 851	±21	1.1%
$Bkgr_{Dec}$[kCnts]	0.0687	±0.0008	1.1%
$Bkgr_{IRF}$[Cnts/Chnl]	6.77	±0.50	7.3%
$Shift_{IRF}$[ps]	12.90	±0.69	5.3%
A_{Scatt}[kCnts]	35.81	±0.71	2.0%
τ_{AvInt}[ns]	6.892	±0.018	0.3%
τ_{AvAmp}[ns]	4.919	±0.032	0.6%
λ_{Det}[nm]	660.0000	---	---

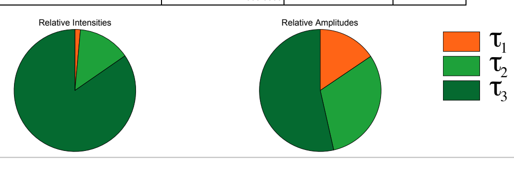

Data Set: 28 / 33

Decay: crv[28]; IRF: crv[0]

Fit

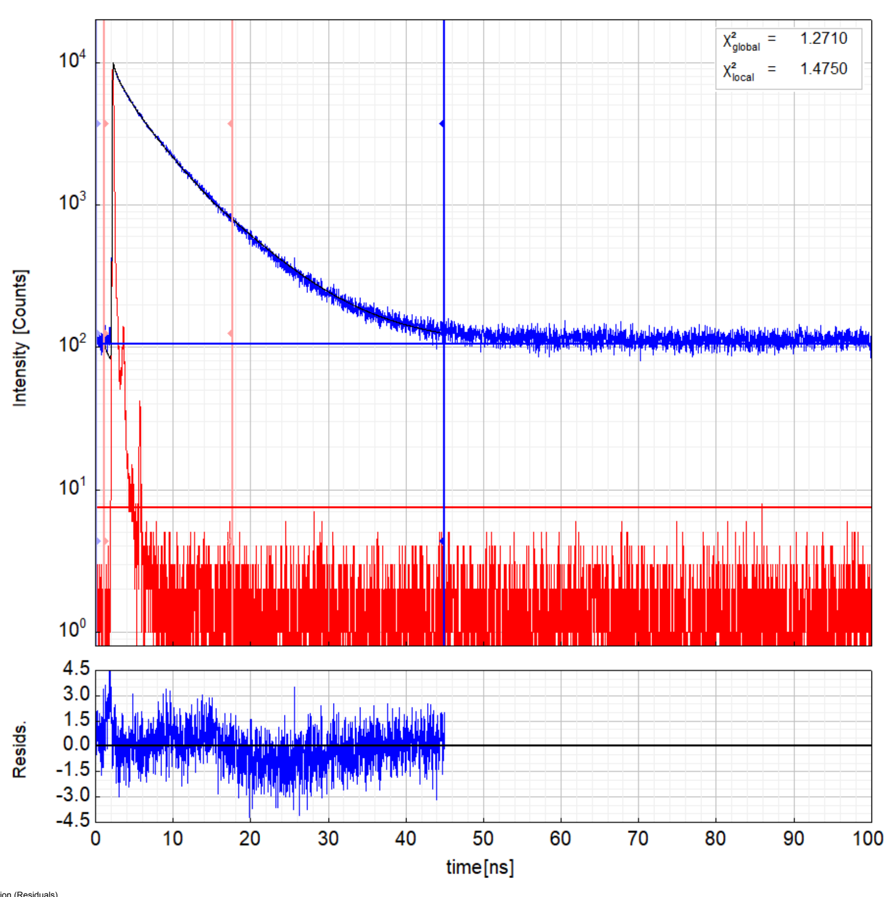

Autocorrelation (Residuals)

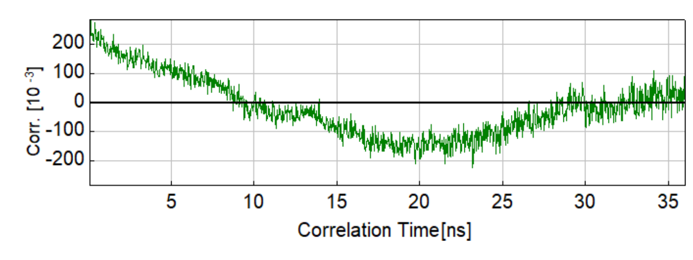

Fitted Parameters

Parameter	Value	Δ	δ
A_1[kCnts/Chnl]	2.14	±0.14	6.4%
τ_1[ns]	0.460	±0.019	4.0%
I_1[kCnts]	39.3	±1.9	4.8%
A_2[kCnts/Chnl]	3.389	±0.058	1.7%
τ_2[ns]	2.220	±0.021	0.9%
I_2[kCnts]	301	±11	3.4%
A_3[kCnts/Chnl]	5.519	±0.039	0.7%
τ_3[ns]	7.772	±0.017	0.2%
I_3[kCnts]	1 716	±13	0.7%
$Bkgr_{Dec}$[kCnts]	0.1049	±0.0006	0.6%
$Bkgr_{IRF}$[Cnts/Chnl]	7.46	±0.36	4.7%
$Shift_{IRF}$[ps]	13.9	±1.2	8.2%
A_{Scatt}[kCnts]	37.03	±0.93	2.5%
τ_{AvInt}[ns]	6.820	±0.018	0.3%
τ_{AvAmp}[ns]	4.656	±0.041	0.9%
λ_{Det}[nm]	670.0000	---	---

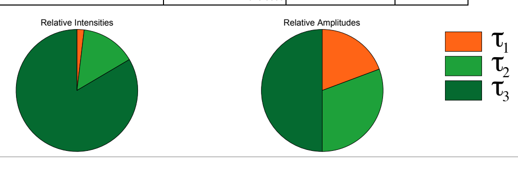

Data Set: 29 / 33

Decay: crv[29]; IRF: crv[0]

Fit

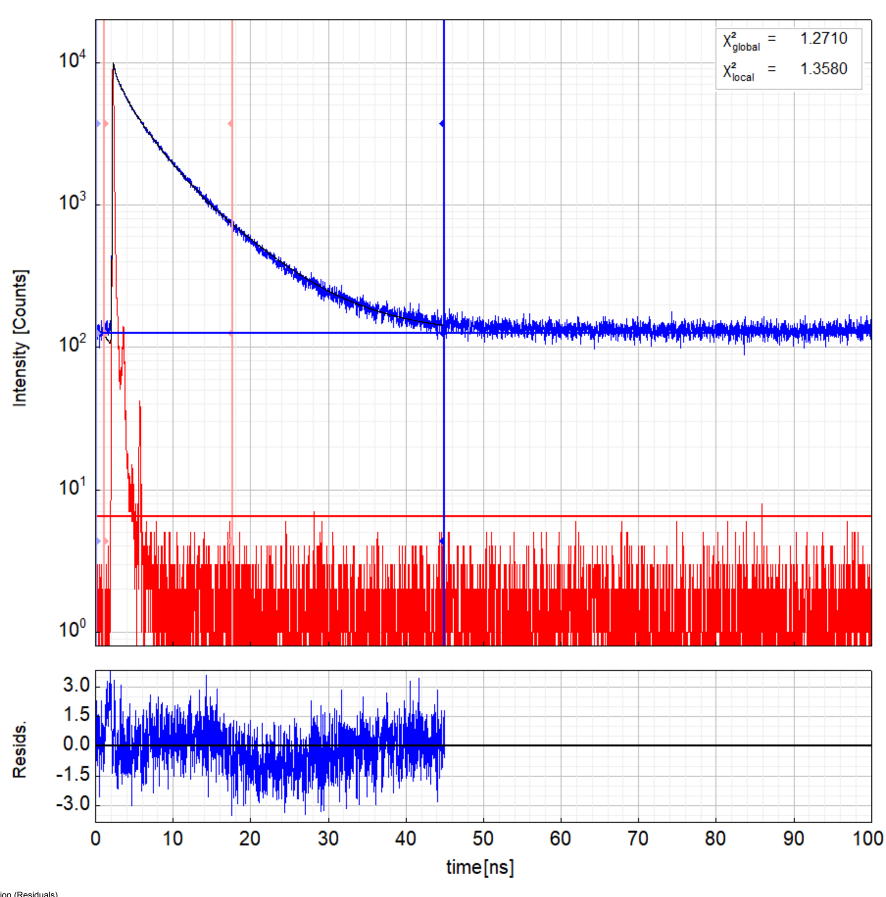

Autocorrelation (Residuals)

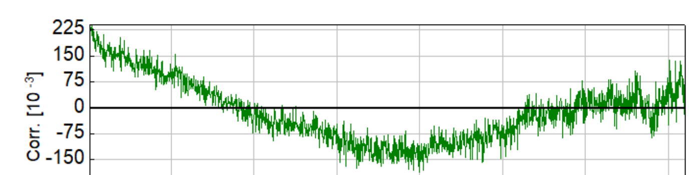

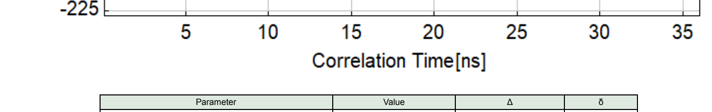

Parameter	Value	Δ	δ
A_1[kCnts/Chnl]	2.33	±0.11	4.4%
τ_1[ns]	0.460	±0.019	4.0%
I_1[kCnts]	42.8	±2.2	5.1%
A_2[kCnts/Chnl]	3.717	±0.070	1.9%
τ_2[ns]	2.220	±0.021	0.9%
I_2[kCnts]	330.1	±7.1	2.1%
A_3[kCnts/Chnl]	4.807	±0.037	0.8%
τ_3[ns]	7.772	±0.017	0.2%
I_3[kCnts]	1 494.0	±8.4	0.6%
$Bkgr_{Dec}$[kCnts]	0.1246	±0.0005	0.4%
$Bkgr_{IRF}$[Cnts/Chnl]	6.43	±0.33	5.1%
$Shift_{IRF}$[ps]	15.26	±0.51	3.3%
A_{Scatt}[kCnts]	39.05	±0.94	2.4%
τ_{AvInt}[ns]	6.6222	±0.0071	0.1%
τ_{AvAmp}[ns]	4.302	±0.022	0.5%
λ_{Det}[nm]	680.0000	---	---

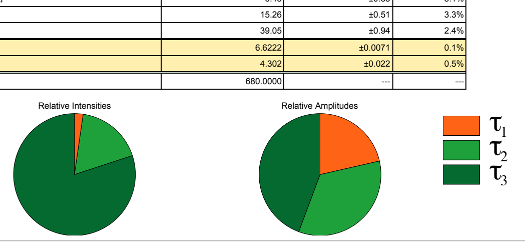

Data Set: 30 / 33

Decay: crv[30]; IRF: crv[0]

Fit

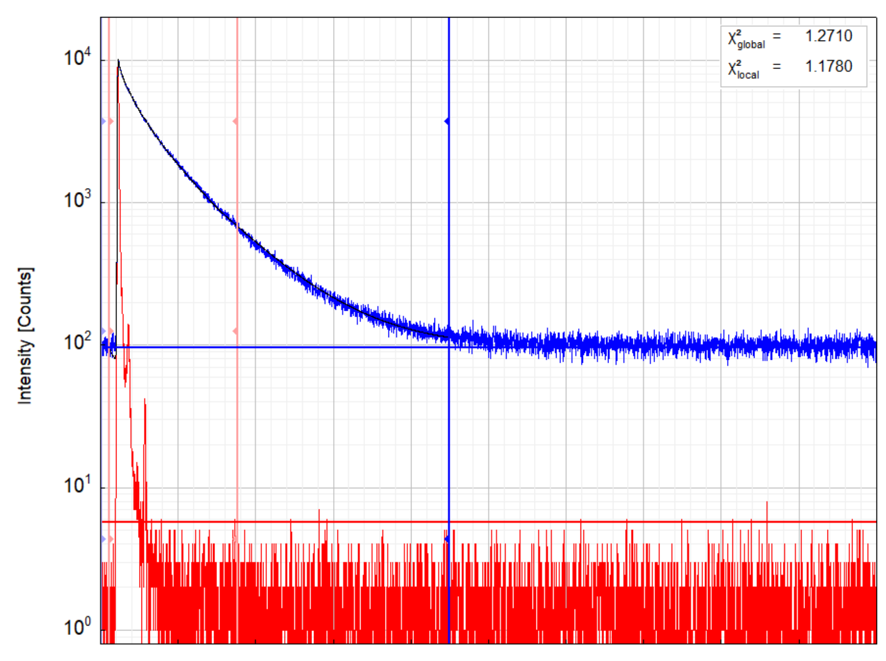

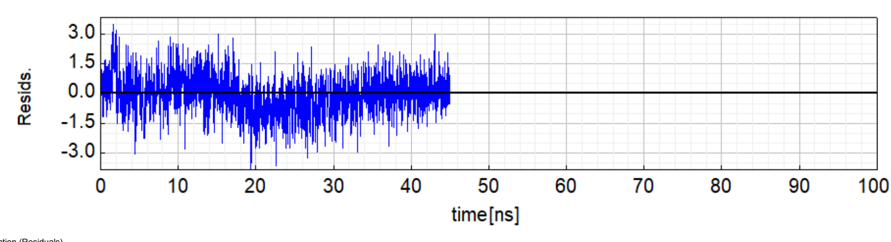

Autocorrelation (Residuals)

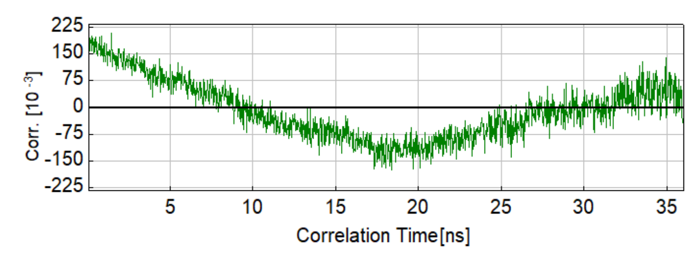

Fitted Parameters

Parameter	Value	Δ	δ
A_1[kCnts/Chnl]	2.528	±0.098	3.9%
τ_1[ns]	0.460	±0.019	4.0%
I_1[kCnts]	46.5	±2.0	4.1%
A_2[kCnts/Chnl]	3.983	±0.063	1.6%
τ_2[ns]	2.220	±0.021	0.9%
I_2[kCnts]	353.7	±8.3	2.3%
A_3[kCnts/Chnl]	4.565	±0.038	0.8%
τ_3[ns]	7.772	±0.017	0.2%
I_3[kCnts]	1 420	±15	1.0%
$Bkgr_{Dec}$[kCnts]	0.0956	±0.0007	0.7%
$Bkgr_{IRF}$[Cnts/Chnl]	5.75	±0.63	11%
$Shift_{IRF}$[ps]	15.80	±0.29	1.8%
A_{Scatt}[kCnts]	38.94	±0.27	0.7%
τ_{AvInt}[ns]	6.506	±0.027	0.4%
τ_{AvAmp}[ns]	4.107	±0.038	0.9%
λ_{Det}[nm]	690.0000	---	---

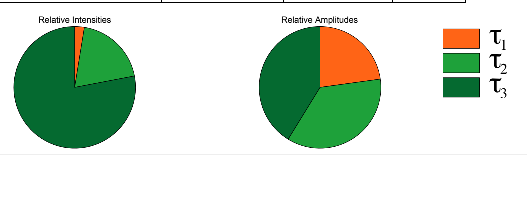

Data Set: 31 / 33

Decay: crv[31]; IRF: crv[0]

Fit

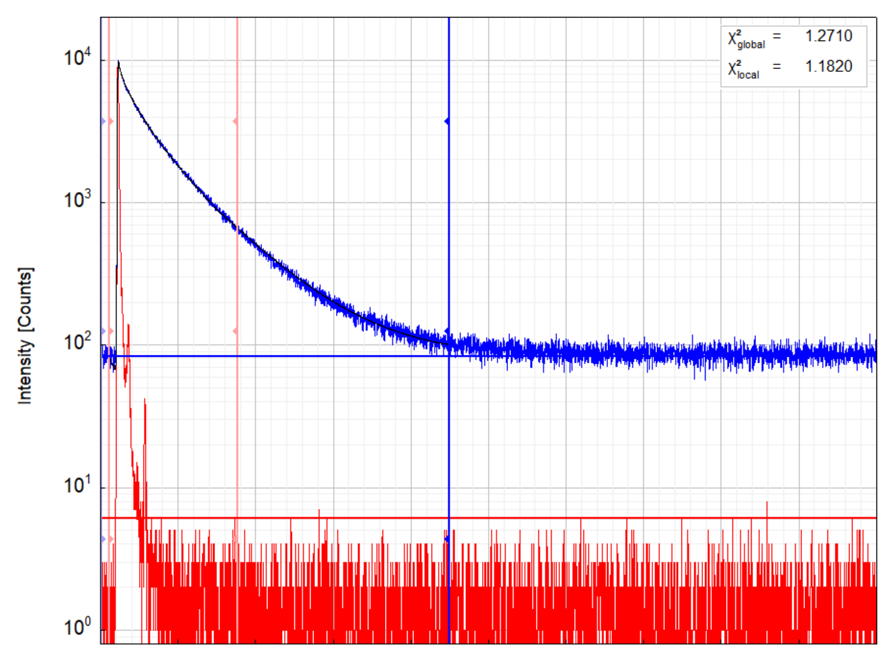

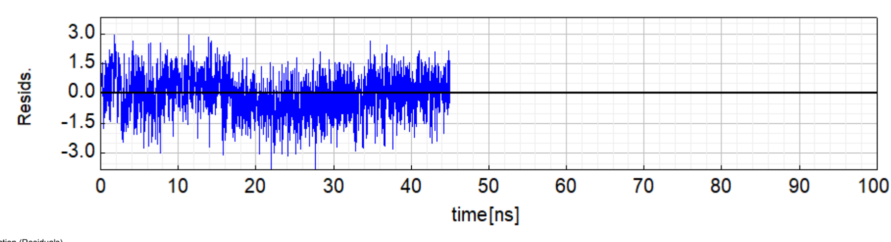

Autocorrelation (Residuals)

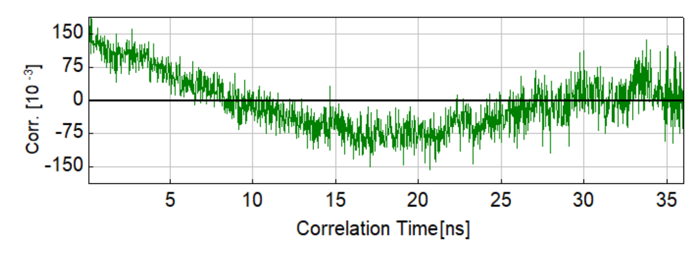

Fitted Parameters

Parameter	Value	Δ	δ
A_1[kCnts/Chnl]	2.61	±0.11	4.1%
τ_1[ns]	0.460	±0.019	4.0%
I_1[kCnts]	48.0	±2.2	4.4%
A_2[kCnts/Chnl]	3.743	±0.077	2.0%
τ_2[ns]	2.220	±0.021	0.9%
I_2[kCnts]	332.4	±6.4	1.9%
A_3[kCnts/Chnl]	4.533	±0.038	0.8%
τ_3[ns]	7.772	±0.017	0.2%
I_3[kCnts]	1 409	±12	0.8%
$Bkgr_{Dec}$[kCnts]	0.0834	±0.0004	0.4%
$Bkgr_{IRF}$[Cnts/Chnl]	6.09	±0.46	7.4%
$Shift_{IRF}$[ps]	16.6	±1.1	6.1%
A_{Scatt}[kCnts]	37.47	±0.90	2.4%
τ_{AvInt}[ns]	6.544	±0.023	0.3%
τ_{AvAmp}[ns]	4.110	±0.036	0.9%
λ_{Det}[nm]	700.0000	---	---

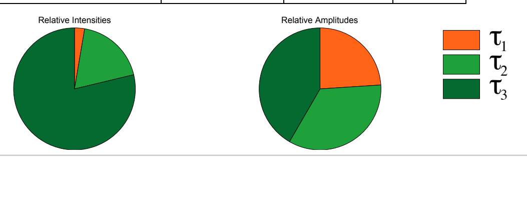

Data Set: 32 / 33

Decay: crv[32]; IRF: crv[0]

Fit

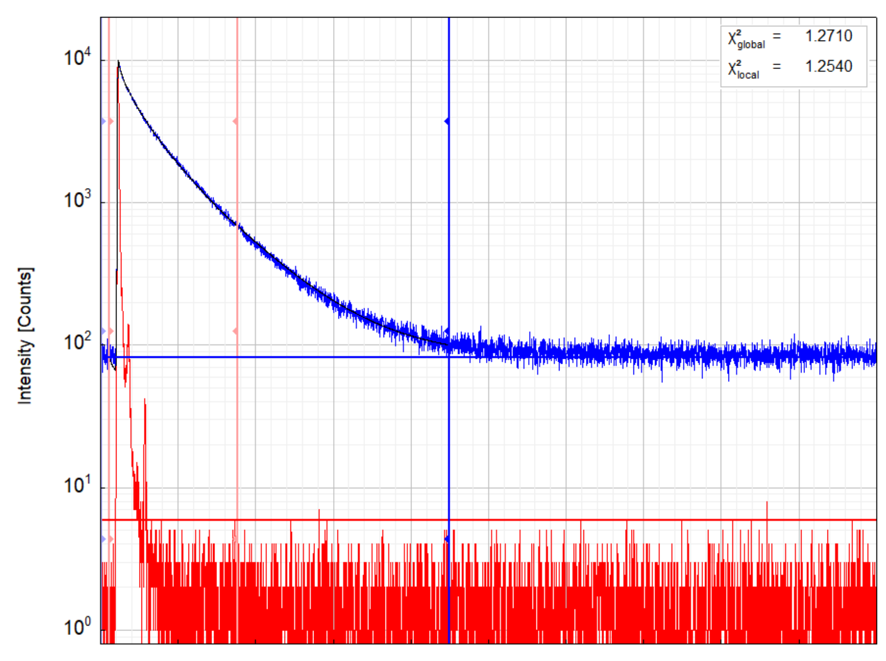

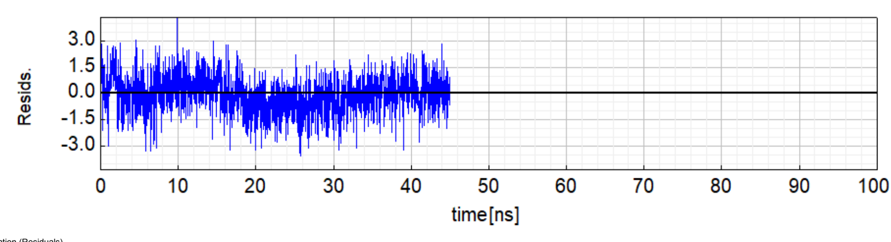

Autocorrelation (Residuals)

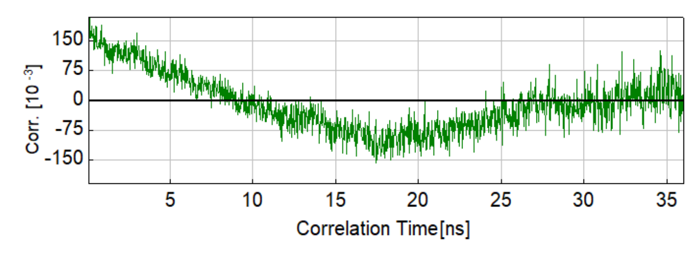

Fitted Parameters

Parameter	Value	Δ	δ
A_1[kCnts/Chnl]	2.623	±0.081	3.1%
τ_1[ns]	0.460	±0.019	4.0%
I_1[kCnts]	48.3	±2.5	5.1%
A_2[kCnts/Chnl]	3.728	±0.080	2.1%
τ_2[ns]	2.220	±0.021	0.9%
I_2[kCnts]	331.1	±7.1	2.1%
A_3[kCnts/Chnl]	4.693	±0.043	0.9%
τ_3[ns]	7.772	±0.017	0.2%
I_3[kCnts]	1 459	±11	0.7%
$Bkgr_{Dec}$[kCnts]	0.0823	±0.0004	0.5%
$Bkgr_{IRF}$[Cnts/Chnl]	5.86	±0.40	6.7%
$Shift_{IRF}$[ps]	17.60	±0.45	2.5%
A_{Scatt}[kCnts]	37.53	±0.80	2.1%
τ_{AvInt}[ns]	6.580	±0.014	0.2%
τ_{AvAmp}[ns]	4.162	±0.032	0.8%
λ_{Det}[nm]	710.0000	---	---

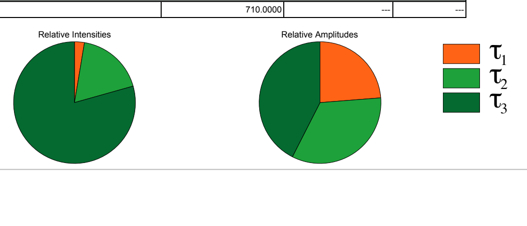

Data Set: 33 / 33

Decay: crv[33]; IRF: crv[0]

Fit

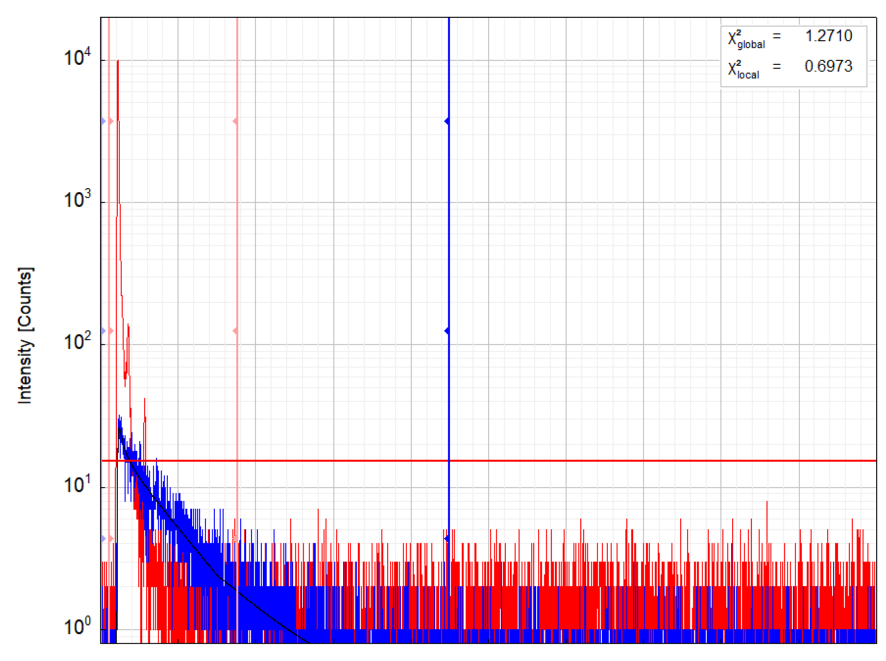

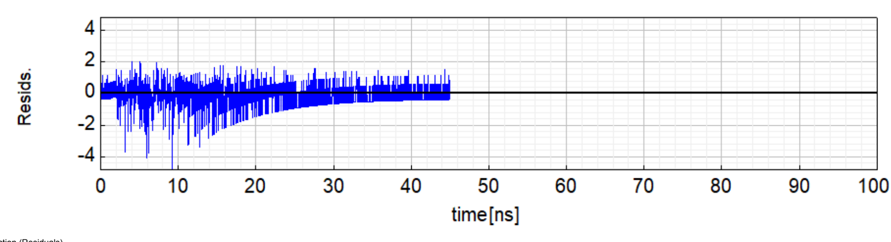

Autocorrelation (Residuals)

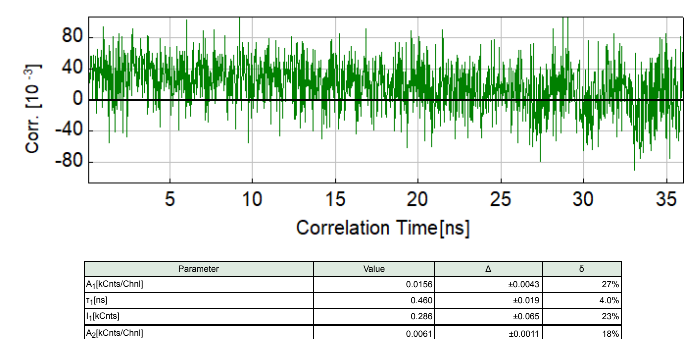

Fitted Parameters

Parameter	Value	Δ	δ
A_1[kCnts/Chnl]	0.0156	±0.0043	27%
τ_1[ns]	0.460	±0.019	4.0%
I_1[kCnts]	0.286	±0.065	23%
A_2[kCnts/Chnl]	0.0061	±0.0011	18%
τ_2[ns]	2.220	±0.021	0.9%
I_2[kCnts]	0.54	±0.11	19%
A_3[kCnts/Chnl]	0.0143	±0.0003	1.7%
τ_3[ns]	7.772	±0.017	0.2%
I_3[kCnts]	4.436	±0.073	1.6%
$Bkgr_{Dec}$[kCnts]	0.0004	±0.0001	8.6%
$Bkgr_{IRF}$[Cnts/Chnl]	15.2	±2.1	14%
$Shift_{IRF}$[ps]	-9	±37	390%
A_{Scatt}[kCnts]	0.025	±0.073	298%
τ_{AvInt}[ns]	6.805	±0.081	1.2%
τ_{AvAmp}[ns]	3.67	±0.36	9.8%
λ_{Det}[nm]	720.0000	---	---

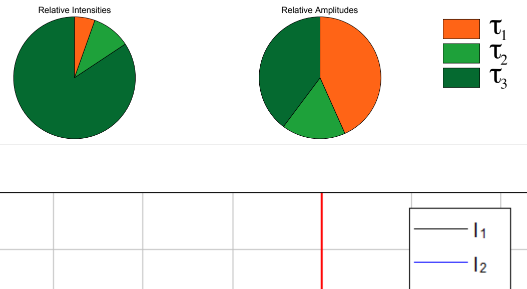

Parameter Plot

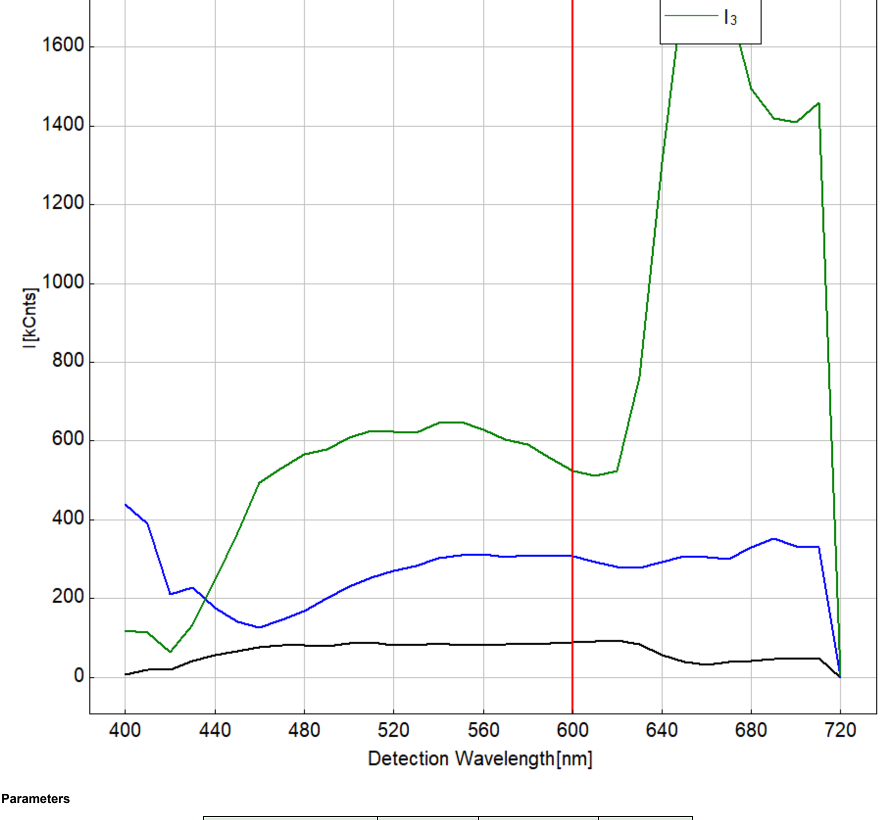

Global Parameters

Parameter	Value	Δ	δ
τ_1[ns]	0.460	±0.019	4.0%
τ_2[ns]	2.220	±0.021	0.9%
τ_3[ns]	7.772	±0.017	0.2%

EasyTau Report

3/11/2022 7:47:16 PM
F:\Paper Xiongfei\CD-1 MeOH tau global.html

Container Curves

TRES+IRF_20201222_1636.etc: crv[0 - 31]

Container Properties

Measurement Context:	Time Resolved Emission Scan
Detection Wavelength:	400.0 - 700.0 (Step: 10.0) nm
Summary:	
Sample:	CD_X_1
Solvent:	Methanol
Excitation:	V pol 376±1nm with LDH-P-C-375
Detection:	M pol 400...700nm 10nm steps<br>10000 peak counts<br>grating 1200/500-<br>detector UV-red [PMT]

Comment

Model: Exponential [Reconv.]

$$\mathrm{Dec}(t)=\left[\int_{-\infty}^{t} dt'\,[\mathrm{IRF}(t-Shift_{IRF})-Bkgr_{IRF}]\left[\sum_{i=1}^{n_{Exp}} A_i\, e^{-\frac{t-t'}{\tau_i}}+A_{Scatt}\,\delta(t-t')\right]\right]+Bkgr_{Dec}$$

$$I_m = A_m \tau_m$$

$$A_{Rel\,m}=A_m \Big/ \sum_{\substack{i=1\\A_i>0}}^{n_{Exp}} A_i \qquad I_{Rel\,m}=I_m \Big/ \sum_{\substack{i=1\\I_i>0}}^{n_{Exp}} I_i$$

$$\tau_{Av\,Amp}=\sum_{\substack{i=1\\A_i>0}}^{n_{Exp}} A_i\tau_i \Big/ \sum_{\substack{i=1\\A_i>0}}^{n_{Exp}} A_i \qquad \tau_{Av\,Int}=\sum_{\substack{i=1\\I_i>0}}^{n_{Exp}} I_i\tau_i \Big/ \sum_{\substack{i=1\\I_i>0}}^{n_{Exp}} I_i$$

Data Set: 1 / 31

Decay: crv[1]; IRF: crv[0]

Fit

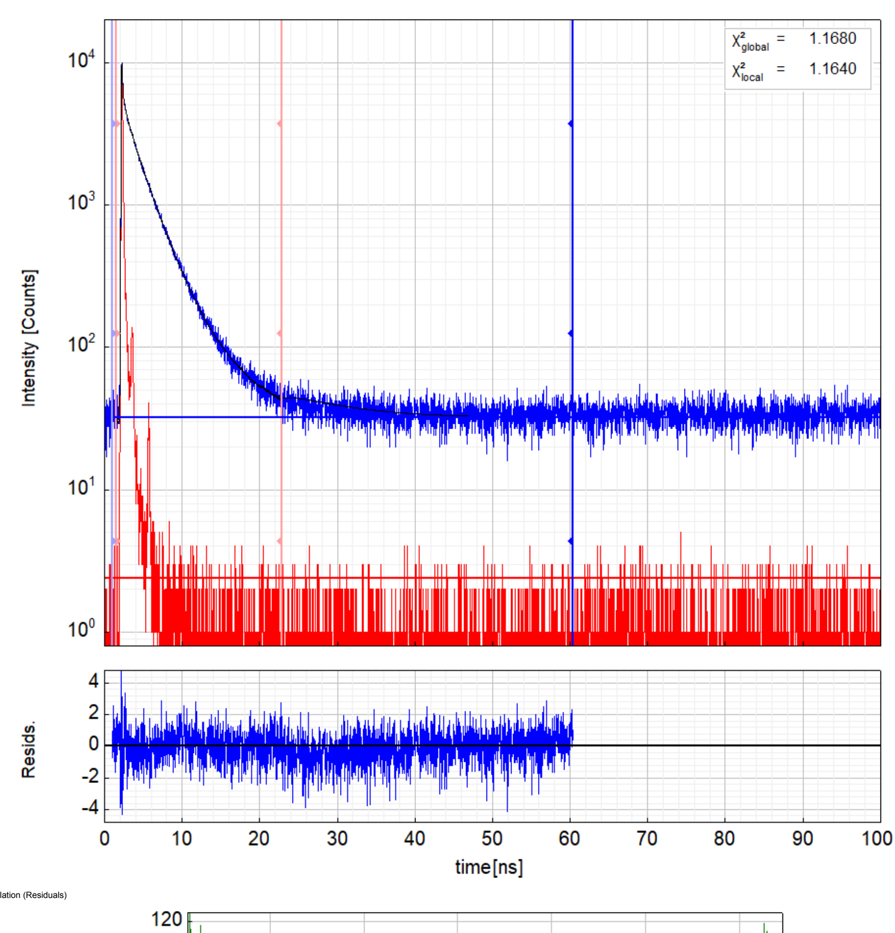

Autocorrelation Residuals

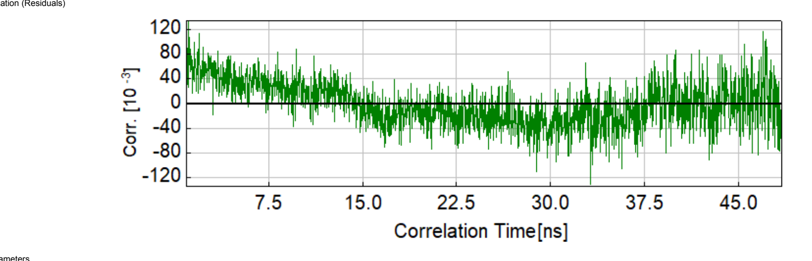

Fitted Parameters

Parameter	Value	Δ	δ
A_1[kCnts/Chnl]	4.557	±0.038	0.8%
τ_1[ns]	2.505	±0.013	0.5%
I_1[kCnts]	456.6	±1.6	0.3%
A_2[kCnts/Chnl]	0.2643	±0.0062	2.3%
τ_2[ns]	8.0397	±0.0067	0.1%
I_2[kCnts]	85.0	±2.3	2.6%
A_3[kCnts/Chnl]	1.77	±0.12	6.7%
τ_3[ns]	0.5435	±0.0070	1.3%
I_3[kCnts]	38.5	±3.2	8.2%
$Bkgr_{Dec}$[kCnts]	0.0320	±0.0002	0.6%
$Bkgr_{IRF}$[Cnts/Chnl]	2.40	±0.13	5.3%
$Shift_{IRF}$[ps]	-9.7	±2.6	26%
A_{Scatt}[kCnts]	59.9	±1.6	2.6%
τ_{AvInt}[ns]	3.186	±0.017	0.5%
τ_{AvAmp}[ns]	2.201	±0.020	0.9%
λ_{Det}[nm]	400.0000	---	---

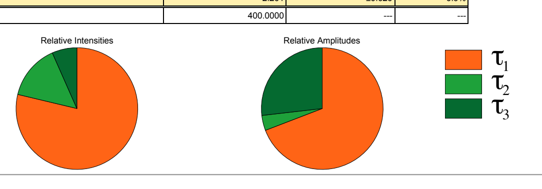

Data Set: 2 / 31

Decay: crv[2]; IRF: crv[0]

Fit

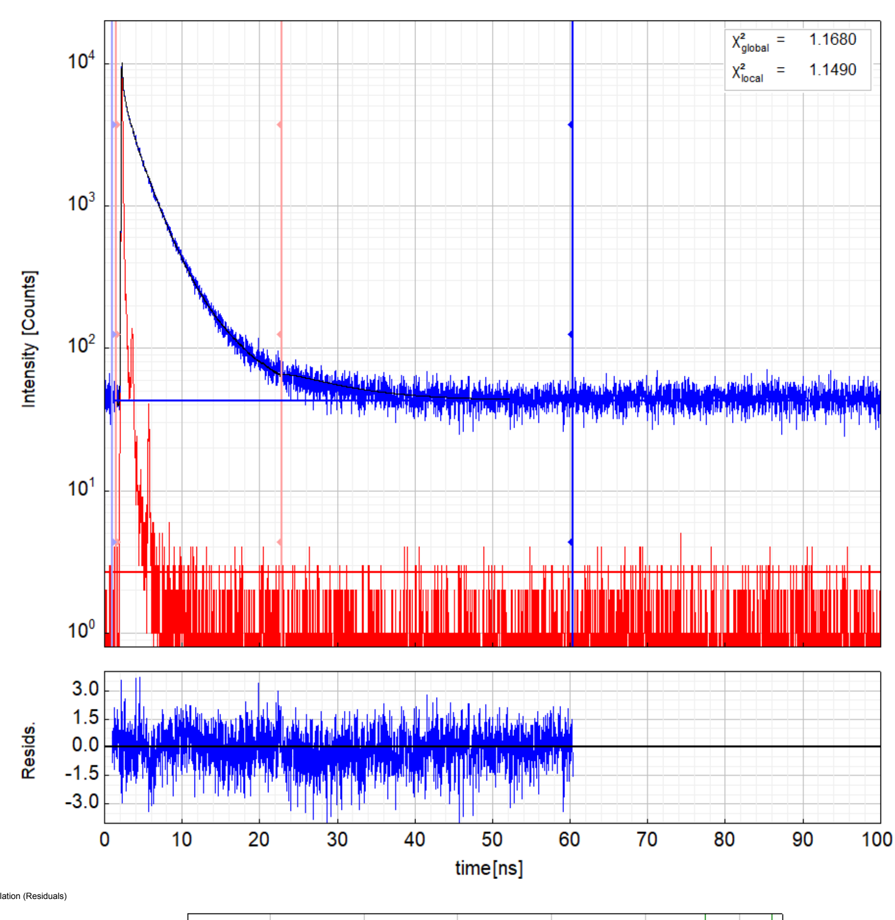

Autocorrelation Residuals

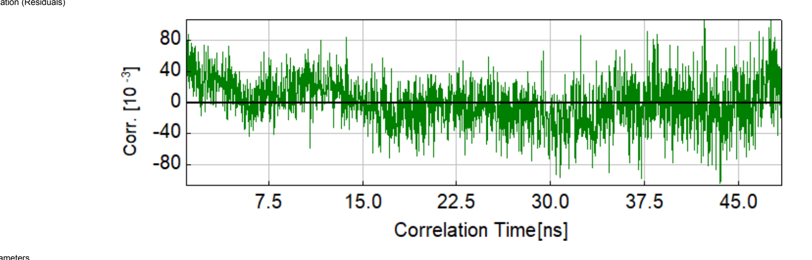

Fitted Parameters

Parameter	Value	Δ	δ
A_1[kCnts/Chnl]	4.764	±0.038	0.8%
τ_1[ns]	2.505	±0.013	0.5%
I_1[kCnts]	477.3	±4.1	0.9%
A_2[kCnts/Chnl]	0.446	±0.017	3.7%
τ_2[ns]	8.0397	±0.0067	0.1%
I_2[kCnts]	143.2	±5.2	3.6%
A_3[kCnts/Chnl]	2.854	±0.082	2.8%
τ_3[ns]	0.5435	±0.0070	1.3%
I_3[kCnts]	62.1	±2.1	3.3%
$Bkgr_{Dec}$[kCnts]	0.0430	±0.0003	0.5%
$Bkgr_{IRF}$[Cnts/Chnl]	2.68	±0.22	7.9%
$Shift_{IRF}$[ps]	-5.76	±0.80	14%
A_{Scatt}[kCnts]	55.43	±0.76	1.4%
τ_{AvInt}[ns]	3.488	±0.031	0.9%
τ_{AvAmp}[ns]	2.117	±0.015	0.7%
λ_{Det}[nm]	410.0000	---	---

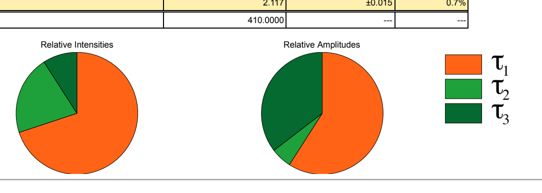

Data Set: 3 / 31

Decay: crv[3]; IRF: crv[0]

Fit

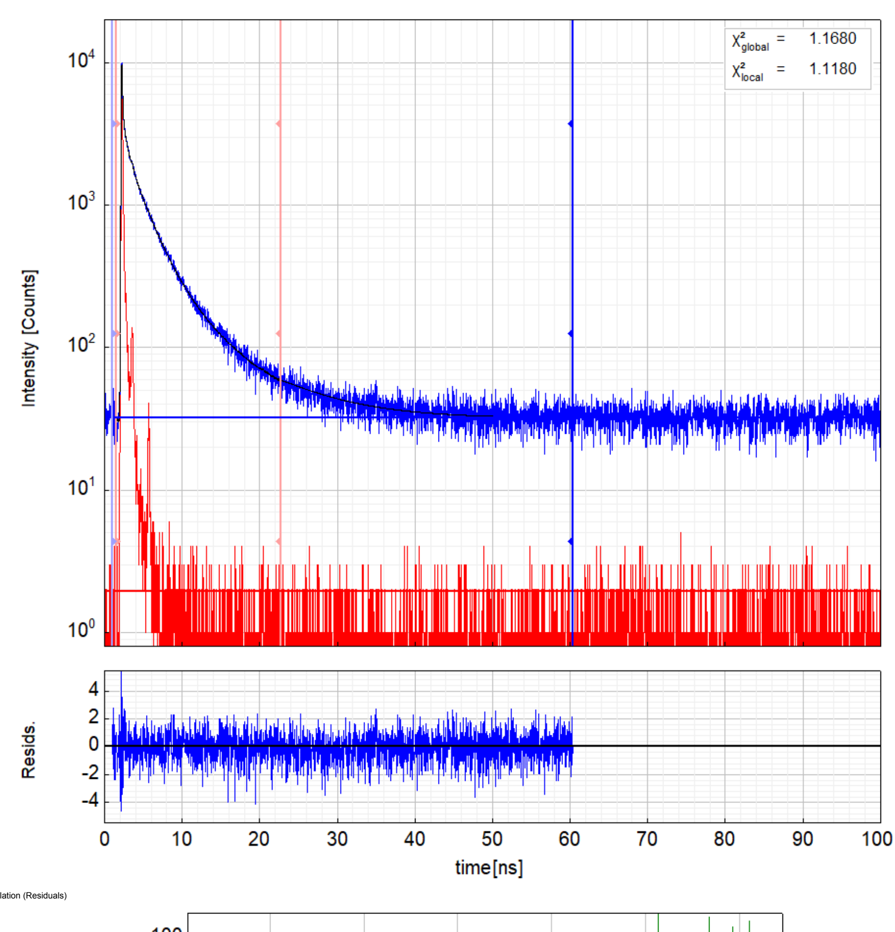

Autocorrelation Residuals

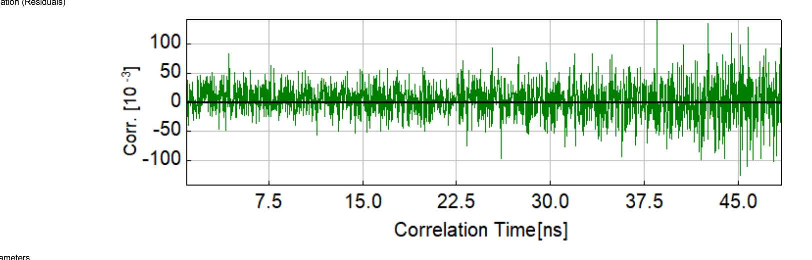

Fitted Parameters

Parameter	Value	Δ	δ
A_1[kCnts/Chnl]	2.290	±0.018	0.8%
τ_1[ns]	2.505	±0.013	0.5%
I_1[kCnts]	229.5	±1.4	0.6%
A_2[kCnts/Chnl]	0.3956	±0.0042	1.1%
τ_2[ns]	8.0397	±0.0067	0.1%
I_2[kCnts]	127.2	±1.3	1.0%
A_3[kCnts/Chnl]	1.856	±0.081	4.3%
τ_3[ns]	0.5435	±0.0070	1.3%
I_3[kCnts]	40.4	±1.9	4.6%
$Bkgr_{Dec}$[kCnts]	0.0320	±0.0003	0.7%
$Bkgr_{IRF}$[Cnts/Chnl]	1.96	±0.11	5.4%
$Shift_{IRF}$[ps]	-13.91	±0.34	2.4%
A_{Scatt}[kCnts]	67.33	±0.87	1.3%
τ_{AvInt}[ns]	4.079	±0.017	0.4%
τ_{AvAmp}[ns]	2.186	±0.026	1.1%
λ_{Det}[nm]	420.0000	---	---

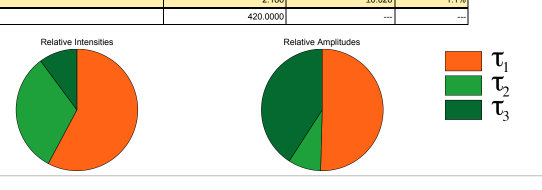

Data Set: 4 / 31

Decay: crv[4]; IRF: crv[0]

Fit

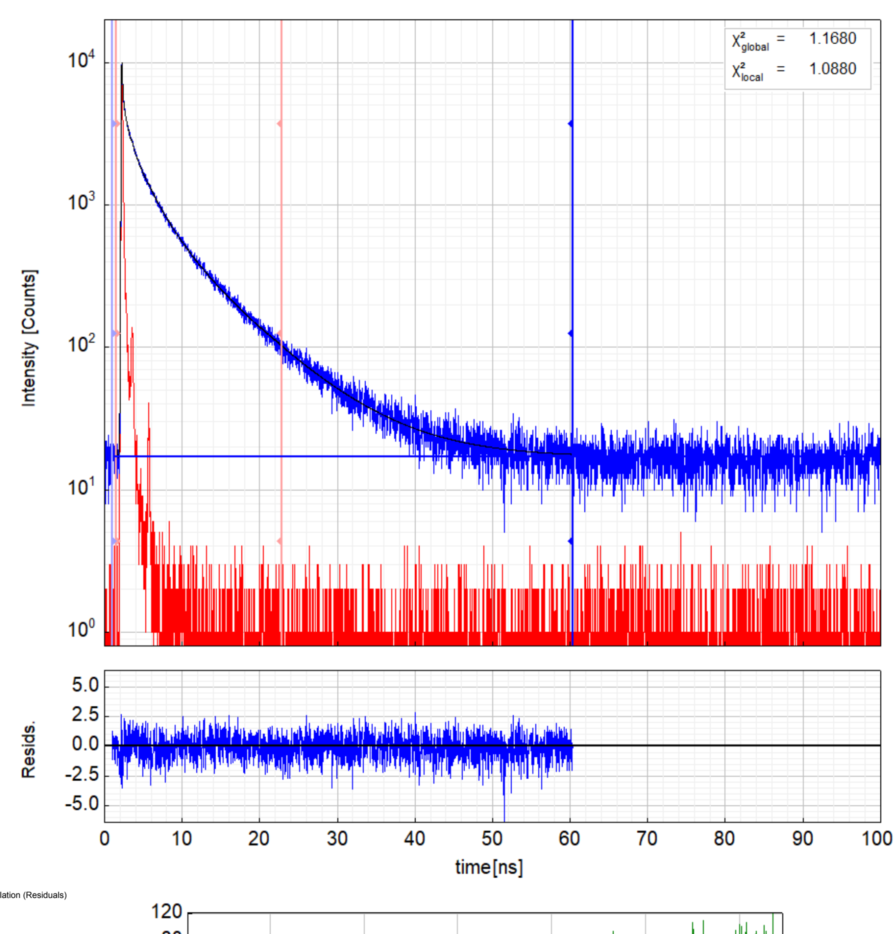

Autocorrelation Residuals

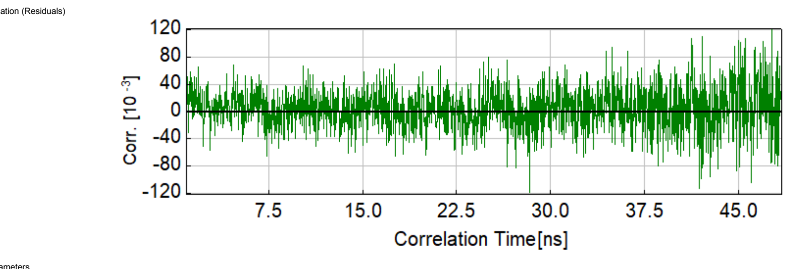

Fitted Parameters

Parameter	Value	Δ	δ
A_1[kCnts/Chnl]	2.525	±0.025	1.0%
τ_1[ns]	2.505	±0.013	0.5%
I_1[kCnts]	253.0	±2.1	0.8%
A_2[kCnts/Chnl]	1.0815	±0.0086	0.8%
τ_2[ns]	8.0397	±0.0067	0.1%
I_2[kCnts]	347.8	±2.8	0.8%
A_3[kCnts/Chnl]	2.872	±0.045	1.5%
τ_3[ns]	0.5435	±0.0070	1.3%
I_3[kCnts]	62.42	±0.91	1.4%
$Bkgr_{Dec}$[kCnts]	0.0170	±0.0002	1.0%
$Bkgr_{IRF}$[Cnts/Chnl]	0.74	±0.16	21%
$Shift_{IRF}$[ps]	-8.1	±1.2	14%
A_{Scatt}[kCnts]	62.39	±0.51	0.8%
τ_{AvInt}[ns]	5.223	±0.017	0.3%
τ_{AvAmp}[ns]	2.560	±0.013	0.5%
λ_{Det}[nm]	430.0000	---	---

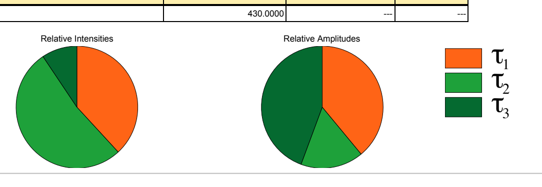

Data Set: 5 / 31

Decay: crv[5]; IRF: crv[0]

Fit

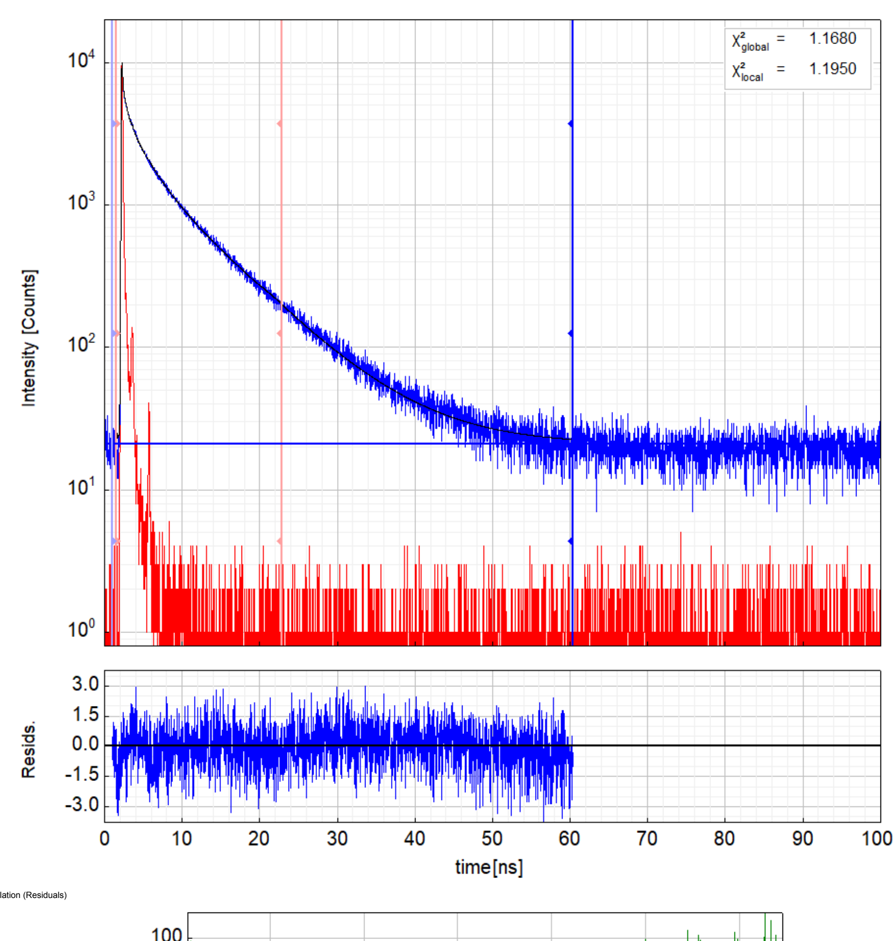

Autocorrelation Residuals

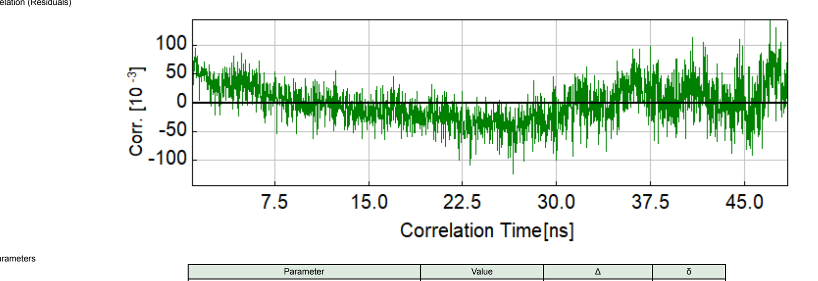

Fitted Parameters

Parameter	Value	Δ	δ
A_1[kCnts/Chnl]	2.475	±0.028	1.1%
τ_1[ns]	2.505	±0.013	0.5%
I_1[kCnts]	248.0	±2.2	0.9%
A_2[kCnts/Chnl]	2.106	±0.012	0.6%
τ_2[ns]	8.0397	±0.0067	0.1%
I_2[kCnts]	677.3	±2.5	0.4%
A_3[kCnts/Chnl]	3.288	±0.045	1.3%
τ_3[ns]	0.5435	±0.0070	1.3%
I_3[kCnts]	71.5	±1.8	2.4%
$Bkgr_{Dec}$[kCnts]	0.0209	±0.0002	0.8%
$Bkgr_{IRF}$[Cnts/Chnl]	-0.67	±0.12	18%
$Shift_{IRF}$[ps]	-2.97	±0.30	9.9%
A_{Scatt}[kCnts]	55.25	±0.41	0.7%
τ_{AvInt}[ns]	6.1253	±0.0097	0.2%
τ_{AvAmp}[ns]	3.1668	±0.0066	0.2%
λ_{Det}[nm]	440.0000	---	---

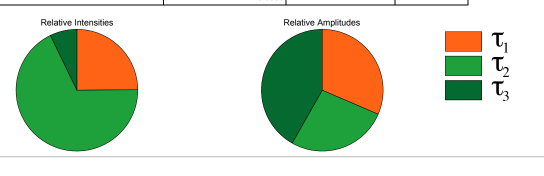

Data Set: 6 / 31

Decay: crv[6]; IRF: crv[0]

Fit

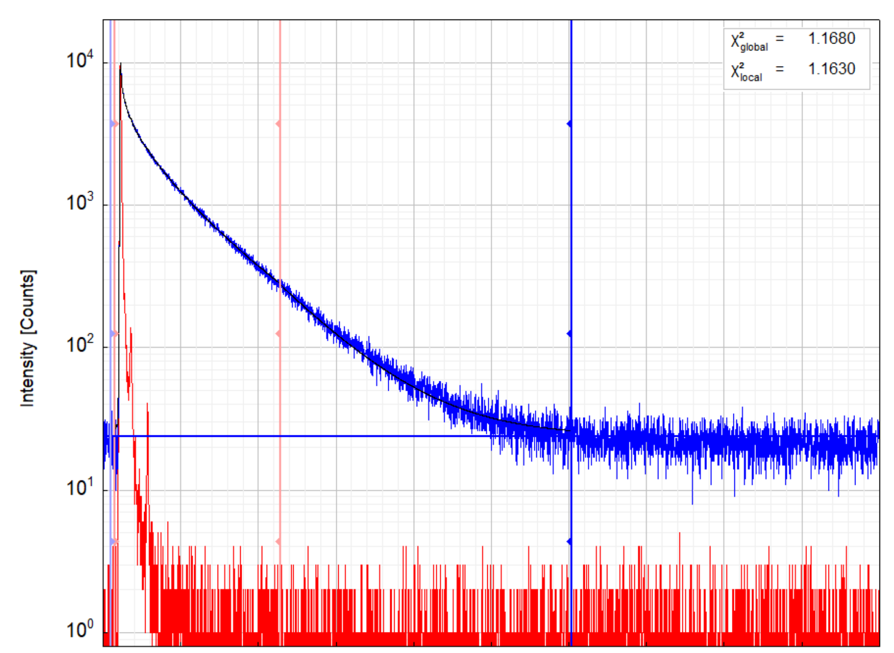

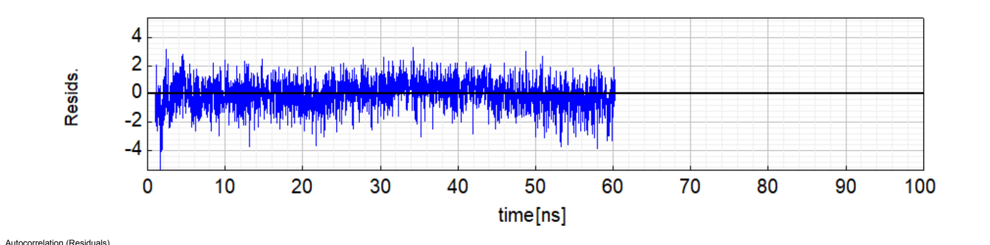

Autocorrelation (Residuals)

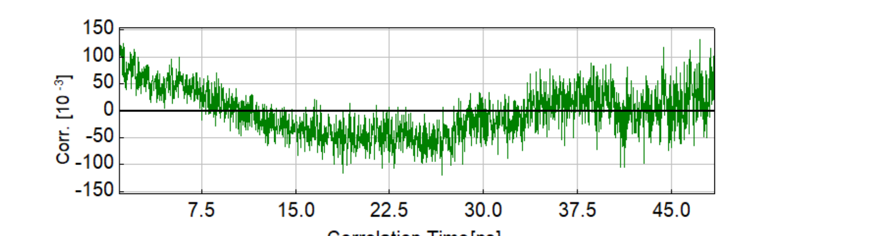

Fitted Parameters

Parameter	Value	Δ	δ
A_1[kCnts/Chnl]	2.085	±0.036	1.7%
$τ_1$[ns]	2.505	±0.013	0.5%
I_1[kCnts]	208.9	±3.6	1.7%
A_2[kCnts/Chnl]	2.863	±0.013	0.4%
$τ_2$[ns]	8.0397	±0.0067	0.1%
I_2[kCnts]	920.6	±5.5	0.6%
A_3[kCnts/Chnl]	3.315	±0.050	1.5%
$τ_3$[ns]	0.5435	±0.0070	1.3%
I_3[kCnts]	72.1	±1.6	2.1%
$Bkgr_{Dec}$[kCnts]	0.0238	±0.0003	1.2%
$Bkgr_{IRF}$[Cnts/Chnl]	-1.52	±0.21	14%
$Shift_{IRF}$[ps]	-1.47	±0.32	22%
A_{Scatt}[kCnts]	53.43	±0.44	0.8%
$τ_{AvInt}$[ns]	6.628	±0.017	0.2%
$τ_{AvAmp}$[ns]	3.636	±0.029	0.8%
$λ_{Det}$[nm]	450.0000	---	---

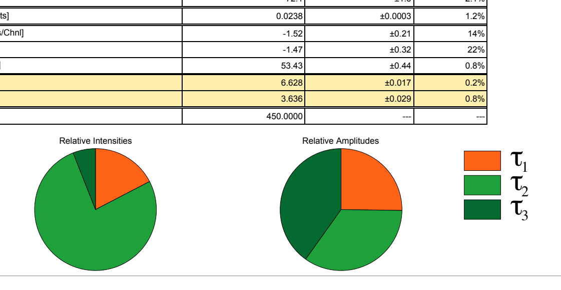

Data Set: 7 / 31

Decay: crv[7]; IRF: crv[0]

Fit

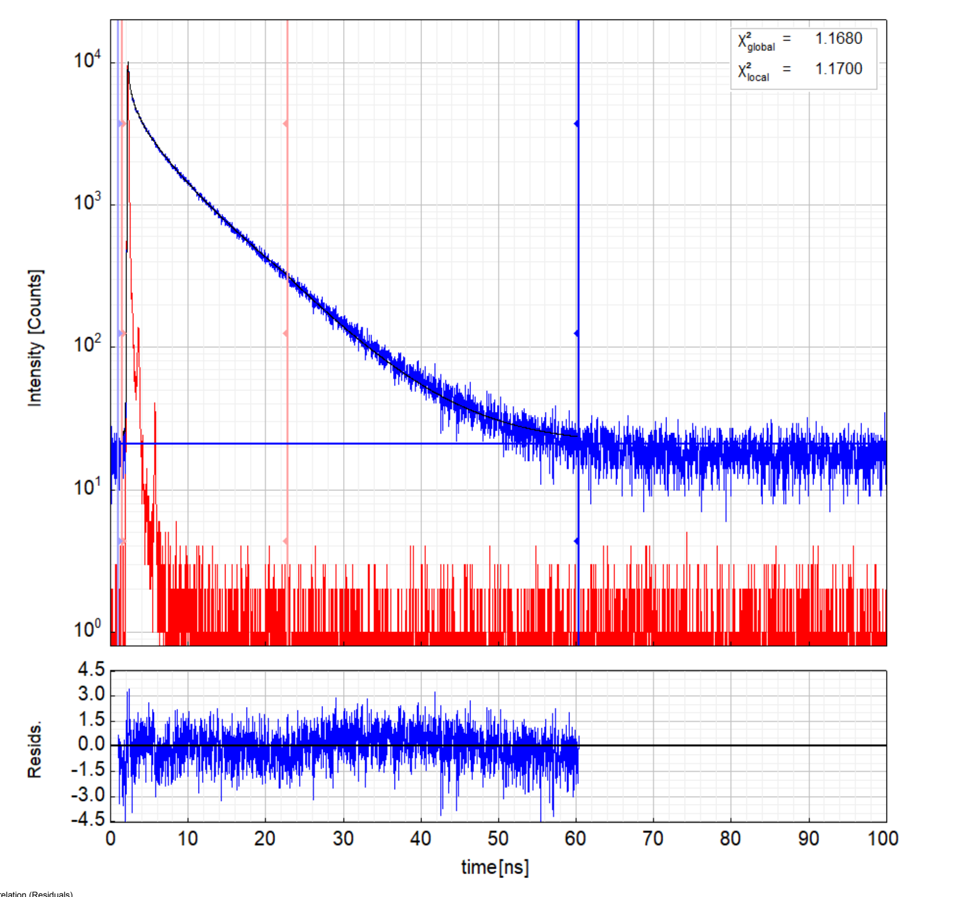

Autocorrelation (Residuals)

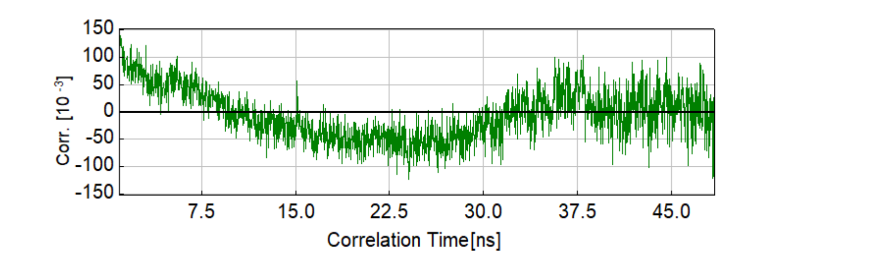

Fitted Parameters

Parameter	Value	Δ	δ
A_1[kCnts/Chnl]	2.000	±0.036	1.8%
$τ_1$[ns]	2.505	±0.013	0.5%
I_1[kCnts]	200.4	±4.1	2.0%
A_2[kCnts/Chnl]	3.3418	±0.0097	0.3%
$τ_2$[ns]	8.0397	±0.0067	0.1%
I_2[kCnts]	1 074.7	±2.3	0.2%
A_3[kCnts/Chnl]	3.382	±0.083	2.4%
$τ_3$[ns]	0.5435	±0.0070	1.3%
I_3[kCnts]	73.6	±2.5	3.3%
$Bkgr_{Dec}$[kCnts]	0.0209	±0.0004	1.5%
$Bkgr_{IRF}$[Cnts/Chnl]	-1.692	±0.086	5.1%
$Shift_{IRF}$[ps]	0.8	±1.3	171%
A_{Scatt}[kCnts]	53.2	±1.3	2.3%
$τ_{AvInt}$[ns]	6.8088	±0.0075	0.1%
$τ_{AvAmp}$[ns]	3.866	±0.028	0.7%
$λ_{Det}$[nm]	460.0000	---	---

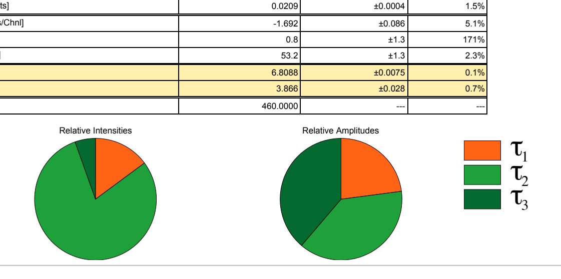

Data Set: 8 / 31

Decay: crv[8]; IRF: crv[0]

Fit

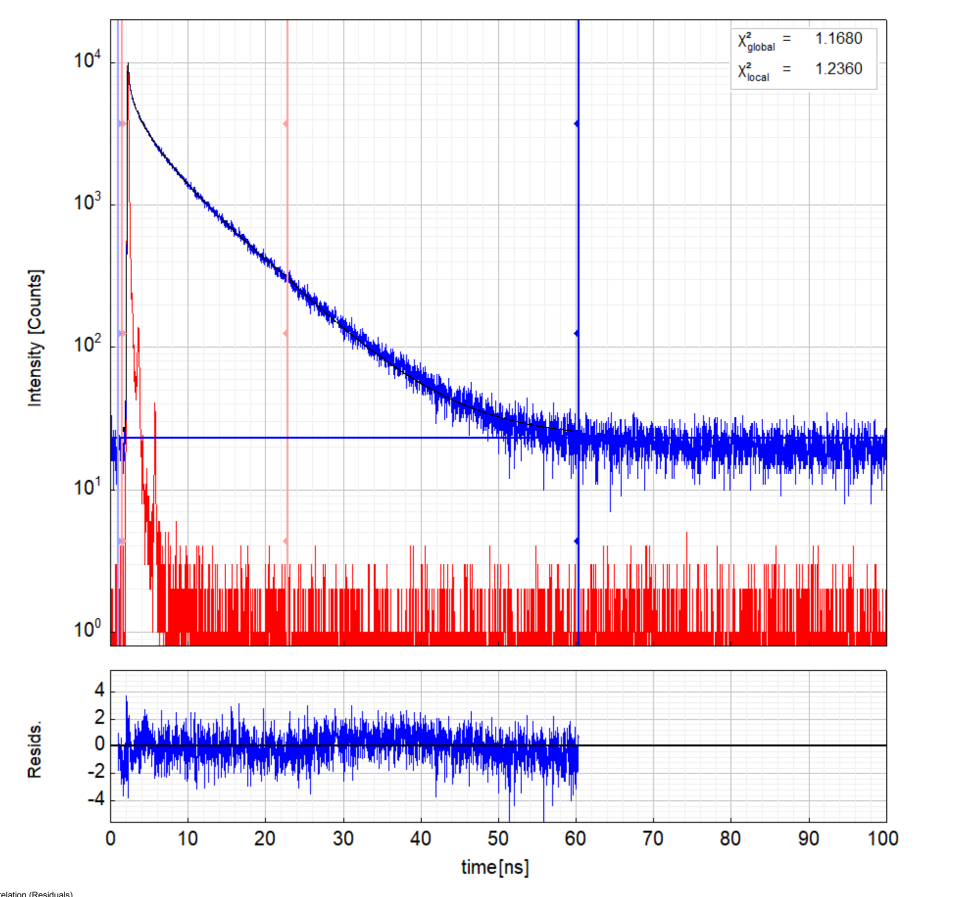

Autocorrelation (Residuals)

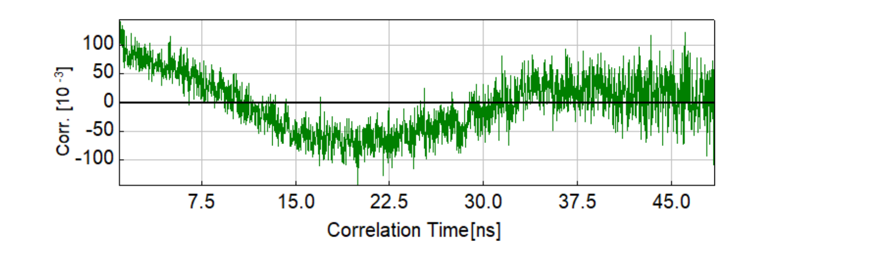

Fitted Parameters

Parameter	Value	Δ	δ
A_1[kCnts/Chnl]	2.200	±0.037	1.7%
$τ_1$[ns]	2.505	±0.013	0.5%
I_1[kCnts]	220.5	±2.7	1.2%
A_2[kCnts/Chnl]	3.253	±0.012	0.4%
$τ_2$[ns]	8.0397	±0.0067	0.1%
I_2[kCnts]	1 045.9	±4.2	0.4%
A_3[kCnts/Chnl]	3.154	±0.091	2.9%
$τ_3$[ns]	0.5435	±0.0070	1.3%
I_3[kCnts]	68.6	±2.5	3.6%
$Bkgr_{Dec}$[kCnts]	0.0230	±0.0002	0.5%
$Bkgr_{IRF}$[Cnts/Chnl]	-1.33	±0.21	15%
$Shift_{IRF}$[ps]	0.8	±1.3	165%
A_{Scatt}[kCnts]	51.3	±1.9	3.6%
$τ_{AvInt}$[ns]	6.741	±0.014	0.2%
$τ_{AvAmp}$[ns]	3.878	±0.032	0.8%
$λ_{Det}$[nm]	470.0000	---	---

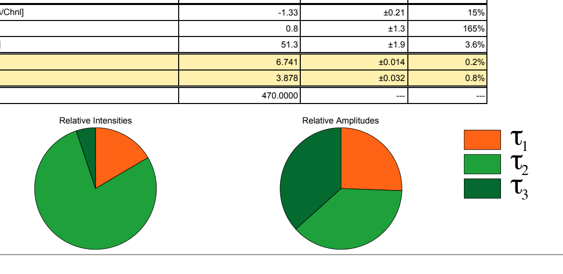

Data Set: 9 / 31

Decay: crv[9]; IRF: crv[0]

Fit

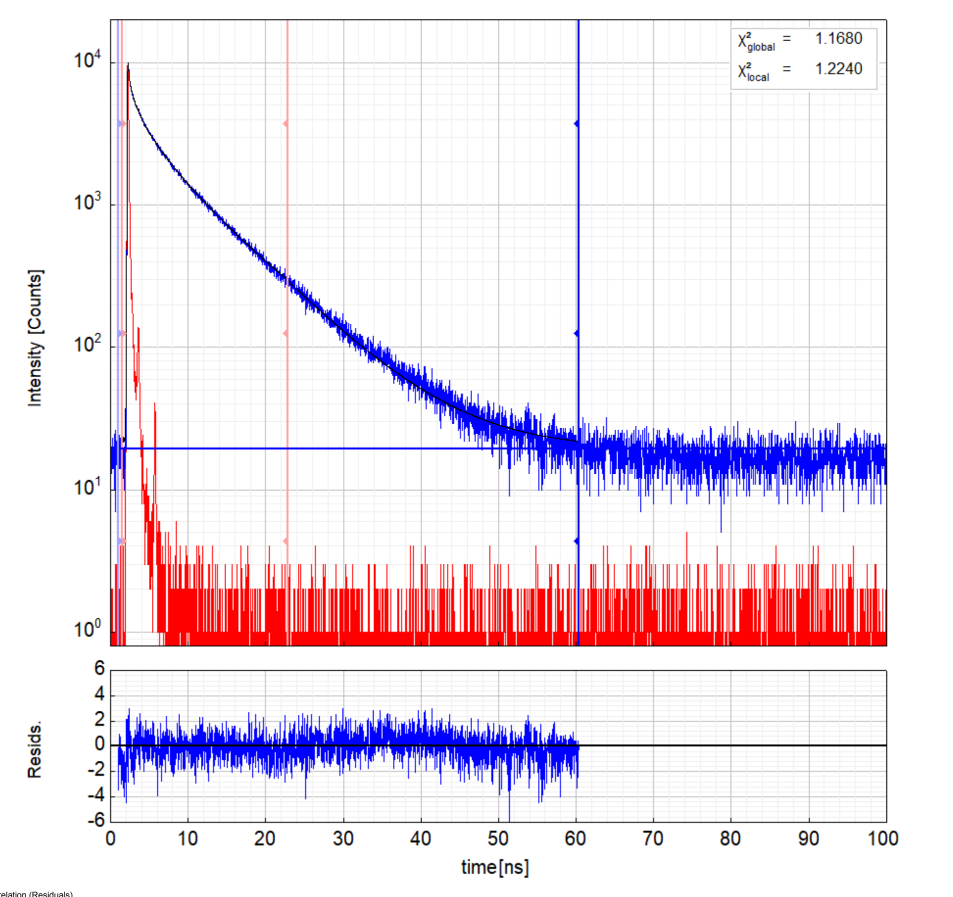

Autocorrelation (Residuals)

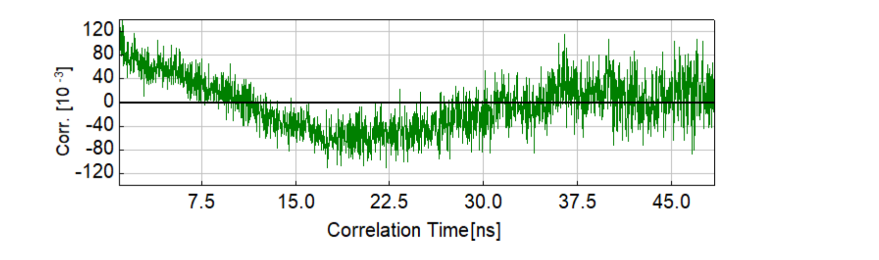

Fitted Parameters

Parameter	Value	Δ	δ
A_1[kCnts/Chnl]	2.574	±0.028	1.1%
$τ_1$[ns]	2.505	±0.013	0.5%
I_1[kCnts]	257.9	±2.0	0.7%
A_2[kCnts/Chnl]	3.2222	±0.0096	0.3%
$τ_2$[ns]	8.0397	±0.0067	0.1%
I_2[kCnts]	1 036.2	±3.4	0.3%
A_3[kCnts/Chnl]	3.484	±0.053	1.5%
$τ_3$[ns]	0.5435	±0.0070	1.3%
I_3[kCnts]	75.8	±2.0	2.6%
$Bkgr_{Dec}$[kCnts]	0.0194	±0.0003	1.1%
$Bkgr_{IRF}$[Cnts/Chnl]	-0.87	±0.12	14%
$Shift_{IRF}$[ps]	0.63	±0.74	117%
A_{Scatt}[kCnts]	49.4	±1.2	2.3%
$τ_{AvInt}$[ns]	6.584	±0.014	0.2%
$τ_{AvAmp}$[ns]	3.691	±0.016	0.4%
$λ_{Det}$[nm]	480.0000	---	---

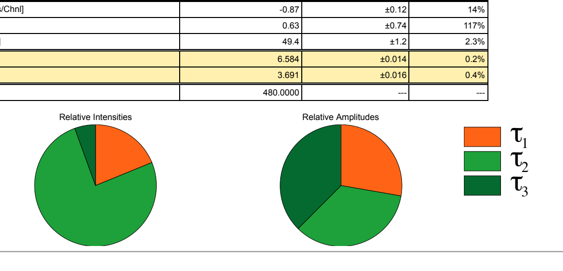

Data Set: 10 / 31

Decay: crv[10]; IRF: crv[0]

Fit

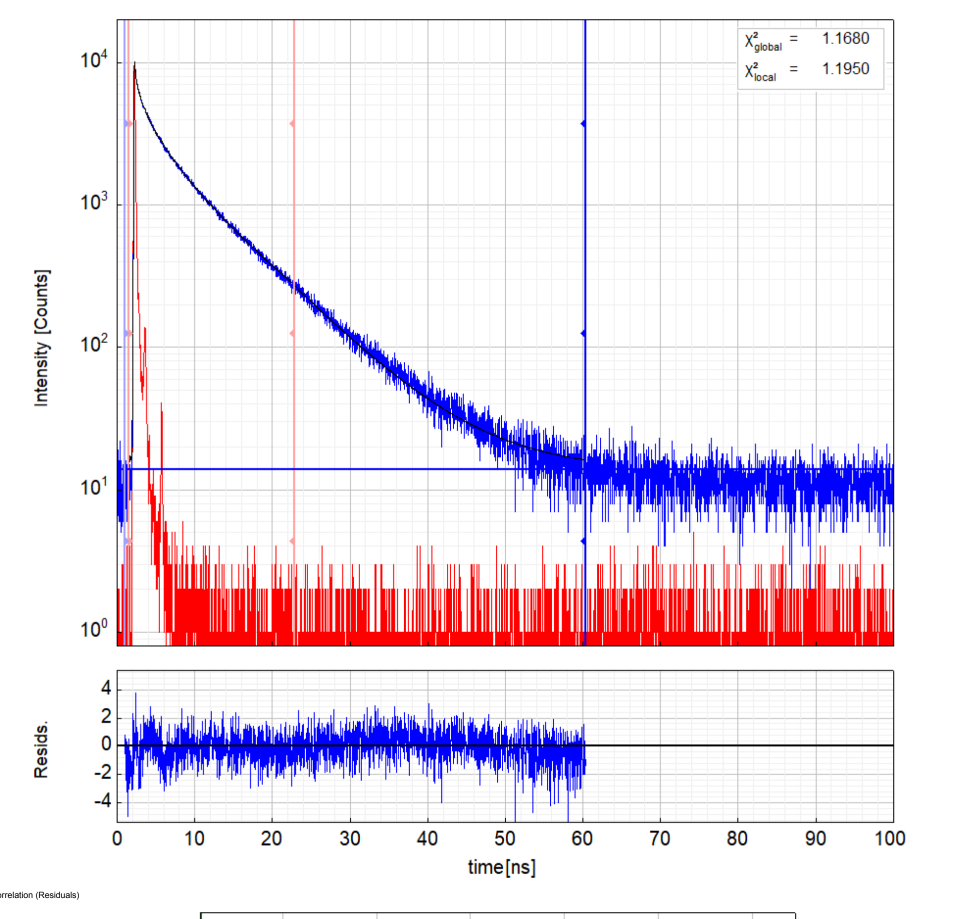

Autocorrelation (Residuals)

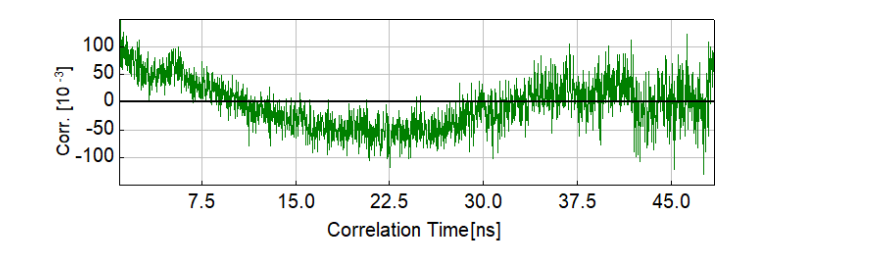

Fitted Parameters

Parameter	Value	Δ	δ
A_1[kCnts/Chnl]	2.975	±0.052	1.7%
$τ_1$[ns]	2.505	±0.013	0.5%
I_1[kCnts]	298.1	±6.0	2.0%
A_2[kCnts/Chnl]	3.053	±0.015	0.5%
$τ_2$[ns]	8.0397	±0.0067	0.1%
I_2[kCnts]	981.6	±5.9	0.6%
A_3[kCnts/Chnl]	3.55	±0.17	4.7%
$τ_3$[ns]	0.5435	±0.0070	1.3%
I_3[kCnts]	77.1	±3.7	4.8%
$Bkgr_{Dec}$[kCnts]	0.0139	±0.0002	1.1%
$Bkgr_{IRF}$[Cnts/Chnl]	-0.59	±0.18	30%
$Shift_{IRF}$[ps]	2.3	±1.1	45%
A_{Scatt}[kCnts]	48.78	±0.60	1.2%
$τ_{AvInt}$[ns]	6.3979	±0.0082	0.1%
$τ_{AvAmp}$[ns]	3.544	±0.031	0.9%
$λ_{Det}$[nm]	490.0000	---	---

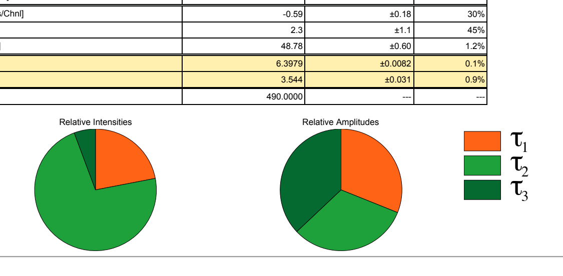

Data Set: 11 / 31

Decay: crv[11]; IRF: crv[0]

Fit

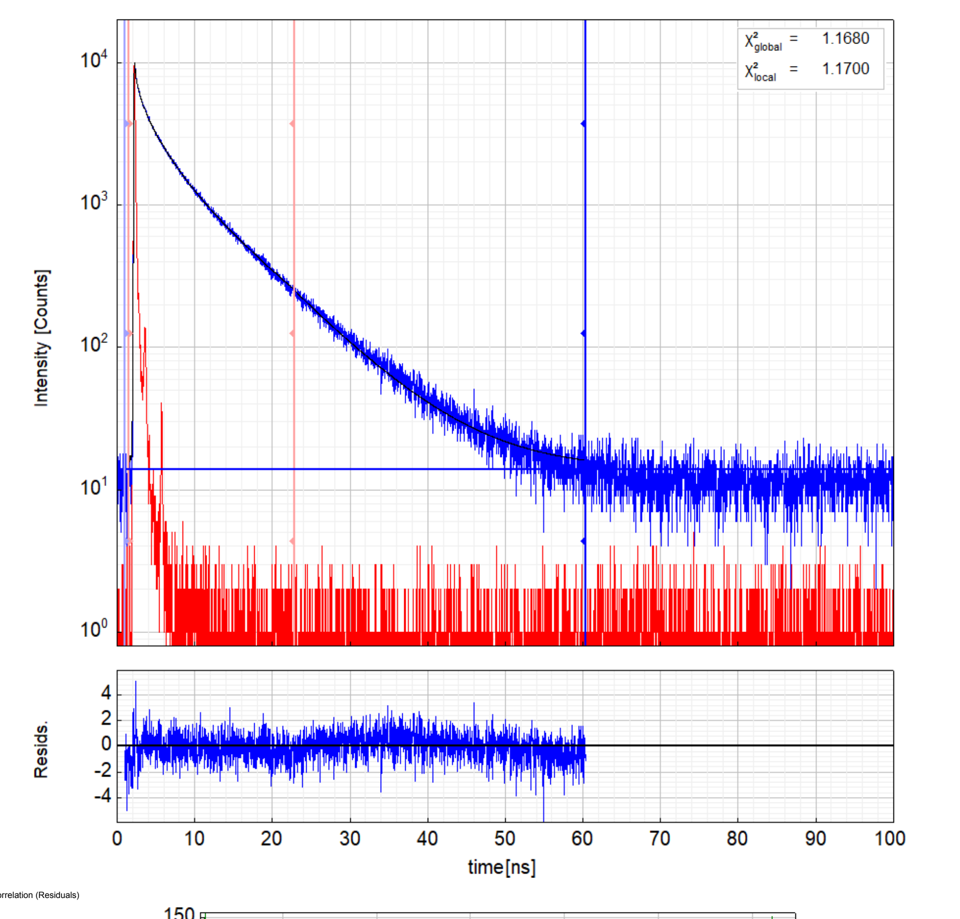

Autocorrelation (Residuals)

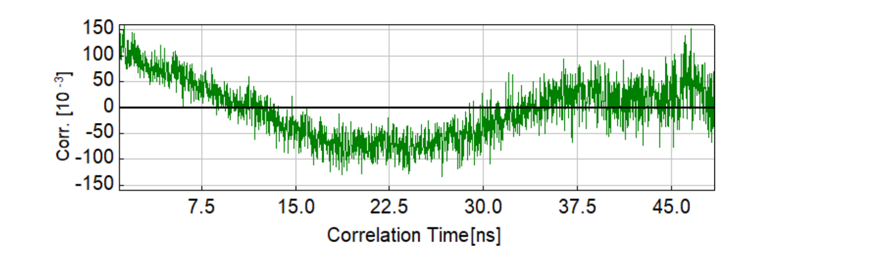

Fitted Parameters

Parameter	Value	Δ	δ
A_1[kCnts/Chnl]	3.356	±0.021	0.6%
$τ_1$[ns]	2.505	±0.013	0.5%
I_1[kCnts]	336.3	±2.8	0.8%
A_2[kCnts/Chnl]	2.7948	±0.0082	0.3%
$τ_2$[ns]	8.0397	±0.0067	0.1%
I_2[kCnts]	898.8	±4.2	0.5%
A_3[kCnts/Chnl]	3.560	±0.037	1.0%
$τ_3$[ns]	0.5435	±0.0070	1.3%
I_3[kCnts]	77.4	±1.2	1.4%
$Bkgr_{Dec}$[kCnts]	0.0140	±0.0002	1.3%
$Bkgr_{IRF}$[Cnts/Chnl]	-0.60	±0.11	18%
$Shift_{IRF}$[ps]	3.3	±1.1	31%
A_{Scatt}[kCnts]	47.23	±0.64	1.3%
$τ_{AvInt}$[ns]	6.180	±0.011	0.2%
$τ_{AvAmp}$[ns]	3.3790	±0.0092	0.3%
$λ_{Det}$[nm]	500.0000	---	---

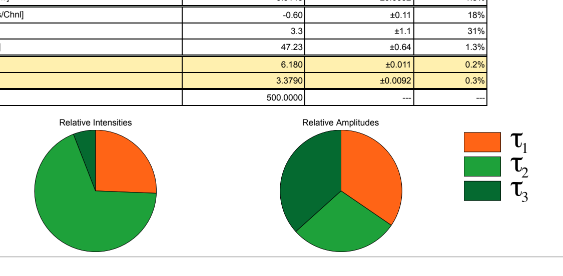

Data Set: 12 / 31

Decay: crv[12]; IRF: crv[0]

Fit

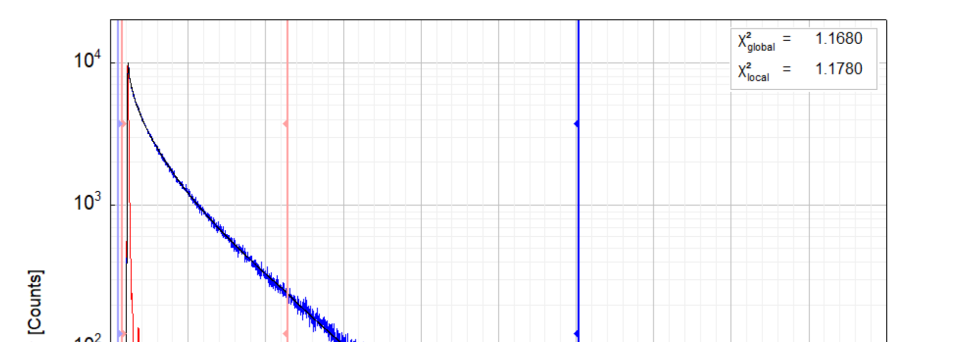

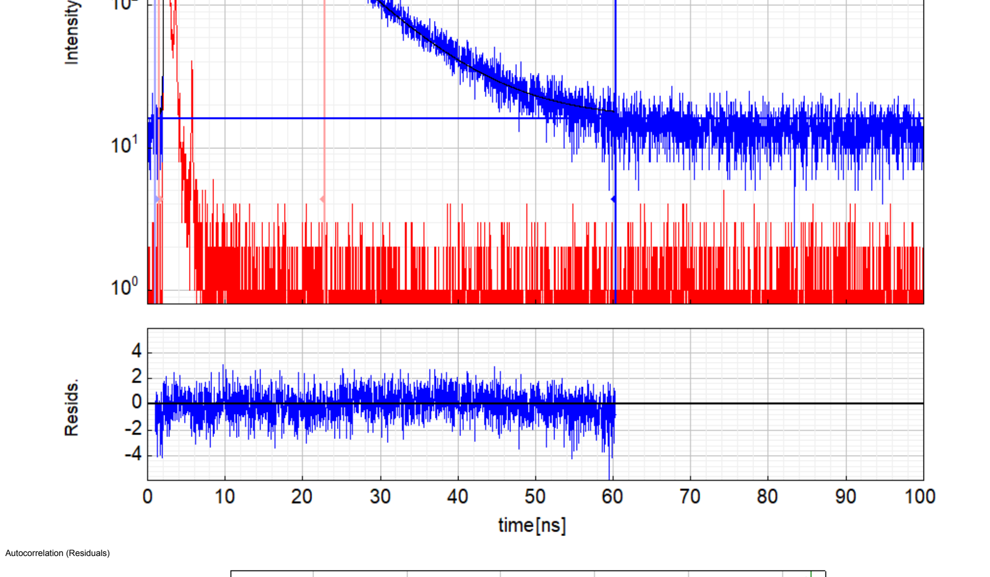

Autocorrelation (Residuals)

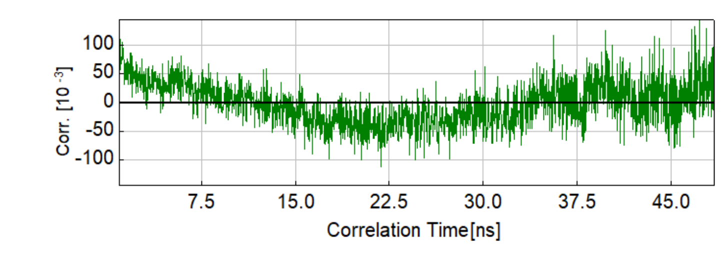

Fitted Parameters

Parameter	Value	Δ	δ
A_1[kCnts/Chnl]	3.592	±0.037	1.0%
τ_1[ns]	2.505	±0.013	0.5%
I_1[kCnts]	359.9	±3.0	0.8%
A_2[kCnts/Chnl]	2.650	±0.015	0.6%
τ_2[ns]	8.0397	±0.0067	0.1%
I_2[kCnts]	852.0	±4.2	0.5%
A_3[kCnts/Chnl]	3.640	±0.079	2.1%
τ_3[ns]	0.5435	±0.0070	1.3%
I_3[kCnts]	79.2	±1.3	1.5%
$Bkgr_{Dec}$[kCnts]	0.0159	±0.0004	2.0%
$Bkgr_{IRF}$[Cnts/Chnl]	-0.34	±0.14	39%
$Shift_{IRF}$[ps]	3.08	±0.82	26%
A_{Scatt}[kCnts]	46.44	±0.53	1.1%
τ_{AvInt}[ns]	6.038	±0.016	0.3%
τ_{AvAmp}[ns]	3.267	±0.019	0.6%
λ_{Det}[nm]	510.0000	---	---

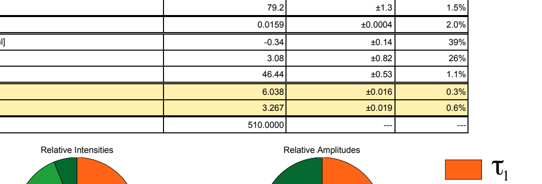

Data Set: 13 / 31

Decay: crv[13]; IRF: crv[0]

Fit

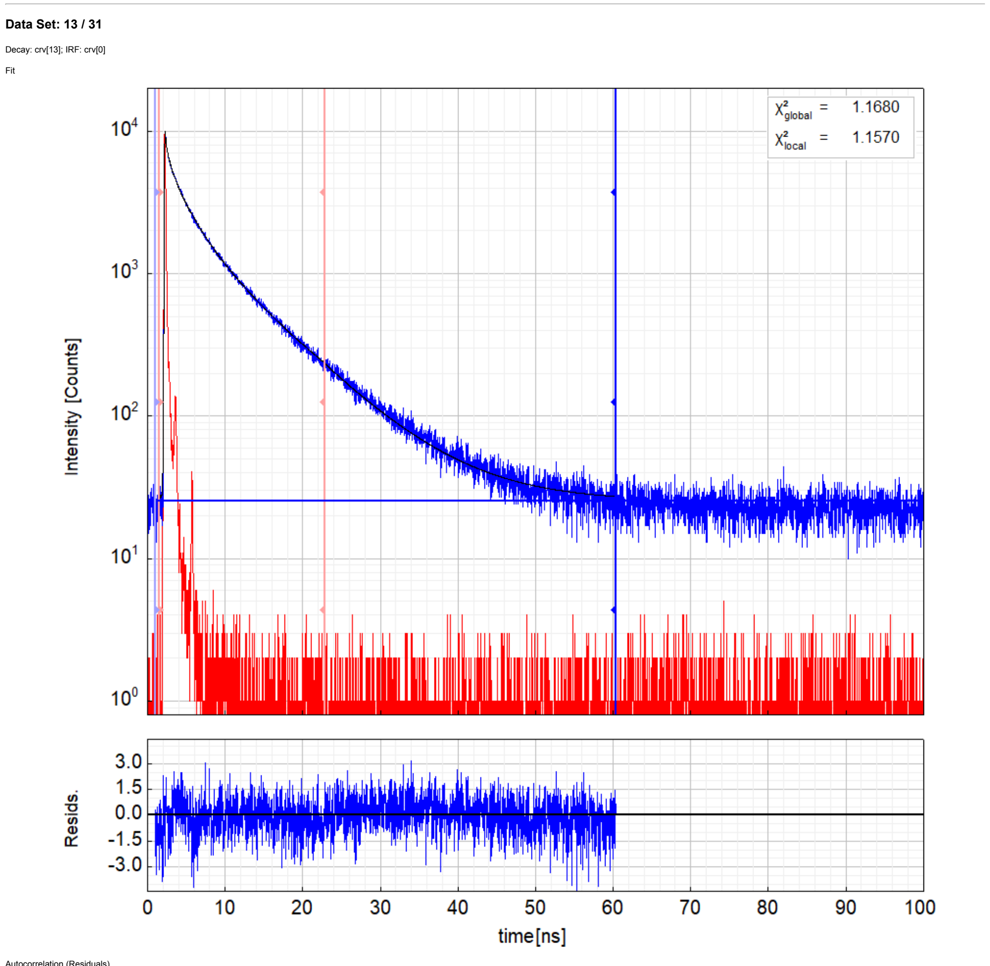

Autocorrelation (Residuals)

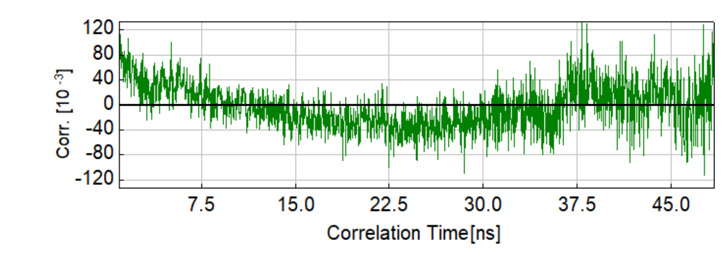

Fitted Parameters

Parameter	Value	Δ	δ
A_1[kCnts/Chnl]	3.636	±0.019	0.5%
τ_1[ns]	2.505	±0.013	0.5%
I_1[kCnts]	364.3	±2.7	0.7%
A_2[kCnts/Chnl]	2.499	±0.014	0.5%
τ_2[ns]	8.0397	±0.0067	0.1%
I_2[kCnts]	803.5	±4.2	0.5%
A_3[kCnts/Chnl]	3.522	±0.059	1.7%
τ_3[ns]	0.5435	±0.0070	1.3%
I_3[kCnts]	76.6	±1.3	1.6%
$Bkgr_{Dec}$[kCnts]	0.0254	±0.0003	0.8%
$Bkgr_{IRF}$[Cnts/Chnl]	-0.17	±0.15	89%
$Shift_{IRF}$[ps]	5.56	±0.33	5.9%
A_{Scatt}[kCnts]	46.65	±0.63	1.3%
τ_{AvInt}[ns]	5.959	±0.011	0.2%
τ_{AvAmp}[ns]	3.222	±0.013	0.4%
λ_{Det}[nm]	520.0000	---	---

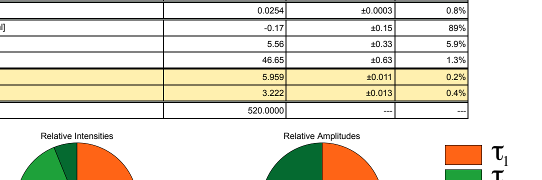

Data Set: 14 / 31

Decay: crv[14]; IRF: crv[0]

Fit

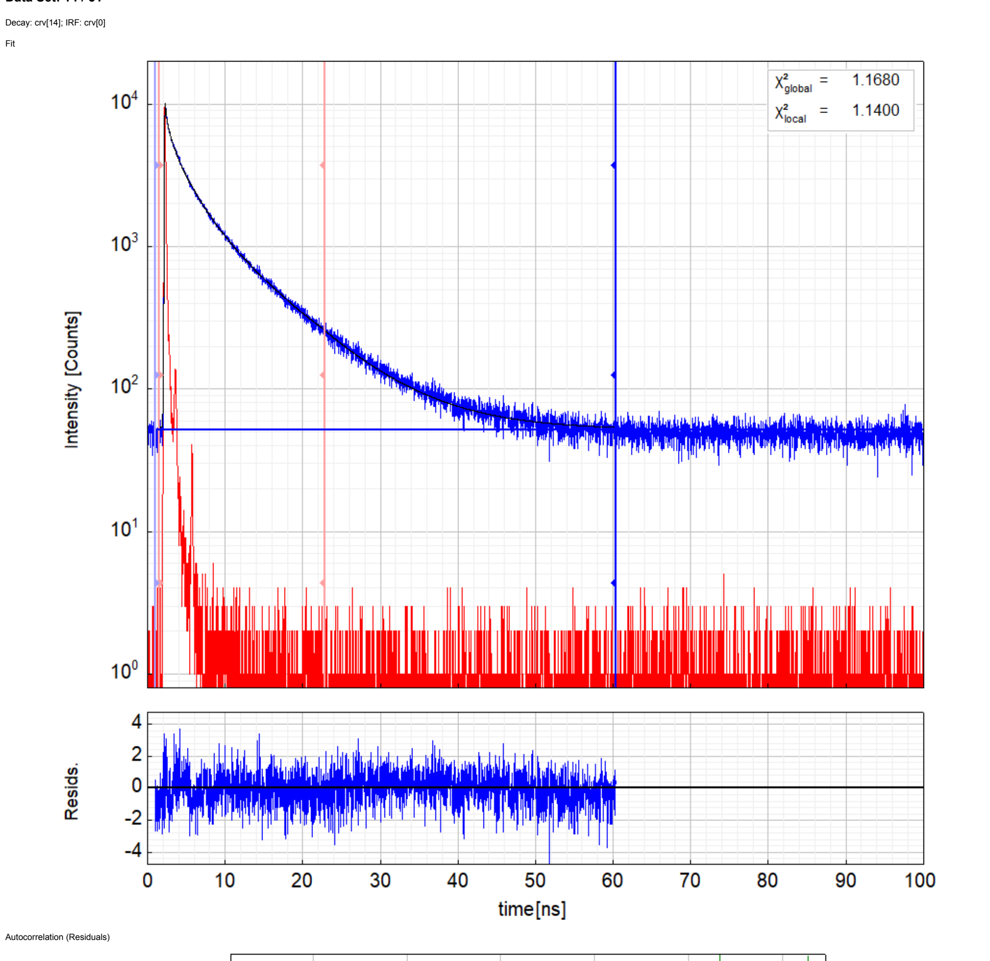

Autocorrelation (Residuals)

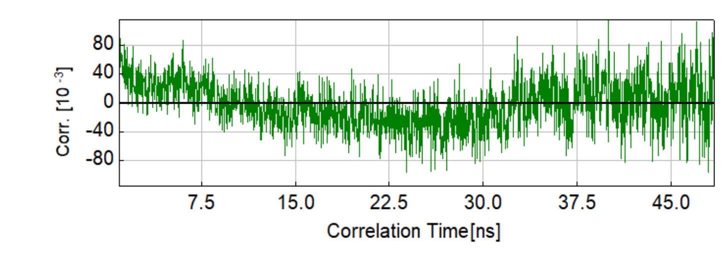

Fitted Parameters

Parameter	Value	Δ	δ
A_1[kCnts/Chnl]	3.792	±0.055	1.4%
τ_1[ns]	2.505	±0.013	0.5%
I_1[kCnts]	379.9	±4.7	1.2%
A_2[kCnts/Chnl]	2.453	±0.015	0.6%
τ_2[ns]	8.0397	±0.0067	0.1%
I_2[kCnts]	788.9	±4.9	0.6%
A_3[kCnts/Chnl]	3.77	±0.11	2.9%
τ_3[ns]	0.5435	±0.0070	1.3%
I_3[kCnts]	81.9	±2.6	3.1%
$Bkgr_{Dec}$[kCnts]	0.0520	±0.0003	0.5%
$Bkgr_{IRF}$[Cnts/Chnl]	-0.21	±0.16	73%
$Shift_{IRF}$[ps]	6.6	±1.1	16%
A_{Scatt}[kCnts]	46.72	±0.48	1.0%
τ_{AvInt}[ns]	5.8677	±0.0065	0.1%
τ_{AvAmp}[ns]	3.123	±0.026	0.8%
λ_{Det}[nm]	530.0000	---	---

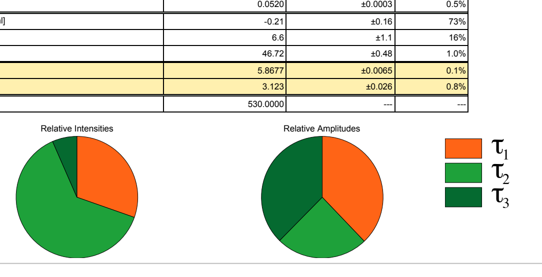

Data Set: 15 / 31

Decay: crv[15]; IRF: crv[0]

Fit

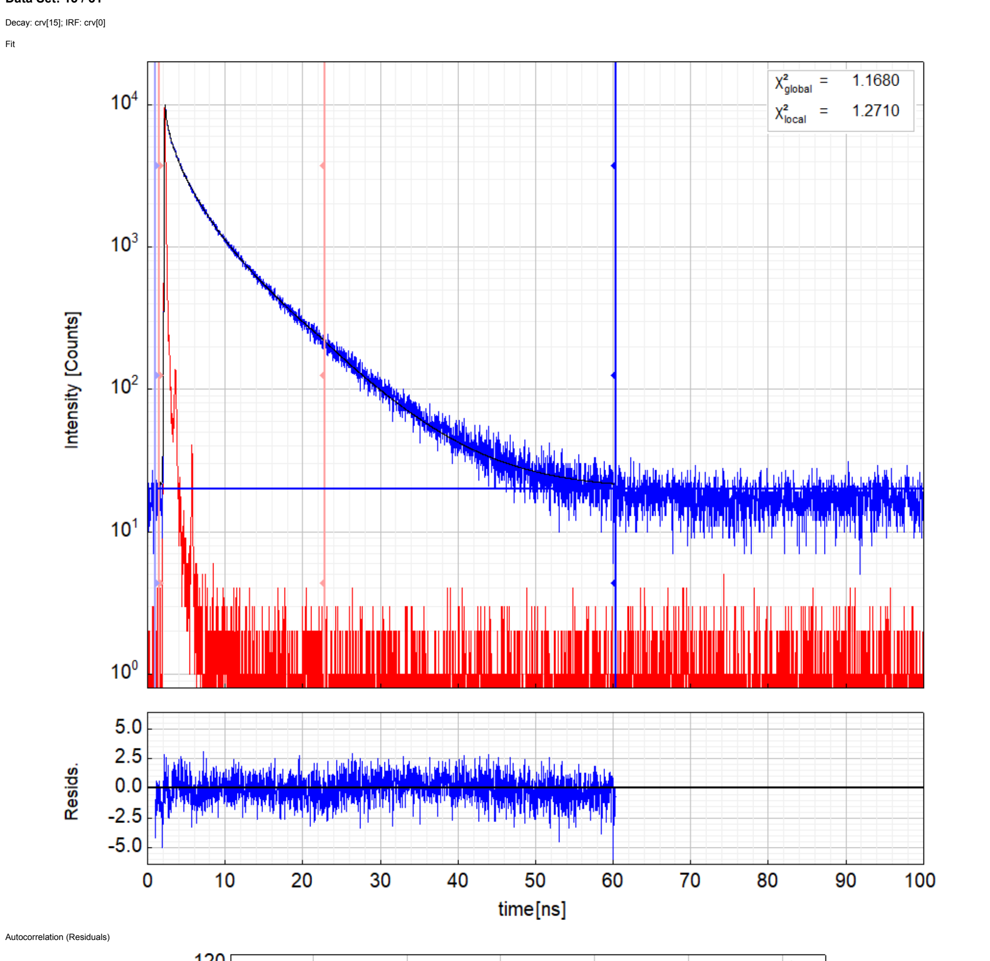

Autocorrelation (Residuals)

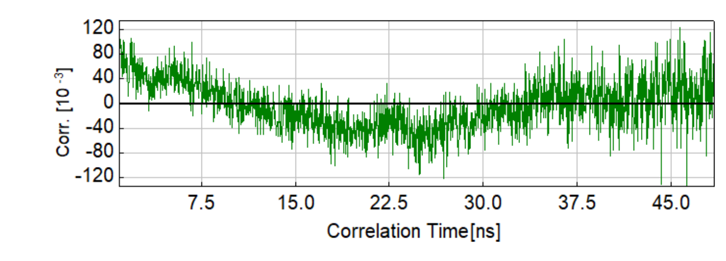

Fitted Parameters

Parameter	Value	Δ	δ
A_1[kCnts/Chnl]	3.755	±0.032	0.8%
τ_1[ns]	2.505	±0.013	0.5%
I_1[kCnts]	376.3	±2.2	0.6%
A_2[kCnts/Chnl]	2.376	±0.012	0.5%
τ_2[ns]	8.0397	±0.0067	0.1%
I_2[kCnts]	764.1	±4.2	0.5%
A_3[kCnts/Chnl]	3.829	±0.093	2.4%
τ_3[ns]	0.5435	±0.0070	1.3%
I_3[kCnts]	83.3	±1.8	2.1%
$Bkgr_{Dec}$[kCnts]	0.0199	±0.0003	1.3%
$Bkgr_{IRF}$[Cnts/Chnl]	-0.11	±0.12	104%
$Shift_{IRF}$[ps]	7.53	±0.74	9.7%
A_{Scatt}[kCnts]	45.94	±0.50	1.1%
τ_{AvInt}[ns]	5.828	±0.015	0.3%
τ_{AvAmp}[ns]	3.072	±0.023	0.7%
λ_{Det}[nm]	540.0000	---	---

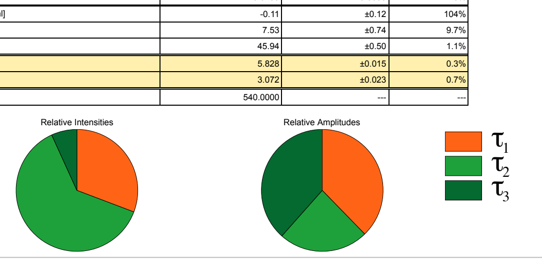

Data Set: 16 / 31

Decay: crv[16]; IRF: crv[0]

Fit

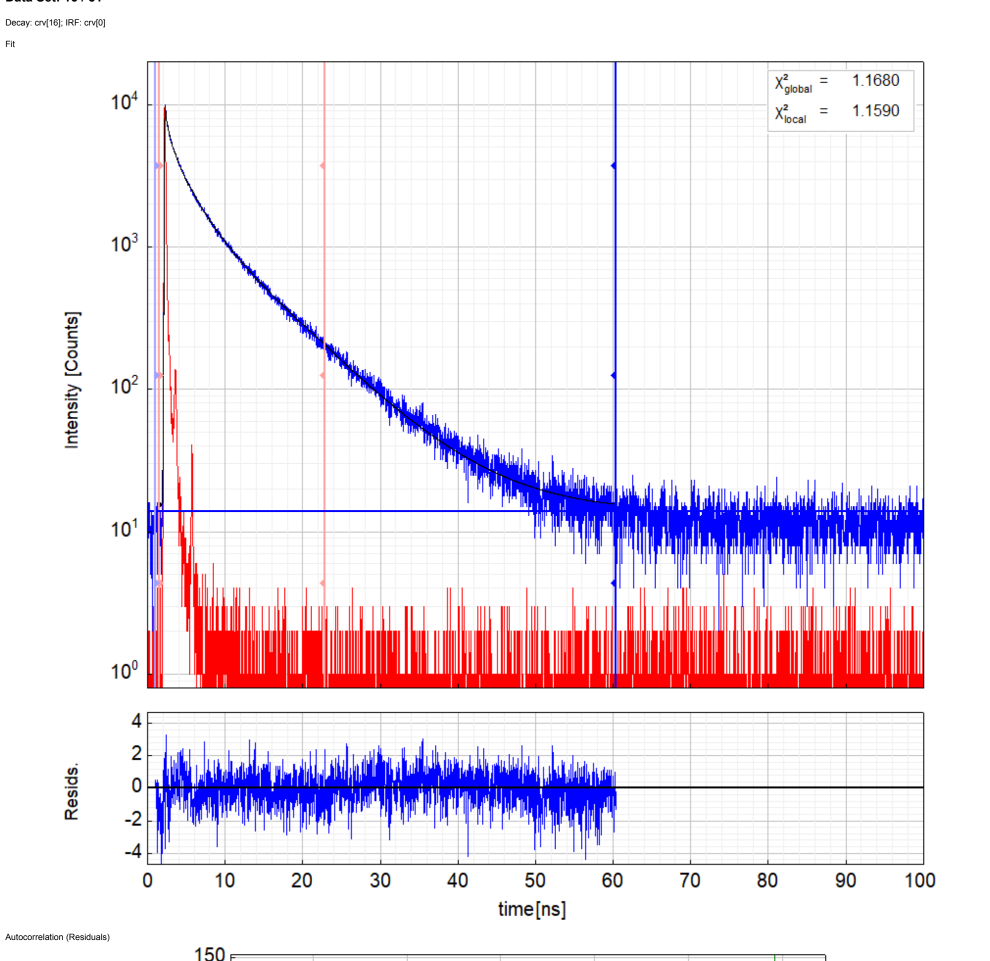

Autocorrelation (Residuals)

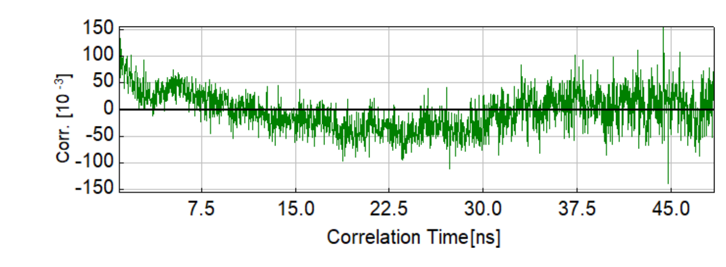

Fitted Parameters

Parameter	Value	Δ	δ
A_1[kCnts/Chnl]	3.640	±0.027	0.7%
τ_1[ns]	2.505	±0.013	0.5%
I_1[kCnts]	364.7	±3.3	0.9%
A_2[kCnts/Chnl]	2.3325	±0.0070	0.3%
τ_2[ns]	8.0397	±0.0067	0.1%
I_2[kCnts]	750.1	±3.6	0.5%
A_3[kCnts/Chnl]	3.799	±0.098	2.6%
τ_3[ns]	0.5435	±0.0070	1.3%
I_3[kCnts]	82.6	±2.5	3.0%
$Bkgr_{Dec}$[kCnts]	0.0139	±0.0002	1.4%
$Bkgr_{IRF}$[Cnts/Chnl]	0.04	±0.13	358%
$Shift_{IRF}$[ps]	7.95	±0.25	3.1%
A_{Scatt}[kCnts]	45.70	±0.51	1.1%
τ_{AvInt}[ns]	5.837	±0.012	0.2%
τ_{AvAmp}[ns]	3.064	±0.024	0.8%
λ_{Det}[nm]	550.0000	---	---

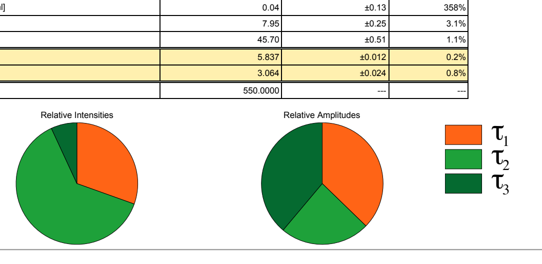

Data Set: 17 / 31

Decay: crv[17]; IRF: crv[0]

Fit

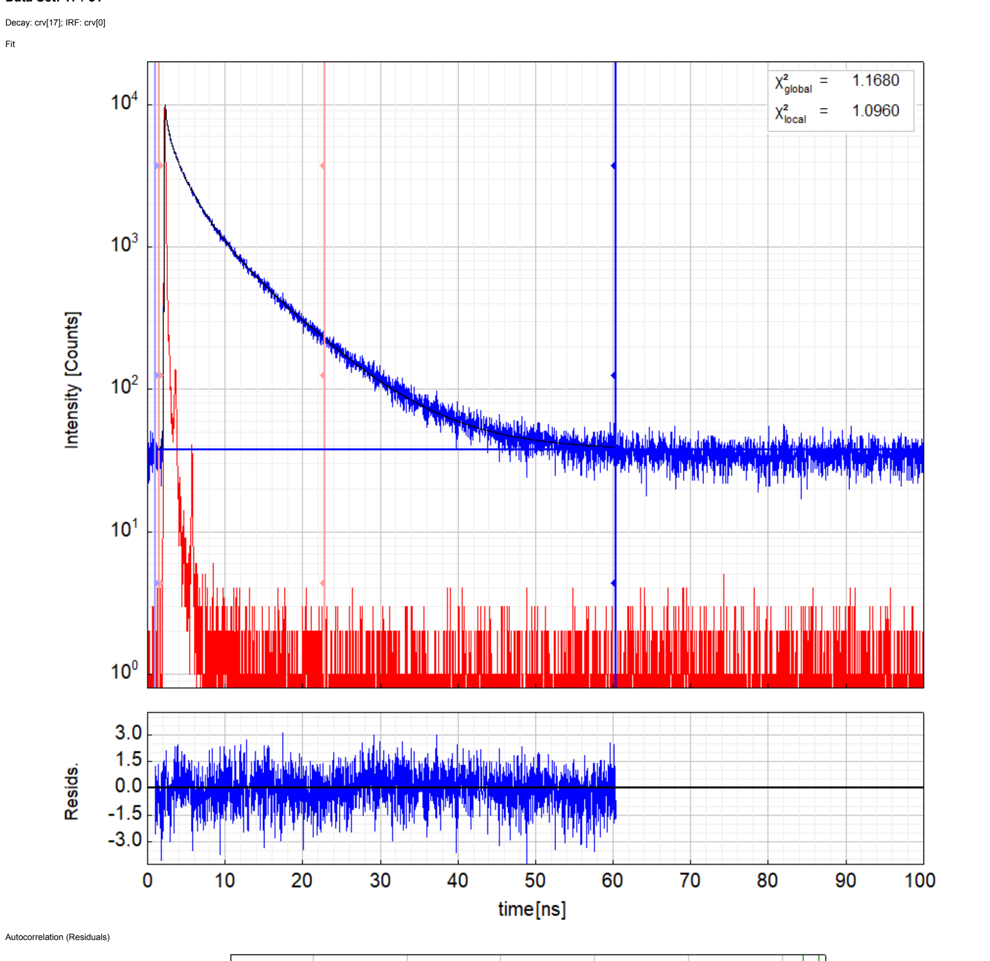

Autocorrelation (Residuals)

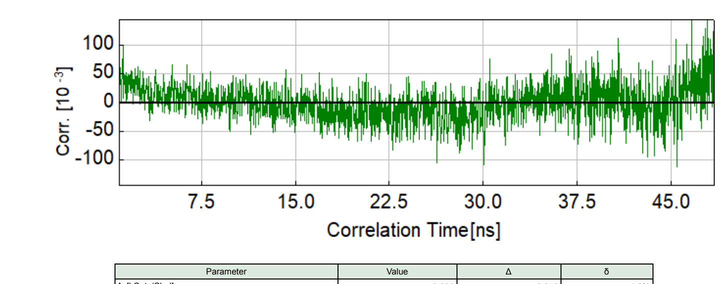

Fitted Parameters

Parameter	Value	Δ	δ
A_1[kCnts/Chnl]	3.633	±0.043	1.2%
τ_1[ns]	2.505	±0.013	0.5%
I_1[kCnts]	364.0	±2.1	0.6%
A_2[kCnts/Chnl]	2.298	±0.011	0.5%
τ_2[ns]	8.0397	±0.0067	0.1%
I_2[kCnts]	738.9	±3.2	0.4%
A_3[kCnts/Chnl]	3.946	±0.026	0.6%
τ_3[ns]	0.5435	±0.0070	1.3%
I_3[kCnts]	85.8	±1.7	2.0%
$Bkgr_{Dec}$[kCnts]	0.0377	±0.0002	0.5%
$Bkgr_{IRF}$[Cnts/Chnl]	0.006	±0.081	1489%
$Shift_{IRF}$[ps]	8.67	±0.57	6.5%
A_{Scatt}[kCnts]	45.74	±0.51	1.1%
τ_{AvInt}[ns]	5.804	±0.012	0.2%
τ_{AvAmp}[ns]	3.009	±0.012	0.4%
λ_{Det}[nm]	560.0000	---	---

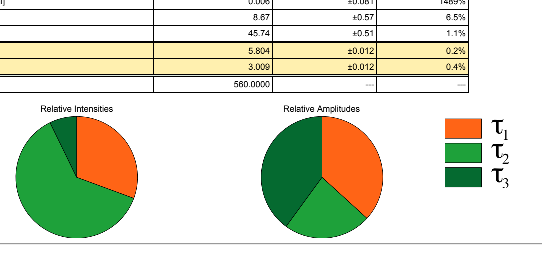

Data Set: 18 / 31

Decay: crv[18]; IRF: crv[0]

Fit

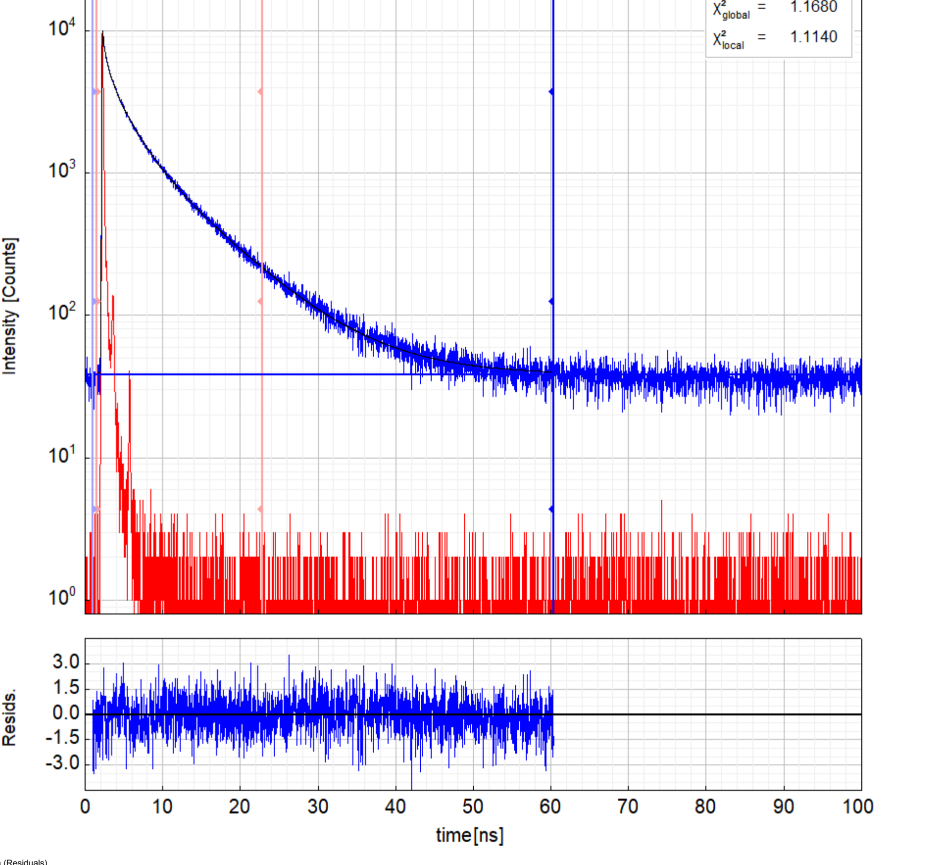

Autocorrelation (Residuals)

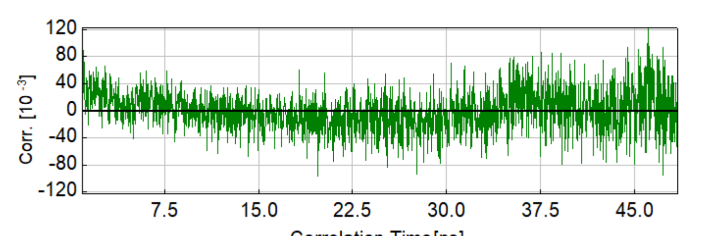

Fitted Parameters

Parameter	Value	Δ	δ
A_1[kCnts/Chnl]	3.493	±0.045	1.3%
$τ_1$[ns]	2.505	±0.013	0.5%
I_1[kCnts]	350.0	±2.8	0.8%
A_2[kCnts/Chnl]	2.226	±0.011	0.5%
$τ_2$[ns]	8.0397	±0.0067	0.1%
I_2[kCnts]	715.7	±2.4	0.3%
A_3[kCnts/Chnl]	4.150	±0.078	1.9%
$τ_3$[ns]	0.5435	±0.0070	1.3%
I_3[kCnts]	90.2	±3.0	3.2%
$Bkgr_{Dec}$[kCnts]	0.0385	±0.0003	0.6%
$Bkgr_{IRF}$[Cnts/Chnl]	0.512	±0.081	16%
$Shift_{IRF}$[ps]	9.09	±0.77	8.5%
A_{Scatt}[kCnts]	45.5	±1.3	2.8%
$τ_{AvInt}$[ns]	5.779	±0.011	0.2%
$τ_{AvAmp}$[ns]	2.929	±0.022	0.7%
$λ_{Det}$[nm]	570.0000	---	---

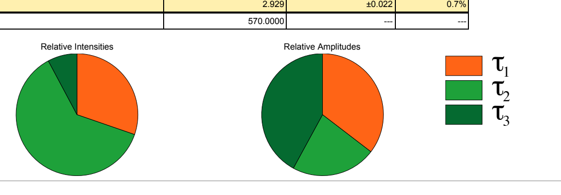

Data Set: 19 / 31

Decay: crv[19]; IRF: crv[0]

Fit

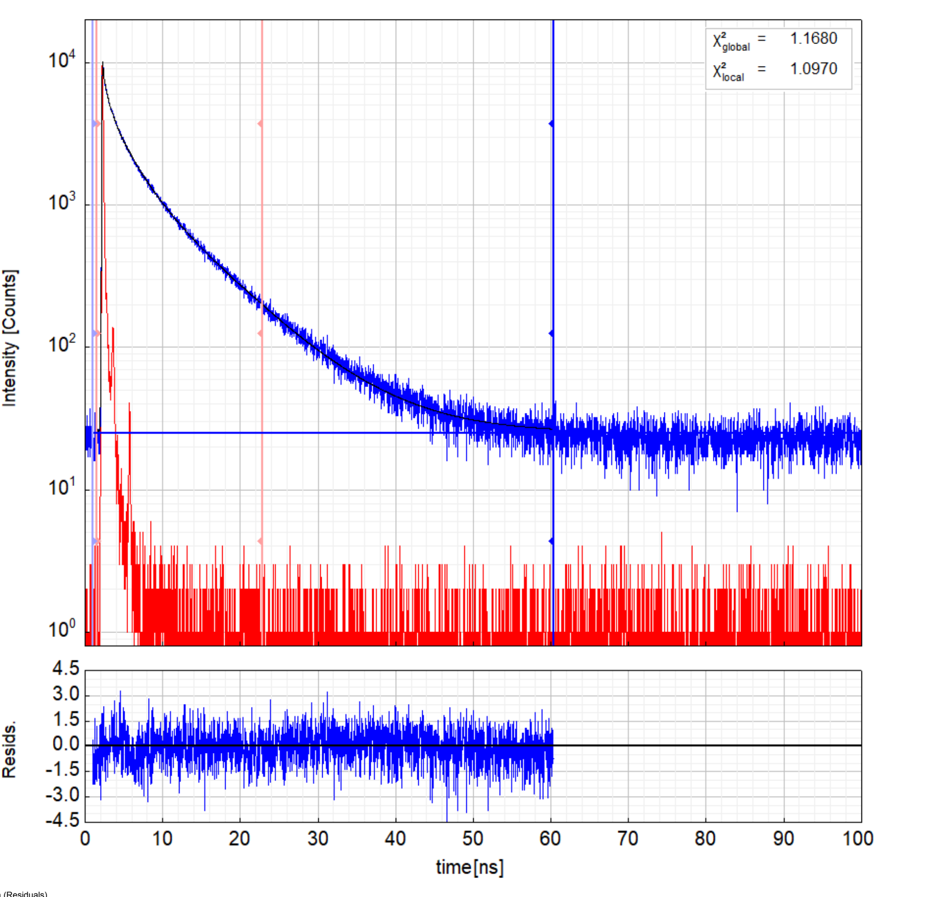

Autocorrelation (Residuals)

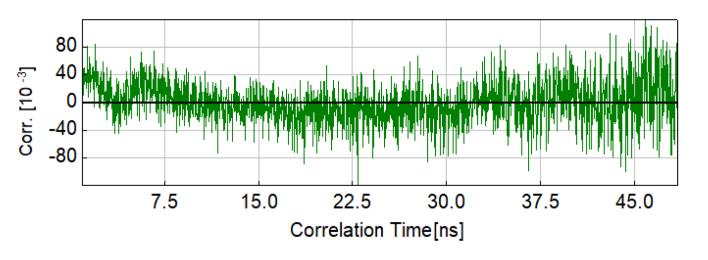

Fitted Parameters

Parameter	Value	Δ	δ
A_1[kCnts/Chnl]	3.463	±0.047	1.4%
$τ_1$[ns]	2.505	±0.013	0.5%
I_1[kCnts]	347.0	±3.8	1.1%
A_2[kCnts/Chnl]	2.178	±0.011	0.5%
$τ_2$[ns]	8.0397	±0.0067	0.1%
I_2[kCnts]	700.3	±3.1	0.4%
A_3[kCnts/Chnl]	4.265	±0.060	1.4%
$τ_3$[ns]	0.5435	±0.0070	1.3%
I_3[kCnts]	92.8	±1.2	1.2%
$Bkgr_{Dec}$[kCnts]	0.0251	±0.0003	1.1%
$Bkgr_{IRF}$[Cnts/Chnl]	0.49	±0.16	33%
$Shift_{IRF}$[ps]	8.92	±0.30	3.3%
A_{Scatt}[kCnts]	47.23	±0.75	1.6%
$τ_{AvInt}$[ns]	5.746	±0.015	0.3%
$τ_{AvAmp}$[ns]	2.8773	±0.0048	0.2%
$λ_{Det}$[nm]	580.0000	---	---

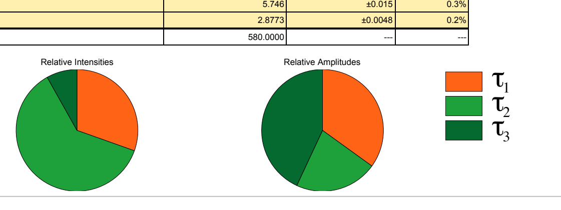

Data Set: 20 / 31

Decay: crv[20]; IRF: crv[0]

Fit

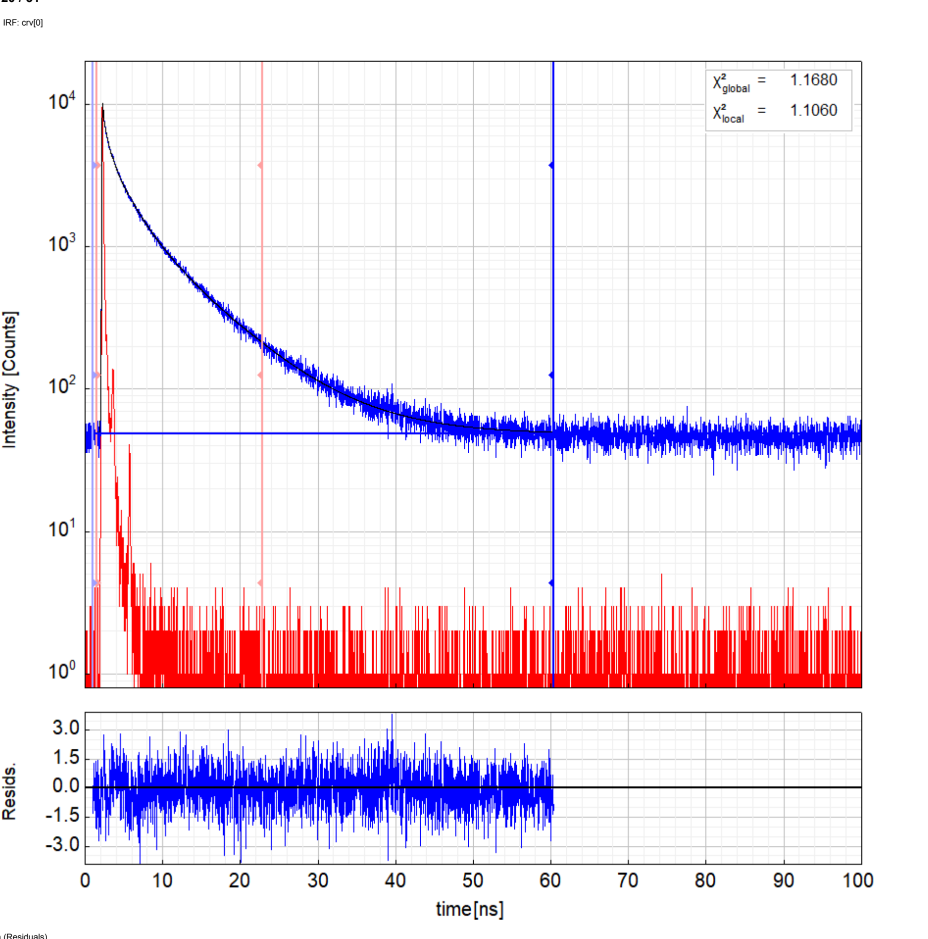

Autocorrelation (Residuals)

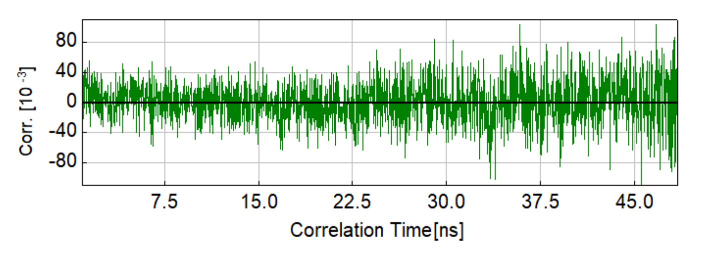

Fitted Parameters

Parameter	Value	Δ	δ
A_1[kCnts/Chnl]	3.295	±0.024	0.7%
$τ_1$[ns]	2.505	±0.013	0.5%
I_1[kCnts]	330.2	±2.6	0.8%
A_2[kCnts/Chnl]	2.057	±0.012	0.6%
$τ_2$[ns]	8.0397	±0.0067	0.1%
I_2[kCnts]	661.3	±3.7	0.5%
A_3[kCnts/Chnl]	4.394	±0.030	0.7%
$τ_3$[ns]	0.5435	±0.0070	1.3%
I_3[kCnts]	95.6	±1.2	1.2%
$Bkgr_{Dec}$[kCnts]	0.0483	±0.0004	0.7%
$Bkgr_{IRF}$[Cnts/Chnl]	0.626	±0.066	10%
$Shift_{IRF}$[ps]	10.12	±0.49	4.8%
A_{Scatt}[kCnts]	48.10	±0.33	0.7%
$τ_{AvInt}$[ns]	5.700	±0.018	0.3%
$τ_{AvAmp}$[ns]	2.7885	±0.0085	0.3%
$λ_{Det}$[nm]	590.0000	---	---

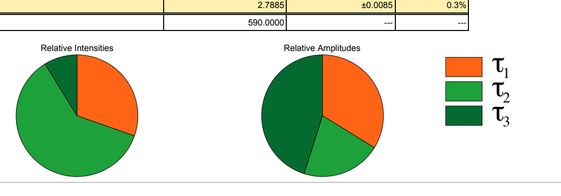

Data Set: 21 / 31

Decay: crv[21]; IRF: crv[0]

Fit

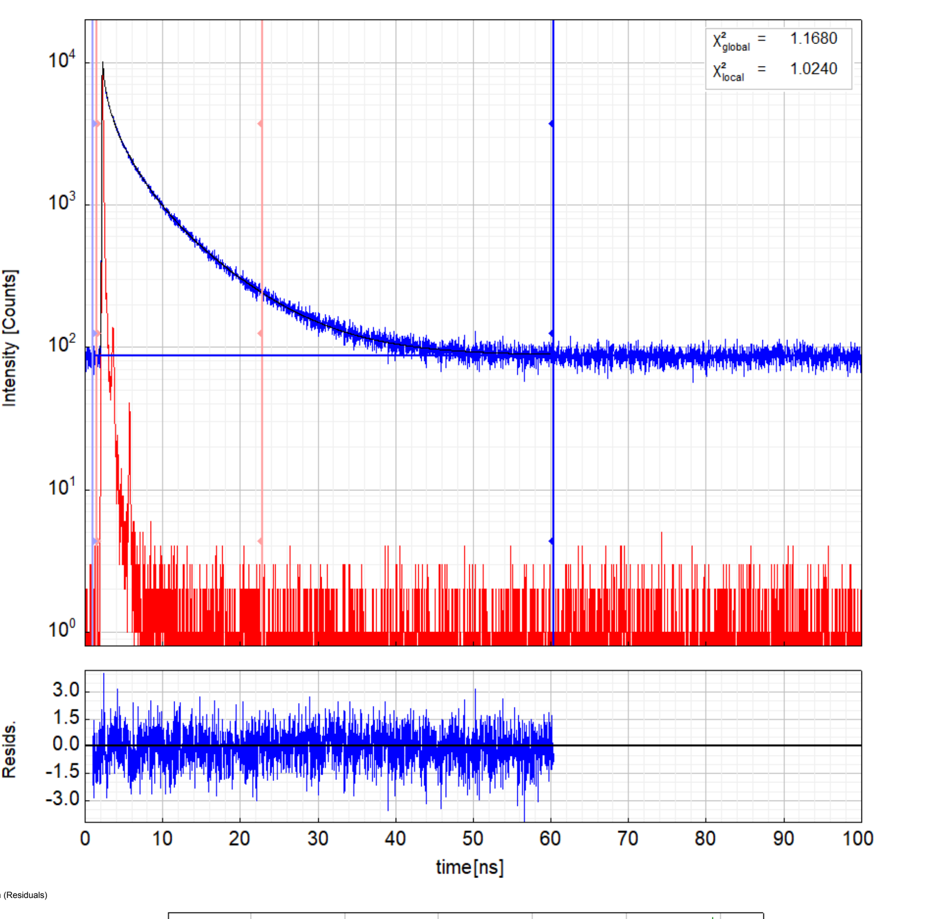

Autocorrelation (Residuals)

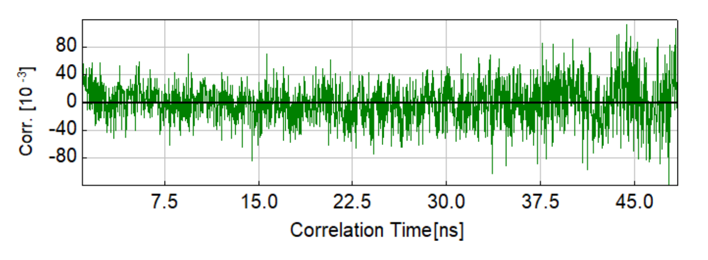

Fitted Parameters

Parameter	Value	Δ	δ
A_1[kCnts/Chnl]	3.214	±0.035	1.1%
$τ_1$[ns]	2.505	±0.013	0.5%
I_1[kCnts]	322.1	±3.3	1.0%
A_2[kCnts/Chnl]	1.945	±0.016	0.8%
$τ_2$[ns]	8.0397	±0.0067	0.1%
I_2[kCnts]	625.3	±5.0	0.8%
A_3[kCnts/Chnl]	4.40	±0.11	2.3%
$τ_3$[ns]	0.5435	±0.0070	1.3%
I_3[kCnts]	95.5	±3.4	3.5%
$Bkgr_{Dec}$[kCnts]	0.0878	±0.0003	0.3%
$Bkgr_{IRF}$[Cnts/Chnl]	0.76	±0.19	25%
$Shift_{IRF}$[ps]	10.93	±0.86	7.9%
A_{Scatt}[kCnts]	49.16	±0.52	1.0%
$τ_{AvInt}$[ns]	5.645	±0.013	0.2%
$τ_{AvAmp}$[ns]	2.730	±0.023	0.8%
$λ_{Det}$[nm]	600.0000	---	---

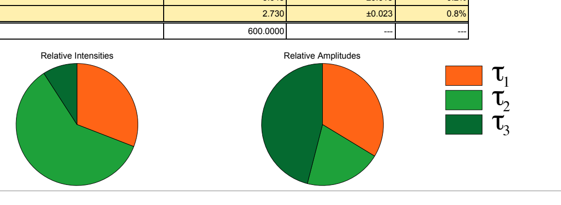

Data Set: 22 / 31

Decay: crv[22]; IRF: crv[0]

Fit

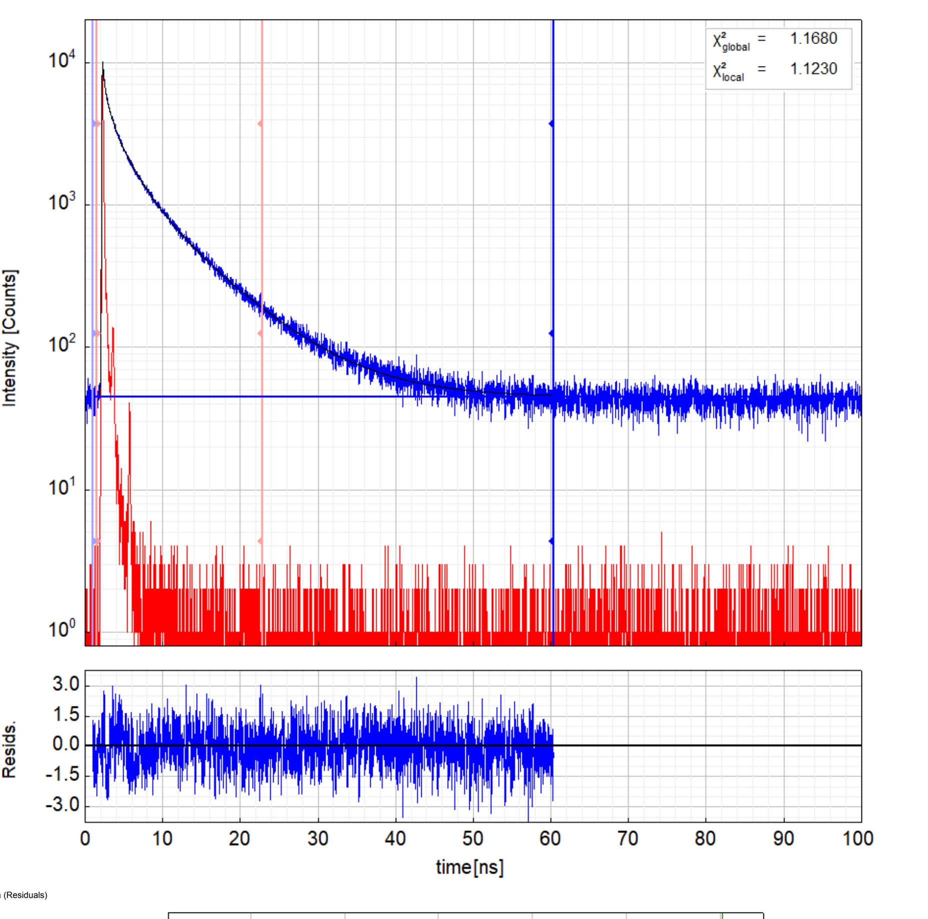

Autocorrelation (Residuals)

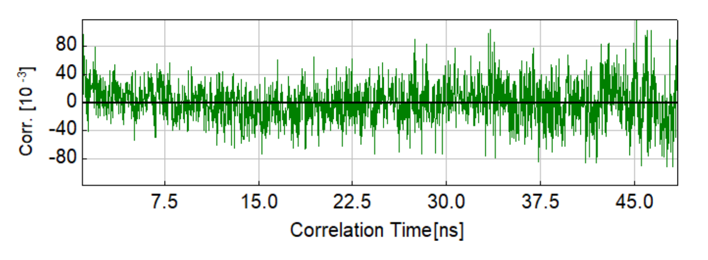

Fitted Parameters

Parameter	Value	Δ	δ
A_1[kCnts/Chnl]	3.108	±0.024	0.8%
$τ_1$[ns]	2.505	±0.013	0.5%
I_1[kCnts]	311.5	±1.7	0.5%
A_2[kCnts/Chnl]	1.8431	±0.0084	0.5%
$τ_2$[ns]	8.0397	±0.0067	0.1%
I_2[kCnts]	592.8	±2.5	0.4%
A_3[kCnts/Chnl]	4.598	±0.063	1.4%
$τ_3$[ns]	0.5435	±0.0070	1.3%
I_3[kCnts]	100.0	±1.5	1.5%
$Bkgr_{Dec}$[kCnts]	0.0446	±0.0004	0.9%
$Bkgr_{IRF}$[Cnts/Chnl]	0.79	±0.11	13%
$Shift_{IRF}$[ps]	10.74	±0.38	3.4%
A_{Scatt}[kCnts]	50.09	±0.48	0.9%
$τ_{AvInt}$[ns]	5.5769	±0.0066	0.1%
$τ_{AvAmp}$[ns]	2.629	±0.017	0.6%
$λ_{Det}$[nm]	610.0000	---	---

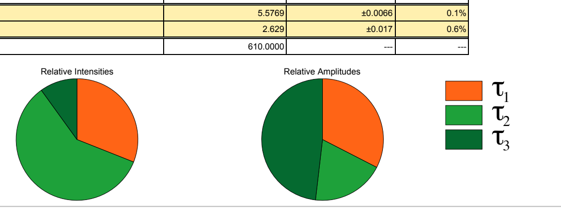

Data Set: 23 / 31

Decay: crv[23]; IRF: crv[0]

Fit

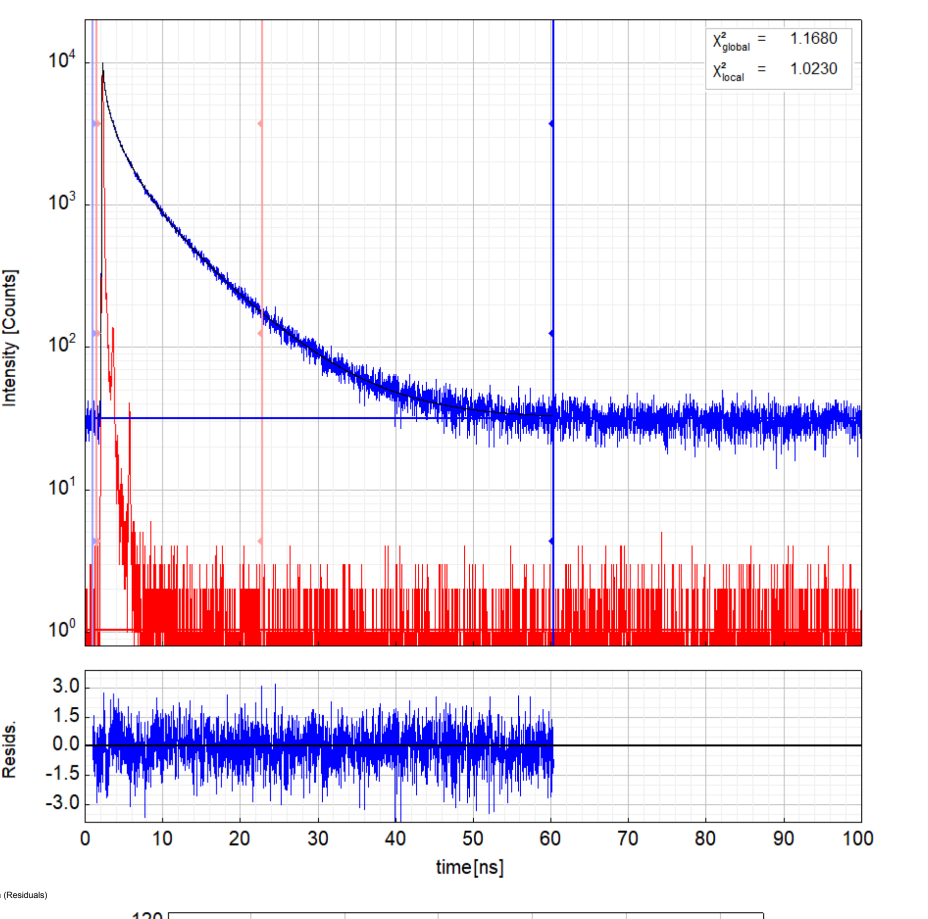

Autocorrelation (Residuals)

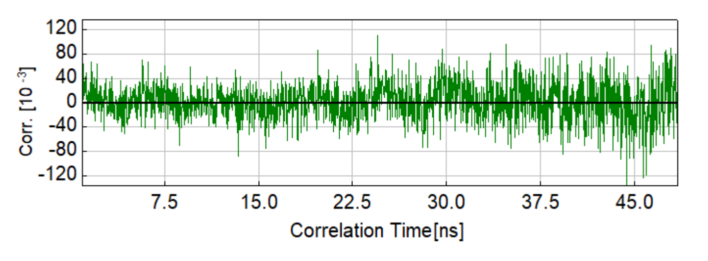

Fitted Parameters

Parameter	Value	Δ	δ
A_1[kCnts/Chnl]	2.908	±0.033	1.1%
$τ_1$[ns]	2.505	±0.013	0.5%
I_1[kCnts]	291.4	±1.6	0.5%
A_2[kCnts/Chnl]	1.8478	±0.0075	0.4%
$τ_2$[ns]	8.0397	±0.0067	0.1%
I_2[kCnts]	594.2	±2.1	0.3%
A_3[kCnts/Chnl]	4.470	±0.046	1.0%
$τ_3$[ns]	0.5435	±0.0070	1.3%
I_3[kCnts]	97.2	±1.7	1.7%
$Bkgr_{Dec}$[kCnts]	0.0316	±0.0004	1.0%
$Bkgr_{IRF}$[Cnts/Chnl]	1.04	±0.15	14%
$Shift_{IRF}$[ps]	12.85	±0.43	3.3%

A_{Scatt}[kCnts]	50.30	±0.24	0.5%
τ_{AvInt}[ns]	5.6577	±0.0100	0.2%
τ_{AvAmp}[ns]	2.664	±0.012	0.4%
λ_{Det}[nm]	620.0000	---	---

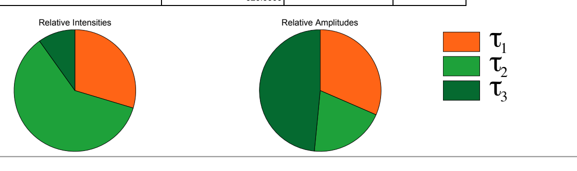

Data Set: 24 / 31

Decay: crv[24]; IRF: crv[0]

Fit

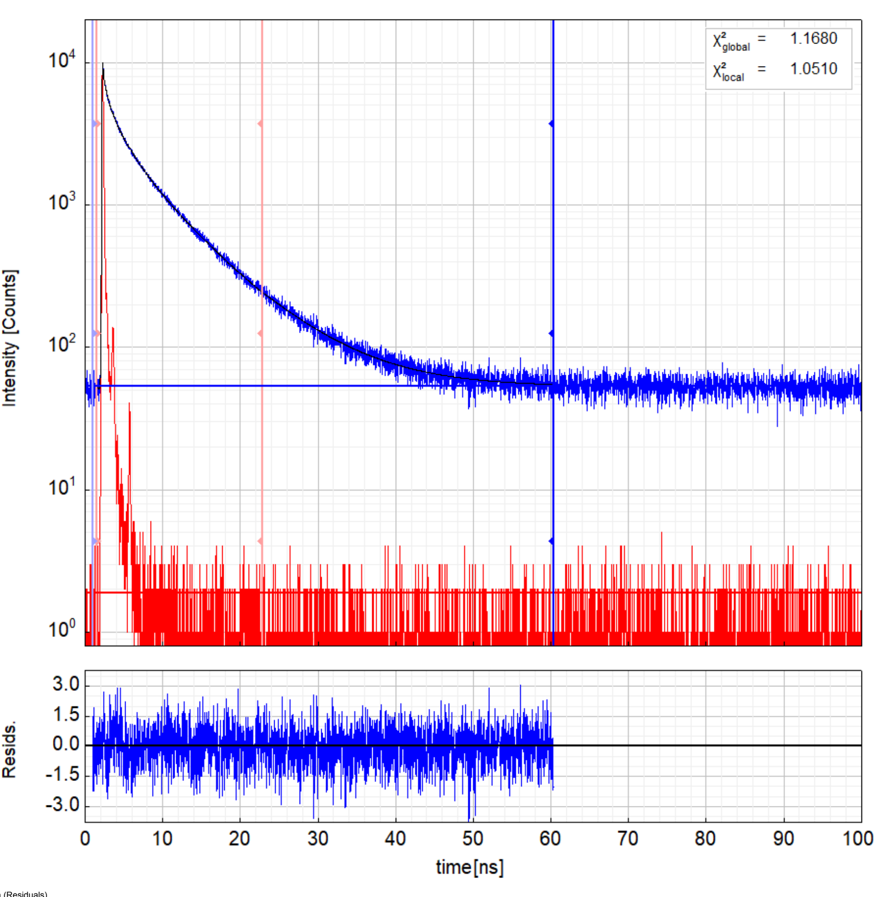

Autocorrelation (Residuals)

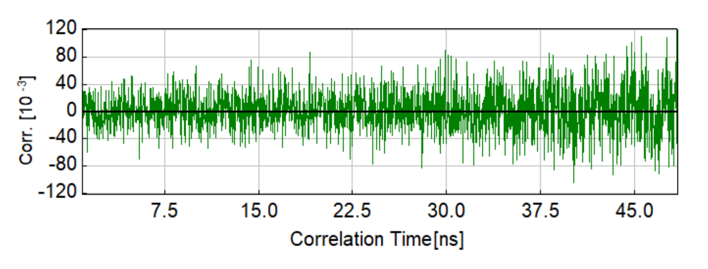

Fitted Parameters

Parameter	Value	Δ	δ
A_1[kCnts/Chnl]	2.943	±0.021	0.7%
τ_1[ns]	2.505	±0.013	0.5%
I_1[kCnts]	294.8	±2.8	0.9%
A_2[kCnts/Chnl]	2.610	±0.011	0.4%
τ_2[ns]	8.0397	±0.0067	0.1%
I_2[kCnts]	839.2	±3.6	0.4%
A_3[kCnts/Chnl]	4.011	±0.087	2.2%
τ_3[ns]	0.5435	±0.0070	1.3%
I_3[kCnts]	87.2	±1.6	1.8%
$Bkgr_{Dec}$[kCnts]	0.0531	±0.0002	0.3%
$Bkgr_{IRF}$[Cnts/Chnl]	1.90	±0.14	6.9%
$Shift_{IRF}$[ps]	12.89	±0.70	5.4%
A_{Scatt}[kCnts]	47.00	±0.53	1.1%
τ_{AvInt}[ns]	6.169	±0.013	0.2%
τ_{AvAmp}[ns]	3.193	±0.020	0.6%
λ_{Det}[nm]	630.0000	---	---

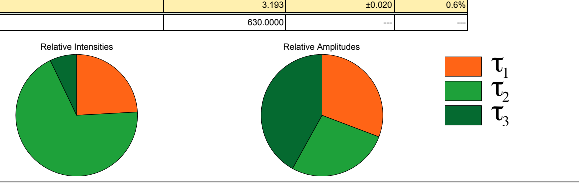

Data Set: 25 / 31

Decay: crv[25]; IRF: crv[0]

Fit

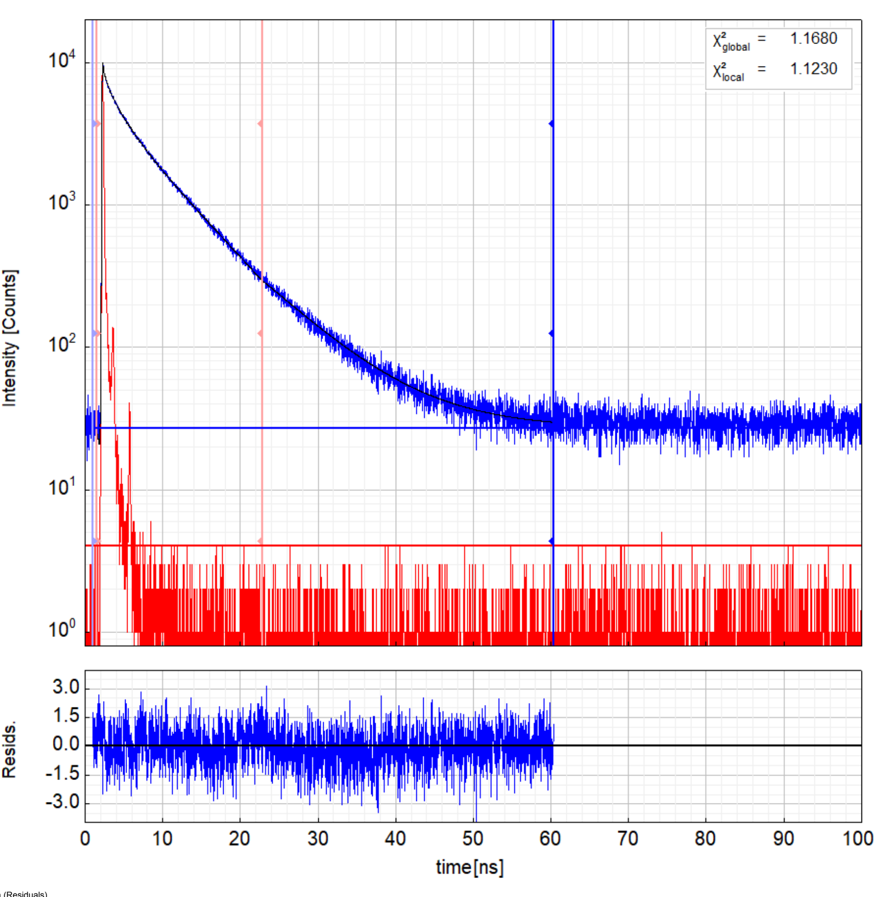

Autocorrelation (Residuals)

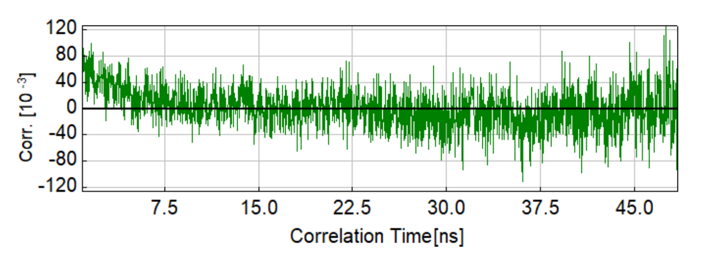

Fitted Parameters

Parameter	Value	Δ	δ
A_1[kCnts/Chnl]	3.271	±0.030	0.9%
τ_1[ns]	2.505	±0.013	0.5%
I_1[kCnts]	327.7	±2.2	0.6%
A_2[kCnts/Chnl]	4.1844	±0.0072	0.2%
τ_2[ns]	8.0397	±0.0067	0.1%
I_2[kCnts]	1 345.7	±5.5	0.4%
A_3[kCnts/Chnl]	2.969	±0.073	2.5%
τ_3[ns]	0.5435	±0.0070	1.3%
I_3[kCnts]	64.6	±1.4	2.1%
$Bkgr_{Dec}$[kCnts]	0.0272	±0.0002	0.5%
$Bkgr_{IRF}$[Cnts/Chnl]	4.01	±0.16	3.8%
$Shift_{IRF}$[ps]	12.82	±0.62	4.8%
A_{Scatt}[kCnts]	41.93	±0.95	2.3%
τ_{AvInt}[ns]	6.718	±0.013	0.2%
τ_{AvAmp}[ns]	4.169	±0.031	0.7%
λ_{Det}[nm]	640.0000	---	---

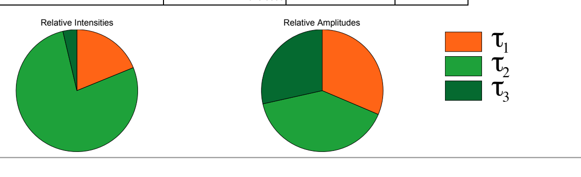

Data Set: 26 / 31

Decay: crv[26]; IRF: crv[0]

Fit

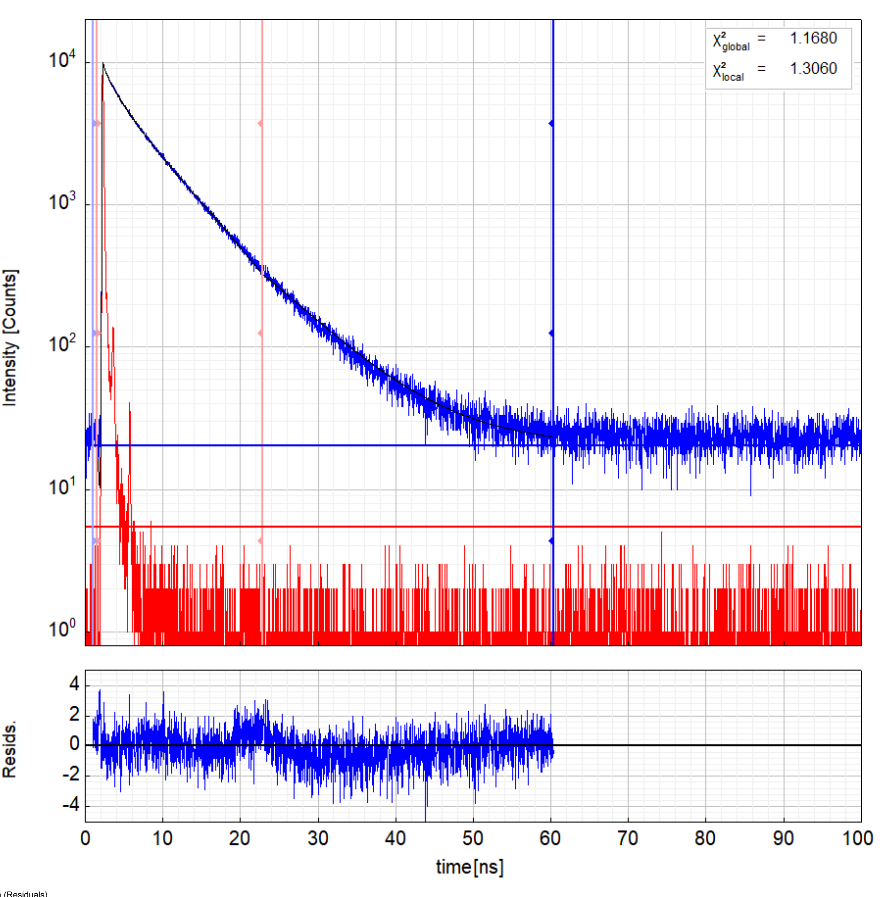

Autocorrelation (Residuals)

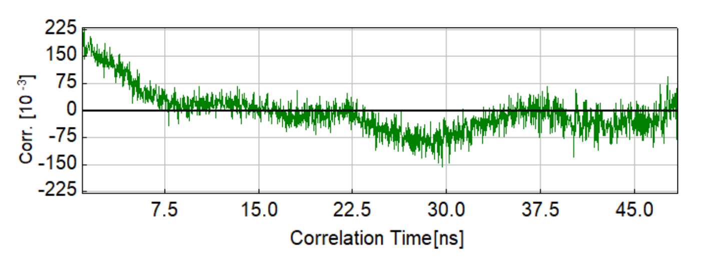

Fitted Parameters

Parameter	Value	Δ	δ
A_1[kCnts/Chnl]	3.690	±0.037	1.0%
τ_1[ns]	2.505	±0.013	0.5%
I_1[kCnts]	369.8	±4.6	1.2%
A_2[kCnts/Chnl]	5.290	±0.015	0.3%
τ_2[ns]	8.0397	±0.0067	0.1%
I_2[kCnts]	1 701.2	±4.9	0.3%
A_3[kCnts/Chnl]	1.913	±0.068	3.5%
τ_3[ns]	0.5435	±0.0070	1.3%
I_3[kCnts]	41.6	±1.2	2.8%
$Bkgr_{Dec}$[kCnts]	0.0204	±0.0003	1.5%
$Bkgr_{IRF}$[Cnts/Chnl]	5.47	±0.11	1.9%
$Shift_{IRF}$[ps]	14.50	±0.63	4.3%
A_{Scatt}[kCnts]	38.75	±0.74	1.9%
τ_{AvInt}[ns]	6.9234	±0.0085	0.1%
τ_{AvAmp}[ns]	4.849	±0.017	0.3%
λ_{Det}[nm]	650.0000	---	---

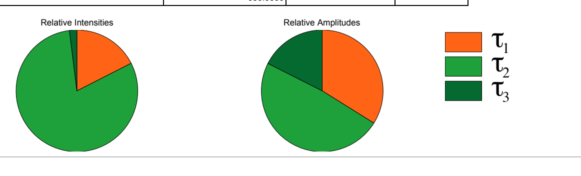

Data Set: 27 / 31

Decay: crv[27]; IRF: crv[0]

Fit

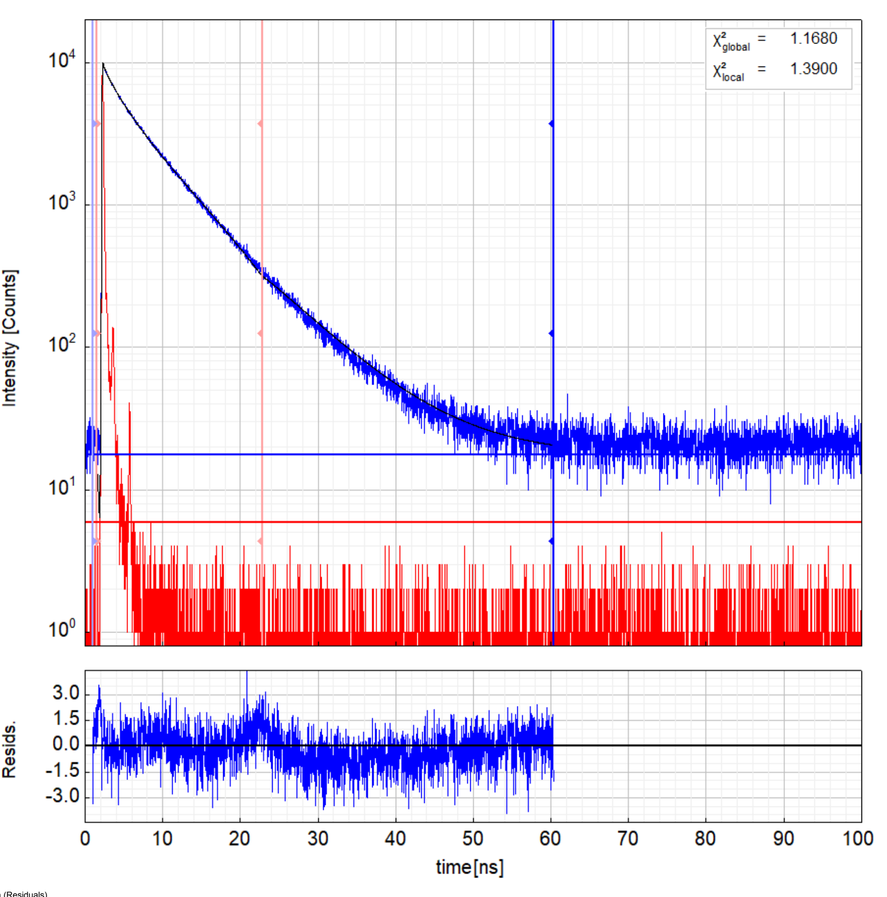

Autocorrelation (Residuals)

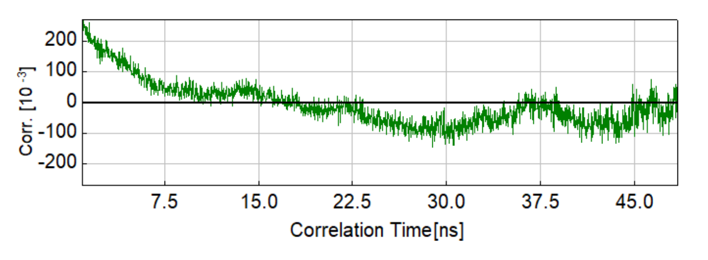

Fitted Parameters

Parameter	Value	Δ	δ
A_1[kCnts/Chnl]	3.921	±0.023	0.6%
τ_1[ns]	2.505	±0.013	0.5%
I_1[kCnts]	392.8	±2.9	0.7%
A_2[kCnts/Chnl]	5.373	±0.015	0.3%
τ_2[ns]	8.0397	±0.0067	0.1%
I_2[kCnts]	1 727.9	±6.1	0.4%
A_3[kCnts/Chnl]	1.753	±0.084	4.8%
τ_3[ns]	0.5435	±0.0070	1.3%
I_3[kCnts]	38.2	±1.3	3.3%
$Bkgr_{Dec}$[kCnts]	0.0176	±0.0004	1.8%
$Bkgr_{IRF}$[Cnts/Chnl]	5.906	±0.094	1.6%
$Shift_{IRF}$[ps]	14.40	±0.79	5.5%
A_{Scatt}[kCnts]	36.94	±0.42	1.1%
τ_{AvInt}[ns]	6.9003	±0.0077	0.1%
τ_{AvAmp}[ns]	4.886	±0.027	0.5%
λ_{Det}[nm]	660.0000	---	---

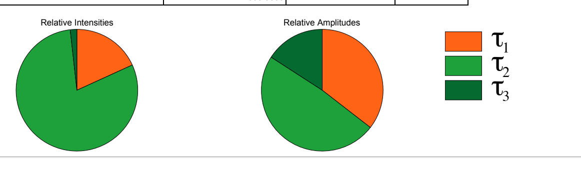

Data Set: 28 / 31

Decay: crv[28]; IRF: crv[0]

Fit

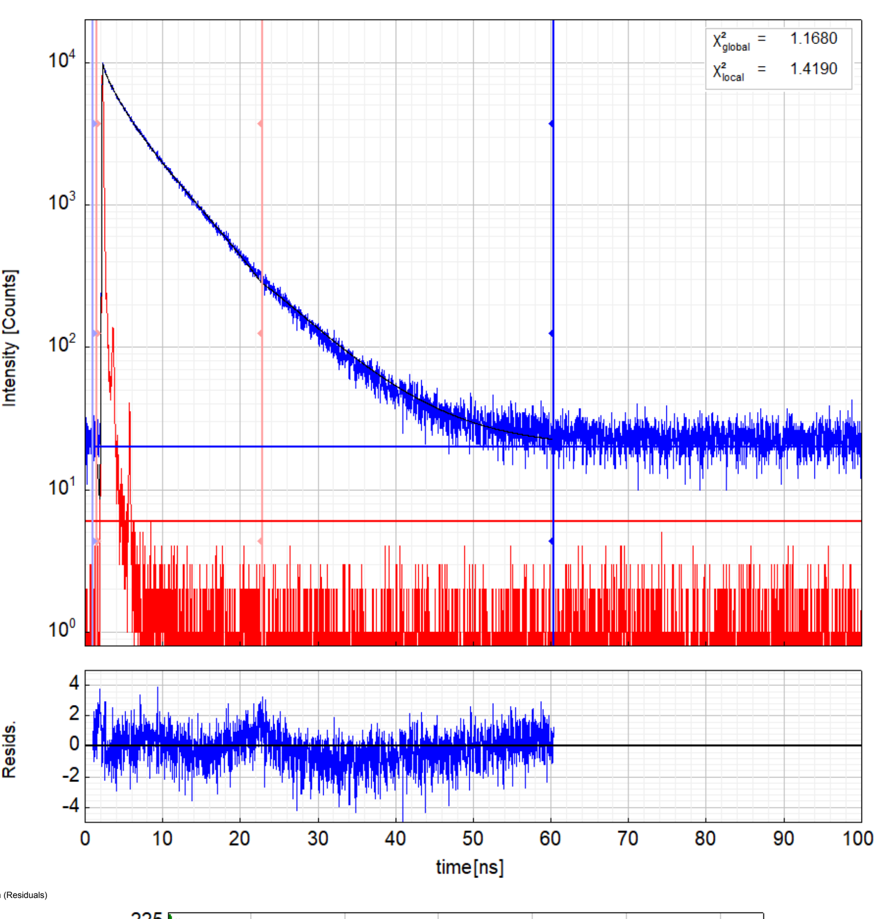

Autocorrelation (Residuals)

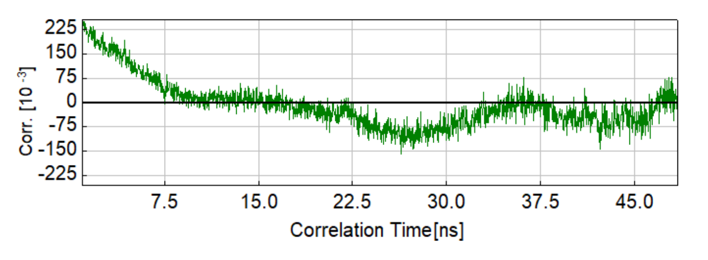

Fitted Parameters

Parameter	Value	Δ	δ
A_1[kCnts/Chnl]	4.064	±0.032	0.8%
τ_1[ns]	2.505	±0.013	0.5%
I_1[kCnts]	407.2	±3.0	0.7%
A_2[kCnts/Chnl]	4.818	±0.018	0.4%
τ_2[ns]	8.0397	±0.0067	0.1%
I_2[kCnts]	1 549.3	±5.6	0.4%
A_3[kCnts/Chnl]	1.887	±0.060	3.2%
τ_3[ns]	0.5435	±0.0070	1.3%
I_3[kCnts]	41.1	±1.9	4.5%
$Bkgr_{Dec}$[kCnts]	0.0200	±0.0004	1.9%
$Bkgr_{IRF}$[Cnts/Chnl]	6.031	±0.086	1.4%
$Shift_{IRF}$[ps]	14.42	±0.62	4.2%
A_{Scatt}[kCnts]	38.12	±0.45	1.2%
τ_{AvInt}[ns]	6.7576	±0.0087	0.1%
τ_{AvAmp}[ns]	4.638	±0.017	0.4%
λ_{Det}[nm]	670.0000	---	---

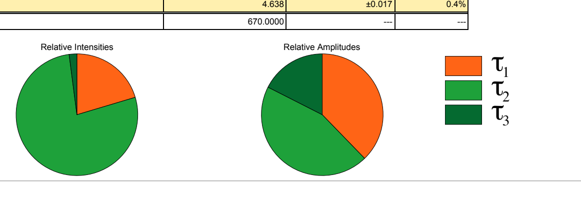

Data Set: 29 / 31

Decay: crv[29]; IRF: crv[0]

Fit

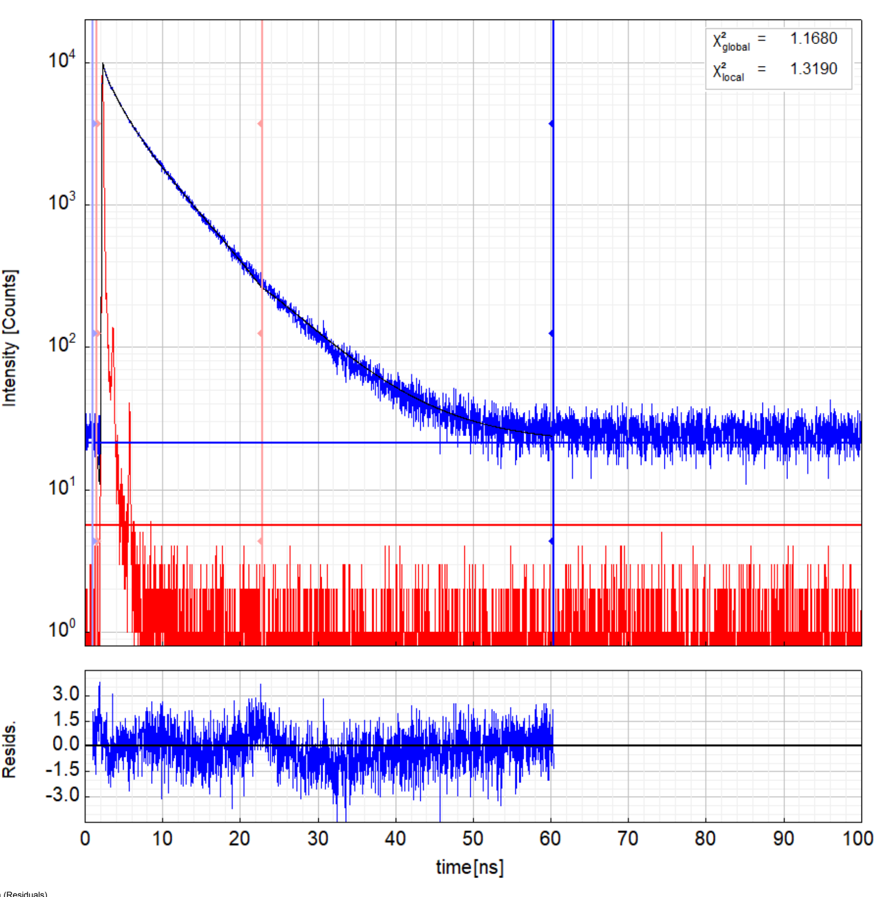

Autocorrelation (Residuals)

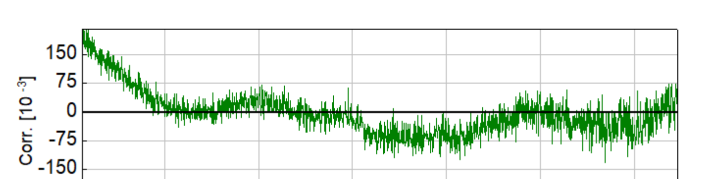

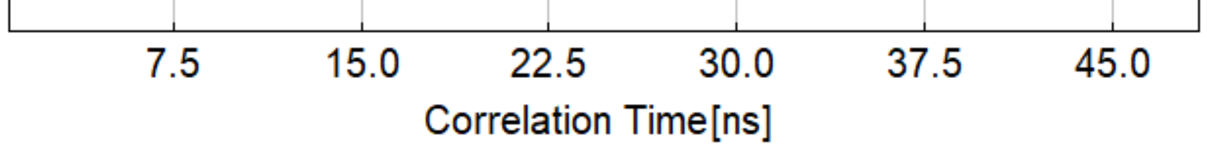

Fitted Parameters

Parameter	Value	Δ	δ
A_1[kCnts/Chnl]	4.403	±0.033	0.7%
$τ_1$[ns]	2.505	±0.013	0.5%
I_1[kCnts]	441.2	±3.9	0.9%
A_2[kCnts/Chnl]	4.351	±0.021	0.5%
$τ_2$[ns]	8.0397	±0.0067	0.1%
I_2[kCnts]	1 399.2	±6.6	0.5%
A_3[kCnts/Chnl]	2.077	±0.057	2.7%
$τ_3$[ns]	0.5435	±0.0070	1.3%
I_3[kCnts]	45.2	±1.3	2.8%
$Bkgr_{Dec}$[kCnts]	0.0214	±0.0003	1.1%
$Bkgr_{IRF}$[Cnts/Chnl]	5.65	±0.17	2.9%
$Shift_{IRF}$[ps]	15.18	±0.43	2.8%
A_{Scatt}[kCnts]	39.47	±0.46	1.1%
$τ_{AvInt}$[ns]	6.566	±0.016	0.2%
$τ_{AvAmp}$[ns]	4.353	±0.012	0.3%
$λ_{Det}$[nm]	680.0000	---	---

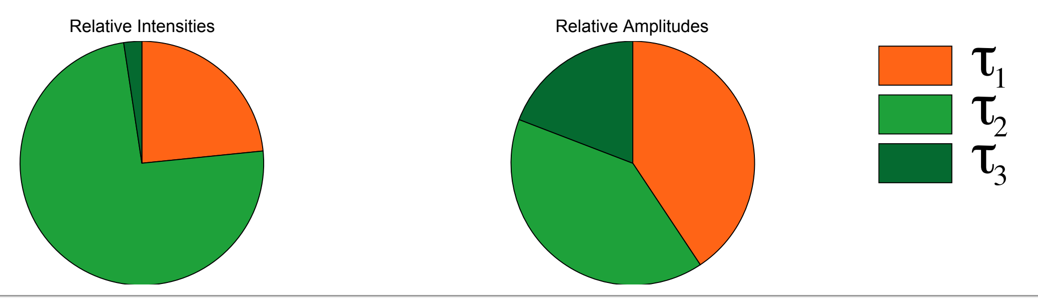

Data Set: 30 / 31

Decay: crv[30]; IRF: crv[0]

Fit

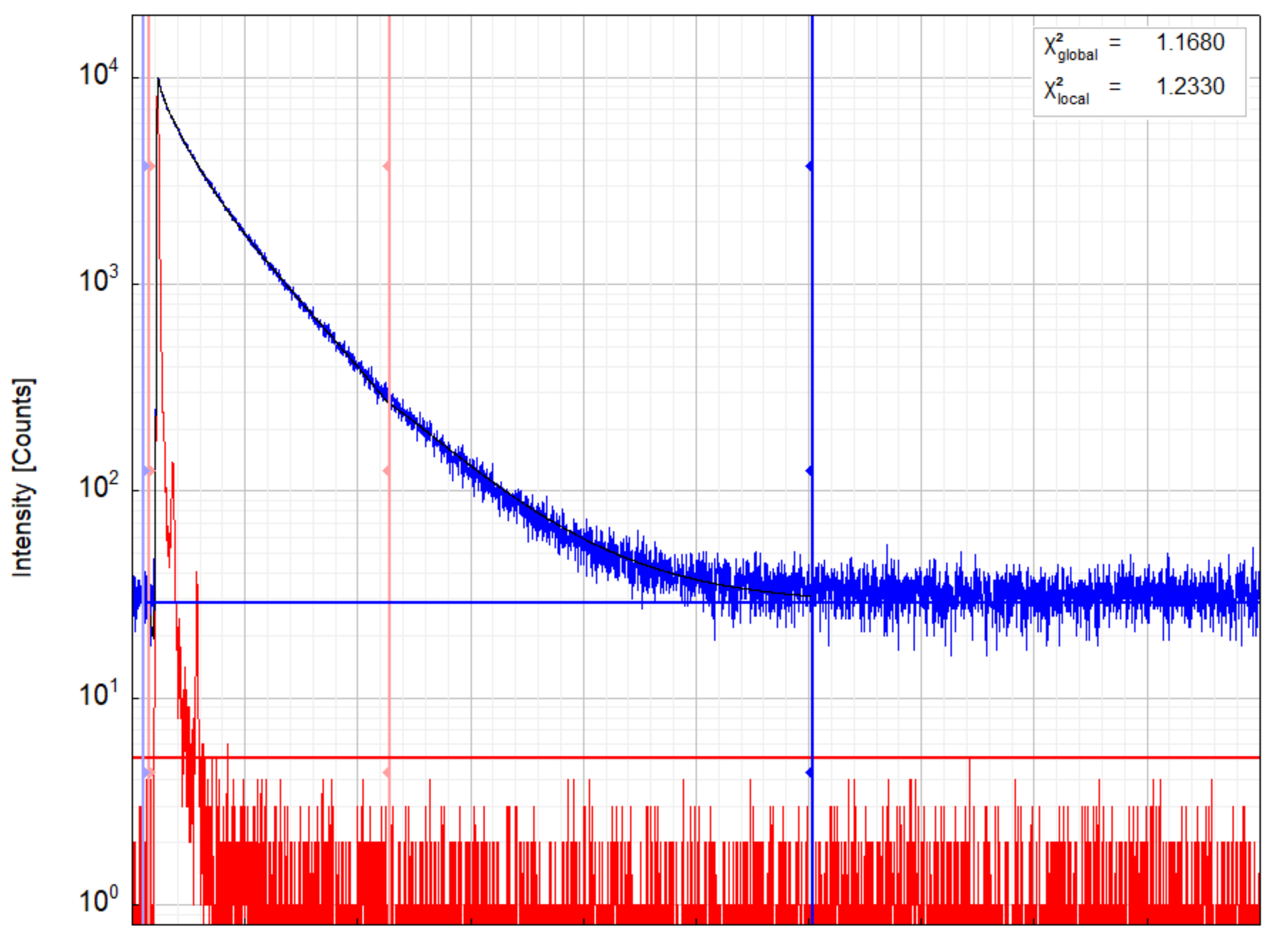

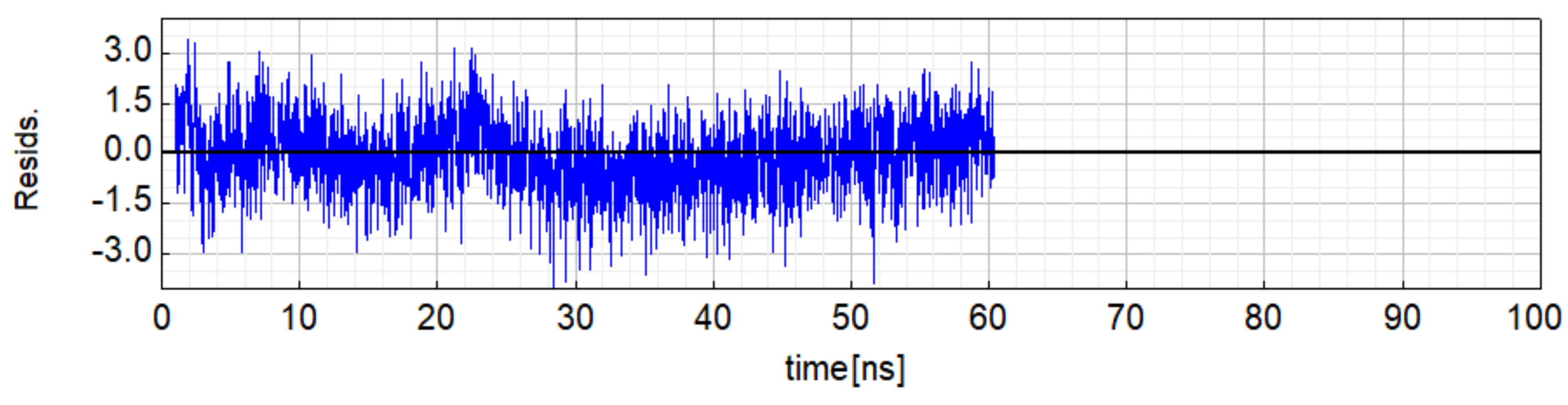

Autocorrelation (Residuals)

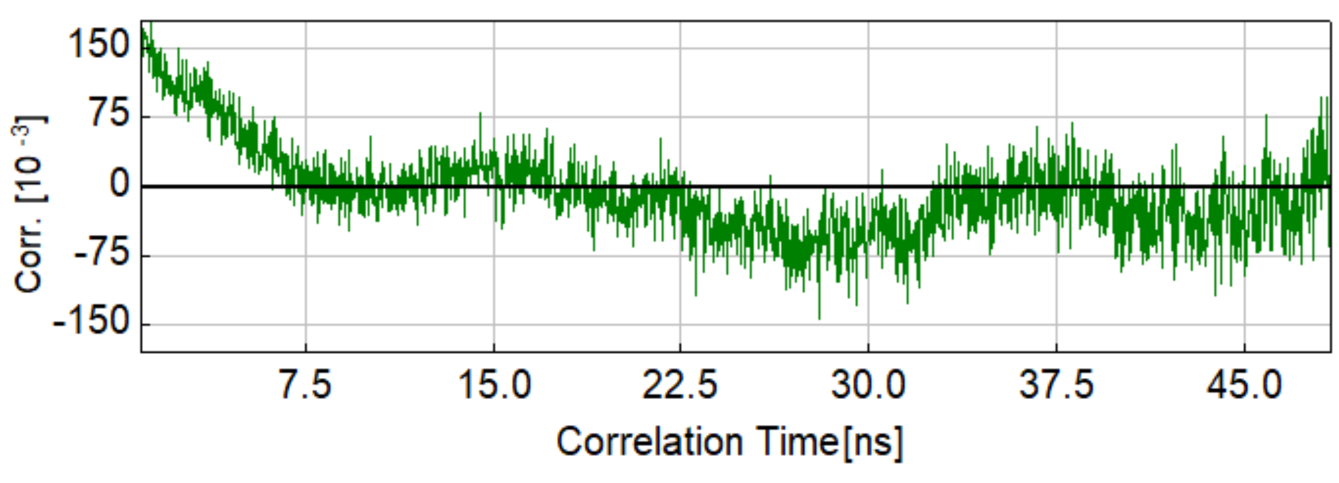

Fitted Parameters

Parameter	Value	Δ	δ
A_1[kCnts/Chnl]	4.577	±0.029	0.6%
$τ_1$[ns]	2.505	±0.013	0.5%
I_1[kCnts]	458.6	±3.5	0.8%
A_2[kCnts/Chnl]	4.094	±0.012	0.3%
$τ_2$[ns]	8.0397	±0.0067	0.1%
I_2[kCnts]	1 316.5	±3.9	0.3%
A_3[kCnts/Chnl]	2.151	±0.077	3.5%
$τ_3$[ns]	0.5435	±0.0070	1.3%
I_3[kCnts]	46.8	±2.1	4.3%
$Bkgr_{Dec}$[kCnts]	0.0286	±0.0003	0.8%
$Bkgr_{IRF}$[Cnts/Chnl]	5.09	±0.11	2.0%
$Shift_{IRF}$[ps]	15.65	±0.64	4.1%
A_{Scatt}[kCnts]	39.29	±0.97	2.5%
$τ_{AvInt}$[ns]	6.455	±0.014	0.2%
$τ_{AvAmp}$[ns]	4.209	±0.035	0.8%
$λ_{Det}$[nm]	690.0000	---	---

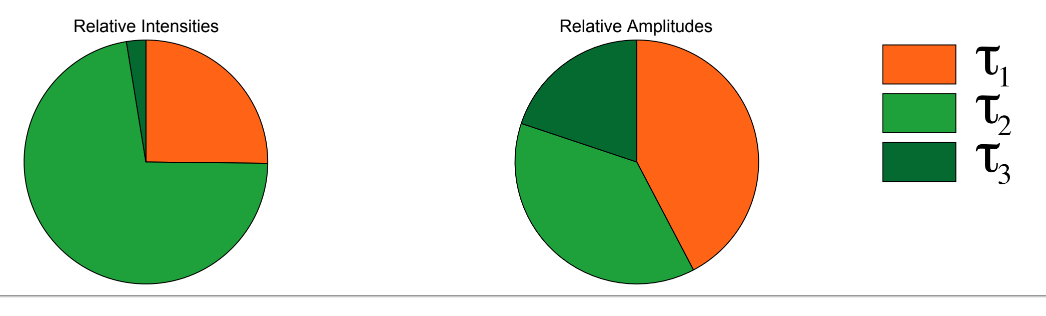

Data Set: 31 / 31

Decay: crv[31]; IRF: crv[0]

Fit

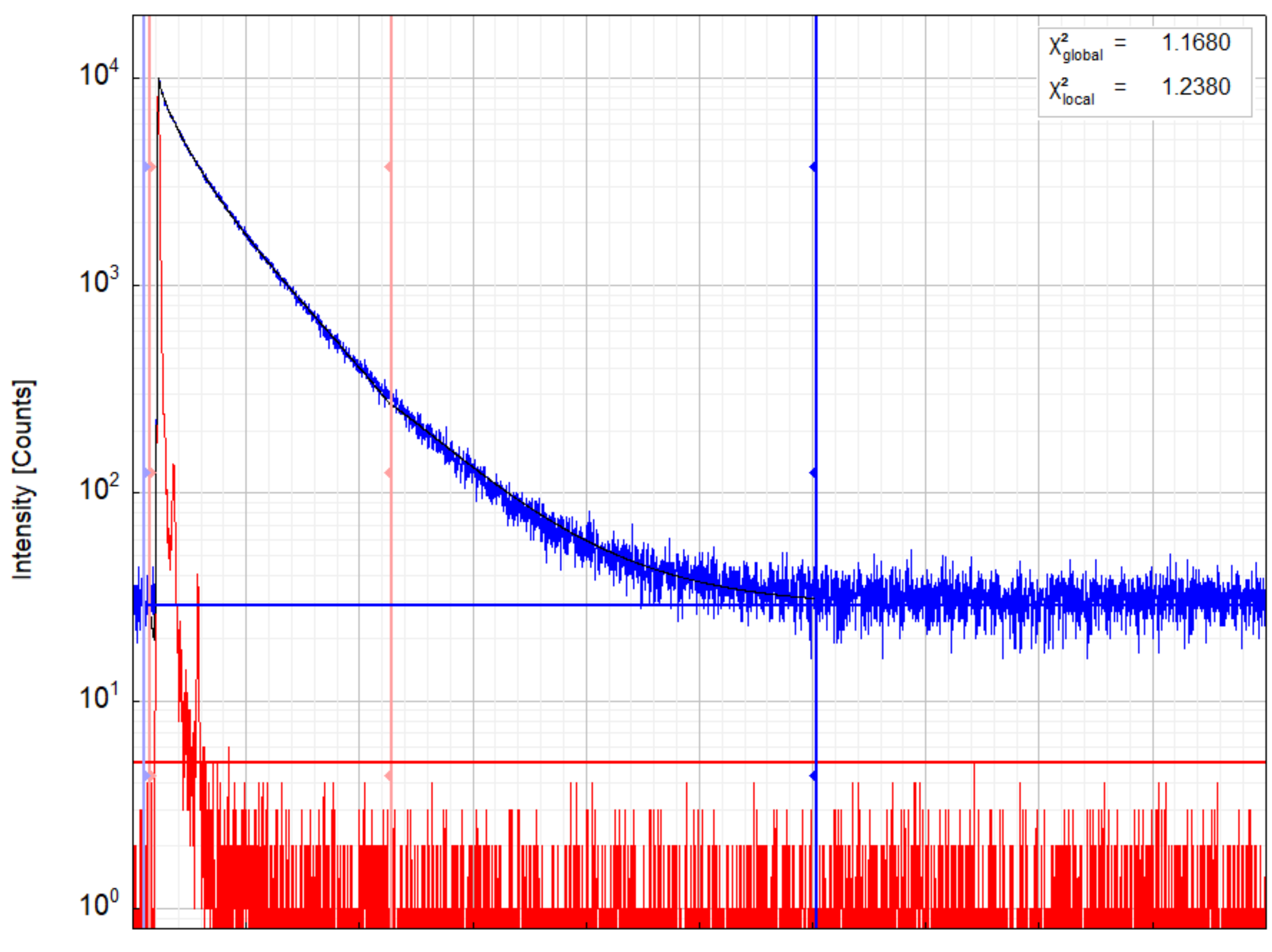

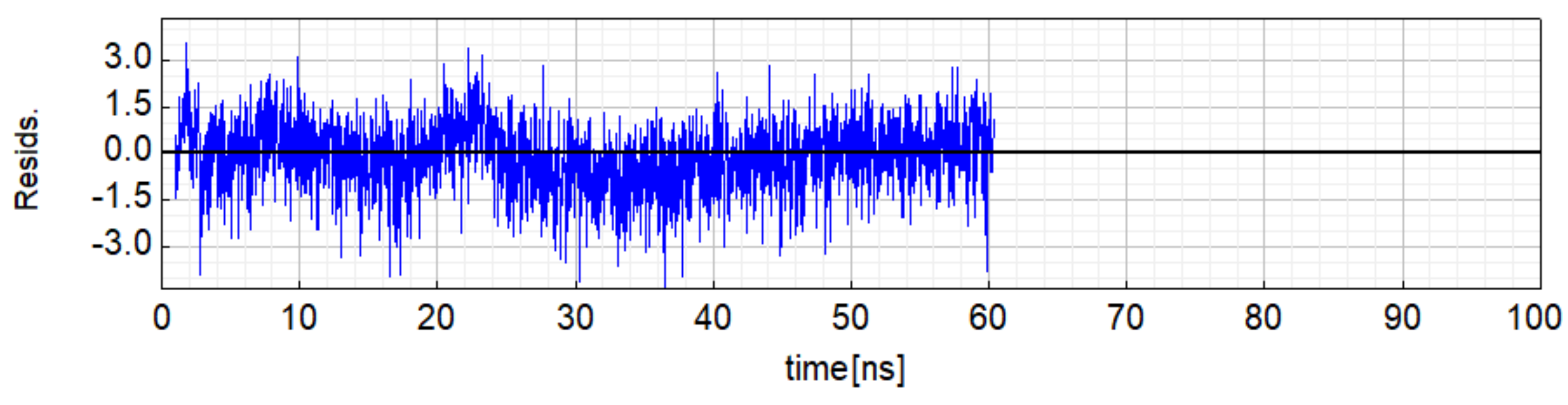

Autocorrelation (Residuals)

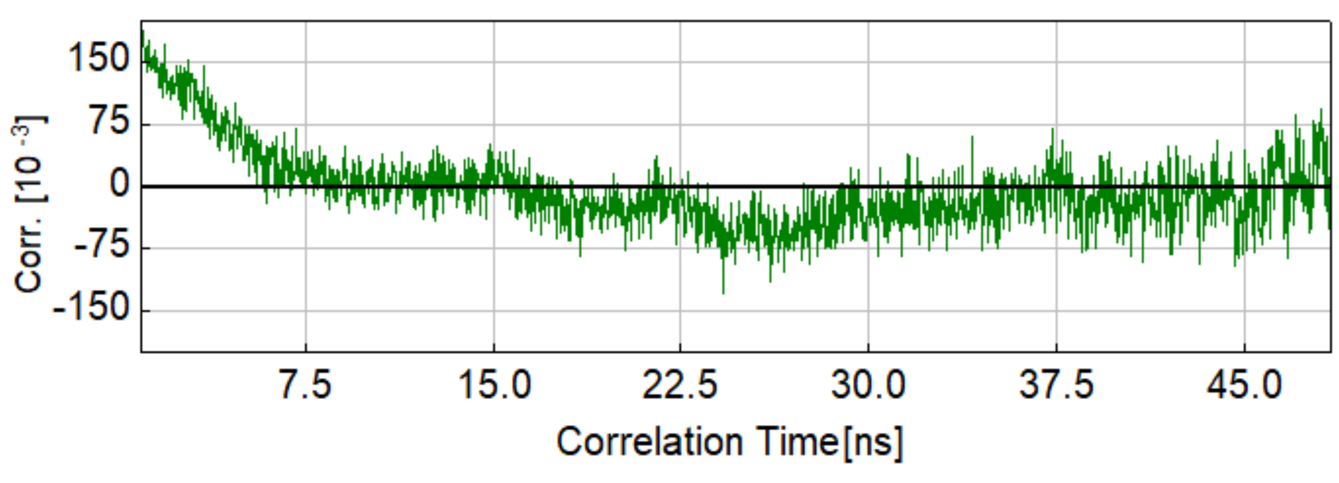

Fitted Parameters

Parameter	Value	Δ	δ
A_1[kCnts/Chnl]	4.458	±0.025	0.5%
$τ_1$[ns]	2.505	±0.013	0.5%
I_1[kCnts]	446.7	±4.2	0.9%
A_2[kCnts/Chnl]	4.106	±0.015	0.4%
$τ_2$[ns]	8.0397	±0.0067	0.1%
I_2[kCnts]	1 320.3	±4.4	0.3%
A_3[kCnts/Chnl]	2.059	±0.075	3.6%
$τ_3$[ns]	0.5435	±0.0070	1.3%
I_3[kCnts]	44.8	±1.6	3.5%
$Bkgr_{Dec}$[kCnts]	0.0287	±0.0003	0.9%
$Bkgr_{IRF}$[Cnts/Chnl]	5.03	±0.15	3.0%
$Shift_{IRF}$[ps]	17.59	±0.60	3.4%
A_{Scatt}[kCnts]	39.58	±0.76	1.9%
$τ_{AvInt}$[ns]	6.490	±0.012	0.2%
$τ_{AvAmp}$[ns]	4.264	±0.025	0.6%
$λ_{Det}$[nm]	700.0000	---	---

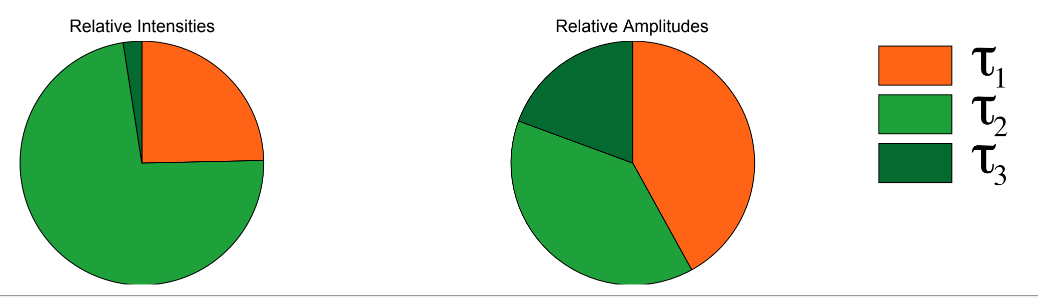

Parameter Plot

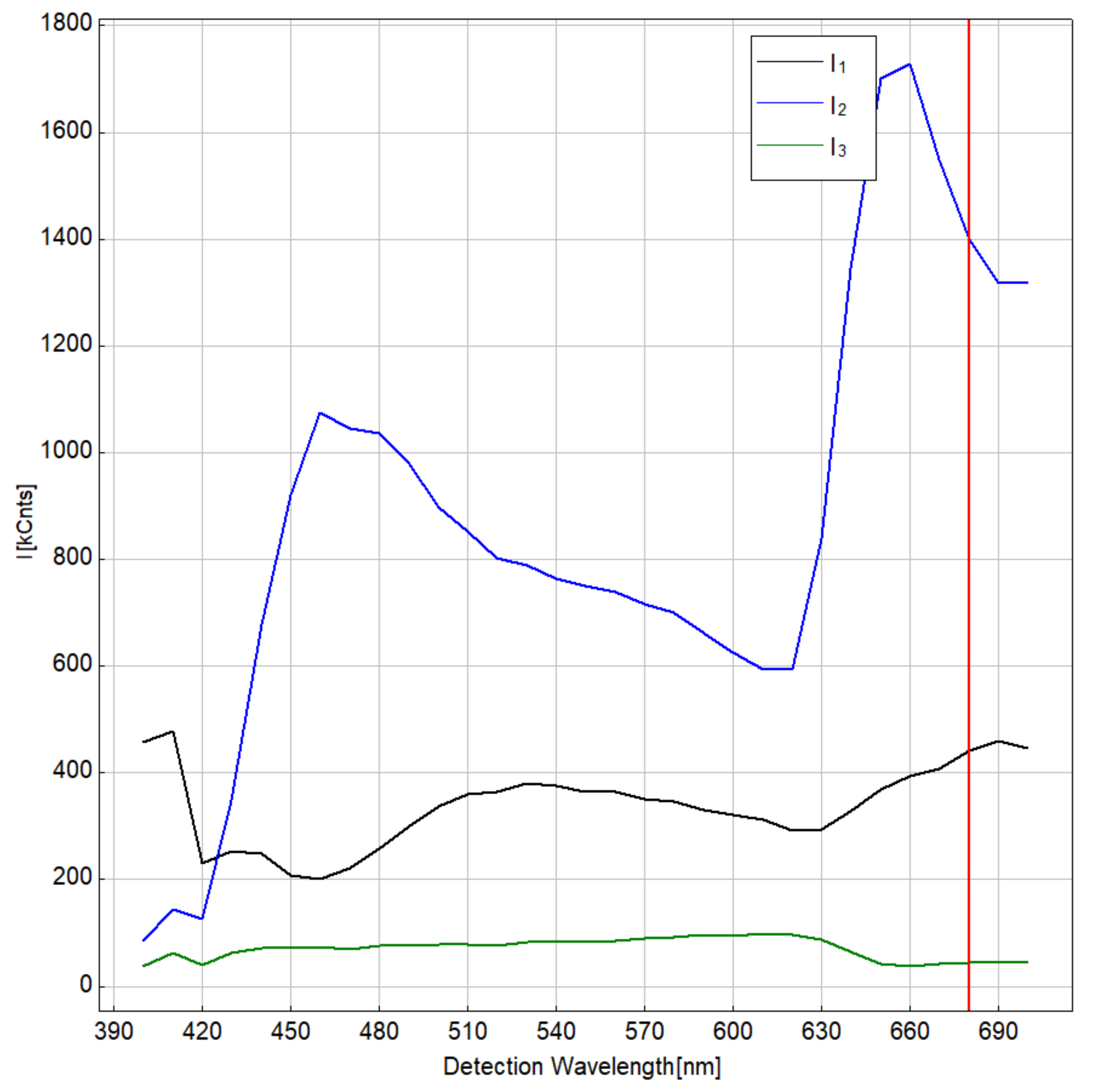

Global Parameters

Parameter	Value	Δ	δ
$τ_1$[ns]	2.505	±0.013	0.5%
$τ_2$[ns]	8.0397	±0.0067	0.1%
$τ_3$[ns]	0.5435	±0.0070	1.3%

EasyTau Report

3/11/2022 7:51:21 PM
F:\Paper Xiongfei\CD-1 THF tau global.html

Container Curves

TRES+IRF_20201222_1824.etc: crv[0 - 16]

Container Properties

Measurement Context: Time Resolved Emission Scan
Detection Wavelength: 400.0 - 700.0 (Step: 20.0) nm
Summary:

Sample:	CD_X_1
Solvent:	Tetrahydrofuran
Excitation:	V pol 376±1nm with LDH-P-C-375
Detection:	M pol 400...700nm 20nm steps<br>10000 peak counts<br>grating 1200/500-<br>detector UV-red [PMT]

Comment

Model: Exponential [Reconv.]

$$\mathrm{Dec}(t)=\left[\int_{-\infty}^{t} dt'\,[\mathrm{IRF}(t-Shift_{IRF})-Bkgr_{IRF}]\left[\sum_{i=1}^{n_{Exp}} A_i\, e^{-\frac{t-t'}{\tau_i}}+A_{Scatt}\,\delta(t-t')\right]\right]+Bkgr_{Dec}$$

$$I_m = A_m \tau_m$$

$$A_{Rel\,m}=A_m \Big/ \sum_{\substack{i=1\\A_i>0}}^{n_{Exp}} A_i \qquad I_{Rel\,m}=I_m \Big/ \sum_{\substack{i=1\\I_i>0}}^{n_{Exp}} I_i$$

$$\tau_{Av\,Amp}=\sum_{\substack{i=1\\A_i>0}}^{n_{Exp}} A_i\tau_i \Big/ \sum_{\substack{i=1\\A_i>0}}^{n_{Exp}} A_i \qquad \tau_{Av\,Int}=\sum_{\substack{i=1\\I_i>0}}^{n_{Exp}} I_i\tau_i \Big/ \sum_{\substack{i=1\\I_i>0}}^{n_{Exp}} I_i$$

Data Set: 1 / 16

Decay: crv[1]; IRF: crv[0]

Fit

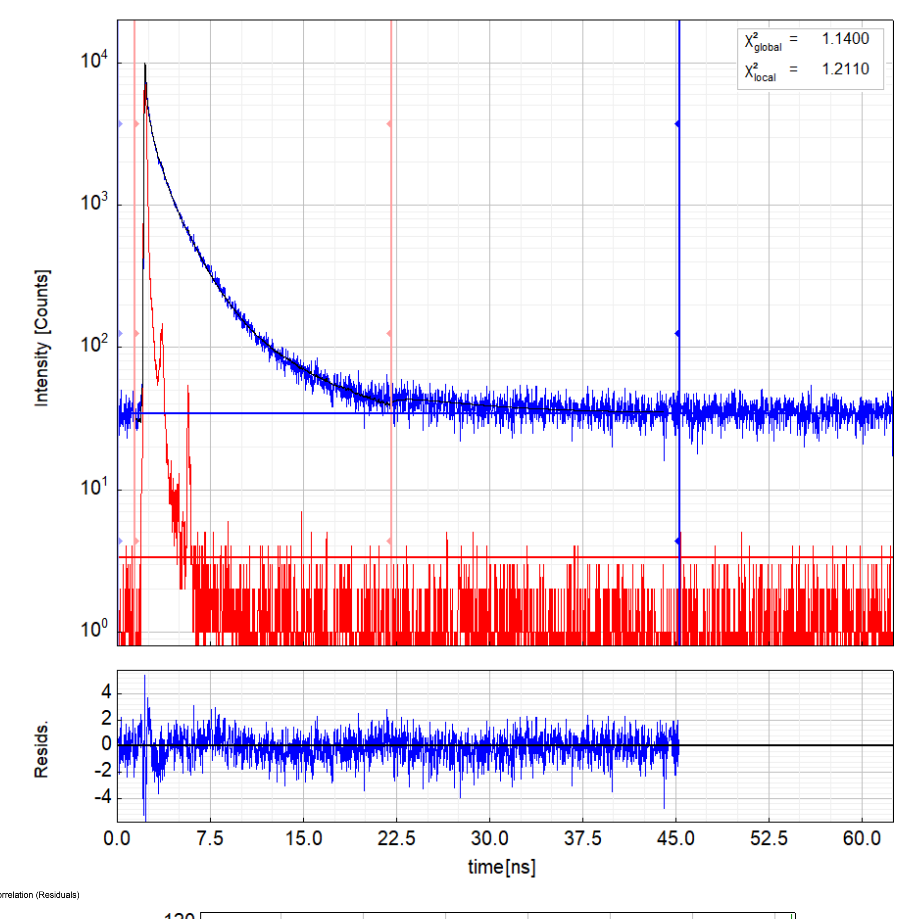

Autocorrelation (Residuals)

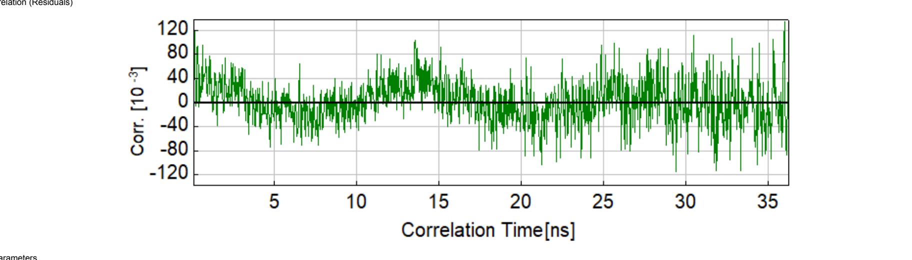

Fitted Parameters

Parameter	Value	Δ	δ
A_1[kCnts/Chnl]	0.2478	±0.0084	3.4%
τ_1[ns]	7.166	±0.030	0.4%
I_1[kCnts]	71.1	±2.1	2.9%
A_2[kCnts/Chnl]	2.521	±0.070	2.8%
τ_2[ns]	1.943	±0.028	1.4%
I_2[kCnts]	195.9	±2.1	1.1%
A_3[kCnts/Chnl]	3.30	±0.11	3.3%
τ_3[ns]	0.534	±0.025	4.6%
I_3[kCnts]	70.20	±1.00	1.4%
$Bkgr_{Dec}$[kCnts]	0.0344	±0.0002	0.4%
$Bkgr_{IRF}$[Cnts/Chnl]	3.34	±0.17	4.8%
$Shift_{IRF}$[ps]	-10.1	±1.3	13%
A_{Scatt}[kCnts]	63.9	±2.2	3.4%
τ_{AvInt}[ns]	2.750	±0.014	0.5%
τ_{AvAmp}[ns]	1.391	±0.028	2.0%
λ_{Det}[nm]	400.0000	---	---

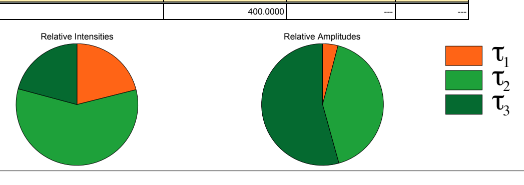

Data Set: 2 / 16

Decay: crv[2]; IRF: crv[0]

Fit

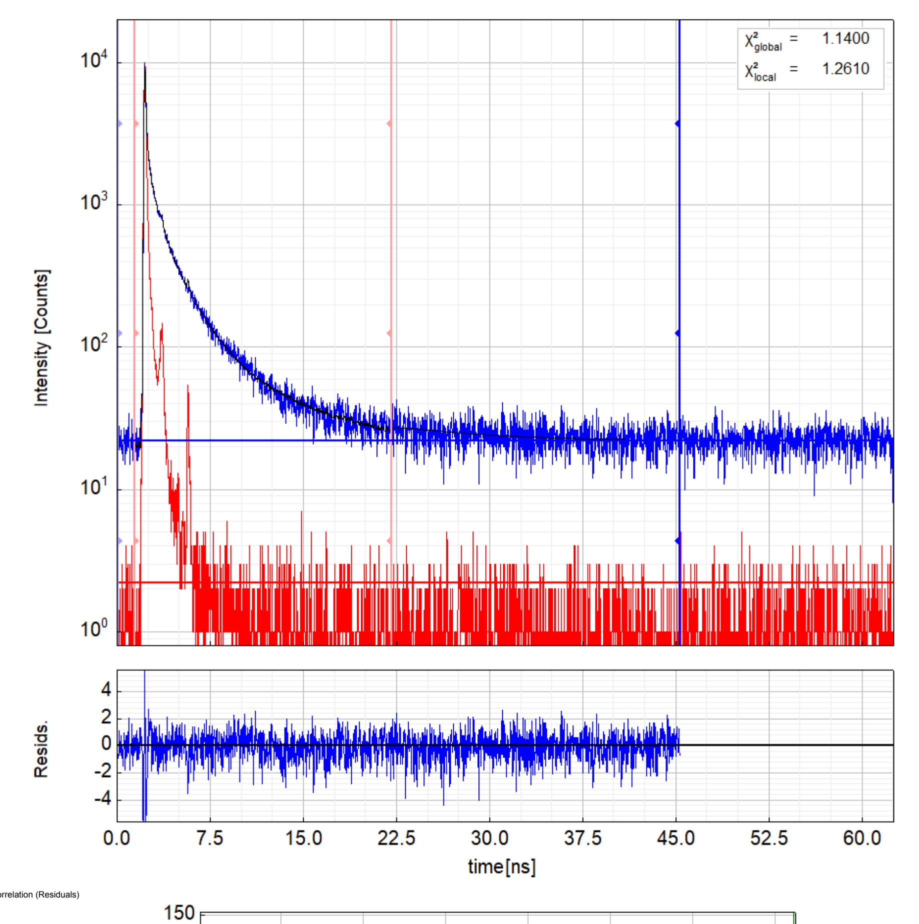

Autocorrelation (Residuals)

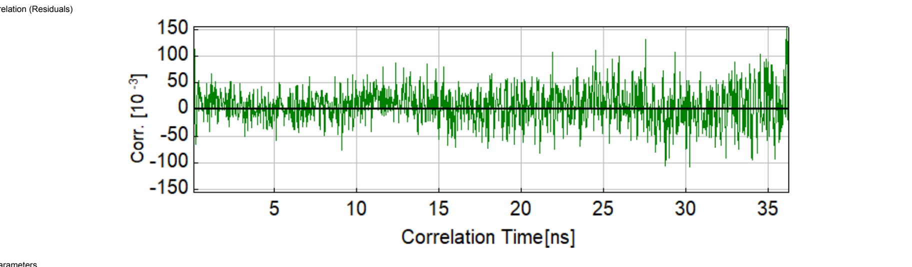

Fitted Parameters

Parameter	Value	Δ	δ
A_1[kCnts/Chnl]	0.1107	±0.0040	3.6%
τ_1[ns]	7.166	±0.030	0.4%
I_1[kCnts]	31.8	±1.1	3.5%
A_2[kCnts/Chnl]	0.927	±0.027	2.8%
τ_2[ns]	1.943	±0.028	1.4%
I_2[kCnts]	72.0	±1.2	1.6%
A_3[kCnts/Chnl]	1.577	±0.049	3.1%
τ_3[ns]	0.534	±0.025	4.6%
I_3[kCnts]	33.7	±1.1	3.2%
$Bkgr_{Dec}$[kCnts]	0.0221	±0.0002	0.7%
$Bkgr_{IRF}$[Cnts/Chnl]	2.21	±0.16	7.2%
$Shift_{IRF}$[ps]	-18.16	±0.92	5.0%
A_{Scatt}[kCnts]	72.9	±1.7	2.2%
τ_{AvInt}[ns]	2.804	±0.016	0.6%
τ_{AvAmp}[ns]	1.314	±0.026	1.9%
λ_{Det}[nm]	420.0000	---	---

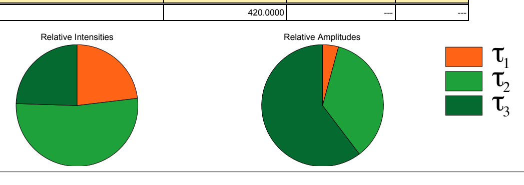

Data Set: 3 / 16

Decay: crv[3]; IRF: crv[0]

Fit

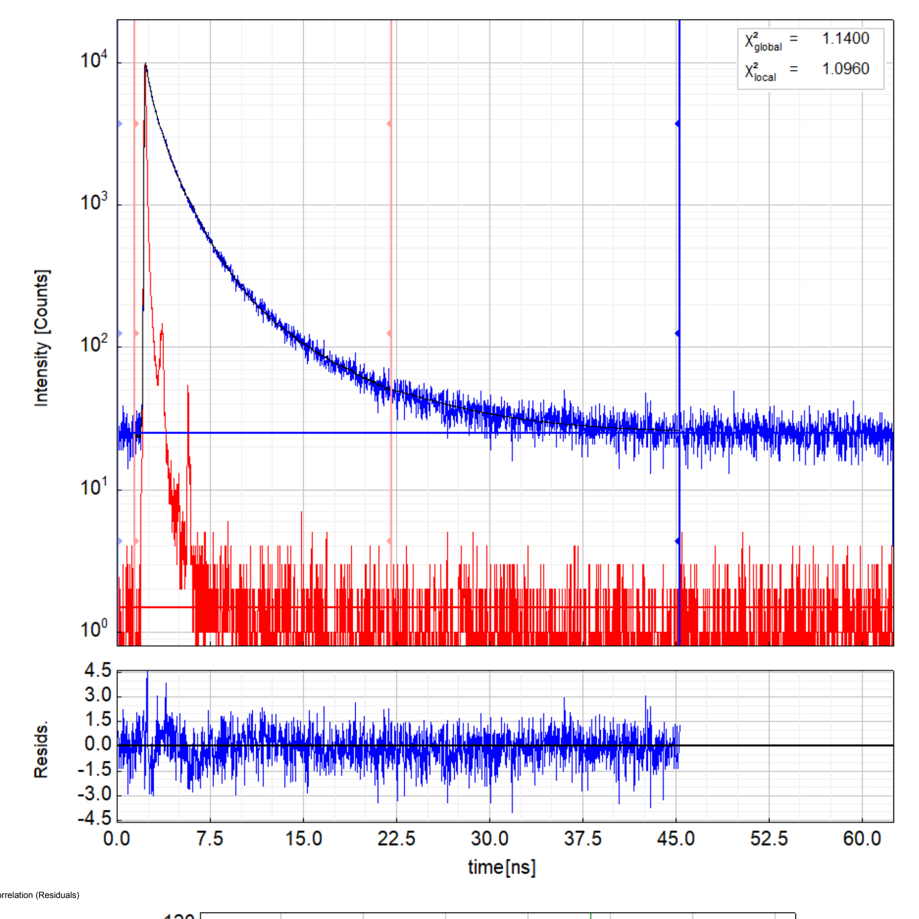

Autocorrelation (Residuals)

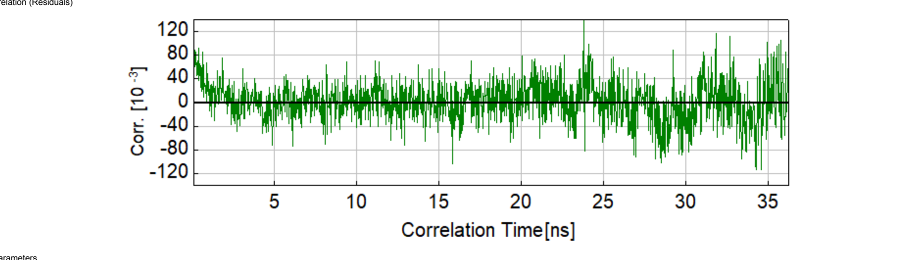

Fitted Parameters

Parameter	Value	Δ	δ
A_1[kCnts/Chnl]	0.453	±0.013	2.7%
τ_1[ns]	7.166	±0.030	0.4%
I_1[kCnts]	129.7	±3.0	2.2%
A_2[kCnts/Chnl]	4.43	±0.11	2.3%
τ_2[ns]	1.943	±0.028	1.4%
I_2[kCnts]	343.9	±3.4	1.0%
A_3[kCnts/Chnl]	6.37	±0.13	2.0%
τ_3[ns]	0.534	±0.025	4.6%
I_3[kCnts]	135.7	±4.1	3.0%
$Bkgr_{Dec}$[kCnts]	0.0250	±0.0003	1.0%
$Bkgr_{IRF}$[Cnts/Chnl]	1.49	±0.13	8.4%
$Shift_{IRF}$[ps]	4.32	±0.90	21%
A_{Scatt}[kCnts]	40.2	±1.5	3.5%
τ_{AvInt}[ns]	2.741	±0.011	0.4%
τ_{AvAmp}[ns]	1.355	±0.024	1.8%
λ_{Det}[nm]	440.0000	---	---

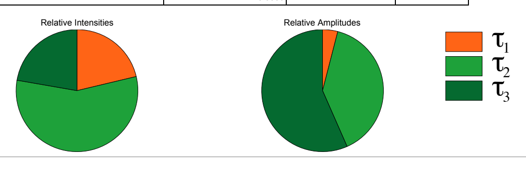

Data Set: 4 / 16

Decay: crv[4]; IRF: crv[0]

Fit

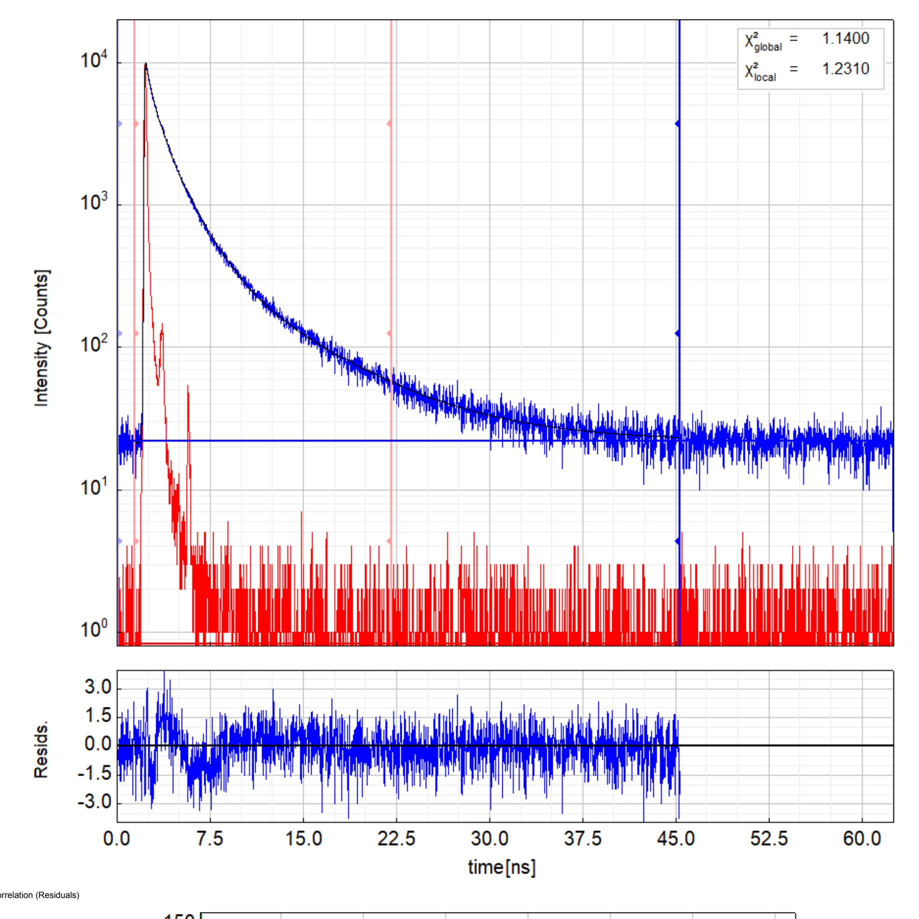

Autocorrelation (Residuals)

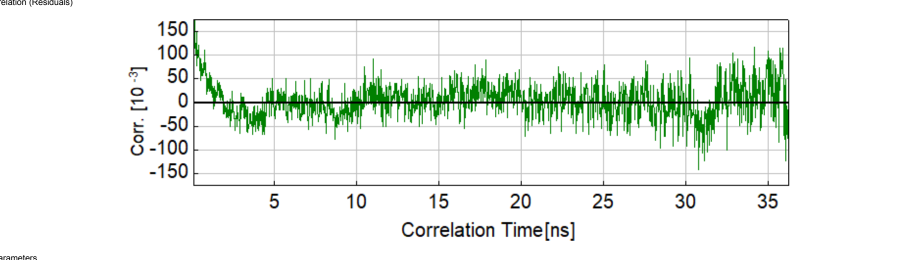

Fitted Parameters

Parameter	Value	Δ	δ
A_1[kCnts/Chnl]	0.5378	±0.0097	1.8%
τ_1[ns]	7.166	±0.030	0.4%
I_1[kCnts]	154.2	±2.6	1.6%
A_2[kCnts/Chnl]	4.73	±0.15	3.1%
τ_2[ns]	1.943	±0.028	1.4%
I_2[kCnts]	367.4	±4.9	1.3%
A_3[kCnts/Chnl]	6.26	±0.12	1.8%
τ_3[ns]	0.534	±0.025	4.6%
I_3[kCnts]	133.4	±5.4	4.0%
$Bkgr_{Dec}$[kCnts]	0.0221	±0.0003	1.2%
$Bkgr_{IRF}$[Cnts/Chnl]	0.829	±0.098	12%
$Shift_{IRF}$[ps]	9.52	±0.88	9.2%
A_{Scatt}[kCnts]	38.06	±0.78	2.0%
τ_{AvInt}[ns]	2.885	±0.015	0.5%
τ_{AvAmp}[ns]	1.421	±0.022	1.5%
λ_{Det}[nm]	460.0000	---	---

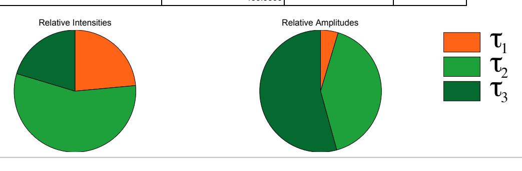

Data Set: 5 / 16

Decay: crv[5]; IRF: crv[0]

Fit

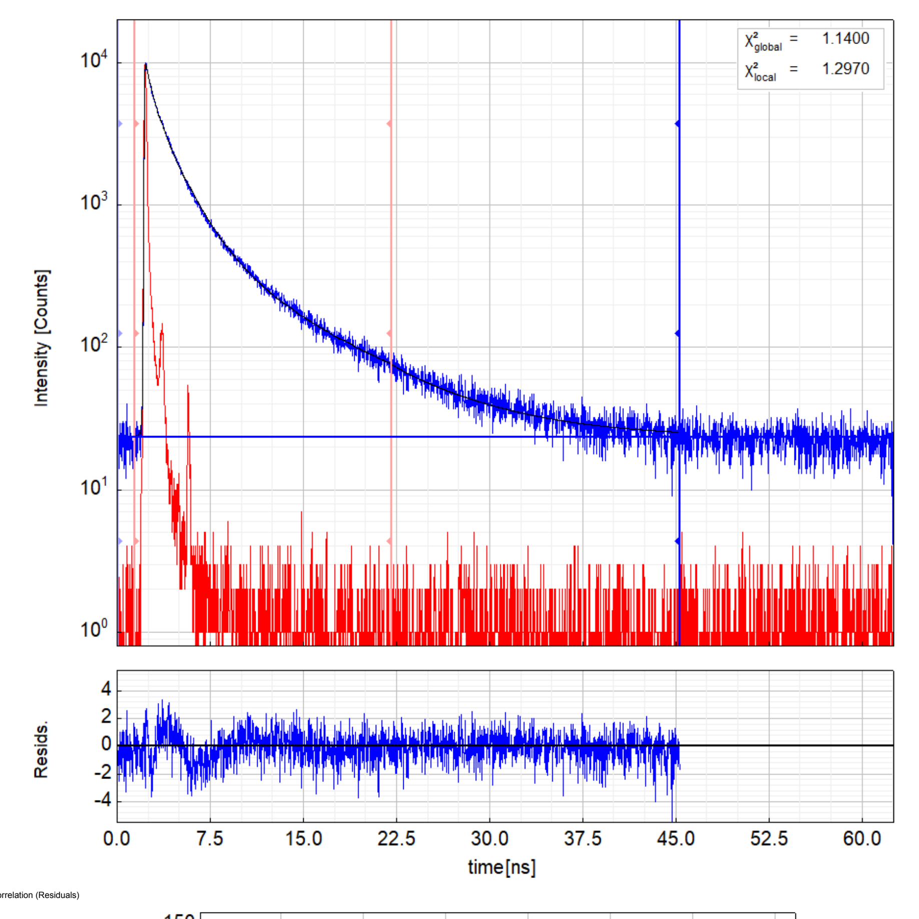

Autocorrelation (Residuals)

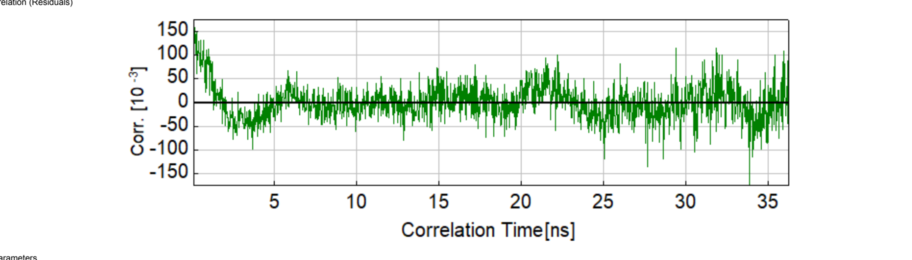

Fitted Parameters

Parameter	Value	Δ	δ
A_1[kCnts/Chnl]	0.7340	±0.0068	0.9%
τ_1[ns]	7.166	±0.030	0.4%
I_1[kCnts]	210.4	±2.7	1.3%
A_2[kCnts/Chnl]	5.04	±0.13	2.5%
τ_2[ns]	1.943	±0.028	1.4%
I_2[kCnts]	391.4	±5.2	1.3%
A_3[kCnts/Chnl]	5.68	±0.12	2.1%
τ_3[ns]	0.534	±0.025	4.6%
I_3[kCnts]	121.1	±6.9	5.7%
$Bkgr_{Dec}$[kCnts]	0.0235	±0.0003	1.2%
$Bkgr_{IRF}$[Cnts/Chnl]	0.28	±0.12	42%
$Shift_{IRF}$[ps]	10.0	±1.1	11%
A_{Scatt}[kCnts]	36.7	±1.2	3.2%
τ_{AvInt}[ns]	3.227	±0.013	0.4%
τ_{AvAmp}[ns]	1.579	±0.016	1.0%
λ_{Det}[nm]	480.0000	---	---

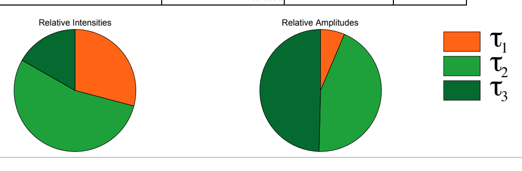

Data Set: 6 / 16

Decay: crv[6]; IRF: crv[0]

Fit

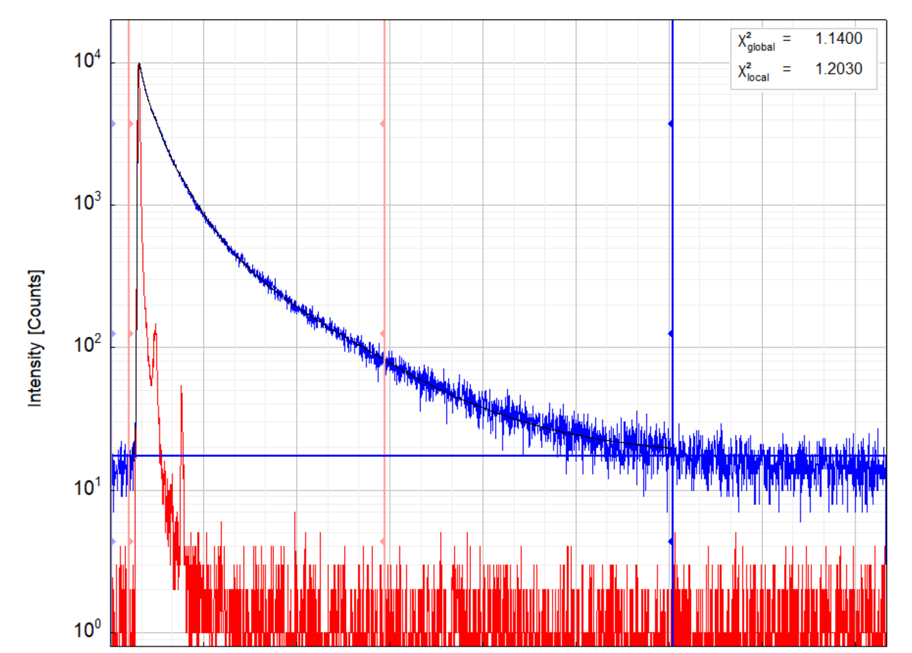

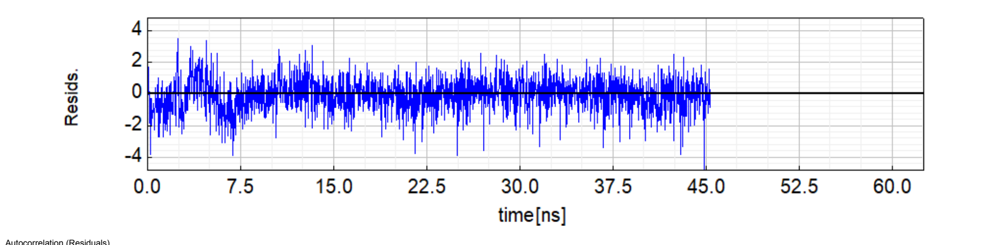

Autocorrelation (Residuals)

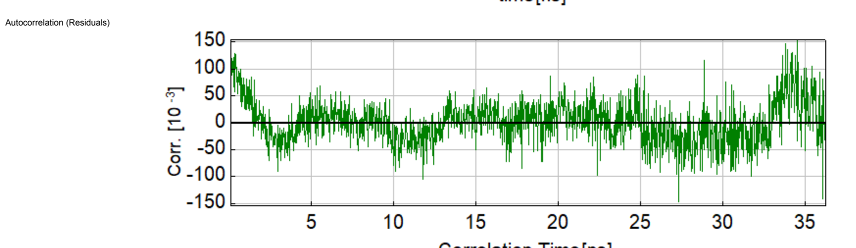

Fitted Parameters

Parameter	Value	Δ	δ
A_1[kCnts/Chnl]	0.953	±0.018	1.8%
τ_1[ns]	7.166	±0.030	0.4%
I_1[kCnts]	273.1	±4.3	1.6%
A_2[kCnts/Chnl]	5.27	±0.14	2.6%
τ_2[ns]	1.943	±0.028	1.4%
I_2[kCnts]	409.3	±5.1	1.2%
A_3[kCnts/Chnl]	5.291	±0.080	1.5%
τ_3[ns]	0.534	±0.025	4.6%
I_3[kCnts]	112.9	±7.6	6.7%
$Bkgr_{Dec}$[kCnts]	0.0173	±0.0002	0.8%
$Bkgr_{IRF}$[Cnts/Chnl]	0.47	±0.13	27%
$Shift_{IRF}$[ps]	12.60	±0.82	6.4%
A_{Scatt}[kCnts]	37.42	±0.83	2.2%
τ_{AvInt}[ns]	3.537	±0.013	0.4%
τ_{AvAmp}[ns]	1.7269	±0.0094	0.5%
λ_{Det}[nm]	500.0000	---	---

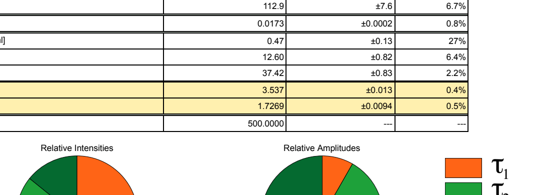

Data Set: 7 / 16

Decay: crv[7]; IRF: crv[0]

Fit

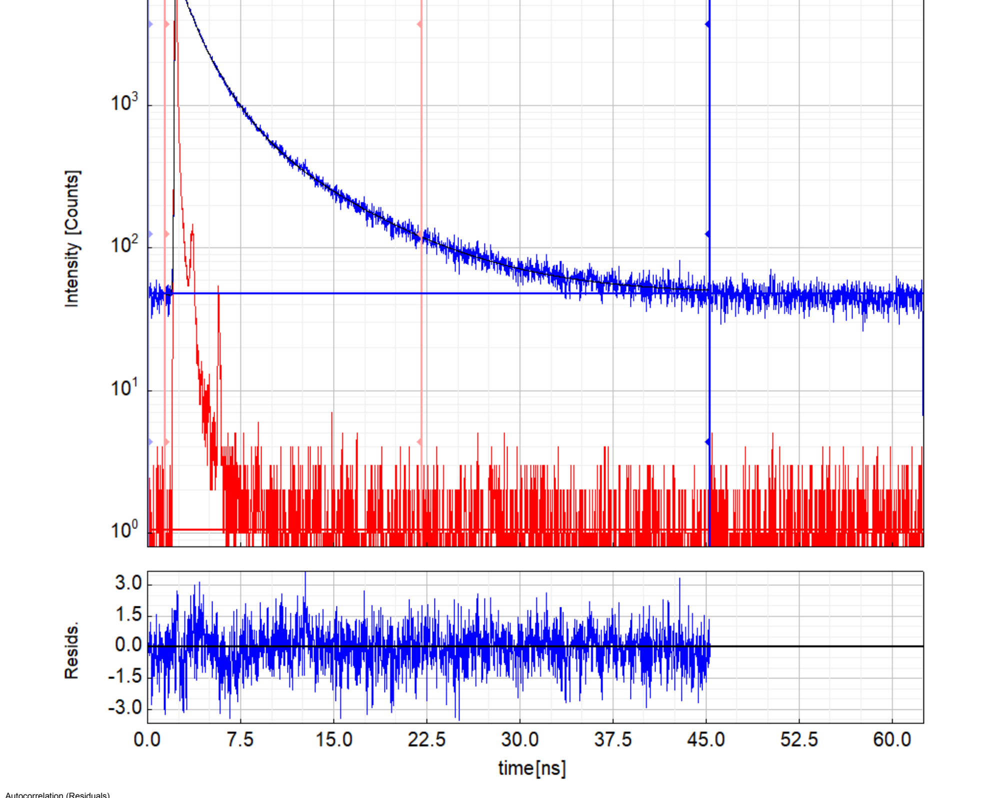

Autocorrelation (Residuals)

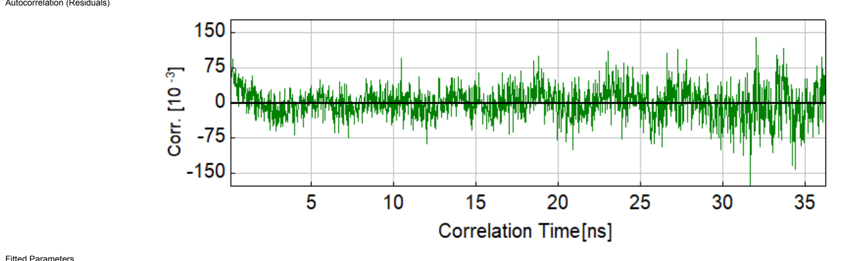

Fitted Parameters

Parameter	Value	Δ	δ
A_1[kCnts/Chnl]	1.133	±0.019	1.6%
τ_1[ns]	7.166	±0.030	0.4%
I_1[kCnts]	324.7	±3.8	1.1%
A_2[kCnts/Chnl]	5.54	±0.13	2.3%
τ_2[ns]	1.943	±0.028	1.4%
I_2[kCnts]	429.9	±3.5	0.8%
A_3[kCnts/Chnl]	4.82	±0.15	2.9%
τ_3[ns]	0.534	±0.025	4.6%
I_3[kCnts]	102.7	±5.5	5.3%
$Bkgr_{Dec}$[kCnts]	0.0478	±0.0002	0.3%
$Bkgr_{IRF}$[Cnts/Chnl]	1.042	±0.080	7.6%
$Shift_{IRF}$[ps]	11.05	±0.69	6.2%
A_{Scatt}[kCnts]	37.05	±0.86	2.3%
τ_{AvInt}[ns]	3.7518	±0.0043	0.1%
τ_{AvAmp}[ns]	1.867	±0.019	1.0%
λ_{Det}[nm]	520.0000	---	---

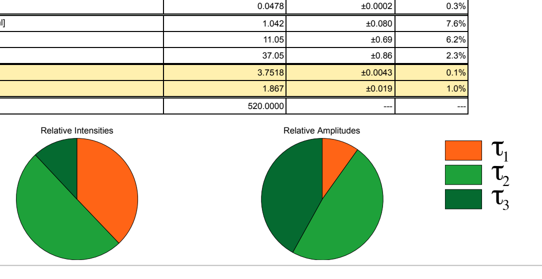

Data Set: 8 / 16

Decay: crv[8]; IRF: crv[0]

Fit

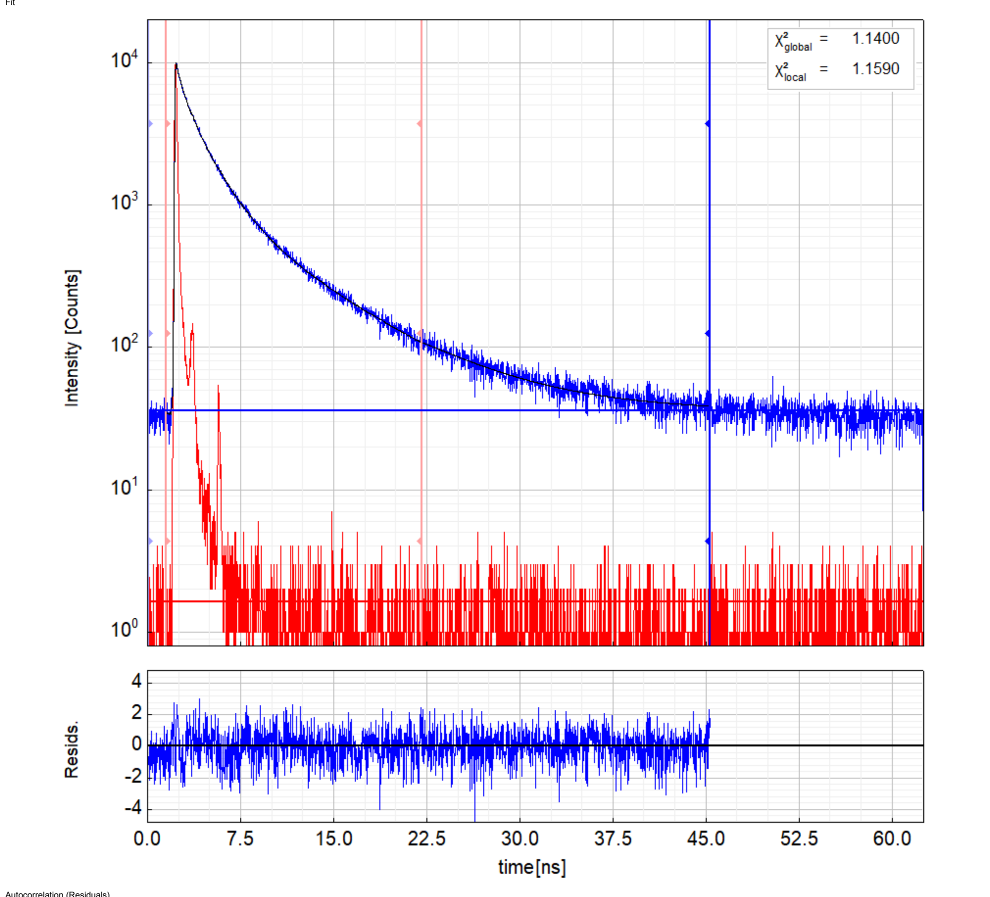

Autocorrelation (Residuals)

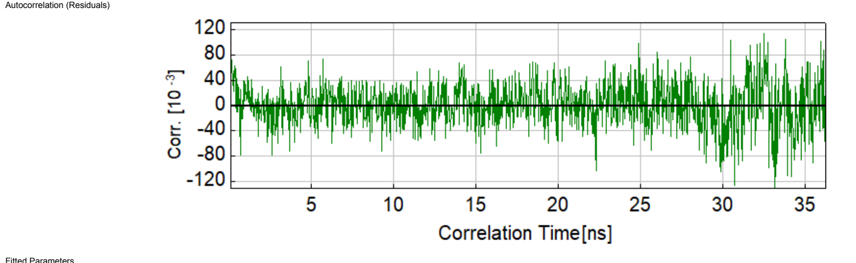

Fitted Parameters

Parameter	Value	Δ	δ
A_1[kCnts/Chnl]	1.241	±0.017	1.4%
τ_1[ns]	7.166	±0.030	0.4%
I_1[kCnts]	355.7	±4.4	1.2%
A_2[kCnts/Chnl]	5.51	±0.14	2.5%
τ_2[ns]	1.943	±0.028	1.4%
I_2[kCnts]	427.5	±3.9	0.9%
A_3[kCnts/Chnl]	4.402	±0.076	1.7%
τ_3[ns]	0.534	±0.025	4.6%
I_3[kCnts]	93.9	±6.9	7.3%
$Bkgr_{Dec}$[kCnts]	0.0357	±0.0003	0.7%
$Bkgr_{IRF}$[Cnts/Chnl]	1.63	±0.15	8.6%
$Shift_{IRF}$[ps]	12.88	±0.67	5.2%
A_{Scatt}[kCnts]	38.44	±0.67	1.7%
τ_{AvInt}[ns]	3.910	±0.011	0.3%
τ_{AvAmp}[ns]	1.968	±0.014	0.7%
λ_{Det}[nm]	540.0000	---	---

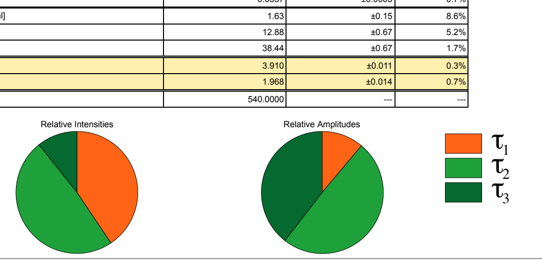

Data Set: 9 / 16

Decay: crv[9]; IRF: crv[0]

Fit

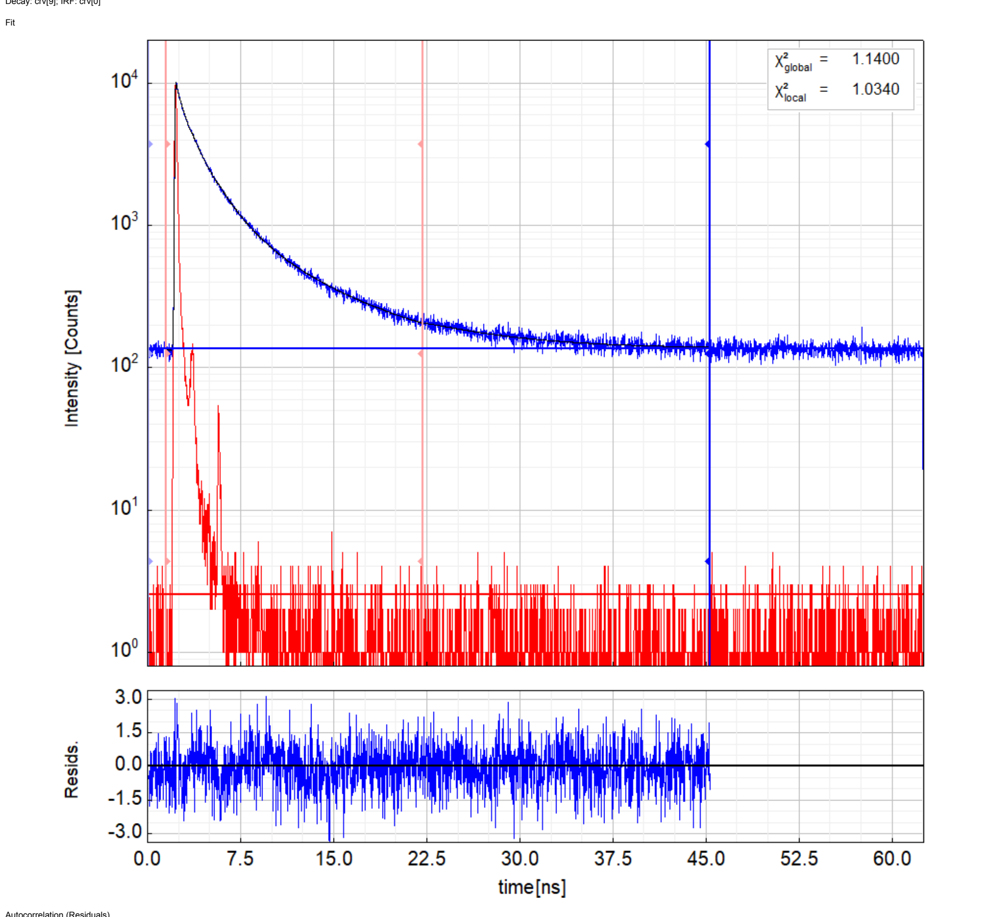

Autocorrelation (Residuals)

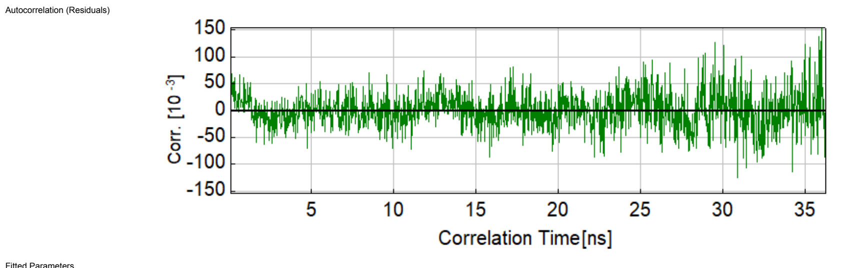

Fitted Parameters

Parameter	Value	Δ	δ
A_1[kCnts/Chnl]	1.356	±0.016	1.2%
τ_1[ns]	7.166	±0.030	0.4%
I_1[kCnts]	388.7	±3.6	0.9%
A_2[kCnts/Chnl]	5.39	±0.11	2.0%
τ_2[ns]	1.943	±0.028	1.4%
I_2[kCnts]	418.3	±3.6	0.8%
A_3[kCnts/Chnl]	4.31	±0.14	3.0%
τ_3[ns]	0.534	±0.025	4.6%
I_3[kCnts]	91.8	±4.6	5.0%
$Bkgr_{Dec}$[kCnts]	0.1361	±0.0005	0.3%
$Bkgr_{IRF}$[Cnts/Chnl]	2.531	±0.099	3.9%
$Shift_{IRF}$[ps]	13.16	±0.78	5.9%
A_{Scatt}[kCnts]	39.70	±0.93	2.3%
τ_{AvInt}[ns]	4.058	±0.017	0.4%
τ_{AvAmp}[ns]	2.035	±0.023	1.1%
λ_{Det}[nm]	560.0000	---	---

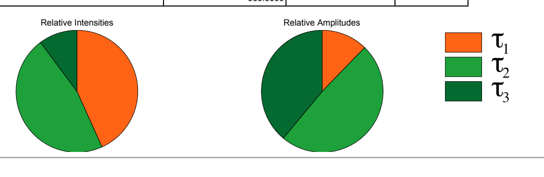

Data Set: 10 / 16

Decay: crv[10]; IRF: crv[0]

Fit

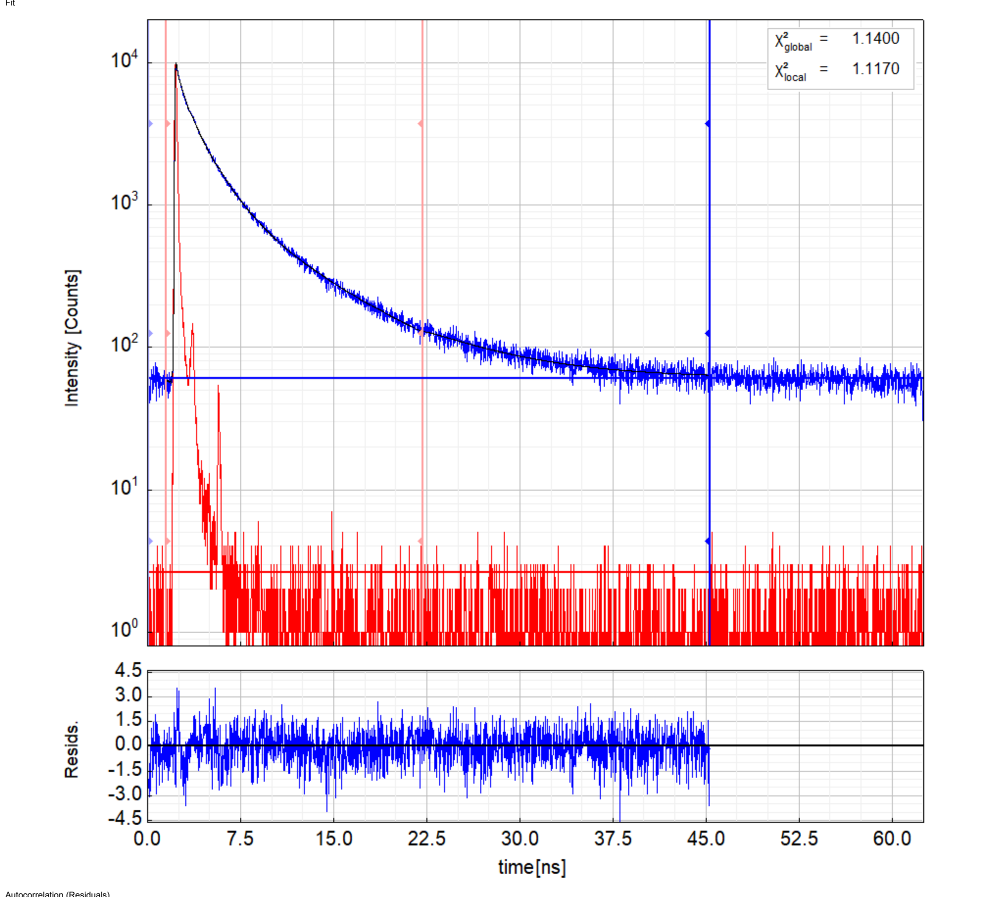

Autocorrelation (Residuals)

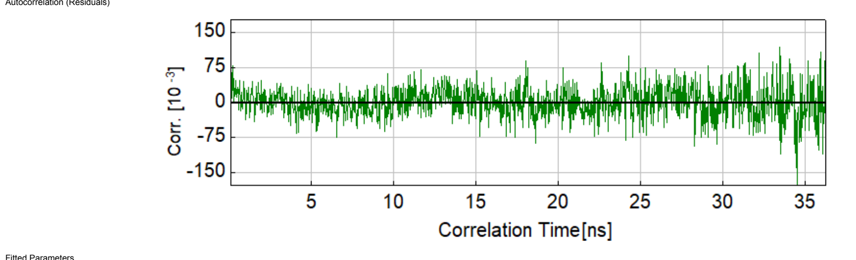

Fitted Parameters

Parameter	Value	Δ	δ
A_1[kCnts/Chnl]	1.347	±0.023	1.7%
τ_1[ns]	7.166	±0.030	0.4%
I_1[kCnts]	386.0	±4.5	1.2%
A_2[kCnts/Chnl]	5.17	±0.14	2.5%
τ_2[ns]	1.943	±0.028	1.4%
I_2[kCnts]	401.6	±3.6	0.9%
A_3[kCnts/Chnl]	4.03	±0.12	2.8%
τ_3[ns]	0.534	±0.025	4.6%
I_3[kCnts]	85.9	±5.7	6.6%
$Bkgr_{Dec}$[kCnts]	0.0608	±0.0004	0.5%
$Bkgr_{IRF}$[Cnts/Chnl]	2.64	±0.11	4.1%
$Shift_{IRF}$[ps]	14.6	±1.2	8.0%
A_{Scatt}[kCnts]	42.6	±1.4	3.1%
τ_{AvInt}[ns]	4.113	±0.012	0.3%
τ_{AvAmp}[ns]	2.072	±0.022	1.0%
λ_{Det}[nm]	580.0000	---	---

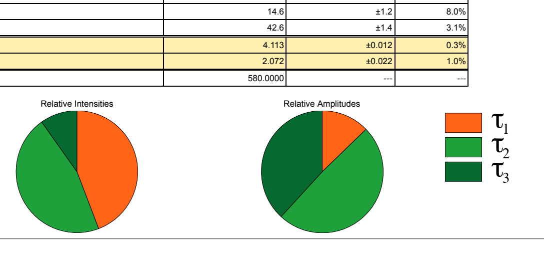

Data Set: 11 / 16

Decay: crv[11]; IRF: crv[0]

Fit

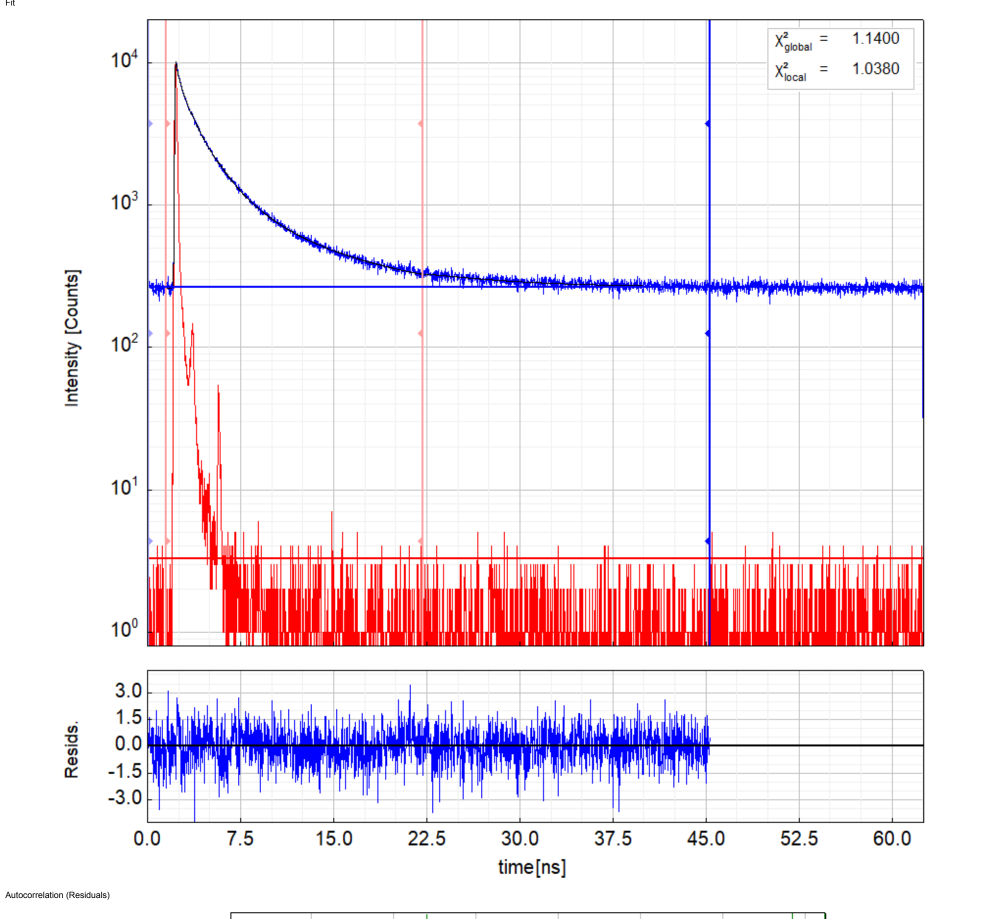

Autocorrelation (Residuals)

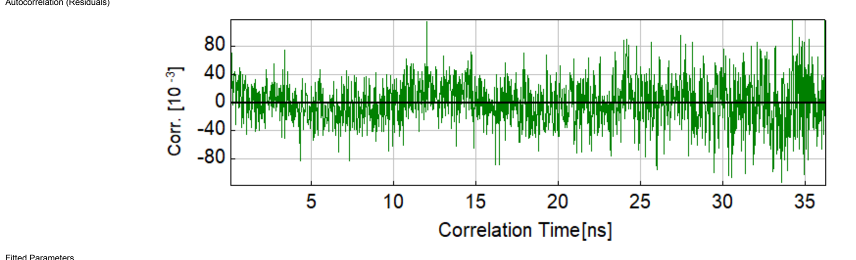

Fitted Parameters

Parameter	Value	Δ	δ
A_1[kCnts/Chnl]	1.328	±0.024	1.8%
τ_1[ns]	7.166	±0.030	0.4%
I_1[kCnts]	380.6	±5.1	1.3%
A_2[kCnts/Chnl]	5.03	±0.13	2.4%
τ_2[ns]	1.943	±0.028	1.4%
I_2[kCnts]	390.2	±4.1	1.0%
A_3[kCnts/Chnl]	3.915	±0.072	1.8%
τ_3[ns]	0.534	±0.025	4.6%
I_3[kCnts]	83.5	±5.5	6.5%
$Bkgr_{Dec}$[kCnts]	0.2645	±0.0004	0.1%
$Bkgr_{IRF}$[Cnts/Chnl]	3.26	±0.14	4.2%
$Shift_{IRF}$[ps]	14.53	±0.27	1.8%
A_{Scatt}[kCnts]	43.81	±0.64	1.4%
τ_{AvInt}[ns]	4.132	±0.012	0.3%
τ_{AvAmp}[ns]	2.081	±0.019	0.9%
λ_{Det}[nm]	600.0000	---	---

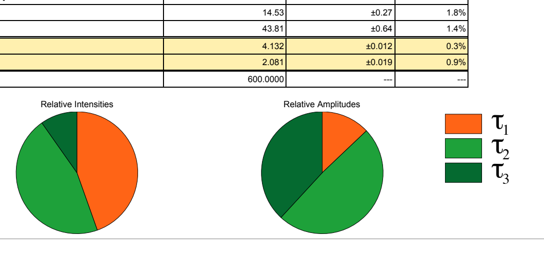

Data Set: 12 / 16

Decay: crv[12]; IRF: crv[0]

Fit

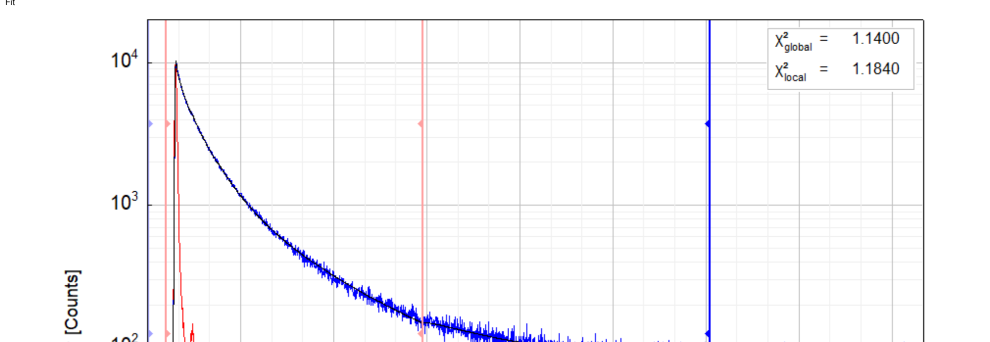

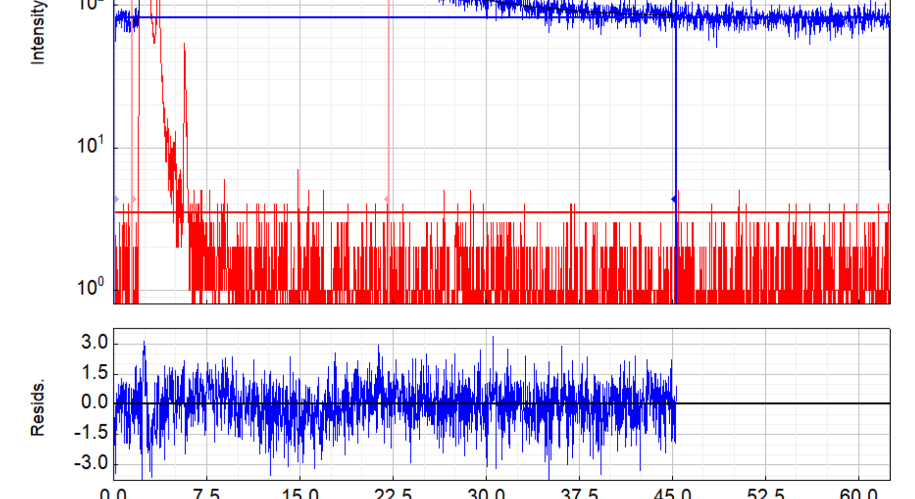

Autocorrelation (Residuals)

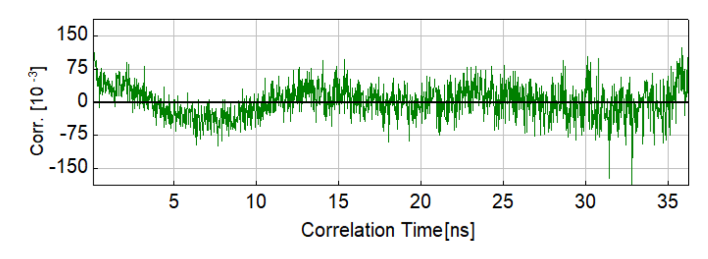

Fitted Parameters

Parameter	Value	Δ	δ
A_1[kCnts/Chnl]	1.496	±0.020	1.3%
$τ_1$[ns]	7.166	±0.030	0.4%
I_1[kCnts]	428.7	±5.0	1.2%
A_2[kCnts/Chnl]	5.249	±0.096	1.8%
$τ_2$[ns]	1.943	±0.028	1.4%
I_2[kCnts]	407.8	±2.3	0.6%
A_3[kCnts/Chnl]	3.50	±0.17	4.6%
$τ_3$[ns]	0.534	±0.025	4.6%
I_3[kCnts]	74.5	±5.8	7.8%
$Bkgr_{Dec}$[kCnts]	0.0817	±0.0006	0.7%
$Bkgr_{IRF}$[Cnts/Chnl]	3.48	±0.21	5.9%
$Shift_{IRF}$[ps]	13.68	±0.93	6.7%
A_{Scatt}[kCnts]	45.8	±1.2	2.4%
$τ_{AvInt}$[ns]	4.286	±0.014	0.3%
$τ_{AvAmp}$[ns]	2.225	±0.013	0.6%
$λ_{Det}$[nm]	620.0000	---	---

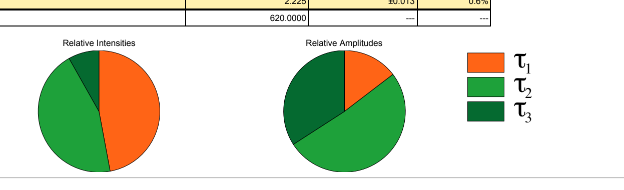

Data Set: 13 / 16

Decay: crv[13]; IRF: crv[0]

Fit

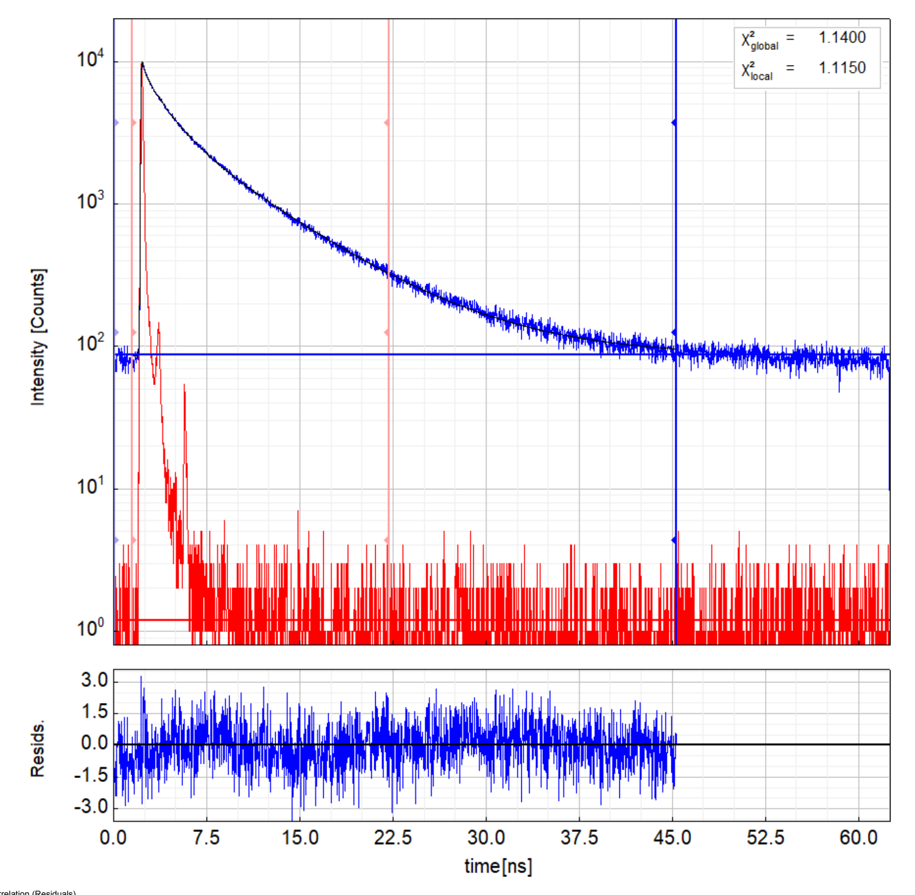

Autocorrelation (Residuals)

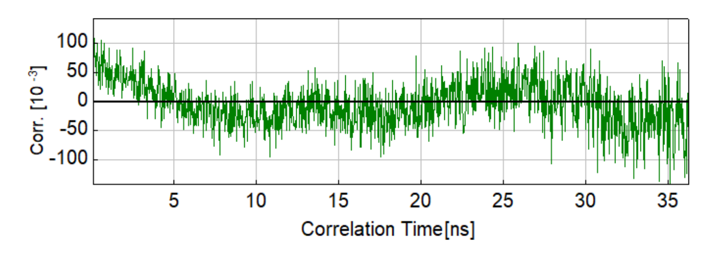

Fitted Parameters

Parameter	Value	Δ	δ
A_1[kCnts/Chnl]	3.837	±0.022	0.6%
$τ_1$[ns]	7.166	±0.030	0.4%
I_1[kCnts]	1 099.8	±3.0	0.3%
A_2[kCnts/Chnl]	4.415	±0.091	2.0%
$τ_2$[ns]	1.943	±0.028	1.4%
I_2[kCnts]	343.0	±3.3	0.9%
A_3[kCnts/Chnl]	2.403	±0.088	3.6%
$τ_3$[ns]	0.534	±0.025	4.6%
I_3[kCnts]	51.3	±4.0	7.7%
$Bkgr_{Dec}$[kCnts]	0.0867	±0.0004	0.4%
$Bkgr_{IRF}$[Cnts/Chnl]	1.188	±0.080	6.7%
$Shift_{IRF}$[ps]	15.74	±0.72	4.5%
A_{Scatt}[kCnts]	40.34	±0.97	2.4%
$τ_{AvInt}$[ns]	5.740	±0.013	0.2%
$τ_{AvAmp}$[ns]	3.506	±0.017	0.5%
$λ_{Det}$[nm]	640.0000	---	---

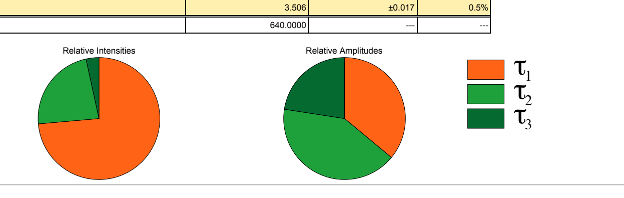

Data Set: 14 / 16

Decay: crv[14]; IRF: crv[0]

Fit

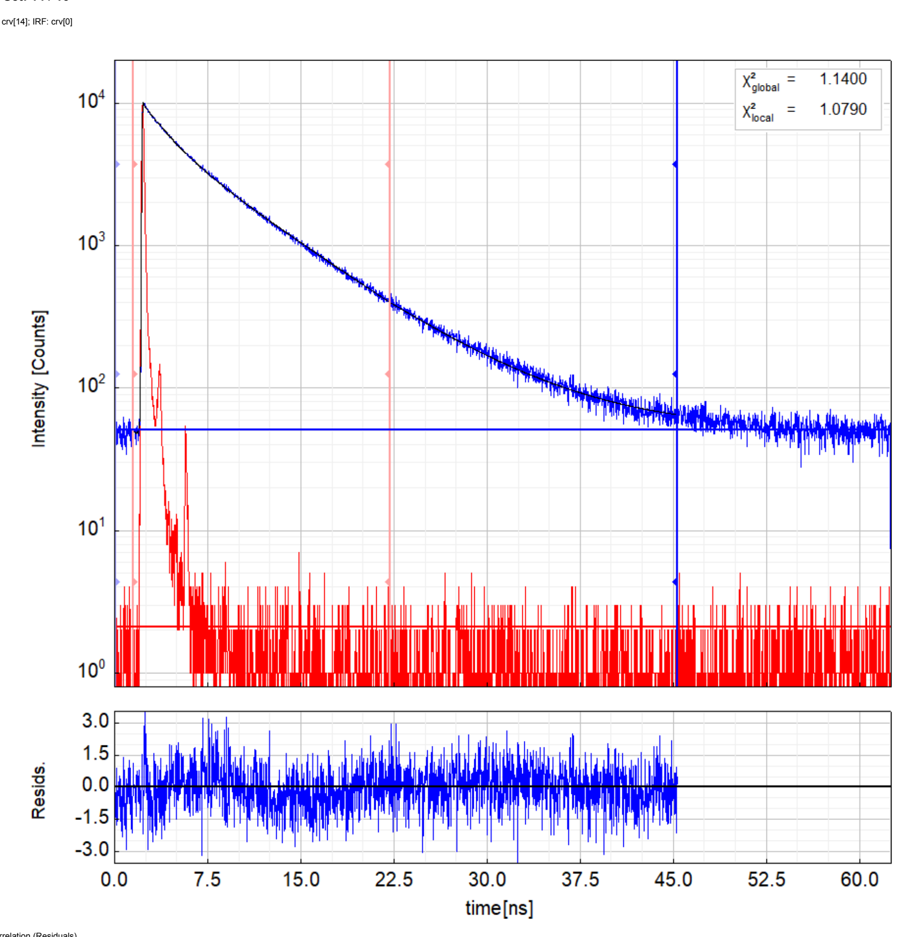

Autocorrelation (Residuals)

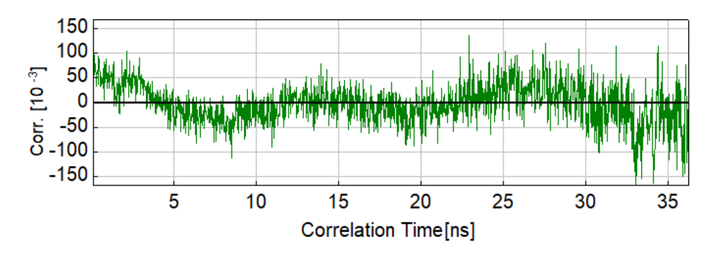

Fitted Parameters

Parameter	Value	Δ	δ
A_1[kCnts/Chnl]	5.951	±0.033	0.5%
$τ_1$[ns]	7.166	±0.030	0.4%
I_1[kCnts]	1 705.7	±5.5	0.3%
A_2[kCnts/Chnl]	4.196	±0.061	1.4%
$τ_2$[ns]	1.943	±0.028	1.4%
I_2[kCnts]	326.0	±3.4	1.0%
A_3[kCnts/Chnl]	0.919	±0.088	9.5%
$τ_3$[ns]	0.534	±0.025	4.6%
I_3[kCnts]	19.6	±2.7	13%
$Bkgr_{Dec}$[kCnts]	0.0511	±0.0003	0.5%
$Bkgr_{IRF}$[Cnts/Chnl]	2.109	±0.094	4.4%
$Shift_{IRF}$[ps]	16.88	±0.74	4.4%
A_{Scatt}[kCnts]	36.1	±1.1	3.1%
$τ_{AvInt}$[ns]	6.273	±0.019	0.3%
$τ_{AvAmp}$[ns]	4.635	±0.024	0.5%
$λ_{Det}$[nm]	660.0000	---	---

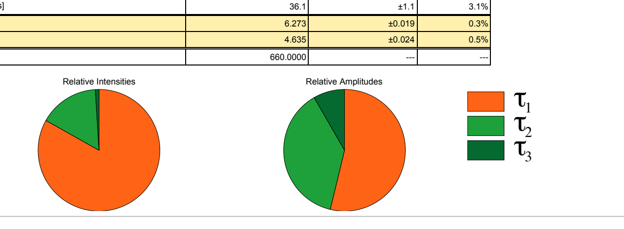

Data Set: 15 / 16

Decay: crv[15]; IRF: crv[0]

Fit

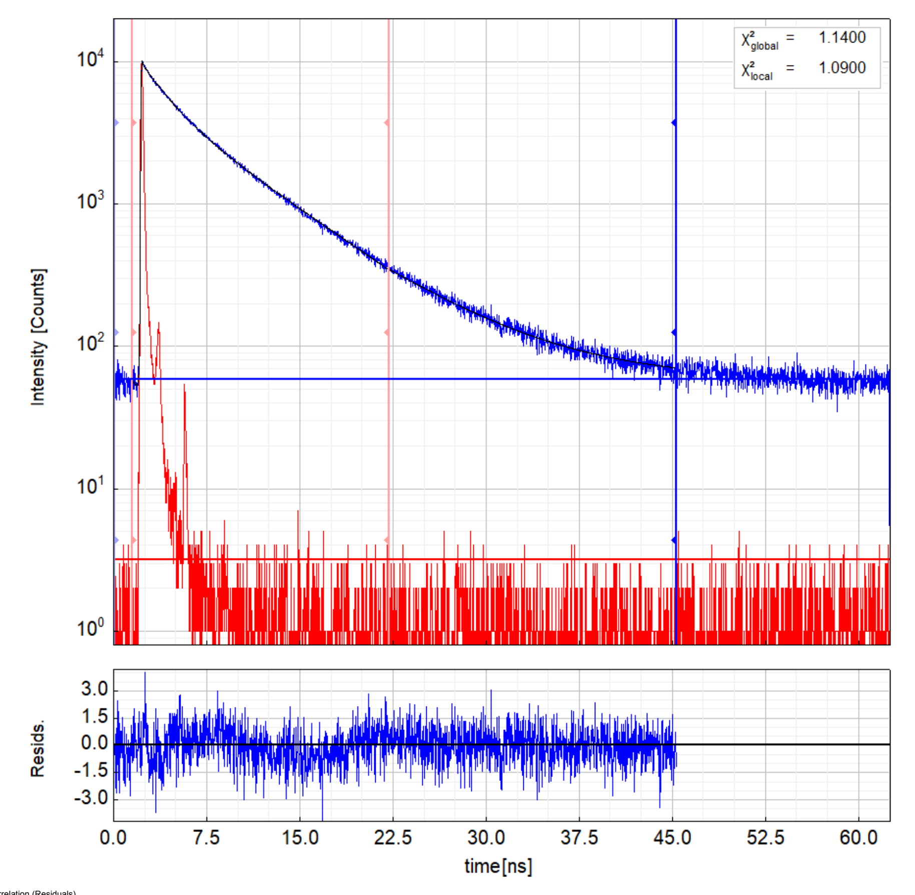

Autocorrelation (Residuals)

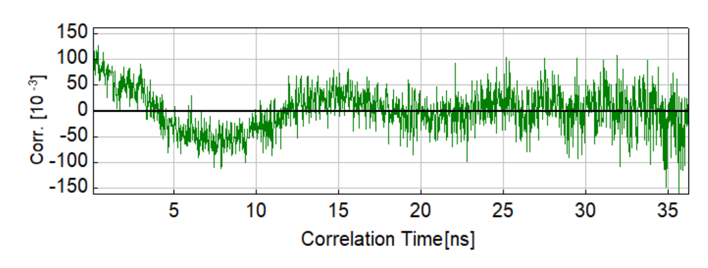

Fitted Parameters

Parameter	Value	Δ	δ
A_1[kCnts/Chnl]	5.311	±0.048	0.9%
$τ_1$[ns]	7.166	±0.030	0.4%
I_1[kCnts]	1 522.1	±7.8	0.5%
A_2[kCnts/Chnl]	4.641	±0.090	1.9%
$τ_2$[ns]	1.943	±0.028	1.4%
I_2[kCnts]	360.5	±8.7	2.4%
A_3[kCnts/Chnl]	0.87	±0.12	13%
$τ_3$[ns]	0.534	±0.025	4.6%
I_3[kCnts]	18.5	±2.0	10%
$Bkgr_{Dec}$[kCnts]	0.0589	±0.0004	0.6%
$Bkgr_{IRF}$[Cnts/Chnl]	3.20	±0.17	5.1%
$Shift_{IRF}$[ps]	18.24	±0.36	2.0%
A_{Scatt}[kCnts]	38.04	±0.54	1.4%
$τ_{AvInt}$[ns]	6.111	±0.015	0.2%
$τ_{AvAmp}$[ns]	4.395	±0.022	0.5%
$λ_{Det}$[nm]	680.0000	---	---

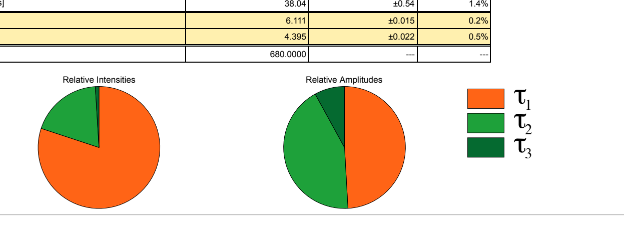

Data Set: 16 / 16

Decay: crv[16]; IRF: crv[0]

Fit

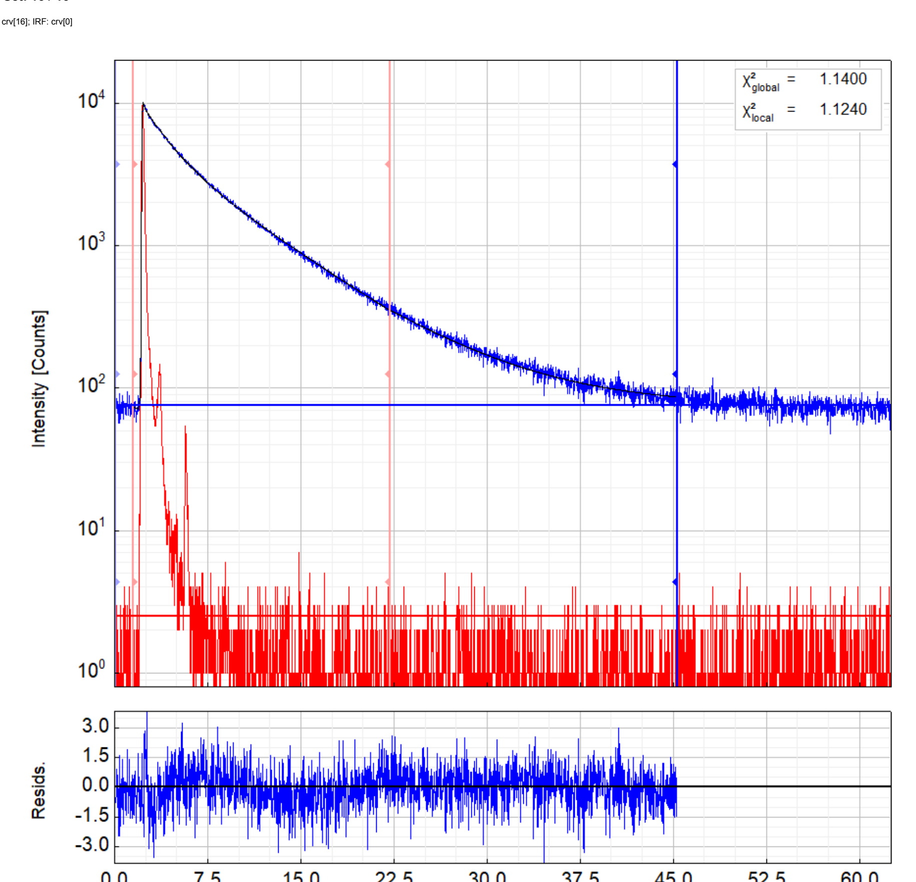

Autocorrelation (Residuals)

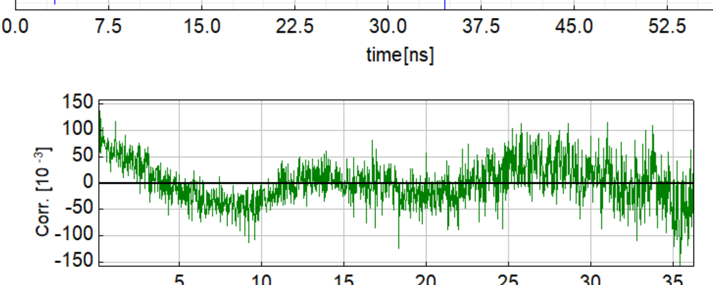

Fitted Parameters

Parameter	Value	Δ	δ
A_1[kCnts/Chnl]	4.881	±0.039	0.8%
$τ_1$[ns]	7.166	±0.030	0.4%
I_1[kCnts]	1 399.0	±6.1	0.4%
A_2[kCnts/Chnl]	4.695	±0.030	0.6%
$τ_2$[ns]	1.943	±0.028	1.4%
I_2[kCnts]	364.8	±5.9	1.6%
A_3[kCnts/Chnl]	1.13	±0.11	9.5%
$τ_3$[ns]	0.534	±0.025	4.6%
I_3[kCnts]	24.1	±3.4	14%
$Bkgr_{Dec}$[kCnts]	0.0756	±0.0005	0.6%
$Bkgr_{IRF}$[Cnts/Chnl]	2.53	±0.17	6.7%
$Shift_{IRF}$[ps]	18.55	±0.54	2.9%
A_{Scatt}[kCnts]	39.31	±0.81	2.0%
$τ_{AvInt}$[ns]	6.011	±0.012	0.2%
$τ_{AvAmp}$[ns]	4.176	±0.030	0.7%
$λ_{Det}$[nm]	700.0000	---	---

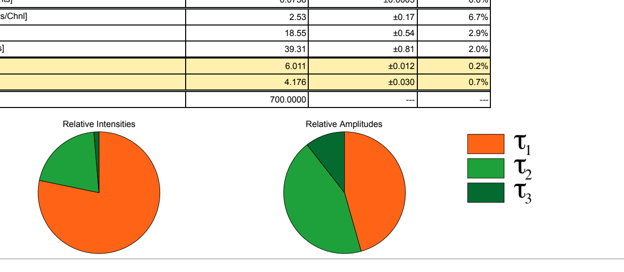

Parameter Plot

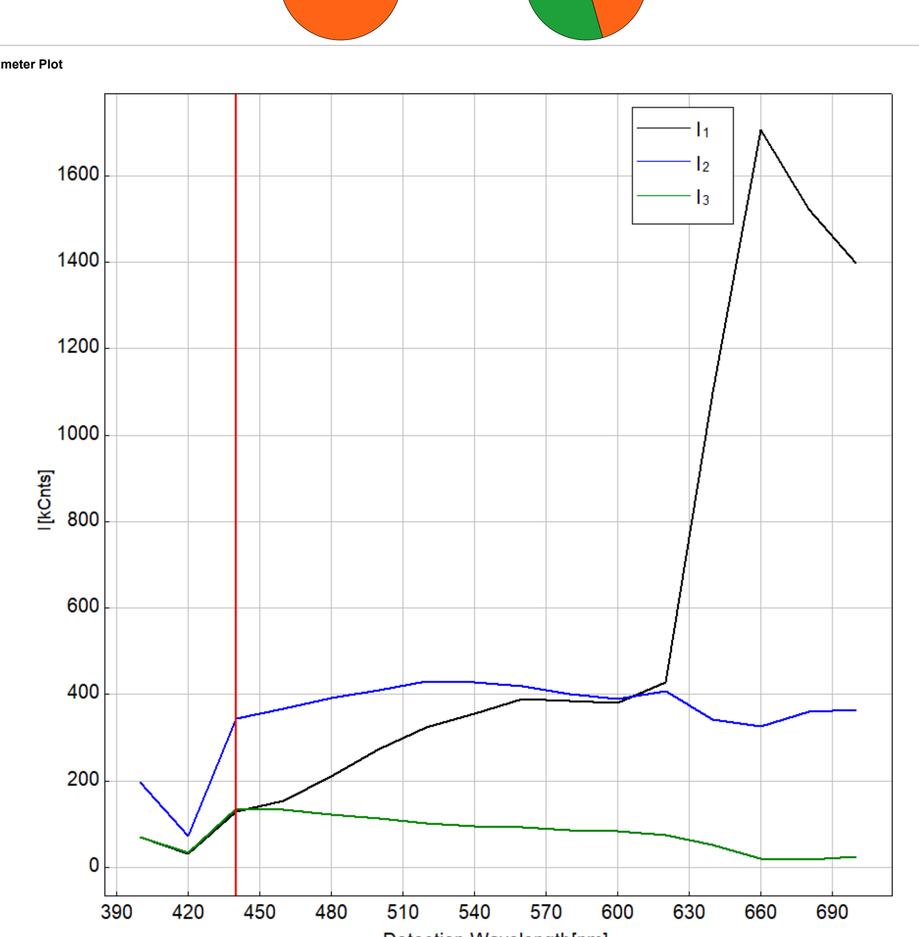

Global Parameters

Parameter	Value	Δ	δ
$τ_1$[ns]	7.166	±0.030	0.4%
$τ_2$[ns]	1.943	±0.028	1.4%
$τ_3$[ns]	0.534	±0.025	4.6%

References

[1] P. Wang, D. Zheng, S. Liu, M. Luo, J. Li, S. Shen, S. Li, L. Zhu, Z. Chen, *Carbon* **2021**, *171*, 946-952.

[2] C. Kütahya, P. Wang, S. Li, S. Liu, J. Li, Z. Chen, B. Strehmel, *Angew. Chem. Int. Ed.* **2020**, *59*, 3166-3171.

[3] C. Kütahya, Y. Zhai, S. Li, S. Liu, J. Li, V. Strehmel, Z. Chen, B. Strehmel, *Angew. Chem. Int. Ed.* **2021**, *60*, 10983-10991.

[4] T. Brömme, D. Oprych, J. Horst, P. S. Pinto, B. Strehmel, *RSC Advances* **2015**, *5*, 69915-69924.

Author Contributions

Xiongfei Luo: made material design, conducted experiments, writing of manuscript draft; Jianyong Wan: investigation, writing - original draft; Nicolai Meckbach: investigation, GPC analysis; Veronika Strehmel: GPC analysis, manuscript editing, reviewing of experimental results; Shujun Li: conceptual design, manuscript editing; Zhijun Chen: general project design, manuscript editing, coordination of project in China, writing, reviewing of experimental results, supervision; Bernd Strehmel: writing manuscript, reviewing of experimental results, coordination of project in Germany, supervision.
